# Guide to the littoral zone vascular flora of Carolina bay lakes (U.S.A.)

**DOI:** 10.3897/BDJ.4.e7964

**Published:** 2016-04-05

**Authors:** Nathan Howell, Alexander Krings, Richard R Braham

**Affiliations:** ‡ Department of Plant and Microbial Biology, North Carolina State University, Raleigh, United States of America; § Department of Forestry and Environmental Resources, North Carolina State University, Raleigh, United States of America

**Keywords:** North American southeastern Coastal Plain lakes, floristics, aquatic, emersed vegetation

## Abstract

**Background:**

Carolina bays are elliptic, directionally aligned basins of disputed origin that occur on the Atlantic Coastal Plain from the Delmarva Peninsula to southern Georgia. In southeastern North Carolina, several large, natural, lacustrine systems (i.e., Carolina bay lakes) exist within the geomorphological features known as Carolina bays. Within the current distribution of Carolina bays, Bladen and Columbus counties (North Carolina) contain the only known examples of Carolina bay lakes. The Carolina bay lakes can be split into two major divisions, the “Bladen Lakes Group” which is characterized as being relatively unproductive (dystrophic – oligotrophic), and Lake Waccamaw, which stands alone in Columbus County and is known for its high productivity and species richness. Although there have been several studies conducted on these unique lentic systems, none have documented the flora comprehensively.

**New information:**

Over the 2013−2014 growing seasons, the littoral zone flora of Carolina bay lakes was surveyed and vouchered. Literature reviews and herbarium crawls complemented this fieldwork to produce an inventory of the vascular plant species. This survey detected 205 taxa (species/subspecies and varieties) in 136 genera and 80 vascular plant families. Thirty-one species (15.2%) are of conservation concern. Lake Waccamaw exhibited the highest species richness with 145 catalogued taxa and 26 species of conservation concern. Across all sites, the Cyperaceae (25 spp.), Poaceae (21 spp.), Asteraceae (13 spp.), Ericaceae (8 spp.), Juncaceae (8 spp.), and Lentibulariaceae (6 spp.) were the six most species-rich vascular plant families encountered. A guide to the littoral zone flora of Carolina bay lakes is presented herein, including dichotomous keys, species accounts (including abundance, habitat, phenology, and exsiccatae), as well as images of living species and vouchered specimens.

## Introduction

Carolina bays are shallow elliptical depressions of disputed origin aligned in a northwest-southeast direction on the Atlantic Coastal Plain of the eastern United States from the Delmarva Peninsula to southern Georgia (Tuomey 1848, Glenn 1895, Melton and Schriever 1933, Prouty 1952, LeBlond 1995, Sharitz 2003). In southeastern North Carolina, several large, natural, lacustrine systems exist within the geomorphological features known as Carolina bays. Within the current distribution of Carolina bays, Bladen and Columbus counties in North Carolina contain the only known examples of Carolina bay lakes. Carolina bay lakes can be split into two major divisions, the “Bladen Lake Group”, which are dystrophic to oligotrophic and relatively unproductive, and Lake Waccamaw, which stands alone in Columbus County and is known for its high productivity, species richness, and rates of endemism (Weiss and Kuenzler 1976, Casterlin et al. 1984, LeBlond 1995, North Carolina Division of Water Quality, Environmental Sciences Section, Intensive Survey Unit 2009, North Carolina Division of Water Quality, Environmental Sciences Section, Intensive Survey Unit 2012, Schafale 2012).

Although there have been several studies conducted on these unique lentic (freshwater) systems (Prouty 1935, Eyles 1941, Hubbs and Raney 1946, Frey 1949, Frey 1951a, Frey 1951b, Frey 1954, Louder 1962, Casterlin et al. 1984, Newman and Schalles 1990; see also Suppl. material 1), none have focused comprehensively on their vegetation. Several manuals, guides, and broader floristic works are available on wetlands of North Carolina and the eastern United States (Suppl. material 2), but few floras have followed the guidelines and recommendations of Palmer et al. (1995) and Denslow et al. (2010) and documented the site-specific aquatic flora of wetlands, streams, rivers, ponds, or lakes in North Carolina (Sieren and Warr 1992, Warren et al. 2004). Nifong (1998) estimated the occurrence of 620 functionally intact, unaltered, Carolina bays remaining in the Coastal Plain of the Carolinas, and an annual rate of loss of about 36 functionally intact North Carolina bays to development, agriculture, silviculture, and other means. It is imperative that the few remaining unaltered bays be studied, especially Carolina bay lakes, considering that with increasing demotechnic growth (Wetzel 2001) and insecure protection status of isolated wetlands (Sharitz 2003), many freshwater systems, including Carolina bays and bay lakes, face an uncertain future.

A narrow time frame exists to study the few remaining natural freshwater systems not affected by severe degradation. Denslow et al. (2010) found only one aquatic flora (Sieren and Warr 1992) within the state of North Carolina between the years 1834−2009, showing a neglect of aquatic systems in floristic studies within the state. To help fill this gap in knowledge, the objectives of this study were to (1) inventory the littoral zone vascular flora of Carolina bay lakes through the collection of voucher specimens, (2) provide a comprehensive checklist of the littoral zone vascular flora based on integration of new and historic collections and reports, and (3) create an illustrated guide based on the checklist.

### Background

***Lake Ecosystems and Abiotic Factors***

*Catchment Area*

Lakes (also referred to as lentic systems along with ponds) exhibit physical and chemical characteristics unique to the soils, vegetation, and land use activities present on immediately surrounding lands; thus, no two lakes are exactly the same (Moss et al. 1996, Brönmark and Hansson 2005). All lakes occur within *catchment areas*. A catchment area can also be referred to as a watershed or drainage basin, which is simply the zone of land surrounding a lake that drains precipitation into the lake basin (Brönmark and Hansson 2005). The area, geology, edaphic (soil) properties, land use, and vegetation of catchment areas affect the acidity, water color, nutrient input, and chemical composition of lakes (Wetzel 2001, Brönmark and Hansson 2005). Large catchment areas have a more pronounced impact on the chemical properties of lakes because they drain more precipitation, and thus the potential for more nutrients, into the lake basin. Consequently, land use activities that release excessive nutrient inputs into large catchment areas (e.g., intensive agriculture) are likely to cause eutrophication (Casterlin et al. 1984, Brönmark and Hansson 2005).

*Water Color*

The observed color of natural lake waters is caused by the selective absorption of wavelengths as light penetrates through the water column (Wetzel 2001). Organic matter (i.e., dead and decomposing plant and animal parts) is the principal determinant of water color in lakes (Juday and Birge 1933, Rasmussen et al. 1989, Brönmark and Hansson 2005). Due to differences in wavelength absorption, waters with little dissolved organic matter, such as hardwater lakes or glacial streams, appear blue/green, and, in contrast, lakes containing much dissolved organic matter in the form of humic substances (e.g., Carolina bay lakes and bogs) appear yellow/red or “tea-stained” in color. Humic substances are large molecules formed as a result of decomposing organic matter; they are difficult for the microbial community to degrade and are long-lived within the lake system (Brönmark and Hansson 2005).

*Trophic status*

Trophic status refers to the rate at which organic matter is supplied by or transported into a lake. Humic substances are the most common component in allochthonous organic matter; consequently, wetlands that receive the bulk of their organic matter from allochthonous sources (e.g., Carolina bay lakes, bogs, pocosins) are heavily “tea-stained” and are commonly referred to in the southeastern United States as “black water” lakes, streams, rivers, ponds. Lakes receiving the majority of their organic matter from allochthonous sources have been given the term *dystrophic*. Dystrophic lakes have low productivity and are often acidic due to large quantities of allochthonous humic input.

Phosphorous is limiting in freshwater systems and is therefore a useful determinant for production. Phosphorous concentrations are easier to quantify than carbon content and production, and, as a result, trophic status is often classified based on phosphorous content (Brönmark and Hansson 2005). *Oligotrophic* lakes experience low productivity associated with autochthonous carbon production and low levels of phosphorous and nitrogen. *Eutrophic* lakes experience high productivity associated with autochthonous production and high levels of phosphorous and nitrogen.

*pH*

The unit commonly used to measure acidity is pH. It is technically defined as the reciprocal of the activity of free hydrogen ions (H^+^; Covington et al. 1985). Because pH is measured on a logarithmic scale, a change of one unit in pH corresponds to a ten-fold increase in hydrogen ions (Brönmark and Hansson 2005). pH is measured on a scale of 1–14; most lakes possess a pH between 6 and 9, but extreme cases of acidity (1–5) and alkalinity (10–14) also exist depending upon various abiotic and biotic conditions within a lake’s catchment area (see above; Brönmark and Hansson 2005). Geological and hydrological conditions within catchment areas primarily control the pH of lakes; however, acid rain can also affect the pH of lakes. In North America, coal-fired power plants and other industries emit sulfur dioxide (SO_2_) into the atmosphere. As weather systems make their way across North America from west to east, they pick up this sulfur dioxide (SO_2_) and deposit it across the landscape in the form of precipitation (i.e., acid rain). The cumulative effects of acid rain deposition on both terrestrial and aquatic systems is known to be most severe in the eastern United States; this is due to the region's geographic location in relation to broad-scale weather paterns and industries emitting sulfur dioxides (Schindler 1988).

Photosynthesis and respiration are also known to affect the pH of waters by influencing the amount of carbon dioxide (CO_2_) in the water column. When CO_2_ is taken up and stored by aquatic macrophytes, phytoplankton, and algae during photosynthesis, free hydrogen ions (H^+^) are neutralized or taken up by carbonates, bicarbonates, and hydroxides, causing a reduction in H^+^ and thus a higher pH. Respiration adds CO_2_ into the system, thus releasing free H^+^ into the water column and lowering the pH (Brönmark and Hansson 2005). Because photosynthesis and respiration can cause fluctuating differences in pH within a 24-hour cycle, alkalinity is typically considered to be a better measurement of a lake's acidification status (Brönmark and Hansson 2005).

*Alkalinity*

Alkalinity refers to a lake's ability to neutralize strong inorganic acids (i.e., it is a measure of how sensitive a lake is to acidification). It is now used synonymously with acid neutralizing capacity (ANC; Wetzel 2001). Today, alkalinity is generally expressed in milliequivalents per liter (meq/L), but has commonly been recorded in the past in milligrams per liter (mg/L; Brönmark and Hansson 2005). Lakes with an alkalinity above 0.5 meq/L have good buffering capacities, whereas lakes with alkalinities below 0.01 meq/L have little or no buffering capacities ([Bibr B2205550][Bibr B2143725]). Lakes with low alkalinities are susceptible to drops in pH with only small additions of acid (H^+^), whereas lakes with high alkalinities can withstand the addition of acid (H^+^) into their systems without proportional drops in pH (Brönmark and Hansson 2005).

Wetzel (2001) noted that the property of alkalinity in most fresh waters is imparted by the presence of carbonates (i.e., carbonate, bicarbonate, calcium carbonate). Carbonates and hydroxides remove hydrogen ions (H^+^) from lakes, thus neutralizing their acidity (i.e., raising the pH to a more basic status). Lake Waccamaw, the largest Carolina bay lake, has a high alkalinity (7.0−12 mg/L or 0.14−0.24 meq/L; Weiss and Kuenzler 1976) due to the presence of both subsurface and surficial limestone deposits within and around the lake. As a result, it possesses a neutral to basic pH (6.8−8.5 s.u.) and has the ability to handle larger additions of acid.

***Carolina Bays, Bay Lakes, and Pocosins***

*Carolina Bays*

**Figure 1.**
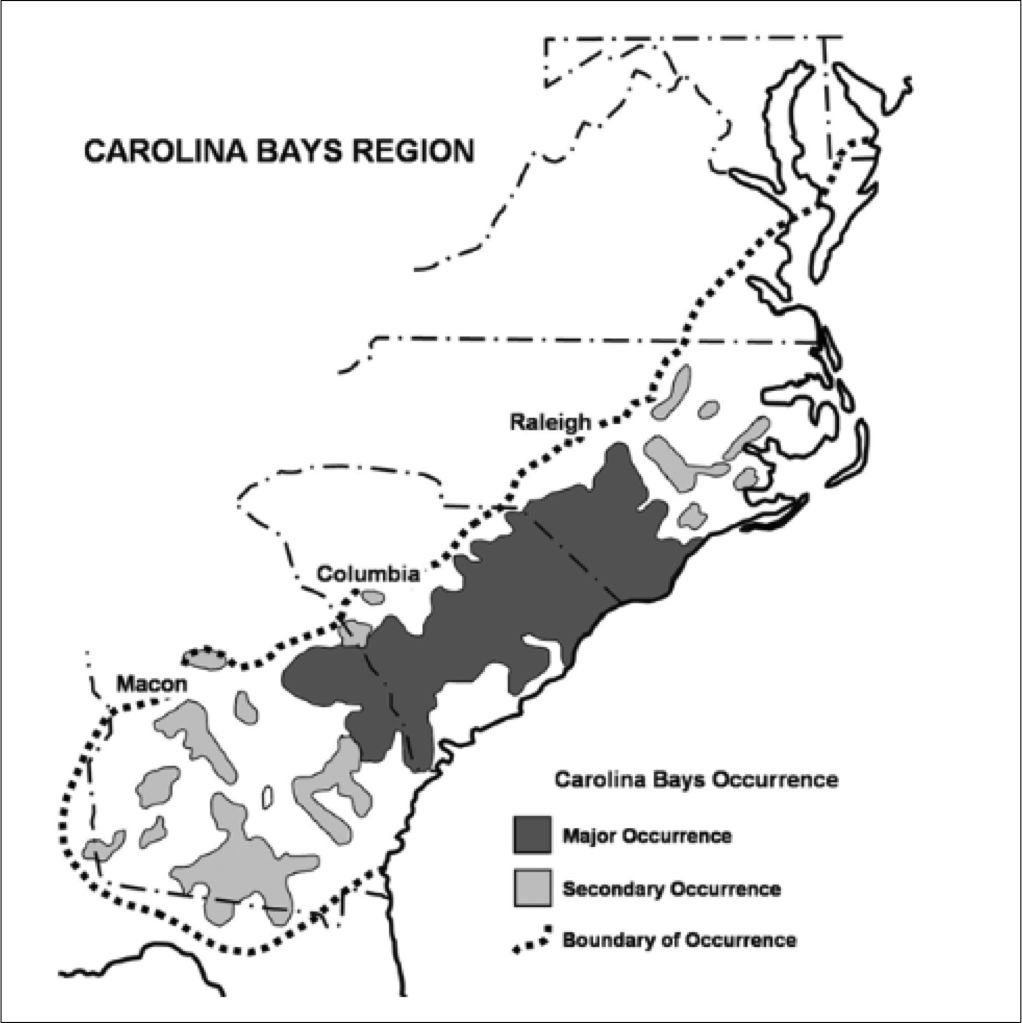
Core distribution of Carolina bays. Carolina bays are known to occur from the Delmarva Peninsula south to southern Georgia. Although many historical texts frequently cite the distribution range of Carolina bays as occurring from New Jersey south to Florida, the more narrow range from the Delmarva Peninsula to southern Georgia is more accurate. Conversations with state agencies and personnel from all states included in the broader range of Carolina bays confirm their “apparent absence” in southern New Jersey and northern Florida. The core distribution of Carolina bays is located in northeast South Carolina and southeast North Carolina (darker gray). The bays in this region would be considered “classic” Carolina bays (i.e., matching all of the well-known and consistent geomorphological criteria in the literature), whose geomorphology is described well by Prouty (1952) and Ross (2003). Toward the peripheries of the known Carolina bay distribution range, the term Carolina bay tends to be used loosely and is not used in its strictest sense (i.e., depression wetlands; Chick Gaddy, pers. comm.). Figure taken from Ross (2003).

The core concentration of Carolina bays occurs in southeastern North Carolina and northeastern South Carolina (Ross 2003; Fig. 1). Although these depressions share the same elliptical shape, they vary dramatically in length along their long axis from 50 m to 8 km (with some as large as 3,600 ha; Prouty 1935, Thom 1970, Savage 1982, Sharitz and Gibbons 1982). Nifong (1982) suggested that there are fewer than 13,000 bays (unaltered and altered) left in the Coastal Plain of the Carolinas, as opposed to the 400,000 proposed by Prouty (1935). It was not until the early 20^th^ century that researchers fully recognized the magnitude and extent of Carolina bay distribution by the use of airplanes and soon-to-be aerial imagery.

Savage (1982) declared that: “When seen from the air, Carolina bays are an astounding, unforgettable revelation. But though hundreds of thousands lie clearly visible, scattered across the Atlantic Coastal Plain from Maryland to northern Florida, they are often all but unrecognizable to the uninitiated eyes of groundlings”. The first aerial images produced of the Atlantic Coastal Plain exposed Carolina bays to both citizens and scientists on a broad scale; moreover, they initiated a flurry of scientific research on Carolina bay distribution, numbers, origin, vegetation, and soils.

The term *bay* is used to describe these landscape features not because they commonly contain hydric soils or are inundated with water, but because of the presence of three species of bay tree typically found within and around their elliptical boundaries (i.e., *Magnolia virginiana* L. [sweetbay; Magnoliaceae], *Persea palustris* (Raf.) Sarg. [swamp bay; Lauraceae], and *Gordonia lasianthus* (L.) J. Ellis [loblollybay; Theaceae]. Traditionally, the term “bay” tree has been used when speaking of the laurel trees within the Lauraceae family. While *Persea palustris* may be properly referred to as a “bay” tree, *Gordonia lasianthus* and *Magnolia virginiana* may not (*sensu stricto*), hence their common names being one word (i.e., loblollybay and sweetbay). *Gordonia lasianthus* and *Magnolia virginiana* bear a noticeable morphological resemblence to the laurels of the Lauraceae; thus, they are generally referred to as “bay” trees (*sensu lato*). North of Virginia, these mysterious landscape features are referred to as *Delmarva potholes*, *bays, or basins* (Tiner and Burke 1995, Lide 1997, Sharitz 2003, Tiner 2003). The inability to agree upon a clear-cut definition and universal name for these unique geological features has caused some discrepancy among estimates of bay numbers (Lide 1997).

Collectively, Carolina bays and pocosins represent the largest total acreage of palustrine wetlands in the Carolinas (Wilson 1962, Richardson 1983, Richardson and Gibbons 1993, Nifong 1998). Pocosins occur on the Atlantic Coastal Plain from southern Virginia to northern Florida (essentially the same range as Carolina bays). Unlike Carolina bays, pocosins have been poorly mapped throughout the whole of their range. Wilson (1962) and Richardson (1981) comprehensively mapped the pocosins of North Carolina. It is estimated that ca. 70% of the nation's pocosin habitat occurs in North Carolina and that over 50% of the state's palustrine wetlands are comprised of pocosins (Richardson and Gibbons 2003). Richardson (1981) suggested that ca. 8,300 km^2 ^(3,200 mi^2^) of unaltered pocosins were drained for other land uses between 1962 and 1979; and ca. 3,700 km^2 ^(1,450 mi^2^) of unaltered pocosins remained in North Carolina in 1980. Based on the presence of wetland soils (i.e., “soils formed under conditions of saturation, flooding, or ponding long enough during the growing season to develop anaerobic conditions in the upper part” [Vepraskas and Richardson 2001]), North Carolina is estimated to have contained nearly 7.5 million acres (3.03 million hectares) of wetlands prior to European settlement of the state; 95% of these wetlands were located in the Coastal Plain (North Carolina Division of Environmental Management 1994).

Geographic location, soil depth, soil type, surrounding land use, varying hydrology, and fire regimes interact to create vastly different vegetative and wetland assemblages within Carolina bays. Nifong (1998) summarized this diversity, noting that bays included “in some form, virtually every non-marine wetland system found on the southeastern Coastal Plain, including brackish marsh, freshwater pond, freshwater marsh, freshwater prairie, pocosin, bay forest, bog, swamp forest, depression meadow, cypress savanna, and longleaf pine savanna communities, among others”. Other communities found within Carolina bays include *Pinus taeda* L. (loblolly pine) plantations, cropland, and open lakes (Carolina bay lakes).

Carolina bays can be divided into two classes based on soil substrate: clay-based bays and peat-based bays. The vast majority of Carolina bay literature has referenced peat-based bays, frequently using terms such as “pocosin” or “evergreen shrub bog” to describe the vegetation growing over deep organic soils. However, there are about 27 bays (as of 1982) located in the Carolinas that contain clay subsoil not overlain with sand or peat (Kelley and Batson 1955, Nifong 1982). These clay-based bays are restricted to Cumberland, Scotland, Hoke, and Robeson Counties in North Carolina. The vegetative physiognomy of clay-based bays differs from peat-based bays in that the structure is more open in the former (i.e., they have a sparse overstory of *Taxodium* and an herbaceous understory composed mostly of herbaceous taxa). However, clay-based bays do share some of the classical Carolina bay morphology features (e.g., elliptical boundaries, varying size, sand rims) with peat-based Carolina bays.

Clay-based bays are species-rich communities, often supporting rare taxa within their boundaries (Nifong 1982). Clay-based bays in high quality condition typically have an open canopy with a species-rich herbaceous understory. Fire and water level fluctuations are two disturbance regimes that account for the diversity found in these bays (Sutter and Kral 1994, Nifong 1998). Peat based bays are more prevalent throughout the Coastal Plain of the Carolinas. Peat-based bays are not as restricted to the inner Coastal Plain and are not as floristically rich as high quality clay-based bays.

Bladen County, North Carolina, is well-known for its many Carolina bays. Nifong (1998) found 617 Carolina bays within Bladen County; of these, 325 were classified as fully vegetated and 292 were classified as cleared (i.e., > 50% of their natural vegetation removed). Bladen County hosts the densest cluster of *unaltered* bays in the state (the county is fourth densest for bays in any condition). The majority of the bays in Bladen County are found in the Cape Fear River Valley, between the Cape Fear River and the South and Black Rivers. All of these bays are considered peat-based bays. Among extent Carolina bay lakes, all but one occur in Bladen County.

Carolina bays should not be confused with pocosins; they are two distinct physiographic features that just so happen to coexist with one another on the Atlantic Coastal Plain. These two landscape features differ from one another and using the terms synonymously is a common mistake among both laymen and professionals (Ross 2003). The term *pocosin* originated as an eastern Algonquian term meaning “*swamp-on-a-hill”* (Richardson 1983). It is defined by Ross (2003) as “a Coastal Plain wetland area of *variable shape and size* in an area of poor surface drainage whose vegetation is mostly broad-leafed evergreen shrubs and *Pinus serotina* Michx. growing on organic peaty soils” and by Brinson (1991) as “ecosystems dominated by woody, predominantly evergreen species and that normally occur on histosols (organic peat or muck soils ≥ 40 cm deep) or on soils with a histic epipedon (uppermost soil horizon used to classify a soil)”. Pocosins typically are located on broad, flat, interstream areas or near estuaries where rising sea levels affect their hydrology and hinder their drainage. Although there may be “pocosin-like vegetation” within a Carolina bay, the features are structurally of different origins. Unlike Carolina bays, the origin of pocosins is generally more understood (Whitehead 1972, Whitehead 1981, Brinson 1991, Richardson and Gibbons 1993).

Brinson (1991) attributed pocosin formation and subsequent persistence to two factors: climate and topography. Climate, he attested, “determines the exchange of matter and thermal energy between pocosins and the atmosphere”. The bulk of this exhange is in the form of precipitation, much of which is lost to evapotranspiration following its input. Brinson (1991) added “while the muted topographic relief of the Atlantic Coastal Plain is probably the main contributor to pocosin formation, the feedback between climate and topography is likely essential”. In summary, pocosins have formed in landscape positions with low topgraphic relief where the regional climate and lack of surficial hydrologic connections with adjacent wetland systems interact to form ombrotrophic conditions. Here, organic matter in the form of dead terrestrial vegetation is deposited onto wetland soils and accumlates at a slow, consistent rate through geologic time, resulting in the formation of pocosins.

Historically, the Atlantic and Gulf Coastal Plains supported a heterogeneous landscape of longleaf pine savannas, xeric sandhills, upland mixed-pine hardwoods, pocosins, Carolina bays, bottomland hardwood forests, natural lakes, and black and brown-water river systems (Garren 1943, Christensen 1999). However, it is now a highly fragmented and fire-suppressed region dominated by agriculture, residential developments, and large cities with few large intact parcels of natural ecosystems remaining. Demotechnic growth (Wetzel 2001, Dudgeon et al. 2006), global warming (Smith and Tirpak 1989), increasing agricultural production (Tilman et al. 2002), fire supression (Nowacki and Abrams 2008, Palmquist et al. 2014), urbanization (Terando et al. 2014), shoreline development (Radomski and Goeman 2001, Ford and Flaspohler 2010, Frost and Hicks 2012), and introduction of invasive species (Pimentel et al. 2005) continue to threaten and encroach upon the few “natural”, intact, terrestrial and freshwater ecosystems remaining in the Southeast, including Carolina bays and bay lakes.

Carolina bays are valuable components of our national and state natural heritage (Nifong 1998). Their variable hydrology and size, presence of rare and endemic taxa, and isolated landscape position, make them valuable habitats for southeastern flora and fauna and provide important ecosystem services (Suppl. material 4). Unfortunately, Carolina bays and other palustrine wetland systems have suffered from extensive habitat loss and degradation during the past three centuries (Bennett and Nelson 1991, Mitsch and Gosselink 1993, North Carolina Division of Environmental Management 1996, Kirkman et al. 1996, Nifong 1998). Using 1988 aerial imagery, Nifong (1998) found 8,057 Carolina bays in the state of North Carolina. Of these 8,057 total bays, 6,331 (79%) had more than half of their natural vegetation removed.

Sharitz and Gibbons (1982) and Nifong (1998) suggested several ways to better preserve and manage Carolina bays in the future. For an excellent review on the copious amount of Carolina bay literature available, see Ross (2000), Ross (2003); and for detail specifically about bays in the Carolinas, see Nifong (1998).

*Carolina Bay Lakes*

**Figure 2.**
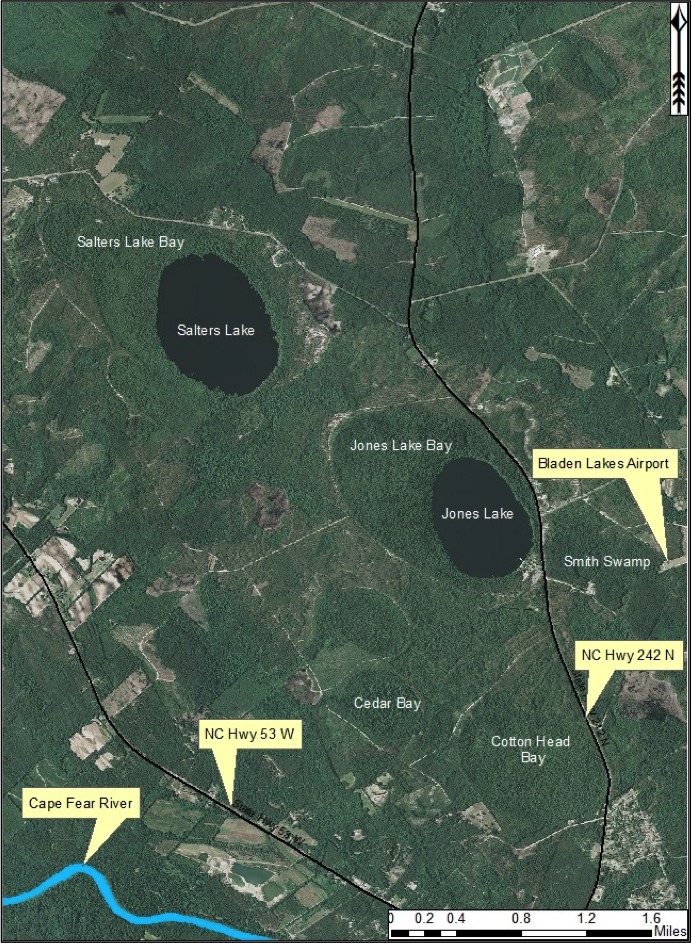
Position of Carolina bay lakes within Carolina bays. Carolina bay lakes are located in the southeasternmost portions of Carolina bays. The northern portions of the bays (i.e., the portion not inundated by lake waters) support shrub-bog plants over organic soils. Here, Salters (top left) and Jones (middle right) Lakes exemplify the typical bay lake position within Carolina bays. Aerial imagery, transportation, and hydrography layers obtained from NRCS Geospatial Data Gateway: https://gdg.sc.egov.usda.gov. Map produced by Nathan Howell using ArcGis Desktop: Version 10.2.2. (Environmental Systems Research Institute (ESRI) 2014).

Several Carolina bays in southeastern North Carolina contain large (i.e., > 50 hectares) natural lakes within their elliptic boundaries (Frey 1949), thereby giving them the name *Carolina bay lakes*. Each lake is located in the southernmost portion of the elliptical feature known as a Carolina bay (Fig. 2). The northern portions of the bays (i.e., the portions not inundated by lake waters) contain organic, peaty soils and a unique vegetative assemblage comprised of bay trees (*Gordonia lasianthus*, *Magnolia virginiana*, *Persea palustris)*, ericaceous shrubs (e.g., *Chamaedaphne calyculata* (L.) Moench, *Eubotrys racemosa* (L.) Nutt., *Kalmia* L., *Lyonia* Nutt., *Rhododendron* L., *Vaccinium* L., *Zenobia pulverulenta* (W. Bartram ex Willd.) Pollard), and several other species well-associated with nutrient-poor soils (e.g., *Chamaecyparis thyoides* (L.) Britton, Sterns & Poggenb., *Nyssa biflora* Walter, *Pinus serotina*, and *Smilax laurifolia* L.).

**Figure 3.**
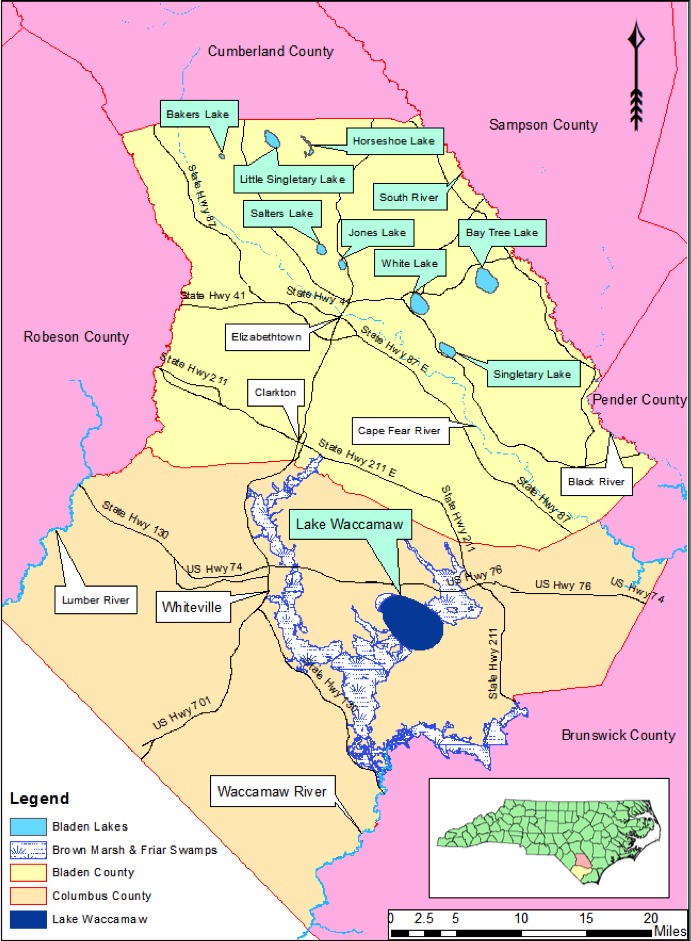
Geographic location of all nine Carolina bay lakes (green text boxes). Bladen County (light yellow) supports eight of the nine Carolina bay lakes known to exist; all eight lakes occur within the Cape Fear River Valley between the Cape Fear River and South River. Bay Tree Lake is the largest Carolina bay lake in Bladen County; the smallest is Bakers Lake. Lake Waccamaw is the largest Carolina bay and bay lake in North Carolina and is the only bay lake known to exist in Columbus County (tan). Baseline vector data obtained from NRCS Geospatial Data Gateway: https://gdg.sc.egov.usda.gov. Map Produced by Nathan Howell using ArcGis Desktop: Version 10.2.2. (Environmental Systems Research Institute (ESRI) 2014).

Nine Carolina bay lakes (i.e., Bakers Lake, Bay Tree Lake, Horseshoe Lake, Jones Lake, Lake Waccamaw, Little Singletary Lake, Salters Lake, Singletary Lake and White Lake) are known to exist within the known distribution of Carolina bays. All nine lakes occur in Bladen and Columbus counties, North Carolina (Frey 1949, LeBlond 1995, LeBlond and Grant 2005; Fig. 3). Carolina bay lakes, with the exception of Lake Waccamaw and White Lake, are nutrient poor because they receive the bulk of their hydrologic inputs in the form of precipitation. These lakes are also characteristically dystrophic due to the dominance of organic soils within their catchment area. Organic soils do not allow for the rapid decomposition of plant and animal matter, resulting in the high amount of humic substances found in the water column.

Although some Carolina bays may contain shallow marshes or ponds (Bennett and Nelson 1991, Nifong 1998), these are not considered *lakes.* There is no universally accepted technical definition that distinguishes a *lake* from a *pond* (Heinonen et al. 2008); however, it seems reasonable to accept as distinguishing that lakes have a clearly defined littoral and profundal zone, a larger overall size (>8 hectares), a shoreline exposed to wave dynamics, greater water depth, a mixing of the water column by wind induced turbulence, and the ability to retain the bulk of their water volume even in years of drought (Cowardin et al. 1979, Moss et al. 1996, Williams et al. 2004, Biggs et al. 2005, Brönmark and Hansson 2005).

Carolina bays are considered to be geographically isolated wetlands with their primary water source coming directly from precipitation (Sharitz 2003, Tiner 2003). Although the vast majority of Carolina bays lack surface water connections to outside aquatic systems, Carolina bay *lakes* are an exception. Carolina bay lakes all contain drainage outlets--usually along their southern shorelines, but in the northwest for White Lake (Frey 1949)--that release excess water into the Cape Fear and Waccamaw River drainages during periods of high precipitation. However, during years of scarce rainfall, these lakes are more or less isolated from surrounding lotic systems and are confined to their basins (N. Howell, pers. obs.).

***Lacustrine Zonation*** (derived from Wetzel 2001)

**Figure 4.**
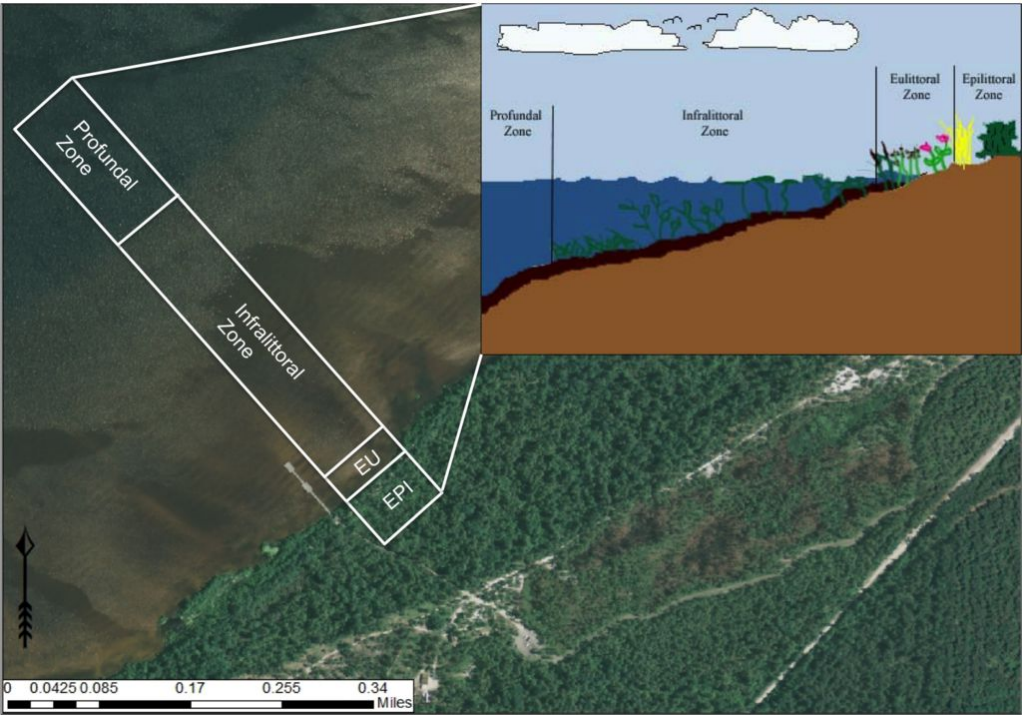
Lacustrine zonation. EPI = epilittoral zone, EU = eulittoral zone. Aerial imagery obtained from NRCS Geospatial Data Gateway: https://gdg.sc.egov.usda.gov. Map Produced by Nathan Howell using ArcGis Desktop: Version 10.2.2. (Environmental Systems Research Institute (ESRI) 2014). Illustration (top right) by Nathan Howell.

Lakes, including Carolina bay lakes, can be divided into distinct transitional zones, moving from the shoreline to the center of the lake (Fig. 4).

(1) *Epilittoral zone*: The zone that lies entirely above the lake surface and is not influenced by the spray of surf. This zone can be thought of as the terrestrial or upland zone; the highest water levels never reach it and it is not affected by lakeshore dynamics or hydrology.

(2) *Supralittoral zone*: The zone that lies entirely above the lake surface and is influenced by the spray of the surf.

(3) *Eulittoral zone*: The zone encompassing the entire region of the shoreline from the highest and lowest seasonal water levels. This zone experiences natural disturbances such as water level fluctuations and wave dynamics.

(4) *Infralittoral zone*: This zone is subdivided into three zones in relation to the occurrence and distribution of the major classes of aquatic macrophytes: *upper infralittoral* zone where emergent rooted macrophytes persist; *middle infralittoral* zone where floating-leaved rooted macrophytes occur; and *lower infralittoral* zone where submersed-rooted, adnate, or free-floating macrophytes occur. The eulittoral and infralittoral zones collectively constitute the littoral zone.

(5) *Littoriprofundal zone*: The zone occupied by photosynthetic algae and bacteria, often associated with the metalimnion (i.e., the stratum between the epilimnion and hypolimnion representing a marked thermal change; also synonymous with thermocline) of stratified lakes.

(6) *Profundal zone*: The zone that consists of the remainder of the vegetation free sediments.

*The Littoral Zone*

The littoral zone of lakes (i.e., the eulittoral and infralittoral zones) is an important transition zone between adjacent uplands and the deeper pelagic area of the lake. This zone contains vascular macrophytes (i.e., aquatic vascular plants large enough to see with the naked eye) that have evolved from their terrestrial ancestors to cope with the physical and physiological demands of persisting in an aquatic environment (Sculthorpe 1967, Wetzel 2001, Brönmark and Hansson 2005, Keddy 2010). The vascular macrophytes and coarse woody debris that exist in this zone provide critical habitat for zooplankton, photosynthetic and heterotrophic microflora, macroinvertebrates, herpetofauna, avifauna, fish, and mammals (Brusnyk and Gilbert 1983, Pieczynska 1990, North Carolina Division of Environmental Management 1996, Wetzel 2001, Keddy 2010, Ewert et al. 2011). The littoral zone is characterized by having high productivity, including some of the highest rates of organic matter synthesis in the biosphere (Wetzel 2001).

***Aquatic Macrophytes*** (derived from Wetzel 2001)

Aquatic macrophytes may be divided into four classes. Moving from the shoreline out to deeper water, these classes are as follows [taxa vouchered or reported from Carolina bay lakes are indicated by ^c^]:

(1) *Emergent macrophytes*: Species rooted in saturated and inundated soils with a water depth up to 1.5 meters; root systems remain in anoxic soil conditions while leaves and reproductive organs stay above the water surface. These plants are often rhizomatous, stoloniferous, or cormous with the potential to reproduce asexually. Heterophyllous (i.e., when a plant exhibits vegetative polymorphism, having morphologically different submersed and aerial organs) species may also be emergent. Examples of genera that may be grouped in this category include *Carex* L.^c^, *Cephalanthus* L.^c^, *Cladium* P. Browne^c^, *Juncus* L.^c^, *Panicum* L.^c^, *Pontederia* L.^c^, *Rhynchospora* Vahl^c^, *Scirpus* L.^c^, and *Typha* L.

(2) *Floating-leaved macrophytes*: Species rooted in the substratum with floating leaves attached to long flexible petioles or on short petioles attached to an ascending stem.

Submersed leaves precede the floating leaves in heterophyllous species. Reproductive organs remain atop or above the water surface. Examples of genera grouped into this category include *Brasenia* Schreb.^c^, *Nelumbo* Adans.^c^, *Nuphar* Sm.^c^, *Nymphaea* L.^c^, *Nymphoides* Ség.^c^, and *Potamogeton* L^c^.

(3) *Submersed macrophytes*: Species that remain completely submersed in the water column, but are rooted to the substratum. Leaf morphology is highly variable in this group, from finely dissected to very broad, and reproductive organs may be emersed, floating, or submersed. Examples of genera included in this group are *Ceratophyllum* L., *Isoetes* L., and *Myriophyllum* L^c^.

(4) *Freely floating macrophytes*: Species that remain unattached to the substratum and are completely dependent upon the nutrients in the water column for survival. Reproductive organs may be floating or aerial. Examples of genera include *Azolla* Lam., *Eichhornia* Kunth, *Hydrocharis* L., *Limnobium* Rich., *Trapa* L., and *Utricularia* L^c^.

***Factors affecting Aquatic Macrophyte Richness in Lakes***

Lacoul and Freedman (2006) provided a thorough review on how various environmental influences affect aquatic plants in freshwater systems. A few of these environmental factors are reviewed below.

*Latitude*

It is well known that generally the number of species occuring at the equator greatly exceeds that of the temperate and northern latitudes (Edmonds 1997). Although this general rule applies across most groups of taxa, it does not seem to apply to aquatic plants. Crow (1993) found that aquatic plants are more diverse in temperate rather than tropical latitudes. When comparing temperate wetland floras to those of tropical climes, this pattern is reinforced (Stuckey 1975, Henry and Scott 1984, Peet and Allard 1993, Ruch et al. 2009). Because Carolina bay lakes differ little in latitude, this factor does not significantly affect species richness in these systems.

*pH and Alkalinity*

Peat-based Carolina bays are known to have acidic (< 7 pH), nutrient poor, organic soils (Daniels et al. 1984, Leab 1990, Newman and Schalles 1990). In many respects, these isolated wetlands of the Southeast are quite similar to the peatlands of the northern United States and Canada. Floristic diversity in peatlands has been shown to increase with increased levels of calcium and alkalinity in the groundwater (Glaser et al. 1990, Vitt and Chee 1990). Similarly, aquatic macrophyte richness of lakes tends to be lower in unproductive lakes with low pH (e.g., Carolina bay lakes) and higher in more productive lakes with higher alkalinities (Roelofs 1983, Roberts et al. 1985, Rørslett 1991, Dodson et al. 2000, Vestergaard and Sand-Jensen 2000, Søndergaard et al. 2005).

*Water Color*

Waters with increased levels of humic substances are typically, dystrophic, acidic, and tea-stained. Tea-stained waters are not as transparent as lakes with low humic substances, thus humic lakes have a shallow euphotic zone and a narrow littoral zone, reducing the abundance and depth at which aquatic macrophytes may grow (Spence 1982). Vestergaard and Sand-Jensen (2000) also saw decreased richness in aquatic macrophytes when water transparency was low. An excellent example of how increased humic substances affect water transparency and macrophyte richness and composition can be seen when comparing White Lake to the other Carolina bay lakes. White Lake is an oligotrophic lake with transparent water due to the presence of natural springs on the lake floor. Secchi depths commonly reach to the bottom of the lake (3m/10 ft) and submerged aquatic macrophytes are able to colonize the deepest portions of the lake with ease (i.e., the euphotic zone is deep compared to the other bay lakes).

*Hydrography*

**Figure 5.**
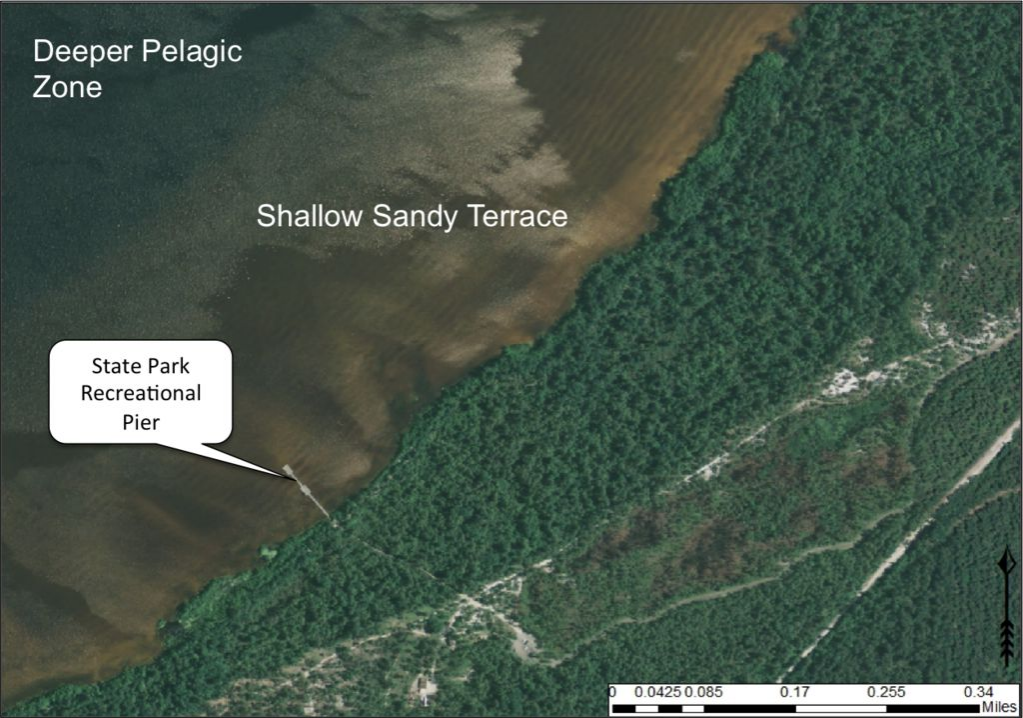
Broad, shallow, sandy terrace along Lake Waccamaw’s southern shoreline. The gentle relief of this terrace creates a wide littoral zone. Wide littoral zones are more floristically diverse and contain more available area for the establishment of aquatic macrophytes. Alternatively, narrow littoral zones do not have much area for the establishment of aquatic macrophytes and are species-poor. Aerial imagery obtained from NRCS Geospatial Data Gateway: https://gdg.sc.egov.usda.gov. Map produced by Nathan Howell using ArcGis Desktop: Version 10.2.2. (Environmental Systems Research Institute (ESRI) 2014).

Frey (1949) documented the morphometry and hydrography of the Carolina bay lakes and determined that the southern portions of the lakes possessed a gentle, tapering hydrography while the northern portions possessed a steep hydrography. Floristic inventories by the first author confirm that aquatic macrophyte richness is higher along southern shorelines; so much so, that the surveying of northern shorelines was abandoned early in the life of the project. A broad sandy terrace occurring along the southern shore of Lake Waccamaw (Fig. 5) creates a wide littoral zone compared to other Carolina bay lakes. This stretch of shoreline, with its gentle hydrography, is known to support over 140 species of wetland plants, while the Bladen lakes, with their comparatively steeper hydrography, are known to support < 55 wetland plant taxa (see floristic summary).

*Lake Size*

As a general rule, species richness usually increases with increasing area (Arrhenius 1921, Williams 1964, Connor and McCoy 1979, Rosenzweig and M.L. 1995, Søndergaard et al. 2005). Findlay and Houlahan (1997) found that species richness increased with area sampled for birds, mammals, hepertofauna, and plants in southeastern Ontario wetlands. Results from this work also support these findings with Bakers Lake (i.e., the smallest bay lake) supporting the least diverse littoral zone flora and Lake Waccamaw (i.e., the largest bay lake) supporting the most species-rich littoral zone flora. Other large natural lakes of North Carolina Coastal Plain (e.g., Lake Phelps, Lake Mattamuskeet, Lake Waccamaw) are known to support diverse shoreline floras, more so than the smaller lakes of the region (Lynch and Peacock 1982, Schafale 2012; N. Howell, pers. obs.).

*Water Level Variation, Disturbance, and Soil Fertility*

Keddy and Fraser (2000) summarized factors that govern littoral zone diversity irrespective of geographic location or size. Three environmental factors (i.e., water levels, soil fertility, and disturbance) govern the composition and floral diversity of littoral zones. Shorelines exposed to intermediate levels of natural disturbances will support a richer flora than those experiencing little to no disturbances and those experiencing extremely harsh disturbances. Natural disturbances may include wave action, ice scour, water level fluctuations, fire, or grazing. If water level fluctuations were absent from a lake or similar waterbody (e.g., in a permanently impounded pond), a two-staged littoral zone would result, with aquatic macrophytes in the aquatic zone and shrubs and trees in the terrestrial zone. Under long-term water level fluctuations, a multi-staged littoral zone would result, leading to increased heterogeneity and a richer flora. Keddy and Fraser (2000) attested that “simply changing water levels from one year to the next doubles the number of vegetation types”. Rørslett (1991) observed that northern European lakes experiencing water level fluctuations of 1–2 meters per year showed greater macrophyte richness than sites experiencing little or intense disturbances. Carolina bay lakes historically would have experienced long-term water level fluctuations, but the installation of water control structures (i.e., dams) in some of the lakes outlet channels has resulted in more stabilized systems (N. Howell, pers. obs.).

Shorelines exposed to frequent disturbances typically have silt and clay stripped from them; and consequently, contain few nutrients. Sheltered shorelines receive clay and silt deposits and therefore contain a higher nutrient content. Foreshores will have a distinct vegetative community characterized as having low biomass and rare species, while backshores (bays or backwater areas sheltered from disturbance) will support a higher biomass community composed of a few clonal dominants (Keddy 2010). Macrophyte richness is always higher in areas of intermediate disturbance. Eutrophification of littoral zones causes increased soil fertility, which increases biomass and negatively impacts macrophyte richness and rare plant taxa.

### Study Sites

***Bakers Lake***

**Figure 6.**
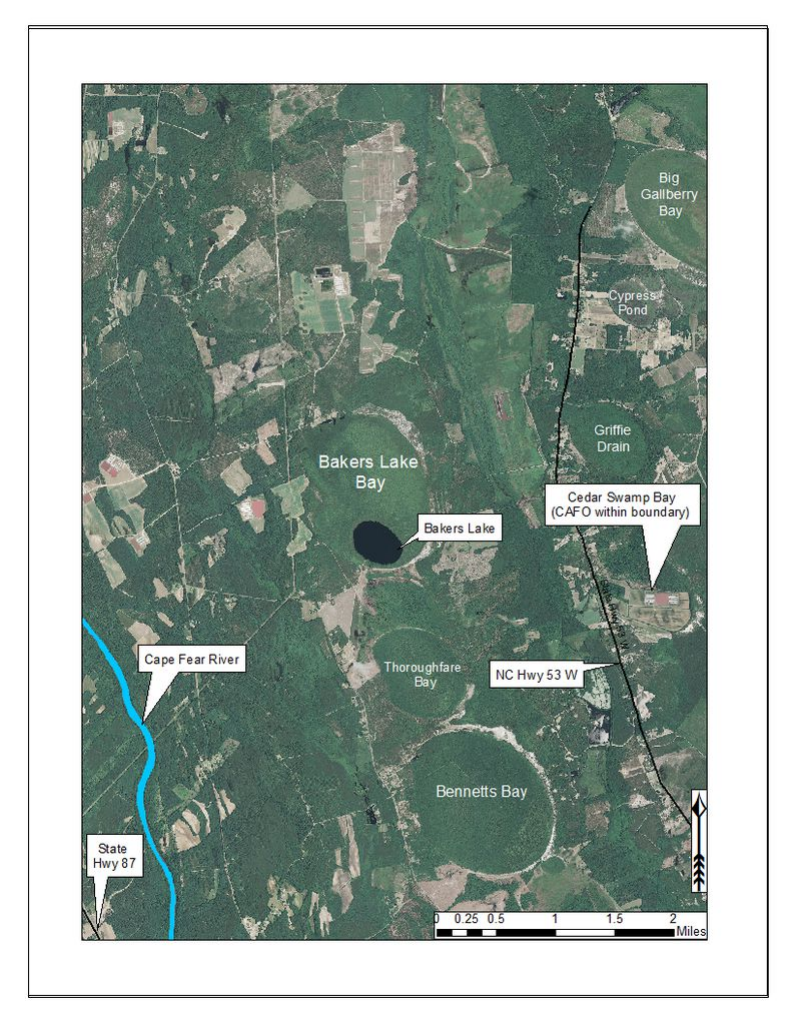
Bakers Lake and surrounding lands. Bakers Lake is located in northern Bladen County and is surrounded by a mix of agriculture and forestland. Aerial imagery, transportation, and hydrography layers obtained from NRCS Geospatial Data Gateway: https://gdg.sc.egov.usda.gov. Map produced by Nathan Howell using ArcGis Desktop: Version 10.2.2. (Environmental Systems Research Institute (ESRI) 2014).

**Figure 7.**
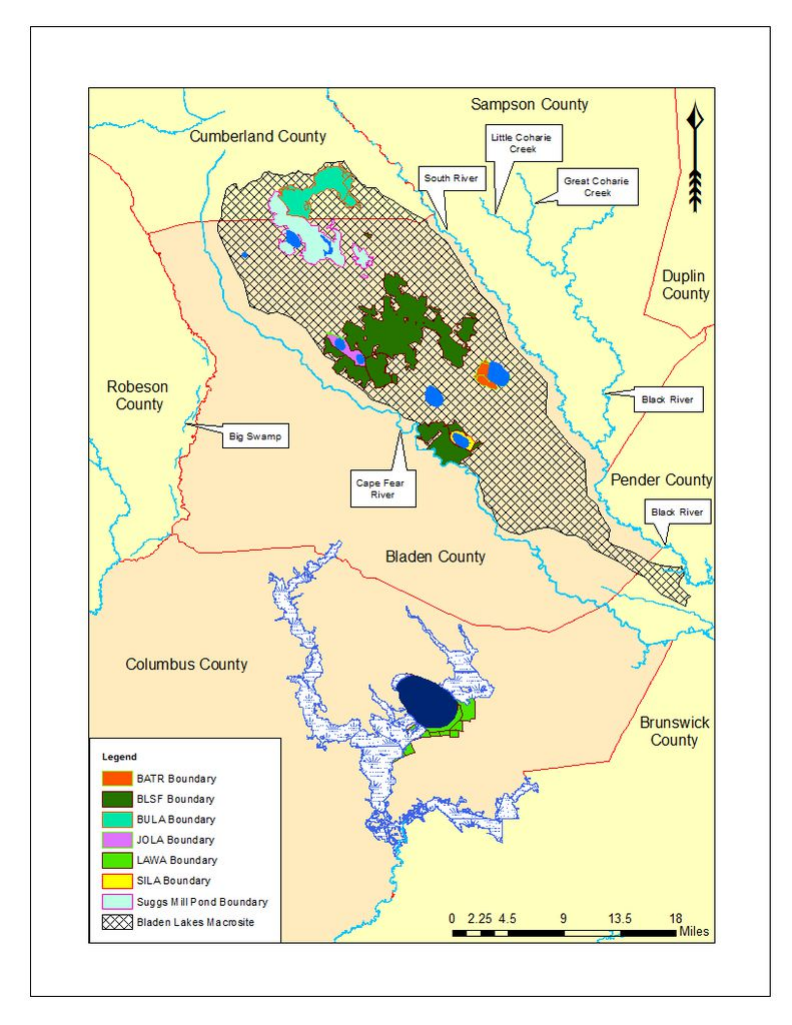
Bladen Lakes Macrosite (vector). The Bladen Lakes Macrosite (hatched pattern) is a large area encompassing parts of southern Cumberland County, eastern Bladen County, and northwest Pender County. Historically, macrosites were established by the North Carolina Natural Heritage Program (NCNHP) in efforts to identify large, intact, natural areas that withheld numerous other smaller natural areas within their boundaries. The NCNHP no longer uses macrosites as viable natural area boundaries, but it is useful to show the extent of the Bladen Lakes Macrosite boundary. When moving from north to south, the lands are as follows: Bushy Lake State Natural Area (teal green), Suggs Mill Pond Gameland (light mint green), Bladen Lakes State Forest (forest green), Jones Lake State Park (pink), Bay Tree Lake State Park (orange), and Singletary Lake State Park (yellow). Lake Waccamaw State Park (neon green) can be seen farther south along with Friar and Brown Marsh Swamps in Columbus County. Baseline vector data obtained from NRCS Geospatial Data Gateway: https://gdg.sc.egov.usda.gov. Map produced by Nathan Howell using ArcGis Desktop: Version 10.2.2. (Environmental Systems Research Institute (ESRI) 2014).

**Figure 8.**
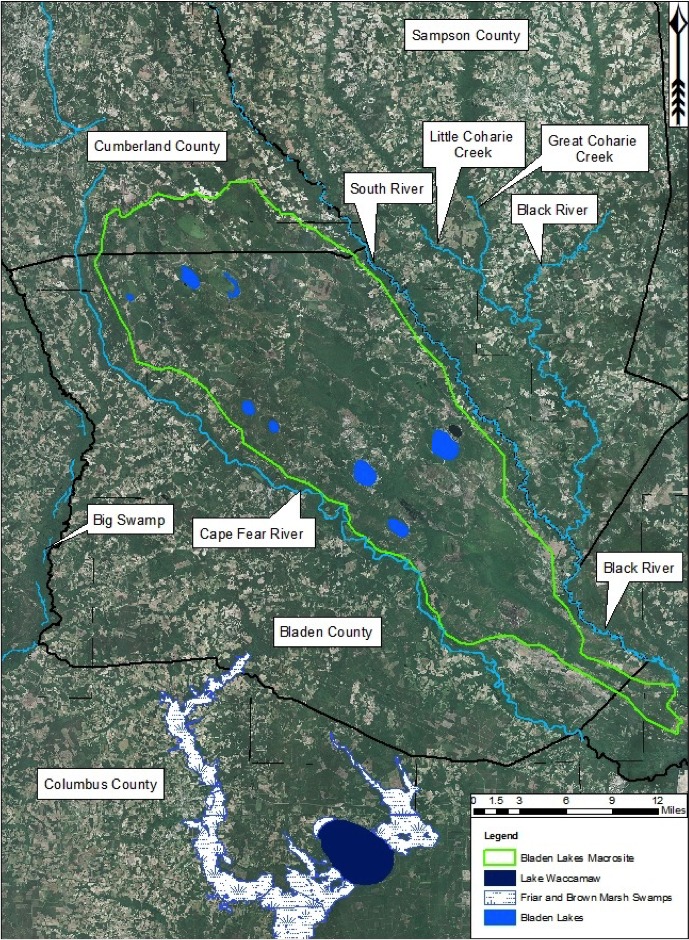
Bladen Lakes Macrosite (ortho). The North Carolina Natural Heritage Program no longer uses macrosites as viable natural area boundaries, but here it is useful to show the extent of the Bladen Lakes Macrosite boundary. Note the large areas of fragmented land surrounding the macrosite and the relatively unfragmented land within the boundaries of the macrosite. This large tract of land contains one of the largest remaining portions of intact unaltered Carolina bay complexes known to exist. Aerial imagery, transportation, and hydrography layers obtained from NRCS Geospatial Data Gateway: https://gdg.sc.egov.usda.gov. Map produced by Nathan Howell using ArcGis Desktop: Version 10.2.2. (Environmental Systems Research Institute (ESRI) 2014).

Bakers Lake (30.35 hectares; 75 acres) is a small, privately owned, Carolina bay lake, located in northwestern Bladen County between Little Singletary Lake and the Cape Fear River north of Thoroughfare Bay, ca. 1.5−2 miles east of the intersection of SR 1318 (Old River Road) and SR 1320 (Middle Road; LeBlond and Grant 2005; Fig. 6). This site is located along the northwest boundary of the Bladen Lakes Macrosite, a large tract of undeveloped and relatively unfragmented land between the Cape Fear, South, and Black River systems (LeBlond and Grant 2005; Figs 7, 8). The macrosite extends from southern Cumberland County, through Bladen County, and into southwestern Pender County. This large area is given the name “macrosite” because it contains numerous “standard sites” (i.e., smaller tracts of land with high ecological integrity) that are strongly geographically associated with one another. The majority of the macrosite is located in Bladen County and contains the largest concentration of unaltered, intact, Carolina bays.

Dr. Clemuel Johnson and wife Nancy Johnson, of Elizabethtown, have owned Bakers Lake and surrounding lands (451.40 hectares; 1,155.45 acres) since 1980. Prior to the Johnson’s ownership, Agnes Holden Williams owned the lake and surrounding lands. Ms. Williams’ father acquired the land from an unknown seller during the early 20^th^ century. This seller was able to successfully purchase the lake before 1929, when North Carolina legislation mandated that all lakes greater than 50 acres in size be made property of the state.

Bakers Lake forms the headwaters of Phillips Creek, which drains southward into the Cape Fear River. Bakers Lake Natural Area (i.e., Bakers Lake bay and immediate surrounding lands) is known to support five natural community types (i.e., Pond Pine Woodland – Typic Subtype (S3,G3), Peatland Atlantic White Cedar Forest (S1,G2), Low Pocosin – Gallberry/Fetterbush Subtype (S2,G2), Sand Barren – Typic Subtype (S2,G2), and Natural Lake Shoreline – Cypress Subtype (S2,G3; LeBlond and Grant 2005). Bakers Lake has been known to support heron rookeries and small populations of the state rare Anhinga (*Anhinga anhinga* [W2; S3B, G5]; LeGrand et al. 2014) during the spring and summer months (S. Clark, pers. comm.; N. Howell, pers. obs.). In addition, the site provides important stopover habitat for large flocks of migrating waterfowl (e.g., *Aix sponsa* [Wood Duck], *Anas americana* [American Widgeon], *Anas clypeata* [Northern Shoveler], *Anas crecca* [Green-winged Teal], *Anas discors* [Blue-winged Teal], *Anas platyrhynchos* [Mallard], *Anas strepera* [Gadwall], *Aythya collaris* [Ring-necked Duck], *Aythya valisineria* [Canvasback], *Branta canadensis* [Canada Goose], *Bucephela albeola* [Bufflehead], *Lyphodytes cucullatus* [Hooded Merganser], *Oxyura jamaicensis* [Ruddy Duck; G. German and S. Clark, pers. comm; N. Howell pers. obs.).

Anthropogenic disturbances (i.e., silvicultural practices, dam installation in the outflow channel, agricultural fields, confined animal feeding operations (CAFOs), fire supression, and rural residential development) have either been documented on site or on adjacent properties (LeBlond and Grant 2005; S. Clark, pers. comm.). These disturbances have lowered the integrity of several of the aforementioned natural community types within and adjacent to Bakers Lake Natural Area (N. Howell, pers. obs.), but restoration potential is still relatively high. The installation of a flashboard riser system in the outflow channel has altered the natural hydrology of the lake and caused natural water level fluctuations to essentially cease. Following the installation of the dam, the lake consistently stays at a high level, thus narrowing the littoral zone and forcing aquatic macrophytes to occur at or just below the maximum annual high water mark (N. Howell, pers. obs.).

The water quality of Baker’s Lake has not been formally tested by state agencies, but appears high in humic substances (N. Howell, pers. obs.) and the chemistry is likely similar to that of the other Bladen lakes. The lake is here considered dystrophic and relatively unproductive.

***Bay Tree Lake***

**Figure 9.**
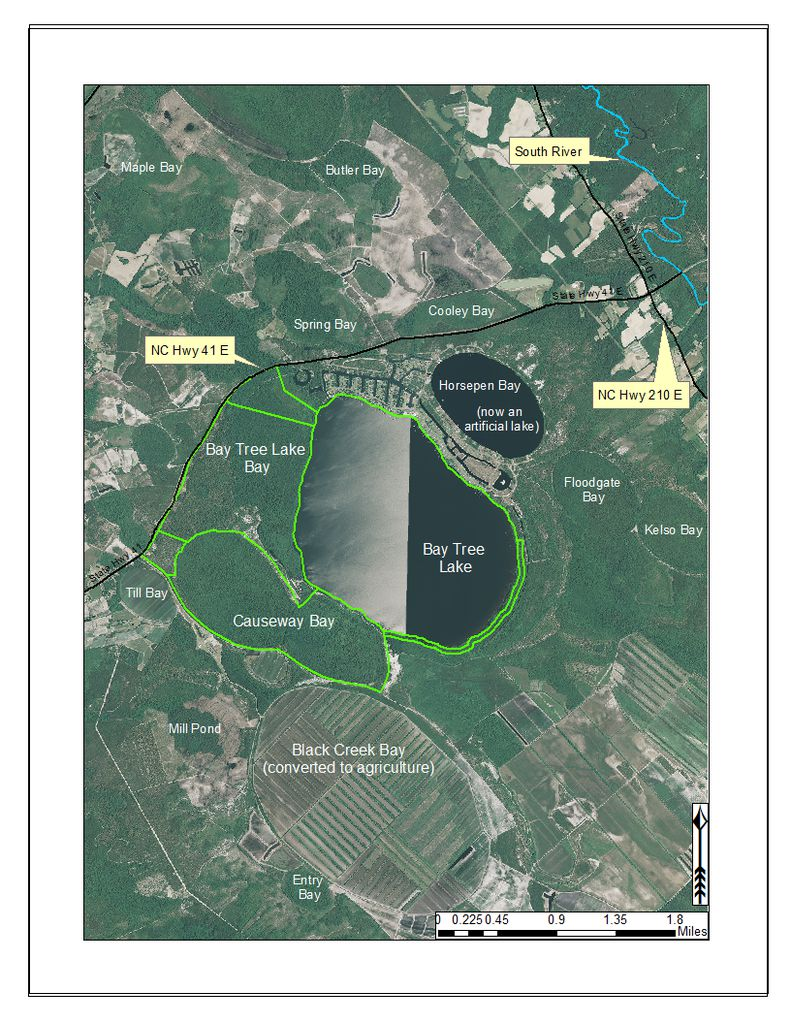
Bay Tree Lake State Park (highlighted in green) and surrounding lands. Lands surrounding Bay Tree Lake State Park to the south are privately owned and have been partially converted to agriculture. Black Creek Bay and several others in the vicinity have been cleared of their original vegetation and converted to agriculture (primarily blueberry farms in this area). Historically, Horsepen Bay was a peat-filled Carolina bay. During the development of the residential community seen along the northeast shoreline (Bay Tree Resorts), it was turned into a body of open water. Aerial imagery, transportation, and hydrography layers obtained from NRCS Geospatial Data Gateway: https://gdg.sc.egov.usda.gov. Map produced by Nathan Howell using ArcGis Desktop: Version 10.2.2. (Environmental Systems Research Institute (ESRI) 2014).

Bay Tree Lake (formerly Black Lake; 588.81 hectares; 1,455 acres) is a large, state-owned Carolina bay lake, located in east-central Bladen County along NC Hwy 41 east of White Lake and west of NC Hwy 210. Bay Tree Lake is part of Bay Tree Lake State Park, a 1,006.85 hectare (2,488 acre) park that includes Bay Tree Lake bay and large parcels of land lying to the north and west of Bay Tree Lake (Fig. 9).

The North Carolina General Assembly passed legislation in 1911 confirming the status of Bay Tree Lake as a state-owned public trust resource (North Carolina Division of Parks and Recreation, Planning and Development Section 2006b). Historically, Bay Tree Lake was not included within the original natural area site boundary determined by the North Carolina Natural Heritage Program (NCNHP) due to high levels of shoreline disturbance. Today, the lake is considered part of the natural area due to the presence of three rare dragonflies (*Gomphus australis* [Clearlake Clubtail], *Gomphus cavillaris brimleyi* [Brimley’s “Sandhill” Clubtail], and *Progomphus bellei* [Belle’s Sanddragon]) that utilize the lake throughout their life cycle.

In January 1965, a private land development group had the option to purchase 5,665.59 hectares (14,000 acres) of land surrounding Bay Tree Lake with the intent of creating an inland resort community (North Carolina Division of Parks and Recreation, Planning and Development Section 1996a). Later that year, a proposal was constructed and sent to the North Carolina Department of Conservation and Development concerning the drainage of Bay Tree Lake. The purpose for draining the lake was to improve the quality of the water and lake bottom for recreational purposes (e.g., swimming and boating). Permission to lower lake levels 4 feet was granted in 1965 and in January of 1966, the development group made a request to completely drain the lake where peat deposits and debris could be taken from the lake bottom (North Carolina Division of Parks and Recreation, Planning and Development Section 1996a).

The purpose of the drainage project was to release tannic, tea-colored, waters from the lake and divert all incoming tannic waters from a northerly adjacent swamp to below the outflow channel. Drainage of the lake was completed in the winter of 1966. The lake remained dry for 5 years while developers removed debris and peat deposits and imported large quantities of white sand, which would later be distributed around the entirety of the lakeshore. In 1970, the lakes outflow channel was plugged and the lake began to refill (North Carolina Division of Parks and Recreation, Planning and Development Section 1996a). After two years, the lake had nearly reached its original water levels. Shortly after residential lots went for sale, a breach of the lake rim occurred and tea-stained waters were allowed to re-enter the lake. The breach was plugged within 24 hours, but the lake had already returned to its original dystrophic condition (North Carolina Division of Parks and Recreation, Planning and Development Section 1996a). The lake has not been significantly altered since and remains in a dystrophic condition to this day.

Bay Tree Lake State Park contains five natural community types (Mesic Pine Savanna – Coastal Plain Subtype [S2,G2G3]; Sand Barren – Typic Subtype [S2,G2]; Small Depression Drawdown Meadow – Typic Subtype [S2S3,G2?]; Small Depression Pocosin – Blueberry Subtype [S2,G3?]; and Xeric Sandhill Scrub – Typic Subtype [S3S4,G3?]. A Natural Lake Shoreline community was not assigned to Bay Tree Lake by the NCNHP due to the shoreline’s disturbance history. The present authors agree with this determination and have chosen not to assign a natural lake shoreline community to this site. However, it is worth noting that the shoreline flora of Bay Tree Lake differs only slightly from the other Bladen Lakes.

Bay Tree Lake forms the headwaters of Lake Creek, a small blackwater creek that drains southeast to the South River (the boundary between Bladen and Sampson counties). Much of the land surounding Bay Tree Lake State Park has been cleared for agriculture (particularly blueberry farms) and has limited the landscape connectivity between it and other intact natural areas. Several bay complexes occur in the immediate vicinity of Bay Tree Lake including Beagle Bay, Black Creek Bay, Causeway Bay, Cooley Bay, Horsepen Bay (now an artificially created lake/pond), Floodgate Bay, Kelso Bay, and Spring Bay. A residential resort community is located along the north and east shorelines of the lake. The boundaries of this community have continued to extend around the east and southeast shorelines. Residential development, agricultural expansion, severe offroad vehicle use, and fire supression are the primary threats to biological diversity within and around Bay Tree Lake State Park (N. Howell pers. obs.). Available water quality parameters for Bay Tree Lake are provided in Table 1.

***Horseshoe Lake***

**Figure 10.**
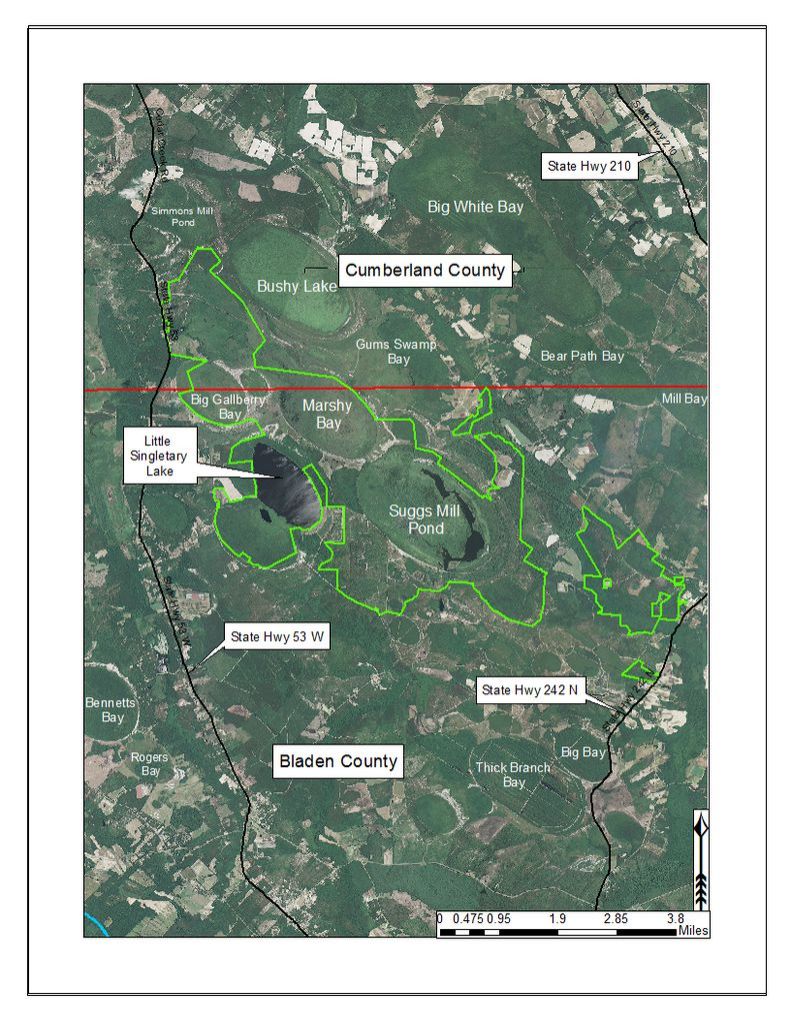
Suggs Mill Pond Game Land (outlined in green) and surrounding lands. Lands north of the red dividing line occur in Cumberland County while lands south of the red line occur in Bladen County. Suggs Mill Pond Game Land contains two large bay lakes within its boundary. Little Singletary Lake is located along the western boundary of the property and Horseshoe Lake (aka Suggs Mill Pond) is located in the center of the property. Aerial imagery, transportation, and hydrography layers obtained from NRCS Geospatial Data Gateway: https://gdg.sc.egov.usda.gov. Map produced by Nathan Howell using ArcGis Desktop: Version 10.2.2. (Environmental Systems Research Institute (ESRI) 2014).

Horseshoe Lake (also known as Suggs Mill Pond; 109 hectares; ca. 270 acres) is an irregularly shaped Carolina bay lake located in northern Bladen County south of Bushy Lake State Natural Area, east of Little Singletary Lake, north of SR 1325 (Gum Springs Rd), and west of SR 1002 (Old Fayetteville Rd). Horseshoe Lake is one of two Carolina bay lakes within Suggs Mill Pond Game Land (4469.34 hectares; 11,044 acres; Fig. 10), the other being Little Singletary Lake. Suggs Mill Pond Game Land is owned by the State of North Carolina and the North Carolina Wildlife Resources Commission (NCWRC) and is located in northern Bladen County and southern Cumberland County. This game land is located in the northwestern portion of the Bladen Lakes Macrosite and contains one of the largest remaining examples of unaltered Carolina bay complexes.

The state first gained rights to the property in 1994 when a 62-acre (25 ha) parcel was donated to the NCWRC from Canal Woods Industries. Thereafter, much of the remaining property was purchased from Canal Woods. The fact that Horseshoe Lake and Little Singletary lake were not owned by the state of North Carolina until the mid-1990s suggests that these lakes were involved in a similar ownership situation as Bakers Lake (i.e., these lakes must have been privately owned prior to 1929 when legislation mandated that all lakes greater than 50 acres (20.2 ha) in size be released to the state of North Carolina). Suggs Mill Pond Game Land is one of four North Carolina game lands enrolled in the Cooperative Upland habitat Restoration and Enhancement program (CURE), where management for early successional habitat is the top priority (Allen et al. 2015). Traditionally, hunters and fishermen were primary users of Suggs Mill Pond Game Land, but an increasing number of non-traditional users (i.e., birders, canoers, hikers, photographers, and researchers) visit the site regularly.

The largest bay on site contains a horsehoe-shaped artificial impoundment (Horseshoe Lake). Horseshoe Lake forms the headwaters of Ellis Creek, which drains southwest to the Cape Fear River. Although an old milldam currently maintains Horseshoe Lake, it is thought that a smaller body of open water may have been present prior to the dam’s installation in the late 19^th^ or early 20^th^ centuries. Horseshoe Lake was formed subsequent to the dam installation, as water levels began to rise into the peat-filled Carolina bay. Today, it is best described as a semi-permanent impoundment; however, the presence of floating bogs within the lake makes it unique from other semi-permanent impoundments in North Carolina. Parts of the lake support patches of the rare floating bog community (the largest extent known from the state), which is dominated by sedges, orchids, carnivorous plants, and ericaceous shrubs. Other portions comprise the Coastal Plain Semipermanent Impoundment community, which is characterized by open water, dominated by floating-leaved macrophytes, and a sparse overstory of *Taxodium ascendens* Brongn.

The floating bog community type is quite unique. Manifestations of this community type occur just above the water surface and range in size from ca. 10 × 10 m to a few hectares in size (N. Howell, pers. obs.). Some bogs may contain well-developed herbaceous vegetation in addition to small (e.g., < 3 m tall) trees of *Chamaecyparis thyoides*, *Nyssa biflora*, and *Taxodium ascendens*, while others contain a strictly herbaceous component. Exposed portions of peat can be seen around the peripheries of some bogs; here, *Drosera intermedia* Hayne, *Eleocharis baldwinii* (Torr.) Chapm. */E. vivipara* Link, *Pogonia ophioglossoides* (L.) Ker Gawl, *Utricularia striata* Leconte ex Torr., *Utricularia purpurea* Walter, and other small-statured herbaceous plants can be seen colonizing the apparently young peat formations. Isolated floating bogs (i.e., bogs surrounded by open water and separated from adjacent bogs and upland habitats) of varying size show a consistent zonation pattern. Small statured herbaceous taxa colonize the outer periphery and are slowly replaced by larger herbaceous taxa (*Andropogon glaucopsis*, *Dulichium arundinaceum* (L.) Britton, *Hypericum virginicum* L., *Rhexia nashii* Small, *Rhynchospora alba* (L.) Vahl, *Rhynchospora inundata* (Oakes) Fernald, *Xyris fimbriata* Elliott, and *Xyris smalliana* Nash) and woody species (*Acer rubrum* L., *Chamaecyparis thyiodes*, *Decodon verticillatus* (L.) Elliott, *Nyssa biflora*, and *Taxodium ascendens*) when moving toward the center. Thus, a dome-shaped appearance is typically seen.

Few examples of floating bogs or mats of vegetation are known to science. The floating peat mats of New Hampshire are most similar to those of Horseshoe Lake. These peat mats possess the same general structure and abiotic conditions as those of Horseshoe Lake and are known to contain several overlapping taxa, inculding *Drosera intermedia*, *Dulichium arundinaceum*, *Eleocharis* R. Br. spp., *Hypericum virginicum*, *Nymphaea odorata* W.T. Aiton, *Rhynchospora alba*, and *Utricularia* spp. (New Hampshire Division of Forests and Lands 2015).

A separate but similar case of floating vegetation mats, forming as a result of dam installation, has been observed at Goose Creek Reservoir in South Carolina (Hunt 1943). In 1933, a dam was installed on Goose Creek, ca. 12 miles north of Charleston, subsequently flooding historic rice plantations that had reverted to brackish marsh vegetation. Hunt (1943) described the zonation (looking across to the center of the mat from the outer periphery) of a typical floating mat as follows: (1) pioneer zone (i.e., the outer margins of the mats): *Alternanthera philoxeroides* (Mart.) Griseb., *Bidens laevis* (L.) Britton, Sterns & Poggenb., *Boehmeria cylindrica* (L.) Sw., *Habenaria repens*, *Hydrocotyle ranunculoides* L.f., *Persicaria glabra* (Willd.) M. Gómez, and *Sacciolepis striata* (L.) Nash, (2) the cat-tail/shrub zone: *Kosteletzkya pentacarpos* (L.) Ledeb., *Typha latifolia* L., and *Salix nigra* Marshall, and (3) the main body: *Acer rubrum*, *Apios americana* Medik., *Decodon verticillatus*, *Mikania scandens* (L.) Willd., Panicum virgatum L. var. virgatum, *Persea palustris*, *Rubus* L. spp., and *Taxodium distichum* (L.) Rich.

The floating “sudd” vegetation of the upper Nile River is also somewhat similar, forming large floating mats of marsh vegetation both along the margins and within the river. Denny (1984) gave a general description of the sudd vegetation as seen only from a boat. Several taxa commonly observed along the margins of the Sudd included: *Ceratophyllum demersum* L., *Cyperus papyrus* L., *Eichhornia crassipes* (Mart.) Solms, *Phragmites karka* (Retz.) Trin. ex Steud., *Typha domingensis* Pers., *Vossia cuspidata* (Roxb.) Griff. A complete checklist of the vascular plants collected from this vegetative study can be found in the attached appendix of Denny (1984).

Eleven natural community types exist within Suggs Mill Pond Game Land, but the low and high pocosin communities are dominant, comprising 48% (2,119.74 hectares; 5,238 acres) of the site (Allen et al. 2015). Lakes and impoundments make up 8.6% (381.21 hectares; 942 acres) of the total acreage of the game land. Fair to high quality landscape connections exist between Suggs Mill Pond Game Land and adjacent natural areas within the Bladen Lakes Macrosite (i.e., Bushy Lake State Natural Area, Charlie Long Mill Pond/Big Colly Bay Natural Area, Jessups Pond, Mill Pond Bay Natural Area, and White Pond Bay Natural Area; LeBlond and Grant 2005). These connections to other large natural areas provide relatively uninterrupted habitat for the movement of plants and animals. Records of Horseshoe Lake’s water quality are lacking, but the lakes water appears high in humic substances and the chemistry is more than likely similar to the other Bladen Lakes. The lake is dystrophic and probably exhibits a pH of < 5.

***Jones Lake***

**Figure 11.**
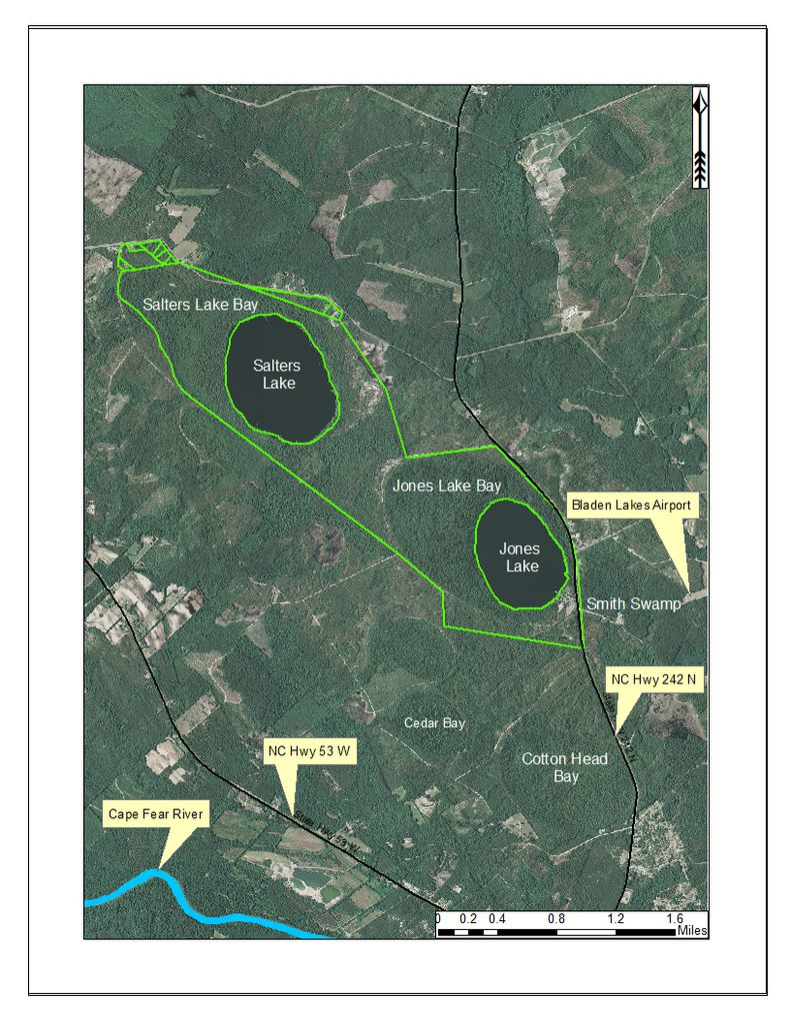
Jones Lake State Park (outlined in green) and surrounding lands. Jones Lake State Park is located between state highways 53 and 242, north of the Cape Fear River. Aerial imagery, transportation, and hydrography layers obtained from NRCS Geospatial Data Gateway: https://gdg.sc.egov.usda.gov. Map produced by Nathan Howell using ArcGis Desktop: Version 10.2.2. (Environmental Systems Research Institute (ESRI) 2014).

Jones Lake (91.05 hectares; 225 acres) is one of two dystrophic Carolina bay lakes located within Jones Lake State Park (893.54 hectares; 2,208 acres; Fig. 11), the other being Salters Lake. This lake is located in central Bladen County four miles north of Elizabethtown west of NC Hwy 242 and east of NC Hwy 53. Jones Lake State Parkforms the headwaters of an unnamed tributary of Turnbull Creek, which drains into the Cape Fear River. The state park sits on a sandy terrace (of Upper Pleistocene age) of the Cape Fear River (Soller 1988). Jones Lake was originally referred to as Woodward’s Lake, after Samuel Woodward, justice of the peace for the area in 1734 (North Carolina Division of Parks and Recreation, Planning and Development Section 2006b). It is believed that the lake later received its current name from Isaac Jones, an adjacent landowner to Samuel Wooodward, on whose land Elizabethtown was later established in 1773. Jones Lake State Park was established in 1939 and became the first state park specifically devoted to African Americans (North Carolina Department of Conservation and Development 1940).

LeBlond and Grant (2005) described both Jones and Salters Lakes as “among the very best examples of Carolina bay lakes in nearly pristine condition”. Jones Lake State Park is connected by fair to high quality landscape connections to Bethel Flatwoods, Cotton Bay Sand Ridge, Tatum Mill Pond/Cypress Bay, and Turnbull Creek Swamp natural areas.

Eleven natural community types have been described from Jones Lake State Park (i.e., Bay Forest, Coastal Plain Small Stream Swamp, High Pocosin, Low Pocosin, Natural Lake Shoreline, Peatland Atlantic White Cedar Forest, Pine/Scrub Oak Sandhill Mixed Oak Variant, Pond Pine Woodland, Wet Pine Flatwoods Wet Spodosol Variant, Xeric Sandhill Scrub Coastal Plain Variant, Xeric Sandhill Scrub Sandbarren Variant; LeBlond and Grant 2005, Schafale 2012), several of which are of extremely high quality and globally rare, such as the Low Pocosin, Peatland Atlantic White Cedar Forest, and Xeric Sandhill Scrub (LeBlond and Grant 2005, Schafale 2012). Available water quality parameters for Jones Lake are provided in Table 2.

***Lake Waccamaw***

**Figure 12.**
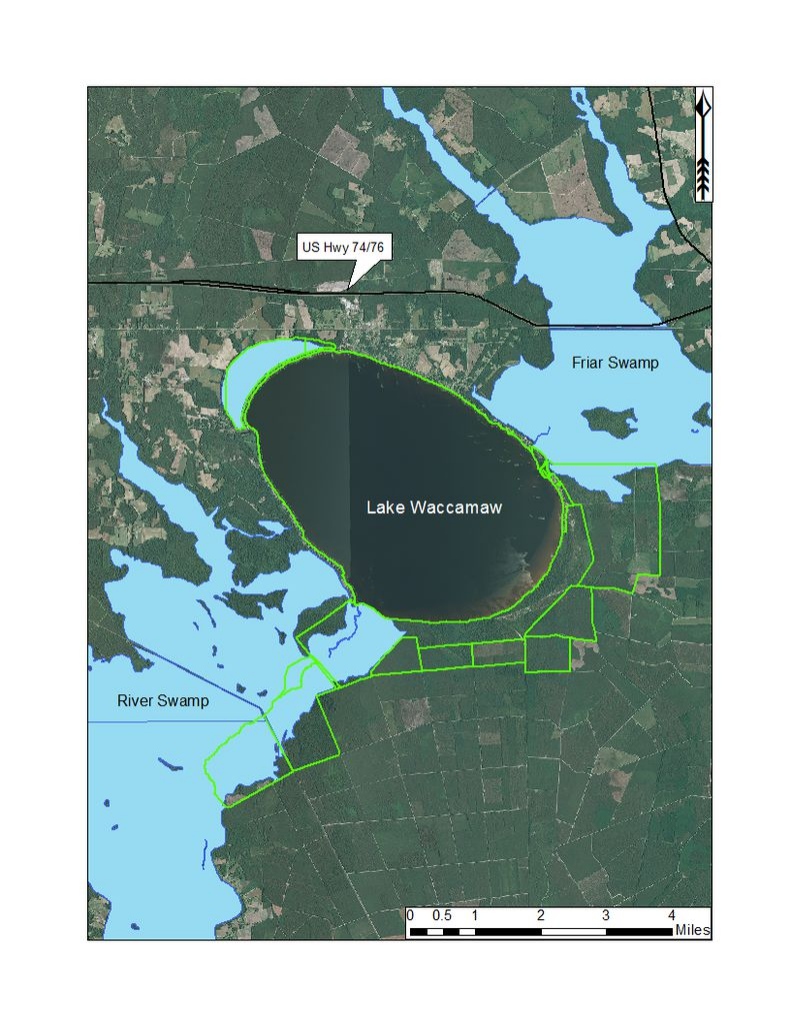
Lake Waccamaw State Park (outlined in green) and surrounding lands. Lake Waccamaw State Park is a large state park encompassing Lake Waccamaw and adjacent swampland and uplands. Aerial imagery, transportation, and hydrography layers obtained from NRCS Geospatial Data Gateway: https://gdg.sc.egov.usda.gov. Map produced by Nathan Howell using ArcGis Desktop: Version 10.2.2. (Environmental Systems Research Institute (ESRI) 2014).

Lake Waccamaw is located south of the township of Lake Waccamaw, between Friar Swamp to the northeast, and the Waccamaw River to the south. It is the only Carolina bay lake located in Columbus County and is the largest Carolina bay and bay lake (3,617.48 hectares; 8,939 acres) in North Carolina (LeBlond 1995). Lake Waccamaw is the third largest lake in North Carolina behind Lake Mattamuskeet and Lake Phelps. The lake is part of Lake Waccamaw State Park (4,327.70 hectares; 10,694 acres; Fig. 12), which also includes lands directly abutting the lake’s southern shoreline. Stager and Cahoon (1987) estimated Lake Waccamaw to be ca. 15,000 years old or less.

Prior to European civilization in the Southeast, the Waccamaw-Sioux Native American peoples, one of five Native American tribes known to inhabit the Cape Fear Region, inhabited the lands surrounding the lake (North Carolina Division of Parks and Recreation, Planning and Development Section 2006a). Native American artifacts, including dugout canoes, dating back to 1015−315 B.P. have been found within and around Lake Waccamaw. In the early 18^th^ century, an unknown young man traveled through Columbus County on his way from north Georgia and, upon seeing Lake Waccamaw, described it as “the most pleasantest place that ever I saw in my life. It is at least eighteen miles round, surrounded with exceeding good land, as oak of all sorts, hickory and fine cypress swamps” (Gentleman 1737).

This bay lake differs from the Bladen lakes in its larger size, neutral pH, mesotrophic status, and presence of alluvial hydrologic inputs (Big Creek). Tea-stained waters from Friar Swamp are delivered into northeast Lake Waccamaw via Big Creek, the largest of several creeks draining into the lake from Friar Swamp. Lake Waccamaw forms the headwaters of the Waccamaw River, a species-rich river system known to support several rare plant (e.g., *Fimbristylis perpusilla* R.M. Harper ex Small & Britton, *Ilex amelanchier* M.A. Curtis ex Chapm., *Lipocarpha micrantha* (Vahl) G.C. Tucker, *Oldenlandia boscii* (DC.) Chapm., *Rhynchospora decurrens* Chapm., and *Sabatia kennedyana* Fernald) and animal taxa (*Alligator mississipiensis* [American Alligator], *Elliptio folliculata* [Pod Lance], *Etheostoma perlongum* [Waccamaw Darter], *Lampsilis ochracea* [Tidewater Mucket], *Menidia extensa* [Waccamaw Silverside], *Noturus* spp. *2* [Broadtail Madtom], and *Procambarus leptodactylus* [Pee Dee Lotic Crayfish; LeBlond 1995]).

Much of the land surrounding Lake Waccamaw has been converted to agriculture (north of the lake) and loblolly pine plantations (south of the lake). A small portion of Lake Waccamaw’s bay is still present on the northern end.

The Coastal Plain Marl Outcrop occurs along a roughly 394 m (1,000 ft.) stretch of northern shoreline and is characterized by having vertical and overhanging low cliffs in the supralittoral zone of the lake. Portions of these cliffs are submerged in the upper eulittoral zone, but local residents privately own terrestrial portions. This marl community is known for supporting the only naturally occuring population of Venus hair fern (*Adiantum capillus-veneris* L.) in the state.

Shoreline residential development extends along the northern shores of the lake from the lake outlet (southwest corner of lake) to just south of Big Creek. These shorelines support the globally rare Natural Lake Shoreline Marsh (Lake Waccamaw Pondlily Subtype) community. Undeveloped shorelines (i.e., Natural Lake Shoreline Swamp – Lake Waccamaw Subtype) occur from just south of Big Creek to the lake’s outlet. Historically, Lake Waccamaw experienced wide-ranging water level fluctuations determined by precipitation. In 1925, a poorly constructed dam was built at the lakes outlet in an effort to stabilize lake levels for increased recreational use. Before construction began, lake levels were so low that vehicles could be driven to the construction site on the dried lake bed (North Carolina Division of Parks and Recreation, Planning and Development Section 2006a).

The physical and hydrographic nature of Lake Waccamaw’s shoreline also differs from the other bay lakes. Lake Waccamaw’s shoreline is sandy around its entire periphery (Frey 1949), whereas the Bladen lakes may be either sandy or peaty along their shorelines.

A broad, sandy, terrace (lacking in Bladen lakes) is also present along the southeast shoreline of Lake Waccamaw (Fig. 5). This shallow underwater terrace extends perpendicularly out into the lake as far as 305 m (1,000 ft.; Frey 1949). The gentle relief of the terrace gradually extends shoreward resulting in a shallow, broad, littoral zone. This littoral zone is the most floristically rich of all Carolina bay lakes and is rivaled only by Lake Phelps in Washington County, North Carolina (N. Howell, pers. obs.). Varying water depths in the littoral zone of Lake Waccamaw result in the temporary and sometimes permanent presence of offshore sandbars and islands. This hydrographical heterogeneity in the littoral zone increases the floristic richness. A more detailed review of the lakes shoreline flora is provided in the floristic summary section and in Suppl. material 6.

The buffering effect of subsurface and surficial limestone on the naturally acidic waters of Lake Waccamaw result in an unusually diverse fauna. Lake Waccamaw contains the largest number of endemic animal species (i.e., endemic to this lake and nowhere else in the world; 10 taxa) of any site in North Carolina (Hubbs and Raney 1946, LeBlond 1995). An additional species, *Fundulus waccamawensis* (Waccamaw Killfish), is found only in waters within and adjacent to Lake Waccamaw and Lake Phelps (Washington County, North Carolina). Six other faunal taxa known to be rare but not endemic also occur within or adjacent to the lake. Available water quality parameters for Lake Waccamaw are provided in Table 3.

***Little Singletary Lake***

Little Singletary Lake (626 acres; 253.33 hectares) is located in the western half of Suggs Mill Pond Game Land (Fig. 10). Unlike Horseshoe Lake, Little Singletary Lake is natural in origin and exhibits a more “typical” bay lake physiognomy. Little Singletary Lake forms the headwaters of Lake Run, a tributary of Ellis Creek, which drains into the Cape Fear River. Relatively intact landscape connections exist to the northeast (Horseshoe Lake), southeast (Marshy Bay Natural Area), and southwest (Cedar Swamp Seep Natural Area) from Little Singletary Lake.

Lands abutting the southern shoreline are privately owned and were once subject to residential development. Remnants of bulkheads and recreational piers can still be seen today along the southeast shoreline. The North Carolina Wildlife Resources Commission gained property rights to all remaining lands surrounding Little Singletary Lake before residential development could ensue. On June 20, 2011, a lightning caused wildfire (Simmons Road Fire) started just west of Little Singletary Lake and by August 18^th^, had burned over 2,023 hectares (5,000 acres) of Carolina bay and pocosin habitat, much of which surrounded Little Singletary Lake. During growing seasons of extreme drought, water levels have been known to recede low enough to reveal a clean sandy lake bottom 90−275 m (100−300 yds) out into the lake (G. Lewis, pers. comm.). Native American projectile points have been found on this lake bottom during drought years (G. Lewis, pers. comm.).

The water quality of Little Singletary Lake has not been documented by state agencies. The water appears high in humic substances and is likely similar to the other Bladen lakes (i.e., dystrophic, acidic, shallow, nutrient poor).

***Salters Lake***

Salters Lake (127.47 hectares; 315 acres) is the larger of the two Carolina bay lakes located in Jones Lake State Park (Fig. 11). Salters Lake was named after Sallie Salter, a revolutionary war hero who spied on the Tories while encamped at Elizabethtown. Her spying played a role in the defeat over the Tories on August 28, 1771, at the battle of Elizabethtown, where 70 Whigs defeated 400 Tories (JNorth Carolina Division of Parks and Recreation, Planning and Development Section 2006b).

Salters Lake is similar to Jones Lake in many respects, but quite possibly could be the most “pristine” of all Carolina bay lakes. Salters Lake has no shoreline development, appreciable recreational activities (e.g., outboard motor use), *immediate* surrounding agricultural (crop or animal production) land use, water level control structures, or historical manipulation of any kind. Natural communities and landscape features for Salters Lake are the same as those for Jones Lake (above). Available water quality parameters for Salters Lake are provided in Table 4.

***Singletary Lake***

**Figure 13.**
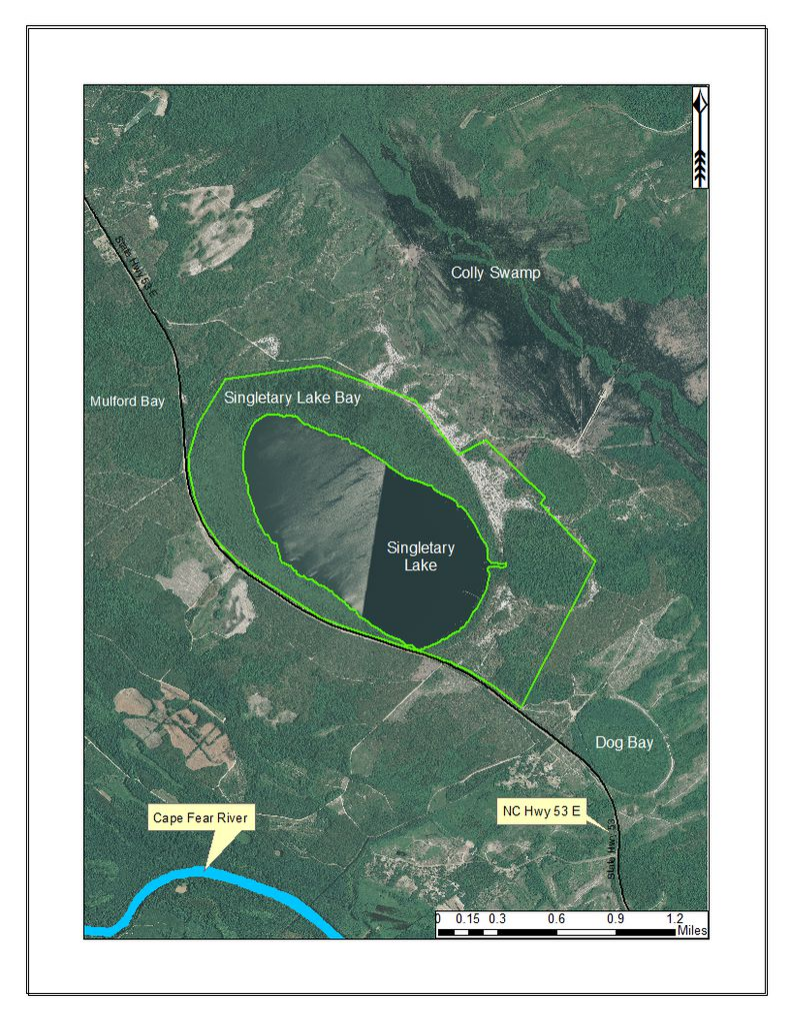
Singletary Lake State Park (outlined in green) and surrounding lands. Singletary Lake State Park is primarily comprised of lands immediately surrounding Singletary Lake. In addition to the lands surrounding Singletary Lake, White Lake is also managed by Singletary Lake State Park. Singletary Lake State Park is located north of the Cape Fear River and State Hwy 53 and southeast of White Lake. Aerial imagery, transportation, and hydrography layers obtained from NRCS Geospatial Data Gateway: https://gdg.sc.egov.usda.gov. Map produced by Nathan Howell using ArcGis Desktop: Version 10.2.2. (Environmental Systems Research Institute (ESRI) 2014).

Singletary Lake (233.09 hectares; 576 acres) is located within Singletary Lake State Park (494.12 hectares; 1,221 acres; Fig. 13). This lake was named after Richard Singletary, who received the grant of land in 1729 (North Carolina Division of Parks and Recreation, Planning and Development Section 1996b). Singletary Lake State Park is located just southeast of White Lake in central-southeast Bladen County between the Cape Fear River and Colly Swamp. Singletary Lake forms the headwaters of Lake Drain Creek, which drains into Big Colly Creek, which drains to the Black River, which drains into the Cape Fear River.

Singletary Lake is similar to the other Bladen lakes in that it is dystrophic, acidic, and nutrient poor. It contains high quality examples of the Natural Lake Shoreline Swamp (Cypress Subtype) and Natural Lake Shoreline Marsh (Typic Subtype) communities. LeBlond and Grant (2005) described this lake’s shoreline community as “one of the most aesthetically pleasing natural communities in the North Carolina Coastal Plain”. A direct landscape connection exists between Singletary Lake and Colly Swamp and the Black River to the northeast. Fair quality landscape connections exist between the state park and the Cape Fear River to the southwest. Available water quality parameters for Singletary Lake are provided in Table 5.

***White Lake***

**Figure 14.**
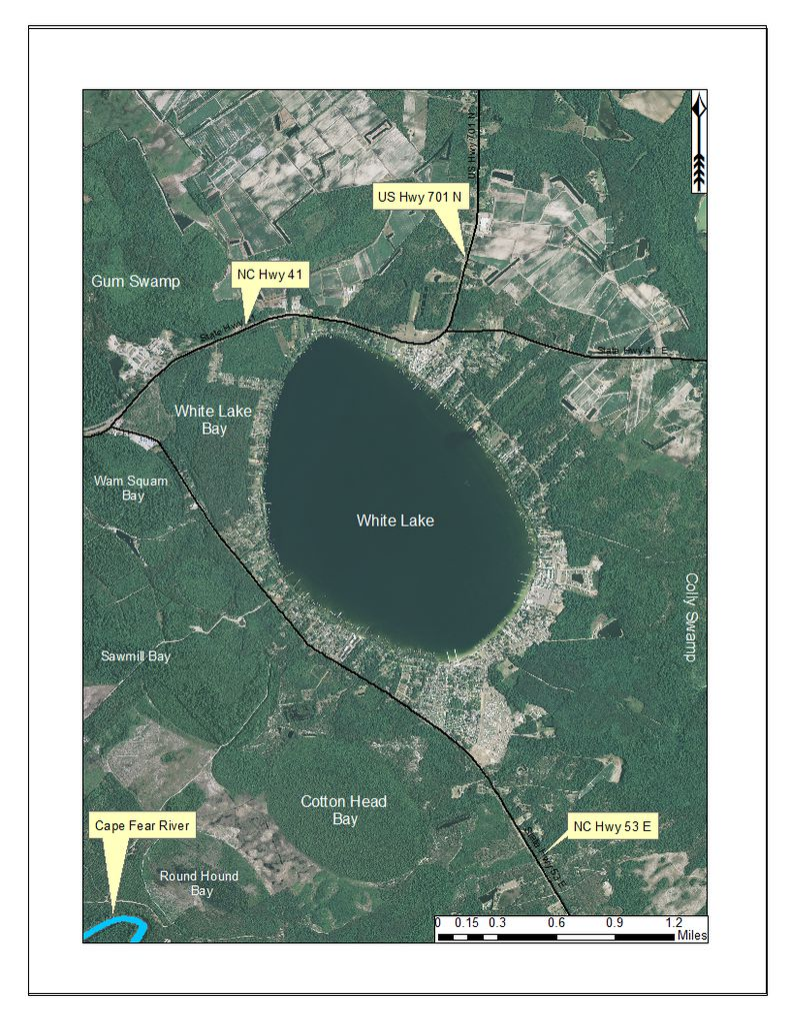
White Lake and surrounding lands. Like the majority of Carolina bay lakes, White Lake is a state-owned lake. All but a very small portion of White Lake’s shoreline has been altered. Aerial imagery, transportation, and hydrography layers obtained from NRCS Geospatial Data Gateway: https://gdg.sc.egov.usda.gov. Map produced by Nathan Howell using ArcGis Desktop: Version 10.2.2. (Environmental Systems Research Institute (ESRI) 2014).

Although not included in the sampling aspect of this study, White Lake is unique and deserves a brief summary. White Lake (432.20 hectares; 1,068 acres) is a large Carolina bay lake located in east-central Bladen County about 6 miles east of Elizabethtown, just east of the intersection of NC Hwy 53 and U.S. Hwy 701 (Fig. 14). White Lake is owned by the state of North Carolina, and is managed by Singletary Lake State park. Unlike all of the remaining bay lakes, White lake’s water is clear and not tea-stained. This feature has made it an incredibly attractive location for development and vacationers. This lake is primarily used for recreation (e.g., water sports, swimming, fishing) and essentially all of its shoreline is residentially and commercially developed.

White Lake’s remarkable water clarity is attributed to the presence of artesian springs on the lake bottom (Wells and Boyce 1953). The clarity of the lake’s water yields a deep euphotic zone (i.e., sunlight can penetrate through the entirety of the water column) with submerged aquatic macrophytes (e.g., *Myriophyllum humile* (Raf.) Morong; N. Howell pers. obs.) present at the lakes deepest depths. White Lake receives its hydrologic inputs principally in two forms, precipitation and groundwater (through springs). Although this lake is primarily fed by springs, its overall water levels are determined by the regional water table (i.e., during drought years, White Lake’s water levels will drop just like all other bay lakes). Another unique feature of White Lake is the location of its outlet channel. White Lake’s outlet channel is located in the northwestern section of the lake as opposed to the southeastern section where it occurs in all other bay lakes. Frey (1954) reported that William Bartram, a renowned naturalist who documented the flora, fauna, and Native American culture of the southeastern United States in the 18^th^ century, operated a sawmill on White Lake during the 20 years following 1770. A map in Bartram and Harper (1942) shows that White Lake was formerly called Lake Bartram. Available water quality parameters for White Lake are provided in Table 6.

### Climate

***Bladen Lake Group (Bladen County, NC)***

Climate data from the nearest weather station to the Bladen County bay lakes, ca. 1.6 kilometers away in Elizabethtown, North Carolina (Bladen County: 34.68° N, -78.58°W; 30.5 m elev.), show that during the thirty-year period between 1971-2000, the average annual temperature was 16.44 °C (61.6 °F) and mean annual precipitation 1,254.76 mm (49.4 in). Average daily maximum and minimum temperatures were 22.83 °C (73.1 °F) and 10.11 °C (50.2 °F; State Climate Office of North Carolina 2014; Fig. 15).

The lowest temperature recorded for Bladen County was -14.4 °C (6 °F) on January 17, 1977 (Leab 1990). The highest recorded temperature for Bladen County was 37.7 °C (100 °F) on July 20, 1977 (Leab 1990). Monthly average temperatures were highest in July and August and lowest in December and January. Monthly precipitation amounts were also highest in July and August, while the lowest monthly precipitation amounts were in April and November (State Climate Office of North Carolina 2014; Fig. 15). The annual growing season, defined as the number of days in five out of ten years during which the daily minimum air temperature exceeds -2.2 °C (28 °F), is 243 days in Bladen County (weather data recorded from 1957-1979; Leab 1990).

***Lake Waccamaw (Columbus County, NC)***

Climate data from the nearest weather station to Lake Waccamaw, ca. 16 km away in Whiteville, North Carolina (Columbus County: 34.27287° N, -78.71499° W; 29.8 meters above sea level), show that for the 30-year period between 1971 and 2000, the average annual temperature was 17.16 °C (62.9 °F) and mean annual precipitation 1,275.08 mm (50.2 in). The average daily maximum and minimum temperatures over the same thirty-year period were 24.3 °C (75.8 °F) and 10 °C (50 °F; State Climate Office of North Carolina 2014; Fig. 15).

The lowest temperature recorded for Columbus County was -15 °C (5 °F) on February 12, 1973 (Spruill 1990). The highest recorded temperature for Columbus County was 40.5 °C (105 °F) on June 27, 1954 (Spruill 1990). Monthly average temperatures were highest in July and August and lowest in January and February. Monthly precipitation amounts were also highest in July and August, while the lowest monthly precipitation amounts were in April and November (State Climate Office of North Carolina 2014; Fig. 15). The annual growing season, defined as the number of days in five out of ten years during which the daily minimum air temperature exceeds -2.2 °C (28 °F), is 240 days in Columbus County (weather data recorded from 1951-1981; Spruill 1990).

### Plant Communities

Four plant community types and two subtypes can be distinguished within the littoral zone of Carolina bay lakes (Schafale 2012; Table 7). Of these four community types and subtypes, three are globally critically imperiled (Natural Lake Shoreline Swamp – Lake Waccamaw Subtype; Natural Lake Shoreline Marsh – Typic Subtype; Natural Lake Shoreline Marsh − Lake Waccamaw Pondlily Subtype), while the others do not have a conservation ranking (Table 7).

**Natural Lake Shoreline Swamp (Cypress Subtype; S2G3) [*Taxodium distichum* – *T. ascendens* / *Panicum hemitomon* Schult. Woodland (CES203.044)].**

This natural community type covers Carolina bay lake shorelines with narrow littoral zones characterized by an absent to sparse herbaceous component and a nearly closed canopy of *Chamaecyparis* Spach, *Nyssa* L., or *Taxodium* Rich. in the upper eulittoral zone. If a cross-section of this littoral zone were to be drawn, the epilittoral vegetation would abruptly coincide with the littoral zone (i.e., a zone of emergent herbaceous vegetation is lacking where it typically would occur between the epilittoral and infralittoral zones). This “two-staged” zonation pattern typical of this community type is directly attributable to the steeper hydrography and narrow littoral zone. The Natural Lake Shoreline Swamp (Lake Waccamaw Subtype) and the Natural Lake Shoreline Marsh community types can be distinguished from the depauperate Natural Lake Shoreline Swamp (Cypress Subtype) community type by having a broader littoral zone, a well-developed zone of herbaceous emergent macrophytes, a sparse to open canopy of *Nyssa*, *Taxodium*, or other obligate wetland hardwoods, and the absence of *Nuphar sagittifolia* (Walter) Pursh. Examples of this community type are found at Bakers Lake, and the western, northern, and eastern shorelines of Jones, Salters, Little Singletary, and Singletary Lakes.

**Natural Lake Shoreline Swamp (Lake Waccamaw Subtype; S1G1) [*Taxodium distichum* – *T. ascendens* / *Panicum hemitomon* – *Sclerolepis uniflora* (Walter) Britton, Sterns & Poggenb. Woodland (CEGL004465)].**

This natural community type covers the southern shoreline of Lake Waccamaw located between Big Creek and the lake’s outlet on the southwest shore. This stretch of natural shoreline is characterized by gentle hydrography, which results in a broad littoral zone, and a species-rich flora dominated by emergent herbaceous macrophytes, many of which are rare. Emergent macrophytes typical of this community type include *Cladium mariscoides* (Muhl.) Torr., *Eriocaulon aqutaicum* (Hill) Druce, *Panicum hemitomon*, *Sclerolepis uniflora*, and *Xyris smalliana*, among others. This community type can be distinguished from the species-poor Natural Lake Shoreline Swamp (Cypress Subtype) community type by its broader littoral zone and species-rich herbaceous component (95 taxa). It can be distinguished from the Natural Lake Shoreline Marsh community types by the absence or only irregular presence of *Nuphar sagittifolia* and the unique assemblage of diverse herbaceous taxa (e.g., *Bacopa caroliniana* (Walter) B.L. Rob., Boltonia asteroides (L.) L’Hér. var. glastifolia, *Cladium mariscoides*, *Ludwigia brevipes* (B.H. Long ex Britton, A. Braun & Small) Eames, *L. sphaerocarpa* Elliott, and *Sclerolepis uniflora*).

**Natural Lake Shoreline Marsh (Typic Subtype; S1G1) [*Panicum hemitomon* – *Juncus* spp. Coastal Plain Lakeshore Herbaceous Vegetation (CEGL004307)].**

This natural community type covers the southern shorelines of the Bladen Lakes. The southern shorelines have a broader littoral zone than the remaining portions of the lakes. Consequently, they support a more diverse emergent herbaceous component. Herbs found in this community type include *Eleocharis baldwinii*, *E. equisetoides* (Elliott) Torr., *E. vivipara*, *Juncus pelocarpus* E. Mey., *Panicum hemitomon*, *Panicum verrucosum* Muhl., *Rhexia nashii*, *Rhynchospora distans*, *Saccharum giganteum* (Walter) Pers., *Sacciolepis striata*, *Scirpus cyperinus* (l.) Kunth, and *Xyris smalliana*. This community type is also characterized as having a sparse to open canopy of *Nyssa* and *Taxodium*. This community type can be distinguished from the Natural Lake Shoreline Marsh (Lake Waccamaw Pondlily Subtype) by the absence of *Nuphar sagittifolia* and from the Natural Lake Shoreline Swamp (Lake Waccamaw Subtype) by the occurence of < 30 herbaceous taxa, none of which include the unique and rare herbs found at Lake Waccamaw. Examples of this community type include the southern shorelines of Jones, Little Singletary, Salters, and Singletary Lakes.

**Natural Lake Shoreline Marsh (Lake Waccamaw Pond-lily Subtype; S1G1) [*Nuphar sagittifola – Eriocaulon aquaticum* Lakeshore Herbaceous Vegetation (CEGL004297)].**

This natural community type covers the western, northern, and eastern shorelines of Lake Waccamaw (i.e., where residential and commercial development is present). It is the only Natural Lake Shoreline community type dominated by *Nuphar sagittifolia* (a distinguishing feature) and *Eriocaulon aquaticum*. *Nuphar sagittifolia* is essentially absent from the Natural Lake Shoreline Swamp (Lake Waccamaw Subtype) community type save for small stands around the mouth of Big Creek and around the dam at the lakes outlet.

**floating Bog [*Rhynchospora alba* Saturated Herbaceous Vegetation (CEGL004463)]**

This natural community type covers the rare examples of vegetation occuring on floating peat mats in deep water of natural or artificial ponds and lakes. Horseshoe Lake is the only Carolina bay lake known to support floating bogs. The floating bogs of Horseshoe Lake are the largest in the state. These floating bogs are saturated and nutrient-poor, supporting taxa that characteristically inhabit such stressful conditions (e.g., *Calopogon tuberosus* (L.) Britton, Sterns & Poggenb., *Drosera intermedia*, *Dulichium arundinaceum*, *Hypericum virginicum*, *Pogonia ophioglossoides*, *Rhynchospora alba*, *R. inundata*, and *Xyris fimbriata*). This community type’s “floating” nature and the presence of the aforementioned plant taxa sets it apart from all others.

**Coastal Plain Semipermanent Impoundment (Cypress-Gum Subtype; G4G5) [*Taxodium distichum* / *Lemna minor* L. Forest (CEGL002420)]**

All portions of Horseshoe Lake not considered floating Bog fall into the Coastal Plain Semipermanent Impoundment community type. This community type is characterized by a sparse to absent canopy of *Taxodium ascendens* with sporadically occurring beds of floating-leaved and submersed aquatics (e.g., *Brasenia schreberi* J.F. Gmel, *Cabomba caroliniana* A. Gray, Nymphaea odorata ssp. odorata, and *Utricularia* spp.). This community type can be distinguished from all others by the sparse presence of *Taxodium* throughout the lake with floating-leaved and submersed aquatics occurring underneath.

### Floristic Summary

***Across All Sites***

The littoral zone vascular flora of Carolina bay lakes, based on vouchered collections, reports, and personal observations, consists of 205 taxa (170 species, 4 subspecies, 30 varieties, 1 hybrid) in 136 genera and 80 vascular plant families (Table 8; Suppl. material 6). Of these 205 taxa, 186 (90.7%) are vouchered and 19 (9.3%) are known only from reports (Peet et al. 2013a, Peet et al. 2013b, North Carolina Natural Heritage Program 2014; NCSU Crop Science Department [Rob Richardson and Justin Nawrocki, pers. comm., April 9, 2015]). Of the 186 vouchered taxa, 157 (84.4%) were collected by the first author; the remaining 29 (15.6%) vouchered taxa were collected from Carolina bay lake shorelines by others and were found by completing systematic searches of major herbaria (DUKE, NCSC, and NCU). Nineteen taxa (9.3%) are listed as significantly rare and twelve taxa (5.8%) are on the NCNHP Watch List (Table 9). Four taxa (1.9%) are Federal Species of Concern (*Ludwigia brevipes*; *Nuphar sagittifolia*; *Rhexia aristosa* Britton; *Sagittaria weatherbiana*). Pair-wise comparisons of species similarity for all bays are provided in Table 10.

**Figure 16.**
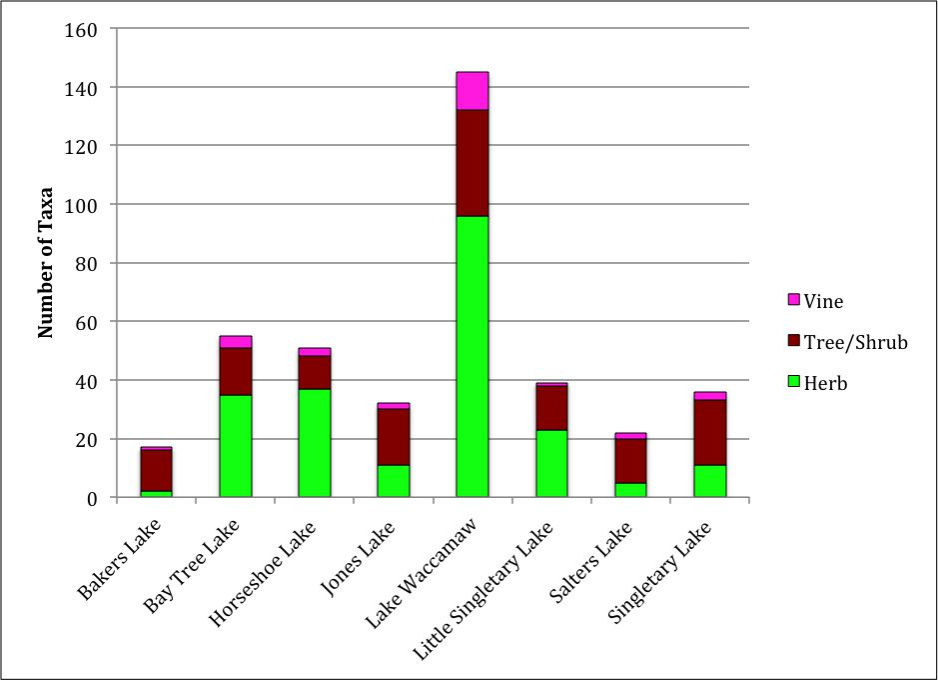
Distribution of plant habit across all Carolina bay lakes. Lakes dominated by herbs have broader littoral zones, which encourage the establishment of herbaceous emergent macrophytes. Lakes dominated by trees and shrubs have narrow littoral zones, which discourage the establishment of herbaceous emergent macrophytes.

**Figure 17.**
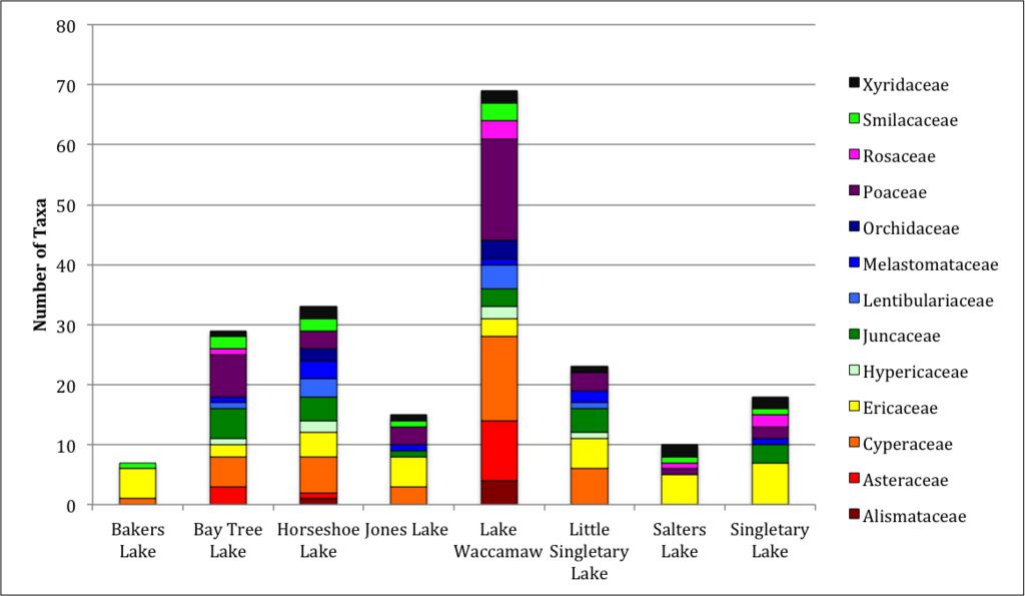
The thirteen most species-rich vascular plant families across all Carolina bay lakes. Cyperaceae (orange), Ericaceae (yellow), Juncaceae (dull green), Poaceae (purple), Smilacaceae (neon green), and Xyridaceae (black) consistently occur across all sites.

Among all taxa treated in this guide, the major vascular plant groups consisted of the following total taxa: Eudicotyledons (101 taxa; 86 species, 1 subspecies, 13 varieties, 1 hybrid), monocotyledons (86 taxa; 71 species, 1 subspecies, 14 varieties), pteridophytes (7 taxa; 6 species and 1 subspecies), gymnosperms (5 species), basal angiosperms (4 taxa; 3 species and 1 subspecies), and magnoliids (2 taxa; 1 species and 1 variety; Table 8; Fig. 16). The richest families in the eudicotyledons are Asteraceae (13 taxa; 11 species, 1 variety, 1 hybrid), Ericaceae (8 taxa; 6 species, 2 varieties), Lentibulariaceae (6 taxa), Melastomataceae (5 taxa; 4 species, 1 variety), Hypericaceae (4 taxa; 3 species, 1 variety), and Rosaceae (4 taxa; Fig. 17). The richest genera in the eudicotyledons are *Utricularia* (6 taxa), *Rhexia* L. (5 taxa), and *Hypericum* L. (4 taxa). The richest families in the monocotyledons are Cyperaceae (25 taxa; 20 species, 5 varieties), Poaceae (21 taxa; 17 species, 4 varieties), Juncaceae (8 taxa), Orchidaceae (5 taxa; 4 species, 1 variety), Alismataceae (4 taxa), Smilacaceae (4 taxa), and Xyridaceae (4 taxa: Fig. 17). The richest genera in the monocotyledons are *Rhynchospora* (9 taxa; 8 species, 1 variety), *Juncus* (8 taxa), *Dichanthelium* (Hitchc. & Chase) Gould (6 taxa; 5 species, 1 variety), *Carex* (4 taxa; 3 species, 1 variety), *Eleocharis* (4 taxa; 3 species, 1 variety), *Sagittaria* L. (4 taxa), *Smilax* L. (4 taxa), and *Xyris* L. (4 taxa).

Among all taxa treated in this guide, the most species-rich habit is herbs (140 taxa; 119 species, 2 subspecies, 18 varieties, 1 hybrid), followed by trees and shrubs (51 taxa; 42 species, 1 subspecies, 8 varieties), and vines (14 taxa, 12 species, 2 varieties; Fig. 16). Among the herbs, Cyperaceae (25 taxa), Poaceae (20 taxa), Asteraceae (11 taxa), Juncaceae (8 taxa), Lentibulariaceae (6 taxa), Melastomataceae (5 taxa), and Orchidaceae (5 taxa) are the most species-rich families. Among trees and shrubs, the Ericaceae (8 taxa) and Rosaceae (4 taxa) were the most species-rich families. Among vines, the Smilacaceae (4 taxa) and Vitaceae (2 taxa) were the most species rich families.

Among the natural community types included in this work, the Natural Lake Shoreline Swamp (Lake Waccamaw Subtype) is the most species-rich (145 taxa) and the Natural Lake Shoreline Marsh (Lake Waccamaw Pondlily Subtype) is the least species-rich (< 10 taxa; Table 7). Five exotic taxa are known to occur in the bay lakes, four (*Alternanthera philoxeroides* [Amaranthaceae], *Colocasia esculenta* (L.) Schott [Araceae], *Hydrilla verticillata* (L.F.) Royle [Hydrocharitaceae], *Triadica sebifera* (L.) Small [Euphorbiaceae]) from Lake Waccamaw and one (*Hypochaeris radicata* L. [Asteraceae]) from Bay Tree Lake.

***Individual Lakes***

Among the lakes, the largest number of littoral zone taxa (i.e., species, subspecies, and varieties) occurred in Lake Waccamaw (145 taxa), followed by Bay Tree Lake (56 taxa) and Horseshoe Lake (52 taxa; Table 11). The least number of littoral zone taxa occurred in Bakers Lake (18 taxa).

***Bakers Lake***

The littoral zone vascular flora of Bakers Lake is depauperate with respect to the other bay lakes (Table 11). A total of 18 taxa (14 species, 4 varieties) in 17 genera and 14 vascular plant families were found in this lake’s littoral zone (Suppl. material 6). All but one taxon (*Tillandsia usneoides*) from Bakers Lake were collected by the first author (i.e., there were no reports or historical vouchers). The richest eudicotyledonous family was Ericaceae (5 taxa; Fig. 17).

The most species-rich habit class was trees and shrubs (14 taxa; 10 species, 4 varieties), followed by herbs (3 taxa), and vines (1 taxa; Fig. 16). Among the trees and shrubs, the Ericaceae (5 taxa) is the most species-rich family. No exotic taxa or taxa of conservation concern occured at this site. One species (*Rhus copallinum* L.) was unique to this Carolina bay lake (i.e., it was not found/reported from any other bay lake in this study; Suppl. material 5).

***Bay Tree Lake***

The littoral zone vascular flora of Bay Tree Lake is comprised of 56 taxa (48 species, 2 subspecies, and 6 varieties), in 47 genera and 34 vascular plant families (Table 11; Suppl. material 6). All but 2 taxa from Bay Tree Lake were vouchered; *Decodon verticillatus* and Pontederia cordata L. var. cordata were personal observations. No species of conservation concern were collected or reported from Bay Tree Lake’s littoral zone. One exotic taxon (*Hypochaeris radicata*) was collected from this site (Suppl. material 6). Twelve taxa are unique to this bay lake (i.e., they were not found/reported from any other bay lake in this study; Suppl. material 6: [*Amelanchier canadensis* (L.) Medik., *Carex longii* Mack., Cyperus odoratus L. var. odoratus, *Diodia virginiana* L., *Fuirena pumila* (Torr.) Spreng., *Hypochaeris radicata*, *Juncus acuminatus* Michx., *Krigia virginica* (L.) Willd., *Nuttallanthus canadensis* (L.) D.A. Sutton, *Panicum virgatum*, *Rumex hastatulus* Baldwin, *Smilax glauca* Walter, and Stipulicida setacea Michx. var. setacea]).

The richest eudicotyledon families are Asteraceae (3 taxa), followed by Ericaceae (2 taxa) and Aquifoliaceae (2 taxa;) . The richest monocotyledonous families are Poaceae (7 taxa; 6 species, 1 subspecies), Cyperaceae (5 taxa; 4 species, 1 variety), and Juncaceae (5 taxa). The richest monocotyledon genera are *Juncus* (5 taxa; 3 species, 1 subspecies, 1 variety) and *Panicum* (3 taxa).

The most species-rich habit class was herbs (35 taxa; 29 species, 2 subspecies, 4 varieties), followed by trees and shrubs (16 taxa; 15 species, 1 variety), and vines (4 species, 1 variety; Fig. 16). Among the herbs, Poaceae (7 taxa; 6 species, 1 subspecies), Cyperaceae (5 taxa; 4 species, 1 variety), Juncaceae (5 taxa), and Asteraceae (3 taxa) are the most species-rich families. Among the trees and shrubs, Cupressaceae (3 taxa), Aquifoliaceae (2 taxa), and Ericaceae (2 taxa) are the most species-rich families.

***Horseshoe Lake***

The littoral zone vascular flora of Horseshoe Lake is comprised of 52 taxa (45 species, 2 subspecies, and 5 varieties), in 41 genera and 29 vascular plant families (Table 11; Suppl. material 6). All but three taxa from Horseshoe Lake were vouchered; *Eleocharis baldwinii*/*vivipara*, *Rhexia aristosa*, and *Tillandsia usneoides* were the only taxa not vouchered from the site. No exotic taxa were collected from this site. Sixteen taxa are unique to this bay lake (i.e., they were not found/reported from any other bay lake in this study; Suppl. material 6). Five taxa of conservation concern were collected or reported from this site (*Rhexia aristosa*, *Rhynchospora alba*, *Rhynchospora inundata*, *Sagittaria isoetiformis* J.G. Sm., and *Xyris smalliana*; Table 9).

The richest eudicotyledon families are Ericaceae (4 taxa), Lentibulariaceae (3 taxa) and Melastomataceae (3 taxa). The richest eudicotyledonous genera are *Rhexia* (3 taxa), *Utricularia* (3 taxa), followed by *Hypericum* (2 taxa). The richest monocotyledonous families are Cyperaceae (5 taxa), Juncaceae (4 taxa), Poaceae (3 taxa), followed by Orchidaceae (2 taxa), Smilacaceae (2 taxa) and Xyridaceae (2 taxa). The richest monocotyledonous genera are *Juncus* (4 taxa), followed by *Rhynchospora* (2 taxa), *Smilax* (2 taxa), and *Xyris* (2 taxa).

The most species-rich habit class was herbs (38 taxa; 31 species, 2 subspecies, 4 varieties), followed by trees and shrubs (11 taxa; 10 species, 1 variety), and vines (3 taxa; Fig. 16). Among the herbs, Cyperaceae (6 taxa), Juncaceae (4 taxa), followed by Lentibulariaceae (3 taxa), Melastomataceae (3 taxa), Poaceae (3 taxa), Orchidaceae (2 taxa), and Xyridaceae (2 taxa) are the most species-rich families. Among the trees and shrubs, the most species-rich family is Ericaceae (4 taxa).

***Jones Lake***

The littoral zone vascular flora of Jones Lake is comprised of 33 taxa (29 species, 1 subspecies, and 3 varieties), in 31 genera and 23 vascular plant families (Table 11; Suppl. material 6). All taxa, save for *Cyrilla racemiflora*, were vouchered by the first author or others. No exotic taxa were collected from this site. Two taxa are unique to this bay lake (i.e., they were not found/reported from any other bay lake in this study; Suppl. material 6: [*Cyperus polystachyos* Rottb., *Rhynchospora inexpansa* (Michx.) Vahl]). *Xyris smalliana* was the only species of conservation concern collected from this site (Table 9).

The richest eudicotyledonous family is Ericaceae (5 taxa). The richest eudicotyledonous genus is *Lyonia* (2 taxa; 1 species, 1 variety). The richest monocotyledonous families are Cyperaceae (3 taxa) and Poaceae (3 taxa). Monocotyledons are comprised of ten different genera.

The most species-rich habit class was trees and shrubs (20 taxa; 16 species, 1 subspecies, 3 varieties), followed by herbs (11 taxa), and vines (2 taxa; Fig. 16). Among the herbs, Cyperaceae (3 taxa) and Poaceae (3 taxa) are the most species-rich families. Among the trees and shrubs, Ericaceae (5 taxa) and Cupressaceae (3 taxa) are the most species-rich families.

***Lake Waccamaw***

The littoral zone vascular flora of Lake Waccamaw is comprised of 145 taxa (122 species, 3 subspecies, 19 varieties, 1 hybrid), in 111 genera and 72 vascular plant families (Table 11; Suppl. material 6). Of the 145 total catalogued taxa, 127 are vouchered and 18 are known only from reports (Suppl. material 6). Twenty-six species of conservation concern were collected or reported from Lake Waccamaw’s littoral zone. Four exotic taxa (*Alternanthera philoxeroides* [Amaranthaceae], *Colocasia esculenta* [Araceae], *Hydrilla verticillata* [Hydrocharitaceae], *Triadica sebifera* [Euphorbiaceae]) are known from this site. Ninety-five taxa are unique to Lake Waccamaw (i.e., they were not found/reported from any other bay lake in this study; Suppl. material 6).

The richest eudicotyledonous families are Asteraceae (10 taxa; 8 species, 1 variety, 1 hybrid), followed by Lentibulariaceae (4 taxa), Ericaceae (3 taxa), Rosaceae (3 taxa), and Salicaceae (3 taxa). The richest eudicotyledonous genera are *Utricularia* (4 taxa), *Eupatorium* L. (2 taxa), *Hypericum* (2 taxa), *Ludwigia* L. (2 taxa), *Nyssa* (2 taxa), and *Salix* L. (2 taxa). The richest monocotyledonous families are Poaceae (17 taxa; 13 species, 1 subspecies, 3 varieties), Cyperaceae (14 taxa; 11 species, 3 varieties), Alismataceae (4 taxa), Juncaceae (3 taxa), Orchidaceae (3 taxa), and Smilacaceae (3 taxa). The richest monocotyledonous genera are *Dichanthelium* (Hitchc. & Chase) Gould (6 taxa; 5 species and 1 variety), *Rhynchospora* (6 taxa; 5 species and 1 variety), *Sagittaria* L. (4 taxa), *Juncus* (3 taxa; 3 species, 1 subspecies, 1 variety) and *Smilax* L. (3 taxa).

The most species-rich habit class was herbs (96 taxa; 80 species, 3 subspecies, 13 varieties, 1 hybrid), followed by trees and shrubs (36 taxa; 32 species, 4 varieties), and vines (13 taxa; 11 species, 2 varieties; Fig. 16). Among the herbs, the Poaceae (16 taxa; 13 species, 1 subspecies, 2 varieties), Cyperaceae (14 taxa; 11 species, 3 varieties), Asteraceae (8 taxa; 6 species, 1 variety, 1 hybrid), Alismataceae (4 taxa), Lentibulariaceae (4 taxa), Juncaceae (3 taxa), and Orchidaceae (3 taxa) are the most species-rich families. Among the trees and shrubs, the Ericaceae (3 taxa), Rosaceae (3 taxa), Salicaceae (3 taxa), Betulaceae (2 taxa), Cupressaceae (2 taxa), Nyssaceae (2 taxa), and Sapindaceae (2 taxa) are the most species-rich families.

***Little Singletary Lake***

The littoral zone flora of Littoral Singletary Lake is comprised of 39 taxa (35 species, 1 subspecies, 3 varieties), in 32 genera and 21 vascular plant families (Table 11; Suppl. material 6). All of the 39 total catalogued taxa were vouchered (i.e., no taxa were known strictly from reports or observations; Suppl. material 6). Two species of conservation concern (i.e., *Eleocharis equisetoides* and *Eleocharis vivipara*) were collected from Little Singletary Lake’s littoral zone (Table 9). No exotic taxa are known from this site. Three taxa are unique to Little Singletary Lake (i.e., they were not found/reported from any other bay lake in this study; Suppl. material 6: [*Agrostis hyemalis* (Walter) Britton, Sterns & Poggenb., *Rhexia virginica* L., and *Xyris jupicai* Rich.])

The richest eudicotyledonous genus is *Rhexia* (2 taxa). The richest monocotyledonous families are Cyperaceae (6 taxa; 5 species and 1 variety), Juncaceae (4 taxa; 2 species, 1 subspecies, 1 variety), and Poaceae (3 taxa). The richest monocotyledonous genera are *Juncus* (4 taxa), *Eleocharis* (3 taxa), and *Panicum* (2 taxa).

The most species-rich habit class was herbs (23 taxa; 20 species, 1 subspecies, 2 varieties), followed by trees and shrubs (15 taxa; 14 species and 1 variety), and vines (1 taxon; Fig. 16). Among the herbs, the Cyperaceae (6 taxa), Juncaceae (4 taxa), and Poaceae (3 taxa) are the most species-rich families. Among the trees and shrubs, the Ericaceae (5 taxa) is the most species-rich family.

***Salters Lake***

The littoral zone flora of Salters Lake is comprised of 22 taxa (16 species, 2 subspecies, 4 varieties), in 18 genera and 16 vascular plant families (Table 11; Suppl. material 6). Twenty of the twenty-three total catalogued taxa were vouchered; *Decodon verticillatus*, *Nyssa biflora*, and *Xyris iridifolia*, were reports or personal observations (Suppl. material 6). Two species of conservation concern (i.e., *Xyris iridifolia* and *Xyris smalliana*) were collected/reported from Salters Lake’s littoral zone (Suppl. material 6; Table 9). No exotic taxa are known from this site. One taxon is unique to Salters Lake (i.e., not found/reported from any other bay lake in this study; Suppl. material 5: [*Xyris iridifolia*])

The richest eudicotyledon family is Ericaceae (5 taxa). The richest eudicotyledonous genera are *Lyonia* (2 taxa) and *Vaccinium* (2 taxa). The richest monocotyledonous family is Xyridaceae (2 taxa). The richest monocotyledon genus is *Xyris* (2 taxa).

The most species-rich habit class was trees and shrubs (15 taxa; 11 species, 1 subspecies, 3 varieties), herbs (5 taxa; 4 species and 1 subspecies), and vines (2 taxa; Fig. 16). Among the trees and shrubs, the Ericaceae (5 taxa) and Cupressaceae (2 taxa) are the most species-rich families. Among the herbs, the Xyridaceae (2 taxa) is the most species-rich family.

***Singletary Lake***

The littoral zone vascular flora of Singletary Lake is comprised of 36 taxa (32 species, 1 subspecies, 3 varieties), in 30 genera and 22 vascular plant families (Table 11; Suppl. material 6). All thirty-six total catalogued taxa were vouchered (i.e., none were reports or personal observations; Suppl. material 6). One taxon from Singletary Lake’s littoral zone is of conservation concern (i.e., *Xyris smalliana*; Suppl. material 6; Table 9). No exotic taxa are known from this site. One taxon is unique to Salters Lake (i.e., not found/reported from any other bay lake in this study; Suppl. material 6: [Rhododendron viscosum (L.) Torr. var. serrulatum (Small) H.E. Ahles]).

The richest eudicotyledonous families are Ericaceae (7 taxa) and Rosaceae (2 taxa). The richest eudicotyledonous genus is *Vaccinium* (2 taxa). The richest monocotyledonous families are Juncaceae (3 taxa), Poaceae (2 taxa), and Xyridaceae (2 taxa). The richest monocotyledonous genera are *Juncus* (3 taxa) and *Xyris* (2 taxa).

The most species-rich habit class was trees and shrubs (22 taxa; 19 species and 3 varieties), herbs (11 taxa; 10 species and 1 subspecies), and vines (3 taxa; Fig. 16). Among the trees and shrubs, the Ericaceae (7 taxa), Cupressaceae (3 taxa), Pinaceae (2 taxa), and Rosaceae (2 taxa) are the most species-rich families. Among the herbs, the Juncaceae (3 taxa), Poaceae (2 taxa), and Xyridaceae (2 taxa) are the most species-rich families.

***White Lake***

White Lake was not included in this study due to the severity of the lake’s shoreline development. A provisional checklist of plants known to occur within the littoral zone of White Lake (from historical vouchers, personal observation, and literature review) is provided in Suppl. material 7. The intent of the provisional checklist is to provide a baseline for future research in this lake.

## Materials and methods

This work is restricted to the littoral zone vascular flora of unaltered Carolina bay lake shorelines. The littoral zone was defined as the zone of vegetation occurring between the maximum annual high water mark and the point at which submerged aquatic plants cease to persist (Fig. 4). Unaltered shorelines were defined as those lacking residential or commercial development (therefore, the entirety of White Lake and the developed shorelines of Lake Waccamaw and Bay Tree Lake were not included in this inventory).

During the 2013 and 2014 growing seasons, 36 total visits were made to the eight study sites meeting the criteria articulated above (i.e., Bakers Lake, Bay Tree Lake, Horseshoe Lake, Jones Lake, Lake Waccamaw, Little Singletary Lake, Salters Lake, Singletary Lake), resulting in 121 field hours and the identification of 204 taxa (species, subspecies, and varieties). A 10-foot aluminum boat with a transom-mounted trolling motor was used to transport equipment along Carolina bay lake shorelines. Where water was too shallow for the use of the trolling motor, we walked and pulled the boat by rope. GPS locations (NAD 83) were taken at numerous intervals and associated with all specimens collected within 30 m of each point. Digital photographs of plant habit and overall morphology were taken prior to collection using a Panasonic Lumix FZ−150. Plant specimens were pressed while in the field. Tissue samples were taken in the field and dessicated with blue indicating silica gel (purchased from Delta Enterprises Inc.) in ziploc bags. Voucher specimens and tissue samples were deposited respectively at the North Carolina State University Vascular Plant Herbarium (NCSC) and its DNA bank. The entirety of Carolina bay lake shorelines was surveyed, but it was quickly observed that all shorelines, save for the southernmost, were relatively depauperate. All taxa occurring along western, northern, and eastern shorelines could be found within the littoral zone of the southern shoreline, but the inverse did not hold true. The significantly gentler hydrography (see Frey 1949 for lake longitudinal profiles), and consequently wider littoral zone of southern shorelines, produces a more species-rich macrophyte community. Consequently, survey time was much longer on the southern, more diverse shorelines of Carolina bay lakes.

The flora is organized by the following major vascular plant groups: (1) pteridophytes, (2) gymnosperms, (3) monocots, and (4) basal angiosperms, magnoliids, and eudicotyledons. Dichotomomous keys are provided to each major group, as well as to families, genera, and species within each group. Notes are provided above some keys to aid in the identification process. Within each group, taxa are arranged alphabetically, by family, then genus, then species.

The following information is provided for each taxon account: taxon concept mapping, basionym, conservation status, habit, habitat, flowering and fruiting phenology, abundance, and presence/absence data for each site (Suppl. material 3). Unless stated otherwise, accepted taxon concepts follow Weakley (2012) and are tied to those in the following major works: RAB = Radford et al. (1968); GW = Godfrey and Wooten (1979), Godfrey and Wooten (1981); FNA = *Flora of North America* (pteridophytes: Blechnaceae [Cranfill 1993], Dryopteridaceae [Smith 1993b, Wagner and Montgomery 1993, Smith 1993b], Lycopodiaceae [Wagner and Beitel 1993], Osmundaceae [Whetstone and Atkinson 1993, Polypodiaceae [Andrews and Windham 1993]; gymnosperms: Cupressaceae [Michener 1993, Watson 1993, Watson and Eckenwalder 1993], Pinaceae [Kral 1993]; monocots: Alismataceae [Durand 2000], Araceae [Thompson 2000], Bromeliaceae [Luther and Brown 2000], Burmanniaceae [Lewis 2002], Cyperaceae [Ball and Reznicek 2002, Ball et al. 2002b, Kral 2002a, Kral 2002b, Kral and Persoon 2002, Mastrogiuseppe 2002, Mastrogiuseppe et al. 2002, Reznicek 2002, Reznicek and Catling 2002, Smith et al. 2002, Tucker 2002, Tucker et al. 2002], Eriocaulaceae [Kral 2000a], Haemodoraceae [Robertson 2002], Hydrocharitaceae [Haynes 2000a, Haynes 2000b], Hypoxidaceae [Herndon 2002], Juncaceae [Brooks and Clemants 2000], Mayacaceae [Faden 2000], Orchidaceae [Goldman et al. 2002, Hágsater et al. 2002, Romero-Gonzáles et al. 2002, Sheviak 2002, Sheviak and Brown 2002, Sheviak and Catling 2002], Poaceae [Barkworth 2003a, Barkworth 2003b, Campbell 2003, Clark and Triplett 2007, Daniel 2007, Freckmann and Lelong 2003a, Freckmann and Lelong 2003b, Harvey 2007, Peterson 2003, Terrell 2007, Wipff 2003], Pontederiaceae [Adanson et al. 2002], Smilacaceae [Holmes 2002], Xyridaceae [Kral 2000a]; basal angiosperms, magnoliids, and eudicots: Altingiaceae [Meyer 1997a], Amaranthaceae [Clemants 2003], Asteraceae [Bogler 2006, Chambers and O'Kennon 2006, Haines 2006, Holmes 2006, Karaman-Castro and Urbatsch 2006, Lamont 2006, Nesom 2006a, Nesom 2006b, Semple and Cook 2006, Siripun and Schilling 2006, Strother and Weedon 2006, Sundberg and Bogler 2006], Betulaceae [Furlow 1997], Cabombaceae [Wiersema 1997b], Caryophyllaceae [Swanson and Rabeler 2005], Clethraceae [Tucker and Jones 2009], Cyrillaceae [Lemke 2009], Ebenaceae [Eckenwalder 2009], Ericaceae [Dorr 2009, Fabijan 2009, Judd 2009, Judd and Kron 2009, Tucker 2009b, Tucker 2009a, Vander Kloet, S.P. 2009], Fagaeae [Jensen 1997], Iteaceae [Morin 2009], Juglandaceae [Stone 1997], Lauraceae [Wofford 1993], Magnoliaceae [Meyer 1997b], Myricaceae [Bornstein 1997], Nelumbonaceae [Wiersema 1997a], Nymphaeaceae [Wiersema and Hellquist 1997], Platanaceae [Kaul 1997], Polygonaceae [Mosyaking 2005], Ranunculaceae [Pringle 1997], Salicaceae [Argus et al. 2010], Sarraceniaceae [Mellichamp and Chase 2009], Theaceae [Prince 2009], Ulmaceae [Sherman-Broyles 1997]). Three symbols are used to relate whether our taxon concepts used here are equivalent (=), narrower (<), or broader (>) than those of other works. For example, the statement “= RAB, FNA” means that the taxon concept, as well as the species name used here, is the same as that used in RAB and FNA (see *Dryopteris ludoviciana* (Kunze) Small). The use of a “less than” symbol (e.g., “< *Onoclea sensibilis* L. – RAB, FNA”), indicates that the taxon concept used here is narrower than that used by RAB and FNA (alternatively, a “greater than” symbol would mean that the concept of a particular taxon is broader than in the cited works). An equals symbol followed by a different species name than the one bolded, indicates that the taxon concept used here is the same as in the work cited, except that the taxon was treated under a different name in the work cited (see *Sagittaria filiformis* J.G. Sm. vs. *Sagittaria stagnorum* Small).

Abundance estimates following the recommendations of Palmer et al. (1995) are provided for each lake in which a taxon was collected or observed by the current author (Table 12; Suppl. material 3). Taxa designated as “exotic” are not native to North America and are indicated by an asterisk preceding the scientific name. The conservation status and rank of species of conservation concern precede the habitat description in each taxon entry (e.g., E, FSC; S1, G2. “Habitat description”). Conservation status and rank of species are designated according to NatureServe (2012), the North Carolina Plant Conservation Program (2010), and the North Carolina Natural Heritage Program List of Rare Plants (Robinson and Finnegan 2014). Unvouchered taxa (i.e., those known only from reports or personal observations) are given one of four symbols in taxon entries (• = the first author observed the species while in the field, but was not able to collect a viable voucher specimen, ♦ = the taxon was reported by the Carolina Vegetation Survey (Peet et al. 2013a, Peet et al. 2013b), = ► the taxon was reported by the North Carolina Natural Heritage Program (North Carolina Natural Heritage Program 2014), ¤ = the taxon was reported by the North Carolina State University Crop Science Program; Rob Richardson and Justin Nawrocki, pers. comm, April 9, 2015).

When available, digital photographs and line drawings were obtained from: Britton and Brown (1913), Center for Aquatic and Invasive Plants, University of Florida, IFAS (2015), Hitchcock and Chase (1951), Mickel (1979), and United States Department of Agriculture, Natural Resources Conservation Service (USDA- NRCS) (2015).

In addition, relevant historical vouchers are cited based on systematic searches of the three major herbaria−DUKE, NCSC, and NCU. Unfortunately, it is not uncommon to find historical specimens containing vague habitat or locality descriptions. For a taxon to be included in the present study, a clear label statement referencing Carolina bay lake shoreline habitat was required (e.g., “collected from peat-drained lake bed of Suggs Mill Pond”). Herbarium vouchers meeting this criterion were annotated (following taxon concepts accepted here) and their label information was subsequently entered into spreadsheets for organization. Label information for new collections resulting from this study was captured in a DarwinCore compliant spreadsheet for upload to the online portal of the Southeastern Regional Network of Expertise and Collections (www.sernecportal.org), which feeds into iDigBio and the Global Biodiversity Data Facility (GBIF).

## Checklists

### PTERIDOPHYTES

#### Families represented: 6

### Blechnaceae

#### Anchistea virginica

(L.) C. Presl

Anchistea virginica Basionym: *Blechnum virginicum* L.
Anchistea virginica Taxon concept: [= *Woodwardia virginica* (L.) Sm. − RAB, FNA, Weakley]

##### Distribution

Bakers Lake (Infrequent): *Howell BALA−14* (NCSC!)

Bay Tree Lake (Occasional): *Howell BATR−4, 24* (NCSC!)

Jones Lake (Rare): *Howell JOLA−44* (NCSC!)

Lake Waccamaw (Infrequent): *Howell LAWA−59* (NCSC!)

Little Singletary Lake (Occasional): *Howell LISI−42* (NCSC!)

##### Notes

Perennial herbs. Upper eulittoral zone; typically found in saturated soils or rooted on logs, stumps, and other debris (NLSS–C, NLSS–LW, NLSM–T). Jun–Sep. Fig. 18

#### Lorinseria areolata

(L.) C. Presl

Lorinseria areolata Basionym: *Acrostichum areolatum* L.
Lorinseria areolata Taxon concept: [= *Woodwardia areolata* (L.) T. Moore − RAB, FNA, Weakley]

##### Distribution

Bay Tree Lake (Occasional): *Howell BATR−5, 26* (NCSC!)

Lake Waccamaw: *Wilbur 84200* (DUKE!)

Little Singletary Lake (Occasional): *Howell LISI–6* (NCSC!)

Singletary Lake: *Hueske* *s.n.* (NCU!)

##### Notes

Perennial herbs. Upper eulittoral zone; typically found in saturated soils or rooted on logs, stumps, and other debris (NLSS–C, NLSS–LW, NLSM–T) . May–Sep. Fig. 19

### Dryopteridaceae

#### Dryopteris ludoviciana

(Kunze) Small

Dryopteris ludoviciana Basionym: *Aspidium ludovicianum* Kunze
Dryopteris ludoviciana Taxon concept: [= RAB, FNA, Weakley]

##### Distribution

Lake Waccamaw: *Bennedict 1247 & 2298* (NCU!); *Blomquist & Correll 7625* (NCU!)

##### Notes

**Figure 20.**
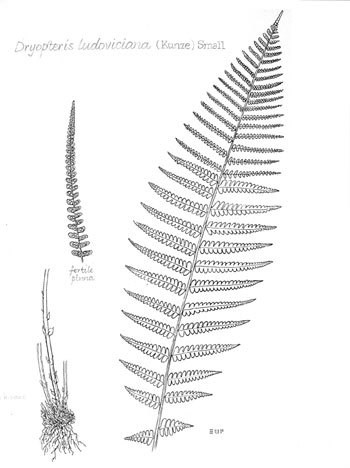
*Dryopteris ludoviciana* (from Mickel 1979)

Perennial herbs. Juncture of eulittoral and supralittoral zones (NLSS–LW). Jun–Sep. This species was not encountered by the first author, but voucher specimens (see above) place it within close proximity of Lake Waccamaw’s shoreline (i.e., it has the potential to occur at the uppermost portions of the littoral zone where the swamp forest adjoins the shoreline community on the southwest side of the lake). Fig. 20

### Lycopodiaceae

#### Lycopodiella appressa

(Chapm.) Cranfill

Lycopodiella appressa Basionym: Lycopodium inundatum L. var. appressum Chapm.
Lycopodiella appressa Taxon concept: [= *Lycopodium appressum* (Chapm.) F.E. Lloyd & Underw. − RAB; = FNA, Weakley]

##### Distribution

Bay Tree Lake: *Wilbur 48656* (DUKE!)

Horseshoe Lake (Infrequent): *Howell HOLA−52* (NCSC!)

Lake Waccamaw (Infrequent): *Howell LAWA−110* (NCSC!)

##### Notes

Perennial herbs. Upper eulittoral zone; usually in association with saturated peaty to sandy soils (NLSS–LW, CPSI–CG). Jul–Sep. Fig. 21

### Onocleaceae

#### Onoclea sensibilis

L.

Onoclea sensibilis Taxon concept: [< *O. sensibilis* L. – RAB, FNA; = Weakley]

##### Distribution

Lake Waccamaw: *Wilbur 84220* (DUKE!)

##### Notes

**Figure 22.**
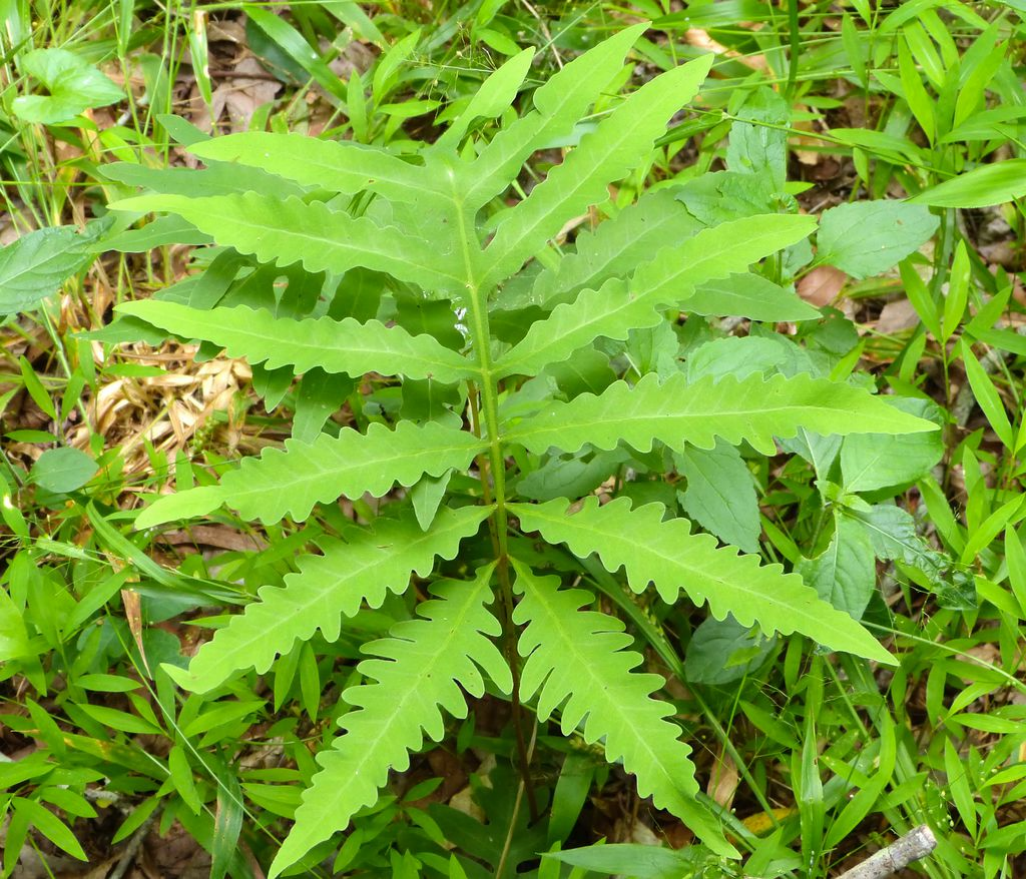
*Onoclea sensibilis* (digital photograph taken by Nathan Howell)

Perennial herbs. Upper eulittoral zone (NLSS−LW). May−Jun. This species was not encountered by the first author in the field, but a single voucher (see above) places it within close proximity to Lake Waccamaw’s southwest shoreline. Fig. 22

### Osmundaceae

#### Osmunda spectabilis

Willd.

Osmunda spectabilis Taxon concept: [< O. regalis L. var. spectabilis (Willd.) A. Gray − RAB, FNA; = Weakley]

##### Distribution

Lake Waccamaw (Infrequent): *Howell LAWA−58, 87, 90* (NCSC!)

##### Notes

Perennial herbs. Eulittoral zone; sometimes establishing itself on old stumps and logs (NLSS–LW). Mar–Jun. Fig. 23

### Polypodiaceae

#### Pleopeltis polypodioides michauxianavar.michauxiana

(Weath.) E.G. Andrews & Windham

Pleopeltis polypodioides michauxianavar.michauxiana Basionym: Polypodium polypodioides (L.) Watt var. michauxianum Weath.
Pleopeltis polypodioides michauxianavar.michauxiana Taxon concept: [< *Polypodium polypodioides* (L.) Watt – RAB; = FNA, Weakley]

##### Distribution

Lake Waccamaw (Infrequent): *Howell LAWA−47* (NCSC!)

Salters Lake (Infrequent): *Howell SALA−1* (NCSC!)

##### Notes

Perennial, frequently epiphytic, herbs. Eulittoral zone; commonly on large limbs and trunks of *Taxodium* and *Nyssa* (NLSS−C, NLSS−LW). Jun−Oct. Fig. 24

### GYMNOSPERMS

#### Families represented: 2

### Cupressaceae

#### Chamaecyparis thyoides

(L.) Britton, Sterns, & Poggenb.

Chamaecyparis thyoides Basionym: *Cupressus thyoides* L.
Chamaecyparis thyoides Taxon concept: [= RAB, FNA, Weakley]

##### Distribution

Bay Tree Lake (Infrequent): *Howell BATR−2* (NCSC!)

Horseshoe Lake (Occasional): *Howell HOLA−2, 13* (NCSC!)

Jones Lake (Occasional): *Brown* *s.n.* (NCSC!); *Howell JOLA−1, 23* (NCSC!); *Lance* *s.n.* (NCU!); *Russell 1304* (NCSC!)

Little Singletary Lake (Occasional): *Howell LISI−8, 26* (NCSC!)

Singletary Lake (Infrequent): *Howell SILA−14* (NCSC!)

##### Notes

Trees. At or just below the juncture of the supralittoral and eulittoral zones; often in saturated peaty or sandy soil (NLSS–C, NLSS–LW, NLSM–T). Mar–Apr; Oct– Nov. Fig. 25

#### Taxodium ascendens

Brongn.

Taxodium ascendens Taxon concept: [= RAB; < T. distichum L. var. imbricarium (Nutt.) Croom − FNA; = Weakley]

##### Distribution

Bakers Lake (Abundant): *Howell BALA−15* (NCSC!)

Bay Tree Lake (Abundant): *Howell BATR−7* (NCSC!)

Horseshoe Lake (Abundant): *Howell HOLA−10* (NCSC!)

Jones Lake (Abundant): *Howell JOLA−3, 22* (NCSC!); *Krings 508* (NCSC!); *Wilbur 57584* (DUKE!)

Lake Waccamaw (Abundant): *Howell LAWA−13* (NCSC!)

Little Singletary Lake (Abundant): *Howell LISI−4, 20* (NCSC!)

Salters Lake (Abundnat): *Howell SALA−8* (NCSC!)

Singletary Lake (Abundant): *Howell SILA−13* (NCSC!); *Wilbur 27966* (DUKE!)

##### Notes

Trees. Eulittoral zone (NLSS–C, NLSS–LW, NLSM–T, NLSM−LWP, CPSI–CG). Mar– Apr; Oct. Fig. 26

#### Taxodium distichum

(L.) Rich.

Taxodium distichum Basionym: *Cupressus disticha* L.
Taxodium distichum Taxon concept: [= RAB; < T. distichum (L.) Rich. var. distichum – FNA; = Weakley]

##### Distribution

Bay Tree Lake: *Wilbur 61464* (DUKE!)

Jones Lake: *Stone 3704* (DUKE!)

Lake Waccamaw: ♦

Salters Lake: *Beckman & Linnenburger 38* (DUKE!)

Singletary Lake: *Crosby 4032* (DUKE!)

##### Notes

**Figure 27.**
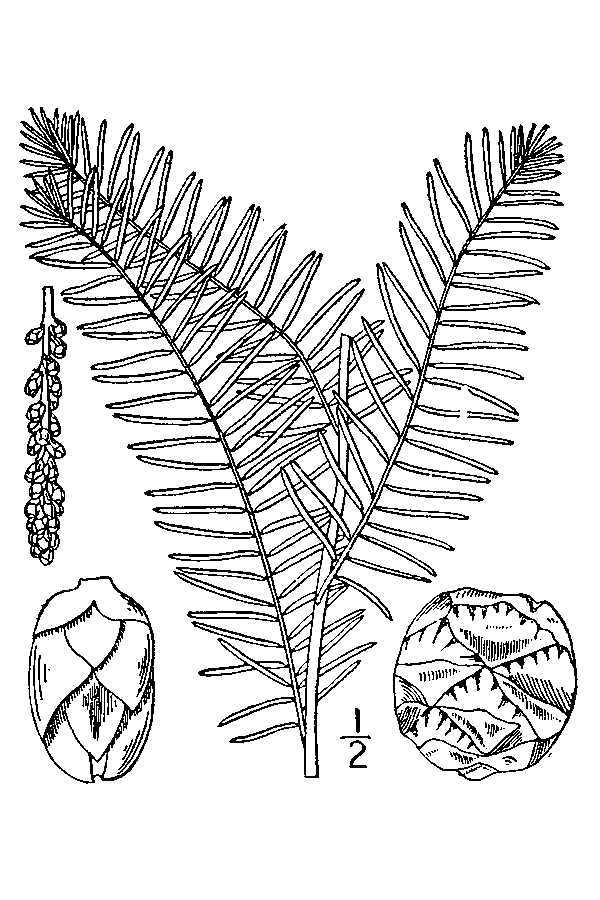
*Taxodium distichum* (from Britton and Brown 1913)

Trees. Eulittoral zone (NLSS–C, NLSS–LW, NLSM−T). Infrequent. Mar–Apr; Oct. Fig. 27

### Pinaceae

#### Pinus serotina

Michx.

Pinus serotina Taxon concept: [= RAB, FNA, Weakley]

##### Distribution

Jones Lake (Rare): *Howell JOLA−14* (NCSC!)

Singletary Lake (Rare): *Howell SILA−37* (NCSC!)

##### Notes

Trees. Juncture of supralittoral and eulittoral zones (NLSS–C). Apr–Aug (or any time of the year in response to fire). Fig. 28

#### Pinus taeda

L.

Pinus taeda Taxon concept: [= RAB, FNA, Weakley]

##### Distribution

Lake Waccamaw (Rare): *Howell LAWA−71* (NCSC!)

Little Singletary Lake (Infrequent): *Howell LISI−27* (NCSC!)

Singletary Lake (Rare): *Howell SILA−12* (NCSC!)

##### Notes

Trees. Juncture of supralittoral and eulittoral zones (NLSS–C, NLSS–LW). Mar–Apr; Oct– Nov. Fig. 29

### MONOCOTYLEDONS

#### Families represented: 17

### Alismataceae

#### Sagittaria filiformis

J.G. Sm.

Sagittaria filiformis Taxon concept: [= *S. stagnorum* Small – GW; S. subulata L. Buchenau var. gracillima (S. Watson) J.G. Sm.; = FNA, Weakley]

##### Ecological interactions

###### Conservation status

SR−P; SH, G4G5.

##### Distribution

Lake Waccamaw: *Blomquist & Schuster 16191* (DUKE!)

##### Notes

**Figure 30.**
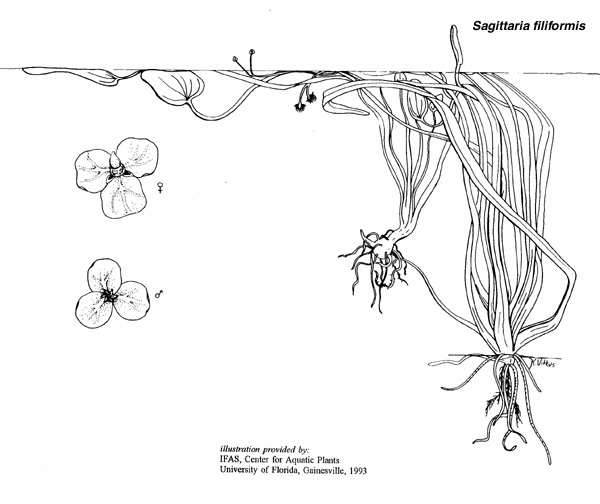
*Sagittaria filiformis* (from Center for Aquatic and Invasive Plants, University of Florida, IFAS 2015)

Perennial herbs. Eulittoral and infralittoral zones (NLSS−LW, NLSM−LWP). May−Sep. The first author has not encountered this taxon in the field, but a single voucher specimen (see above) confirms its historic presence within the lake. Fig. 30

#### Sagittaria graminea

Michx.

Sagittaria graminea Taxon concept: [= S. graminea Michx. var. graminea – RAB, GW; = S. graminea Michx. ssp. graminea – FNA; = Weakley]

##### Distribution

Lake Waccamaw (Frequent): *Howell LAWA−19, 57* (NCSC!); *Radford* *s.n.* (NCU!); ♦

##### Notes

**Figure 31.**
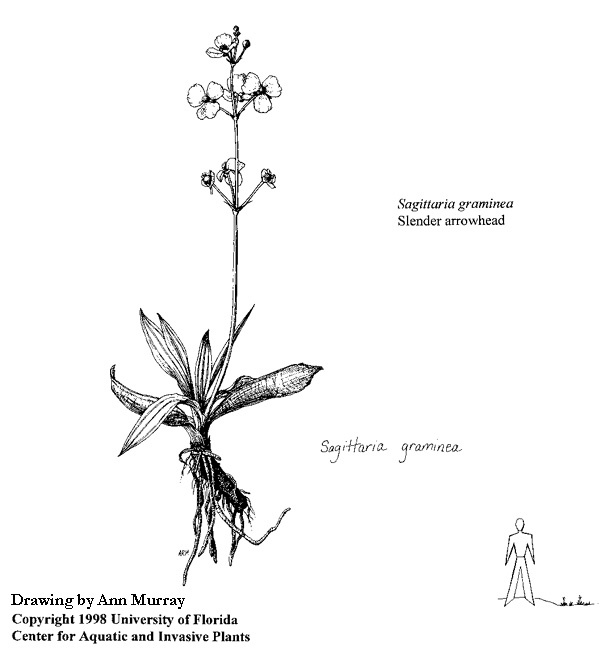
*Sagittaria graminea* (from Center for Aquatic and Invasive Plants, University of Florida, IFAS 2015)

Perennial herbs. Eulittoral zone (NLSS−LW, NLSM−LWP). May−Nov. Fig. 31

#### Sagittaria isoetiformis

J.G. Sm.

Sagittaria isoetiformis Taxon concept: [< *S. teres* S. Watson (misapplied) – RAB; = GW, FNA, Weakley]

##### Ecological interactions

###### Conservation status

State T; S2, G4?.

##### Distribution

Horseshoe Lake (Infrequent): *Grant* *s.n.* (NCU!); *Howell HOLA−34* (NCSC!)

Lake Waccamaw: *LeBlond 5792D* (NCU!)

##### Notes

Perennial herbs. Eulittoral zone (NLSS−LW, NLSM−LWP, CPSI−CG, FB). Jun−Sep. Fig. 32

#### Sagittaria weatherbiana

Fernald

Sagittaria weatherbiana Taxon concept: [= S. graminea Michx. var. weatherbiana – RAB, GW; = S. graminea Michx. ssp. weatherbiana – FNA; = Weakley]

##### Ecological interactions

###### Conservation status

State E, FSC; S2, G3G4.

##### Distribution

Lake Waccamaw: *Adams* *s.n.* (NCSC!)

##### Notes

Perennial herbs. Eulittoral zone (NLSS−LW, NLSM−LWP). Apr−Jun.

### Araceae

#### Colocasia esculenta

(L.) Schott

Colocasia esculenta Basionym: *Arum esculentum* L.
Colocasia esculenta Taxon concept: [= GW, FNA, Weakley]

##### Distribution

Lake Waccamaw (Infrequent): *Howell LAWA−93* (NCSC!)

##### Notes

Perennial herbs. Eulittoral zone (NLSS−LW, NLSM−LWP). “Generally infertile in our area” (Weakley 2012). This species is exotic and has become naturalized in roadside ditches, canals, and portions of the lakes shoreline. It spreads by way of rhizome dispersal, which is almost cartainly caused by residential homeowners digging up rhizomes from their flower beds and either tossing them into the lake or into the canals that surround the lake. Fig. 33

#### Wolffia

Horkel ex Schleid.

##### Notes

The first author has not encountered taxa within this genus in the field; however, the Carolina Vegetation Survey reported “*Wolffia* spp.” from the southwest side Lake Waccamaw. Although a species-level identification has not been made, a key to the two species most likely to inhabit this location is provided in the Identification Keys section below.

### Bromeliaceae

#### Tillandsia usneoides

(L.) L.

Tillandsia usneoides Basionym: *Renealmia usneoides* L.
Tillandsia usneoides Taxon concept: [= RAB, FNA, Weakley]

##### Distribution

Bakers Lake (Occasional): •

Bay Tree Lake (Frequent): *Howell BATR−49* (NCSC!)

Horseshoe Lake (Occasional): •

Jones Lake (Occasional): *Howell JOLA–33* (NCSC!)

Lake Waccamaw (Abundant): *Howell LAWA−46, 84* (NCSC!)

Little Singletary Lake (Occasional): *Howell LISI−18* (NCSC!)

Salters Lake (Occasional): *Howell SALA−9* (NCSC!)

Singletary Lake (Frequent): *Howell SILA−6, 20* (NCSC!)

##### Notes

Perennial, epiphytic herbs. Eulittoral zone; common in low-hanging limbs of *Taxodium* or *Nyssa* (NLSS−C, NLSS−LW, NLSM−T, CPSI−CG). Apr−Jun. Fig. 34

### Burmanniaceae

#### Burmannia capitata

(Walter ex J.F. Gmel.) Mart.

Burmannia capitata Basionym: *Vogelia capitata* Walter ex J.F. Gmel.
Burmannia capitata Taxon concept: [= RAB, GW, FNA, Weakley]

##### Distribution

Lake Waccamaw: *LeBlond & Franklin 6578* (NCU!)

##### Notes

**Figure 35.**
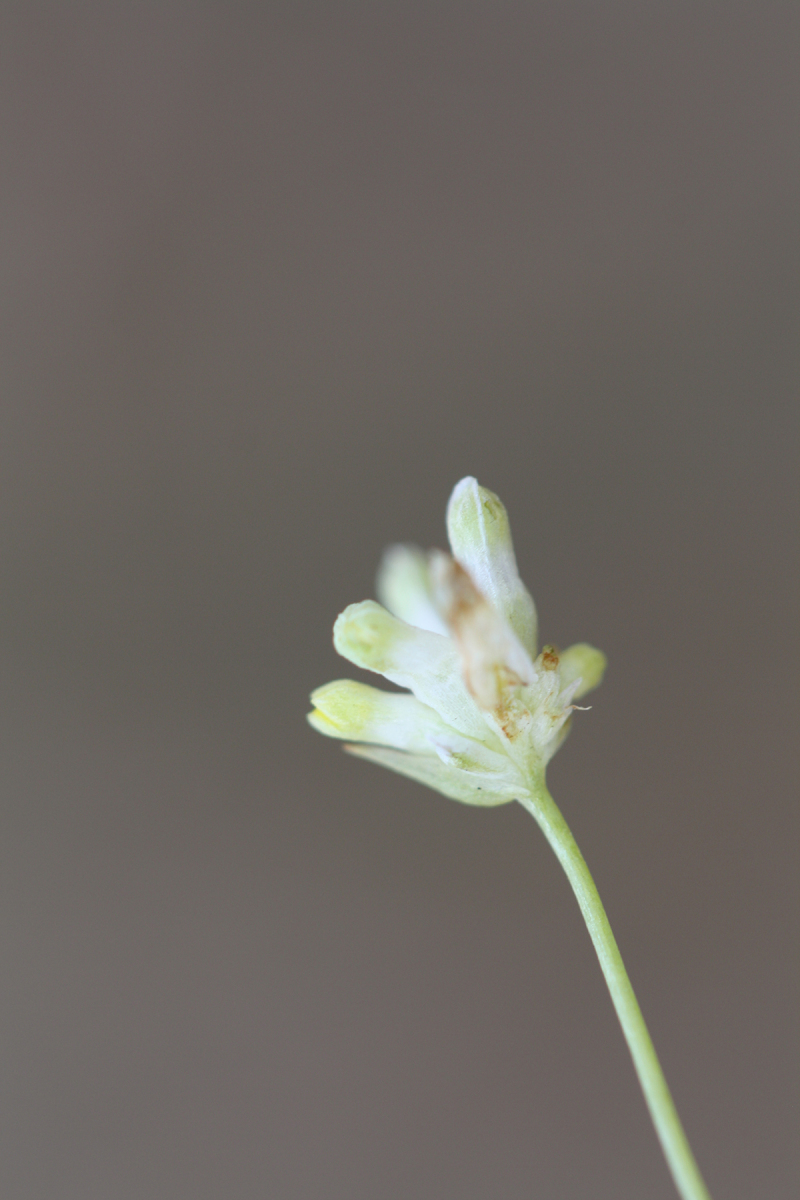
*Burmannia capitata* (digital photograph taken by Alexander Krings)

Annual herbs. Eulittoral zone (NLSS−LW). Jul−Nov. Fig. 35

### Cyperaceae

#### Carex alata

Torr.

Carex alata Taxon concept: [= RAB, GW, FNA, Weakley]

##### Distribution

Lake Waccamaw (Rare): *Howell LAWA−98* (NCSC!)

##### Notes

Perennial herbs. Eulittoral zone; usually at or just below the juncture of the supralittoral and eulittoral zones (NLSS−LW). May−Jun. Fig. 36

#### Carex longii

Mack.

Carex longii Taxon concept: [< *C. albolutescens* Schwein. – RAB, GW; = FNA, Weakley]

##### Distribution

Bay Tree Lake (Rare): *Howell BATR−34* (NCSC!)

##### Notes

Perennial herbs. Eulittoral zone; typically at or just below the juncture of the supralittoral and eulittoral zones. May−Jun. Fig. 37

#### Carex lupulina

Muhl. ex Willd.

Carex lupulina Taxon concept: [= RAB; < *C. lupulina* Muhl. ex Willd. – GW (see *C. lupuliformis*); = FNA, Weakley]

##### Distribution

Lake Waccamaw (Rare): *Howell LAWA−136* (NCSC!)

##### Notes

Perennial herbs. Juncture of the eulittoral and supralittoral zones (NLSS−LW). Jun−Sep. A taxon of bottomland forests throughout the state, this large-fruited sedge occurs where bottomland swamp forests abut the shoreline of Lake Waccamaw. Fig. 38

#### Carex striatavar.brevis

L.H. Bailey

Carex striatavar.brevis Taxon concept: [< *C. walteriana* L.H. Bailey – RAB, GW; = FNA, Weakley]

##### Distribution

Horseshoe Lake: *Buell 2279* (DUKE!, NCSC!)

##### Notes

**Figure 39.**
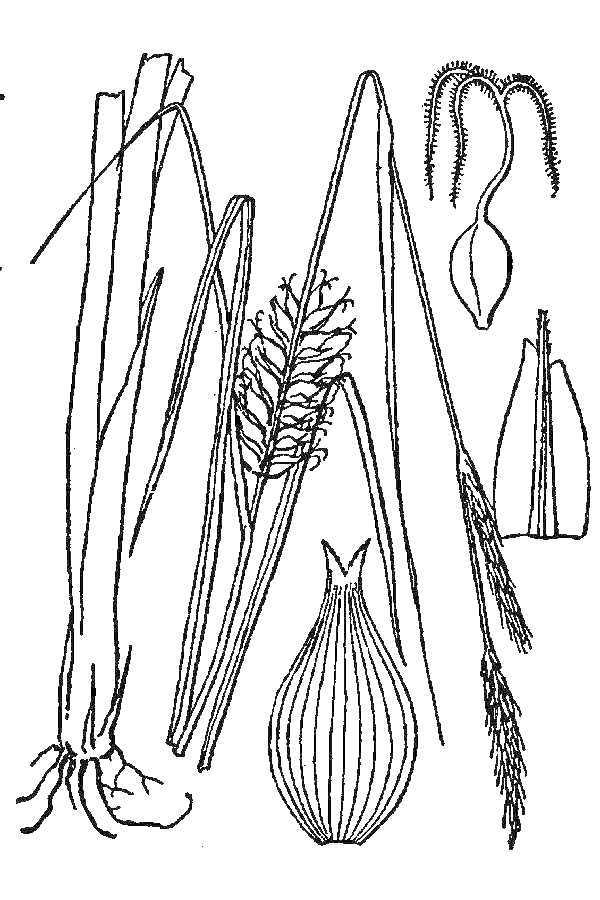
Carex striata var. brevis (from Britton and Brown 1913)

Perennial herbs. Eulittoral zone; typically in acidic, saturated, peaty soils (CPSI−CG, FB). May−Jun. Fig. 39

#### Cladium mariscoides

(Muhl.) Torr.

Cladium mariscoides Basionym: *Schoenus mariscoides* Muhl.
Cladium mariscoides Taxon concept: [= RAB, FNA, Weakley]

##### Ecological interactions

###### Conservation status

SR–O; S3, G5.

##### Distribution

Lake Waccamaw (Abundant): *Howell LAWA−16, 146* (NCSC!); *LeBlond 3862* (NCU!); *Wilbur 49778, 49789* (DUKE!)

##### Notes

Perennial herbs. Eulittoral zone (NLSS−LW). Jul−Sep. This taxon is the principal sedge component of the natural shoreline community of Lake Waccamaw. Fig. 40

#### Cyperus erythrorhizos

Muhl.

Cyperus erythrorhizos Taxon concept: [= RAB, GW, FNA, Weakley]

##### Distribution

Horseshoe Lake: *Buell 2263* (DUKE!); *Rothfels*, *Burge*, *Duke Natural History Society 2403* (DUKE!)

##### Notes

**Figure 41.**
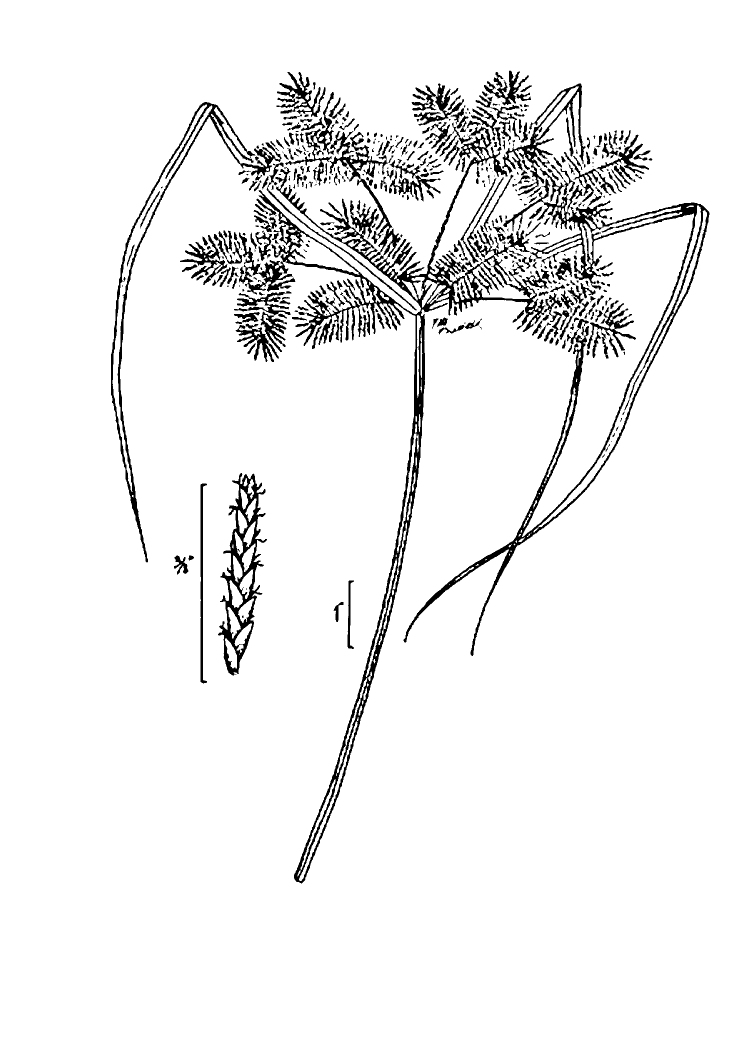
*Cyperus erythrorhizos* (from United States Department of Agriculture, Natural Resource Conservation Service PLANTS Database / United States Department of Agriculture, Natural Resource Conservation Service 2015)

Annual herbs. floating bogs; saturated, acidic, peaty soil (FB). Jul−Sep. Fig. 41

#### Cyperus odoratusvar.odoratus

Cyperus odoratusvar.odoratus Taxon concept: [= *C. odoratus* L. – RAB, GW; < *C. odoratus* L. – FNA; = Weakley]

##### Distribution

Bay Tree Lake (Rare): *Howell BATR−63* (NCSC!)

##### Notes

Annual or short-lived perennial herbs. Eulittoral zone; typically on moist sandy beaches at or just below the maximum annual high water mark. Jul−Sep. Fig. 42

#### Cyperus polystachyos

Rottb.

Cyperus polystachyos Taxon concept: [> C. polystachyos Rottb. var. texensis (Torr.) Fernald – RAB; < *C. polystachyos* – GW; = FNA, Weakley]

##### Distribution

Jones Lake (Rare): *Howell JOLA−43* (NCSC!)

##### Notes

Perennial herbs. Eulittoral zone; usually in sandy moist soil just below the maximum annual high water mark (NLSM−T). Jul−Oct. Fig. 43

#### Dulichium arundinaceumvar.arundinaceum

Dulichium arundinaceumvar.arundinaceum Basionym: *Cyperus arundinaceus* L.
Dulichium arundinaceumvar.arundinaceum Taxon concept: [< *D. arundinaceum* (L.) Britton – RAB, GW; = FNA, Weakley]

##### Distribution

Horseshoe Lake (Occasional): *Beal 4345* (NCSC!); *Buell* *s.n.* (DUKE!); *Howell HOLA−32* (NCSC!)

Lake Waccamaw (Occasional): *Howell LAWA−26, 77* (NCSC!)

Little Singletary Lake (Infrequent): *Howell LISI−41* (NCSC!)

##### Notes

Perennial herbs. Eulittoral zone; calm, quiet waters along shorelines or on floating bogs (NLSS−C, NLSS−LW, CPSI−CG, FB). Jul–Oct. Fig. 44

#### Eleocharis baldwinii

Chapm.

Eleocharis baldwinii Taxon concept: [= RAB, GW, FNA, Weakley]

##### Distribution

Bay Tree Lake (Infrequent): *Howell BATR−36, 40* (NCSC!)

Horseshoe Lake: • (The first author has observed *Eleocharis baldwinii/vivipara* around the peripheries of floating bogs and along saturated peaty shores, but voucher specimens were not collected. These two species are unidentifiable from a distance and the use of a hand lens is needed to distinguish one from the other.)

Little Singletary Lake (Rare): *Howell LISI−43* (NCSC!)

##### Notes

Annual (?) herbs. Eulittoral zone and infralittoral zones; typically submersed in shallow water or on sarurated organic to sandy soils above current lake levels (NLSS−C, NLSM−T). Jul−Sep. Fig. 45

#### Eleocharis equisetoides

(Elliott) Torr.

Eleocharis equisetoides Basionym: *Scirpus equisetoides* Elliott
Eleocharis equisetoides Taxon concept: [= RAB, GW, FNA, Weakley]

##### Ecological interactions

###### Conservation status

W1; S3, G4.

##### Distribution

Lake Waccamaw (Infrequent): *Howell LAWA−67, 155* (NCSC!)

Little Singletary Lake (Rare): *Howell LISI−38* (NCSC!)

##### Notes

Perennial herbs. Eulittoral and infralittoral zones; calm, quiet waters along shorelines (NLSS−C, NLSS−LW). Jun−Sep. Fig. 46

#### Eleocharis olivaceavar.olivacea

Eleocharis olivaceavar.olivacea Taxon concept: [< *E. flavescens* (Poir.) Urb. – RAB; < *E. olivacea* Torr. – GW; < E. flavescens (Poir.) Urb. var. olivacea (Torr.) Gleason – FNA; = Weakley]

##### Distribution

Lake Waccamaw (Rare): *Howell LAWA−78* (NCSC!); *LeBlond 3987* (NCU!)

##### Notes

Perennial herbs. Eulittoral and infralittoral zones; calm, quiet waters along shorelines (NLSS−LW). Jun−Sep. Fig. 47

#### Eleocharis vivipara

Link

Eleocharis vivipara Taxon concept: [= RAB, GW, FNA, Weakley]

##### Ecological interactions

###### Conservation status

State E; S1, G5.

##### Distribution

Horseshoe Lake: • (The first author has observed *Eleocharis baldwinii/vivipara* around the peripheries of floating bogs and along saturated peaty shores, but voucher specimens were not collected. These two species are unidentifiable from a distance and the use of a hand lens is needed to distinguish one from the other.)

Little Singletary Lake (Rare): *Howell LISI−53* (NCSC!)

##### Notes

Perennial herbs. Eulittoral zone (calm, quiet waters) or boggy, saturated, organic soils at or just below the maximum annual high water mark (NLSS−C, NLSM−T). Jul−Sep. Fig. 48

#### Fimbristylis autumnalis

(L.) Roem. & Schult.

Fimbristylis autumnalis Basionym: *Scirpus autumnalis* L.
Fimbristylis autumnalis Taxon concept: [= RAB, GW, FNA, Weakley]

##### Distribution

Lake Waccamaw: *Radford 677* (NCU!)

##### Notes

**Figure 49.**
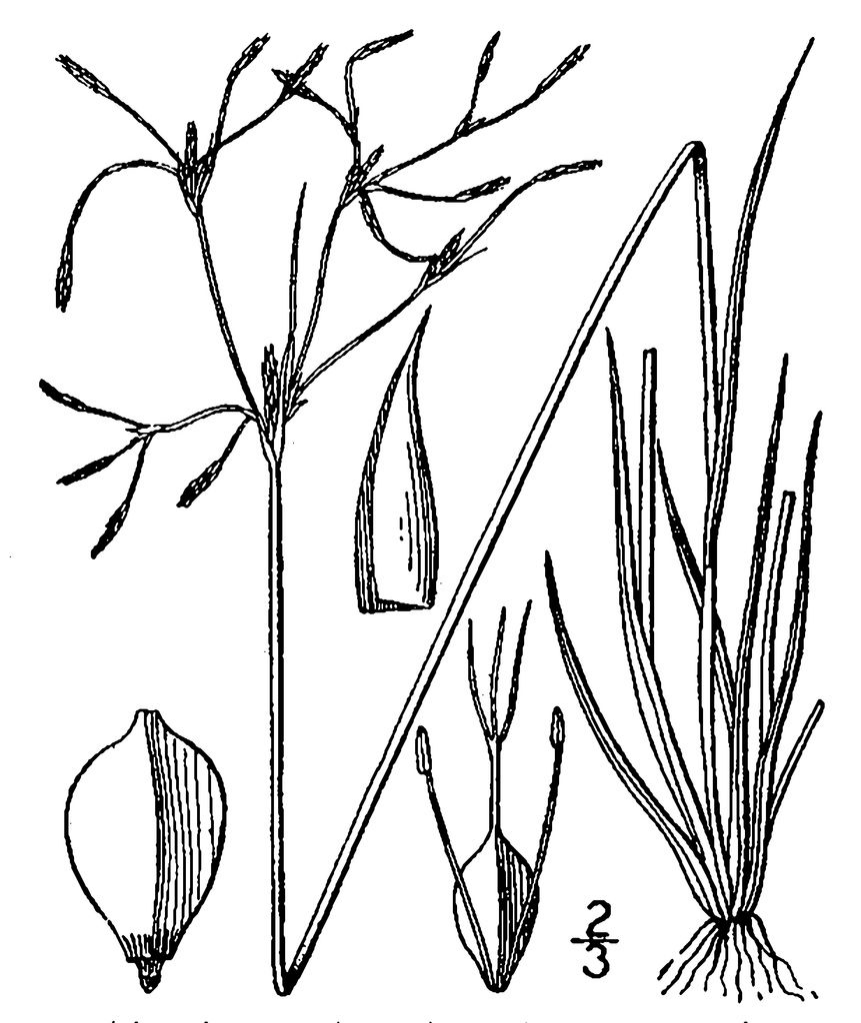
*Fimbristylis autumnalis* (from Britton and Brown 1913)

Annual herbs. Eulittoral zone; wet, sandy, disturbed areas (NLSS−LW). Jun−Oct. Fig. 49

#### Fuirena pumila

(Torr.) Spreng.

Fuirena pumila Basionym: Fuirena squarrosa Michx. var. pumila Torr.
Fuirena pumila Taxon concept: [= RAB, GW, FNA, Weakley]

##### Distribution

Bay Tree Lake (Rare): *Howell BATR−62* (NCSC!); *Wilbur 57396* (DUKE!)

##### Notes

Annual herbs. Eulittoral zone; typically in moist sandy soil at high water mark. Jul−Oct. Fig. 50

#### Rhynchospora alba

(L.) Vahl

Rhynchospora alba Basionym: *Schoenus albus* L.
Rhynchospora alba Taxon concept: [= RAB, GW, FNA, Weakley]

##### Ecological interactions

###### Conservation status

SR−P; S2, G5.

##### Distribution

Horseshoe Lake (Occasional): *Howell HOLA−45* (NCSC!)

##### Notes

Perennial herbs. floating bogs of Horseshoe Lake (FB). Jul−Oct. Fig. 51

#### Rhynchospora corniculata

(Lam.) A. Gray

Rhynchospora corniculata Basionym: *Schoenus corniculatus* Lam.
Rhynchospora corniculata Taxon concept: [= RAB, GW, FNA; < R. corniculata (L.) A. Gray var. corniculata − Weakley]

##### Distribution

Lake Waccamaw (Occasional): *Howell LAWA−135, 163* (NCSC!)

##### Notes

Perennial herbs. Eulittoral zone (NLSS−LW). Jul−Sep. Fig. 52

#### Rhynchospora distans

(Michx.) Vahl

Rhynchospora distans Basionym: *Schoenus distans* Michx.
Rhynchospora distans Taxon concept: [< *R. fascicularis* (Michx.) Vahl – RAB, GW, FNA; = Weakley]

##### Distribution

Bakers Lake (Rare): *Howell BALA−2* (NCSC!)

Lake Waccamaw: *Wilbur 49814* (DUKE!)

Little Singletary Lake (Rare): *Howell LISI−33* (NCSC!)

##### Notes

**Figure 53.**
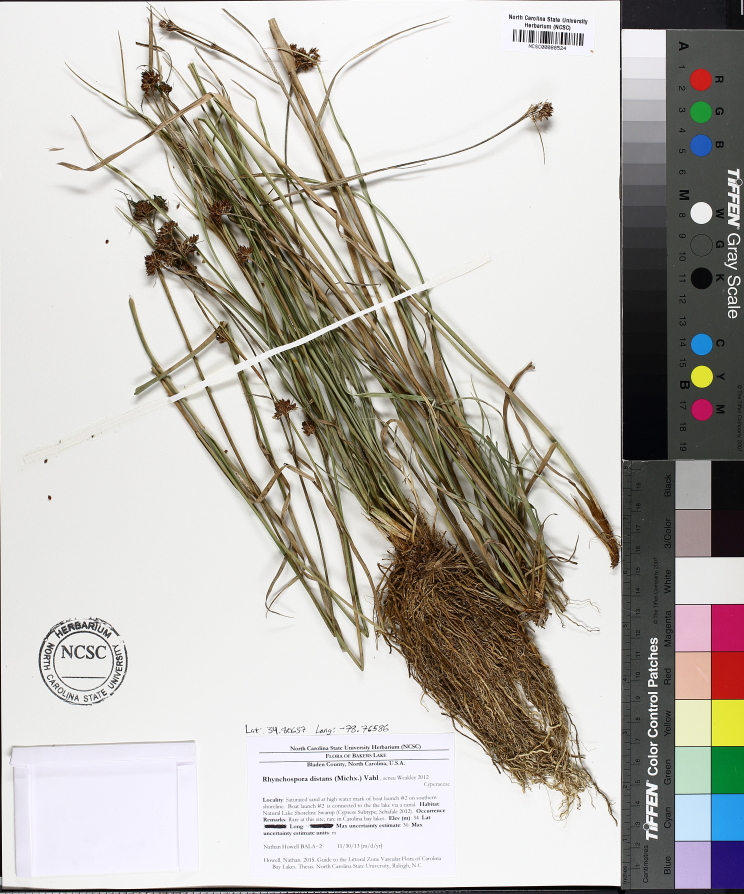
*Rhynchospora distans* (*Howell BALA-2*, NCSC)

Perennial herbs. Eulittoral zone; typically at the high water mark in moist sandy soil (NLSS−C). Jun−Sep. Fig. 53

#### Rhynchospora elliottii

A. Dietr.

Rhynchospora elliottii Taxon concept: [= *R. schoenoides* (Elliott) Wood – RAB; = GW, FNA, Weakley]

##### Distribution

Lake Waccamaw: ♦

##### Notes

Perennial herbs. Eulittoral zone (NLSS−LW). Jul−Sep.

#### Rhynchospora inexpansa

(Michx.) Vahl

Rhynchospora inexpansa Basionym: *Schoenus inexpansus* Michx.
Rhynchospora inexpansa Taxon concept: [= RAB, GW, FNA, Weakley]

##### Distribution

Jones Lake: *Beal 799* (NCSC!)

##### Notes

**Figure 54.**
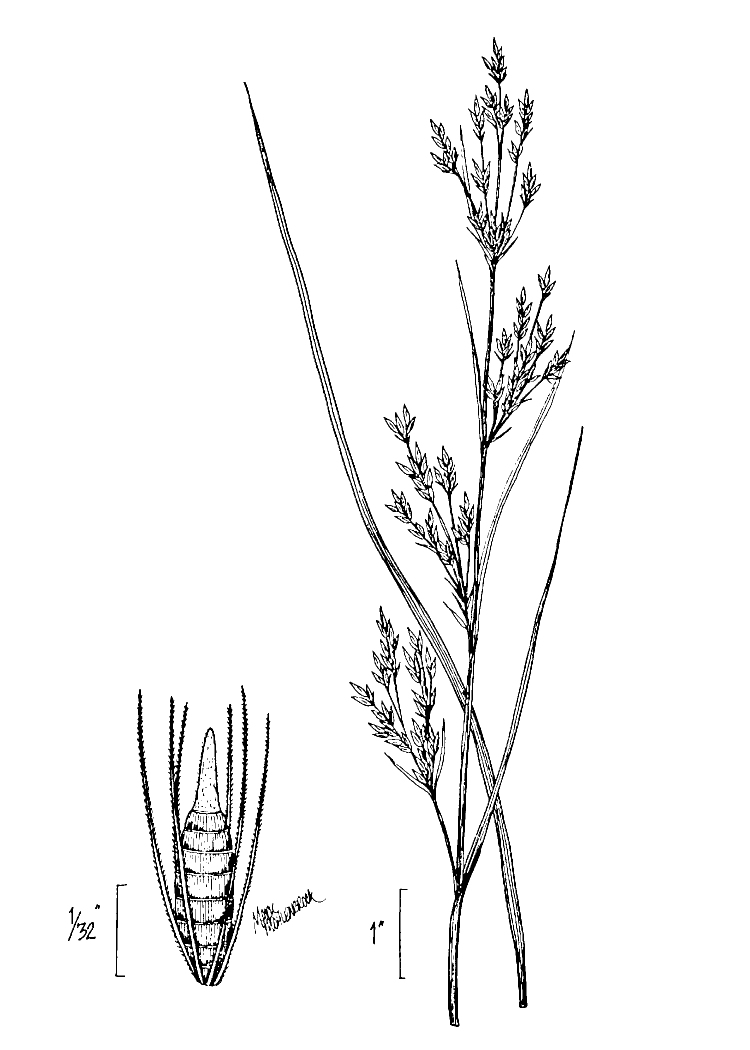
*Rhynchospora inexpansa* (illustration from United States Department of Agriculture, Natural Resource Conservation Service PLANTS Database / United States Department of Agriculture, Natural Resource Conservation Service 2015)

Perennial herbs. Eulittoral zone (NLSS–C). Jul–Sep. Fig. 54

#### Rhynchospora inundata

(Oakes) Fernald

Rhynchospora inundata Basionym: Ceratoschoenus macrostachyus (Torr. ex A. Gray) A. Gray var. inundatus Oakes
Rhynchospora inundata Taxon concept: [= RAB, GW, FNA, Weakley]

##### Ecological interactions

###### Conservation status

W1; S3, G4?

##### Distribution

Horseshoe Lake (Infrequent): *Howell HOLA−53* (NCSC!); *Grant* *s.n.* (NCU!); *Rothfels*, *Burge*, *Duke Nat. Hist. Soc. 2401* (DUKE!)

##### Notes

Perennial herbs. Eulittoral zone of shorelines and on floating bogs (CPSI−CG, FB). Jul−Sep. Fig. 55

#### Rhynchospora latifolia

(Baldwin) W.W. Thomas

Rhynchospora latifolia Basionym: *Dichromena latifolia* Baldwin
Rhynchospora latifolia Taxon concept: [= *Dichromena latifolia* Baldwin ex Elliott – RAB, GW; = FNA, Weakley]

##### Distribution

Lake Waccamaw: *Radford 723* (NCU!)

##### Notes

**Figure 56.**
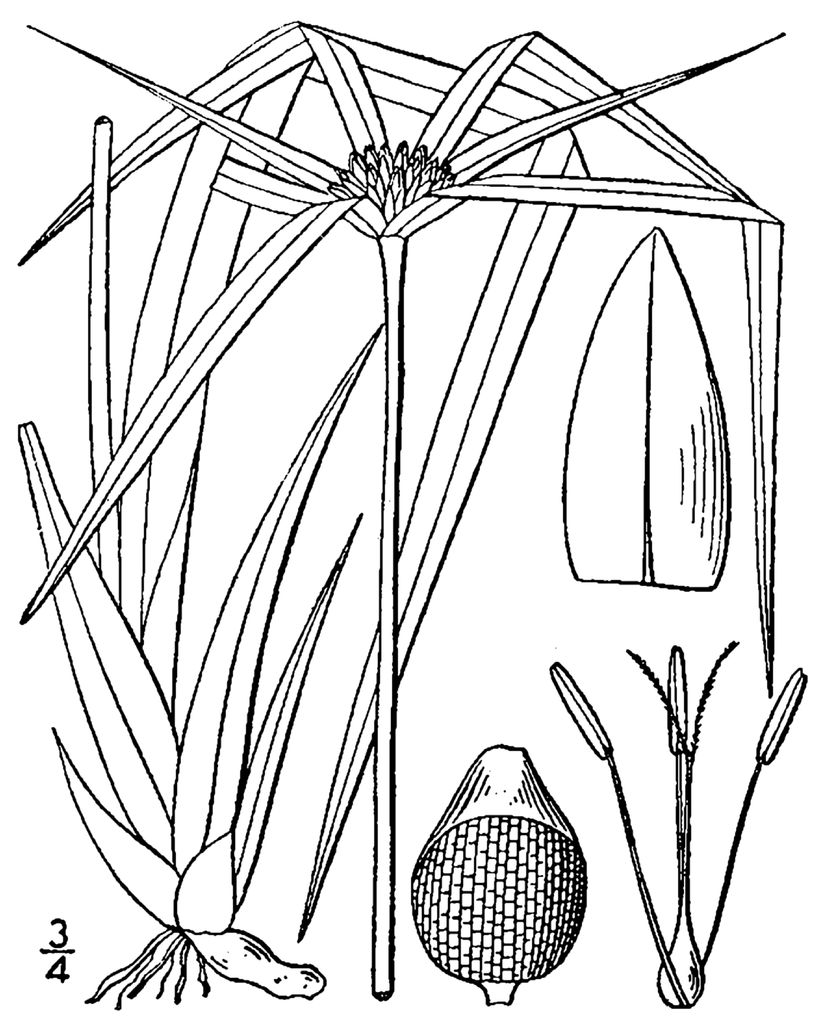
*Rhynchospora latifolia* (illustration from Britton and Brown 1913)

Perennial herbs. Eulittoral zone (NLSS−LW). May−Sep. Fig. 56

#### Rhynchospora macrostachya

Torr. ex A. Gray

Rhynchospora macrostachya Taxon concept: [= RAB, GW, FNA, Weakley]

##### Distribution

Lake Waccamaw (Infrequent): *Howell LAWA−130* (NCSC!)

##### Notes

Perennial herbs. Eulittoral zone (NLSS−LW). Jul−Sep. Fig. 57

#### Rhynchospora nitens

(Vahl) A. Gray

Rhynchospora nitens Basionym: *Scirpus nitens* Vahl
Rhynchospora nitens Taxon concept: [= *Psilocarya nitens* (Vahl) Alph. Wood – RAB, GW; = FNA, Weakley]

##### Ecological interactions

###### Conservation status

W1; S3, G4?

##### Distribution

Lake Waccamaw: *Wilbur 49781* (DUKE!)

##### Notes

**Figure 58.**
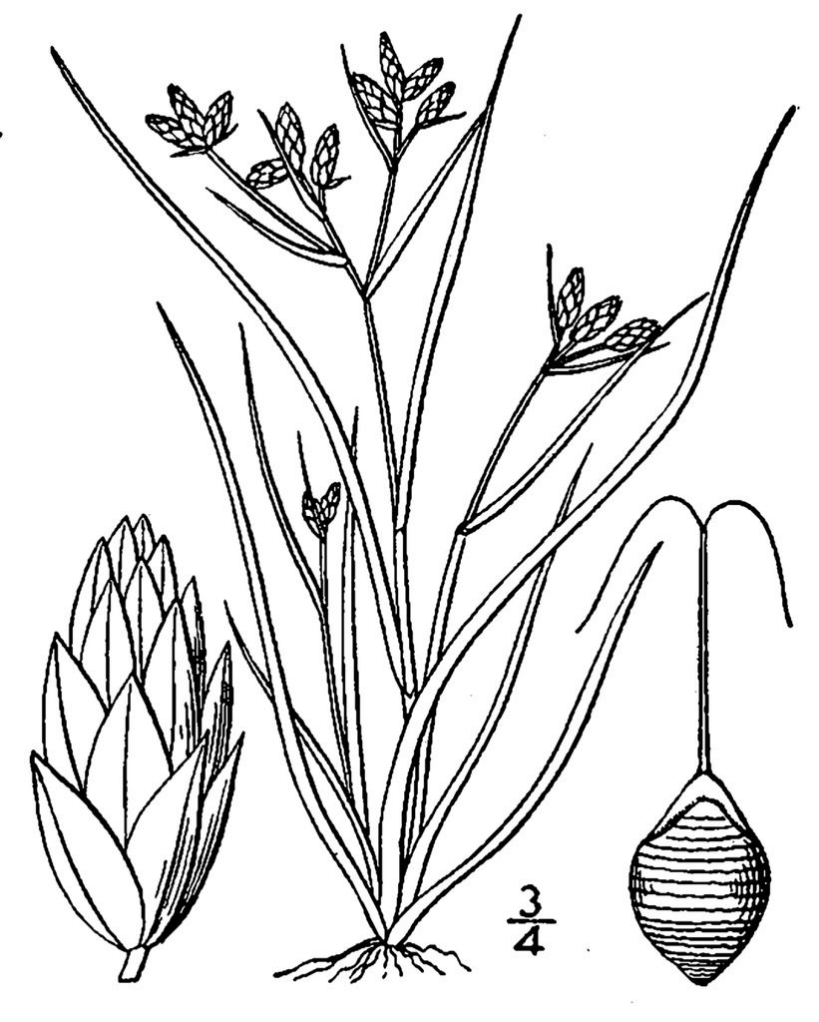
*Rhynchospora nitens* (illustration from Britton and Brown 1913)

Annual herbs. Eulittoral zone (NLSS−LW). Jul−Aug. Fig. 58

#### Scirpus cyperinus

(L.) Kunth

Scirpus cyperinus Basionym: *Eriophorum cyperinum* L.
Scirpus cyperinus Taxon concept: [= RAB, GW, FNA, Weakley]

##### Distribution

Bay Tree Lake (Occasional): *Howell BATR−58* (NCSC!)

Jones Lake (Occasional): *Howell JOLA−4, 45* (NCSC!)

Lake Waccamaw (Occasional): *Howell LAWA−166* (NCSC!)

Little Singletary Lake (Occasional): •

##### Notes

Perennial herbs. Eulittoral zone (NLSS−C, NLSS−LW, NLSM−T). Jul−Sep. Fig. 59

### Eriocaulaceae

#### Eriocaulon aquaticum

(Hill) Druce

Eriocaulon aquaticum Basionym: *Cespa aquatica* Hill
Eriocaulon aquaticum Taxon concept: [> *E. pellucidum* Michx. – RAB; = *E. septangulare* – GW; = FNA, Weakley]

##### Ecological interactions

###### Conservation status

SC−V; S2, G5.

##### Distribution

Lake Waccamaw (Abundant): *Howell LAWA−5, 52* (NCSC!); *Lynch 185* (NCSC!); *Wilbur 49802* (DUKE!)

##### Notes

Perennial herbs. Eulittoral and infralittoral zones (NLSS−LW, NLSM−LWP). Jul−Oct. A dominant species in the littoral zone of Lake Waccamaw. Fig. 60

### Haemodoraceae

#### Lachnanthes caroliniana

(Lam.) Dandy

Lachnanthes caroliniana Basionym: *Dilatris caroliniana* Lam.
Lachnanthes caroliniana Taxon concept: [= RAB, GW, FNA, Weakley]]

##### Distribution

Bay Tree Lake (Infrequent): *Howell BATR−50, 51* (NCSC!)

Horseshoe Lake (Infrequent): *Howell HOLA−51* (NCSC!)

Lake Waccamaw (Occasional): *Howell LAWA−107* (NCSC!)

Little Singletary Lake (Infrequent): *Howell LISI−25, 51* (NCSC!)

##### Notes

Perennial herbs. Eulittoral zone; typically in saturated soils at or below the maximum annual high water mark (NLSS–C, NLSS–LW, NLSM–T, CPSI–CG). Jun–early Sep; Sep–Nov. Fig. 61

### Hydrocharitaceae

#### Hydrilla verticillata

(L. f.) Royle

Hydrilla verticillata Basionym: *Serpicula verticillata* L.f.
Hydrilla verticillata Taxon concept: [= GW, FNA, Weakley]

##### Distribution

Lake Waccamaw: ¤

##### Notes

**Figure 62.**
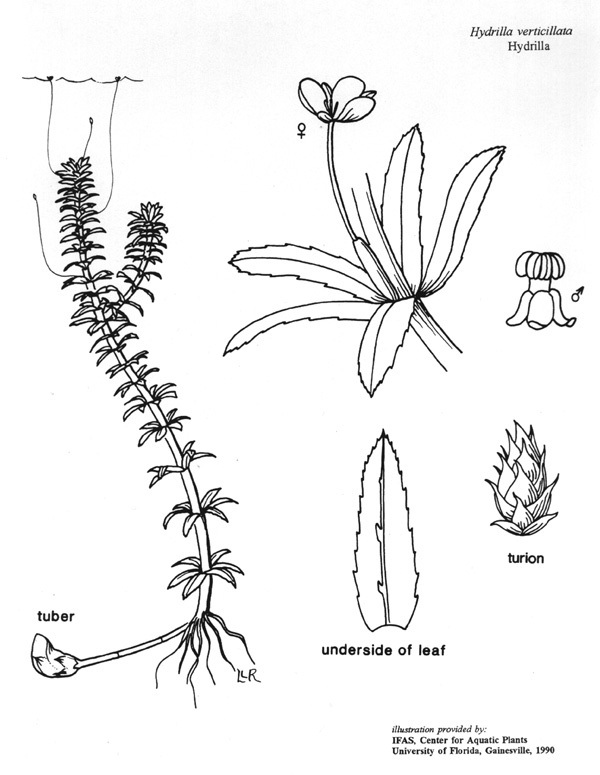
*Hydrilla verticillata* (from Center for Aquatic and Invasive Plants, University of Florida, IFAS 2015)

Perennial herbs. Infralittoral zone (NLSS−LW, NLSM−LWP). Jun−Aug. This exotic, invasive taxon is native to warm climates of the Old World. *Hydrilla verticillata* was introduced to Florida in 1950 as an ornamental and has since become a terrible aquatic invasive throughout the Southeast. Where introduced, *H. verticillata* chokes out native submersed aquatic vegetation (e.g., *Ceratophyllum*, *Myriophyllum*, *Najas*, *Potomogeton*, *Vallisneria*), negatively impacts recreational activities and alters natural hydrology and water chemistry (Ramey and Peichel 2001). Fig. 62

#### Najas guadalupensisvar.guadalupensis

Najas guadalupensisvar.guadalupensis Basionym: *Caulinia guadalupensis* Spreng.
Najas guadalupensisvar.guadalupensis Taxon concept: [< *N. guadalupensis* (Spreng.) Magnus – RAB, GW; = N. guadalupensis ssp. guadalupensis – FNA; = Weakley]

##### Distribution

Lake Waccamaw: *Blomquist & Schuster 16190* (DUKE!)

##### Notes

**Figure 63.**
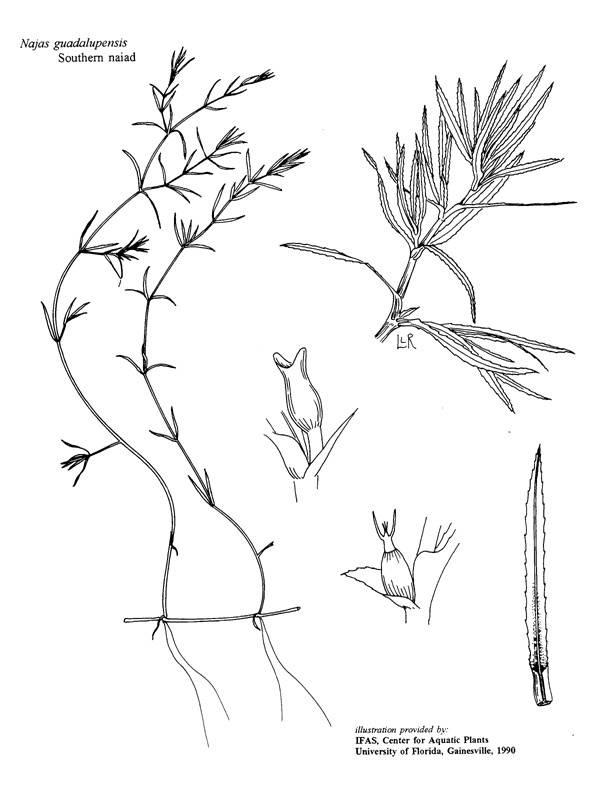
*Najas guadalupensis* (from Center for Aquatic and Invasive Plants, University of Florida, IFAS 2015)

Annual herbs. Infralittoral zone (NLSS−LW, NLSM−LWP). Jul−Sep. Fig. 63

### Hypoxidaceae

#### Hypoxis curtissii

Rose

Hypoxis curtissii Taxon concept: [= H. hirsuta (L.) Coville var. leptocarpa (Engelm. & A. Gray) Fernald – RAB; = *H. leptocarpa* (Engelm. & A. Gray) Small – GW; = FNA, Weakley]

##### Distribution

Lake Waccamaw (Rare): *Howell LAWA−60* (NCSC!)

##### Notes

Perennial herbs. Eulittoral zone; at the high water mark in moist to saturated soil (NLSS−LW). Mar−Jun; May−Jul. Fig. 64

### Juncaceae

#### Juncus acuminatus

Michx.

Juncus acuminatus Taxon concept: [= RAB, GW, FNA, Weakley]

##### Distribution

Bay Tree Lake (Rare): *Howell BATR−15* (NCSC!)

##### Notes

Perennial herbs. Eulittoral zone. May−Aug. Fig. 65

#### Juncus biflorus

Elliott

Juncus biflorus Taxon concept: [= RAB; < *J. marginatus Rostk.* – GW, FNA; = Weakley]

##### Distribution

Little Singletary Lake (Rare): *Howell LISI−58* (NCSC!)

Singletary Lake: *Beal 796* (NCSC!)

##### Notes

Perennial herbs. Juncture of the eulittoral and supralittoral zones; usually in wet soils at or just below the high water mark (NLSM−T, NLSS−C). Jun−Oct. Fig. 66

#### Juncus canadensis

J. Gay ex Laharpe

Juncus canadensis Taxon concept: [= RAB, GW, FNA, Weakley]

##### Distribution

Lake Waccamaw (Infrequent): *Howell LAWA−32, 170* (NCSC!)

##### Notes

Perennial herbs. Eulittoral zone (NLSS−LW). Jul−Oct. Fig. 67

#### Juncus coriaceus

Mack.

Juncus coriaceus Taxon concept: [= RAB, GW, FNA, Weakley]

##### Distribution

Horseshoe Lake: *Beal 828* (NCSC!)

##### Notes

**Figure 68.**
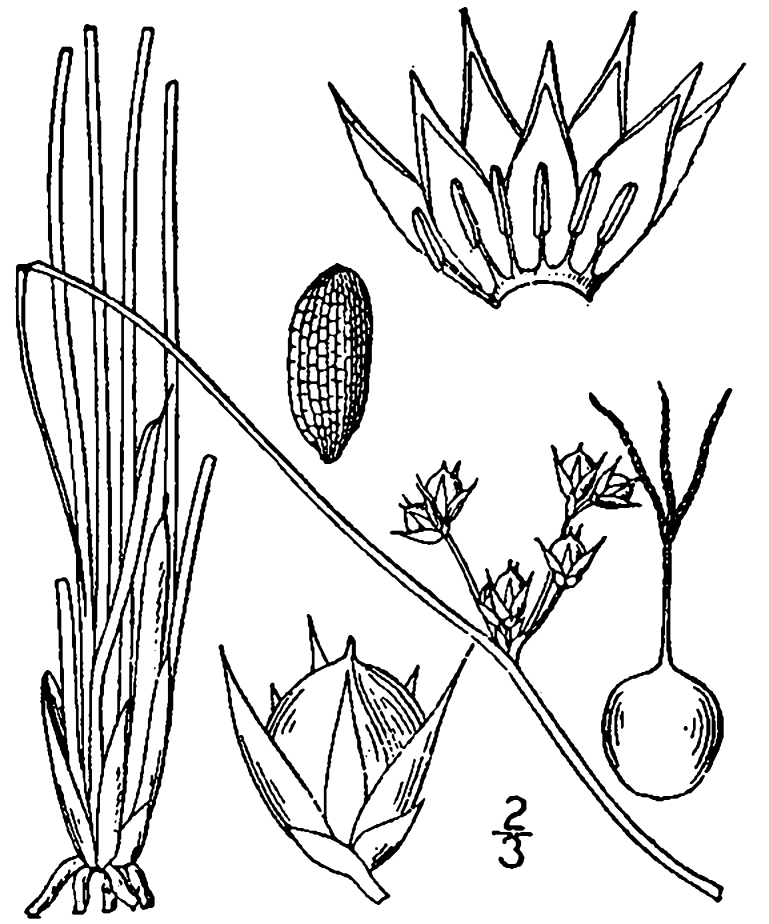
*Juncus coriaceus* (from Britton and Brown 1913)

Perennial herbs. Juncture of eulittoral and supralittoral zones (CPSI−CG). Jun−Sep. Fig. 68

#### Juncus effusus solutusvar.effusus

(Fernald & Wiegand) Hämet-Ahti

Juncus effusus solutusvar.effusus Taxon concept: [< *J. effusus* – RAB, GW, FNA; = Weakley]

##### Distribution

Bay Tree Lake (Occassional): *Howell BATR−6* (NCSC!)

Little Singletary Lake (Occassional): *Howell LISI−3* (NCSC!)

Horseshoe Lake (Occassiona): *Howell HOLA−8* (NCSC!)

##### Notes

Perennial herbs. Eulittoral zone (NLSS−C, NLSM−T, CPSI−CG). Jun−Sep. Fig. 69

#### Juncus pelocarpus

E. Mey.

Juncus pelocarpus Taxon concept: [> *J. abortivus* Chapm. – RAB, GW; = FNA, Weakley]

##### Distribution

Bay Tree Lake (Frequent): *Howell BATR−61* (NCSC!); *Wilbur 57415* (DUKE!)

Horseshoe Lake: *Wilbur 2264, 81465* (DUKE!)

Jones Lake (Occasional): *Howell JOLA−17, 35* (NCSC!); *Wilbur 57582* (DUKE!)

Lake Waccamaw (Frequent): *Howell LAWA−3* (NCSC!); *Wilbur s. n.*, *84188* (DUKE!)

Singletary Lake (Occasional): *Howell SILA−31* (NCSC!)

##### Notes

Perennial herbs. Eulittoral zone (NLSS−C, NLSS−LW, NLSM−T, CPSI−CG). Jul−Oct. Fig. 70

#### Juncus repens

Michx.

Juncus repens Taxon concept: [= RAB, GW, FNA, Weakley]

##### Distribution

Bay Tree Lake (Occasional): *Howell BATR−8* (NCSC!); *Wilbur 57395* (DUKE!)

Horseshoe Lake (Occasional): *Beal 4348* (NCSC!); *Howell HOLA−14* (NCSC!); *Wilbur & Menchi Ho 83792* (DUKE!)

Lake Waccamaw (Occasional): *Howell LAWA−30, 31* (NCSC!)

Little Singletary Lake (Occasional): *Howell LISI−19, 44* (NCSC!)

Singletary Lake (Occasional): *Howell SILA−1, 32* (NCSC!)

##### Notes

Perennial herbs. Eulittoral zone (NLSS−C, NLSS−LW, NLSM−T, CPSI−CG). Jun−Oct. Fig. 71

#### Juncus scirpoidesvar.scirpoides

Juncus scirpoidesvar.scirpoides Taxon concept: [< *J. scirpoides* Lam. – RAB, GW, FNA, Weakley]

##### Distribution

Bay Tree Lake (Infrequent): *Howell BATR−27, 66* (NCSC!)

Little Singletary Lake (Infrequent): *Howell LISI−55* (NCSC!)

##### Notes

Perennial herbs. Eulittoral zone (NLSS−C, NLSM−T). Jun−Sep. Fig. 72

### Mayacaceae

#### Mayaca fluviatilis

Aubl.

Mayaca fluviatilis Taxon concept: [> *M. aubletii* Michx. – RAB; > *M. fluviatilis* Aubl. – RAB; = GW, FNA, Weakley]

##### Distribution

Lake Waccamaw: ¤

##### Notes

**Figure 73.**
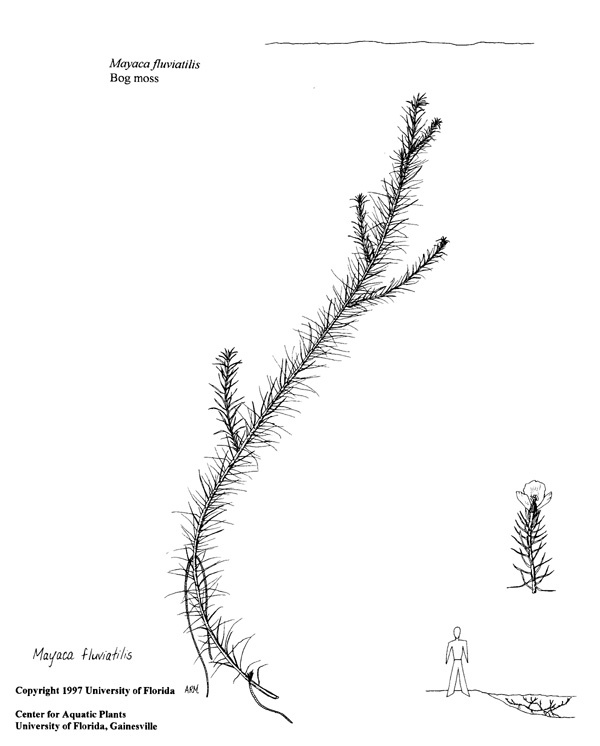
*Mayaca fluviatilis* (from Center for Aquatic and Invasive Plants, University of Florida, IFAS 2015)

Perennial herbs. Eulittoral and infralittoral zones (NLSS−LW). May−Jul. Fig. 73

### Orchidaceae

#### Calopogon tuberosusvar.tuberosus

Calopogon tuberosusvar.tuberosus Basionym: *Limodorum tuberosum* L.
Calopogon tuberosusvar.tuberosus Taxon concept: [< *C. pulchellus* R. Brown − RAB; < *C. tuberosus* (L.) Britton, Sterns, & Poggenb. – GW; = FNA, Weakley]

##### Distribution

Horseshoe Lake (Occasional): *Howell HOLA−24, 39* (NCSC!)

##### Notes

Perennial herbs. floating bogs (CPSI–CG, FB). Apr–Jul. Fig. 74

#### Epidendrum magnoliae

Muhl.

Epidendrum magnoliae Taxon concept: [< *E. conopseum* R. Br. – RAB; = FNA, Weakley]

##### Ecological interactions

###### Conservation status

T; S1S2, G4.

##### Distribution

Lake Waccamaw: *Correll & Blomquist 4900* (DUKE!)

##### Notes

Perennial, epiphytic herbs. Eulittoral zone; typically on limbs and trunks of *Taxodium ascendens*, *Taxodium distichum*, *Nyssa aquatica*, *Nyssa biflora*, *Liquidambar styraciflua*, and possibly other bottomland tree species in the shoreline of Lake Waccamaw (NLSS– LW, NLSS–C). Jul–Oct. This species usually co-occurs with *Pleopeltis polypodiodes*. The first author observed a vegetative specimen on the edge of Big Creek ca. 50–70 meters from the shoreline of Lake Waccamaw. The specimen was on a large *Nyssa aquatica* limb, ca. 25–30 meters above the water, and was co- occuring with *Pleopeltis polypodioides*.

#### Habenaria repens

Nutt.

Habenaria repens Taxon concept: [= RAB, GW, FNA, Weakley]

##### Ecological interactions

###### Conservation status

W1; S2, G5.

##### Distribution

Lake Waccamaw: ►

##### Notes

Perennial herbs. Eulittoral zone (NLSS–LW). Apr–Nov.

#### Pogonia ophioglossoides

(L.) Ker Gawl.

Pogonia ophioglossoides Basionym: *Arethusa ophioglossoides* L.
Pogonia ophioglossoides Taxon concept: [= RAB, GW, FNA, Weakley]

##### Distribution

Horseshoe Lake (Occasional): *Howell HOLA−30* (NCSC!)

##### Notes

Perennial herbs. floating bogs (CPSI–CG, FB). Mar–Jun. Fig. 75

#### Spiranthes laciniata

(Small) Ames

Spiranthes laciniata Basionym: *Gyrostachys laciniata* Small
Spiranthes laciniata Taxon concept: [= RAB; < S. × laciniata – GW; = FNA, Weakley]

##### Ecological interactions

###### Conservation status

SC−V; S2, G4,G5.

##### Distribution

Lake Waccamaw (Occasional): *Howell LAWA−105, 106, 116* (NCSC!)

##### Notes

Perennial herbs. Eulittoral zone (NLSS–LW). May–Aug. Fig. 76

### Poaceae

#### Agrostis hyemalis

(Walter) Britton, Sterns & Poggenb.

Agrostis hyemalis Basionym: *Cornucopiae hyemalis* Walter
Agrostis hyemalis Taxon concept: [< *A. hyemalis* (Walter) Britton, Sterns & Poggenb. – RAB; = *A. hiemalis* (Walter) Britton, Sterns & Poggenb. – GW; = FNA, Weakley]

##### Distribution

Little Singletary Lake (Rare): *Howell LISI−37* (NCSC!)

##### Notes

Perennial herbs. Juncture of supralittoral and eulittoral zones; typically in moist sandy soils (NLSM−T). Mar−Jul. Fig. 77

#### Andropogon glaucopsis

Steud.

Andropogon glaucopsis Taxon concept: [< *A. virginicus* L. – RAB; = GW; = A. glomeratus var. glaucopsis (Elliott) C. Mohr − FNA; = Weakley]

##### Distribution

Horseshoe Lake: *Buell* *s.n.* (DUKE!, NCSC!)

Jones Lake (Infrequent): *Howell JOLA–16* (NCSC!)

##### Notes

**Figure 78.**
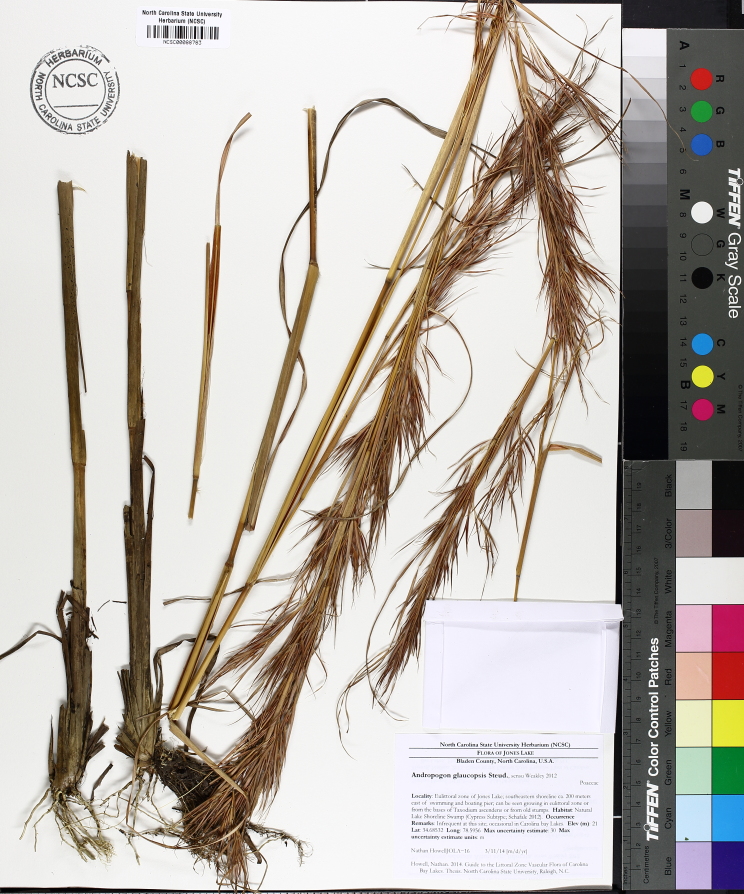
*Andropogon glaucopsis* (*Howell JOLA-16*, NCSC)

Perennial herbs. Eulittoral zone (CPSI−CG, FB). Sep−Oct. Fig. 78

#### Andropogon virginicusvar.virginicus

Andropogon virginicusvar.virginicus Taxon concept: [< *A. virginicus* L. – RAB; = FNA, Weakley]

##### Distribution

Lake Waccamaw: ♦

##### Notes

**Figure 79.**
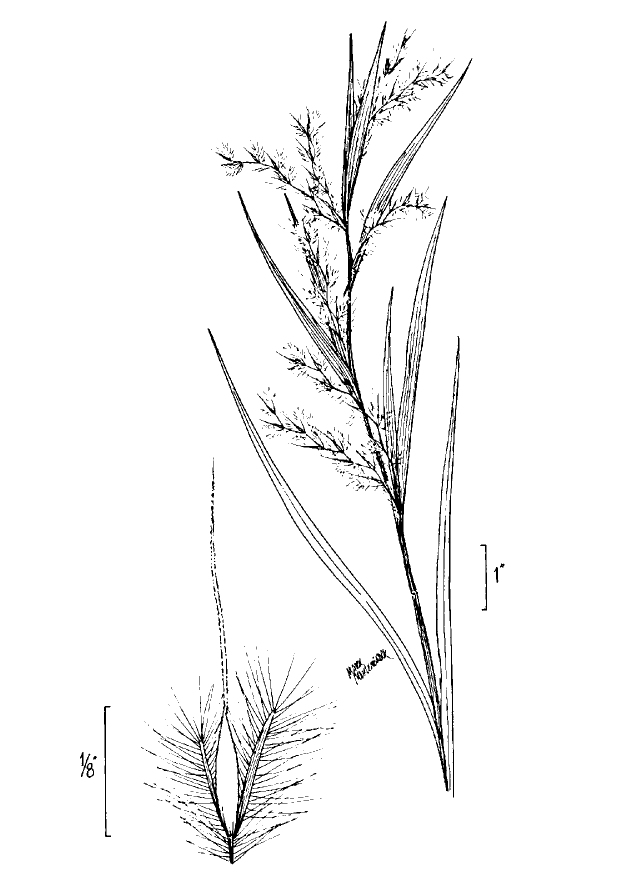
*Andropogon virginicus* (illustration from United States Department of Agriculture, Natural Resource Conservation Service PLANTS Database / United States Department of Agriculture, Natural Resource Conservation Service 2015)

Perennial herbs. Eulittoral zone (NLSS−LW). Sep−Oct. Fig. 79

#### Arundinaria tecta

(Walter) Muhl.

Arundinaria tecta Basionym: *Arundo tecta* Walter
Arundinaria tecta Taxon concept: [< *A. gigantea* (Walter) Muhl. – RAB, GW; = FNA, Weakley]

##### Distribution

Lake Waccamaw: *Bennedict 4350* (DUKE!)

##### Notes

**Figure 80.**
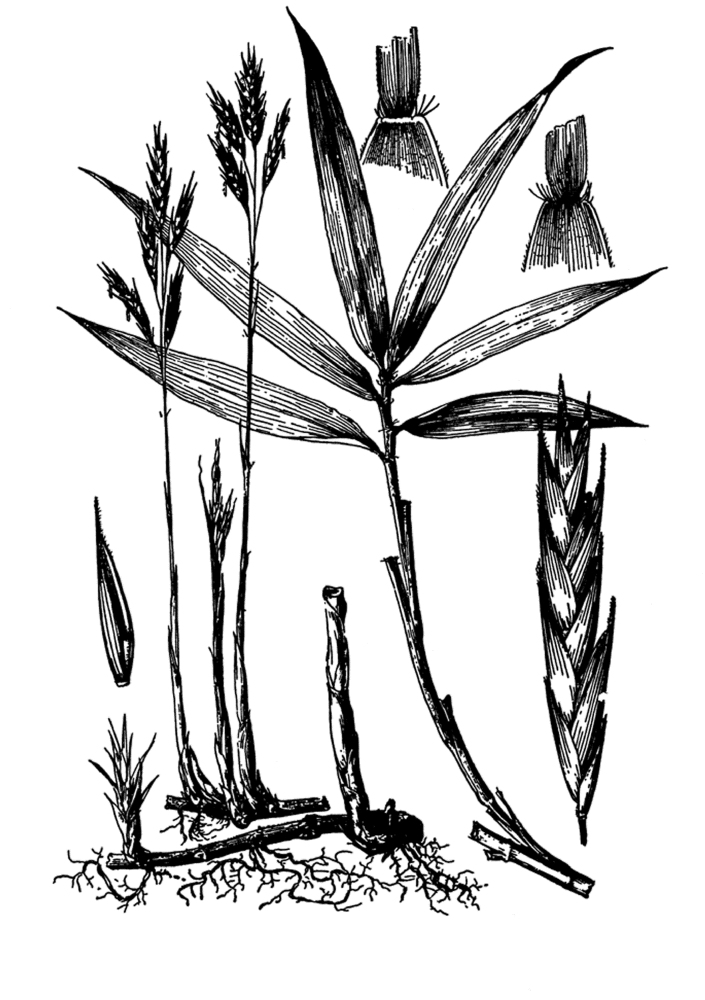
*Arundinaria tecta* (from Hitchcock and Chase 1951)

Arborescent herbs. Eulittoral zone; at or just below the mean annual high water mark (NLSS−LW). Apr−Jul. The first author has not encountered this taxon in the field, but a single voucher specimen (see above) places it within the immediate vicinity. Fig. 80

#### Coleataenia longifoliavar.longifolia

Coleataenia longifoliavar.longifolia Basionym: *Panicum longifolium* Torr.
Coleataenia longifoliavar.longifolia Taxon concept: [= Panicum longifolium Torr. var. longifolium – RAB; < *Panicum longifolium* Torr. – GW; = Panicum rigidulum Bosc ex Nees ssp. pubescens (Vasey) Freckmann & Lelong − FNA; = Weakley]

##### Distribution

Bay Tree Lake (Infrequent): *Howell BATR – 68* (NCSC!)

Lake Waccamaw (Occasional): *Howell LAWA −145, 147, 164, 168* (NCSC!)

##### Notes

**Figure 81.**
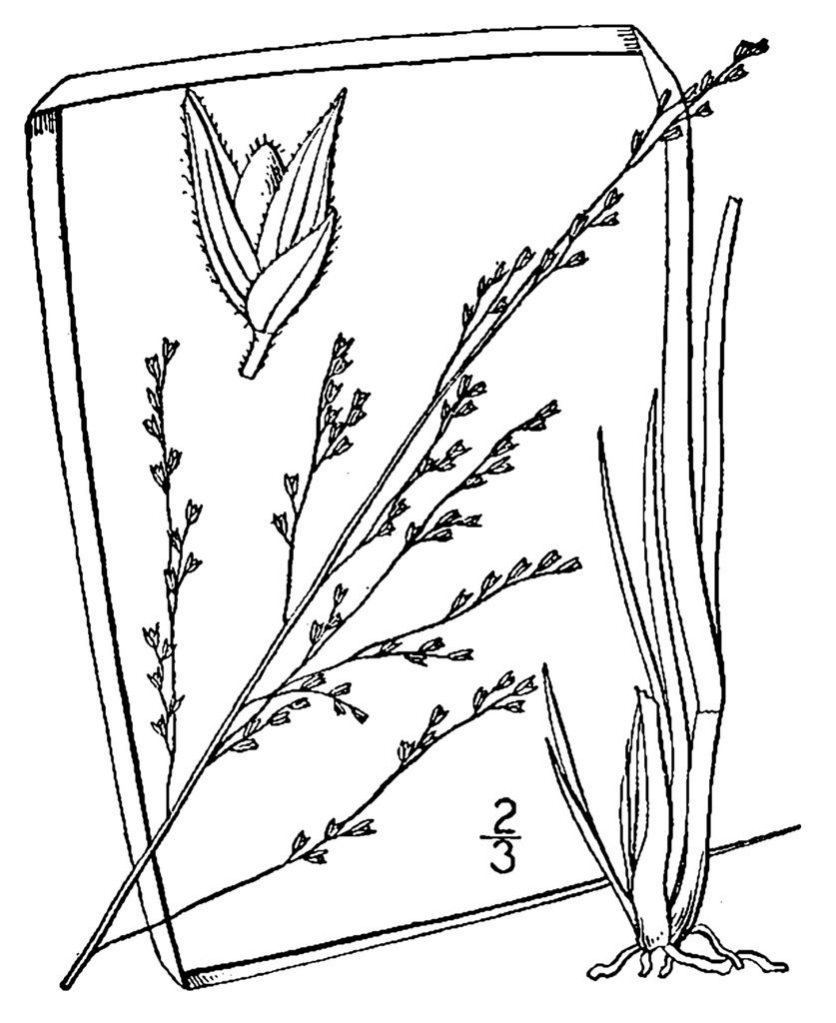
*Coleataenia longifolia* (illustration from Britton and Brown 1913)

Perennial herbs. Eulittoral zone (NLSS−LW). Jul−Oct. Fig. 81

#### Coleataenia tenera

(Beyr. ex Trin.) Soreng

Coleataenia tenera Basionym: *Panicum tenerum* Bey. ex Trin.
Coleataenia tenera Taxon concept: [= *Panicum tenerum* Bey. ex Trin. – RAB, GW, FNA; = Weakley]

##### Distribution

Lake Waccamaw: ►

##### Notes

Perennial herbs. Eulittoral zone (NLSS−LW). Jun−Sep.

#### Dichanthelium boreale

(Nash) Freckmann

Dichanthelium boreale Basionym: *Panicum boreale* Nash
Dichanthelium boreale Taxon concept: [> *Panicum bicknellii* Nash– RAB; > *D. boreale* (Nash) Freckmann – FNA; = Weakley]

##### Distribution

Lake Waccamaw: *Blomquist 957* (DUKE!)

##### Notes

**Figure 82.**
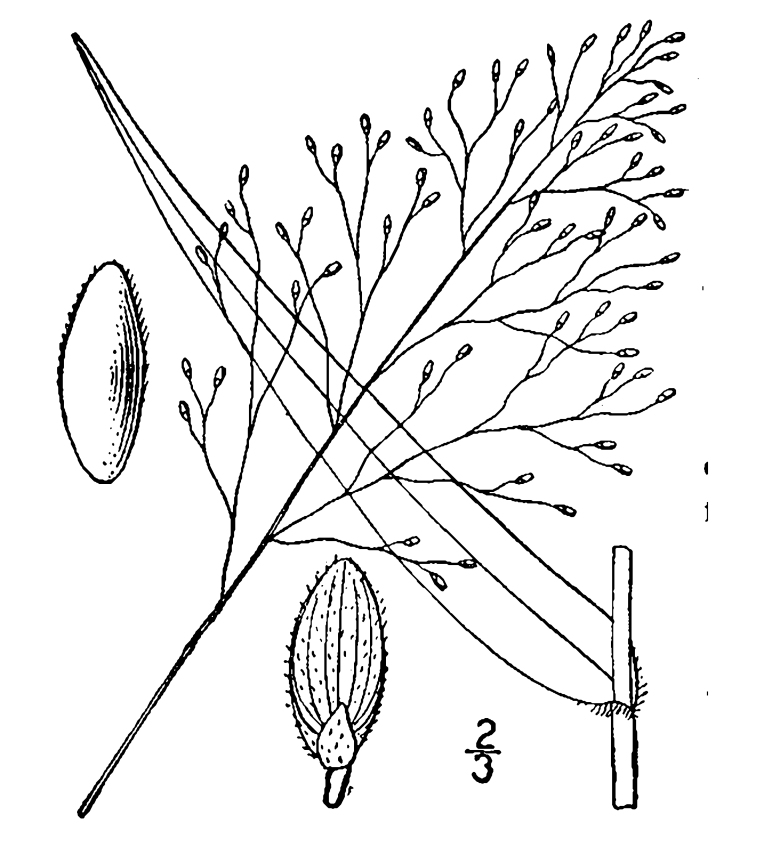
*Dichanthelium boreale* (illustration from Britton and Brown 1913)

Perennial herbs. Eulittoral zone (NLS−LW). Apr−Sep. Fig. 82

#### Dichanthelium dichotomumvar.roanokense

(Ashe) LeBlond

Dichanthelium dichotomumvar.roanokense Basionym: *Panicum roanokense* Nash
Dichanthelium dichotomumvar.roanokense Taxon concept: [< *D. dichotomum* (L.) Gould – RAB, GW; < *D. dichotomum* (L.) Gould) ssp. *roanokense* (Ashe) Freckmann & Lelong – FNA; = Weakley]

##### Distribution

Lake Waccamaw: *Ashe* *s.n.* (NCU!)

##### Notes

Perennial herbs. Eulittoral zone; moist to peaty lakeshores (NLSS−LW). May−Sep.

#### Dichanthelium erectifolium

(Nash) Gould & C.A. Clark

Dichanthelium erectifolium Basionym: *Panicum erectifolium* Nash
Dichanthelium erectifolium Taxon concept: [= *Panicum erectifolium* Nash – RAB, GW; = FNA, Weakley]

##### Ecological interactions

###### Conservation status

W2; S2, G4.

##### Distribution

Lake Waccamaw (Infrequent): *Howell LAWA−111* (NCSC!)

##### Notes

Perennial herbs. Eulittoral zone; moist sandy to peaty shores (NLSS−LW). May−Aug. Fig. 83

#### Dichanthelium lancearium

Dichanthelium lancearium Taxon concept: [= *Panicum lancearium* Trinius – RAB; < D. portoricense (Desv. ex Ham.) B.F. Hansen & Wunderlin ssp. patulum (Scribner & Merrill) Freckmann & Lelong – FNA; = Weakley]

##### Distribution

Lake Waccamaw: *Blomquist & Correll 9383* (NCU!)

##### Notes

Perennial herbs. Eulittoral zone (NLSS−LW). May−Sep.

#### Dichanthelium mattamuskeetense

(Ashe) Mohlenbr.

Dichanthelium mattamuskeetense Basionym: *Panicum mattamuskeetense* Ashe
Dichanthelium mattamuskeetense Taxon concept: [< *Panicum dichotomum* L. – RAB, GW; < D. dichotomum (L.) Gould ssp. mattamuskeetense (Ashe) Freckmann & Lelong – FNA; = Weakley]

##### Distribution

Lake Waccamaw: *Blomquist & Correll 9385* (DUKE!)

##### Notes

**Figure 84.**
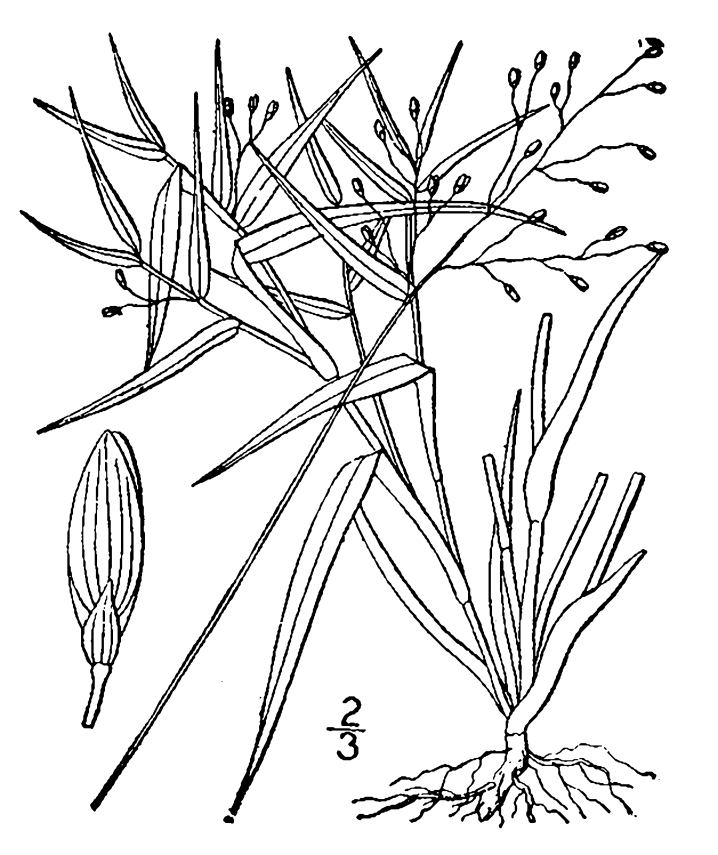
*Dichanthelium mattamuskeetense* (illustration from Britton and Brown 1913)

Perennial herbs. Eulittoral zone (NLSS−LW). May−Oct. Fig. 84

#### Dichanthelium portoricense

(Desv. ex Ham.) B.F. Hansen & Wunderlin

Dichanthelium portoricense Basionym: *Panicum portoricense* Desv. ex Ham.
Dichanthelium portoricense Taxon concept: [= *Panicum portoricense* Desv. ex Ham. – RAB; = D. portoricense (Desv. ex Ham.) B.F. Hansen & Wunderlin ssp. portoricense – FNA; = Weakley]

##### Distribution

Bay Tree Lake (Infrequent): *Howell BATR – 52* (NCSC!)

Lake Waccamaw: *Blomquist & Correll 9383* (NCU!)

##### Notes

**Figure 85.**
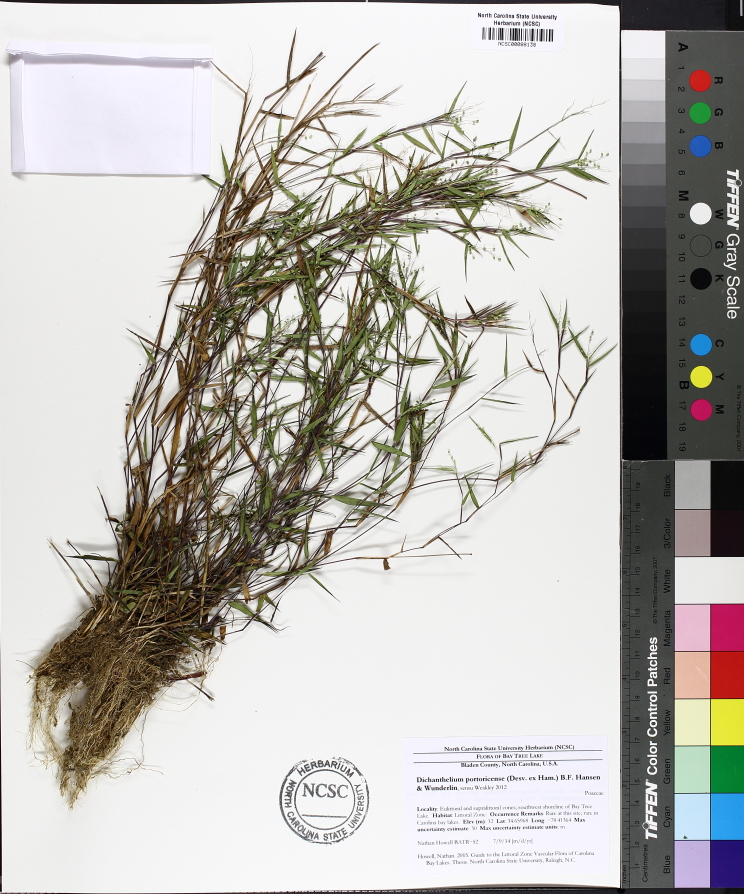
*Dichanthelium portoricense* (*Howell BATR-52*, NCSC)

Perennial herbs. Eulittoral zone; moist sandy to peaty shores (NLSS−LW). May−Sep. Fig. 85

#### Eragrostis elliottii

S. Watson

Eragrostis elliottii Taxon concept: [= RAB, GW, FNA, Weakley]

##### Distribution

Bay Tree Lake (Rare): *Howell BATR−67* (NCSC!)

Lake Waccamaw: ►

##### Notes

**Figure 86.**
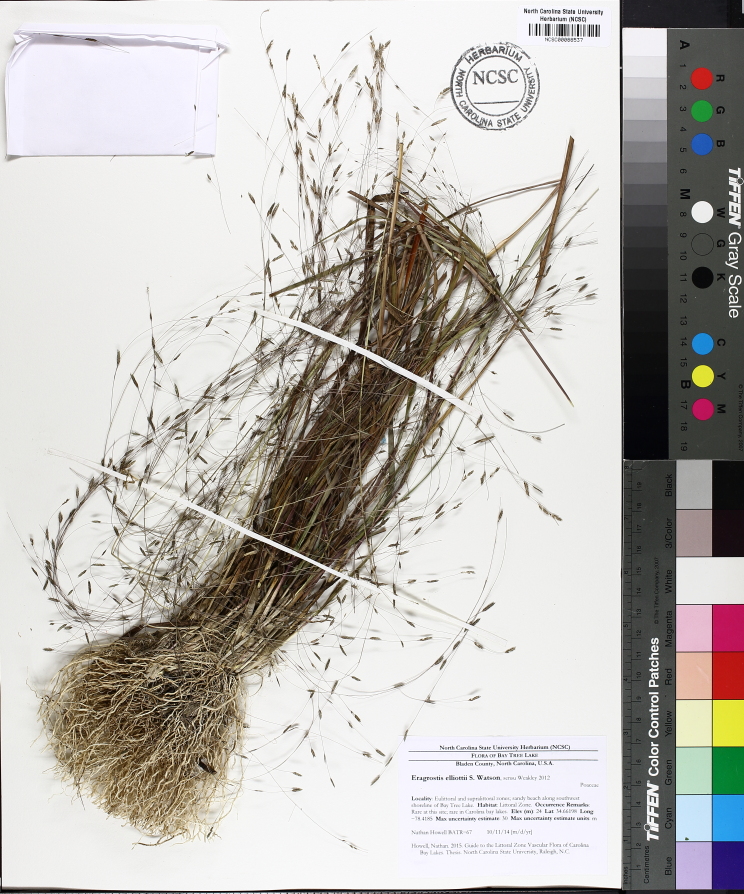
*Eragrostis elliottii* (*Howell BATR-67*, NCSC)

Perennial herbs. Eulittoral zone (NLSS−LW). Sep−Oct. Fig. 86

#### Eragrostis refracta

(Muhl. ex Elliott) Scribn.

Eragrostis refracta Basionym: *Poa refracta* Muhl. ex Elliott
Eragrostis refracta Taxon concept: [= RAB, GW, FNA, Weakley]

##### Distribution

Lake Waccamaw: ►

##### Notes

Perennial herbs. Eulittoral zone (NLSS−LW). Jul−Oct.

#### Luziola fluitansvar.fluitans

Luziola fluitansvar.fluitans Basionym: *Zizania fluitans* Michx.
Luziola fluitansvar.fluitans Taxon concept: [= *Hydrochloa carolinensis* P. Beauv. – RAB, GW; = FNA, Weakley]

##### Distribution

Lake Waccamaw (Occasional): *Bolser MEH107* (NCU!); *Howell LAWA−51* (NCSC!)

##### Notes

Perennial herbs. Eulittoral zone (NLSS−LW). Aug−Oct. Fig. 87

#### Panicum hemitomon

Schult.

Panicum hemitomon Taxon concept: [= RAB, GW, FNA, Weakley]

##### Distribution

Bay Tree Lake (Abundant): *Howell BATR−18* (NCSC!)

Horseshoe Lake (Infrequent): *Howell HOLA−23* (NCSC!)

Lake Waccamaw (Abundant): *Blomquist 1399* (DUKE!); *Blomquist & Correll 9379* (DUKE!, NCU!); *Howell LAWA−79* (NCSC!)

Little Singletary Lake (Infrequent): *Howell LISI−35* (NCSC!)

Salters Lake (Occasional): *Howell SALA−14* (NCSC!)

Singletary Lake (Occasional): *Blomquist 1400* (DUKE!); *Howell SILA−17* (NCSC!); *Wilbur 60947* (DUKE!)

##### Notes

Perennial herbs. Eulittoral and infralitoral zones (NLSS−C, NLSS−LW, NLSM−T, CPSI−CG). Jun−Jul. Fig. 88

#### Panicum verrucosum

Muhl.

Panicum verrucosum Taxon concept: [= RAB, GW, FNA, Weakley]

##### Distribution

Bay Tree Lake (Infrequent): *Howell BATR−53* (NCSC!)

Jones Lake (Rare): *Howell JOLA−40, 41* (NCSC!)

Little Singletary Lake (Rare): *Howell LISI−50* (NCSC!)

##### Notes

Annual herbs. Eulittoral zone (NLSM−T, NLSS−C). Aug−Oct. Fig. 89

#### Panicum virgatumvar.virgatum

Panicum virgatumvar.virgatum Taxon concept: [< *P. virgatum* – RAB, GW, FNA; = Weakley]

##### Distribution

Bay Tree Lake: *Wilbur 57420* (DUKE!)

##### Notes

**Figure 90.**
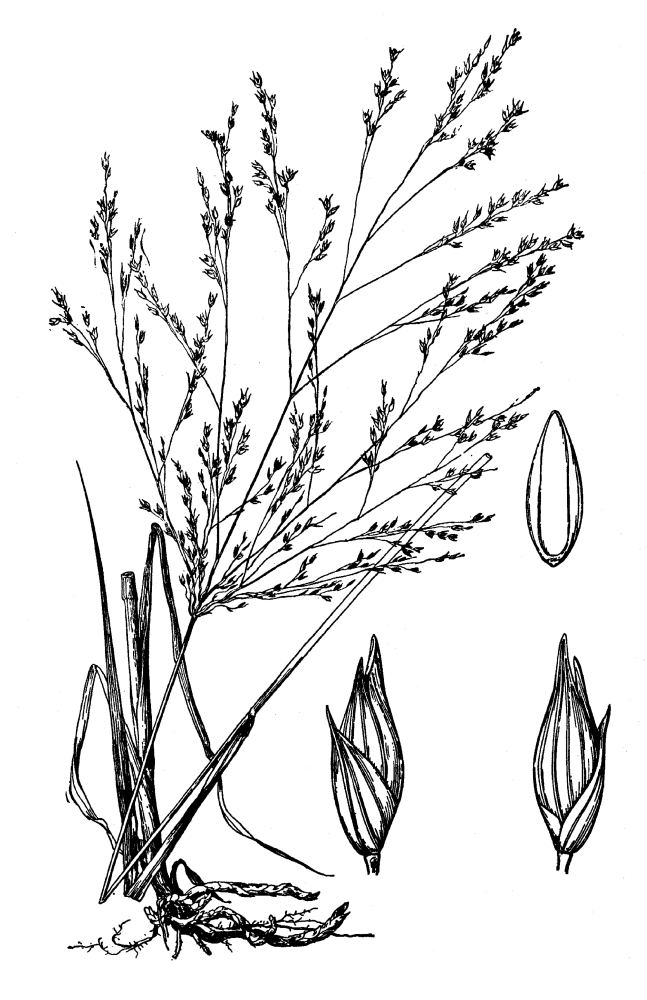
*Panicum virgatum* (illustration from Hitchcock and Chase 1951)

Perennial herbs. Eulittoral zone. Jun−Oct. Fig. 90

#### Saccharum giganteum

(Walter) Pers.

Saccharum giganteum Basionym: *Anthoxanthum giganteum* Walter
Saccharum giganteum Taxon concept: [= *Erianthus giganteus* (Walter) P. Beauv. – RAB, GW; = FNA. Weakley]

##### Distribution

Jones Lake (Rare): *Howell JOLA−37* (NCSC!)

Lake Waccamaw (Occasional): *Howell LAWA−7, 160* (NCSC!)

##### Notes

Perennial herbs. Eulittoral zone (NLSS−C, NLSS−LW). Sep−Oct. Fig. 91

#### Sacciolepis striata

(L.) Nash

Sacciolepis striata Basionym: *Holcus striatus* L.
Sacciolepis striata Taxon concept: [= RAB, GW, FNA, Weakley]

##### Distribution

Bay Tree Lake (Occasional): *Howell BATR−43, 54, 55* (NCSC!); *Wilbur 48657, 57394* (DUKE!)

Horseshoe Lake: *Rothfels*, *Burge*, *Duke Natural History Society 2398* (DUKE!)

Lake Waccamaw (Occasional): *Howell LAWA−131* (NCSC!)

Singletary Lake (Occasional): *Beal 3225* (NCSC!); *Frey* *s.n.* (NCU!); *Howell SILA−38* (NCSC!)

##### Notes

Perennial herbs. Eulittoral zone (NLSS−C, NLSS−LW, NLSM−T, CPSI−CG). Jul−Oct. Fig. 92

#### Sphenopholis obtusata

(Michx.) Scribn.

Sphenopholis obtusata Basionym: *Aria obtusata* Michx.
Sphenopholis obtusata Taxon concept: [= RAB, FNA, Weakley]

##### Distribution

Lake Waccamaw: *Blomquist 1492* (DUKE!)

##### Notes

**Figure 93.**
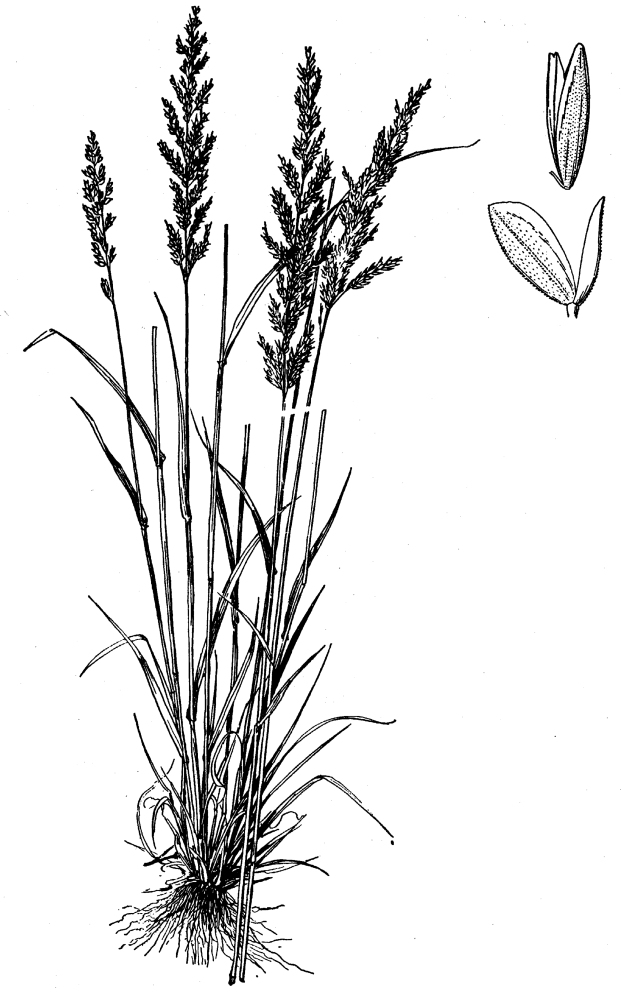
*Sphenopholis obtusata* (illustration from Hitchcock and Chase 1951)

Perennial herbs. Eulittoral zone (NLSS−LW). Apr−May. Fig. 93

### Pontederiaceae

#### Pontederia cordatavar.cordata

Pontederia cordatavar.cordata Taxon concept: [< *P. cordata* − RAB; = GW; < *P. cordata* – FNA; = Weakley]

##### Distribution

Bay Tree Lake (Rare): •Lake Waccamaw (Occasional): *Howell LAWA−15, 50, 159* (NCSC!); *Matthews* *s.n.* (NCU!); *Wilbur 59382* (DUKE!)

##### Notes

Perennial herbs. Eulittoral zone (NLSS−LW). May−Oct. Fig. 94

#### Pontederia cordatavar.lancifolia

(Muhl.) Torr.

Pontederia cordatavar.lancifolia Taxon concept: [< *P. cordata* – RAB; = GW; < *P. cordata* – FNA; = Weakley]

##### Distribution

Lake Waccamaw: ♦

##### Notes

Perennial herbs. Eulittoral zone (NLSS−LW). May−Oct.

### Potamogetonaceae

#### Potamogeton pulcher

Tuck.

Potamogeton pulcher Taxon concept: [= RAB, GW, FNA, Weakley]

##### Distribution

Lake Waccamaw: ►

##### Notes

**Figure 95.**
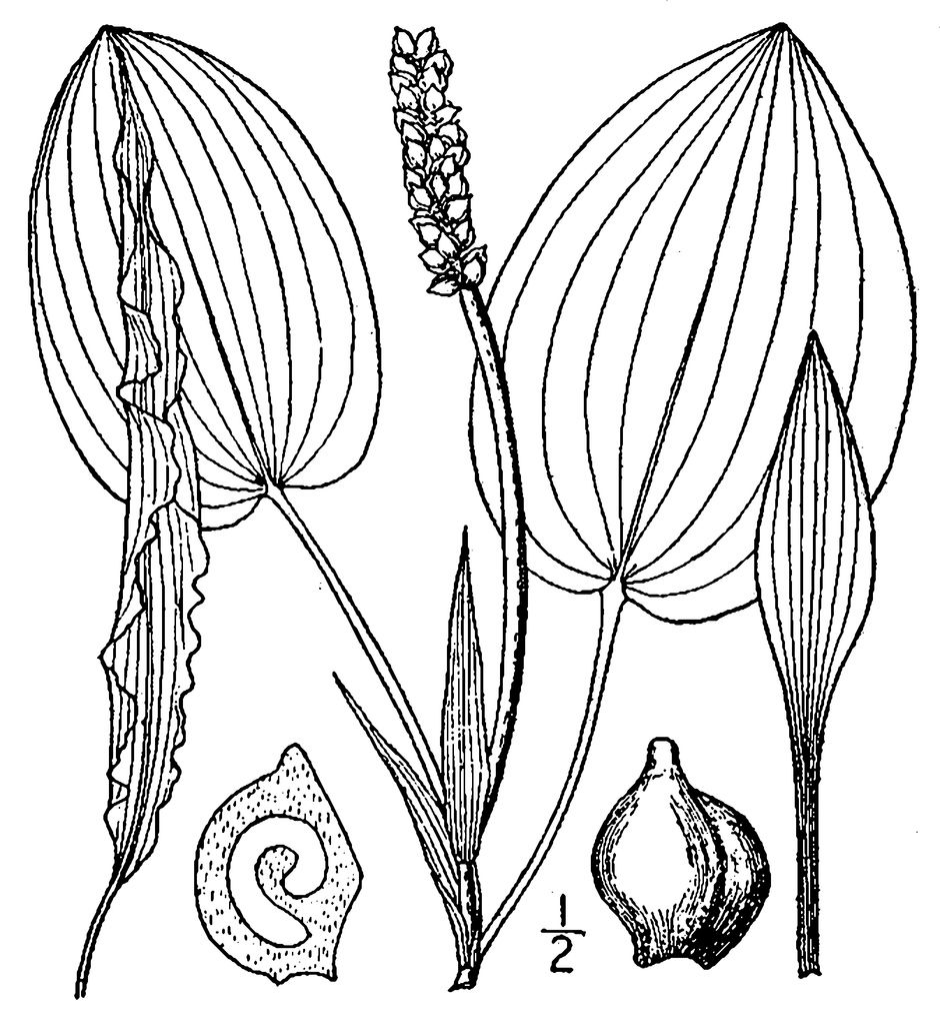
*Potamogeton pulcher* (illustration from Britton and Brown 1913)

Perennial herbs. Eulittoral and infralittoral zones (NLSS−LW, NLSM−LWP). Jun−Sep. Fig. 95

#### Potamogeton pusillus

L.

Potamogeton pusillus Taxon concept: [< *P. berchtoldii* Fieber – RAB; = GW; > P. pusillus L. ssp. pusillus – FNA; > P. pusillus L. var. pusillus − Weakley]

##### Distribution

Lake Waccamaw: ¤

##### Notes

Annual herbs. Eulittoral and infralittoral zones (NLSS−LW, NLSM−LWP). May−Sep.

### Smilacaceae

#### Smilax glauca

Walter

Smilax glauca Taxon concept: [= RAB, GW, FNA, Weakley]

##### Distribution

Bay Tree Lake (Rare): *Howell BATR−29* (NCSC!)

##### Notes

Perennial vines. Juncture of eulittoral and supralittoral zones. Late Apr−Early Jun; Sep−Nov and persisting. Fig. 96

#### Smilax laurifolia

L.

Smilax laurifolia Taxon concept: [= RAB, GW, FNA, Weakley]

##### Distribution

Bakers Lake (Frequent): *Howell BALA−13* (NCSC!)

Bay Tree Lake (Occasional): *Howell BATR−44* (NCSC!)

Horseshoe Lake (Occasional): *Howell HOLA−3* (NCSC!)

Jones Lake (Frequent): *Howell JOLA−7* (NCSC!)

Lake Waccamaw (Infrequent): *Howell LAWA−34* (NCSC!)

Salters Lake (Frequent): *Howell SALA−4, 15* (NCSC!)

Singletary Lake (Frequent): *Howell SILA−9* (NCSC!)

##### Notes

Perennial vines. Eulittoral zone; typically at the maximum annual high water mark in saturated organic to sandy soils (NLSS−C, NLSS−LW, NLSM−T, CPSI−CG). Jul−Aug; Sep−Oct and persisting. Fig. 97

#### Smilax rotundifolia

L.

Smilax rotundifolia Taxon concept: [= RAB, GW, FNA, Weakley]

##### Distribution

Lake Waccamaw: ♦

##### Notes

Perennial vines. Juncture of eulittoral and supralittoral (NLSS−LW). Apr−May; Sep−Oct and persisting. Fig. 98

#### Smilax walteri

Pursh

Smilax walteri Taxon concept: [= RAB, GW, FNA, Weakley]

##### Distribution

Horseshoe Lake (Occasional): *Howell HOLA−13, 21, 25* (NCSC!)

Lake Waccamaw (Occasional): *Howell LAWA−29, 55, 162* (NCSC!)

##### Notes

Perennial vines. Eulittoral zone (NLSS−LW, CPSI−CG). Late Apr−May; Sep−Nov and persisting. Fig. 99

### Xyridaceae

#### Xyris fimbriata

Elliott

Xyris fimbriata Taxon concept: [= RAB, GW, FNA, Weakley]

##### Distribution

Horseshoe Lake: *Rothfels*, *Burge*, *Duke Natural History Society 2400, 2404* (DUKE!)

Lake Waccamaw: ►

Singletary Lake: *Frey* *s.n.* (NCU!)

##### Notes

**Figure 100.**
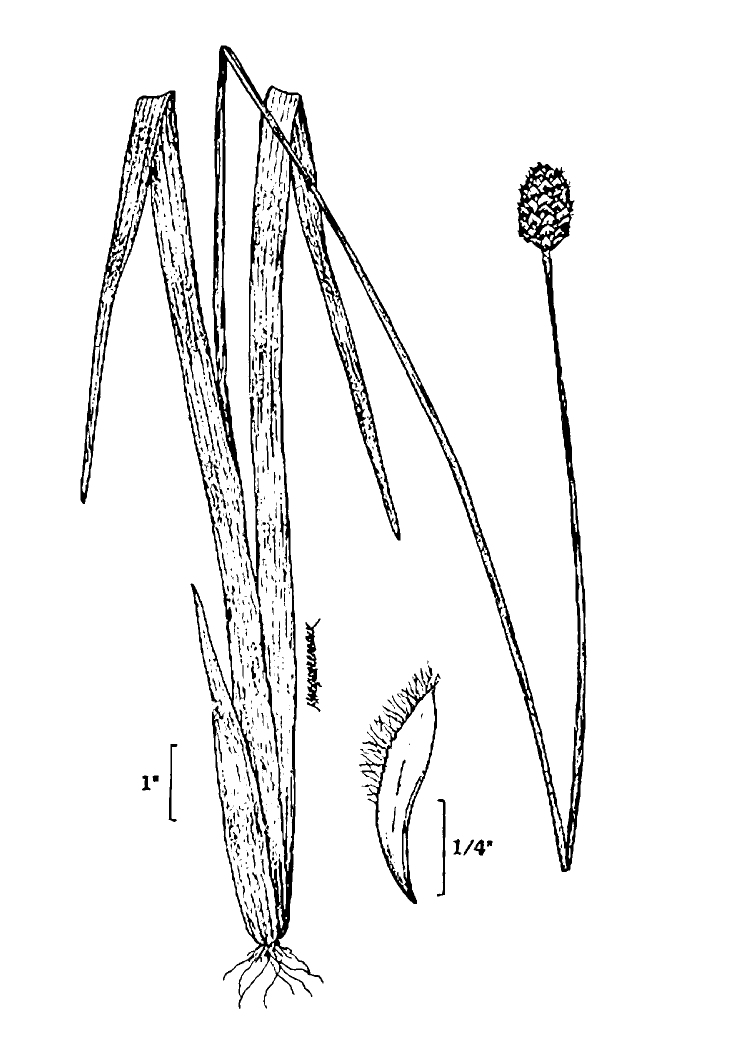
*Xyris fimbriata* (illustration from United States Department of Agriculture, Natural Resource Conservation Service PLANTS Database / United States Department of Agriculture, Natural Resource Conservation Service 2015)

Perennial herbs. Eulittoral zone (NLSS−C, NLSM−T, NLSS−LW, CPSI–CG). Sep−Oct. Fig. 100

#### Xyris iridifolia

Chapm.

Xyris iridifolia Taxon concept: [= RAB, GW; < X. laxifolia Mart. var. iridifolia (Chapm.) Kral – FNA; = Weakley]

##### Ecological interactions

###### Conservation status

W7; S2, G4G5T4T.

##### Distribution

Salters Lake: ♦

##### Notes

Perennial herbs. Eulittoral zone (NLSS–C). Jul–Sep.

#### Xyris jupicai

Rch.

Xyris jupicai Taxon concept: [= RAB, GW, FNA, Weakley]

##### Distribution

Little Singletary Lake (Infrequent): *Howell LISI−46* (NCSC!)

##### Notes

Annual, rarely biennial, herbs. Eulittoral zone (NLSS−C, NLSM−T). Jul−Sep. Fig. 101

#### Xyris smalliana

Nash

Xyris smalliana Taxon concept: [= RAB, GW, FNA, Weakley]

##### Ecological interactions

###### Conservation status

W1; S3, G5.

##### Distribution

Horseshoe Lake: *R.L Wilbur 81092* (DUKE!)

Jones Lake (Occasional): *Howell JOLA−42* (NCSC!)

Lake Waccamaw (Abundant): *Howell LAWA−114, 125, 142, 144* (NCSC!); *LeBlond 3996* (NCU)

Salters Lake: *Beckman & Linnenburger 24* (DUKE!); *Grant* *s.n.* (NCU)

Singletary Lake (Occasional): *Howell SILA−29* (NCSC!); *Rothfels & O’ Reilly, Shaw Lab s.n.* (DUKE!)

##### Notes

Perennial herbs. Eulittoral zone (NLSS−C, NLSS−LW, NLSM–T, CPSI–CG). Jul−Aug. Fig. 102

### BASAL ANGIOSPERMS, MAGNOLIIDS, and EUDICOTYLEDONS

#### Families represented: 55 (BA: 2; M: 2; E: 51)

### Altingiaceae

#### Liquidambar styraciflua

L.

Liquidambar styraciflua Taxon concept: [= RAB, GW, FNA, Weakley]

##### Distribution

Bay Tree Lake (Infrequent): *Howell BATR−47* (NCSC!)

Lake Waccamaw (Occasional): *Howell LAWA−45, 133* (NCSC!)

##### Notes

Trees. Juncture of eulittoral and supralittoral zones (NLSS–LW). Apr–May; Aug–Sep. Fig. 103

### Amaranthaceae

#### Alternanthera philoxeroides

(Mart.) Griseb.

Alternanthera philoxeroides Basionym: *Bucholzia philoxeroides* Mart.
Alternanthera philoxeroides Taxon concept: [= RAB, FNA, Weakley]

##### Distribution

Lake Waccamaw (Infrequent): *Beal 543, 1776* (DUKE!); *Howell LAWA−65* (NCSC!)

##### Notes

Perennial herbs. Eulittoral zone; calm, quiet waters (NLSS–LW). Apr–Oct. Fig. 104

### Anacardiaceae

#### Rhus copallinumvar.copallinum

Rhus copallinumvar.copallinum Taxon concept: [< *R. copallina* L. – RAB; = Weakley]

##### Distribution

Bakers Lake (Rare): *Howell BALA−10* (NCSC!)

##### Notes

Shrubs or small trees. Juncture of eulittoral and supralittoral zones (NLSS−C). Jul−Sep; Aug−Oct. Fig. 105

#### Toxicodendron radicansvar.radicans

Toxicodendron radicansvar.radicans Taxon concept: [< *Rhus radicans* L. – RAB; < *T. radicans* (L.) Kuntze – GW; = Weakley]

##### Distribution

Bay Tree Lake (Infrequent): •

Lake Waccamaw (Occasional): *Howell LAWA−82, 152* (NCSC!)

##### Notes

Shrubs or lianas. Eulittoral zone; typically growing on woody shrubs and trees at or just below the high water mark (NLSS−LW, NLSM−LWP). Late Apr−May; Aug−Oct. Fig. 106

### Apiaceae

#### Centella asiatica

(L.) Urb.

Centella asiatica Basionym: *Hydrocotyle asiatica* L.
Centella asiatica Taxon concept: [= RAB, GW, FNA, Weakley]

##### Distribution

Lake Waccamaw (Frequent): *Howell LAWA−25, 115* (NCSC!)

##### Notes

Perennial herbs. Eulittoral zone (NLSS–LW). Jun–Aug; Jul–Sep. Fig. 107

#### Cicuta maculatavar.maculata

Cicuta maculatavar.maculata Taxon concept: [= *C. maculata* L. – RAB, GW; = Weakley]

##### Distribution

Lake Waccamaw (Rare): *Howell LAWA−121* (NCSC!)

##### Notes

Perennial herbs. Eulittoral zone; moist soils at or just below the mean annual high water mark, also on sandbars and peninsular islands stranded in the littoral zone (NLSS–LW). May−Aug; Jul−Sep. Fig. 108

### Aquifoliaceae

#### Ilex coriacea

(Pursh) Chapm.

Ilex coriacea Basionym: *Prinos coriaceus* Pursh
Ilex coriacea Taxon concept: [= RAB, GW, Weakley]

##### Distribution

Bay Tree Lake (Rare): *Howell BATR−16* (NCSC!)

Jones Lake (Occasional): *Howell JOLA−10, 32* (NCSC!)

##### Notes

Shrubs. Juncture of eulittoral and supralittoral zones (NLSS−C). Apr−May; Sep−Oct. Fig. 109

#### Ilex glabra

(L.) A. Gray

Ilex glabra Basionym: *Prinos glaber* L.
Ilex glabra Taxon concept: [= RAB, GW, Weakley]

##### Distribution

Bakers Lake (Rare): *Howell BALA−9* (NCSC!)

Bay Tree Lake (Rare): *Howell BATR−3, 59* (NCSC!)

Lake Waccamaw (Infrequent): *Howell LAWA−9, 153* (NCSC!)

##### Notes

Shrubs. Juncture of eulittoral and supralittoral zones (NLSS−C, NLSS−LW). May−Jun; Sep−Nov. Fig. 110

### Araliaceae

#### Hydrocotyle umbellata

L.

Hydrocotyle umbellata Taxon concept: [= RAB, GW, RAB, Weakley]

##### Distribution

Bay Tree Lake (Rare): *Howell BATR−45* (NCSC; this specimen is sterile and tentatively referred here)

Lake Waccamaw (Frequent): *Howell LAWA−24, 53* (NCSC; these specimens are sterile and tentatively referred here)

##### Notes

Perennial herbs. Eulittoral zone (NLSS–LW). Apr–Sep. Fig. 111

### Asteraceae

#### Baccharis halimifolia

L.

Baccharis halimifolia Taxon concept: [= RAB, GW, FNA, Weakley]

##### Distribution

Lake Waccamaw (Infrequent): *Howell LAWA−88* (NCSC!)

##### Notes

Shrubs. Eulittoral zone; can be found on saturated soil at or just below the mean annual high water mark or growing from the bases of *Taxodium* in the littoral zone (NLSS−LW). Aug−Oct. Fig. 112f

#### Bidens laevis

(L.) Britton, Sterns & Poggenb.

Bidens laevis Basionym: *Helianthus laevis* L.
Bidens laevis Taxon concept: [= RAB, GW, FNA, Weakley]

##### Distribution

Lake Waccamaw: *Radford 683* (NCU!)

##### Notes

**Figure 113.**
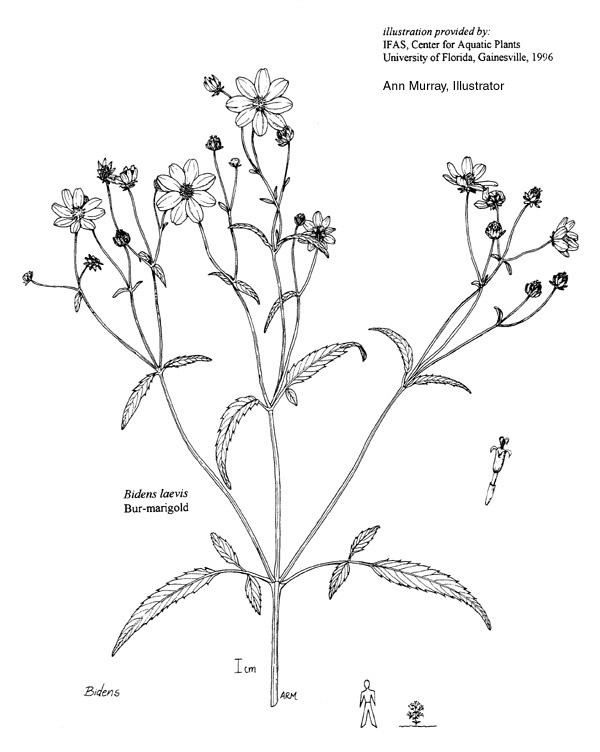
*Bidens laevis* (illustration from Center for Aquatic and Invasive Plants, University of Florida, IFAS 2015)

Annual herbs. Eulittoral zone; wet sandy beaches and sand bars (NLSS−LW). (Aug−Nov). The first author did not encounter this taxon in the field, but a single voucher confirms its historical presence. Fig. 113

#### Boltonia asteroidesvar.glastifolia

(Hill) Fernald

Boltonia asteroidesvar.glastifolia Basionym: *Matricaria glastifolia* Hill
Boltonia asteroidesvar.glastifolia Taxon concept: [< *B. asteroides* (L.) L’Hér – RAB; < *Boltonia* spp. – GW (formal treatment of the genus lacking); < B. asteroides (L.) L’Hér var. asteroides – FNA; = Weakley]

##### Ecological interactions

###### Conservation status

SR−O; S2,G5TNR.

##### Distribution

Lake Waccamaw (Infrequent): *Howell LAWA−1, 158* (NCSC!)

##### Notes

Perennial herbs. Eulittoral zone (NLSS−LW). Aug−Sep. Fig. 114

#### Erigeron vernus

(L.) Torr. & A. Gray

Erigeron vernus Basionym: *Aster vernus* L.
Erigeron vernus Taxon concept: [= RAB, GW, FNA, Weakley]

##### Distribution

Lake Waccamaw: ♦

##### Notes

**Figure 115.**
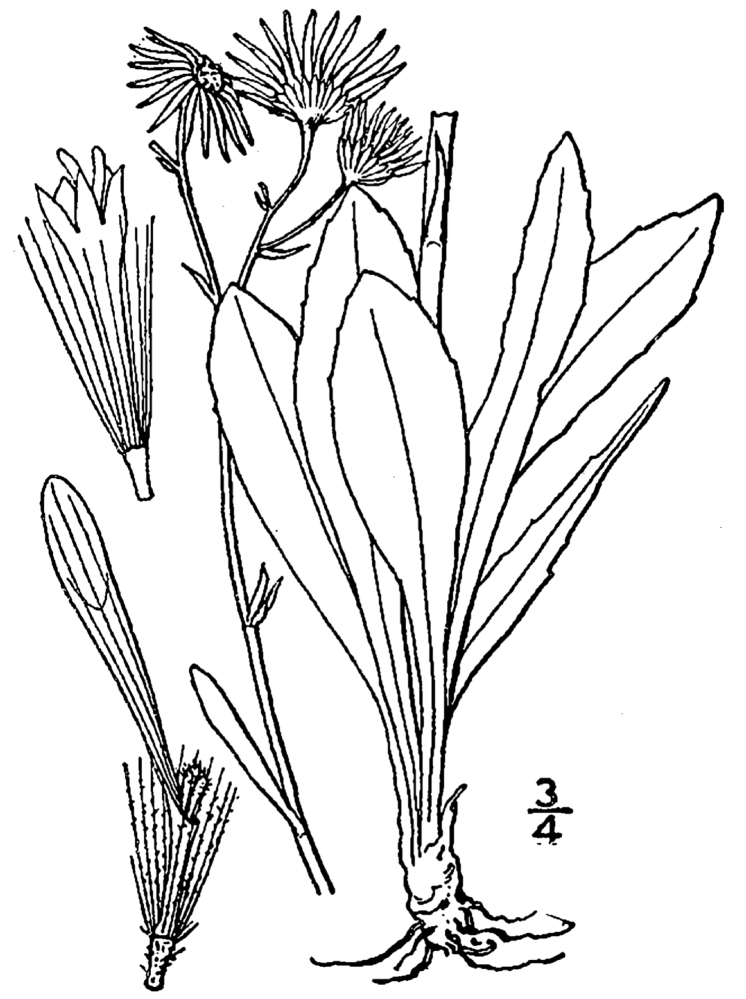
*Erigeron vernus* (illustration from Britton and Brown 1913)

Biennial or short-lived perennial herbs. Eulittoral zone (NLSS−LW). Late Mar−Jun. Fig. 115

#### Eupatorium capillifolium

(Lam.) Small ex Porter & Britton

Eupatorium capillifolium Basionym: *Artemisia capillifolia* Lam.
Eupatorium capillifolium Taxon concept: [= E. capillifolium (Lam.) Small ex Porter & Britton var. capillifolium – RAB; = GW, FNA, Weakley]

##### Distribution

Lake Waccamaw (Infrequent): *Howell LAWA– 141* (NCSC!)

##### Notes

Perennial herbs. Eulittoral zone; usually in a stunted form where detritus has washed ashore just under the maximum annual high water mark (NLSS−LW). Sep−Nov. Fig. 116

#### Eupatorium mohrii paludicola

E.E. Schilling & LeBlond

##### Distribution

Lake Waccamaw (Rare): *Howell LAWA−6* (NCSC!)

##### Notes

Perennial herbs. Eutlittoral zone (NLSS−LW). Aug−Oct. Fig. 117

#### Euthamia caroliniana

(L.) Greene ex Porter & Britton

Euthamia caroliniana Basionym: *Erigeron carolinianus* L.
Euthamia caroliniana Taxon concept: [> *Solidago microcephala* (Nutt.) Bush – RAB; > *Solidago tenuifolia* Pursh – RAB; < *E. tenuifolia* – GW (also see *E. hirtipes*); > *E. minor* (Michx.) Greene – GW; = FNA, Weakley]

##### Distribution

Lake Waccamaw (Rare): *Howell LAWA−12* (NCSC!)

##### Notes

Perennial herbs. Eulittoral zone (NLSS−LW). Sep−Dec. Fig. 118

#### Hypochaeris radicata

L.

Hypochaeris radicata Taxon concept: [= *Hypochoeris radicata* L. – RAB; = FNA, Weakley]

##### Distribution

Bay Tree Lake (Rare): *Howell BATR−32* (NCSC!)

##### Notes

Perennial herbs. Eulittoral zone; moist sandy shores. Apr−Oct. Fig. 119

#### Krigia virginica

(L.) Willd.

Krigia virginica Basionym: *Hyoseris virginica* L.
Krigia virginica Taxon concept: [= RAB, GW, FNA, Weakley]

##### Distribution

Bay Tree Lake (Rare): *Howell BATR−20* (NCSC!)

##### Notes

Annual herbs. Eulittoral zone; moist sandy shores. Late Mar−Jul. Fig. 120

#### Mikania scandens

(L.) Willd.

Mikania scandens Basionym: *Eupatorium scandens* L.
Mikania scandens Taxon concept: [= RAB, GW, Weakley]

##### Distribution

Lake Waccamaw (Infrequent): *Howell LAWA−161* (NCSC!)

##### Notes

Perennial, sometimes lianescent, vines. Eulittoral zone; usually sprawling and climbing on small shrubs and trees (NLSS−LW). Jun−Oct. Fig. 121

#### Pluchea baccharis

(Mill.) Pruski

Pluchea baccharis Basionym: *Conyza baccharis* Mill.
Pluchea baccharis Taxon concept: [= *P. rosea* R.K. Godfrey – RAB; = P. rosea R.K. Godfrey var. rosea – GW; = FNA, Weakley]

##### Distribution

Lake Waccamaw (Frequent): *Howell LAWA−2, 101, 148* (NCSC!)

##### Notes

Perennial herbs. Eulittoral zone (NLSS−LW). Jun−Jul. Fig. 122

#### Sclerolepis uniflora

(Walter) Britton, Sterns & Poggenb.

Sclerolepis uniflora Basionym: *Ethulia uniflora* Walter
Sclerolepis uniflora Taxon concept: [= RAB, GW, FNA, Weakley]

##### Distribution

Lake Waccamaw (Frequent): *Howell LAWA−18, 23, 103, 108* (NCSC!)

##### Notes

Perennial herbs. Eulittoral zone (NLSS−LW). May−Aug; Jul−Oct. Fig. 123

#### Solidago fistulosa

Mill.

Solidago fistulosa Taxon concept: [= RAB, GW, FNA, Weakley]

##### Distribution

Bay Tree Lake (Rare): *Howell BATR−64, 65* (NCSC!)

Horseshoe Lake: *Buell 2266* (NCSC!)

##### Notes

Perennial herbs. Eulittoral zone; saturated peaty to sandy soils at or just below the mean annual high water mark (CPSI−CG). Aug−Nov. Fig. 124

### Betulaceae

#### Alnus serrulata

(Aiton) Willd.

Alnus serrulata Basionym: *Betula serrulata* Aiton
Alnus serrulata Taxon concept: [= RAB, GW, FNA, Weakley]

##### Distribution

Lake Waccamaw (Occasional): *Howell LAWA−39, 170* (NCSC!); *Matthews 683* (NCU!)

##### Notes

Shrubs. Eulittoral zone (NLSS–LW). Feb–Mar; Aug–Oct. Fig. 125

#### Betula nigra

L.

Betula nigra Taxon concept: [= RAB, GW, FNA, Weakley]

##### Distribution

Bay Tree Lake (Occasional): *Howell BATR−13, 19* (NCSC!)

Lake Waccamaw (Occasional): *Blomquist 15004* (DUKE!); *Howell LAWA−37, 63* (NCSC!)

Little Singletary Lake (Infrequent): *Howell LISI−29* (NCSC!)

##### Notes

Trees. Eulittoral zone (NLSS–LW). Mar–Apr; May–Jun. Fig. 126

### Bignoniaceae

#### Campsis radicans

(L.) Bureau

Campsis radicans Basionym: *Bignonia radicans* L.
Campsis radicans Taxon concept: [= RAB, GW, Weakley]

##### Distribution

Lake Waccamaw (Infrequent): *Howell LAWA−81* (NCSC!)

##### Notes

Lianas. Eulittoral zone; climbing on young trees and shrubs at or just below the mean annual high water mark (NLSS−LW). Jun−Jul; Sep−Oct. Fig. 127

### Cabombaceae

#### Brasenia schreberi

J.F. Gmel.

Brasenia schreberi Taxon concept: [= RAB, GW, FNA, Weakley]

##### Distribution

Horseshoe Lake (Infrequent): *Howell HOLA−43* (NCSC!)

##### Notes

Perennial herbs. Infralittoral zone (CPSI–CG). Jun–Oct. Fig. 128

#### Cabomba caroliniana

A. Gray

Cabomba caroliniana Taxon concept: [= RAB, GW, FNA, Weakley]

##### Distribution

Horseshoe Lake (Infrequent): *Howell HOLA−26* (NCSC!)

##### Notes

Perennial herbs. Infralittoral Zone (CPSI–CG). May–Sep. Fig. 129

### Campanulaceae

#### Lobelia glandulosa

Walter

Lobelia glandulosa Taxon concept: [= RAB, GW, Weakley]

##### Distribution

Lake Waccamaw: ¤

##### Notes

Perennial herbs. Eulittoral zone (NLSS−LW). Sep−Oct.

#### Lobelia nuttallii

Roem. & Schult.

Lobelia nuttallii Taxon concept: [= RAB, GW, Weakley]

##### Distribution

Horseshoe Lake (Rare): *Howell HOLA−47* (NCSC!)

##### Notes

Perennial herbs. Eulittoral zone; moist sandy soil at or just below the high water mark (CPSI−CG). May−Nov. Fig. 130

### Caryophyllaceae

#### Stipulicida setaceavar.setacea

Stipulicida setaceavar.setacea Taxon concept: [< *S. setacea* Michx. – RAB; = FNA, Weakley]

##### Distribution

Bay Tree Lake (Rare): *Howell BATR−21* (NCSC!)

##### Notes

Annual, or short-lived perennial, herbs. Juncture of eulittoral and supralittoral zones. May−Aug. Fig. 131

### Clethraceae

#### Clethra alnifolia

L.

Clethra alnifolia Taxon concept: [< C. alnifolia L. var. alnifolia – RAB; = GW, FNA, Weakley]

##### Distribution

Bay Tree Lake (Occasional): *Howell BATR−12* (NCSC!)

Jones Lake (Occasional): *Howell JOLA−5* (NCSC!)

Lake Waccamaw (Occasional): *Howell LAWA−42, 132, 150* (NCSC!)

Little Singletary Lake (Occasional): *Howell LISI−7, 57* (NCSC!)

Singletary Lake (Occasional): *Howell SILA−15, 28* (NCSC!)

##### Notes

Shrubs. Juncture of supralittoral and eulittoral zones; can also establish itself on stumps, logs, and tree bases in the eulittoral zone (NLSS−C, NLSS−LW, NLSM−T, CPSI−CG). Jun−Jul; Sep−Oct. Fig. 132

### Cyrillaceae

#### Cyrilla racemiflora

L.

Cyrilla racemiflora Taxon concept: [= RAB, GW, FNA, Weakley]

##### Distribution

Bay Tree Lake (Frequent): *Howell BATR−38, 41, 60* (NCSC!)

Horseshoe Lake (Occasional): *Howell HOLA−37* (NCSC!)

Jones Lake (Occasional): •

Lake Waccamaw (Frequent): *Howell LAWA−100* (NCSC!)

Little Singletary Lake (Frequent): *Howell LISI−32* (NCSC!)

Singletary Lake (Frequent): *Howell SILA−11, 23* (NCSC!)

##### Notes

Shrubs or small trees. Juncture of eulittoral and supralittoral zones (NLSS−C, NLSM−T, NLSS−LW, CPSI−CG). May−Jul; Sep−Oct. Fig. 133

### Droseraceae

#### Drosera intermedia

Hayne

Drosera intermedia Taxon concept: [= RAB, GW, Weakley]

##### Distribution

Horseshoe Lake (Frequent): *Howell HOLA−28, 40, 49* (NCSC!)

Little Singletary Lake (Occasional): *Howell LISI−52, 56* (NCSC!)

##### Notes

Perennial herbs. Eulittoral zone and floating bogs (NLSS−C, NLSM−T, CPSI−CG, FB). Jul–Sep. Fig. 134

### Ebenaceae

#### Diospyros virginiana

L.

Diospyros virginiana Taxon concept: [= RAB, GW, FNA, Weakley]

##### Distribution

Lake Waccamaw (Rare): *Howell LAWA−80* (NCSC!)

##### Notes

Trees. Juncture of eulittoral and supralittoral zones (NLSS−LW). May−Jun; Sep−Dec. Fig. 135

### Ericaceae

#### Chamaedaphne calyculata

(L.) Moench

Chamaedaphne calyculata Basionym: *Andromeda calyculata* L.
Chamaedaphne calyculata Taxon concept: [= *Cassandra calyculata* (L.) D. Don − RAB; = FNA, Weakley]

##### Distribution

Bakers Lake (Occasional): *Howell BALA−1* (NCSC!)

Horseshoe Lake (Occasional): *Howell HOLA−1, 6, 42* (NCSC!)

Little Singletary Lake (Occasional): *Howell LISI−15, 24* (NCSC!)

Singletary Lake: *Fox*, *Wells*, *Sharp*, *Whitford*, *Fairchild s*. *n.* (NCSC!)

##### Notes

Shrubs. Eulittoral zone, either in shallow water or in saturated organic soils at the high water mark (NLSS–C, NLSM-T, CPSI–CG, FB). Mar–Apr; Jun–Oct. Fig. 136

#### Eubotrys racemosa

(L.) Nutt.

Eubotrys racemosa Basionym: *Andromeda racemosa* L.
Eubotrys racemosa Taxon concept: [= *Leucothoe racemosa* (L.) A. Gray − RAB; = FNA, Weakley]

##### Distribution

Horseshoe Lake (Occasional): *Howell HOLA−12* (NCSC!)

Lake Waccamaw (Occasional): *Howell LAWA−40, 151* (NCSC!); *Matthews* *s.n.* (NCU!)

Jones Lake (Occasional): *Howell JOLA−30* (NCSC!)

Little Singletary Lake (Occasional): *Howell LISI−1* (NCSC!)

Salters Lake (Occasional): *Howell SALA−12* (NCSC!)

Singletary Lake (Occasional): *Howell SILA−7* (NCSC!)

##### Notes

Shrubs. Juncture of eulittoral and supralittoral zones; sometimes on the bases of large Taxodium trunks (NLSS–C, NLSS–LW, NLSM–T, CPSI–CG). Late Mar–early Jun; Sept–Oct. Fig. 137

#### Lyonia ligustrinavar.foliosiflora

(Michx.) Fernald

Lyonia ligustrinavar.foliosiflora Basionym: Andromeda paniculata var. foliosiflora Michx.
Lyonia ligustrinavar.foliosiflora Taxon concept: [< *L. ligustrina* (L.) DC. – RAB; = GW, FNA, Weakley]

##### Distribution

Bakers Lake (Infrequent): *Howell BALA−17* (NCSC!)

Jones Lake (Infrequent): *Howell JOLA−31* (NCSC!)

Salters Lake (Infrequent): *Howell SALA−17* (NCSC!)

##### Notes

Shrubs. Juncture of eulittoral and supralittoral zones; sometimes growing from the bases of large *Taxodium* (NLSS–C). Late Apr–Jul; Sep–Oct. Two varieties of *Lyonia ligustrina* are commonly recognized: var. *foliosiflora* (Michx.) Fernald, with numerous and conspicuous leaf-like bracts in the inflorescence, and var. *ligustrina*, with no or few leaf-like bracts in the inflorescence. The material collected by the first author is var. *foliosiflora*, the more common variety found in the North Carolina Coastal Plain. Fig. 138

#### Lyonia lucida

(Lam.) K. Koch

Lyonia lucida Basionym: *Andromeda lucida* Lam.
Lyonia lucida Taxon concept: [= RAB, GW, FNA, Weakley]

##### Distribution

Bakers Lake (Occasional): *Howell BALA−8* (NCSC!)

Bay Tree Lake (Occasional): *Howell BATR−17* (NCSC!)

Horseshoe Lake (Occasional): *Howell HOLA−7* (NCSC!)

Jones Lake (Frequent): *Howell JOLA−11, 19* (NCSC!)

Lake Waccamaw (Occasional): *Howell LAWA−35* (NCSC!)

Little Singletary (Occasional): *Howell LISI−5, 36* (NCSC!)

Salters Lake (Frequent): *Howell SALA−5, 11* (NCSC!)

Singletary Lake (Frequent): *Howell SILA−3* (NCSC!)

##### Notes

Shrubs. Juncture of eulittoral and supralittoral zones; sometimes growing from the bases of mature *Taxodium* (NLSS–C, NLSS–LW, NLSM–T, CPSI–CG). Apr–early Jun; Sep–Oct. Fig. 139

#### Rhododendron viscosumvar.serrulatum

(Small) H.E. Ahles

Rhododendron viscosumvar.serrulatum Basionym: *Azalea serrulata* Small
Rhododendron viscosumvar.serrulatum Taxon concept: [= RAB; < *R. viscosum* – GW, FNA; = Weakley]

##### Distribution

Singletary Lake (Rare): *Howell SILA−33, 34* (NCSC!)

##### Notes

Shrubs. Juncture of eulittoral and supralittoral zones (NLSS–C). Late May–Jun; Jul–Oct. Fig. 140

#### Vaccinium formosum

Andrews

Vaccinium formosum Taxon concept: [< *V. corymbosum* L. – RAB; = *V. australe* Small – GW; < *V. corymbosum* L. – FNA; = Weakley]

##### Distribution

Bay Tree Lake (Occasional): *Howell BATR−33* (NCSC!)

Jones Lake (Occasional): *Howell JOLA−13, 24, 26* (NCSC!)

Salters Lake (Occasional): *Howell SALA−10* (NCSC!)

Singletary Lake (Occasional): *Howell SILA−18* (NCSC!)

##### Notes

Shrubs. Juncture of eulittoral and supralittoral zones (NLSS−C, NLSM−T, NLSS−LW). Late Feb−May; Jun−Aug. Fig. 141

#### Vaccinium fuscatum

Aiton

Vaccinium fuscatum Taxon concept: [= *V. atrococcum* (Gray) Heller – RAB; = GW; < *V. corymbosum* L. –FNA; Weakley]

##### Distribution

Bakers Lake (Occasional): *Howell BALA−7* (NCSC!)

Lake Waccamaw (Occasional): *Howell LAWA−49* (NCSC!)

Little Singletary Lake (Occasional): *Howell LISI−14, 39, 40* (NCSC!)

Salters Lake (Occasional): *Howell SALA−16* (NCSC!)

Singletary Lake (Occasional): *Howell SILA−21* (NCSC!)

##### Notes

Shrubs. Juncture of eulittoral and supralittoral zones (NLSS−C, NLSM−T, NLSS−LW). Late Feb−May; Jun−Aug. Fig. 142

#### Zenobia pulverulenta

(W. Bartram ex Willd.) Pollard

Zenobia pulverulenta Basionym: *Andromeda pulverulenta* W. Bartram ex Willd.
Zenobia pulverulenta Taxon concept: [= RAB, GW, FNA, Weakley]

##### Distribution

Bakers Lake (Infrequent): *Howell BALA−5* (NCSC!)

Horseshoe Lake (Occasional): *Howell HOLA−17, 22, 35* (NCSC!)

Jones Lake (Occasional): *Howell JOLA−25* (NCSC!)

Little Singletary Lake (Occasional): *Howell LISI−10, 23* (NCSC!)

Singletary Lake: *Fox & Boyce 3781* (NCSC!); *Fox*, *Wells*, *Sharp*, *Whitford*, *Fairchild 1708* (NCSC!)

##### Notes

Shrubs. Juncture of supralittoral and eulittoral zones; sometimes growing on the bases of mature *Taxodium* (NLSS–C, NLSM–T, CPSI–CG, FB). Apr–Jun; Sep–Oct. Fig. 143

### Euphorbiaceae

#### Triadica sebifera

(L.) Small

Triadica sebifera Basionym: *Croton sebifer* L.
Triadica sebifera Taxon concept: [= *Sapium sebiferum* (L.) Roxb. – RAB, GW; = Weakley]

##### Distribution

Lake Waccamaw (Infrequent): *Howell LAWA−92* (NCSC!)

##### Notes

Trees. Eulittoral zone (NLSS–LW). May–Jun; Aug–Nov. Fig. 144

### Fabaceae

#### Wisteria frutescens

(L.) Poir.

Wisteria frutescens Basionym: *Glycine frutescens* L.
Wisteria frutescens Taxon concept: [= RAB, GW, Weakley]

##### Distribution

Bay Tree Lake (Occasional): *Howell BATR−37* (NCSC!)

Lake Waccamaw (Occasional): *Howell LAWA−99, 117* (NCSC!)

Singletary Lake (Infrequent): *Howell SILA−35* (NCSC!)

##### Notes

Lianas. Eulittoral zone (NLSS–LW). Apr–May; Jun–Sep. Fig. 145

### Fagaceae

#### Quercus nigra

L.

Quercus nigra Taxon concept: [= RAB, GW, FNA, Weakley]

##### Distribution

Lake Waccamaw: *Godfrey 6320* (NCSC!)

##### Notes

Trees. Juncture of eulittoral and supralittoral zones (NLSS–LW). Apr; Sep–Nov. Fig. 146

### Gelsemiaceae

#### Gelsemium sempervirens

(L.) J. St.−Hil.

Gelsemium sempervirens Basionym: *Bignonia sempervirens* L.
Gelsemium sempervirens Taxon concept: [= RAB, GW, Weakley]

##### Distribution

Bay Tree Lake (Occasional): *Howell BATR−10, 25* (NCSC!)

Horseshoe Lake (Rare): *Howell HOLA−4* (NCSC!)

Jones Lake (Infrequent): *Howell JOLA−34* (NCSC!)

Lake Waccamaw (Occasional): *Howell LAWA−41* (NCSC!)

Little Singletary Lake (Infrequent): *Howell LISI−34* (NCSC!)

Singletary Lake (Infrequent): *Howell SILA−36* (NCSC!)

##### Notes

Lianas. Eulittoral zone (NLSS–C, NLSS–LW, NLSM–T, CPSI–CG). Mar–early May; Sept–Nov. Fig. 147

### Hydrangeaceae

#### Decumaria barbara

L.

Decumaria barbara Taxon concept: [= RAB, GW, FNA, Weakley]

##### Distribution

Lake Waccamaw (Rare): *Dumond 1621* (NCU!); *Godfrey 52278* (NCSC!); *Howell LAWA−86* (NCSC!)

##### Notes

Lianas. Eulittoral zone; climbing on trees and shrubs at or just below the maximum annual high water mark (NLSS−LW, NLSM−LWP). May−Jun; Jul−Oct. Fig. 148

### Hypericaceae

#### Hypericum canadense

L.

Hypericum canadense Taxon concept: [= RAB, GW, Weakley]

##### Distribution

Horseshoe Lake (Rare): *Howell HOLA−48* (NCSC!)

##### Notes

Annual or perennial herbs. Eulittoral zone; moist sandy soils at or just below the maximum annual high water mark (CPSI−CG). Jul−Sep. Fig. 149

#### Hypericum mutilumvar.mutilum

Hypericum mutilumvar.mutilum Taxon concept: [< *H. mutilum* L. – RAB, GW; = Weakley]

##### Distribution

Lake Waccamaw (Rare): *Howell LAWA−139* (NCSC!)

##### Notes

Annual or perennial herbs. Eulittoral zone; at or just below the maximum annual high water mark (NLSS−LW). Jun−Oct. Fig. 150

#### Hypericum virginicum

L.

Hypericum virginicum Taxon concept: [= RAB; = *Triadenum virginicum* (L.) Raf. – GW; = Weakley]

##### Distribution

Bay Tree Lake (Occasional): *Howell BATR−9, 56* (NCSC!)

Horseshoe Lake (Occasional): *Howell HOLA−33* (NCSC!)

Little Singletary Lake (Occasional): *Howell LISI−54* (NCSC!)

##### Notes

Perennial herbs. Eulittoral zone (NLSS−C, NLSM−T, CPSI−CG, FB). Jul−Sep. Fig. 151

#### Hypericum walteri

J.F. Gmel.

Hypericum walteri Taxon concept: [= RAB; = *Triadenum walteri* (J.F. Gmel.) Gleason – GW; = Weakley]

##### Distribution

Lake Waccamaw (Occasional): *Howell LAWA−20, 134, 149* (NCSC!); *Wilbur 9363* (DUKE!)

##### Notes

Perennial herbs. Eulittoral zone (NLSS−LW). Jul−Sep. Fig. 152

### Iteaceae

#### Itea virginica

L.

Itea virginica Taxon concept: [= RAB, GW, FNA, Weakley]

##### Distribution

Bakers Lake (Infrequent): *Howell BALA−6* (NCSC!)

Horseshoe Lake (Occasional): *Howell HOLA−9* (NCSC!)

Jones Lake (Occasional): *Howell JOLA−27* (NCSC!)

Lake Waccamaw (Occasional): *Howell LAWA−85* (NCSC!)

Singletary Lake (Occasional): *Howell SILA−2* (NCSC!)

##### Notes

Shrubs. Juncture of eulittoral and supralittoral zones; sometimes establishing itself on stumps, logs, and bases of trees in the eulittoral zone (NLSS−C, NLSS−LW, CPSI−CG). May−Jun. Fig. 153

### Juglandaceae

#### Carya glabra

(Mill.) Sweet

Carya glabra Basionym: *Juglans glabra* Mill.
Carya glabra Taxon concept: [= RAB, GW; < *C. glabra* (Mill.) Sweet – FNA; = Weakley]

##### Distribution

Lake Waccamaw (Rare): *Howell LAWA−96* (NCSC!); *Matthews* *s.n.* (DUKE!)

##### Notes

Trees. Juncture of eulittoral and supralittoral zones (NLSS−LW). Apr−May. Fig. 154

### Lamiaceae

#### Lycopus angustifolius

Elliott

Lycopus angustifolius Taxon concept: [< L. rubellus Moench var. angustifolius (Elliott) H.E. Ahles – RAB, GW; = Weakley]

##### Ecological interactions

###### Conservation status

SR−P; S1, G4?Q.

##### Distribution

Lake Waccamaw (Infrequent): *Howell LAWA−4, 156, 157* (NCSC!)

##### Notes

Perennial herbs. Eulittoral zone (NLSS–LW). Jun–Sep. Fig. 155

### Lauraceae

#### Persea palustris

(Raf.) Sarg.

Persea palustris Basionym: *Tamala palustris* Raf.
Persea palustris Taxon concept: [< *P. borbonia* – RAB; = GW, FNA, Weakley]

##### Distribution

Bakers Lake (Occasional): *Howell BALA−12* (NCSC!)

Jones Lake (Occasional): *Howell JOLA−6, 18* (NCSC!)

Lake Waccamaw (Occasional): *Howell LAWA−61, 69* (NCSC!)

Little Singletary Lake (Occasional): *Howell LISI−6* (NCSC!)

Salters Lake (Occasional): *Buell* *s.n.* (DUKE!, NCSC!); *Howell SALA−6, 20* (NCSC!)

Singletary Lake (Occasional): *Howell SILA−10, 25, 27* (NCSC!)

##### Notes

Shrubs or small trees. Juncture of eulittoral and supralittoral zones (NLSS−C, NLSS−LW). May−Jun; Sep−Oct. Fig. 156

### Lentibulariaceae

#### Utricularia cornuta

Michx.

Utricularia cornuta Taxon concept: [= RAB, GW, Weakley]

##### Distribution

Lake Waccamaw (Rare): *Howell LAWA−109* (NCSC!)

##### Notes

Annual or perennial herbs. Eulittoral zone; commonly in saturated sandy to peaty soils just above current water levels or in 1−4 inches of water (NLSS−LW). May−Sep. Fig. 157

#### Utricularia gibba

L.

Utricularia gibba Taxon concept: [= RAB; = *U. biflora* Lam. – GW; = Weakley]

##### Distribution

Horseshoe Lake (Occasional): *Howell HOLA−19* (NCSC!)

##### Notes

Annual or perennial herbs. Eulittoral and infralittoral zones; Godfrey and Wooten (1981) described the habit as “very much intertwined, forming large floating bunches or mats” (CPSI−CG). May−Sep. Fig. 158

#### Utricularia purpurea

Walter

Utricularia purpurea Taxon concept: [= RAB, GW, Weakley]

##### Distribution

Horseshoe Lake (Occasional): *Howell HOLA−36* (NCSC!)

##### Notes

Annual or perennial herbs. Eulittoral and infralittoral zones; floating bogs (CPSI−CG). May−Sep. Fig. 159

#### Utricularia resupinata

B.D. Greene ex Bigelow

Utricularia resupinata Taxon concept: [= GW, Weakley]

##### Distribution

Lake Waccamaw (Rare): *Howell LAWA−123* (NCSC!)

##### Notes

Annual or perennial herbs. Eulittoral zone; commonly in saturated sandy to peaty soils above current lake levels or in 1−4 inches of water (NLSS−LW). Jun−Aug. Fig. 160

#### Utricularia striata

Leconte ex Torr.

Utricularia striata Taxon concept: [= *U. fibrosa* Walter – RAB, GW; = Weakley]

##### Distribution

Bay Tree Lake (Occasional): *Howell BATR−35, 42* (NCSC!)

Horseshoe Lake (Occasional): *Howell HOLA−27* (NCSC!)

Lake Waccamaw (Occasional): *Howell LAWA−14, 122* (NCSC!)

##### Notes

Perennial herbs. Eulittoral zone; typically seen in shallow water or stranded on saturated organic soils (NLSS−LW, CPSI−CG, FB). May−Nov. Fig. 161

#### Utricularia subulata

L.

Utricularia subulata Taxon concept: [= RAB, GW, Weakley]

##### Distribution

Lake Waccamaw: ♦

Little Singletary Lake (Infrequent): *Howell LISI−28, 49* (NCSC!)

##### Notes

Annual or perennial herbs. Eulittoral zone; typically found in saturated sands and peats (NLSS−C, NLSS−LW, NLSM−T). Mar−Aug. Fig. 162

### Linderniaceae

#### Lindernia dubiavar.dubia

Lindernia dubiavar.dubia Basionym: *Gratiola dubia* L.
Lindernia dubiavar.dubia Taxon concept: [= *L. dubia* (L.) Pennell – RAB, GW; = Weakley]

##### Distribution

Lake Waccamaw: *Radford & Stewart 679* (NCU!)

##### Notes

**Figure 163.**
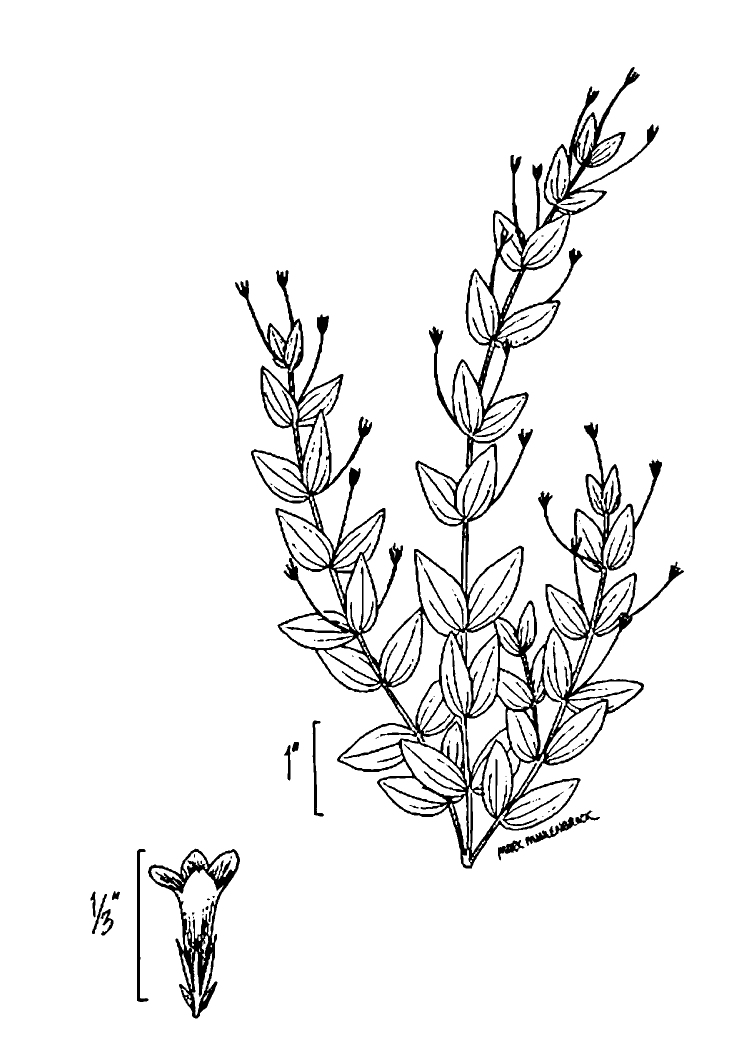
*Lindernia dubia* (illustration from United States Department of Agriculture, Natural Resource Conservation Service PLANTS Database / United States Department of Agriculture, Natural Resource Conservation Service 2015)

Annual or biennial herbs. Eulittoral zone; saturated sandy soils (NLSS−LW). May−Nov. (Fig. 159). The first author did not encounter this taxon in the field, but a single voucher confirms its historic presence (see above). Fig. 163

### Loganiaceae

#### Mitreola petiolata

(Walter ex J.F. Gmelin) Torr. & A. Gray

Mitreola petiolata Basionym: *Cynoctonum petiolatum* Walter ex J.F. Gmelin
Mitreola petiolata Taxon concept: [= *Cynoctonum mitreola* (L.) Britton – RAB; = GW, Weakley]

##### Distribution

Lake Waccamaw (Infrequent): *Howell LAWA−140, 154* (NCSC!)

##### Notes

Annual herbs. Eulittoral zone; shallow water (1−6 inches) or saturated soils above current lake levels (NLSS−LW). Jul−Sep; Sep−Nov. Fig. 164

### Lythraceae

#### Decodon verticillatus

(L.) Elliott

Decodon verticillatus Basionym: *Lythrum verticillatum* L.
Decodon verticillatus Taxon concept: [= RAB, GW, Weakley]

##### Distribution

Bay Tree Lake (Infrequent): •

Horseshoe Lake (Occasional): *Howell HOLA−31* (NCSC!)

Jones Lake (Infrequent): •

Salters Lake (Infrequent): •

##### Notes

Shrubs. Eulittoral zone (NLSS−C, NLSM−T, FB, CPSI−CG). Jul−Sep. Fig. 165

### Magnoliaceae

#### Magnolia virginianavar.virginiana

Magnolia virginianavar.virginiana Taxon concept: [< *M. virginiana* – RAB, GW, FNA; = Weakley]

##### Distribution

Bakers Lake (Occasional): *Howell BALA−3* (NCSC!)

Jones Lake (Occasional): *Howell JOLA−8, 29* (NCSC!)

Lake Waccamaw (Occasional): *Howell LAWA−62* (NCSC!)

Salters Lake (Occasional): *Howell SALA−13* (NCSC!)

Singletary Lake (Occasional): *Howell SILA−5, 19* (NCSC!)

##### Notes

Trees. Juncture of eulittoral and supralittoral zones (NLSS−C, NLSS−LW, NLSM−T). Fig. 166

### Melastomataceae

#### Rhexia aristosa

Britton

Rhexia aristosa Taxon concept: [= RAB, GW, Weakley]

##### Ecological interactions

###### Conservation status

SC–V, FSC; S3, G3G4.

##### Distribution

Horseshoe Lake: ►

##### Notes

Perennial herbs. floating bogs (CPSI–CG, FB). Jun–Sep. Fig. 167

#### Rhexia cubensis

Griseb.

Rhexia cubensis Taxon concept: [= RAB, GW, Weakley]

##### Ecological interactions

###### Conservation status

W1; S3, G4G5.

##### Distribution

Lake Waccamaw (Occasional): *Howell LAWA–113, 126, 129* (NCSC!); *LeBlond 3990* (NCU!)

##### Notes

Perennial herbs. Eulittoral zone (NLS–LW). Jun–Sep. Fig. 168

#### Rhexia marianavar.exalbida

Michx.

Rhexia marianavar.exalbida Taxon concept: [= RAB; < R. mariana var. mariana – GW; = Weakley]

##### Distribution

Horseshoe Lake (Rare): *Howell HOLA−46* (NCSC!)

##### Notes

Perennial herbs. Eulittoral zone (CPSI–CG). Jun–Sep. Fig. 169

#### Rhexia nashii

Small

Rhexia nashii Taxon concept: [< R. mariana var. purpurea Michx. – RAB; = GW, Weakley]

##### Distribution

Bay Tree Lake (Occasional): *Howell HOLA−39, 57* (NCSC!)

Horseshoe Lake (Occasional): *Howell HOLA−44* (NCSC!)

Jones Lake (Infrequent): *Howell JOLA−38,39* (NCSC!)

Little Singletary Lake (Occasional): *Howell LISI−45* (NCSC!)

Singletary Lake (Infrequent): *Howell SILA−26* (NCSC!)

##### Notes

Perennial herbs. Eulittoral zone (NLSS–C, NLSS–LW, NLSM–T, CPSI–CG, FB). May–Oct. Fig. 170

#### Rhexia virginica

L.

Rhexia virginica Taxon concept: [> R. virginica L. var. purshii – RAB; > R. virginica L. var. virginica; = GW, Weakley]

##### Distribution

Little Singletary Lake (Infrequent): *Howell LISI – 47* (NCSC!)

##### Notes

Perennial herbs. Eulittoral zone (NLSM–T, NLSS–C). May–Oct. Fig. 171

### Menyanthaceae

#### Nymphoides aquatica

(J.F. Gmel.) Kuntze

Nymphoides aquatica Basionym: *Villarsia aquatica* J.F. Gmel.
Nymphoides aquatica Taxon concept: [= RAB, GW, Weakley]

##### Distribution

Lake Waccamaw (Occasional): *Harper 954* (NCU!); *Howell LAWA−28, 54* (NCSC!)

##### Notes

Perennial herbs. Eulittoral zone (NLSS−LW). Late Apr−Sep. Fig. 172

### Myricaceae

#### Morella cerifera

(L.) Small

Morella cerifera Basionym: *Myrica cerifera* L.
Morella cerifera Taxon concept [< Myrica cerifera L. var. cerifera – RAB; < *Myrica cerifera* L. – GW, FNA; = Weakley]

##### Distribution

Bay Tree Lake (Infrequent): *Howell BATR−11* (NCSC!)

Jones Lake (Infrequent): *Howell JOLA−15* (NCSC!)

Lake Waccamaw (Occasional): *Dennis 66-15* (DUKE!); *Howell LAWA−36, 169* (NCSC!)

Salters Lake (Infrequent): *Howell SALA−3* (NCSC!)

##### Notes

Shrubs or small trees. Juncture of eulittoral and supralittoral zones (NLSS−C, NLSS−LW). Apr; Aug–Oct. Fig. 173

### Nelumbonaceae

#### Nelumbo lutea

Willd.

Nelumbo lutea Taxon concept: [= RAB, GW, FNA, Weakley]

##### Ecological interactions

###### Conservation status

W7; S2, G4.

##### Distribution

Lake Waccamaw: *Bell 12836* (NCU!); *Leonard*, *Burnham & Ripperton 1748* (NCU!); *Radford 6078* (NCU!); *Schallert 10662* (DUKE!)

##### Notes

**Figure 174.**
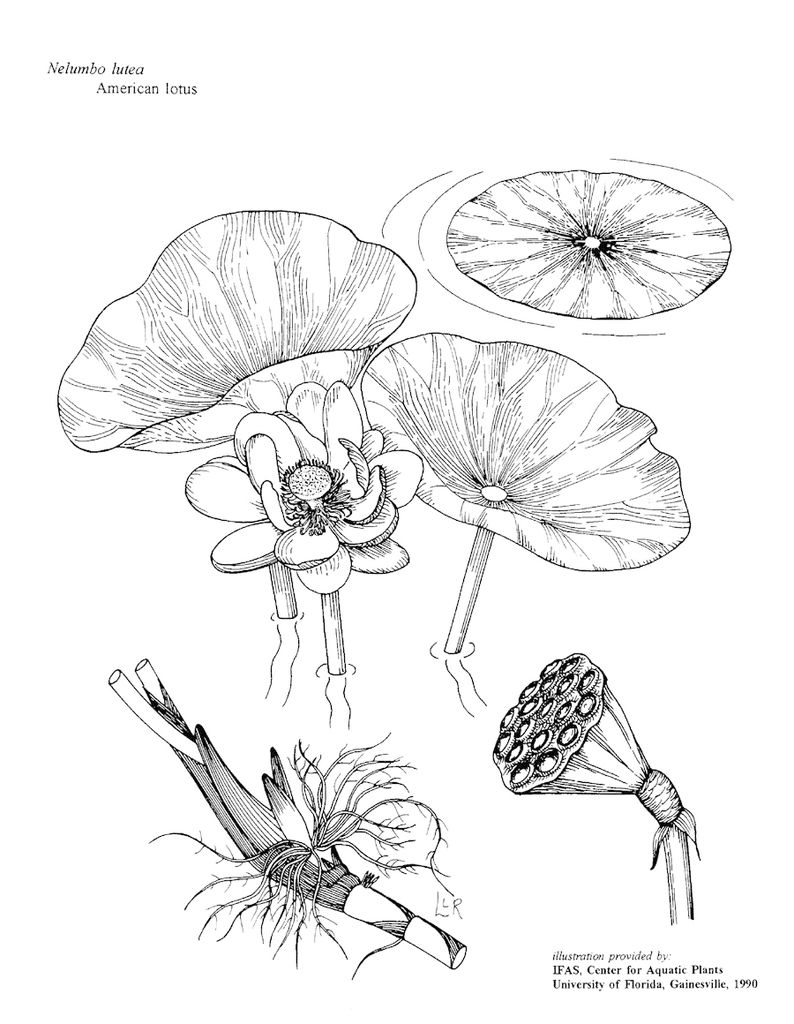
*Nelumbo lutea* (illustration from Center for Aquatic and Invasive Plants, University of Florida, IFAS 2015)

Perennial herbs. Eulittoral and infralittoral zones (NLSS−LW, NLSM−LWP). Jun−Sep. Fig. 174

### Nymphaeaceae

#### Nuphar sagittifolia

(Walter) Pursh

Nuphar sagittifolia Basionym: *Nymphaea sagittifolia* Walter
Nuphar sagittifolia Taxon concept: [< N. luteum (L.) Sibth. & J.E. Smith ssp. sagittifolium (Walter) E.O. Beal – RAB, GW; = FNA, Weakley]

##### Ecological interactions

###### Conservation status

W1, FSC; S2, G5T2.

##### Distribution

Lake Waccamaw (Rare along south and southwest shorelines; frequent elsewhere): *Buell & Godfrey 3505* (NCSC!); *Fox 1878* (NCSC!); *Godfrey & Buell 3505* (NCU!); *Howell LAWA−83* (NCSC!); *Leconte 1085* (DUKE!); *Matthews* *s.n.* (DUKE!, NCU!); *Radford 681, 4348* (NCU!)

##### Notes

Perennial herbs. Infralittoral zone; encountered around dam, northern shorelines, and offshore (NLSS–LW, NLSM–LWP). Apr–Oct. Fig. 175

#### Nymphaea odoratavar.odorata

Nymphaea odoratavar.odorata Taxon concept: [< *N. odorata* – RAB, GW; = FNA, Weakley]

##### Distribution

Horseshoe Lake (Frequent): *Beal 4349* (NCSC!); *Buell & Whitford 1851* (DUKE!, NCSC!); *Howell HOLA−18* (NCSC!)

Lake Waccamaw (Occasional): *Howell LAWA−17, 27, 76* (NCSC!)

Singletary Lake: *Wilbur 60946* (DUKE!)

##### Notes

Perennial herbs. Eulittoral and infralittoral zones (NLSS–LW, NLSM–T, NLSM–LWP, CPSI–CG). Jun–Sep. Fig. 176

### Nyssaceae

#### Nyssa aquatica

L.

Nyssa aquatica Taxon concept: [= RAB, GW, Weakley]

##### Distribution

Lake Waccamaw (Infrequent): *Howell LAWA−56, 89* (NCSC!)

##### Notes

Trees. Eulittoral zone (NLSS−LW). Apr−May; Sep−Oct. Fig. 177

#### Nyssa biflora

Walter

Nyssa biflora Taxon concept: [= N. sylvatica Marshall var. biflora (Walter) Sarg. – RAB, GW; = Weakley]

##### Distribution

Bakers Lake (Frequent): *Howell BALA−11* (NCSC!)

Bay Tree Lake (Occasional): *Howell BATR−48* (NCSC!)

Horseshoe Lake (Occasional): *Howell HOLA−15* (NCSC!)

Lake Waccamaw (Occasional): *Howell LAWA−10* (NCSC!); *Totten* *s.n.* (NCU!)

Little Singletary Lake (Occasional): *Howell LISI−12, 17* (NCSC!)

Salters Lake (Occasional): •

Singletary Lake (Occasional): *Howell SILA−4* (NCSC!)

##### Notes

Trees. Eulittoral zone (NLSS–C, NLSS–LW, NLSM–T, CPSI–CG). Apr–Jun; Aug–Oct. Fig. 178

### Oleaceae

#### Fraxinus caroliniana

Mill.

Fraxinus caroliniana Taxon concept: [= RAB, GW, Weakley]

##### Distribution

Lake Waccamaw (Infrequent): *Howell LAWA−70, 75* (NCSC!)

##### Notes

Trees. Eulittoral zone (NLSS–LW). May; Jul–Oct. Fig. 179

### Onagraceae

#### Ludwigia brevipes

(Long) Eames

Ludwigia brevipes Basionym: *Ludwigiantha brevipes* Long
Ludwigia brevipes Taxon concept: [= RAB, GW, Weakley]

##### Ecological interactions

###### Conservation status

SR–T, FSC; S1S2, G2G3.

##### Distribution

Lake Waccamaw (Infrequent): *Howell LAWA−102, 118* (NCSC!)

##### Notes

Perennial herbs. Eulittoral zone (NLS–LW). Jul–Oct. Fig. 180

#### Ludwigia sphaerocarpa

Elliott

Ludwigia sphaerocarpa Taxon concept: [= RAB, GW, Weakley]

##### Ecological interactions

###### Conservation status

E; S1, G5.

##### Distribution

Lake Waccamaw (Occasional): *Howell LAWA−33, 143* (NCSC!)

##### Notes

Perennial herbs. Eulittoral zone (NLS–LW). Jun–Sep. Fig. 181

### Plantaginaceae

#### Bacopa caroliniana

(Walter) B.L. Rob.

Bacopa caroliniana Basionym: *Obolaria caroliniana* Walter
Bacopa caroliniana Taxon concept: [= RAB, GW, Weakley]

##### Ecological interactions

###### Conservation status

T; S1, G4G5.

##### Distribution

Lake Waccamaw (Rare): *Howell LAWA−66, 120* (NCSC!); *LeBlond 3984* (NCU!)

##### Notes

Perennial herbs. Eulittoral zone; calm, quiet waters (NLSS–LW). May–Sep. Fig. 182

#### Nuttallanthus canadensis

(L.) D.A. Sutton

Nuttallanthus canadensis Basionym: *Antirrhhinum canadense* L.
Nuttallanthus canadensis Taxon concept: [< *Linaria canadensis* (L.) Dum. Cours.; = Weakley]

##### Distribution

Bay Tree Lake (Rare): *Howell BATR–28* (NCSC!)

##### Notes

Annual or biennial herbs. Juncture of eulittoral and supralittoral zones. Mar−Jul. Fig. 183

### Platanaceae

#### Platanus occidentalis

L.

Platanus occidentalis Taxon concept: [= RAB, GW, FNA, Weakley]

##### Distribution

Lake Waccamaw (Infrequent): *Howell LAWA−68* (NCSC!)

##### Notes

Trees. Eulittoral zone; on saturated soils of sandbars and shorelines (NLSS−LW). Apr−May; Sep−Nov. Fig. 184

### Polygalaceae

#### Polygala lutea

L.

Polygala lutea Taxon concept: [= RAB, GW, Weakley]

##### Distribution

Horseshoe Lake (Rare): *Howell HOLA−50* (NCSC!)

Lake Waccamaw (Rare): *Howell LAWA−127* (NCSC!)

##### Notes

Biennial herbs. Eulittoral zone; moist sandy soils at or below the maximum annual high water mark (NLSS−LW, CPSI−CG). Apr−Oct. Fig. 185

### Polygonaceae

#### Rumex hastatulus

Baldwin

Rumex hastatulus Taxon concept: [= RAB, GW, FNA, Weakley]

##### Distribution

Bay Tree Lake (Infrequent): *Howell BATR−14, 23, 30* (NCSC!)

##### Notes

Annual or short-lived perennial herbs. Eulittoral zone; moist sandy to peaty shores. Mar−May; May−Jul. Fig. 186

### Ranunculaceae

#### Clematis crispa

L.

Clematis crispa Taxon concept: [= RAB, GW, FNA. Weakley]

##### Distribution

Lake Waccamaw: *Matthews* *s.n.* (NCU!)

##### Notes

Perennial, sometimes lianescent, vines. Eulittoral zone (NLSS–LW). Apr–Aug. Fig. 187

### Rhamnaceae

#### Berchemia scandens

(Hill) K. Koch

Berchemia scandens Basionym: *Rhamnus scandens* Hill
Berchemia scandens Taxon concept: [= RAB, GW, FNA, Weakley]

##### Distribution

Lake Waccamaw (Occasional): *Howell LAWA−38* (NCSC!)

##### Notes

Lianas. Eulittoral zone (NLSS–LW). Apr–May; Aug–Oct. Fig. 188

### Rosaceae

#### Amelanchier canadensis

(L.) Medik.

Amelanchier canadensis Basionym: *Mespilus canadensis* L.
Amelanchier canadensis Taxon concept: [=RAB, GW, FNA, Weakley]

##### Distribution

Bay Tree Lake: *Radford 1354* (NCU!)

##### Notes

**Figure 189.**
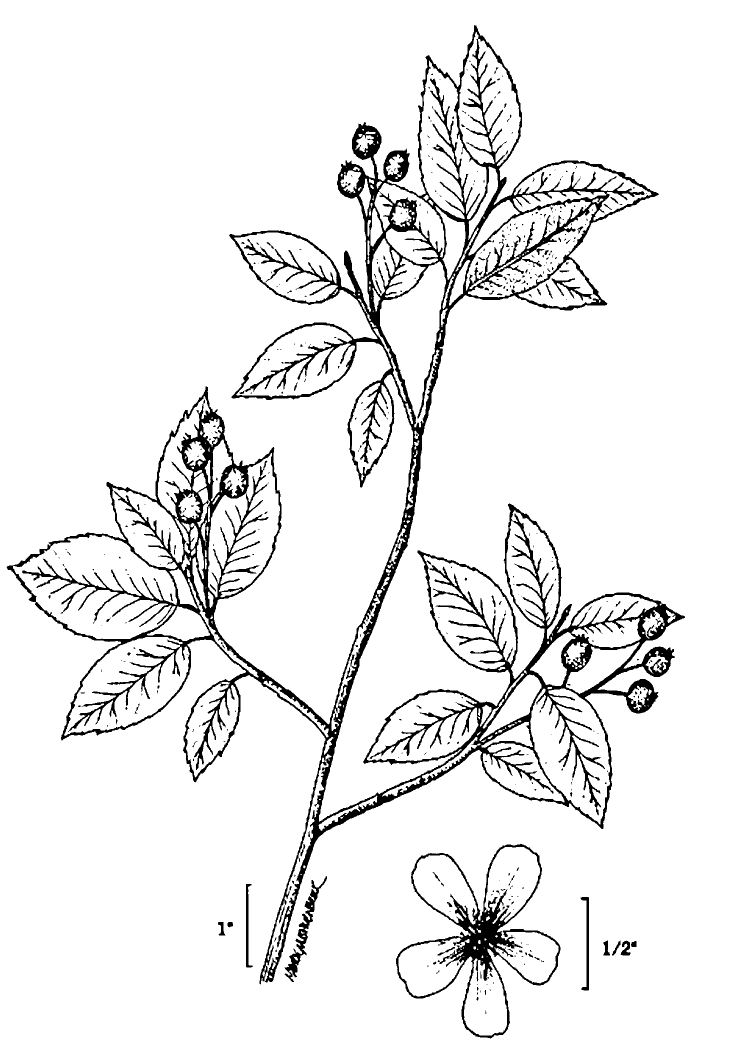
*Amelanchier canadensis* (illustration from United States Department of Agriculture, Natural Resource Conservation Service PLANTS Database / United States Department of Agriculture, Natural Resource Conservation Service 2015)

Shrubs or small trees. Juncture of eulittoral and supralittoral zones. Mar−Apr; May−Jun. Fig. 189

#### Amelanchier obovalis

(Michx.) Ashe

Amelanchier obovalis Basionym: Mespilus canadensis L. var. obovalis Michx.
Amelanchier obovalis Taxon concept: [= RAB, GW, FNA, Weakley]

##### Distribution

Lake Waccamaw (Rare): *Howell LAWA−48* (NCSC!)

##### Notes

Shrubs. Eulittoral zone (NLSS−LW). Mar−Apr; May−Jun. The only specimen encountered by the current author was found in a shallow concave depression in the middle of two boles of Taxodium ascendens arising from the same stump. The shrub established itself in the small amount of soil that had accumulated in the depression through the years. Fig. 190

#### Aronia arbutifolia

(L.) Pers.

Aronia arbutifolia Basionym: *Mespilus arbutifolia* L.
Aronia arbutifolia Taxon concept: [= Sorbus arbutifolia (L.) Hyenh. var. arbutifolia; = RAB; = GW, FNA, Weakley]

##### Distribution

Salters Lake: *Buell* *s.n.* (NCSC!)

Singletary Lake (Rare): *Howell SILA−24* (NCSC!)

##### Notes

Shrubs. Juncture of eulittoral and supralittoral zones (NLSS−C). Mar−May; Sep−Nov. Fig. 191

#### Rosa palustris

Marshall

Rosa palustris Taxon concept: [= RAB, GW, FNA, Weakley]

##### Distribution

Lake Waccamaw (Infrequent): *LAWA−74, 112* (NCSC!)

Singletary Lake: *Fox*, *Wells*, *Sharp*, *Whitford*, *Fairchild s*. *n.* (NCSC!)

##### Notes

Shrubs. Eulittoral zone; sandy to peaty soils at or just below the maximum annual high water mark (NLSS−C, NLSS−LW). May−Jul; Sep−Oct. *Rosa palustris* can be distinguished from *R. multiflora*, a common exotic in the North Carolina Coastal Plain, by its large (adnate portion 13–30 mm long), entire, stipules. Those of *R. multiflora* are up to 21 mm long (adnate portion 3–15 mm long) and pectinate- fringed. Fig. 192

#### Rubus pensilvanicus

Poir.

Rubus pensilvanicus Taxon concept: [> *R. argutus* Link – RAB, GW; > *R. betulifolius* Small – RAB; = Weakley]

##### Distribution

Lake Waccamaw (Infrequent): *Howell LAWA−73, 97* (NCSC!)

##### Notes

Shrubs. Eulittoral zone; sandy to peaty soils at or just below the maximum annual high water mark (NLSS−LW). Apr−May; Late May−Jul. Fig. 193

### Rubiaceae

#### Cephalanthus occidentalis

L.

Cephalanthus occidentalis Taxon concept: [= RAB; < C. occidentalis L. var. occidentalis – GW; = Weakley]

##### Distribution

Lake Waccamaw (Occasional): *Howell LAWA−104, 119, 165* (NCSC!)

##### Notes

Shrubs. Eulittoral zone (NLSS–LW). Jun–Jul. Fig. 194

#### Diodia virginiana

L.

Diodia virginiana Taxon concept: [= RAB, GW, Weakley]

##### Distribution

Bay Tree Lake (Rare): *Howell BATR−22* (NCSC!)

##### Notes

Annual or perennial herbs. Eulittoral zone; sandy soils at or just below the maximum annual high water mark. Jun–Dec. Fig. 195

#### Galium obtusumvar.obtusum

Galium obtusumvar.obtusum Taxon concept: [= RAB; < *G. obtusum* – GW; = Weakley]

##### Distribution

Lake Waccamaw (Rare): *Howell LAWA−138* (NCSC!)

##### Notes

Perennial herbs. Eulittoral zone; at or just below maximum annual high water mark (NLSS–LW). Apr–May. Fig. 196

### Salicaceae

#### Populus heterophylla

L.

Populus heterophylla Taxon concept: [= RAB, GW, FNA, Weakley]

##### Distribution

Lake Waccamaw (Infrequent): *Howell LAWA−91* (NCSC!)

##### Notes

Trees. Eulittoral zone; saturated soils at or just below the maximum annual high water mark (NLSS−LW). Mar−Apr. Fig. 197

#### Salix caroliniana

Michx.

Salix caroliniana Taxon concept: [= RAB, GW, FNA, Weakley]

##### Distribution

Lake Waccamaw: *Harper 970* (NCU!); *Matthews* *s.n.* (DUKE!, NCU!)

##### Notes

Trees. Eulittoral zone; sandbars and sandy shorelines (NLSS−LW). Mar−Apr. This taxon was not encountered by the first author, but voucher specimens confirm its historical presence. Fig. 198

#### Salix nigra

Marshall

Salix nigra Taxon concept: [= RAB, GW, FNA, Weakley]

##### Distribution

Lake Waccamaw (Infrequent): *Howell LAWA−72* (NCSC!)

##### Notes

Trees. Eulittoral zone; sandbars and sandy shorelines (NLSS−LW). Mar−Apr. Fig. 199

### Santalaceae

#### Phoradendron leucarpumvar.leucarpum

Phoradendron leucarpumvar.leucarpum Basionym: *Viscum leucarpum* Raf.
Phoradendron leucarpumvar.leucarpum Taxon concept: [< *P. serotinum* (Raf.) M.C. Johnst. – RAB; = Weakley]

##### Distribution

Jones Lake (Infrequent): *Howell JOLA−12* (NCSC!)

Salters Lake (Infrequent): *Howell SALA−7, 19* (NCSC!)

##### Notes

Epiphytic shrubs. Eulittoral zone; typically on limbs of *Acer* or *Nyssa* (NLSS–C). Oct–Nov; Nov–Jan. Fig. 200

### Sapindaceae

#### Acer rubrumvar.rubrum

Acer rubrumvar.rubrum Taxon concept: [< *A. rubrum* L. – RAB, GW; = Weakley]

##### Distribution

Bakers Lake (Rare): *Howell BALA−4* (NCSC!)

##### Notes

Trees. Eulittoral zone; typically in saturated organic to sandy soils at or just below the maximum annual high water mark (NLSS−C). Jan−Mar; Apr−July. Fig. 201

#### Acer rubrumvar.trilobum

Torr. & A. Gray ex K. Koch

Acer rubrumvar.trilobum Taxon concept: [< *A. rubrum* L. – RAB, GW; = Weakley]

##### Distribution

Bay Tree Lake (Occasional): *Howell BATR−1* (NCSC!)

Horseshoe Lake (Occasional): *Howell HOLA−5, 20* (NCSC!)

Jones Lake (Occasional): *Howell JOLA−9, 21* (NCSC!)

Lake Waccamaw (Occasional): *Howell LAWA−22, 43, 44* (NCSC!)

Little Singletary Lake (Occasional): *Howell LISI−11, 21* (NCSC!)

Salters Lake (Occasional): *Beckman & Linnenburger 27* (DUKE!); *Howell SALA−2, 18* (NCSC!)

Singletary Lake (Occasional): *Howell SILA−8* (NCSC!)

##### Notes

Trees. Eulittoral zone; typically in saturated organic to sandy soils at or just below the maximum annual high water mark (NLSS−C, NLSS−LW, NLSM−T, CPSI−CG). Jan−Mar; Apr−Jun. Fig. 202

#### Aesculus paviavar.pavia

Aesculus paviavar.pavia Taxon concept: [< *A. pavia* L. − RAB; = Weakley]

##### Distribution

Lake Waccamaw: *Harbison 6084* (NCU!); *Harper s*. *n.*, *955, 965* (NCU!); *Matthews* *s.n.* (NCU!); *Oosting 3498* (DUKE!); *Reed & Stites 275* (NCU!)

##### Notes

**Figure 203.**
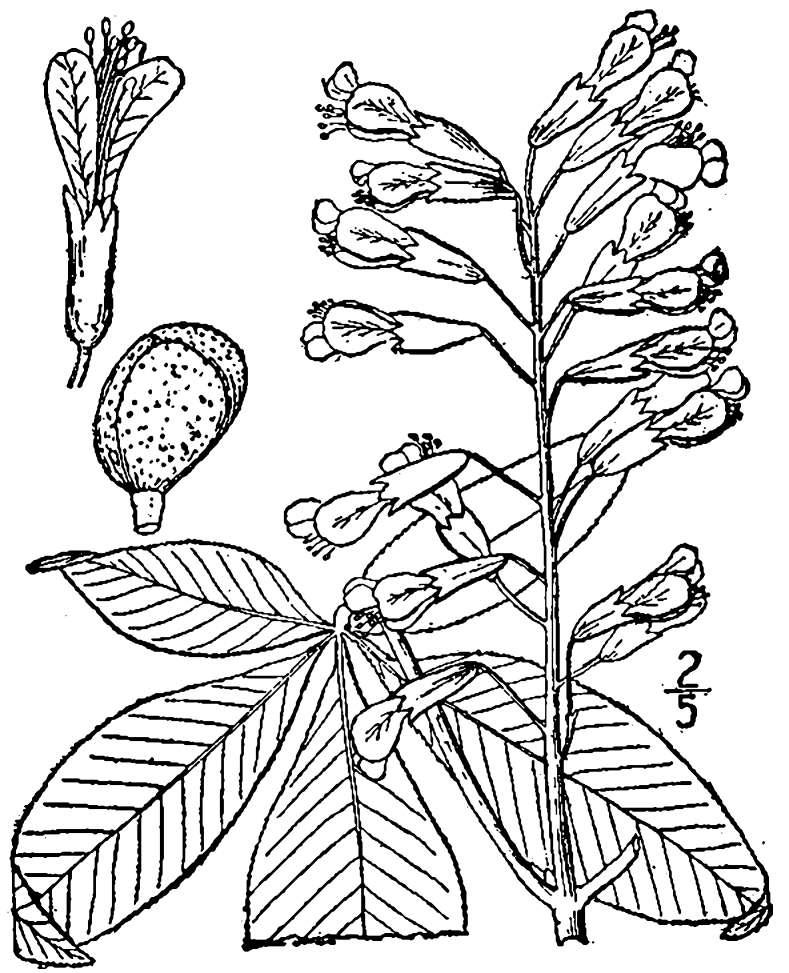
*Aesculus pavia* (illustration from Britton and Brown 1913)

Shrubs or trees. Eulittoral zone (NLSS−LW). Apr−early May; Jul−Aug. Fig. 203

### Sarraceniaceae

#### Sarracenia flava

L.

Sarracenia flava Taxon concept: [= RAB, GW, FNA, Weakley]

##### Distribution

Horseshoe Lake (Abundant): *Buell & Whitford s.n.* (NCSC!); *Howell HOLA−16, 41* (NCSC!)

##### Notes

Perennial herbs. floating bogs (CPSI-CG, FB). Mar–Apr; May–Jun. Fig. 204

### Theaceae

#### Gordonia lasianthus

(L.) J. Ellis

Gordonia lasianthus Basionym: *Hypericum lasianthus* L.
Gordonia lasianthus Taxon concept: [= RAB, GW, FNA, Weakley]

##### Distribution

Bakers Lake (Infrequent): *Howell BALA−16* (NCSC!)

Horseshoe Lake: *Buell 2262* (NCSC!)

Jones Lake (Occasional): *Howell JOLA−2* (NCSC!)

Little Singletary Lake (Infrequent): *Howell LISI−30, 48* (NCSC!)

Singletary Lake (Infrequent): *Howell SILA−30* (NCSC!)

##### Notes

Trees. Juncture of eulittoral and supralittoral zones (NLSS−C). Jul−Sep; Sep−Oct. Fig. 205

### Ulmaceae

#### Ulmus americanavar.americana

Ulmus americanavar.americana Taxon concept: [< *U. americana* L. – RAB, GW, FNA; = Weakley]

##### Distribution

Lake Waccamaw (Rare): *Bell 12839* (NCU!); *Godfrey 6318* (NCSC!); *Howell LAWA−95* (NCSC!)

##### Notes

Trees. Juncture of the eulittoral and supralittoral zones (NLSS−LW). Feb−Mar; Mar−Apr. Fig. 206

### Vitaceae

#### Muscadinia rotundifoliavar.rotundifolia

Muscadinia rotundifoliavar.rotundifolia Basionym: *Vitis rotundifolia* Michx.
Muscadinia rotundifoliavar.rotundifolia Taxon concept: [< *Vitis rotundifolia* Michx. – RAB, GW; = Weakley]

##### Distribution

Bay Tree Lake (Occasional): *Howell BATR−46* (NCSC!)

Lake Waccamaw (Occasional): *Howell LAWA−64, 137* (NCSC!)

Salters Lake (Infrequent): *Howell SALA−21* (NCSC!)

##### Notes

Lianas. Upper eulittoral zone; typically at the high water mark forming dense tangles along the waters edge (NLSS–C, NLSS–LW, NLSM–T). Late Apr–May; late Jul–Sep. Fig. 207

#### Parthenocissus quinquefolia

(L.) Planch.

Parthenocissus quinquefolia Basionym: *Hedera quinquefolia* L.
Parthenocissus quinquefolia Taxon concept: [= RAB, Weakley]

##### Distribution

Lake Waccamaw (Infrequent): *Howell LAWA−94* (NCSC!)

##### Notes

Lianas. Eulittoral zone; growing on fallen trees, shrubs, and erect trees at or just below the maximum annual high water mark (NLSS−LW). May−Jul; Jul−Aug. Fig. 208

## Identification Keys

### Keys to the major vascular plant groups

**Table:** 

1	Plant reproducing by spores	Pteridophytes
–	Plant reproducing by seeds	2
2	Seeds borne in woody cones; leaves needle-like or scale-like, < 3 mm wide	Gymnosperms
–	Seeds borne in fruits; leaves various	3
3	Plant exhibiting ≥ 2 of the following characters: Cotyledon 1; stem vascular bundles scattered; leaves parallel veined; floral parts in 3s	Monocotyledons
–	Plant exhibiting ≥ 2 of the following characters: Cotyledons 2; stem vascular bundles in a ring; leaves without parallel venation; floral parts in 4s and 5s	Basal Angiosperms, Magnoliids, and Eudicotyledons

### PTERIDOPHYTES

**Table:** 

1	Leaves simple, scale-like, < 2 cm long, each leaf with 1, unbranched vein; sporangia borne in strobili at the tips of shoots	Lycopodiaceae [*Lycopodiella appressa*] Fig. 21
–	Leaves pinnatifid to 2-pinnate, “ferny”, > 2 cm long, each leaf bearing numerous pinately-branched veins; sporangia borne in sori on the undersides of modified or unmodified pinnae	2
2	Plant epiphytic, growing on large limbs and tree trunks along shorelines; leaves (not including the petiole) 3−25 × 2.5−5 cm, evergreen, undersides with peltate, gray scales	Polypodiaceae [Pleopeltis polypodioides ssp. michauxiana]Fig. 24
–	Plant not epiphytic, growing in inundated, saturated, or moist soils of shorelines; leaves (not including the petiole) > 25 cm × 5 cm, deciduous or evergreen, undersides lacking peltate, gray scales	3
3	Stipules present, wing-like; leaves 2-pinnate or more divided, pinnae divided to their midribs; sori and indusia lacking	Osmundaceae [*Osmunda spectabilis*] Fig. 23
–	Stipules absent; leaves 1-pinnate-pinatifid or less divided; sori and indusia present	4
4	Leaves 1-pinnatifid, the rachis winged by leaf tissue throughout most or allof its length	5
–	Leaves 1-pinnate-pinnatifid, the pinnae fully divided from one another (the rachis not winged by leaf tissue throughout most or all of its length)	6
5	Fertile leaf woody, with bead-like segments; margins of sterile leaves entire, wavy, the lowermost pinnae sometimes becoming slightly lobed; pinnae with obtuse apices	Onocleaceae [*Onoclea sensibilis*] Fig. 22
–	Fertile leaf herbaceous, not woody or with bead-like segments; margins of sterile pinnae finely serrulate; pinnae mostly with acute apices	Blechnaceae [*Lorinseria areolata*] Fig. 19
6	Rhizomes long-creeping; leaves deciduous, monomorphic, 28–60 cm long, scattered along the rhizome, forming clonal patches; petiole dark purple to black proximally; sori elongate, borne end to end along both sides of main veins, pinnae lobes of sterile leaves with reticulate, chain-like venation on either side of the central vein	Blechnaceae [*Anchistea virginica*] Fig. 18
–	Rhizomes short-creeping; leaves evergreen, somewhat dimorphic (fertile pinnae in distal half of leaves), 35–120 cm long, clustered on the rhizome, not forming clonal patches; petiole not purple to black proximally; sori circular, not borne end to end along the main veins, located midway between main vein and pinnae lobe margins; pinnae lobes of sterile portions of leaves lacking a chain-like venation pattern on either side of the central vein	Dryopteridaceae [*Dryopteris ludoviciana*] Fig. 20

Key adapted from Radford et al. (1968), Smith (1993a), and Weakley (2012).

Note: Successful keying of ferns is greatly facilitated by a basic understanding of fern morphology. Thornhill et al. (2014) provided a useful summary of important morphological terms: “Pinnate indicates lobing of leaves, leaflets, or pinnules entirely to the rachis or midrib. Pinnatifid indicates lobing of leaves, leaflets, or pinnules to near the midrib (i.e., not all the way to the rachis or midrib, as in the leaflets of *Anchistea virginica)*. Pinnate- pinnatifid refers to a leaf blade that is once-pinnate and whose segments (pinnae) are themselves pinnatifid. Sori are the spore-producing structures found on many species of ferns; these may be either exposed or covered by the margin of the leaves (a false indusium) or a separate structure altogether (a true indusium). Leaf-like structures that bear sporangia are called sporophylls; these may be similar to the sterile leaves or be highly modified (e.g., the compact, cone-like structures, or strobili of the Lycopodiaceae)”.

### GYMNOSPERMS

**Table:** 

1	Leaves scale-like or needle-like, < 1.5 cm long, not in fascicles; seed cone scales valvate or imbricate, if imbricate then leaves opposite and scale-like; seeds 1−3 per scale	Cupressaceae
–	Leaves needle-like, (10−) 12−45 cm long, in fascicles of 2−3 leaves; seed cone scales imbricate; seeds 2 per scale	Pinaceae [*Pinus*]

Key adapted from Eckenwalder and Thieret (1993).

### Cupressaceae

**Table:** 

1	Leaves scale-like, 1−3 mm long, opposite or whorled, evergreen; mature seed cones woody, 4−9 mm broad, scales imbricate; seeds 1–2 (−3) per scale	*Chamaecyparis thyoides* Fig. 25
–	Leaves linear, 3−17 mm long, alternate, deciduous; mature seed cones woody, 1.3–3.6 cm broad, scales valvate; seeds (1−) 2 per scale	* Taxodium *

Key adapted from Watson and Eckenwalder (1993) and Weakley (2012).

### *Taxodium* Rich.

**Table:** 

1	Leaves mostly vertically ascending, appressed and overlapping, spirally arranged; branchlets ascending from twigs, secundly erect; bark 1–2.5 cm thick, furrowed, dark- brown, not exfoliating; larger knees short, rarely > 4 dm tall, with thick, compact bark on top; trees of isolated depressions, natural lakes, wet savannas, pocosins, other wet peaty habitats, and, less commonly, blackwater swamps	*Taxodium ascendens* Fig. 26
–	Leaves pendent to horizontally spreading to laterally divergent, spirally arranged but generally appearing distichous (“featherlike”); branchlets not ascending from twigs; bark < 1 cm thick, exfoliating in shreddy, orange-brown strips; larger knees often tall, frequently > 4 dm tall, with thin, shreddy bark on top; trees of blackwater swamps, brownwater swamps, natural lakes, and millponds; usually in riverine situations	*Taxodium distichum* Fig. 27

Key adapted from Watson (1993), Weakley (2012), and Thornhill et al. (2014).

Note: “In the following key, leaf and branchlet characters of *T. ascendens* refer to mature trees; foliage of juvenile trees often mimics that of *T. distichum*. Leaf and branchlet characters of *T. distichum* refer to both mature and juvenile trees; however, in the crowns of mature *T. distichum*, leaf and branchlet characters sometimes mimic those of *T. ascendens*. For these reasons, accurate identification of the two species often requires observation of other, non-foliage features, including the stature of the “knees”, the thickness and texture of the bark, and the habitat in which the trees grow” (Thornhill et al. 2014).

### Pinaceae

**Table:** 

1	Open seed cones about as broad as long, “top-shaped”, 3–6 cm long, serotinous; trunks typically producing epicormic branches, especially in response to fire	*Pinus serotina* Fig. 28
–	Open seed cones distinctly longer than broad, not top-shaped, 6–18(−20) cm long, not serotinous; trunk not producing epicormic branches	*Pinus taeda* Fig. 29

Key adapted from Radford et al. (1968), Kral (1993), and Weakley (2012).

### MONOCOTYLEDONS

**Table:** 

1	Plant an epiphyte, growing on the trunks and limbs of trees in the littoral zone	2
–	Plant not epiphytic, rooted in soil or freely floating	3
2	Plant green, erect, not scurfy; leaves lanceolate; roots present, fibrous; flowers in racemes, petals dimorphic (two similar in size, the third differentiated into a broad lip)	Orchidaceae [*Epidendrum magnoliae*]
–	Plants gray, pendent (often in masses), scurfy; leaves filiform; roots absent; flowers solitary, petals monomorphic	Bromeliaceae [*Tillandsia usneoides* Fig. 34]
3	Plant diminutive ≤ 1.5 mm long in any dimension, floating or submersed in water, sometimes left stranded on mud or debris by receding water levels, plants thallus-like, not differentiated into stems and leaves, rootless or with few simple roots	Araceae [*Wolffia*]
–	Plant not diminutive or thallus-like, > 2 mm in any dimension, differentiated into stems and leaves, rooted in soil or floating on water surface	4
4	Stems woody	5
–	Stems herbaceous	6
5	Leafy stem erect, smooth, lacking prickles; internodes hollow	Poaceae [*Arundinaria tecta* Fig. 80]
–	Leafy stems climbing by stipular tendrils, armed with prickles; internodes solid	Smilacaceae [*Smilax*]
6	Flowers borne in a single compact head terminating an elongate scape	7
–	Flowers not borne in single compact heads atop elongated scapes	8
7	Flowering head involucrate, white to gray, hemispheric, “button-like”, < 1 cm tall; flowers 2−3-merous, unisexual, 1.5−4 mm long, pale to grayish, not subtended by a scale- like bract, sepals and petals partially coated with club-shaped hairs; anthers black, 2-locular	Eriocaulaceae
–	Flowering head not involucrate, brown, globose to cylindrical, “cone-like”, 0.5−3.5 cm tall; flowers 3-merous, bi-sexual, individual petals 3−6 mm long, yellow, subtended by a conspicuous scale-like bract, sepals and petals not coated with white club-shaped hairs; anthers yellow, 2−4-locular	Xyridaceae
8	Flowers and fruits subtended by imbricate or distichous bracts or scales and for the most part hidden by them, usually only the stamens and styles protruding at anthesis; fruit 1-seeded	9
–	Flowers and fruits not subtended by imbricate or distichous scales, or if so, then the flowers exceeding or equalling the bracts or scales and not hidden; fruit > 1- seeded	10
9	Leaves usually 3-ranked, sheaths typically closed; culms typically triangular in cross- section and solid; fruit an achene	Cyperaceae
–	Leaves usually 2-ranked, sheaths open (split lengthwise on the side opposite the blade); culms terete in cross-section, usually hollow; fruit a caryopsis	Poaceae
10	Plants aquatic, wholly submersed (except for Mayaca fluviatilis, which may be found wholly submersed or growing erect in saturated soils along shorelines); inflorescences submersed, floating, or just above the water surface	11
–	Plants terrestrial, or if growing in shallow water then the inflorescences well above the water surface (except during infrequent flooding events)	13
11	Leaves opposite or whorled (if opposite but appearing whorled, then leaf bases dilated and sheathlike); flowers either lacking perianth parts as in *Najas* or inconspicuous as in *Hydrilla*	Hydrocharitaceae
–	Leaves alternate; perianth parts present or not, if so, then conspicuous	12
12	Plant moss-like, habit ranging from wholly submersed to completely emersed; not heterophyllous; leaves 20−200 (−300) × 0.5−1 mm, very numerous and tightly spaced, spirally arranged, apices sometimes slightly bifid; flowers solitary in the leaf axils, petals rose to maroon to lilac, sometimes white basally, obovate	Mayacaceae [*Mayaca fluviatilis* Fig. 73]
–	Plant not moss-like, habit restricted to wholly submersed; heterophyllous or not, if heterophyllous, then the submersed leaves transluscent and with a soft, fragile, texture, the floating leaves coriaceous; leaves 10−160 × 0.5−85 mm, diffusely spaced, somewhat spirally arranged in *P. pusillus*, no so in *P. pulcher*, apices entire; flowers in axillary spikes, perianth lacking	Potamogetonaceae
13	Inflorescence a spadix surrounded by a yellow spathe; leaves 17−70 × 10−40 cm, peltate, bases cordate to sagittate to hastate, adaxial surface glaucous blue-green, typically with a red or purple spot where the petiole attaches to the blade	Araceae [*Colocasia esculenta*]
–	Plant not with the above combination of characters	14
14	Perianth segments densely pubescent abaxially	15
–	Perianth segments not densely pubescent abaxially	16
15	Leaves linear, equitant; corolla yellow; ovary inferior	Haemodoraceae [*Lachnanthes caroliniana* Fig. 61]
–	Leaves cordate to lanceolate, not equitant; corolla blue to purple; ovary superior	Pontederiaceae [*Pontederia cordata*]
16	Corolla stellate, petals white, female flowers exhibiting an apocarpous gynoecium, each pistil ripening into an achene; phyllodia present	Alismataceae [*Sagittaria*]
–	Corolla not stellate (or, if so, then petals not white), female flowers not exhibiting an apocarpous gynoecium, 1 pistil restricted to each flower, ripening into a capsule; phyllodia absent	17
17	Plant annual, diminutive, 5−20 cm tall, stems filiform; leaves minutely scale-like	Burmanniaceae [*Burmannia capitata* Fig. 35]
–	Plant perennial, not diminutive, > 20 cm tall, stems not filiform; leaves not scale-like (though blades not well-developed in *Juncus effusus*)	18
18	Ovary superior; perianth parts bract-like, dry, scarious, persistent, not petal-like; leaves septate or not, terete, or flat and blade-like	Juncaceae
–	Ovary inferior, perianth parts petal-like, neither bract-like, hard, nor scarious, not persistent; leaves flat and blade-like, never septate	19
19	Flowers radially symmetric; androecium and gynoecium in separate whorls, not borne in a column; pollen free	Hypoxidaceae [*Hypoxis curtisii* Fig. 64]
–	Flowers strongly bilaterally symmetric; androecium and gynoecium borne in a column; pollen in pollinia (pollen sacs)	Orchidaceae

Key adapted from Radford et al. (1968), Godfrey and Wooten (1979), and Weakley (2012).

### Alismataceae

**Table:** 

1	Leaf blades floating, cordate basally	*Sagittaria filiformis* Fig. 30
–	Leaf blades not floating, without basal lobes, linear to lanceolate, or modified asbladeless phyllodia, these with a spongy texture	2
2	Stalks of the pistillate flowering heads stout and reflexed in fruit; stamen filaments glabrous	*Sagittaria filiformis*
–	Stalks of the pistillate flowering heads not overly stout and either spreading or ascending in fruit; stamen filaments roughened with minute scales	3
3	Mature leaves all phyllodial, phyllodia terete or very nearly so	*Sagittaria isoetiformis* Fig. 32
–	Mature leaves with blades and petioles, or phyllodia flattened on the adaxial surface or triangular in cross-section	4
4	Plant with corms or stolons, coarse rhizomes lacking; blades of emersed leaves < 3 (−4) mm wide; flowers ≤ 1.3 cm in diam.	*Sagittaria isoetiformis*
–	Coarse rhizomes present, stolons and corms absent; blades of emersed leaves > 1 cm wide; flowers ≤ 2.3 cm in diameter	5
5	Larger phyllodes ≤ 1 cm wide, apices acute; pistillate pedicels 1−4 cm long; median resin duct of mature achene club-shaped, 2× the width of the posterior duct	*Sagittaria graminea* Fig. 31
–	Larger phyllodes 0.8−2.5 cm wide, apices blunt; pistillate pedicels 2−5 (−6.5) cm long; median resin duct of mature achene linear, about as wide as the posterior duct (or ducts absent)	*Sagittaria weatherbiana*

Key adapted from Durand (2000), Godfrey and Wooten (1979), and Weakley (2012).

### Araceae

**Table:** 

1	Plant terrestrial, stems present, rooted in moist to saturated soils; leaf blades to 70 cm long	*Colocasia esculenta* Fig. 33
–	Plant floating, diminutive, thallus-like, stems absent, dropping water levels sometimes leaving some plants stranded; leaf blades < 0.2 cm long	*Wolffia* spp.

Key adapted from Thompson (2000) and Weakley (2012).

### *Wolffia* Horkel ex Schleid.

**Table:** 

1	Fronds nutshell-like, upper surface flattened, 0.5−1 × as deep as wide, a small portion not flattened and with minute central papillae, fronds brownish punctate above (best seen in dead fronds), cells of fronds inflated in the lower portions and becoming progressively smaller and more compact toward the upper surface	*Wolffia brasiliensis* Fig. 209
–	Fronds globoid to ovoid, upper surface convex, 1−1.5 × as deep as wide, a small portion slightly flattened and roughened with minute central papillae, fronds not brownish punctate above, cells of frond uniformly inflated throughout	*Wolffia columbiana*

Key adapted from Weakley (2012).

Note: The first author did not encountered taxa within this genus in the field; however, the Carolina Vegetation Survey reported “*Wolffia* spp.” from the southwest side of Lake Waccamaw. Although a species-level identification has not been made, a key to the two species most likely to inhabit this location is provided below.

### Cyperaceae

**Table:** 

1	Achenes enclosed in a perigynium; flowers unisexual	* Carex *
–	Achenes not enclosed within a perigynium; flowers unisexual or bisexual	2
2	Leaves absent; spikelets 1 per culm, terminal	* Eleocharis *
–	Leaves present; spikelets ≥ 1 per culm, terminal or axillary	3
3	Spikelet scales distichous (two−ranked)	4
–	Spikelet scales spirally arranged, imbricate	5
4	Leaves not 3−ranked, predominantly basal; inflorescence terminal; perianth bristles lacking	* Cyperus *
–	Leaves prominently 3−ranked, cauline; inflorescence axillary; perianth bristles 6−9	*Dulichium arundinaceum* Fig. 44
5	Base of style hardened, differentiated from achene body, persistent as a tubercle at apex of achene	* Rhynchospora *
–	Base of style not hardened; tubercle absent from apex of achene	6
6	Perianth bristles present	7
–	Perianth bristles absent	8
7	Perianth scales 3, stalked, paddle−shaped; perianth bristles 3	*Fuirena pumila* Fig. 50
–	Perianth scales lacking; perianth bristles typically 4−8	*Scirpus cyperinus* Fig. 59
8	Style entire along margins; culms obtusely angled, 50−80 cm tall; leaf blade margins scaberulous; perennial	*Cladium mariscoides* Fig. 40
–	Style fringed along margins; culms flattened, to 40 cm tall; leaf blade margins glabrous; annual	*Fimbristylis autumnalis* Fig. 49

Key adapted from Radford et al. (1968), Ball et al. (2002a), and Weakley (2012).

### *Carex* L.

**Table:** 

1	Achene lenticular (biconvex); stigmas 2; perigynia wing-margined	2
–	Achene trigonous (three-sided); stigmas 3; perigynia not wing-margined	3
2	Pistillate scales in middle to lower portions of spike 2.8−3.5 (3.8) mm long, apices short-aristate; leaf blades 3−7 per fertile culm, 11−50 × 0.25−0.6 cm; spikes 6−20 × 4−9 mm; perigynia faintly 3−8 nerved on each face, obovate, 4−5.5 × 2.5−3.8 mm; achenes oblong, 1.7−2 × 0.9−1.1 mm, 0.3−0.4 mm thick	*Carex alata* Fig. 36
–	Pistillate scales in middle to lower portions of spike 2.2−3.7 mm long, apices mostly obtuse, not short-aristate; leaf blades 2−4 (−6) per fertile culm, 8−30 × 0.25−0.4 cm; spikes 6−13 (−17) × 3.8−7 mm; perigynia conspicuously 5−many- nerved on each face, obovate, 3−4.6 × 1.6−2.6 (2.8) mm; achenes oblong, 1.3−1.7 × 0.7−1 mm, 0.4−0.5 mm thick	*Carex longii* Fig. 37
3	Style jointed near the base, disarticulating at the joint; culms erect 20−100 (−130) cm; pistillate spikes 1.5−6.5 × 1.3−3 cm; perigynia 11−19 × 3−6 mm; pistillate scales about as long as the body of the perigynia; achenes 3−4 (−4.5) × 1.7−2.6 (−2.8) mm	*Carex lupulina* Fig. 38
–	Style not jointed near the base, hardened and persistent, remaining attached to the mature achene; culms erect 40−90 cm; pistillate spikes 2−4 × 0.7−0.8 cm; perigynia 3.9−7 × 2−3.3 mm; lower pistillate scales about as long as the body of the perigynia, upper about 1⁄2 as long; achenes 2−2.5 ×1.5−2 mm	*Carex striata* Fig. 39

Key adapted from Radford et al. (1968), Godfrey and Wooten (1979), and Ball and Reznicek (2002).

### *Cyperus* L.

**Table:** 

1	Stigmas 2; achenes lenticular	*Cyperus polystachyos* Fig. 43
–	Stigmas 3; achenes trigonous	2
2	Mature spikelets shedding scales and achenes individually, leaving the rachilla intact (for at least a short while); roots and lower sheaths conspicuously reddish-purple; culms trigonous to roundly trigonous, (0.5−) 5−25 (−105) cm × 0.1−0.25 (0.75) cm; spikelets 3−8 (−11) × 1−1.5 mm; pistillate scales deciduous, laterally light brown with red speckles and ribless, medially greennish and 3-ribbed, 1.3−1.5 × 0.8−1.2 mm, apex obtuse, mucronulate; achenes sessile, ovoid, (0.4−) 0.7−1 × 0.4−0.6 mm, surface glabrous	*Cyperus erythrorhizos* Fig. 41
–	Mature spikelets disarticulating into segemets, each comprised of a scale, an achene, and a cartilaginously thickened section of the rachilla; roots and lower sheaths not conspicuously reddish-purple; culms trigonous (4−) 10−50 (−130) × (0.05−) 0.1−0.4 cm; spikelets (5−) 8−15 (−38) × 0.8−1.3 (−1.9) mm; floral scales medially green and 2−5 ribbed, laterally straw-colored to reddish and 1−3 ribbed, (2−) 2.2−2.8 (−3.2) × (1.2−) 1.4−1.6 (−1.8) mm, apex entire or emarginate; achene stipitate, narrowly ellipsoid to oblong, (1−) 1.2−1.5 (−1.9) × 0.5−0.6 (−0.75) mm, surface finely papillose	*Cyperus odoratus* Fig. 42

Key adapted from Radford et al. (1968), Godfrey and Wooten (1979), Tucker et al. (2002), and Weakley (2012).

### *Eleocharis* R.Br.

**Table:** 

1	Culm as broad or broader than width of terminal spike, nodose-septate	*Eleocharis equisetoides* Fig. 46
–	Culm narrower than width of terminal spike, not nodose-septate	2
2	Culms strictly producing fertile spikelets, vegetative proliferations absent; achenes lenticular or biconvex; styles 2−branched	Eleocharis olivacea var. olivacea Fig. 47
–	Culms producing vegetative proliferations or fertile spikelets; achenes trigonous or nearly terete; styles 3−branched	3
3	Upper portion of sheath thin and scarious, lacking a noticeable red-dotted band encircling the apex of sheath (i.e, the apex of the sheath is not differently colored than the lower potions of sheath); sheath tips 1−2 mm long; culms usually more thin and capillary; scales of spikes 2-ranked (distichous); spike usually 2−4 flowered; achenes trigonous, smooth, grayish-olive, 0.6−0.9 × 0.4−0.6 mm, apex constricted proximal to tubercle; tubercle pyramidal, trigonous, 0.2−0.3 (−0.4) × 0.2−0.5 mm	*Eleocharis baldwinii* Fig. 45
–	Upper portion of sheath firm, a noticeable red-dotted band encircling the sheath apex present (i.e., the sheath apex a different color than the lower sheath); sheath tips <1 mm long; culms usually more robust and less capillary than E. baldwinii; scales of spike spirally imbricate, not 2-ranked; spike with > 4 flowers; achenes trigonous, finely reticulate, gray to greenish, 0.6−0.9 × 0.55−0.8 mm, apex constricted proximal to tubercle; tubercle pyramidal, trigonous, 0.2−0.5 × 0.4−0.5 mm	*Eleocharis vivipara* Fig. 48

Key adapted from Smith et al. (2002) and Weakley (2012).

Note: Achene measurements in this key do not include the tubercle. *Eleocharis baldwinii* and *E. vivipara* can be difficult to distinguish in the field when they are both in their vegetative forms. One should pay particular attention to the sheaths encircling the culms; the differences are highlighted in the key below.

### *Rhynchospora* Vahl

**Table:** 

1	Tubercle 3−23 mm long; style simple or bifid only at tip	2
–	Tubercle < 3 mm long; style divided into 2 slender branches	4
2	Longest perianth bristles shorter than the achene body	*Rhynchospora corniculata* Fig. 52
–	Longest perianth bristles equaling or exceeding the achene body	3
3	Plants rhizomatous; primary clusters with 1−6 loosely clustered spikelets; achene (3.5−) 4.0−4.8 mm long	*Rhynchospora inundata* Fig. 55
–	Plants cespitose; primary clusters with 10−50 densely clustered spikelets; achene (4.5−) 5−6 mm long	*Rhynchospora macrostachya* Fig. 57
4	Inflorescence bracts several, bright white basally	*Rhynchospora latifolia* Fig. 56
–	Inflorescence bracts 0−several, not white basally	5
5	Perianth bristles retrorsely barbellate (at least distally)	*Rhynchospora alba* Fig. 51
–	Perianth bristles antrorsely barbellate	6
6	Surface of achene smooth, minutely pitted, or finely striate	7
–	Surface of achene transversely ridged, rugose, or honeycomb-reticulate	8
7	Bristles > 1⁄2 as long or exceeding the achene body; larger basal leaves 1.3−2.5 mm wide, achene elliptic, 1.1−1.3 mm wide, tubercle triangular−attenuate	*Rhynchospora distans* Fig. 53
–	Bristles virtually non-existent to 1⁄2 as long as the achene body (rarely > 1⁄2 as long as the achene body); larger basal leaves 2−4 mm wide; achene suborbicular, 1.2−1.5 mm wide, tubercle triangular	[*Rhynchospora fascicularis*]
8	Achenes biconvex, not flat or concave on one side	*Rhynchospora nitens* Fig. 58
–	Achene faces flat or concave, when one face is concave, the other slightly convex	9
9	Achene < 2× as long as wide, obovate, tubercle triangular, 0.2−0.9 mm long	*Rhynchospora elliottii*
–	Achene at least 2× as long as wide, elliptic−oblong, tubercle subulate, 0.8−1.2 mm long	*Rhynchospora inexpansa* Fig. 54

Key adapted from Kral (2002a) and Weakley (2012).

Note: A voucher (*Wilbur 49814*, DUKE) for *Rhynchospora fascicularis* (Michx.) Vahl was collected from the shoreline of Lake Waccamaw; however, this specimen appears referable to *R. distans* (Michx.) Vahl. Nonetheless, though not otherwise reported from the littoral zone of Carolina bay lakes, *R. fascicularis* has the potential to occur in these sites and is therefore included in the key below. Achene measurements in this key do not include the tubercle (i.e., the tubercle and achene should be measured as two separate entities).

### Eriocaulaceae

**Table:** 

1	Plant 4−21 cm tall (−100 cm when submersed); receptacle/base of flowers glabrous or sparingly hairy; heads overall appearing dark gray to white, 4−10 mm in diam. when in full flower and fruit; seeds light-brown or red-brown, ovoid to broadly ellipsoid, faintly reticulate, not papillate; of sandy to peaty shorelines, bogs, and streams	*Eriocaulon aquaticum* Fig. 60
–	Plant 20−70 cm tall; receptacle/base of flowers copiously hairy; heads overall appearing white, 10−20 mm in diam. when in full flower or fruit; seeds dark lustrous brown, broadly ovoid to round but asymmetric, minutely spiny papillate; of seasonally floooded depression ponds, savannas, flatwoods, ditches	[*Eriocaulon compressum*]

Key adapted from Kral (2000a) and Weakley (2012).

Note: Although the first author has only encountered *E. aquaticum* in the field, *E. compressum* was reported from the NCSU Crop Science Department (Rob Richardson and Justin Nawrocki, pers. comm., April 9, 2015) and is therefore included in the key below.

### Hydrocharitaceae

**Table:** 

1	Leaves noticeably rough to the touch, in whorls of (3−) 4−8, 1.2−4 mm wide, lacking sheaths, margins conspicuously serrulate, each serration tipped with 1-celled sharp teeth, 1- nerved, mid-vein keeled below, keels bearing conical protrusions, each armed with sharp teeth; plants dioecious, flowers unisexual (only female plants found in the southeastern United States)	*Hydrilla verticillata* Fig. 62
–	Leaves not rough, opposite or sometimes crowded and appearing whorled, 0.2−2.1 mm wide, sheaths present, margins minutely serrulate, 1-nerved, midvein lacking an abaxial keel and conical protrusions; plants monoecious, flowers unisexual	Najas guadalupensis var. guadalupensis Fig. 63

Key adapted from Godfrey and Wooten (1979), Haynes (2000b), and Weakley (2012).

### Juncaceae

**Table:** 

1	Inflorescence bract exceeding the inflorescence, inflorescence thus appearing lateral	2
–	Inflorescence bract not exceeding the inflorescence, inflorescence appearing terminal	3
2	Basal sheaths (or at least a few) producing elongate well-developed blades; inflorescence bract channeled on one side; capsules subglobose	*Juncus coriaceus* Fig. 68
–	Basal sheaths not producing elongate blades; inflorescence bract not channeled on one side; capsules more or less oblong, 3-sided	Juncus effusus ssp. solutus Fig. 69
3	Leaf blades not septate	4
–	Leaf blades septate	5
4	Stems erect and with a hardened base, never creeping or forming mats; perianth < 6 mm long; plant not cinfined to aquatic settings, may occur in uplands as well as wetland margins, never submersed	*Juncus biflorus* Fig. 66
–	Stems soft, weak, creeping and rooting at the nodes, often forming homogeneous mats or stands in shallow water or saturated soils above current water level; perianth 6−10 mm long; plant strictly aquatic, submersed and sterile or emersed/stranded and fertile	*Juncus repens* Fig. 71
5	Flowers or fruits borne singly (solitary) on the branches of the inflorescence; inflorescence diffuse, with slender flexuous branches; flowers often aborted; seeds without tail-like appendages	*Juncus pelocarpus* Fig. 70
–	Flowers or fruits borne in heads of 3 or more, heads often spherical; inflorescence not diffuse, branches not slender and flexuous; flowers seldom aborted; seeds with or without tail-like appendages	6
6	Mature seeds with elongate tail-like appendages, body of seeds 1.2−2.2 mm long	*Juncus canadensis* Fig. 67
–	Mature seeds lacking elongate tail-like appendages; body of seeds < 0.7 mm long	7
7	Heads turbinate to hemispherical, 3−15-flowered; capsules 2.8−3.5 (−4) mm long, straw-colored, exerted, abruptly contracting at the summit, apex acute, valves separating (dehiscing) at maturity, equaling or just exceeding the perianth; stamens 3 or 6; seeds ellipsoid, clear amber	*Juncus acuminatus* Fig. 65
–	Heads spherical, 15−60-flowered; capsules 2−3 mm long, straw-colored, exerted, apex gradually tapering to the summit, remaining attached at the tip, valves not separating (dehiscing) at maturity, subulate tips of the capsules exceeding the perianth when fully mature; stamens 3; seeds oblong, dark to clear yellow amber	Juncus scirpoides var. compositus Fig. 72

Key adapted from Godfrey and Wooten (1979), Brooks and Clemants (2000), and Weakley (2012).

### Orchidaceae

**Table:** 

1	Plant an epiphyte, typically found on bases, boles, and large limbs of *Taxodium*, *Nyssa*, *Liquidambar*, and other deciduous hardwoods	*Epidendrum magnoliae*
–	Plant not epiphytic, found in the littoral zone and on floating bogs	2
2	Corolla greenish-colored, lip with a spur, spur deeply divided into 3 linear segments; leaves 3−5, basally disposed	*Habenaria repens*
–	Corolla white, pink, purple or magenta, lip not spurred; leaves basally disposed or cauline	3
3	Flowers arranged in distinct spirals (often appearing 3–4 ranked if spiral is “tight”, white, relatively small, 3–5 mm wide	*Spiranthes laciniata* Fig. 76
–	Flowers not in distinct spirals, pink, magenta, purple, larger, typically ≥ 1 cm wide	4
4	Flowers not resupinate, lip oriented upwards, bearing numerous orange or yellow clavellate trichomes reminiscent of stamens	Calopogon tuberosus var. tuberosus Fig. 74
–	Flowers resupinate, lip oriented downwards, not bearing numerous stamen-like trichomes	*Pogonia ophioglossoides* Fig. 75

Key adapted from Romero-Gonzáles et al. (2002) and Weakley (2012).

### Poaceae

**Table:** 

1	Culm perennial, woody, developing complex branching systems from upper culm nodes; [Bambuseae]	*Arundinaria tecta* Fig. 80
–	Culm annual or facultatively perennial, herbaceous, not developing complex branching systems from upper culm nodes	2
2	Spikelets almost always with 2 florets, lower floret in spikelet always sterile or staminate, frequently absent or reduced to lemma, upper floret bisexual, staminate, or sterile, unawned or awned from the lemma apices; [Andropogoneae and Paniceae]	3
–	Spikelets **either** not with 2 florets ***or*** with two florets and the lower bisexual or upper floret awned from lemma backs or bases [various tribes]	10
3	Spikelets in sessile-pedicellate pairs, not arranged in conspicuous rows on one side of the rachis; glumes stiff, indurate; usually subequal in length, one or usually both exceeding the floret (excluding the lemma awn); lemmas hyaline; paleas hyaline or absent; [Andropogoneae]	4
–	Spikelets solitary, or if paired, then forming 2–4 obvious rows on one side of rachis; glumes membranous, lower usually shorter than upper or absent entirely, upper glumes shorter than or nearly equaling upper floret; lower lemmas membranous, upper lemmas typically stiff and indurate, occasionally membranous; upper paleas of similar texture to upper lemmeas; [Paniceae]	5
4	Plant to 1 m tall; spikelets of the pair unalike, sessile bisexual, pedicellate sterile, vestigial, or absent	* Andropogon *
–	Plant to 3 m tall; spikelets of the pair alike, pedicellate spikelet perfect	*Saccharum giganteum* Fig. 91
5	Base of spikelets with rounded, distended, swellings (gibbous)	*Sacciolepis striata* Fig. 92
–	Spikelets not gibbous	6
6	Plant producing simple culms with terminal “spring” paniculate inflorescences before mid-summer, the culms branching and producing lateral “autumnal” inflorescences from mid to lower culm nodes in the summer and autumn, these often his by the fascicles of smaller “autumnal” leaves; upper florets not disarticulating at maturity	* Dichanthelium *
–	Plant producing terminal panicles in late summer and fall; culms usually not branching from mid to lower culm nodes, or, if so, the branches seldom further branched; upper florets disarticulating or not at maturity	7
7	Plant annual, lacking rhizomes or hard knotty crowns; spikelets verrucose	*Panicum* [in part]
–	Plant a perennial, with rhizomes or hard knotty crowns; spikelets not verrucose	8
8	Plant with hard, knotty crowns, lacking rhizomes; upper lemmas 1.2−1.6 mm long	*Coleataenia* [in part]
–	Plant with rhizomes; upper lemmas 1.6−4 mm long	9
9	Culms slightly compressed below; ligules ≤ 0.5 mm tall; spikelets subsecund, usually obliquely bent above the first glume, pedicels appressed; upper lemma apices lacking papillae, with minute tuft of hair	*Coleataenia* [in part]
–	Culms terete, not slightly compressed below; ligules 2−6 mm tall; spikelets not secund, not obliquely bent above first glume, pedicels spreading; upper lamma apices with simple or compound papillae, glabrous	*Panicum* [in part]
10	Plant seldom seen in flower; spikelets composed of a single floret, florets imperfect; culms ≤ 2 mm wide, slender, flexuous, prostrate; leaves conspicuously clustered at the culm apices, floating (lentic system) or streaming (lotic system) on the water surface, or emergent after receding water levels; glumes absent; [Oryzeae]	*Luziola fluitans* Fig. 87
–	Plants regularly seen in flower; spikelets composed of ≥ 1 floret, florets imperfect or perfect; culms > 2 mm wide, slender, flexuous, or prostrate; leaves not conspicuously clustered at the culm apices, not floating or emergent after receding water levels; glumes present	11
11	Spikelets with (4−) 6−30 florets; [Cynodonteae]	* Eragrostis *
–	Spikelets with ≤ 3 florets; [Poaeae]	12
12	Culm 1.5-8.2 dm; sheaths glabrous; ligules (0.7−) 1.2−4 mm tall; blades 3−10 × 0.1−0.2 cm; panicles (5−) 10−25 (36) × (3) 4−24 cm, diffuse, the whole panicle detaching at the base at maturity, the resulting detached panicle resemblig a “tumbleweed”	*Agrostis hyemalis* Fig. 77
–	Culm (0.9−) 2−13 dm; sheaths glabrous, hairy, or scabridulous; ligules (1−) 1.5−2.5 mm tall; blades 5−14 × (0.1-) 0.2-0.8 cm; panicles (2−) 5−15 (−25) × 0.5−2 cm, compact, spike-like, the panicle not detaching at the base at maturity	*Sphenopholis obtusata* Fig. 93

Key adapted from Radford et al. (1968), Godfrey and Wooten (1979), Barkworth (2003a), Barkworth (2003b), Barkworth (2007), and Weakley (2012).

### *Andropogon* L.

**Table:** 

1	Leaves strongly glaucous (appearing powdery-white and leaving white residue on fingers when rubbed), glabrous; ligules (0.9−) 1.5 (−2) mm tall	*Andropogon glaucopsis* Fig. 78
–	Leaves green, not glaucous (never powdery-white), pubescent (at least on the margin near the collar; ligules 0.2−1 mm tall	Andropogon virginicus var. virginicus Fig. 79

Key adapted from Weakley (2012).

### *Coleataenia* Griseb.

**Table:** 

1	Cauline leaf blades 2−8 mm wide; glumes and sterile lemmas keeled along midvein; apices of fertile lemmas with a minute tuft of hairs	Coleataenia longifolia var. longifolia Fig. 81
–	Cauline leaf blades 1−4 mm wide; glumes and sterile lemmas not keeled along midvein; apices of fertile lemmas lacking a minute tuft of stiff hairs	*Coleataenia tenera*

Key adapted from Weakley (2012).

### *Dichanthelium* (Hitchc. et Chase) Gould

**Table:** 

1	Spikelets 0.8−2.0 mm long	2
–	Spikelets 2.1−3.2 mm long	5
2	Internodes glabrous	3
–	Internodes crisp-puberulent	4
3	Plants with hard knotty crowns; culms to 100 cm; nodes without a distinct constricted yellow ring; vernal cauline leaves 15−20× as long as wide (5−12 cm long); ligules < 1 mm tall; spikelets 1.7−2.3 mm, glabrous	Dichanthelium dichotomum var. roanokense
–	Plants cespitose; culms 30−75 cm; nodes with a distinct constricted yellow ring; vernal cauline leaves < 15× as long as wide (5−10 cm long); ligules 0.2−0.5 mm tall; ligules 0.2−0.5 mm; spikelets 0.9−1.2 mm, puberulent to subglabrous	*Dichanthelium erectifolium* Fig. 83
4	Spikelets 1.5−1.8 mm; first glume 0.5−0.8 mm; lower culm blades 2−5 mm wide	*Dichanthelium portoricense* Fig. 85
–	Spikelets (1.8−) 1.9−2.2 (−2.3) mm; first glume 0.8−1.2 mm; lower culm blades 4−8 mm wide	*Dichanthelium* species 3 (=*lancearium*)
5	Larger culm blades usually 6−15 mm wide; spikelets 2−3 mm, pubescent; internodes glabrous	6
–	Larger culm blades usually 3.5−8 mm wide; spikelets 1.7−2.3 mm, pubescent or glabrous; internodes crisp-puberulent or glabrous	7
6	Larger culm blades 6−12 mm wide; lower culm nodes not bearded; spikelets 2−3 mm long; first glumes 0.5–1 mm long	*Dichanthelium boreale* Fig. 82
–	Larger culm blades 13−25 mm wide; lower culm nodes bearded (often retrorsely); spikelets (2−) 2.2−2.8 mm long; first glumes 0.5–1.3 mm long	*Dichanthelium mattamuskeetense* Fig. 84
7	Spikelets 1.7−2.3 mm long, glabrous; first glume 0.6−1.1 mm; largest vernal blades 15−20× as long as wide; internodes glabrous	Dichanthelium dichotomum var. roanokense
–	Spikelets (1.8) 1.9−2.2 (2.3) mm long, pubescent; first glume 0.8−1.2 mm; largest vernal blades < 15× as long as wide; internodes crisp-puberulent	*Dichanthelium* species 3 (=*lancearium*)

Key adapted from Freckmann and Lelong (2003b) and Weakley (2012).

### *Eragrostis* Wolf

**Table:** 

1	Lateral spikelets with widely spreading pedicels; lower pedicles longer than spikelets; disarticulation of the lemmas only, paleas are persistent	*Eragrostis elliottii* Fig. 86
–	Lateral spikelets with appressed pedicels, lower pedicels shorter than spikelets; disarticulation usually of the whole floret	*Eragrostis refracta*

Key adapted from Godfrey and Wooten (1979) and Weakley (2012).

### *Panicum* L.

**Table:** 

1	Glumes and lower lemmas verrucose; ligules 0.2−0.5 mm tall	*Panicum verrucosum* Fig. 89
–	Glumes and lower lemmas smooth, not verrucose; ligules 0.5−6 mm tall	2
2	Panicle < 1 cm wide at maturity; upper glume and lower lemma 3−5 veined; ligule <1 mm tall	*Panicum hemitomon* Fig. 88
–	Panicle 4−20 cm wide at maturity; upper glume and lower lemma 7−11 veined; ligule 2−6 mm tall	*Panicum virgatum* Fig. 90

Key adapted from Freckmann and Lelong (2003a) and Weakley (2012).

### Pontederiaceae

**Table:** 

1	Outside of floral tube villous when young, essentially glabrous to sparsely glandular at maturity; leaves ovate to triangular−lanceolate, 2.2−21 cm wide, base usually cordate or truncate	Pontederia cordata var. cordata Fig. 94
–	Outside of floral tube persistently pubescent with glandular hairs; leaves lanceolate, 0.4−8.3 cm wide, base usually cuneate to truncate	Pontederia cordata var. lancifolia

Key adapted from Godfrey and Wooten (1979) and Weakley (2012).

### Potamogetonaceae

**Table:** 

1	Plant with floating and submersed leaves; submersed to 30 mm wide, linear to narrowly- lanceolate to lanceolate, mid to upper stem leaves translucent, with 4−8 rows of lacunae aon either side of midvein, floating to 85 mm wide, coriaceous, ovate to oblong-elliptic, bases rounded or slightly cordate	*Potamogeton pulcher* Fig. 95
–	Plant with submersed leaves only, leaves linear, thread-like, or ribbonlike, to 3 mm wide, obvious lacunae absent on either side of midvein	*Potamogeton pusillus*

Key adapted from Weakley (2012).

Note: The first author has not encountered *Potamogeton* in the field, but *Potamogeton pusillus* L. and *Potamogeton pulcher* were reported from Lake Waccamaw by the NCSU Crop Science Department (Rob Richardson and Justin Nawrocki, pers. comm., April 9, 2015) and Richard LeBlond with the North Carolia Natural Heritage Program (see specimen label of *LeBlond 3382*, NCU!). A key to these reported taxa is provided below.

### Smilacaceae

**Table:** 

1	Leaves evergreen, blades more or less oblong to linear or narrowly lanceolate, thick, coriaceous, midvein (as seen from the abaxial leaf surface) much more pronounced than the secondary veins, which are not noticeably evident (except perhaps at base of leaf blade)	*Smilax laurifolia* Fig. 97
–	Leaves deciduous or evergreen, blades ovate to suborbicular, membraneous, midvein (as seen from the abaxial leaf surface) little if any more pronounced than the secondary veins, which are noticeably evident	2
2	Abaxial surface of mature leaves strongly glaucous	*Smilax glauca* Fig. 96
–	Abaxial surface of mature leaves not glaucous, usually paler green than the adaxial surface	3
3	Mature berries blue-black, seeds (1−) 2−3 per berry; perianth green; leaves semi- evergreen to evergreen, margins of mature leaf blades usually not revolute, typically with small, flat, tooth-like projections near the base; of various upland and wetland habitats, typically not restricted to sites that are inundated for much of the year	*Smilax rotundifolia* Fig. 98
–	Mature berries bright red, seeds 2−4 per berry; perianth brownish-yellow; leaves deciduous; margins of mature leaf blades usually revolute, lacking small, flat, tooth-like projections near the base; restricted to sites with long hydroperiods	*Smilax walteri* Fig. 99

Key adapted from Godfrey and Wooten (1979), Holmes (2002), and Weakley (2012).

### Xyridaceae

**Table:** 

1	Keel of lateral sepals long–fimbriate towards apex, fimbriate tip conspicuously protruding beyond the subtending bract (sometimes eroded and less evident in older spikes)	*Xyris fimbriata* Fig. 100
–	Keel of lateral sepals lacerate, not ciliate or long-fimbriate	2
2	Lateral sepals longer than and protruding from the subtending bracts; scapes 5−15 dm tall	*Xyris smalliana* Fig. 102
–	Lateral sepals shorter than subtending bracts, hidden (except when spikes open during maturity); scapes 1.5−12 dm tall	3
3	Summit of the scape distinctly flattened and broad relative to the spike; scape ridges 2−3, the two more prominent ridges comprising the flattened edge of the scape, therefore the upper scape ellipsoidal or fusiform in cross-section	*Xyris iridifolia*
–	Summit of the scape not flattened and broad relative to the spike; scape ridges > 3 (at least on the mid to lower portions of the scape), scape much narrower than the spike, terete or slightly flattened in cross-section	*Xyris jupicai* Fig. 101

Key adapted from Godfrey and Wooten (1979), Kral (2000b), and Weakley (2012).

### BASAL ANGIOSPERMS, MAGNOLIIDS, and EUDICOTYLEDONS

**Table:** 

1	Plant epiphytic	Santalaceae [*Phoradendron leucarpum*; Fig. 200]
–	Plant terrestrial or aquatic, not epiphytic	2
2	Plants woody [trees, shrubs, and lianas]	Key 1
–	Plants herbaceous [herbs and vines]	Key 2

### Key 1: Woody plants (trees, shrubs, and lianas)

**Table:** 

1	Plant a liana, climbing by means of tendrils, adventitious roots, or twining stems	2
–	Plant a tree or shrub, not climbing	10
2	Leaves compound	3
–	Leaves simple	7
3	Leaves opposite	4
–	Leaves alternate	5
4	Stems not ribbed; leaves 1-pinnate, leaflets (5−) 7−11 (−15), 4−8 cm long, ovate, unlobed, margins coarsely serrate, rounded at base, apices acute; inflorescence > 1-flowered, flowers erect or spreading, pedicels shorter to slightly longer than the calyx tube; calyx greenish-yellow or orange, campanulate to funnel-shaped, 1.5−2 cm long, lobes ascending, margins entire; corolla showy, orange to scarlet, funnelform, 6−8 cm long; fruit a fusiform, falcate, capsule	Bignoniaceae [*Campsis radicans*; Fig. 127]
–	Stems ribbed; leaves 1−2-pinnate or sometimes simple or trifoliolate, leaflets 4−10 plus a ± tendrilate terminal leaflet, (1.5−) 3−10 cm long, linear to ovate, unlobed or proximally 3−5 lobed, margins entire, bases broadly to narrowly cuneate, truncate, occasionally subcordate, apices acute, obtuse, or acuminate; inflorescence 1-flowered, flower pendent, pedicel > 2x length of the calyx tube; calyx violet-blue, campanulate, 2.5−5 cm long, lobes strongly recurved, margins crisped; corolla lacking; fruit an aggregate of achenes	Ranunculaceae [*Clematis crispa*; Fig. 187]
5	Leaves trifoliolate; plant climbing by adventitious roots, tendrils absent; fruit a drupe, white; plant containing contact poisons	Anacardiaceae [*Toxicodendron radicans*; Fig. 106]
–	Leaves palmately or pinnately compound but not trifoliolate; plant twining or tendrillate; fruit a legume and brown *or* a berry and blue; plant not containing contact poisons	6
6	Plant twining, tendrils absent; leaves 1-pinnate, leaflets 9−15, entire; fruit a legume	Fabaceae [*Wisteria frutescens*; Fig. 145]
–	Plant tendrillate; leaves palmately compound, leaflets (3−) 5 (−7), coarsely serrate on their distal margins; fruit a berry	Vitaceae [*Parthenocissus quinquefolia*; Fig. 208]
7	Leaves opposite	8
–	Leaves alternate	9
8	Plant twining; leaves evergreen, lanceolate to ovate, 3−9 × 1−2.5 cm, apices acute to acuminate, margins entire; flowers solitary or sometimes in 2−3-flowered axillary cymes; petals lemon yellow, connate; ovary superior	Gelsemiaceae [*Gelsemium sempervirens*; Fig. 147]
–	Plant climbing by means of aerial adventitous roots; leaves deciduous, ovate to orbicular 3−12 cm × 1−8 cm, apices abruptly short acuminate, acute, or obtuse, margins distally serrate; flowers numerous, borne in terminal compound cymes; petals white, not connate; ovary inferior	Hydrangeaceae [*Decumaria barbara*; Fig. 148]
9	Tendrils lacking; leaves 3−8 × 1.5−4 cm, elliptic to ovate, margins slightly wavy to entire; fruit a blue-black drupe, 5−7 mm long	Rhamnaceae [*Berchemia scandens*; Fig. 188]
–	Tendrils present, borne opposite the leaves; leaves 5−12 × 5−12 cm, orbicular to ovate, margins prominently dentate-serrate; fruit a blue-black berry, 1−2.5 cm long	Vitaceae [*Muscadinia rotundifolia*; Fig. 207]
10	Leaves opposite or whorled	11
–	Leaves alternate	15
11	Plant exhibiting varying degrees of both opposite and whorled leaf arrangement	12
–	Plant exhibiting strictly opposite leaf arrangement	13
12	Plant woody proximally and herbaceous distally, stem with a soft corky texture when under water, young stems strongly pubescent, green; petioles not connected by a central stipule or stipular scars; leaves lanceolate to elliptic, to 20 × 5 cm, bases and apices acute, glabrous adaxially and pubescent with branched hairs abaxially; flowers in cymose inflorescences; corolla majenta; stamens of 3 possible lengths, 2 of the 3 occuring in any one flower	Lythraceae [*Decodon verticillatus*; Fig. 165]
–	Plants woody entirely, stem not soft and corky when submersed, young stems sometimes short-pilose initially but becoming glabrous with age, reddish-brown; petioles connected by a central stipule or stipular scar; leaves oval, oblong oval, elliptic or ovate, to 15 × 10 cm, bases broadly rounded to cuneate, apices acute or acuminate, glabrous adaxially and short-pilose abaxially (at least on the principal veins); flowers in dense globose heads, corolla white; stamens of one length	Rubiaceae [*Cephalanthus occidentalis*; Fig. 194]
13	Leaves simple	Sapindaceae [*Acer*]
–	Leaves compound	14
14	Leaves 1-pinnate, imparipinnate, leaflets 5−7 (−9); inflorescences borne on old wood of previous growing seasons before the development of new shoots; corolla not scarlet red; fruit a samara	Oleaceae [*Fraxinus caroliniana*; Fig. 179]
–	Leaves palmately compound, leaflets 5−7; inflorescences borne on new shoots of current year; corolla scarlet red; fruit a capsule	Sapindaceae [*Aesculus pavia*; Fig. 203]
15	Leaves compound	16
–	Leaves simple	18
16	Stems arching, trailing, or erect to 2 m tall, armed with numerous prickles; leaves 1-pinnately or 1-palmately compound, leaflets 3−9; fruit an aggregate of drupes or an aggregate of achenes enclosed in a hip	Rosaceae [*Rosa* & *Rubus*]
–	Stems erect, > 2m in height, lacking prickles; leaves 1-pinnately compound, leaflets 5−23; fruit a nut or drupe	17
17	Plant a shrub to small tree, to 7 m tall; stems densely short pubescent; leaflets 9−11 (−23), 3−8 ×1−4 cm, rachis winged; fruit a drupe	Anacardiaceae [Rhus copallinum var. copallinum; Fig. 105]
–	Plant a medium to large tree (unless in juvenile stage of development), to 30 m tall; stems not densely pubescent; leaflets (3−) 5 (−7), 3−22.5 × 1.8−13 cm; rachis not winged; fruit a nut enclosed within a husk	Juglandaceae [*Carya glabra*; Fig. 154]
18	Flowers borne in heads subtended by an involucre of bracts	Asteraceae [*Baccharis halimifolia*; Fig. 112]
–	Flowers not borne in heads subtended by an involucre of bracts	19
19	Leaves palmately lobed, margins glandular-serrate; fruit a multiple of capsules	Altingiaceae [*Liquidambar styraciflua*; Fig. 103]
–	Leaves pinnately or palmately lobed, margins otherwise; fruit otherwise	20
20	Fruit a nut (acorn) bearing a basal cupule (“cap”); buds conspicuously clustered at twig tips, scales imbricate	Fagaceae [*Quercus nigra*; Fig. 146]
–	Fruit otherwise; axillary buds not clusted at twigs tips with scales imbricate	21
21	Stipular scars conspicuous, completely encircling the twig stems or nodes	22
–	Stipular scars (if present) not encircling the twig stems or nodes completely	23
22	Bark of mature specimens gray, intact; leaves 6−15 × 2−6 cm, tardily deciduous, long-elliptic to oblong, adaxial surface light green and glabrous, abaxial surface strongly glaucous, petiole base solid, not covering axillary bud; flowers very showy and fragrant, “magnolia-like”; fruit an aggregate of follicles, elongate, seeds red-arillate	Magnoliaceae [Magnolia virginiana var. virginiana; Fig. 166]
–	Bark of mature specimens brown, furrowed, and scaly proximally, sloughing away to reveal a bright white smooth inner bark distally; leaves to 35 cm long and wide, deciduous, as long as broad, both adaxial and abaxial surfaces green, petiole base hollow, covering axillary bud; flowers neither showy nor fragrant; fruit a multiple of achenes, spherical, seeds brown, not arillate	Platanaceae [*Platanus occidentalis*; Fig. 184]
23	Flowers unisexual and borne in catkins	24
–	Flowers bisexual and not borne in catkins	26
24	Leaves pleasantly aromatic when crushed, glandular punctate abaxially	Myricaceae [*Morella cerifera*; Fig. 173]
–	Leaves not aromatic when crushed; not glandular punctate abaxially	25
25	Leaves < 3× as long as broad, very “neatly” pinnately veined, lateral veins consistently parallel to one another, ovate-triangular, sub-rhombic, elliptic, obovate, or oblong, margins either doubly serrate or slightly wavy; fruit a nutlet, 1-seeded	Betulaceae
–	Leaves > 3× as long as broad, not so neatly pinnately veined, lanceolate, margins serrate; fruit a capsule, 2-valved, many-seeded	Salicaceae [*Salix*]
26	Leaves broadly ovate to rhombic-ovate	27
–	Leaves longer than broad	28
27	Plant exuding milky sap when injured; leaves to 7 (−9) cm long; fruit a 3-valved capsule, maturing after leaf maturation in late summer to fall	Euphorbiaceae [*Triadica sebiferum*; Fig. 144]
–	Plant lacking milky sap; larger leaves ≥ 10 cm long; fruit a 2−4-valved capsule, maturing prior to leaf emergence in the spring	Salicaceae [*Populus heterophylla*; Fig. 197]
28	Fruits dry (capsules, berry-like, samaras)	29
–	Fruits fleshy (berries, drupes, pomes)	34
29	Leaves 2-ranked on the twigs, bases markedly oblique; fruit a samara	Ulmaceae [*Ulmus americana*; Fig. 206]
–	Leaves ≥ 3-ranked on the twigs, bases not oblique; fruit not a samara	30
30	Fruit indehiscent, berry-like; stems typically sharply longitudinally ridged below point of attachment of leaf petioles; leaves spatulate to oblanceolate, margins entire	Cyrillaceae [*Cyrilla racemiflora*; Fig. 133]
–	Fruit a dehiscent capsule; stems not longitudinally ridged below point of attachment of leaf petioles; leaves obovate, elliptic, oblong, or lanceolate, margins toothed (if entire, then blades with a perimarginal vein, lepidote, or with margins ciliate)	31
31	Plant a tree, to 26 m tall; flowers solitary, axillary; stamens > 50	Theaceae [*Gordonia lasianthus*; Fig. 205]
–	Plant a shrub, < 6 m tall; flowers numerous, borne in racemes or spikes; stamens ≤ 10	32
32	Pith chambered; ovary 2-locular	Iteaceae [*Itea virginica*; Fig. 153]
–	Pith solid; ovary ≥ 3-locular	33
33	Young twigs, inflorescence rachises, pedicels, and calyces stellate-pubescent; leaves oblanceolate, widest above middle, margins serrate distally; corolla rotate, petals connate ≤ ½ their length, lobes 5−8 mm long; ovary 3-locular	Clethraceae [*Clethra alnifolia*; Fig. 132]
–	Young twigs, inflorescence rachises, pedicels, and calyces glabrous or pubescent, but not stellate-pubescent; leaves lanceolate, ovate, elliptic, oblanceolate, or narrowly obovate, widest at or below the middle (widest above middle in *Rhododendron viscosum*, but margins finely bristly-ciliate); corolla urceolate, campanulate, globose, rotate, or funnelform, petals connate ≥ ½ their length, lobes either < 4 mm long or 7−24 mm long; ovary 5-locular	Ericaceae [in part]
34	Fruit a pome	Rosaceae [*Amelanchier* and *Aronia*]
–	Fruit a drupe or berry	35
35	Leaves evergreen	36
–	Leaves deciduous or tardily deciduous	37
36	Leaves not aromatic when crushed; margins spinose, crenate, or sometimes entire, lacking deforming galls; drupes containing 4−8 seeds	Aquifoliaceae [*Ilex*]
–	Leaves with a spicy aromatic scent when crushed; margins entire, often with numerous deforming galls (galls a result of red bay psyllid activity); drupes containing 1 seed	Lauraceae [*Persea palustris*; Fig. 156]
37	Plant a shrub, typically < 4 m in height; flowers perfect; fruit a blue, purple, or black berry; seeds ≥ 10, ca. 1.2 mm long	Ericaceae [*Vaccinium*]
–	Plant a small to full sized tree, > 4 m in height; flowers imperfect or perfect; fruit a drupe or berry, if berry then yellow to orange (2−) 3−5 (−7.7) cm in diam, seeds 3−8, > 5 mm long	38
38	Vascular bundle scars 1 per leaf scar; leaves generally widest at or below the middle, margins lacking teeth, fruit a berry, orange at maturity, (2−) 3−5 (−7.7) cm in diam, subtended by a thick leathery calyx	Ebenaceae [*Diospyros virginiana*; Fig. 135]
–	Vascular bundle scars 3 per leaf scar; leaves generally widest at or above the middle, sometimes toothed (as in *Nyssa aquatica*); fruit a drupe, blue-black at maturity 0.7−1.2 cm in diam., a thick leathery calyx lacking	Nyssaceae [*Nyssa*]

Key adapted from Radford et al. (1968), Godfrey and Wooten (1981), and Weakley (2012).

### Key 2: Herbaceous plants (herbs and vines)

**Table:** 

1	Flowers borne in ligulate, radiate, or discoid heads subtended by an involucre of bracts	Asteraceae
–	Flowers various but not borne in heads subtended by an involucre of bracts	2
2	Plant carnivorous, leaves modified into tube-like pitchers (Sarraceniaceae) ***or*** containing small inconspicuous “bladders” (Lentibulariaceae) ***or*** with obvious glandular trichomes (Droseraceae)	3
–	Plant not carnivorous, lacking the above carnivorous characters	5
3	Leaves modified into conspicuous water-storing, tubular pitchers; flowers with a conspicuous style disk, a strong odor of ammonia (somewhat like cat urine) present; stamens 50−100	Sarraceniaceae [*Sarracenia flava*; Fig. 204]
–	Leaves not modified into water-storing, tubular pitchers; flowers lacking a style disk, lacking a strong odor of ammonia; stamens < 50	4
4	Plants terrestrial (occuring in moist to saturated soils), leaves lacking bladder-like traps, instead exhibiting glandular trichomes; corolla actinomorphic, not 2-lipped, white	Droseraceae [*Drosera intermedia*; Fig. 134]
–	Plant terrestrial (occuring in moist to saturated soils) or aquatic (typically found floating on the water surface), leaves bearing small, subterranean, urn-like or bladder-like traps; corolla zygomorphic, 2-lipped, corolla yellow or purple-lavender	Lentibulariaceae [*Utricularia*]
5	Plant a rooted aquatic, having either submersed, floating, or both submersed and floating leaves [Plants included in this section are the “prototypical” truly aquatic plants, exhibiting submersed or floating leaves. However, fluctuating water levels can cause a small degree of ambiguity. Increasing water levels may flood emergent wetland plants and give them the appearance of having submersed or floating leaves. Similarly, receding water levels may leave “prototypical” aquatic plants stranded and give them the appearance of emergents. Taking this into consideration, certain families and genera are included both in this lead and the next to ensure a broad range of environmental conditions are covered]	6
–	Plant terrestrial, emergent, with only roots and/or basal leaves inundated	11
6	Leaves of two types: submersed cauline, opposite, and comprised of dichotomously dissected linear segments, floating alternate, simple, and peltate, blades elongate-rhombic	Cabombaceae [*Cabomba caroliniana*; Fig. 129]
–	Leaves of one type (two in *Nuphar sagittifolia*, but then submersed leaves not cauline and not dichotomously divided): floating (submersed, floating, or erect in *Hydrocotyle umbellata*), peltate or not, blades oval, orbicular, cordate, ovate, reniform, lanceolate, or oblong-lanceolate	7
7	Leaves peltate	8
–	Leaves not peltate, petiole attached to a cuneate, sagittate, or cordate base	10
8	Underwater portions of plant coated with transparent mucilaginous jelly; leaves elliptic	Cabombaceae [*Brasenia schreberi*; Fig. 128]
–	Underwater portions of plant lacking mucilaginous jelly; leaves orbicular	9
9	Leaves < 8 cm in diam., submersed, floating, or emersed at maturity, margins crenate; peduncle (inflorescence stalk) equaling or just exceeding the leaves	Araliaceae [*Hydrocotyle umbellata*; Fig. 111]
–	Leaves > 20 cm in diam., floating (sometimes emersed during falling water levels), margins entire; peduncle (inflorescence stalk) tall, commonly overtopping the leaves	Nelumbonaceae [*Nelumbo lutea*; Fig. 174]
10	Leaf 5−15 cm long, ovate to reniform; petiole often reddish purple-punctate; inflorescence borne amongst or immediately subtended by a cluster of stout, fleshy, tuber-like, bannana-shaped roots; flowers 4−5-merous (eudicot)	Menyanthaceae [*Nymphoides aquatica*; Fig. 172]
–	Leaf (5−) 10−50 cm long, orbicular or lanceolate to oblong-lanceolate; petiole not reddish purple-punctate; inflorescence not amongst or subtended by fleshy tuber-like roots; flowers > 5-merous (basal angiosperm)	Nymphaeaceae
11	Leaves peltate	Araliaceae [*Hydrocotyle umbellata*; Fig. 111]
–	Leaves not peltate	12
12	Plant exuding milky sap when injured	13
–	Plant exuding clear sap when injured	14
13	Plant 2−10 dm tall; cauline leaves .1−15 × .1−.8 cm, linear, narrowly elliptic, or oblanceolate, margins callose toothed; flowers single and relatively distant from one another on the racemes or raceme-like branches; sepals not composed of an inner two (enlarged and wing-like) and an outer three (reduced); corolla blue to bluish-white	Campanulaceae
–	Plant 0.5−4 dm tall; leaves 1.5−6 × 0.5−2 cm, spatulate to obovate, margins lacking callose teeth; flowers in dense racemose terminal heads; sepals 5, composed of an inner two (enlarged and wing-like) and on outer three (reduced); corolla orange	Polygalaceae [*Polygala lutea* Fig. 185]
14	Leaves basal (sprouting form the nodes of a stolon) and simple ***or*** cauline and 1−3-pinnately compound (*Cicuta maculata*; this plant is extremely poisonous and care should be taken when handling plant parts); inflorescence a single or compound umbel; fruit a schizocarp	Apiaceae
–	Leaves various, if basal then not sprouting from nodes of a stolon and if cauline then not compound; inflorescence not umbellate; fruits various but not a schizocarp	15
15	Cauline leaves alternate	16
–	Cauline leaves opposite	18
16	Perianth differentiated into sepals and petals; corolla zygomorphic, bluish-purple and white	Plantaginaceae [*Nuttallanthus canadensis* Fig. 183]
–	Perianth undifferentiated and comprised of green, pinkish, or red tepals, ***or*** comprised solely of sepals (petals lacking)	17
17	Mature stems to 8 dm tall, relatively dainty (herb-like), if submersed then not spongy and thickened, ocrea present; leaves primarily basal, those along stem stem much reduced and distant from one another, bases mostly hastate (sometimes cuneate due to relatively frequent wave disturbance); inflorescence composed of terminal paniculate racemes; sepals 6; fruit a single achene kept inside the inner calyces	Polygonaceae [*Rumex hastatulus* Fig. 186]
–	Mature stems to 10 dm tall, robust (shrub-like), submersed stems becoming very spongy and enlarged, apparently “splitting” in place, ocrea absent; leaves evenly distributed on the stem, not reduced and distant on the stem; inflorescence composed of a solitary flower in the leaf axils; sepals 4; fruit a capsule	Onagraceae [*Ludwigia sphaerocarpa* Fig. 181]
18	Stems dichotomously branched, “wiry” in overall appearance; cauline leaves ≤ 2 mm long, subulate, bases pectinately-fringed	Caryophyllaceae [*Stipulicida setacea* Fig. 131]
–	Stems not dichotomously branched, not “wiry” in overall appearance; cauline leaves > 2 mm long, not subulate, bases not pectinately-fringed	19
19	Stems 4-angled; flowers in dense axillary clusters; corolla < 7 mm long, connate most of length, white, somewhat bi-labiate, 5-lobed, pubescent within; fruit a nutlet	Lamiaceae [*Lycopus angustifolius* Fig. 155]
–	Stems not 4-angled (4-angled in Melastomataceae, but not with the above combination of floral characters); flowers not in dense axilary clusters; corolla not as above; fruits various but not a nutlet	20
20	Corolla ≤ 5 mm long; flowers secund on small branchlets; capsule swollen at the base with two incurving appendages distally, thus having the appearance of “horns”	Loganiaceae [*Mitreola petiolata* Fig. 164]
–	Corolla ≥ 5 mm long; flowers not secund on small branchlets; capsule not swollen at the base with horn-like appendages distally	21
21	Plants commonly creeping and forming small to large mats in shallow water; flowers solitary or in head-like inflorescences arising from leaf axils	22
–	Plants not creeping or forming small to large mats in shallow water; flowers and fruits not borne in leaf axils (except for Linderniaceae and Rubiaceae)	24
22	Leaves to 9 cm long, linear-elliptic, apices acute and tipped with a tiny spine; inflorescence a multi-flowered axillary or terminal white head; fruit an utricle	Amaranthaceae [*Alternanthera philoxeroides* Fig. 104]
–	Leaves to 2 cm long, ovate, oblanceolate, or sometimes elliptic, apices acute to rounded, lacking a tiny spine; inflorescence composed of a single axillary flower, corolla pale or bright blue to violet-blue ***or*** yellow; fruit a capsule	23
23	Plant lacking a pleasant citrus-spicy aroma when crushed; stems without spongy or succulent texture; leaves to 2.5 ×0.6 cm, oblanceolate, apices acute to obtuse, petals separate, ≤ 9 mm long yellow	Onagraceae [*Ludwigia brevipes* Fig. 180]
–	Plant with a very pleasant citrus-spicy aroma when crushed; stems with a spongy and succulent texture; leaves to 2 × 1.5 cm, ovate, apices obtuse to rounded; petals connate, 9−13 mm long, pale or bright blue to violet-blue	Plantaginaceae [*Bacopa caroliniana* Fig. 182]
24	Flowers axillary, solitary, usually in the axils of one of a given pair of leaves (sometimes one in each axil of the pair); sepals linear-attenuate scabrous; corolla funnelform, 5-lobed, upper lip erect and shallowly 2-lobed, lower lip deflexed and 3-lobed, lavender	Linderniaceae [*Lindernia dubia* Fig. 163]
–	Flowers axillary or not, if so, then sometimes having more than 1 flower per axil and always in the axils of both of a given pair of leaves; sepals various, not scabrous; corolla various but if connate then not with the above floral characters, white, lavender, rose, pink, purple, or yellow	25
25	Leaves connected by interposed stipules or foliaceous stipules, if foliaceous, then indistinguishable from the leaves, thus the leaves appearing whorled; corollas white, connate basally to form a tube, or separated into 3–4 distinct petals	Rubiaceae [in part]
–	Leaves not connected by interposed or foliaceous stipules, not appearing whorled; corolla yellow, purple, pink, rose, or lavendar, never connate basally to form a tube, always separated into 4−5 distinct petals	26
26	Leaves glabrous, punctate-dotted, entire, not decussate; petals 4−5, pink (flesh-colored) or yellow; stamens sometimes grouped into fascicles, staminodia sometimes present; ovary superior, fruit a septicidal capsule not enclosed within a hypanthium	Hypericaceae
–	Leaves often pubescent or sparingly pubescent, not punctate-dotted, usually serrated or coarsely toothed, decussate; petals 4, pink, rose, or lavendar; stamens never grouped into fascicles, staminodia lacking; ovary inferior; fruit a loculicidal capsuse enclosed within an urceolate-shaped hypanthium	Melastomataceae

Key adapted from Radford et al. (1968), Godfrey and Wooten (1981), and Weakley (2012).

### Anacardiaceae

**Table:** 

1	Leaves imparipinnate, leaflets ≥ 7, rachis winged; fruits red, glandular pubescent; plant lacking contact poisons; inflorescences terminal	Rhus copallinum var. copallinum Fig. 105
–	Leaves pinnately trifoliolate; fruits white to yellow, glabrous or puberulant, hairs eglandular; plant containing contact poisons; inflorescences axillary	Toxicodendron radicans var. radicans Fig. 106

Key adapted from Radford et al. (1968) and Weakley (2012).

### Apiaceae

**Table:** 

1	Stems elongate-rhizomatous, horizontal, low-growing; leaves simple, blades ovate to oblong, 1.5–5 (−10) × 1.5 −3.5 (−8) cm, apices rounded, base cordate to truncate, margins denticulate; umbels simple, 1−4 (−9) flowers per umbel, pedicels 0.5−3 mm long; fruit strongly flattened laterally, prominently nerved with raised reticulate venation between nerves, corky ribs lacking	*Centella asiatica* Fig. 107
–	Stems erect, not horizontal or low-growing; leaves 1–3 times pinnately compound, blades to 30 × 25 cm, leaflets lanceolate to lance-oblong, 4−7 (−14) cm ×0.6−3 (−5) cm, apices acute, bases cuneate to rounded, frequently asymmetrically so, margins serrate; umbels compound, > 9 flowers per umbel, pedicels 2−10 mm long; fruit somewhat flattened laterally with strong, flattish corky ribs	*Cicuta maculata* Fig. 108

Key adapted from Radford et al. (1968), Godfrey and Wooten (1981), and Weakley (2012).

### Aquifoliaceae

**Table:** 

1	Leaves 1.5−3× as long as wide, ca. 2−3 cm wide, with a few, irregularly spaced, marginal spinose teeth, if present, spreading away from the leaf apex	*Ilex coriacea* Fig. 109
–	Leaves 3−4× as long as wide, ca. 1 cm wide (almost never > 2 cm wide), crenate in the apical 1/2−1/3 of the leaf, marginal teeth pointing toward the leaf apex	*Ilex glabra* Fig. 110

Key adapted from Godfrey and Wooten (1981) and Weakley (2012).

### Araliaceae

**Table:** 

1	Leaves not peltate	*Hydrocotyle ranunculoides*
–	Leaves peltate	2
2	Inflorescence umbellate, leaf blades 1–4 (–7) cm wide	*Hydrocotyle umbellata* Fig. 111
–	Inflorescence verticillate, all flowers borne sessile to subsessile on the unbranched inflorescence axis; leaf blades 1–6 cm wide	*Hydrocotyle verticillata* Fig. 210

Key adapted from Godfrey and Wooten (1981) and Weakley (2012).

Note: The North Carolina Natural Heritage Program (North Carolina Natural Heritage Program 2014) and Carolina Vegetation Survey (Peet et al. 2013a, Peet et al. 2013b) reported *Hydrocotyle umbellata* from Lake Waccamaw; however, reproductive specimens were not encountered by the first author. Three species of *Hydrocotyle* are likely to occur in North Carolina Coastal Plain littoral communities. Two of the three posses peltate leaves. All material collected by the current author possessed peltate leaves and thus can be either *H. umbellata* or *H. verticillata*. Of these, *H. umbellata* is most likely to occur in this habitat, but as a precautionary measure, all three are included in the key below.

### Asteraceae

**Table:** 

1	Plant a woody shrub, with obvious woody growth well above ground level	*Baccharis halimifolia* Fig. 112
–	Plant an herb or twining vine, lacking obvious woody growth above ground level	2
2	Plant a twining vine; leaves opposite, bases cordate, margins coarsely toothed	*Mikania scandens* Fig. 121
–	Plant an herb; leaves opposite, alternate, whorled, or basally disposed, bases and margins various	3
3	Plant exuding milky sap when cut or damaged; heads ligulate (containing only ligulate [ray] flowers)	4
–	Plant exuding clear sap when cut or damaged; heads discoid (only containing disc flowers) or radiate (with both ligulate [ray] and disc flowers in the same head)	5
4	Leaves completely basally disposed in a rosette, the flowering stem therefore being scapose (lacking leaves); involucre of 2 or more series of bracts; rays 1–1.5 cm long; cypselas beaked; pappus composed strictly of bristles	*Hypochaeris radicata* Fig. 119
–	Leaves primarily basally disposed, sometimes a few leaves extending up the stem, these alternate; involucre of 1 series of bracts; rays 0.6–1 cm long; cypselas beakless, pappus composed of 5 bristles and 5 scales	
5	Leaves opposite or whorled (at least on the lower stem nodes)	6
–	Leaves alternate	8
6	Leaves whorled, 8−20 × 0.3−2 mm; inflorescence composed of a single, terminal, pink, discoid head; plants no more than 45 cm tall, mat-forming	*Sclerolepis uniflora* Fig. 123
–	Leaves opposite (at least on lower stem nodes, sometimes becoming alternate distally), leaves > 20 × > 2 mm; inflorescence composed of more than one head, heads discoid or radiate, not pink; mature plants > 45 cm tall, erect, not mat-forming	7
7	Plant an annual; heads radiate, borne singly or in ± corymbiform arrays, rays yellow; leaves simple, (20−) 50−100 (−160+) × (5−) 10−25 (−40+) mm, sessile; phyllaries 8−12, ovate to obovate to lance-oblong, (4−) 6−8 (−10+) mm, tips orange to purplish; disc florets (25−) 60−100 (−150+); cypselae 6−10 mm, pappi of 2−4 retrorsely barbed awns, 3−5 mm long	*Bidens laevis* Fig. 113
–	Plant a perennial; heads discoid, corymbiform or paniculiform arrays, corollas white; leaves simple or pinnate/pinnatifid, 5−100 × 0.2−10 (−15) mm, sessile; phyllaries 8−10, narrowly elliptic, 0.5−8 × 0.2−1.2 mm, tips green; disc florets 5; cypselae 1−3 mm, pappi of 20−40 antrorsely barbed bristles, 2−5 mm long	* Eupatorium *
8	Heads discoid, phyllaries pink, disc corollas rose-pink; stems, leaves, and phyllaries stipitate to sessile glandular (sometimes viscid)	*Pluchea baccharis* Fig. 122
–	Heads radiate, phyllaries green, disc corollas yellow; stems, leaves, and phyllaries eglandular	9
9	Ray florets yellow	10
–	Ray florets white	11
10	Leaves on the middle to distal portions of the stem linear, 24−70 × 1−3 mm, bases attenuate, if sessile, not clasping the stem, abundantly gland-dotted, scabro-villous on mid-nerves; heads corymbose, ray florets 7−17 (−25), disc florets 3−22, corollas 3.3−4.8 mm long; stems sparsely pubescent, 2.5−10 dm tall	*Euthamia caroliniana* Fig. 118
–	Leaves on the middle to distal portions of the stem lanceolate-ovate to ovate-oblong, larger leaves 35−120 × 8−35 mm, bases auriculate, broad and more or less clasping, hirsuto-villous on the midnerves, not gland-dotted; heads paniculate, ray florets (2−) 4−10, disc florets (2−) 4−7, corollas 4−5 mm long; stems conspicuously hirsute, 5−15 dm tall	*Solidago fistulosa* Fig. 124
11	Leaves cauline, linear to lanceolate, 2−22 × 0.2−3 cm, not fleshy thickened; heads 50−100; ray florets 8−20 mm long; involucres 2.4−3.8 × 3.7−8.7 mm; cypselae obovoid, 1−3 mm, pappi comprised of 9 or 18 awns, (0−) 0.4−1.2 mm long; plants 3−20 dm tall, stoloniferous	Boltonia asteroides var. glastifolia Fig. 114
–	Leaves mostly basal, narrowly to broadly oblanceolate to spatulate, 2−10 (−15+) × 0.4−2.5 cm, more or less fleshy thickened; heads (1−) 4−20 (−25); ray florets 5−10 mm long; involucres 3−4 ×5−11 mm; cypselae subterete, 1.2−1.6 mm, pappi comprised of setae (outer) and 16−25 bristles (inner), bristles 2.5−3.3 mm long; plants 1.5−5 dm tall, rhizomatous or fibrous-rooted	*Erigeron vernus* Fig. 115

Key adapted from Radford et al. (1968), Godfrey and Wooten (1981), Barkley et al. (2006), and Weakley (2012).

### Betulaceae

**Table:** 

1	Buds stalked; pistillate catkins becoming hard and woody, forming a persisting cone-like catkin that persists through the winter and into the next growing season; plant a shrub, < 4 m tall; bark tight, not sloughing away from trunk; leaves 3-ranked, blades 5−10 cm × 2.5−5 cm, obovate, elliptic, or oblong, margins entire to serrulate	*Alnus serrulata* Fig. 125
–	Buds sessile; pistillate catkins not becoming woody or hard and not persisting through the winter and into the next season; plant a tree, > 10 m tall; bark loose, sloughing away from trunk, usually with the consistency of paper; leaves 2-ranked, blades 3−10 cm × 1.5−3 cm, ovate-triangular or sub-rhombic, margins coarsely doubly serrate to dentate	*Betula nigra* Fig. 126

Key adapted from Radford et al. (1968), Godfrey and Wooten (1981), and Weakley (2012).

### Cabombaceae

**Table:** 

1	Leaves floating only, blades elliptic, 3.5–11 × 2–6.5 cm, peltate; submersed plant parts coated with a layer of transparent mucilage	*Brasenia schreberi* Fig. 128
–	Leaves floating and submersed, blades of floating leaves elliptic, 0.6–3 × 0.1–0.4 cm, peltate, blades of submersed leaves dichotomously divided into linear segments; submersed plant parts not coated with mucilage	*Cabomba caroliniana* Fig. 129

Key adapted from Godfrey and Wooten (1981) and Weakley (2012).

### Campanulaceae

**Table:** 

1	Plant perennial; stems 7−10 dm tall; stem leaves linear to narrowly oblanceolate, 8−15 × 0.5−0.8 cm, margins callose glandular (sometimes not), often with short translucent trichomes on or near the margins; subtending bracts shorter than or exceeding the pedicels in length; corollas (including hypanthium) 18−33 mm long, fenestrate (with a slit or window on each side of the corolla tube at the base); plant of seasonally wet to inundated soils	*Lobelia glandulosa*
–	Plant annual; stems 2−7.5 dm tall, stem leaves lanceolate to linear, 1−3.5 × 0.1−0.4 cm, margins callose (not glandular), lacking short transluscent trichomes on or near the margins; subtending bracts shorter than or rarely equaling the pedicels in length; corollas (including the hypanthium) 8−14 mm long, not fenestrate; plant of various savnna-like habitats, and occassionally in wetter soils, but never found in inundated soils or wetlands	*Lobelia nuttallii* Fig. 130

Key adapted from Godfrey and Wooten (1981) and Weakley (2012).

### Ericaceae

**Table:** 

1	Ovary inferior; fruit a berry	* Vaccinium *
–	Ovary superior; fruit a capsule	2
2	Leaves evergreen, blades coriaceous, adaxial surface ***either*** dark green and shiny ***or*** dull olive green and lepidote (covered with small, white or yellowish scurfy scales)	3
–	Leaves deciduous, blades membranous or subcoriaceous, deciduous, adaxial surface light to dark green, dull, not lepidote	4
3	Twig and leaf blade surfaces prominently lepidote, adaxial leaf surface dull olive green, lacking a prominent perimarginal vein	*Chamaedaphne calyculata* Fig. 136
–	Twig and adaxial leaf blade surfaces glabrous, not lepidote, adaxial leaf surface dark green and shiny, larger leaves with a prominent perimarginal vein ca. 1 mm from blade margin	*Lyonia* [*Lyonia lucida* Fig. 139]
4	Leaves predominantly obovate or oblanceolate, margins distinctly long-ciliate; corolla funnelform, lobes > 10 mm long; capsule elongate, > 2 × as long as broad, 7–23 mm long	Rhododendron viscosum var. serrulatum Fig. 140
–	Leaves various, margins not long-ciliate; corolla urceolate, campanulate, or globose, lobes < 5 mm long, capsule oblate (spheroidal, but flattened apically and basally), ovoid, globose, or subglobose, nearly as broad as long or broader, 2–6.5 mm long	5
5	Leaf margins crenate; corolla campanulate; capsule oblate	*Zenobia pulverulenta* Fig. 143
–	Leaf margins spinulose-serrate, serrulate, or entire; corolla urceolate or globose; capsule ovoid, globose, or subglobose	6
6	Leaf margins spinulose-serrate; inflorescence of racemes produced along stems of previous year; capsules not thickened and whitish along sutures; seeds 5–10 per capsule	*Eubotrys racemosa* Fig. 137
–	Leaf margins entire to minutely serrulate; inflorescence of terminal panicles produced on stems of current year, proximal inflorescences often with conspicuous leaf-like bracts; capsules thickened and whitish along sutures; seeds 100–300+ per capsule	*Lyonia ligustrina* Fig. 138

Key adapted from Tucker (2009b) and Weakley (2012).

### *Lyonia* Nutt.

**Table:** 

1	Leaves deciduous, blades subcoriaceous, dull, lacking a prominent perimarginal vein, margins serrulate; corollas urceolate 2–4(−4.5) mm long; calyx lobes 0.5–1.5 mm long	Lyonia ligustrina var. foliosiflora Fig. 138
–	Leaves evergreen, blades coriaceous, shiny, with a prominent perimarginal vein, leaf margins entire; corollas cylindric 5–14 mm long; calyx lobes 2–9.5 mm long	*Lyonia lucida* Fig. 139

Key adapted from Judd (2009) and Weakley (2012).

### *Vaccinium* L.

**Table:** 

1	Twigs of the year glabrous; leaves glabrous below, margins eciliate; berries blue	*Vaccinium formosum* Fig. 141
–	Twigs of the year pubescent; leaves pubescent below, margins ciliate; berries black	*Vaccinium fuscatum* Fig. 142

Key adapted from Weakley (2012).

### Hypericaceae

**Table:** 

1	Petals flesh-colored to pink; stamens in 3 fascicles, each fascicle containing 3 stamen; 3 orange staminodial glands alternating with the 3 fascicles of stamen	2
–	Petals yellow, stamens few, not in fascicles; orange staminodial glands lacking	3
2	Leaves sessile, clasping the stem, cordate or subcordate at the base, mostly 2−7 × 1−3 cm; sepals 5−8 mm long at maturity, acute to acuminate; filaments united basally; styles 1.8−3 mm long	*Hypericum virginicum* Fig. 151
–	Leaves petiolate (at least the lower), not clasping the stem, cuneate at the base, up to 15 × 3.5 cm; sepals 3−5 mm long at maturity, apices obtuse; filaments united to above the middle; styles 1.5−3 mm long	*Hypericum walteri* Fig. 152
3	Leaf blades lanceolate to linear, 1−3-nerved, 6−30 mm long, bases attenuate to cuneate, not clasping, apices blunt to acute; petals 5, 6−8 mm long; capsules purplish, slightly exceeding the calyx	*Hypericum canadense* Fig. 149
–	Leaf blades ovate, elliptic, lanceolate, 5-nerved, 10−50 mm long, bases broad, sometimes clasping, apices rounded to blunt; petals 5, 2−3 mm long; capsules not purplish, equaling the calyx	Hypericum mutilum var. mutilum Fig. 150

Key adapted from Godfrey and Wooten (1981) and Weakley (2012).

### Lentibulariaceae

**Table:** 

1	Plants aquatic, floating unattached in water (sometimes stranded on top of soil by receding water levels); bladders 0.7–5 mm long, mostly > than 1.0 mm long; seeds 0.5–2 mm long	2
–	Plants terrestrial, attached to soil (principal branches within the soil); bladders 0.2–1.1 mm long, mostly < 1.0 mm long; seeds 0.2−0.25 mm long	5
2	Flowers purple; leaves divided into verticillate segments with terminal traps	*Utricularia purpurea* Fig. 159
–	Flowers yellow; leaves divided into alternate segments with lateral traps; upper corolla lip larger than the lower, obscurely 3–lobed	3
3	Plant exhibiting vegetative shoots of two types, some bearing leafy segments with few or no traps, others bearing reduced segments and many traps; seeds 1.0–2.5 mm long, with an irregularly deeply lobed or partial wing (plant of shallow water or left stranded on soil surface after receding water levels)	*Utricularia striata* Fig. 161
–	Plant exhibiting uniform vegetative shoots, all bearing sparsely divided leaf segments with traps; seeds 0.8–1.1 mm long, with a continuous, circumferential wing, slightly to irregularly lobed	4
4	Lower corolla lip 8–10 mm long, equaling or slightly shorter than the conical, 5–9 mm long spur; leaves usually forked twice	[*Utricularia biflora* Fig. 211]
–	Lower corolla lip 5–6 mm long exceeding the blunt 3.5–4.5 mm long spur; leaves usually forked once	*Utricularia gibba* Fig. 158
5	Corolla rose pink; inflorescence 1 (−2)-flowered; bract at base of pedicel tubular, attached circumferentially around stem; aerial leaves (when present) terete, septate	*Utricularia resupinata* Fig. 160
–	Corolla yellow (sometimes fading white); inflorescence (1−) 2−15-flowered; bract at base of the pedicel peltate or ovate, attached on one side of the stem; aerial leaves (when present) flattened, not septate	6
6	Pedicels subtended by a single ovate (attached at base) bract; pair of bracteoles present, bracteoles linear to lanceolate, a little longer than the bract; corolla spur oriented downward or backward, at right angle to lower corolla lip	*Utricularia cornuta* Fig. 157
–	Pedicels subtended by a single peltate (attached in middle) bract, unattached at either end; pair of bracteoles absent; corolla spur oriented forward, essentially appressed to lower corolla lip; aerial leaves (when present) with subacute or obtuse apices	*Utricularia subulata* Fig. 162

Key adapted from Godfrey and Wooten (1981), Taylor (1989), and Weakley (2012).

Note: Traditionally, in the southeastern United States, *U. biflora* and *U. gibba* have been recognized as distinct species (Radford et al. 1968). However, Radford et al. (1968) described the two as “doubtfully distinct” and neither Godfrey and Wooten (1981), nor Taylor (1989), recognized a distinction. Here, we follow Weakley (2012) in provisionally recognizing the two species as distinct, pending a world-wide revision. During the present work, only *U. gibba* was encountered in the field, but *U. biflora* (bracketed in key below) is keyed here due to its morphological similarity, overlapping range, and similar habitat requirements.

### Melastomataceae

**Table:** 

1	Sepal lobes aristate, awn tip 0.5–1.5 mm long, hairs 3–5 mm long, yellow, stiff	*Rhexia aristosa* Fig. 167
–	Sepal lobes obtuse to acuminate, not aristate, hairs < 3 mm long, neither yellow nor stiff	2
2	Leaves linear or linear-elliptic, 1– 5(−8) mm wide	3
–	Leaves lanceolate, elliptic, or ovate, (5−) 7–20 (−35) mm wide	4
3	Petals lavender–rose, (1−) 1.5−2 (−2.5) cm long; mature hypanthium 10−14 mm long, hairs glandular; marginal nerves of leaf abaxial surface absent or obscure and discontinuous; anthers 7−10 mm long	*Rhexia cubensis* Fig. 168
–	Petals white to pink, (0.7−) 0.9−1.4 cm long; mature hypanthium 6−10 mm long, glabrous or hairs glandular; marginal nerves of leaf abaxial surface prominent; anthers 5−8 mm long	Rhexia mariana var. exalbida Fig. 169
4	Four stem faces at mid–stem noticeably unequal, one pair of opposite faces broader, convex, darker green, the narrower pair concave or flat, pale, arrangement of broader and narrower faces alternating at each internode up the stem, angles at midstem not winged	*Rhexia nashii* Fig. 170
–	Four stem faces at mid–stem about equal, almost flat, angles at midstem conspicuously winged	*Rhexia virginica* Fig. 171

Key adapted from Radford et al. (1968) and Weakley (2012).

### Nymphaeaceae

**Table:** 

1	Perianth globose at anthesis, 2–5 cm diam.; margin of stigmatic disk crenate to dentate; leaves linear to lanceolate 15−30 (−50) × 5−10 (−11.5) cm, green both adaxially and abaxially, venation essentially pinnate, often of two types, submersed leaves (when present) thinner in texture than floating or emersed leaves, 60–90% of surface area of floating or emersed leaves with vasculature derived from the midrib; sepals 6, green to yellow, petaloid; petals inconspicuous, yellow, stamen-like, shorter than the sepals; rhizome with triangular or winged leaf scars	*Nuphar sagittifolia* Fig. 175
–	Perianth spreading at anthesis, 4–20 cm diam.; margin of stigmatic disk with prominent, distinct, upwardly incurved appendages; leaves ovate to orbiculate (5−) 10−40 × (5−) 10−40 cm, green adaxially and deep reddish-purple abaxially, venation essentially palmate, of one type, floating, 25–40% of surface area with vasculature derived from the midrib; sepals 4, greenish or reddish tinged, not petaloid; petals showy, white to pink, distinctly longer than the sepals; rhizomes with circular leaf scars	Nymphaea odorata var. odorata Fig. 176

Key adapted from Godfrey and Wooten (1981) and Weakley (2012).

### Nyssaceae

**Table:** 

1	Petioles of mature leaves 3−6 cm long; mature leaf blades exceeding 10 cm long, margins with a few irregular teeth; drupes ≥ 20 mm long	*Nyssa aquatica* Fig. 177
–	Petioles of mature leaves < 3 cm long; mature leaves ≤ 10 cm long, margins lacking irregular teeth; drupes 10−15 mm long	*Nyssa biflora* Fig. 178

Key adapted from Godfrey and Wooten (1981) and Weakley (2012).

### Onagraceae

**Table:** 

1	Leaves opposite, oblanceolate to elliptic, 8−20 mm; pedicels conspicuous, 5−16 mm long; petals 4, 4−5 mm long, slightly larger than the calyx segments; capsule obconical, 6−8 mm long, slightly quadrangular in cross-section, curved; seed coat with rectangular reticulations; plants pubescent with short hooked hairs; plant creeping and rooting at the nodes	*Ludwigia brevipes* Fig. 180
–	Leaves alternate, lanceolate to linear-lanceolate, 3−10 cm; pedicels 0−1 mm long; petals lacking; capsules subglobose, 2.5−4.5 mm long, terete in cross-section or with broadly rounded lobes; seed coat with square reticulations, pentagonal or circular; plants glabrous to slightly pubescent, if pubescent, then hairs not hooked; plant erect and ascending, not rooting at the nodes	*Ludwigia sphaerocarpa* Fig. 181

Key adapted from Weakley (2012).

### Plantaginaceae

**Table:** 

1	Plant a true aquatic, forming extensive mats in shallow water; plant parts spicy aromatic when crushed; stems lax, fleshy, semi-succulent, pubescent; leaves of the flowering stem 55−28 × 7–15 mm, opposite, ovate to widely elliptic, with 3−7 palmate veins; inflorescence composed of a single axillary flower; corolla bluish-purple, 9–11 mm long, not spurred, orifice distinct	*Bacopa caroliniana* Fig. 182
–	Plant terrestrial, usually found at the upper margins of the high water mark in moist sandy soil; plant parts not aromatic when crushed; stems not lax, fleshy or semi-succulent, nor pubescent; leaves of the flowering stem 5–20 × 1−3.5 mm, alternate, linear < 3 veins; inflorescence a terminal raceme; corolla bluish-purple and white, 5–15 mm long (including the spur), spurred, orifice obscured	*Nuttallanthus canadensis* Fig. 183

Key adapted from Radford et al. (1968) and Weakley (2012).

### Rosaceae

**Table:** 

1	Leaves simple; fruit a pome	2
–	Leaves compound; fruit achenes enclosed within a hip or an aggregate of drupelets	4
2	Inflorescence corymbose; adaxial surface of leaves with dark glandular trichomes along the midrib, leaf margins finely serrate, teeth tipped with red glands; mature fruit red	*Aronia arbutifolia* Fig. 191
–	Inflorescence racemose; adaxial surface of leaves lacking glandular trichomes, leaf margins serrate, teeth tips lacking red glands; mature fruit blue to purple [*Amelanchier*]	3
3	Plant a small shrub or tree, 8–20 m tall, not rhizomatous; pedicels of varying lengths, the longest > 1 cm long; petals 6–12 mm long	*Amelanchier canadensis* Fig. 189
–	Plant a small shrub, 0.2–2.5 m tall, rhizomatous; pedicels nearly uniform in length, usually < 1 cm long; petals 5.9–7.7 mm long	*Amelanchier obovalis* Fig. 190
4	Leaves odd-pinnately compound, leaflet margins usually crenulate to serrulate; fruit a hip, developing from an urceolate hypanthium, enclosing the ovaries and achenes except for the apical orifice	*Rosa palustris* Fig. 192
–	Leaves palmately compound, leaflet margins usually serrate to doubly serrate; fruit an aggregate of drupelets, developing from a flatish to hemispheric hypanthium, ovaries and druplets exposed, not borne inside a hypanthium	*Rubus pensilvanicus* Fig. 193

Key adapted from Godfrey and Wooten (1981) and Weakley (2012).

### Rubiaceae

**Table:** 

1	Plant woody, a shrub or small tree; inflorescence in dense globose heads; corolla narrowly infundibuliform, white, lobes 4, shorter than the tube; length of exerted style ca. 3× or more the length of a corolla lobe	*Cephalanthus occidentalis* Fig. 194
–	Plant herbaceous; inflorescence of solitary or few-flowered, axillary cymes; corolla salverform to subcampanulate, white, lobes 3−4; length of exerted style ca. 1× the length of a corolla lobe or less	2
2	Plant pubescent to glabrous, erect or spreading; stem often with a reddish tinge; leaves opposite, elliptic-lanceolate to oblanceolate, 2−7 cm × 4−12 mm; flowers sessile, borne in leaf axils, 1 (−2) per axil; corolla salverform, 7−9 mm long, lobes 4, 3−4 mm long, inner surface pubescent; fruit oblong-ellipsoid, pubescent, 3−5 mm wide, prominently ridged	*Diodia virginiana* Fig. 195
–	Plant glabrous, erect or spreading; stem lacking a reddish tinge; leaves whorled, 4 per node, linear-obovate, 8−20 mm × 1.5−4 mm; flowers in branched terminal and axillary cymes, 1−3-flowered; corolla subcampanulate, corolla lobes 3−4, < 3 mm long, inner surface glabrous; fruit orbicular, glabrous, 2.5−4 mm wide, smooth, not ridged	Galium obtusum var. obtusum Fig. 196

Key adapted from Godfrey and Wooten (1981) and Weakley (2012).

### Salicaceae

**Table:** 

1	Buds scales imbricate; leaf blades ovate, < 3 × as long as broad, bases truncate to broadly rounded, slightly cordate; inflorescences pendulous; stamens 5−80	*Populus heterophylla* Fig. 197
–	Bud scales 1; leaf blades lanceolate, > 3 × as long as broad, bases cuneate, not cordate; inflorescences erect or spreading; stamens 1−9	2
2	Mature leaf undersides glaucous, glabrous to sparsely pubescent, blades (4−) average 7.5 (−13) × as long as wide; stipules usually prominent and persisting, to 15 mm long	*Salix caroliniana* Fig. 198
–	Mature leaf undersides green, not glaucous, glabrous, blades (4−) average 9 (−16) × as long as wide; stipules not persisting, to 12 mm long	*Salix nigra* Fig. 199

Key adapted from Godfrey and Wooten (1981), Argus et al. (2010), and Weakley (2012).

### Sapindaceae

**Table:** 

1	Leaves palmately compound; fruit a capsule	Aesculus pavia var. pavia Fig. 203
–	Leaves simple; fruit a schizocarp composed of two 1-seeded samaras	2
2	Leaves (3−) 5 (−9) lobed, central lobe 4−8 cm long, upper two lateral lobes 2−5 cm long, bases generally cordate	Acer rubrum var. rubrum Fig. 201
–	Leaves (0−) 3 (−5) lobed, central lobe 1−5 cm long, upper two lateral lobes (if leaves more than 3-lobed) 0.5−2 (−3) cm long, bases cuneate to rounded to subcordate	Acer rubrum var. trilobum Fig. 202

Key adapted from Radford et al. (1968) and Weakley (2012).

### Vitaceae

**Table:** 

1	Leaves simple, leaf margins prominently dentate-serrate throughout, bases cordate; tendrils unbranched, lacking adhesive pads	*Muscadinia rotundifolia* Fig. 207
–	Leaves palmately compound, leaflet margins coarsely serrate above the middle, entire below middle, bases cuneate; tendrils branched, bearing adhesive pads at the tips	*Parthenocissus quinquefolia* Fig. 208

Key adapted from Radford et al. (1968) and Weakley (2012).

## Supplementary Material

Supplementary material 1 Carolina bay lakes literature Data type: references
Brief description: List of citations regarding Carolina bay lakes
File: oo_72560.doc Nathan Howell

Supplementary material 2 Floras, manuals, guides, and broader floristic works on site-specific and broad-scale aquatic/wetland habitats of the eastern United States. Data type: references
Brief description: List of some floras, manuals, guides, and broader floristic works aquatic/wetland habitats of the eastern United States that may be of interest to readers.
File: oo_72561.doc Nathan Howell

Supplementary material 3 Sample taxon entry with brief descriptions of working parts. Data type: taxon entry components and definitions
File: oo_72562.doc Nathan Howell

Supplementary material 4 Literature highlighting the ecological, biological, and cultural importance of Carolina bays. Data type: references
File: oo_72563.doc Nathan Howell

Supplementary material 5 Suggested collection methods for problematic aquatic taxa and sampling methods of floating bog communities. Data type: collection suggestions
File: oo_72564.doc Nathan Howell

Supplementary material 6 Checklist of the littoral zone vascular flora of unaltered Carolina bay lake shorelines (i.e., Bakers Lake, Bay Tree Lake, Horseshoe Lake, Jones Lake, Lake Waccamaw, Little Singletary Lake, Salters Lake, Singletary Lake). Data type: occurrences
Brief description: Taxa are organized by major plant groups (i.e., pteridophytes, gymnosperms, basal angiosperms, magnoliids, monocotyledons, and eudicotyledons), then alphabetically by family, genus, and species. Parentheses around a taxon indicate that it is not vouchered (i.e., it has been reported by state agencies or has been observed by the first author, but has not been collected as a voucher specimen; see text for details). For taxa collected from Carolina bay lake littoral zones *by the present author*, abundance estimates sensu Palmer et al. (1995) are provided. Abundance estimates in this checklist reflect the abundance in which the taxa occur within each lake. Status and rank designations are also provided for rare taxa monitored by the NC Natural Heritage Program (Robinson and Finnegan 2014). The term “restricted” is used here only to indicate the presence of a taxon within a particular lake among all those surveyed and not in a global sense (e.g., a taxon here considered restricted to Lake Waccamaw has not been found in the other lakes surveyed, but may exist in other localities in the state or country). A = Abundant; F= Frequent; I=Infrequent; O = Occasional; R = Rare; = þ restricted to lake indicated; () = not vouchered (i.e., reported by state agencies or observed by the present author, but not collected as a voucher specimen; see text for details); H = taxon has been collected and vouchered in the past but not by the present author. BALA = Bakers Lake; BATR = Bay Tree Lake; HOLA = Horseshoe Lake; JOLA = Jones Lake; LAWA = Lake Waccamaw; LISI = Little Singletary; SALA = Salters Lake; SILA = Singletary Lake.
File: oo_72565.doc Nathan Howell

Supplementary material 7 Provisional checklist of the littoral zone vascular flora from White Lake based on historical vouchers, personal observations, and literature reviews. Data type: occurrences
Brief description: This checklist does not represent a complete inventory of this locality, but rather serves as a baseline for future research. Taxa are arranged by major groups (i.e., gymnosperms, magnoliids, monocotyledons, and eudicotyledons), then alphabetically by family, genus, and species. Basal angiosperms and pteridophytes were not represented by vouchers, observations, or reports and are therefore not included in the following checklist. Brackets around a taxon indicate that it is unvouchered (i.e., it has been reported by outside agencies or has been observed by the present author, but has not been collected). Status and rank designations are also provided for rare taxa monitored by the NC Natural Heritage Program (Robinson and Finnegan 2014).
File: oo_82955.doc Nathan Howell

Supplementary material 8 Climate data supporting Fig 15 (Walter climate diagrams) Data type: climate
Brief description: Monthly mean temperature and precipitation data for Bladen and Columbus County.
File: oo_75216.xlsx Nathan Howell

Supplementary material 9 Data supporting Fig 16 (Distribution of plant habit across all Carolina bay lakes) Data type: morphological
Brief description: Counts of the number of taxa in the categories of herb, tree/shrub, and vine for each Carolina bay lake flora.
File: oo_75217.xlsx Nathan Howell

Supplementary material 10 Data supporting Fig 17 (The thirteen most species-rich vascular plant families across all Carolina bay lakes) Data type: taxonomic
Brief description: Counts of the number of taxa in each of the thirteen most species-rich vascular plant families in each Carolina bay lake flora.
File: oo_75218.xlsx Nathan Howell

Supplementary material 11 Howell specimen collections Data type: occurrences
Brief description: Comma delimited file of occurrence data (DwC) for the specimens collected by the first author from Carolina Bay Lakes. Precise locality data has been redacted for species of conservation concern. Specimens are deposited at NCSC. Images are available through http://sernecportal.org.
File: oo_83035.csv Nathan Howell

## Figures and Tables

**Figure 15a.**
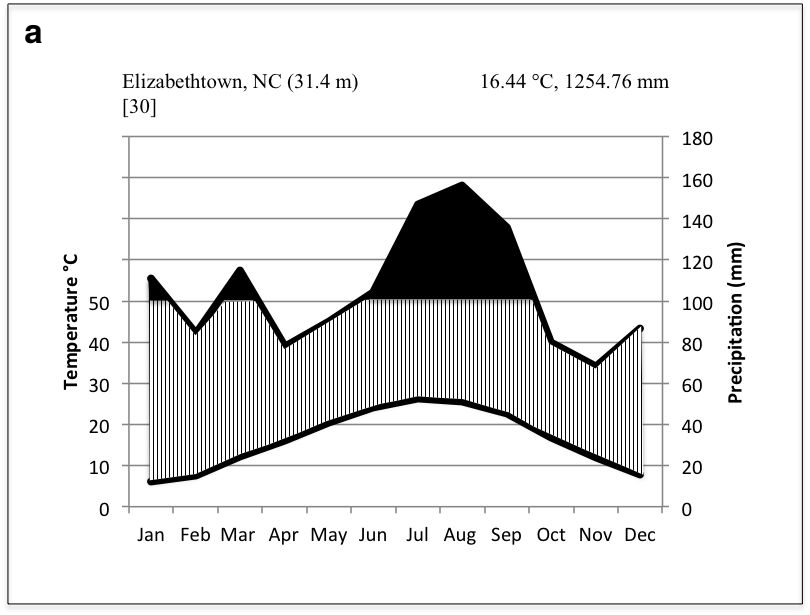


**Figure 15b.**
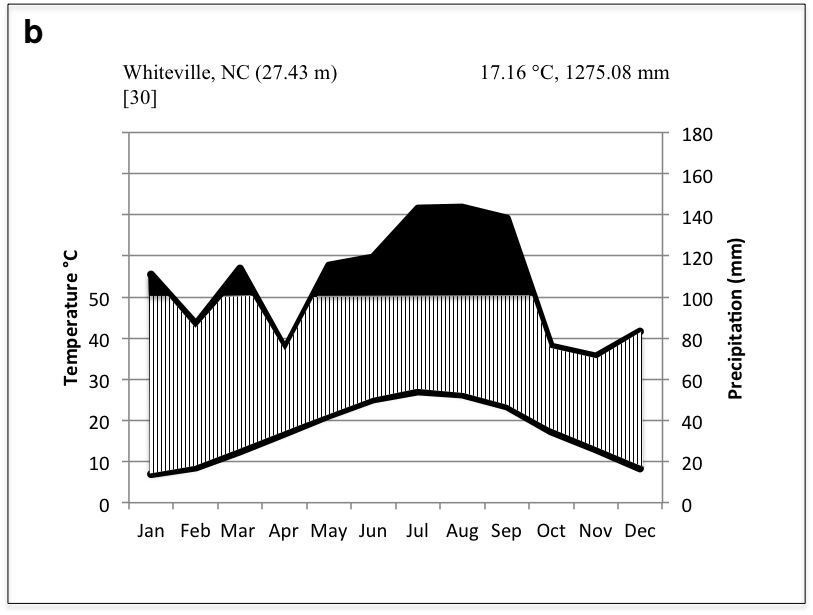


**Figure 18a.**
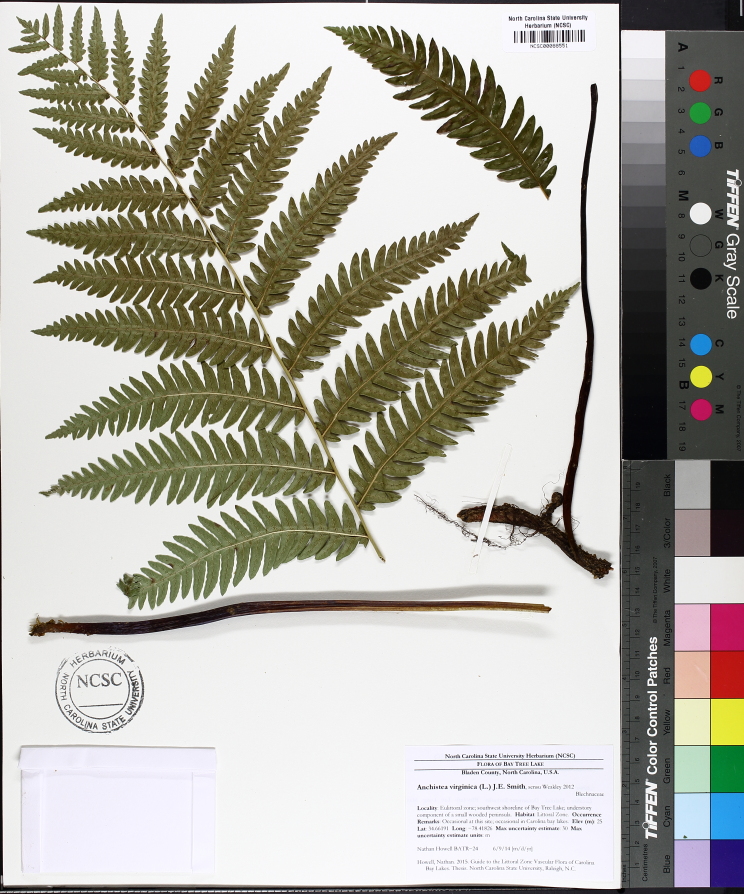
Specimen: *Howell BATR*−*24* (NCSC)

**Figure 18b.**
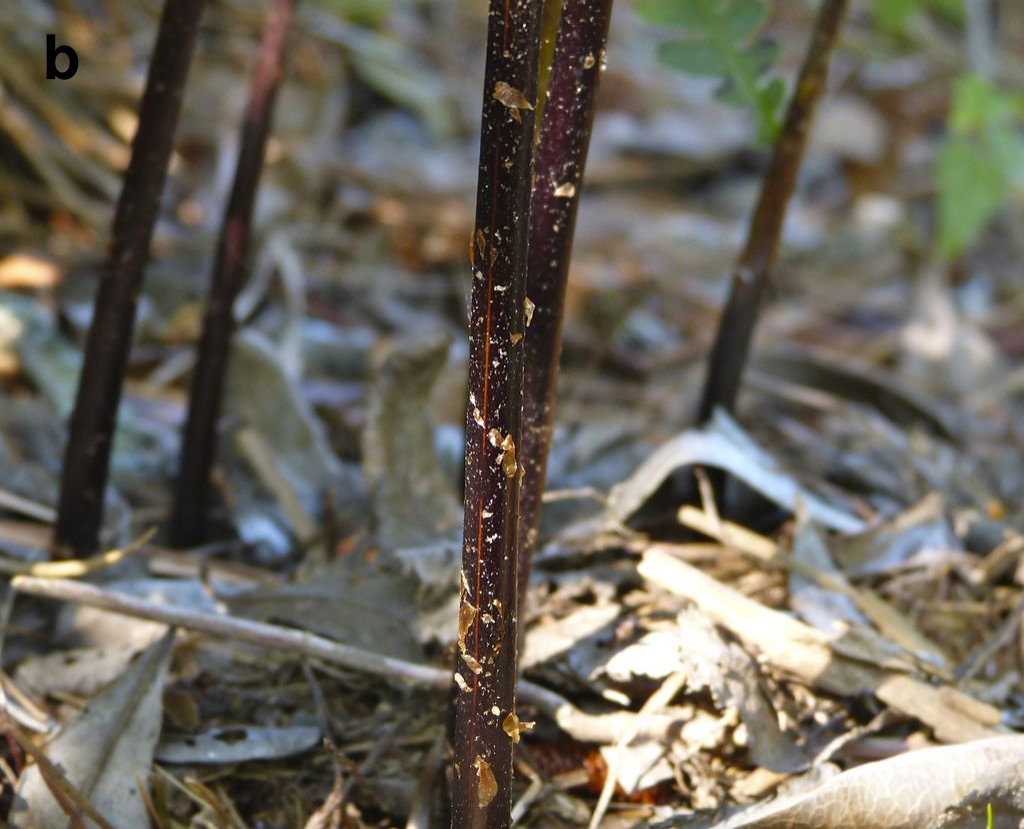
Stipe; note the dark purple coloration.

**Figure 18c.**
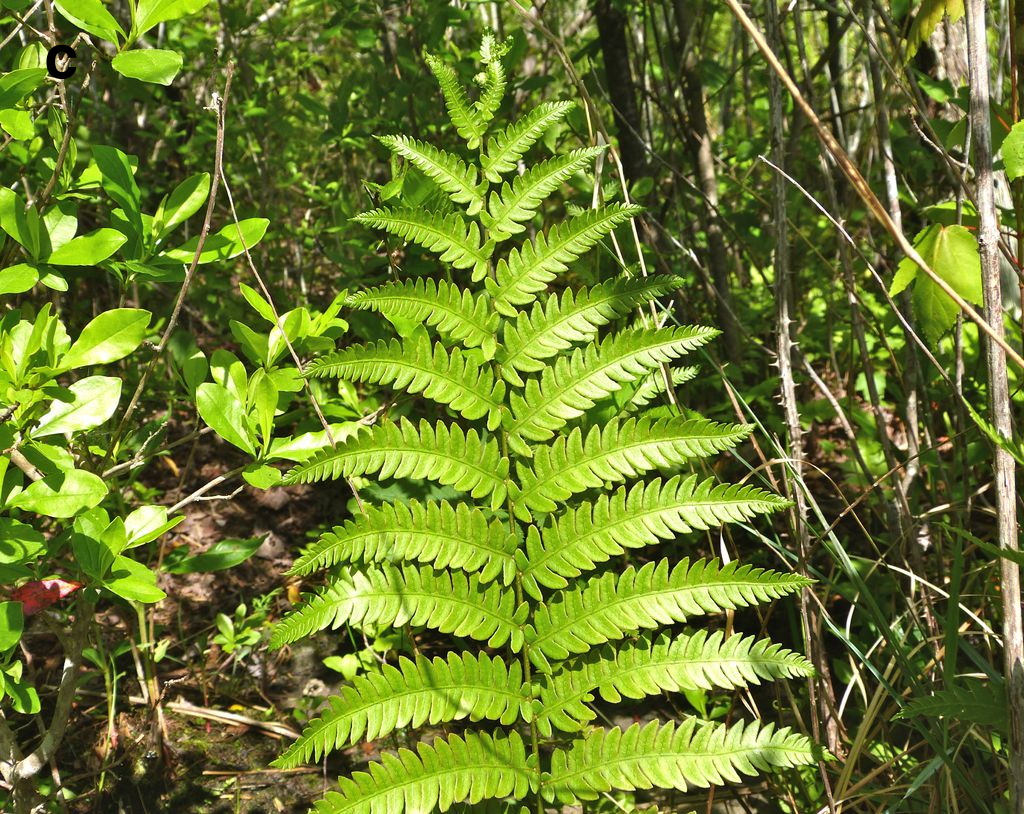
Mature frond

**Figure 18d.**
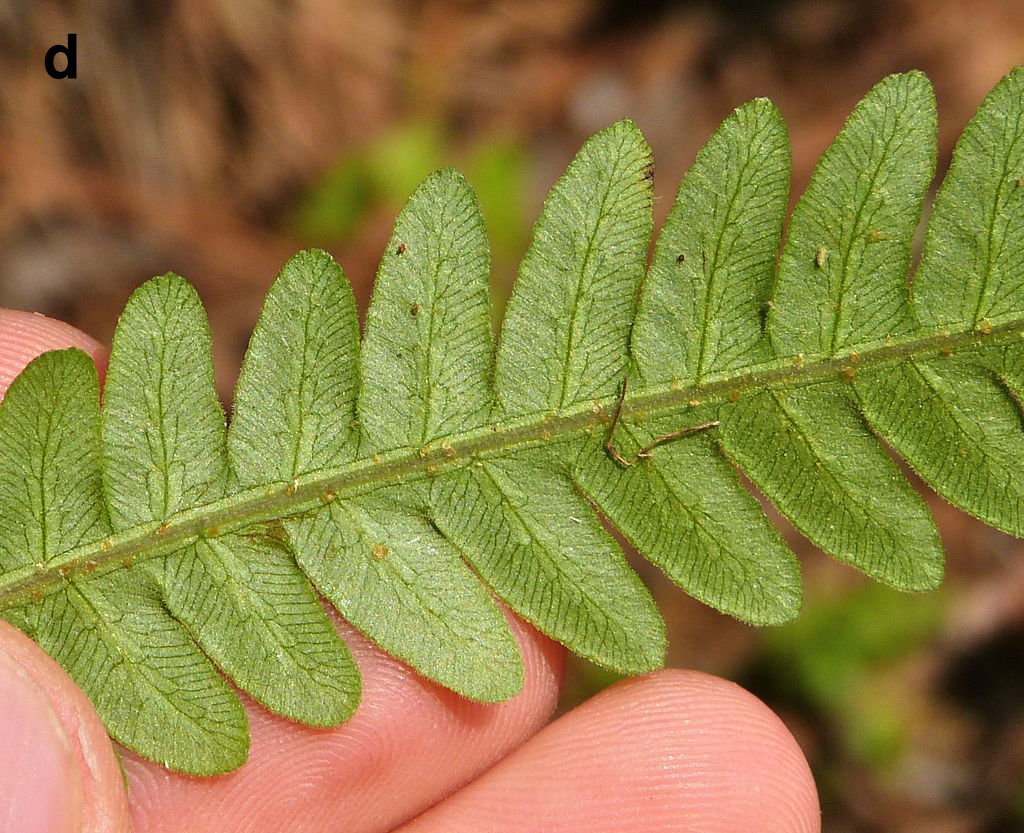
Pinna; note the chain-like venation pattern along the sides of leaflet midveins.

**Figure 19a.**
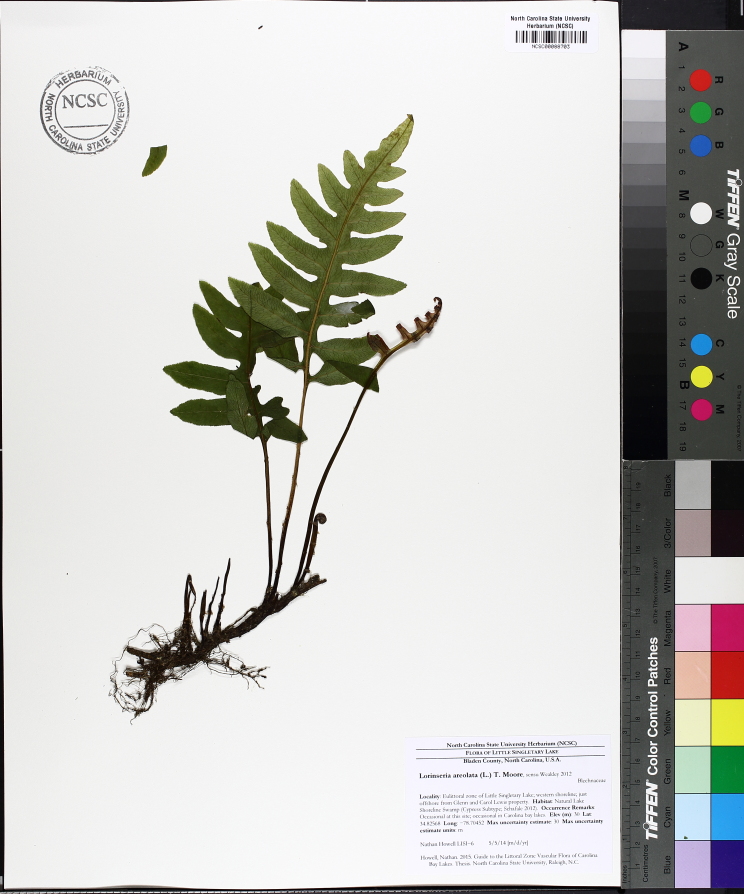
Specimen: *Howell LISI*–*6* (NCSC)

**Figure 19b.**
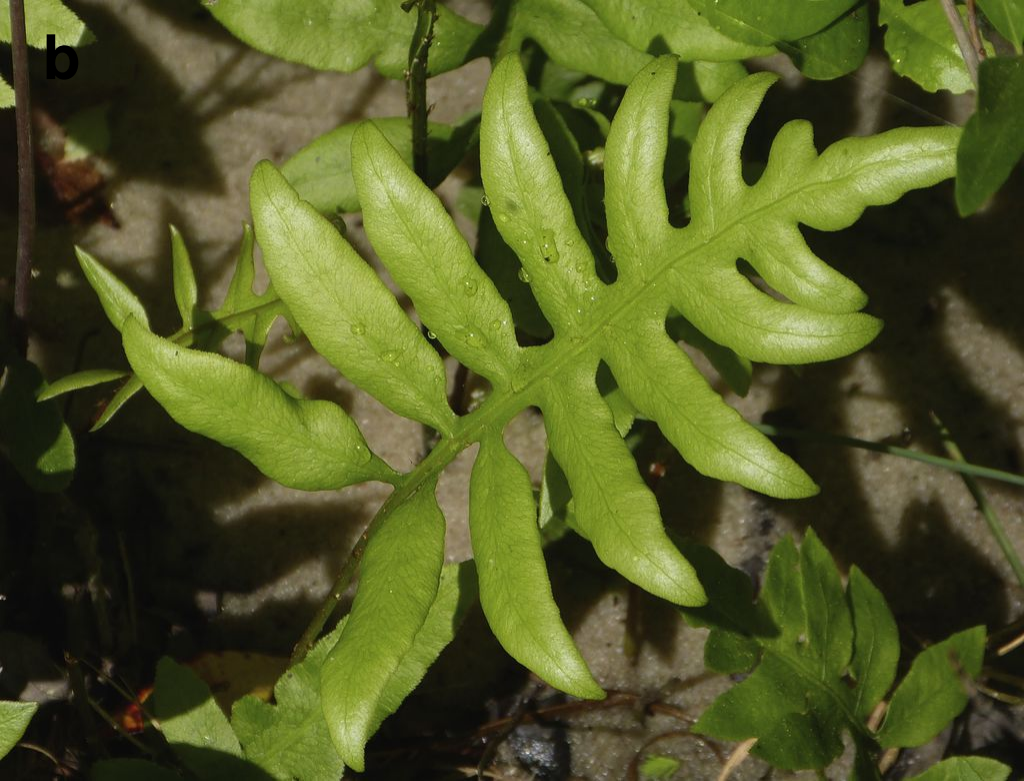
Mature frond

**Figure 19c.**
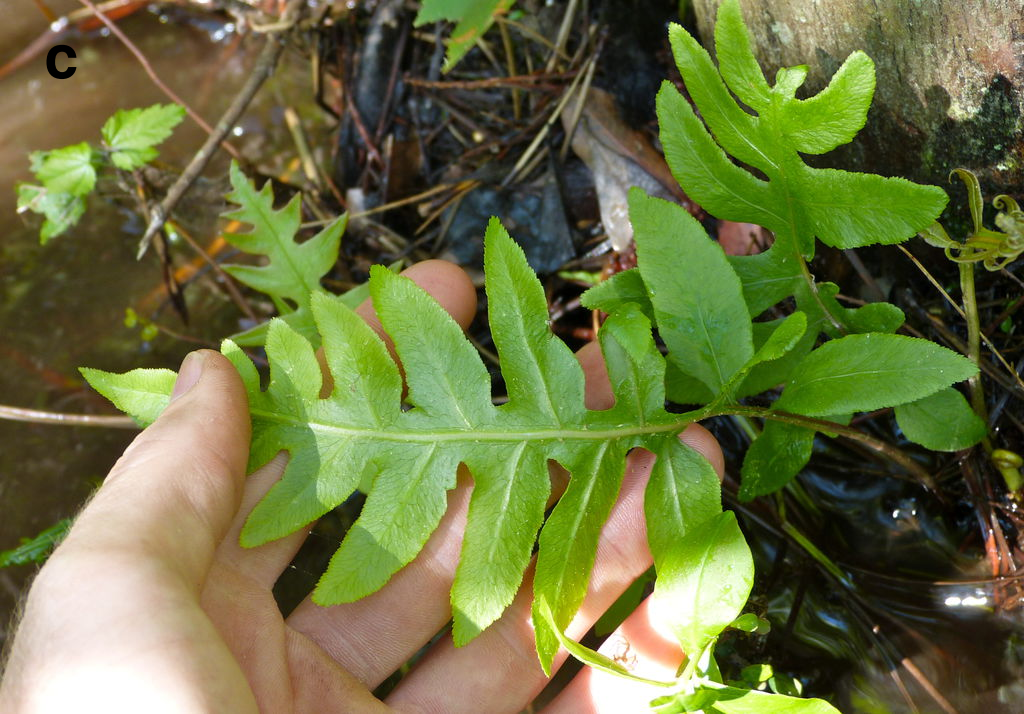
Frond underside

**Figure 19d.**
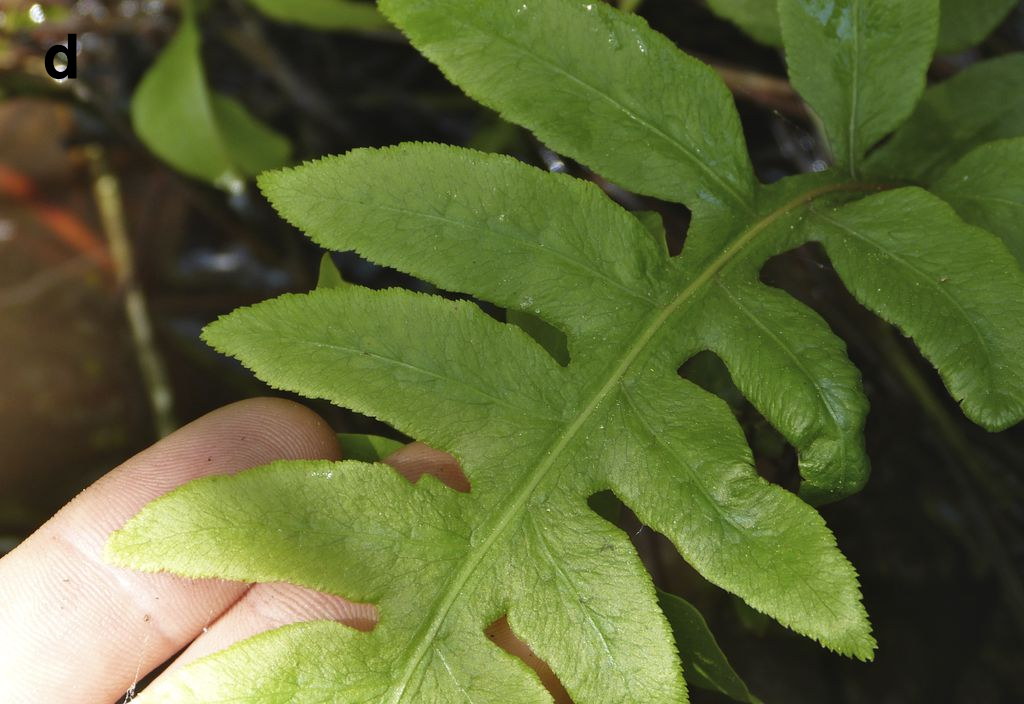
Pinnae

**Figure 21a.**
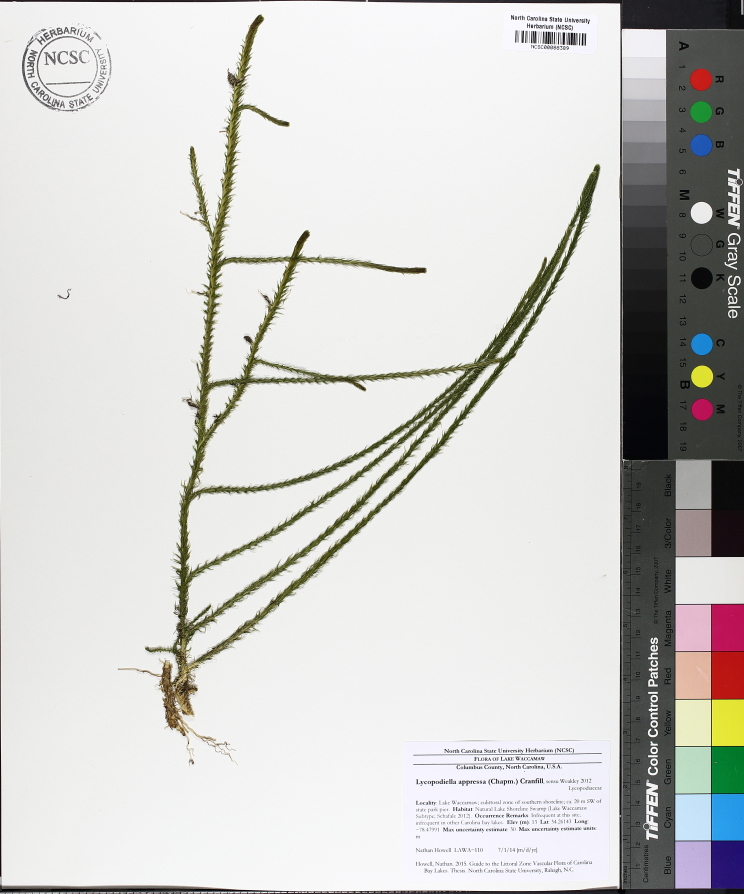
Specimen: *Howell LAWA*–*110* (NCSC)

**Figure 21b.**
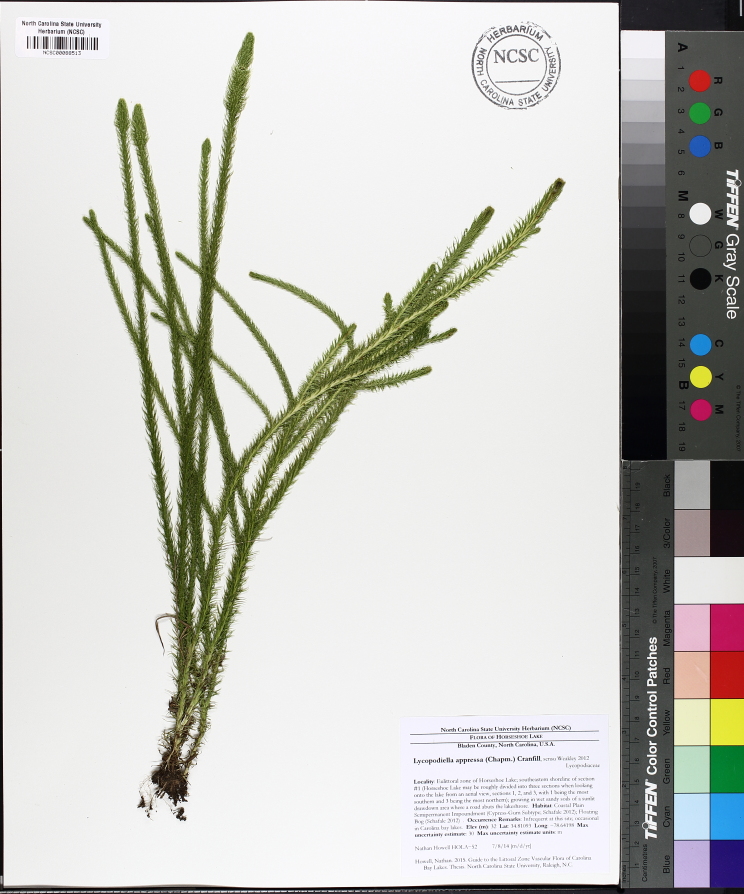
Specimen: *Howell HOLA*–52 (NCSC)

**Figure 21c.**
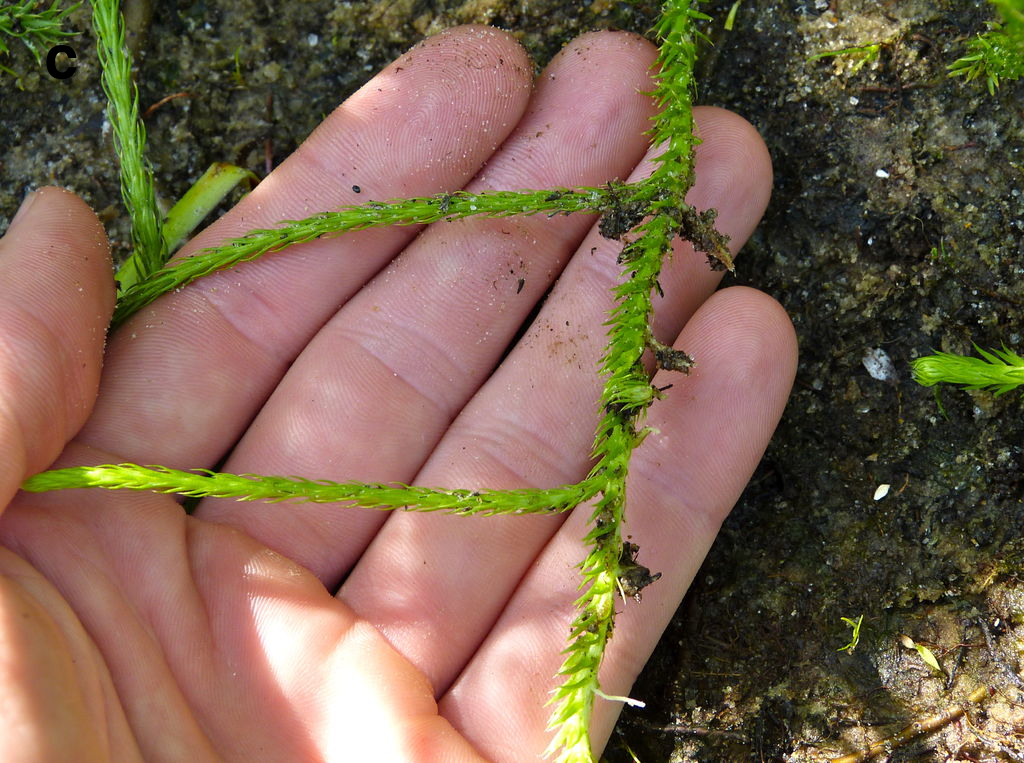
Rooting stems where making contact with soil

**Figure 21d.**
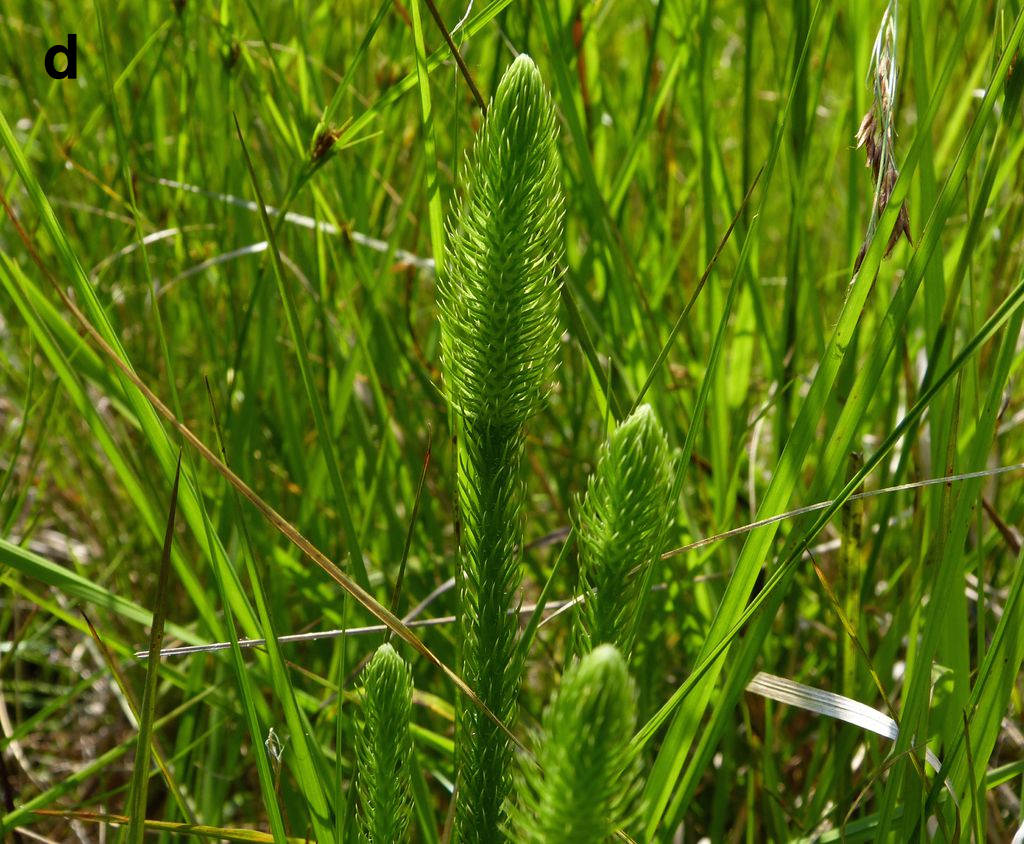
Terminal strobilus

**Figure 23a.**
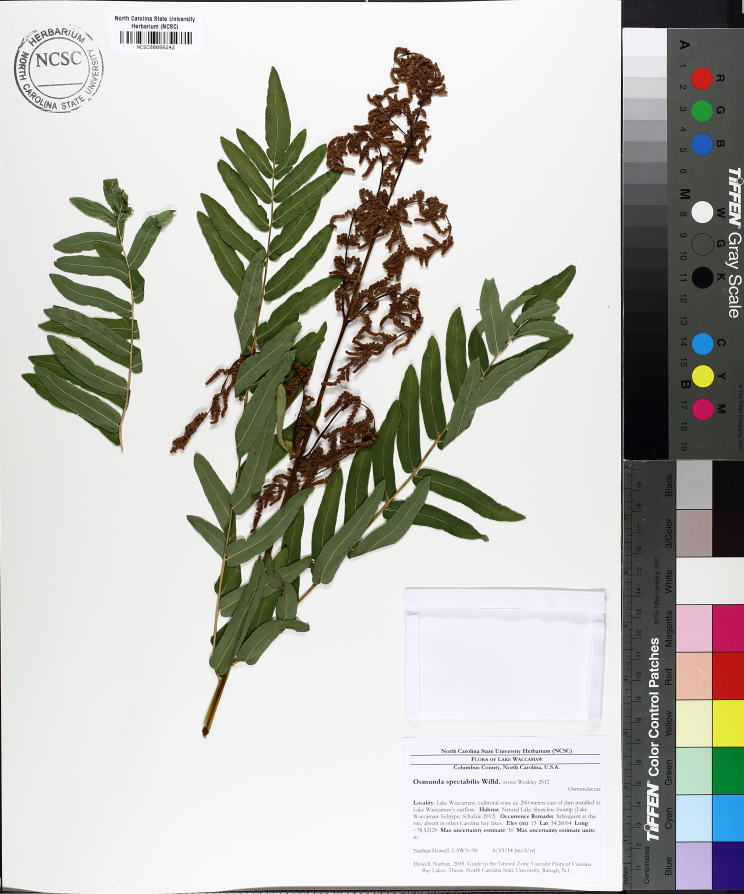
Specimen: *LAWA-90* (NCSC)

**Figure 23b.**
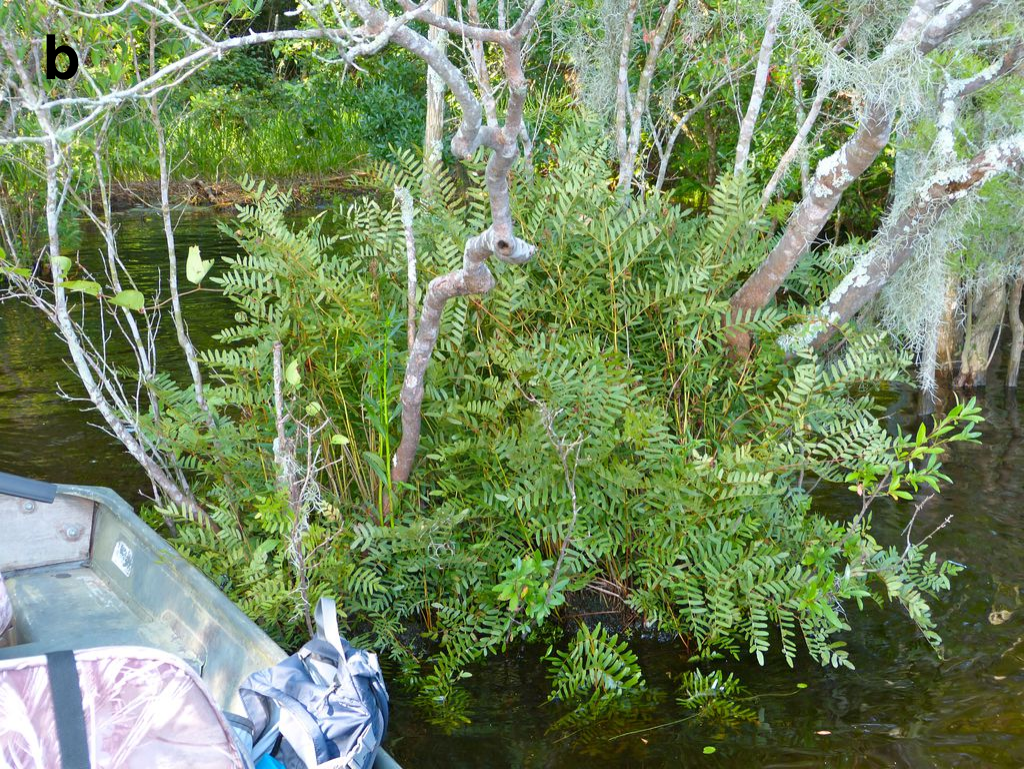
Habit

**Figure 23c.**
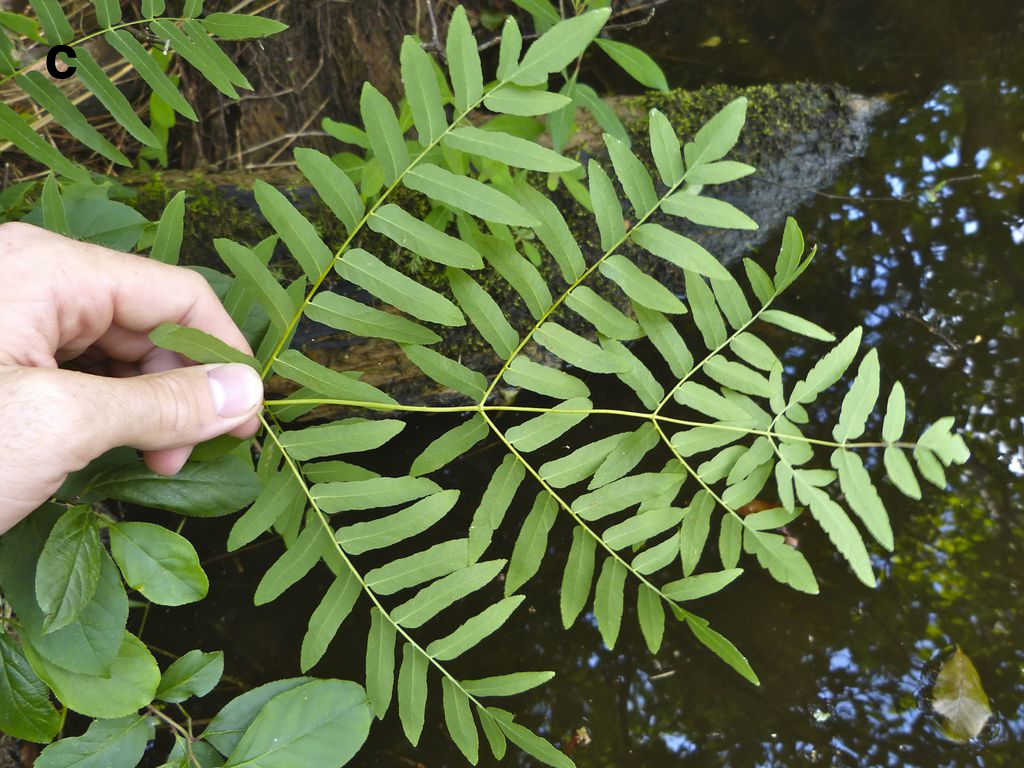
Sterile frond

**Figure 23d.**
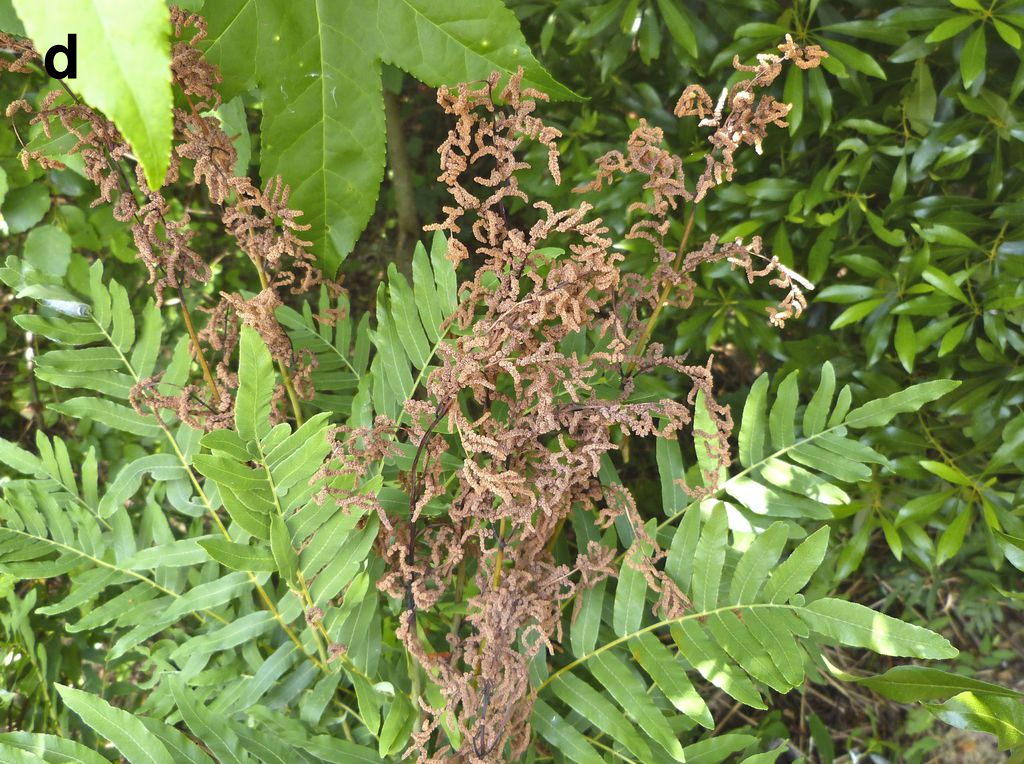
Sterile and fertile fronds

**Figure 24a.**
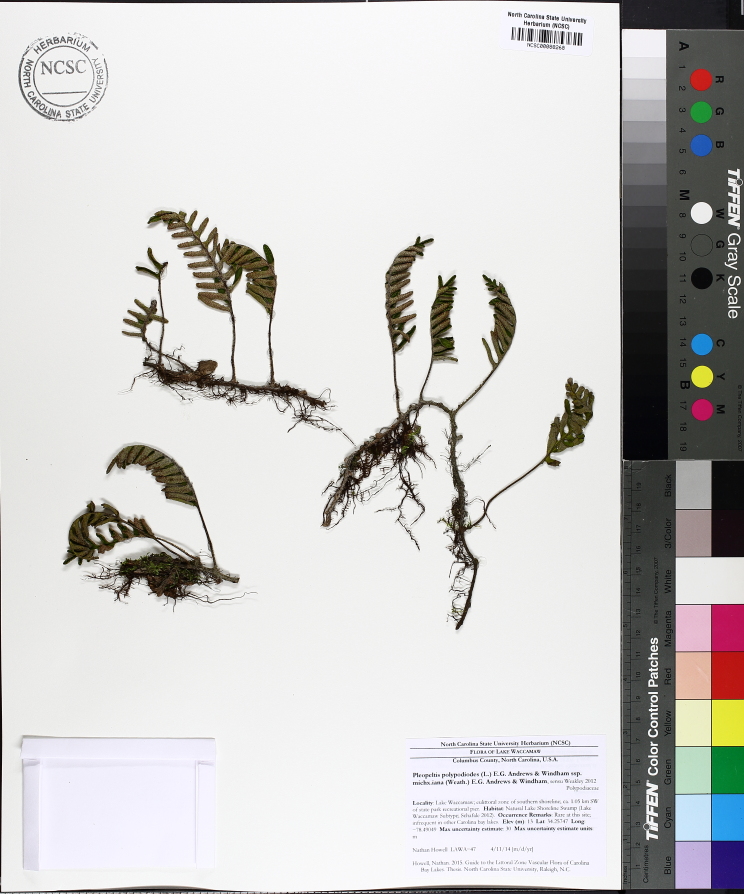
Specimen: *Howell LAWA-47* (NCSC)

**Figure 24b.**
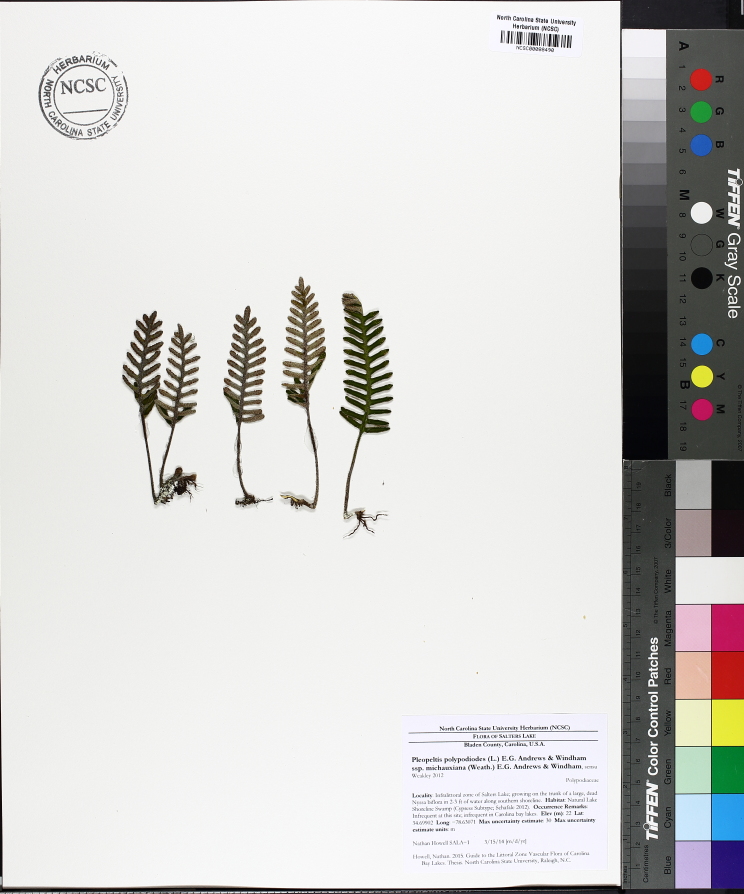
Specimen: *Howell SALA-1* (NCSC)

**Figure 24c.**
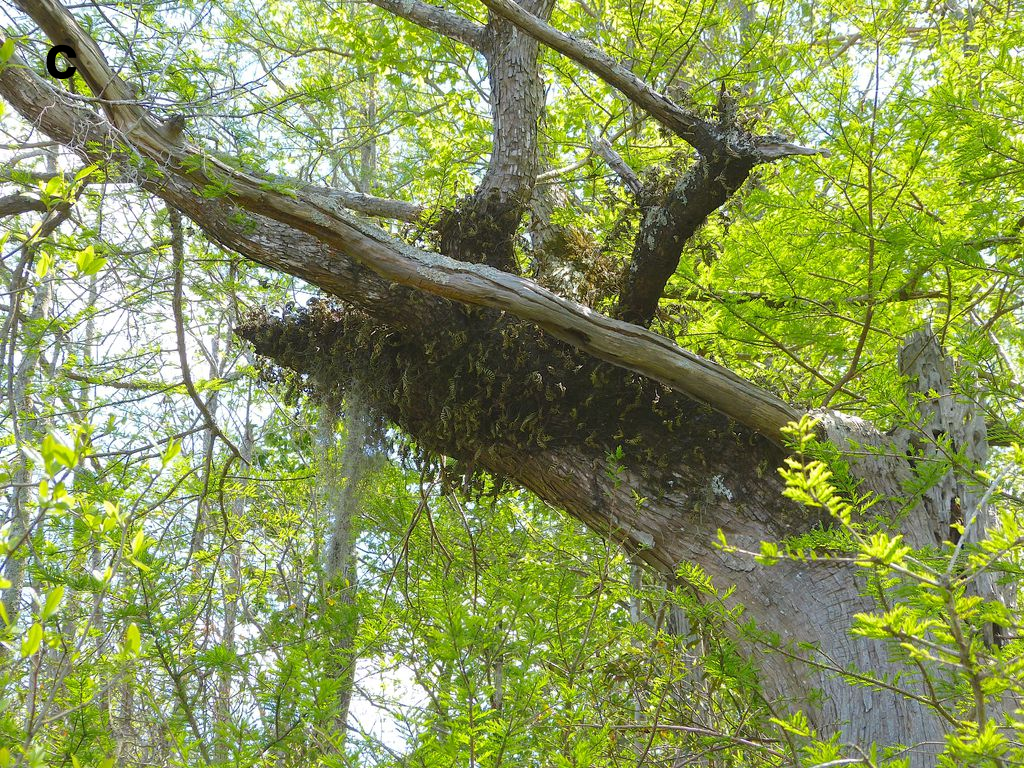
Habit

**Figure 24d.**
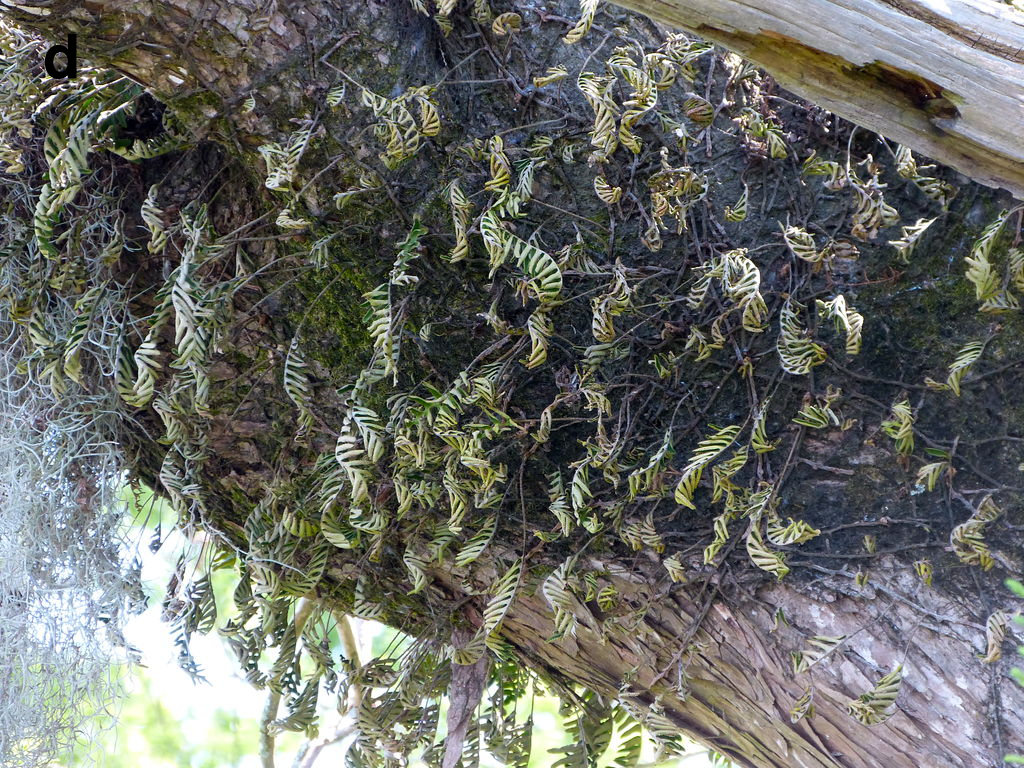
Habit

**Figure 25a.**
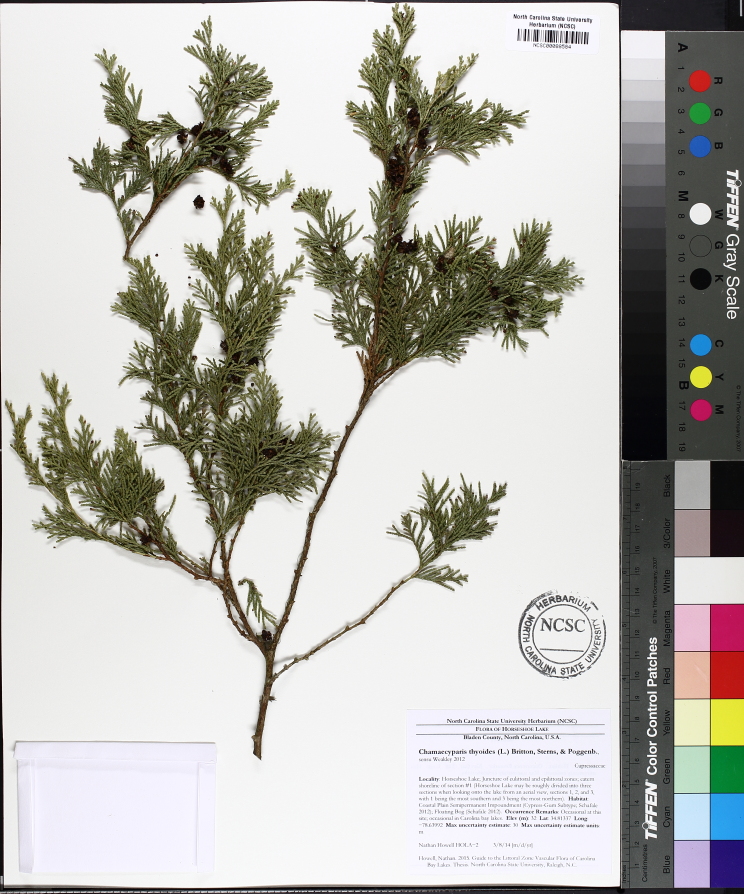
Specimen: *Howell HOLA-2* (NCSC)

**Figure 25b.**
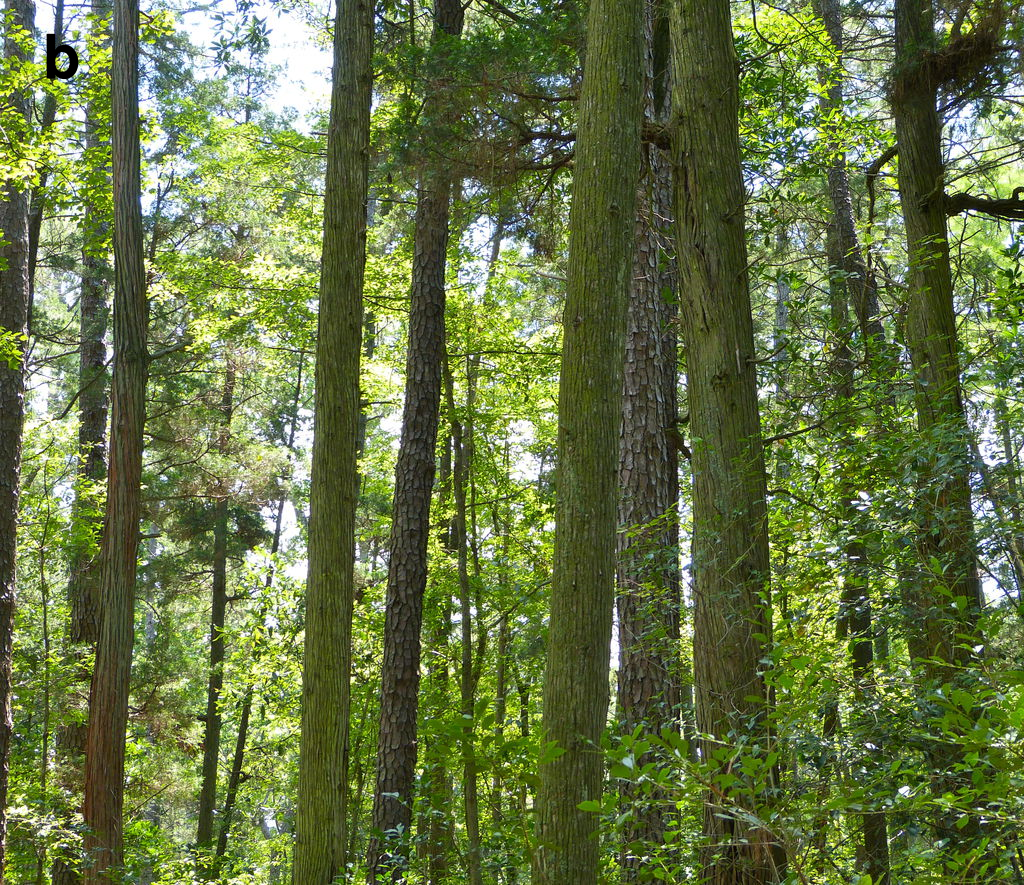
Bark

**Figure 25c.**
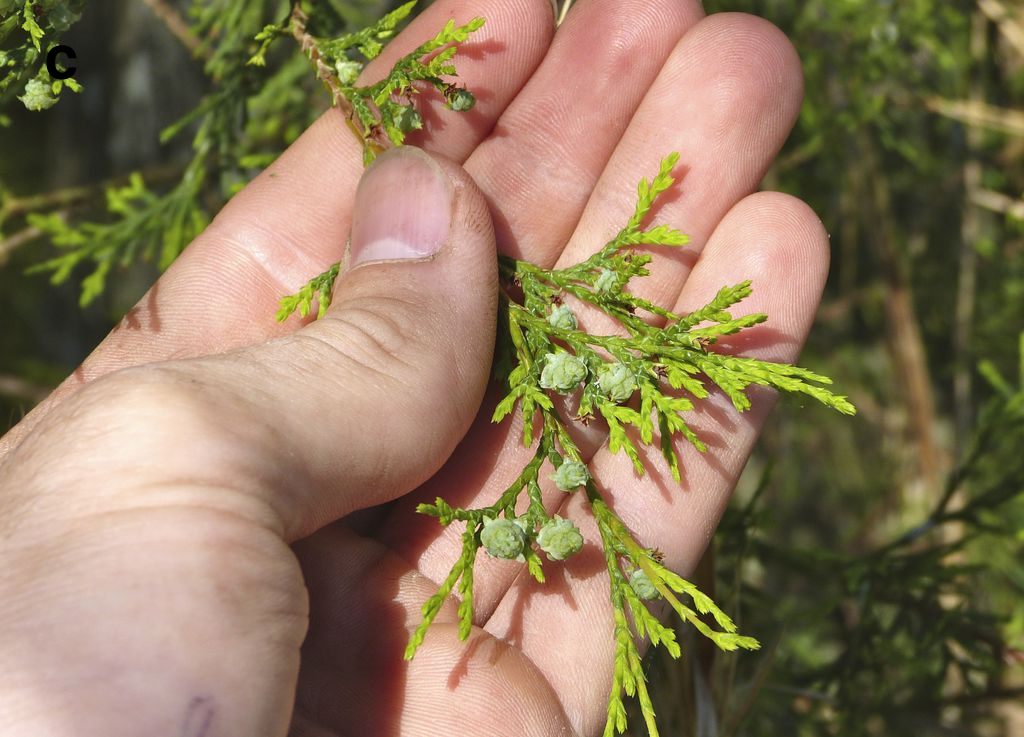
Leaves and developing seed cones

**Figure 25d.**
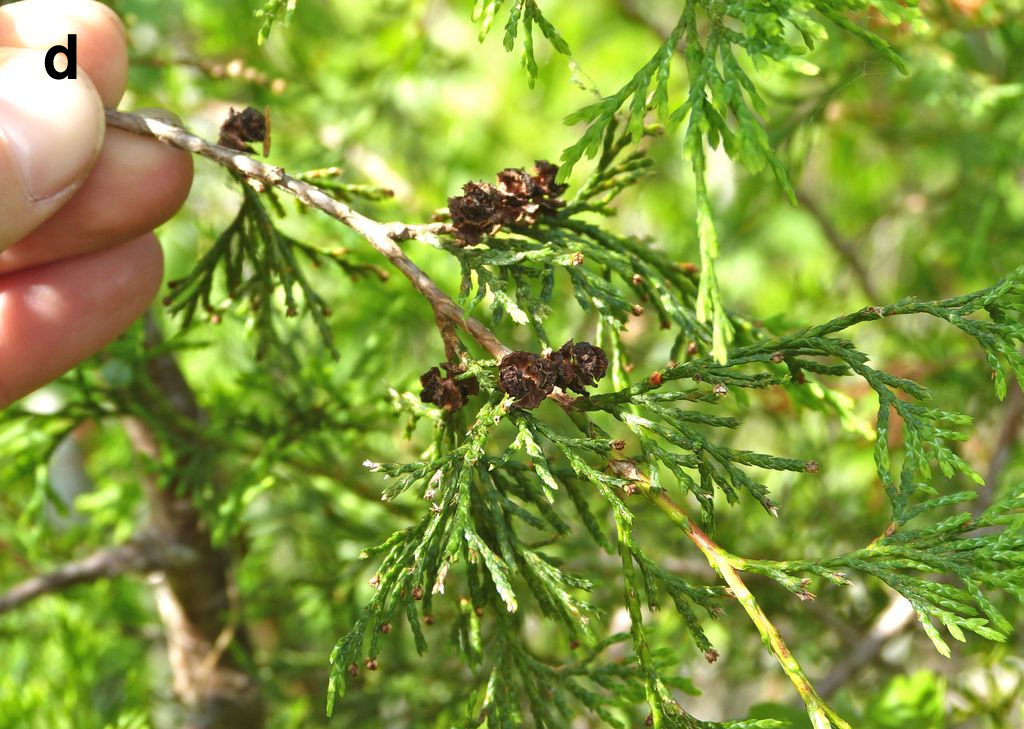
Leaves and mature seed cones

**Figure 26a.**
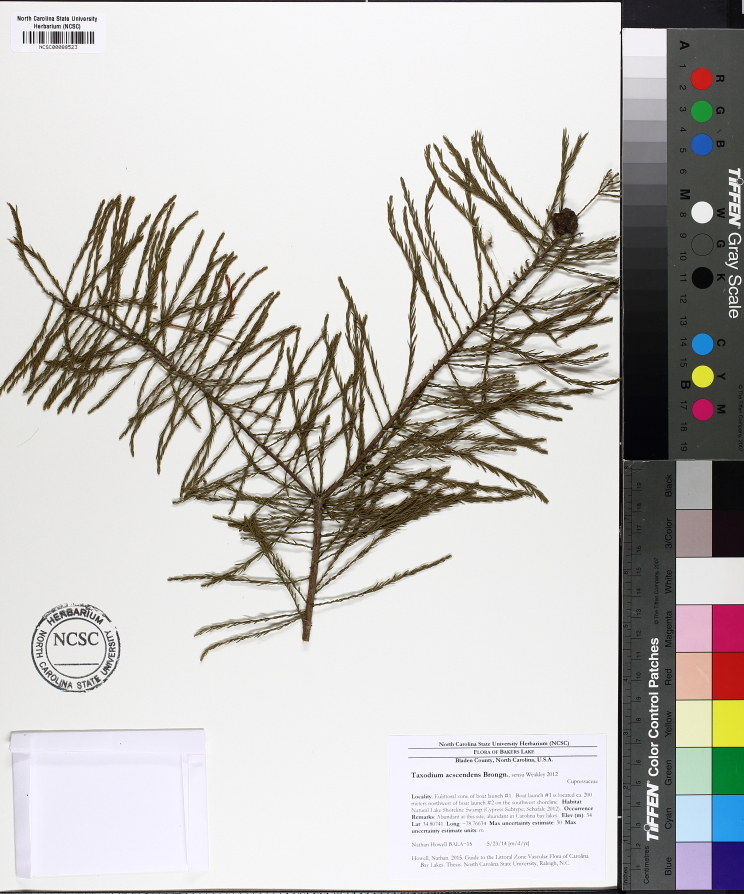
Specimen: *Howell BALA-15* (NCSC)

**Figure 26b.**
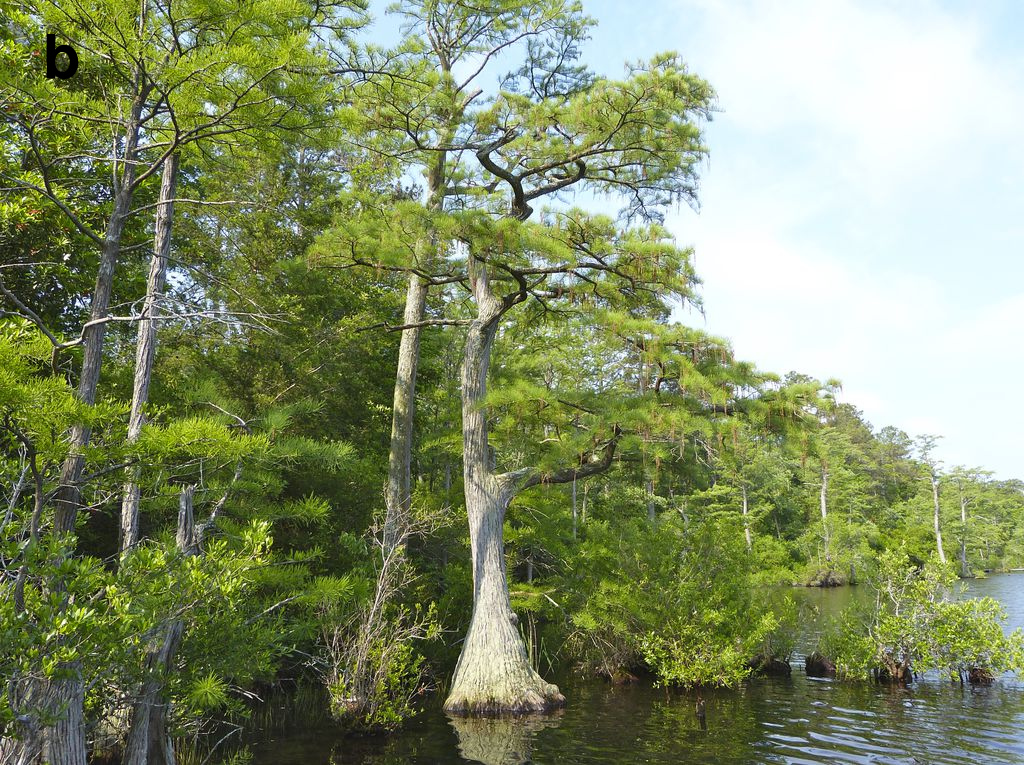
Habit

**Figure 26c.**
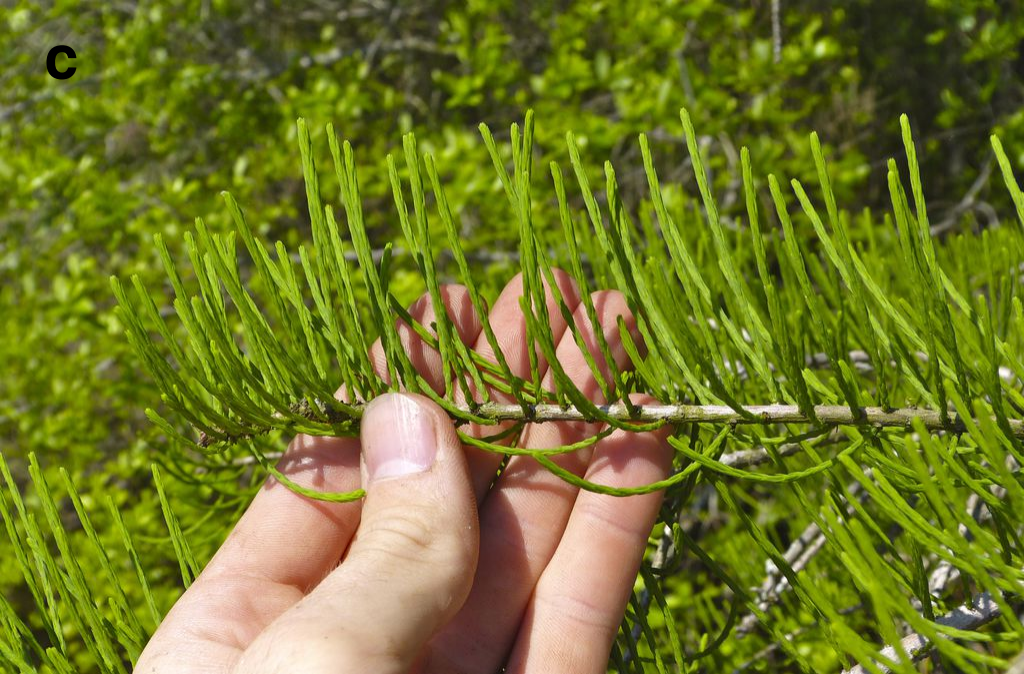
Leaves

**Figure 26d.**
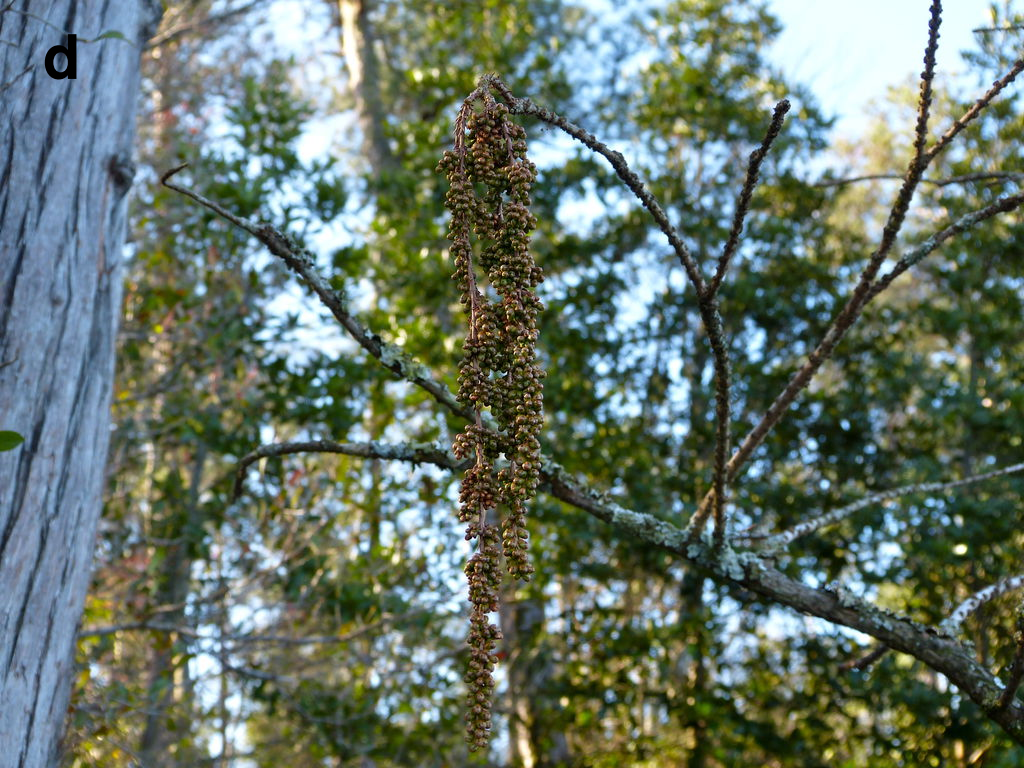
Pollen cones

**Figure 28a.**
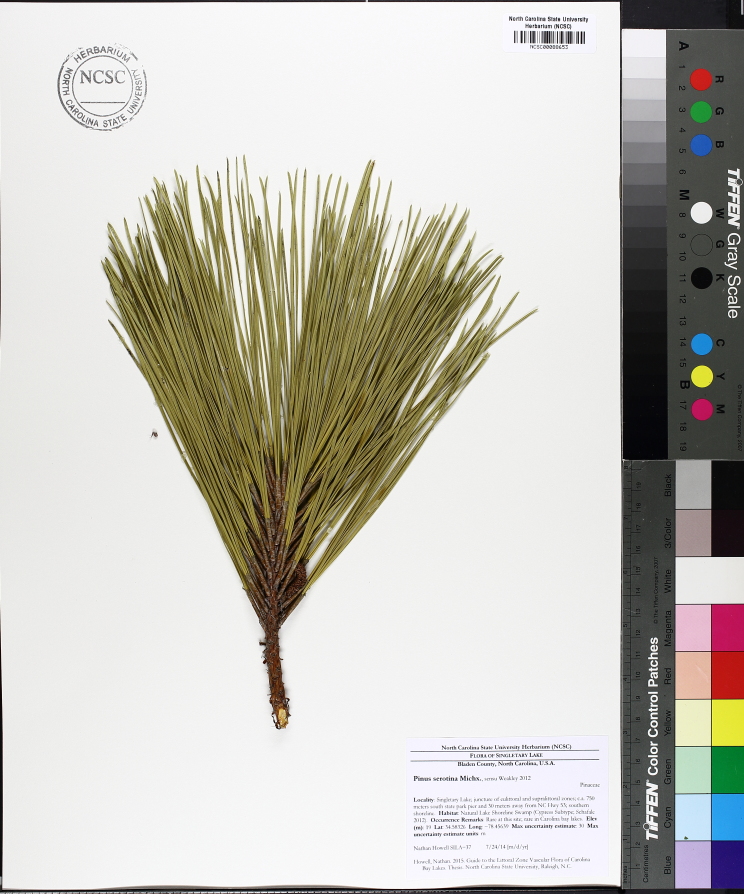
Specimen: *Howell SILA-37* (NCSC)

**Figure 28b.**
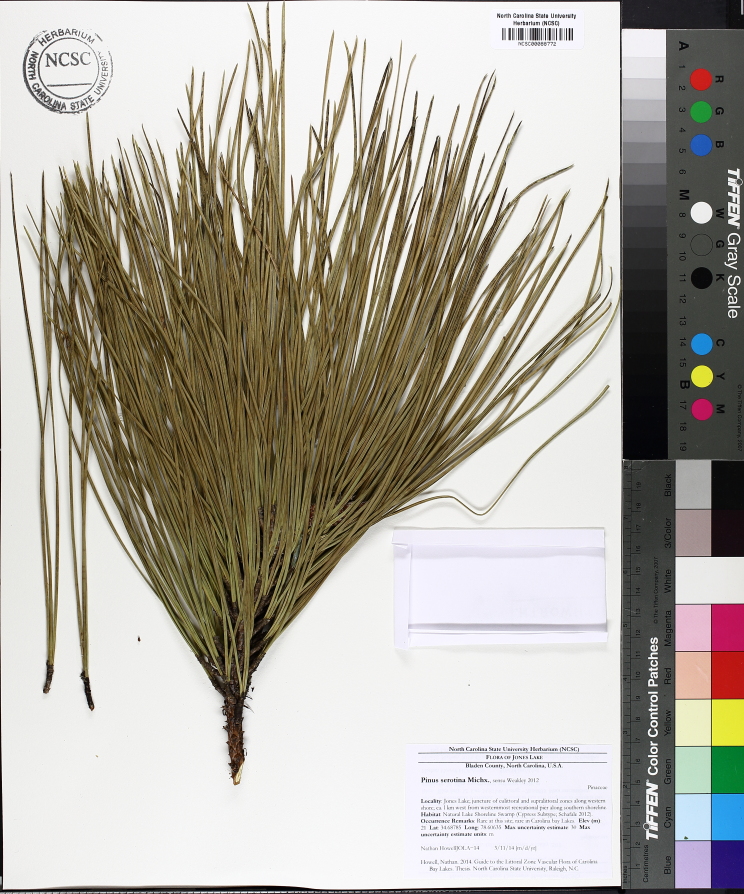
Specimen: *Howell JOLA-14* (NCSC)

**Figure 28c.**
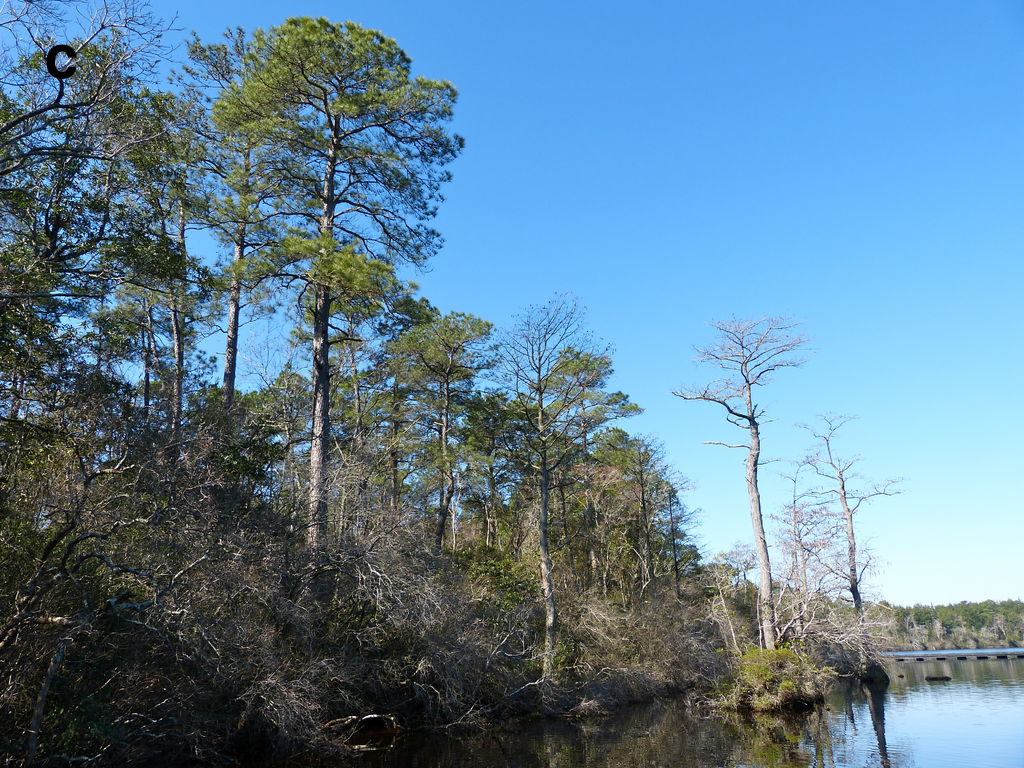
Habit

**Figure 28d.**
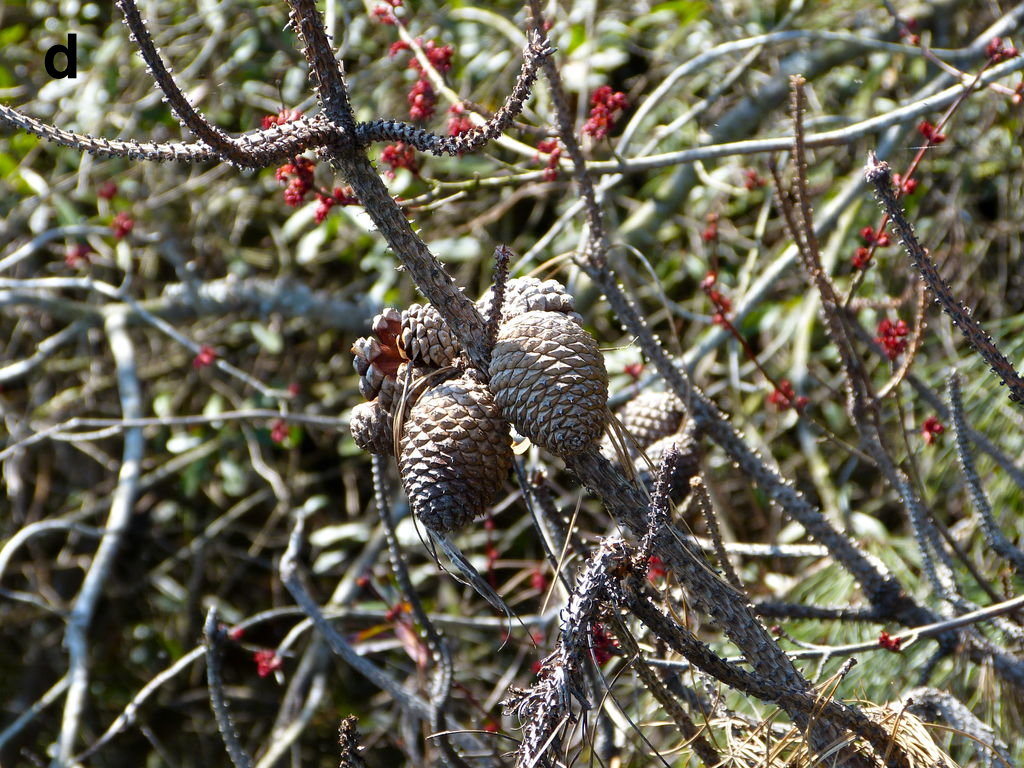
Mature seed cones

**Figure 29a.**
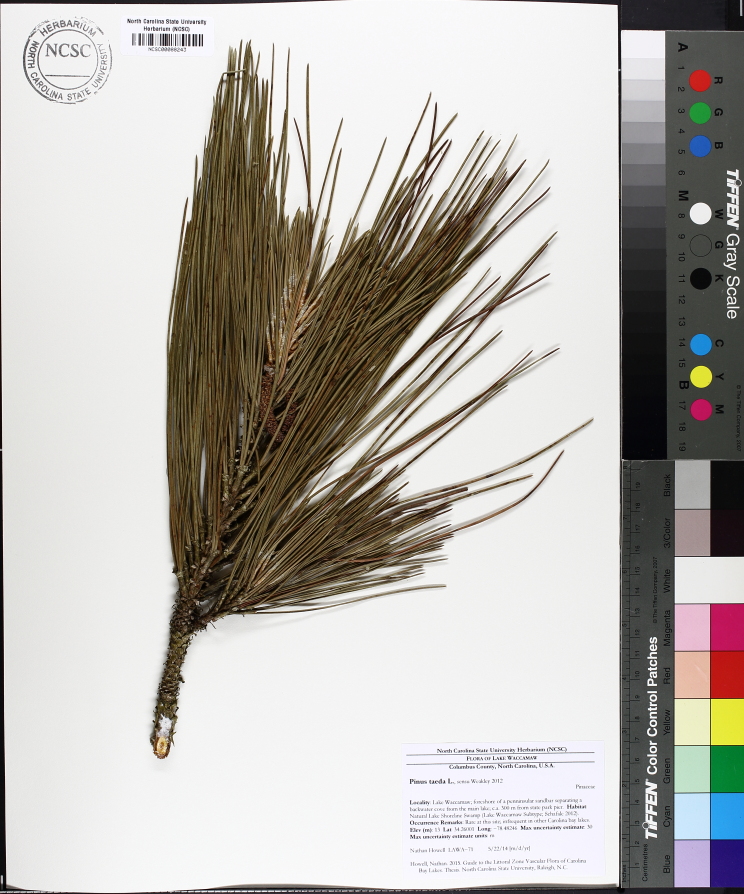
Specimen: *Howell LAWA-71* (NCSC)

**Figure 29b.**
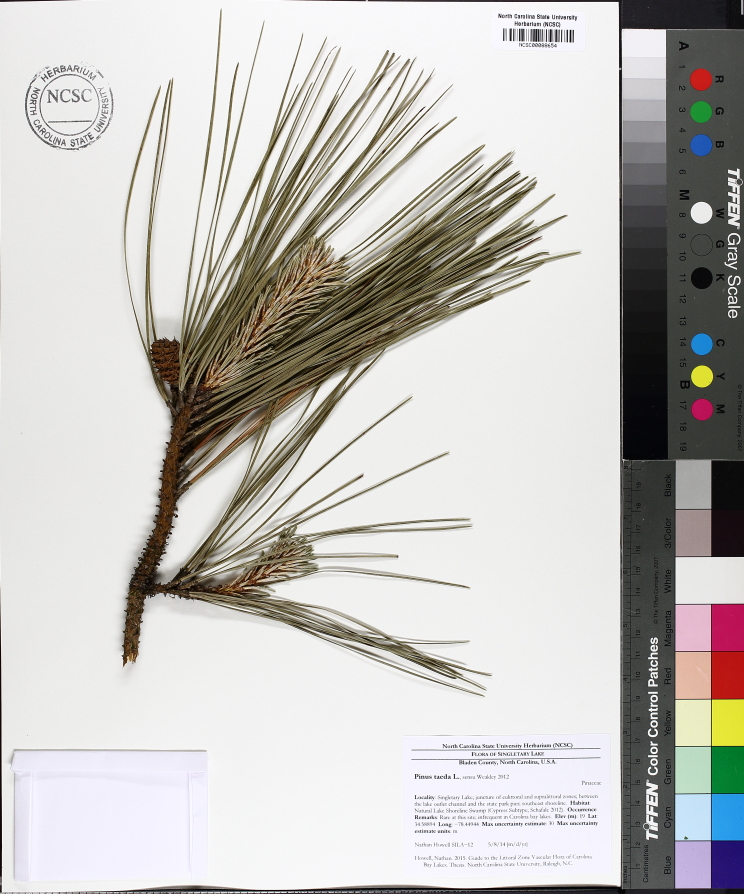
Specimen: *Howell SILA-12* (NCSC)

**Figure 29c.**
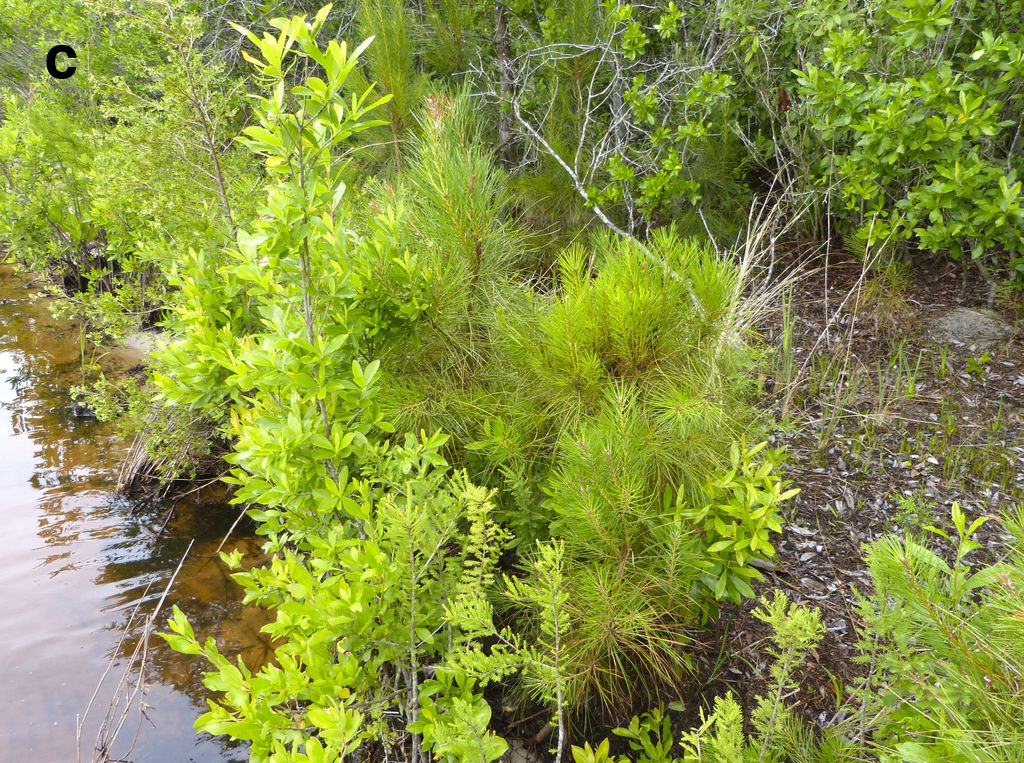
Young trees

**Figure 29d.**
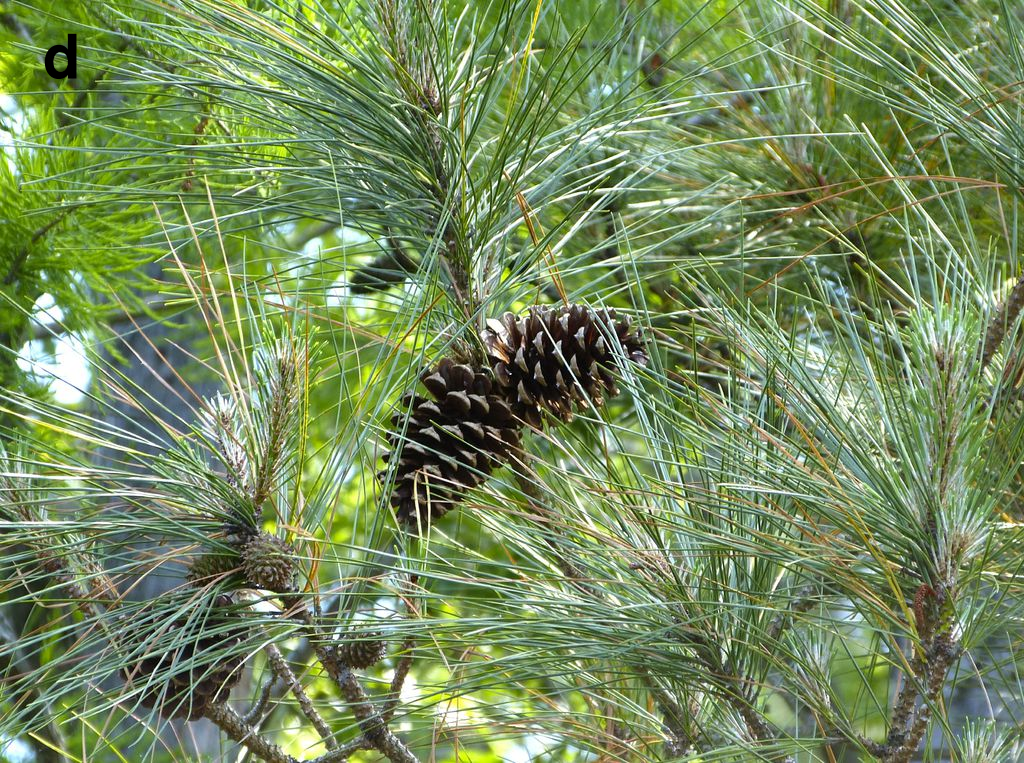
Mature seed cones

**Figure 32a.**
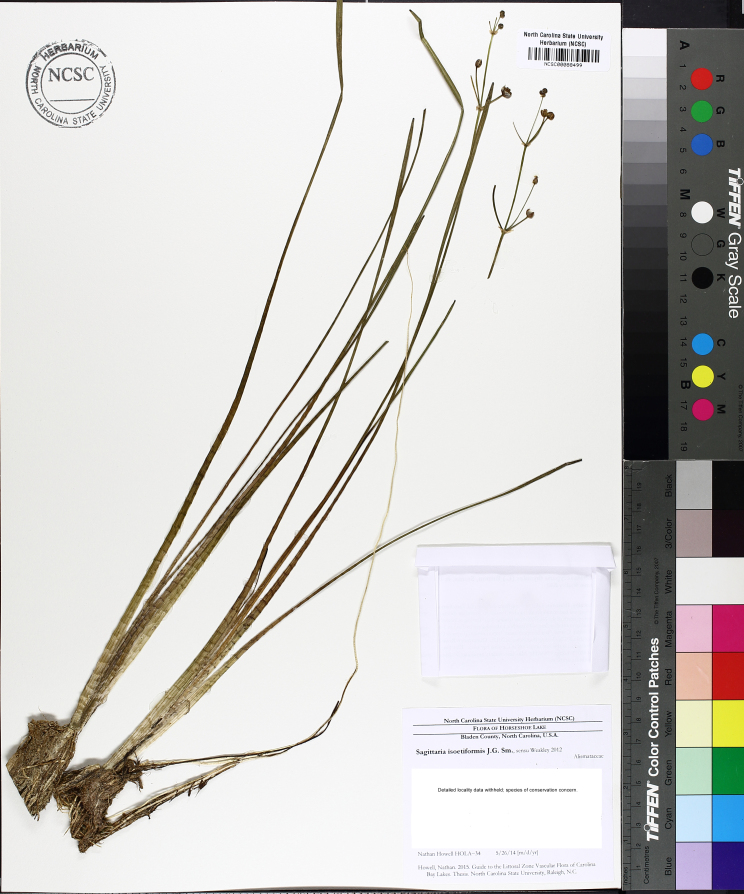
Specimen: *Howell HOLA-34* (NCSC)

**Figure 32b.**
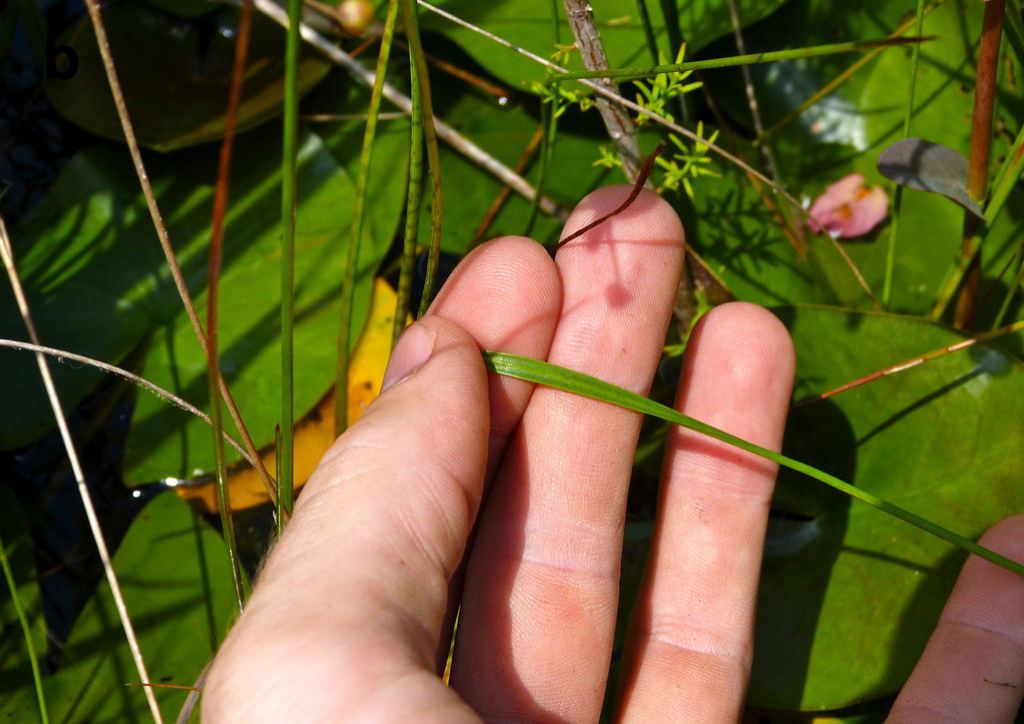
Leaf

**Figure 32c.**
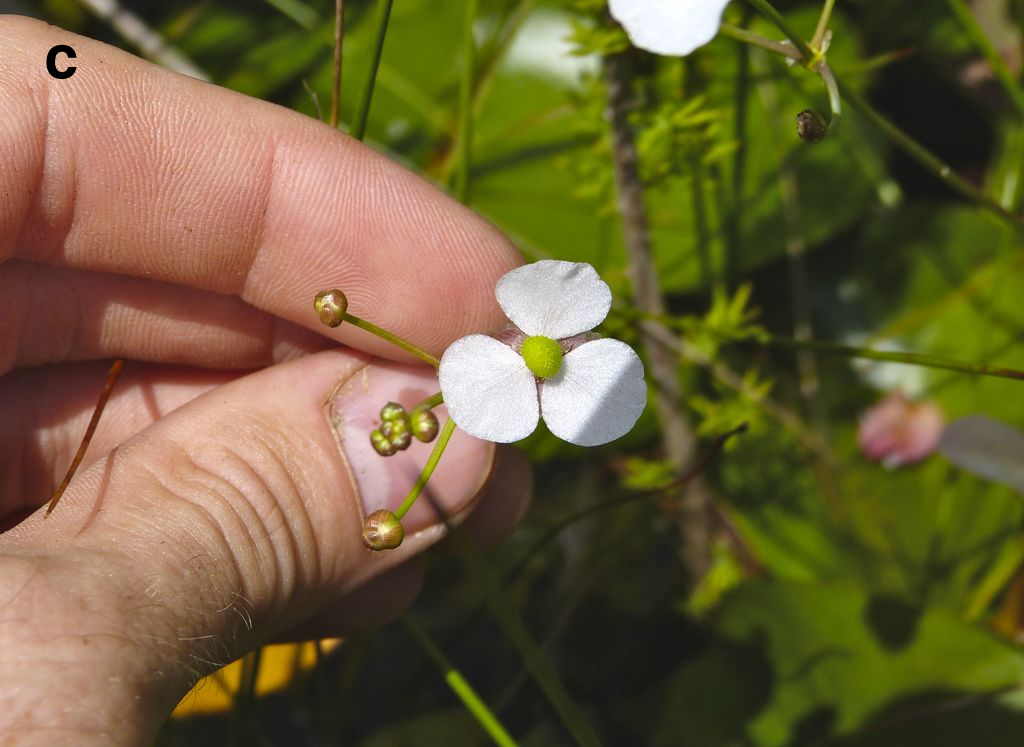
Flower and floral buds

**Figure 32d.**
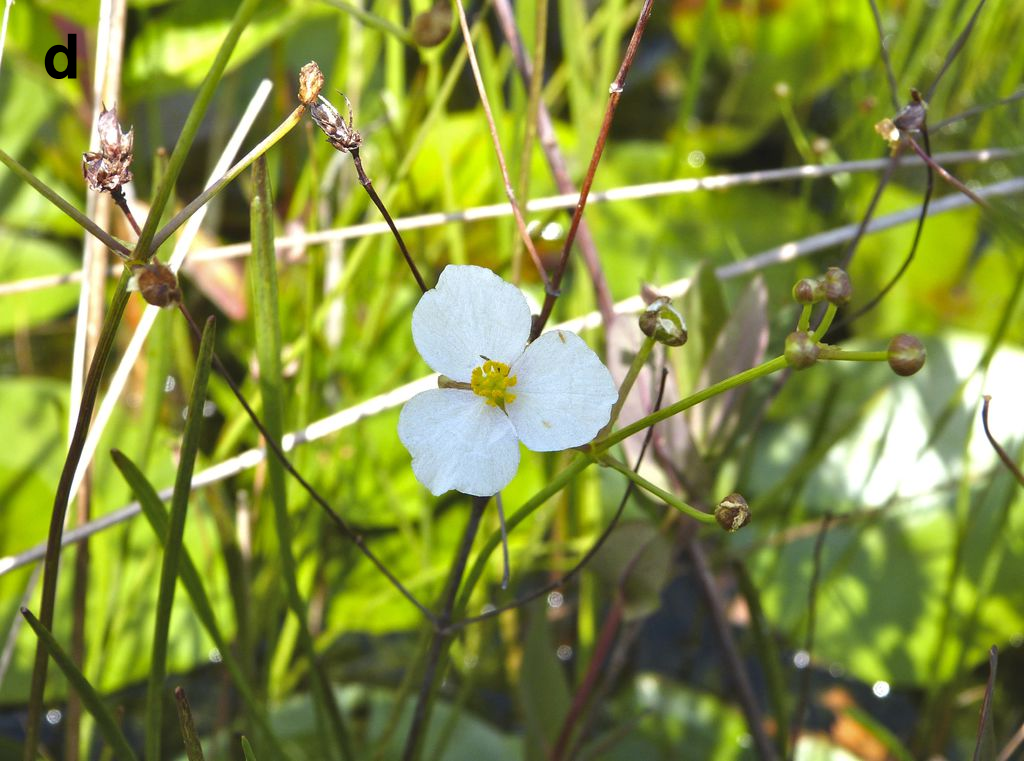
Flower and floral buds

**Figure 32e.**
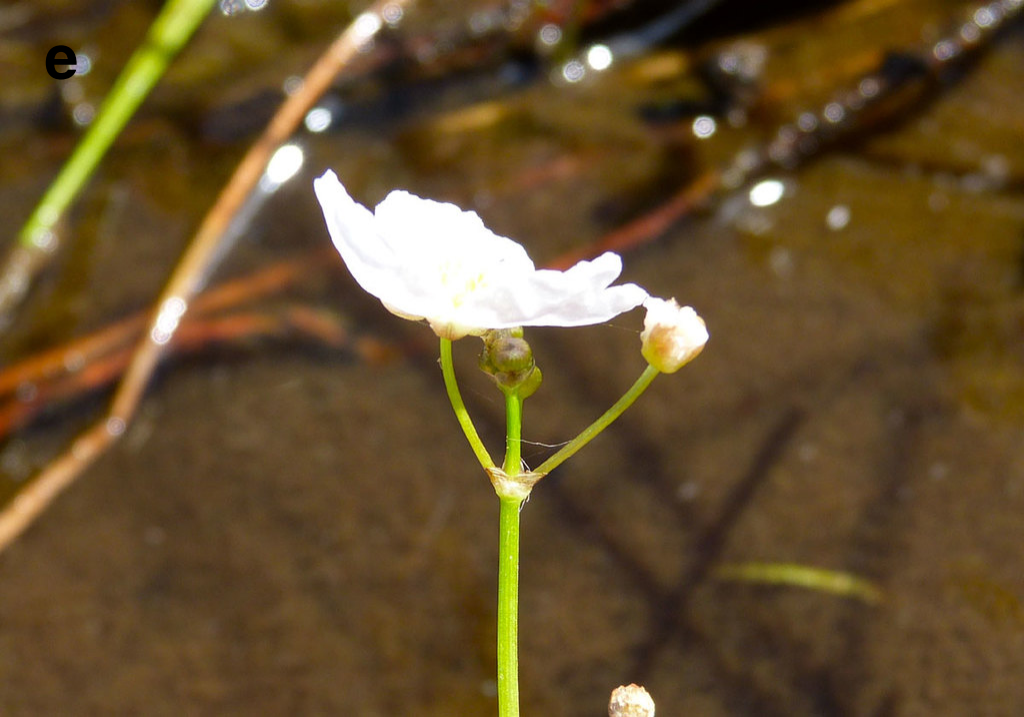
Inflorescence (note bract)

**Figure 32f.**
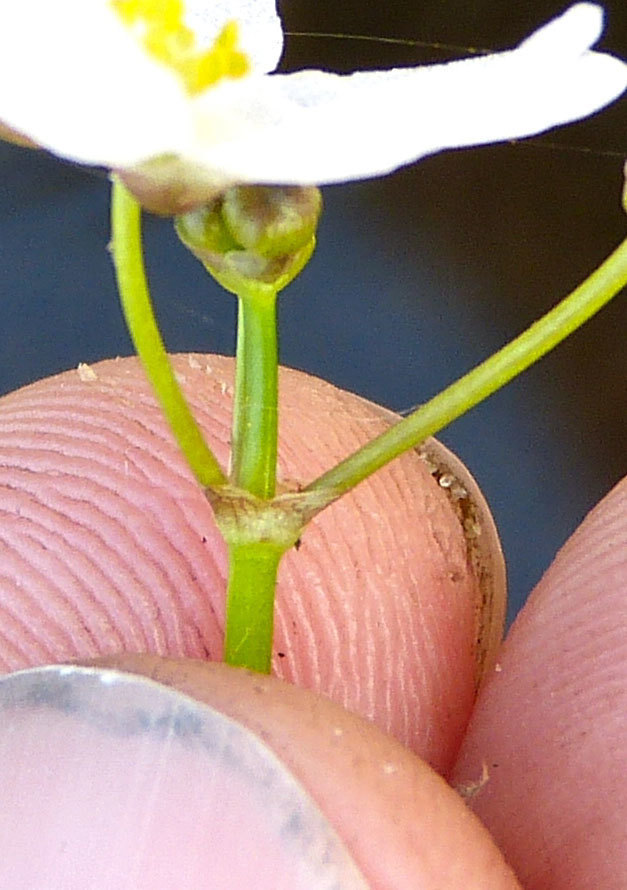
Inflorescence bract detail

**Figure 33a.**
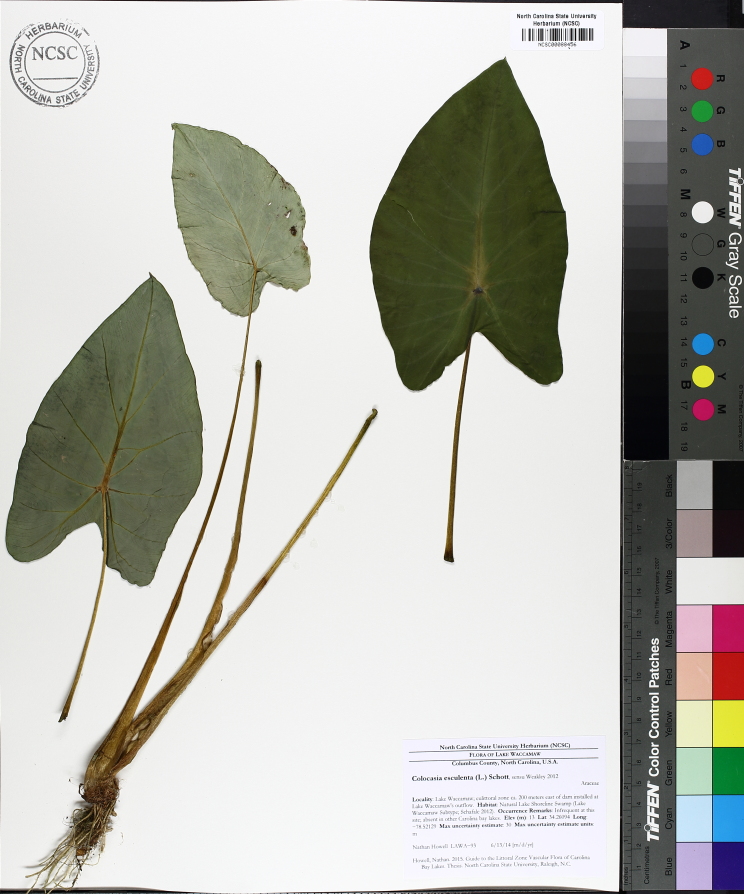
Specimen: *Howell LAWA-93* (NCSC)

**Figure 33b.**
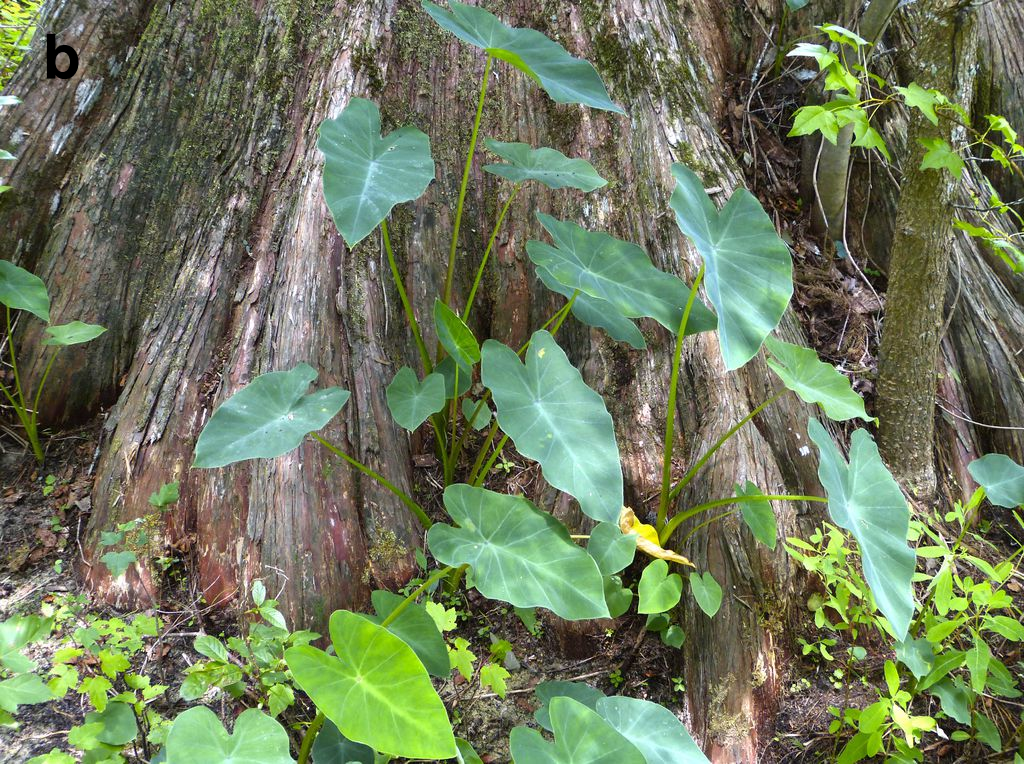
Habit

**Figure 34a.**
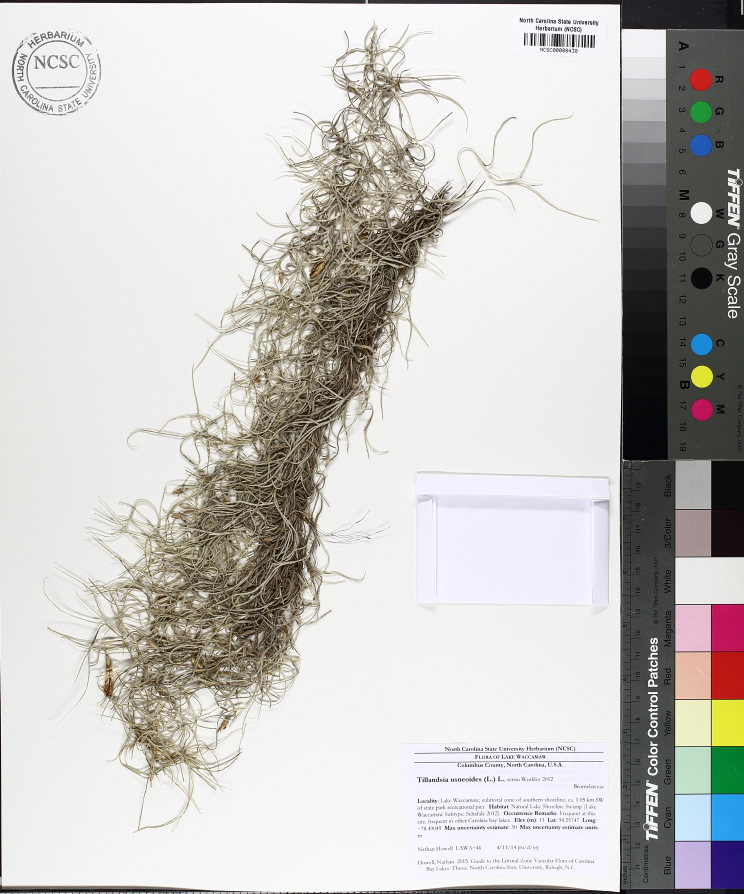
Specimen: *Howell LAWA-46* (NCSC)

**Figure 34b.**
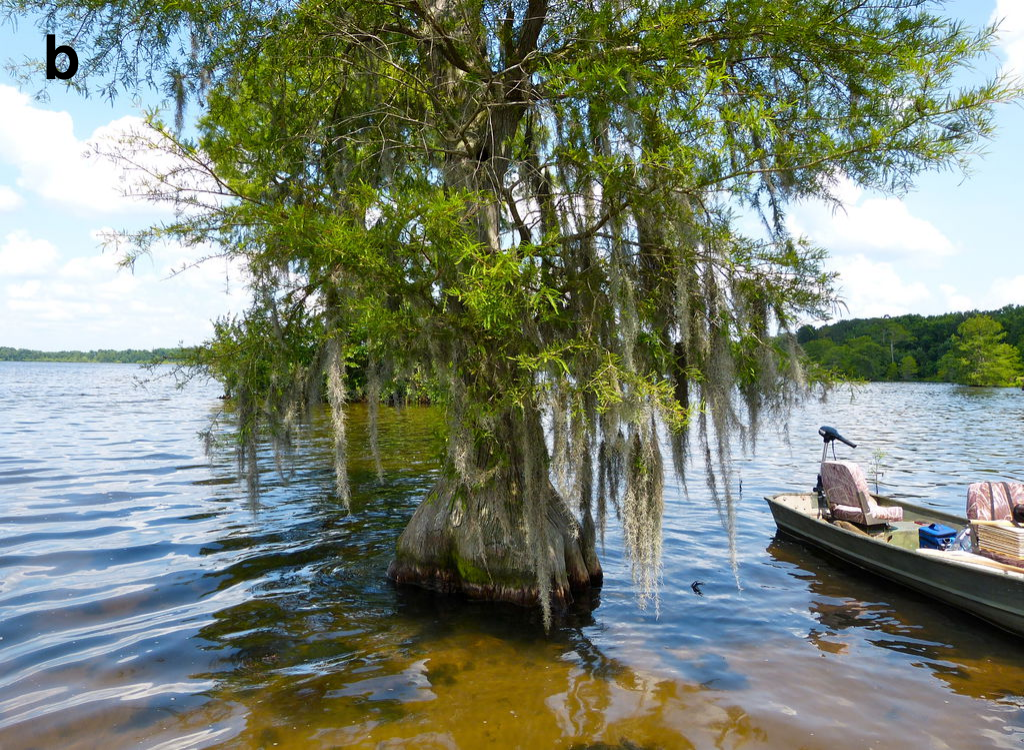
Habit (draped on branches of *Taxodium ascendens*)

**Figure 34c.**
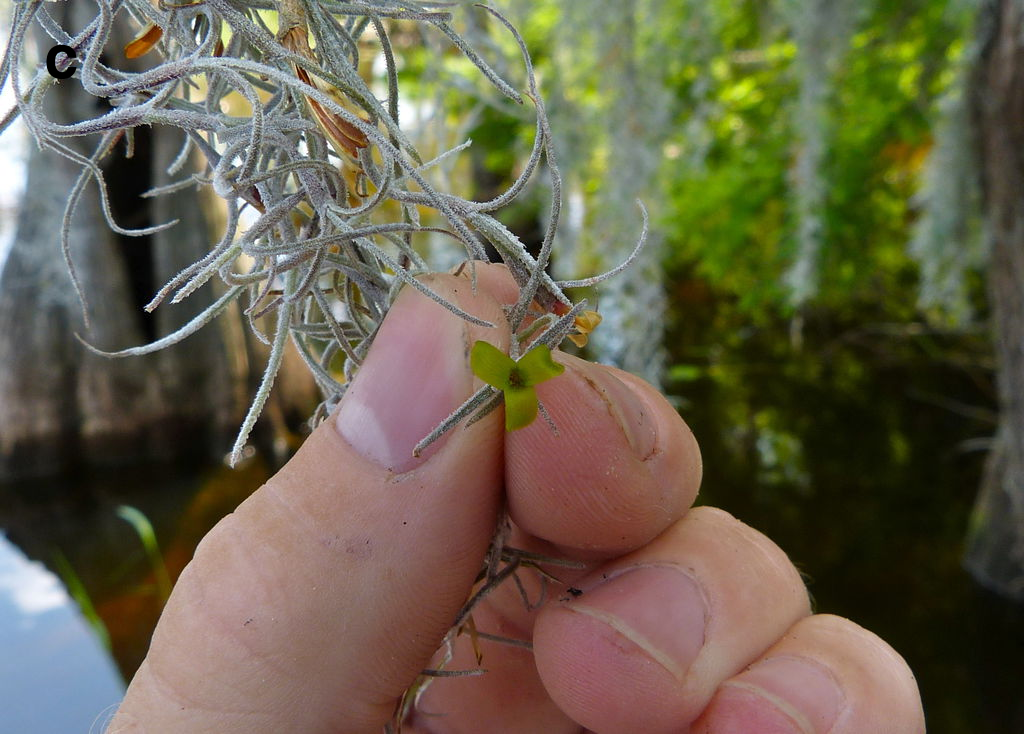
Flower

**Figure 34d.**
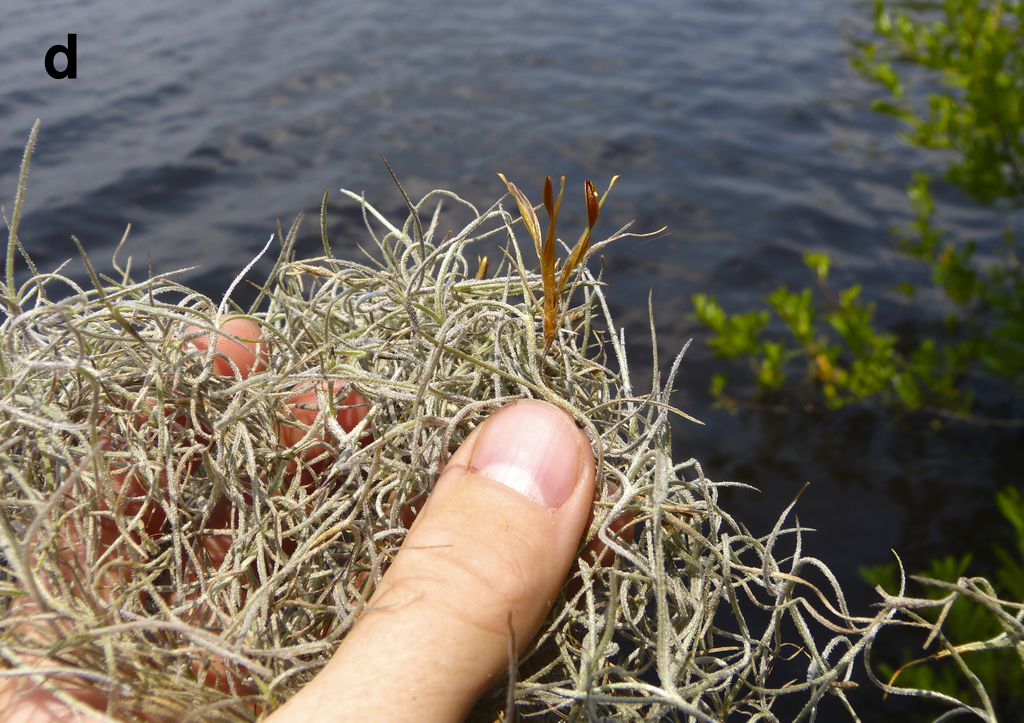
Capsule

**Figure 36a.**
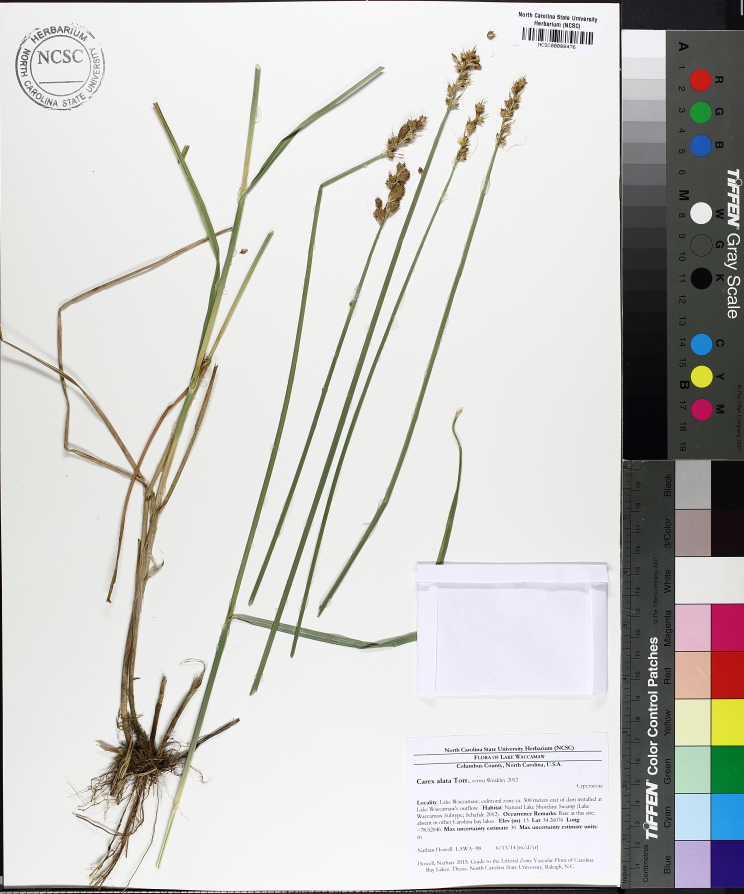
Specimen: *Howell LAWA-98* (NCSC)

**Figure 36b.**
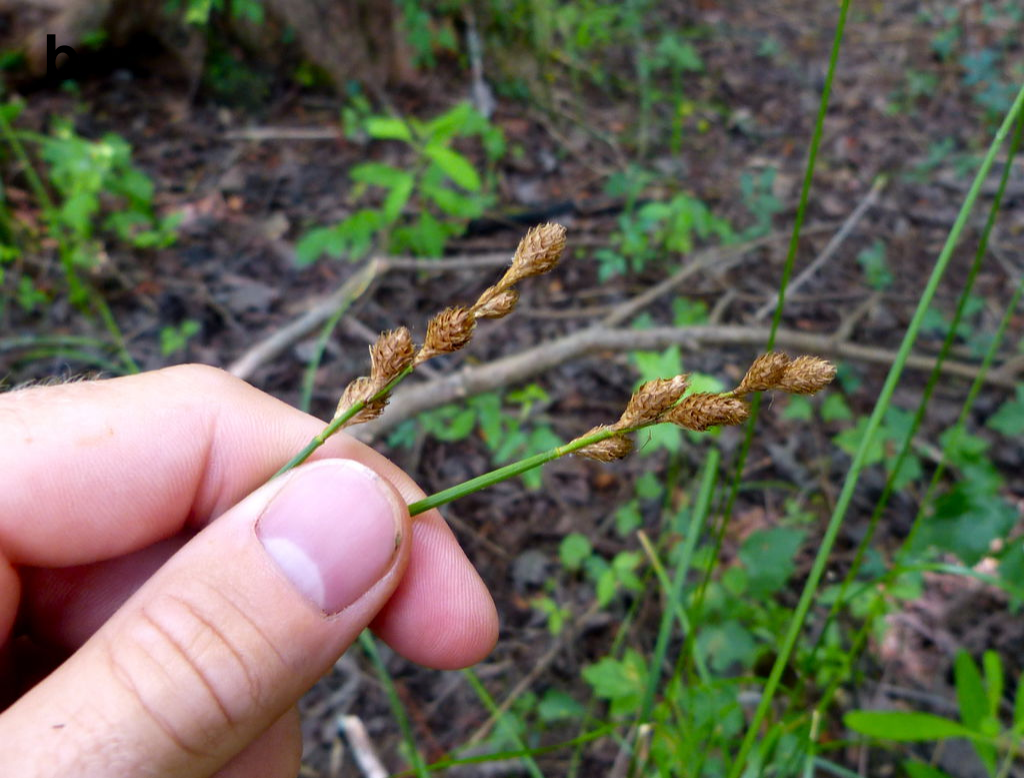
Inflorescence

**Figure 37a.**
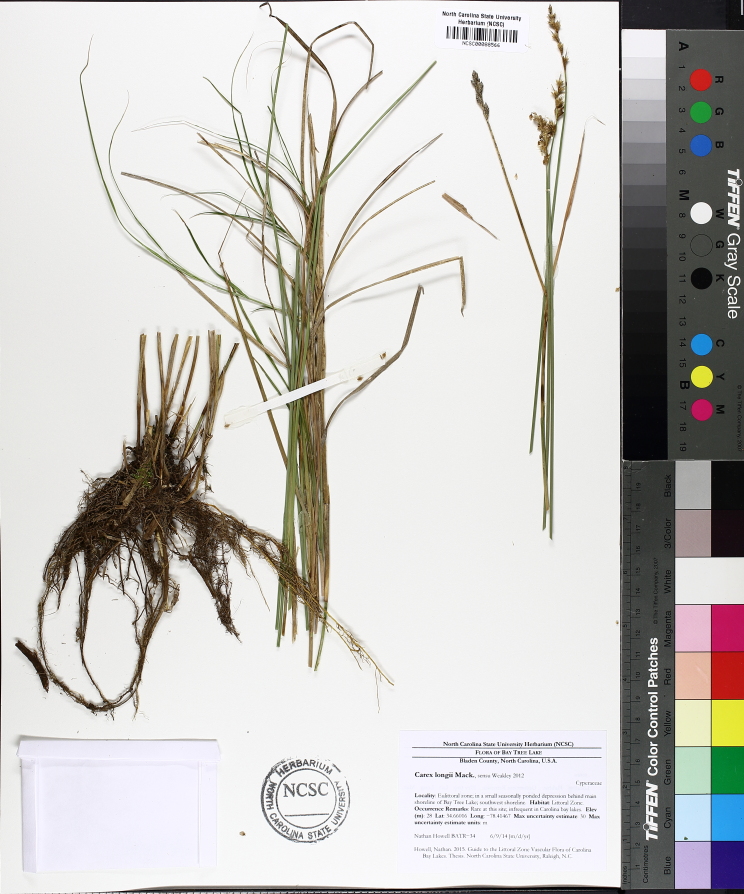
Specimen: *Howell BATR-34* (NCSC)

**Figure 37b.**
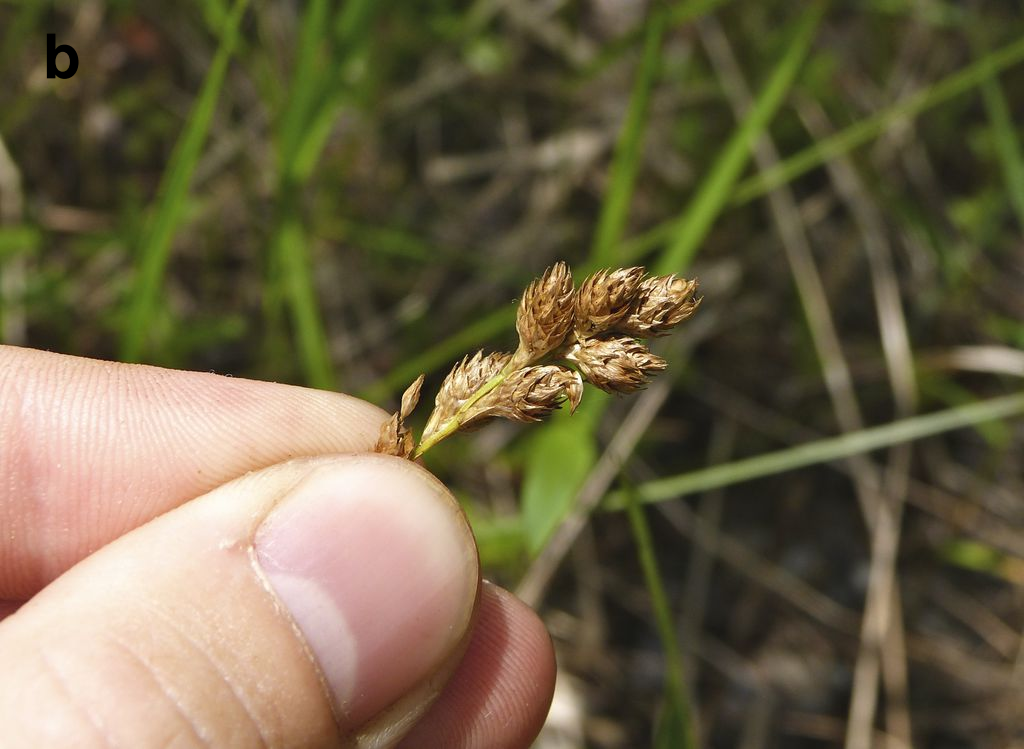
Inflorescence

**Figure 38a.**
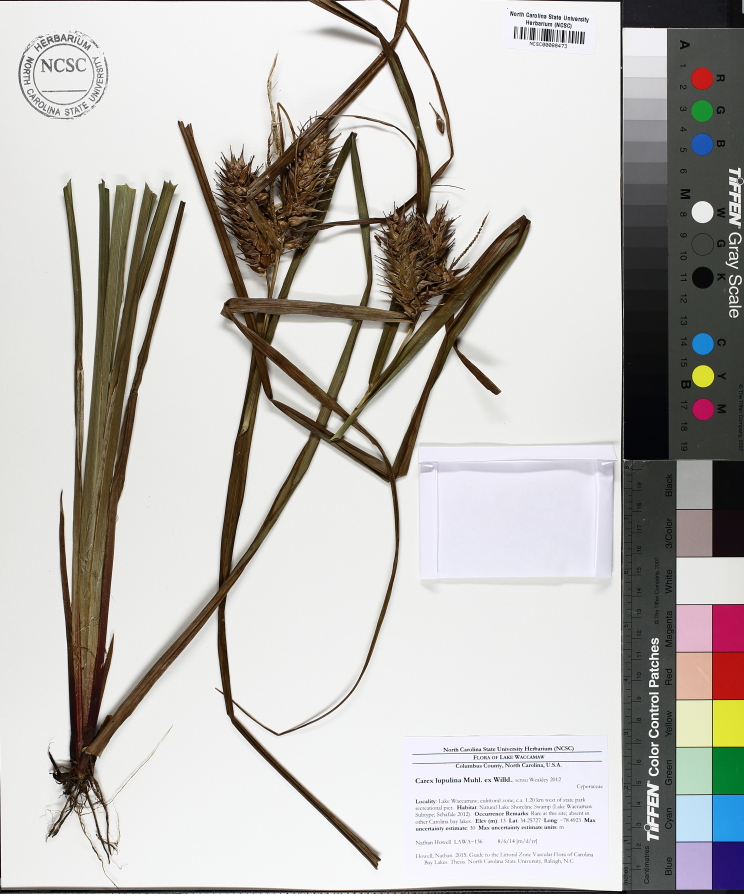
Specimen: *Howell LAWA-136* (NCSC)

**Figure 38b.**
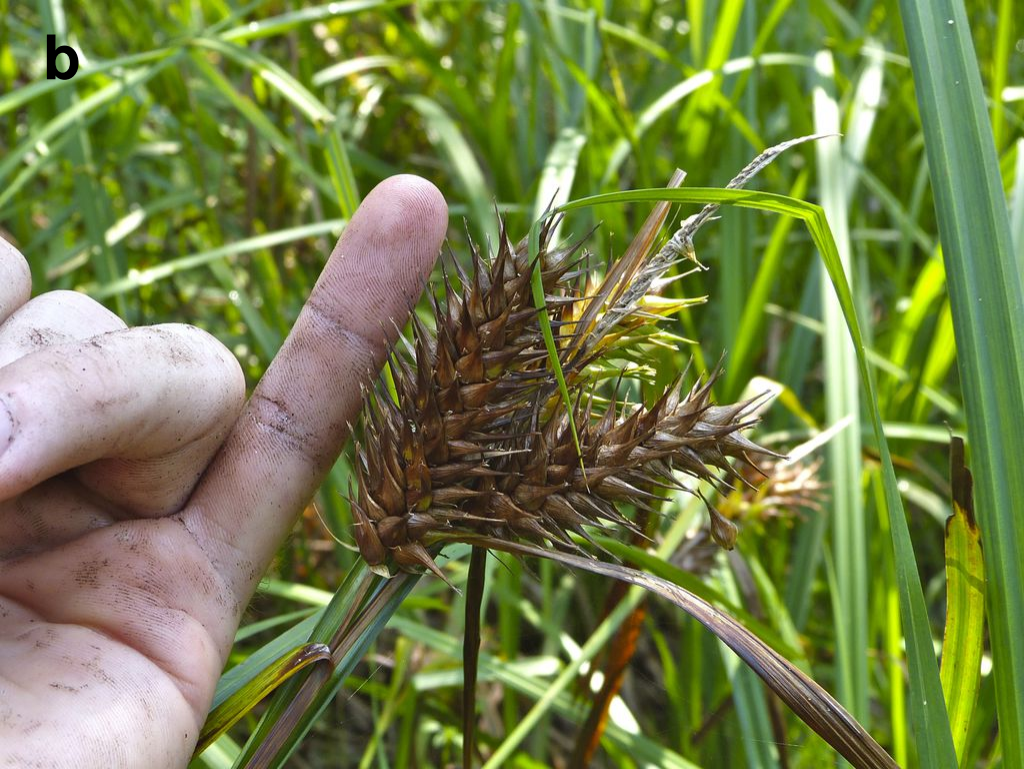
Inflorescence

**Figure 40a.**
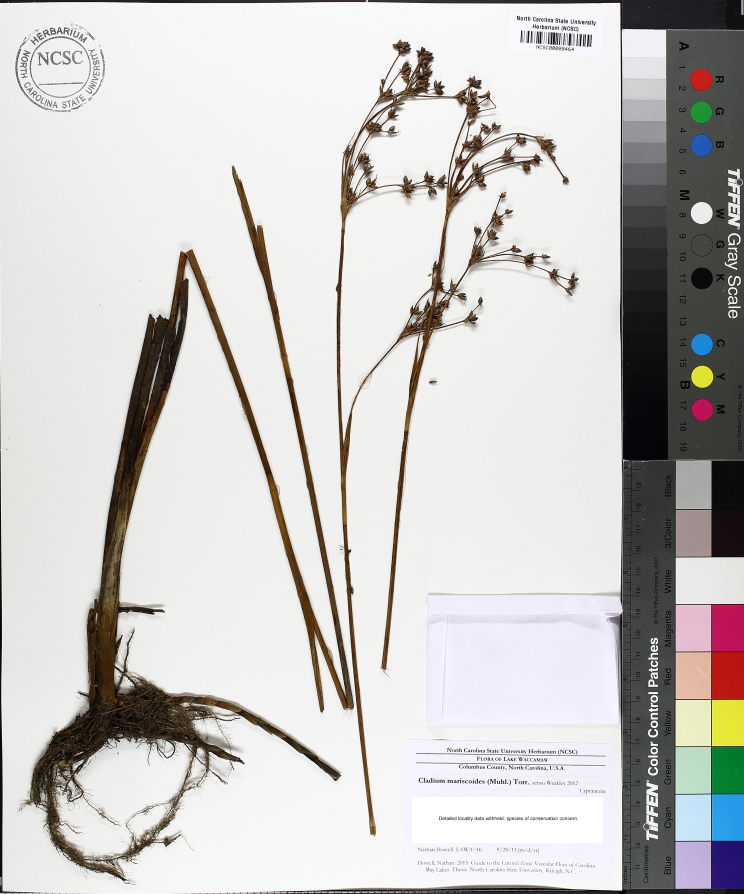
Specimen: *Howell LAWA-16* (NCSC)

**Figure 40b.**
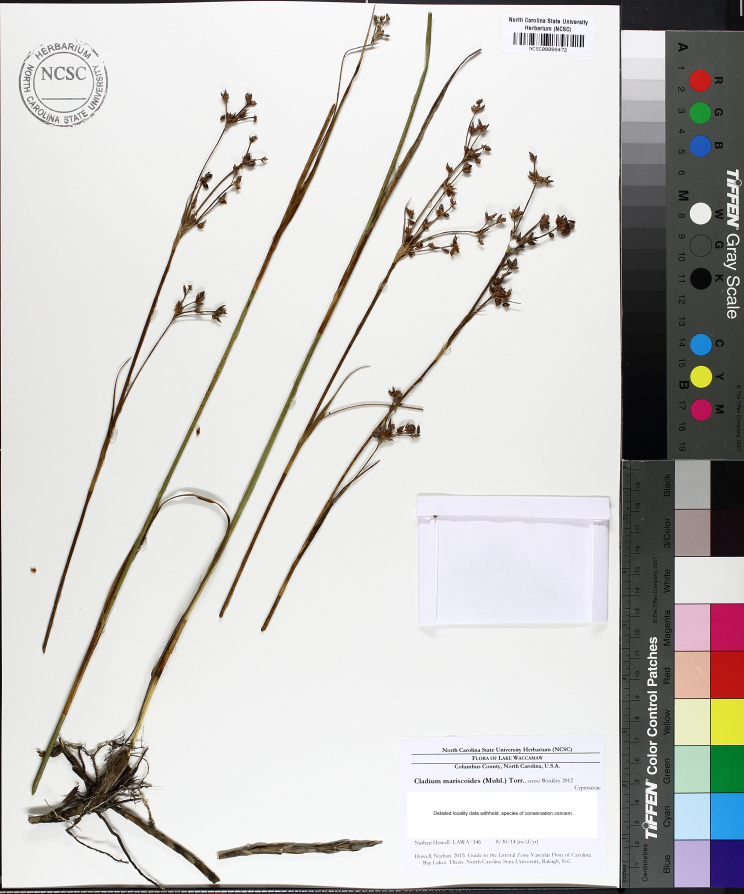
Specimen: *Howell LAWA-146* (NCSC)

**Figure 40c.**
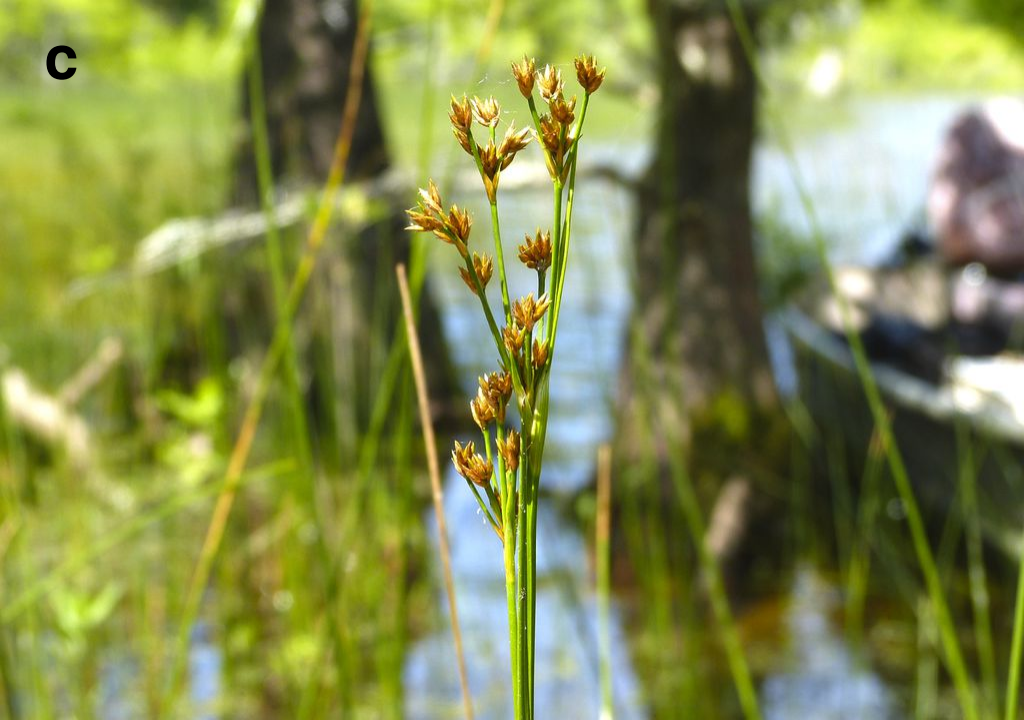
Inflorescence

**Figure 40d.**
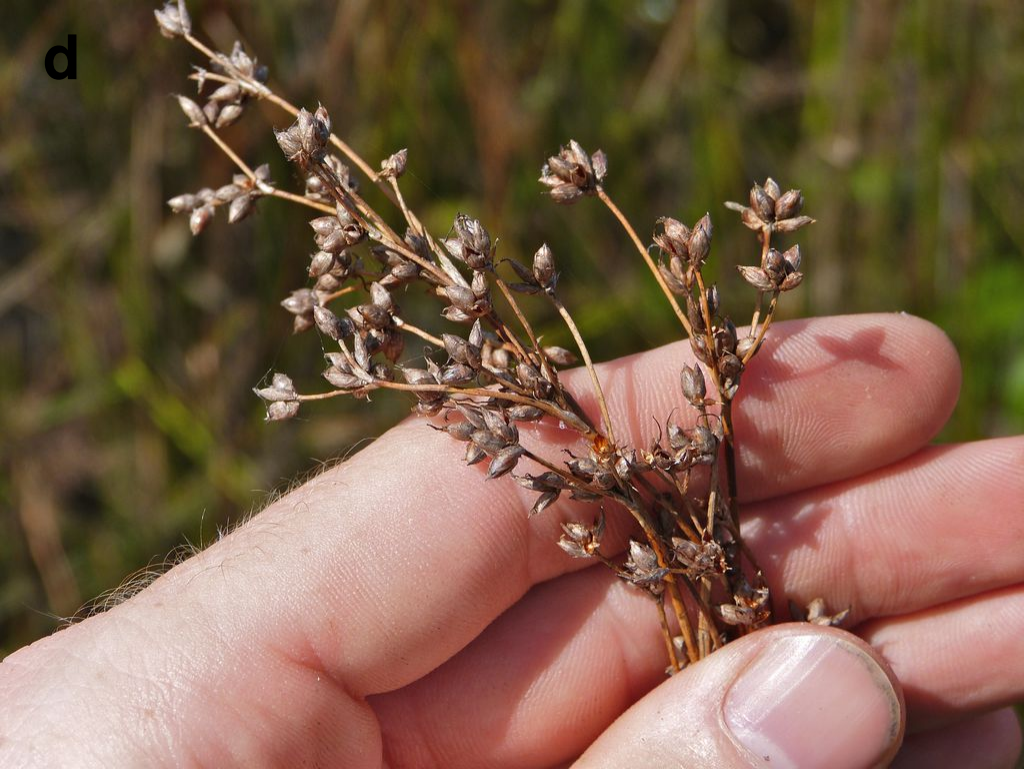
Inflorescence

**Figure 42a.**
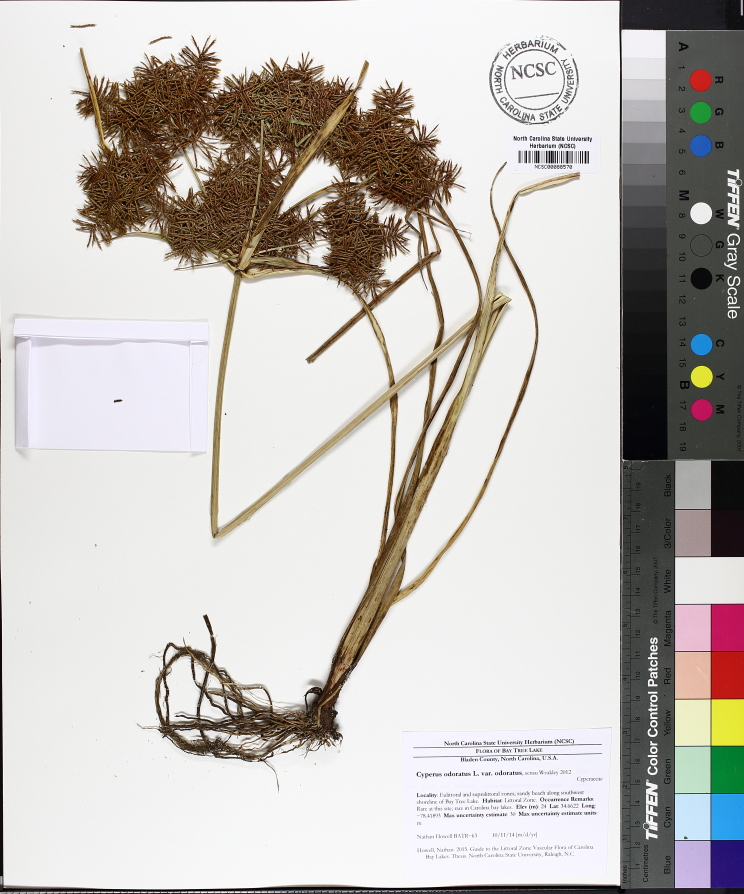
Specimen: *Howell BATR-63* (NCSC)

**Figure 42b.**
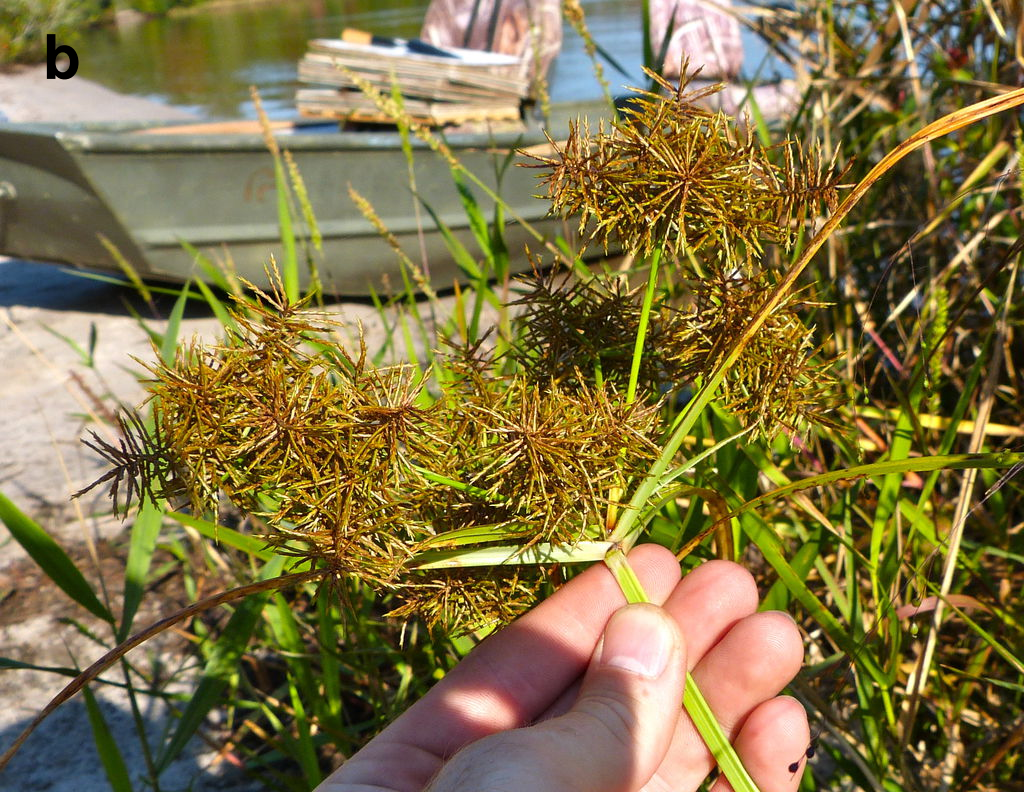
Inflorescence

**Figure 43a.**
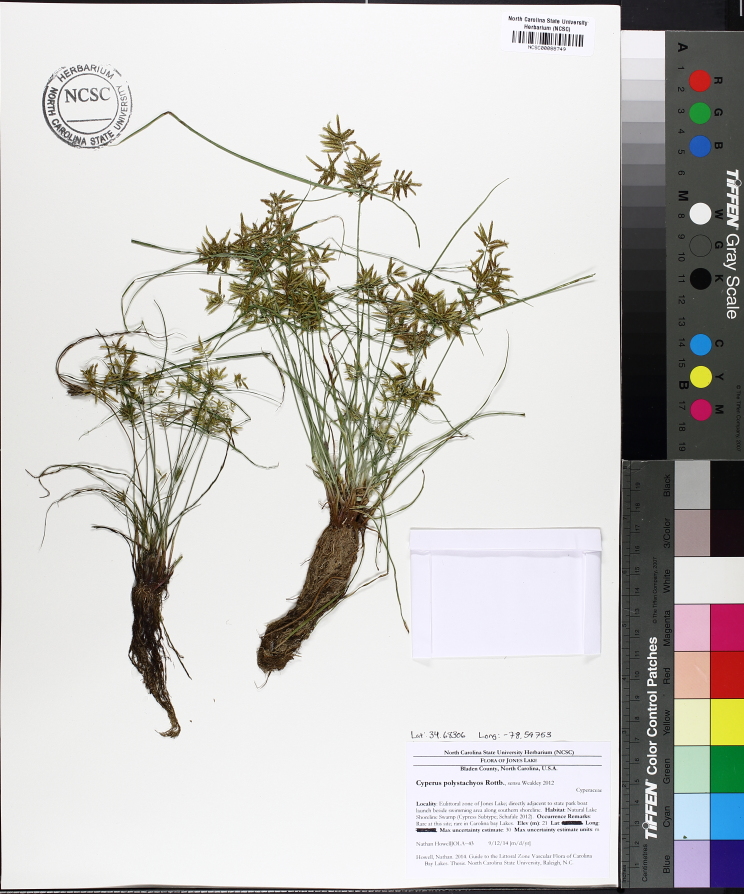
Specimen: *Howell JOLA-43* (NCSC)

**Figure 43b.**
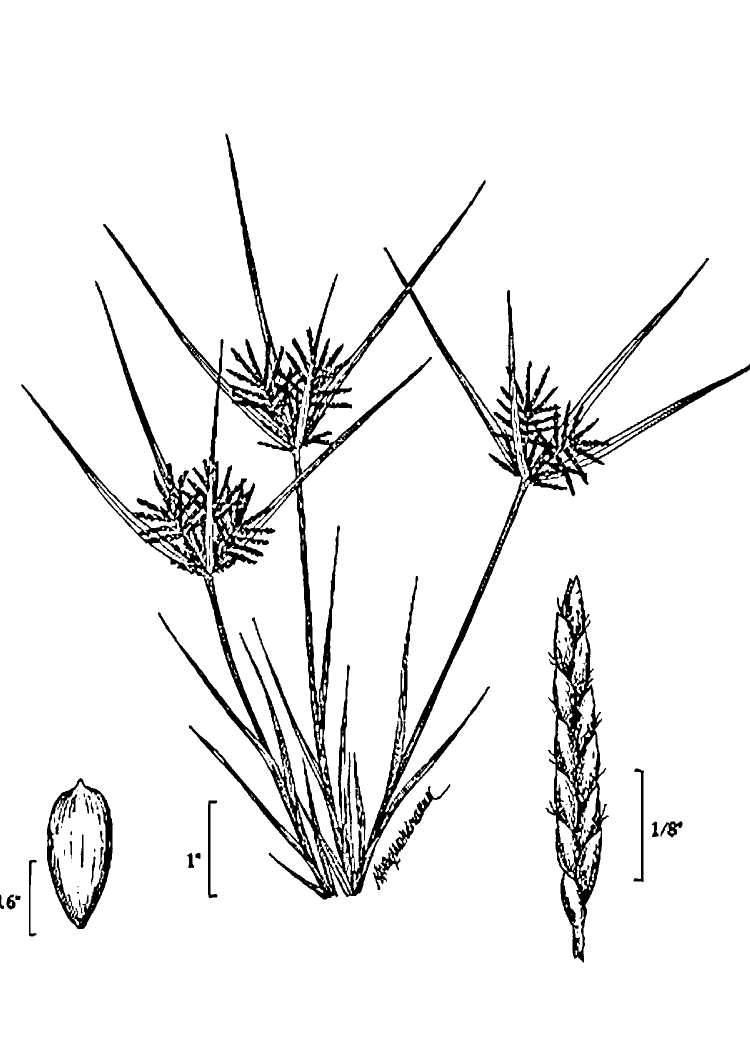
Illustration

**Figure 44a.**
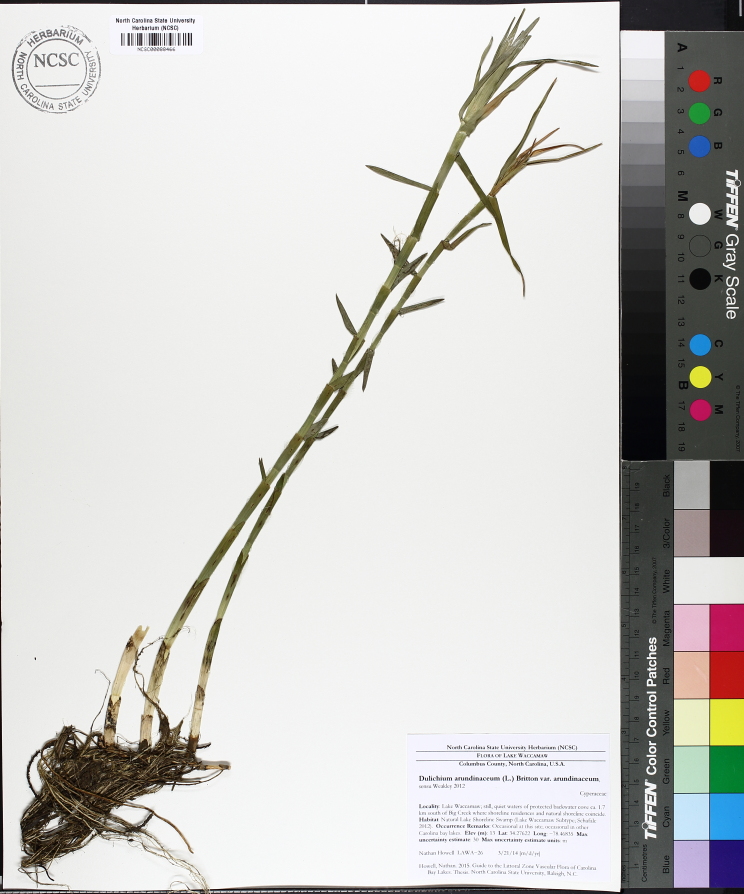
Specimen: *Howell LAWA-26* (NCSC)

**Figure 44b.**
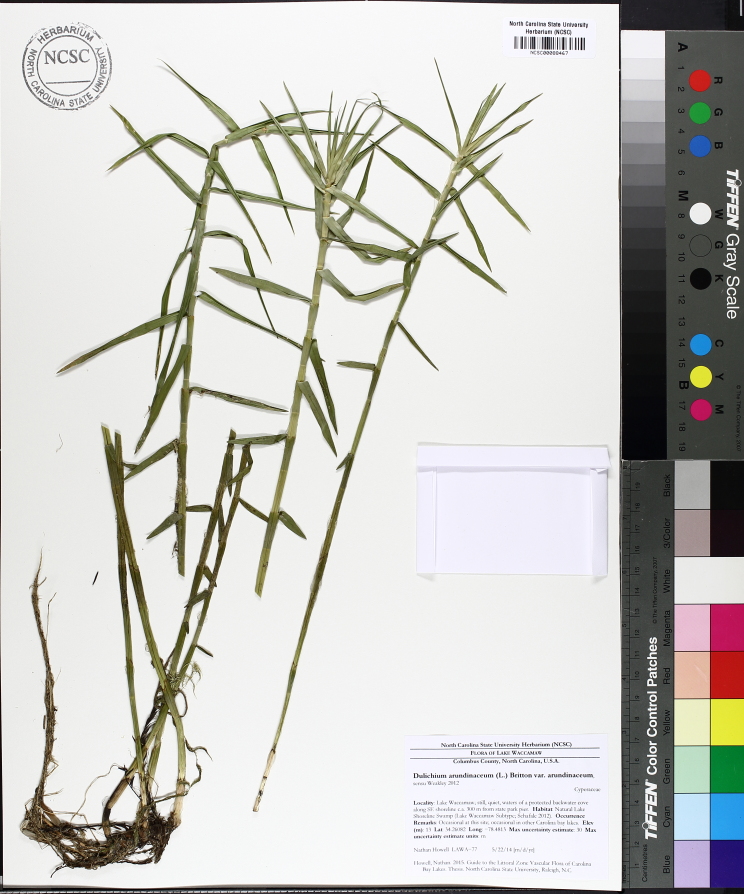
Specimen: *Howell LAWA-77* (NCSC)

**Figure 44c.**
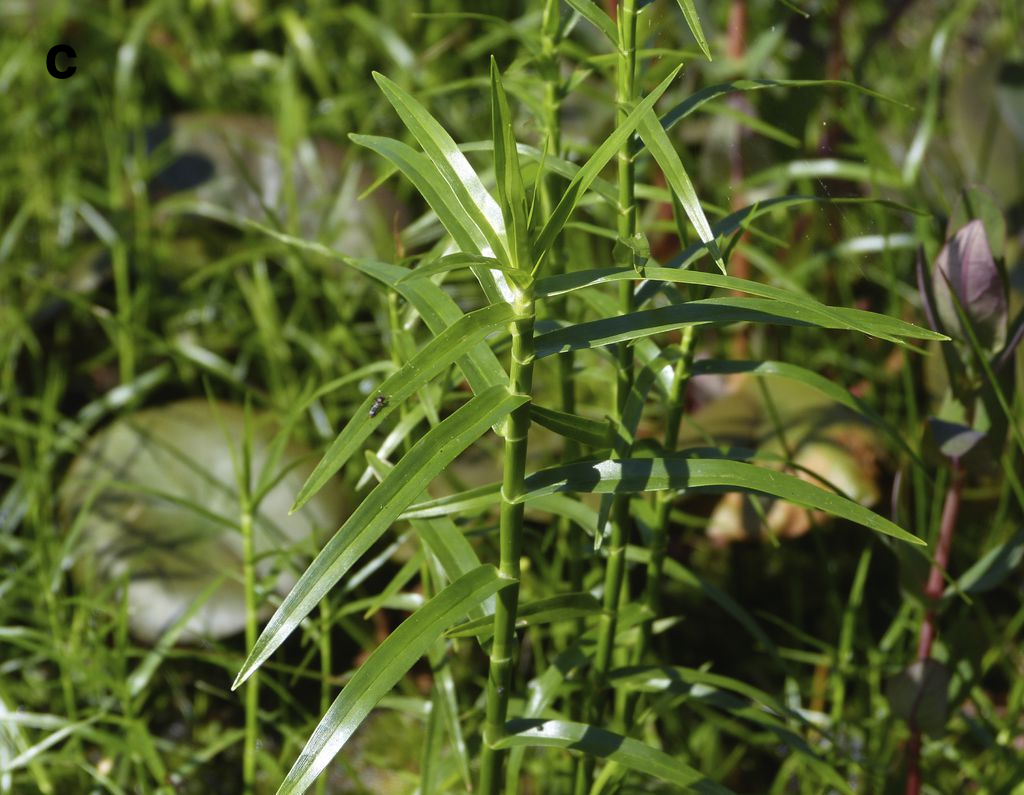
Habit

**Figure 44d.**
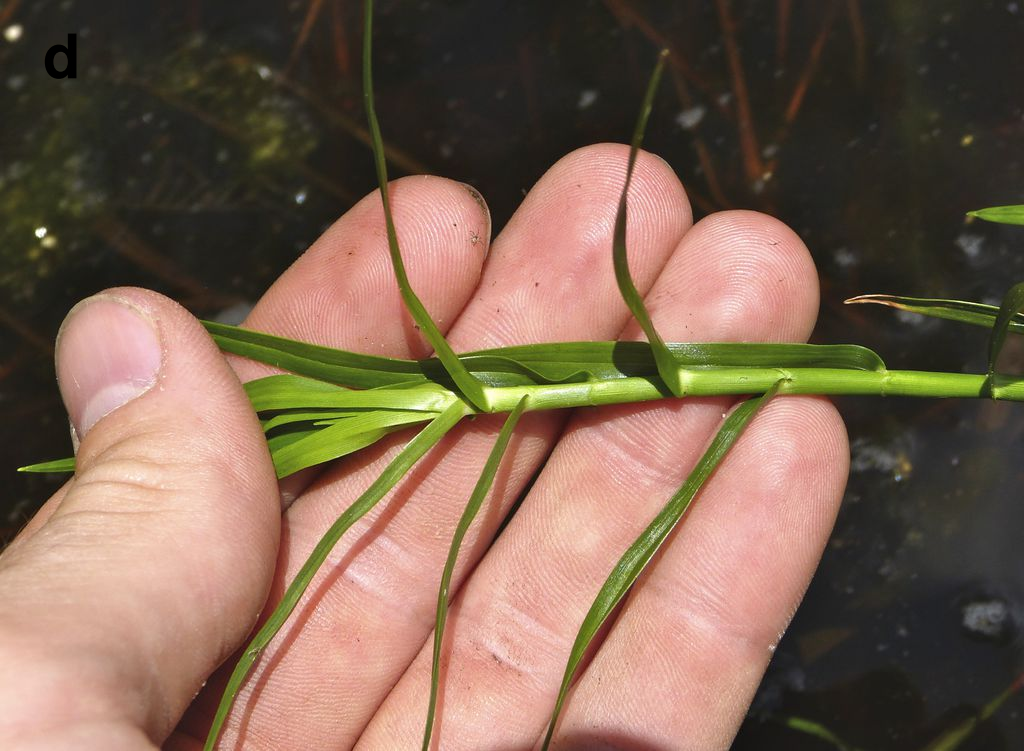
Leaves (3-ranked)

**Figure 45a.**
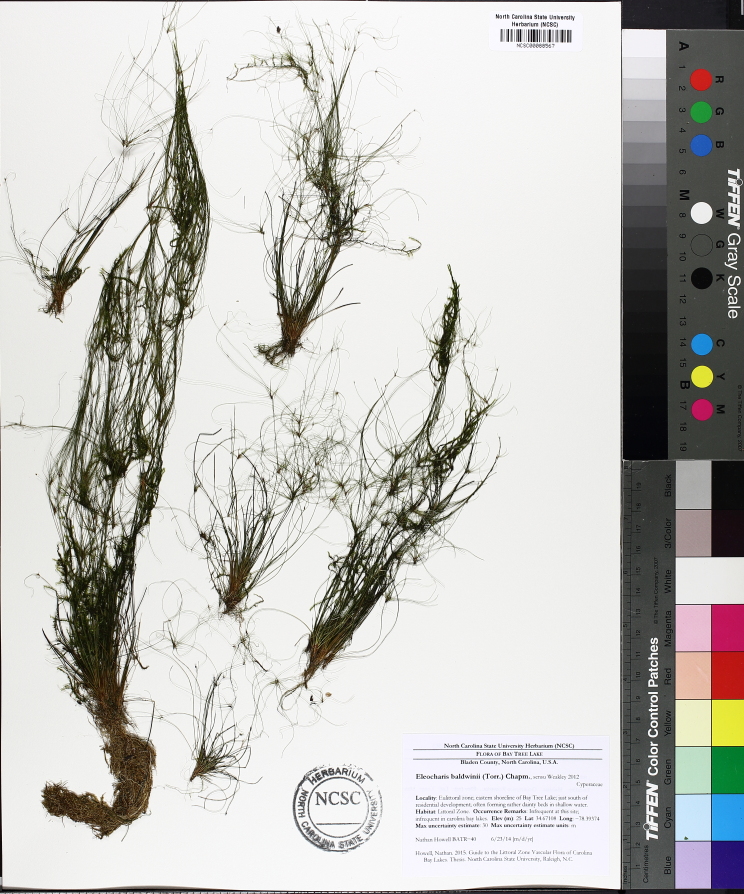
Specimen: *Howell BATR-40* (NCSC)

**Figure 45b.**
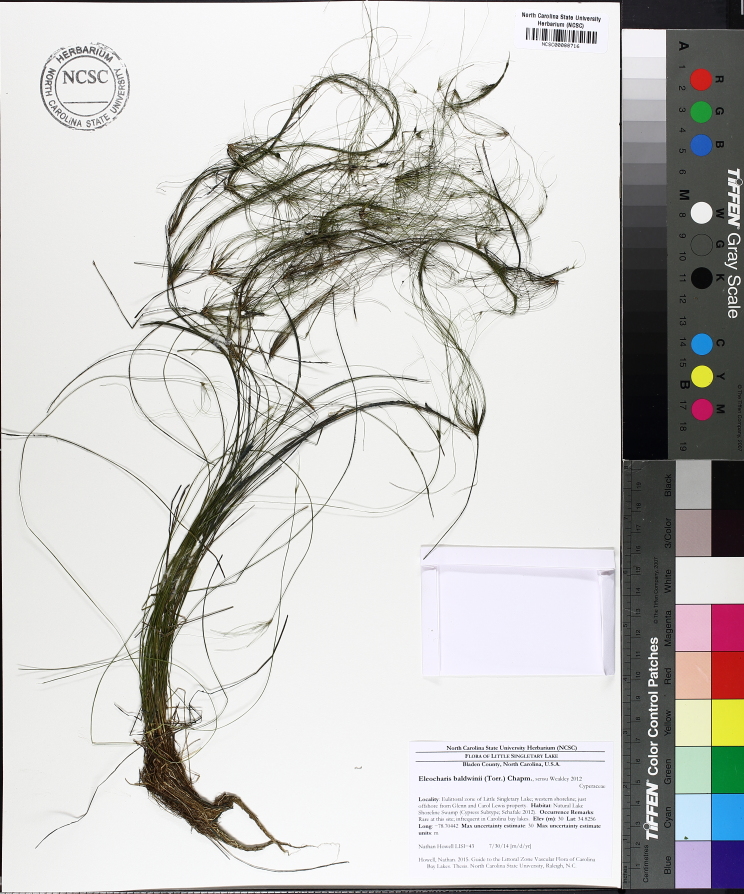
Specimen: *Howell LISI-43* (NCSC)

**Figure 45c.**
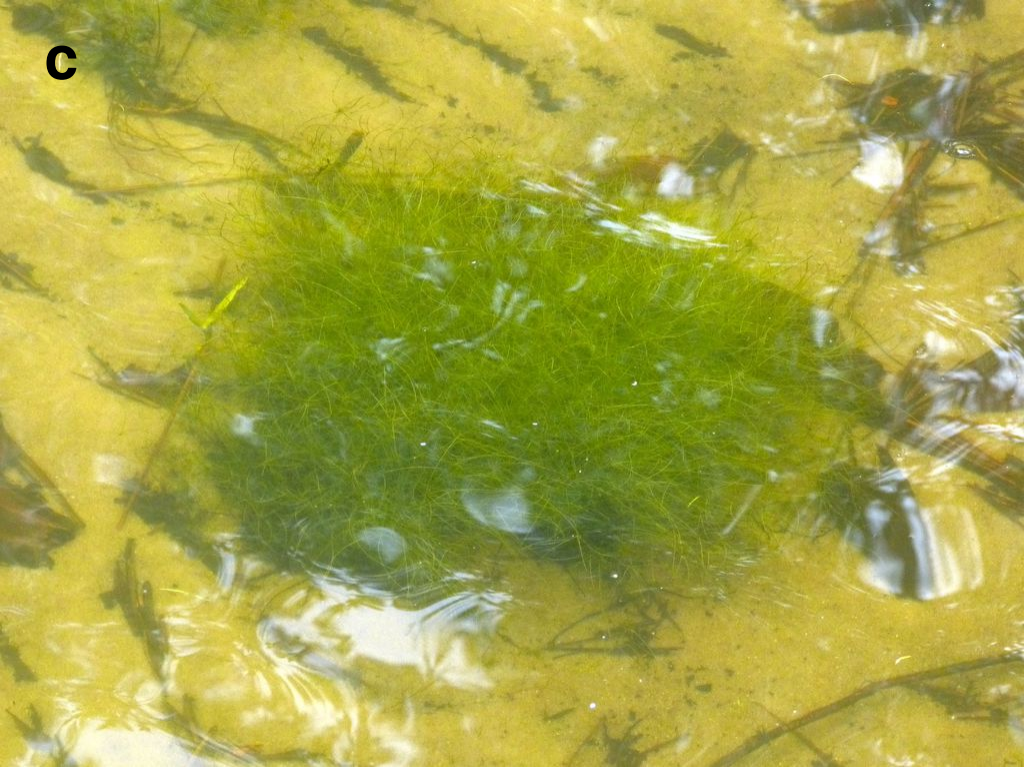
Habit

**Figure 45d.**
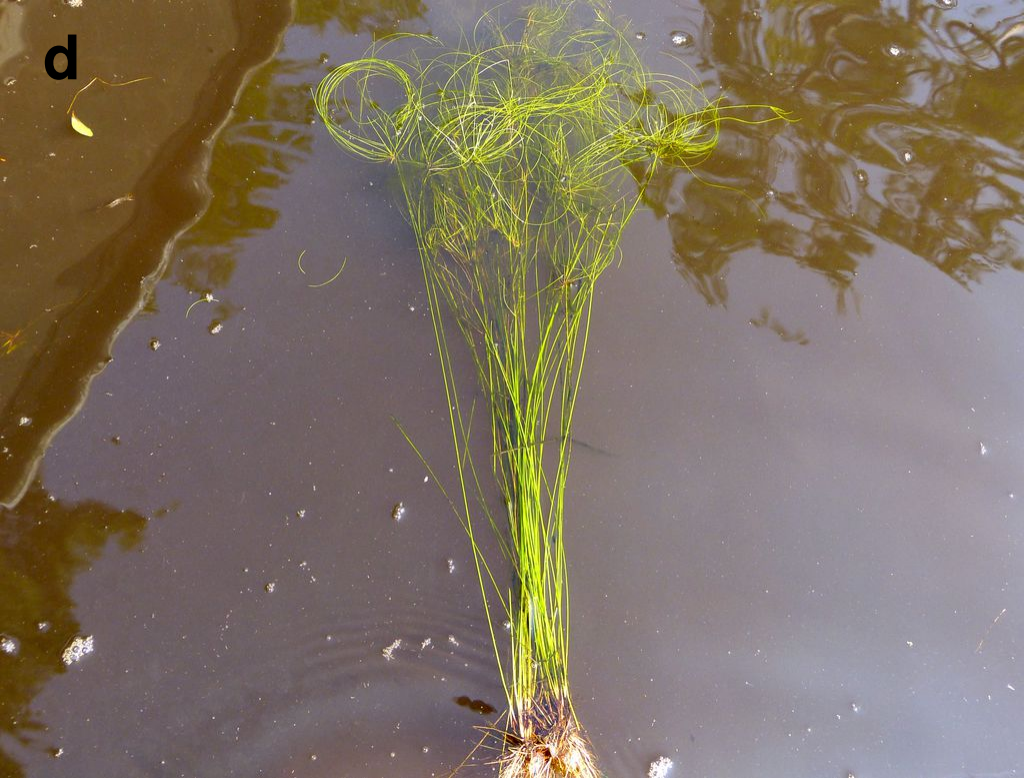
Habit

**Figure 46a.**
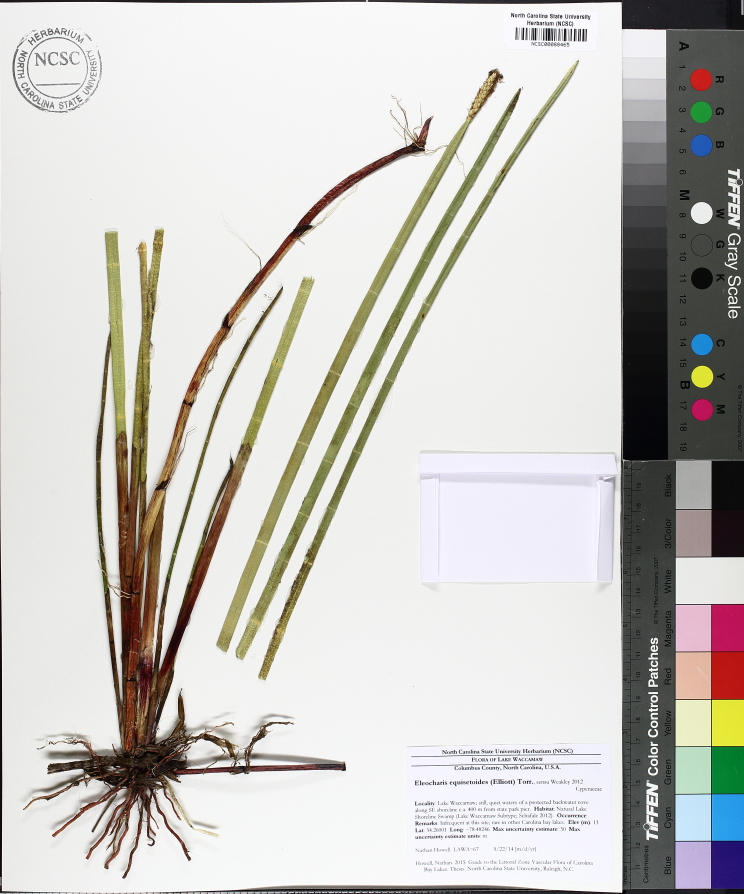
Specimen: *Howell LAWA-67* (NCSC)

**Figure 46b.**
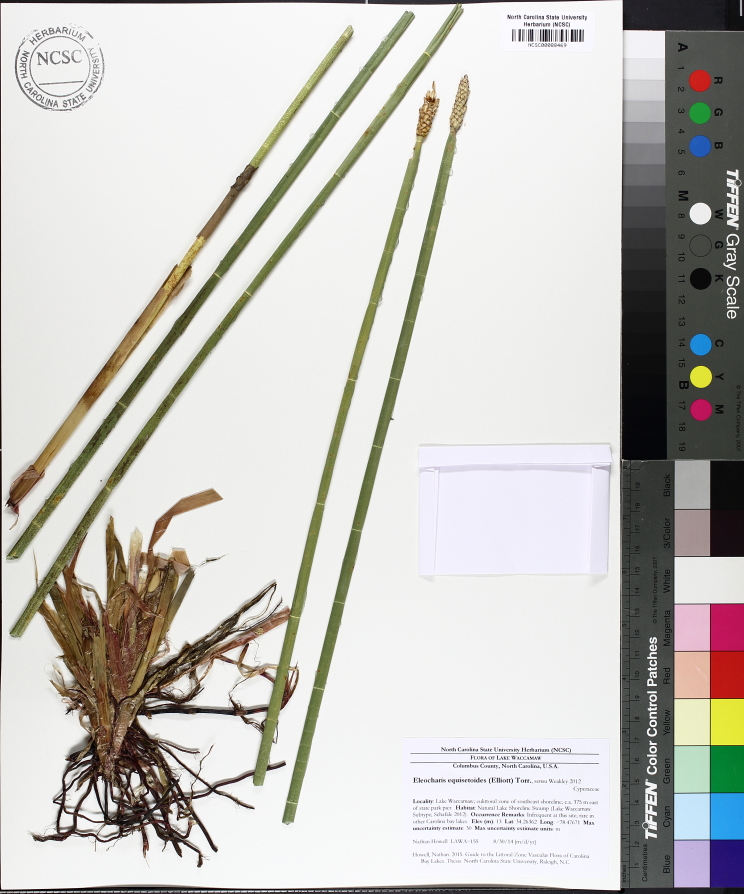
Specimen: *Howell LAWA-155* (NCSC)

**Figure 46c.**
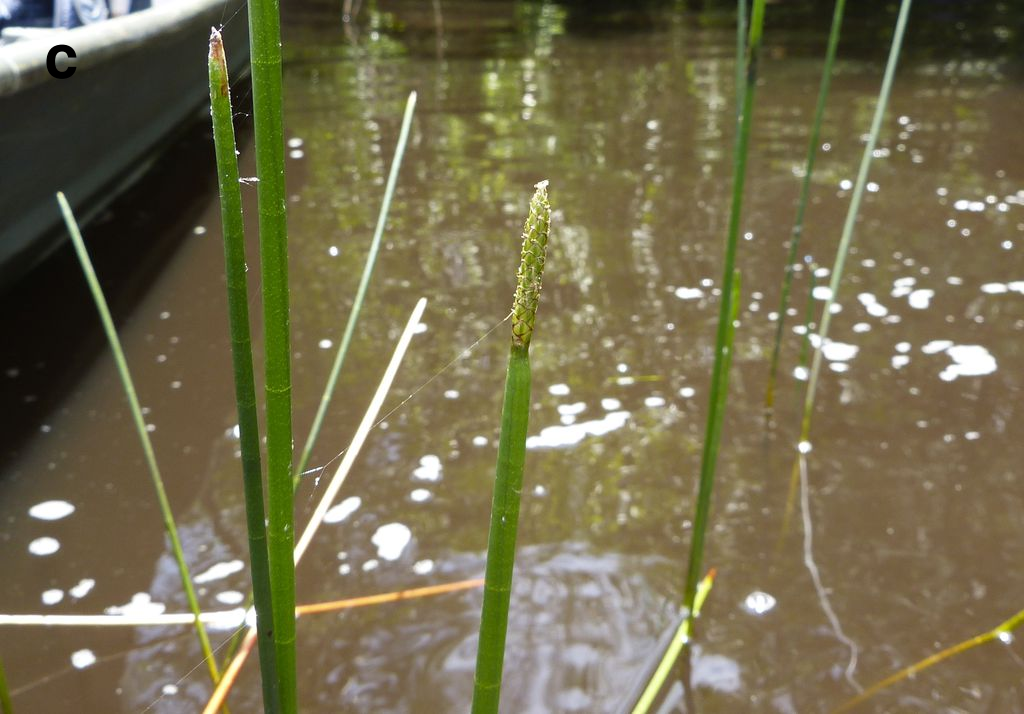
Habit

**Figure 46d.**
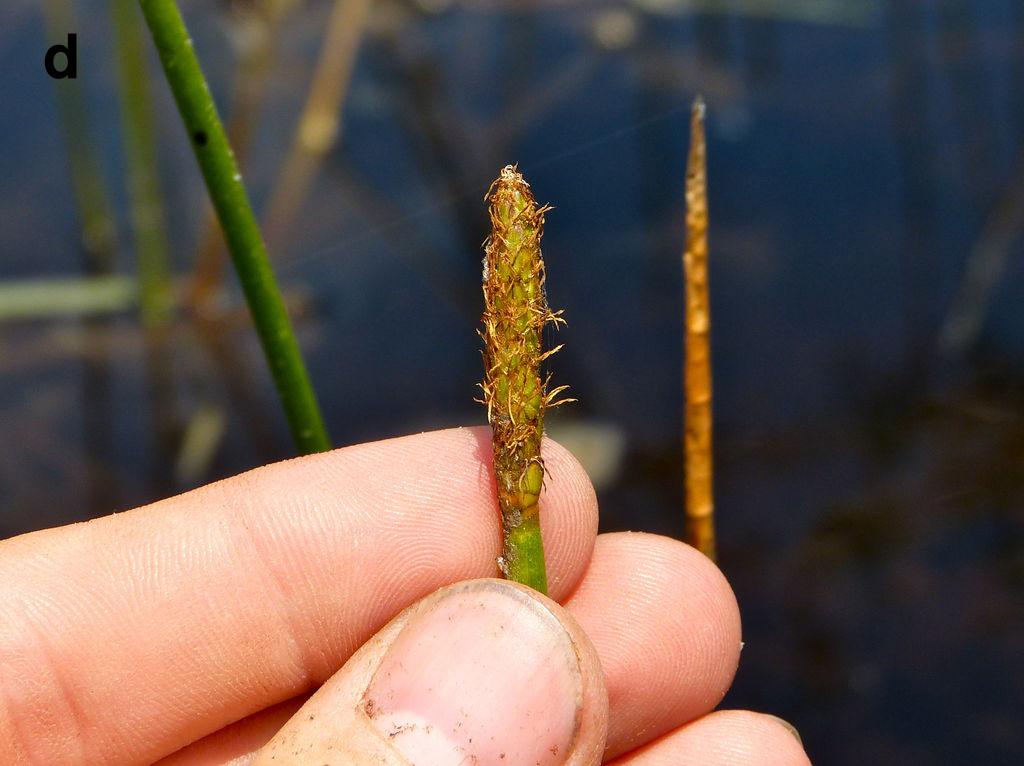
Inflorescence detail

**Figure 47a.**
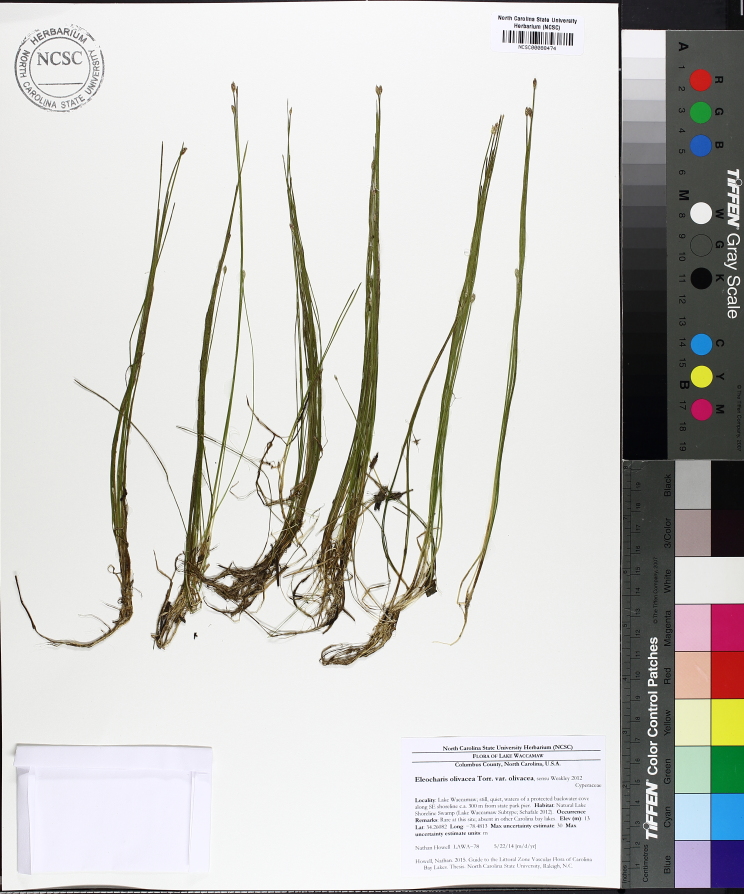
Specimen: *Howell LAWA-78* (NCSC)

**Figure 47b.**
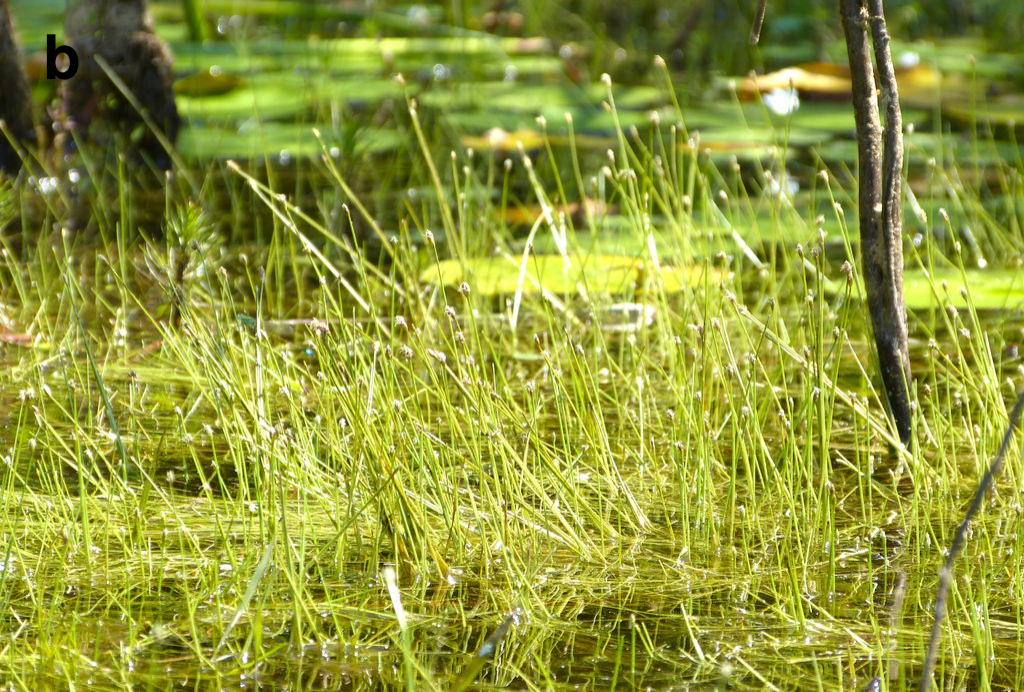
Habit

**Figure 48a.**
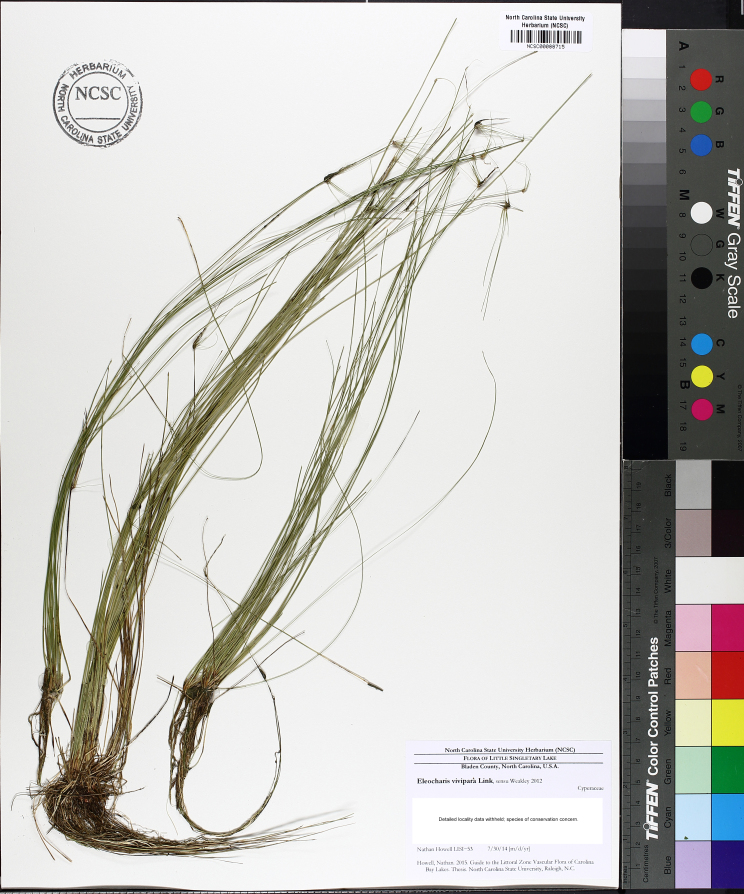
Specimen: *Howell LISI-53* (NCSC)

**Figure 48b.**
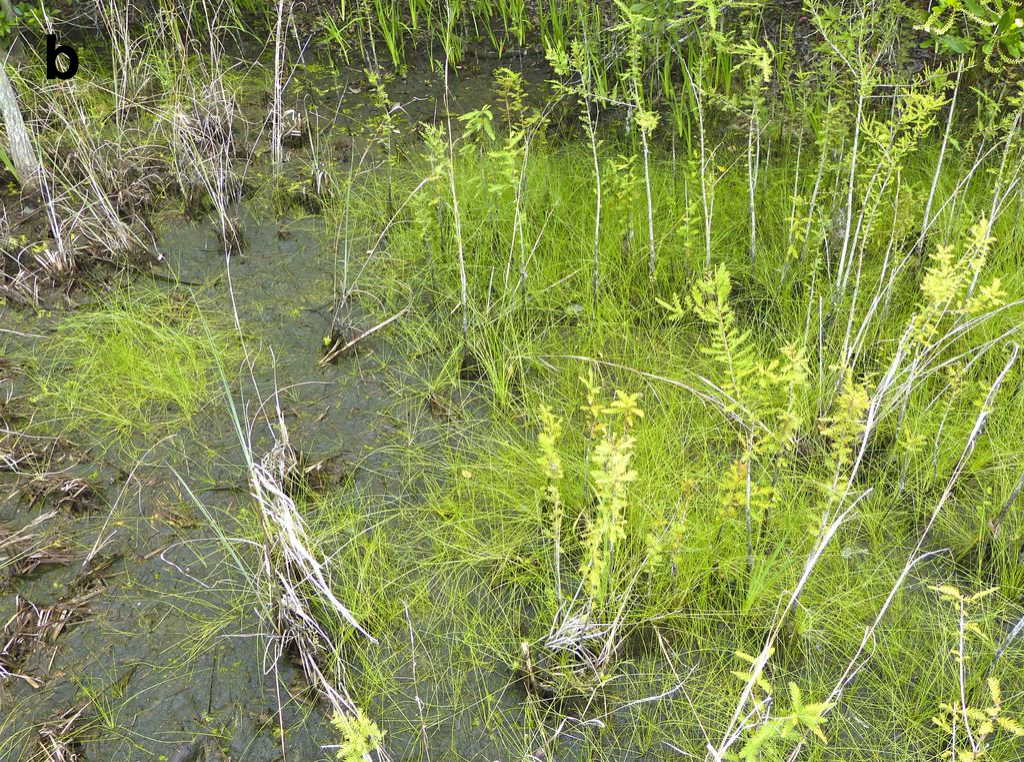
Habit

**Figure 50a.**
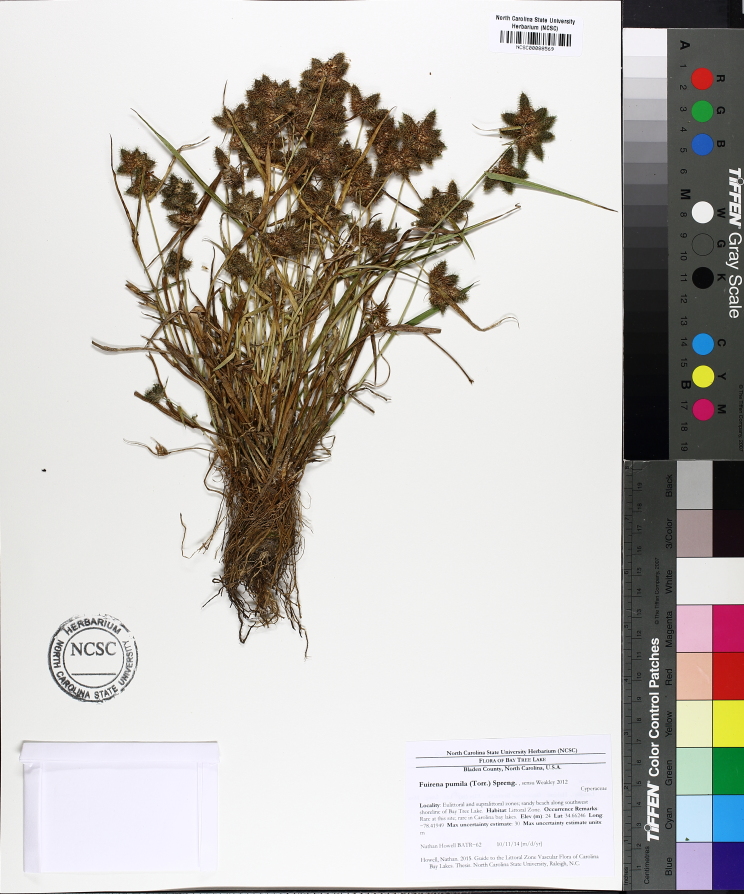
Specimen: *Howell BATR-62* (NCSC)

**Figure 50b.**
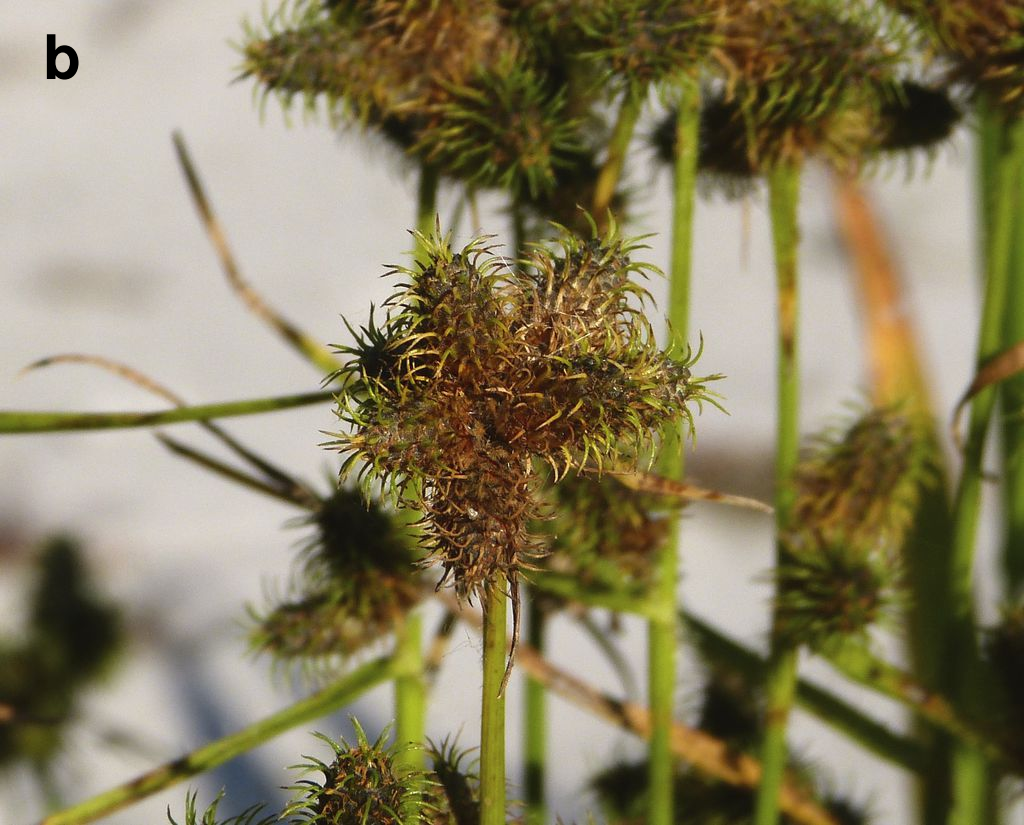
Inflorescence detail

**Figure 51a.**
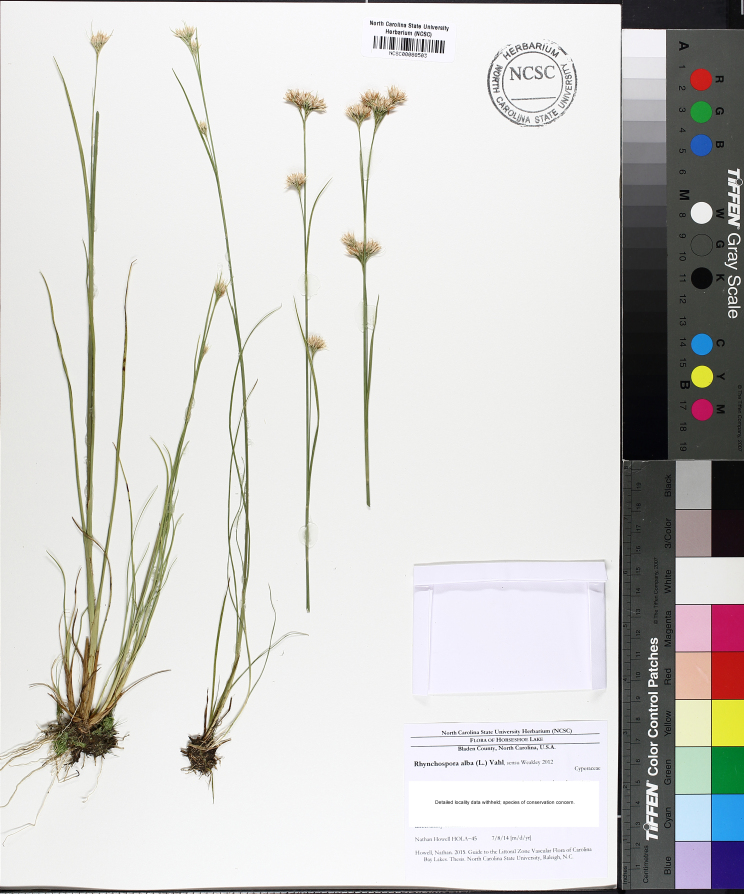
Specimen: *Howell HOLA-45* (NCSC)

**Figure 51b.**
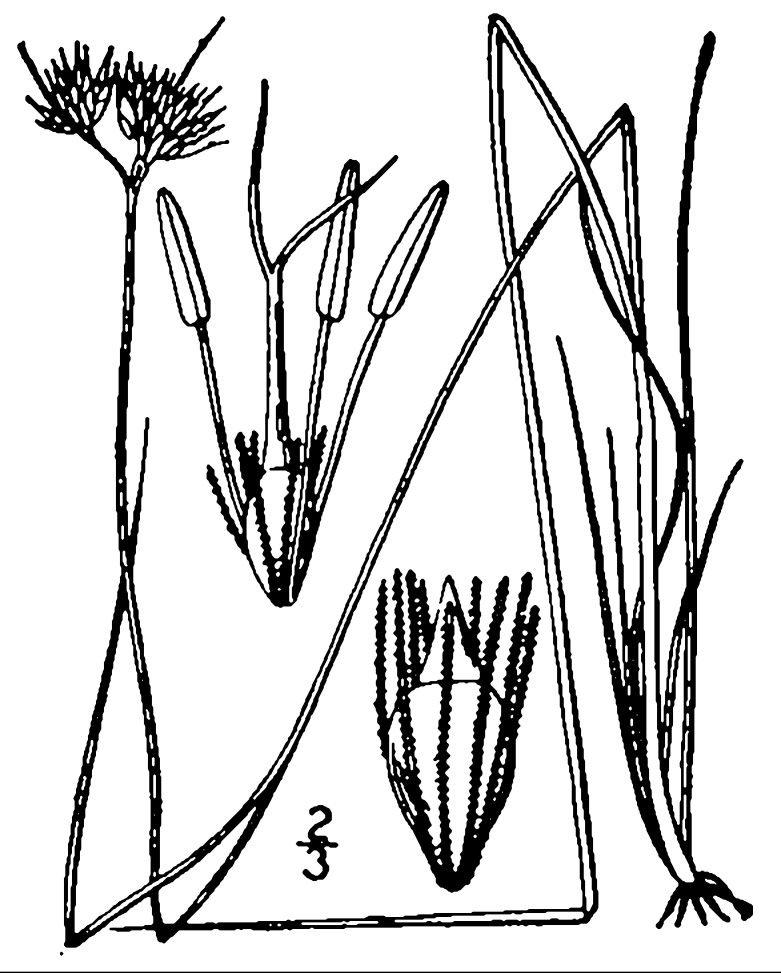
Illustration

**Figure 51c.**
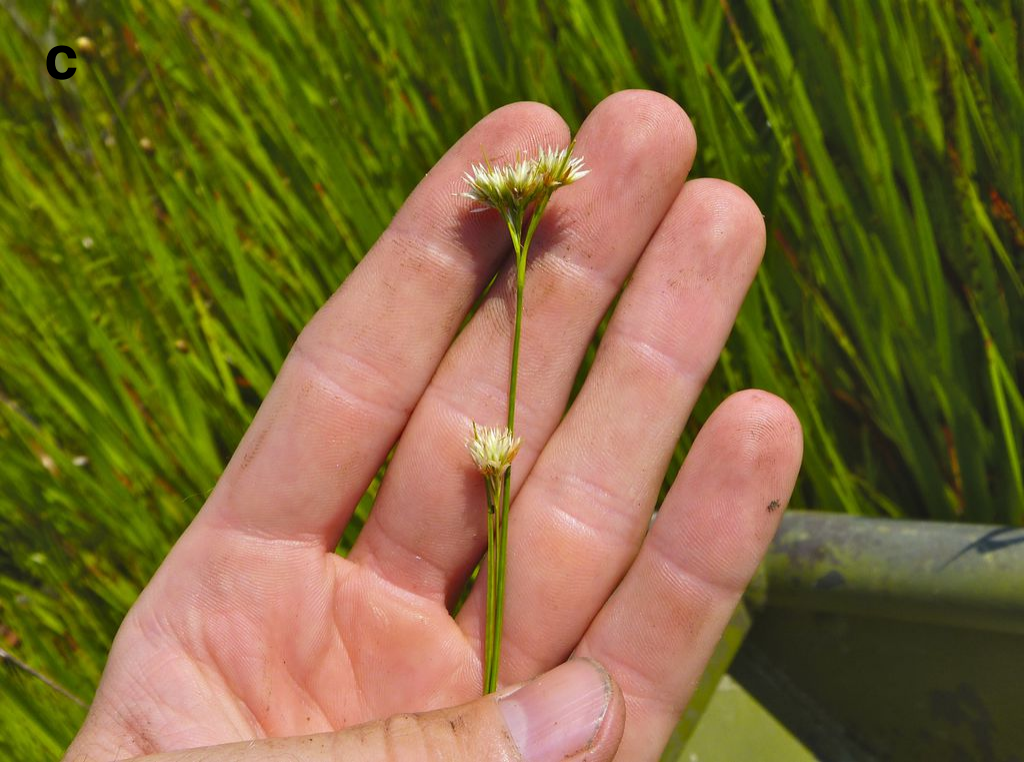
Inflorescences

**Figure 51d.**
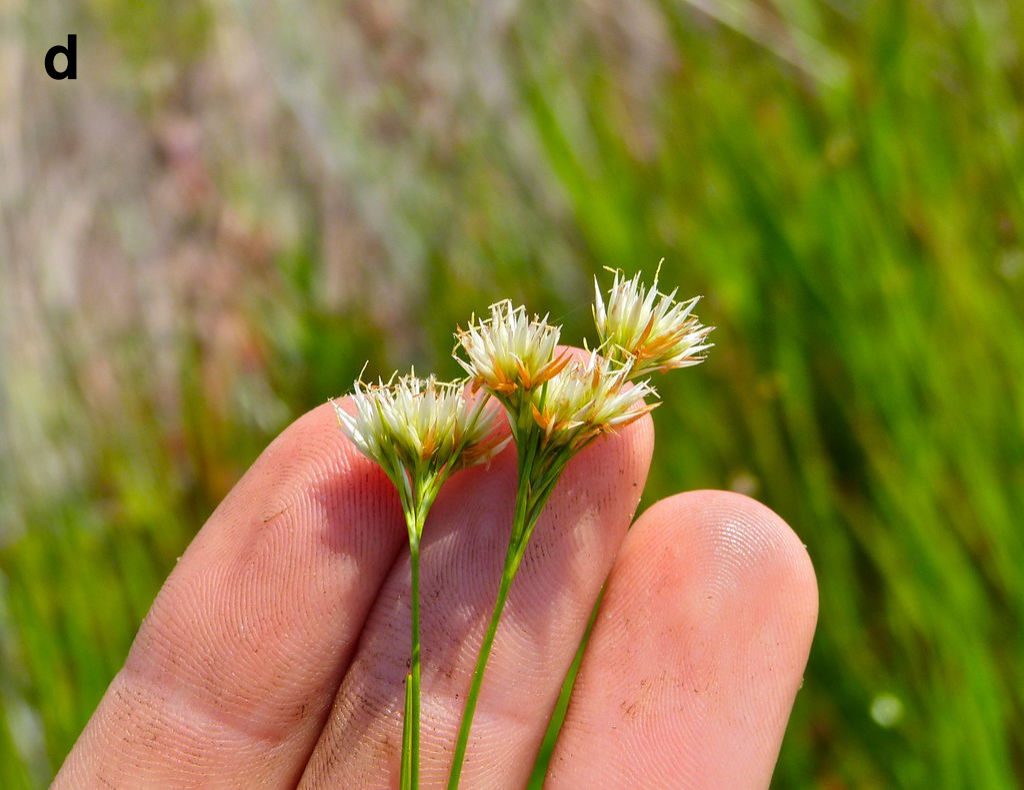
Inflorescences

**Figure 52a.**
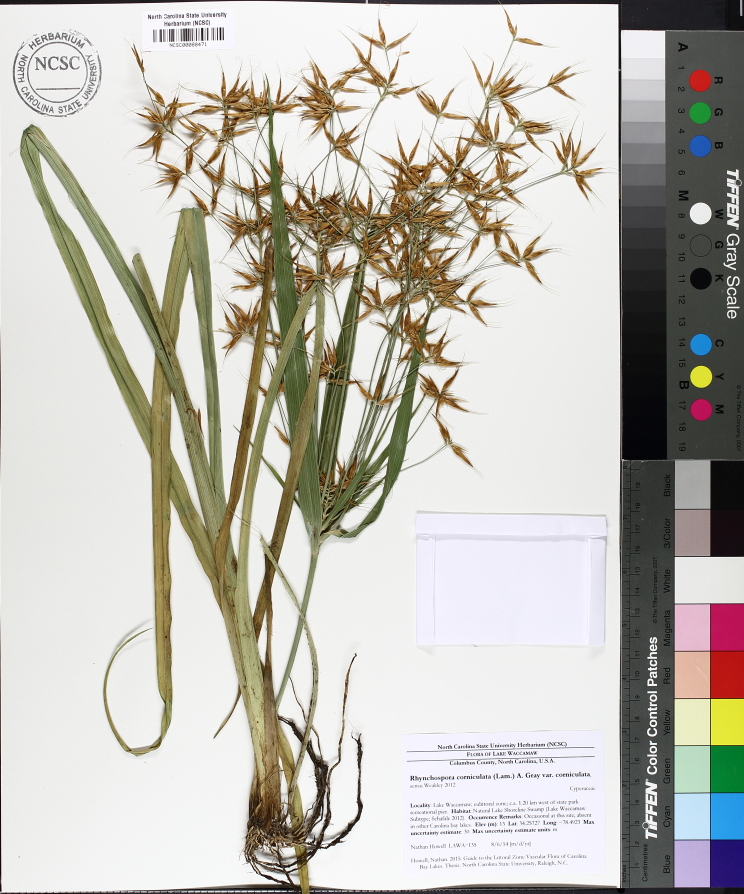
Specimen: *Howell LAWA-135* (NCSC)

**Figure 52b.**
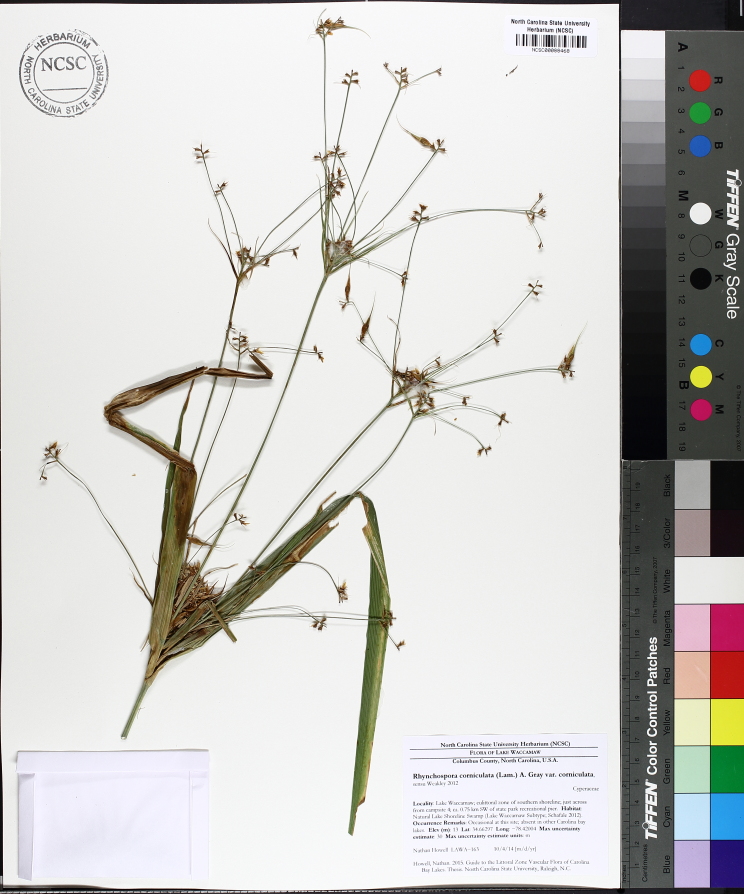
Specimen: *Howell LAWA-163* (NCSC)

**Figure 52c.**
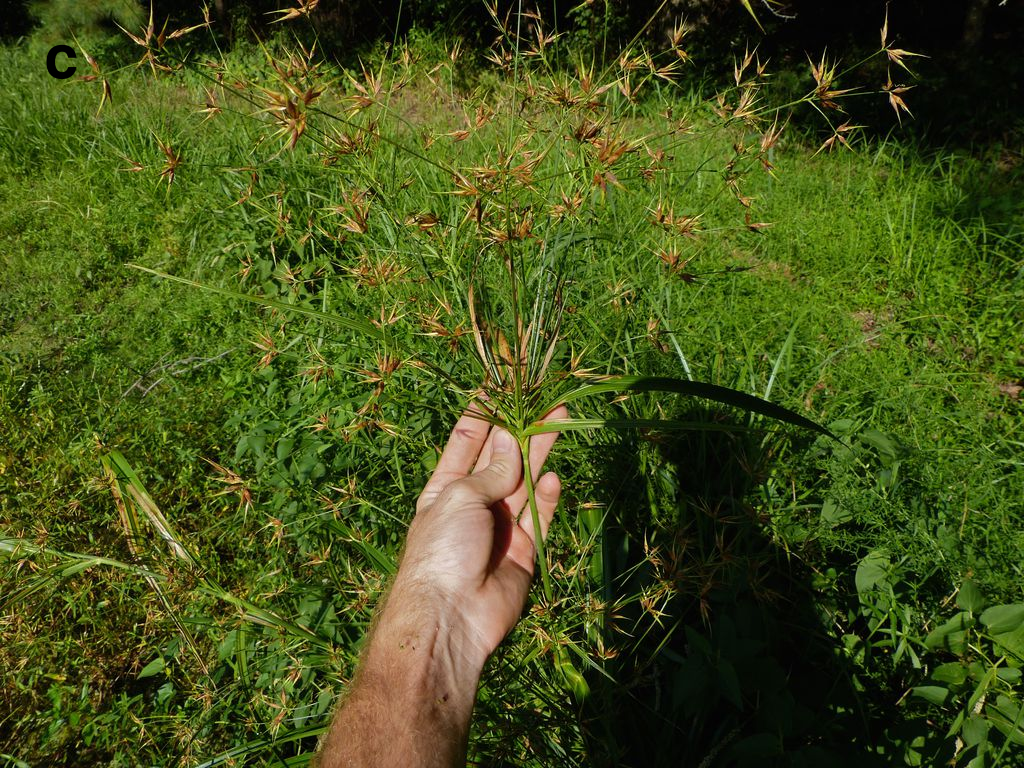
Inflorescence

**Figure 52d.**
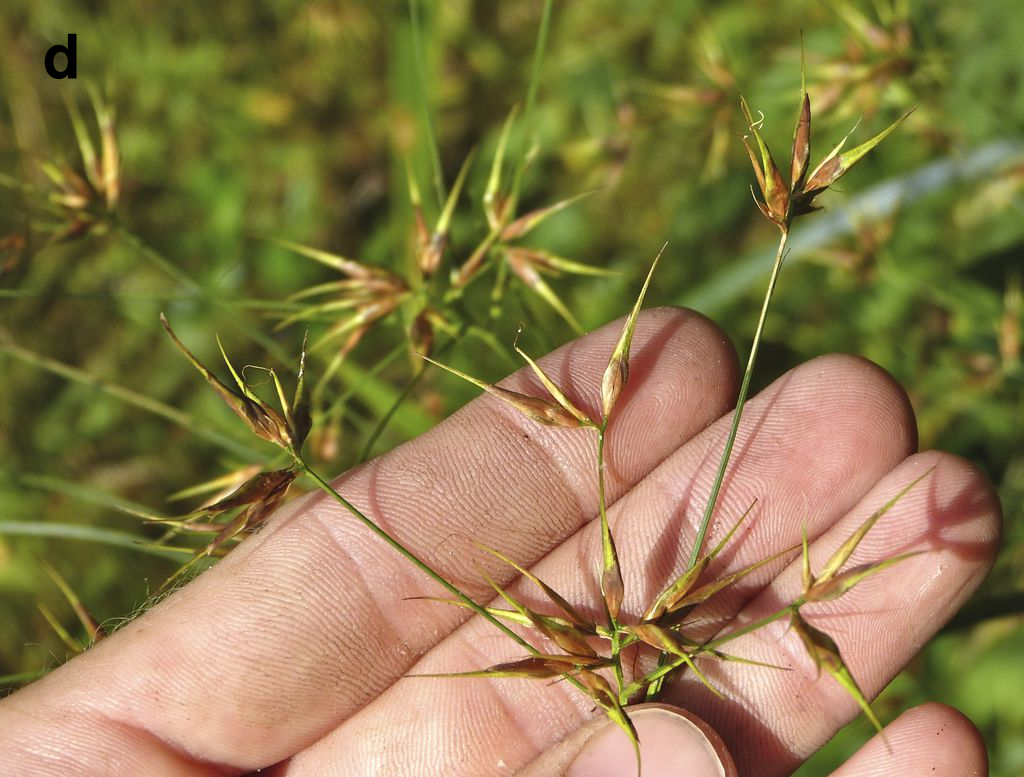
Inflorescence detail

**Figure 55a.**
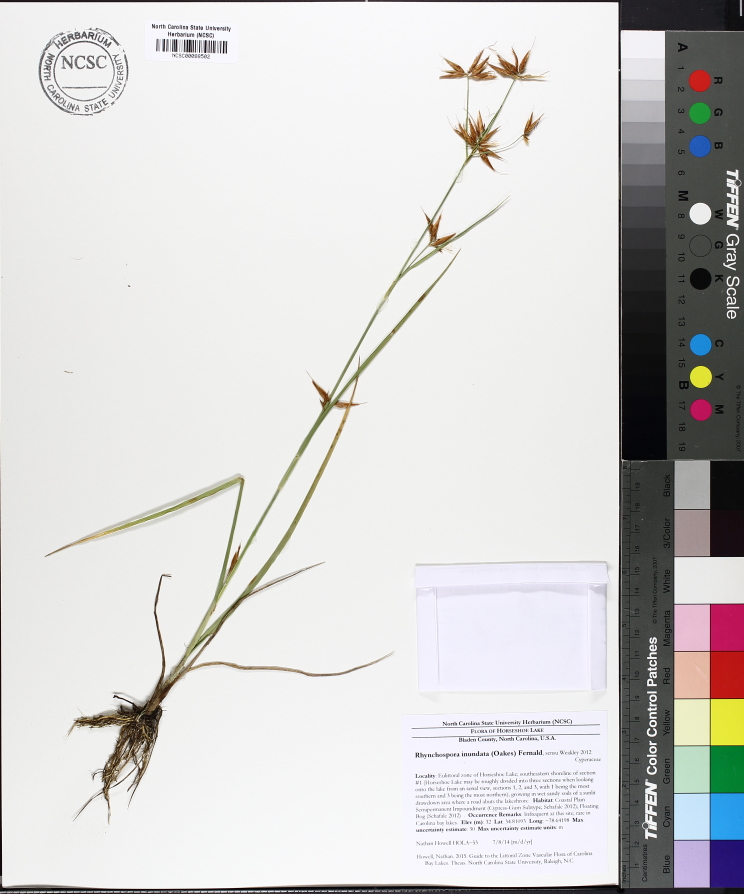
Specimen: *Howell HOLA-53* (NCSC)

**Figure 55b.**
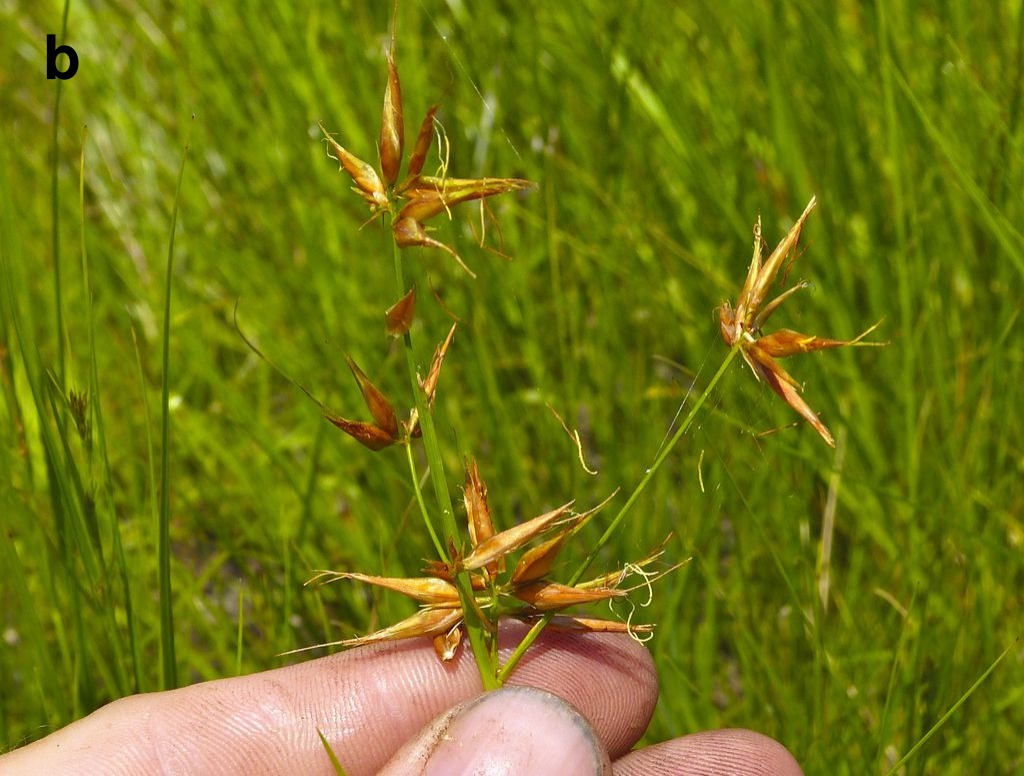
Inflorescence

**Figure 57a.**
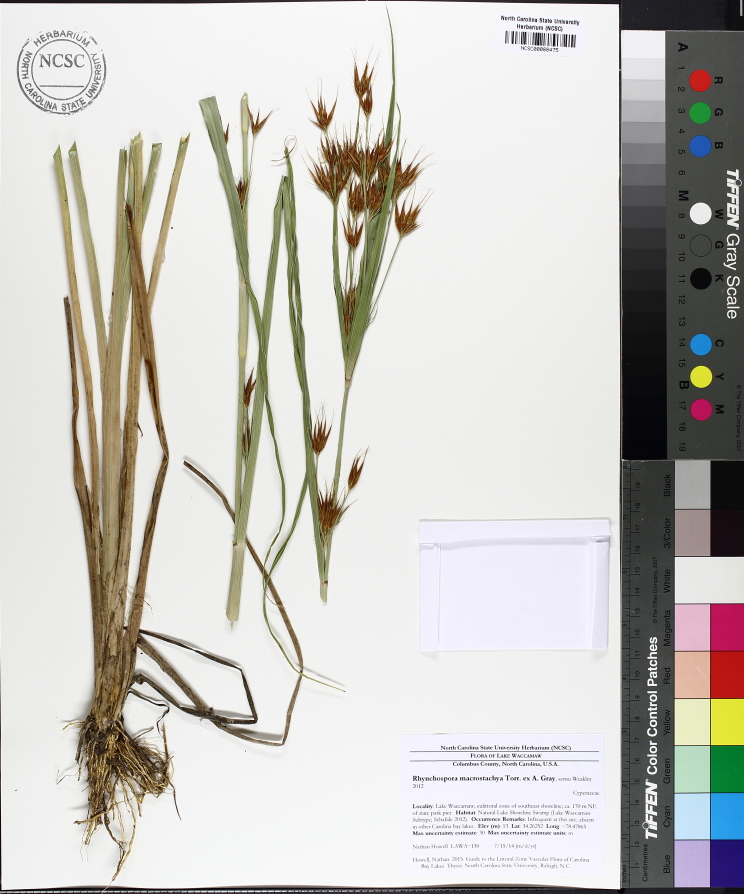
Specimen: *Howell LAWA-130* (NCSC)

**Figure 57b.**
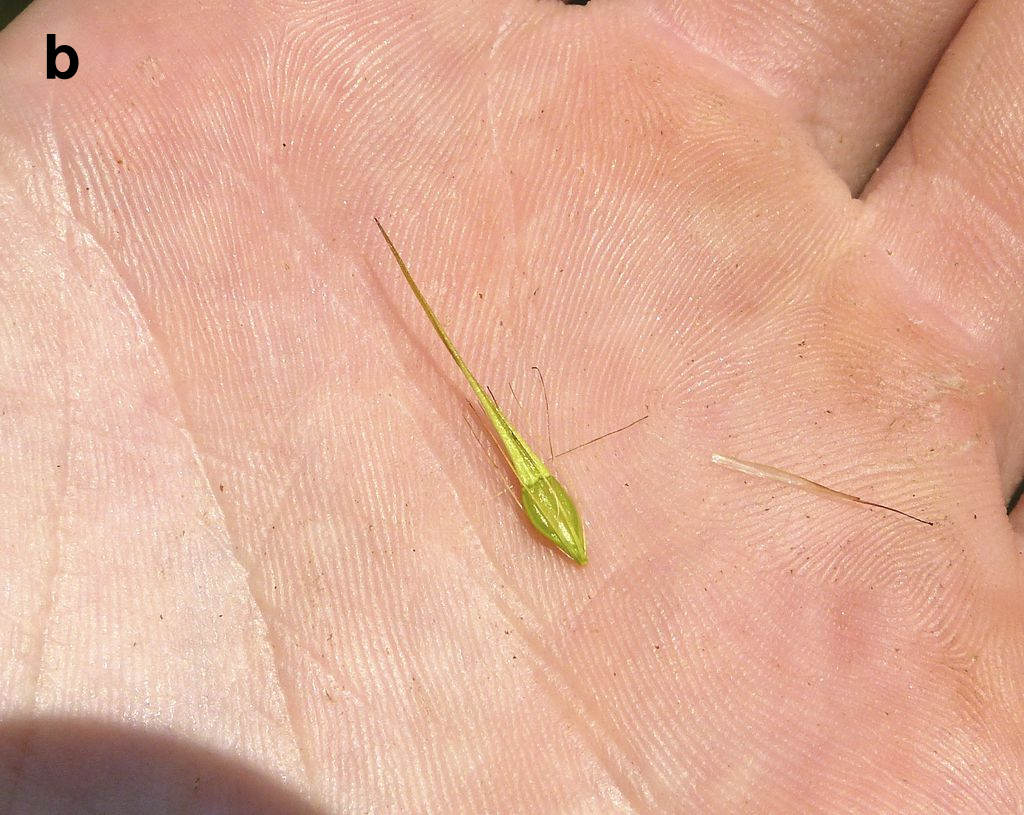
Achene detail

**Figure 59a.**
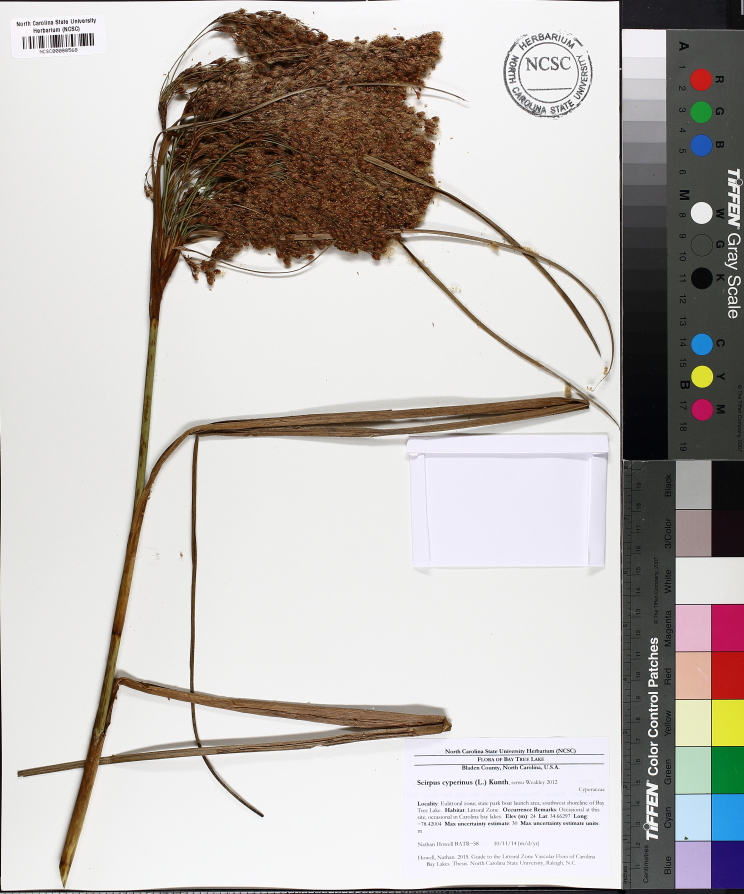
Specimen: *Howell BATR-58* (NCSC)

**Figure 59b.**
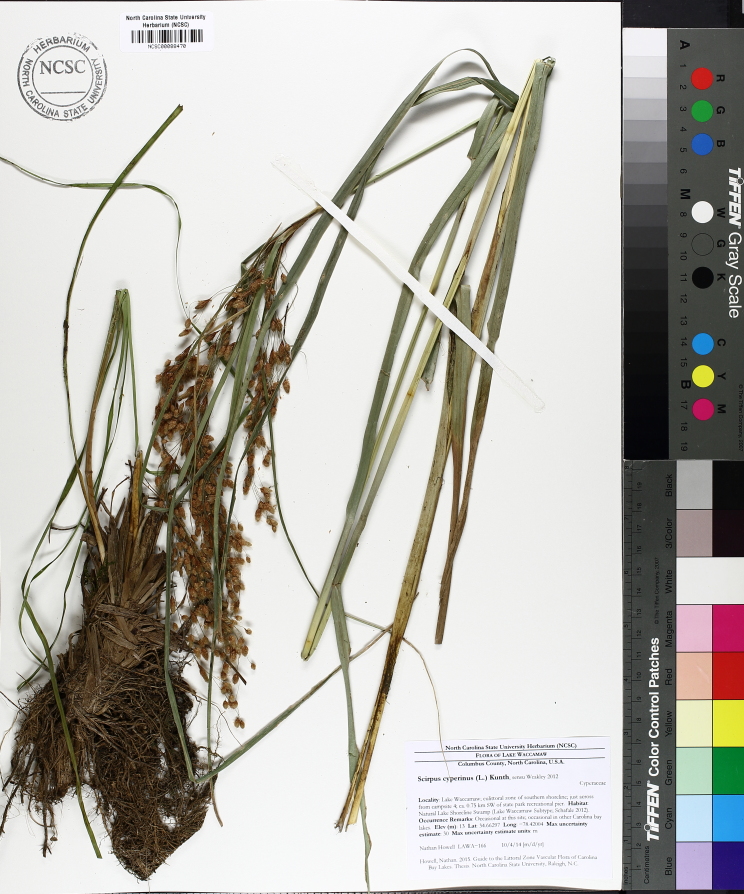
Specimen: *Howell LAWA-166* (NCSC)

**Figure 59c.**
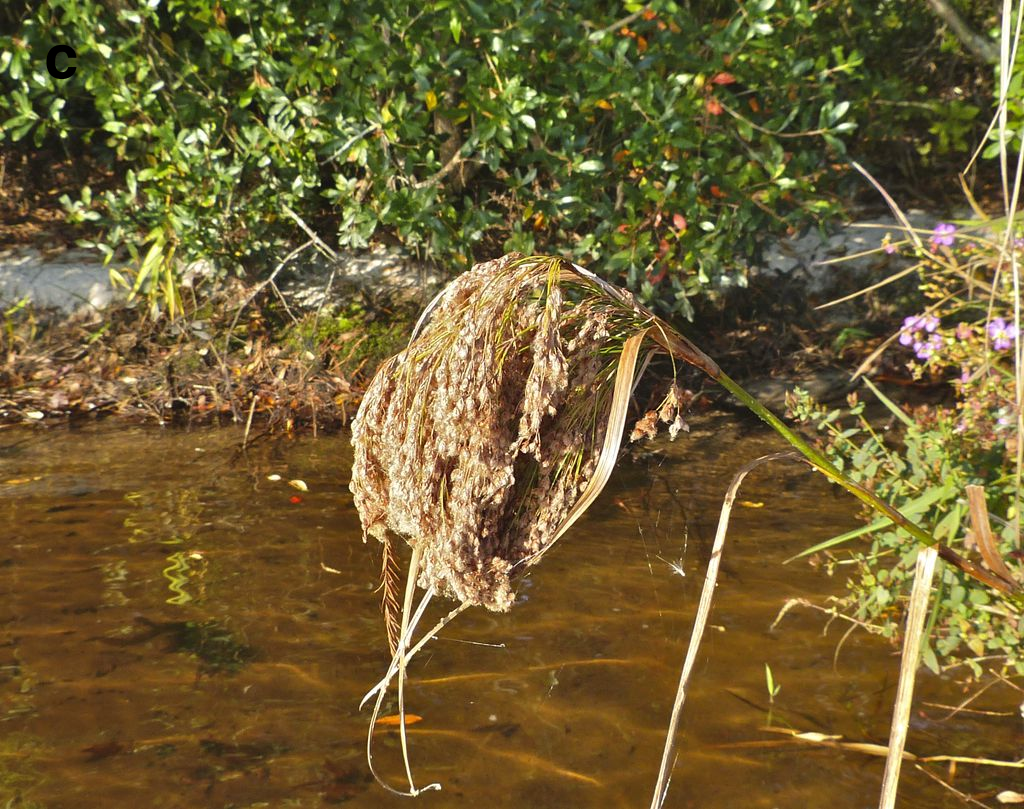
Inflorescence

**Figure 59d.**
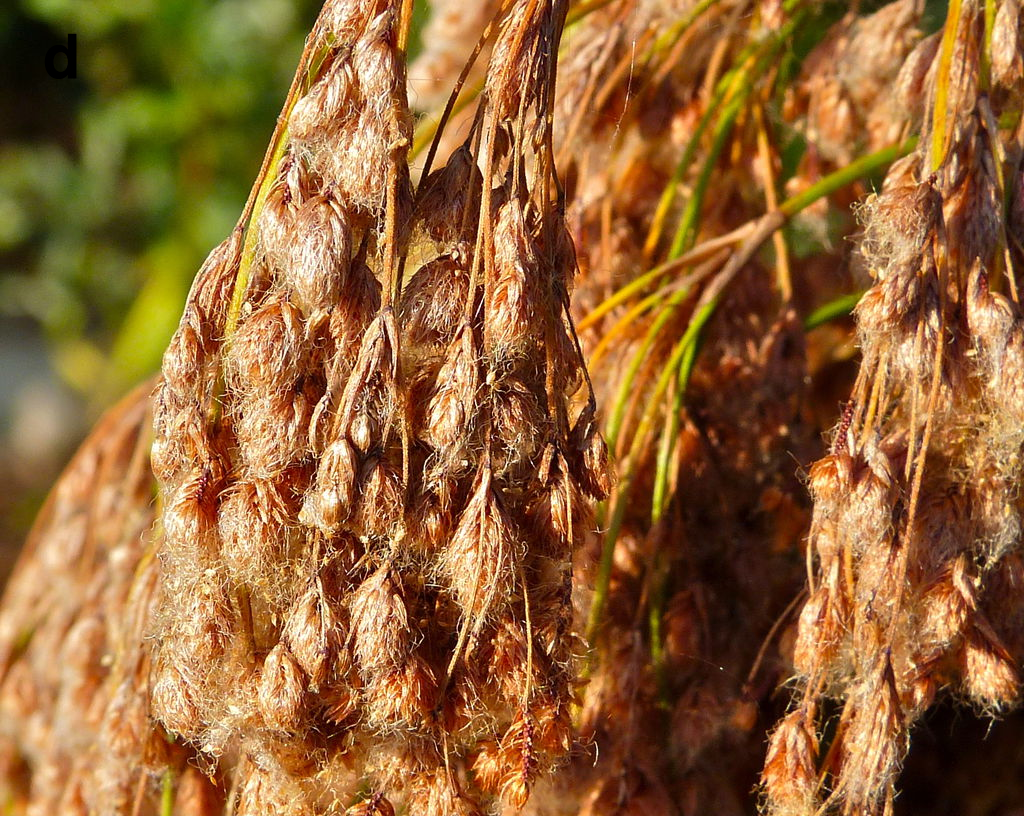
Inflorescence detail

**Figure 60a.**
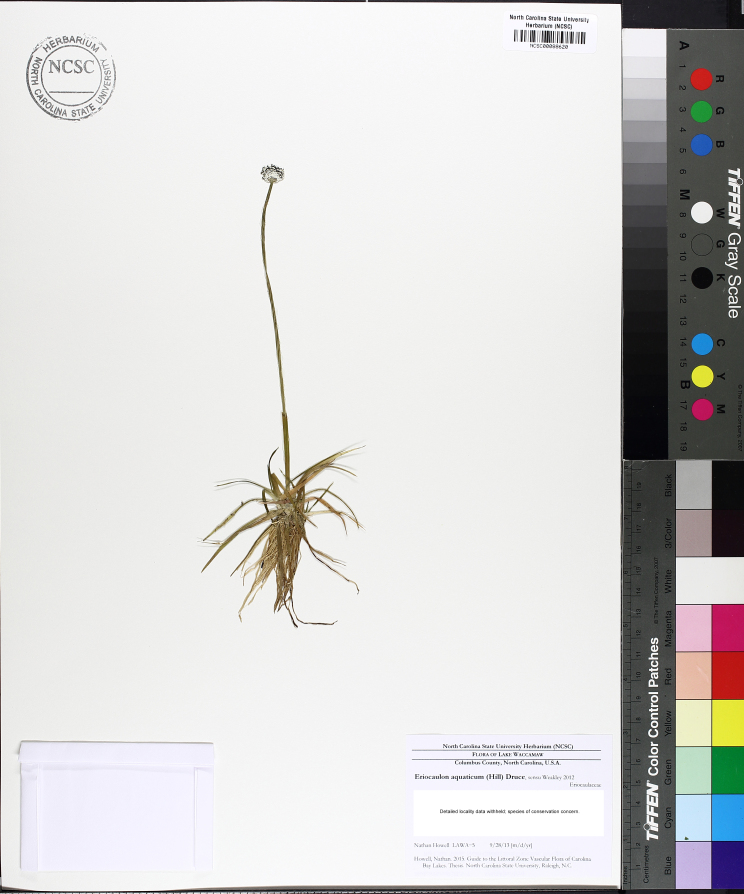
Specimen: *Howell LAWA-5* (NCSC)

**Figure 60b.**
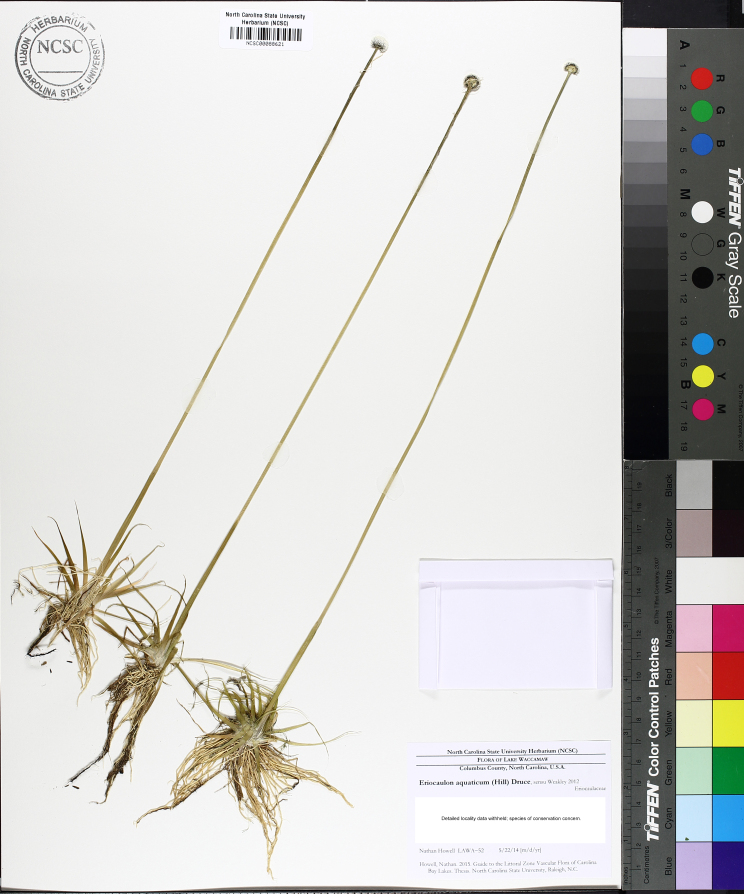
Specimen: *Howell LAWA-52* (NCSC)

**Figure 60c.**
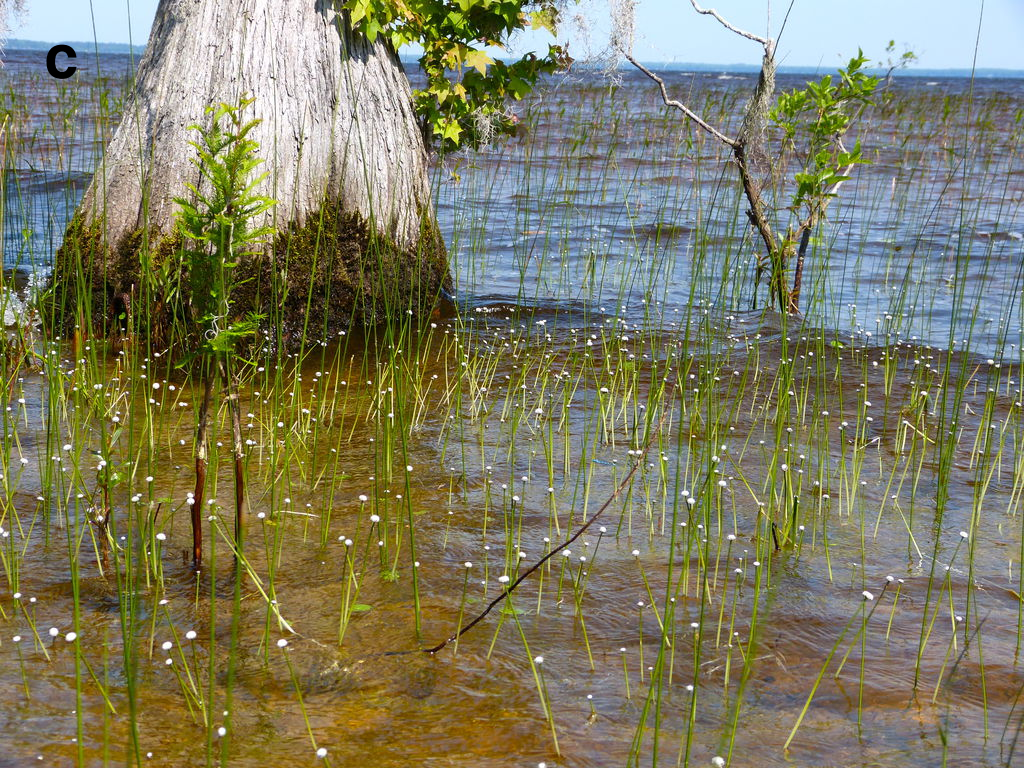
Habit

**Figure 60d.**
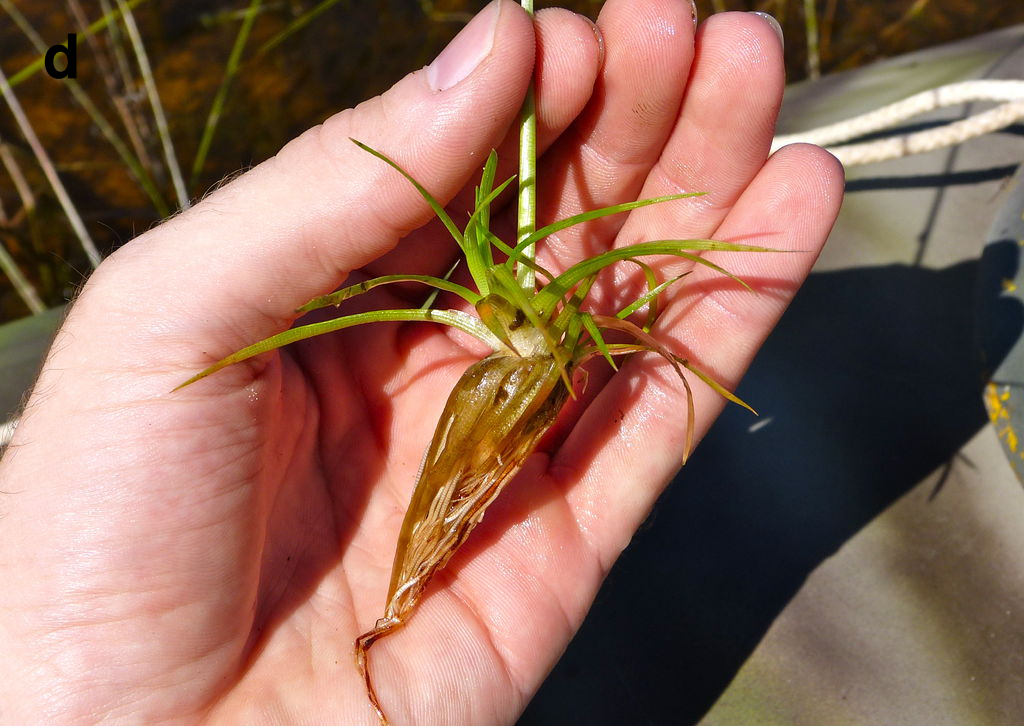
Leaves

**Figure 60e.**
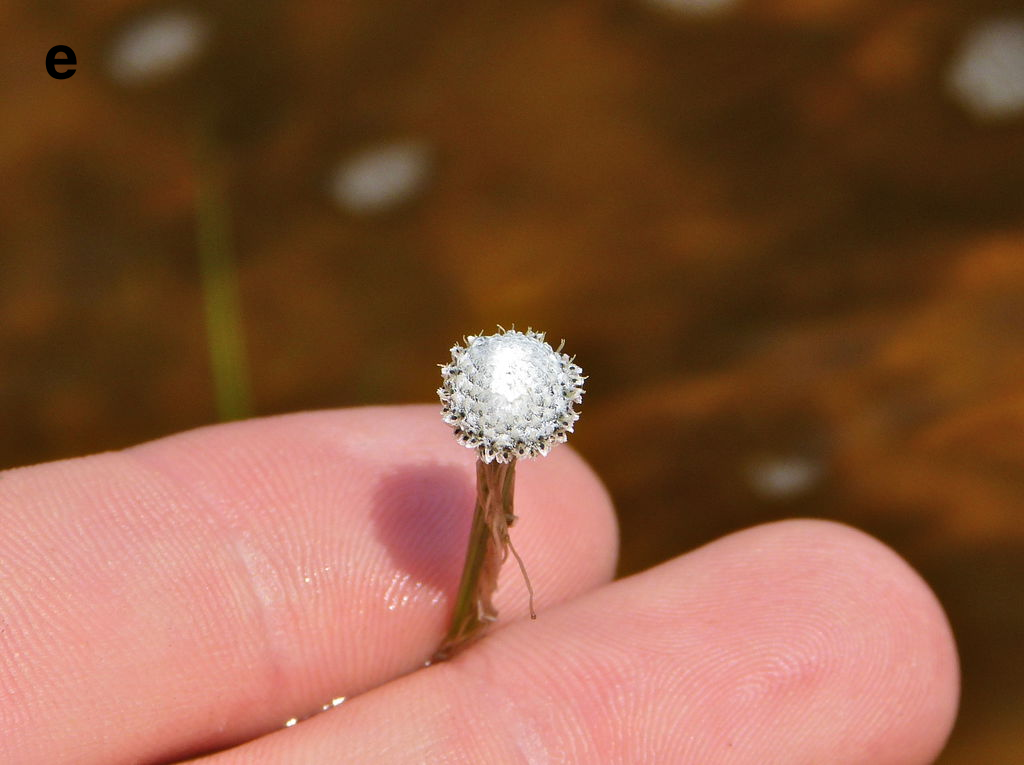
Inflorescence

**Figure 60f.**
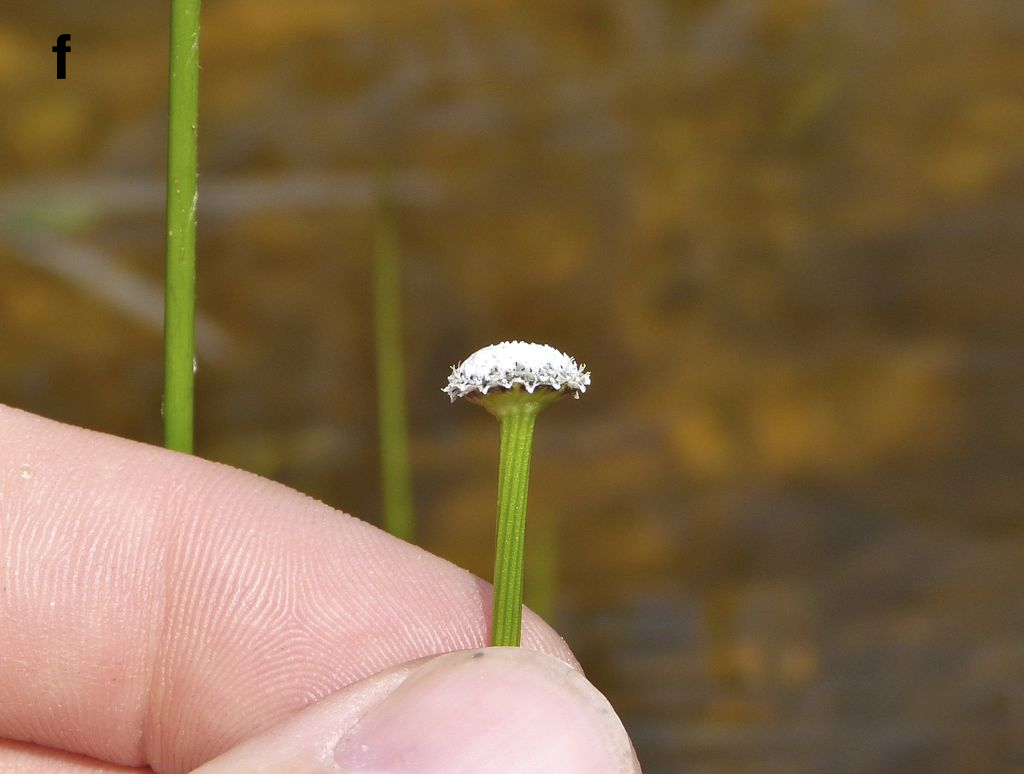
Inflorescence

**Figure 61a.**
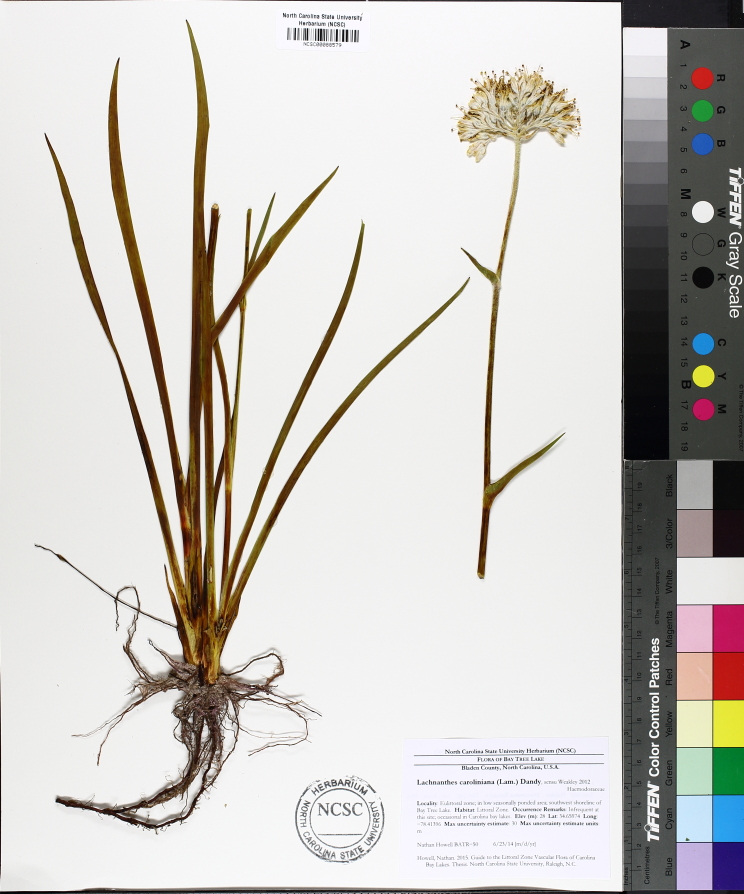
Specimen: *Howell BATR-50* (NCSC)

**Figure 61b.**
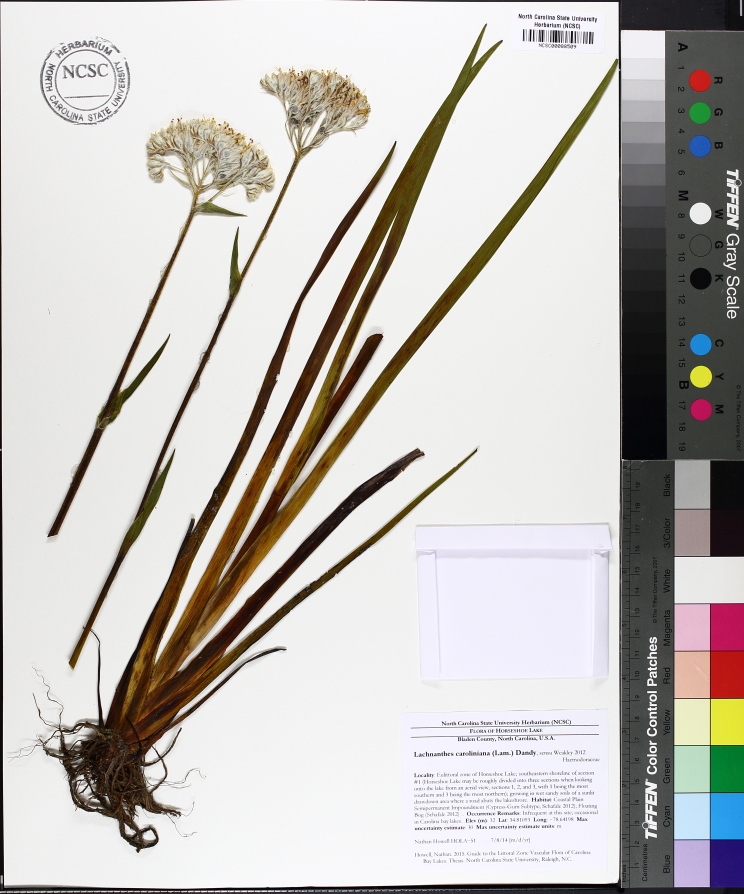
Specimen: *Howell HOLA-51* (NCSC)

**Figure 61c.**
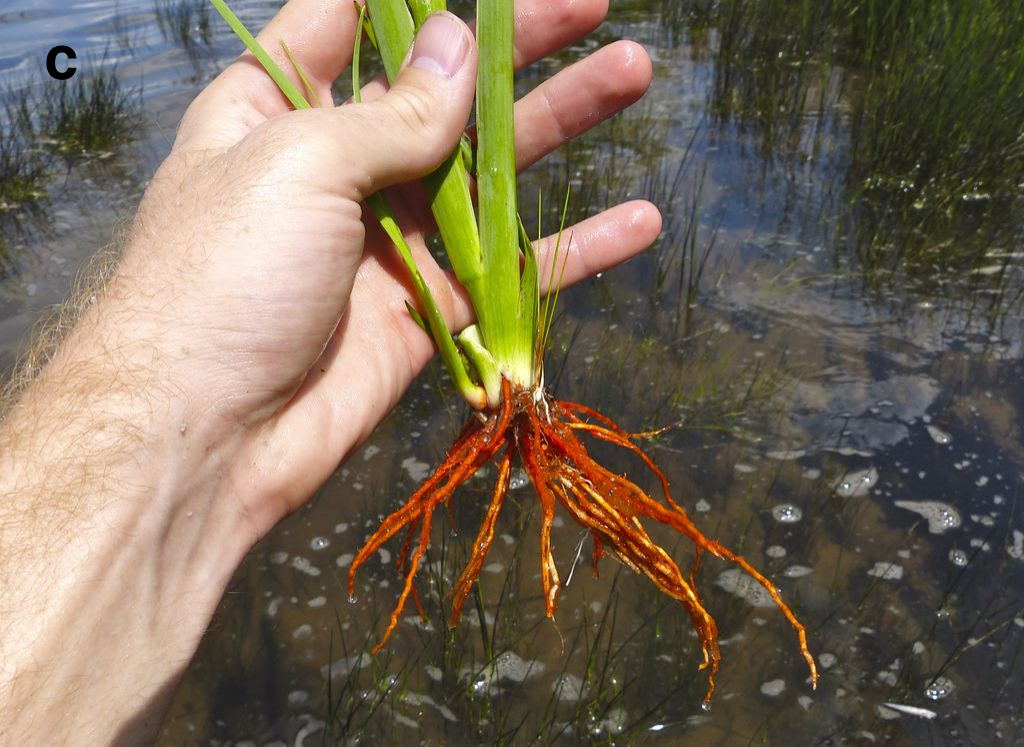
Roots

**Figure 61d.**
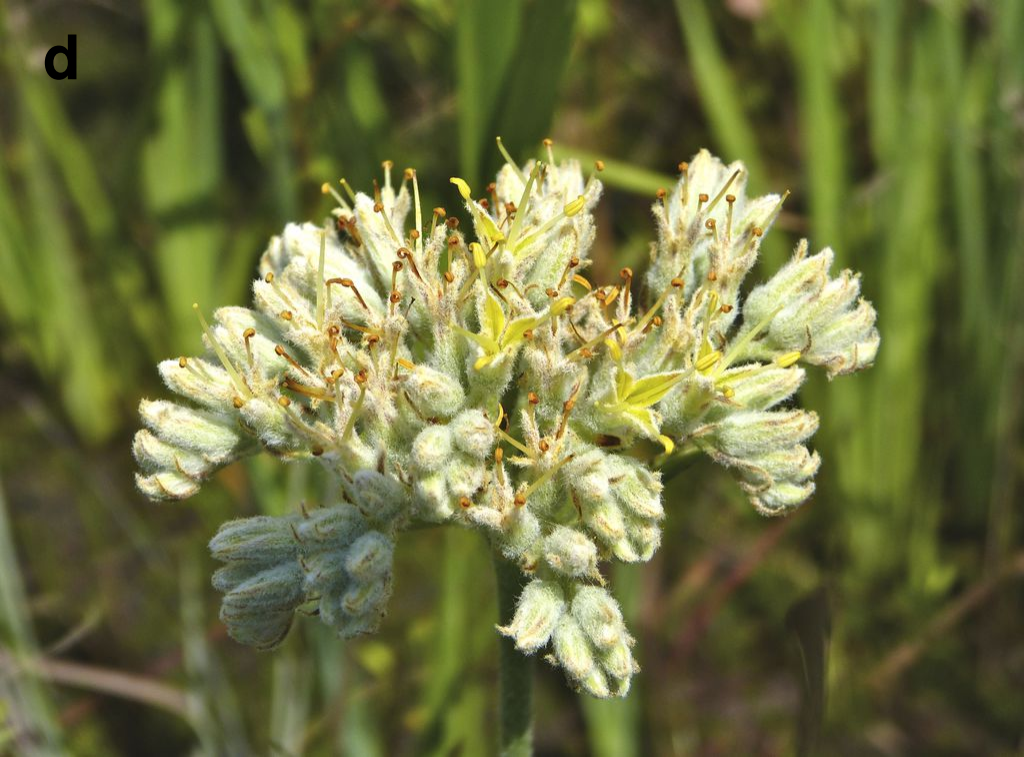
Inflorescence

**Figure 64a.**
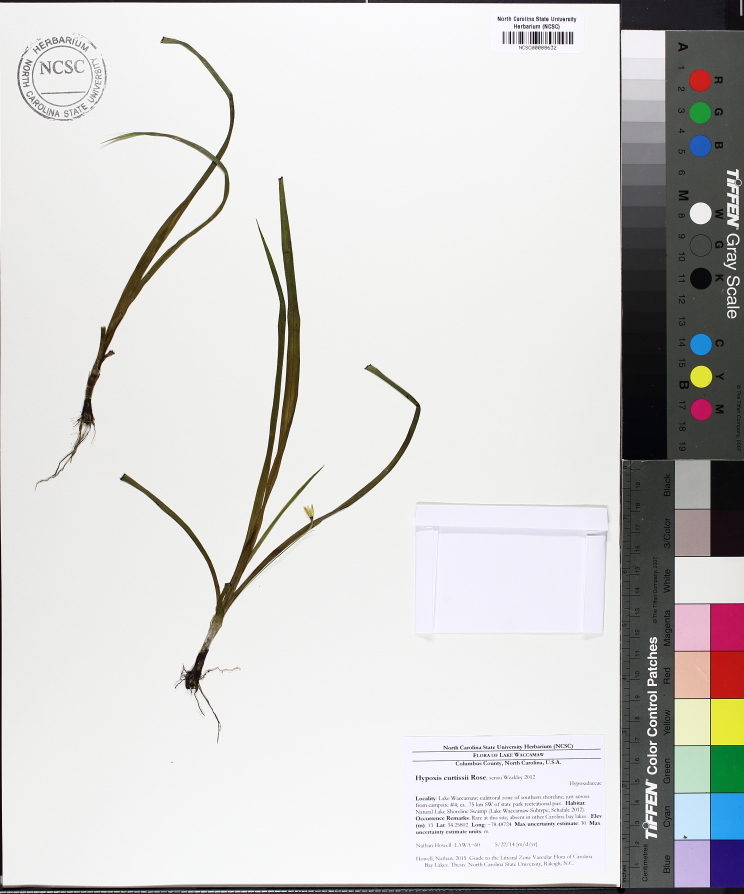
Specimen: *Howell LAWA-60* (NCSC)

**Figure 64b.**
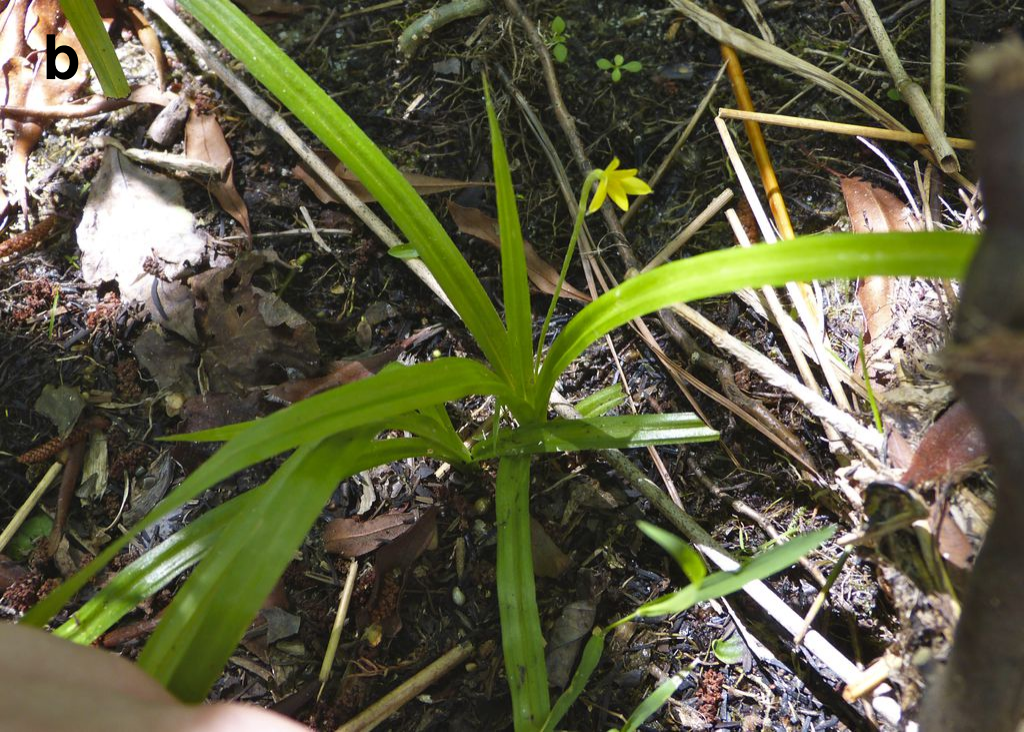
Habit

**Figure 64c.**
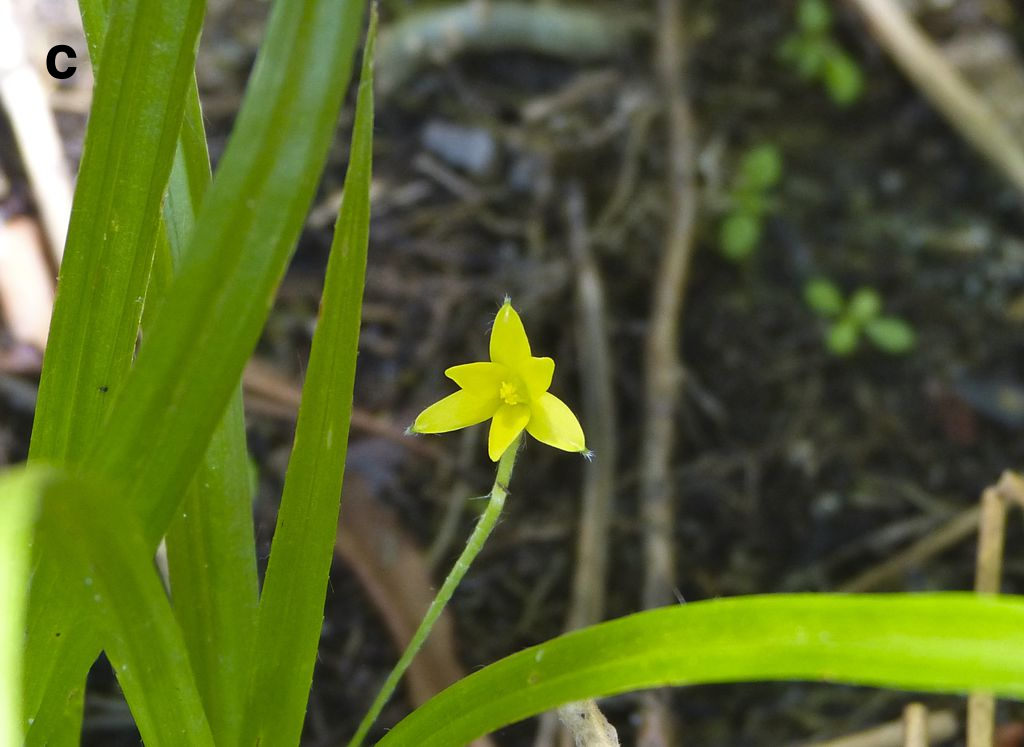
Flower

**Figure 64d.**
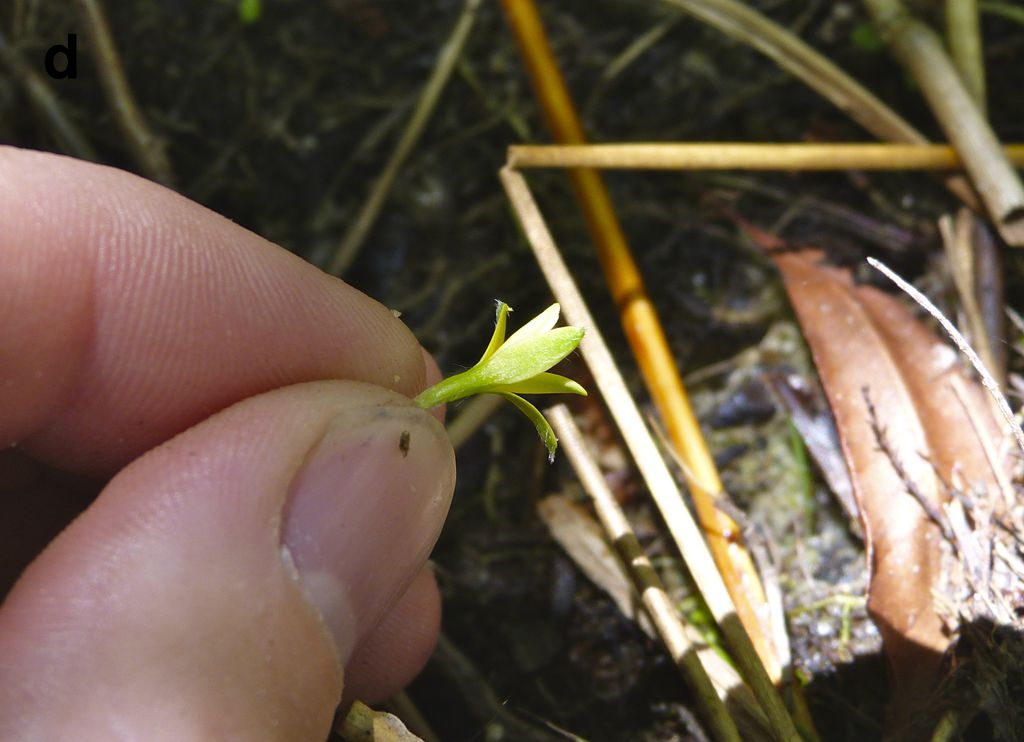
Flower

**Figure 65a.**
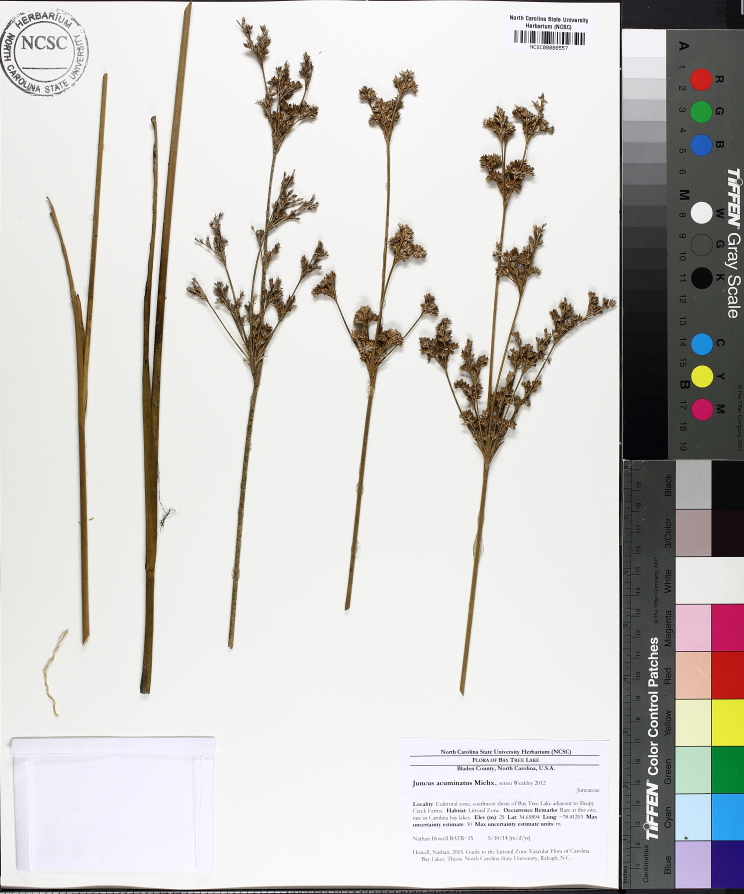
Specimen: *Howell BATR-15* (NCSC)

**Figure 65b.**
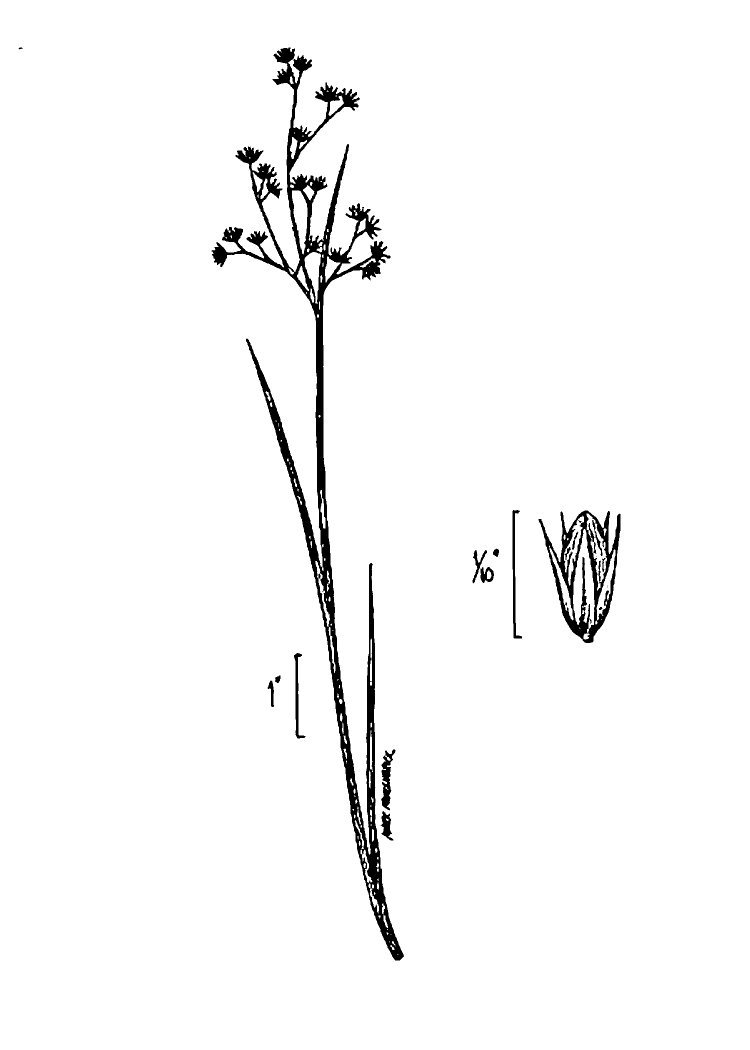
Illustration

**Figure 66a.**
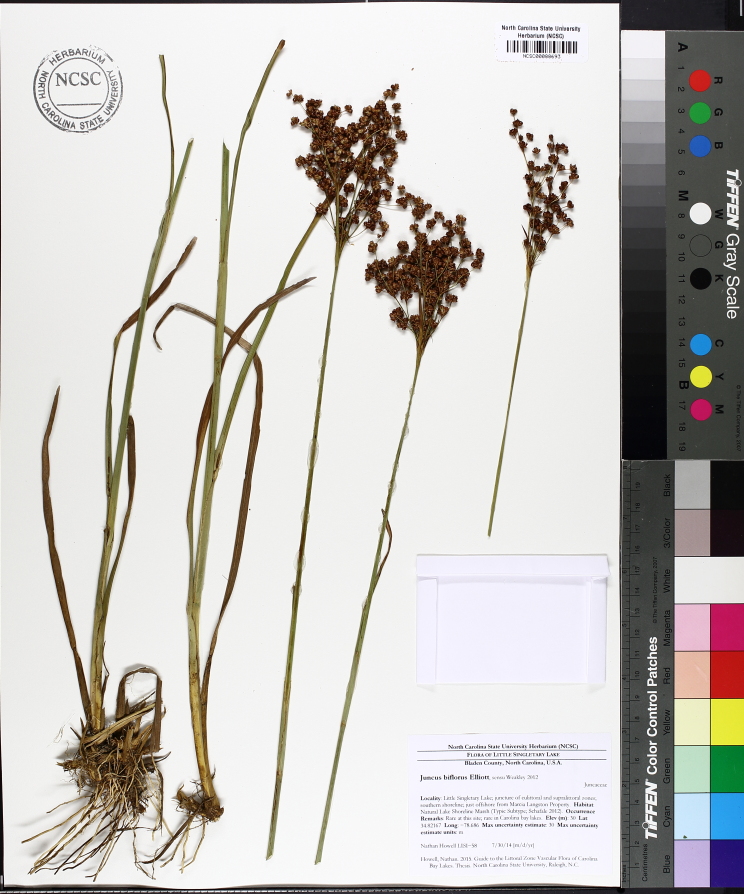
Specimen: *Howell LISI-58* (NCSC)

**Figure 66b.**
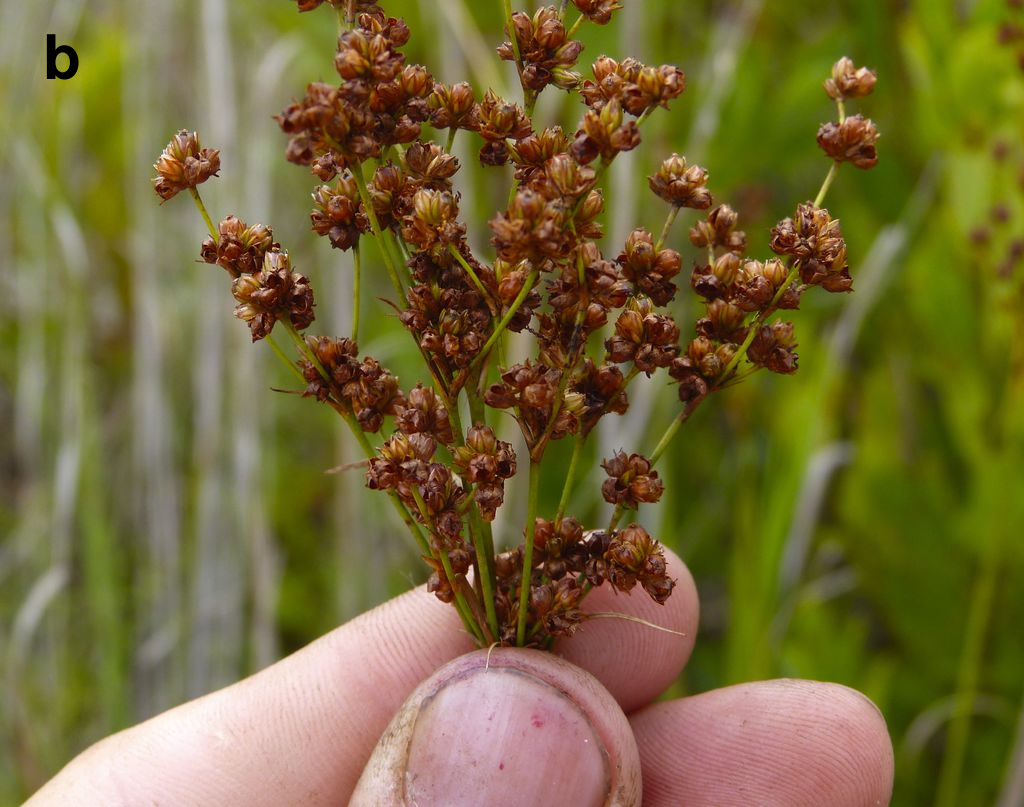
Inflorescence

**Figure 67a.**
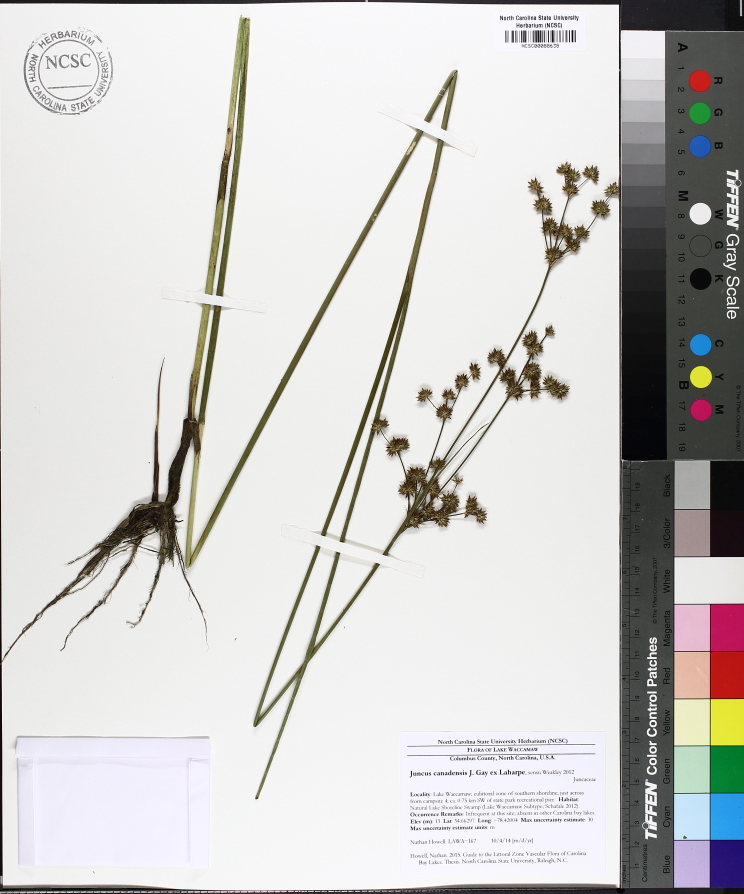
Specimen: *Howell LAWA-167* (NCSC)

**Figure 67b.**
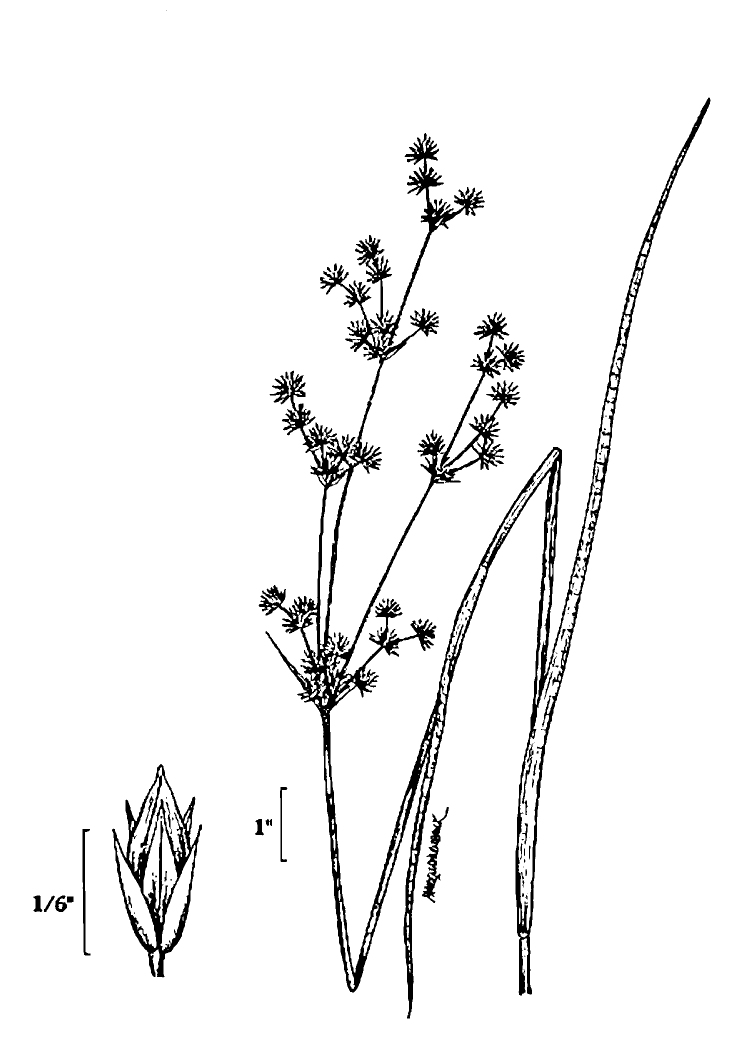
Illustration

**Figure 69a.**
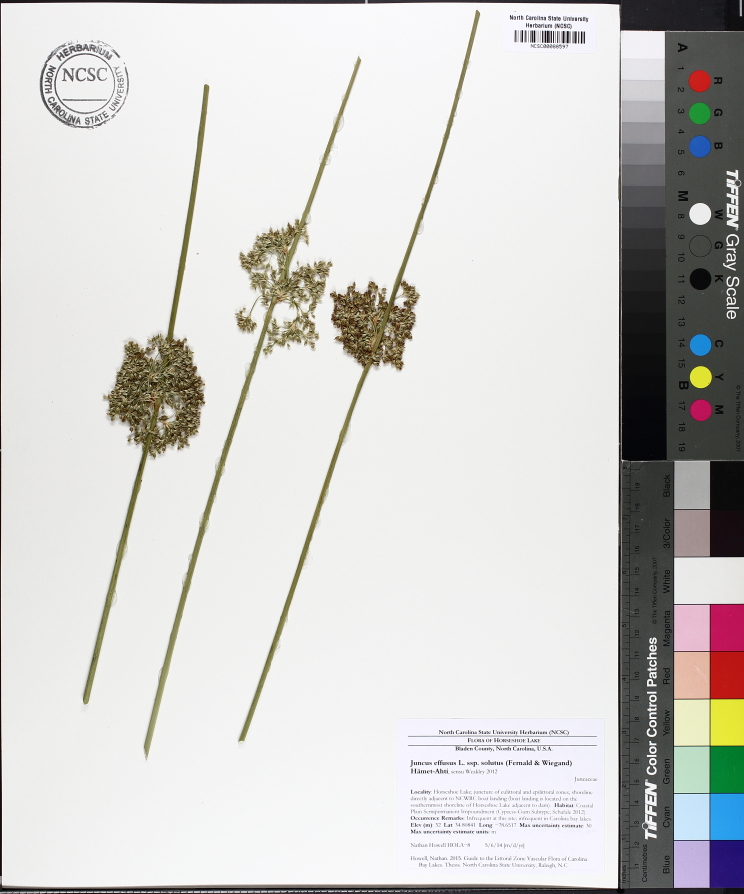
Specimen: *Howell HOLA-8* (NCSC)

**Figure 69b.**
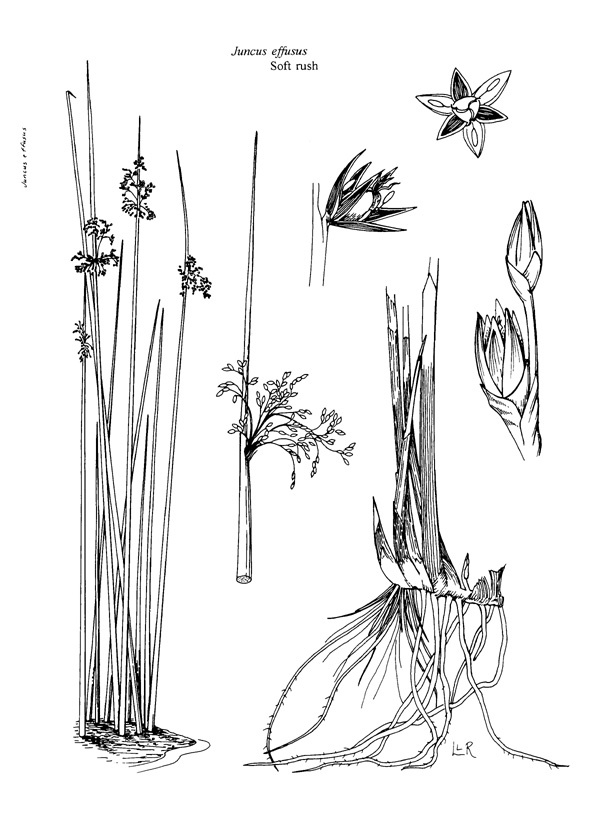
Illustration

**Figure 69c.**
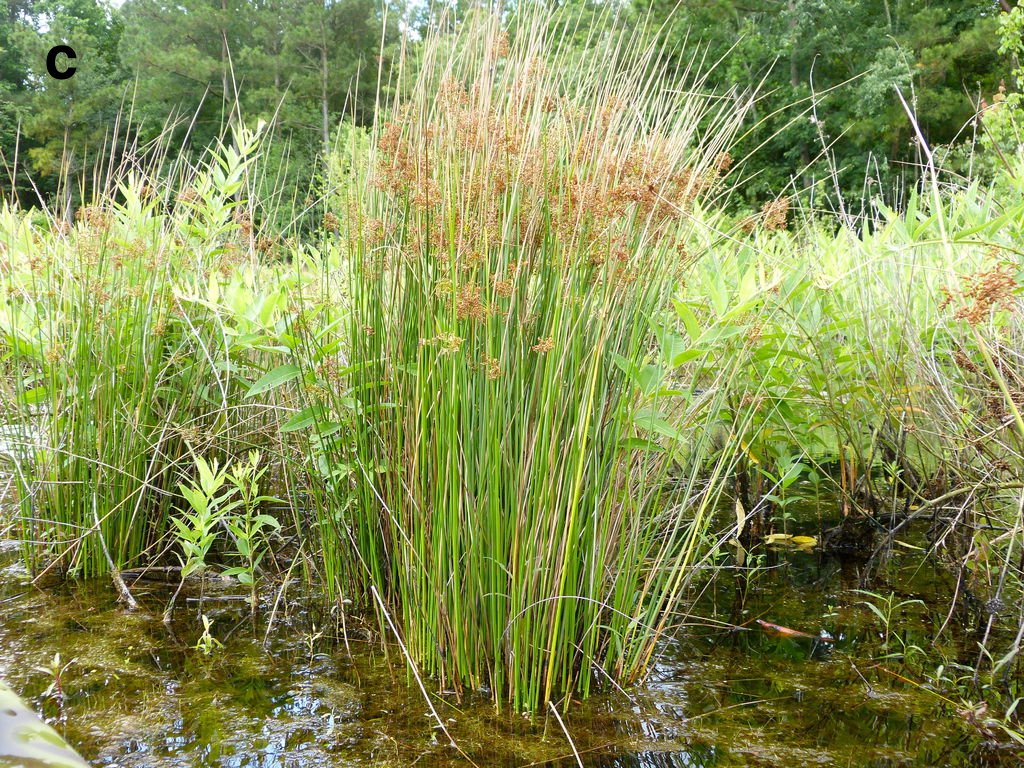
Habit

**Figure 69d.**
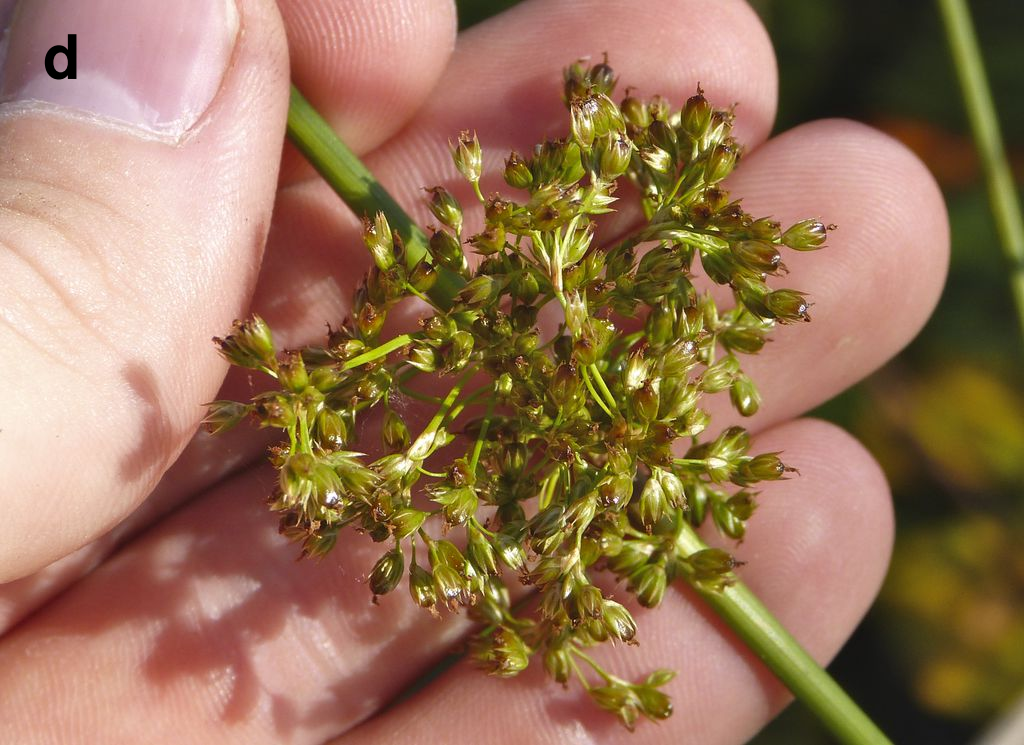
Inflorescence

**Figure 70a.**
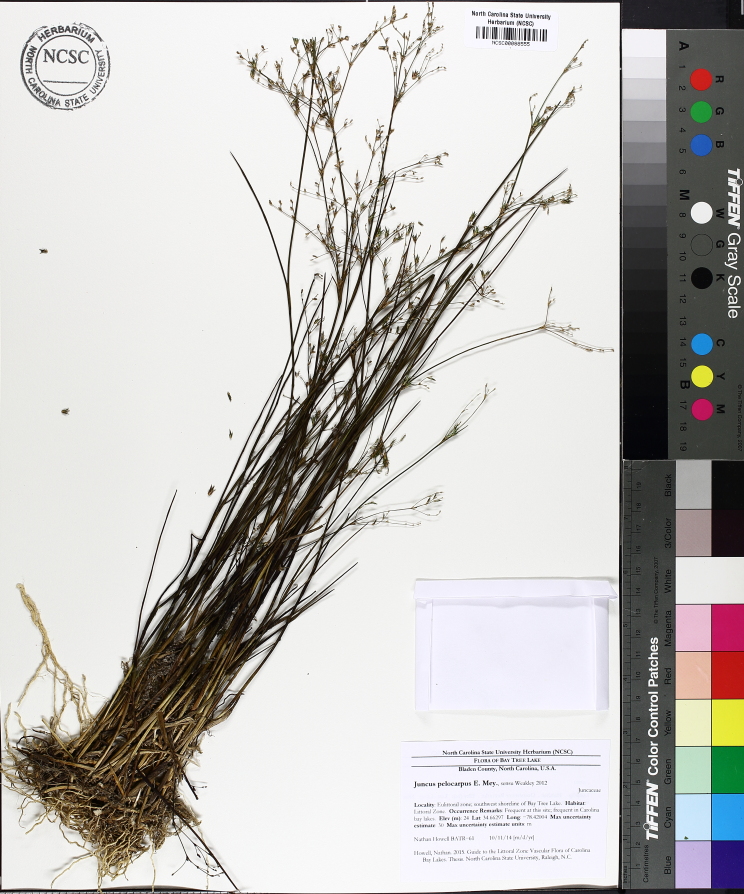
Specimen: *Howell BATR-61* (NCSC)

**Figure 70b.**
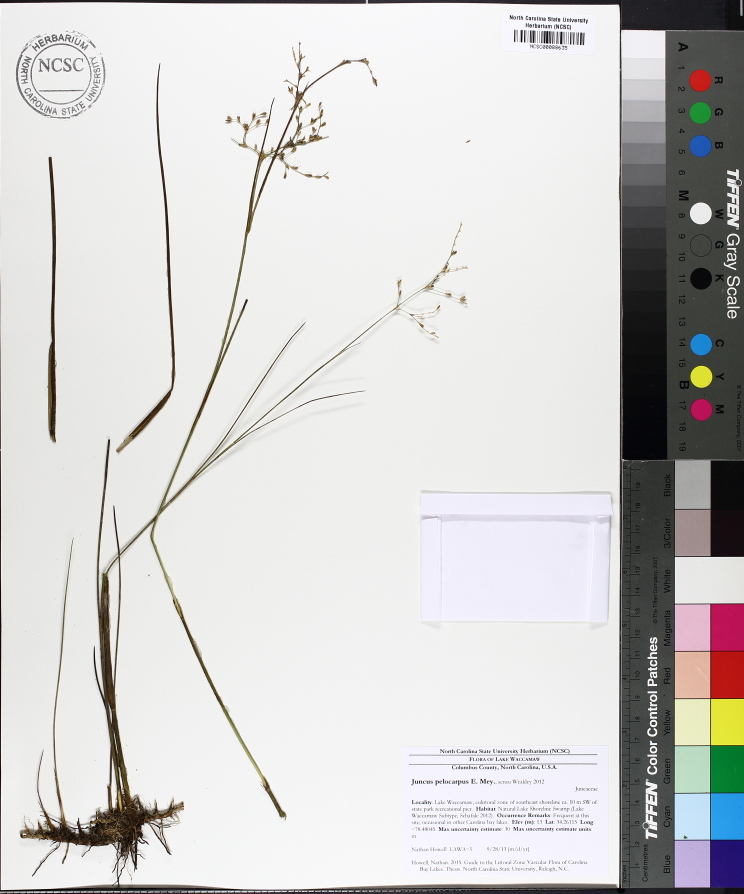
Specimen: *Howell LAWA-3* (NCSC)

**Figure 70c.**
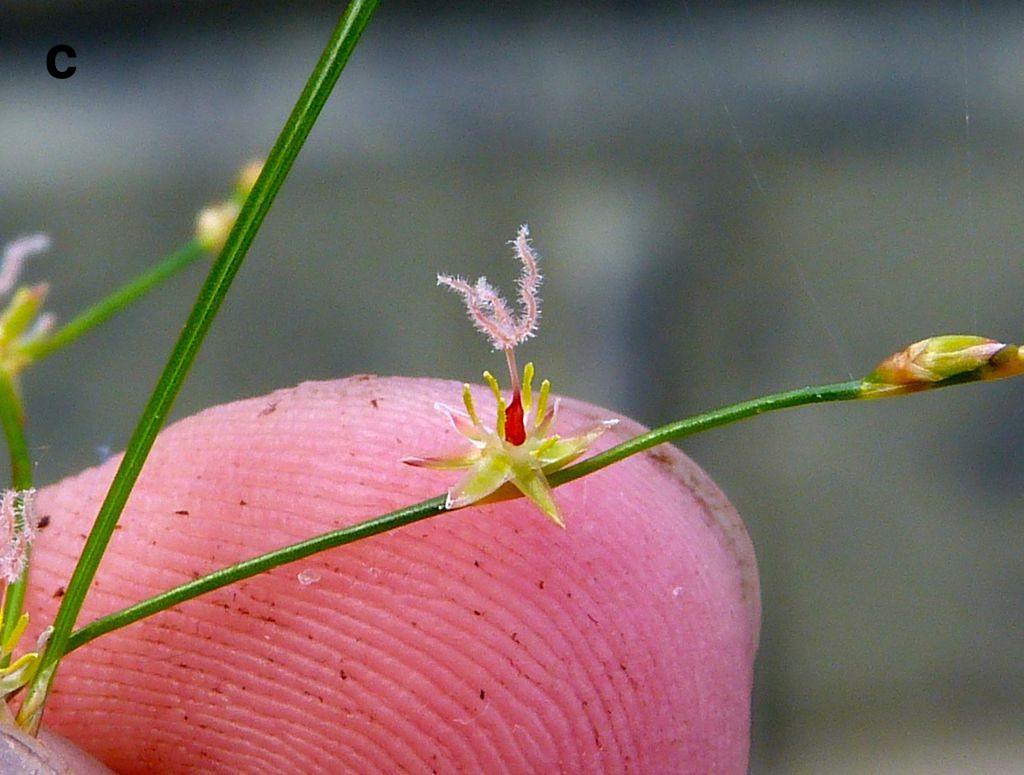
Flower

**Figure 70d.**
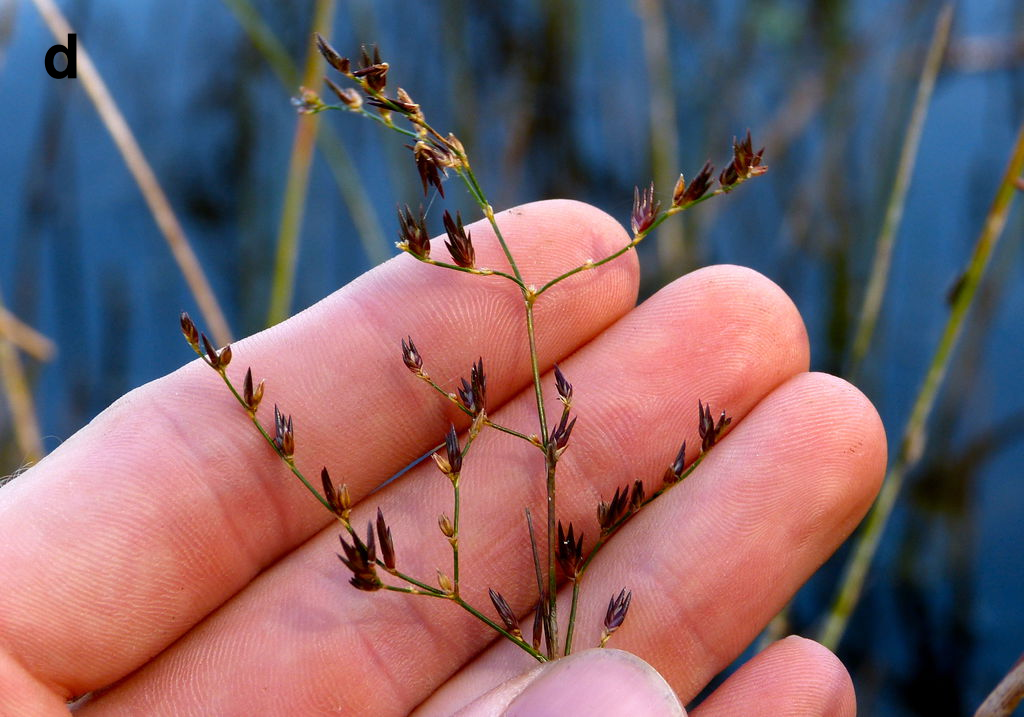
Inflorescence

**Figure 71a.**
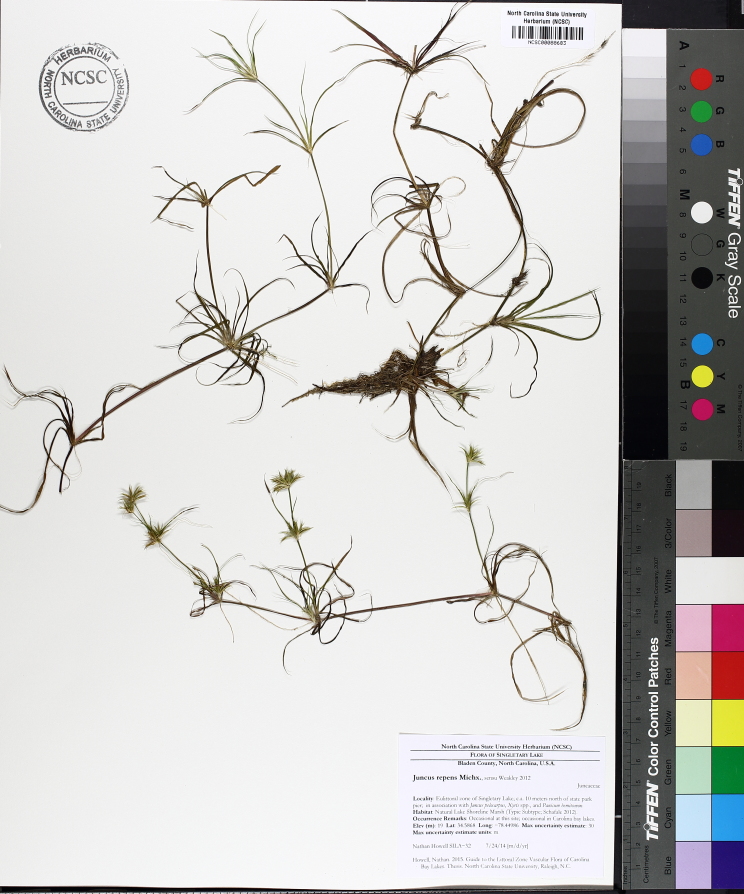
Specimen: *Howell SILA-32* (NCSC)

**Figure 71b.**
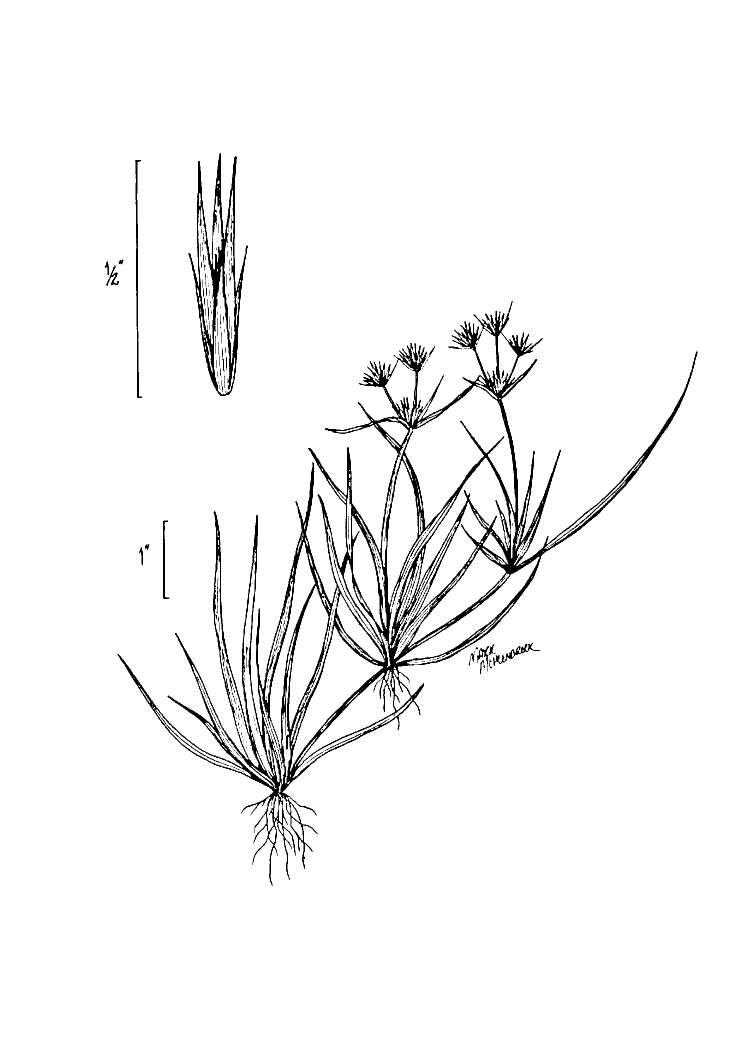
Illustration

**Figure 71c.**
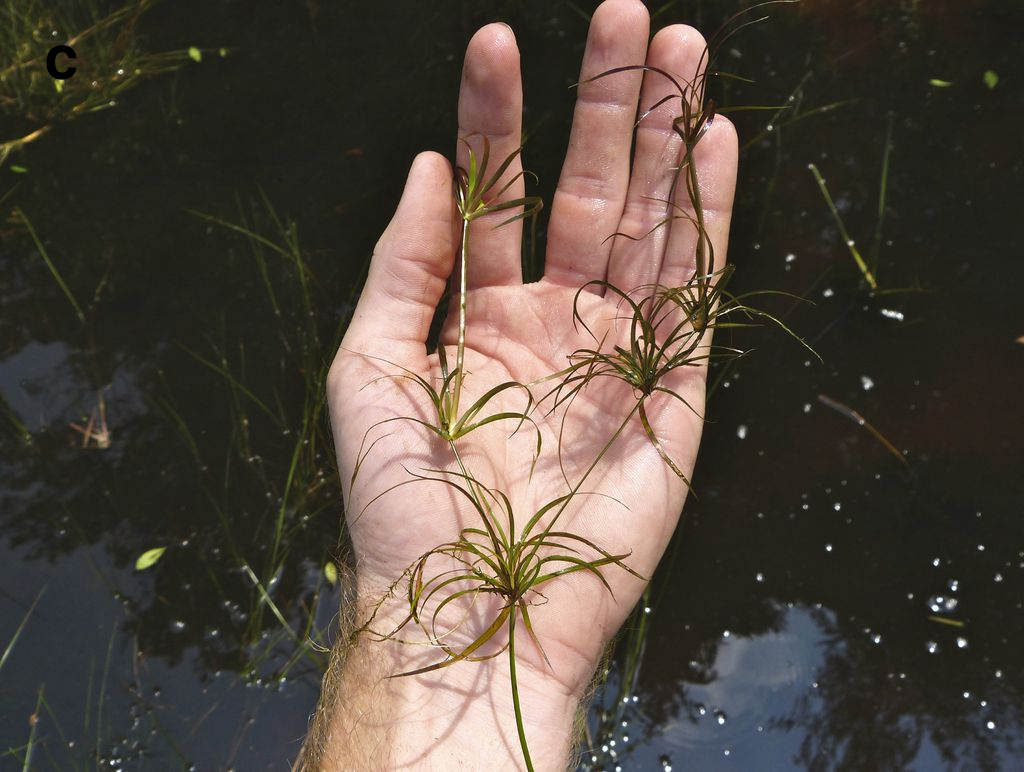
Habit

**Figure 71d.**
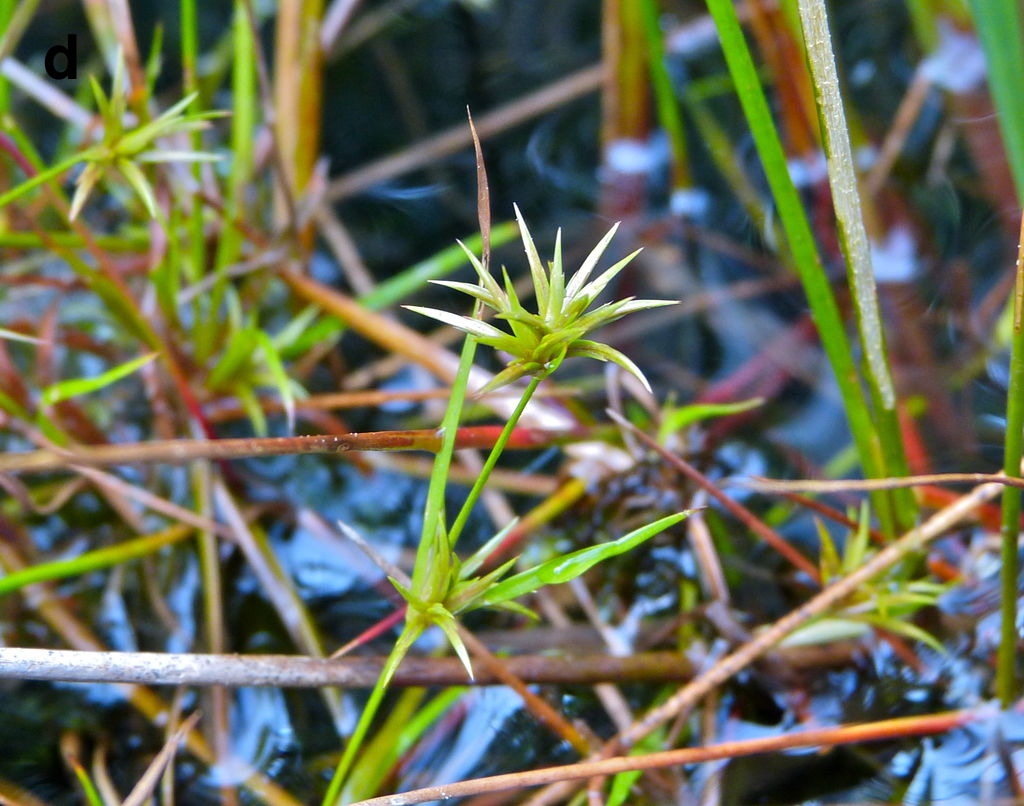
Habit

**Figure 72a.**
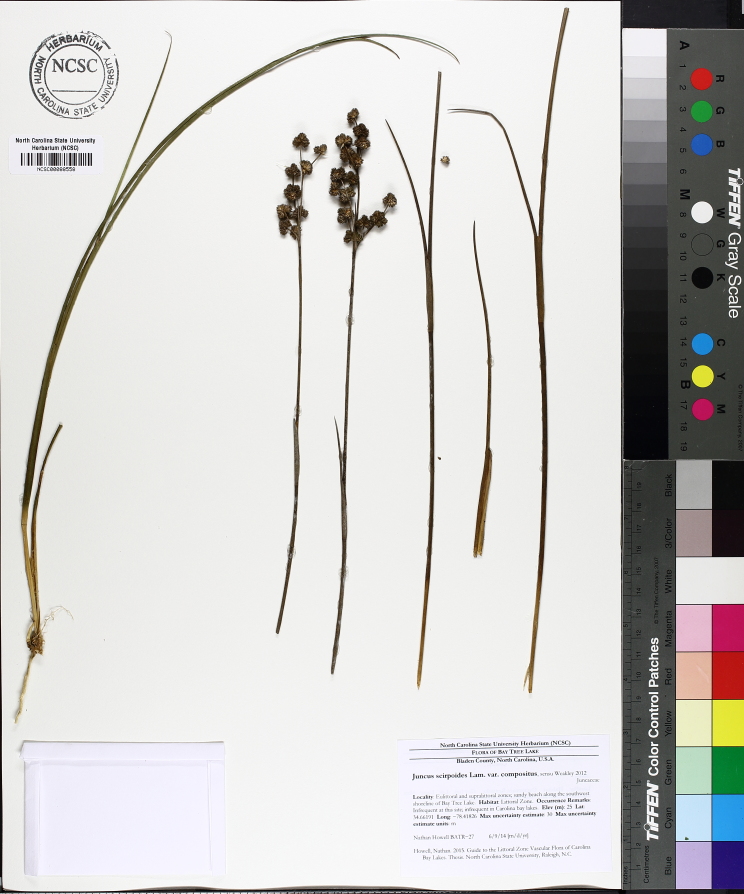
Specimen: *Howell BATR-27* (NCSC)

**Figure 72b.**
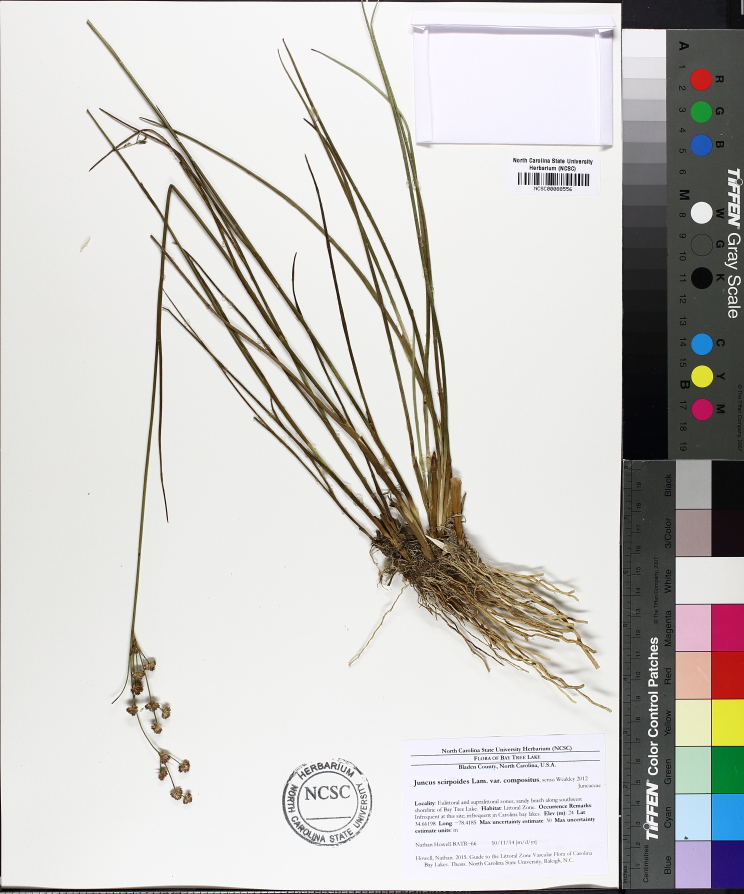
Specimen: *Howell BATR-66* (NCSC)

**Figure 72c.**
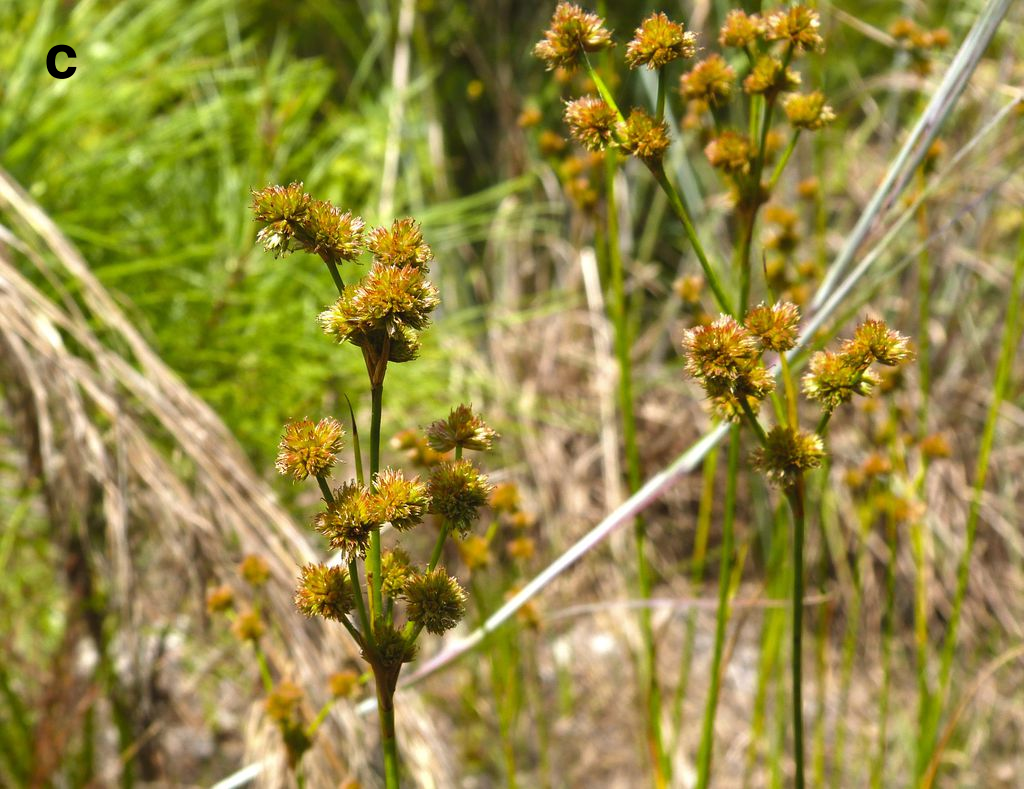
Inflorescence

**Figure 72d.**
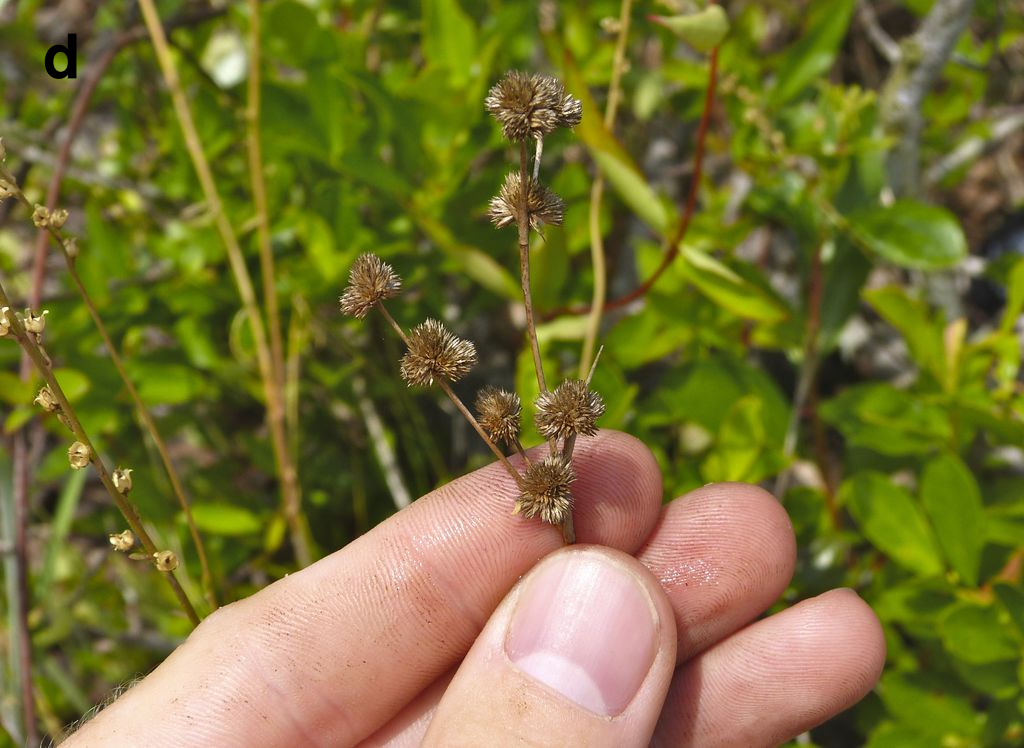
Inflorescence

**Figure 74a.**
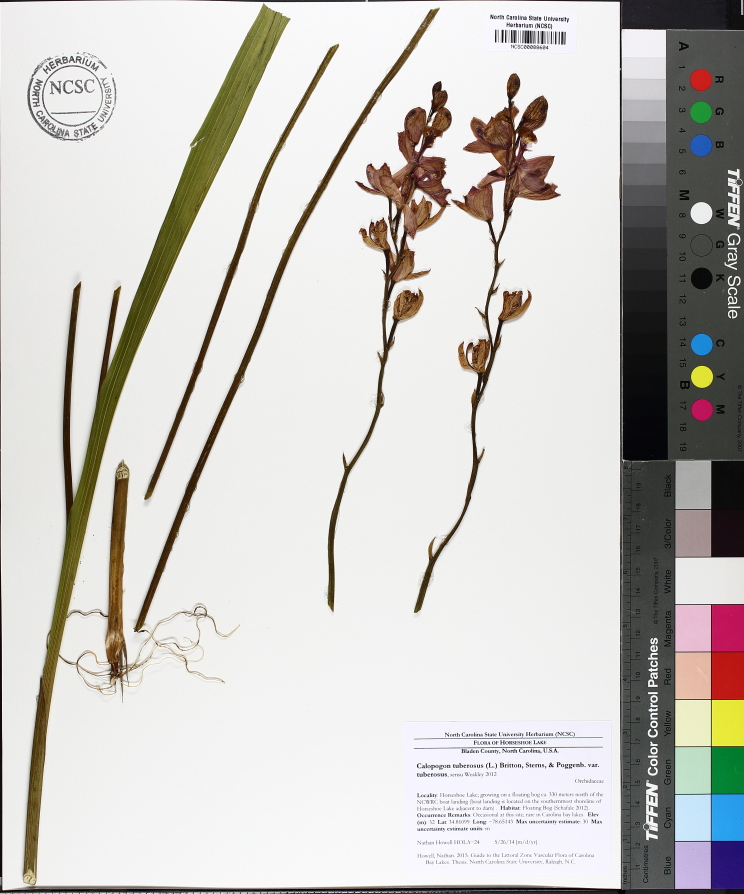
Specimen: *Howell HOLA-24* (NCSC)

**Figure 74b.**
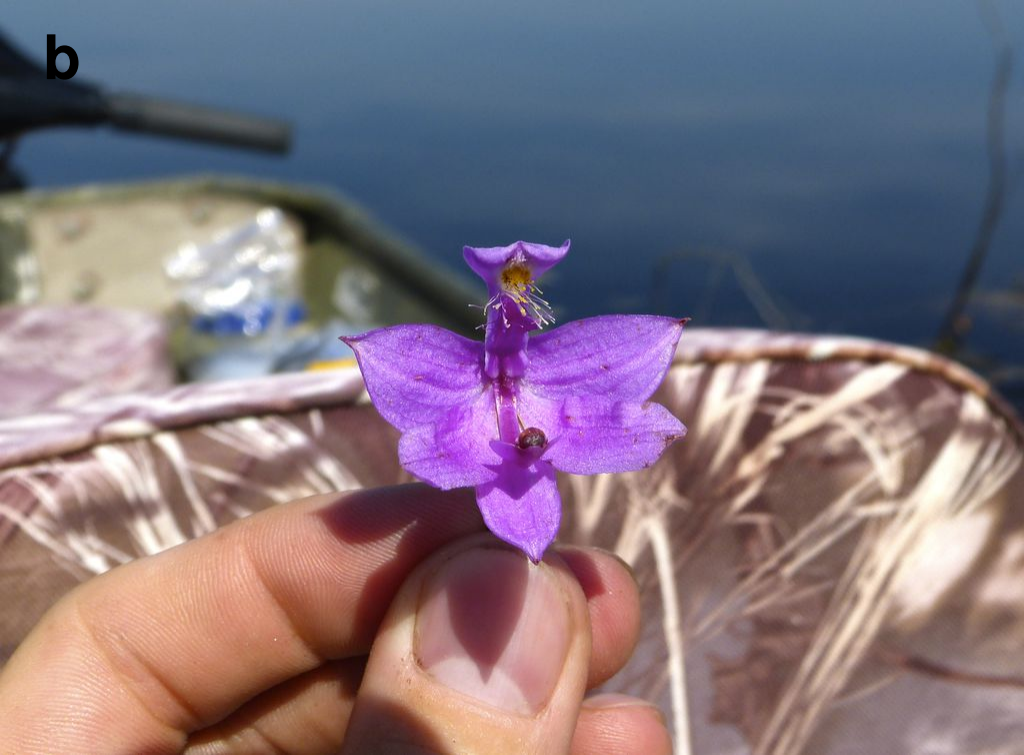
Flower

**Figure 75a.**
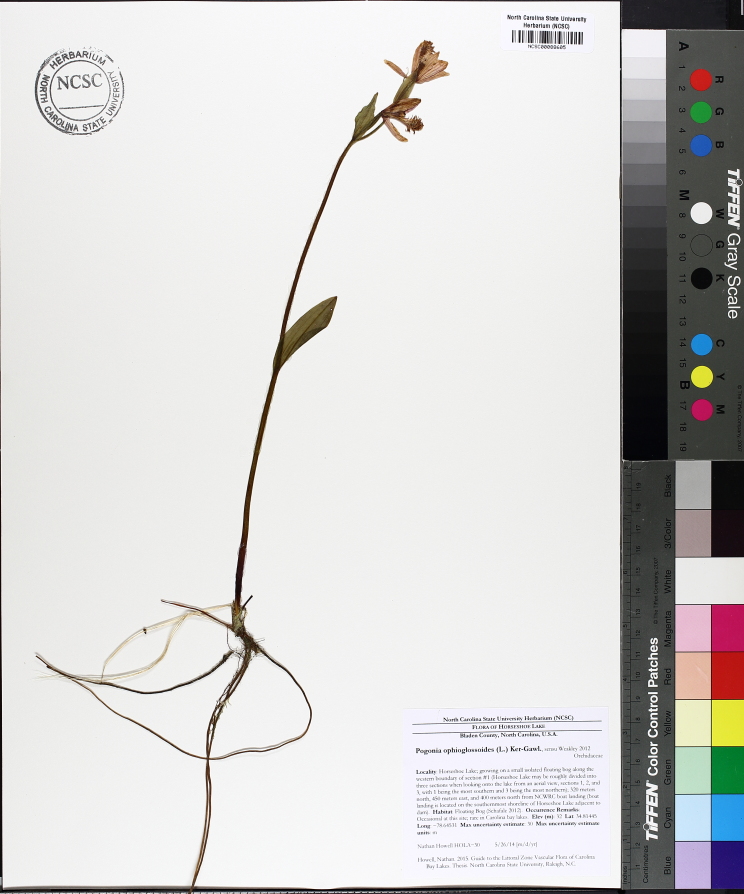
Specimen: *Howell HOLA-30* (NCSC)

**Figure 75b.**
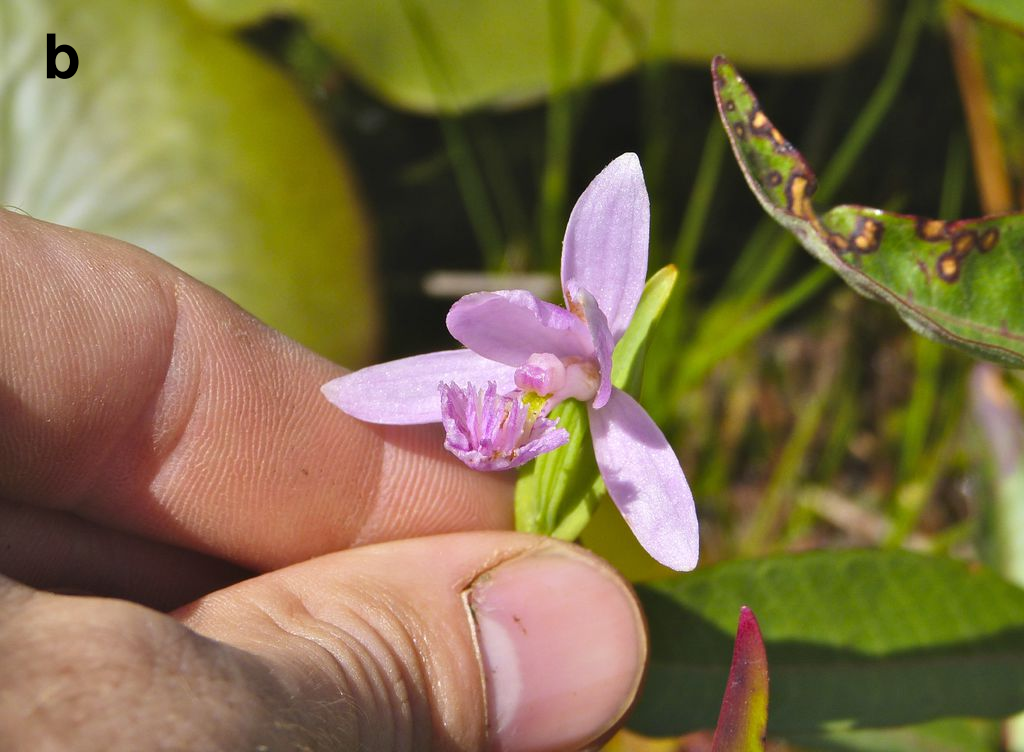
Flower

**Figure 76a.**
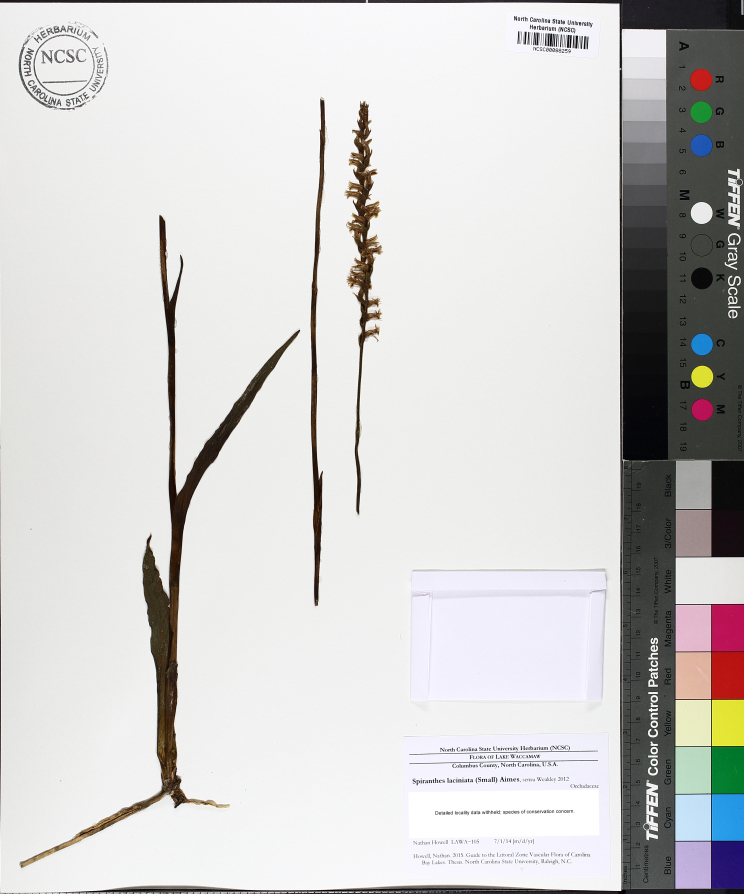
Specimen: *Howell LAWA-105* (NCSC)

**Figure 76b.**
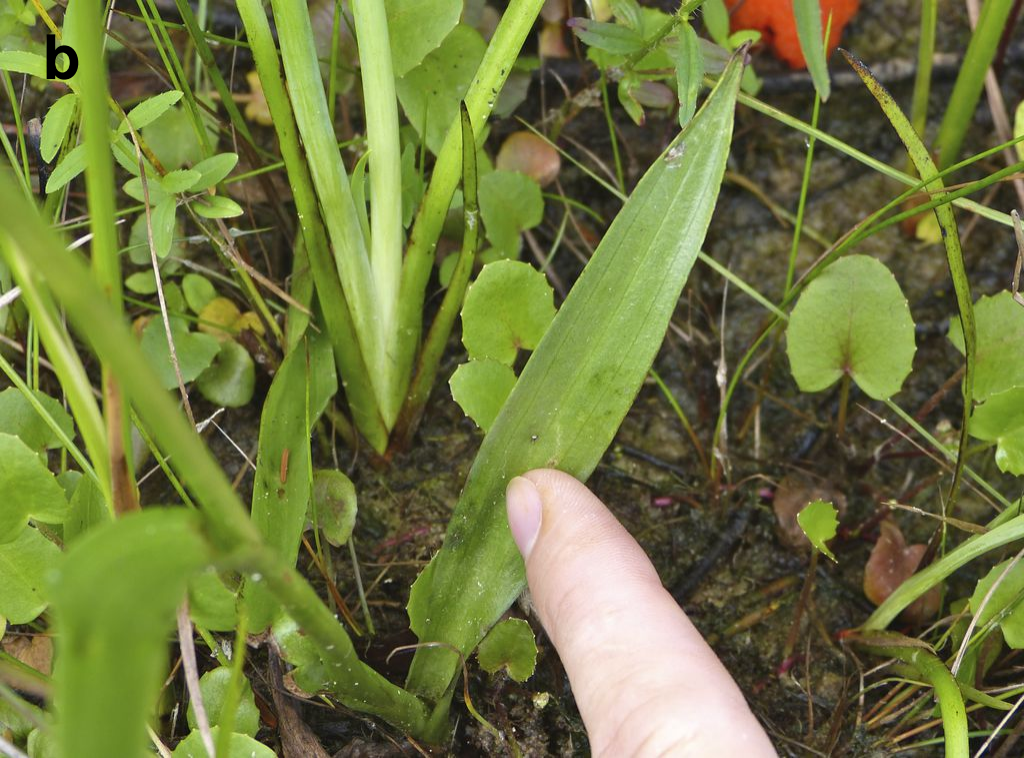
Leaf

**Figure 76c.**
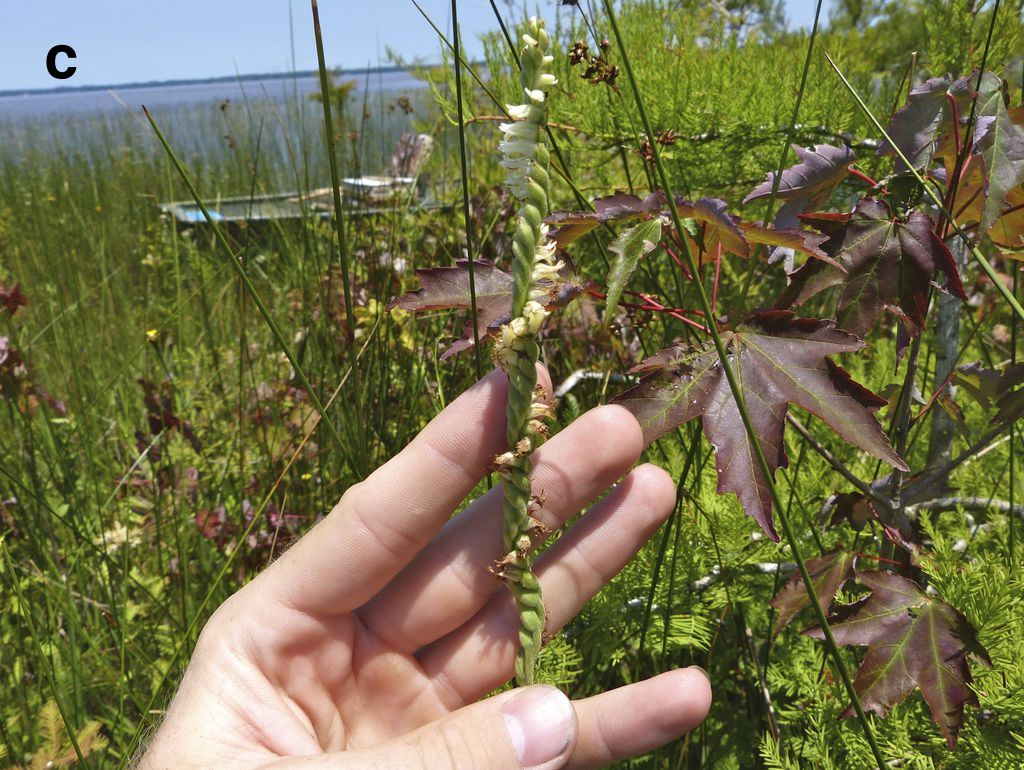
Inflorescence

**Figure 76d.**
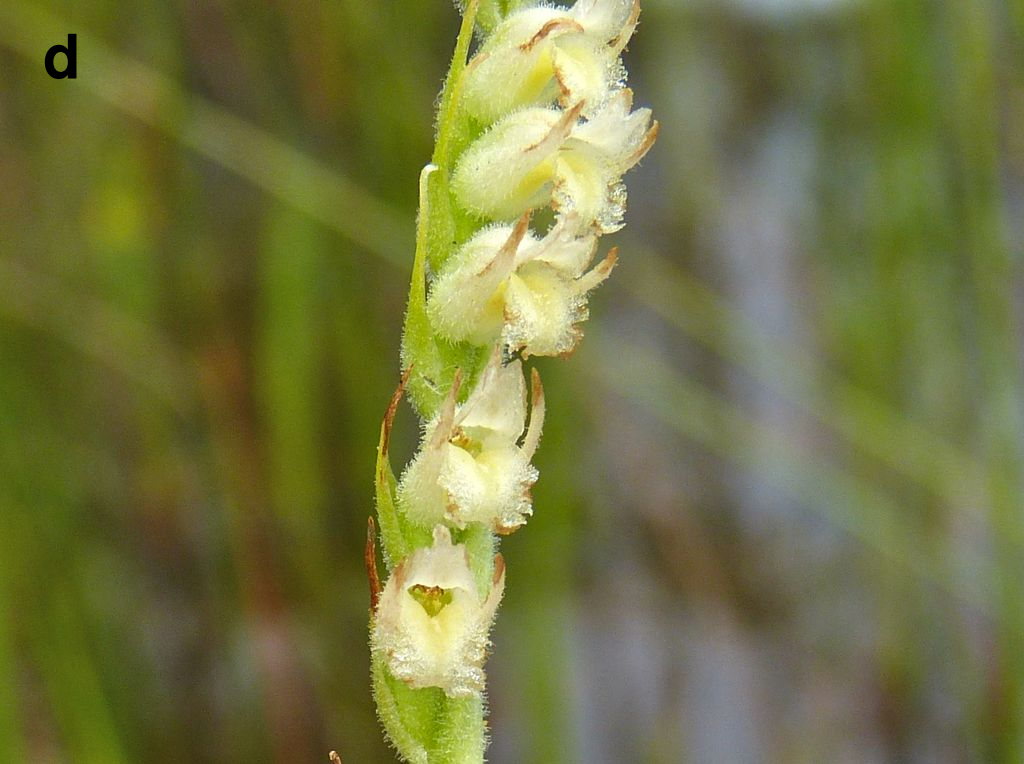
Inflorescence (detail)

**Figure 77a.**
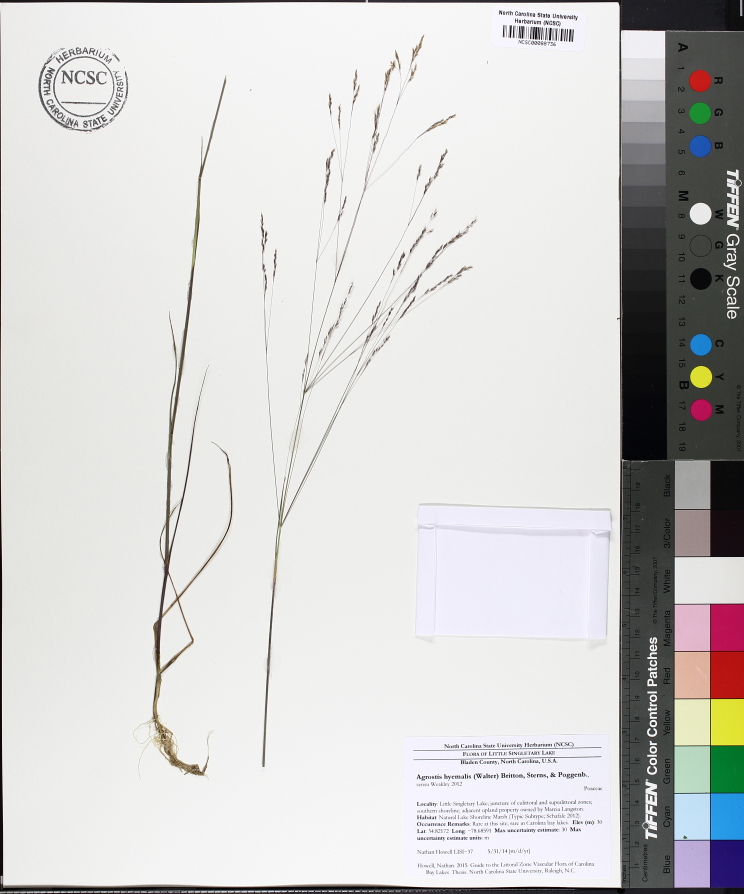
Specimen: *Howell LISI-37* (NCSC)

**Figure 77b.**
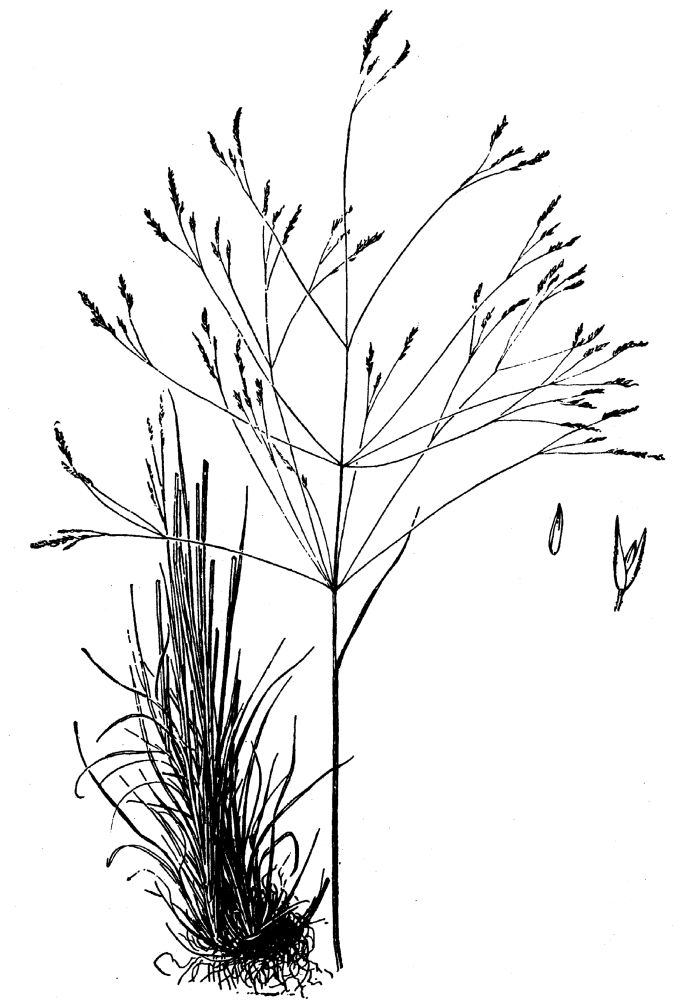
Illustration

**Figure 77c.**
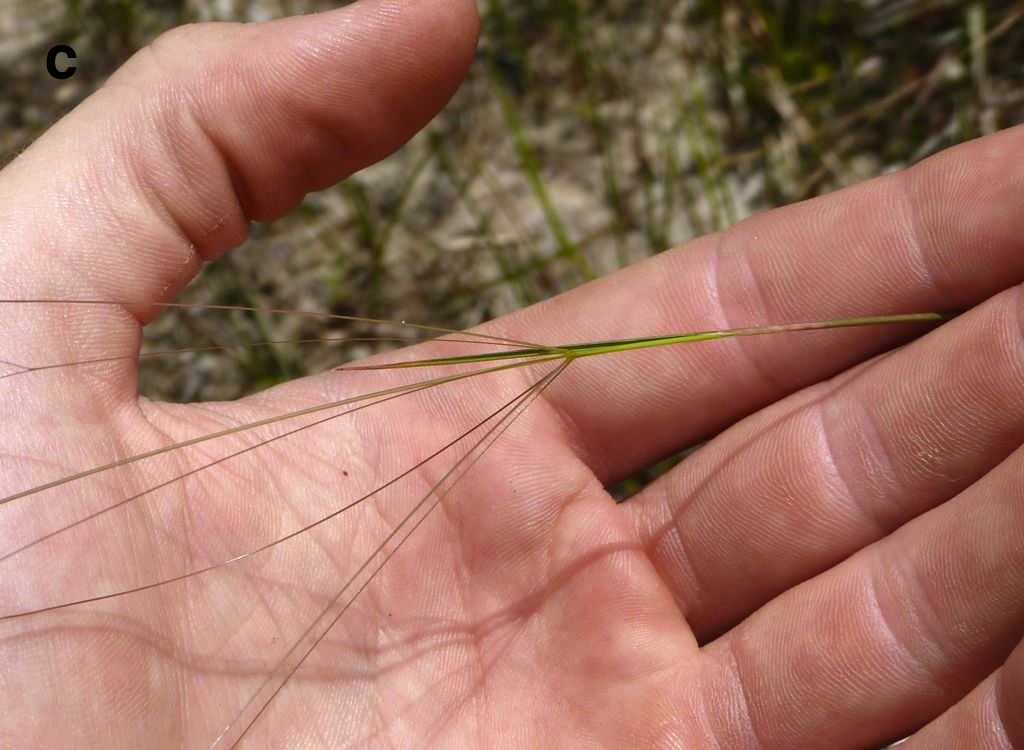
Base of inflorescence

**Figure 77d.**
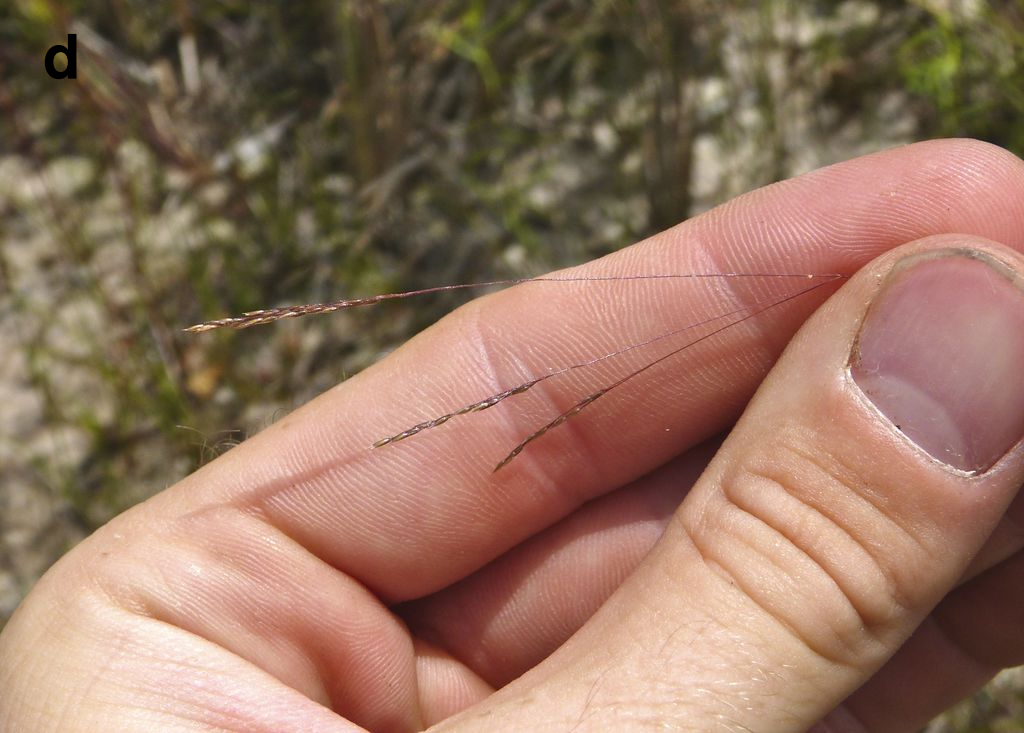
Inflorescence, including spikelets

**Figure 83a.**
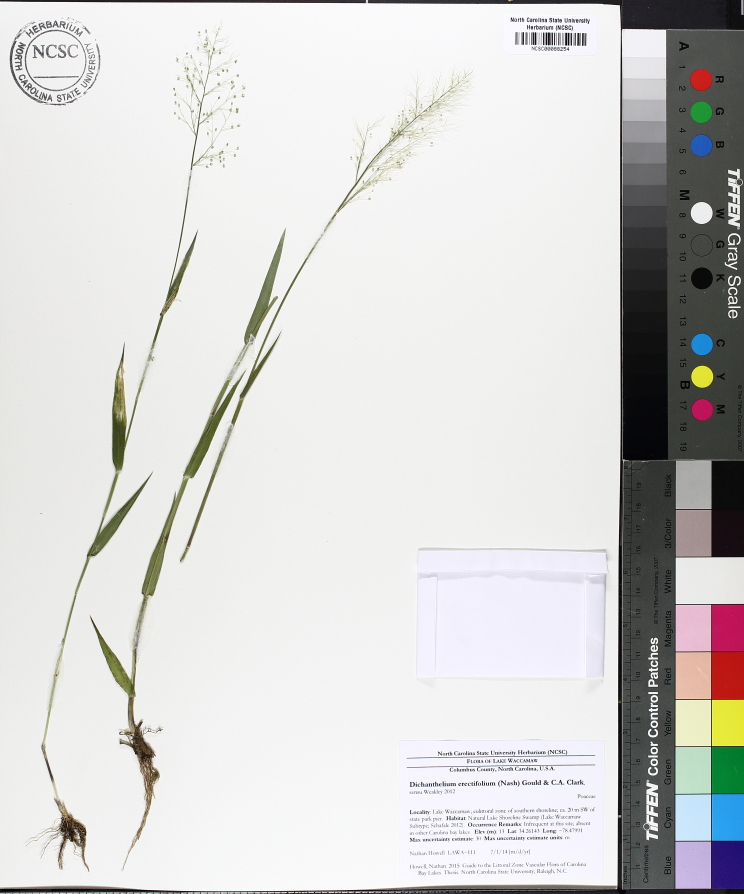
Specimen: *Howell LAWA-111* (NCSC)

**Figure 83b.**
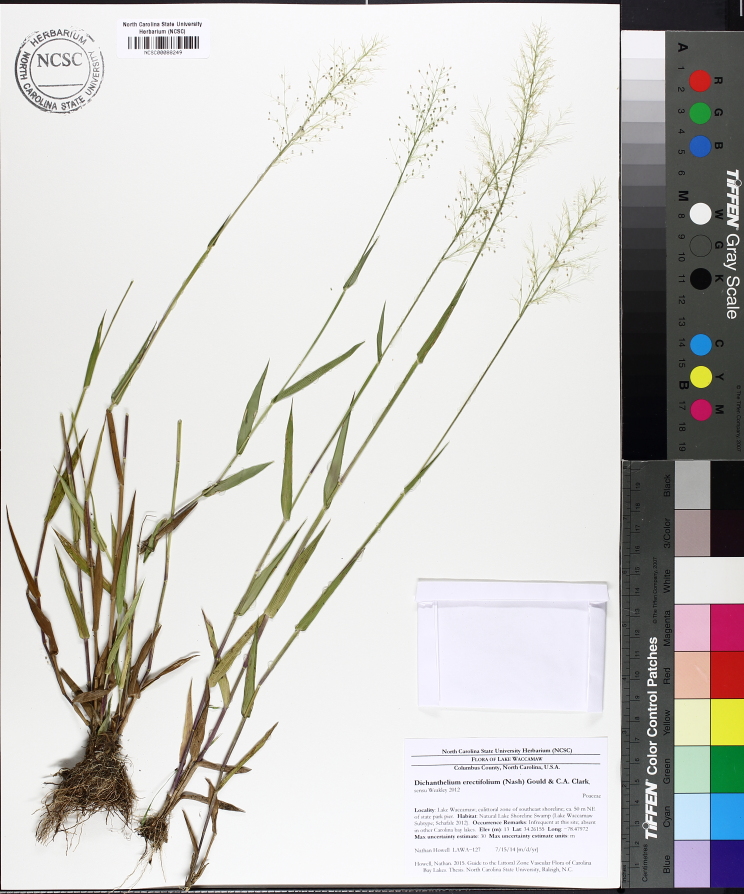
Specimen: *Howell LAWA-127* (NCSC)

**Figure 83c.**
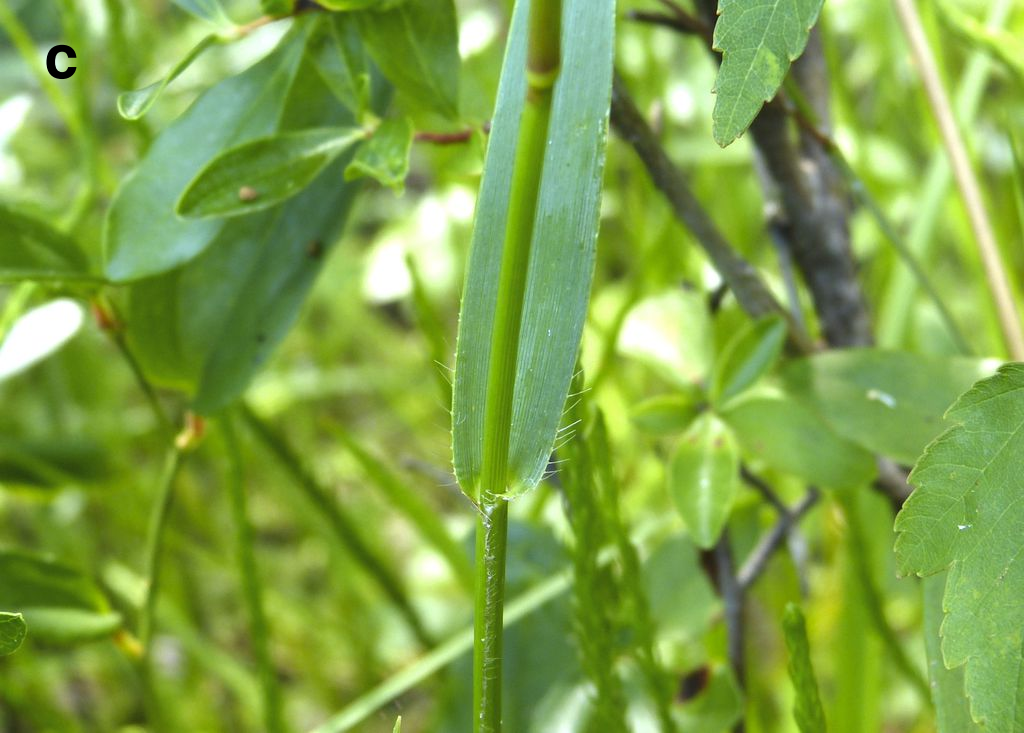
Culm and leaf

**Figure 83d.**
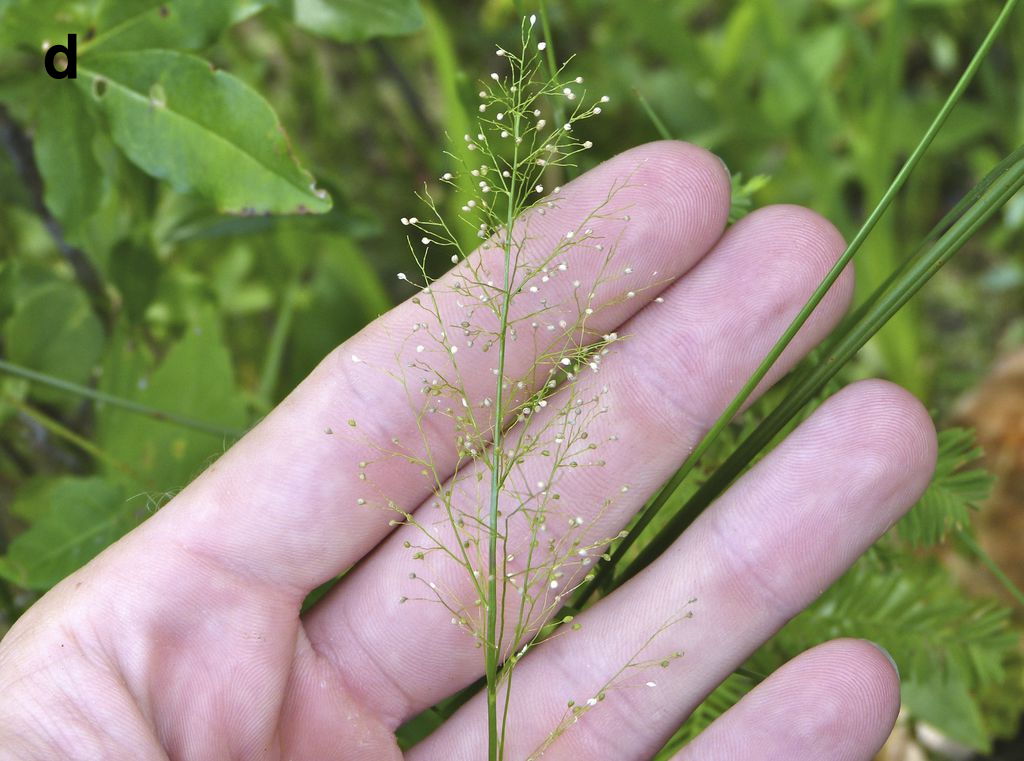
Inflorescence

**Figure 87a.**
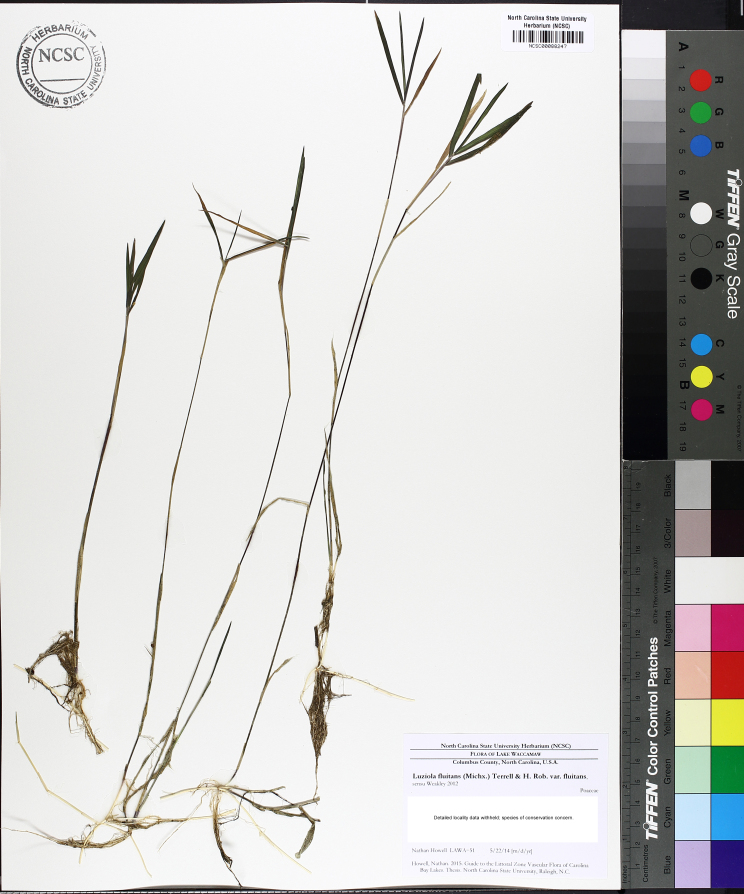
Specimen: *Howell LAWA-51* (NCSC)

**Figure 87b.**
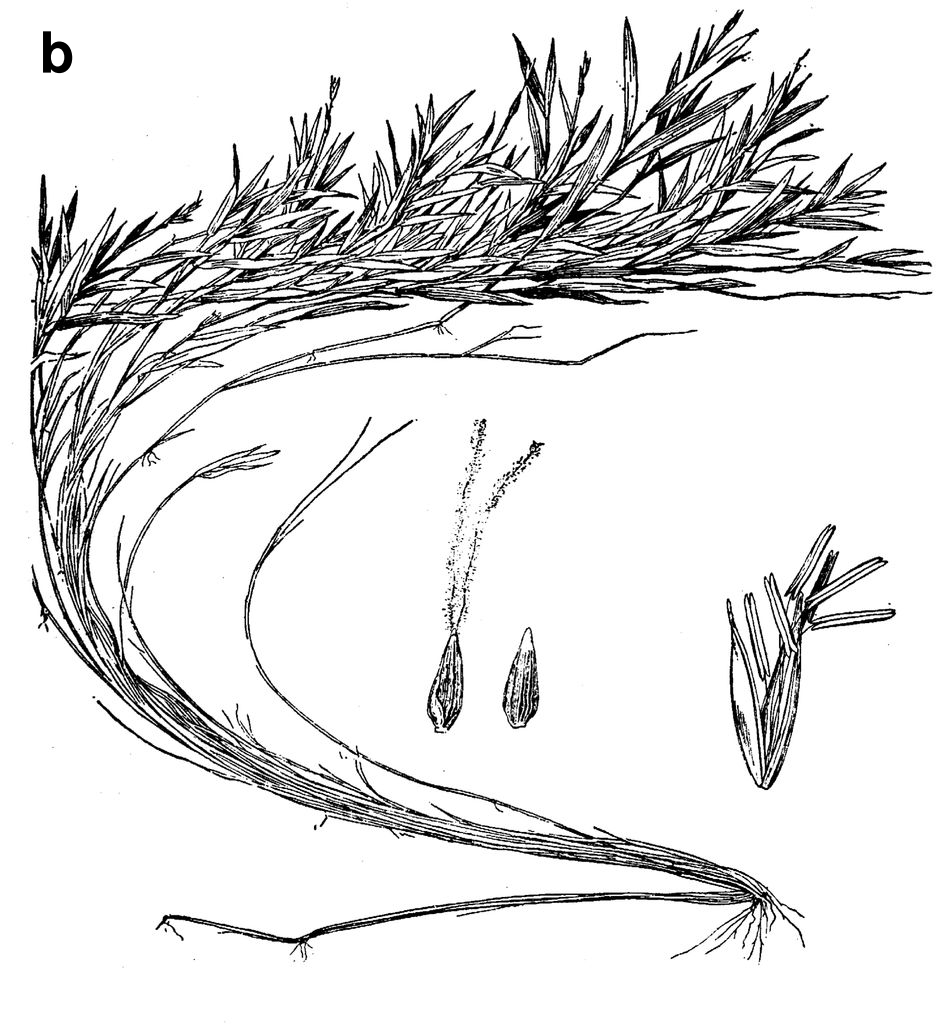
Illustration

**Figure 87c.**
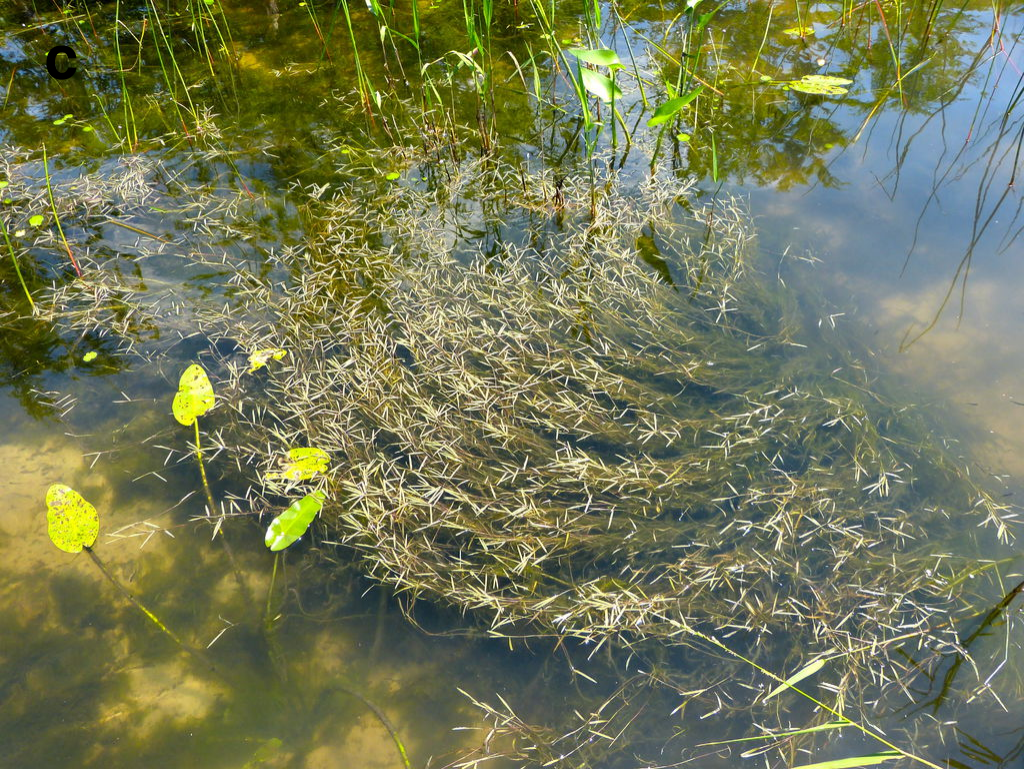
Habit

**Figure 87d.**
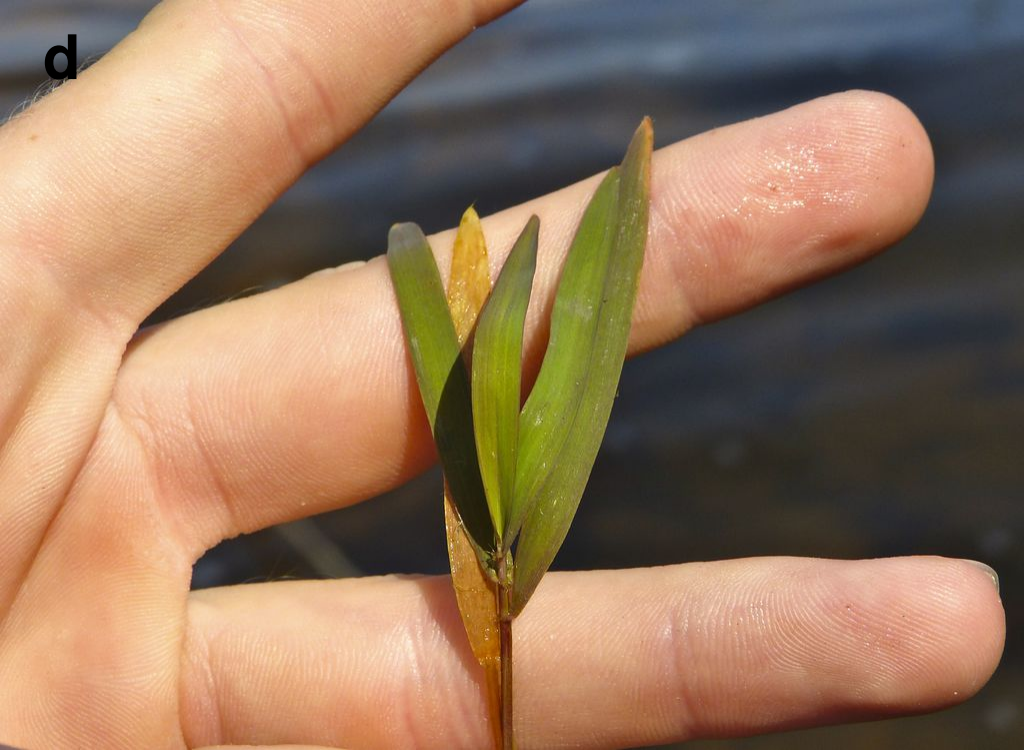
Leaves

**Figure 88a.**
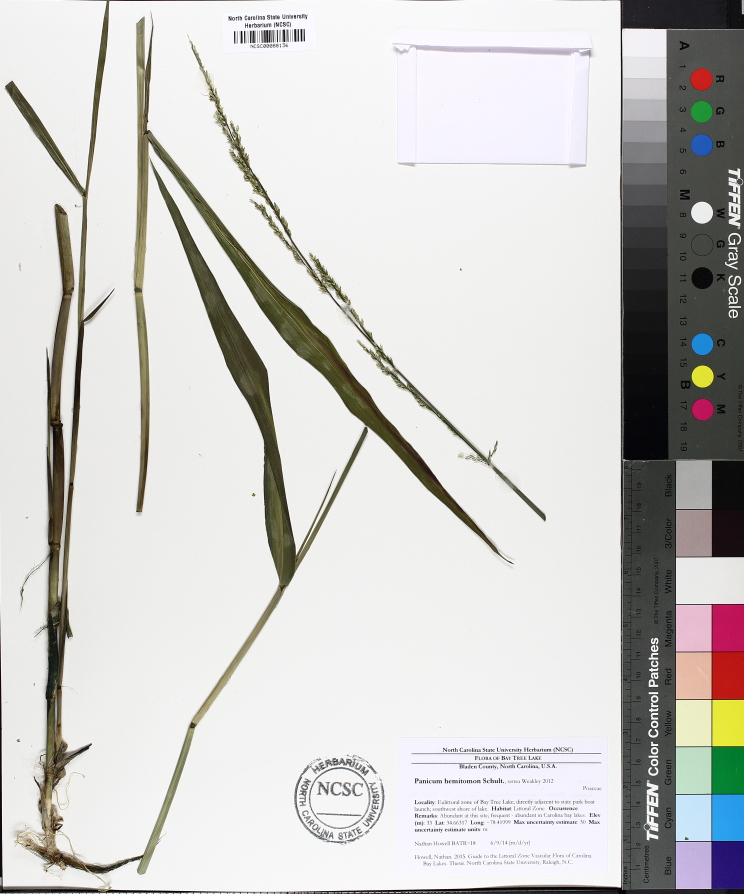
Specimen: *Howell BATR-18* (NCSC)

**Figure 88b.**
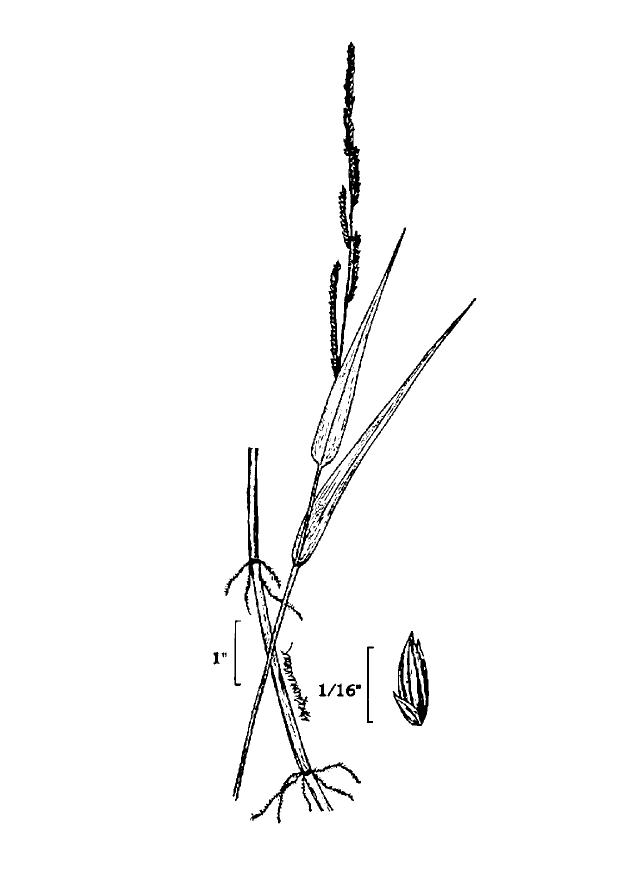
Illustration

**Figure 88c.**
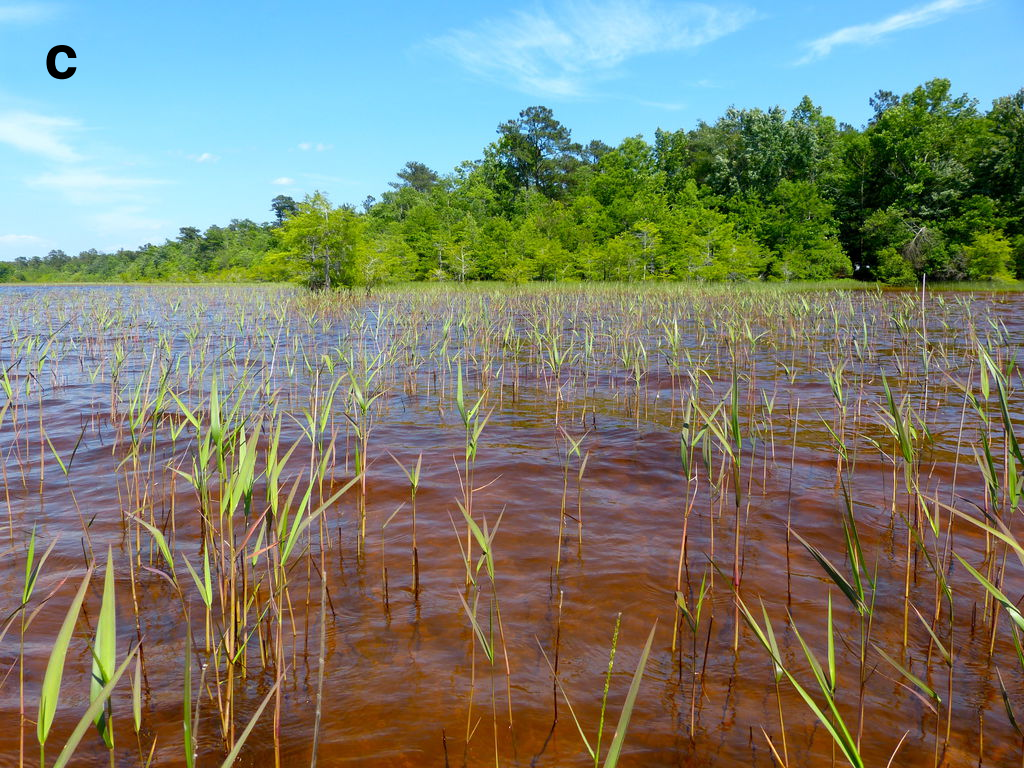
Habit

**Figure 88d.**
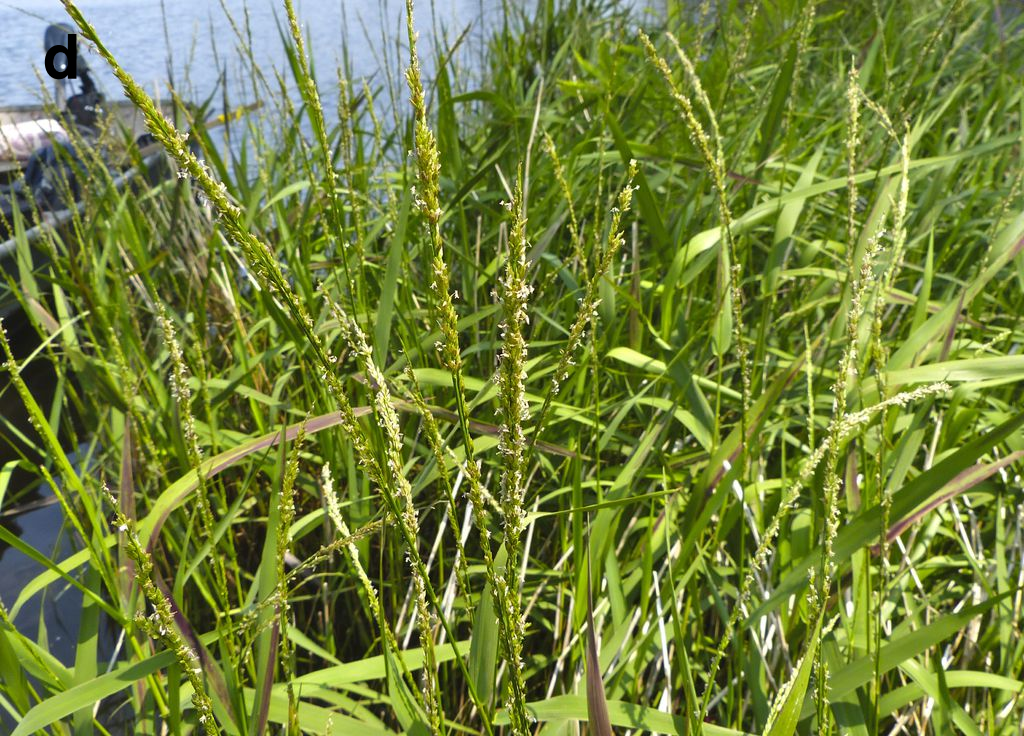
Inflorescences

**Figure 89a.**
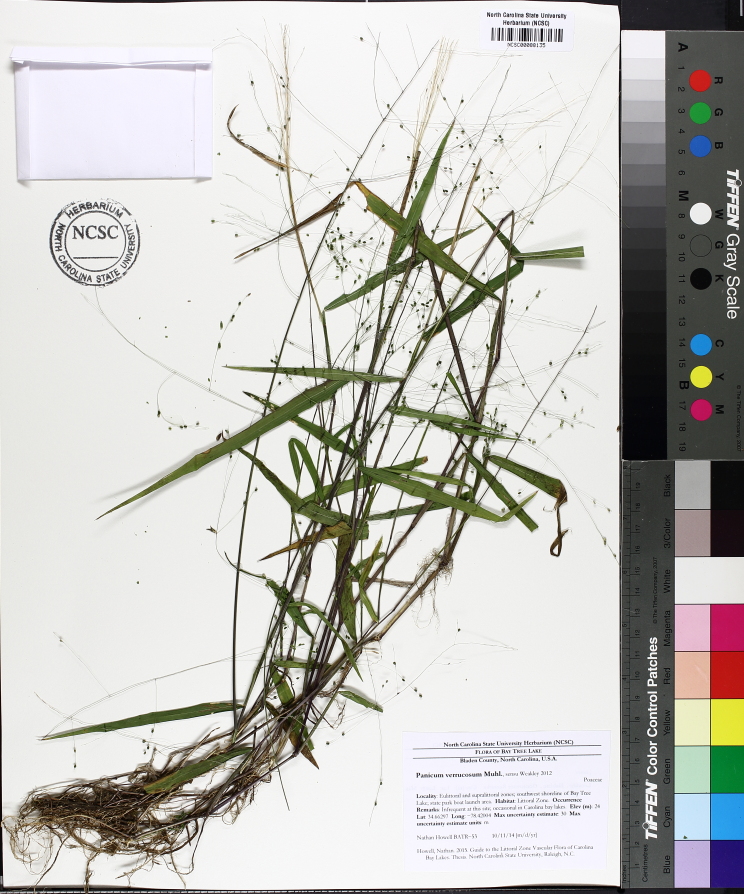
Specimen: *Howell BATR-53* (NCSC)

**Figure 89b.**
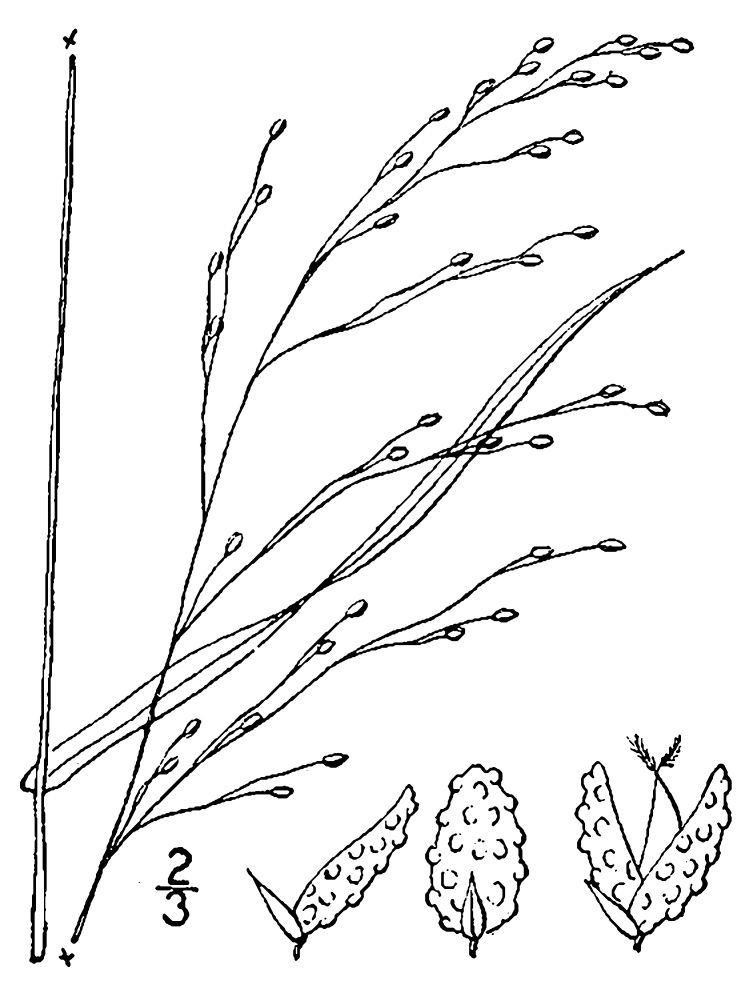
Illustration

**Figure 91a.**
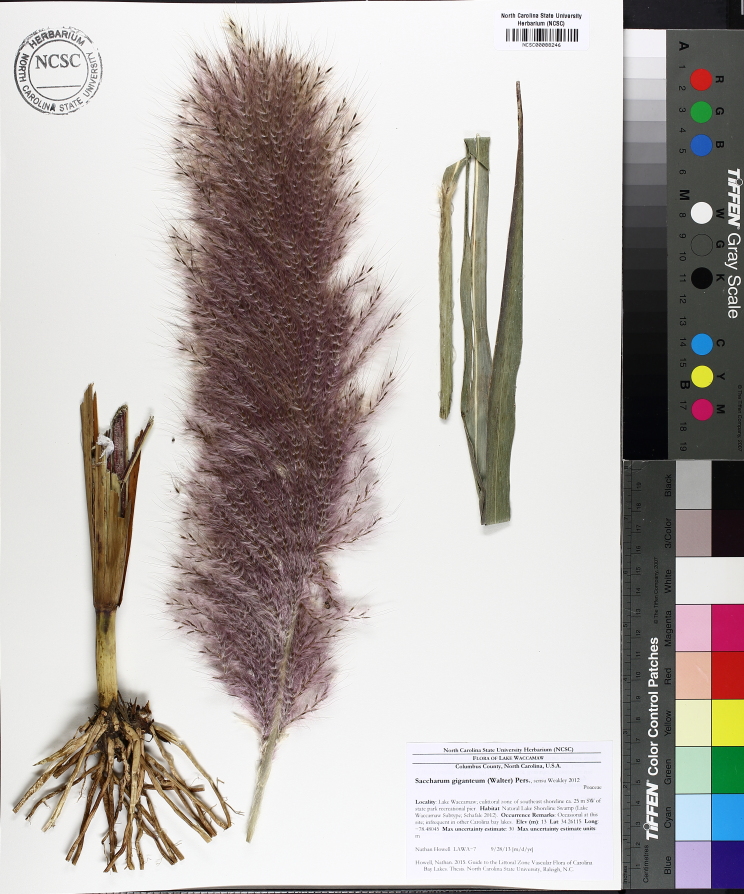
Specimen: *Howell LAWA-7* (NCSC)

**Figure 91b.**
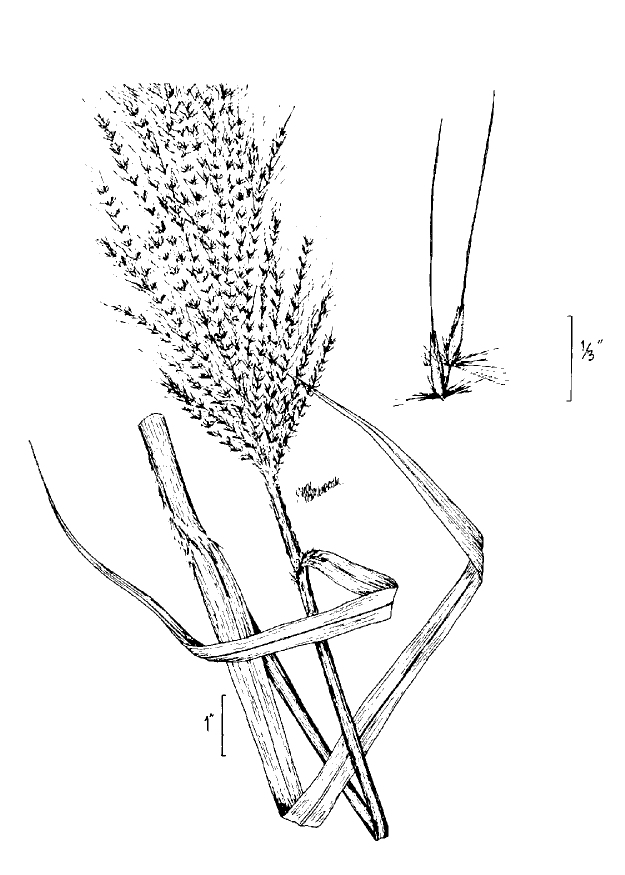
lllustration

**Figure 91c.**
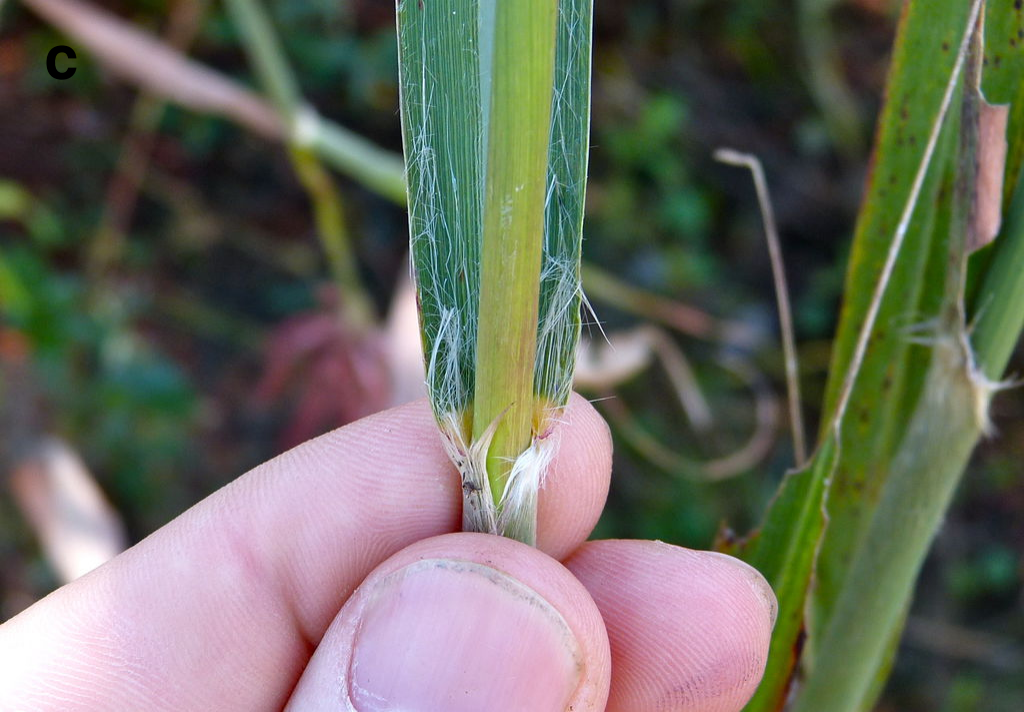
Culm and leaf blade

**Figure 91d.**
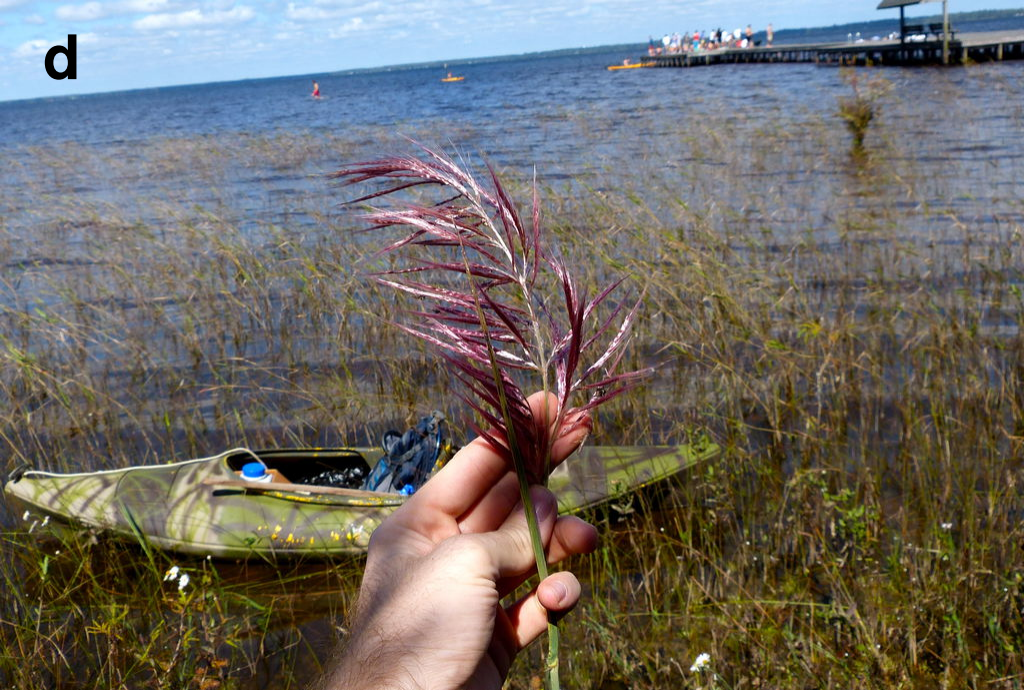
Inflorescence

**Figure 92a.**
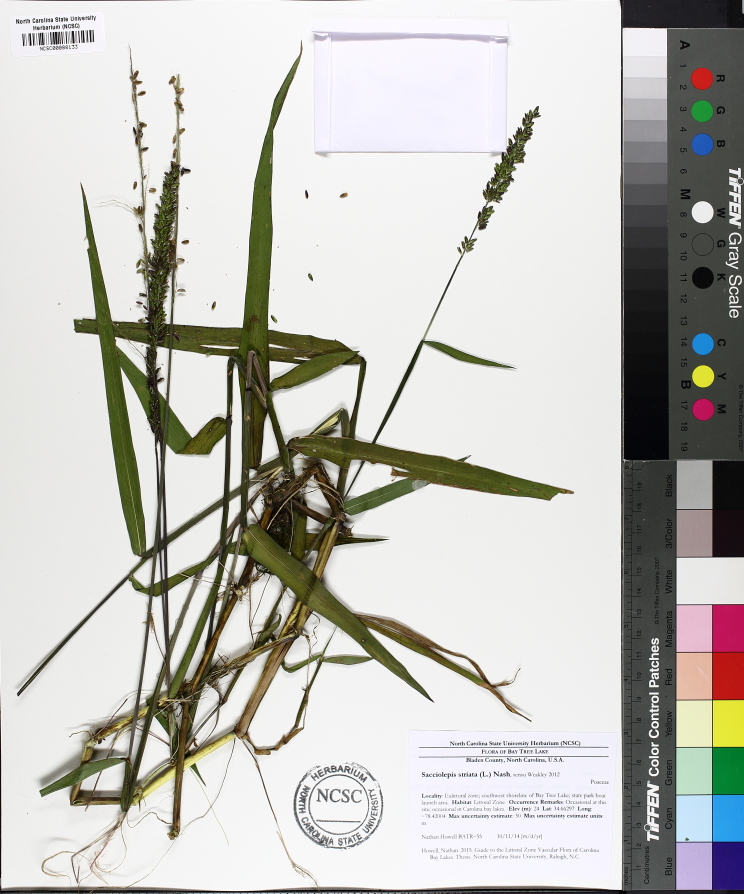
Specimen: *Howell BATR-55* (NCSC)

**Figure 92b.**
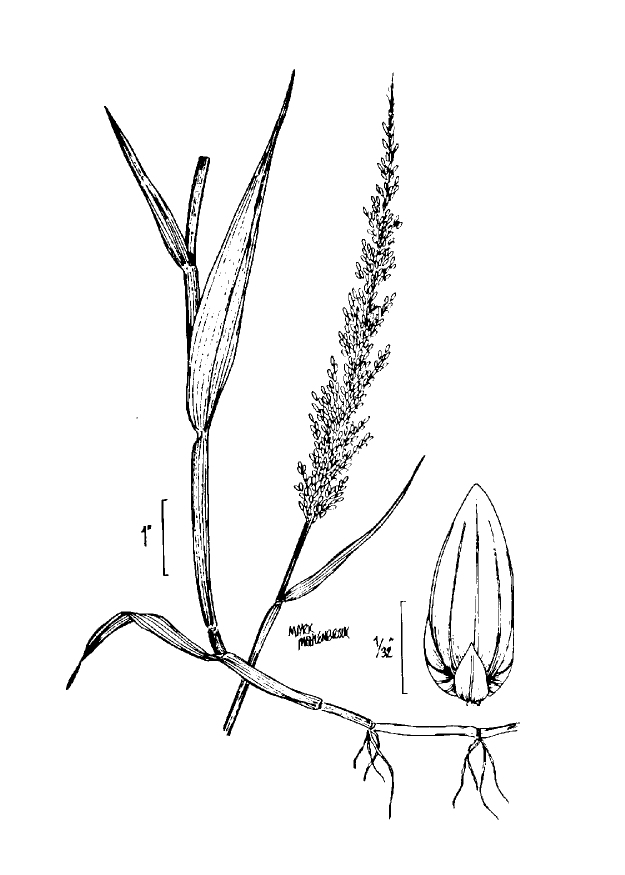
Illustration

**Figure 92c.**
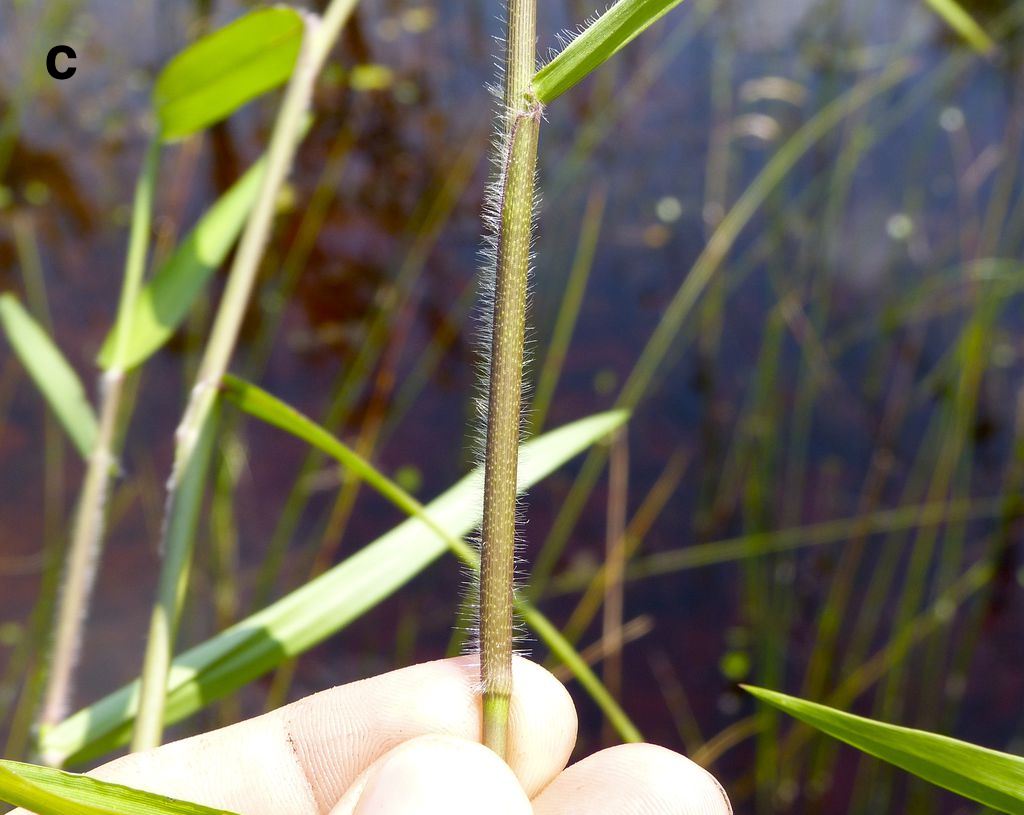
Leaf sheath

**Figure 92d.**
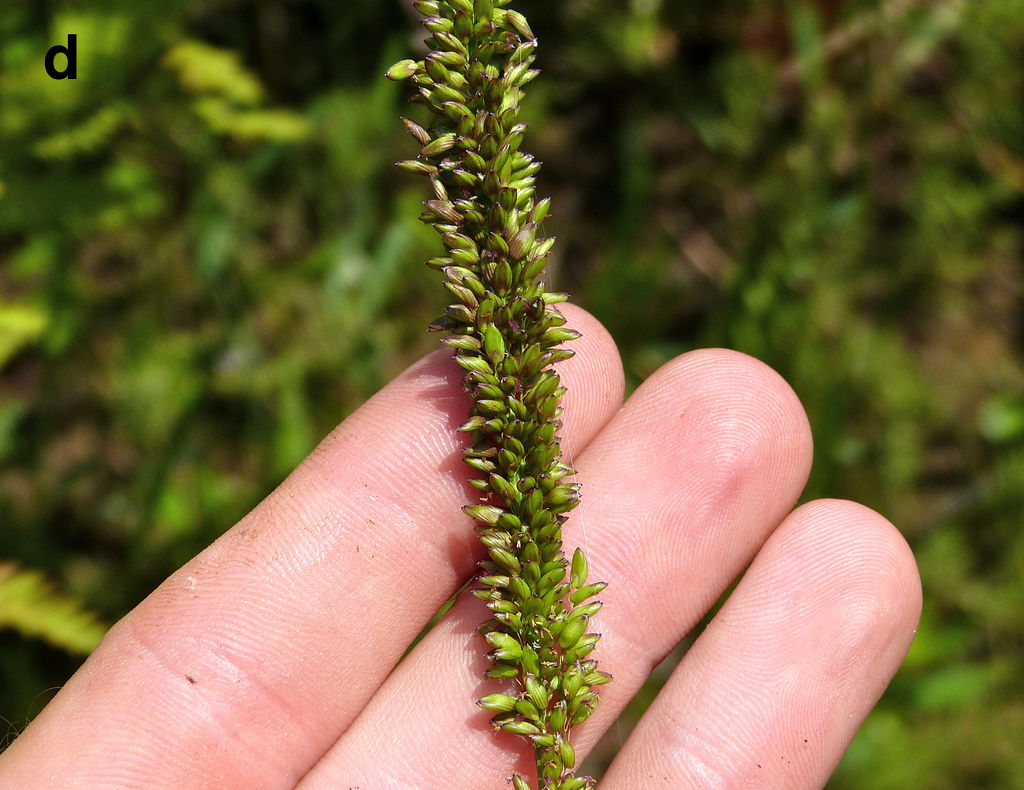
Inflorescence

**Figure 94a.**
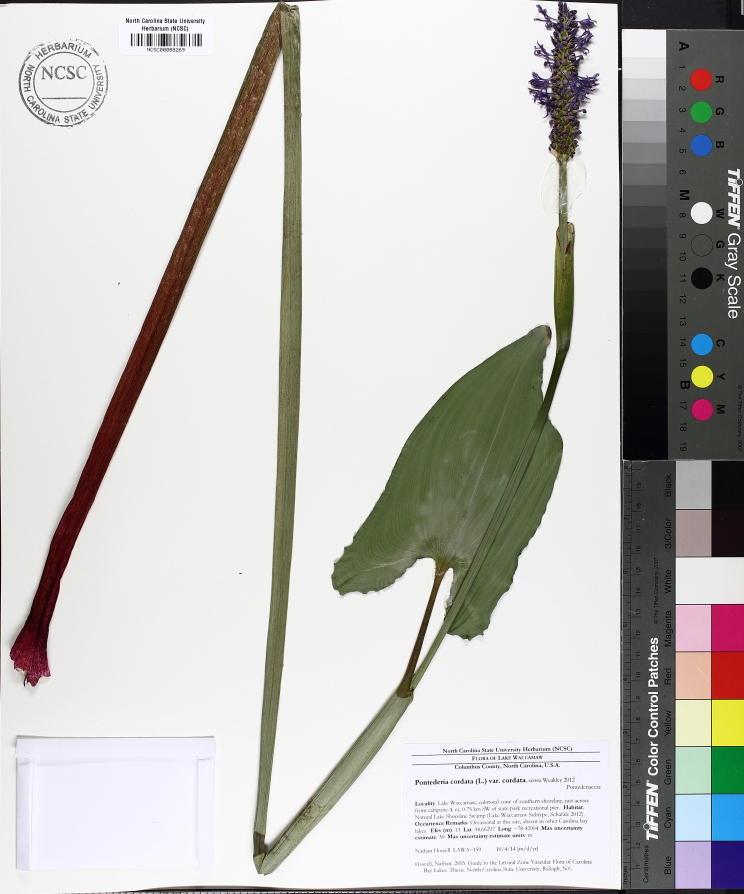
Specimen: *Howell LAWA-159* (NCSC)

**Figure 94b.**
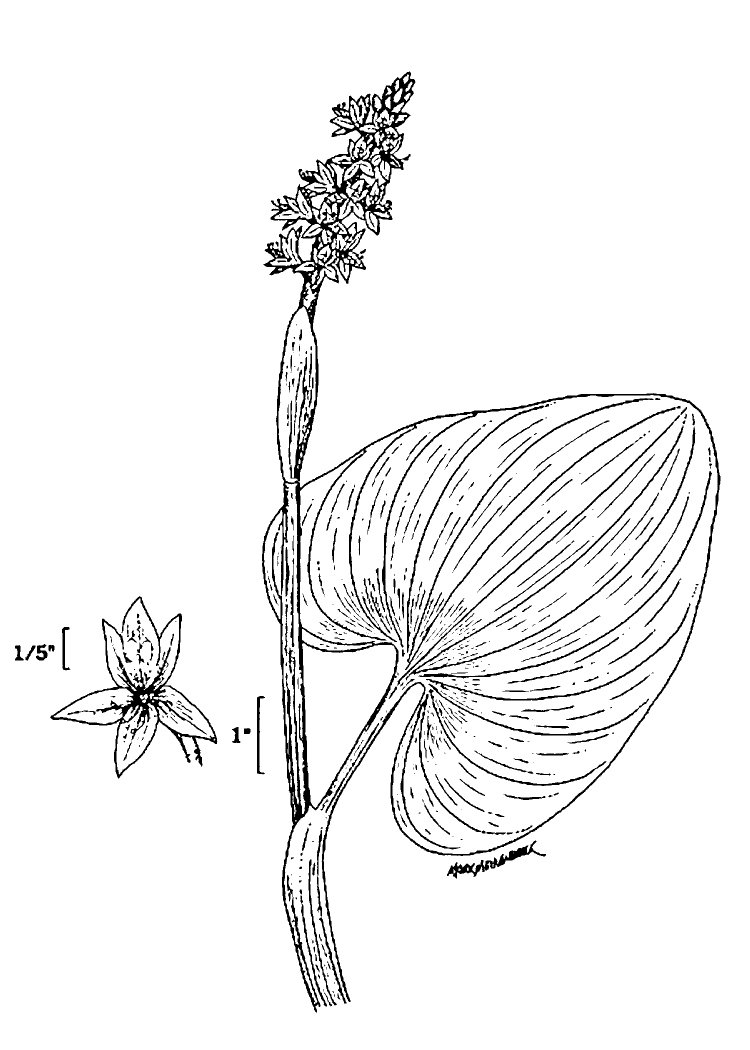
Illustration

**Figure 94c.**
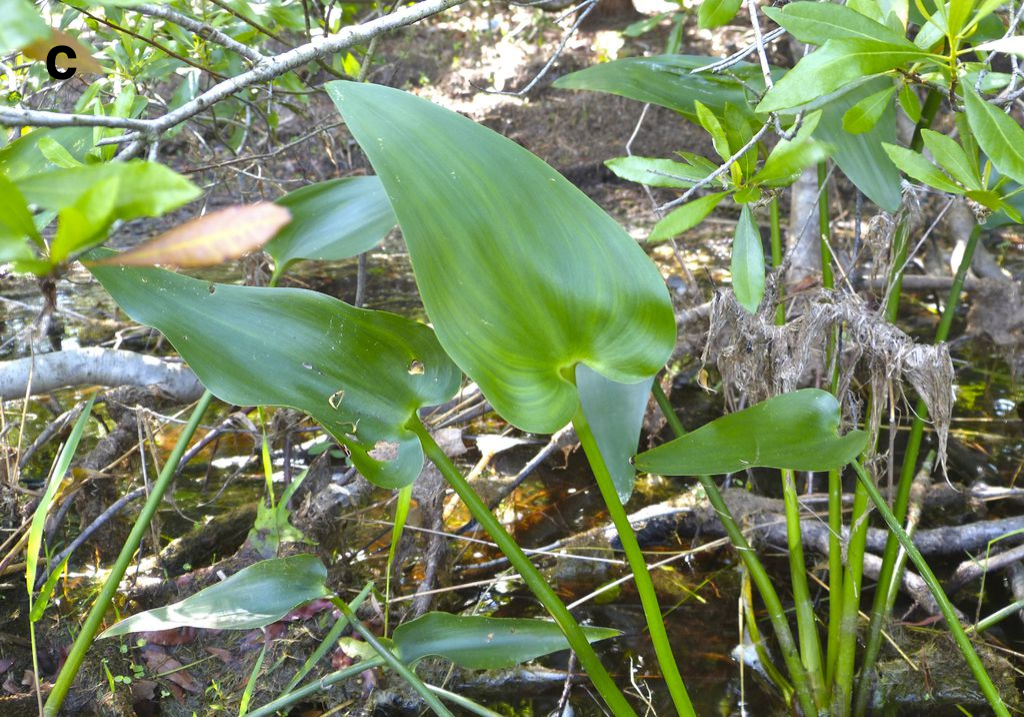
Leaves

**Figure 94d.**
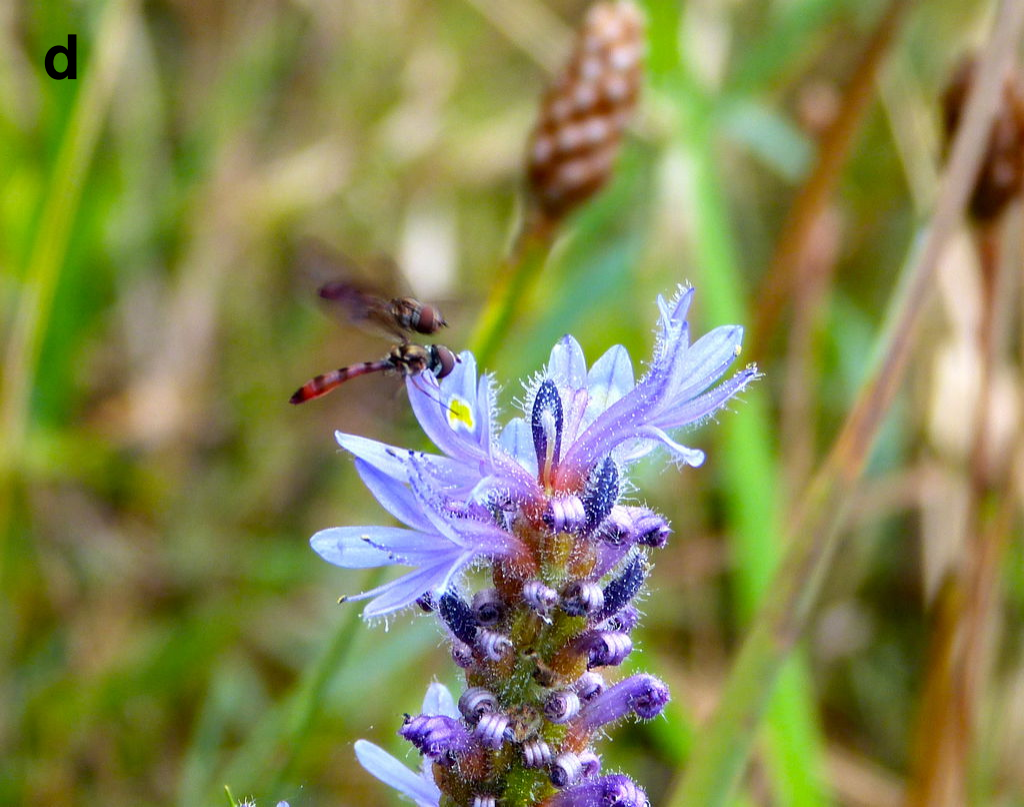
Inflorescence

**Figure 96a.**
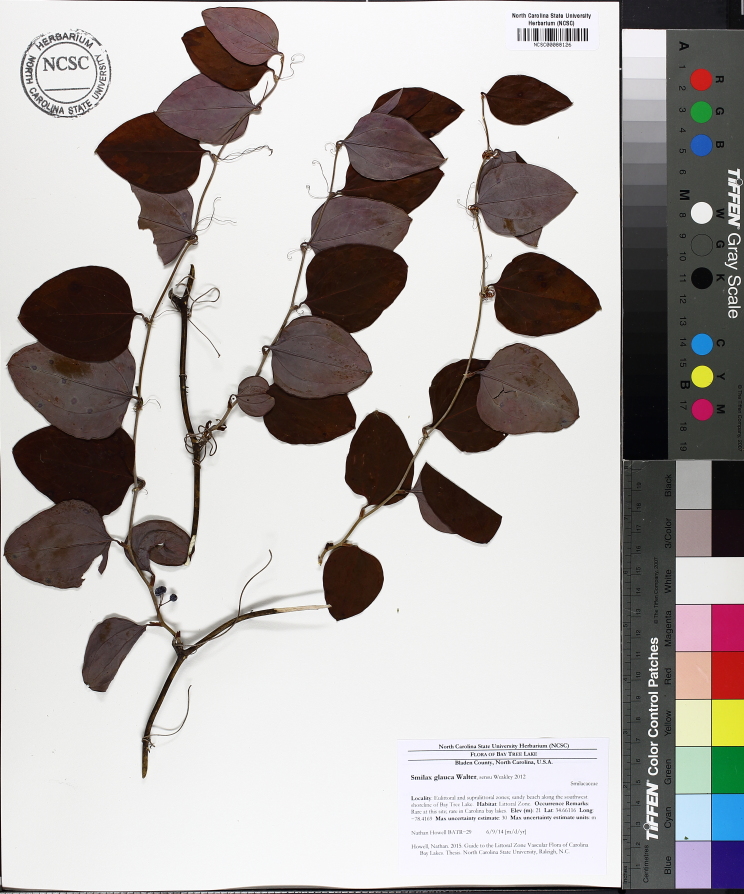
Specimen: *Howell BATR-29* (NCSC)

**Figure 96b.**
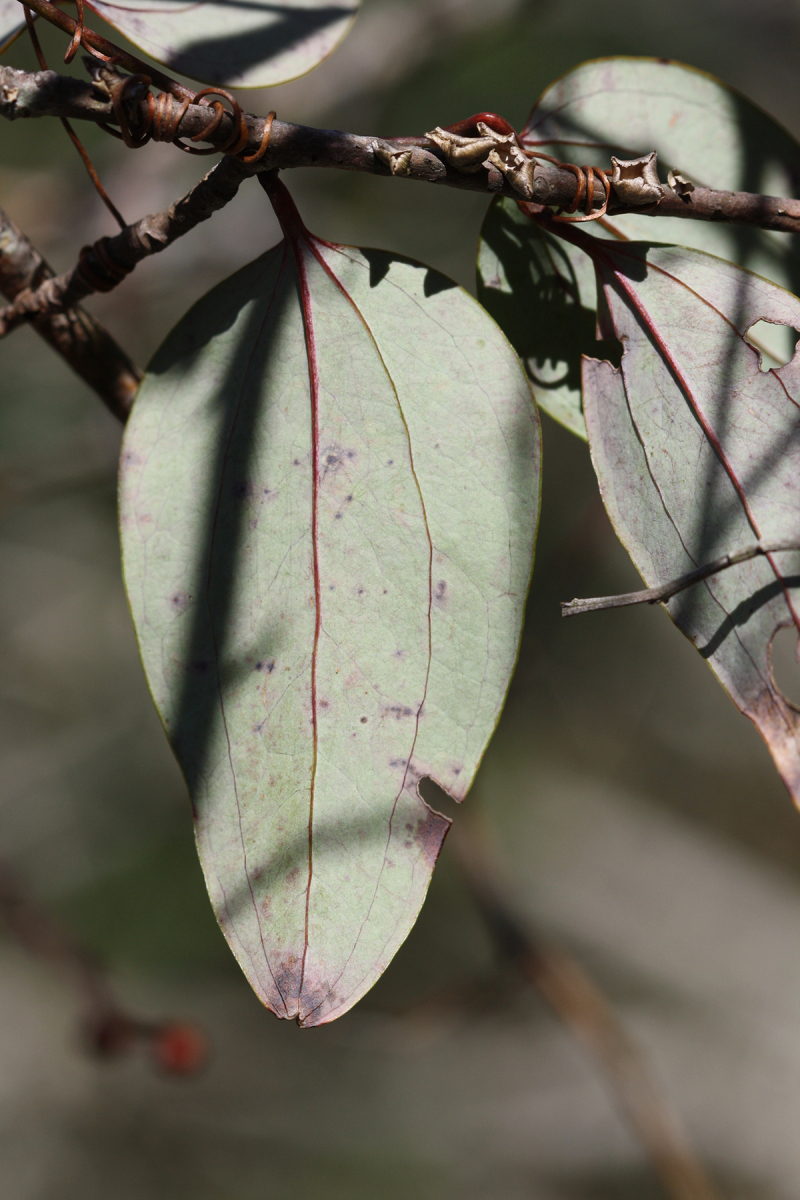
Leaf (abaxial surface)

**Figure 96c.**
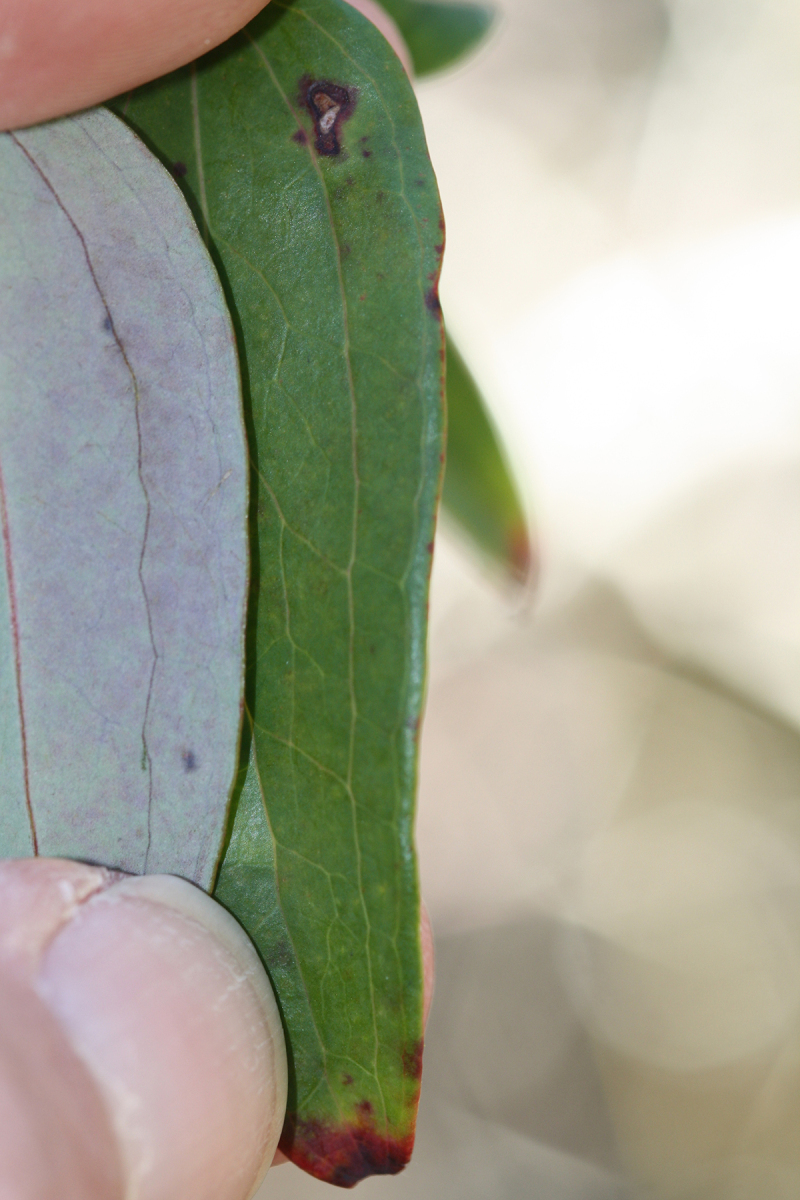
Leaf showing contrast between abaxial surface (left) and adaxial surface (right)

**Figure 96d.**
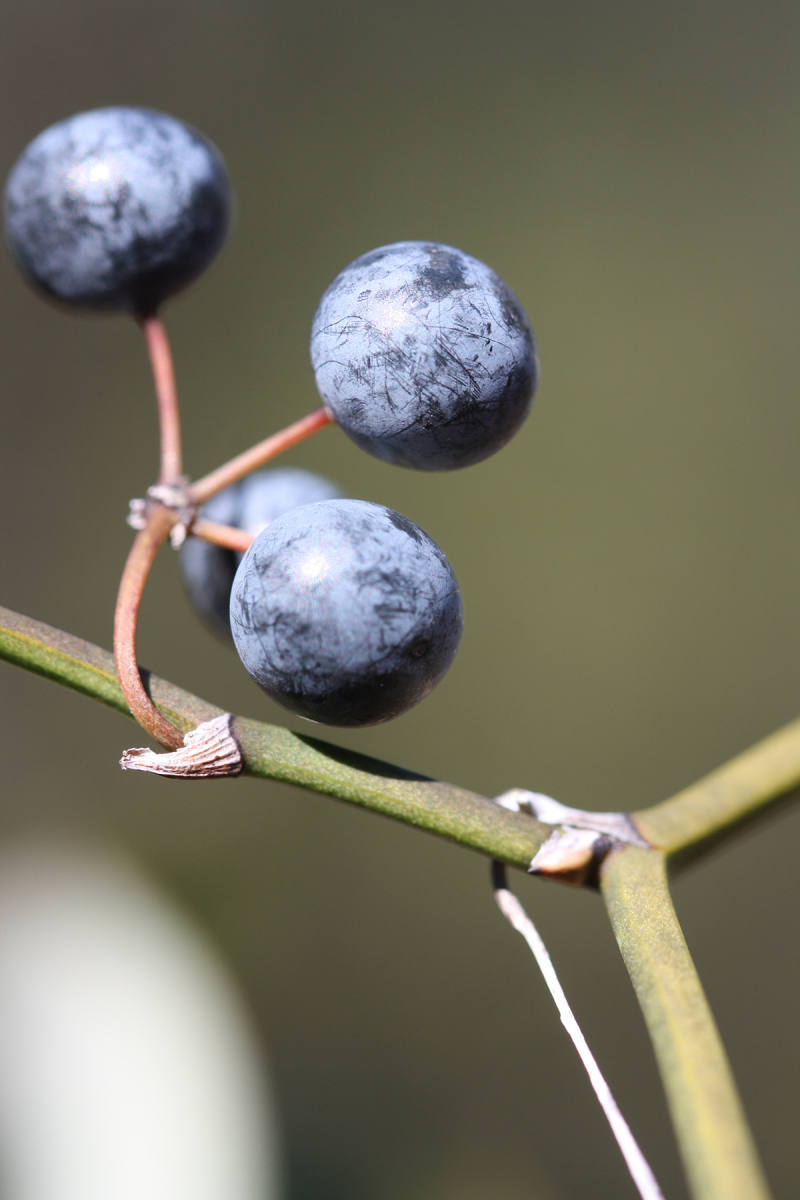
Fruits

**Figure 97a.**
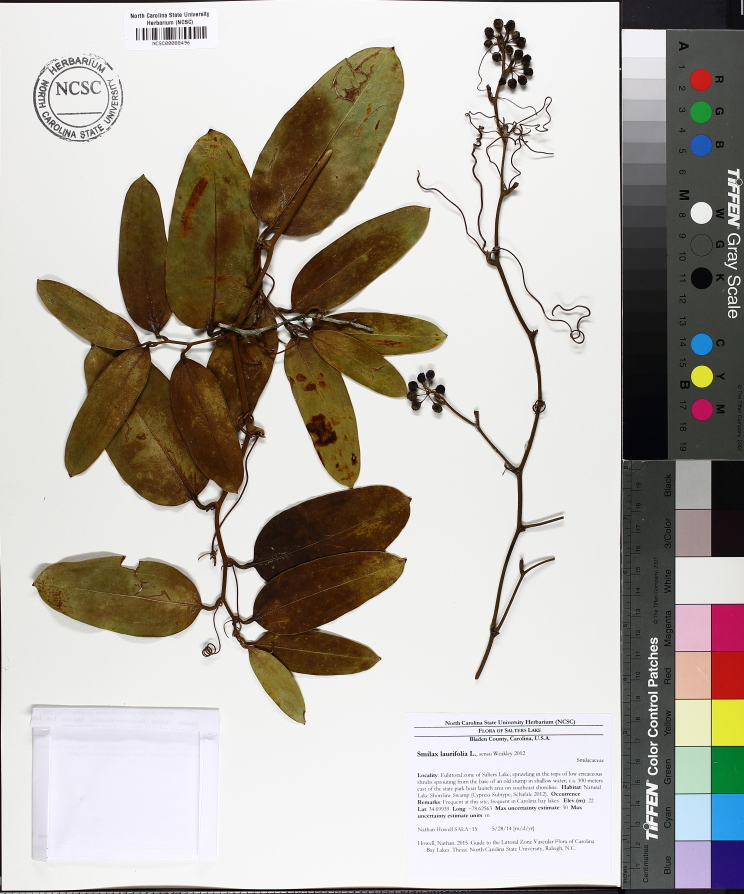
Specimen: *Howell SALA-15* (NCSC)

**Figure 97b.**
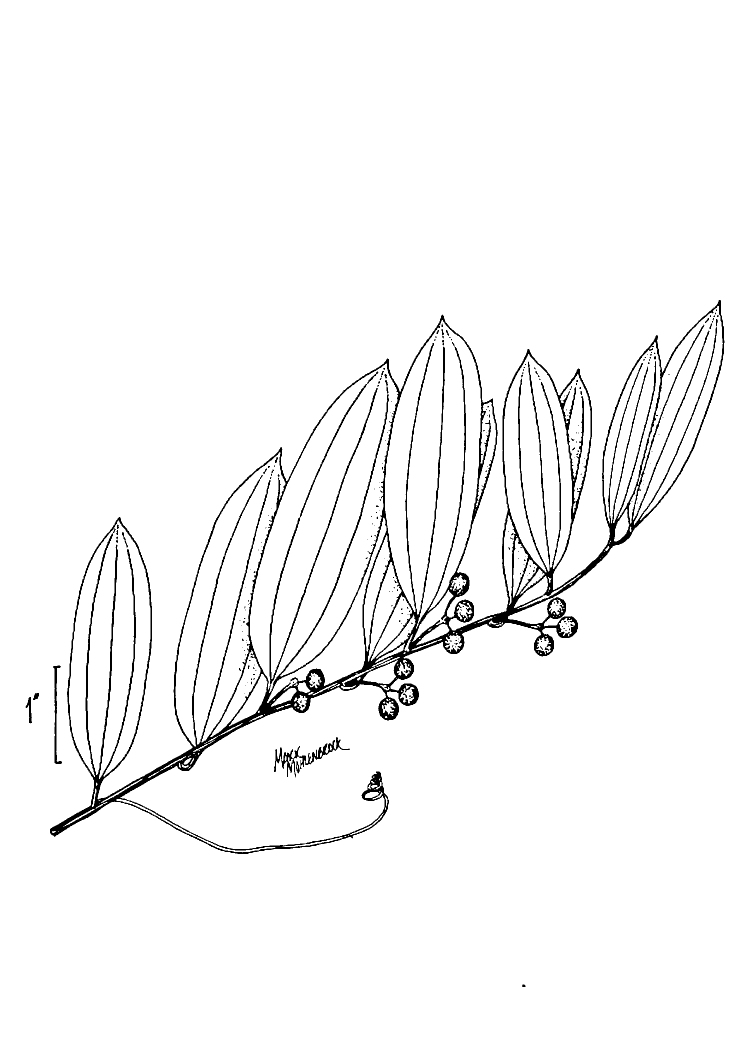
Illustration

**Figure 97c.**
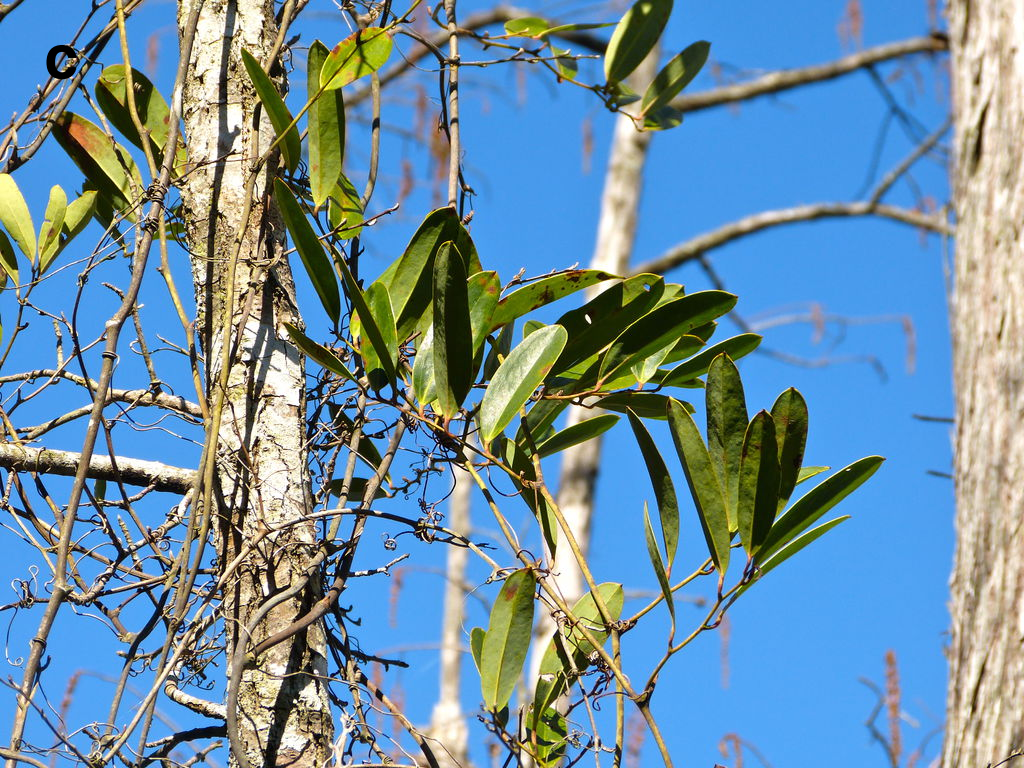
Habit

**Figure 97d.**
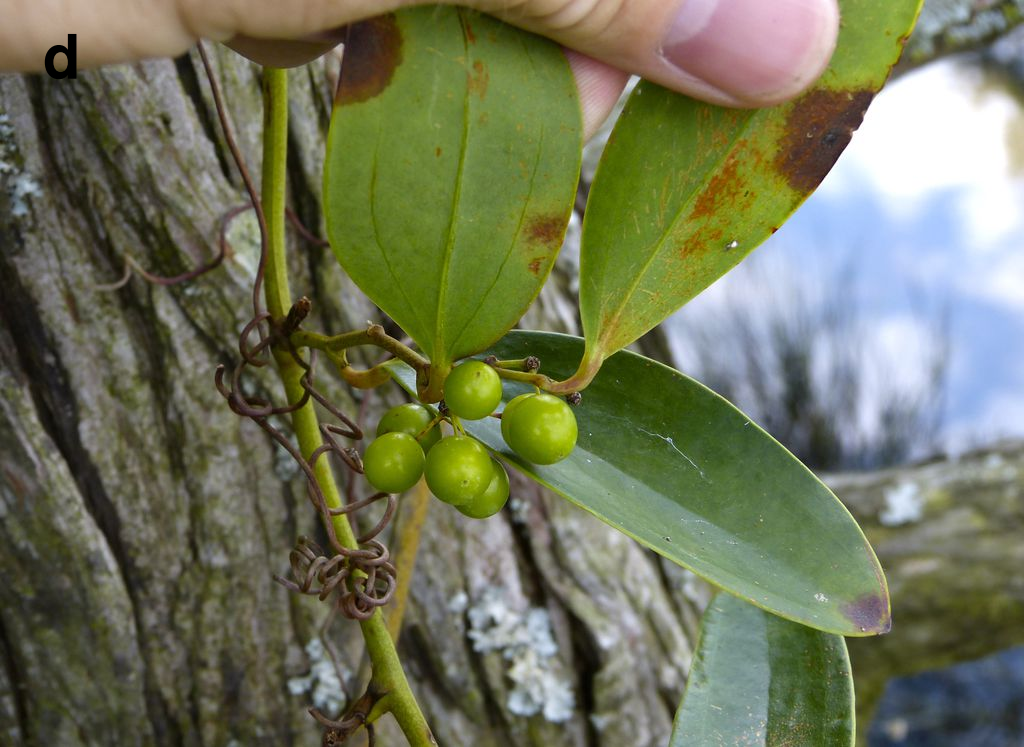
Infructescence

**Figure 98a.**
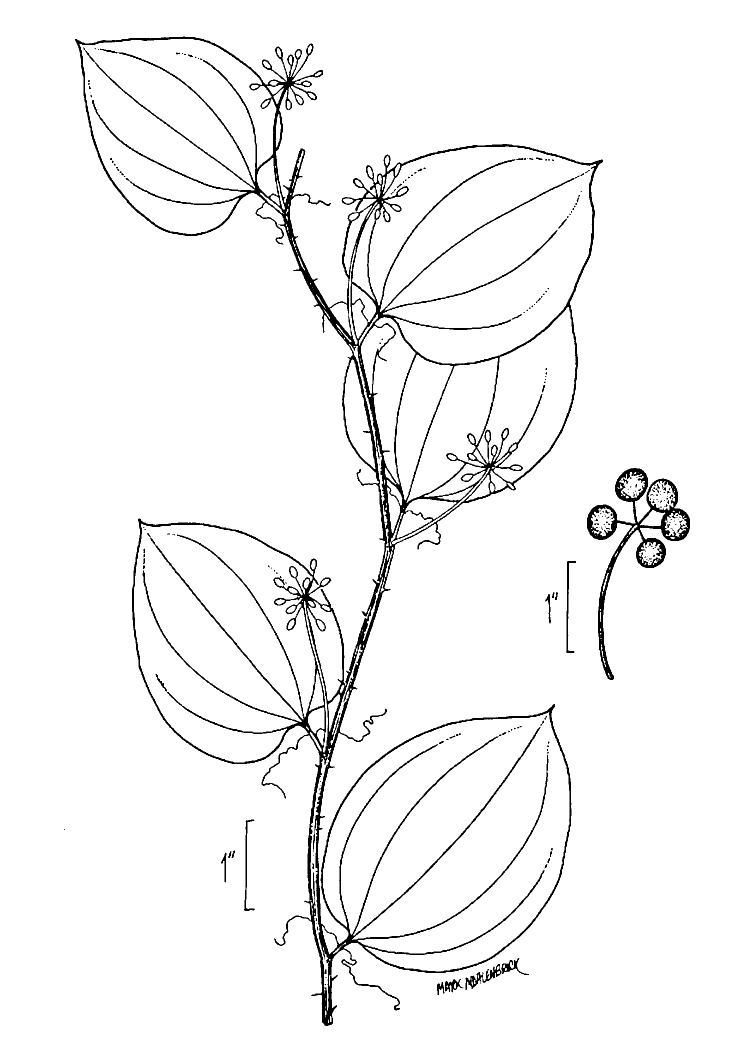
Illustration

**Figure 98b.**
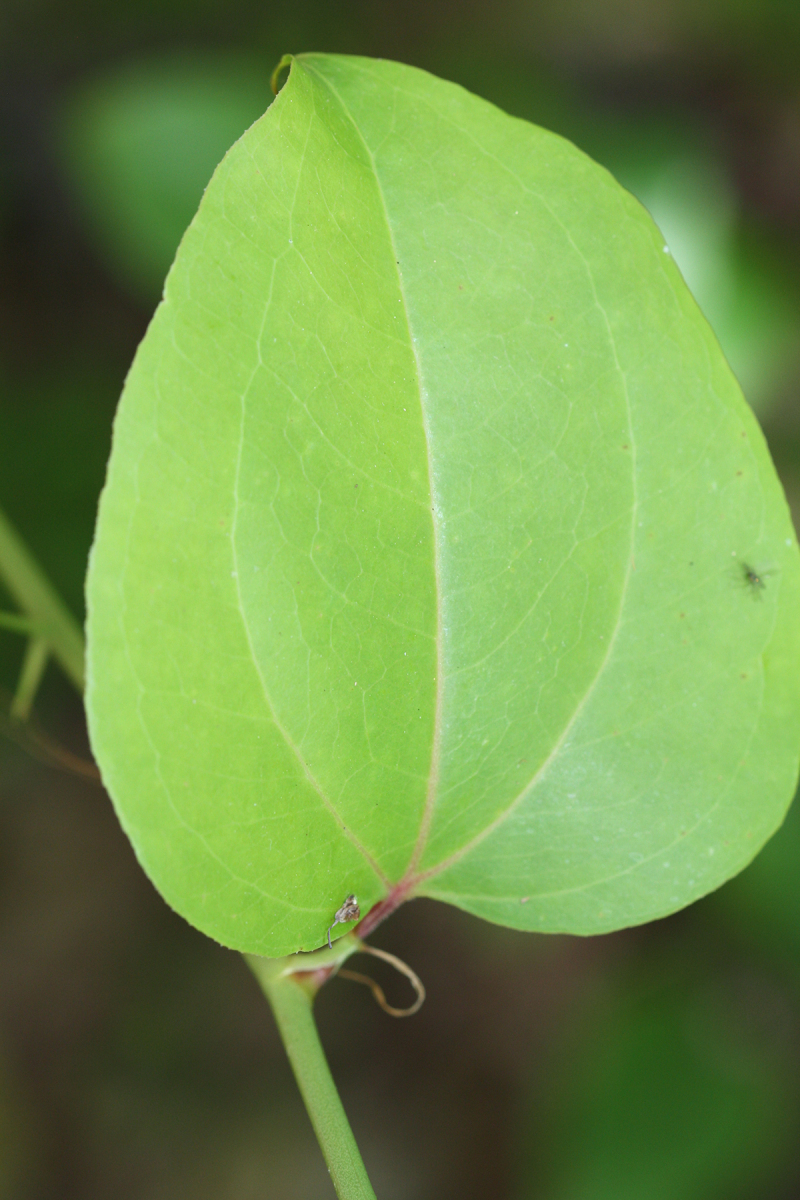
Leaf (adaxial surface)

**Figure 98c.**
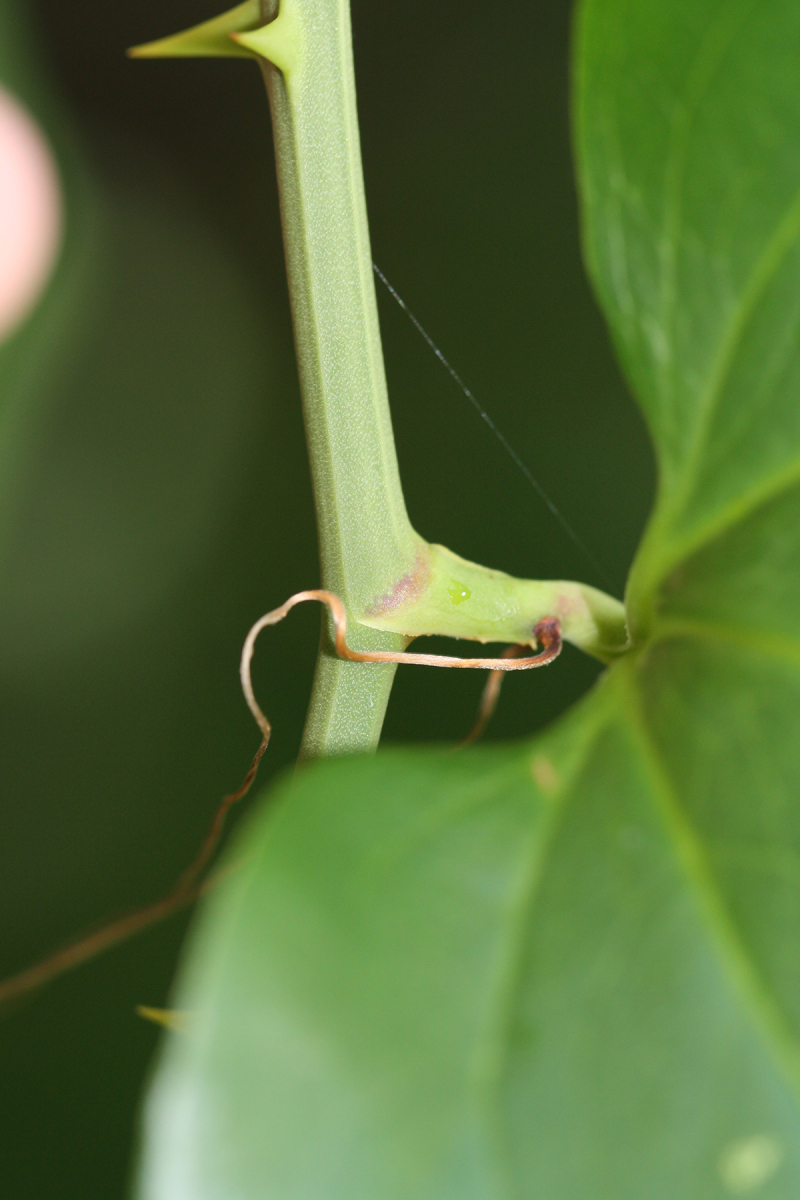
Stem (with prickles), petiole, and withered stipular tendrils (at base of petiole)

**Figure 98d.**
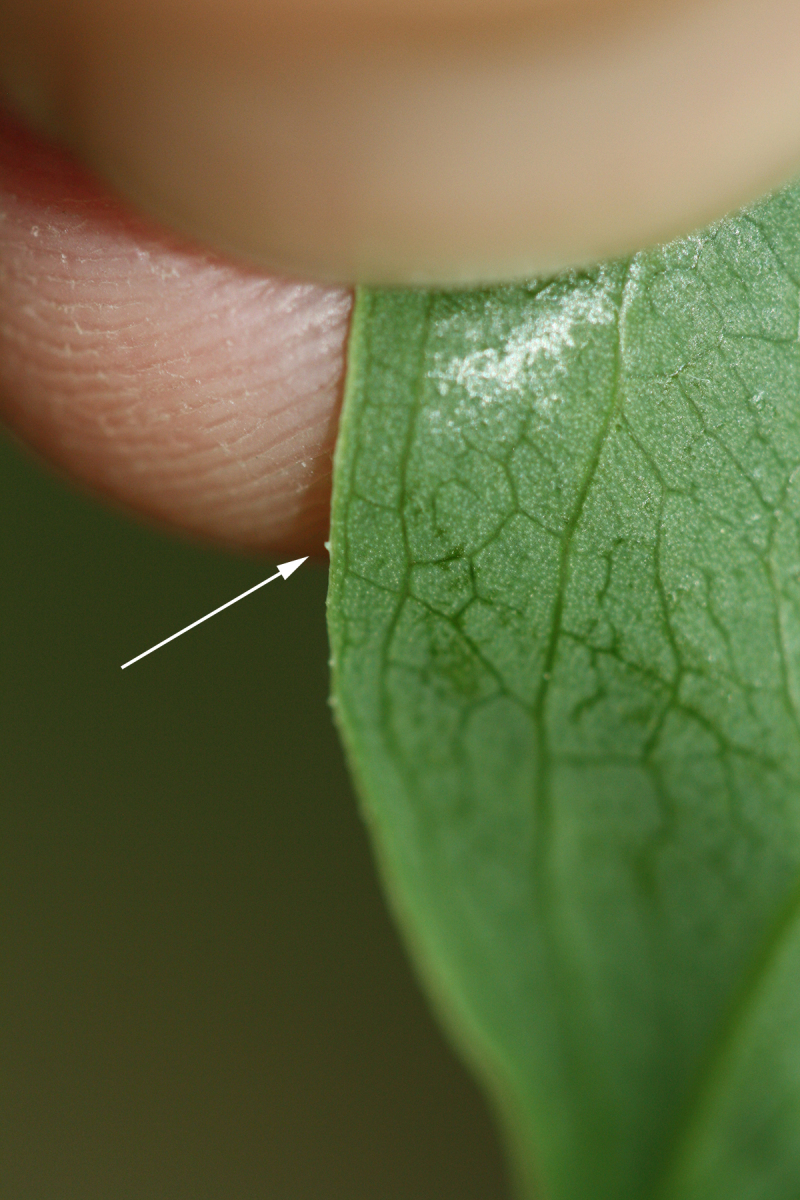
Denticulations at base of leaf blade (arrowed)

**Figure 99a.**
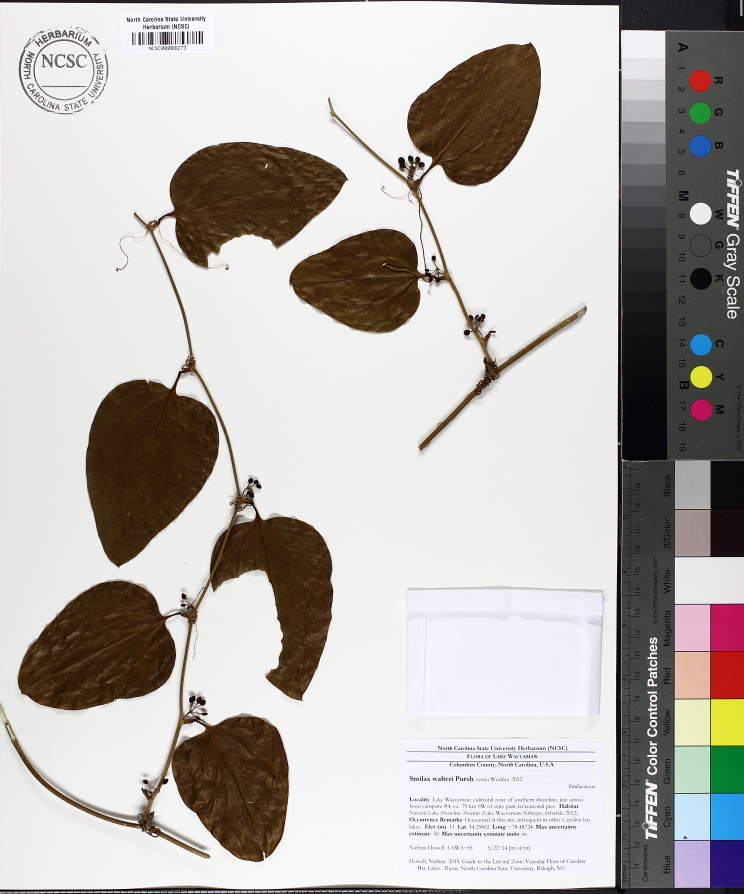
Specimen: *Howell LAWA-55* (NCSC)

**Figure 99b.**
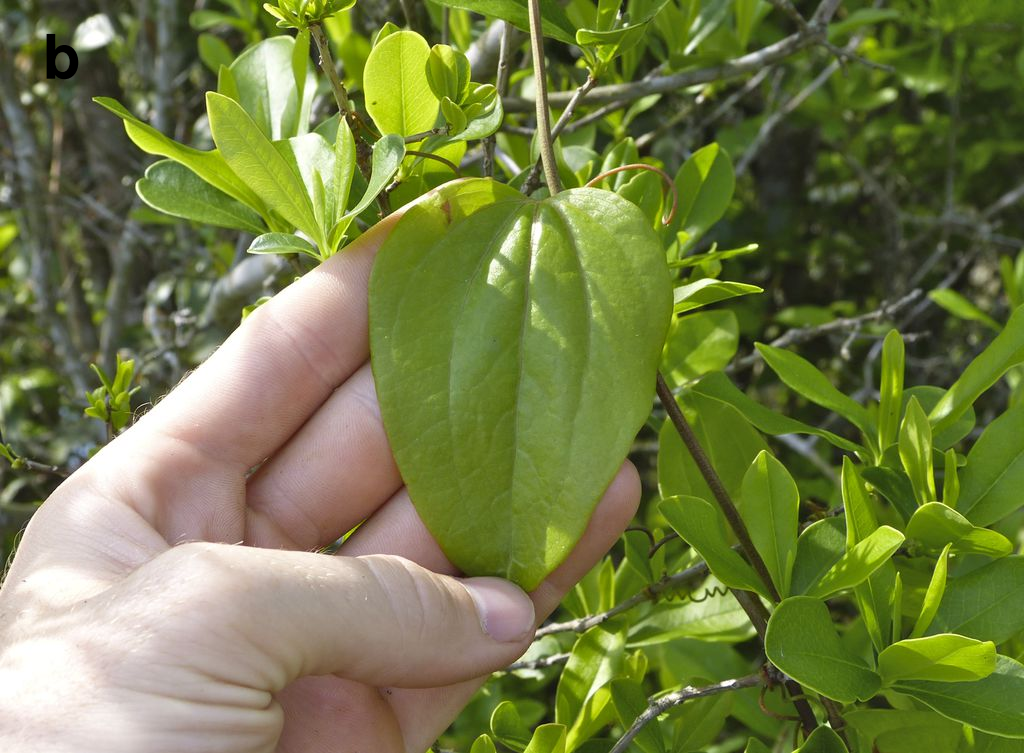
Leaf (adaxial surface)

**Figure 99c.**
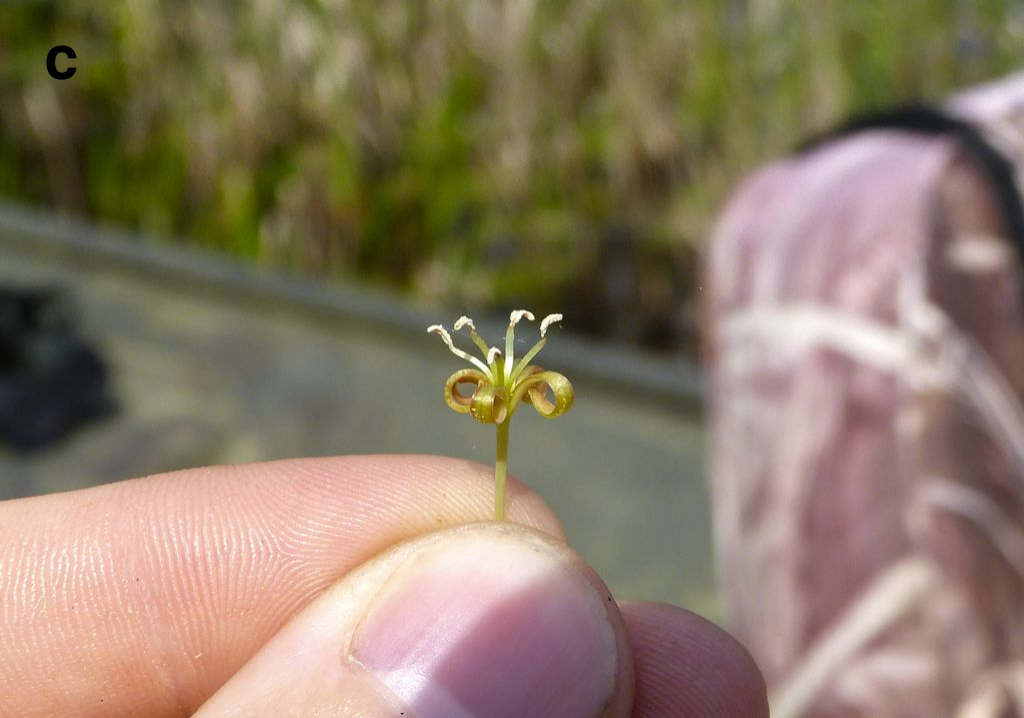
Flower

**Figure 99d.**
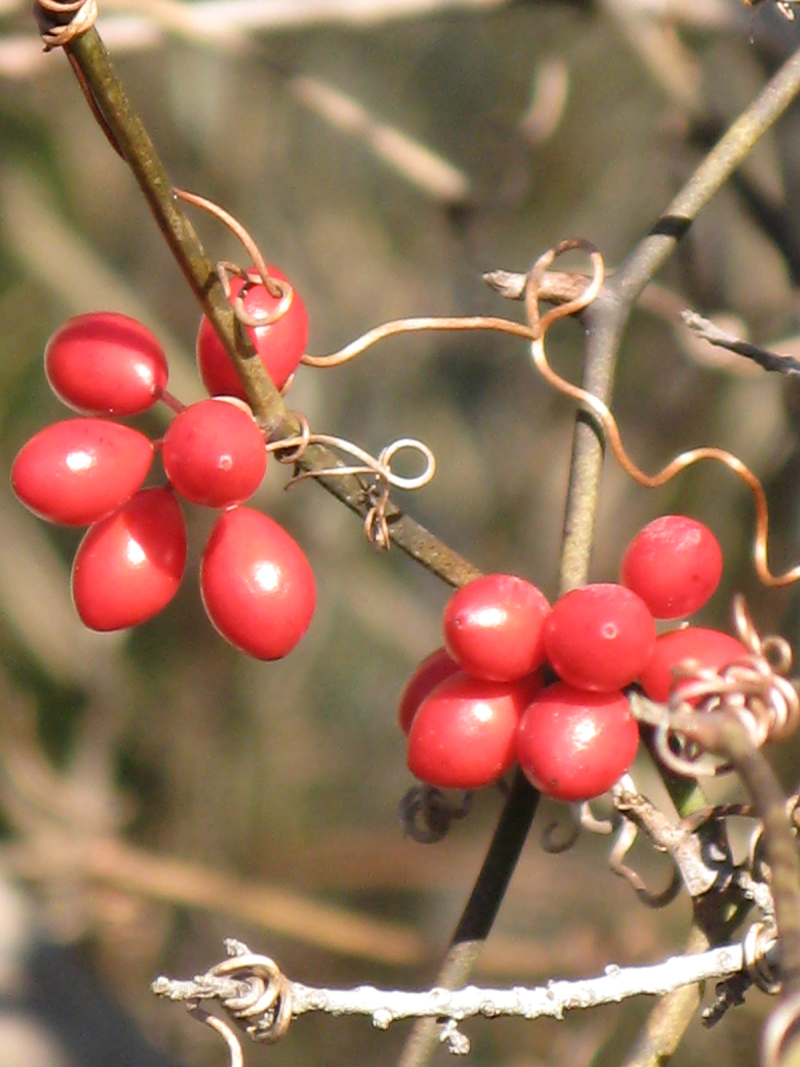
Fruits

**Figure 101a.**
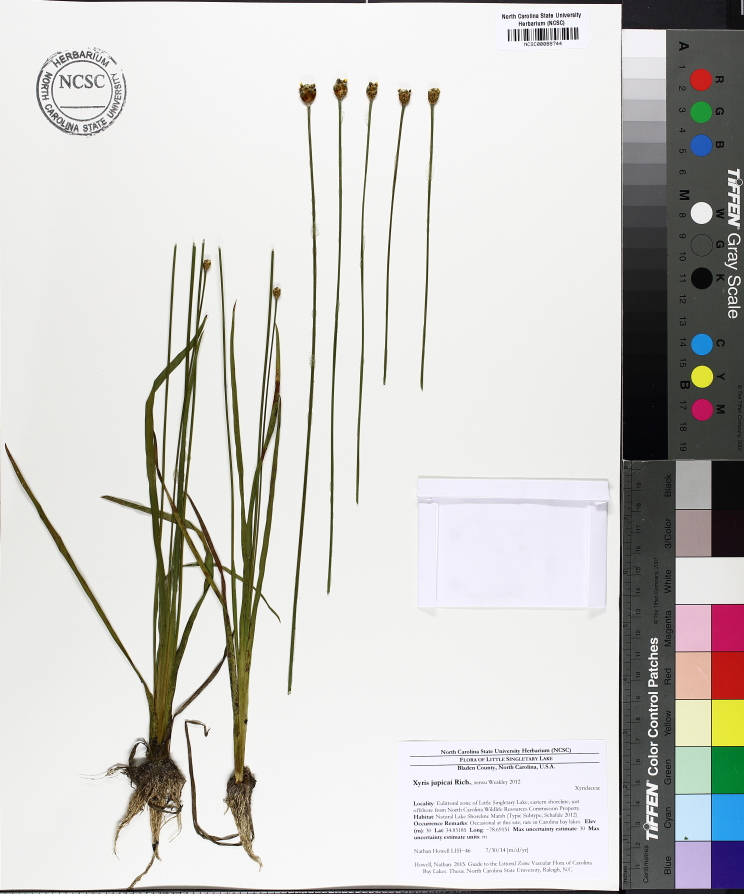
Specimen: *Howell LISI-46* (NCSC)

**Figure 101b.**
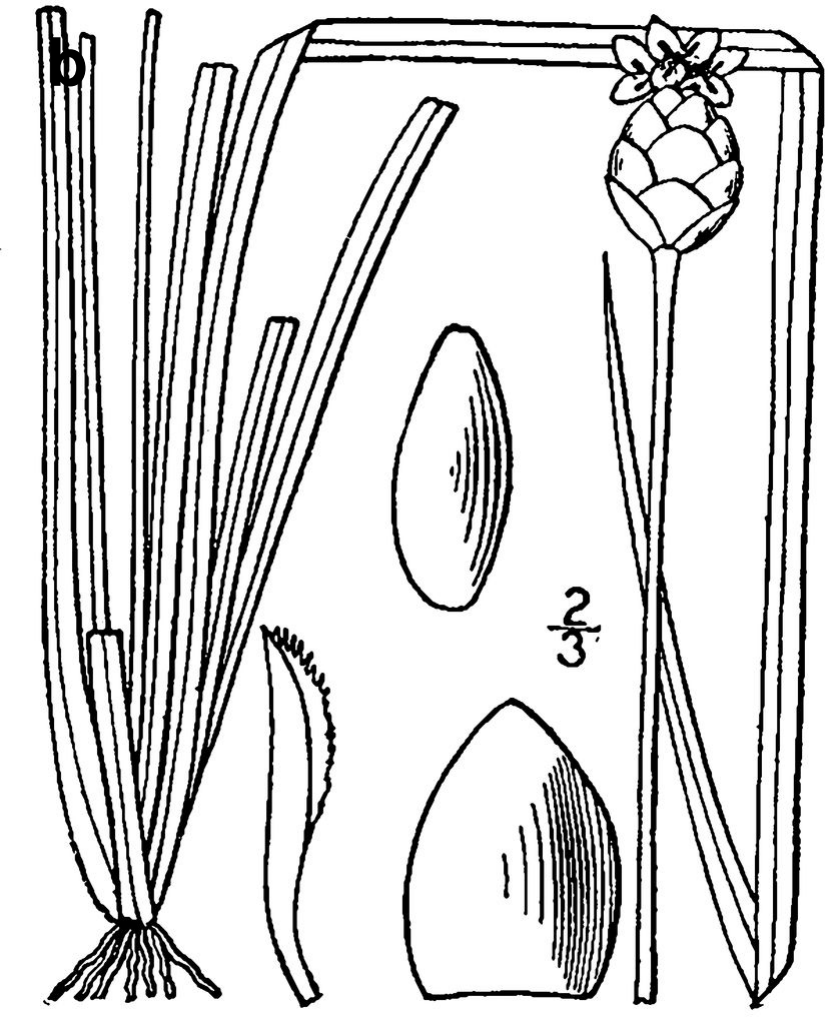
Illustration

**Figure 102a.**
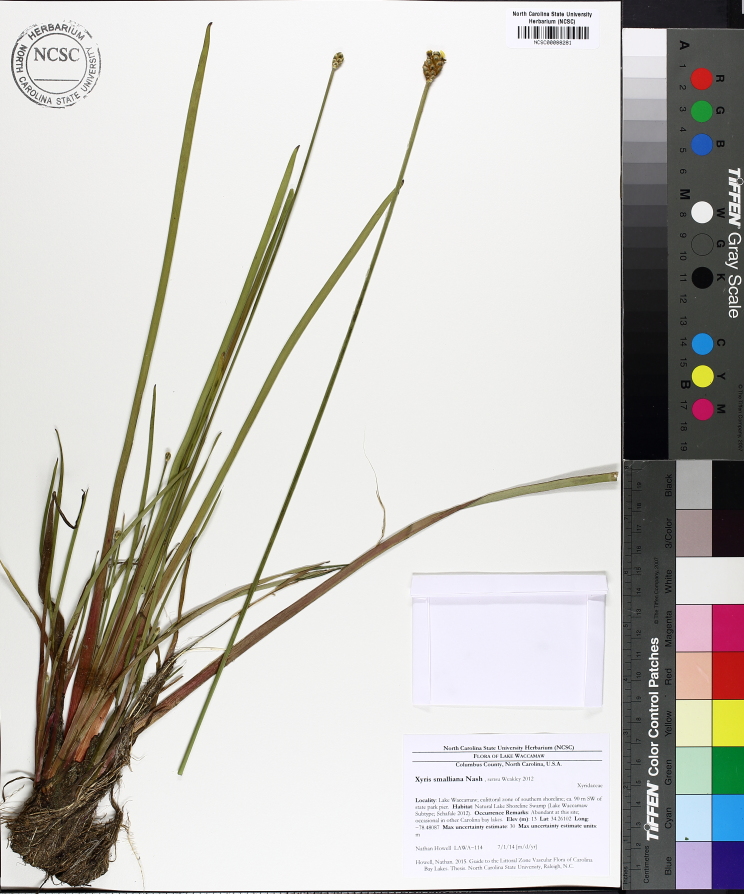
Specimen: *Howell LAWA-114* (NCSC)

**Figure 102b.**
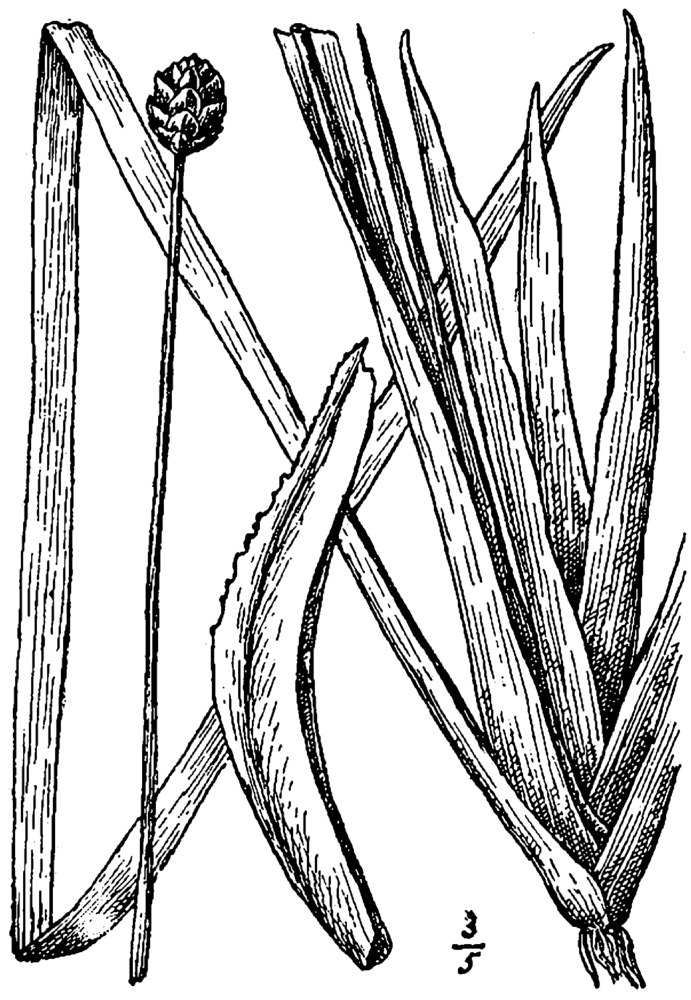
Illustration

**Figure 102c.**
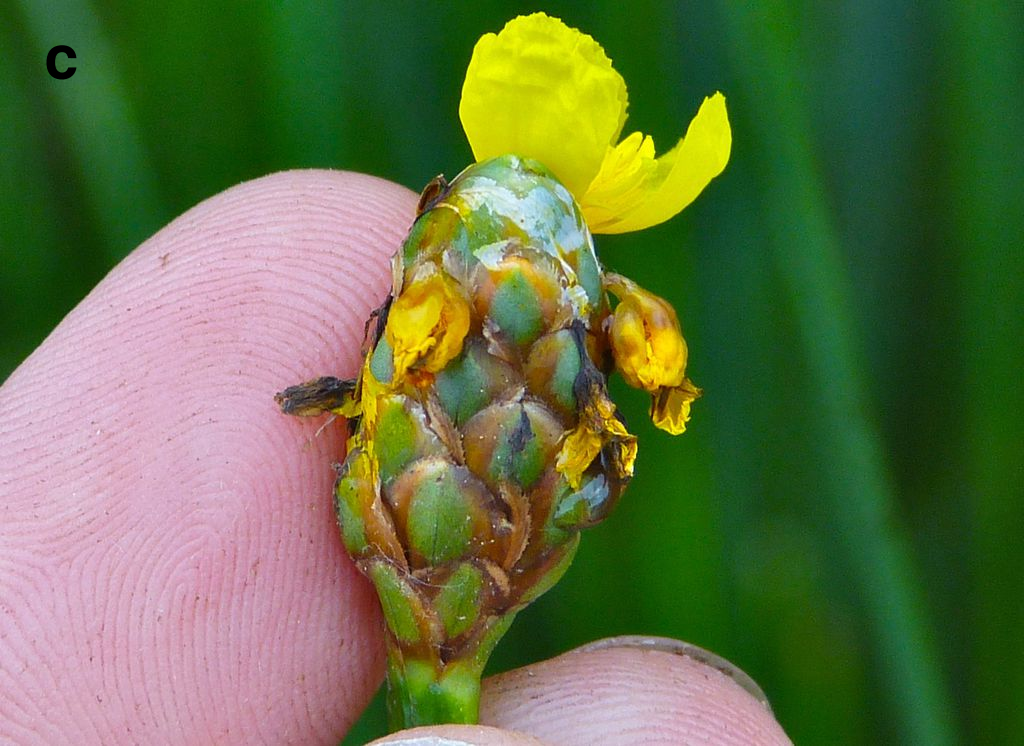
Inflorescence

**Figure 102d.**
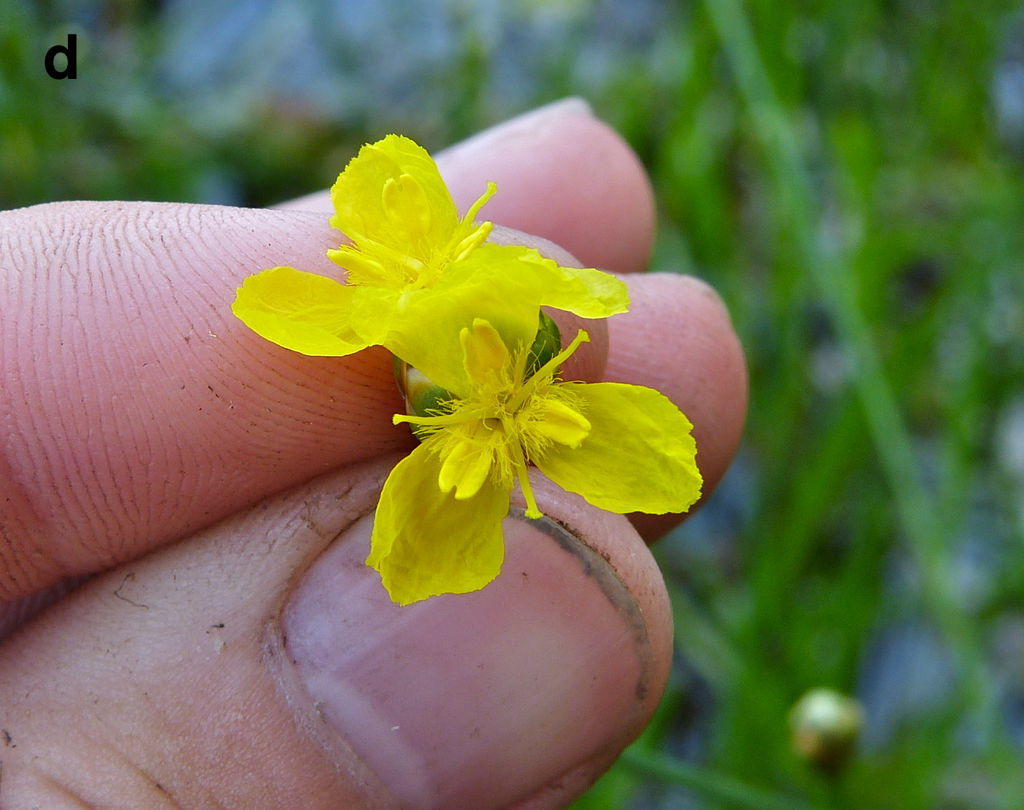
Flowers

**Figure 103a.**
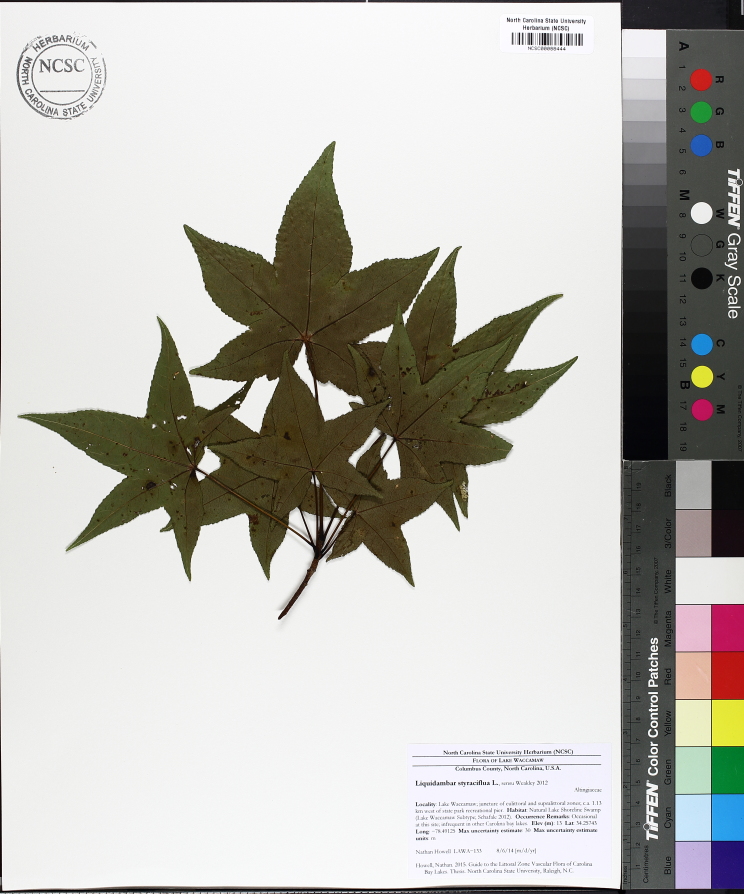
Specimen: *Howell LAWA-133* (NCSC)

**Figure 103b.**
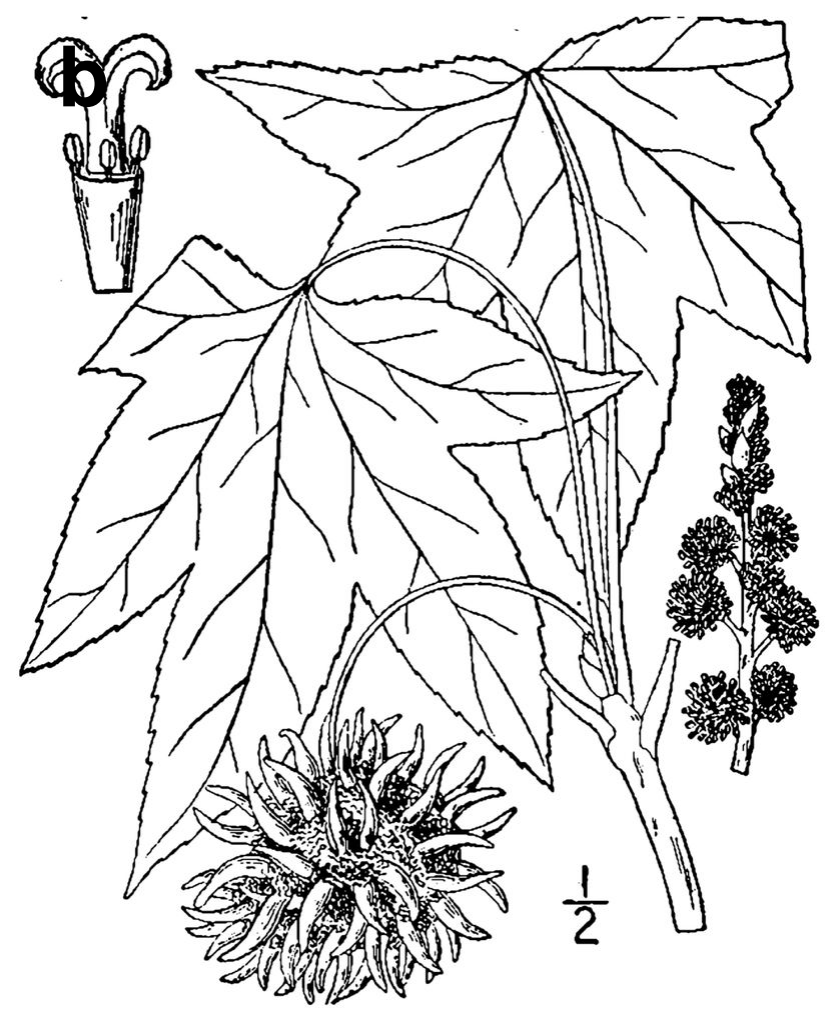
Illustration

**Figure 103c.**
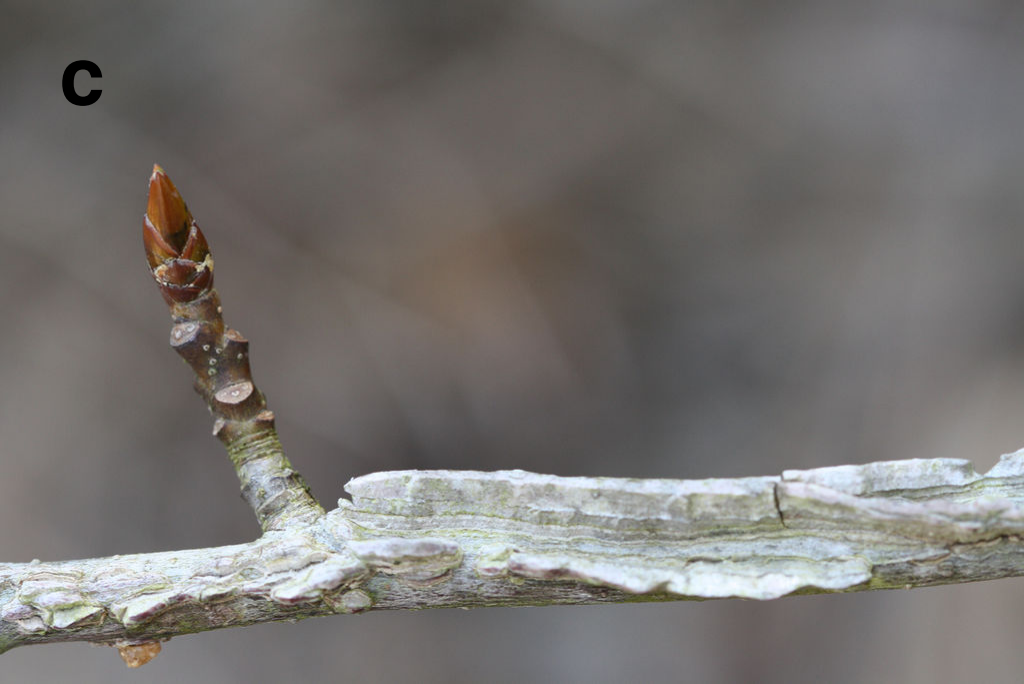
Twig, bud, and leaf scars

**Figure 103d.**
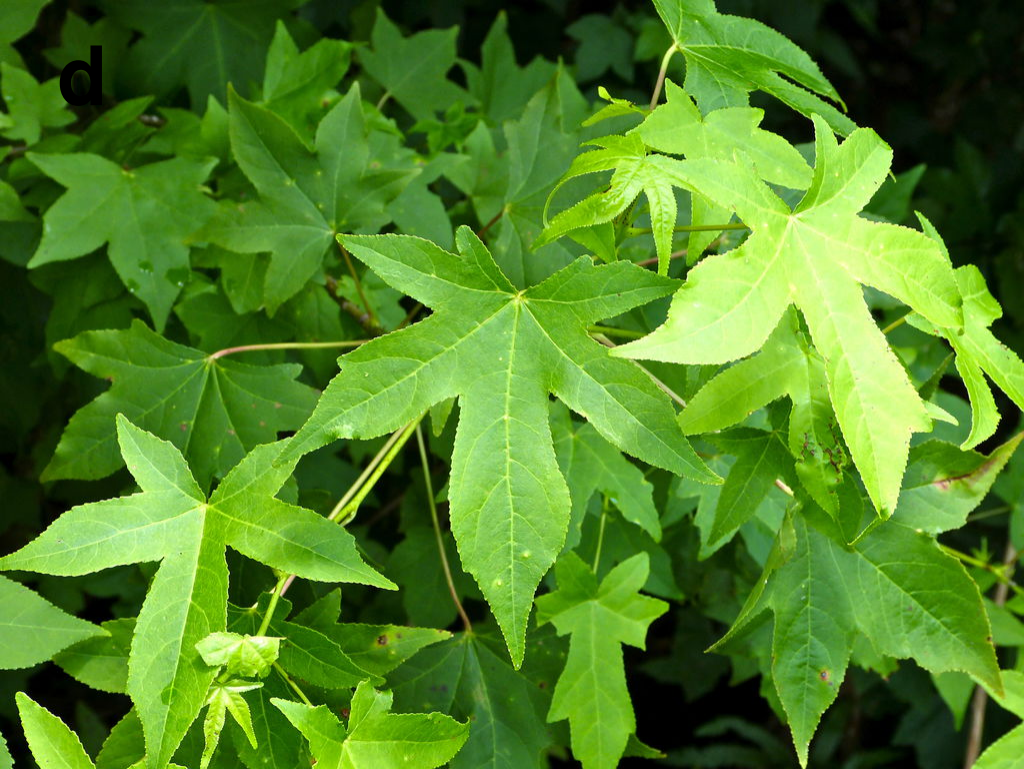
Leaves

**Figure 103e.**
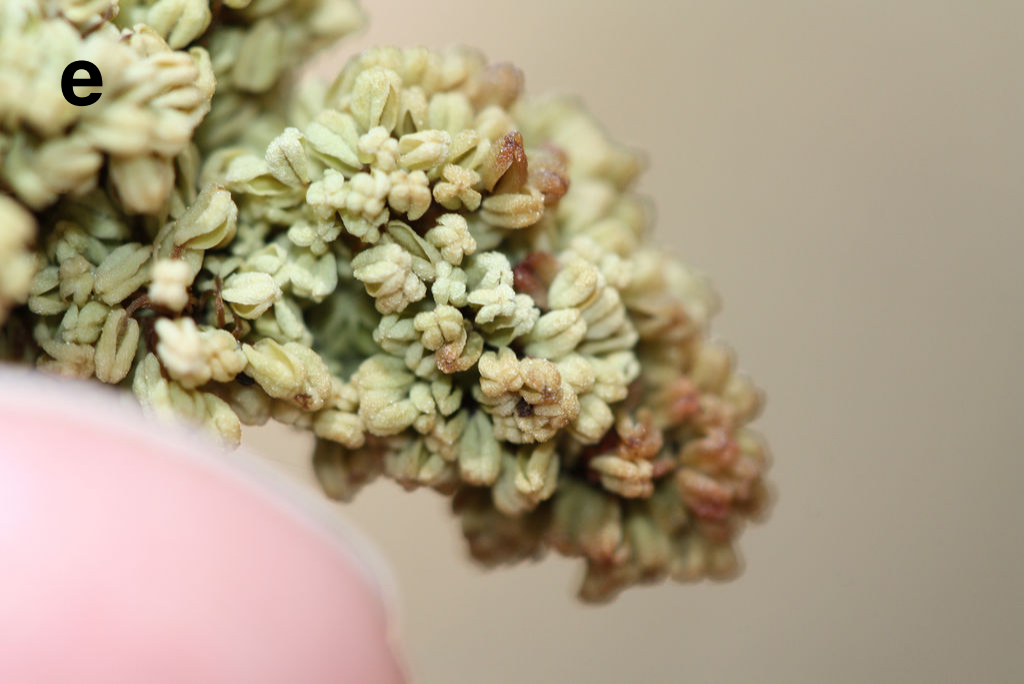
Staminate flowers

**Figure 103f.**
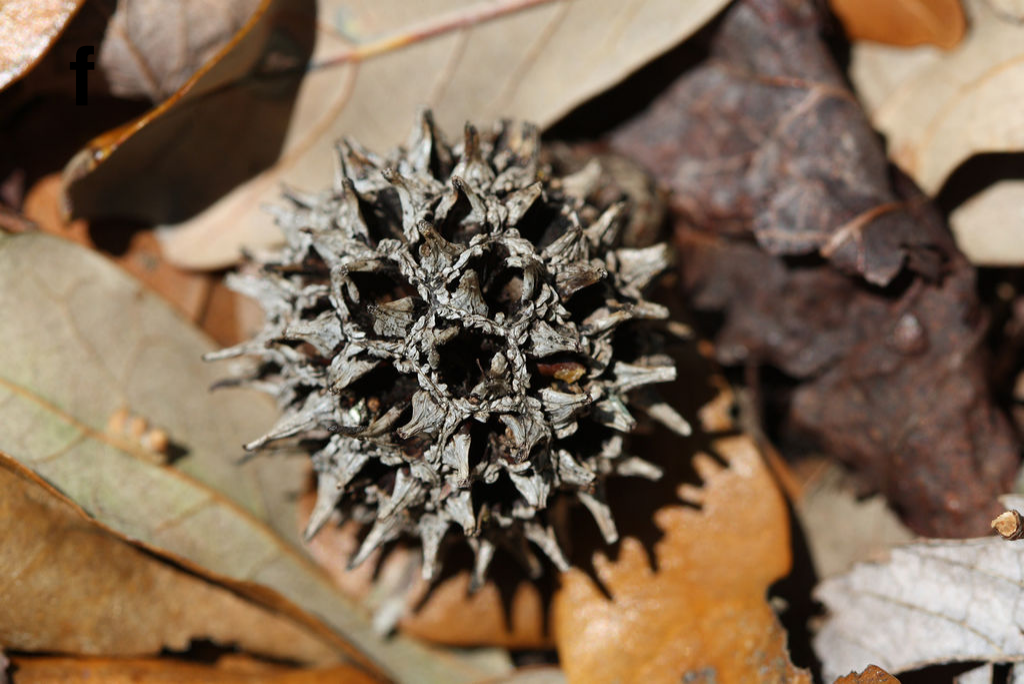
Fruit

**Figure 104a.**
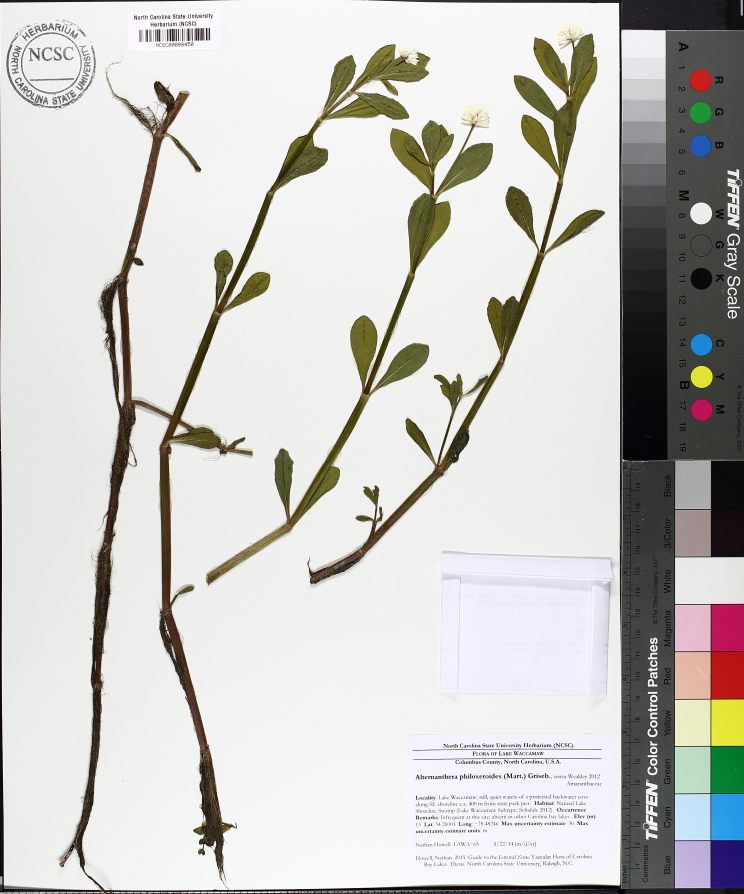
Specimen: *Howell LAWA-65* (NCSC)

**Figure 104b.**
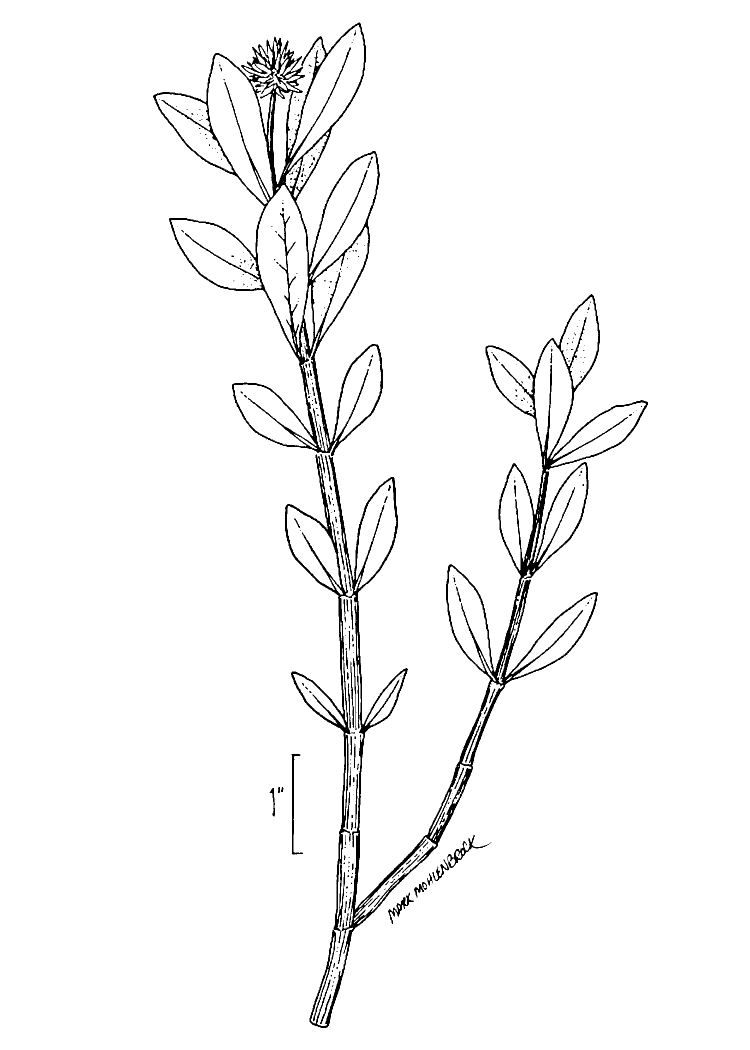
Illustration

**Figure 104c.**
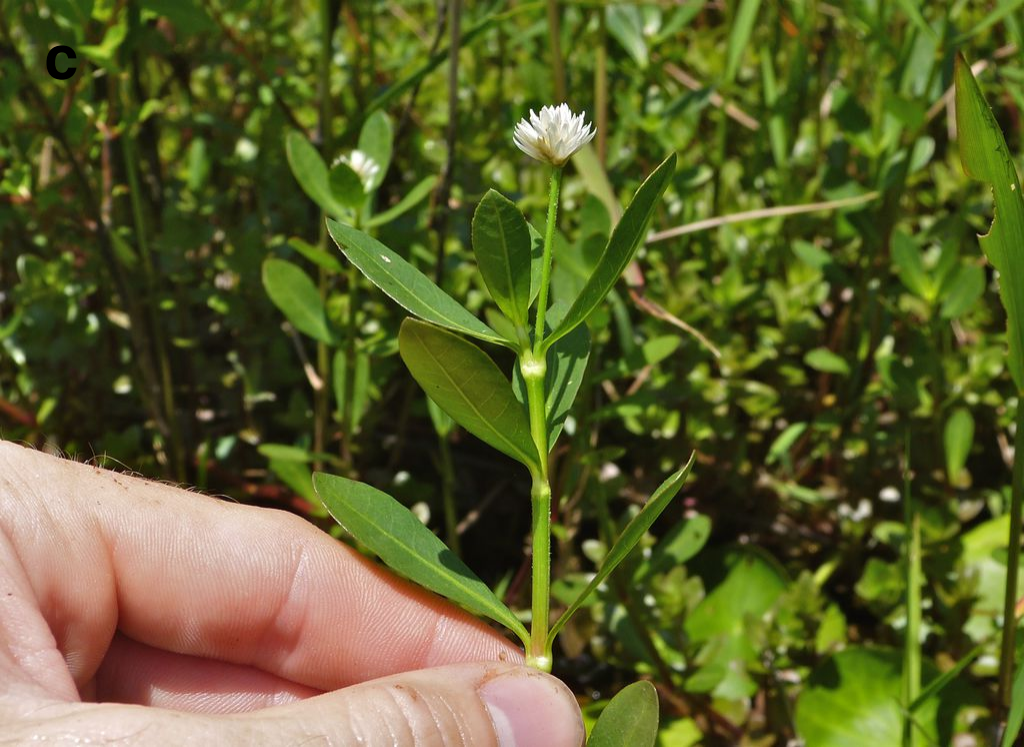
Habit

**Figure 104d.**
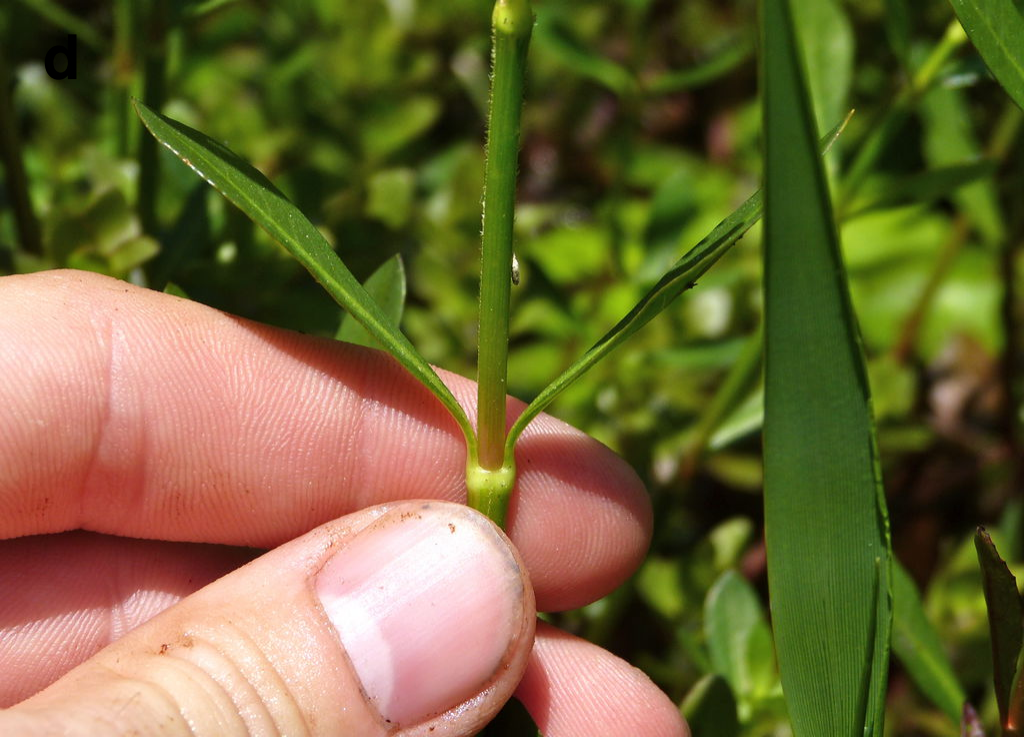
Node

**Figure 104e.**
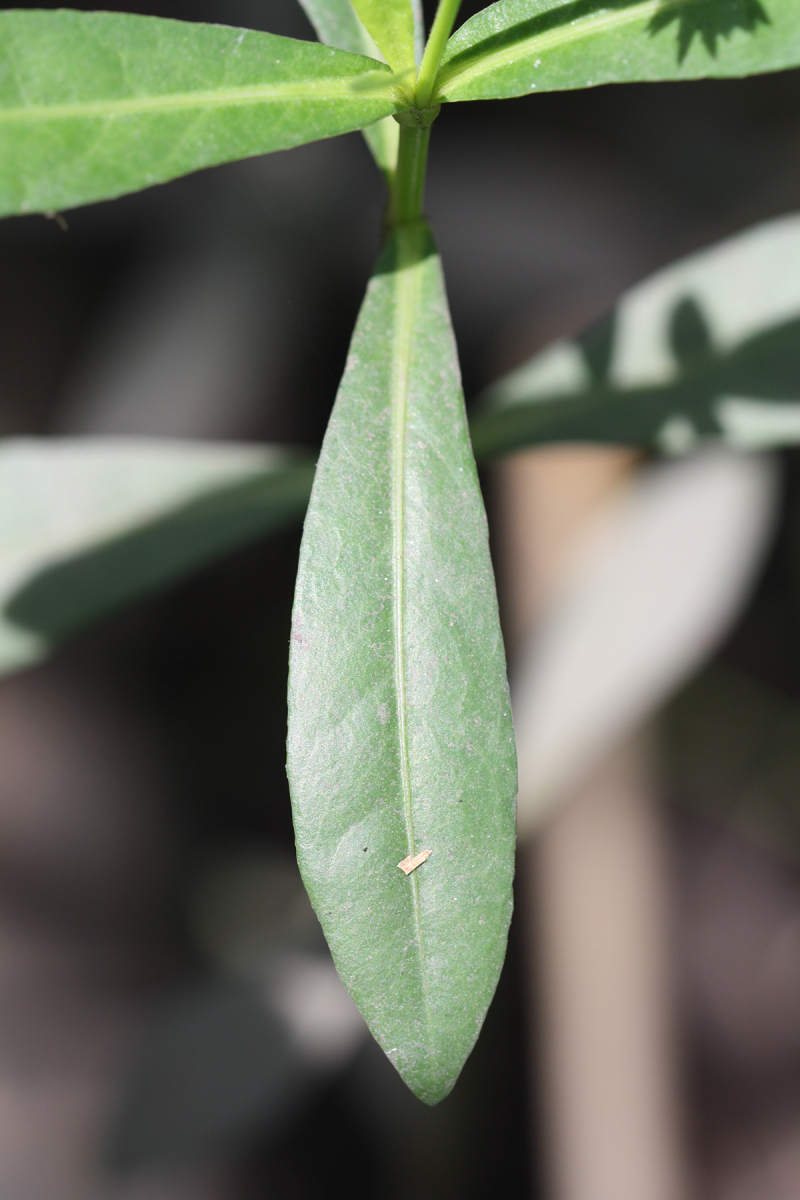
Leaf

**Figure 104f.**
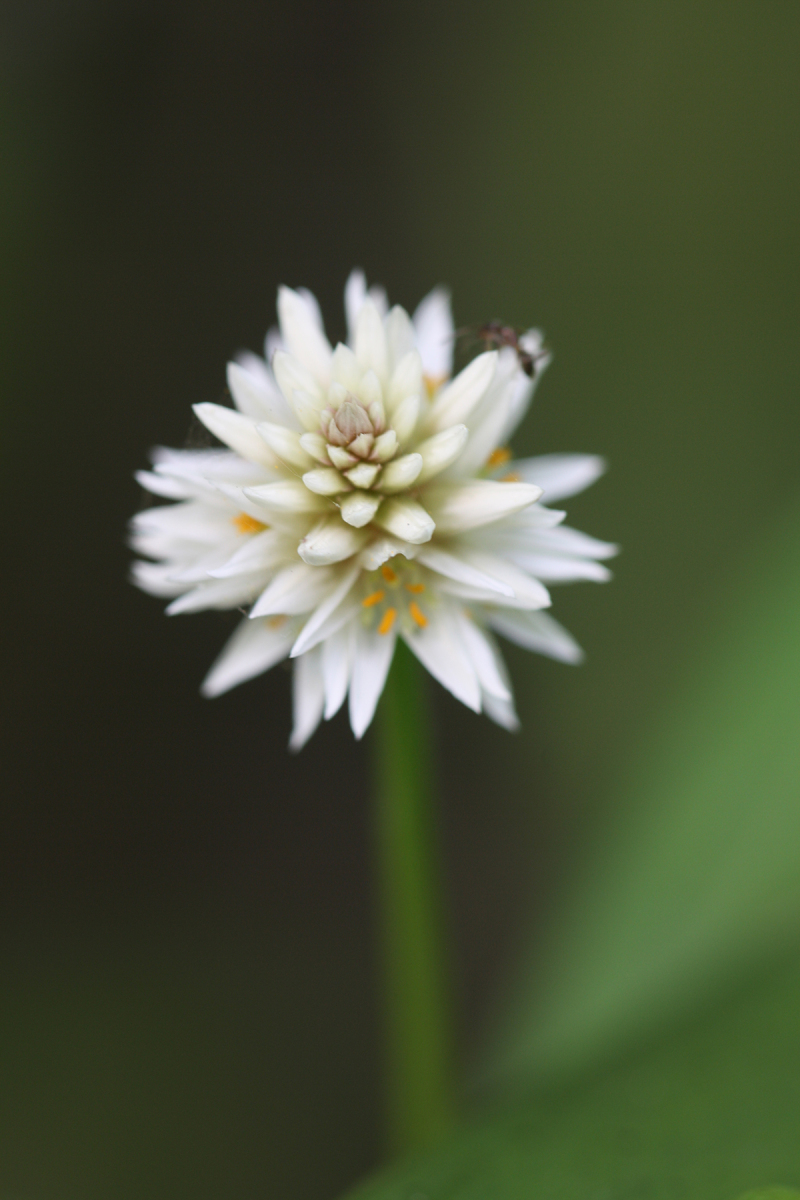
Inflorescence

**Figure 105a.**
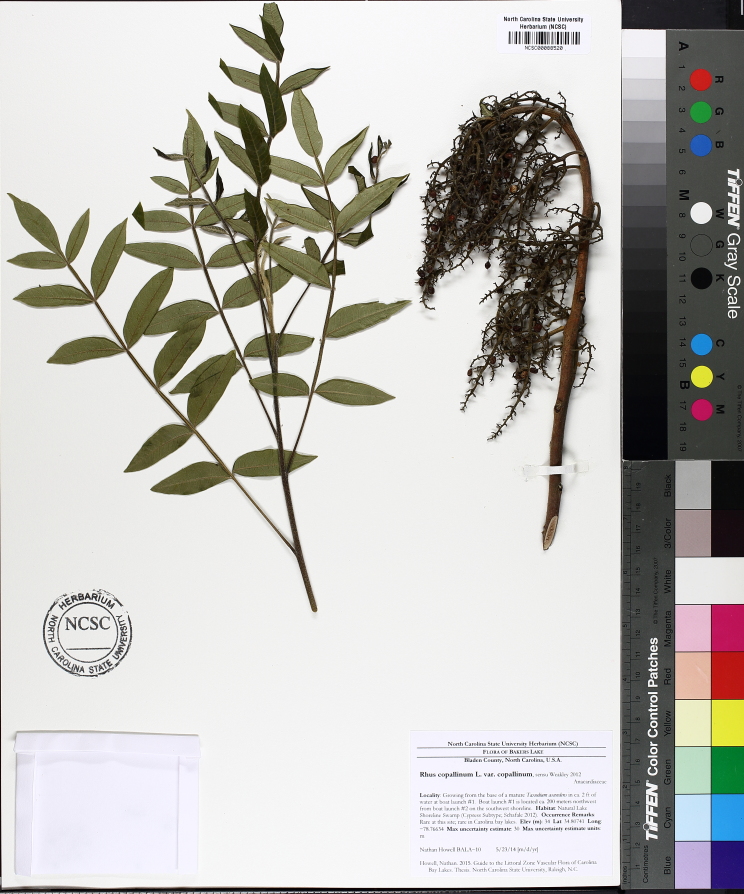
Specimen: *Howell BALA-10* (NCSC)

**Figure 105b.**
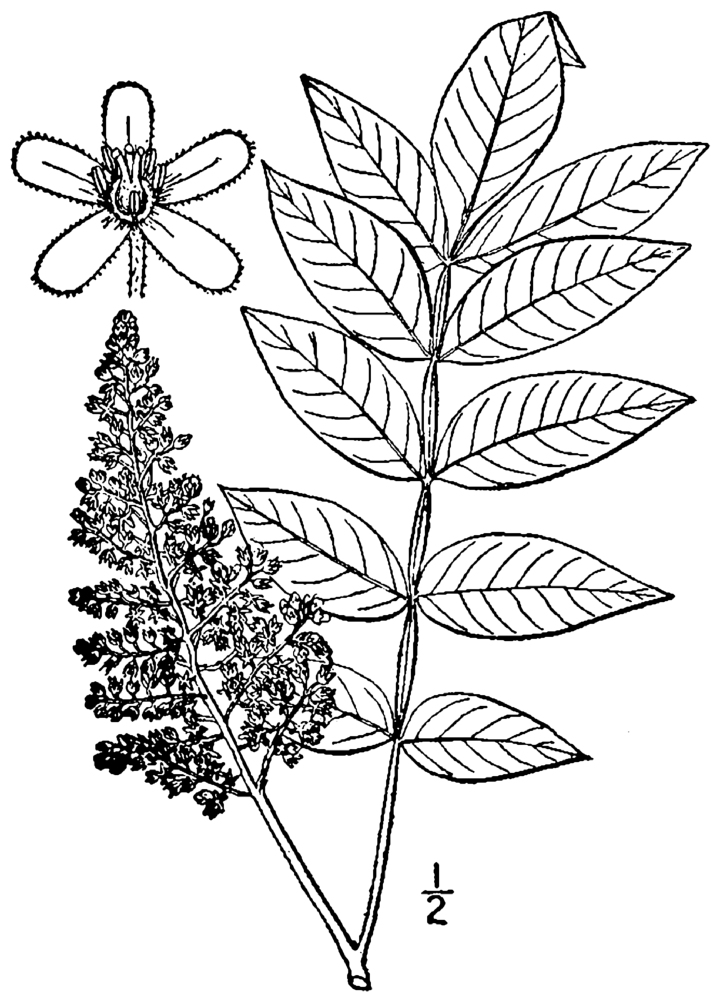
Illustration

**Figure 105c.**
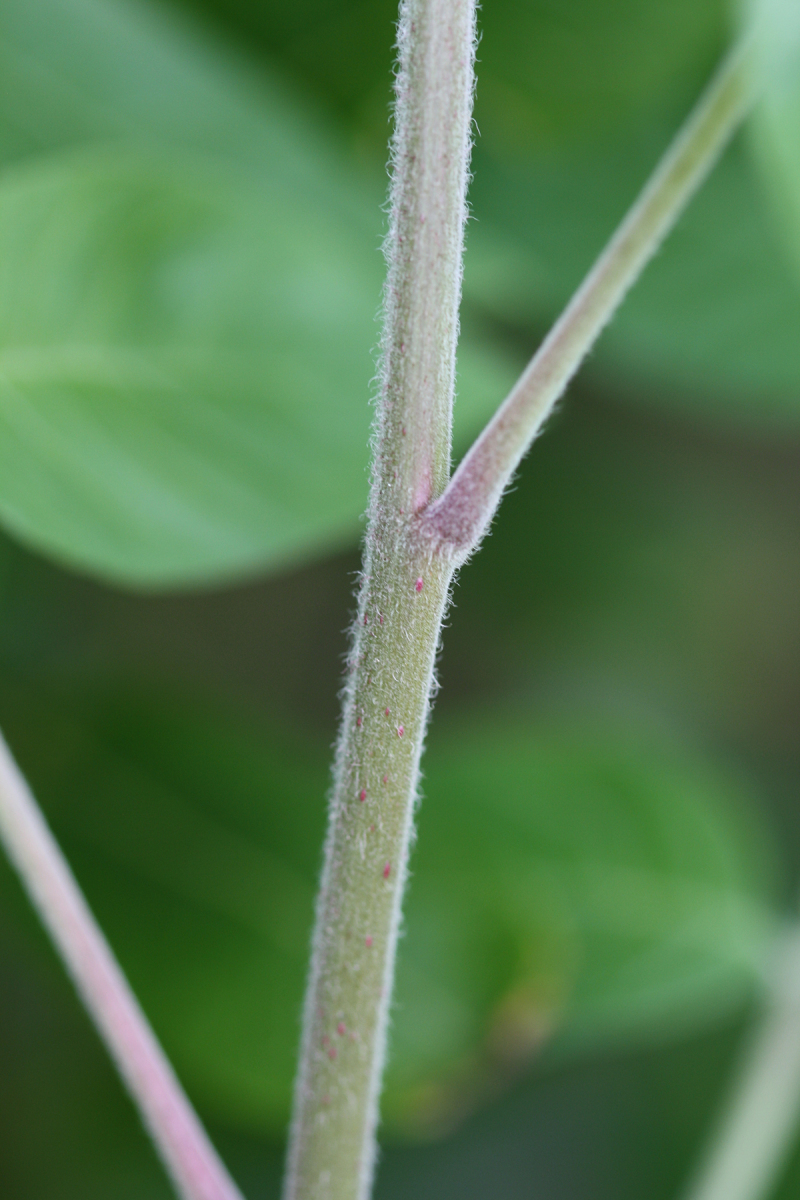
Stem

**Figure 105d.**
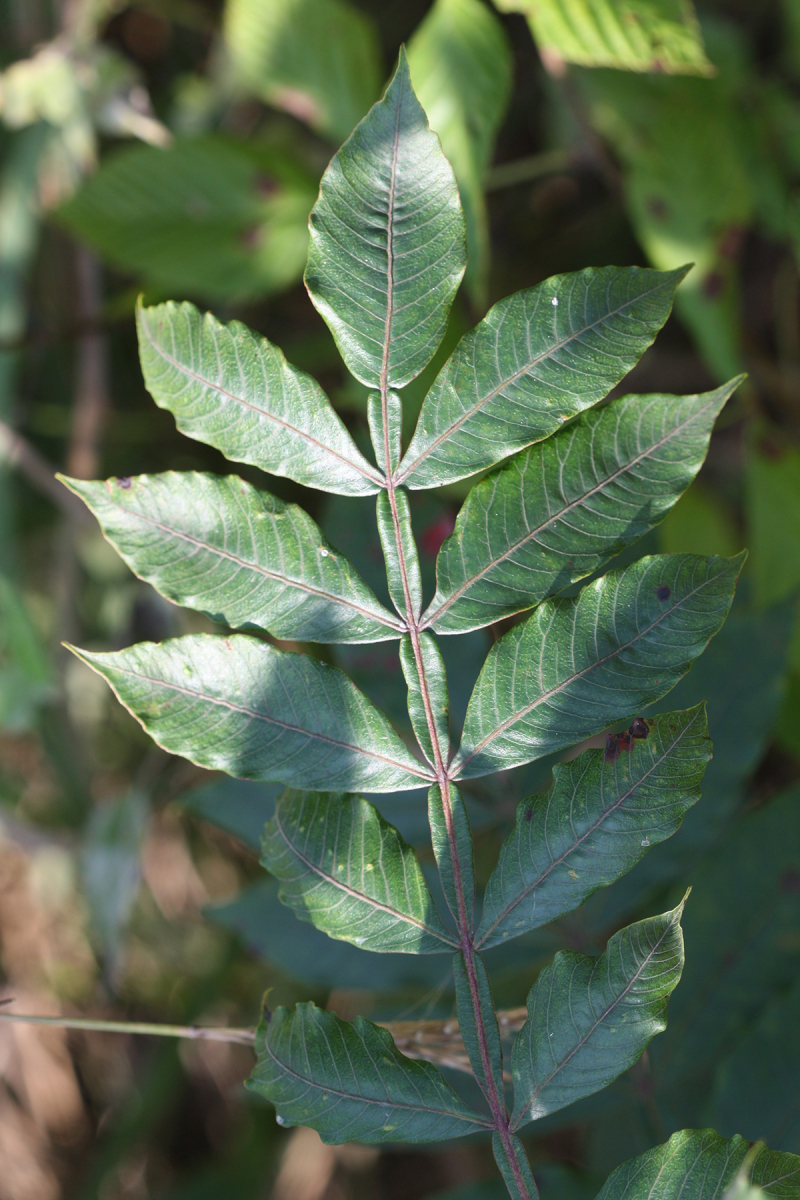
Imparipinnate leaf (with winged rachis)

**Figure 105e.**
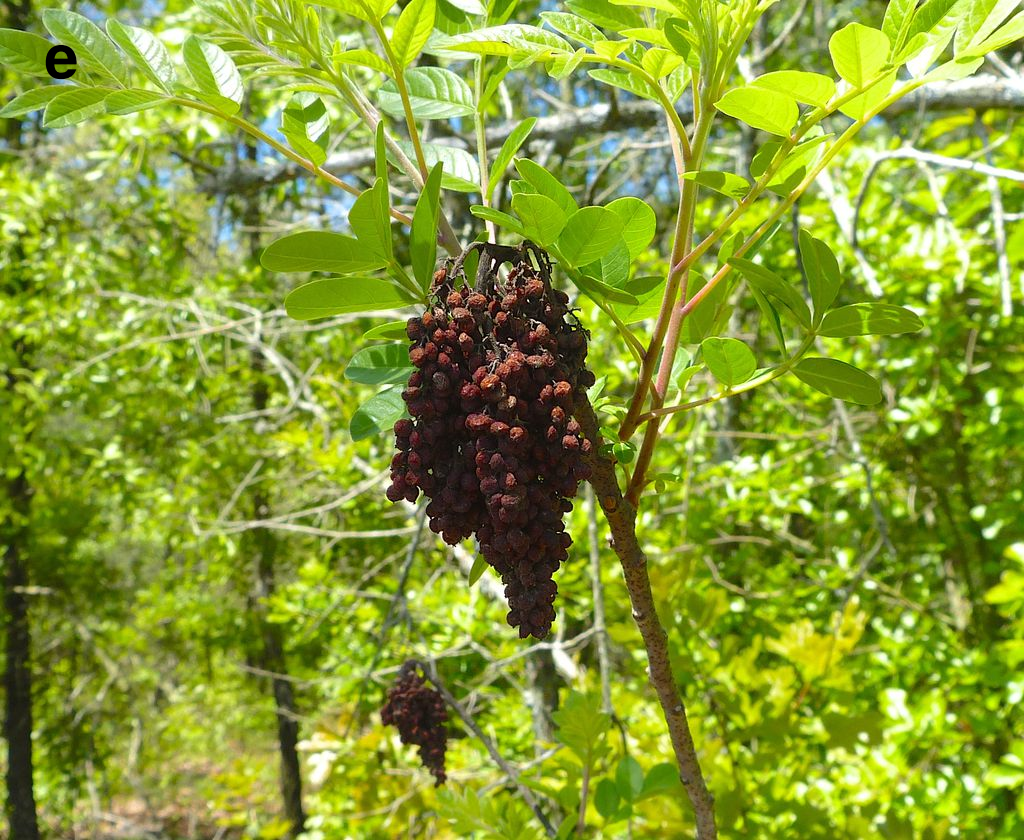
Infructescence

**Figure 105f.**
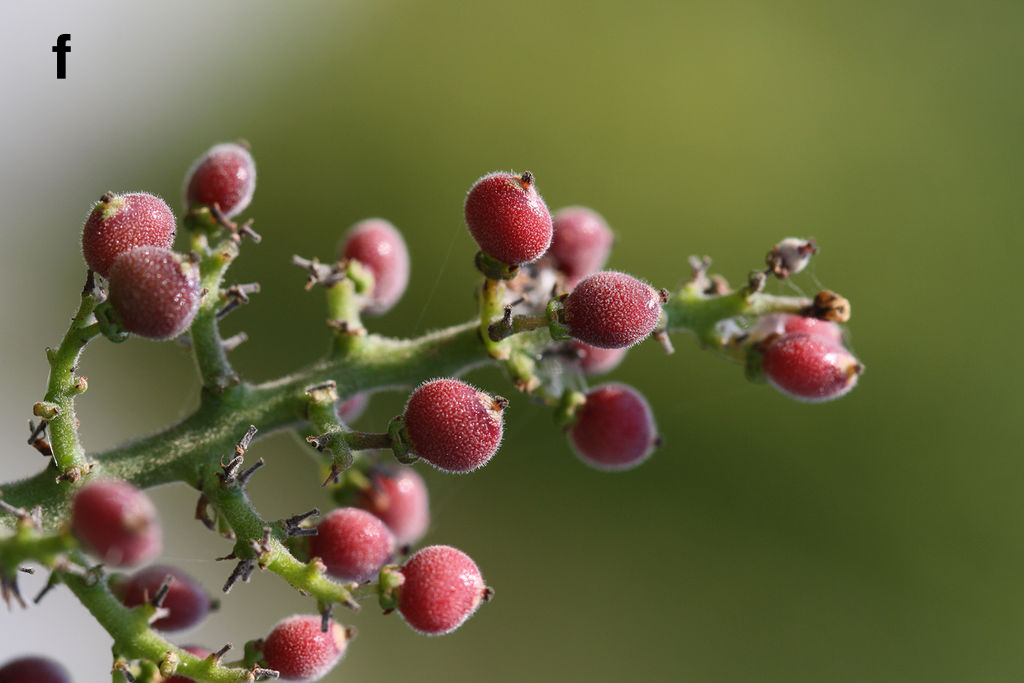
Infructescence (detail)

**Figure 106a.**
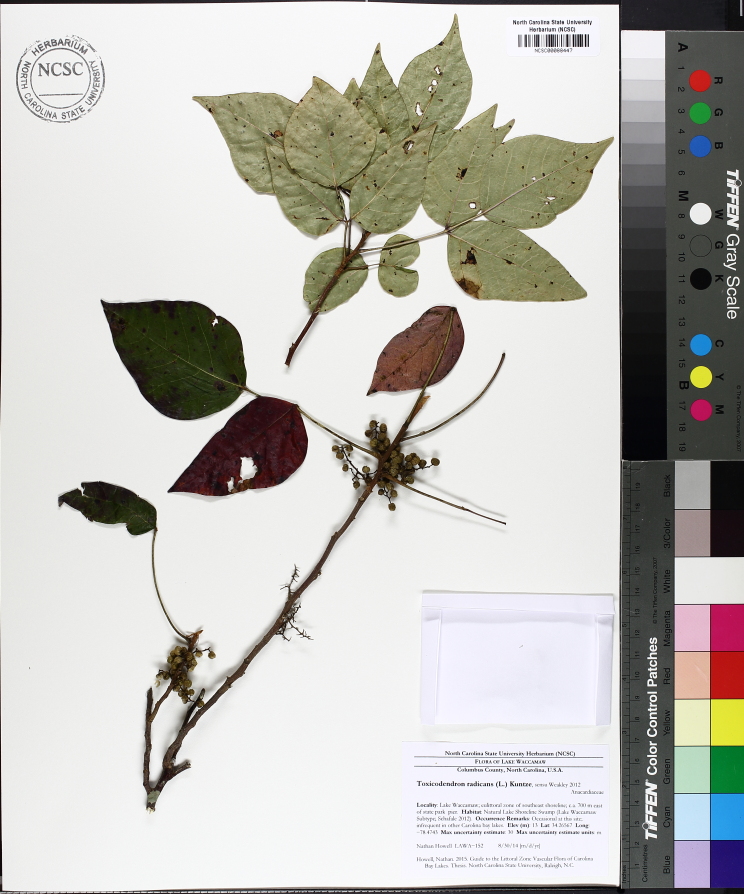
Specimen: *Howell LAWA-152* (NCSC)

**Figure 106b.**
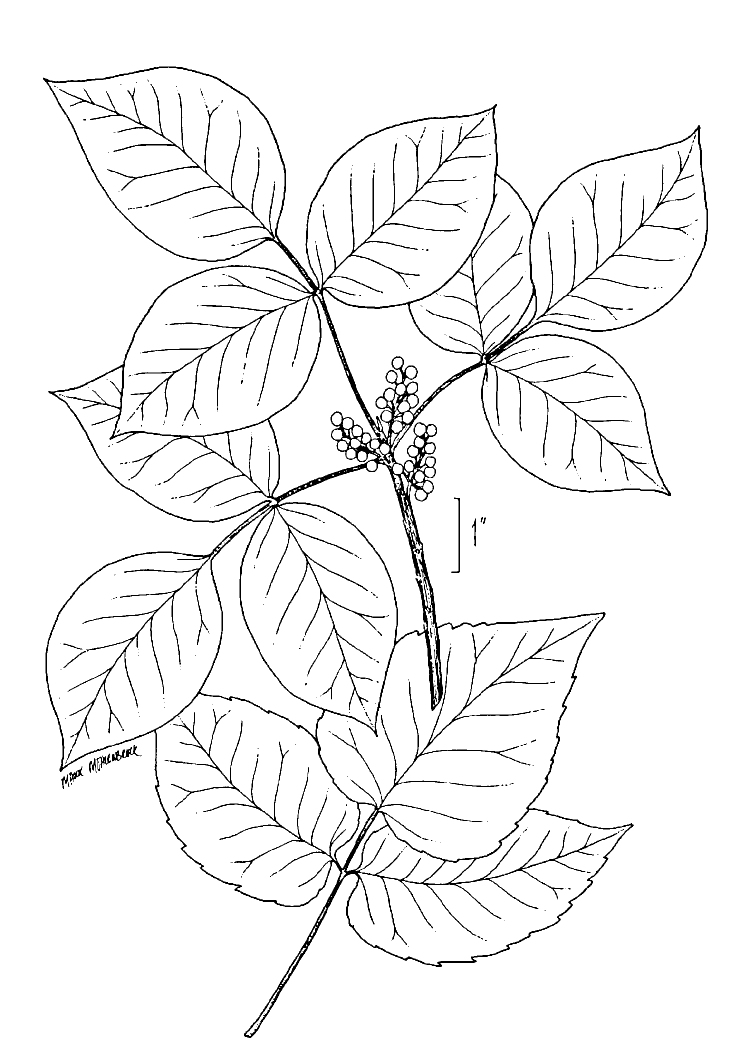
lllustration

**Figure 106c.**
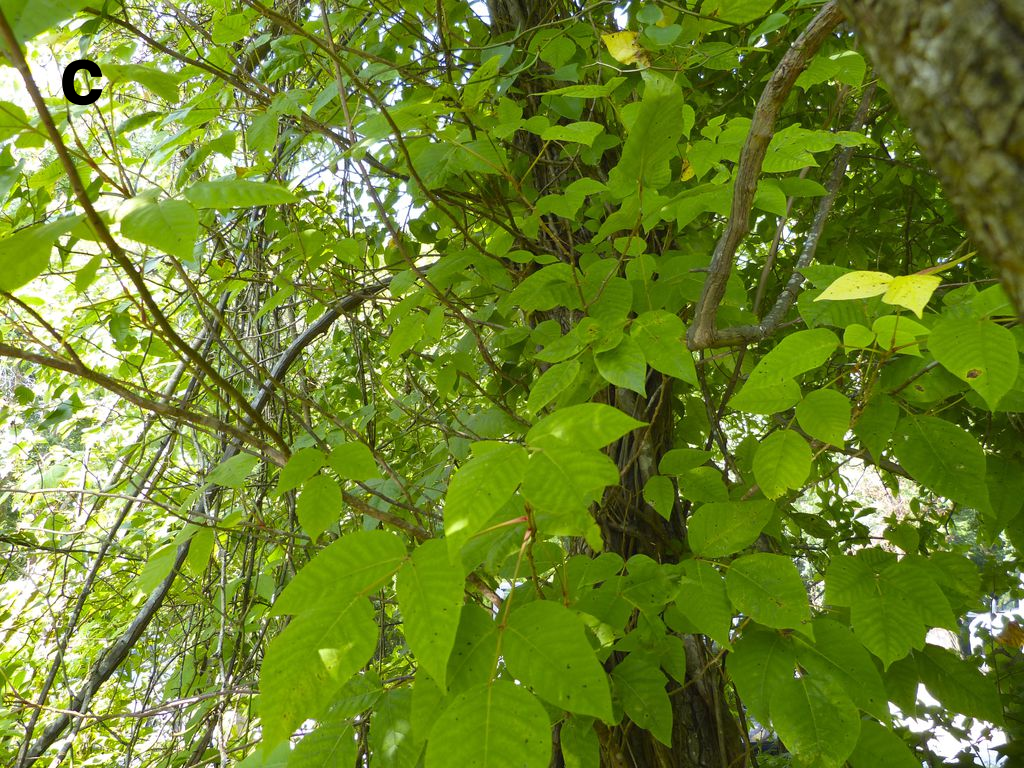
Habit

**Figure 106d.**
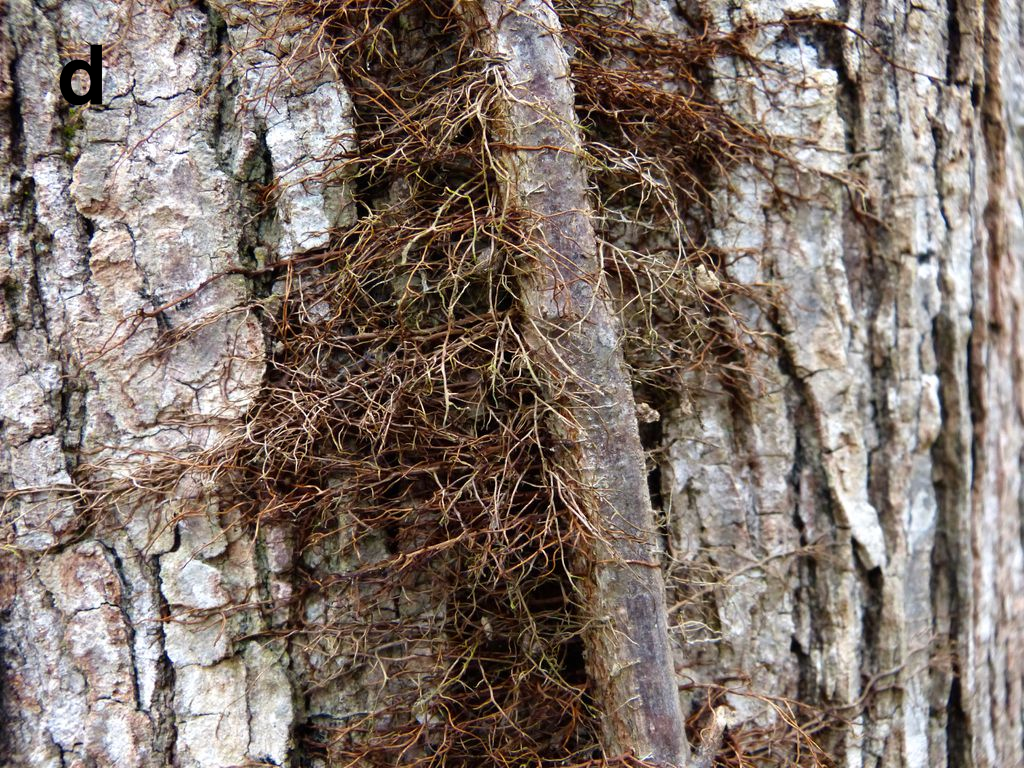
Climbing stem with adventitious roots

**Figure 106e.**
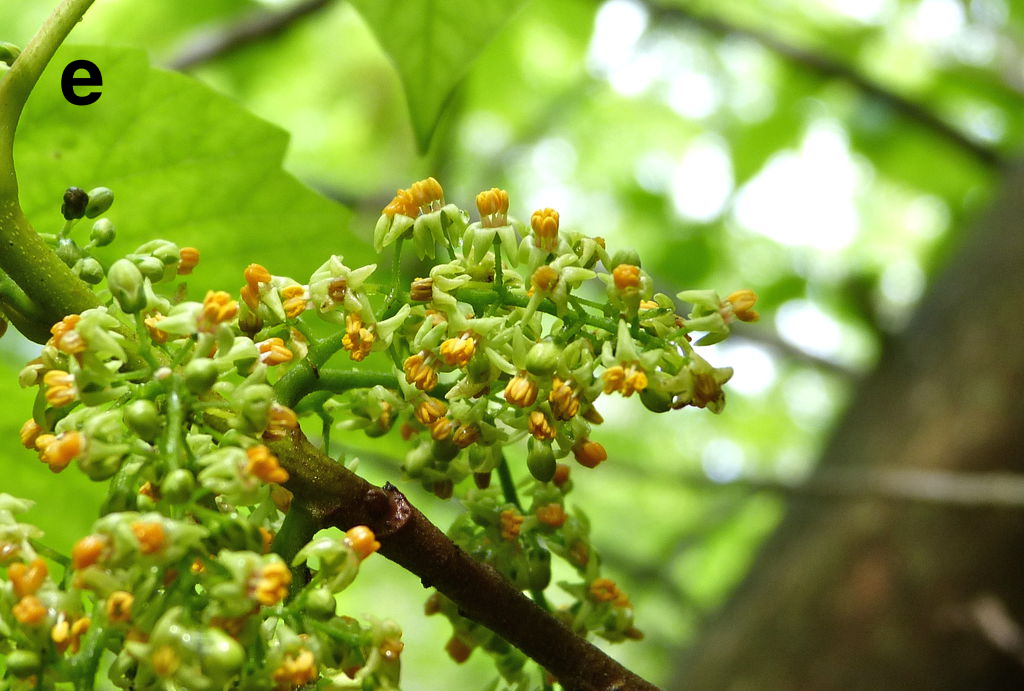
Inflorescence

**Figure 106f.**
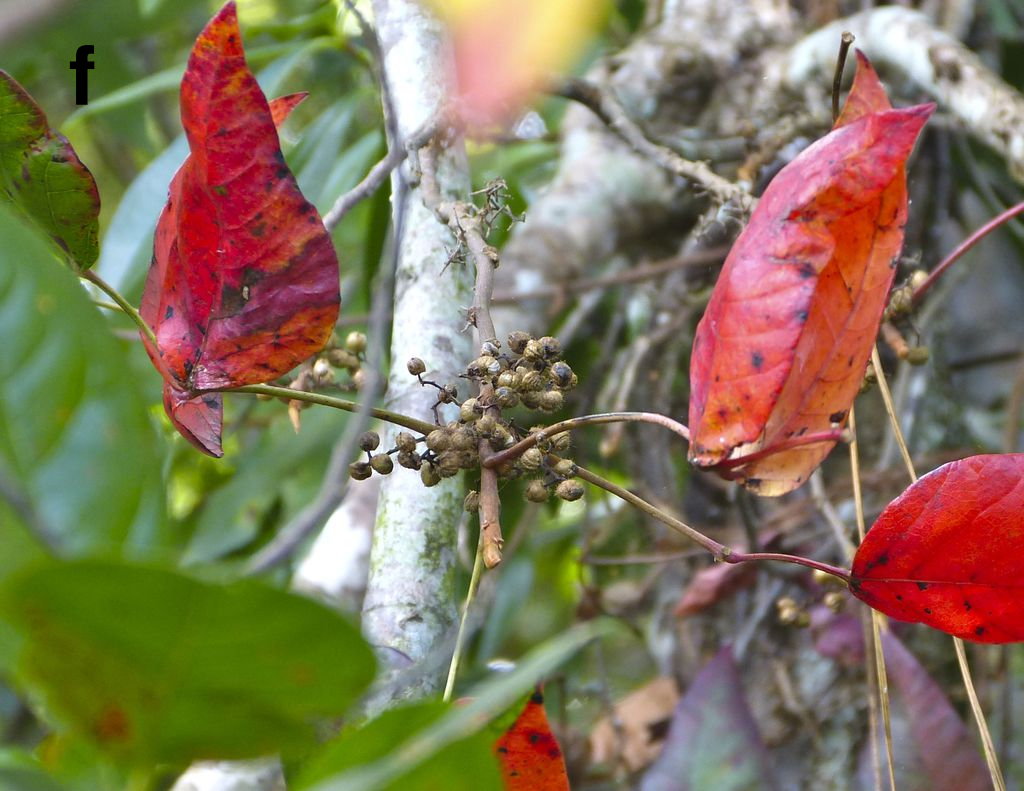
Fruits

**Figure 107a.**
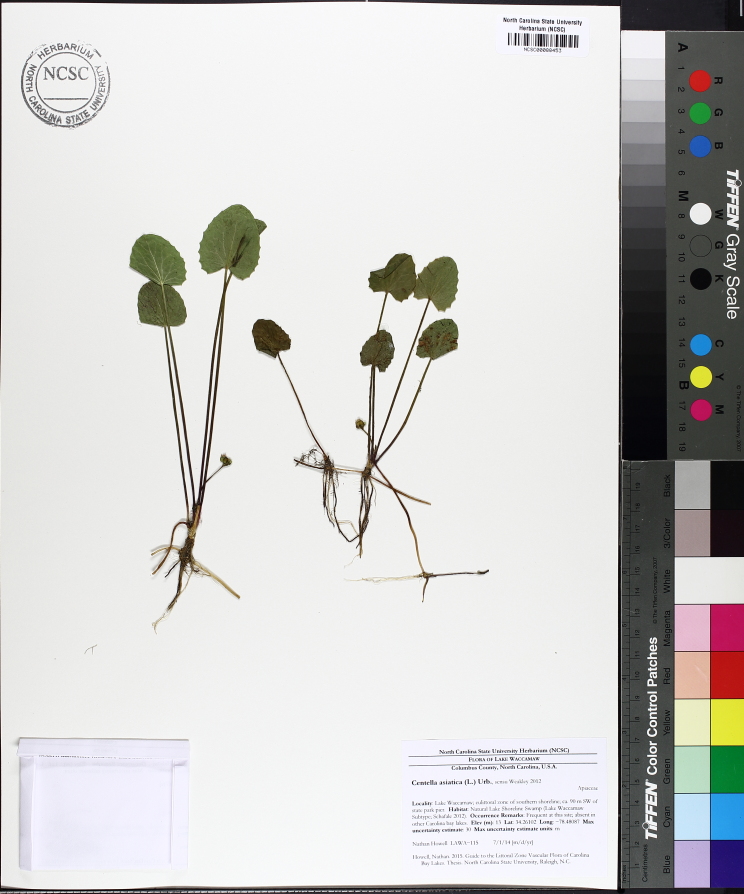
Specimen: *Howell LAWA-115* (NCSC)

**Figure 107b.**
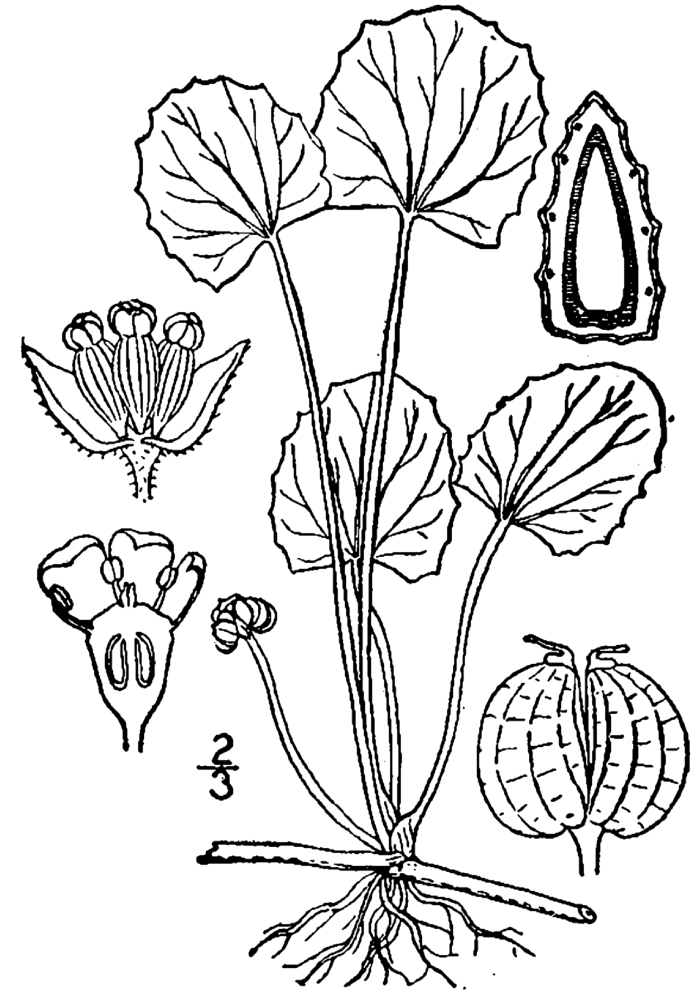
Illustration

**Figure 107c.**
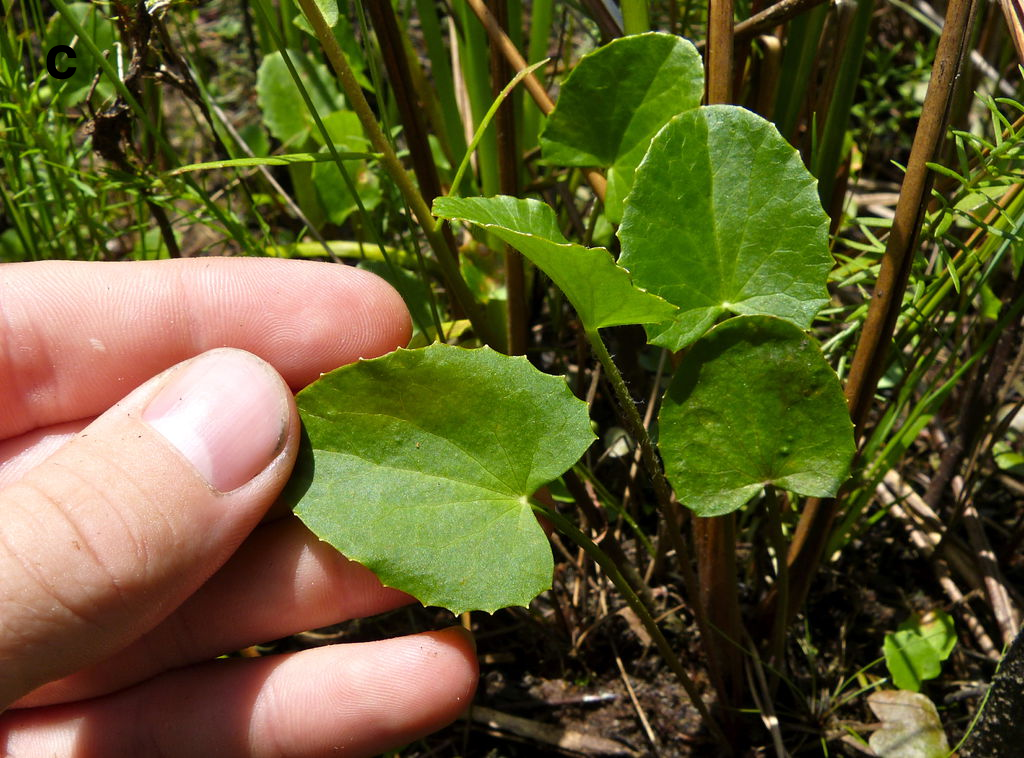
Leaves

**Figure 107d.**
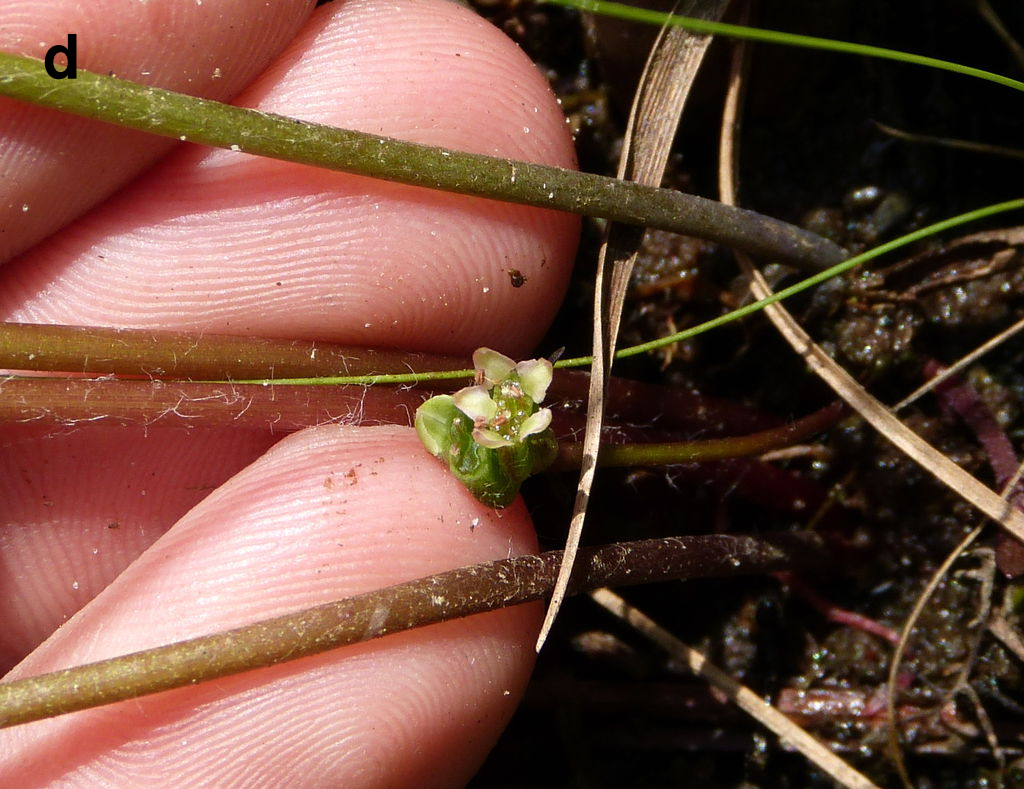
Flower

**Figure 108a.**
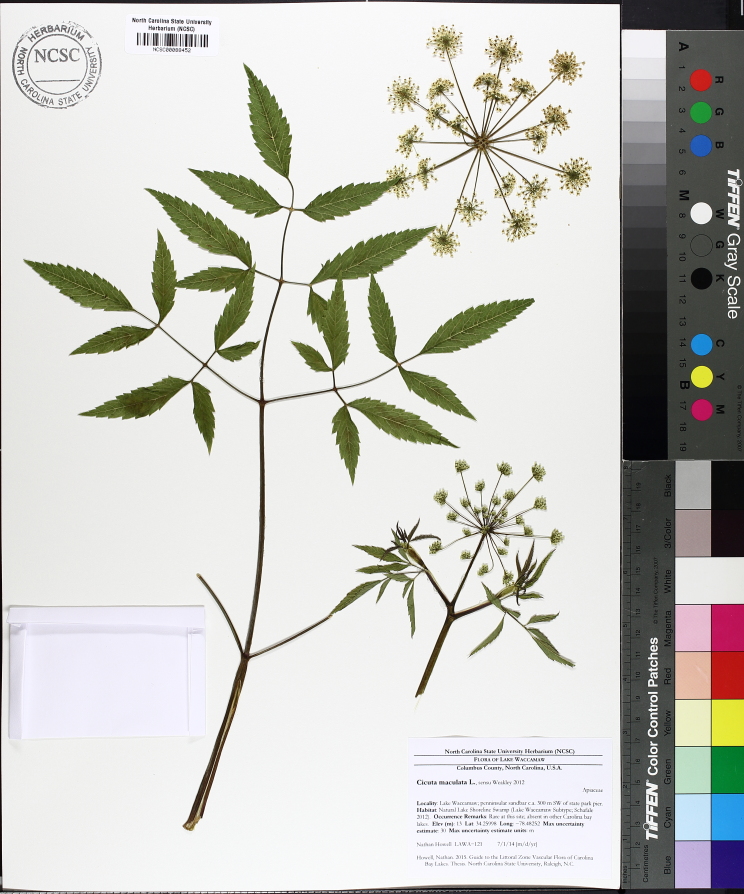
Specimen: *Howell LAWA-121* (NCSC)

**Figure 108b.**
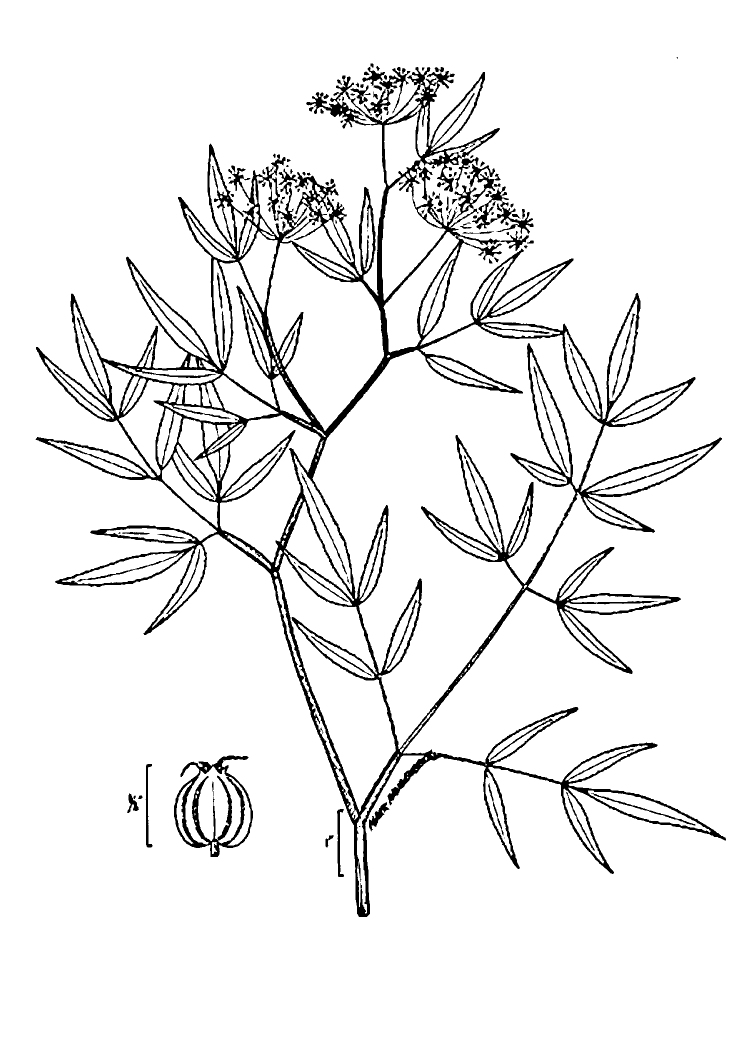
Illustration

**Figure 108c.**
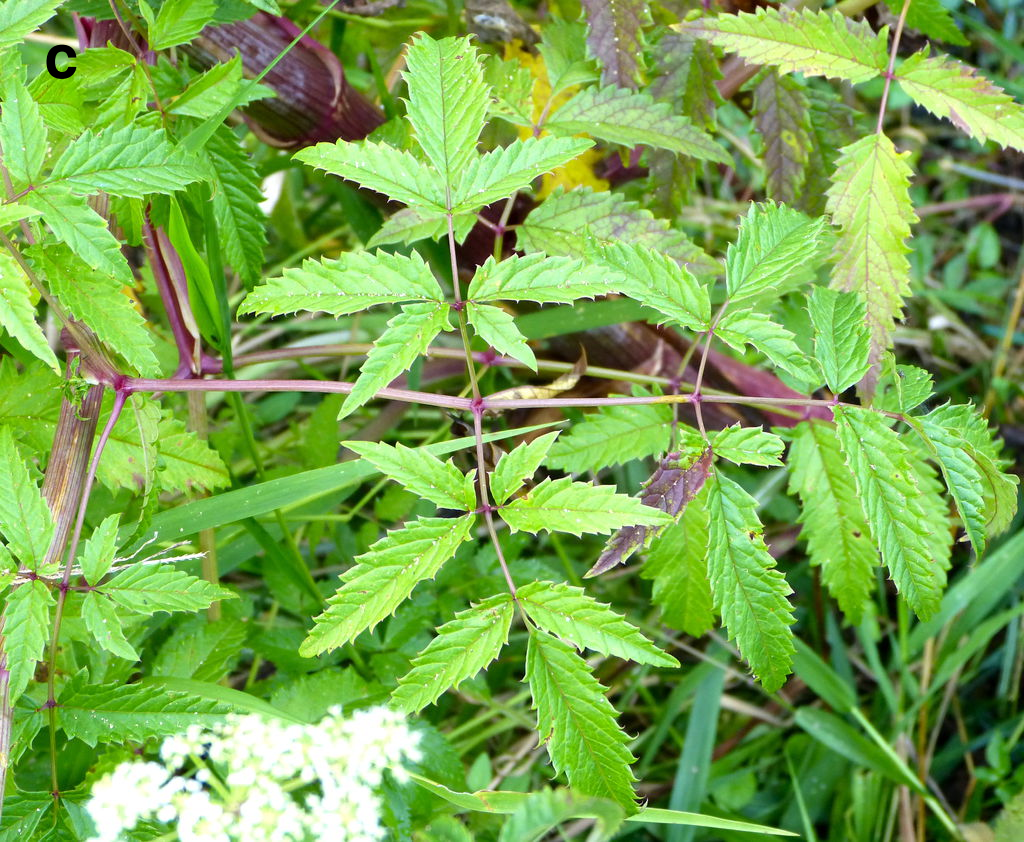
Leaf

**Figure 108d.**
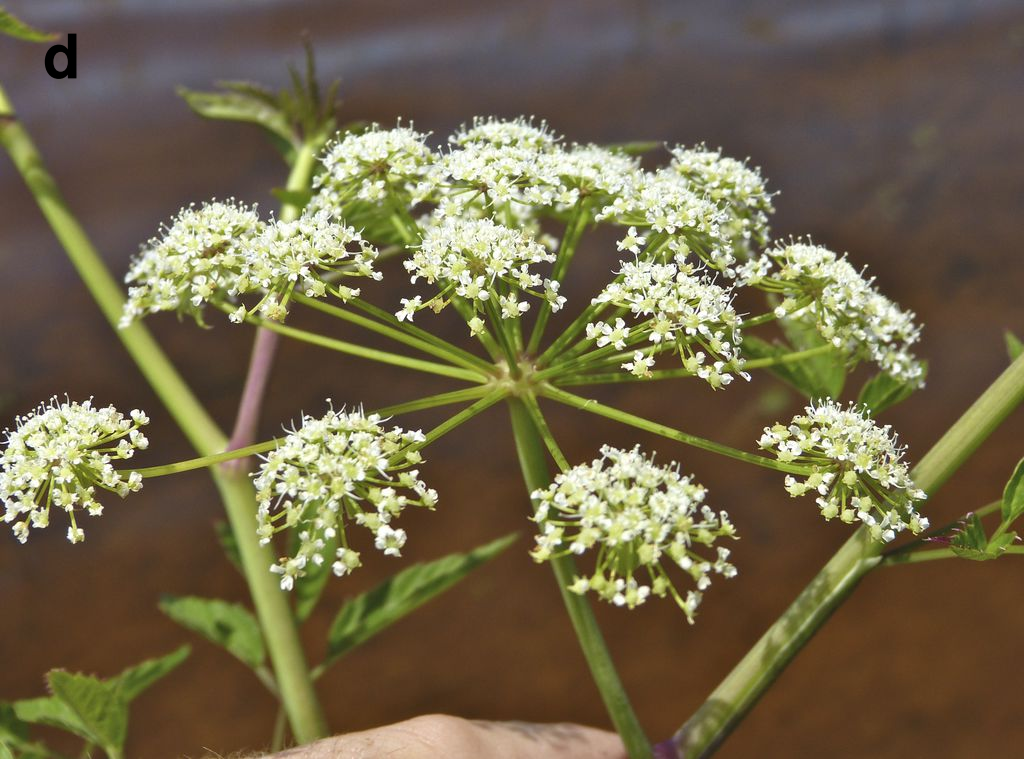
Inflorescence

**Figure 109a.**
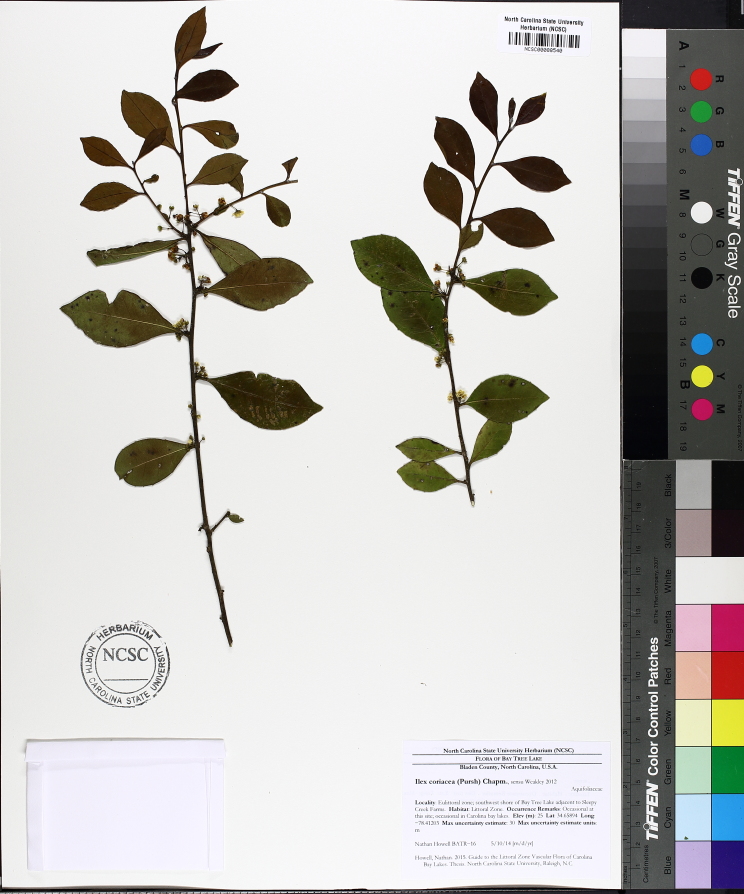
Specimen: *Howell BATR-16* (NCSC)

**Figure 109b.**
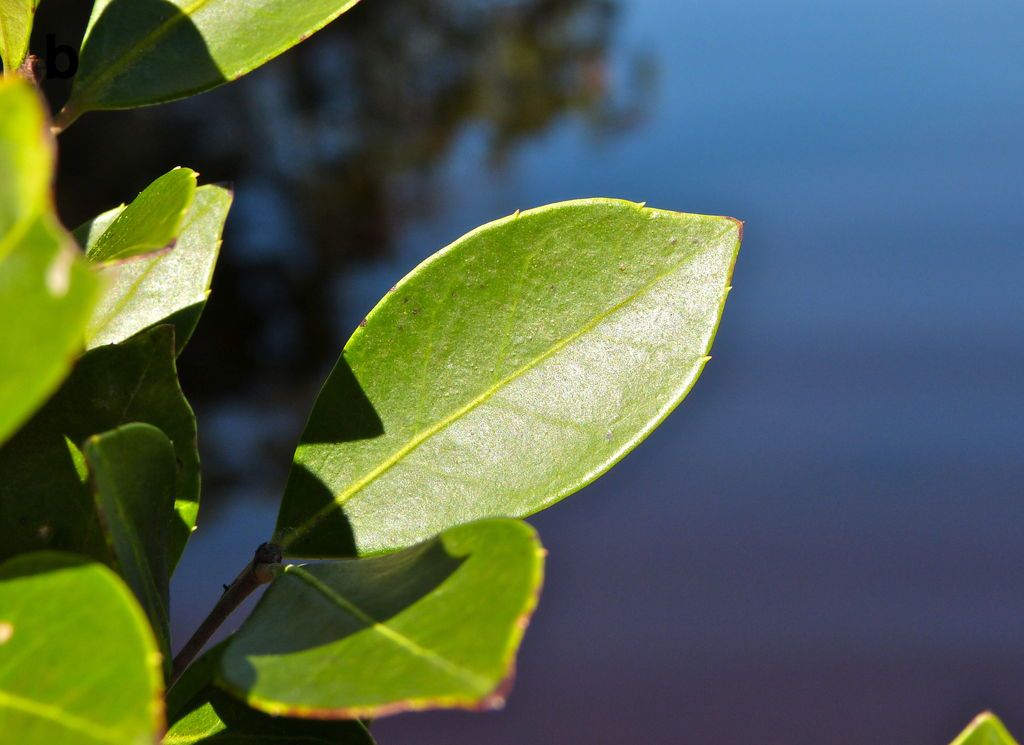
Leaves

**Figure 109c.**
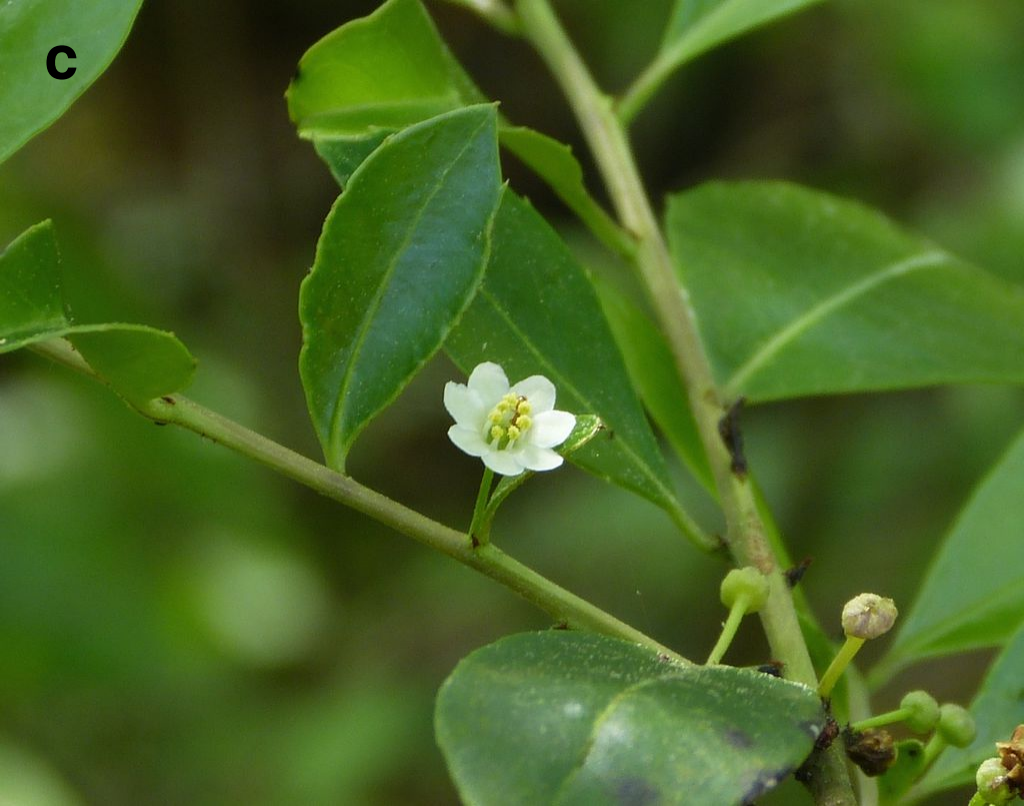
Flower

**Figure 109d.**
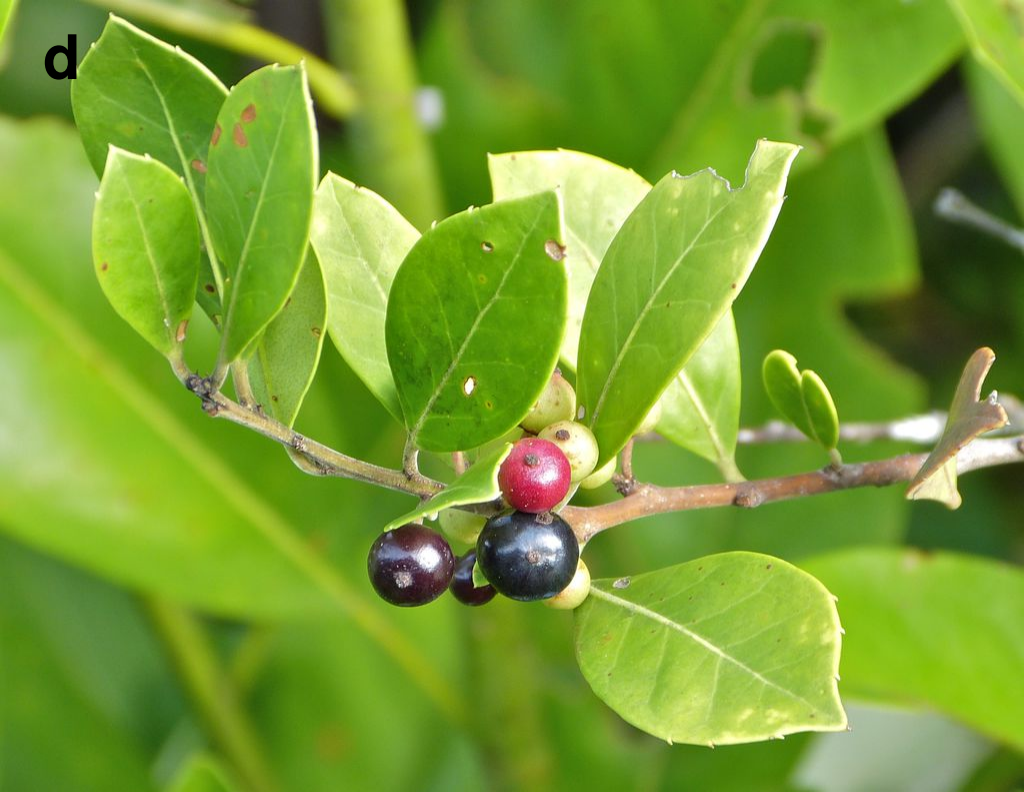
Fruits

**Figure 110a.**
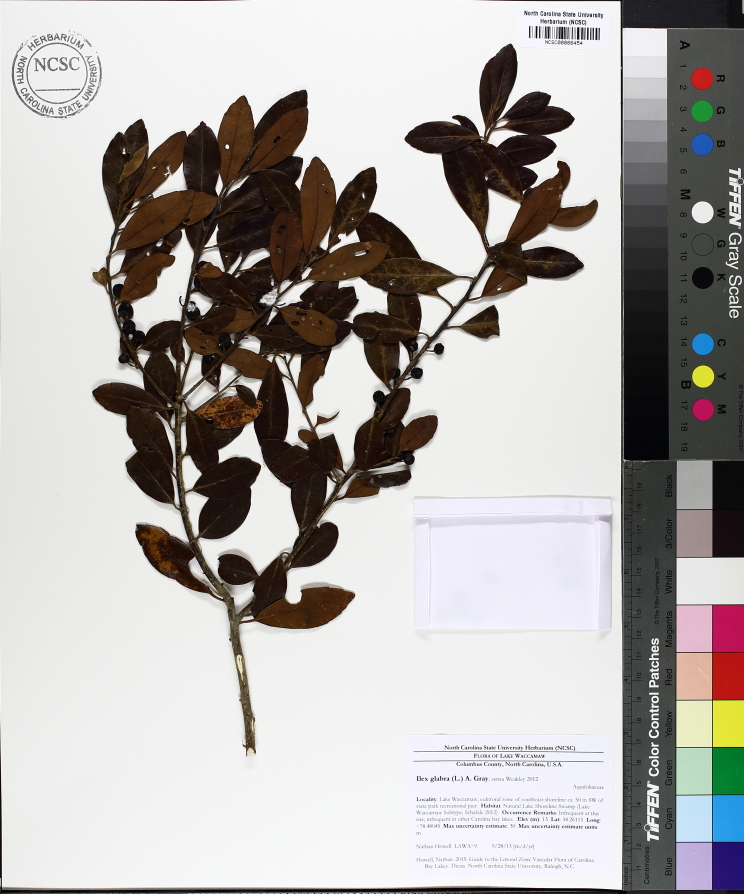
Specimen: *Howell LAWA-9* (NCSC)

**Figure 110b.**
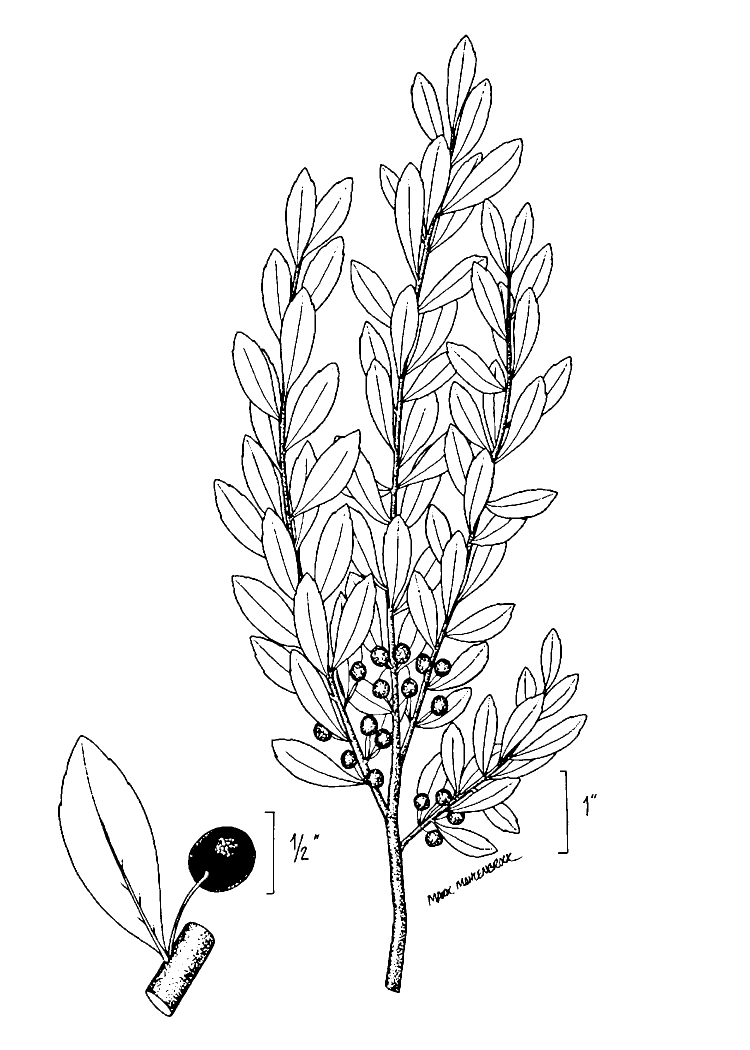
Illustration

**Figure 110c.**
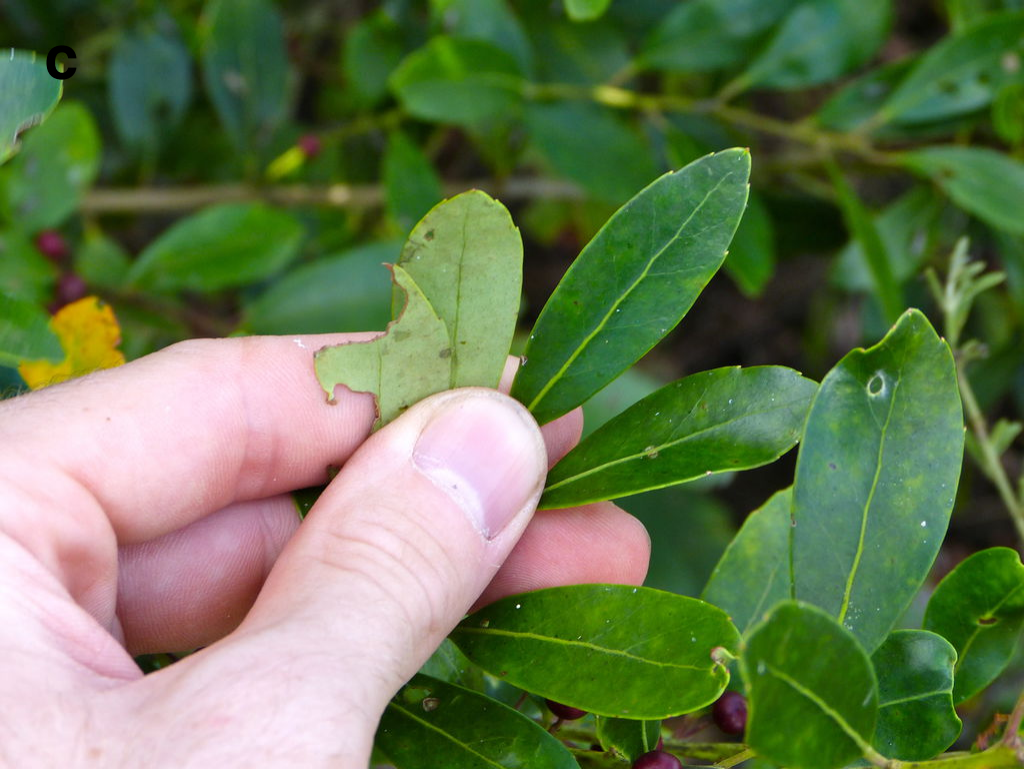
Leaves

**Figure 110d.**
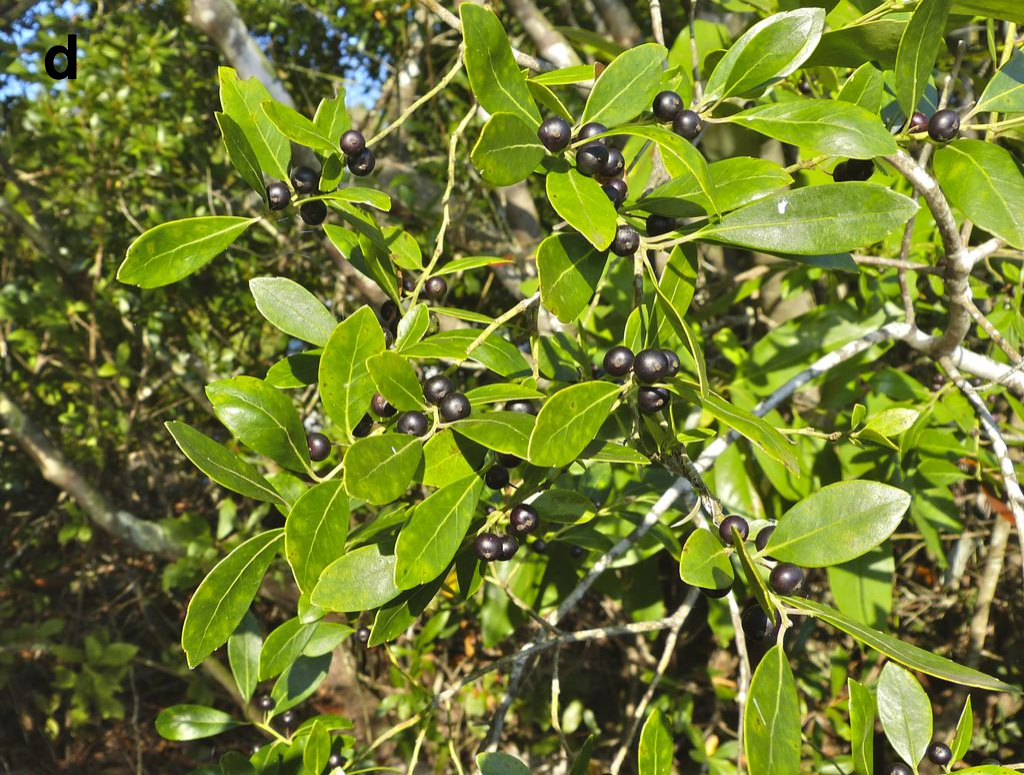
Fruits

**Figure 111a.**
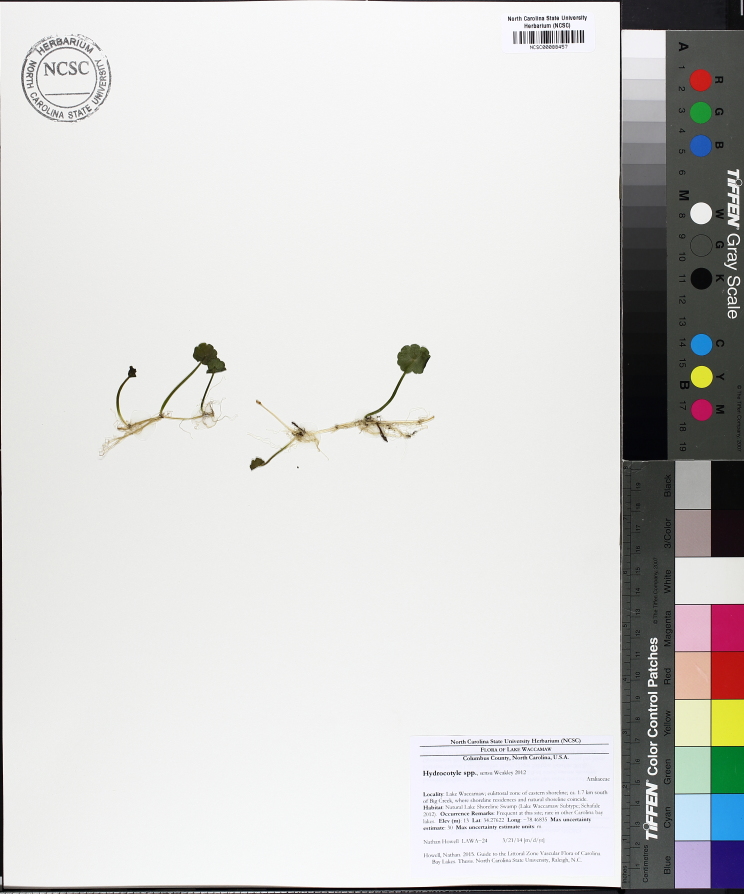
Specimen: *Howell LAWA-24* (NCSC)

**Figure 111b.**
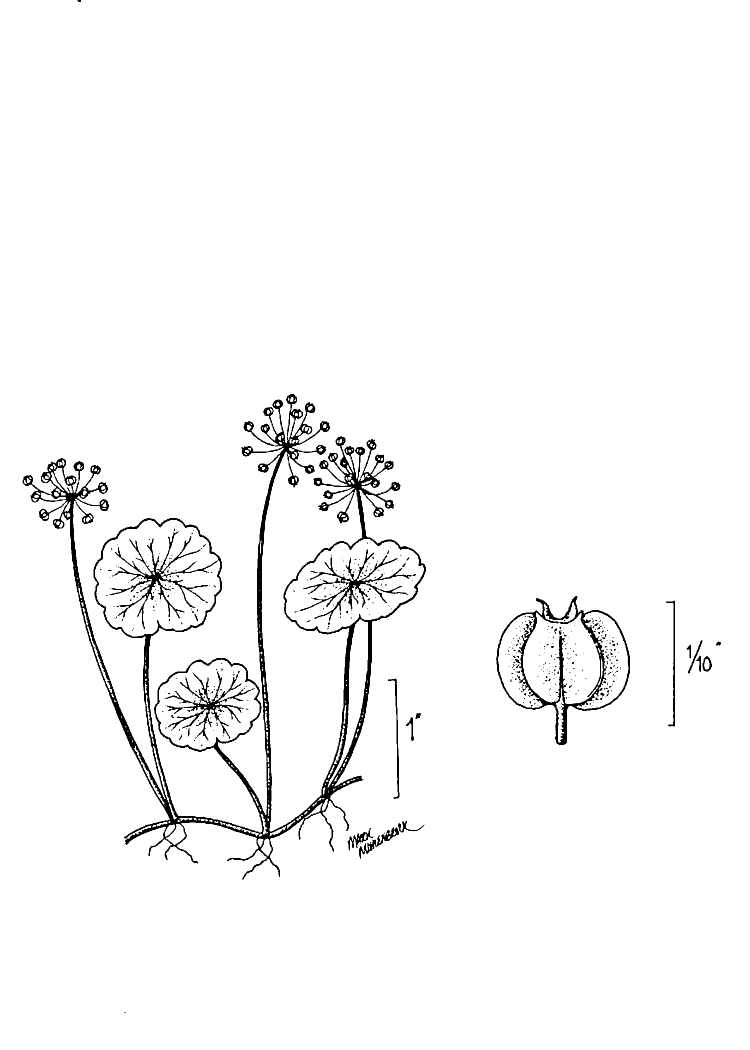
Illustration

**Figure 112a.**
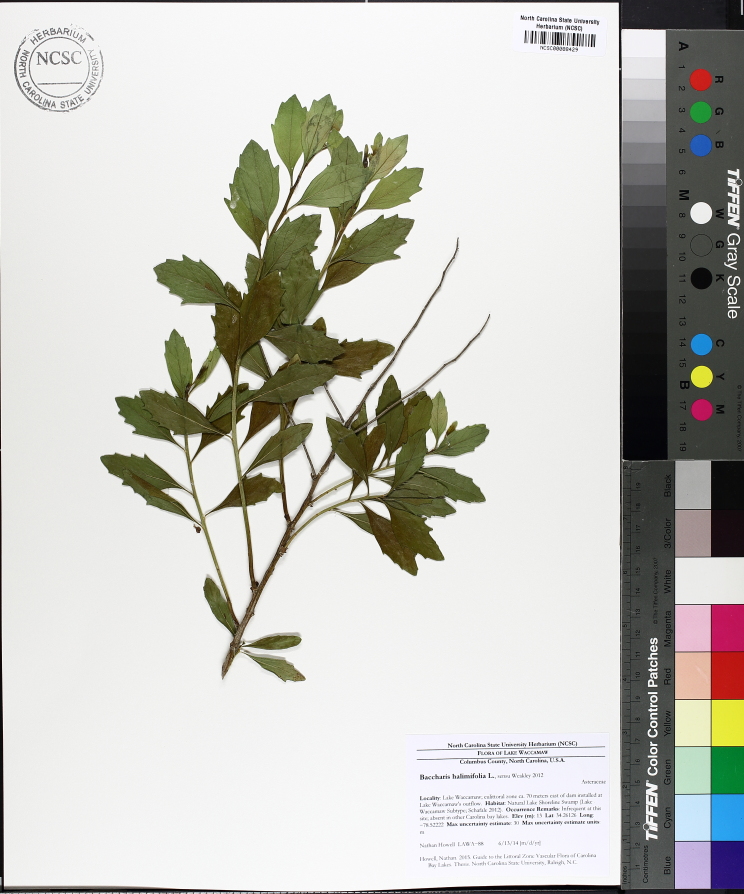
Specimen: *Howell LAWA-88* (NCSC)

**Figure 112b.**
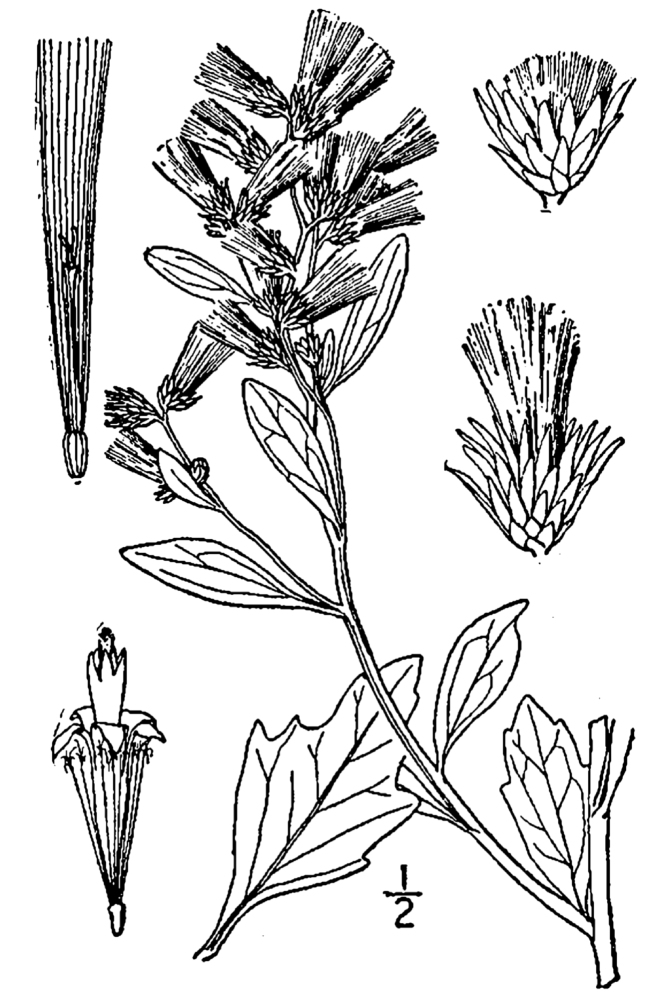
Illustration

**Figure 112c.**
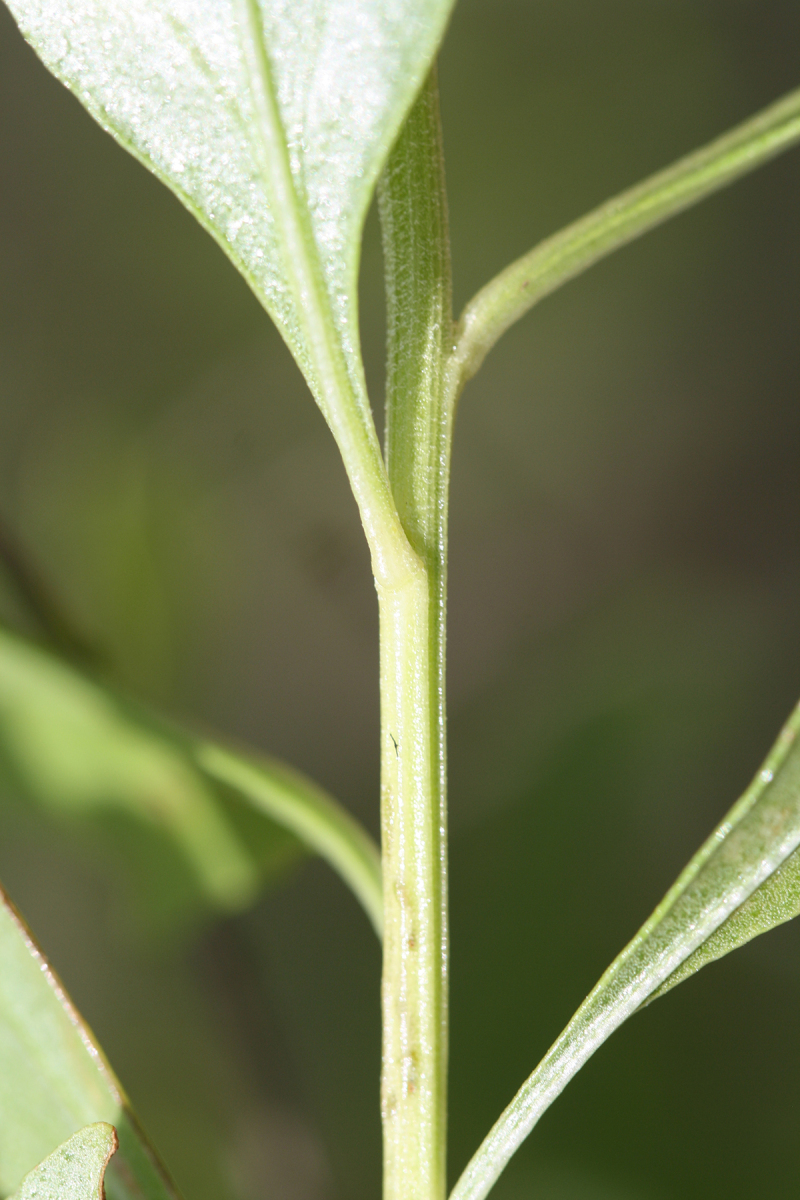
Stem

**Figure 112d.**
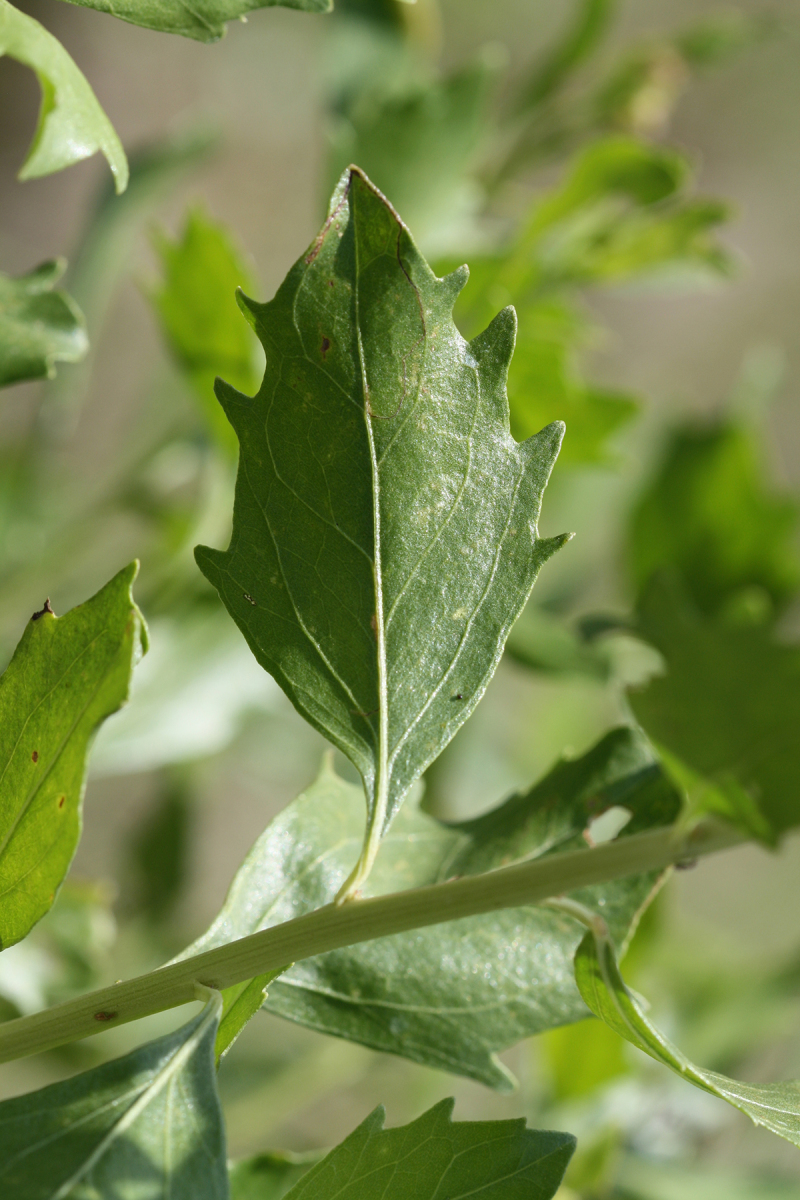
Leaf

**Figure 112e.**
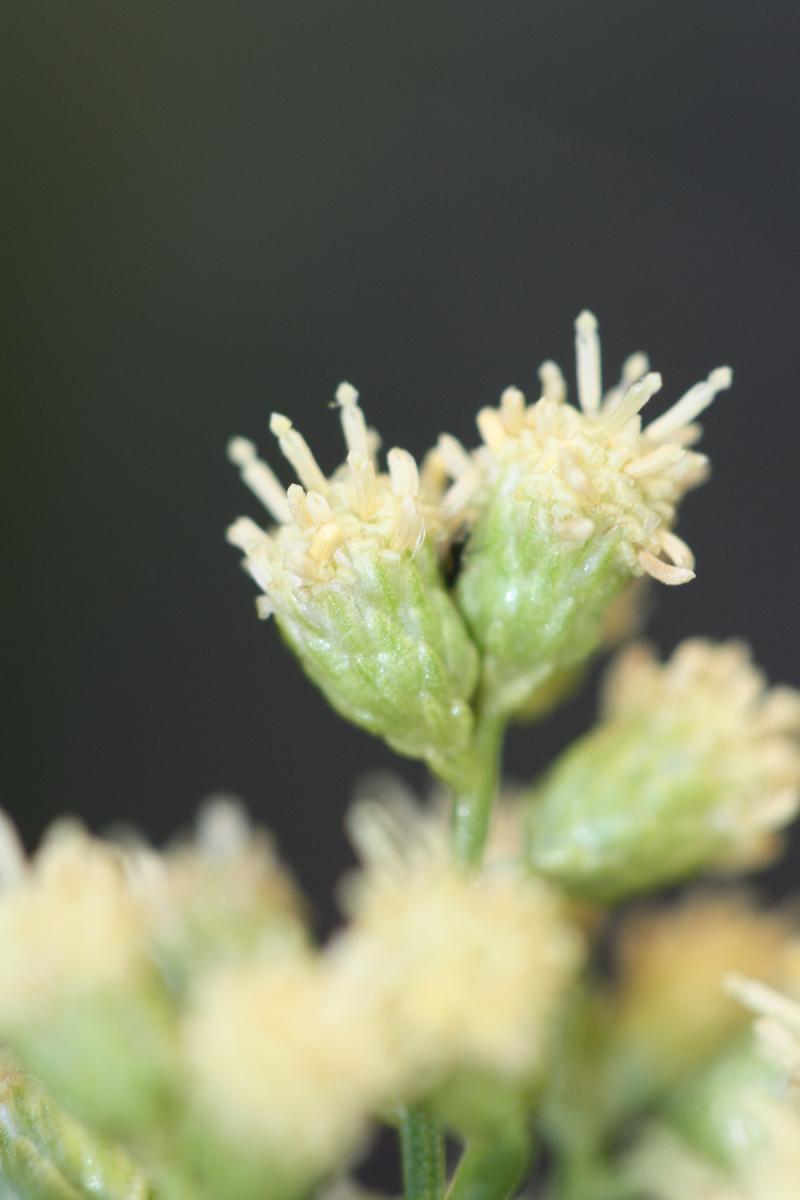
Staminate capitulescence

**Figure 112f.**
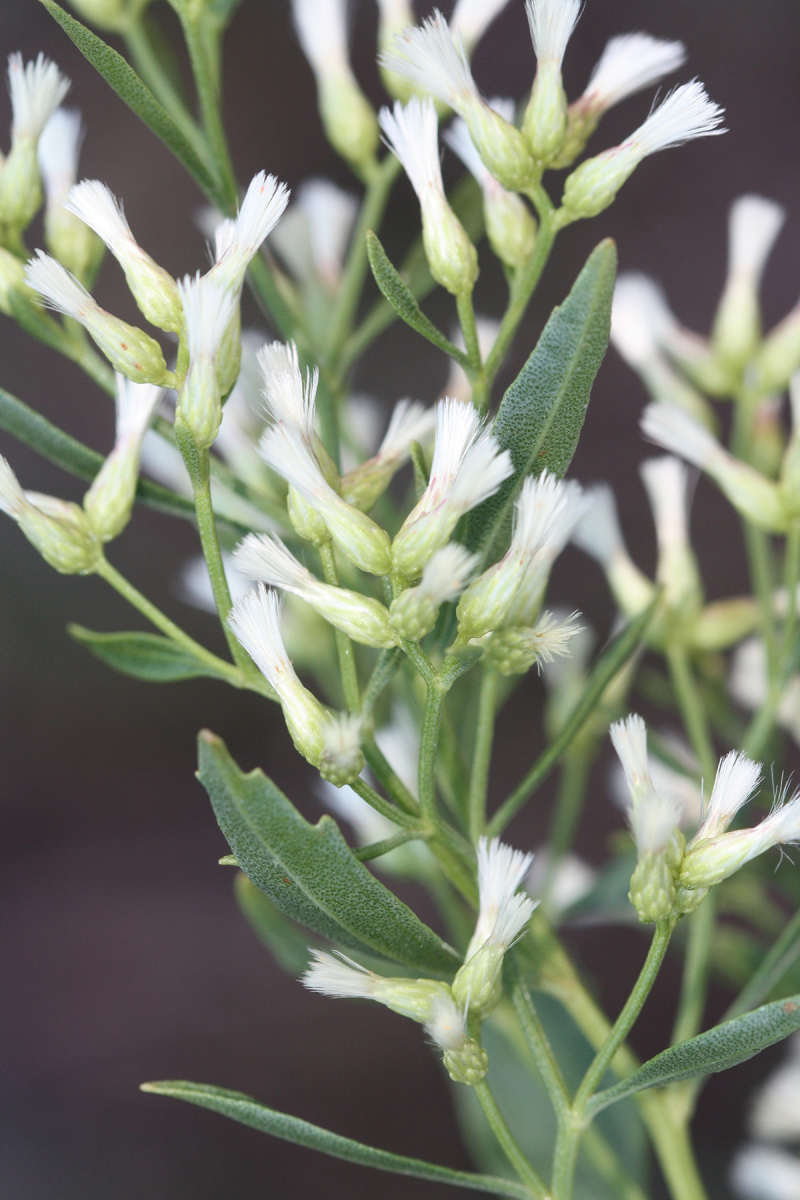
Pistillate capitulescence

**Figure 114a.**
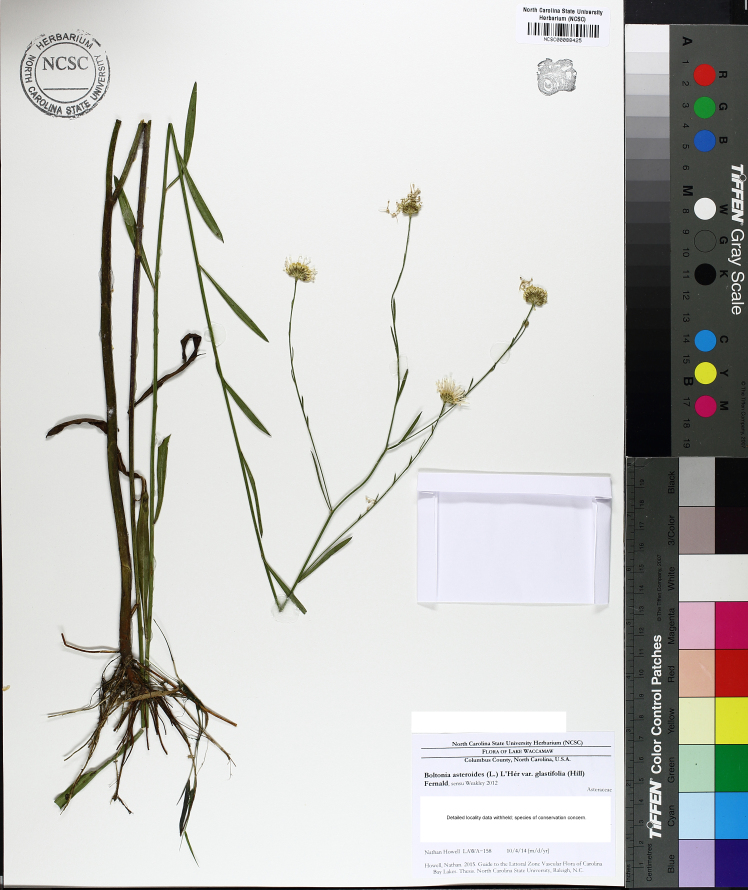
Specimen: *Howell LAWA-158* (NCSC)

**Figure 114b.**
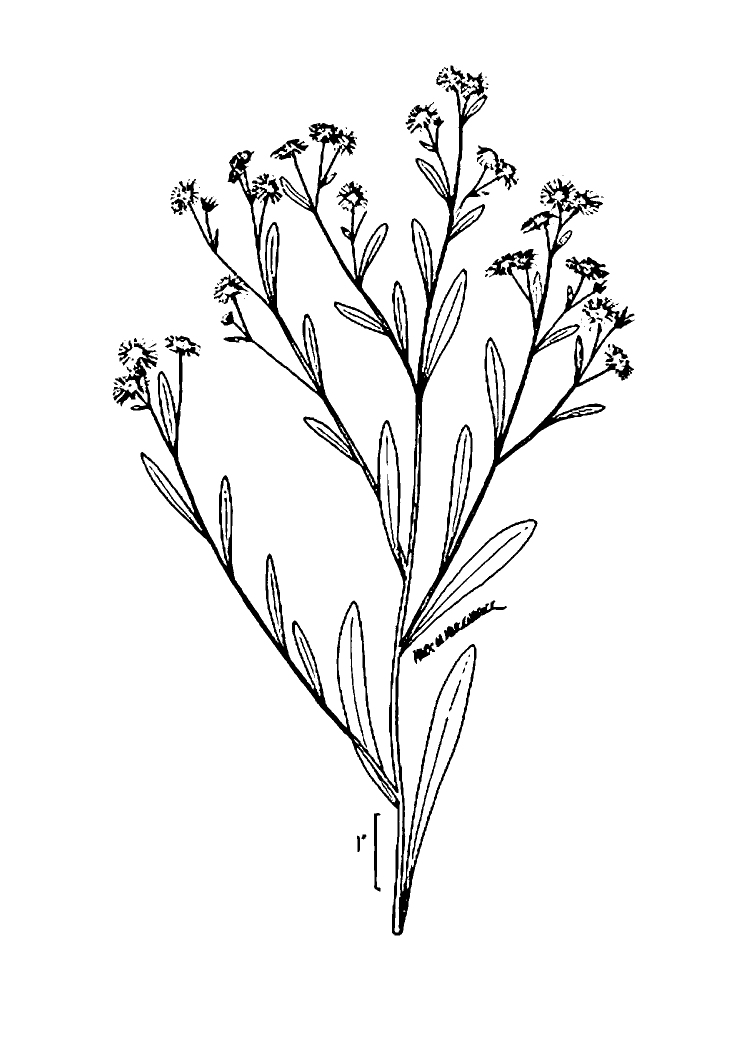
Illustration

**Figure 114c.**
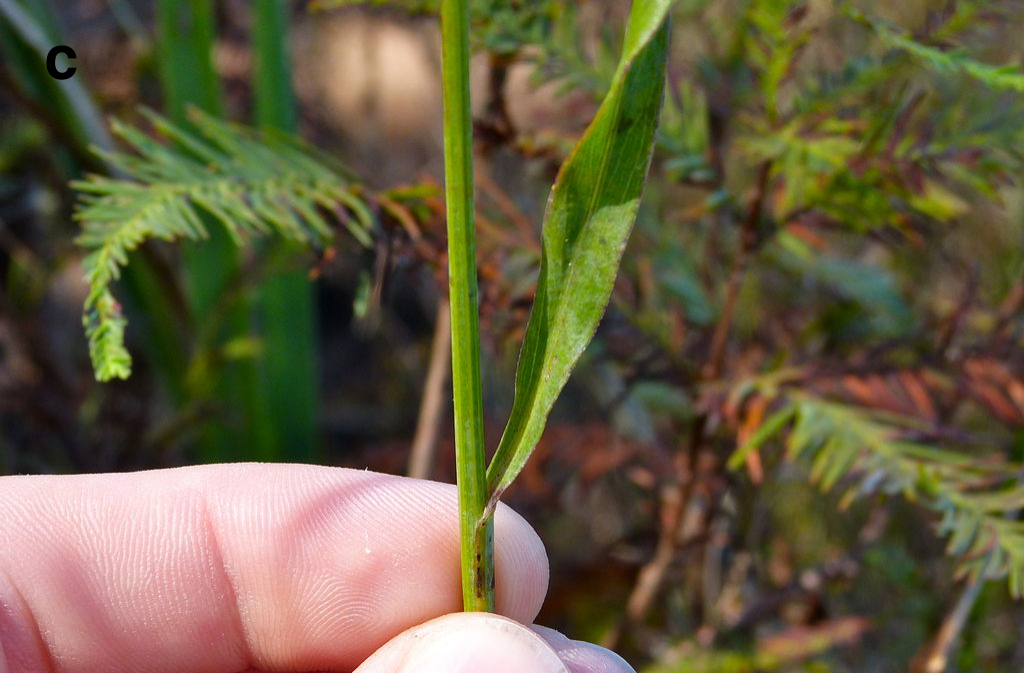
Stem and leaf

**Figure 114d.**
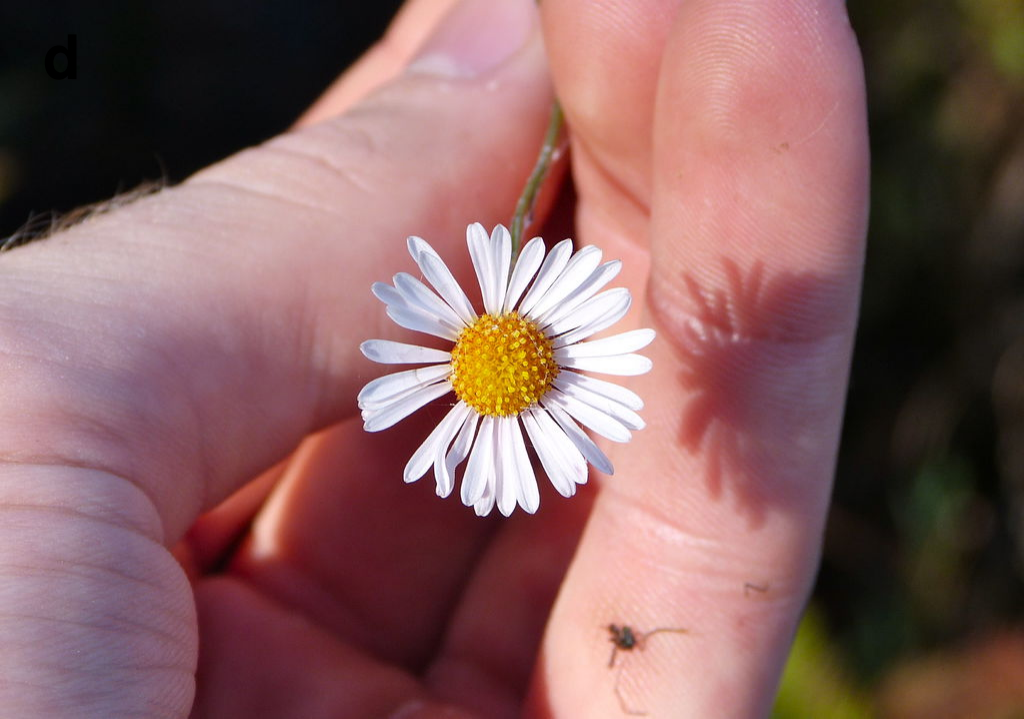
Radiate head

**Figure 116a.**
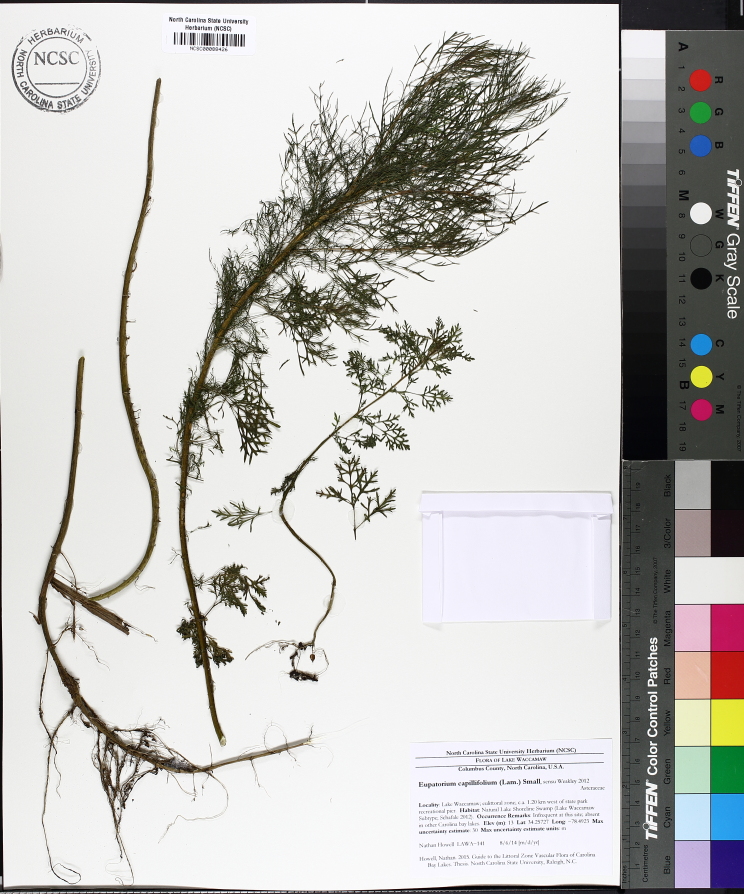
Specimen: *Howell LAWA-141* (NCSC)

**Figure 116b.**
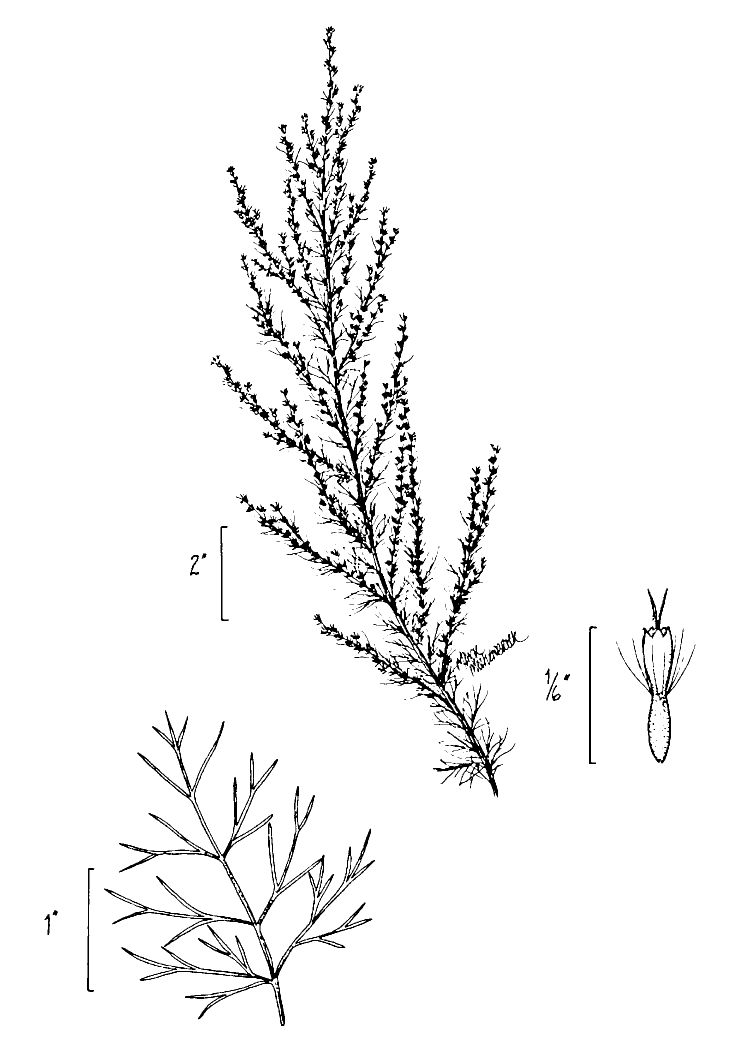
Illustration

**Figure 116c.**
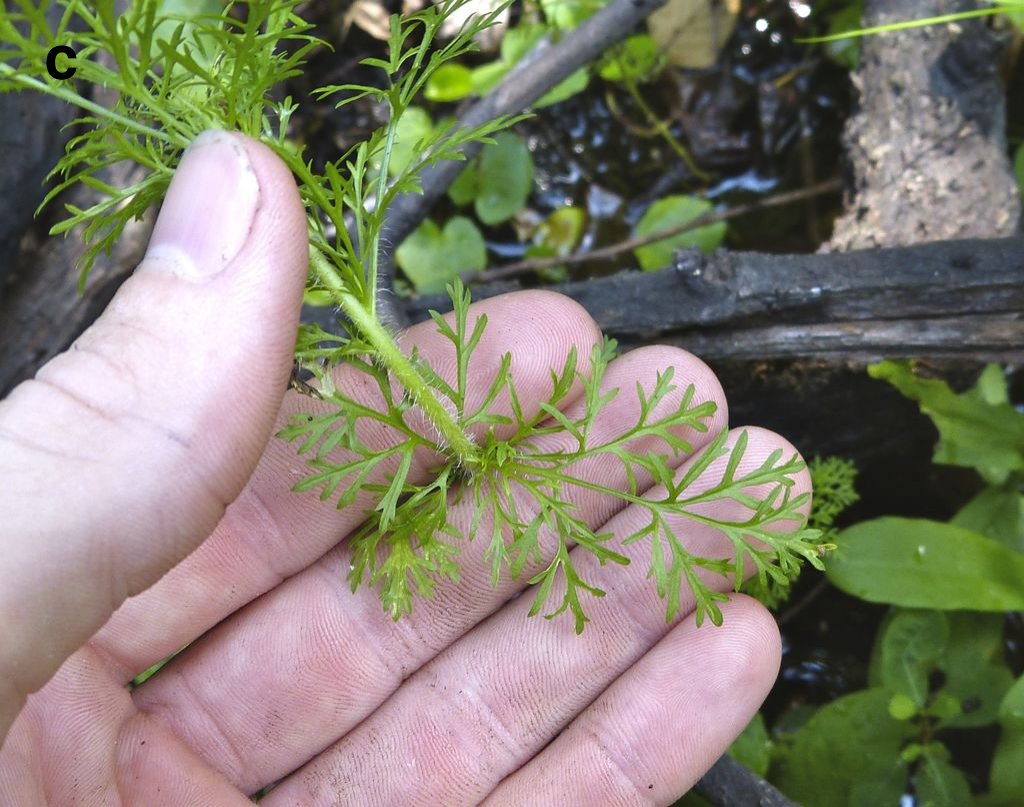
Leaf

**Figure 116d.**
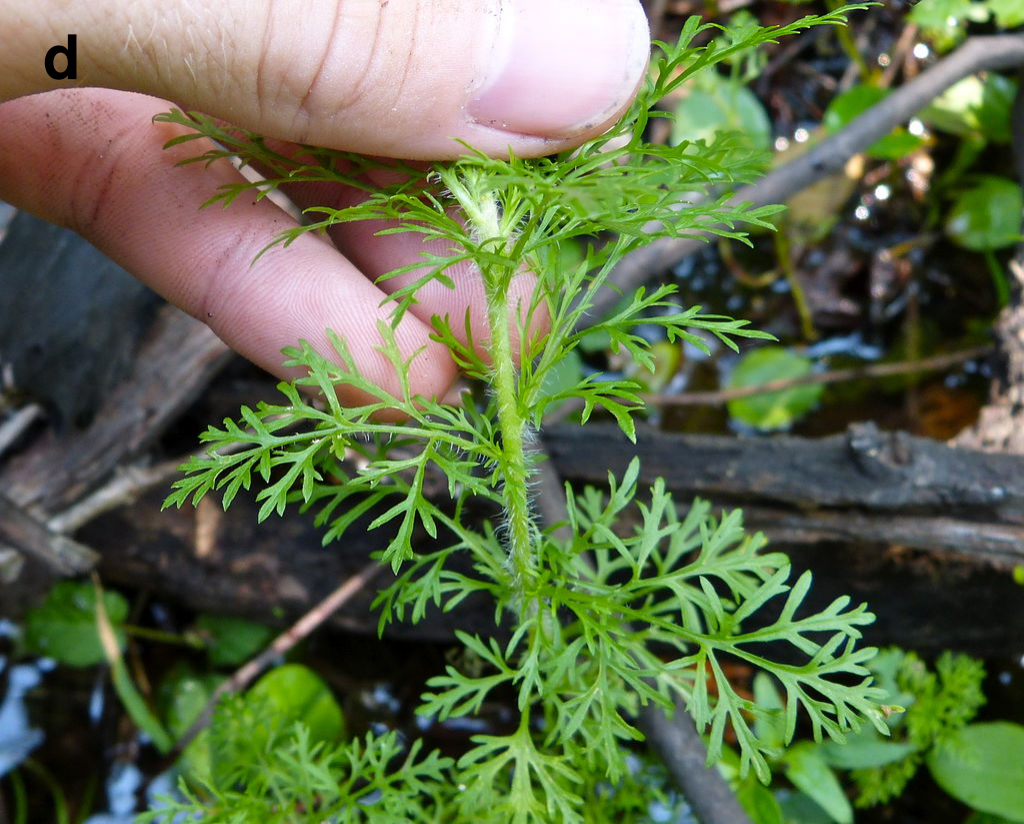
Leaf

**Figure 117a.**
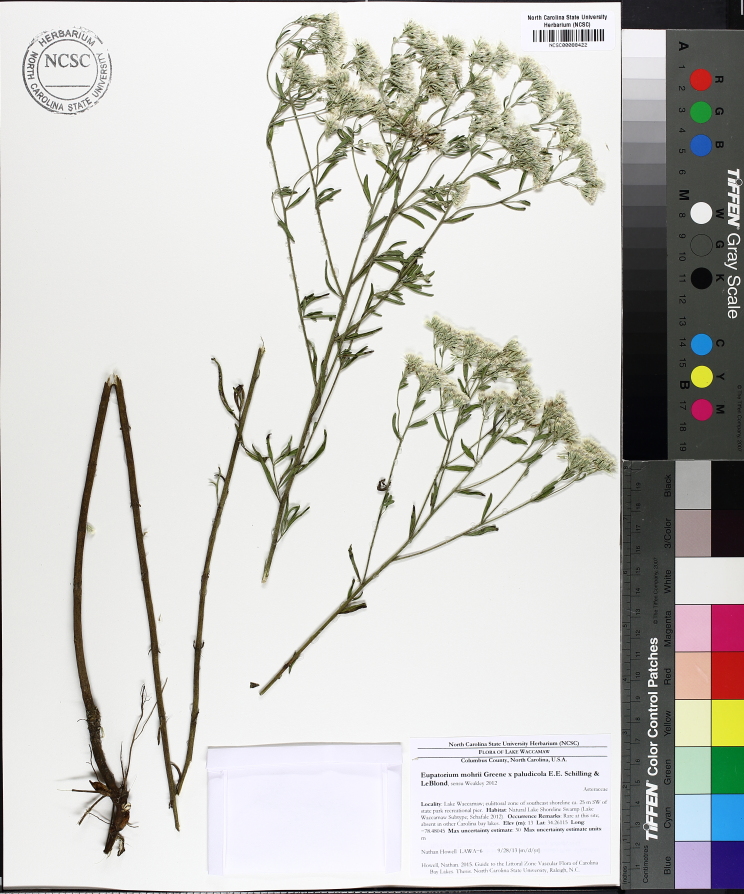
Specimen: *Howell LAWA-6* (NCSC)

**Figure 117b.**
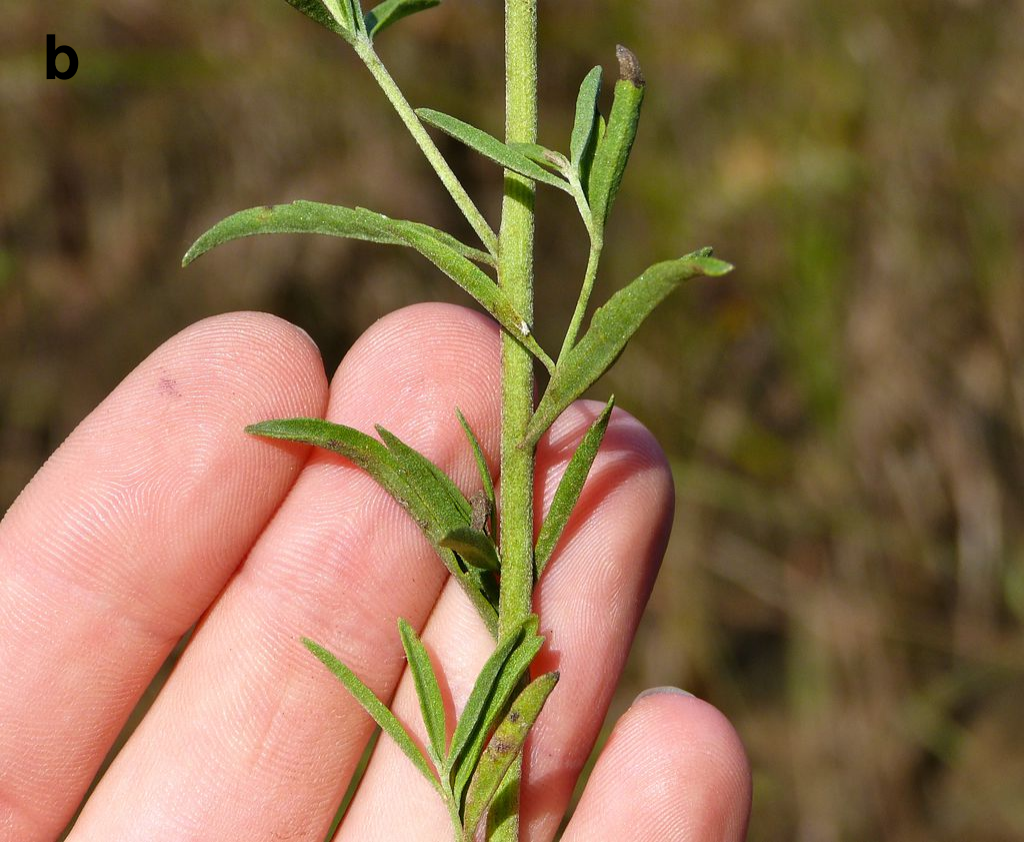
Stem and leaves

**Figure 117c.**
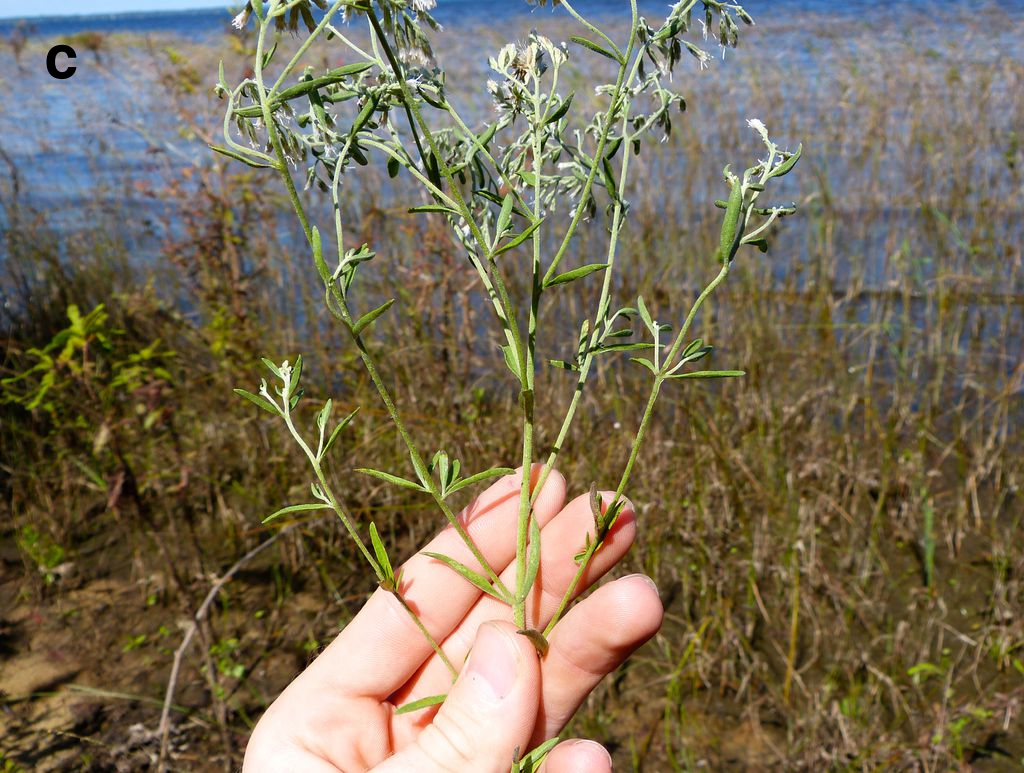
Capitulescence

**Figure 117d.**
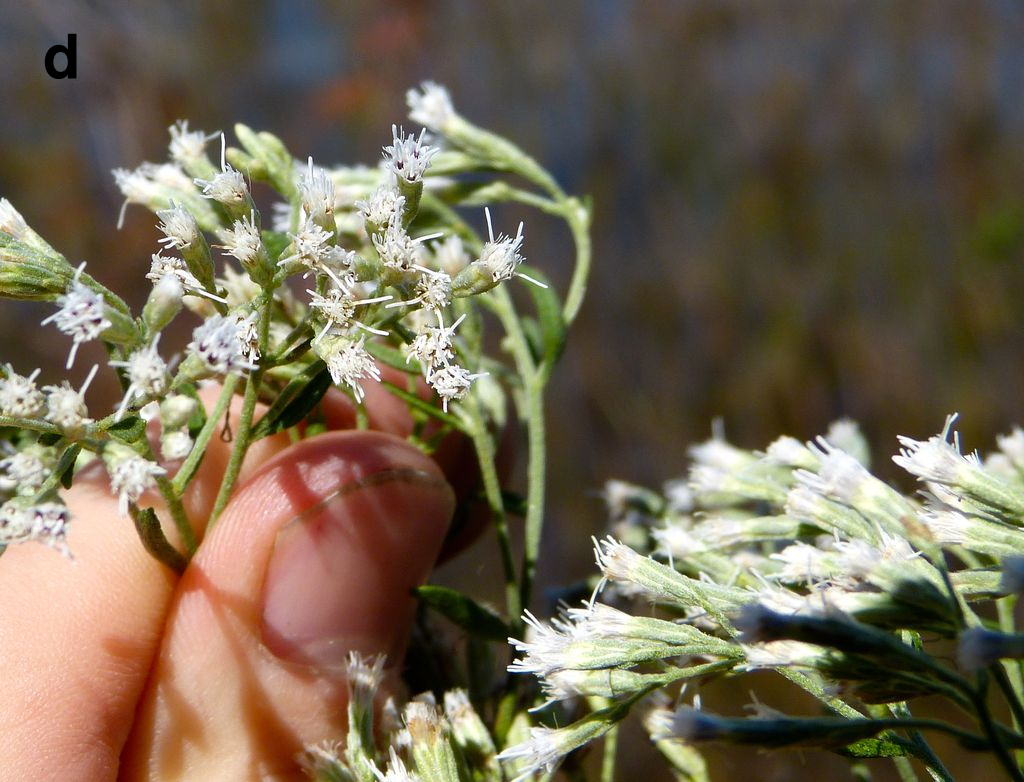
Capitulescence (detail)

**Figure 118a.**
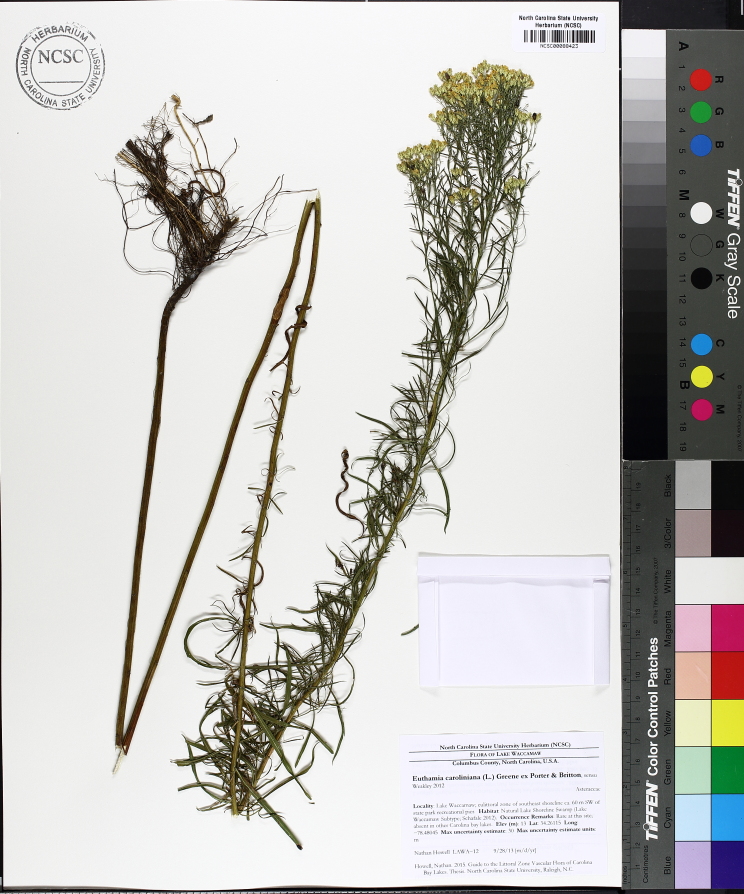
Specimen: *Howell LAWA-12* (NCSC)

**Figure 118b.**
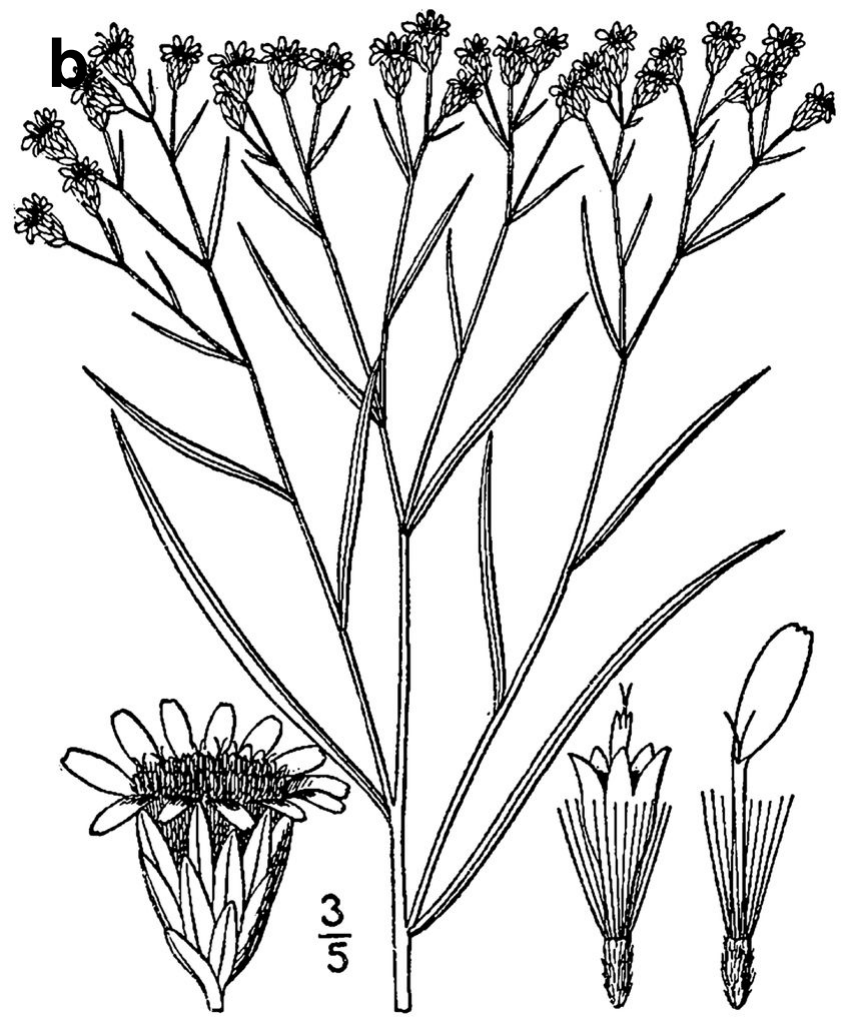
Illustration

**Figure 118c.**
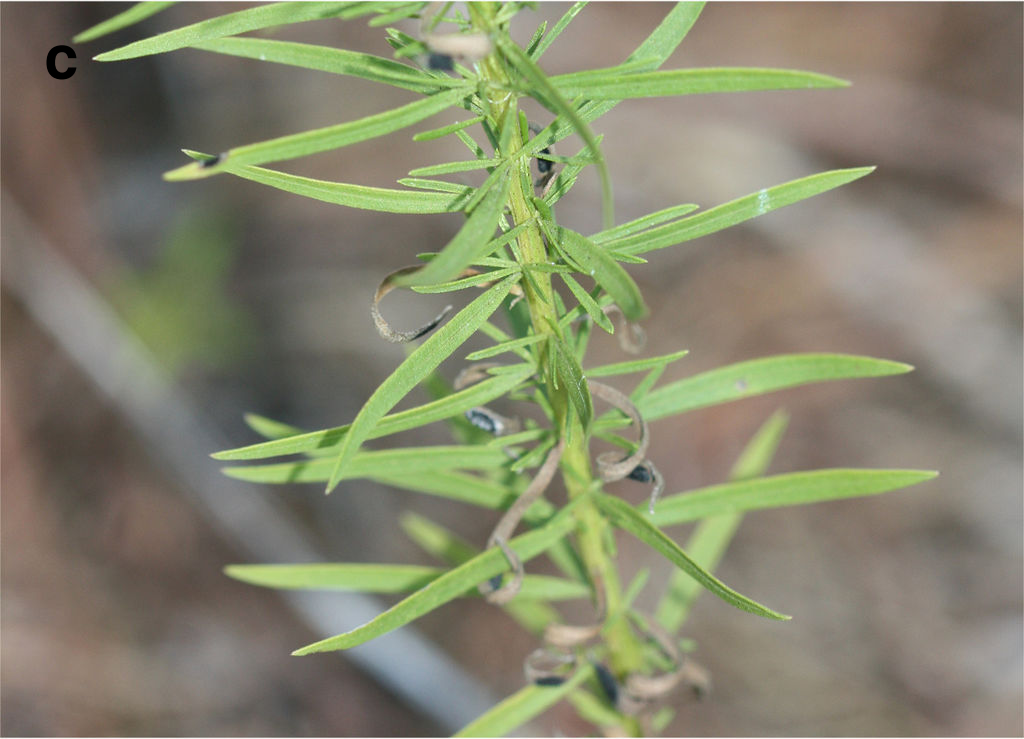
Leaves

**Figure 118d.**
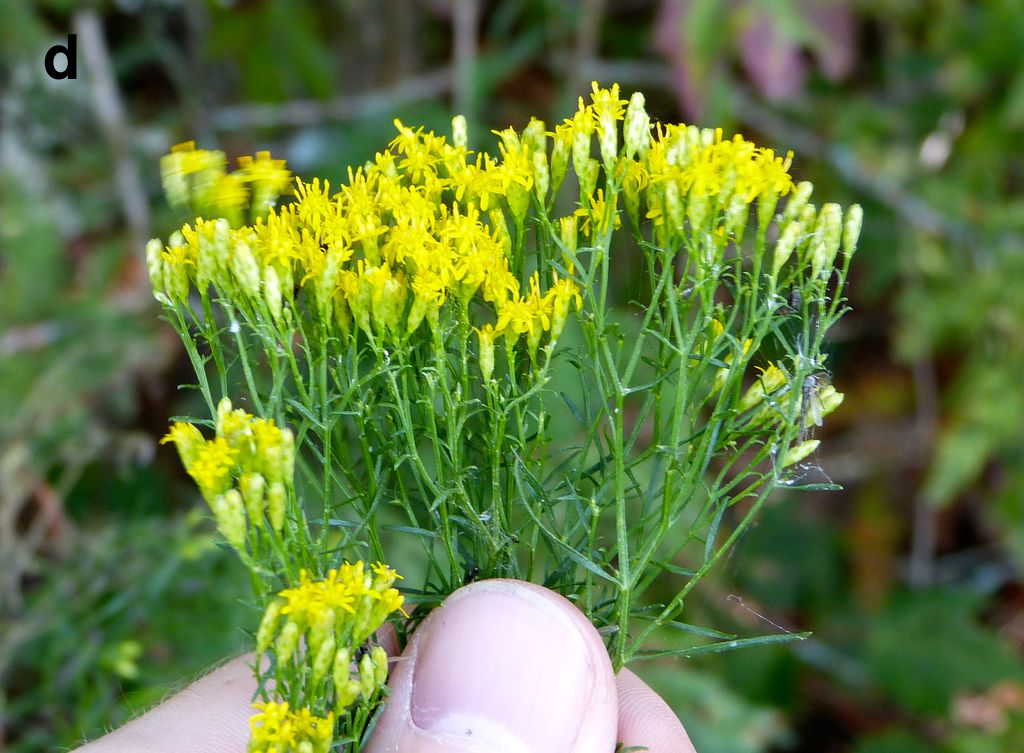
Capitulescence

**Figure 119a.**
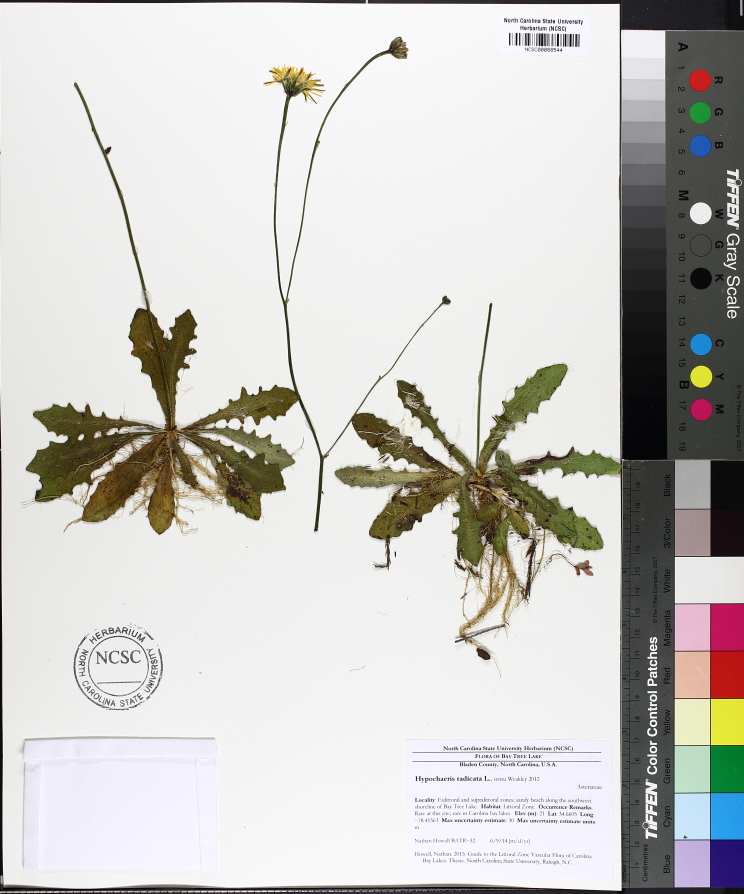
Specimen: *Howell BATR-32* (NCSC)

**Figure 119b.**
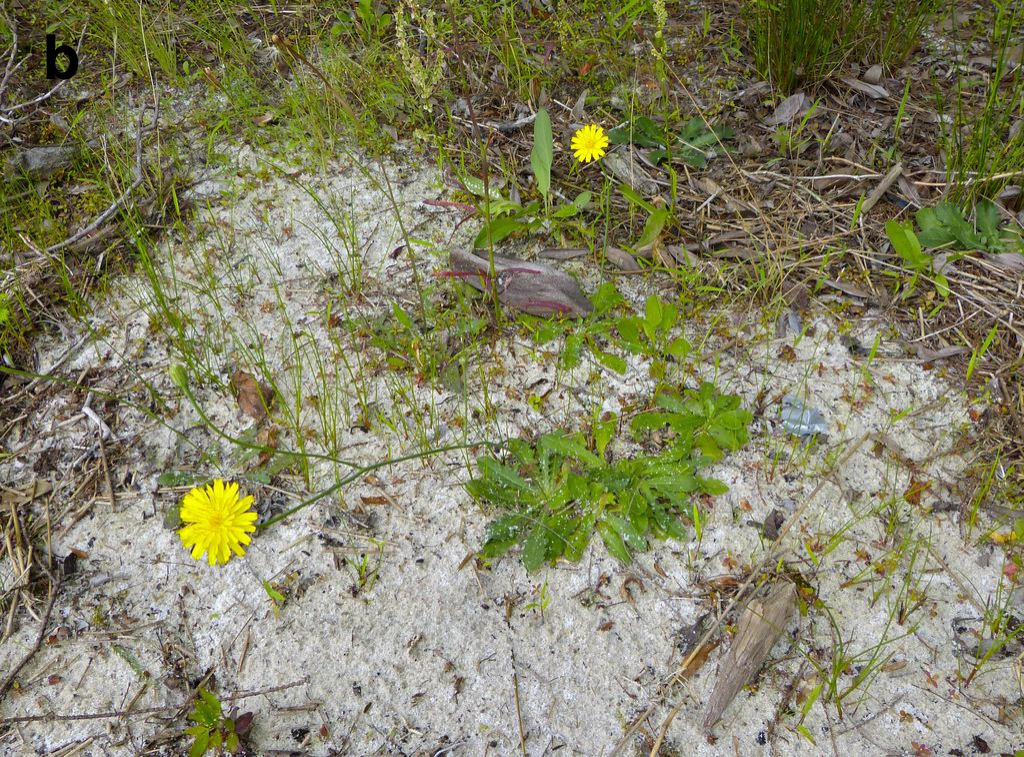
Habit

**Figure 119c.**
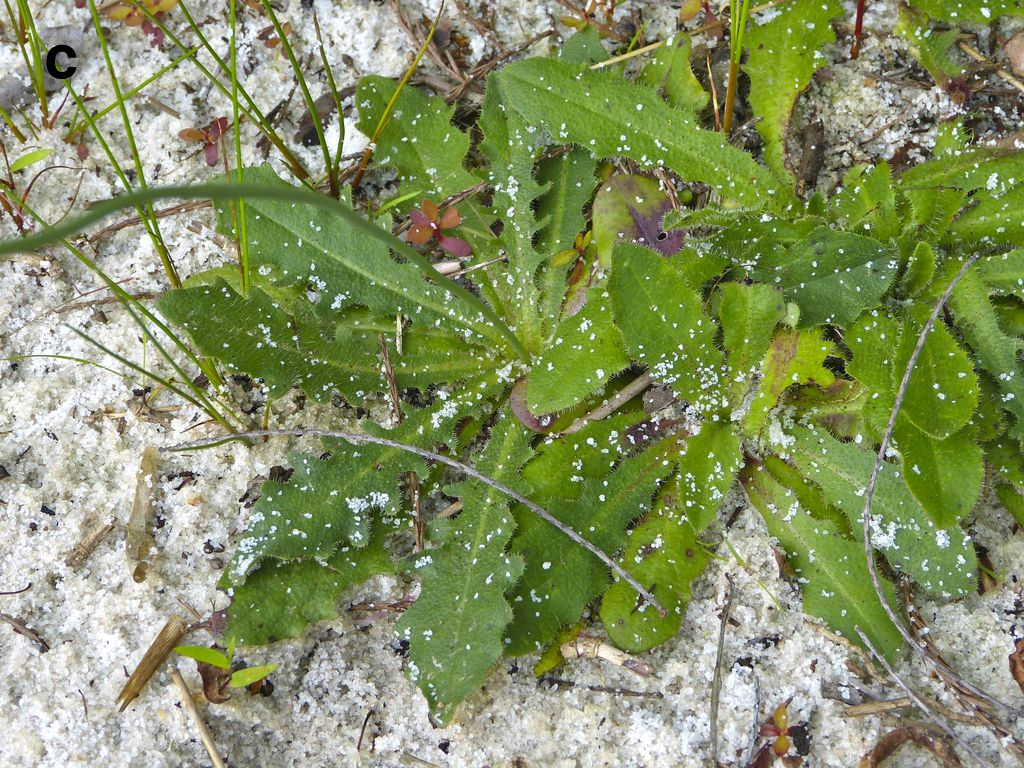
Basal rosette

**Figure 119d.**
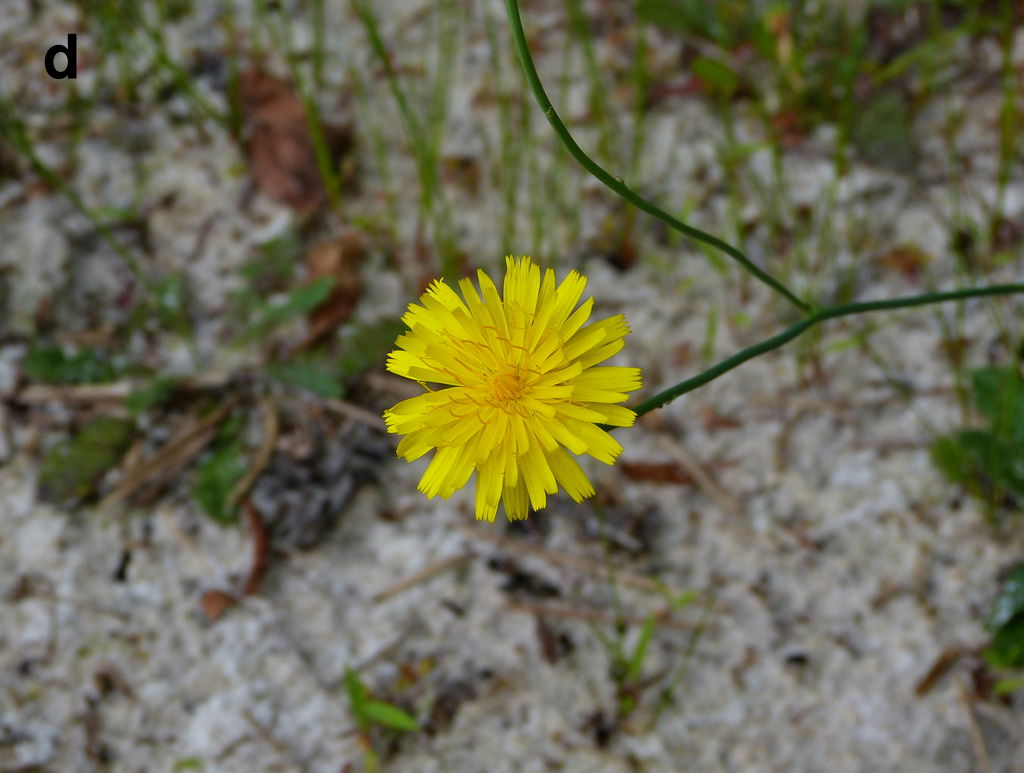
Ligulate head

**Figure 120a.**
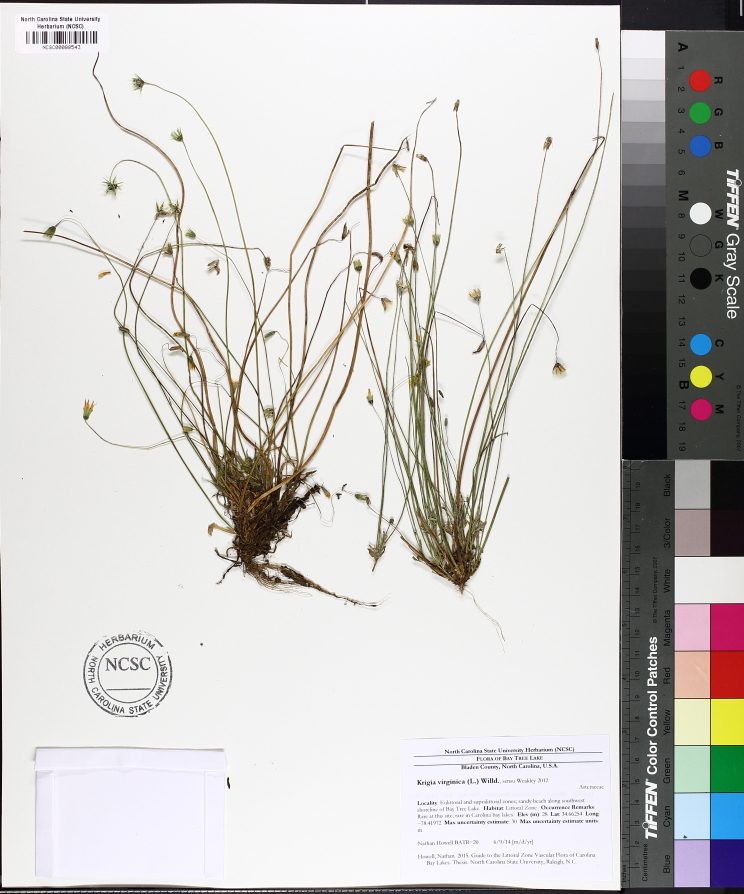
Specimen: *Howell BATR-20* (NCSC)

**Figure 120b.**
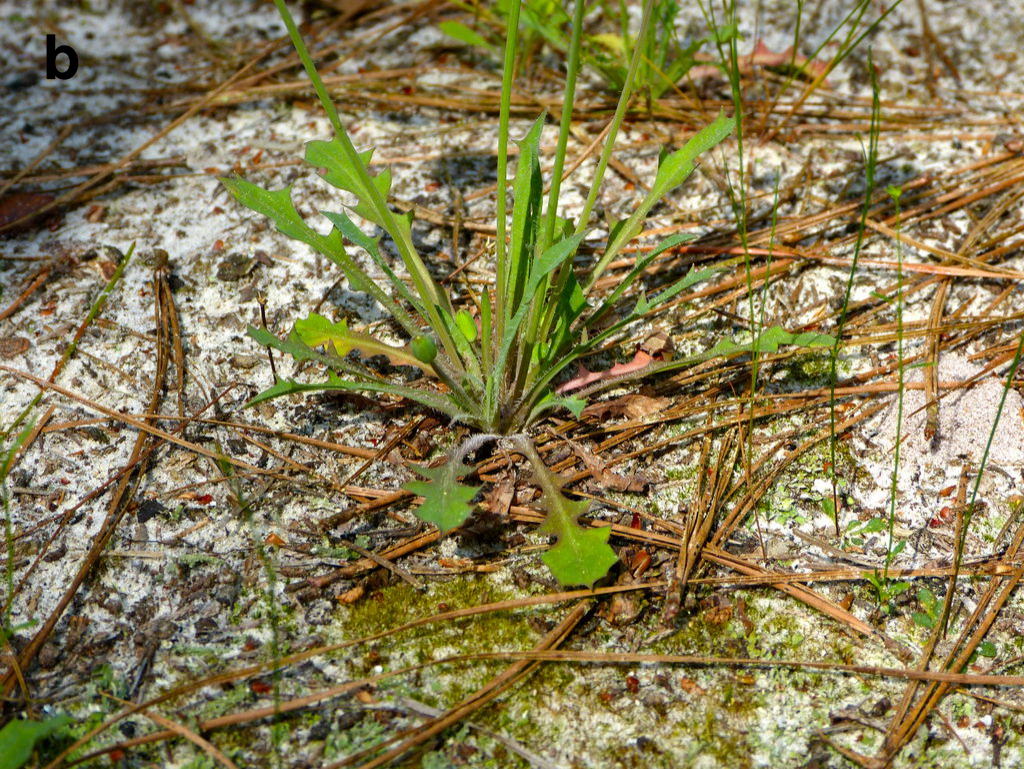
Basal rosette

**Figure 120c.**
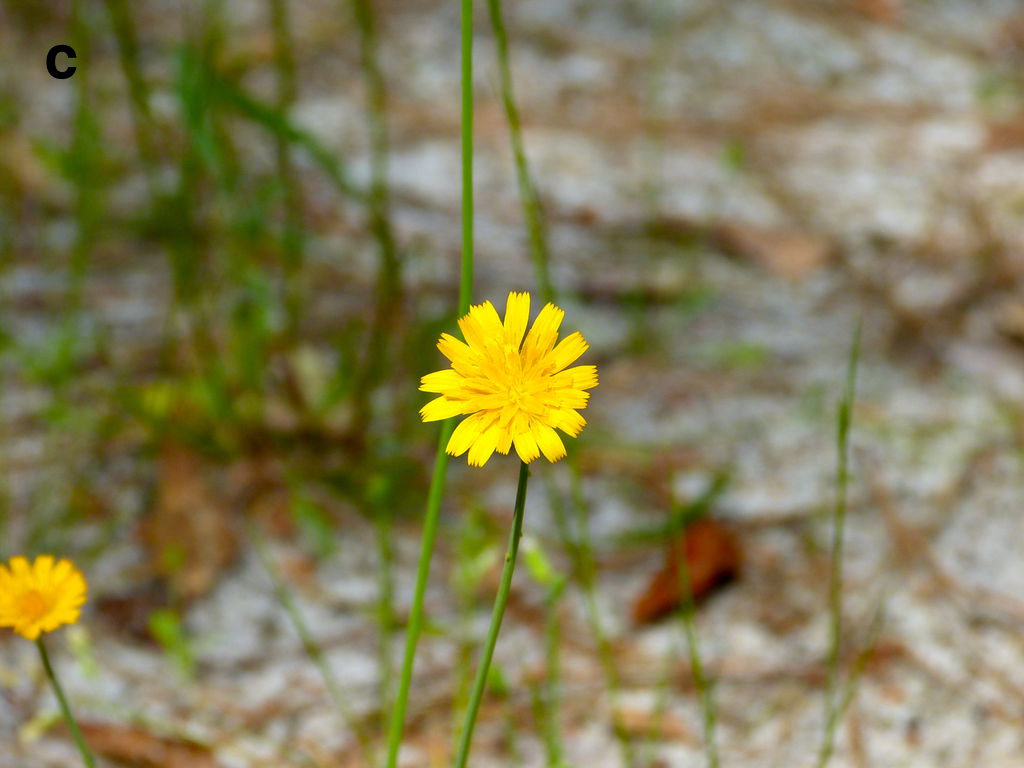
Capitulescence

**Figure 120d.**
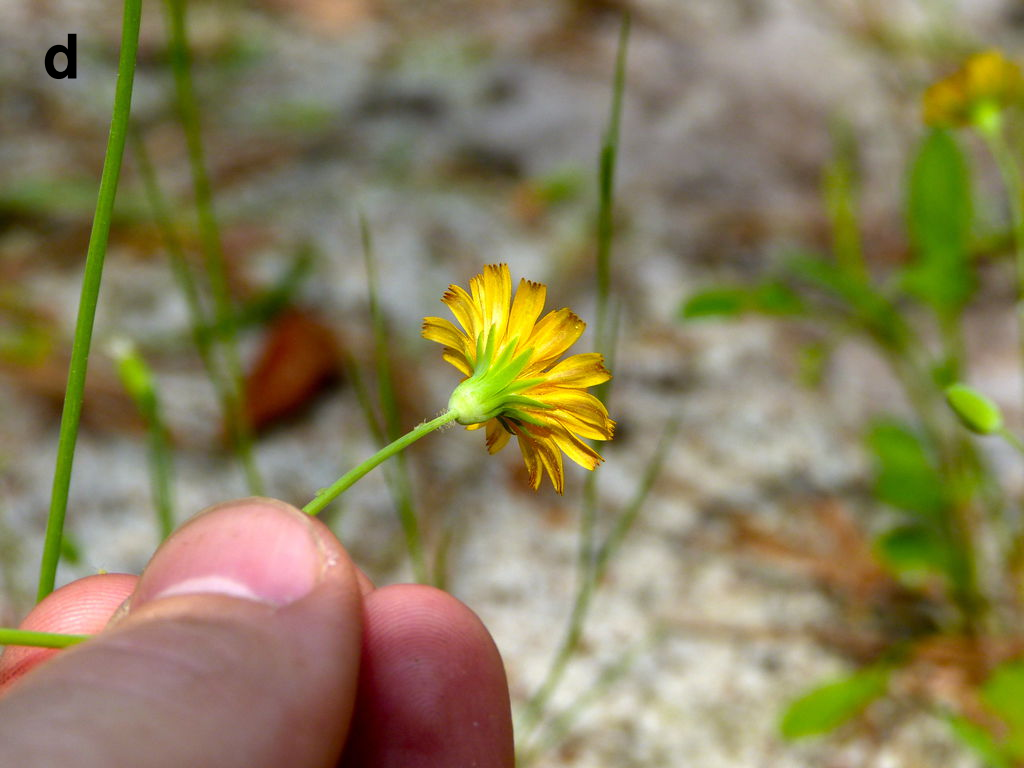
Capitulescence (lateral)

**Figure 121a.**
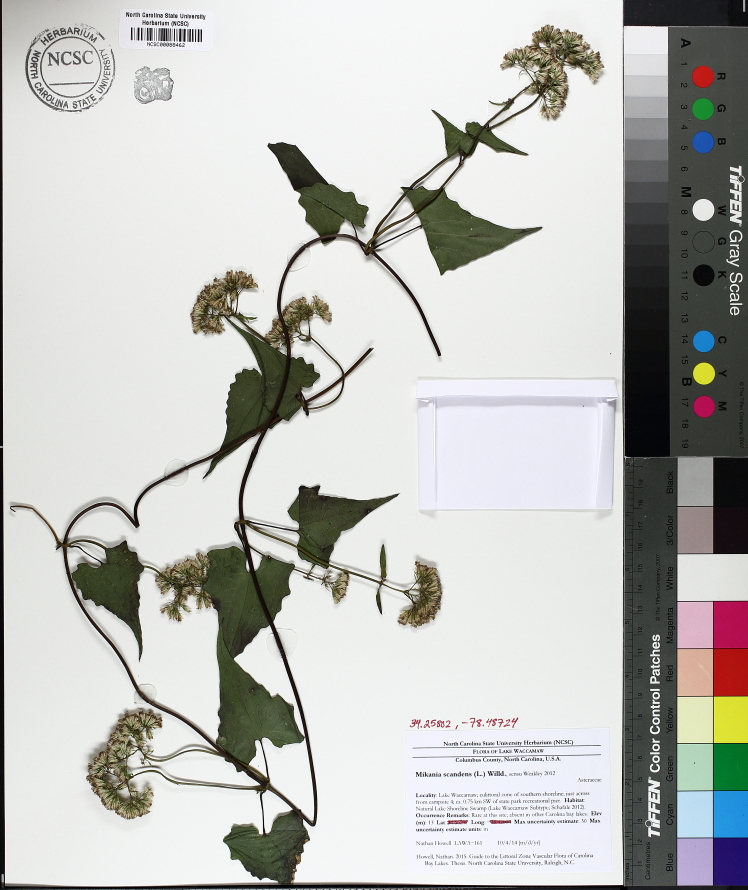
Specimen: *Howell LAWA-161* (NCSC)

**Figure 121b.**
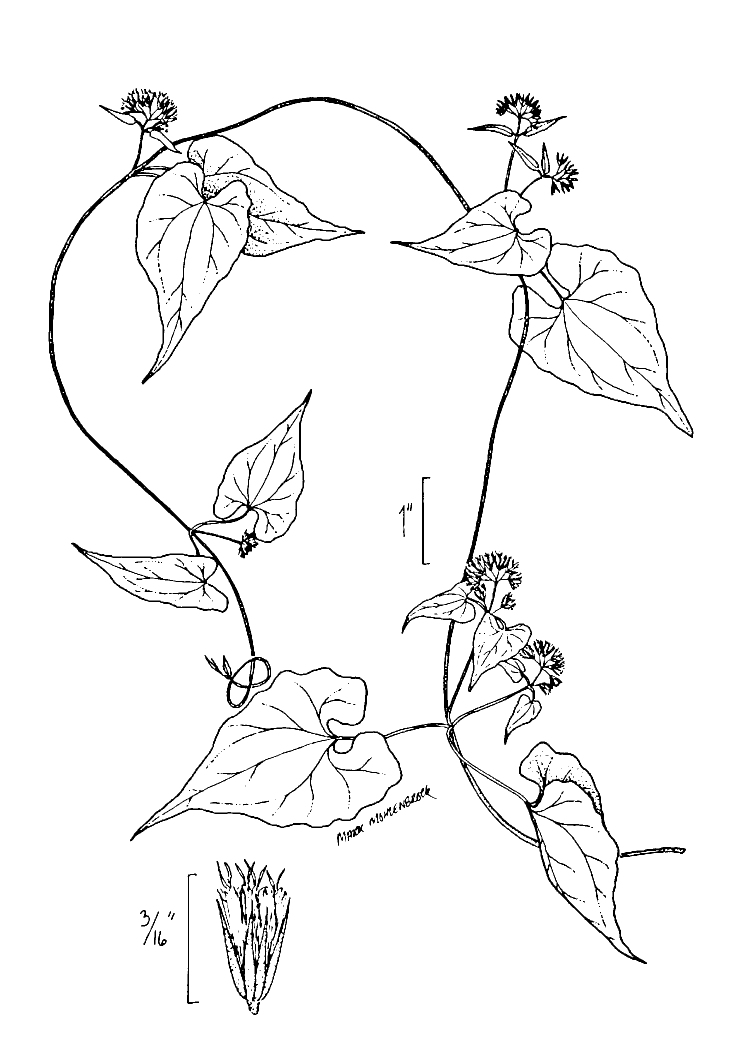
Illustration

**Figure 121c.**
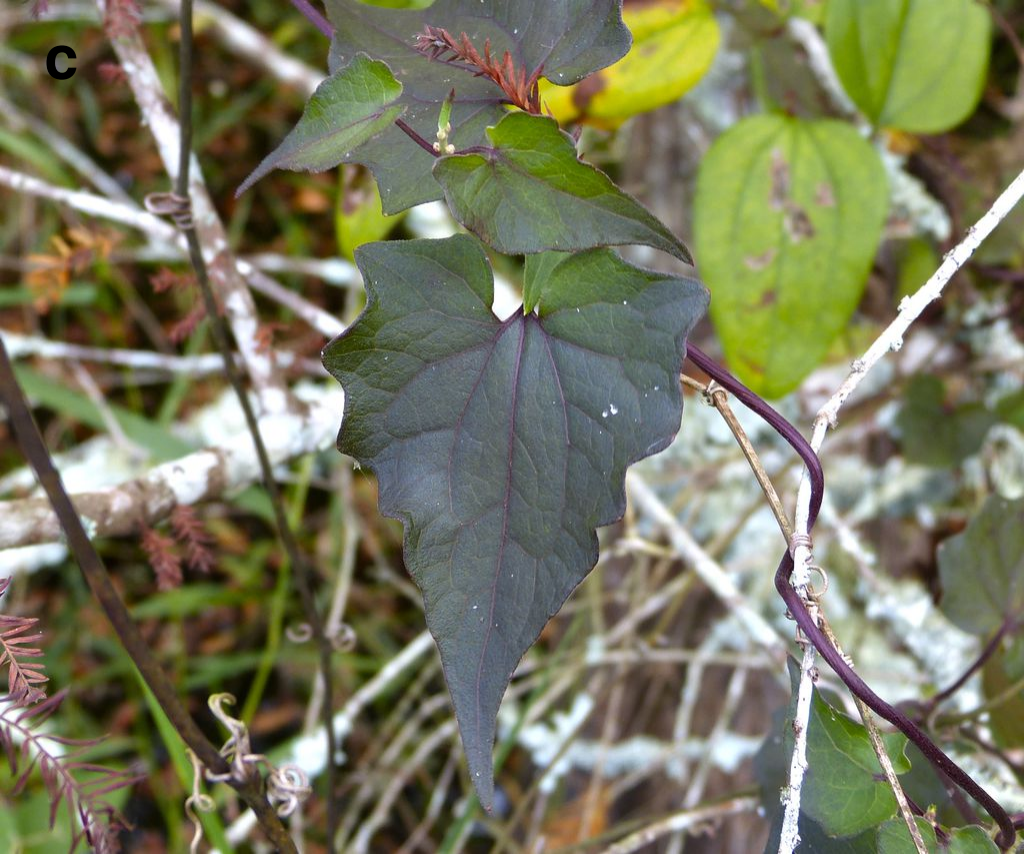
Leaves

**Figure 121d.**
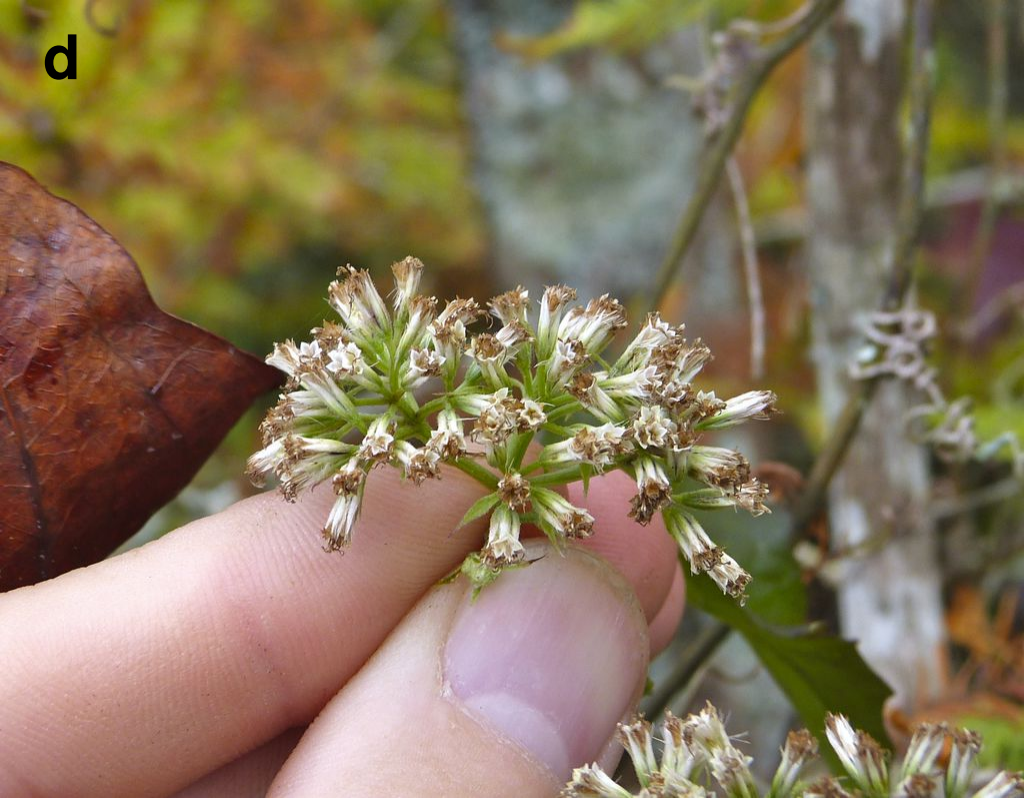
Capitulescence

**Figure 122a.**
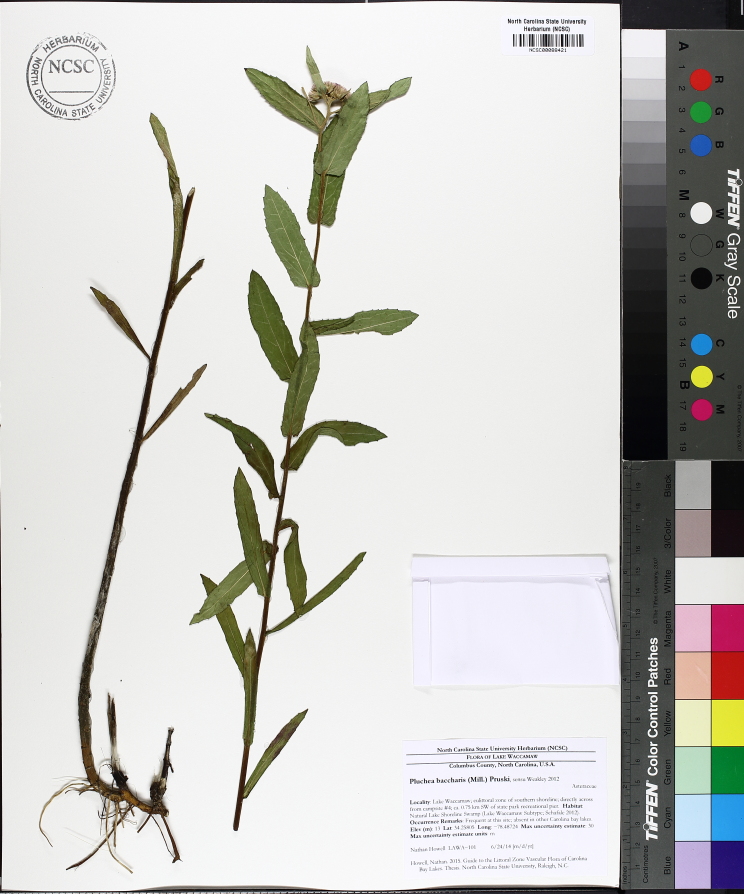
Specimen: *Howell LAWA-101* (NCSC)

**Figure 122b.**
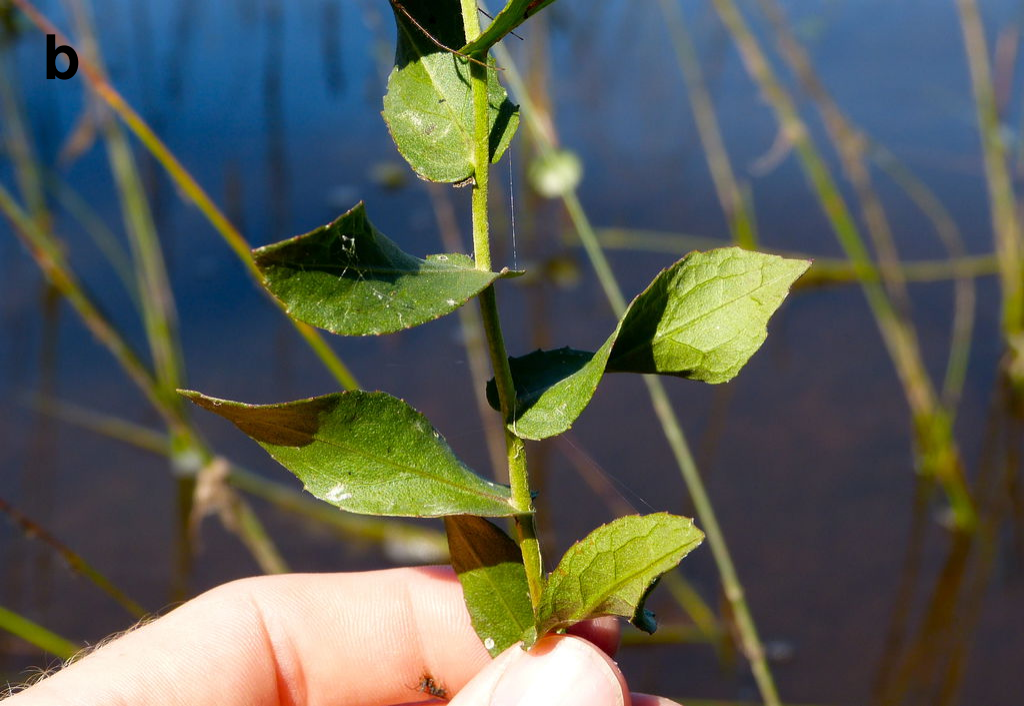
Stem and leaves

**Figure 122c.**
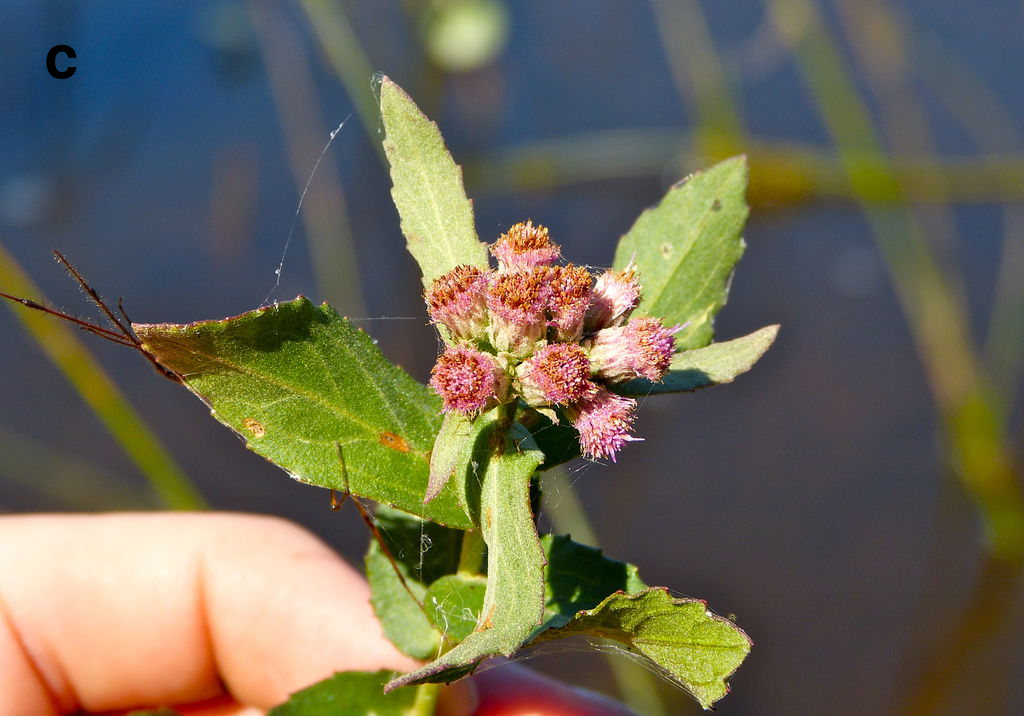
Capitulescence

**Figure 122d.**
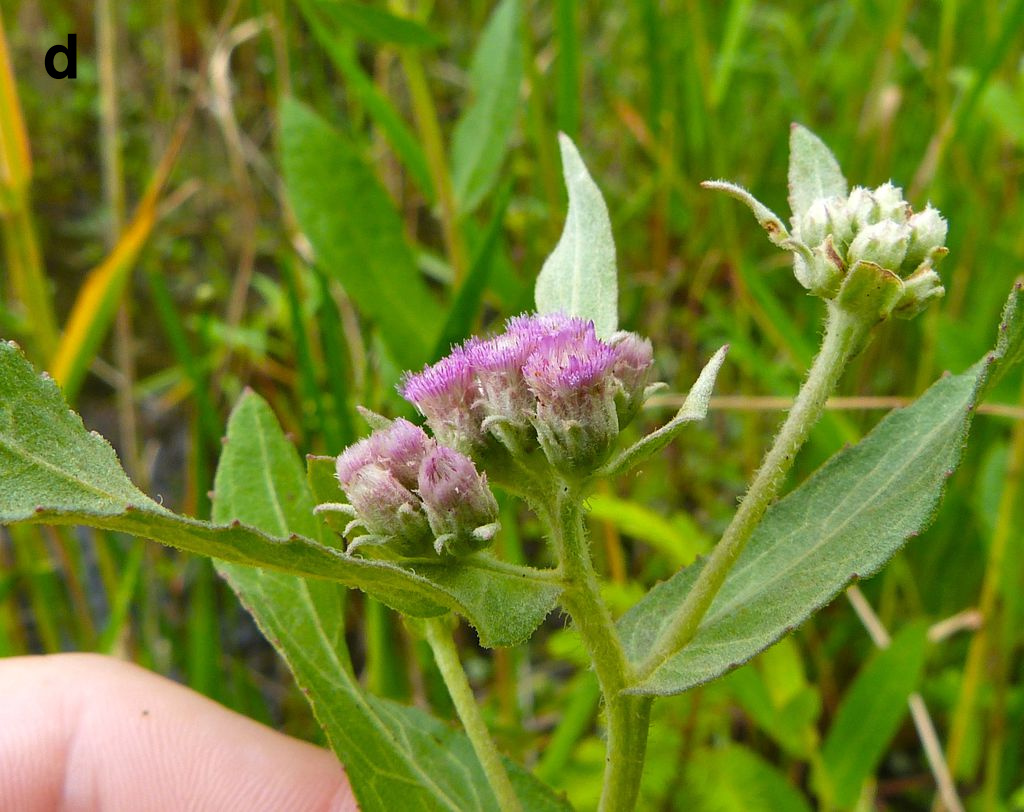
Capitulescence

**Figure 123a.**
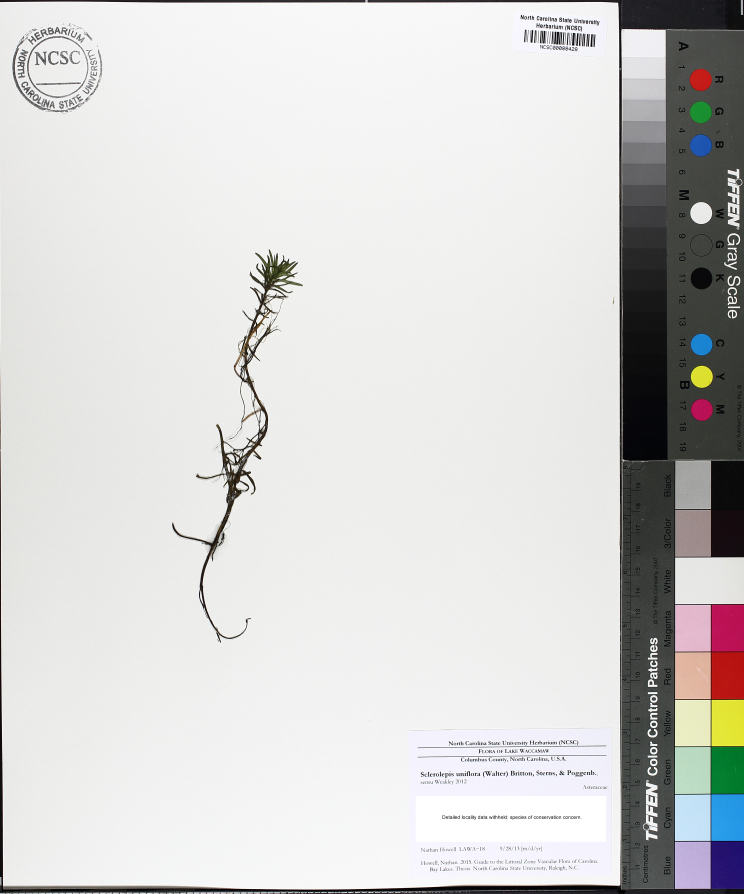
Specimen: *Howell LAWA-18* (NCSC)

**Figure 123b.**
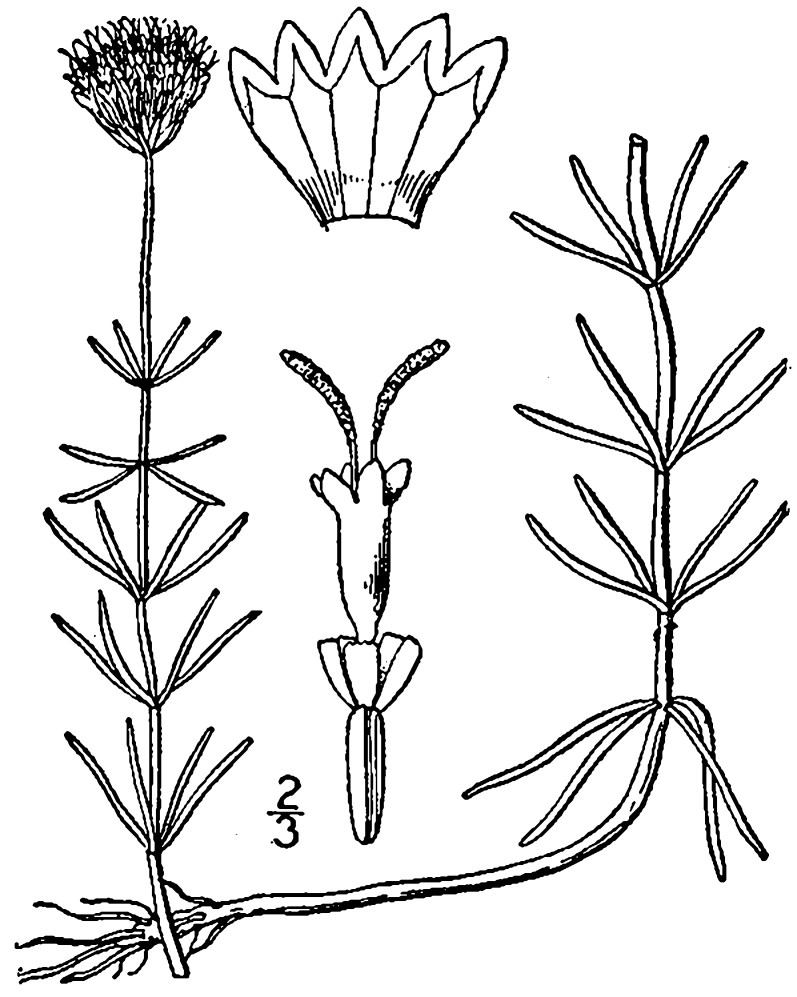
Illustration

**Figure 123c.**
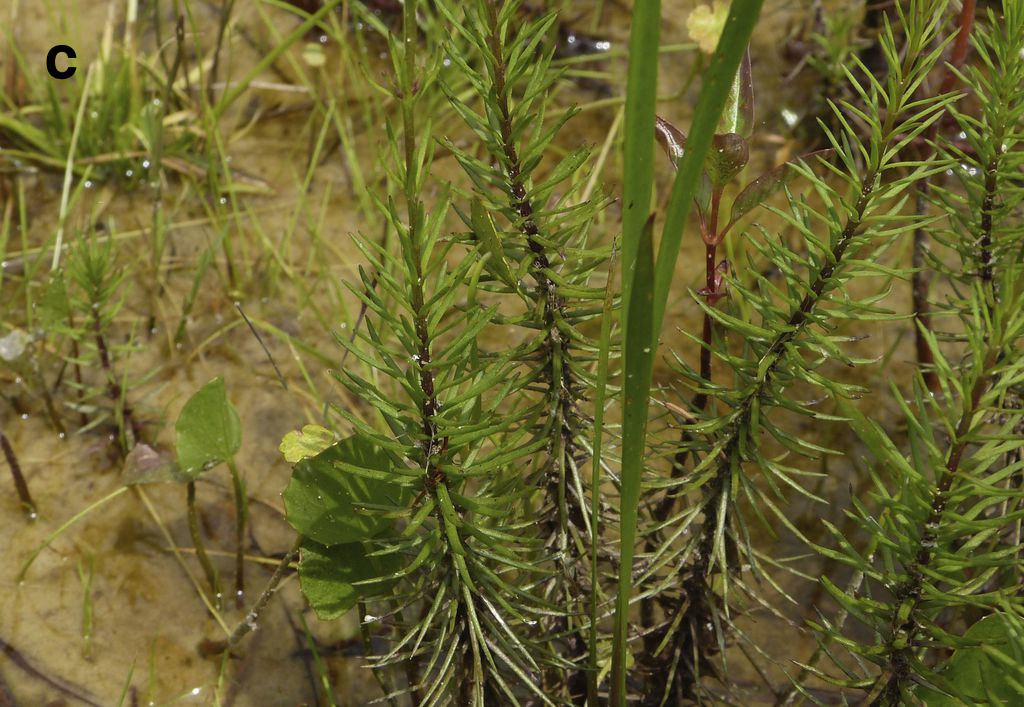
Habit

**Figure 123d.**
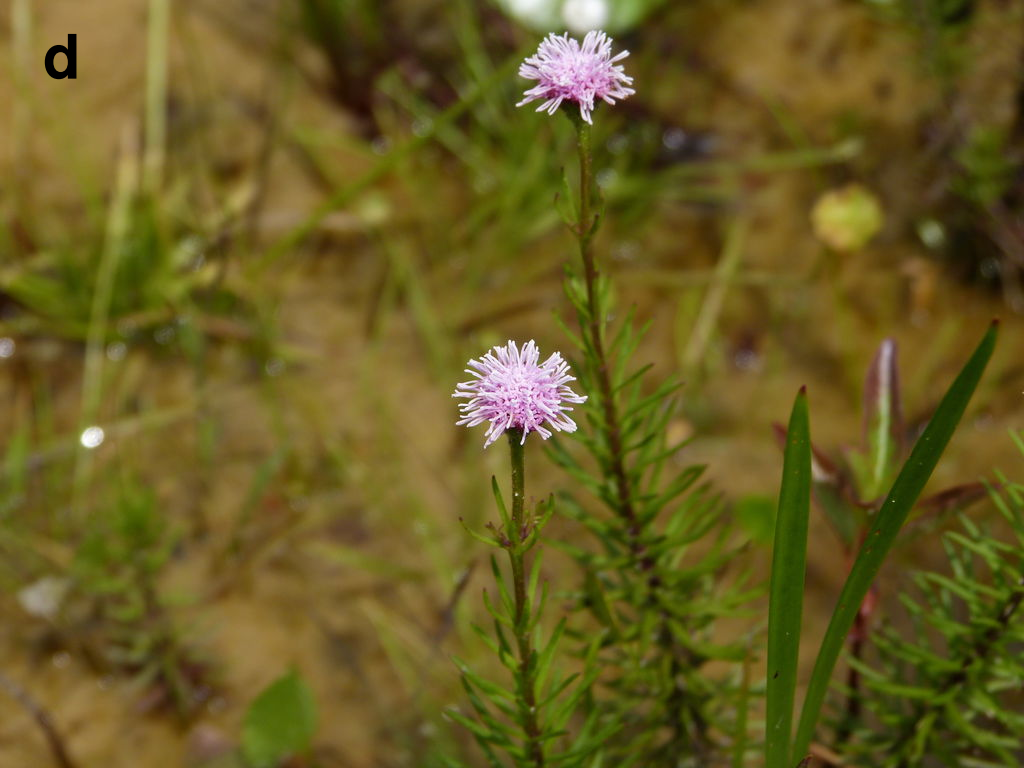
Capitulescence

**Figure 124a.**
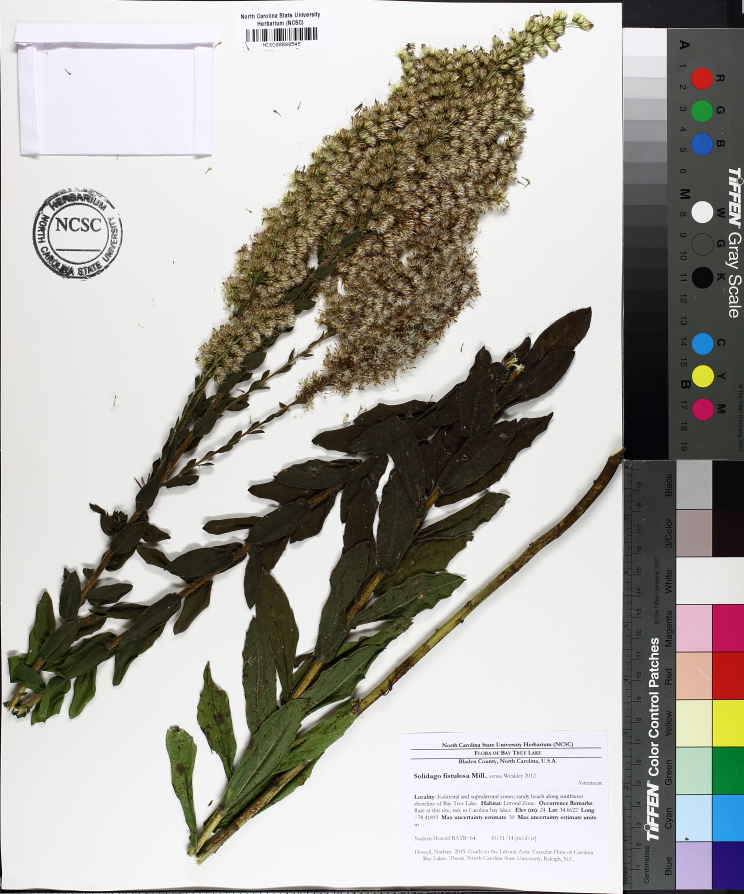
Specimen: *Howell BATR-64* (NCSC)

**Figure 124b.**
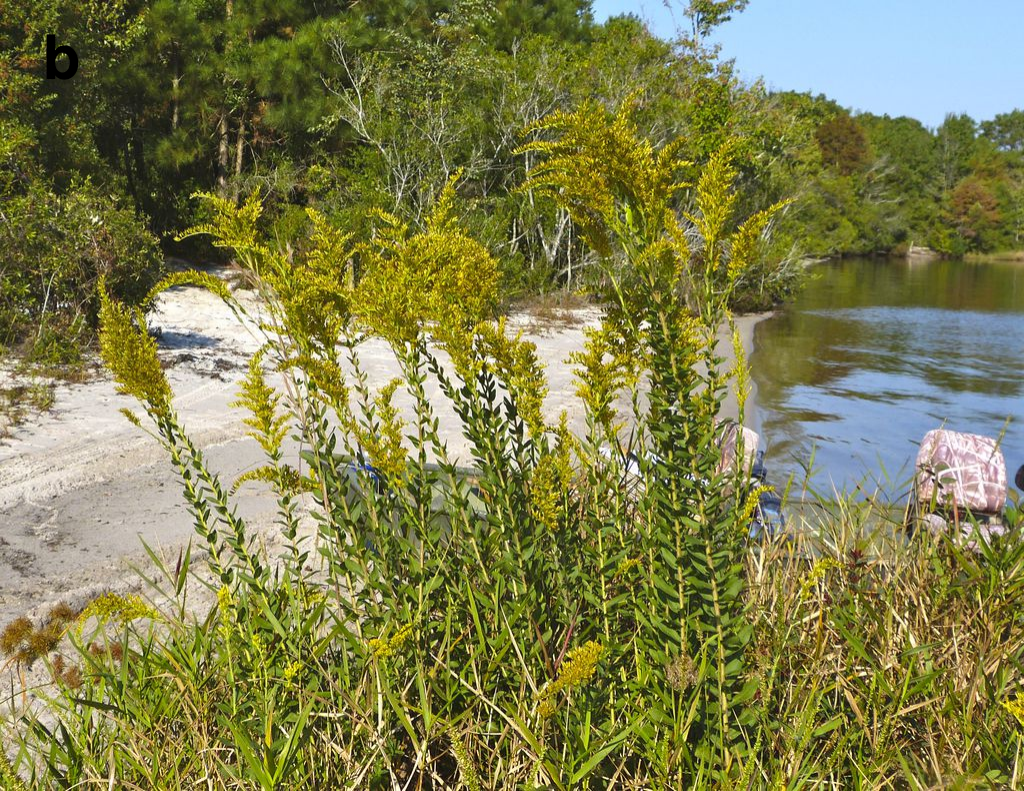
Habit

**Figure 124c.**
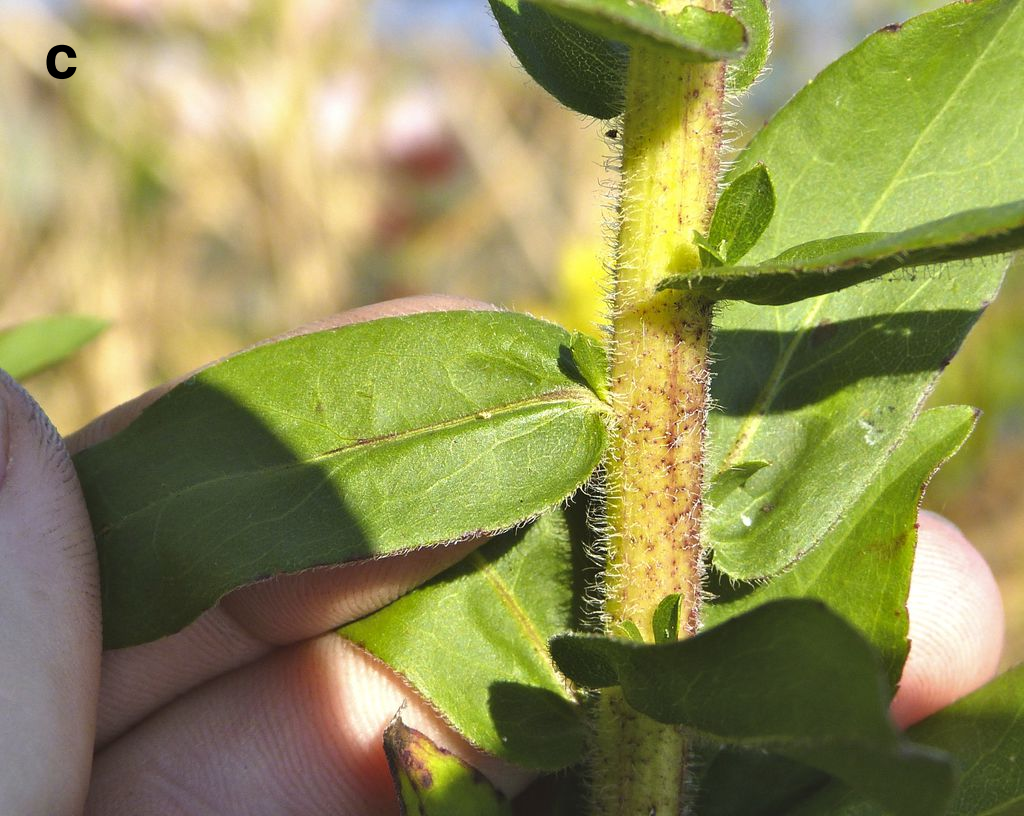
Stem and leaves

**Figure 124d.**
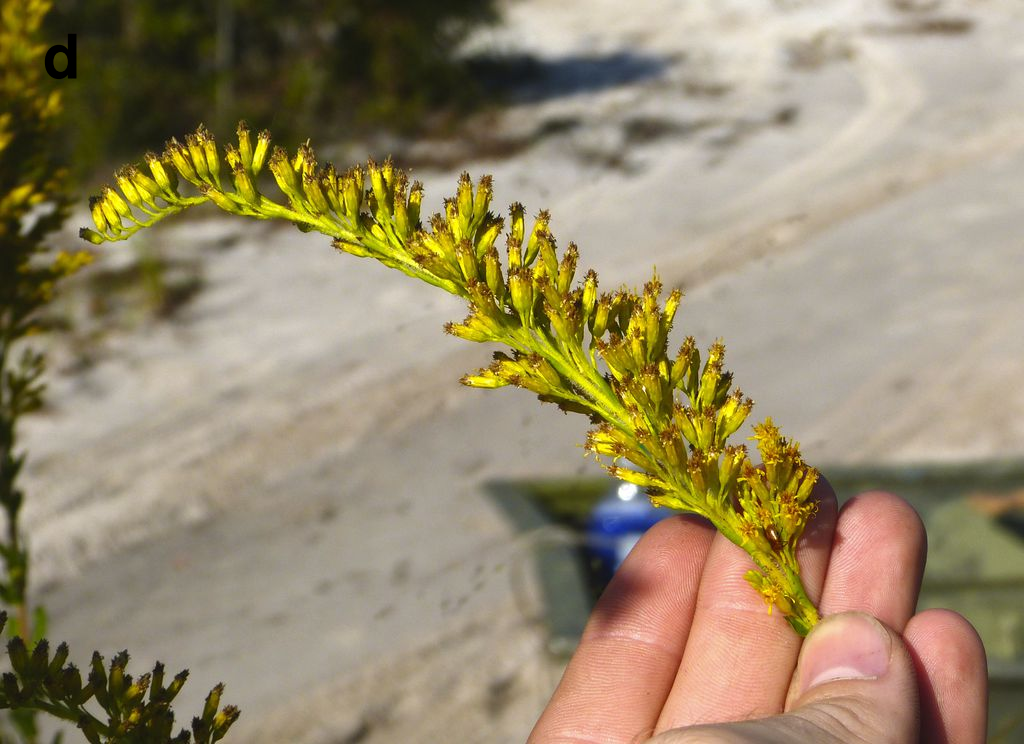
Capitulescence

**Figure 125a.**
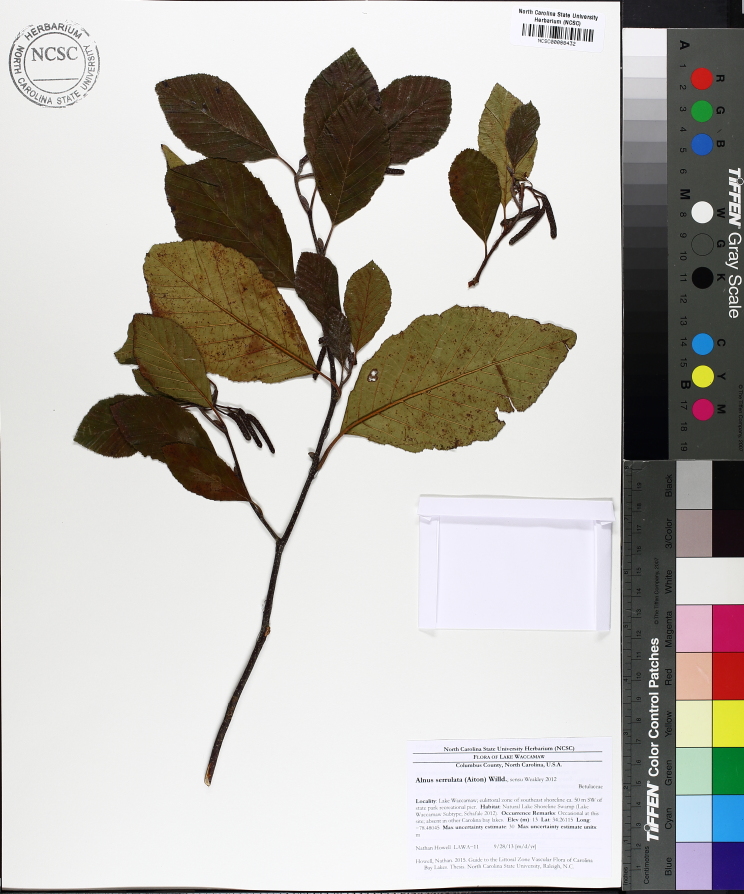
Specimen: *Howell LAWA-11* (NCSC)

**Figure 125b.**
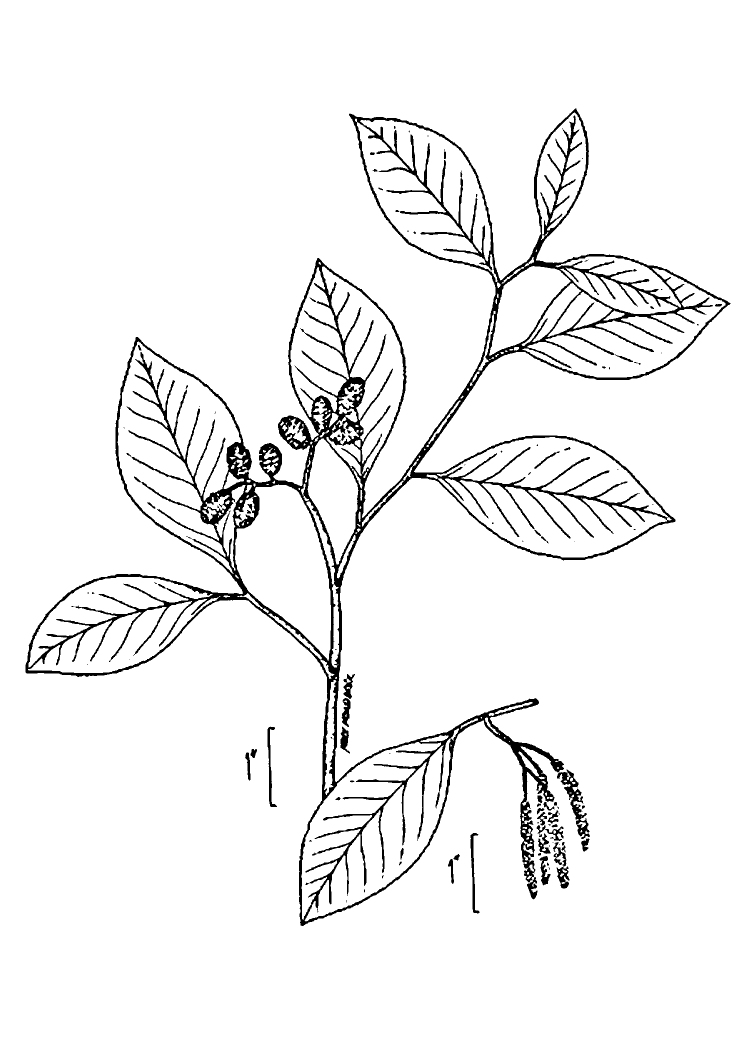
Illustration

**Figure 125c.**
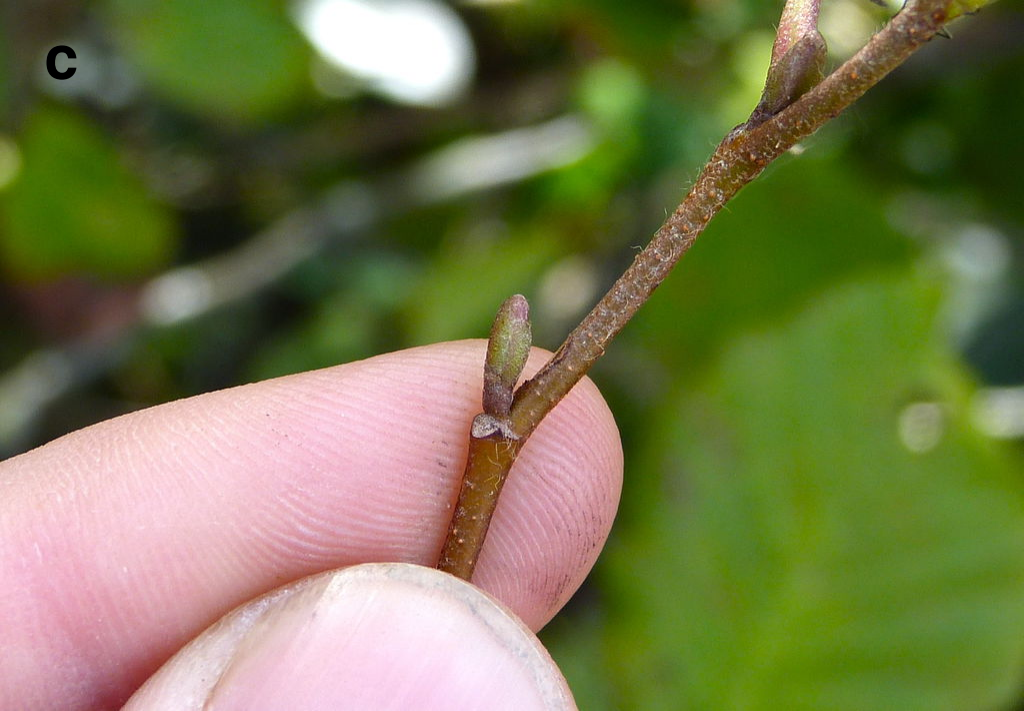
Twig and axillary bud

**Figure 125d.**
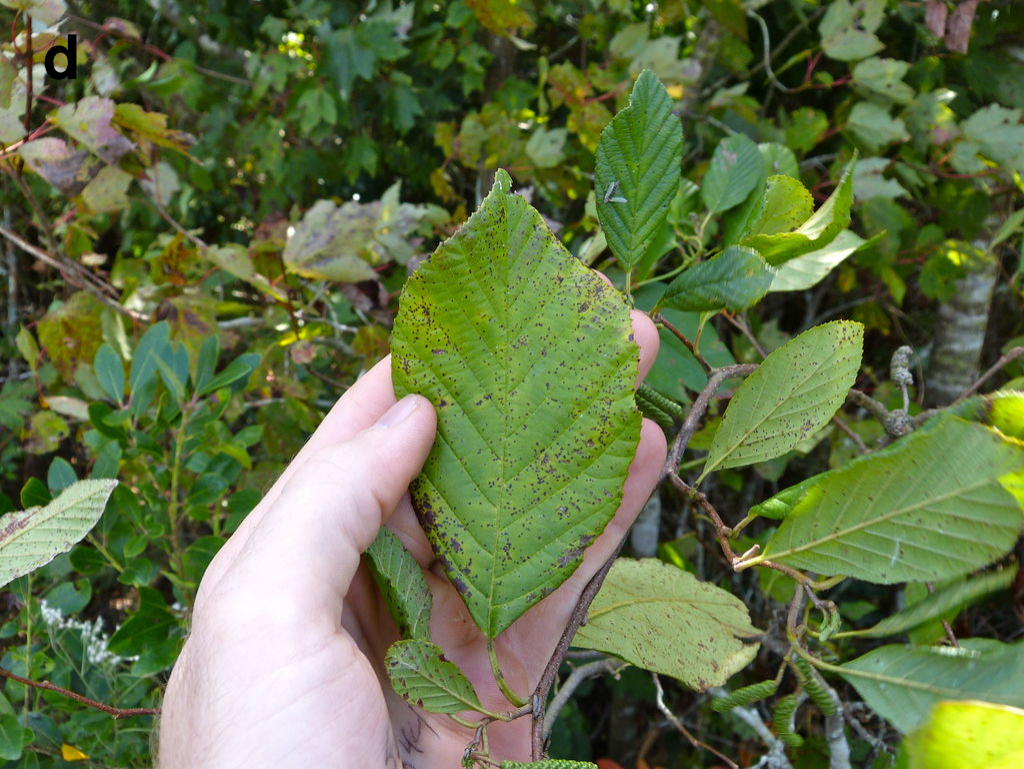
Leaf

**Figure 125e.**
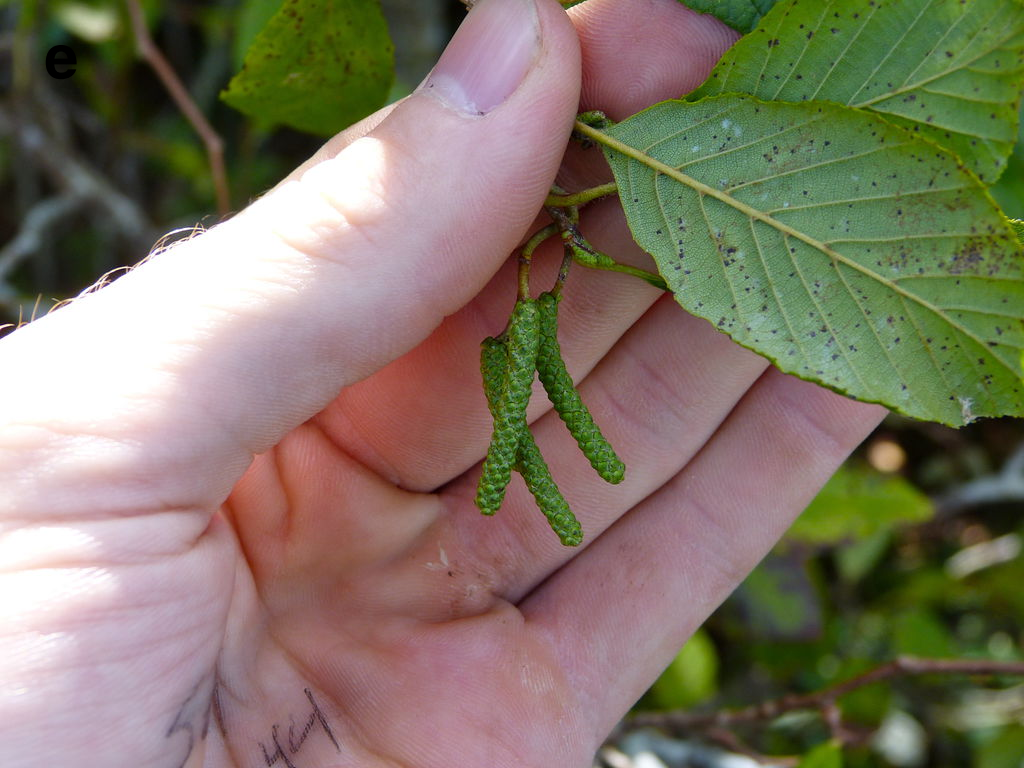
Staminate catkin

**Figure 125f.**
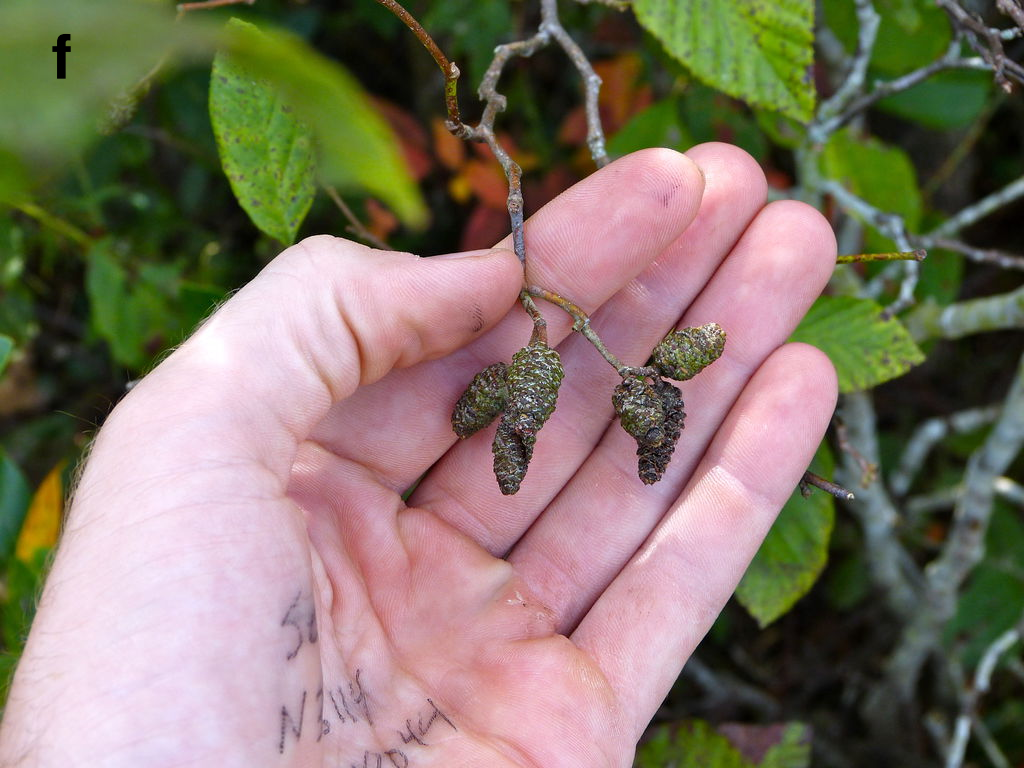
Pistillate catkin

**Figure 126a.**
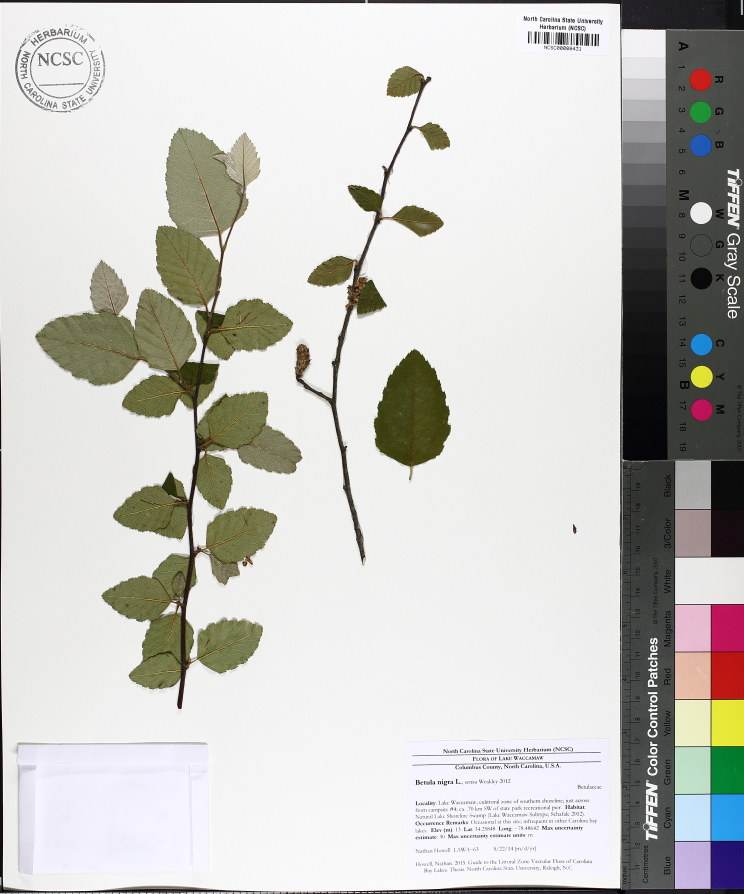
Specimen: *Howell LAWA-63* (NCSC)

**Figure 126b.**
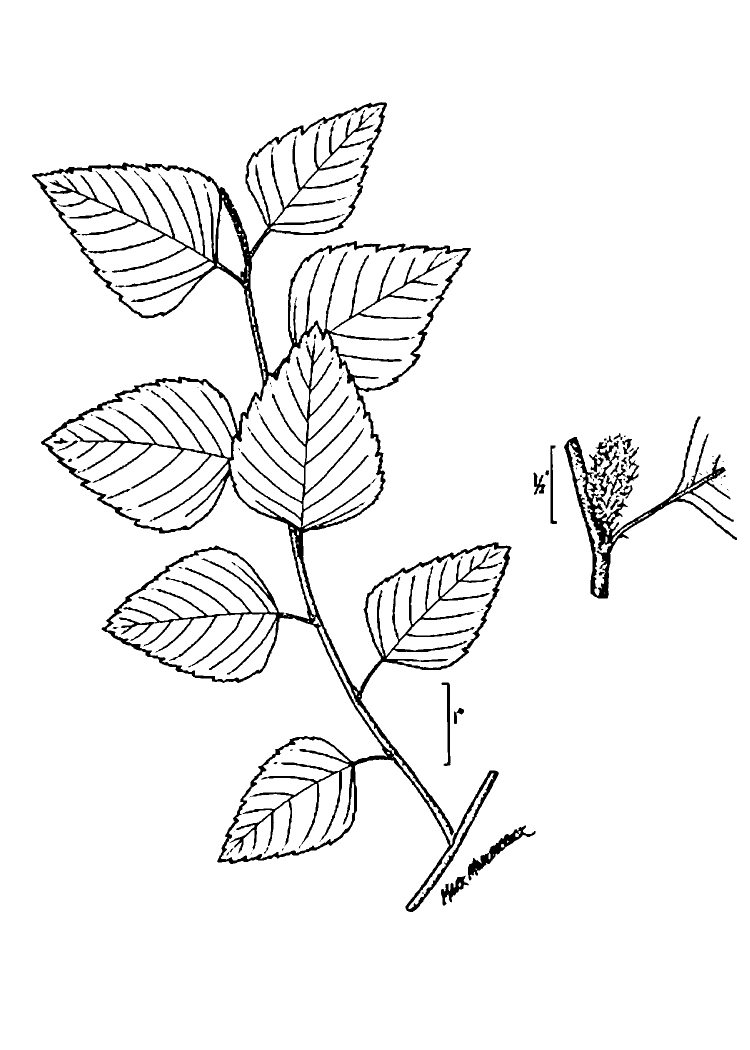
Illustration

**Figure 126c.**
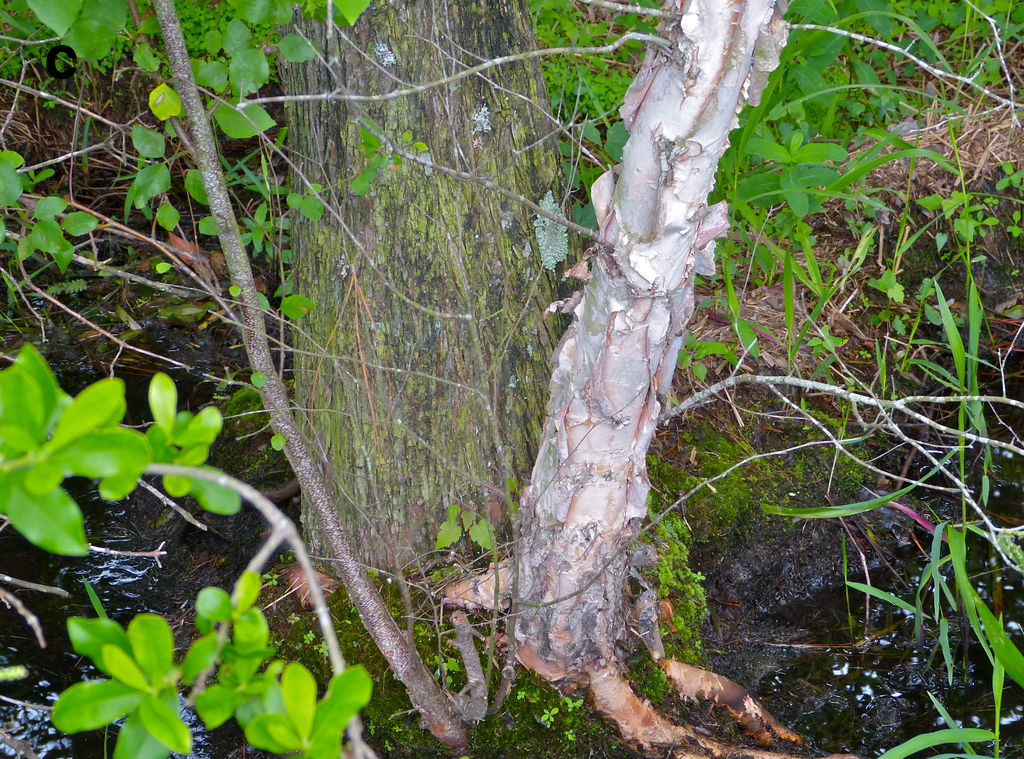
Bark (young tree on right)

**Figure 126d.**
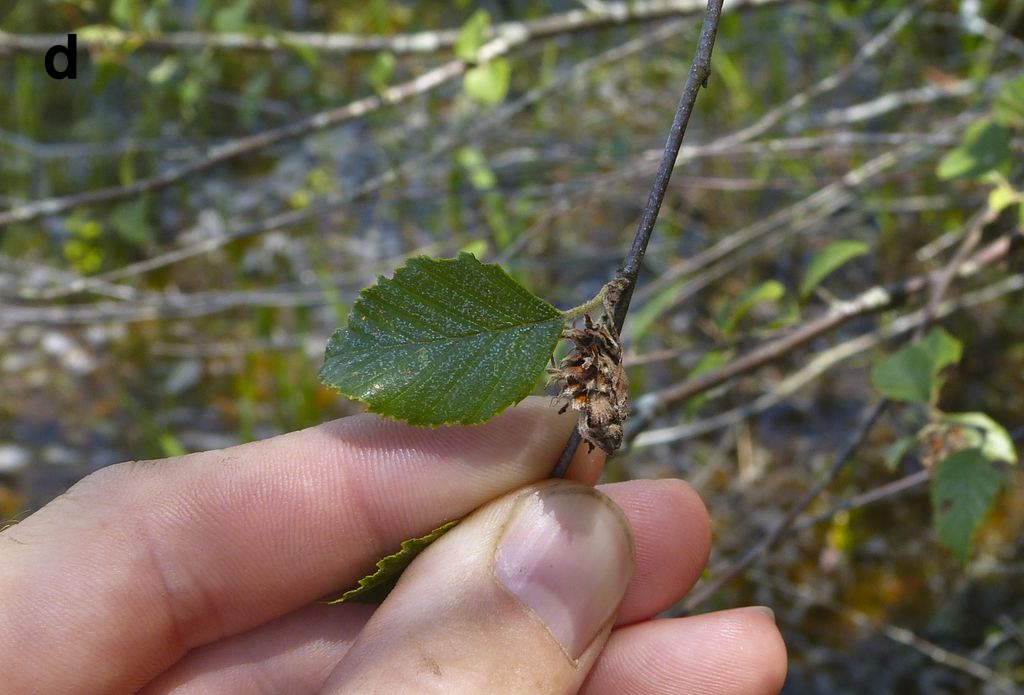
Pistillate catkin

**Figure 127a.**
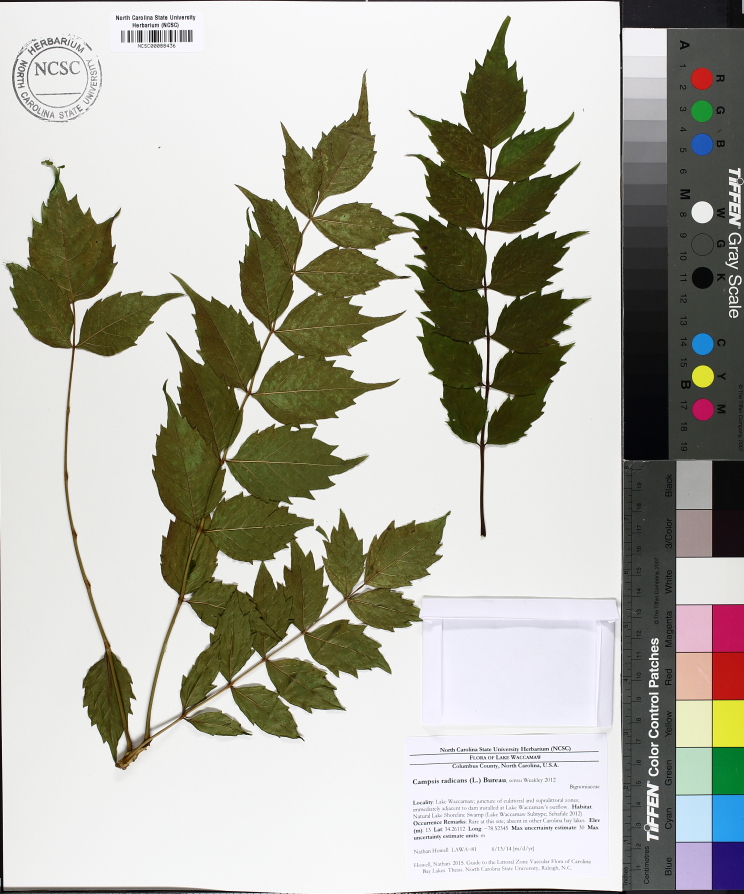
Specimen: *Howell-LAWA 81* (NCSC)

**Figure 127b.**
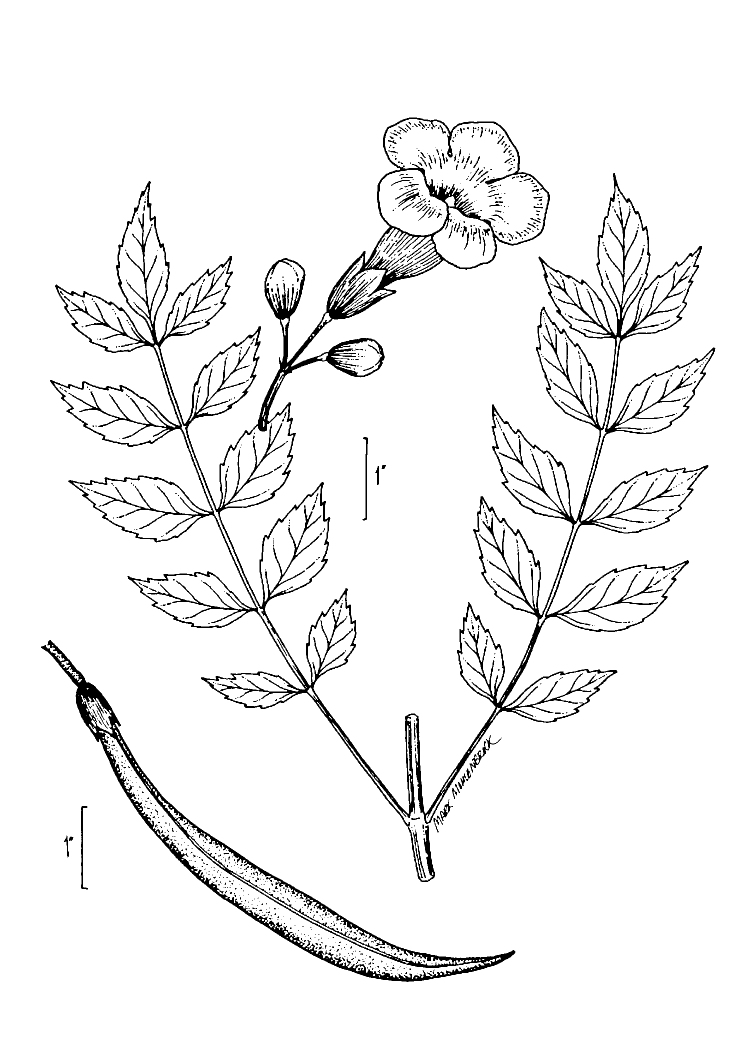
lllustration

**Figure 127c.**
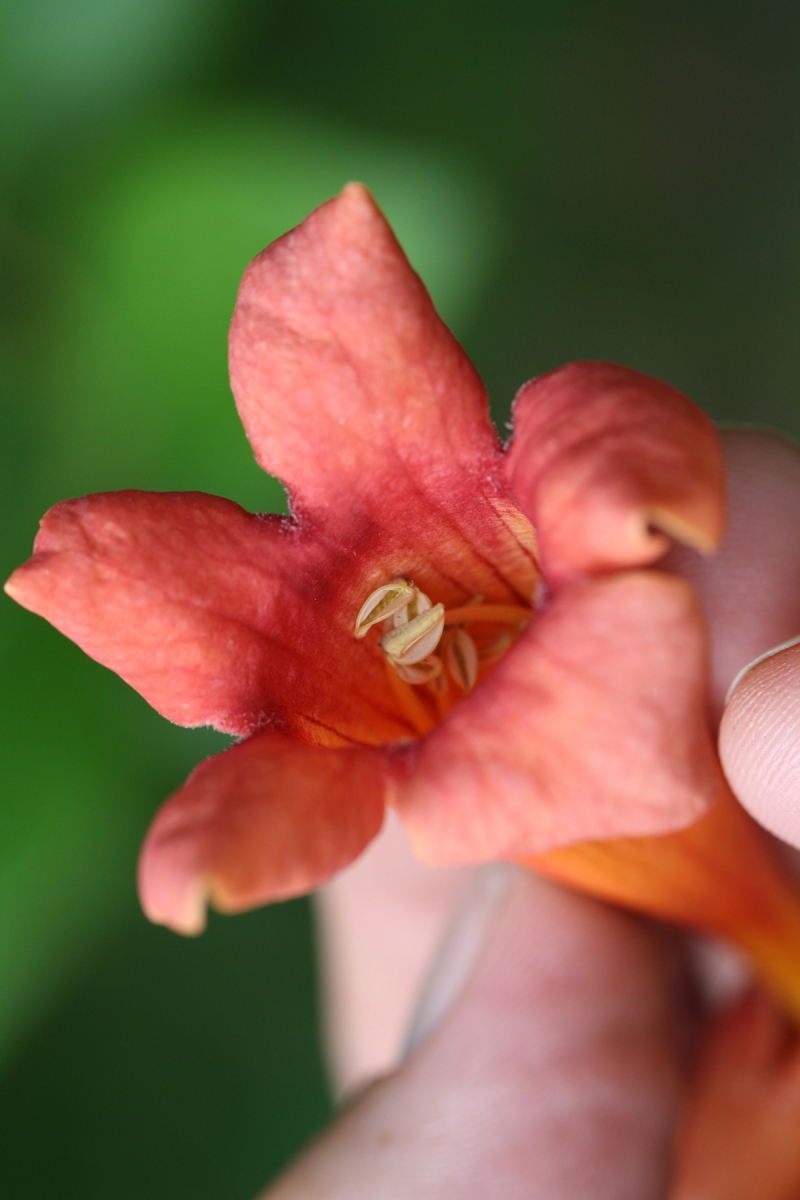
Flower

**Figure 127d.**
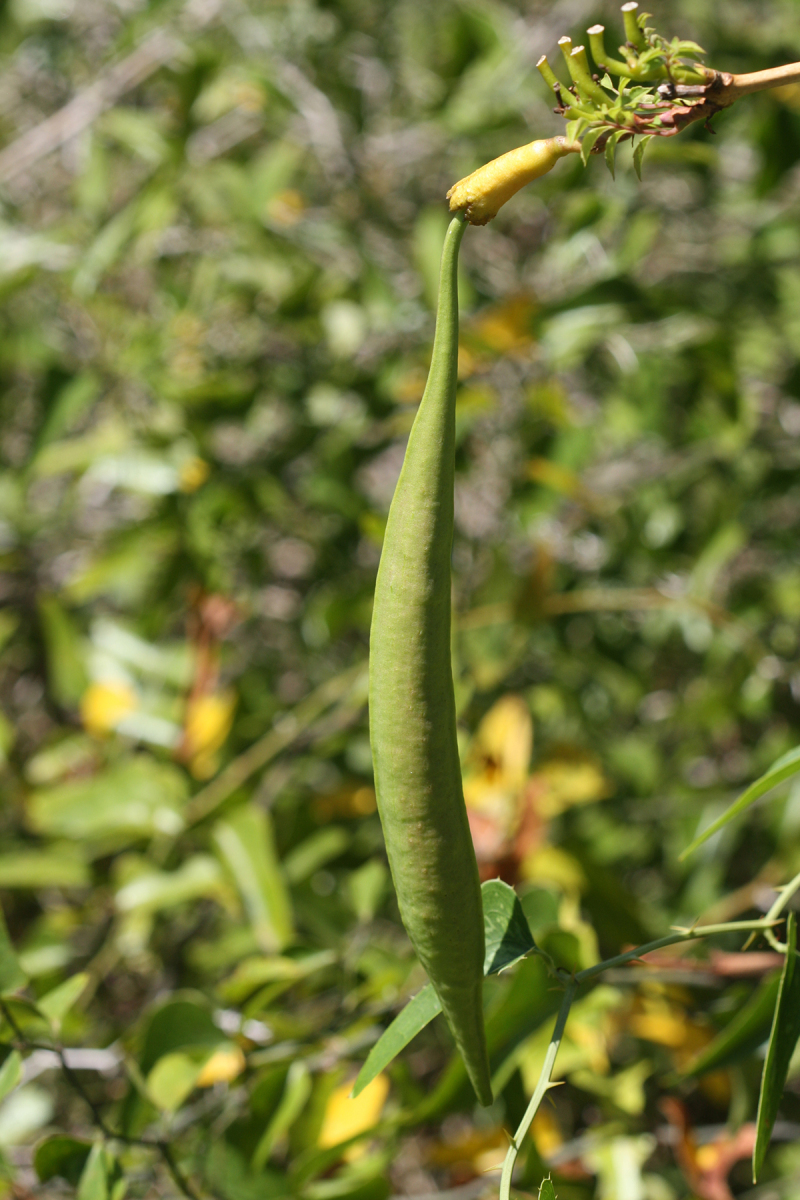
Fruit

**Figure 128a.**
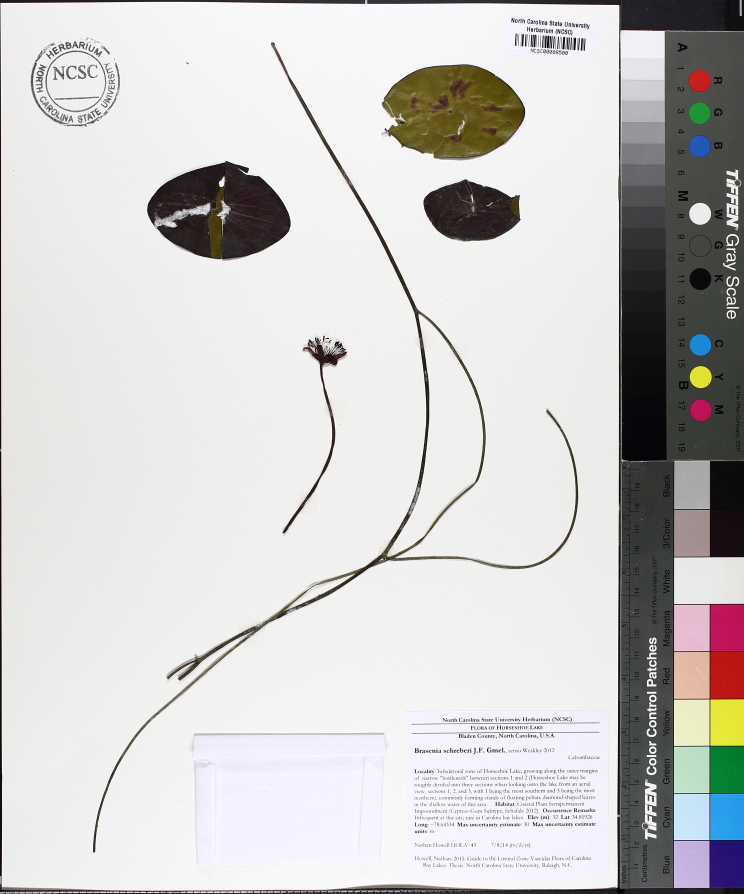
Specimen: *Howell HOLA-43* (NCSC)

**Figure 128b.**
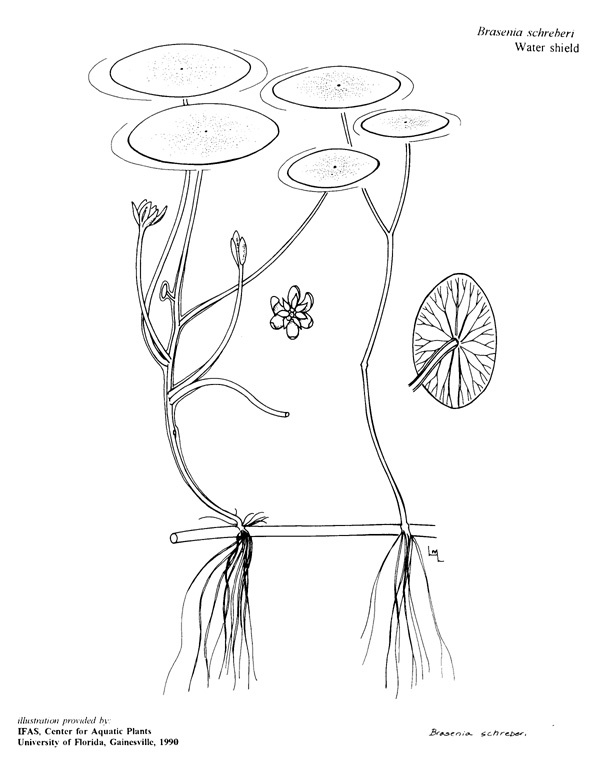
Illustration

**Figure 128c.**
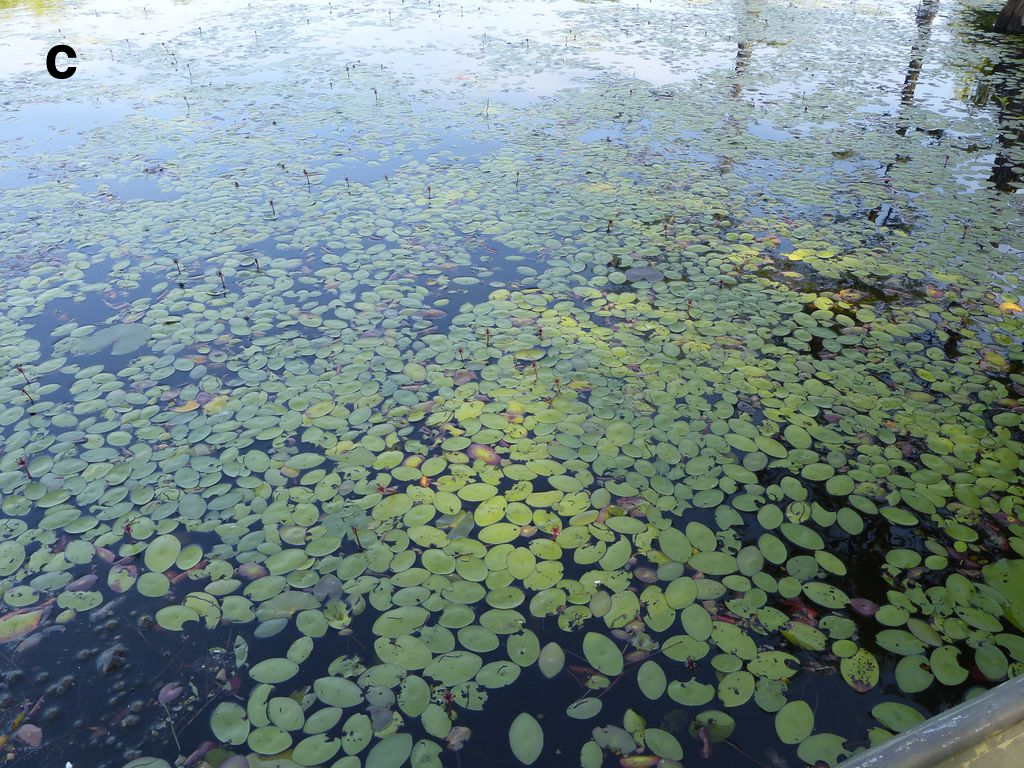
Habit

**Figure 128d.**
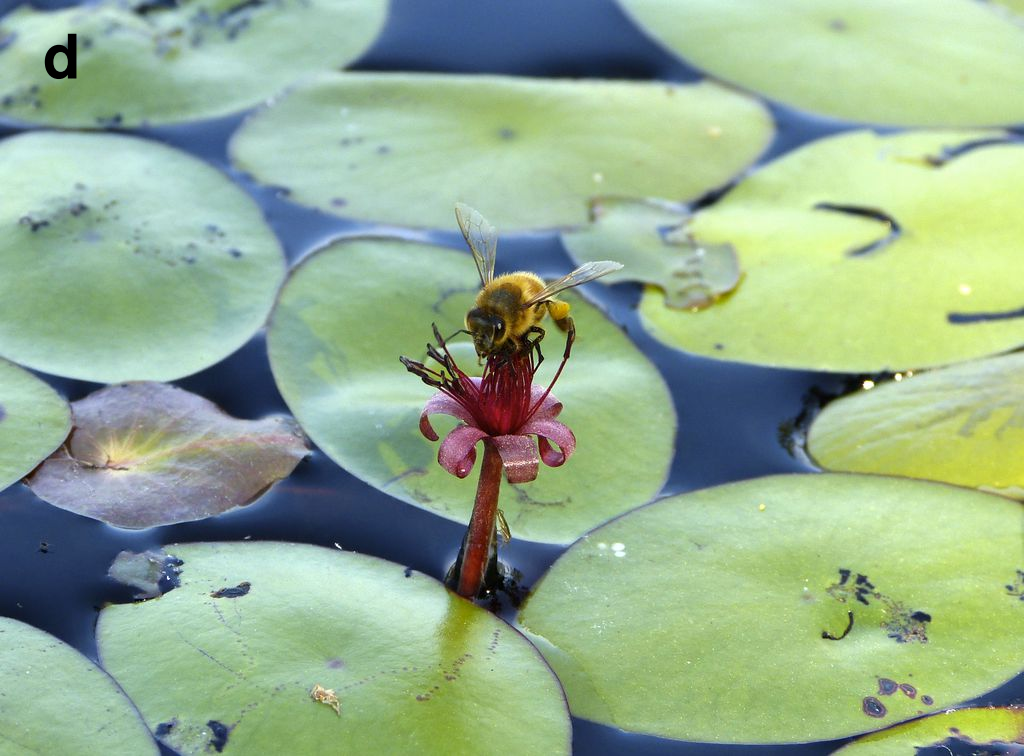
Flower (with hymenopteran visitor)

**Figure 129a.**
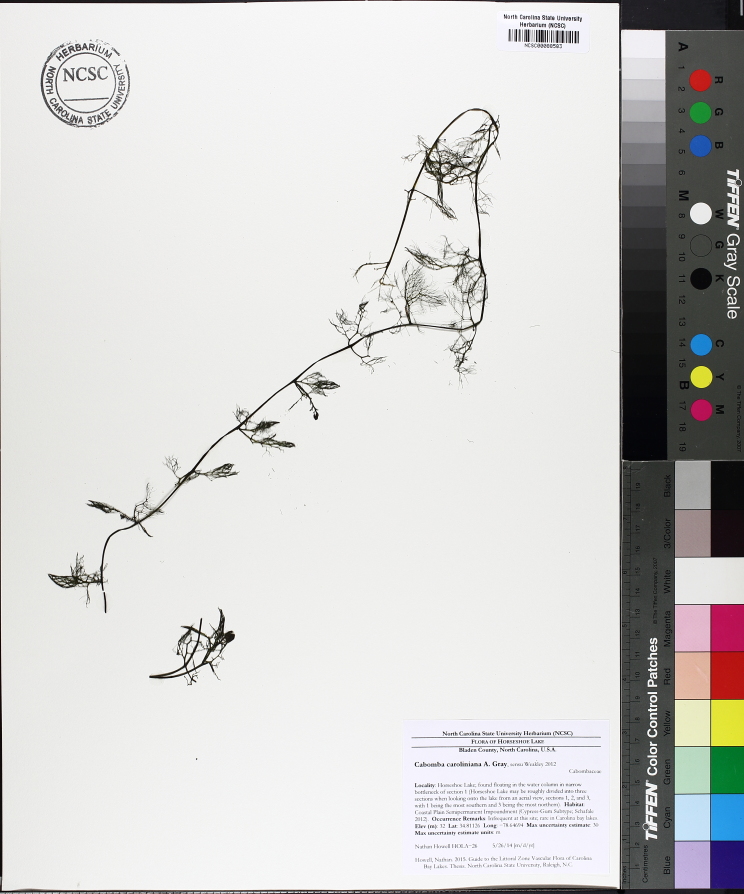
Specimen: *Howell HOLA-26* (NCSC)

**Figure 129b.**
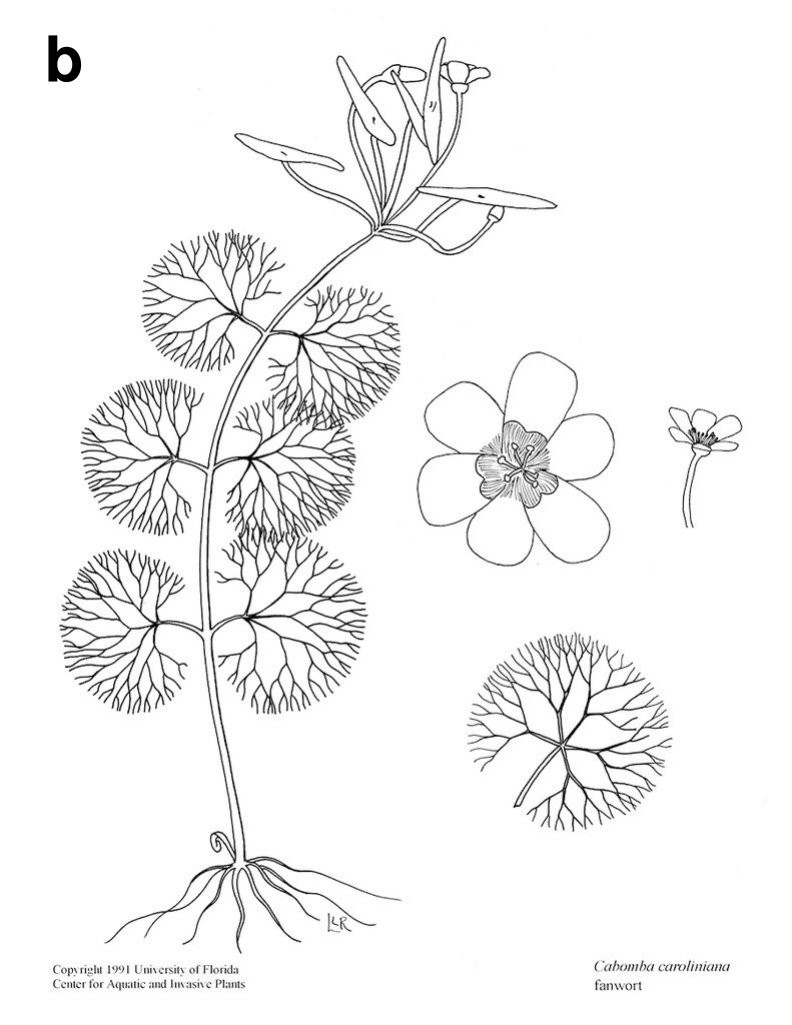
Illustration

**Figure 130a.**
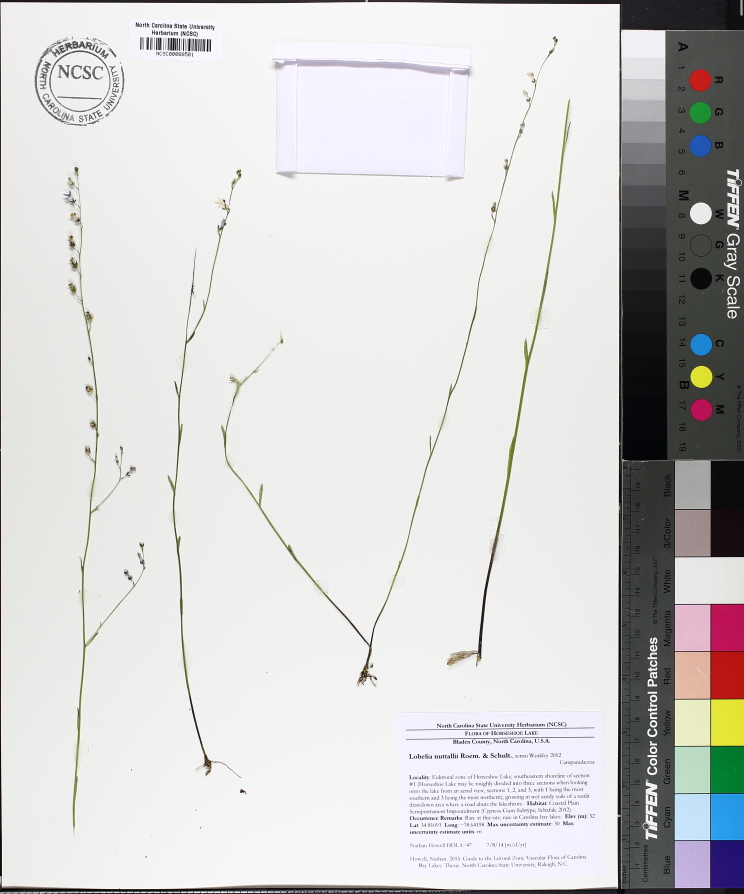
Specimen: *Howell HOLA-47* (NCSC)

**Figure 130b.**
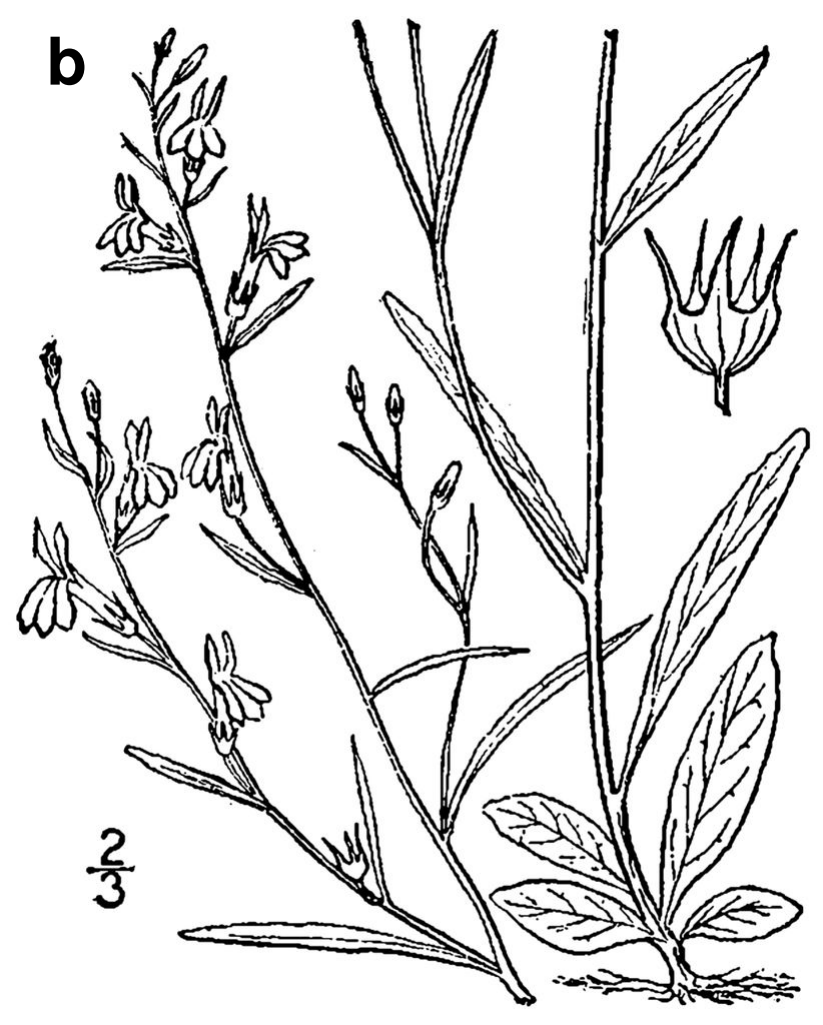
Illustration

**Figure 130c.**
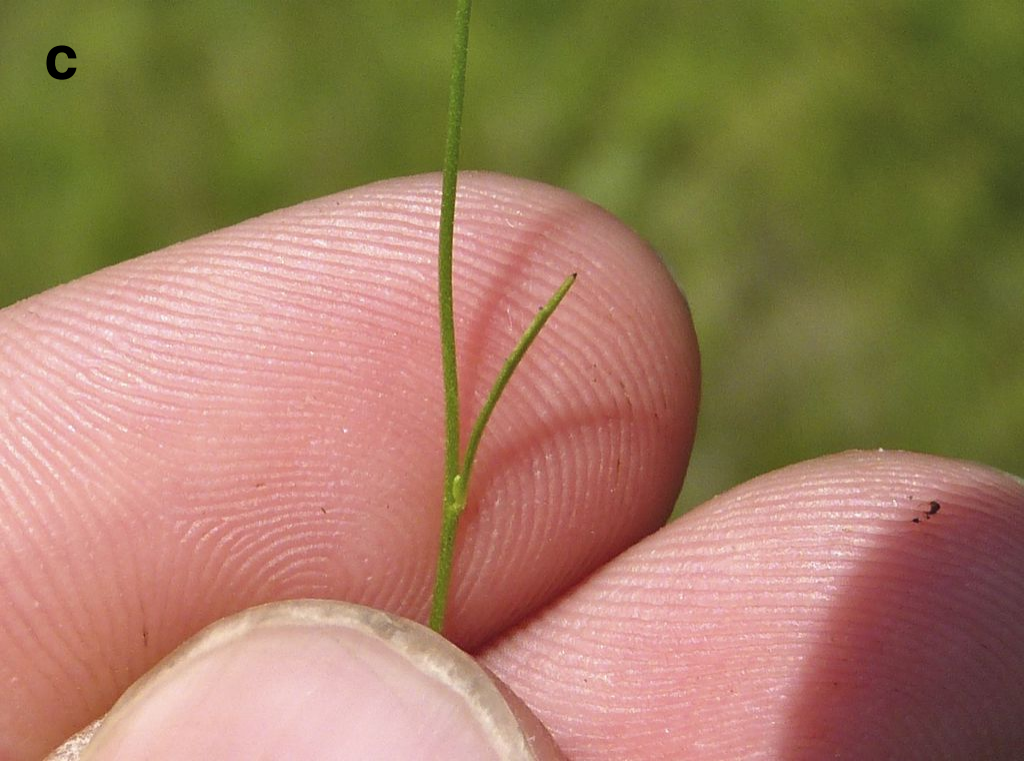
Cauline leaf

**Figure 130d.**
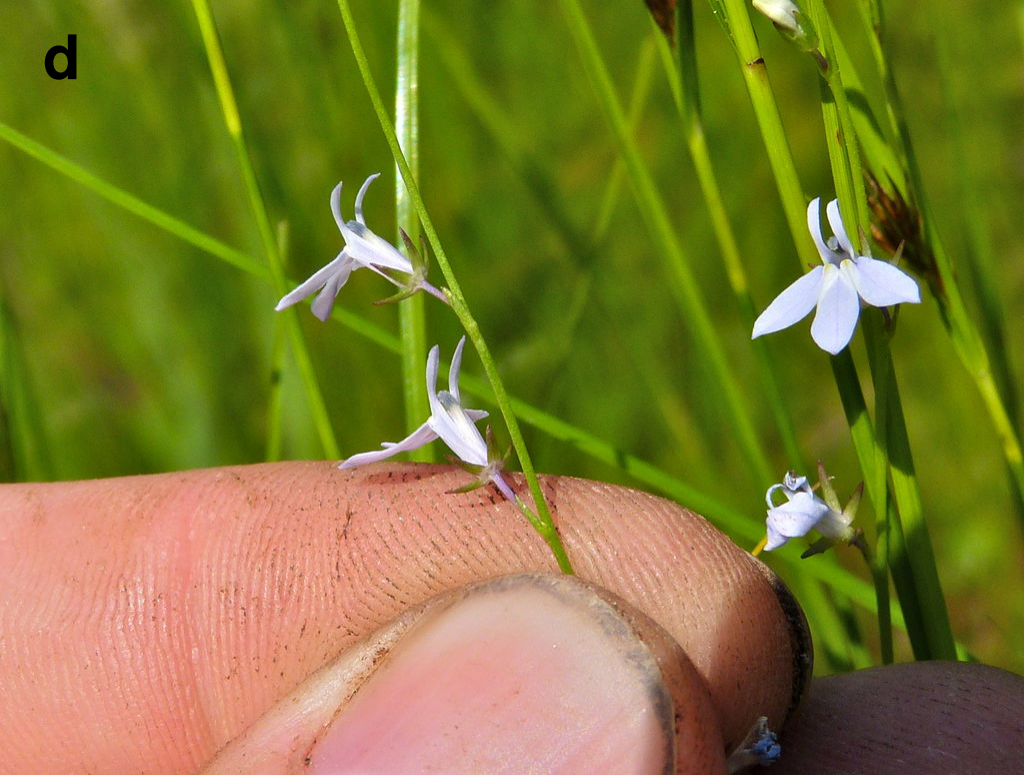
Inflorescences

**Figure 131a.**
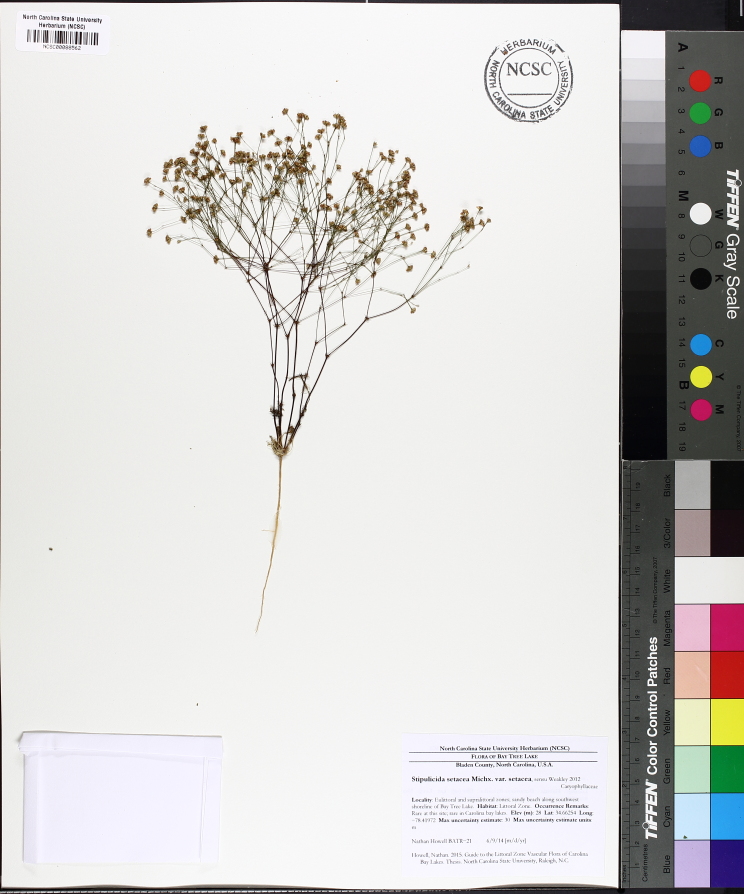
Specimen: *Howell BATR-21* (NCSC)

**Figure 131b.**
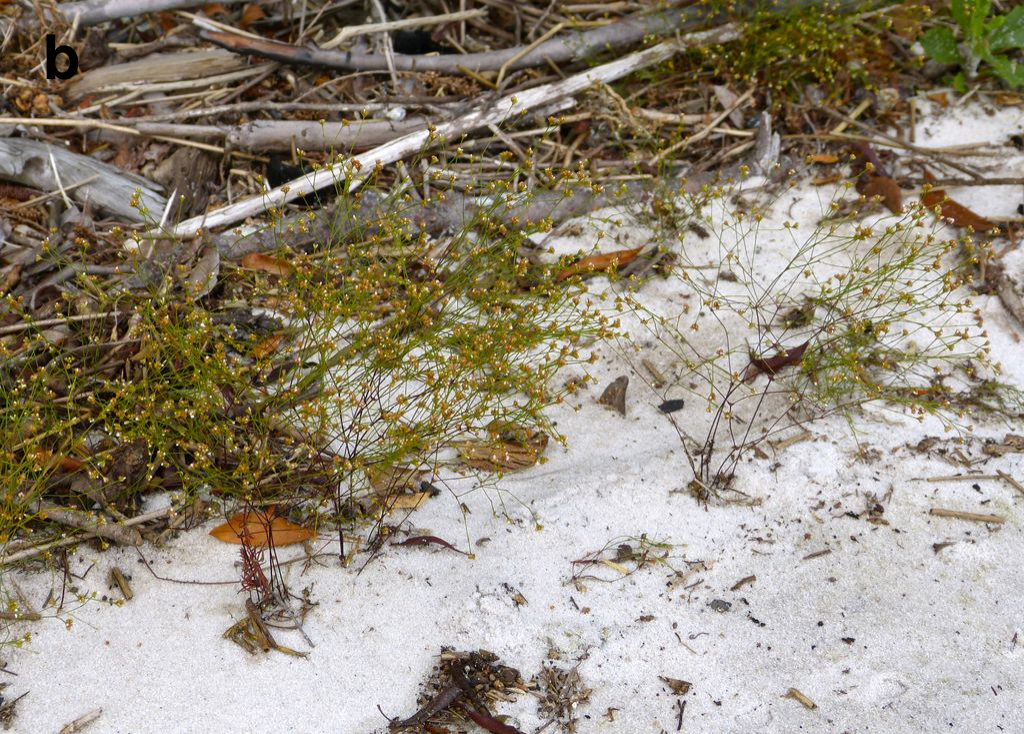
Habit

**Figure 131c.**
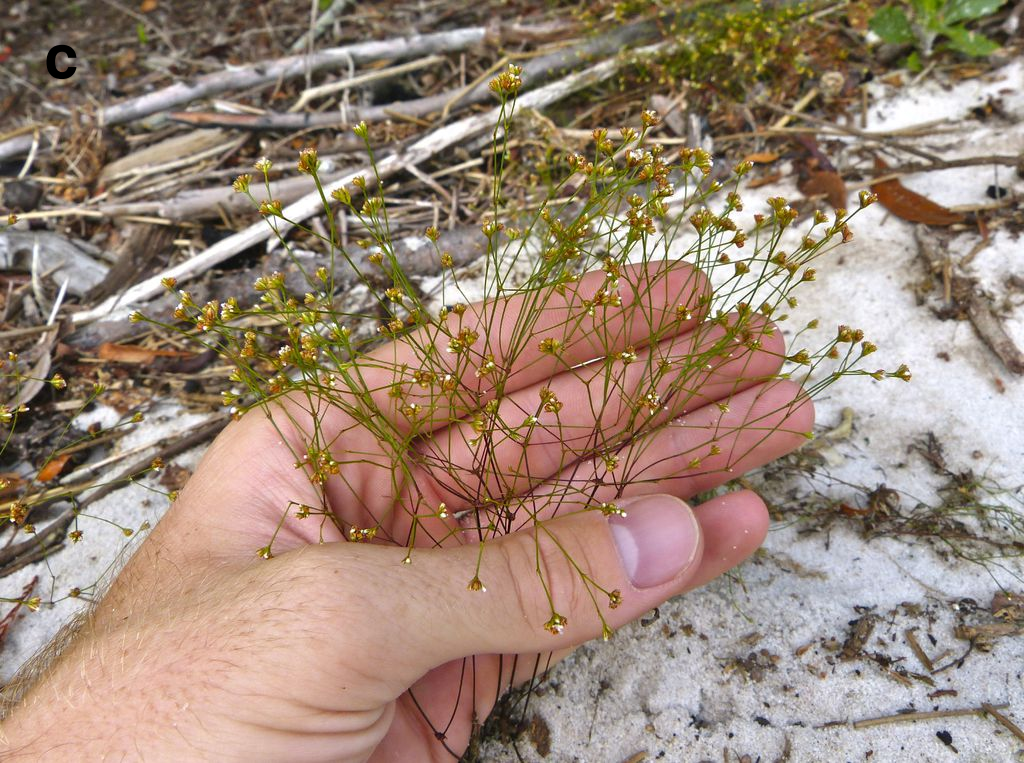
Habit

**Figure 131d.**
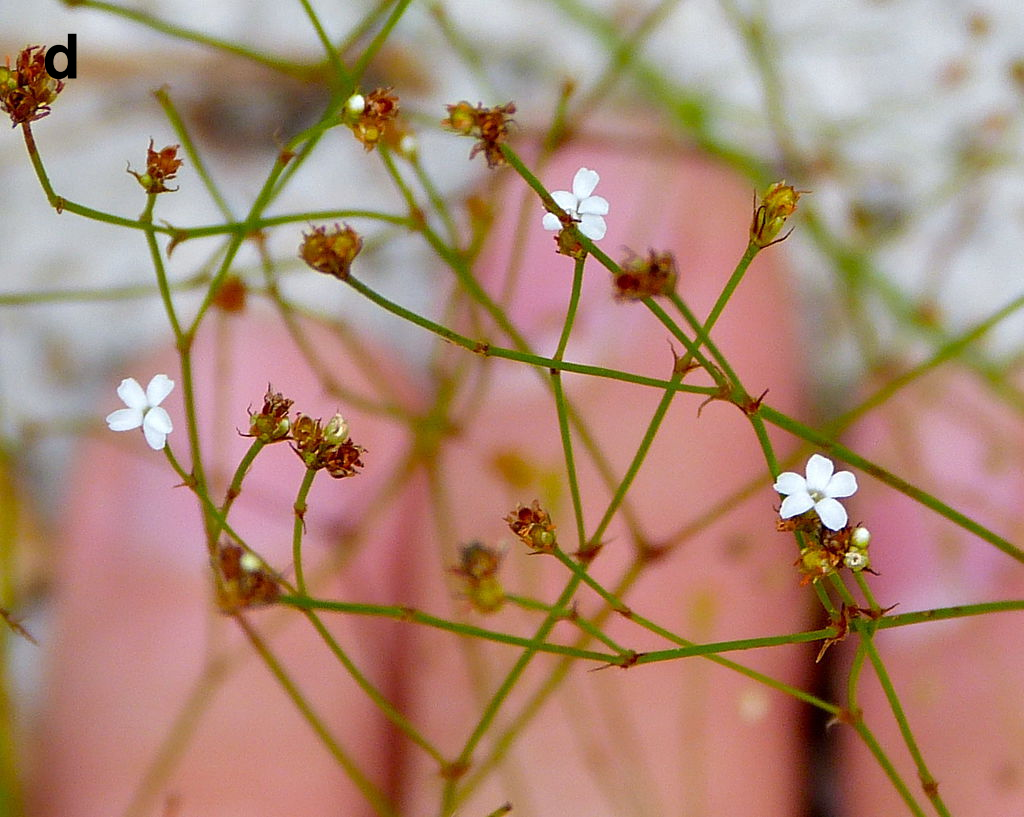
Flowers

**Figure 132a.**
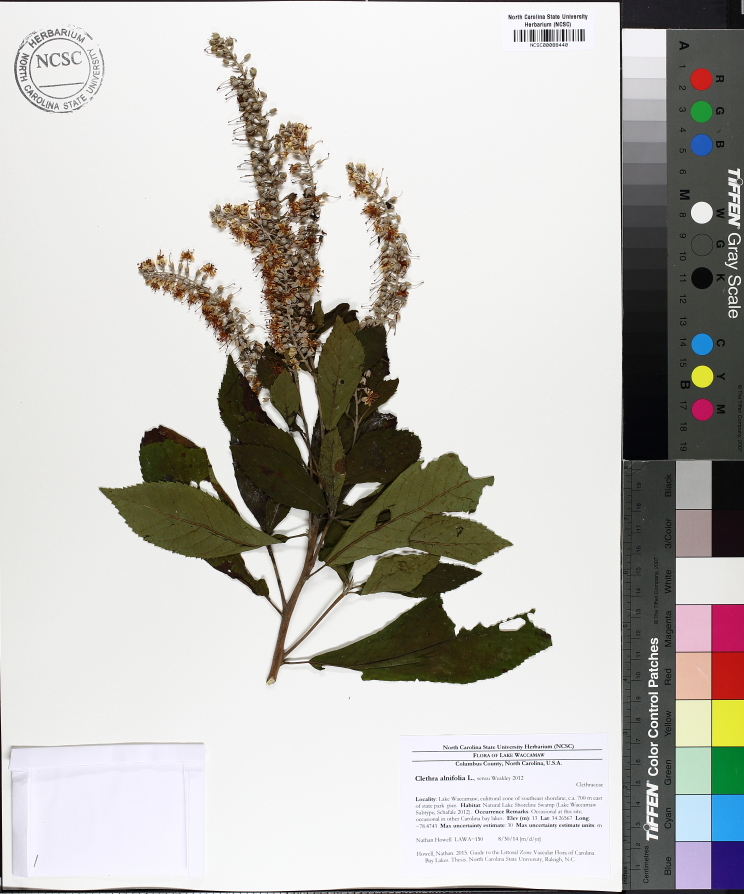
Specimen: *Howell LAWA-150* (NCSC)

**Figure 132b.**
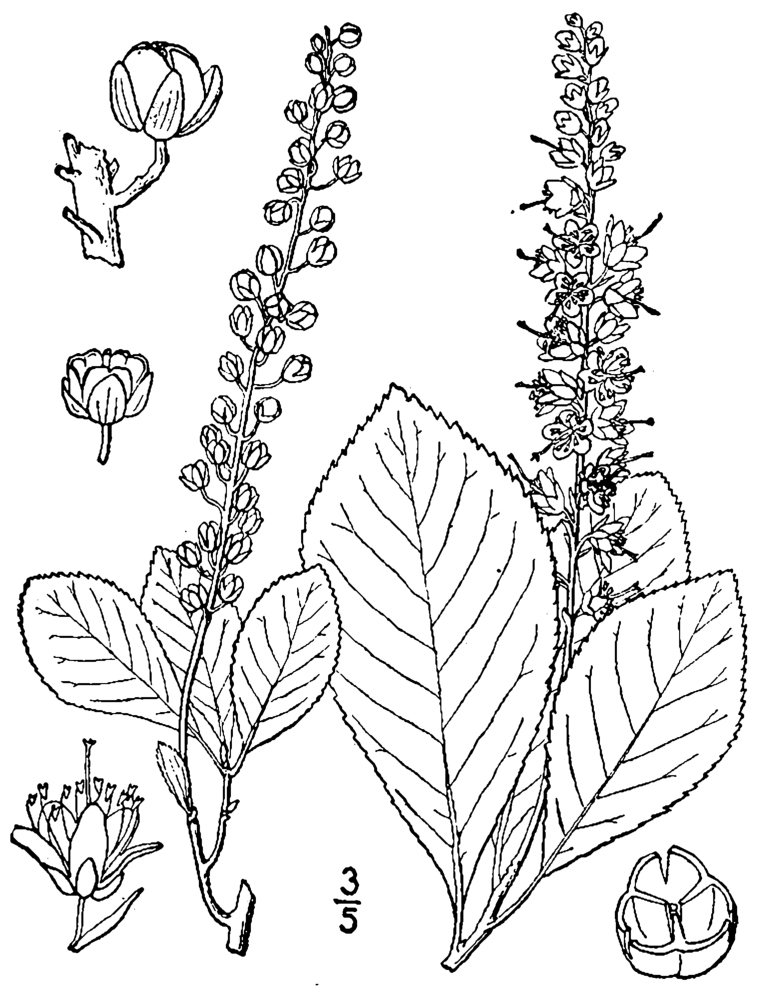
Illustration

**Figure 132c.**
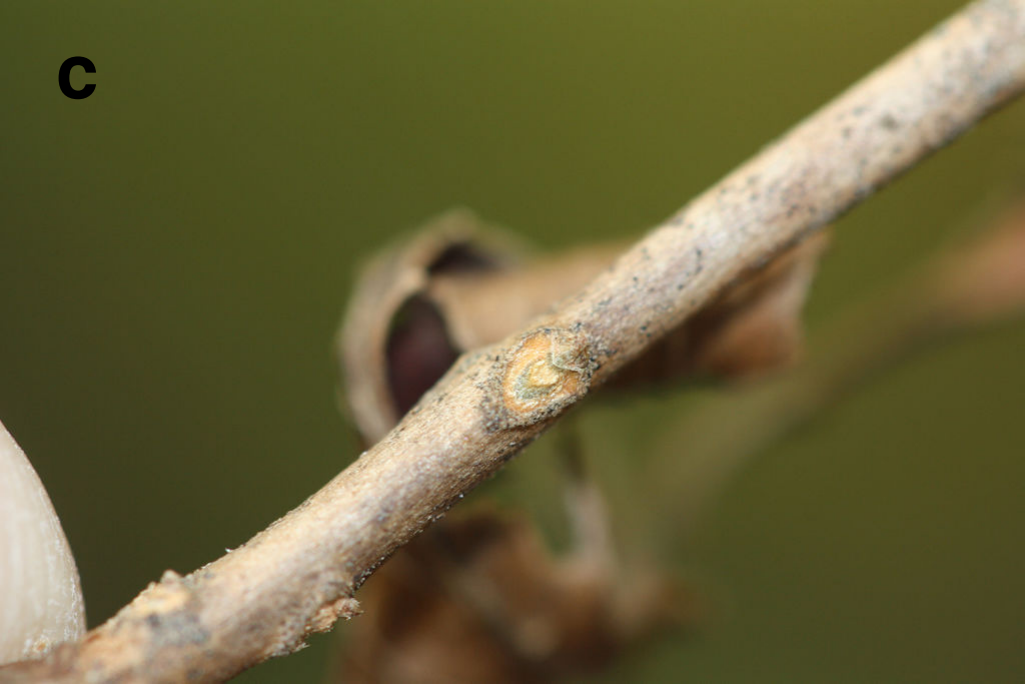
Twig, showing leaf scar

**Figure 132d.**
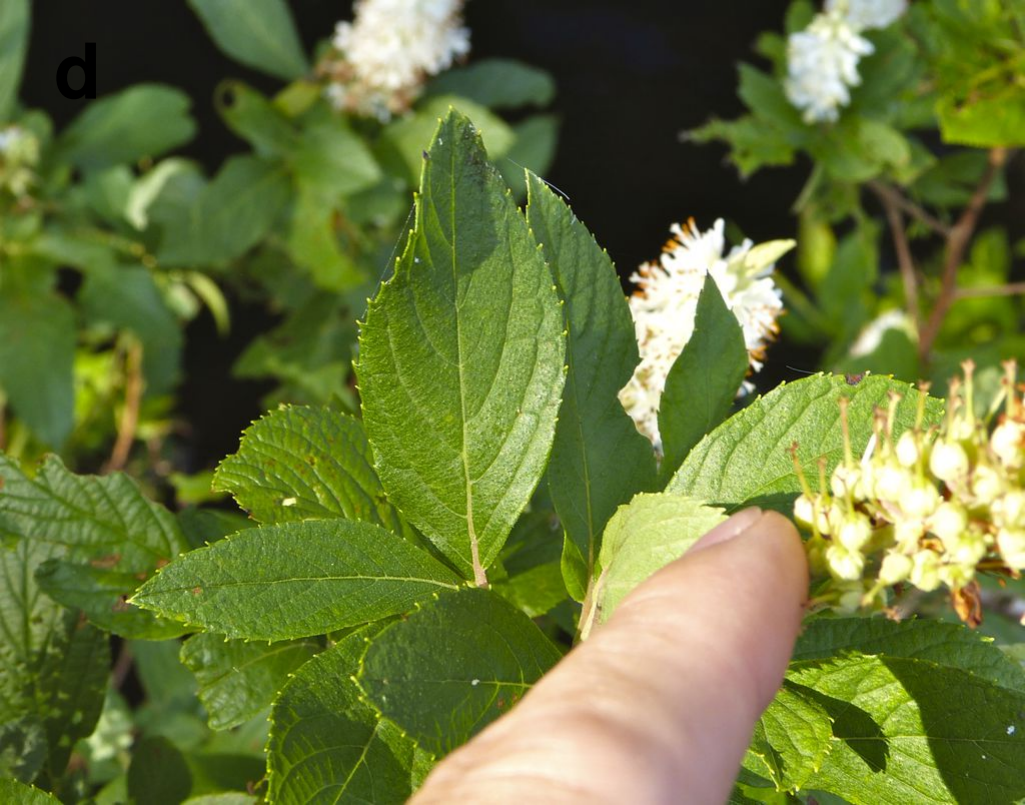
Leaves

**Figure 132e.**
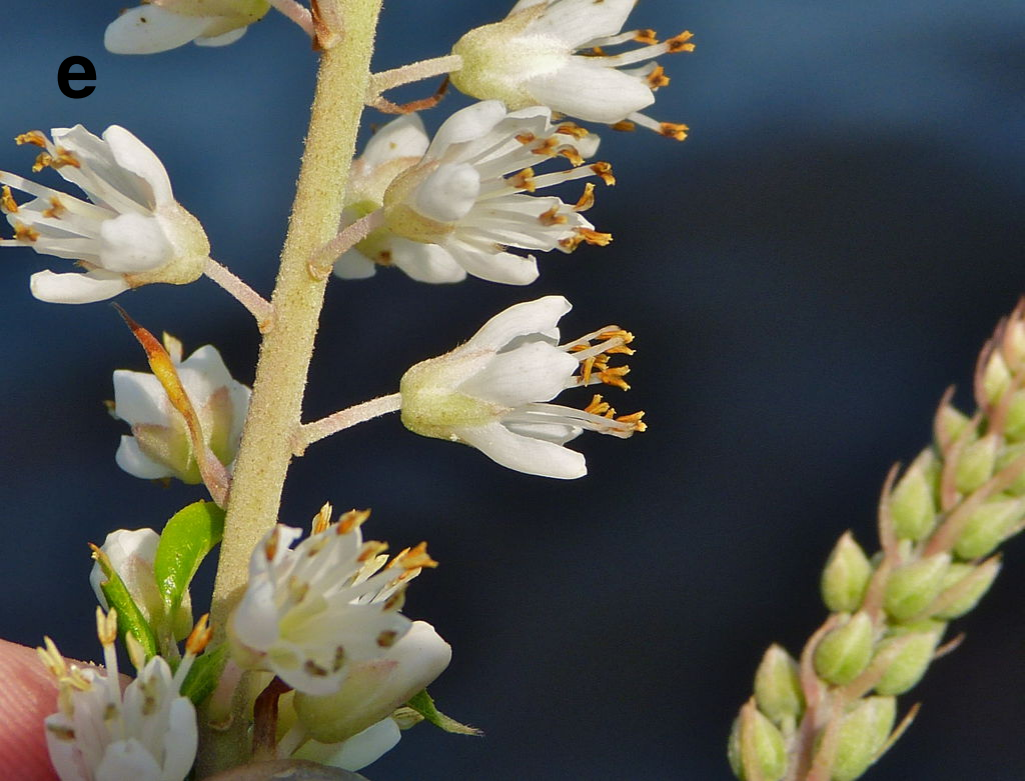
Flowers

**Figure 132f.**
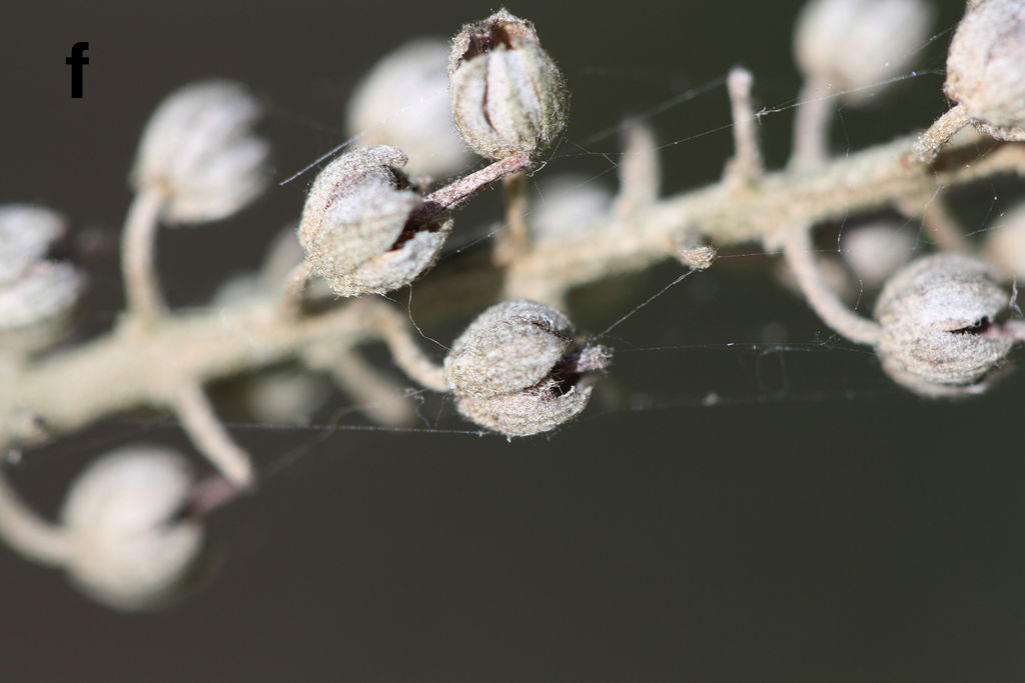
Fruits

**Figure 133a.**
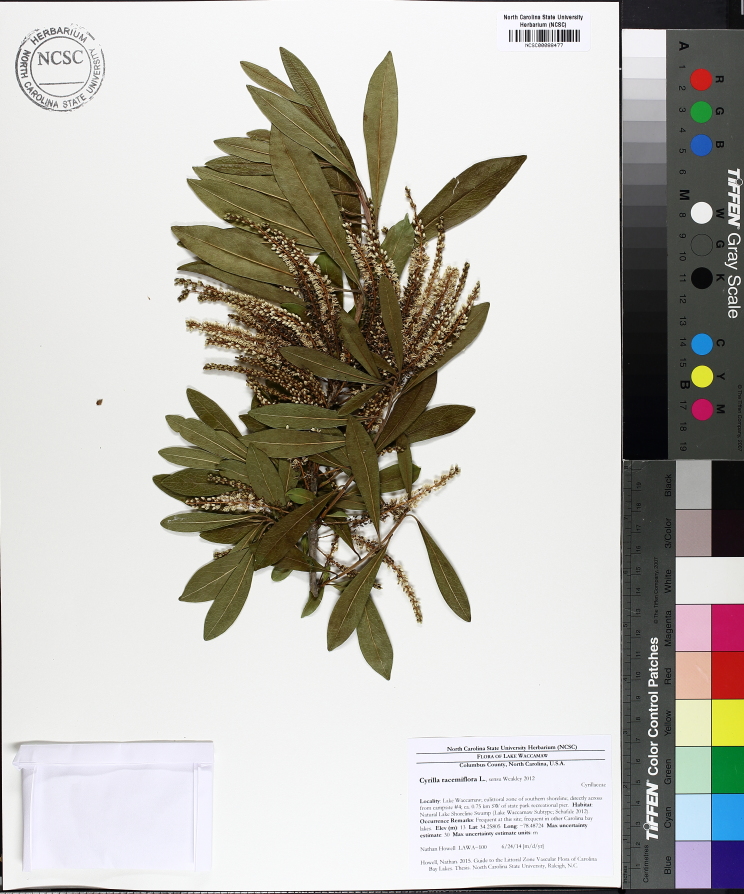
Specimen: *Howell LAWA-100* (NCSC)

**Figure 133b.**
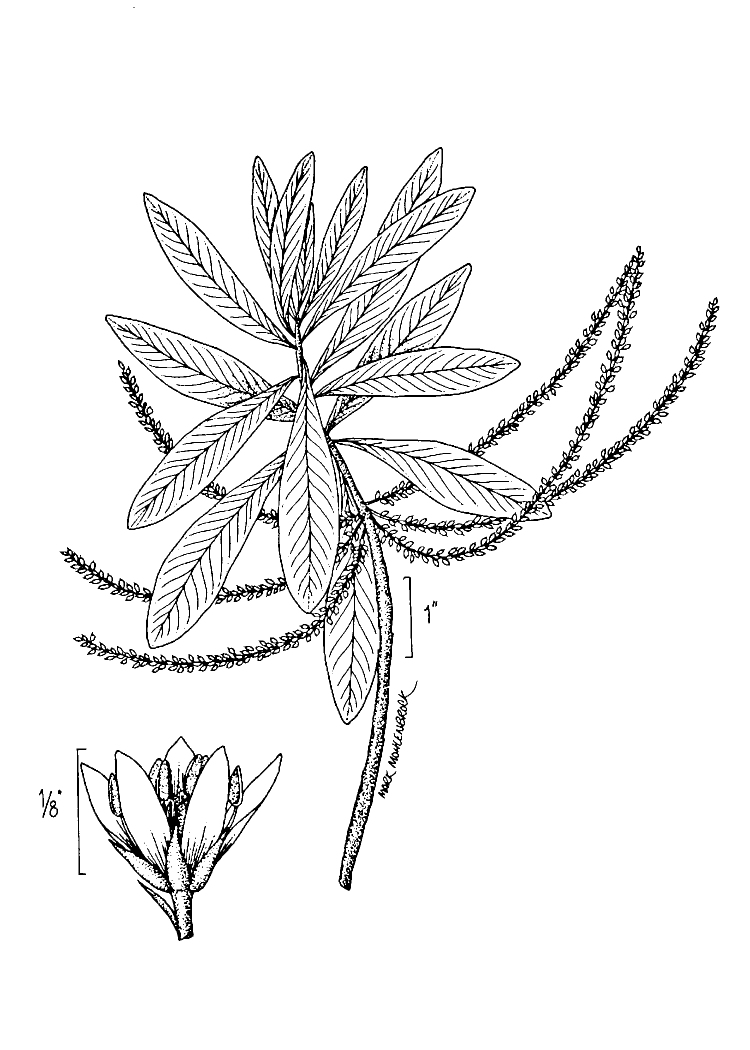
Illustration

**Figure 133c.**
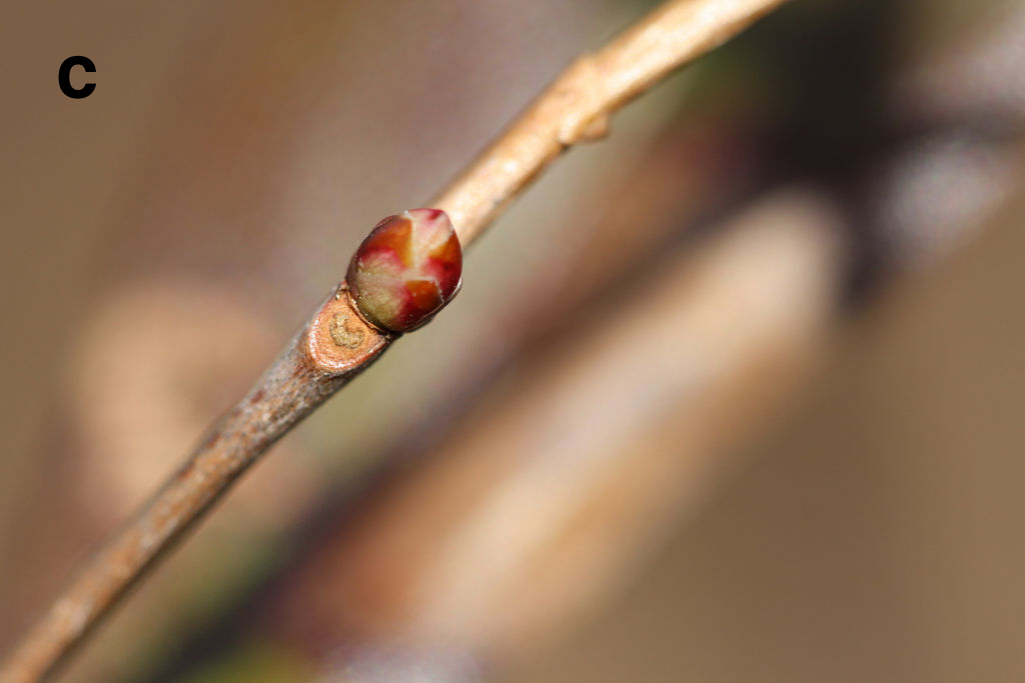
Twig, leaf scar, and bud

**Figure 133d.**
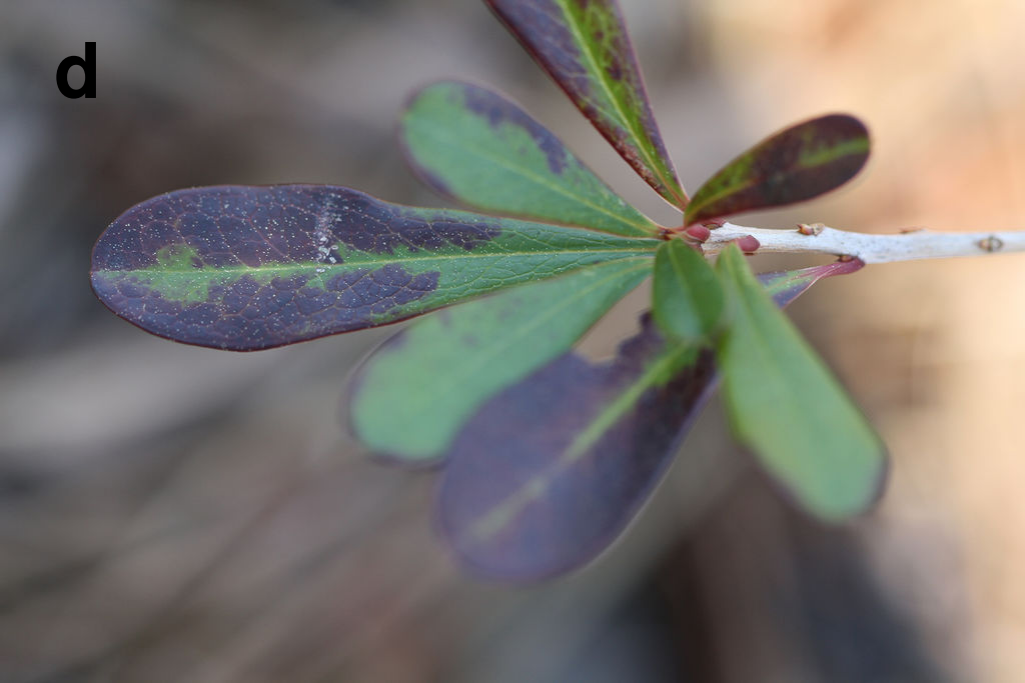
Leaves

**Figure 133e.**
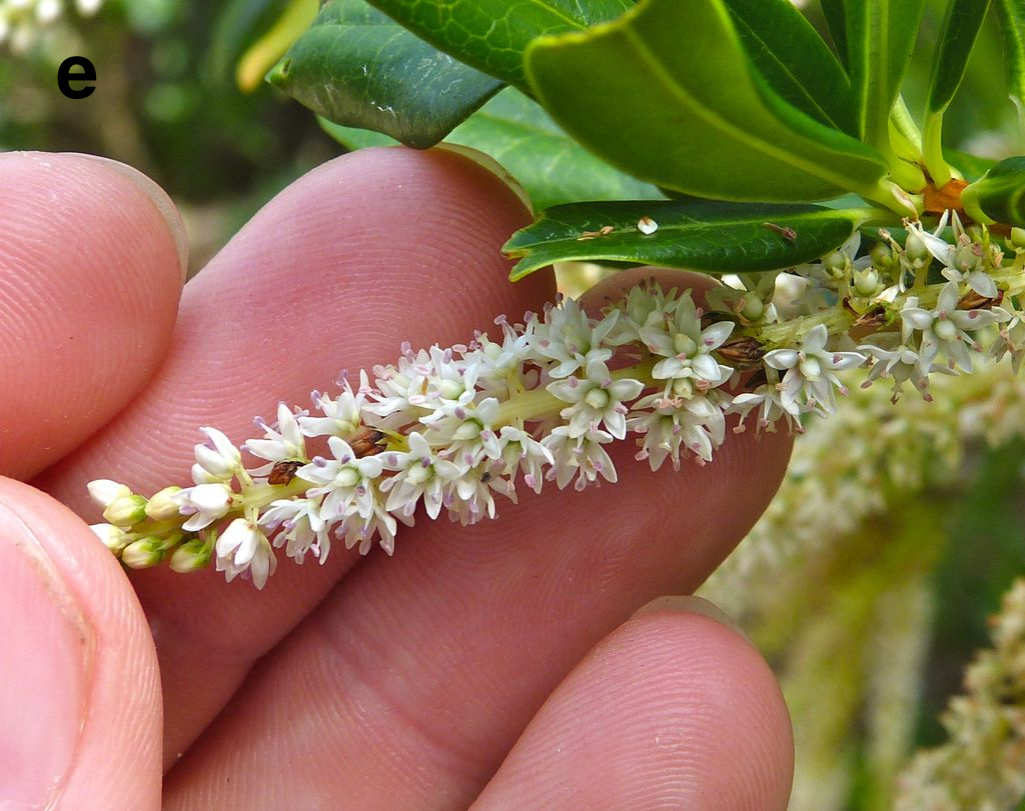
Inflorescence

**Figure 133f.**
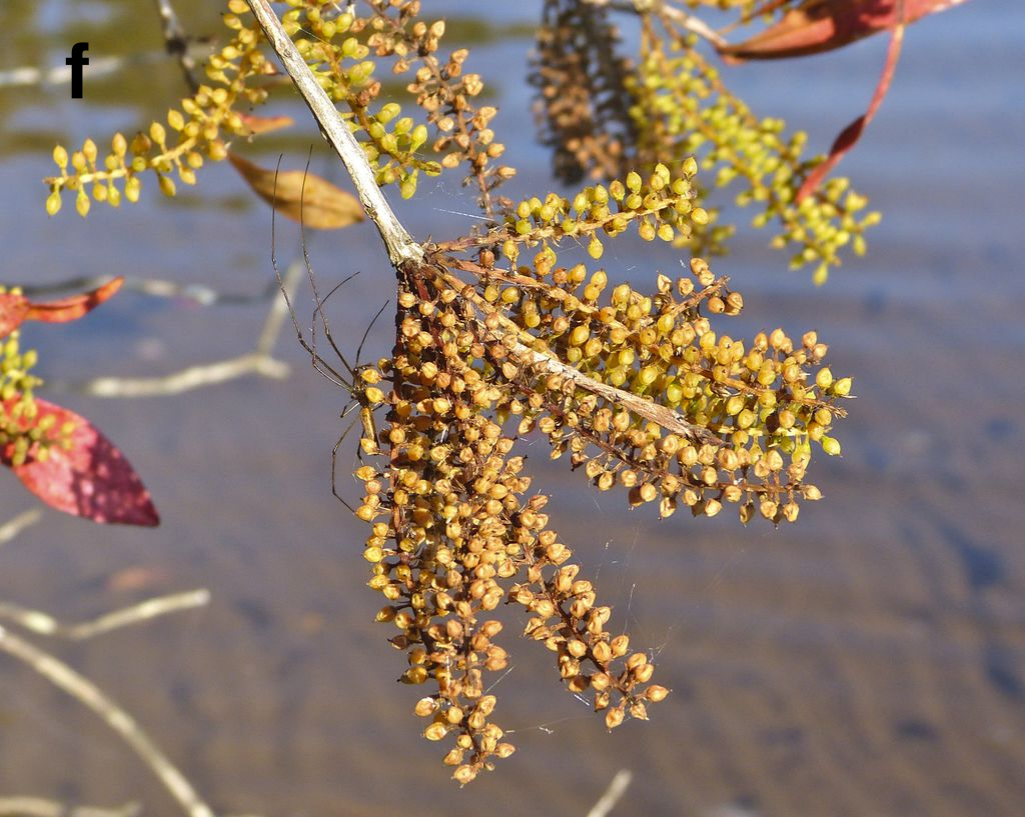
Infructescence

**Figure 134a.**
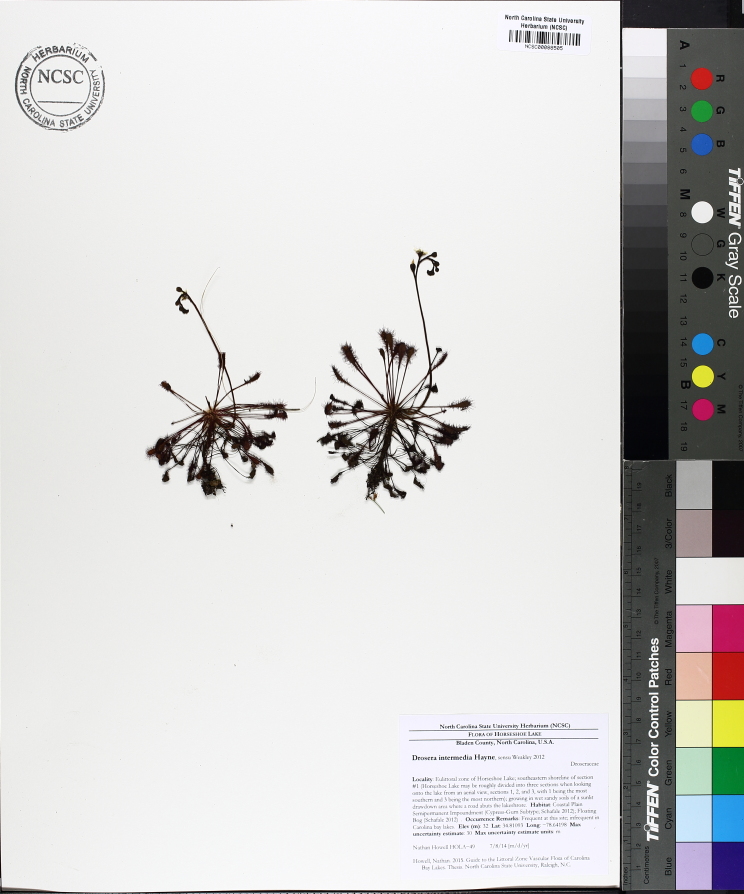
Specimen: *Howell HOLA-49* (NCSC)

**Figure 134b.**
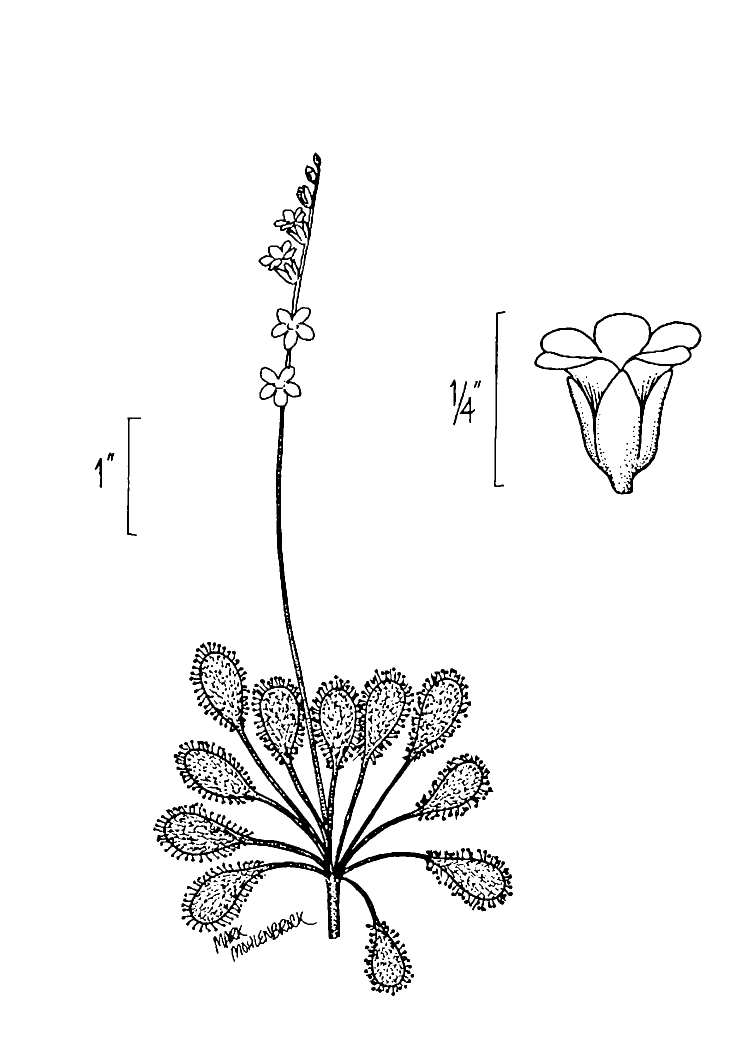
Illustration

**Figure 134c.**
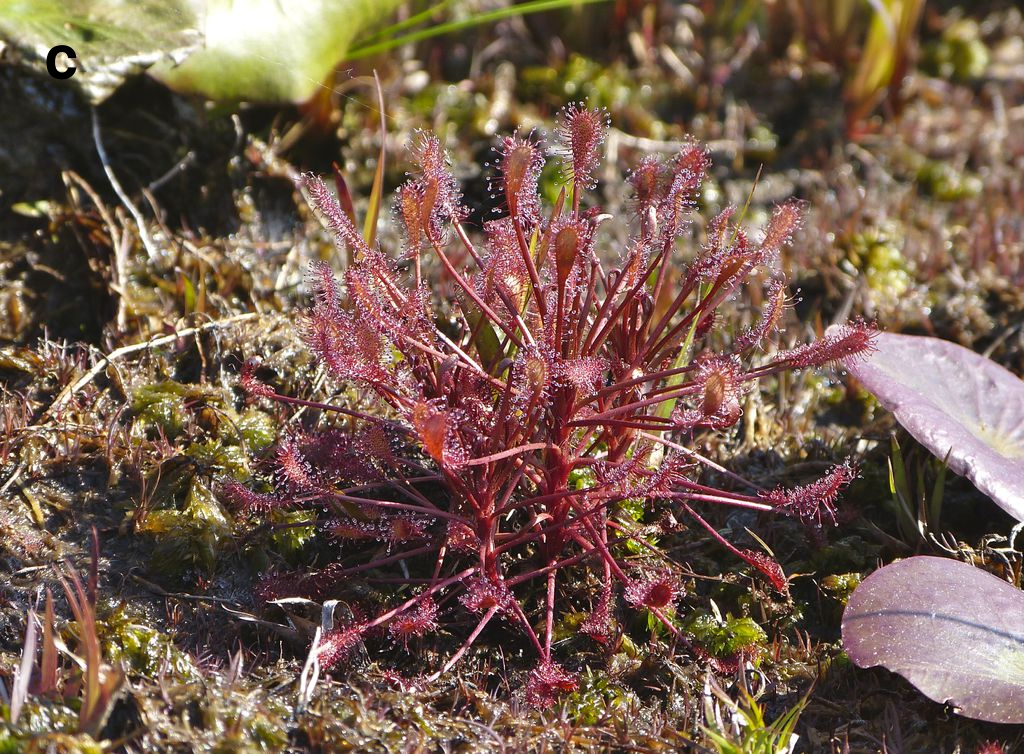
Habit

**Figure 134d.**
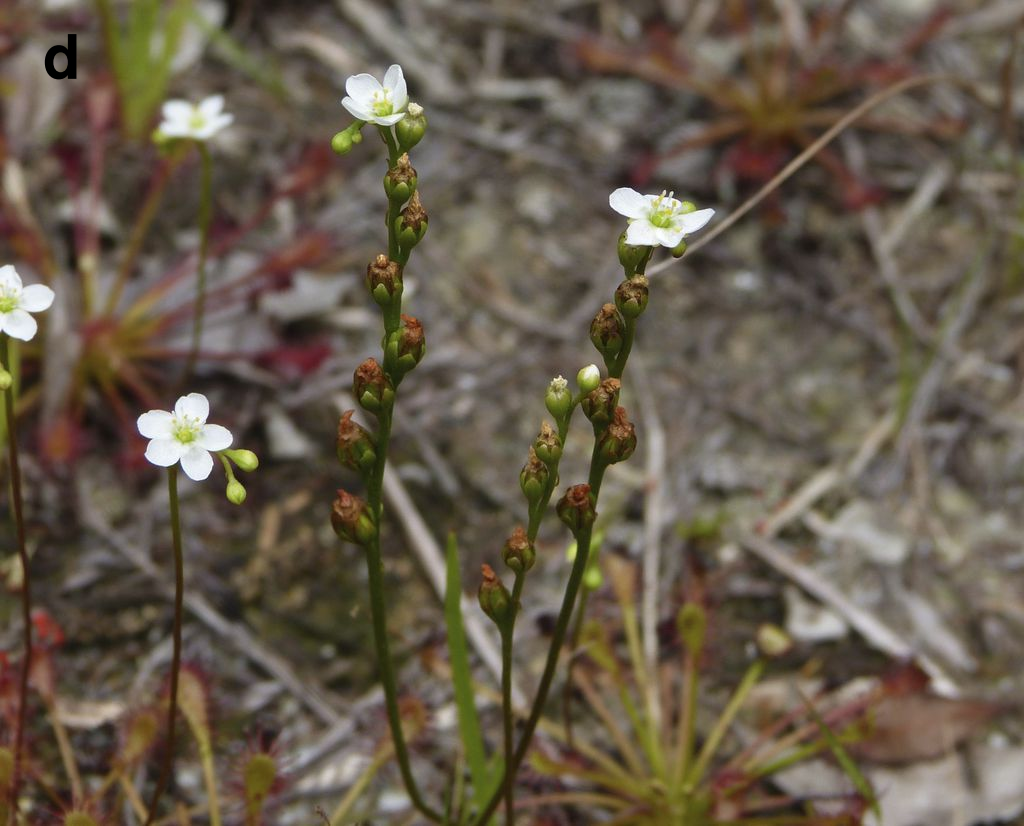
Inflorescences

**Figure 135a.**
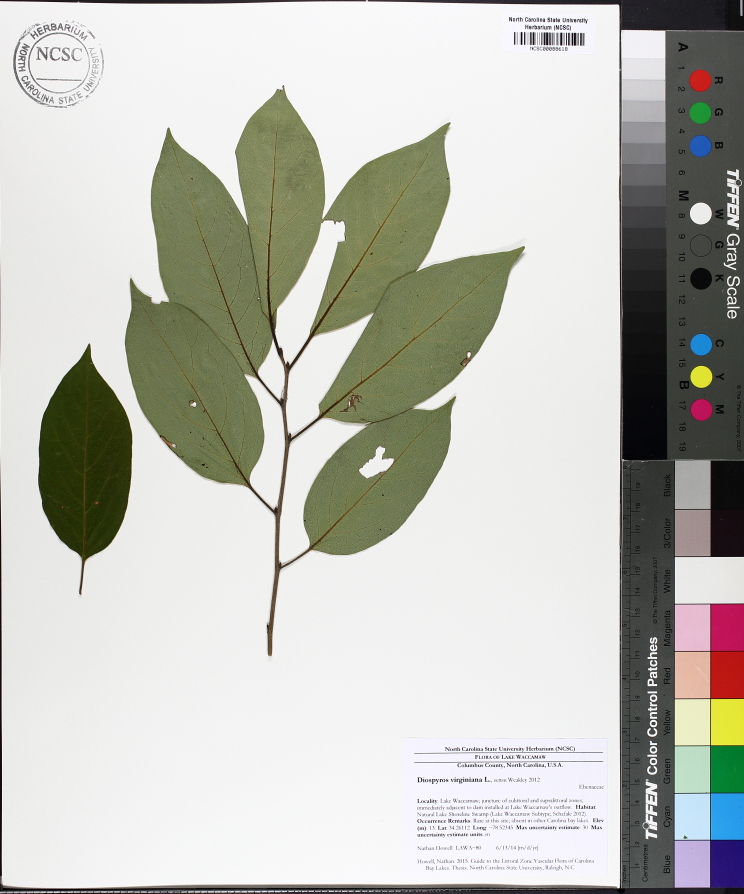
Specimen: *Howell LAWA-80* (NCSC)

**Figure 135b.**
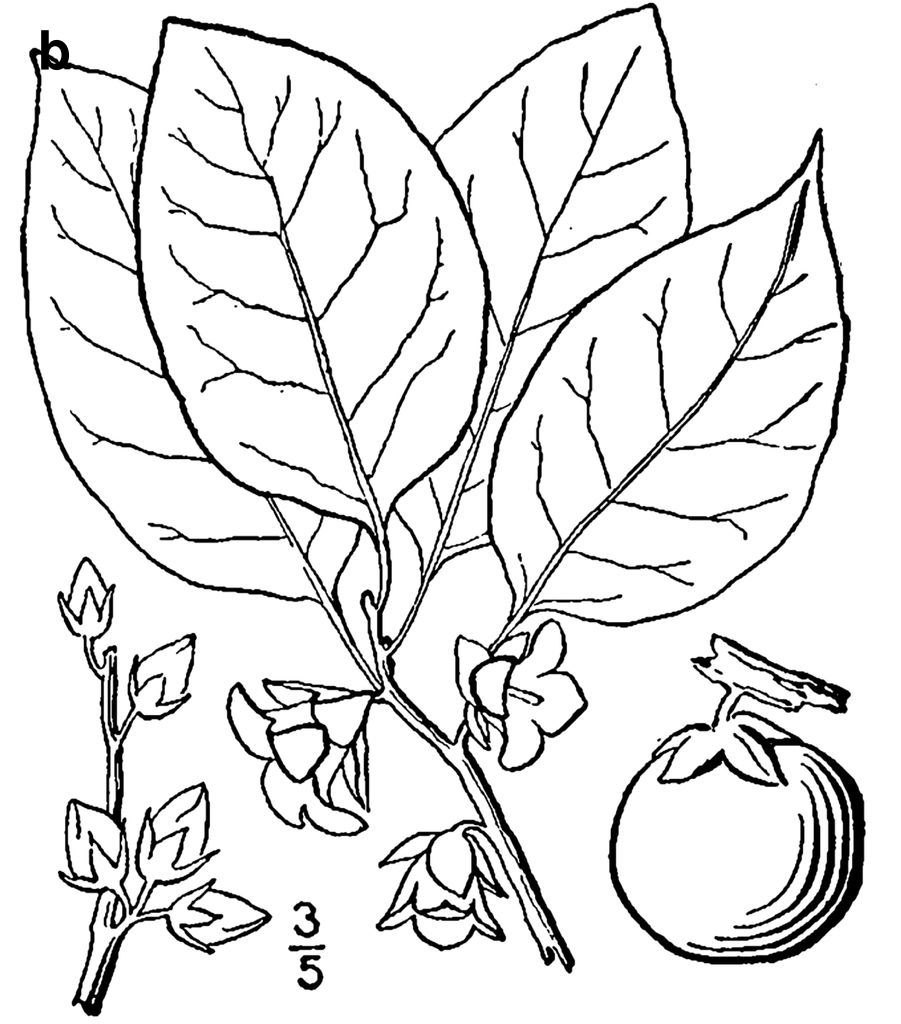
Illustration

**Figure 135c.**
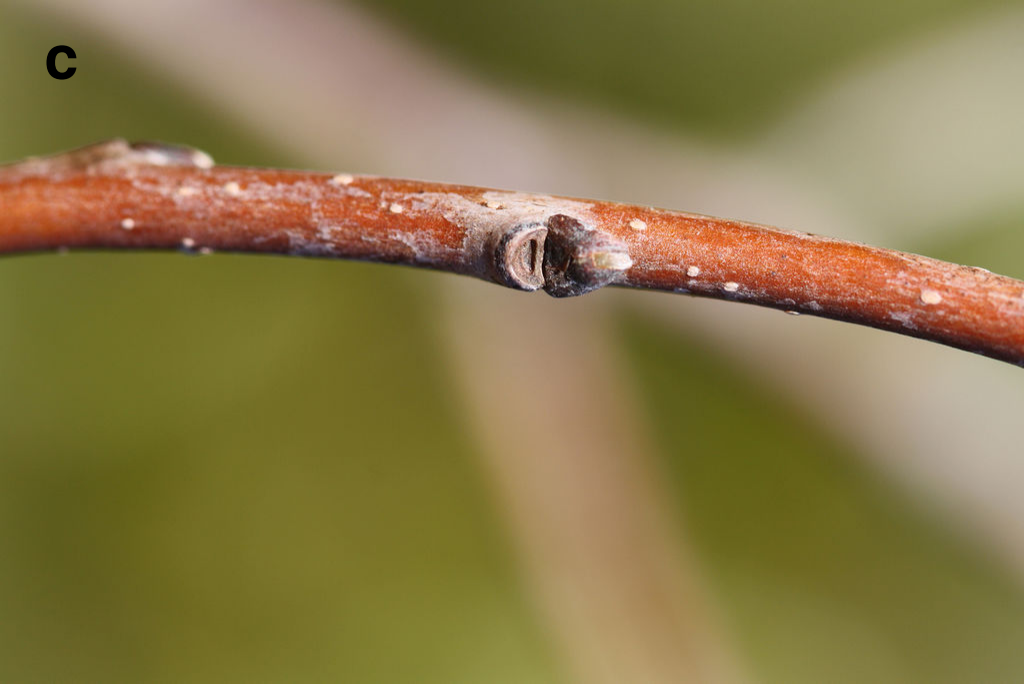
Twig, showing the typical dark bud and a single vascular bundle scar in the leaf scar

**Figure 135d.**
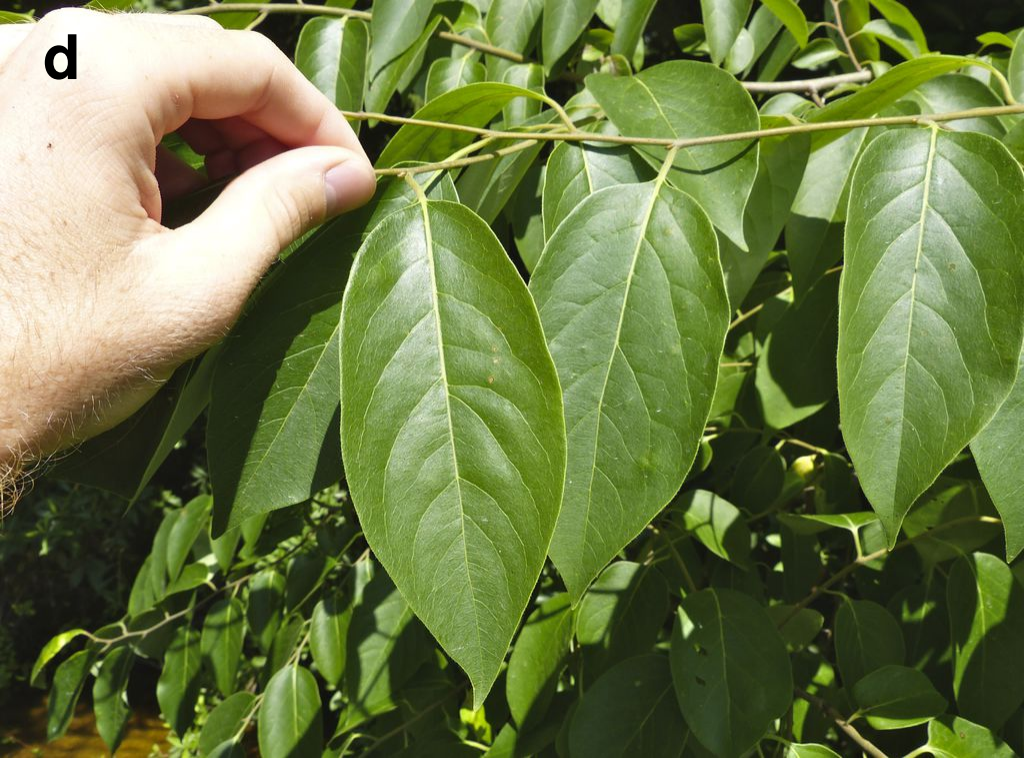
Leaves

**Figure 135e.**
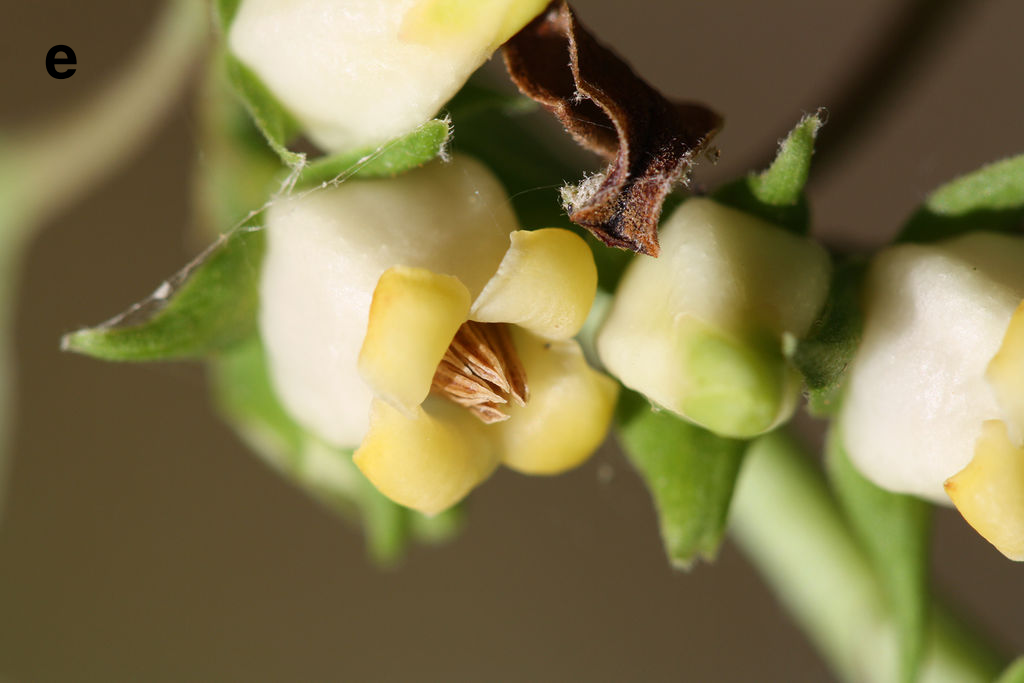
Flowers

**Figure 135f.**
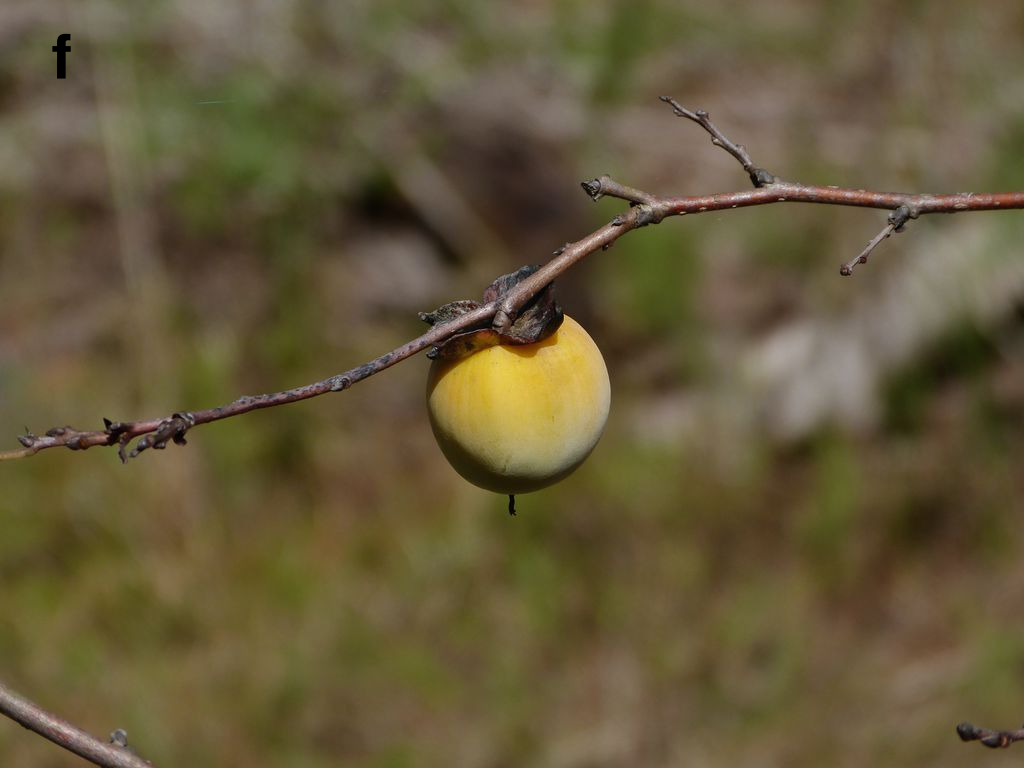
Fruit

**Figure 136a.**
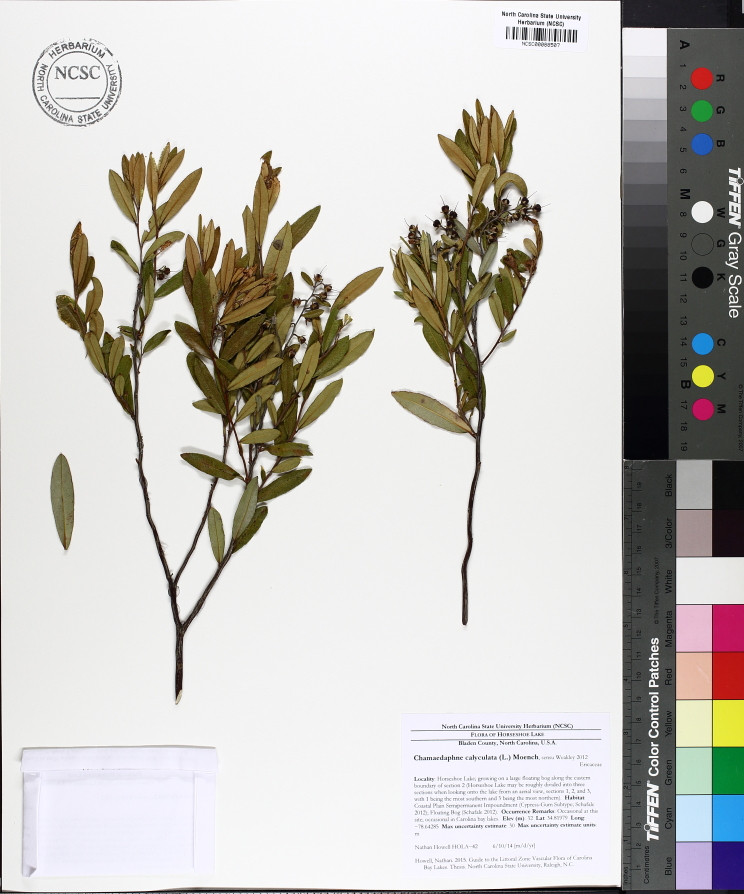
Specimen: *Howell HOLA-42* (NCSC)

**Figure 136b.**
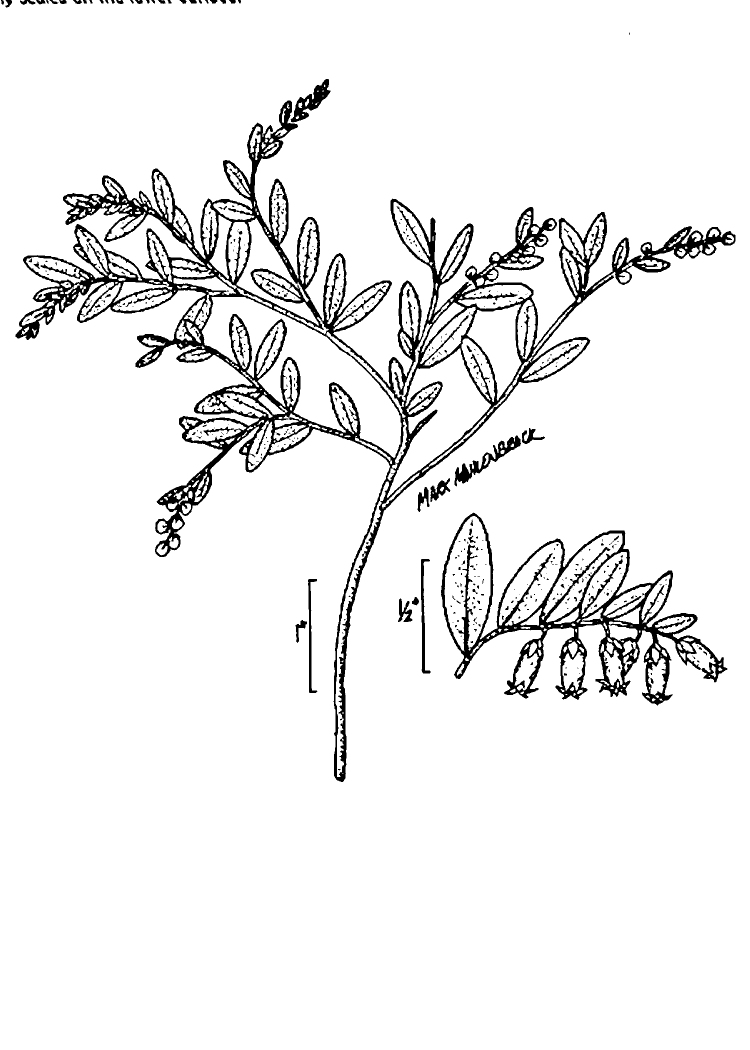
Illustration

**Figure 136c.**
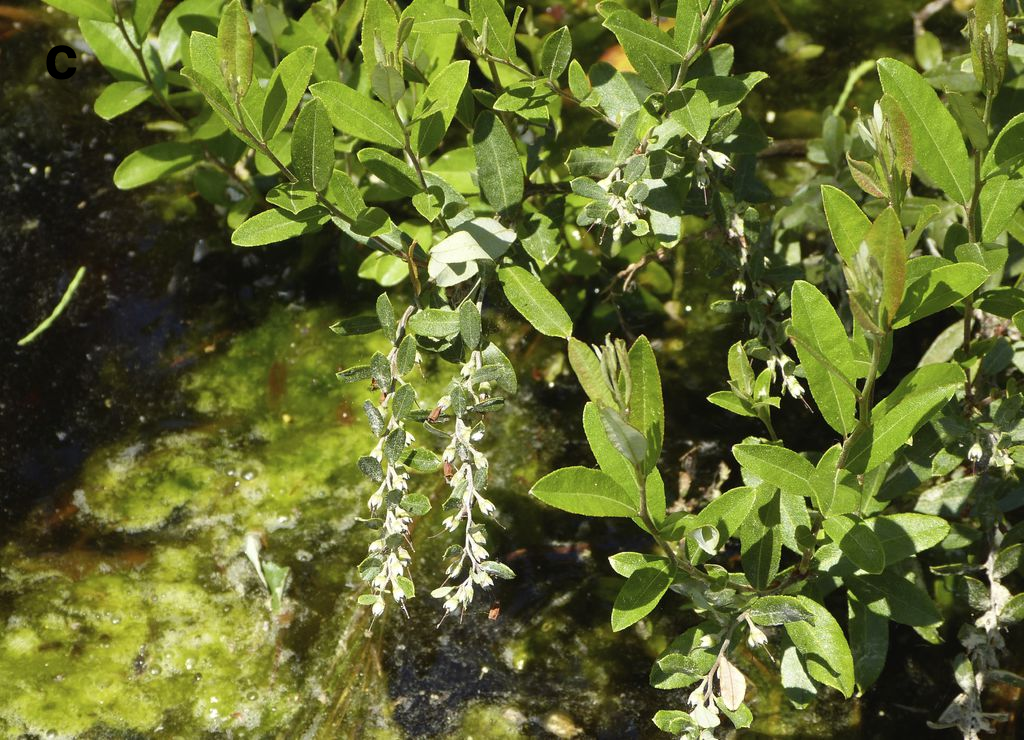
Inflorescences

**Figure 136d.**
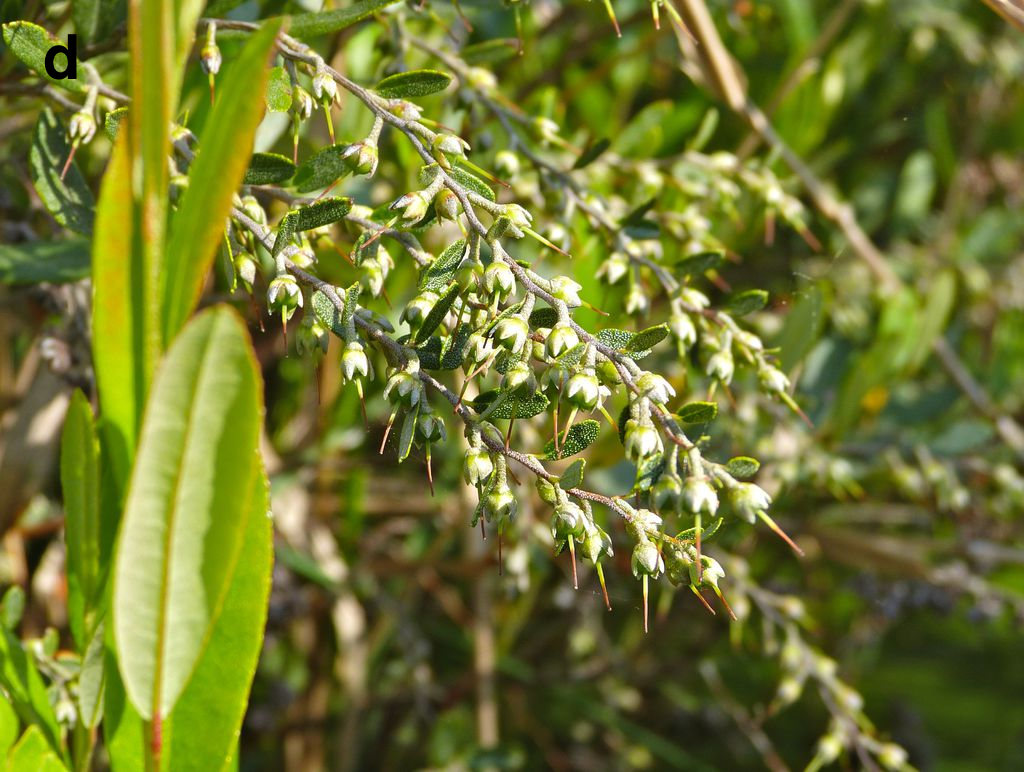
Inflorescences

**Figure 137a.**
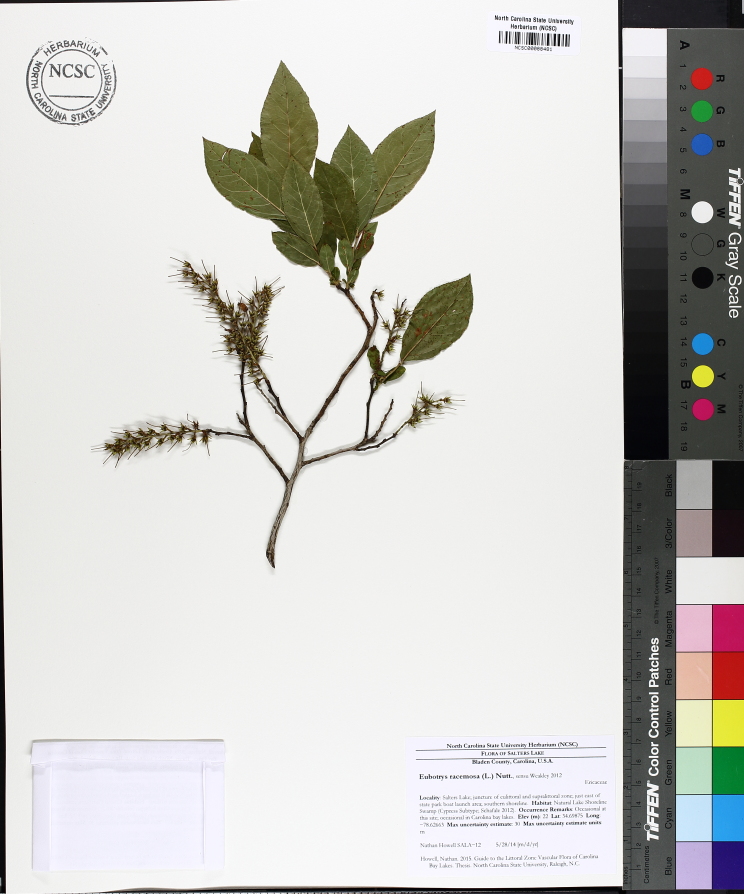
Specimen:*Howell SALA-12* (NCSC)

**Figure 137b.**
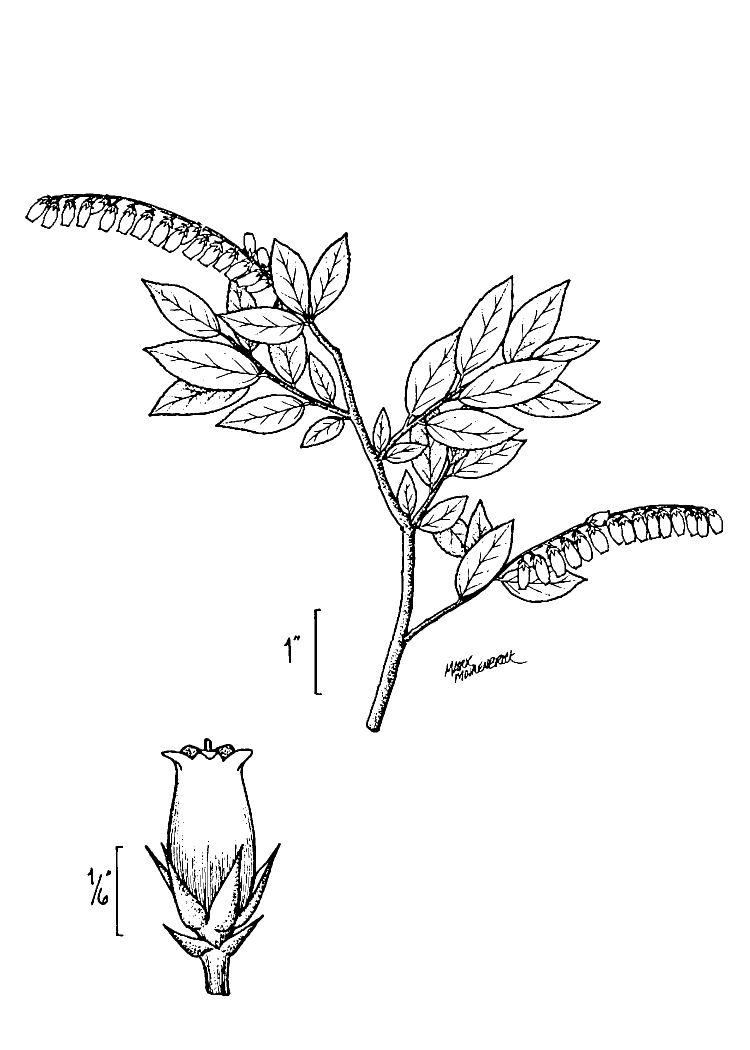
Illustration

**Figure 137c.**
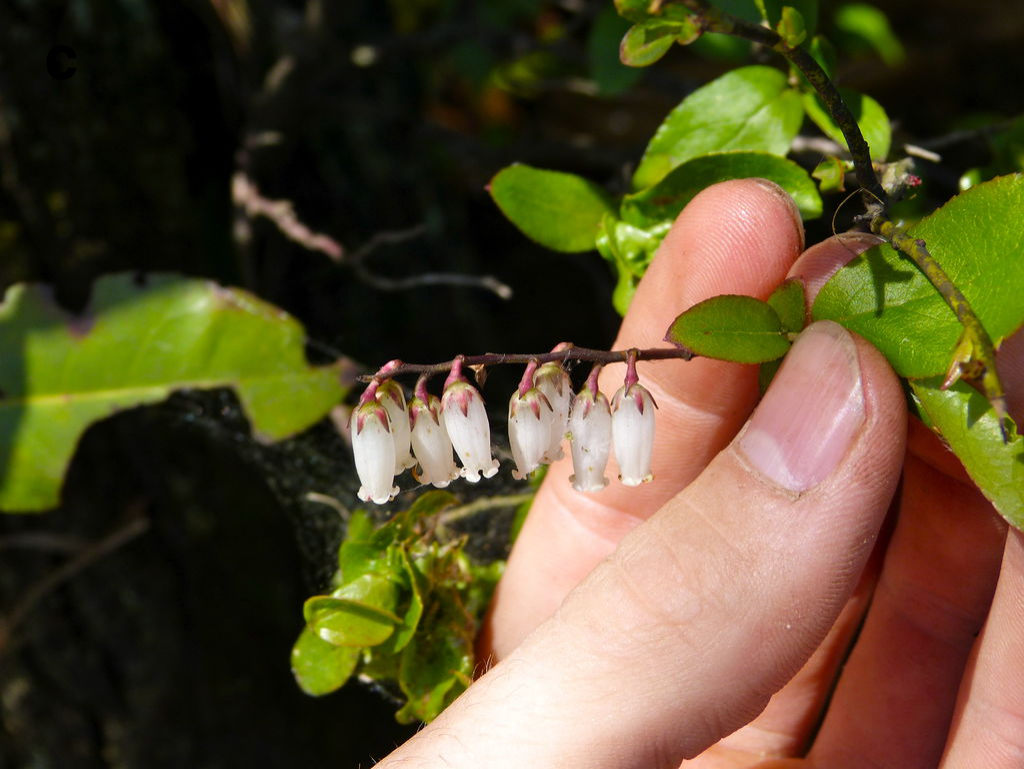
Inflorescence

**Figure 137d.**
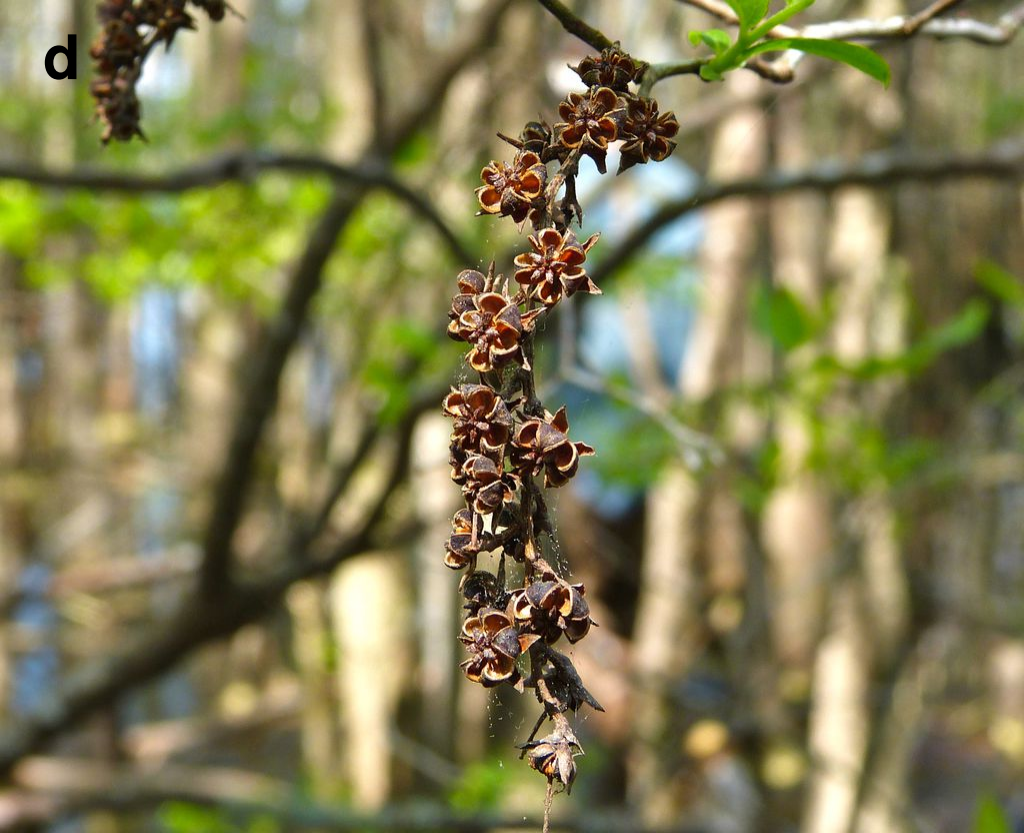
Infructescence

**Figure 138a.**
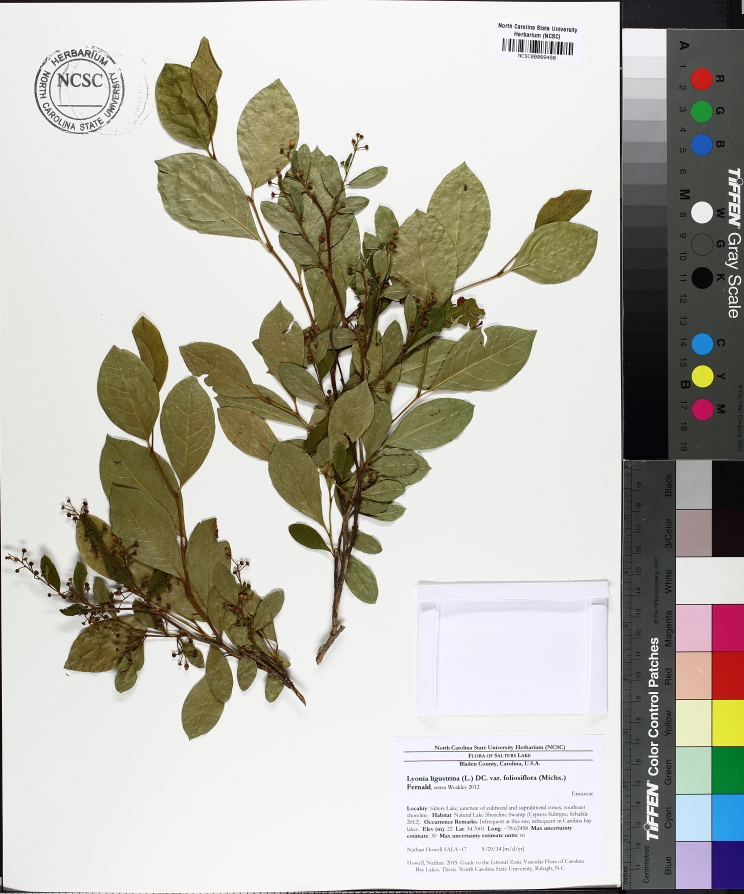
Specimen: *Howell SALA-17* (NCSC)

**Figure 138b.**
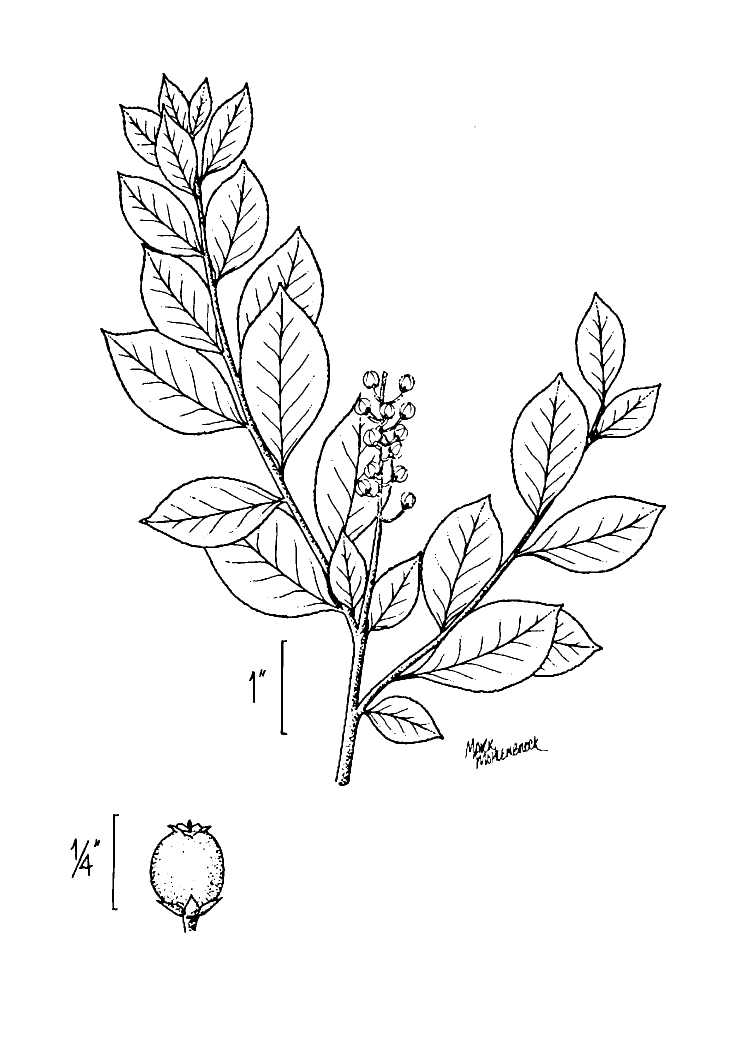
Illustration

**Figure 138c.**
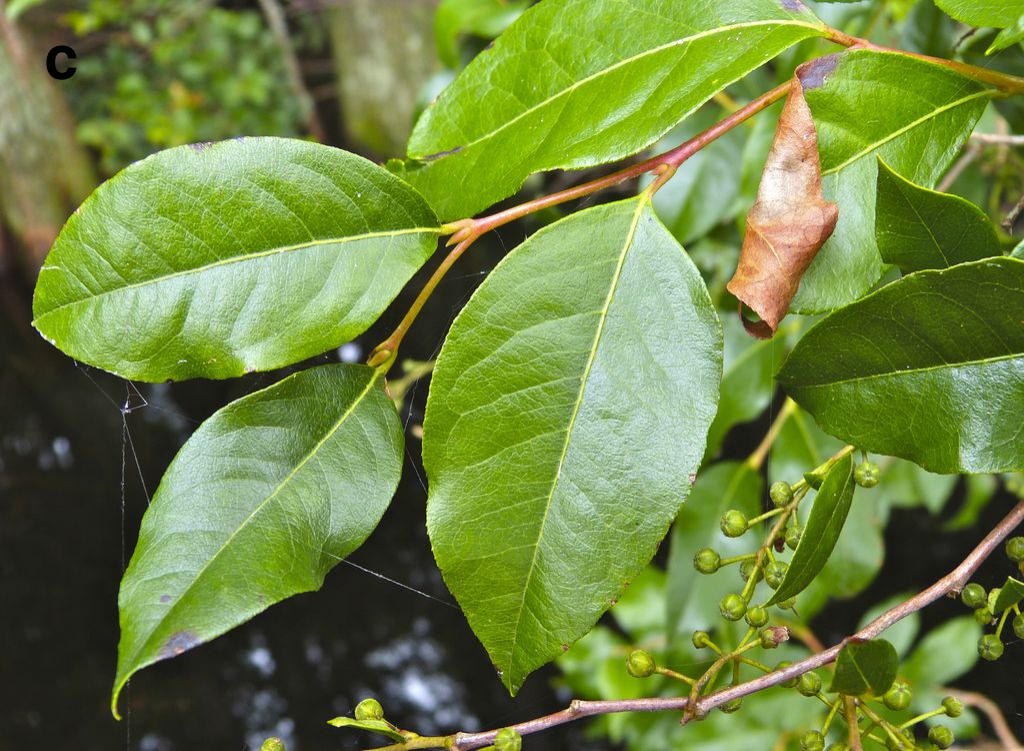
Leaves

**Figure 138d.**
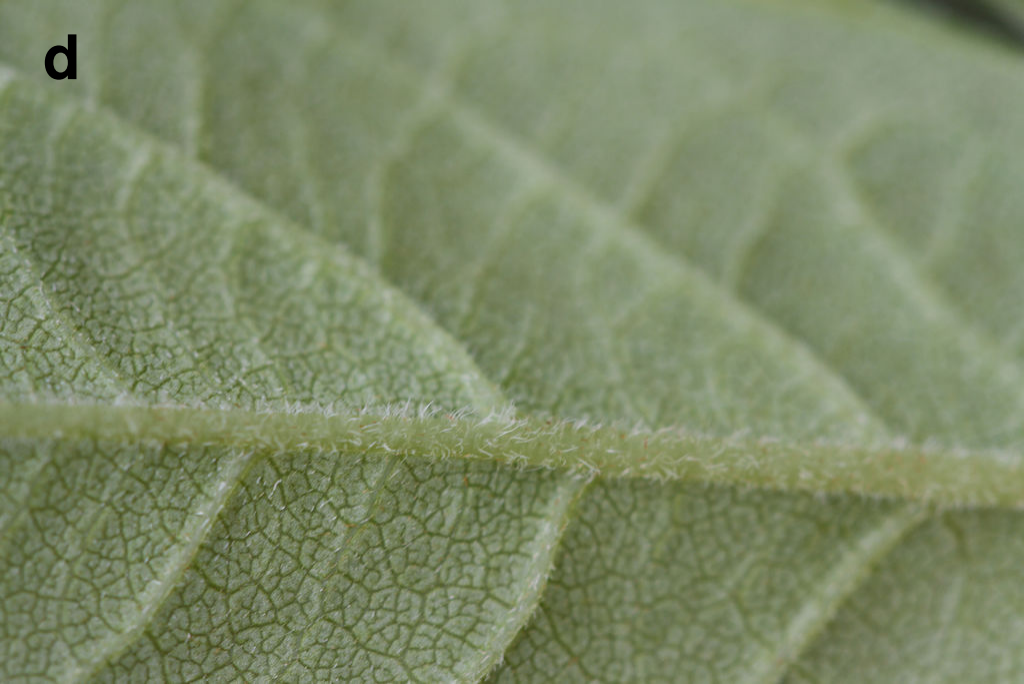
Abaxial leaf surface

**Figure 138e.**
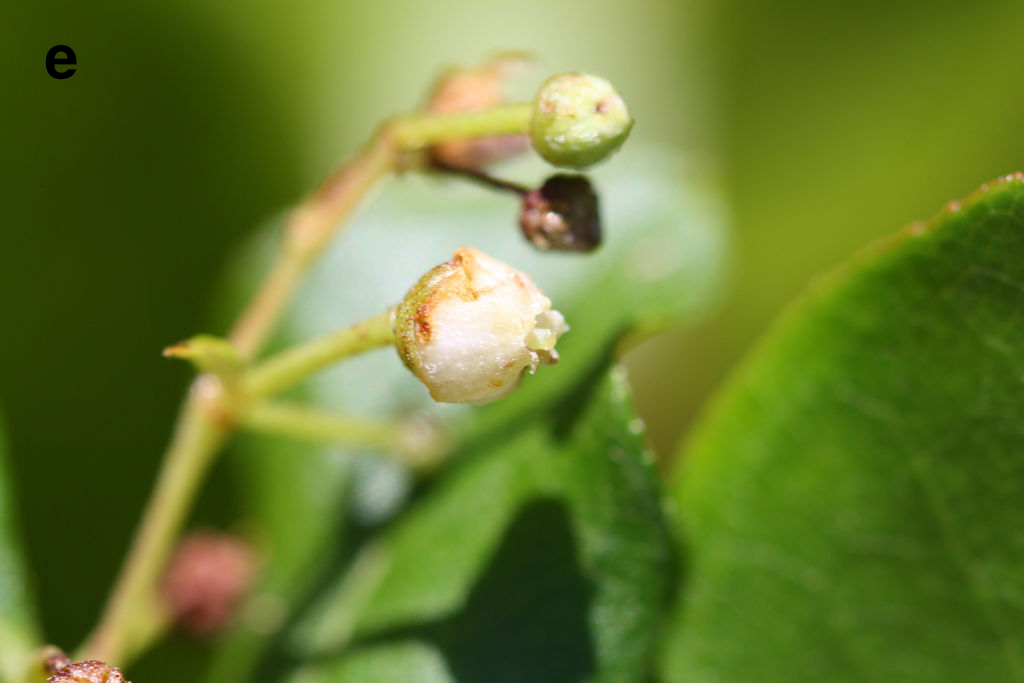
Flower

**Figure 138f.**
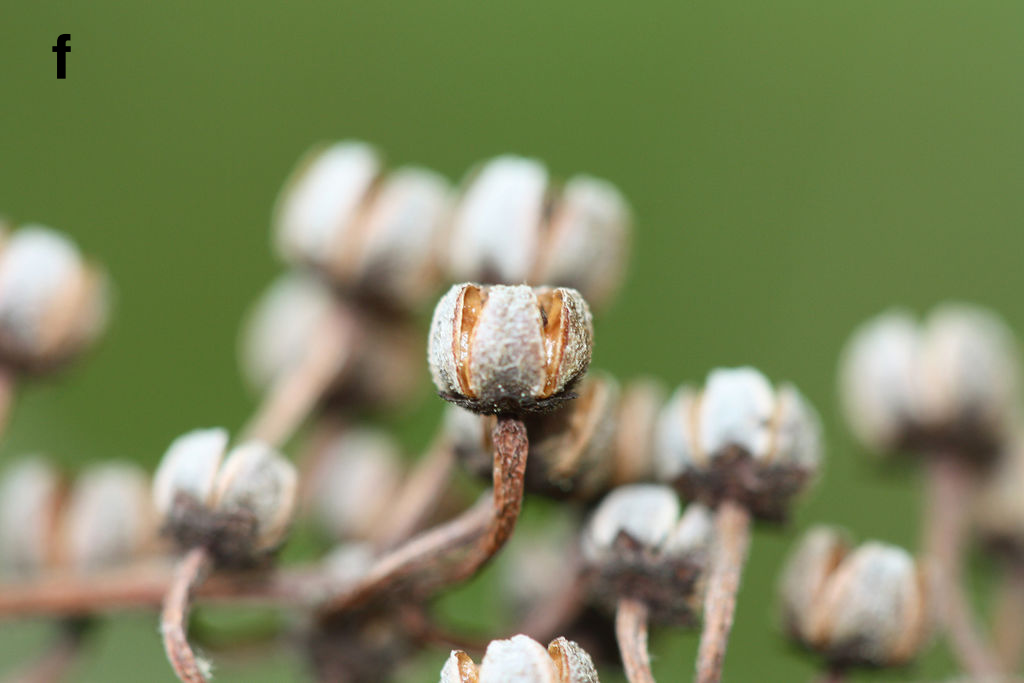
Fruits

**Figure 139a.**
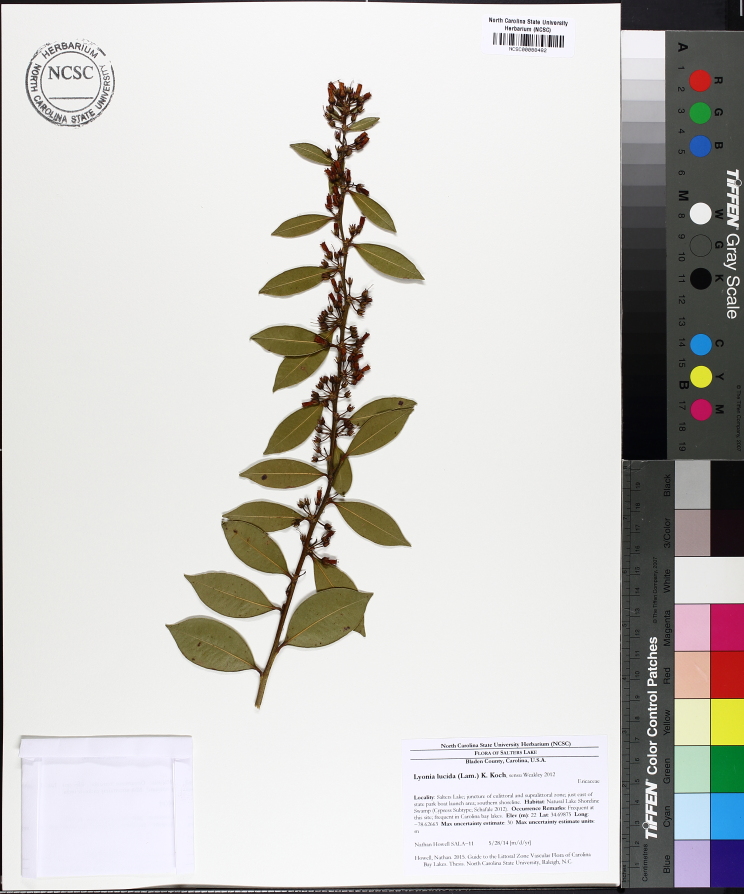
Specimen: *Howell SALA-11* (NCSC)

**Figure 139b.**
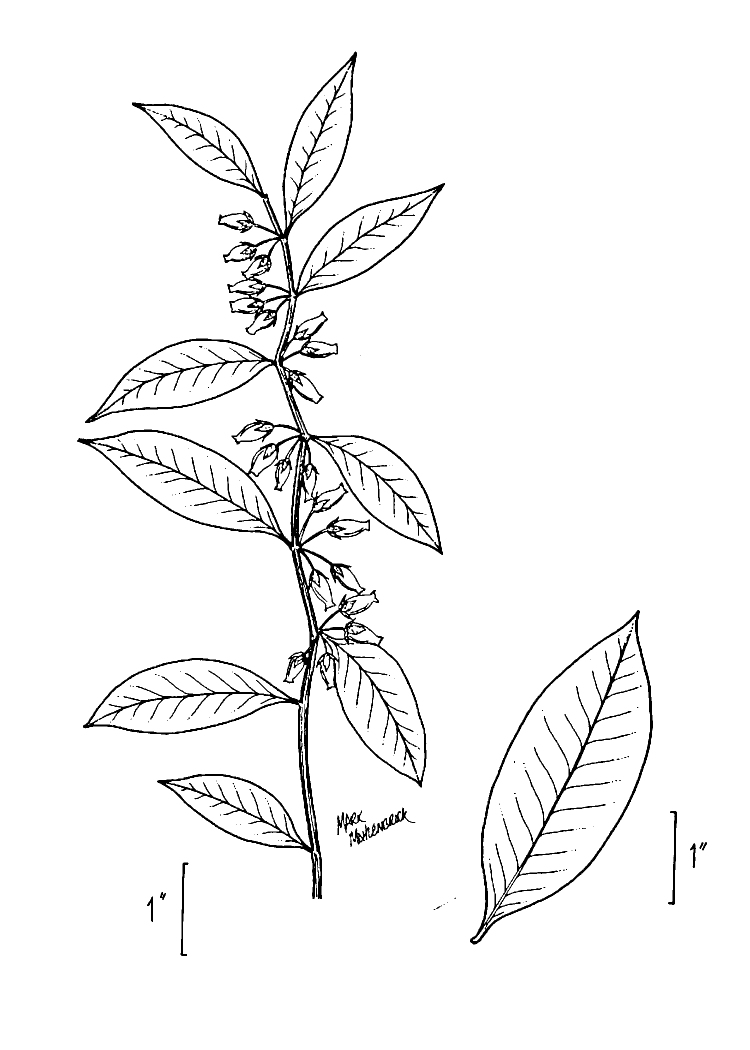
Illustration

**Figure 139c.**
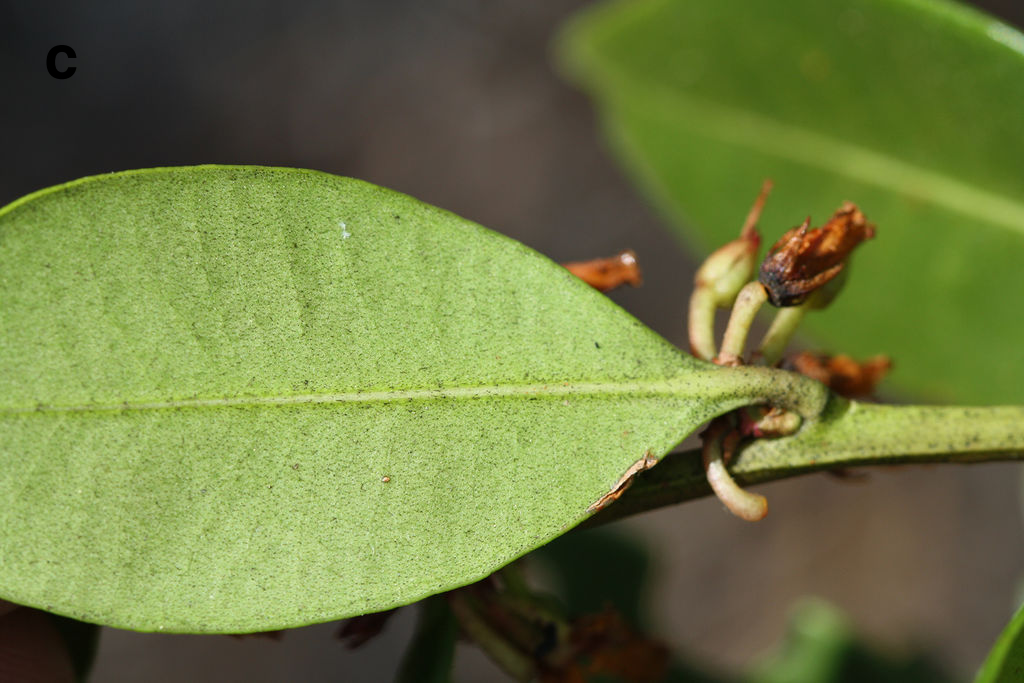
Leaf (abaxial surface)

**Figure 139d.**
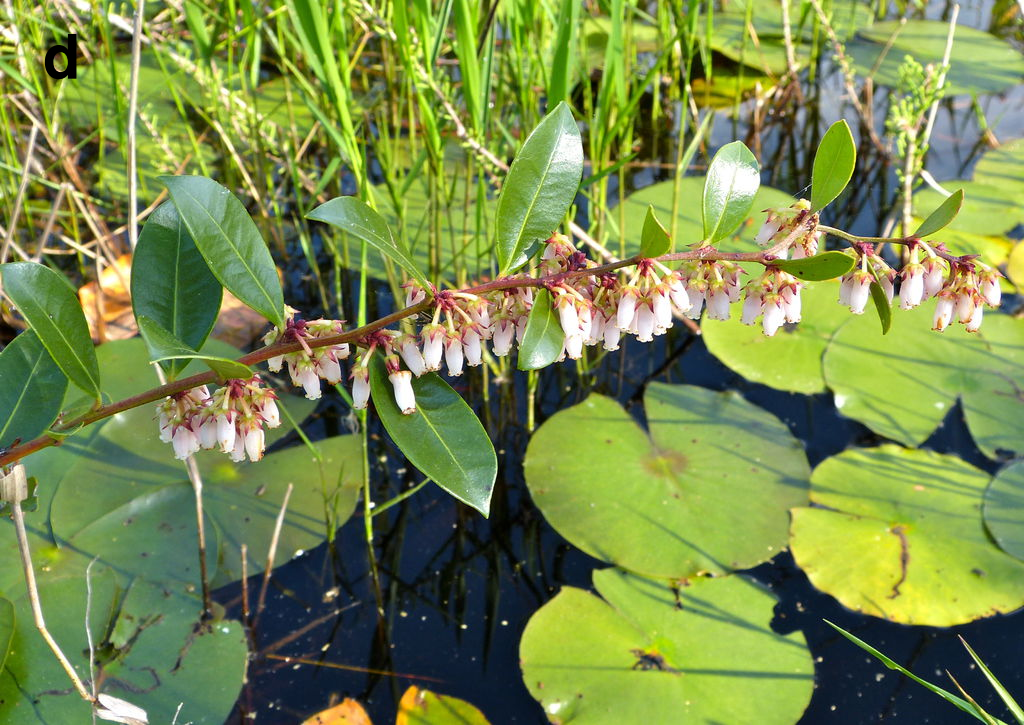
Inflorescence

**Figure 139e.**
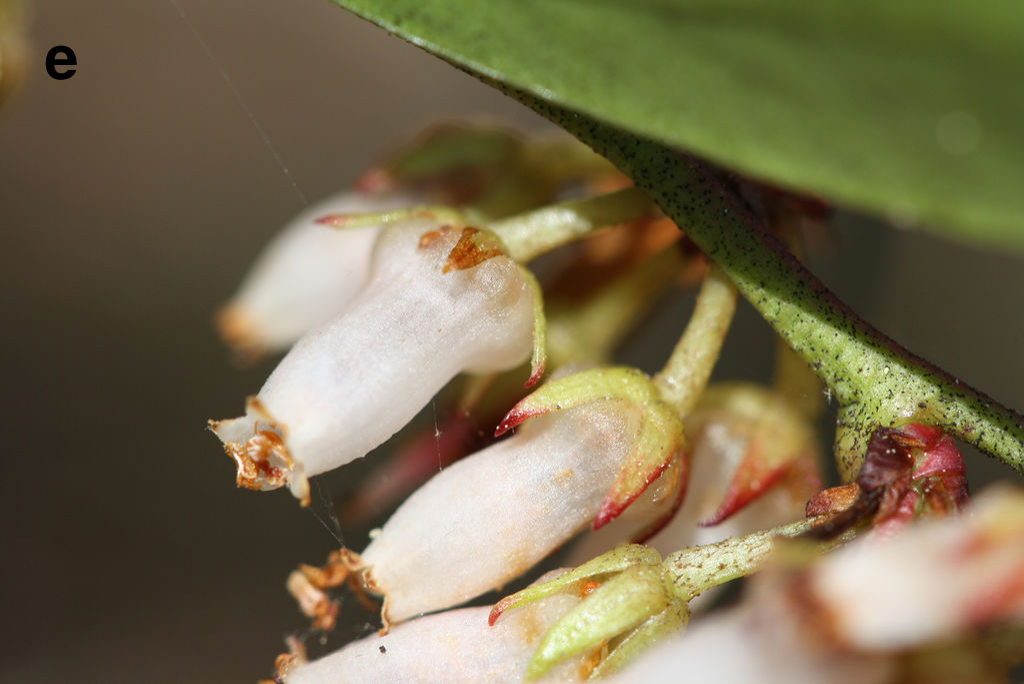
Flowers

**Figure 139f.**
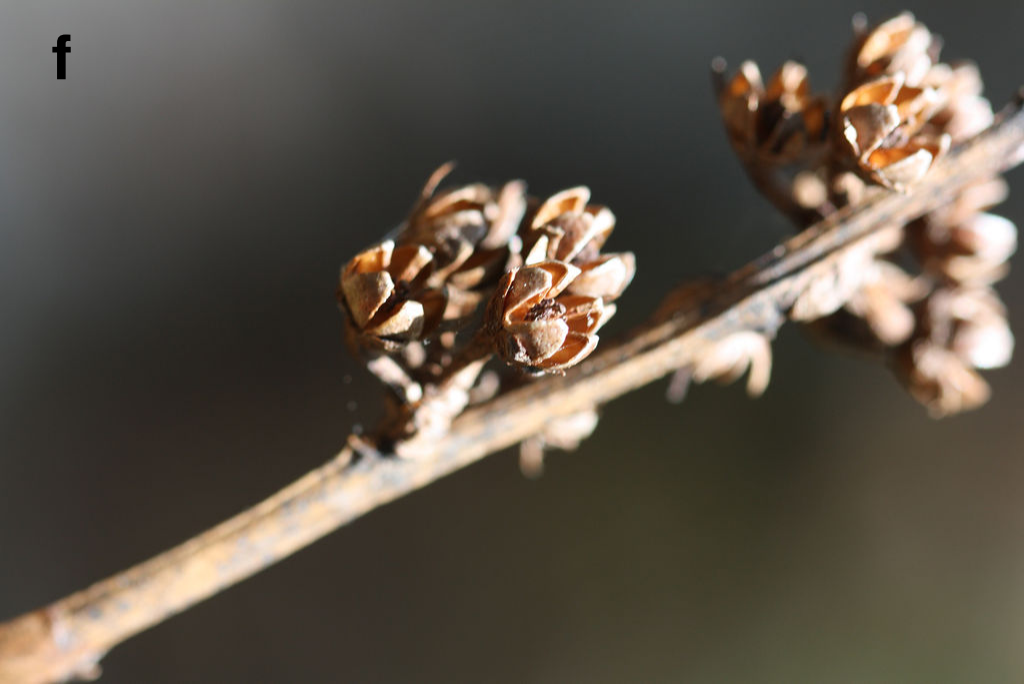
Fruits

**Figure 140a.**
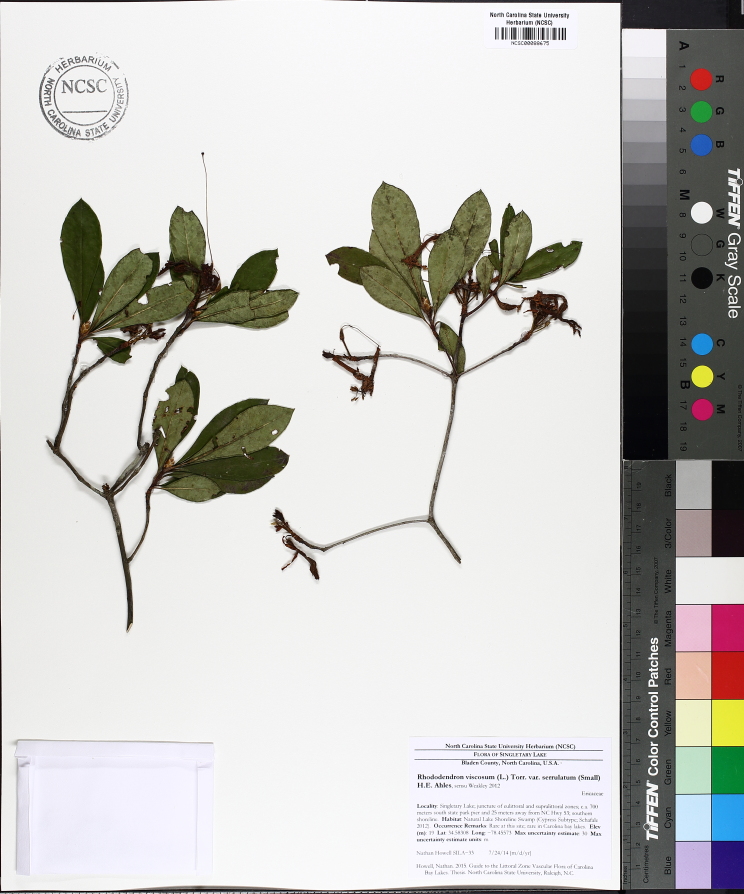
Specimen: *Howell SILA-33* (NCSC)

**Figure 140b.**
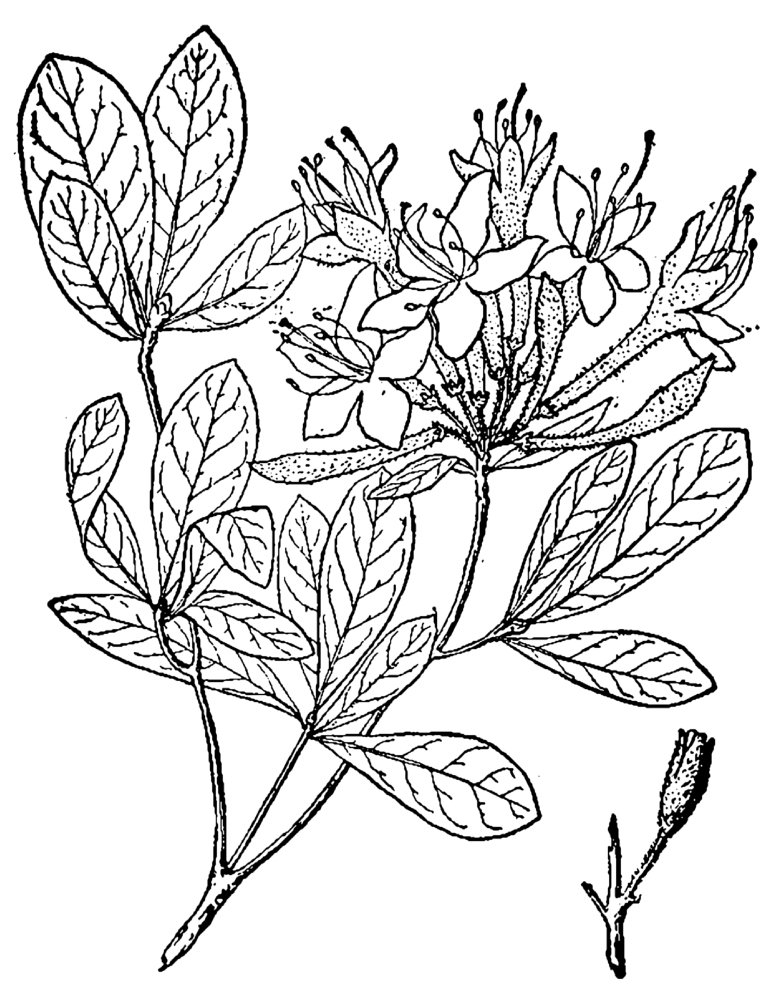
Illustration

**Figure 140c.**
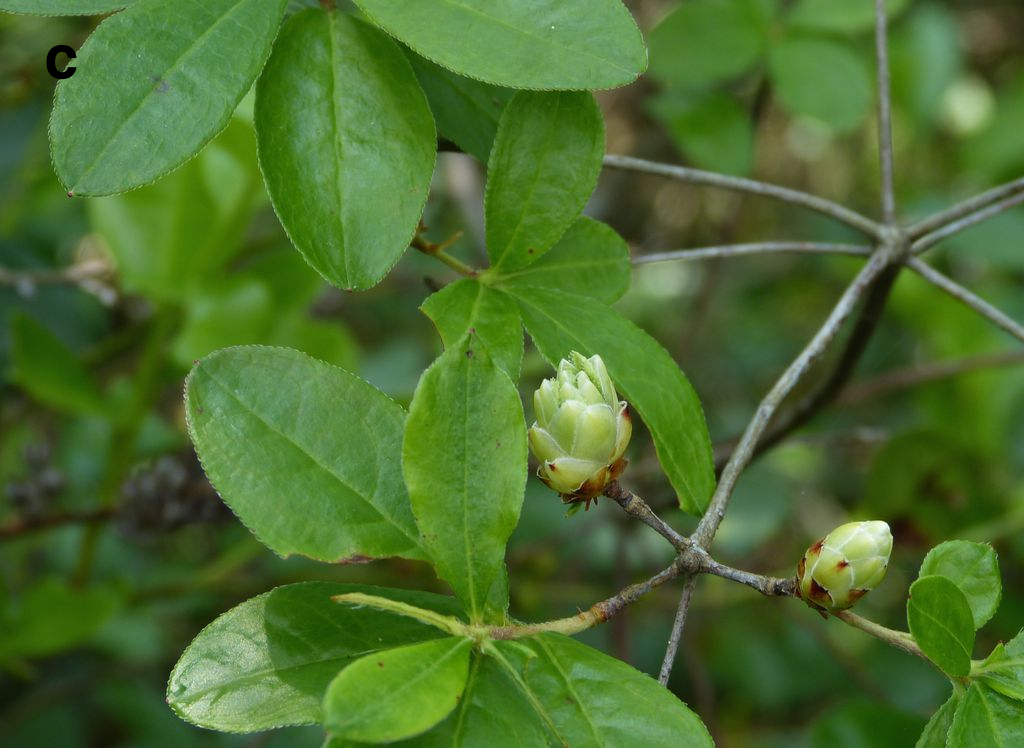
Leaves

**Figure 140d.**
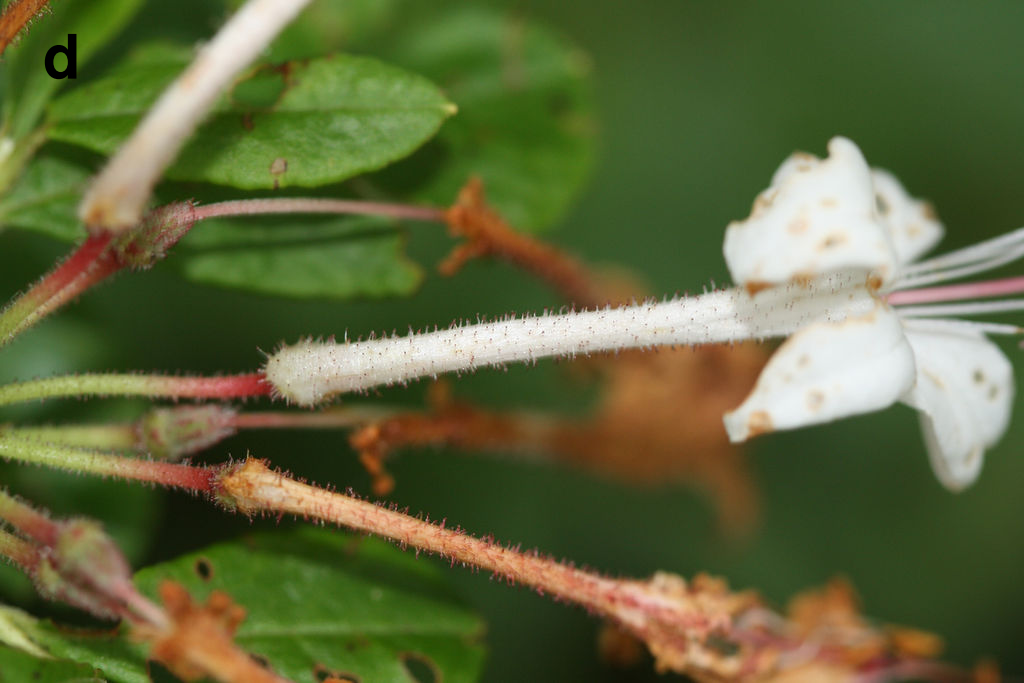
Flower

**Figure 140e.**
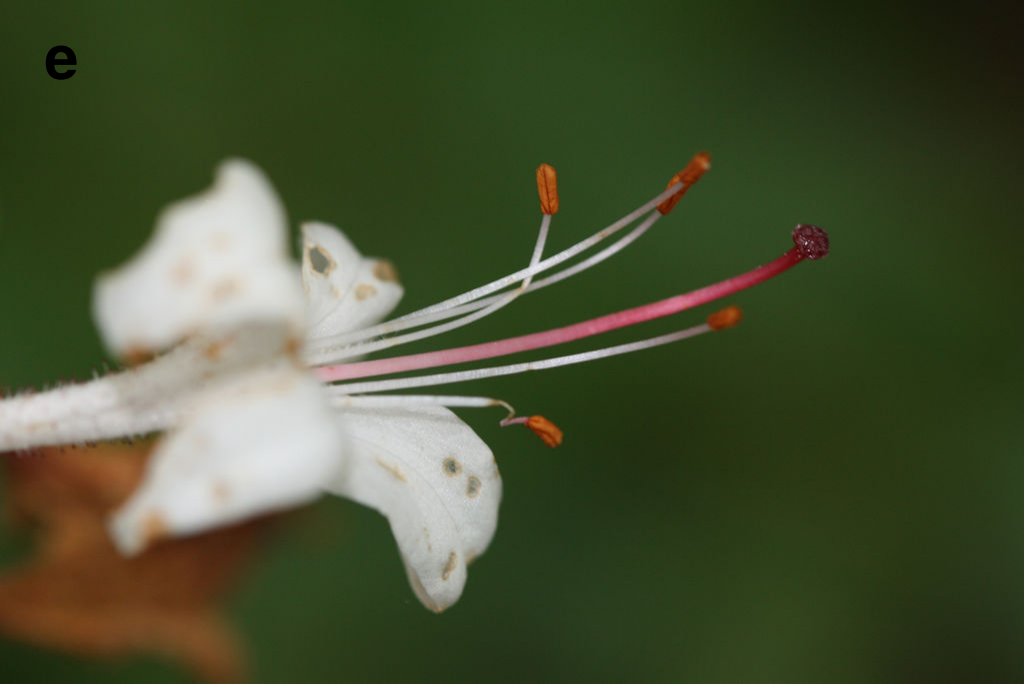
Flower detail

**Figure 140f.**
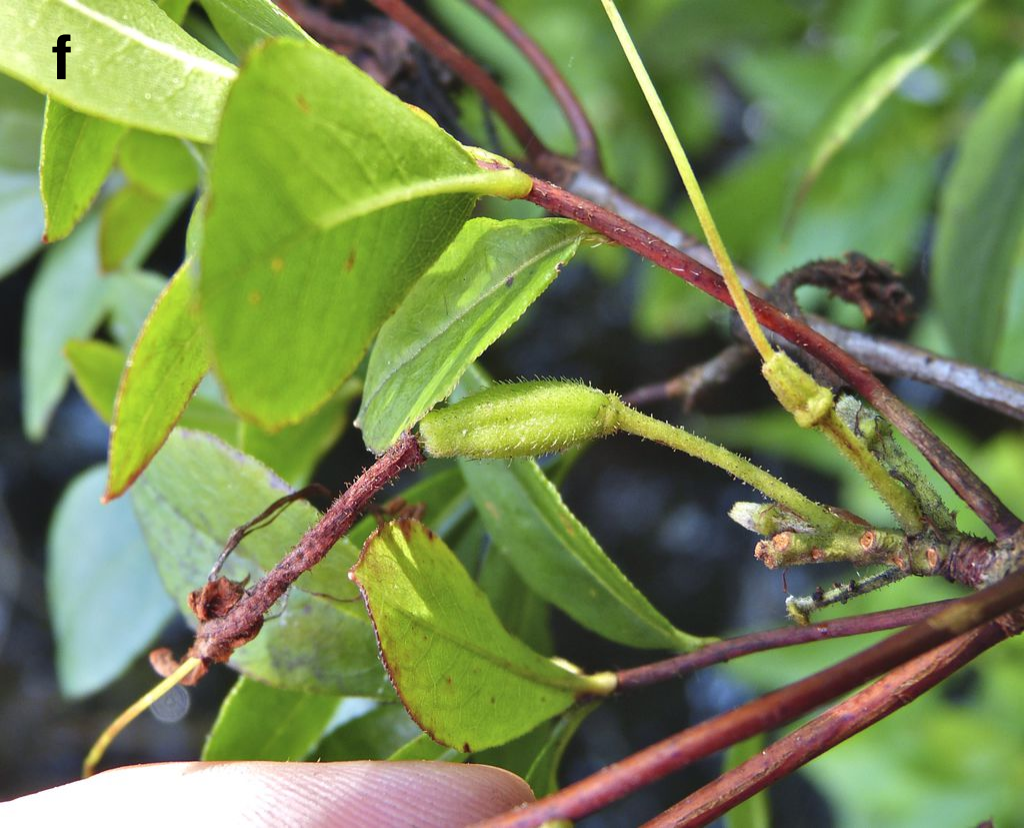
Fruit

**Figure 141a.**
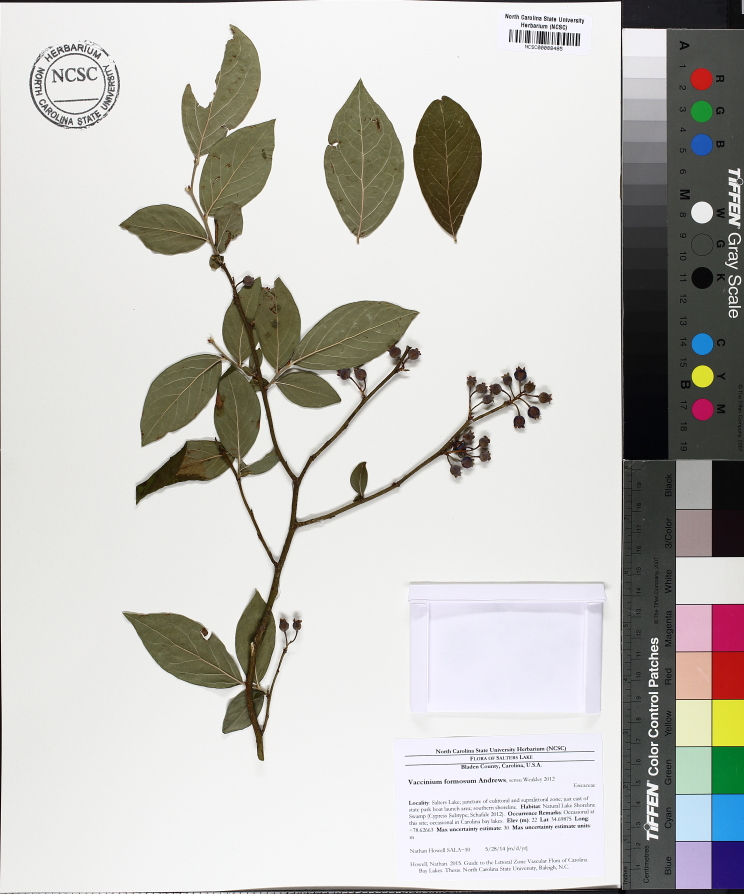
Specimen: *Howell SALA-10* (NCSC)

**Figure 141b.**
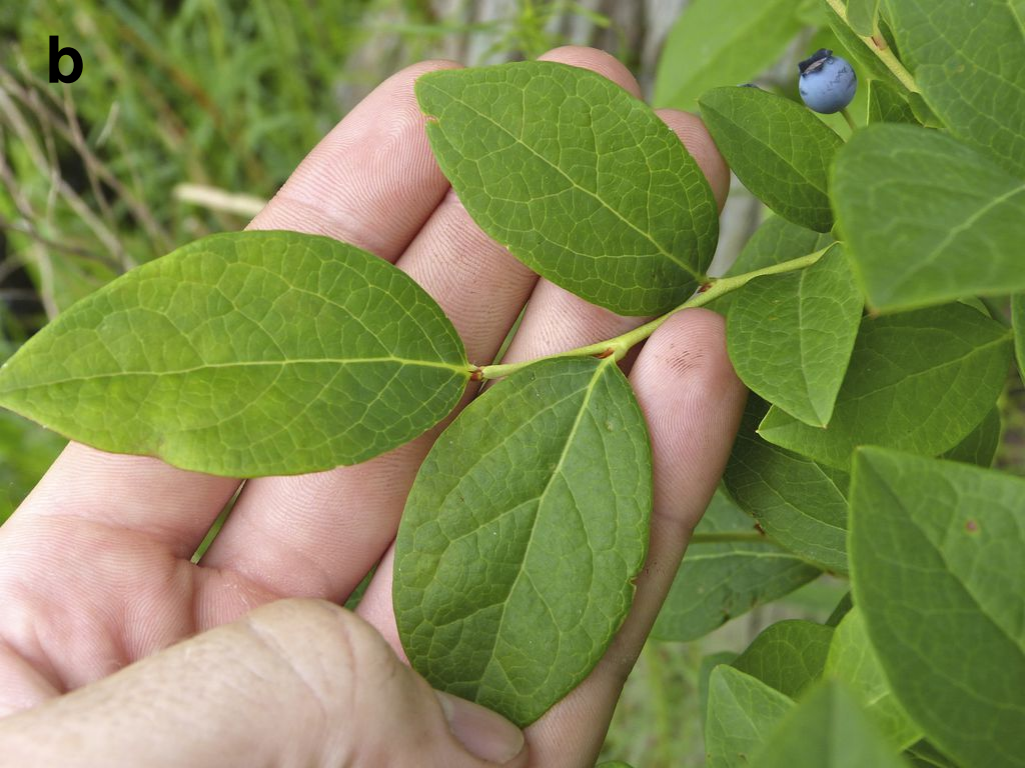
Leaves

**Figure 141c.**
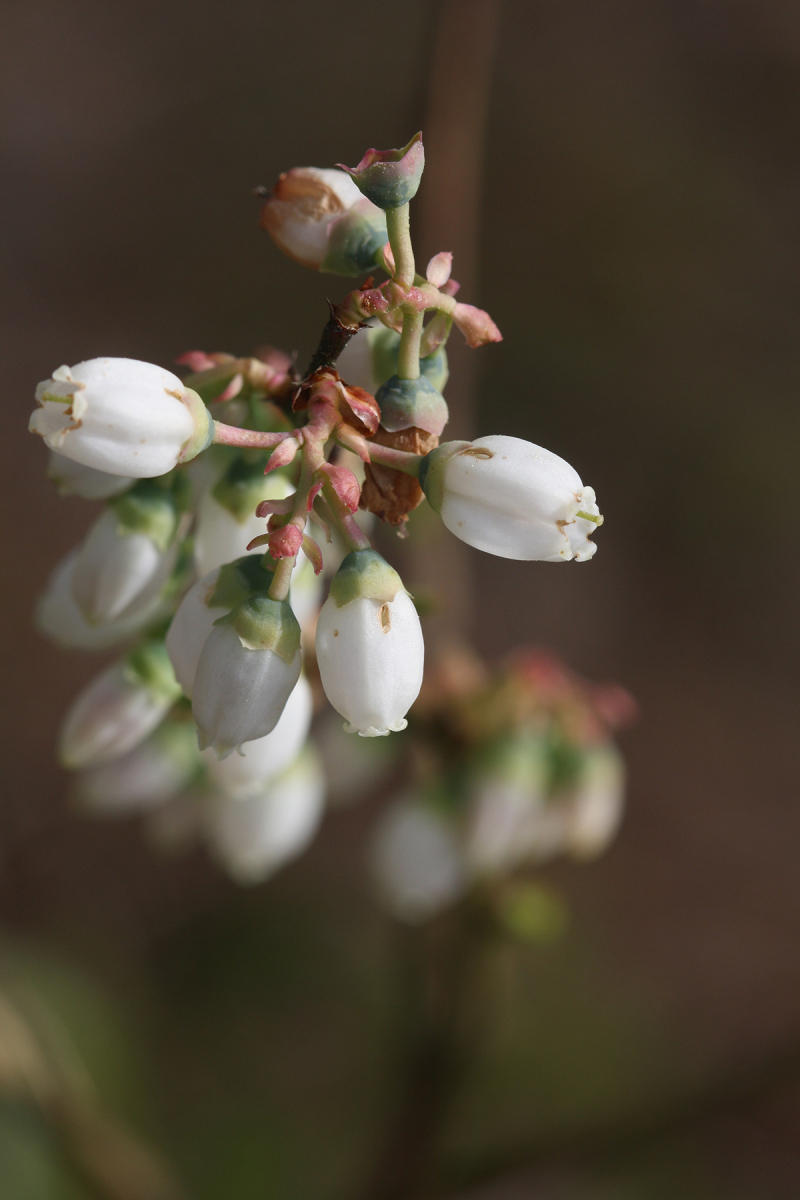
Inflorescence

**Figure 141d.**
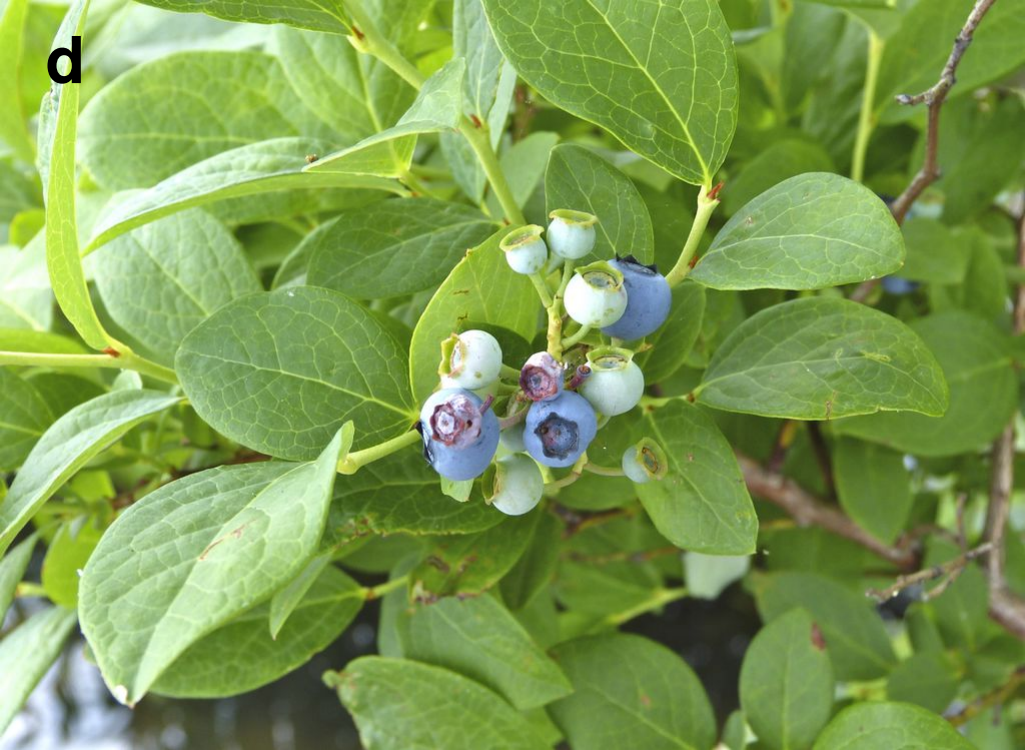
Infructescence

**Figure 142a.**
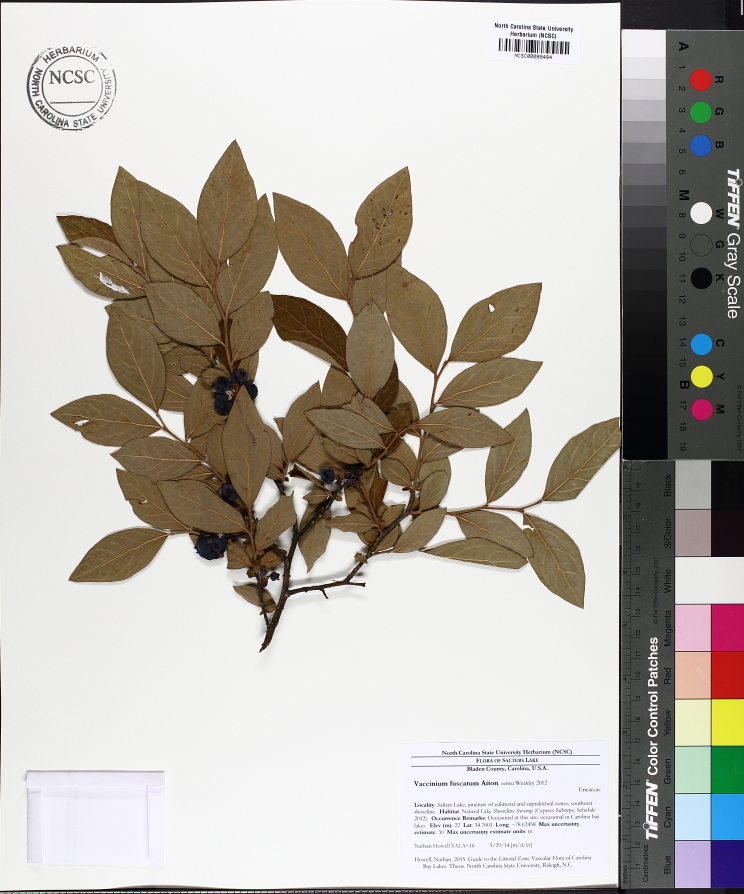
Specimen: *Howell SALA-16* (NCSC)

**Figure 142b.**
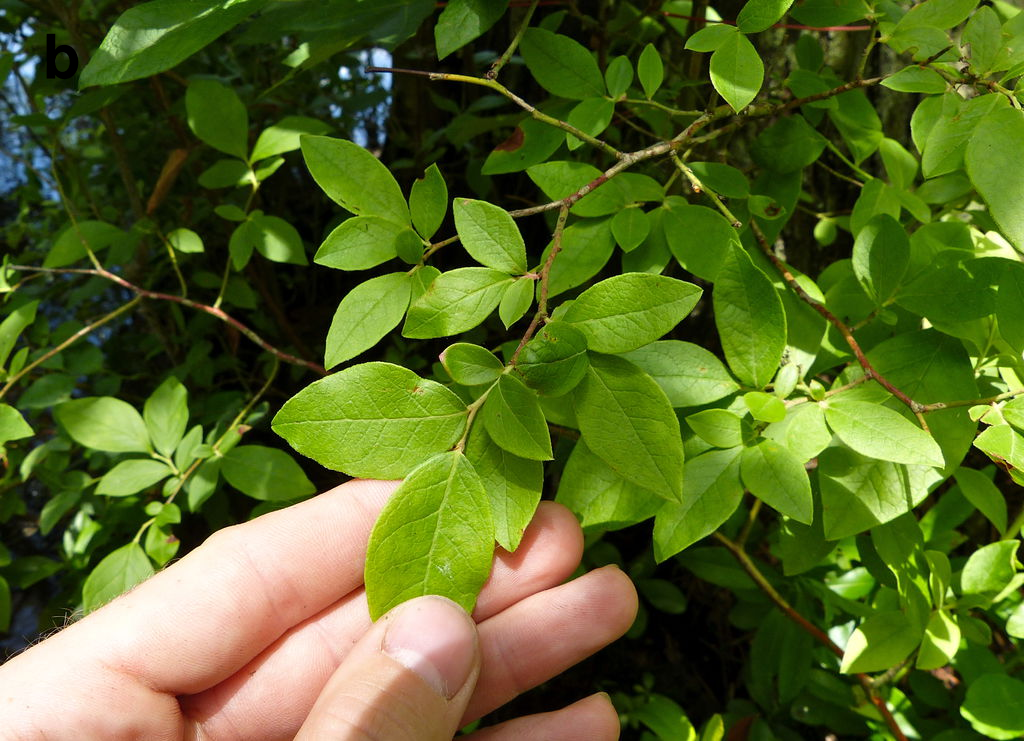
Leaves

**Figure 142c.**
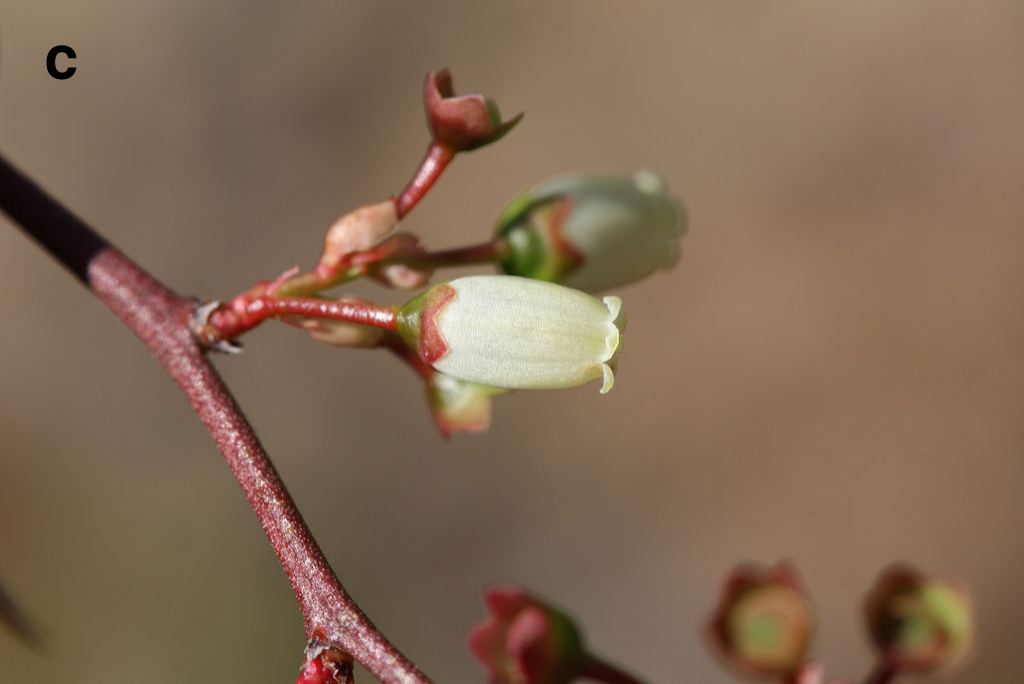
Flower

**Figure 142d.**
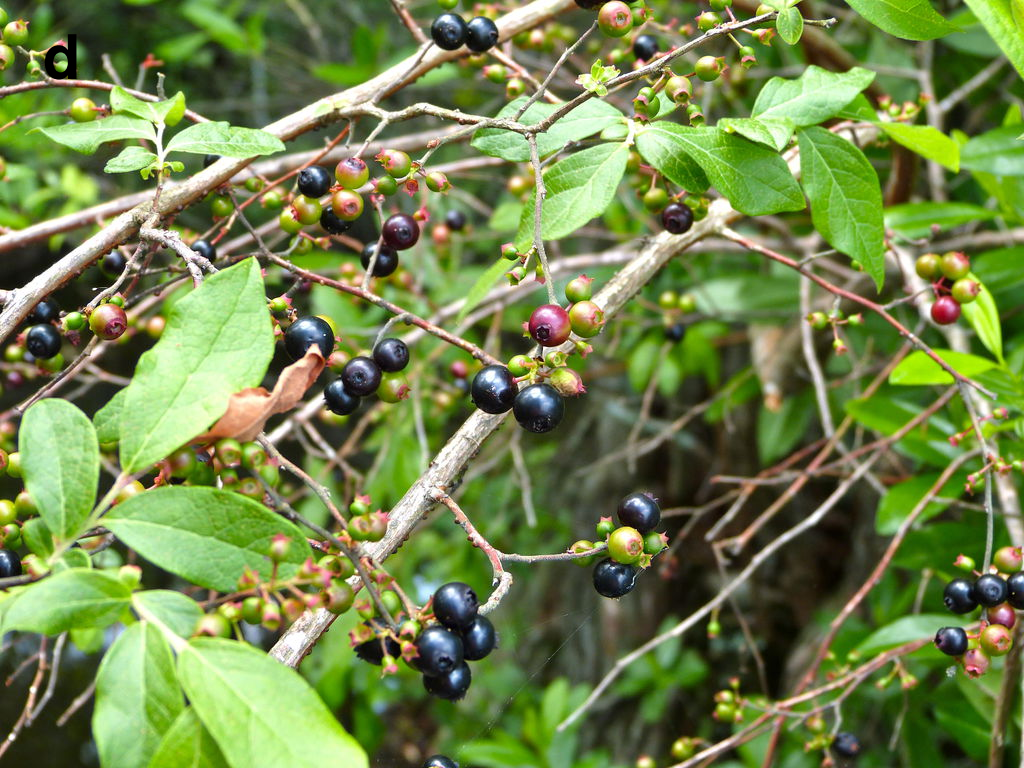
Infructescence

**Figure 143a.**
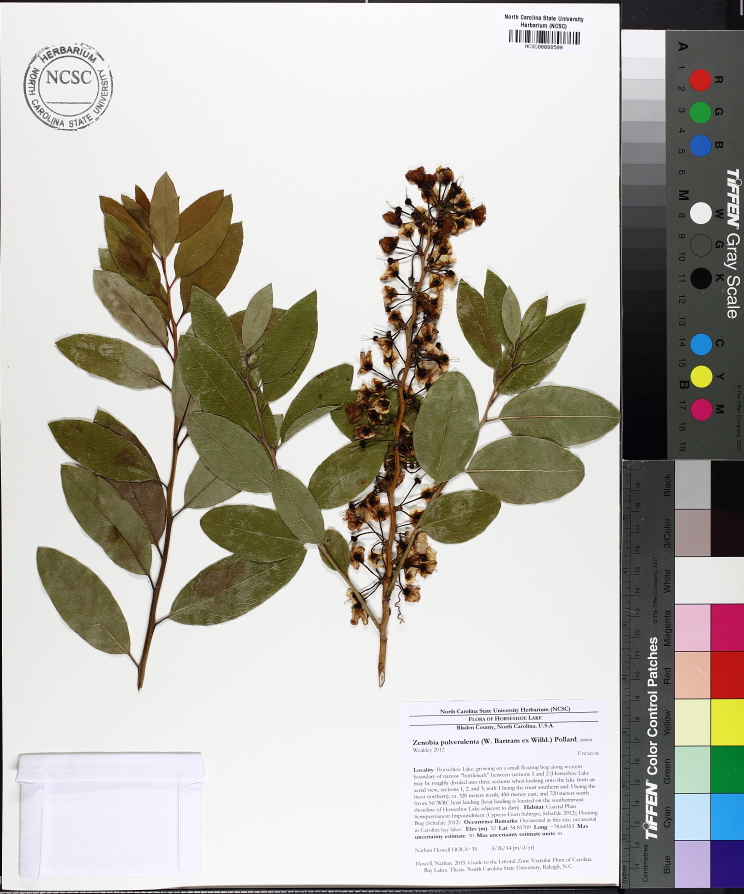
Specimen: *Howell HOLA-35* (NCSC)

**Figure 143b.**
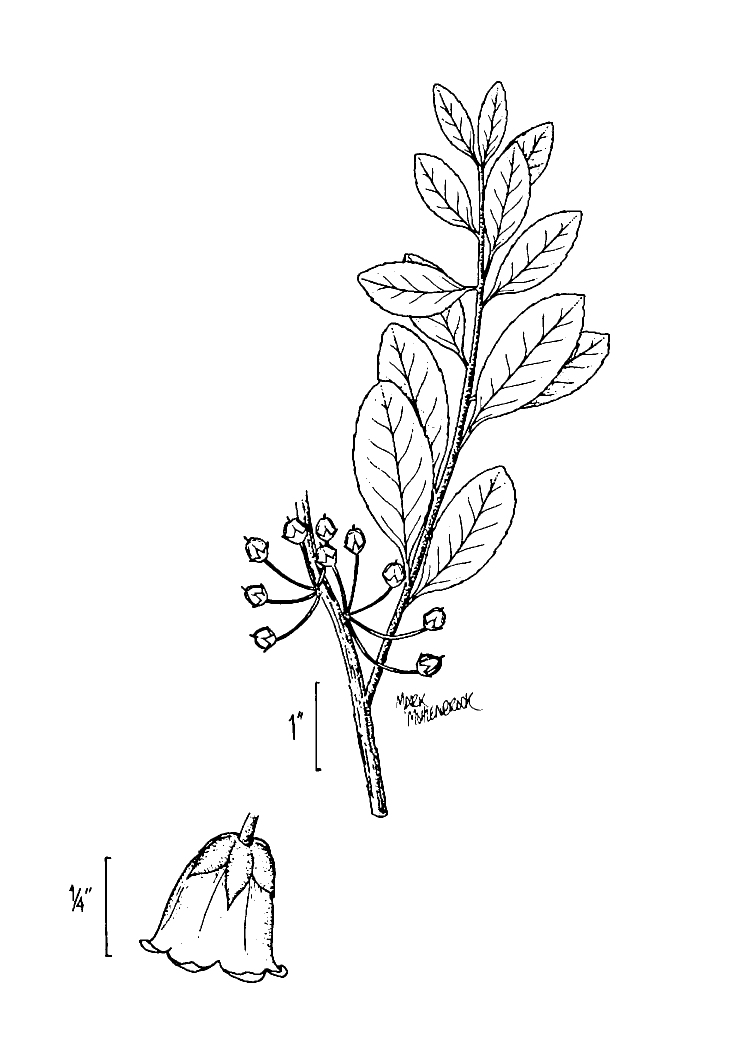
Illustration

**Figure 143c.**
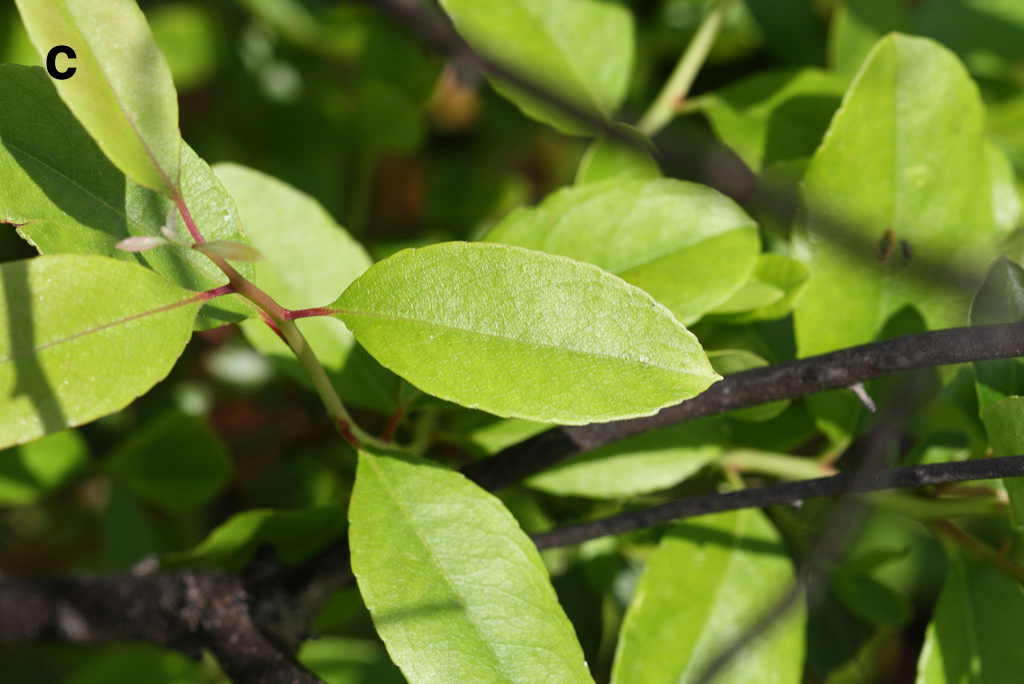
Leaves

**Figure 143d.**
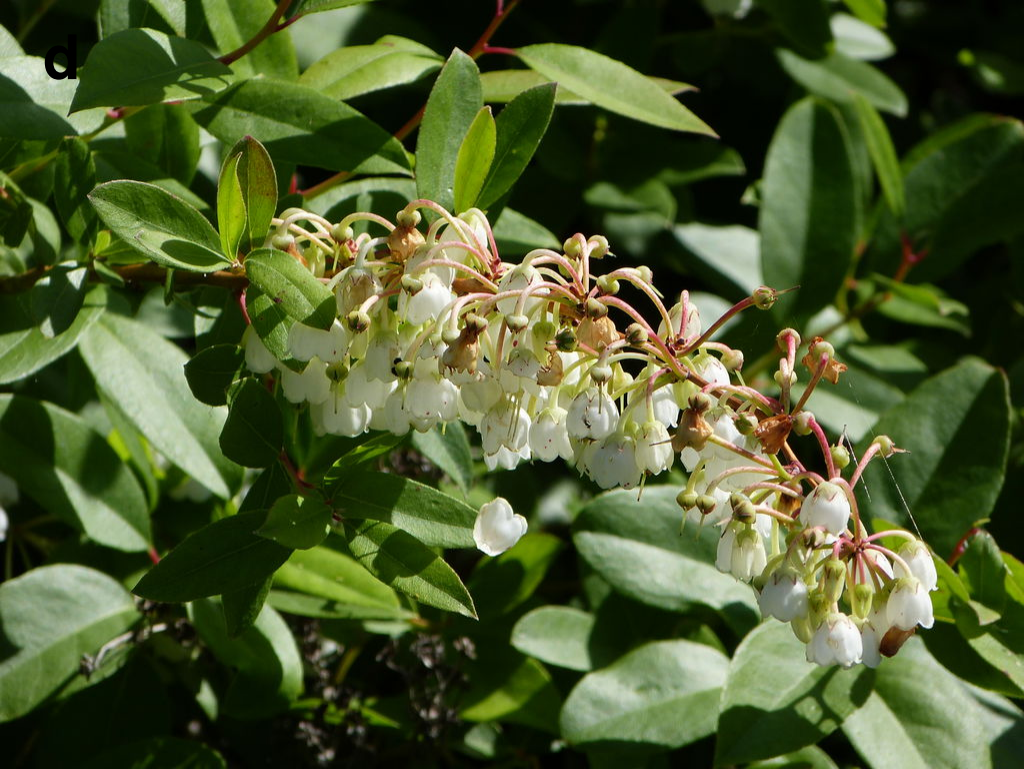
Inflorescence

**Figure 143e.**
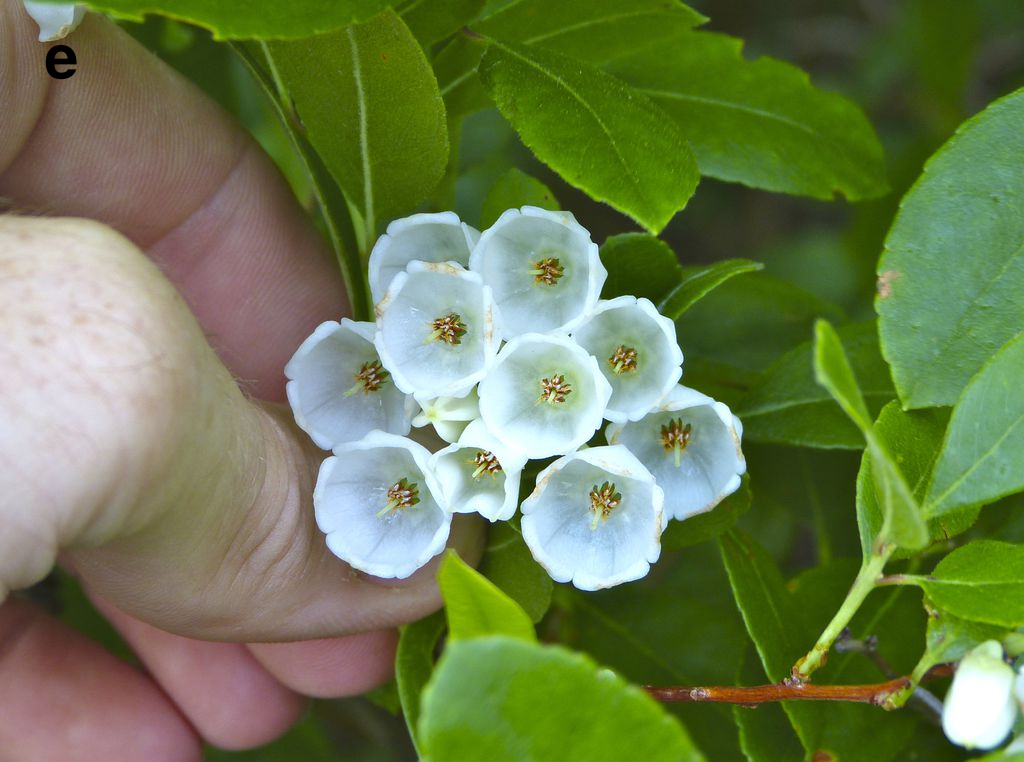
Flowers within

**Figure 143f.**
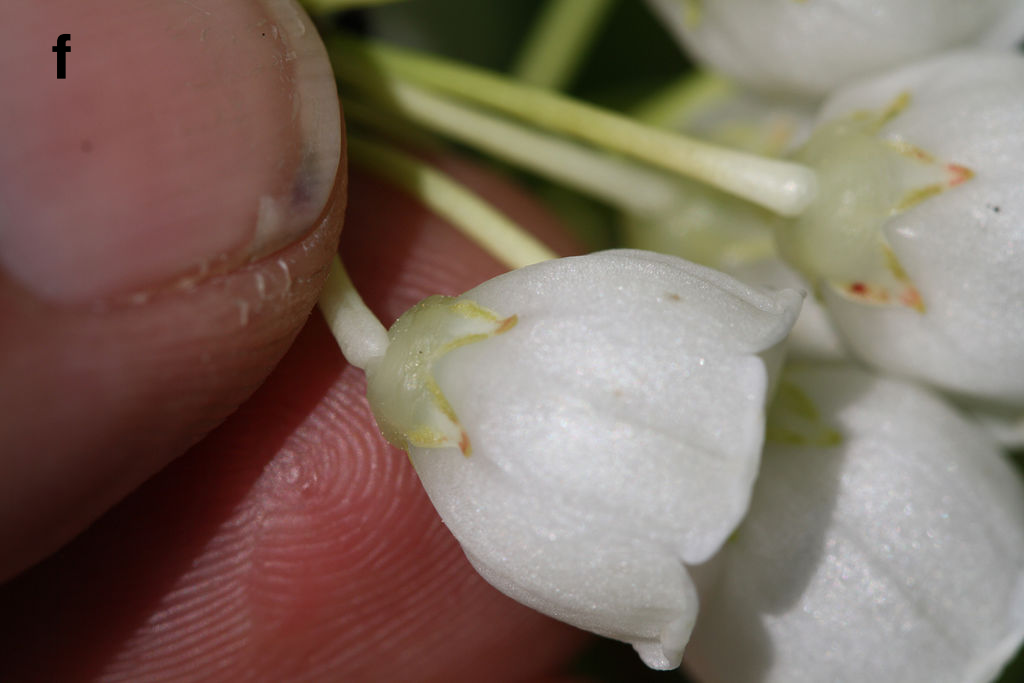
Flower

**Figure 144a.**
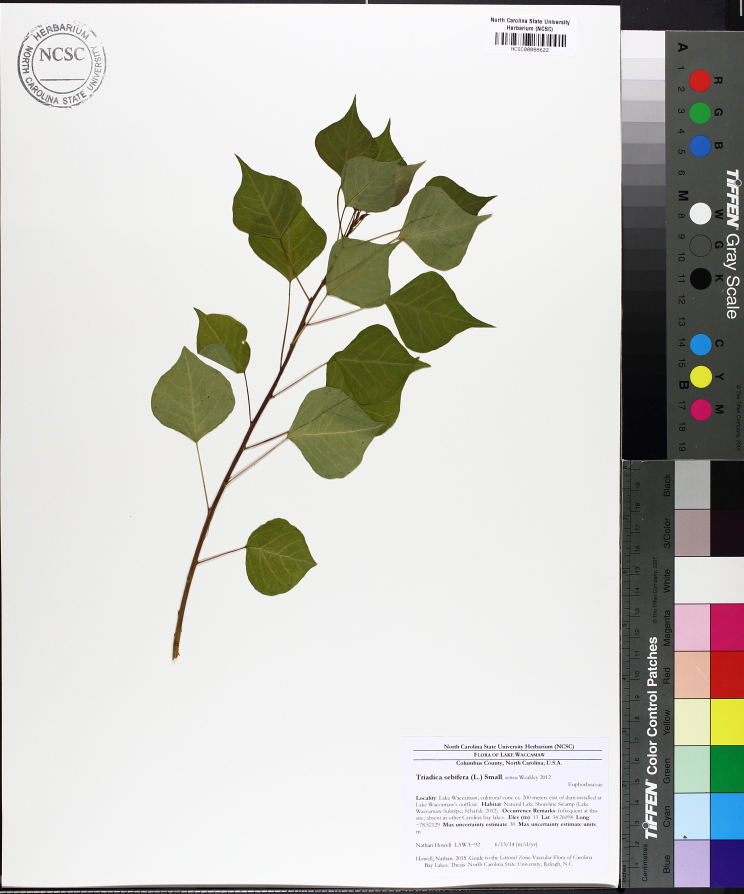
Specimen: *Howell LAWA-92* (NCSC)

**Figure 144b.**
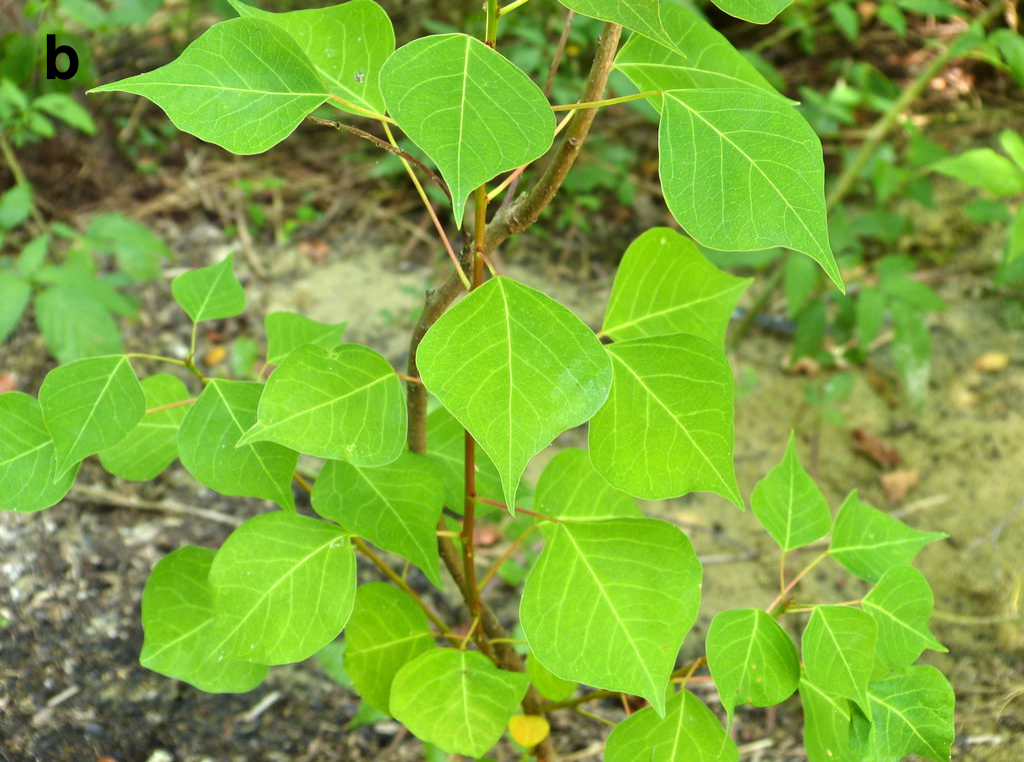
Young tree

**Figure 145a.**
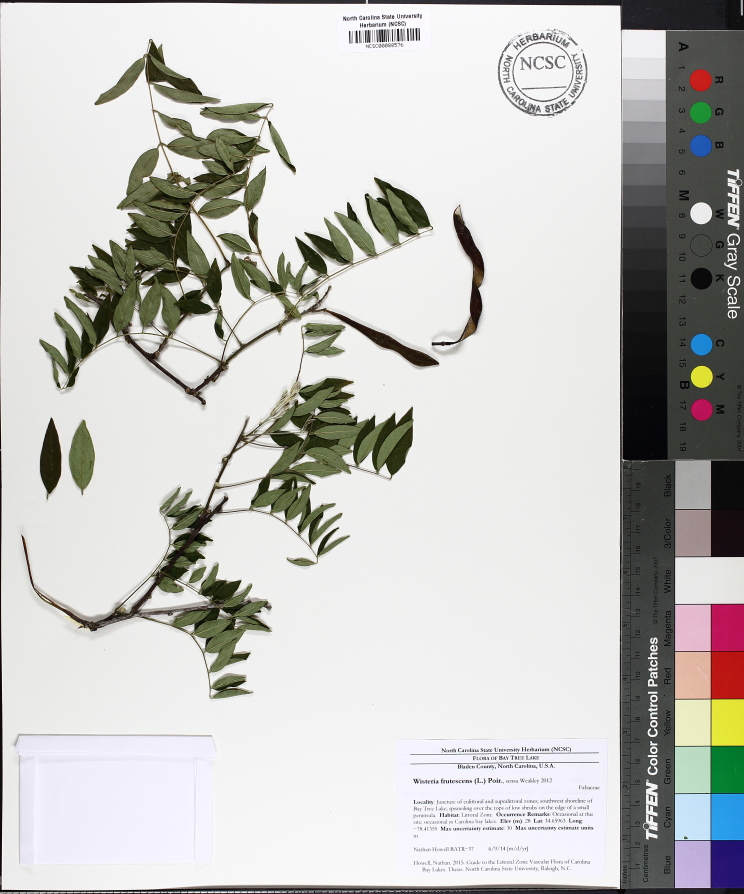
Specimen: *Howell BATR-37* (NCSC)

**Figure 145b.**
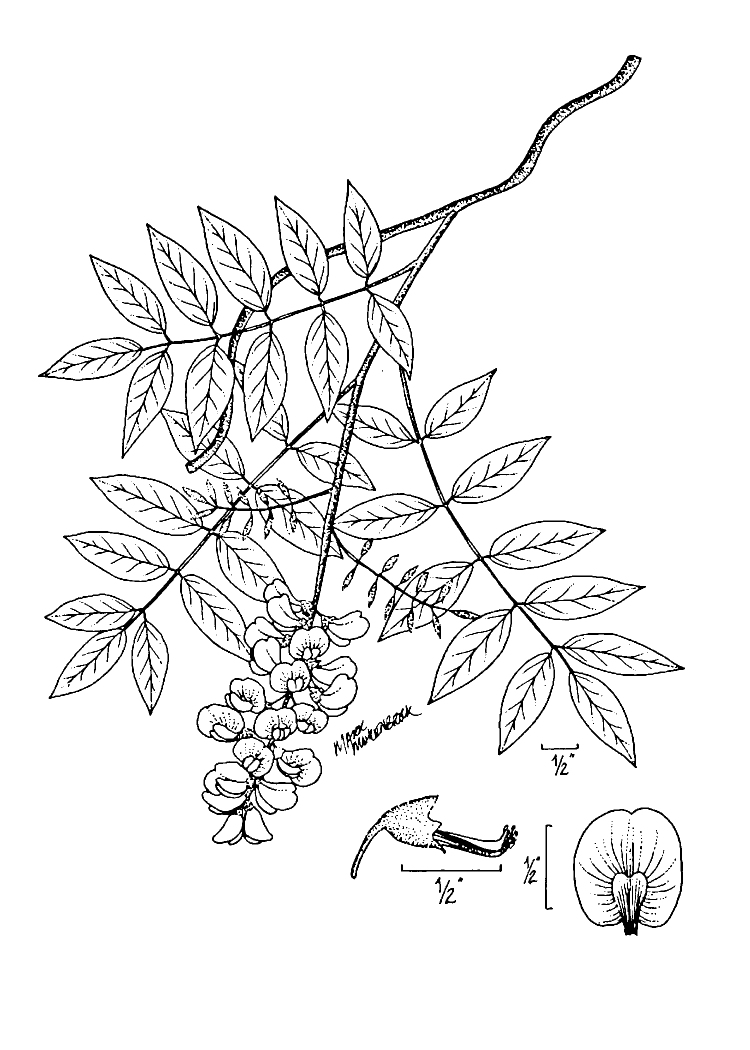
Illustration

**Figure 145c.**
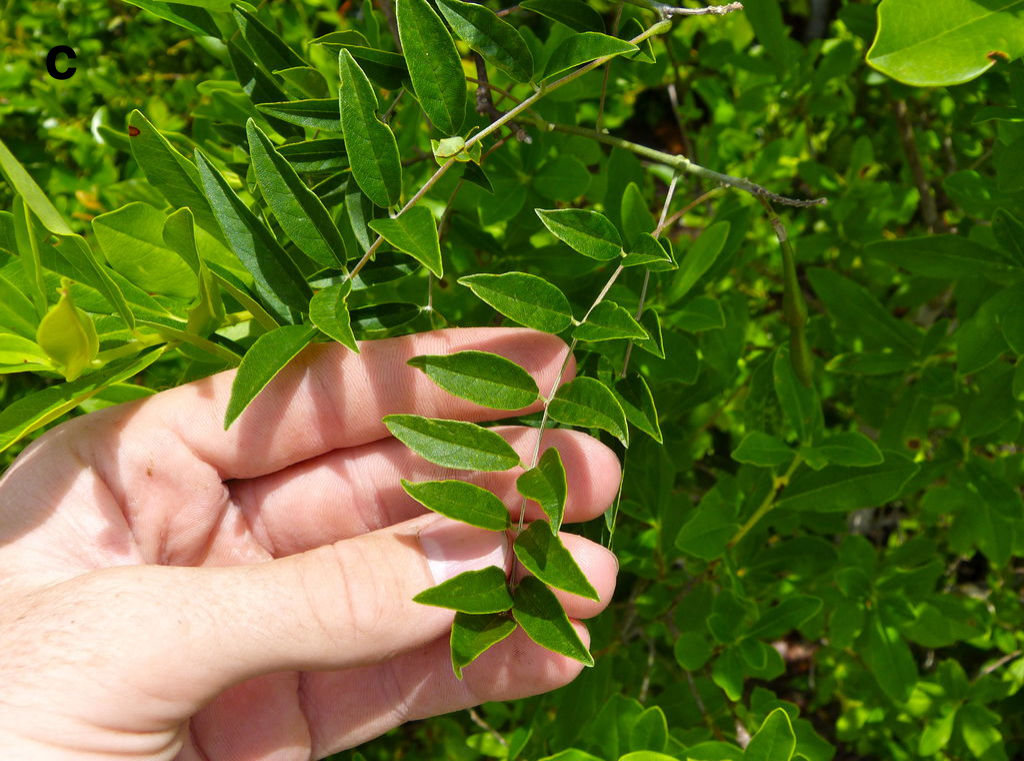
Imparipinnate leaf

**Figure 145d.**
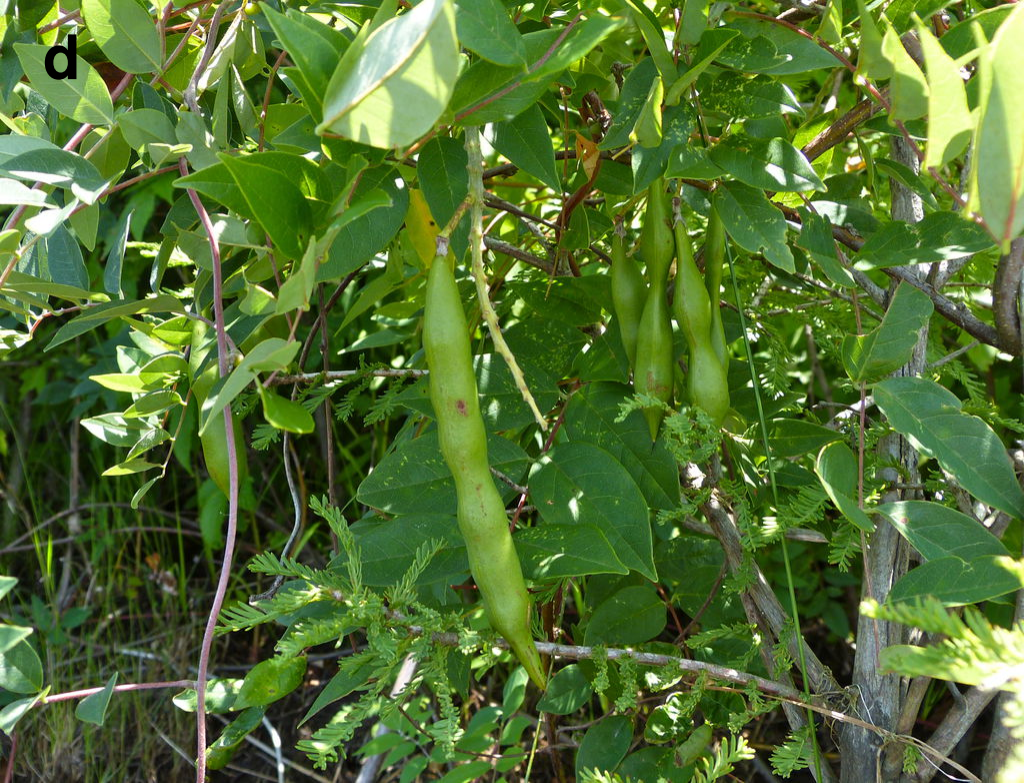
Fruit

**Figure 146a.**
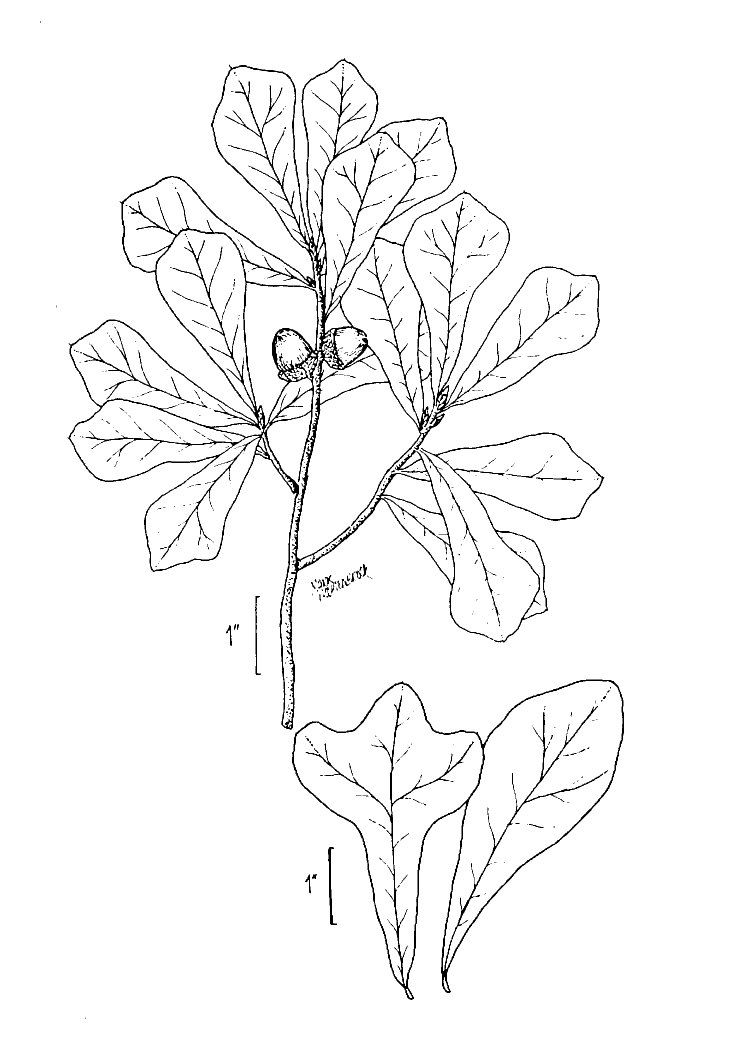
Illustration

**Figure 146b.**
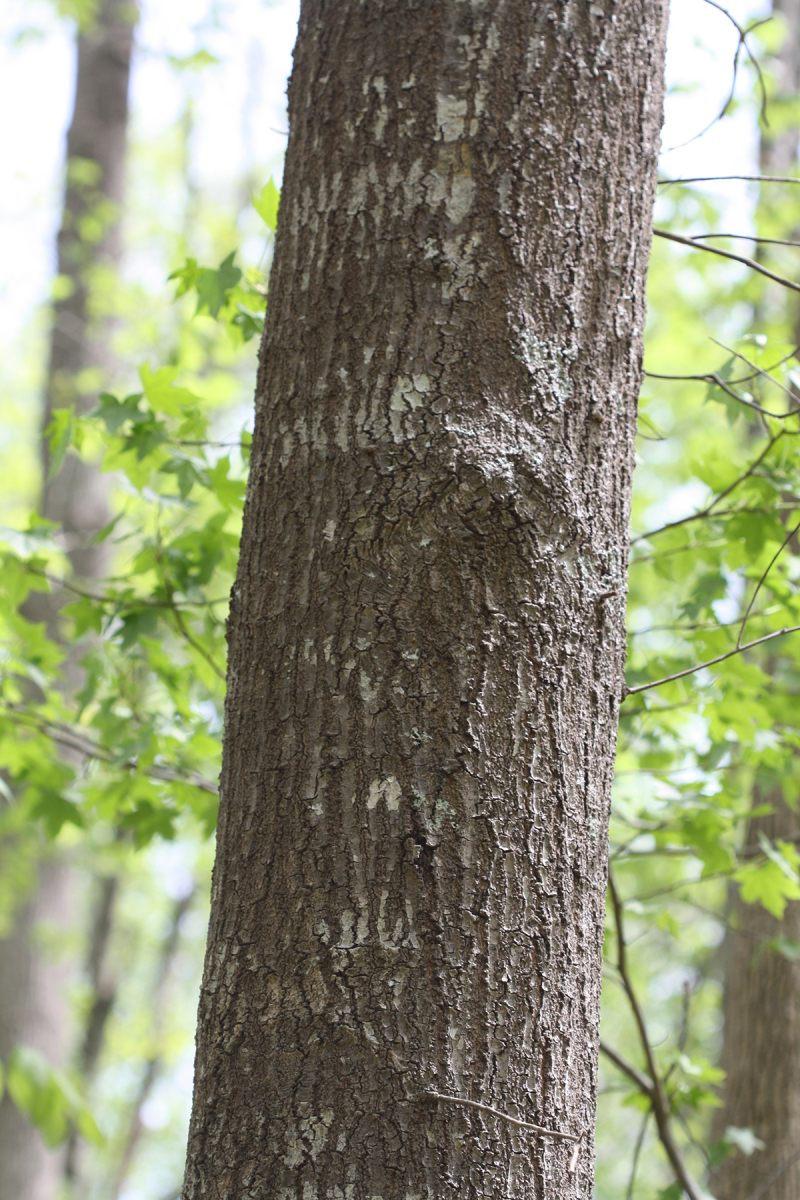
Bark

**Figure 146c.**
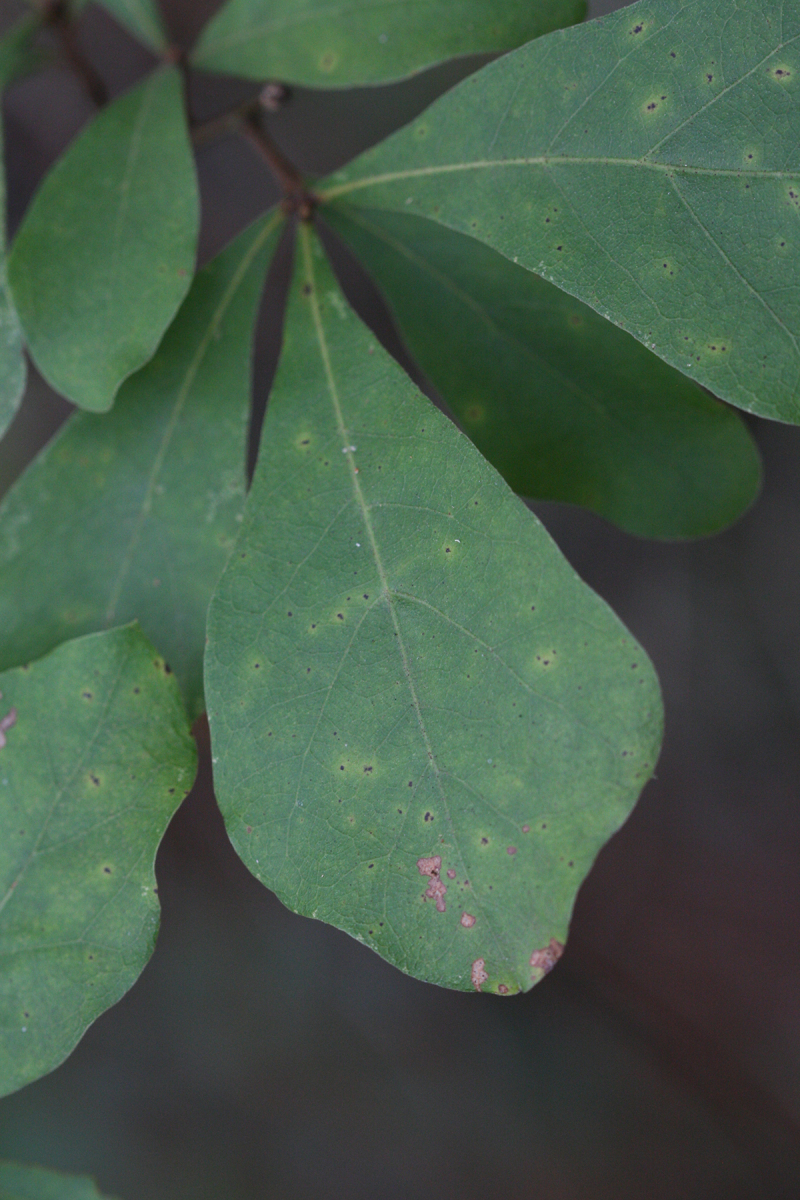
Leaves

**Figure 146d.**
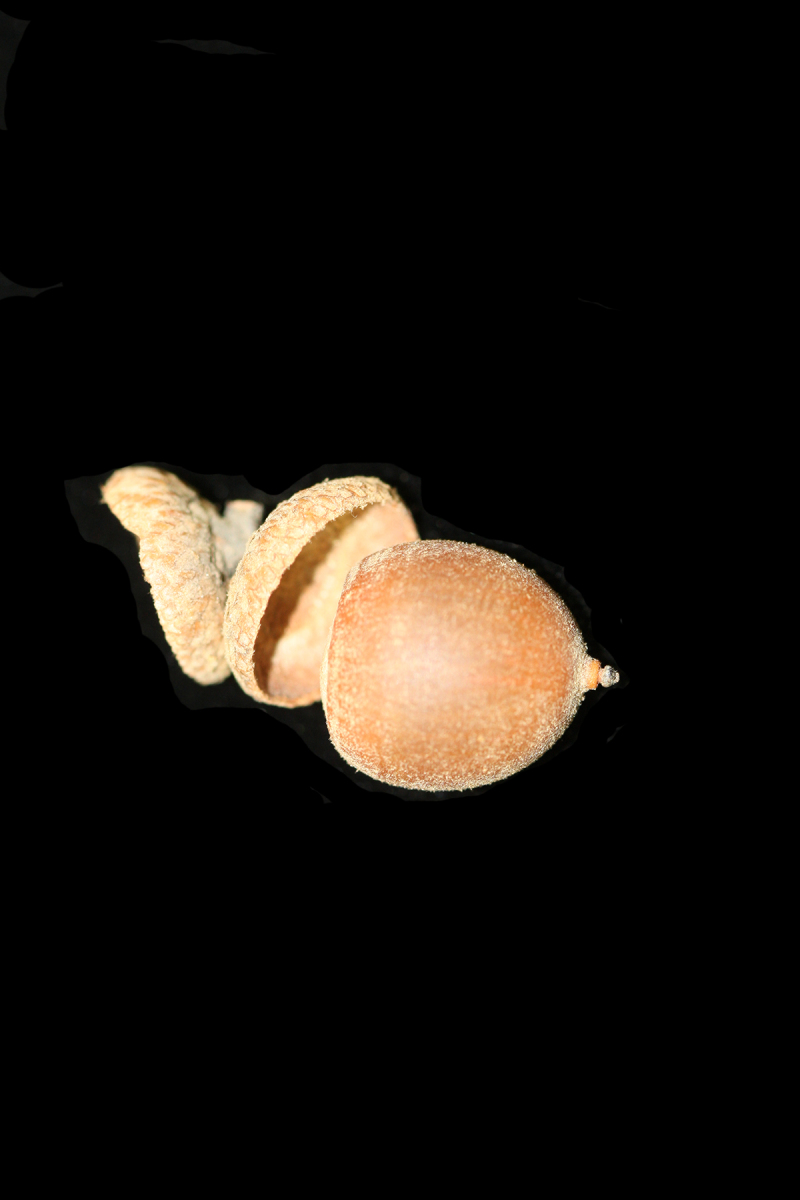
Fruit

**Figure 147a.**
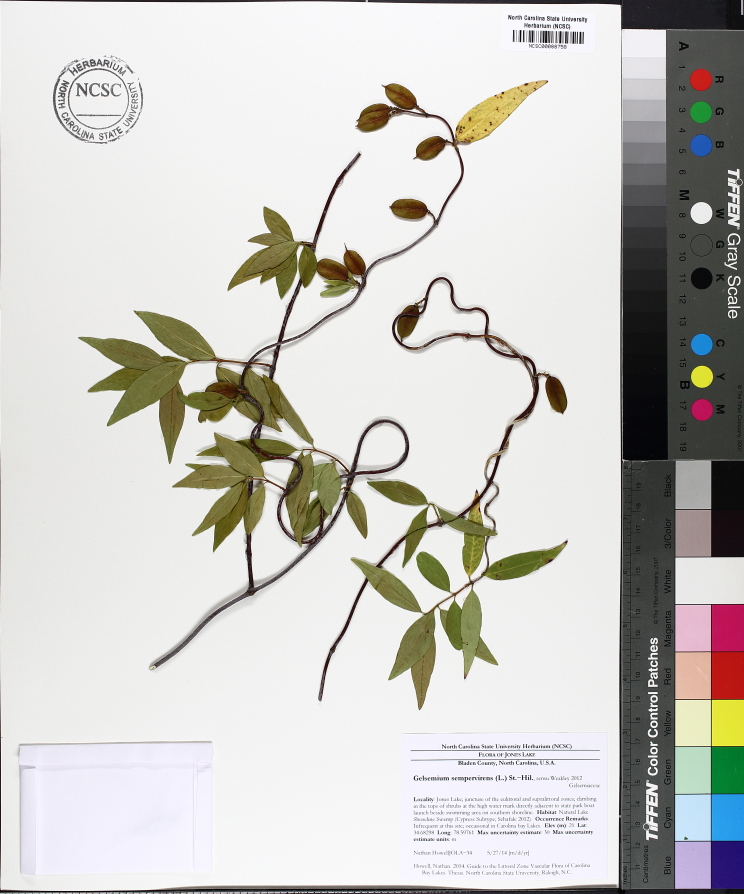
Specimen: *Howell JOLA-34* (NCSC)

**Figure 147b.**
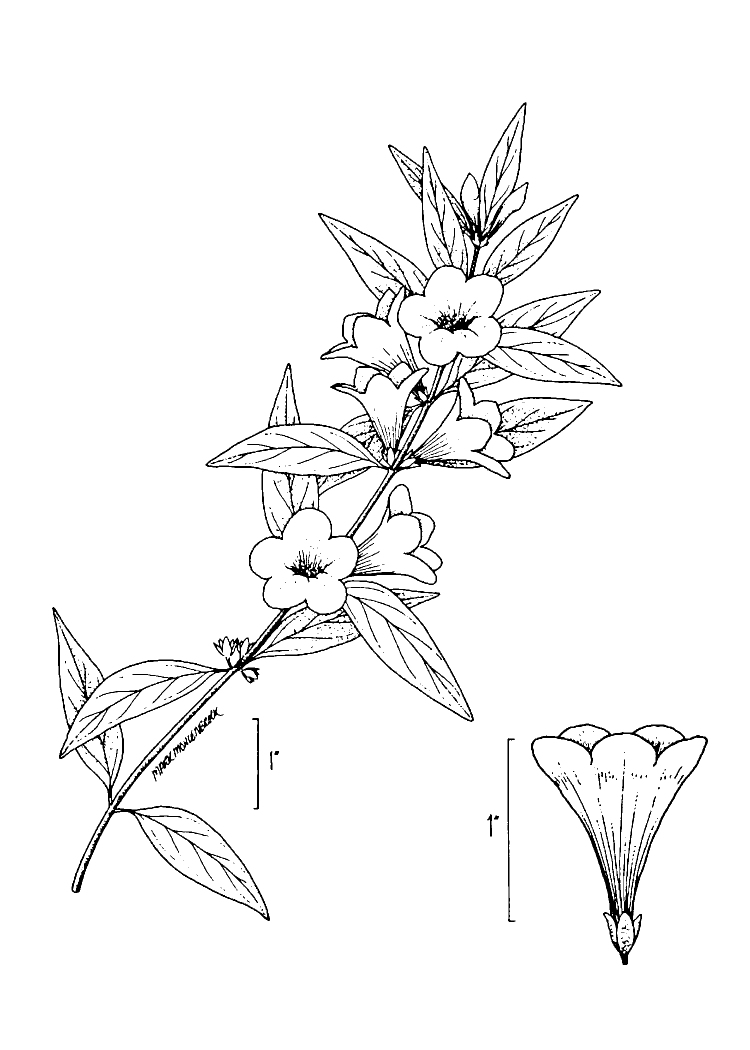
lllustration

**Figure 147c.**
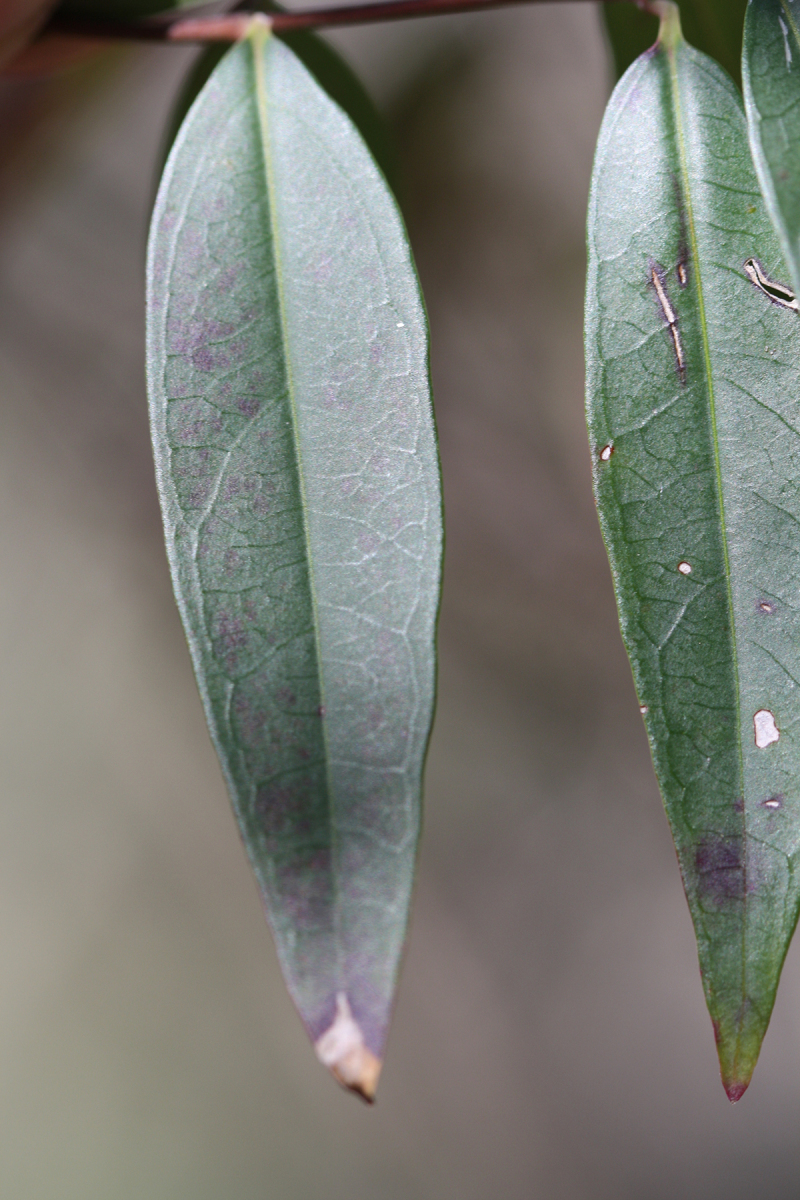
Leaves

**Figure 147d.**
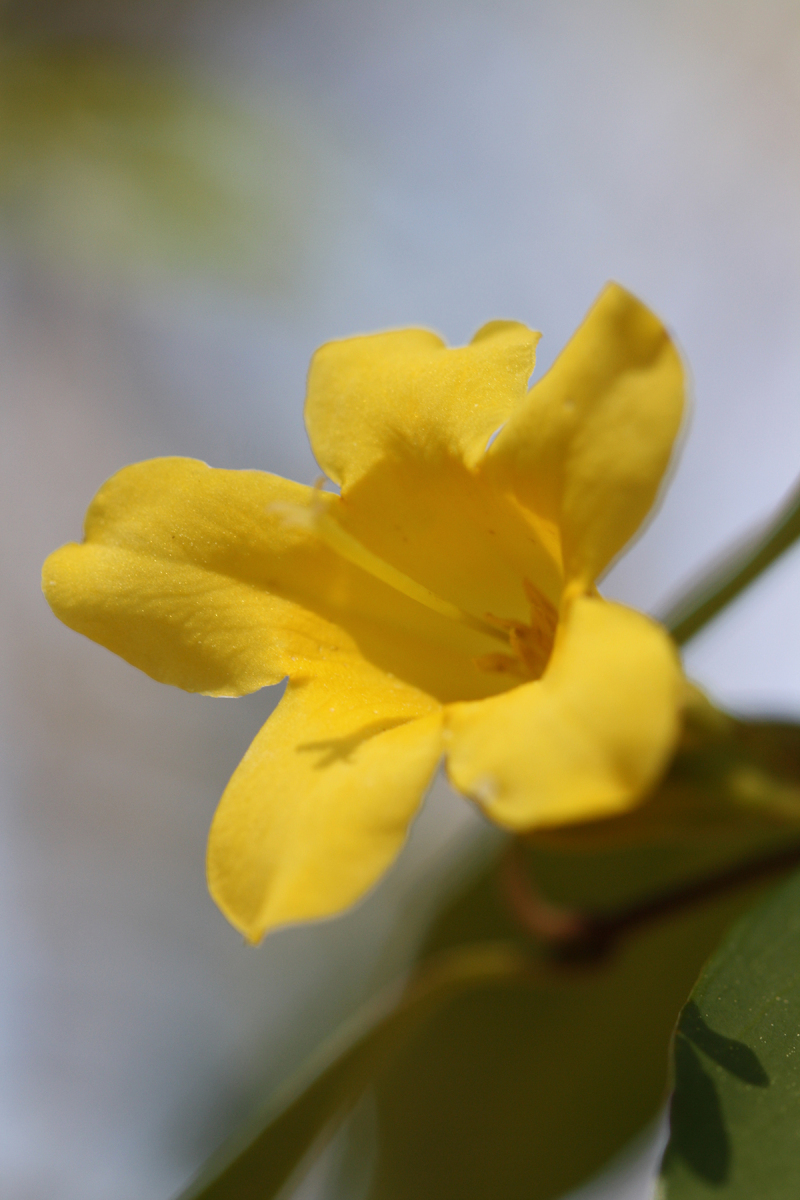
Flower

**Figure 147e.**
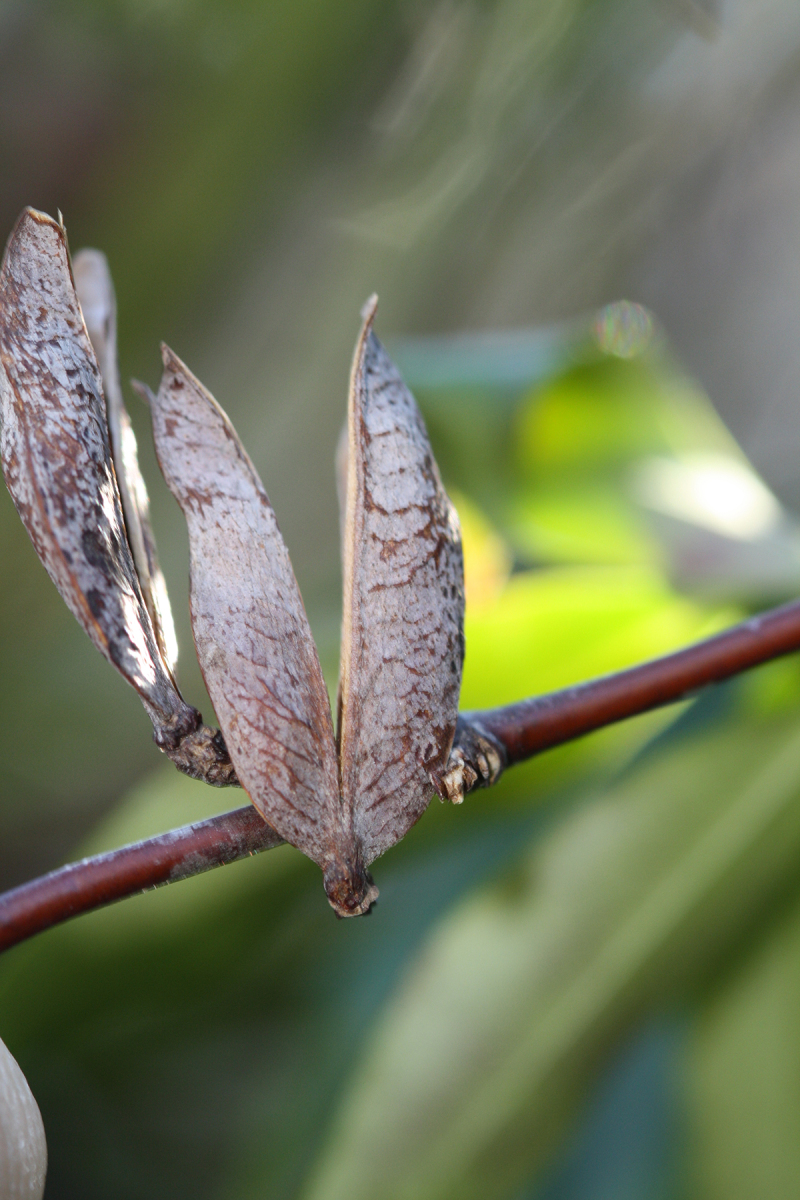
Fruit

**Figure 147f.**
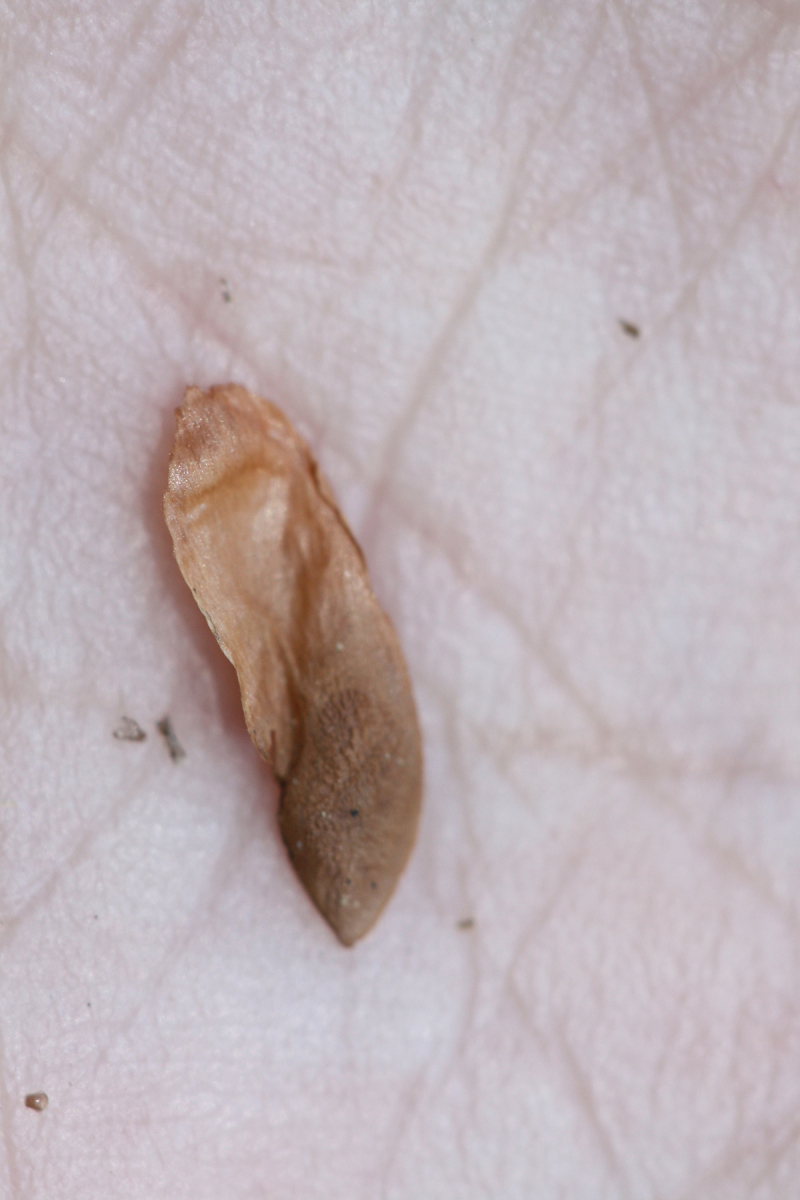
Seed

**Figure 148a.**
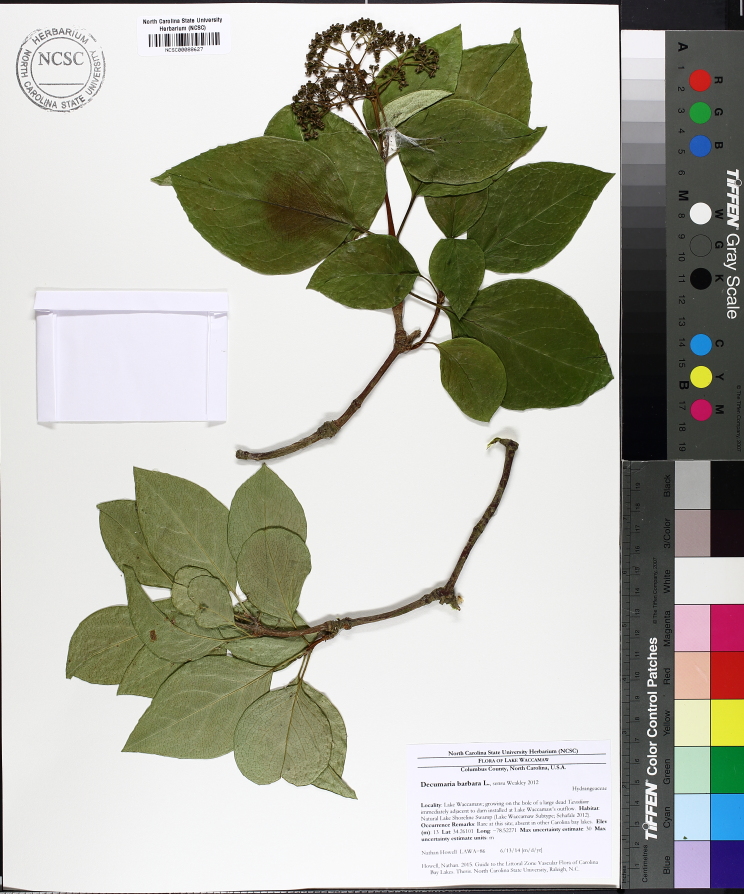
Specimen: *Howell LAWA-86* (NCSC)

**Figure 148b.**
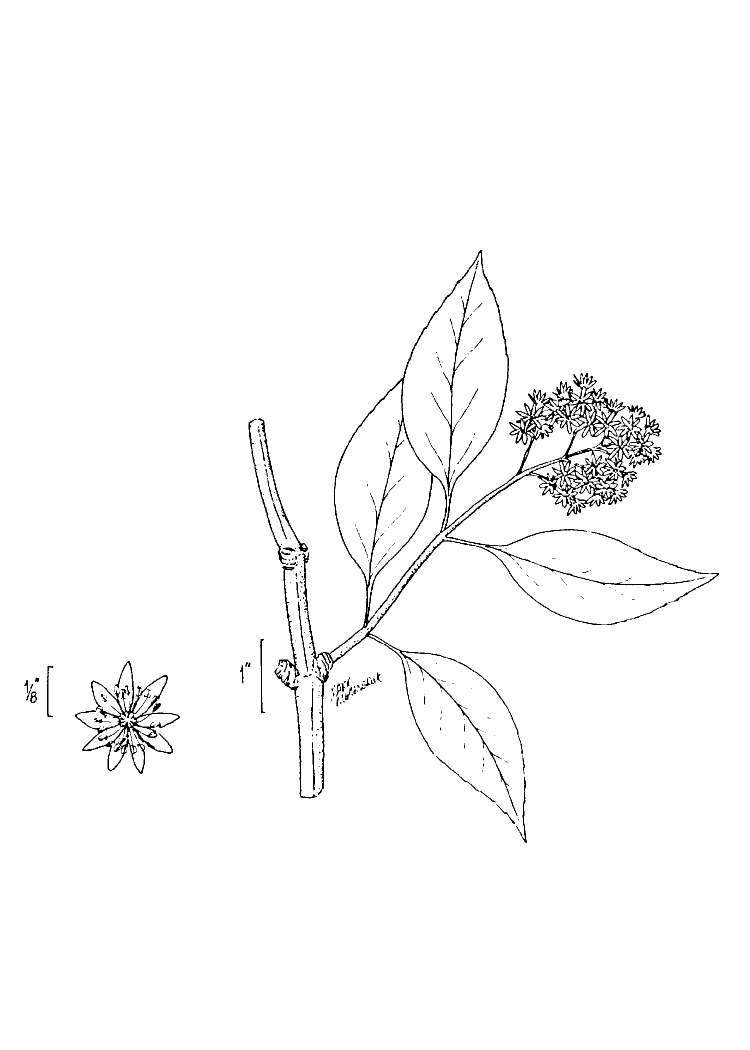
Illustration

**Figure 148c.**
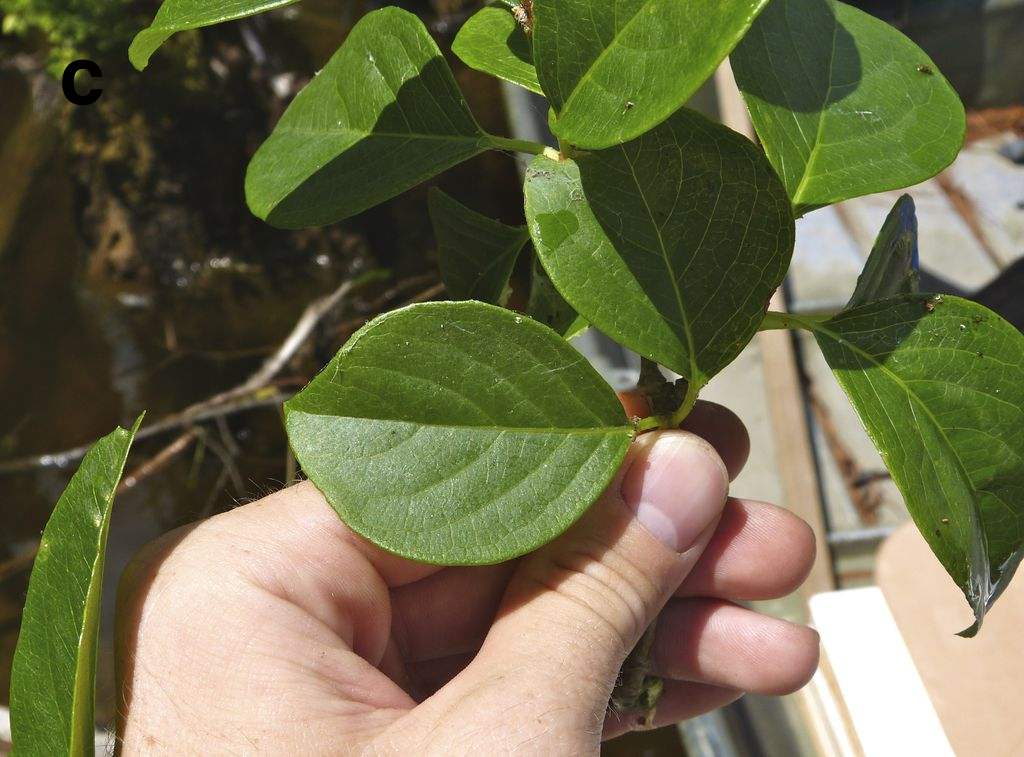
Leaves

**Figure 148d.**
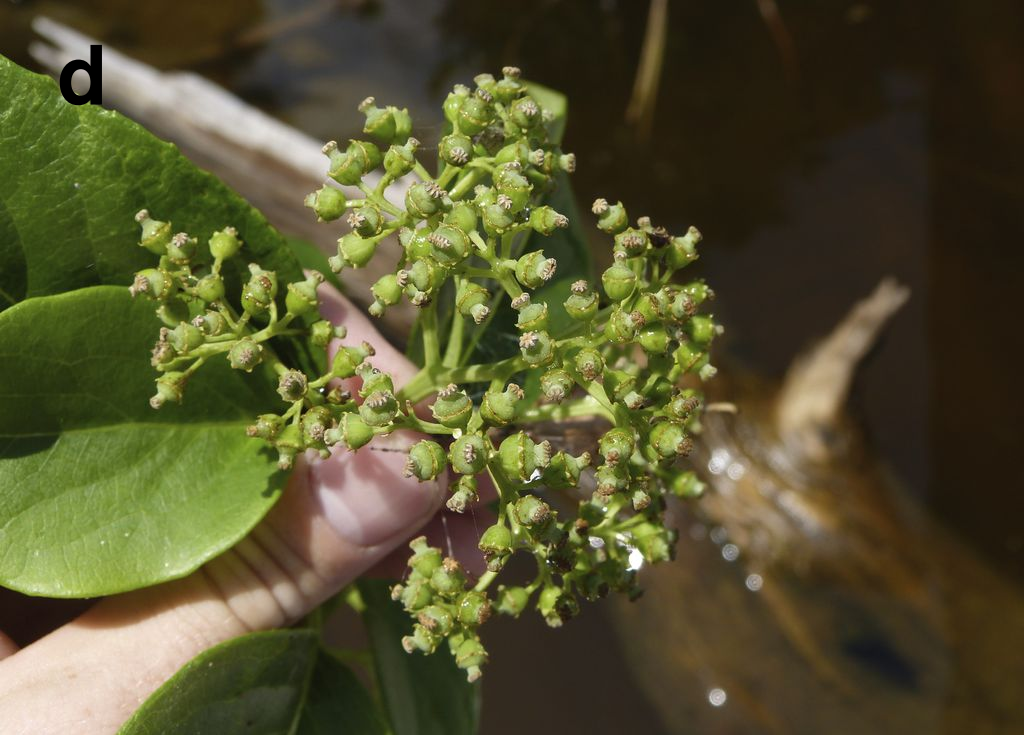
Fruits

**Figure 149a.**
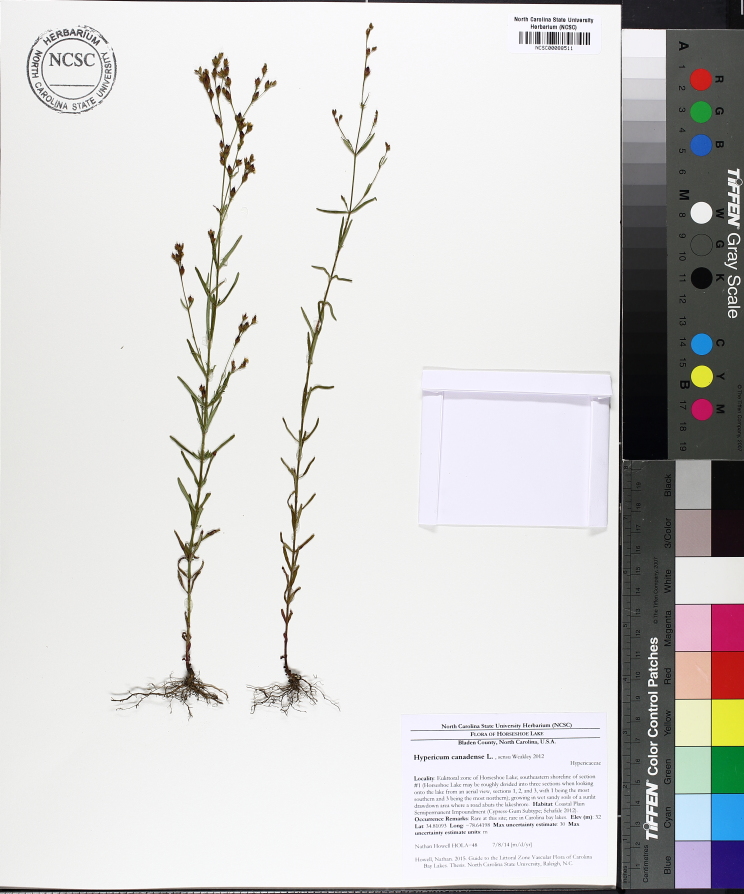
Specimen: *Howell HOLA-48* (NCSC)

**Figure 149b.**
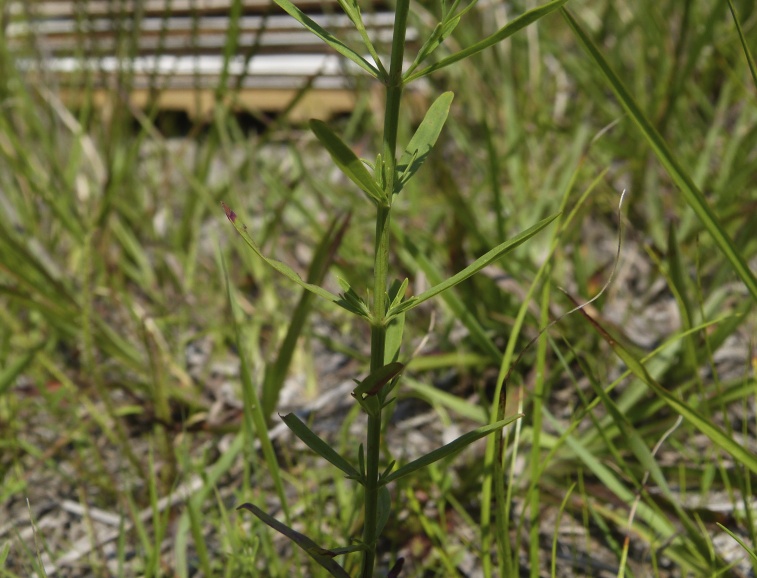
Stem and leaves

**Figure 149c.**
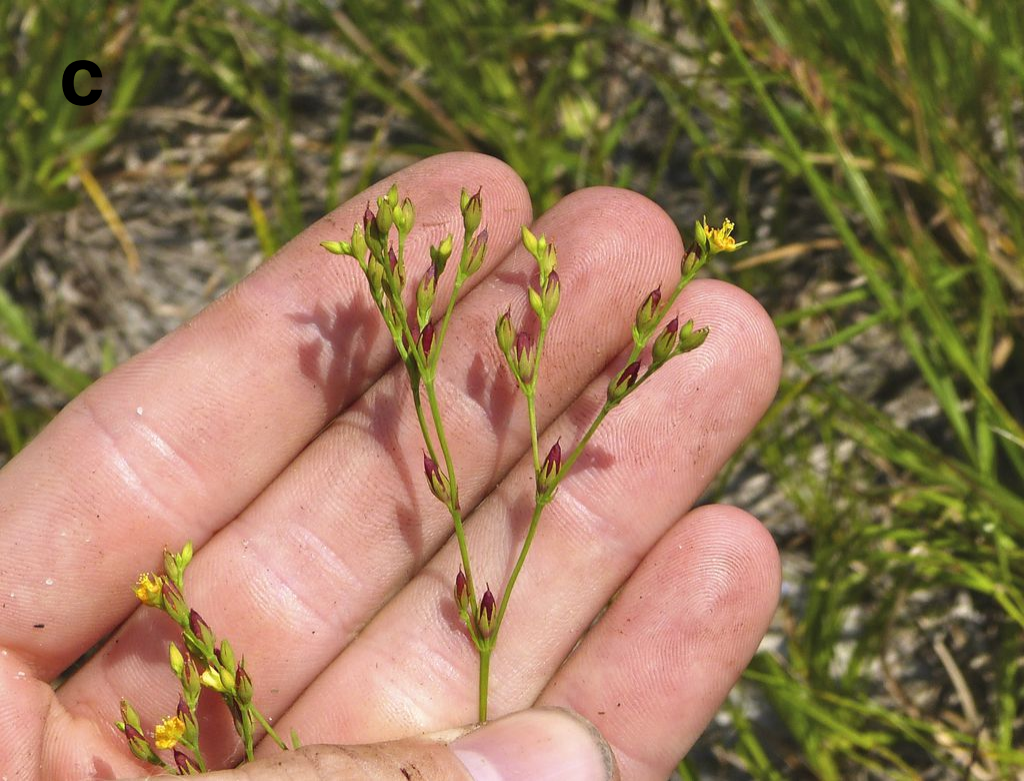
Inflorescence

**Figure 149d.**
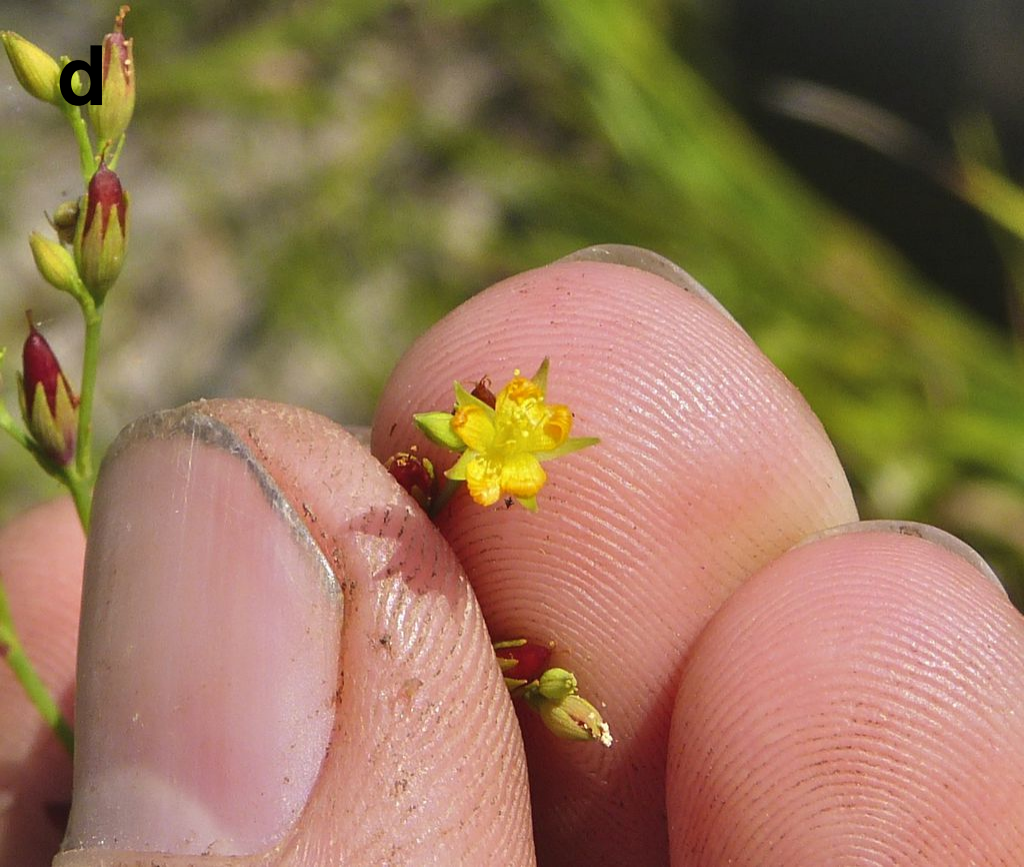
Flower

**Figure 150a.**
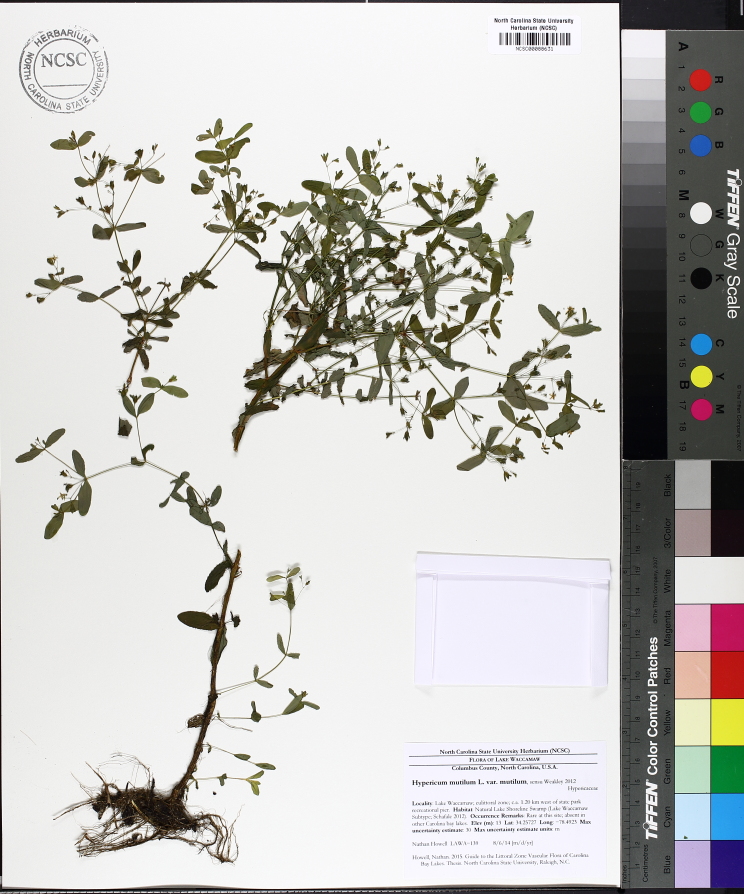
Specimen: *Howell LAWA-139* (NCSC)

**Figure 150b.**
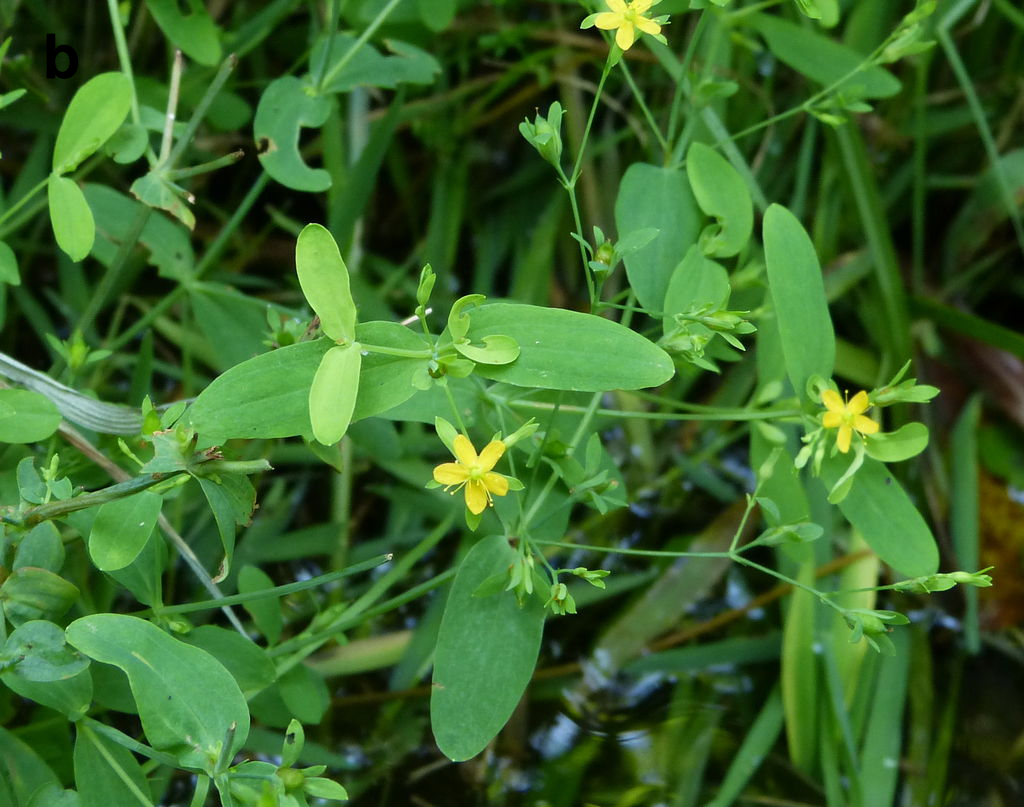
Habit

**Figure 150c.**
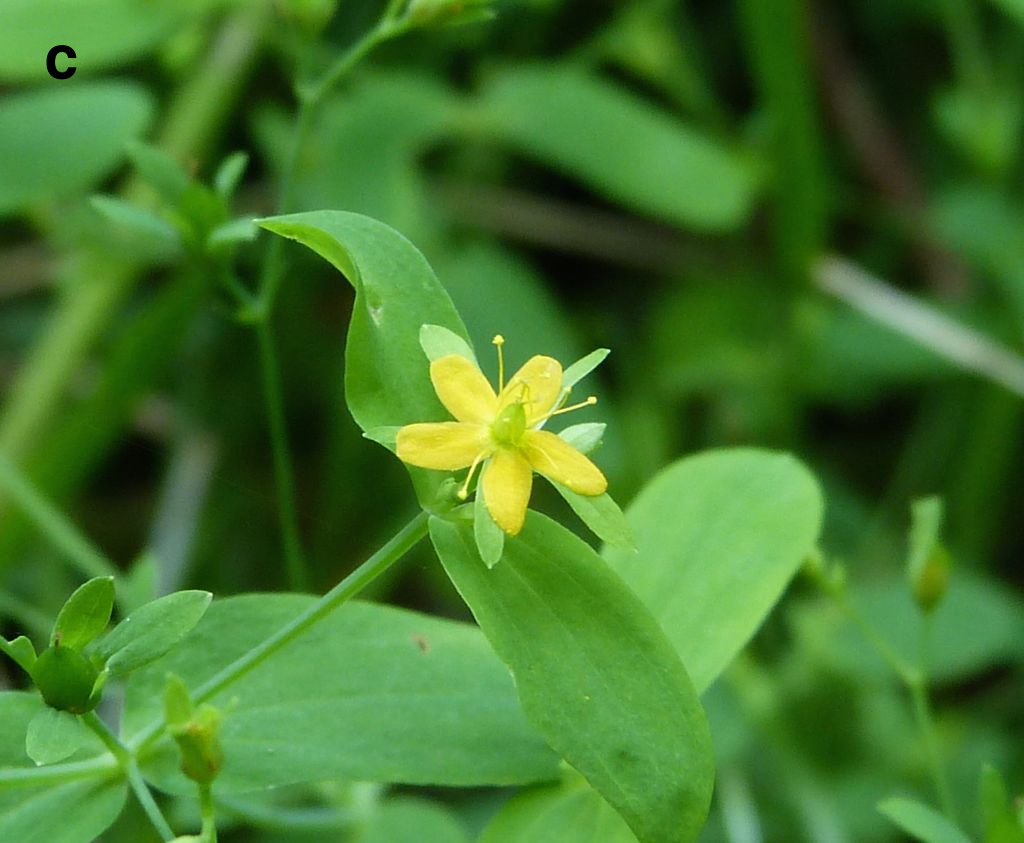
Flower (front)

**Figure 150d.**
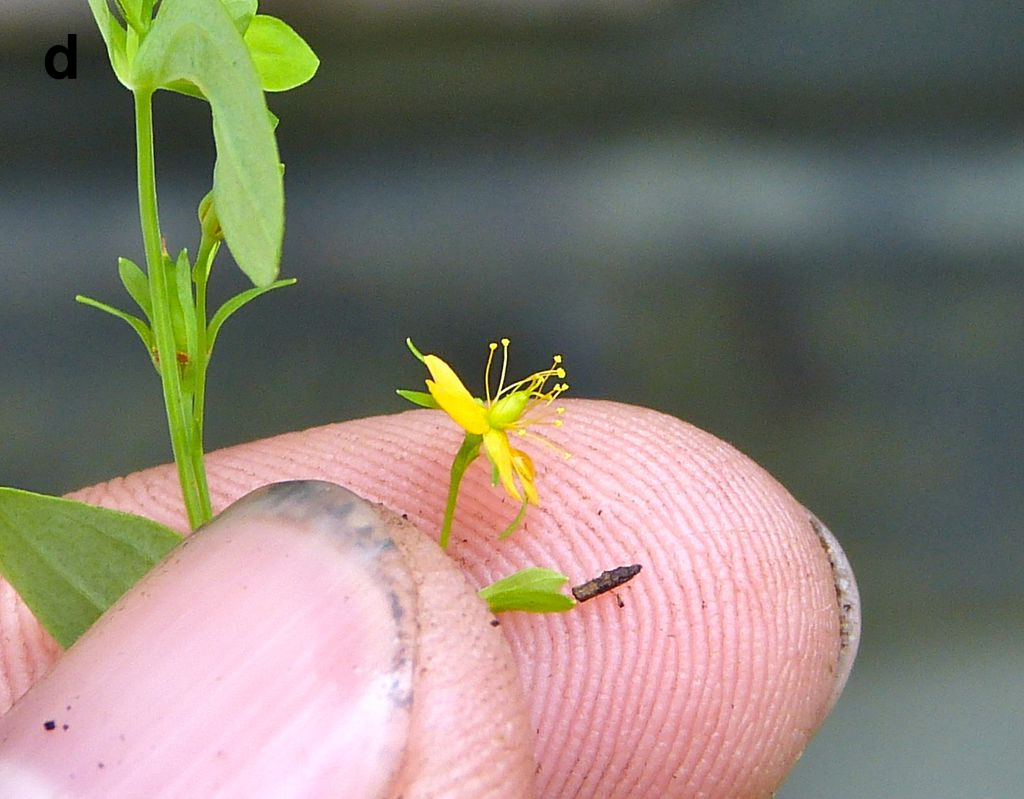
Flower (side)

**Figure 151a.**
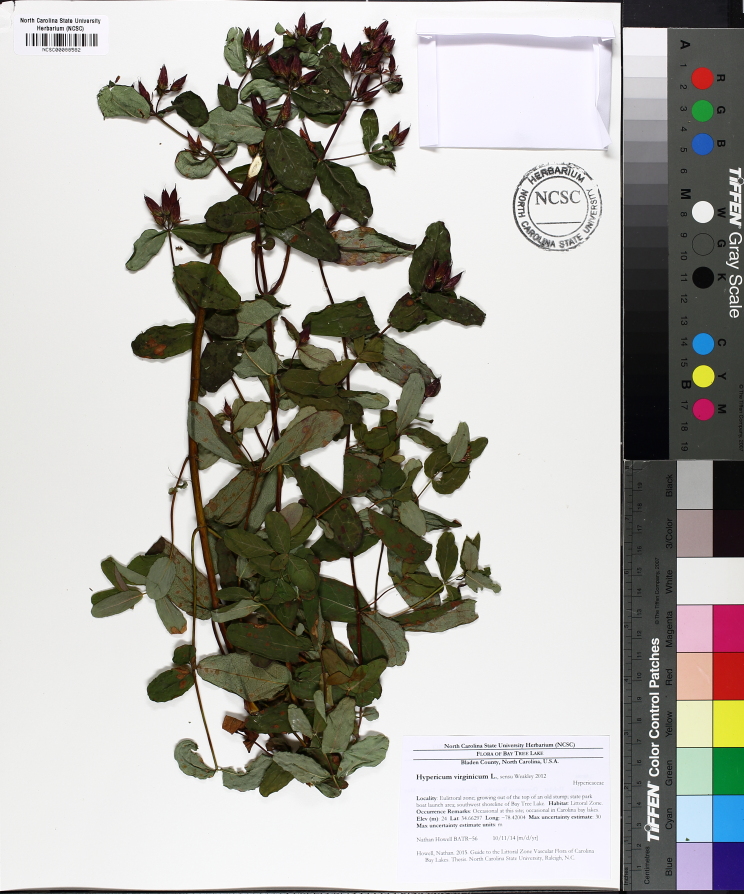
Specimen: *Howell BATR-56* (NCSC)

**Figure 151b.**
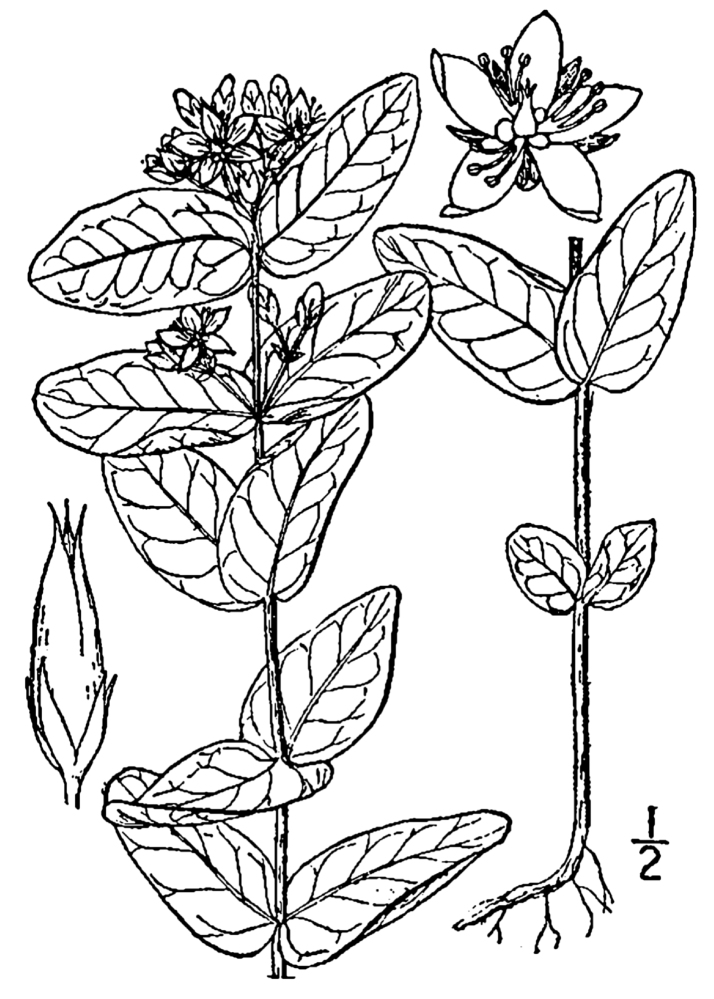
Illustration

**Figure 151c.**
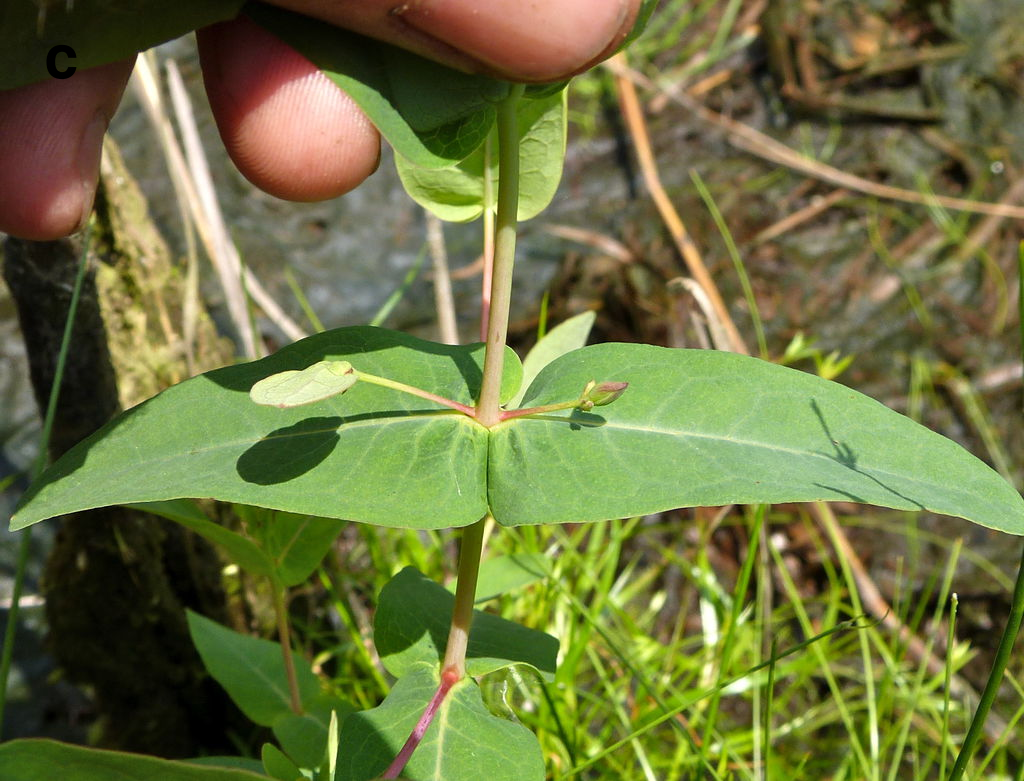
Leaves

**Figure 151d.**
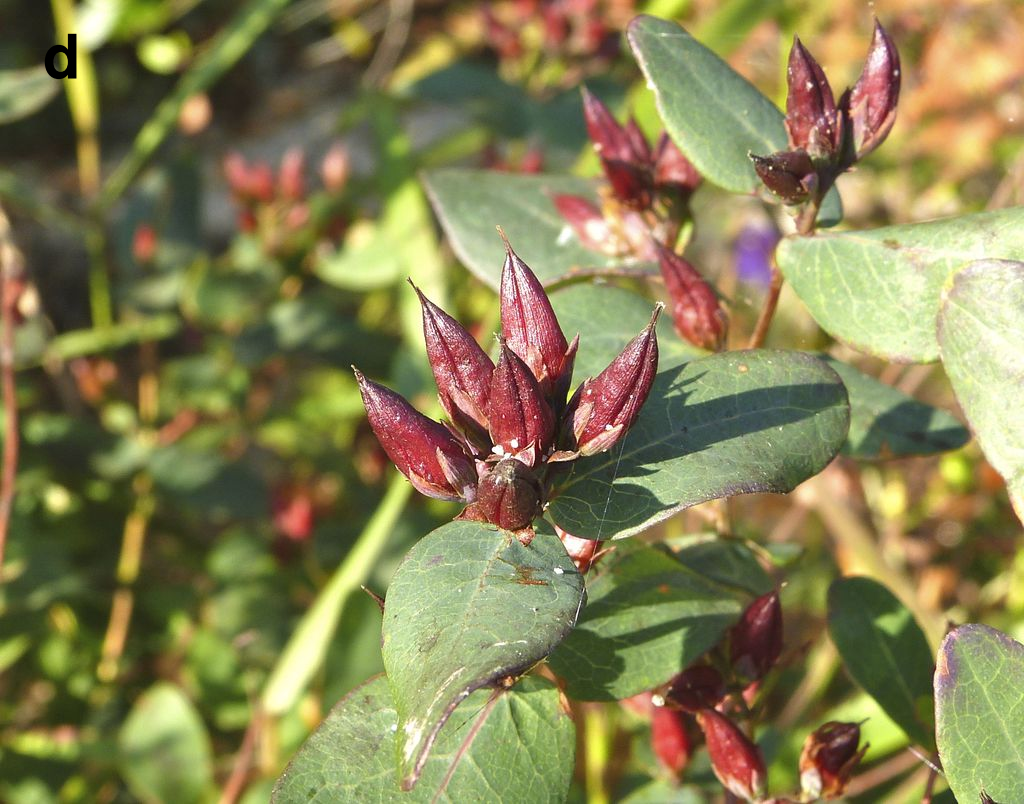
Infructescence

**Figure 152a.**
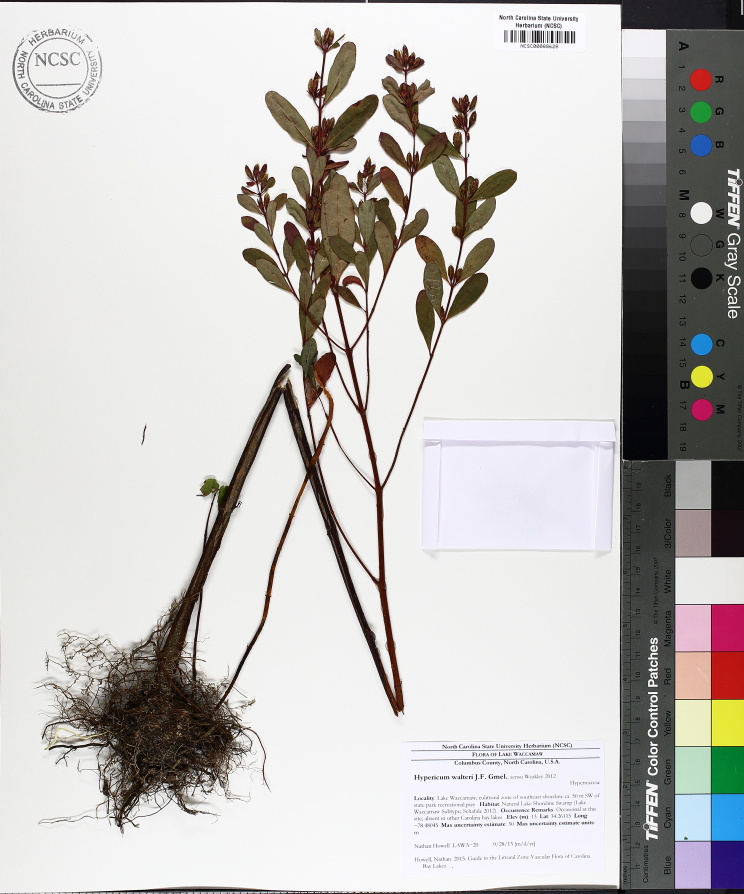
Specimen: *Howell LAWA-20* (NCSC)

**Figure 152b.**
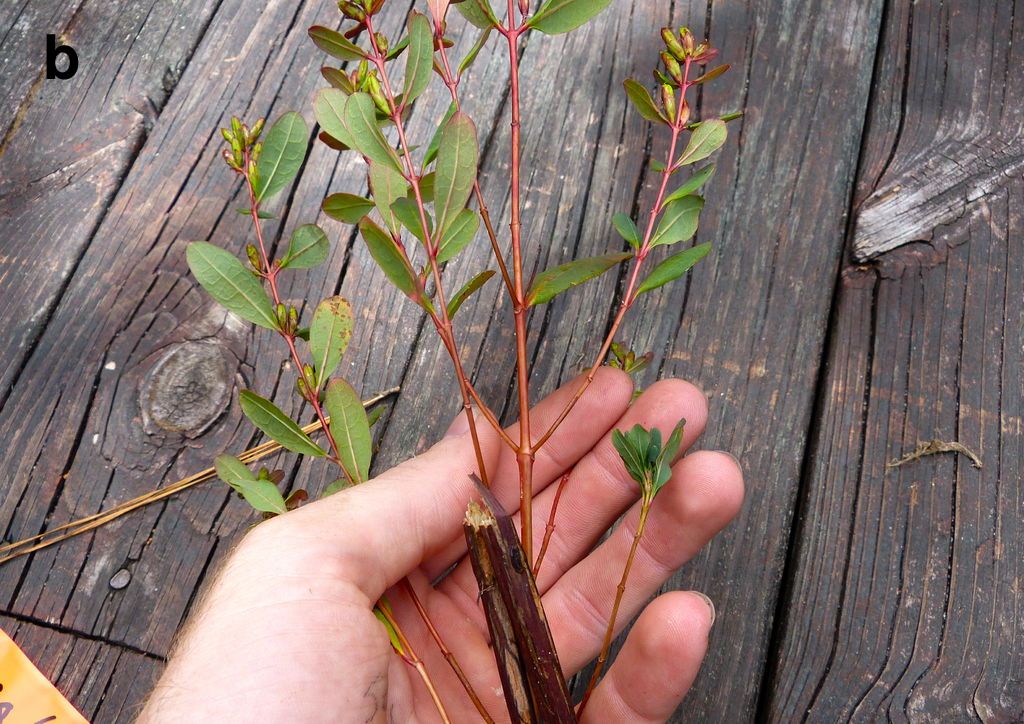
Habit

**Figure 152c.**
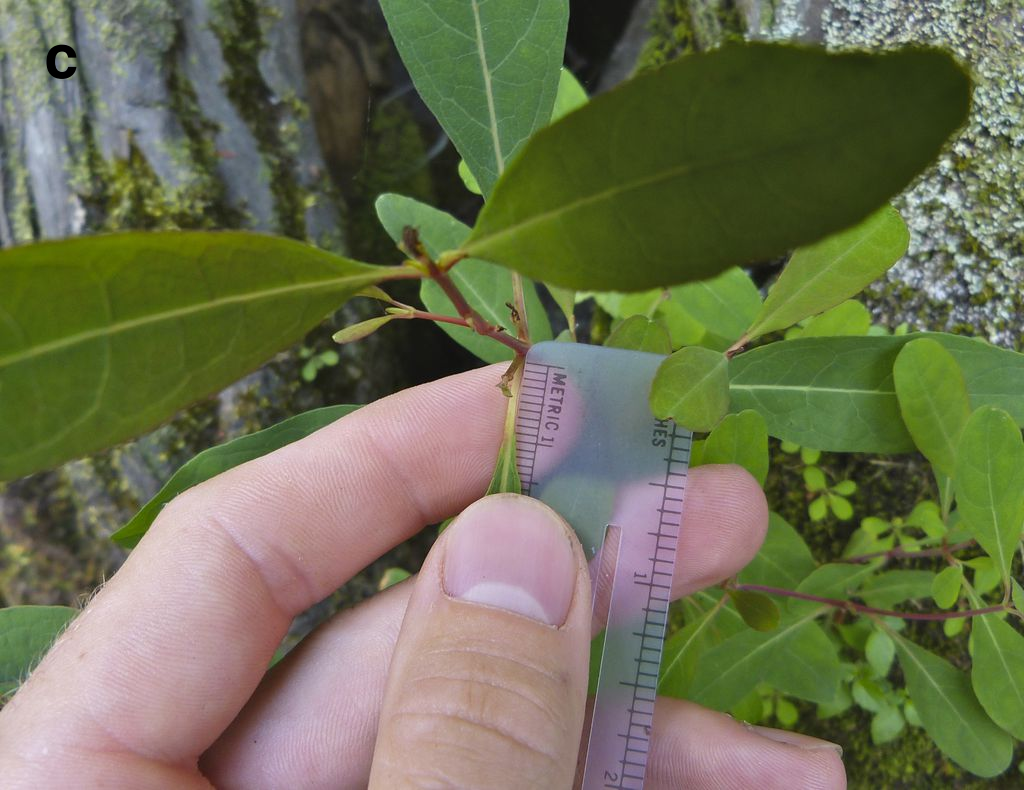
Leaves, showing distinct petioles

**Figure 152d.**
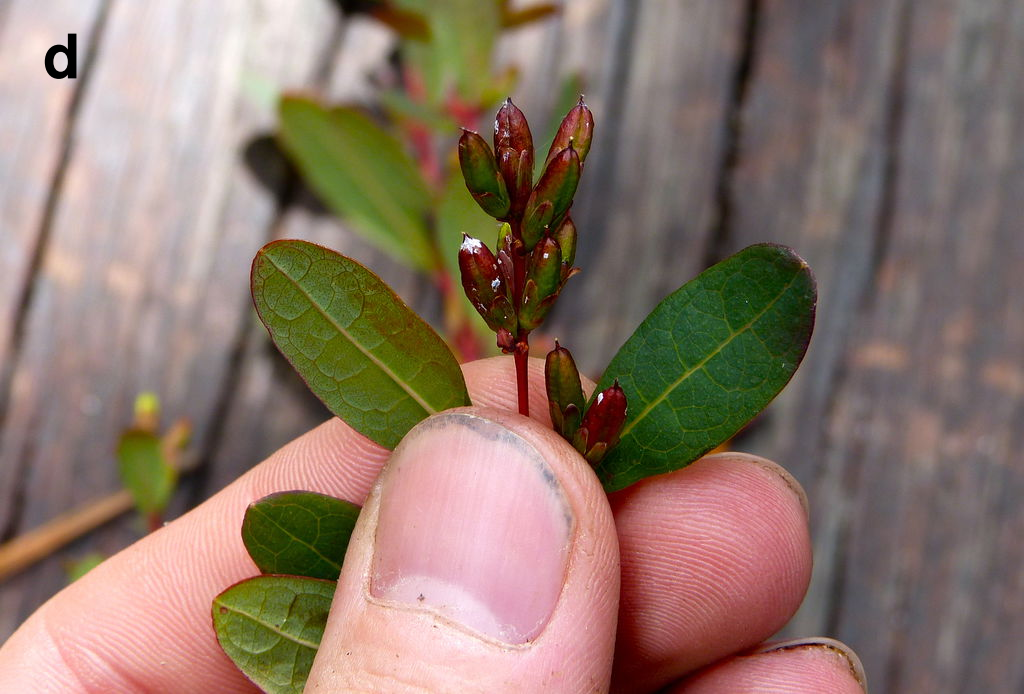
Infructescence

**Figure 153a.**
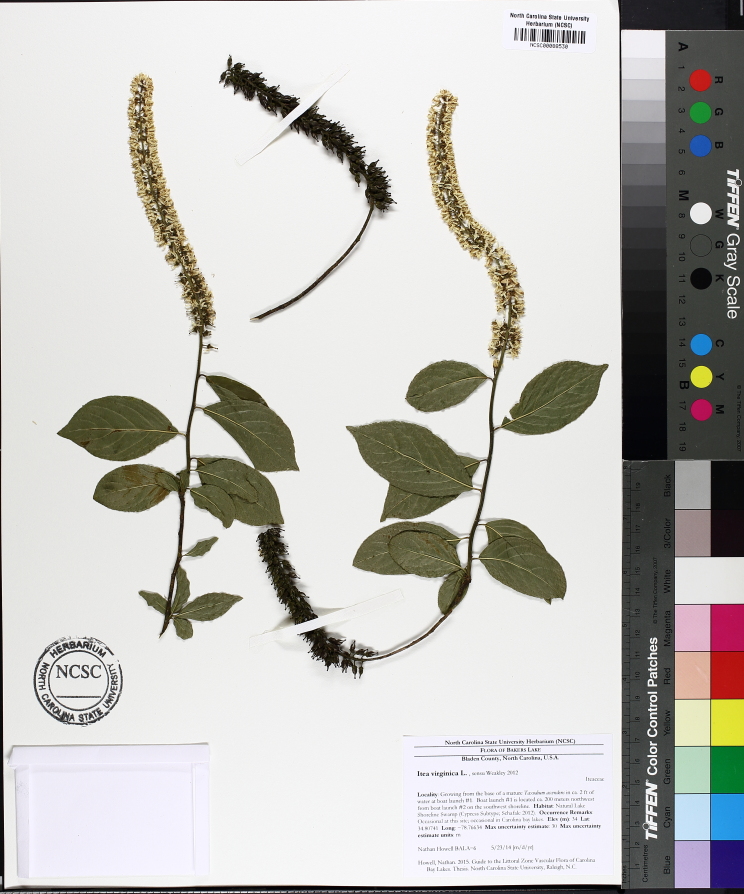
Specimen: *Howell BALA-6* (NCSC)

**Figure 153b.**
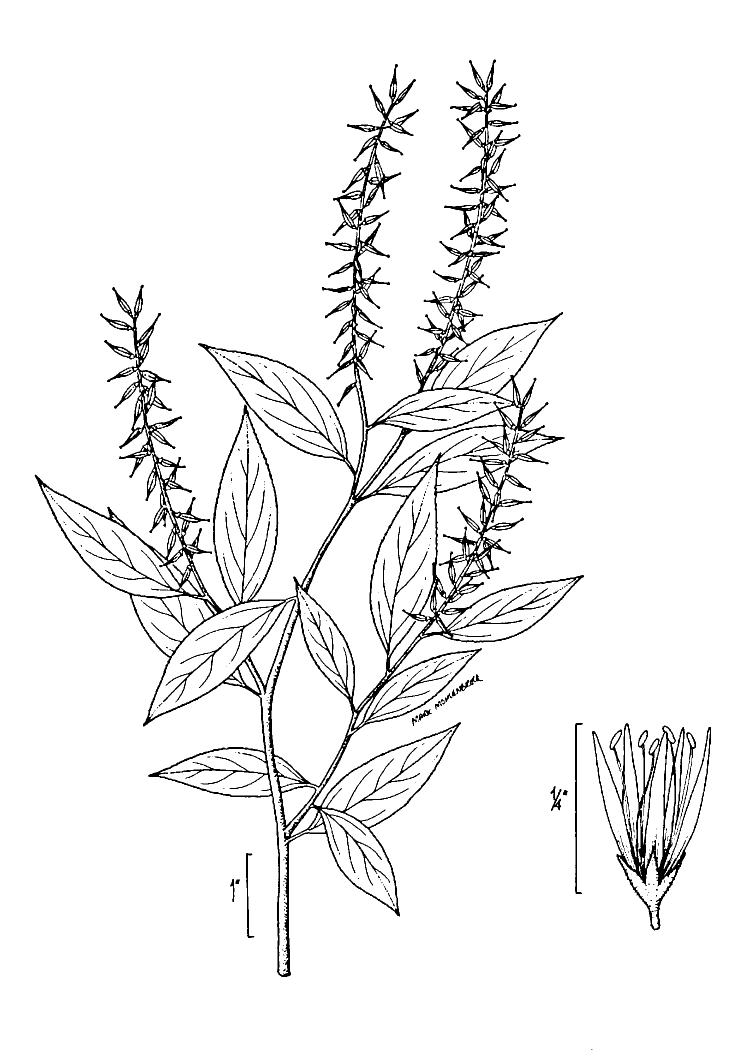
Illustration

**Figure 153c.**
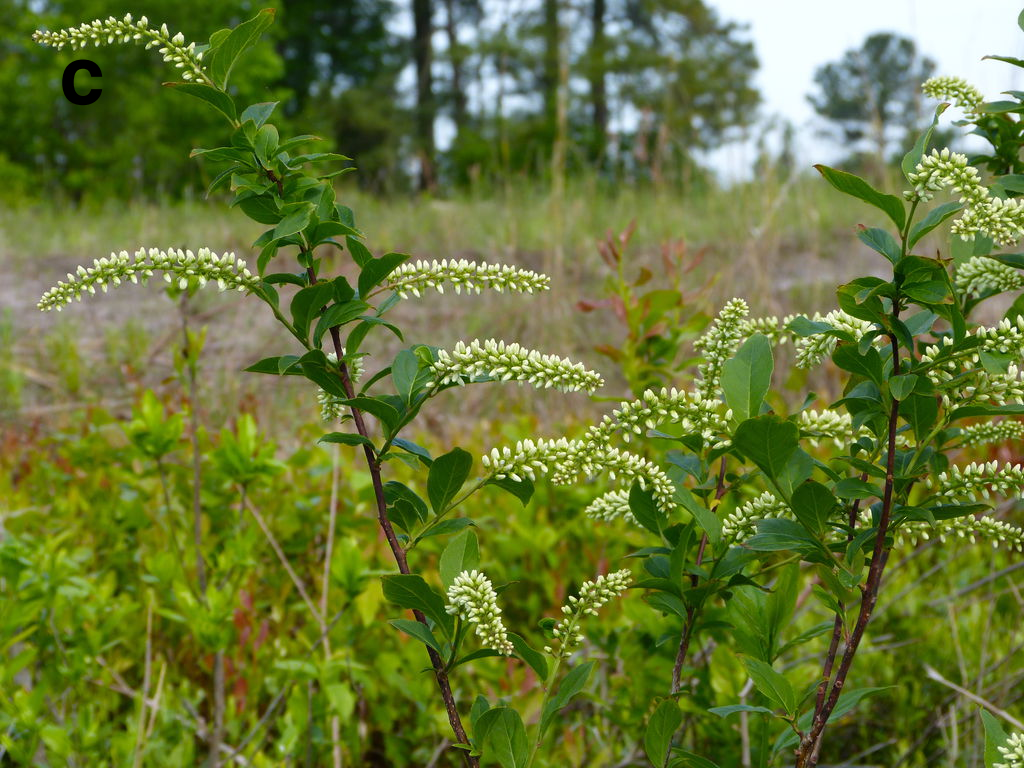
Habit

**Figure 153d.**
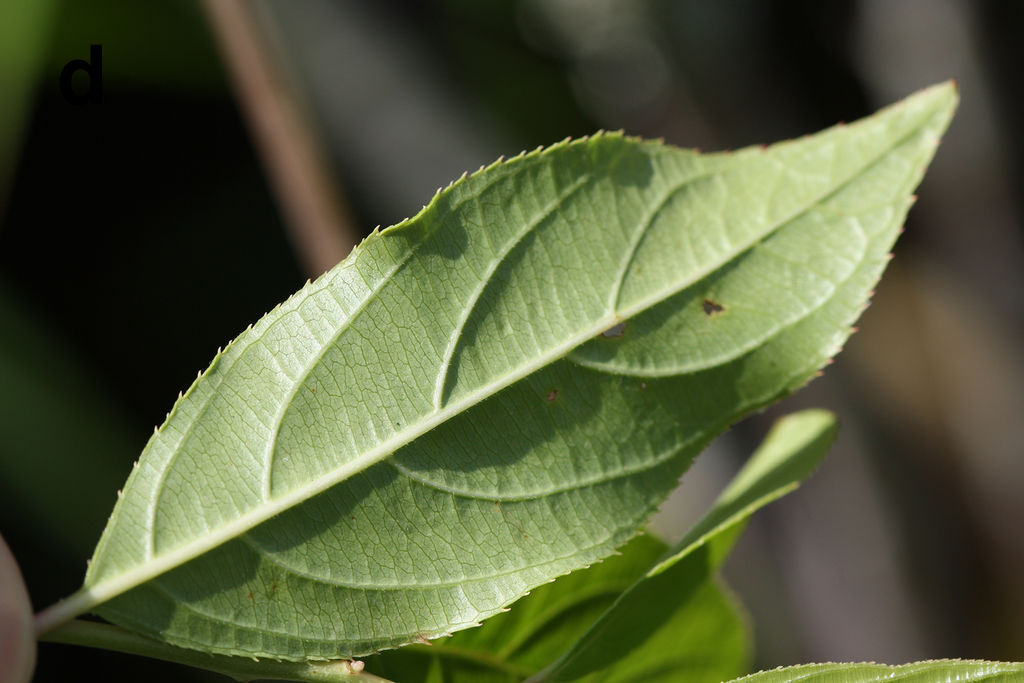
Leaf

**Figure 153e.**
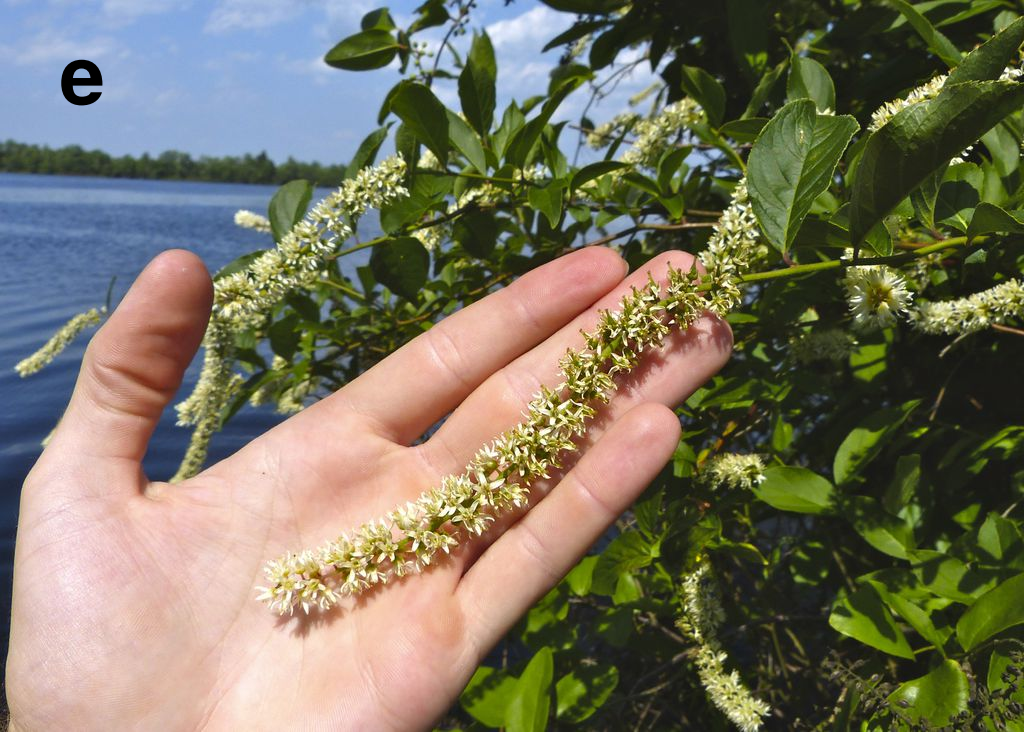
Inflorescence

**Figure 153f.**
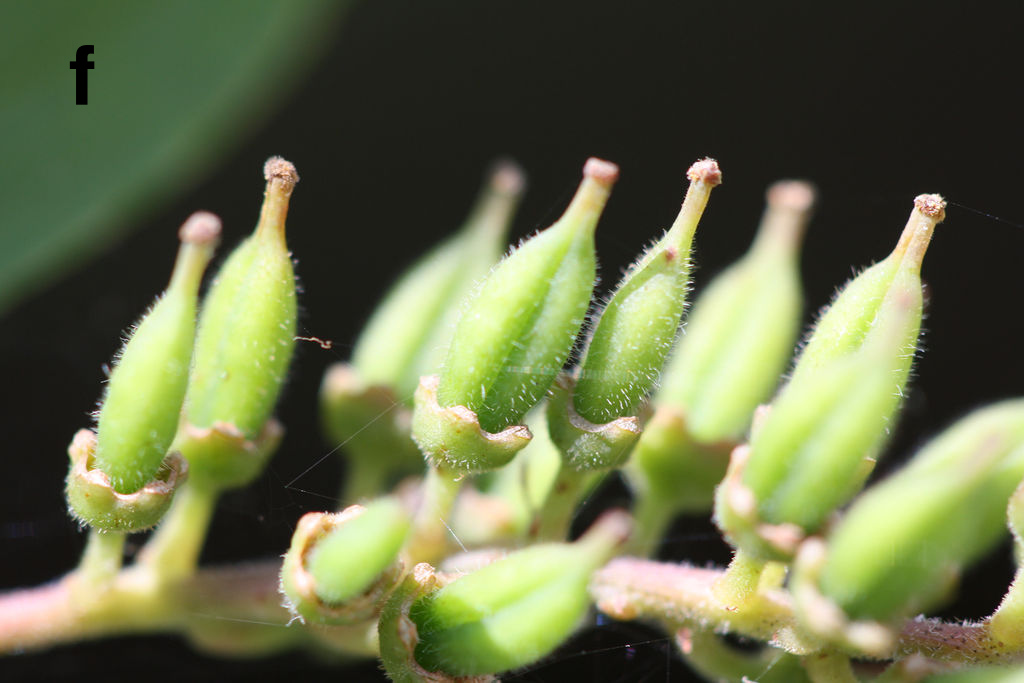
Fruits

**Figure 154a.**
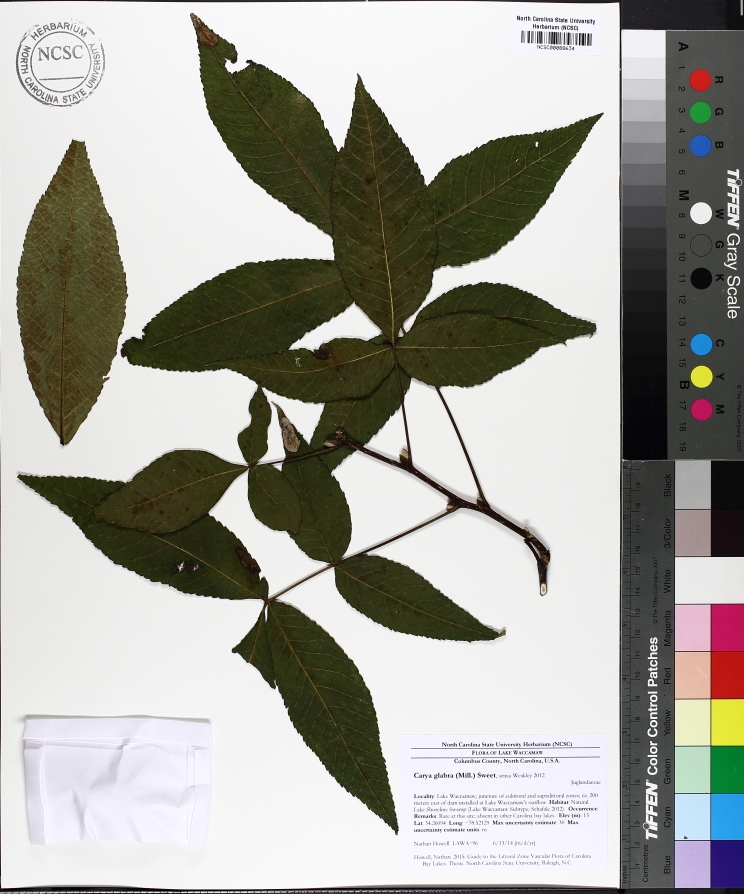
Specimen: *Howell LAWA-96* (NCSC)

**Figure 154b.**
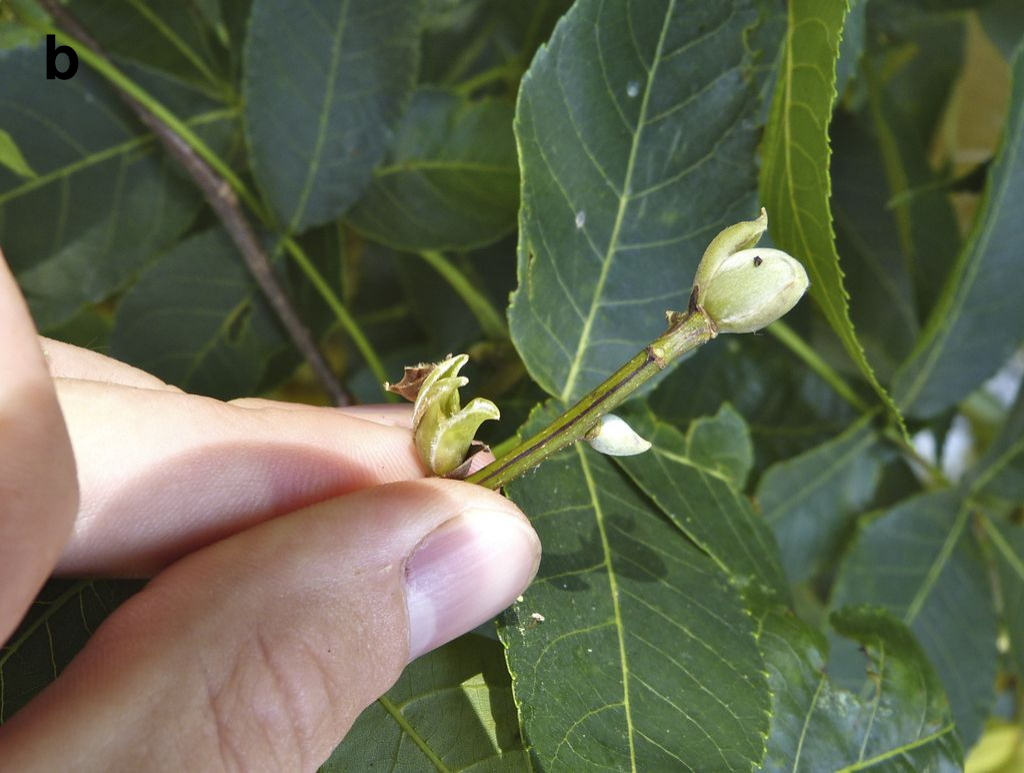
Stem and buds

**Figure 154c.**
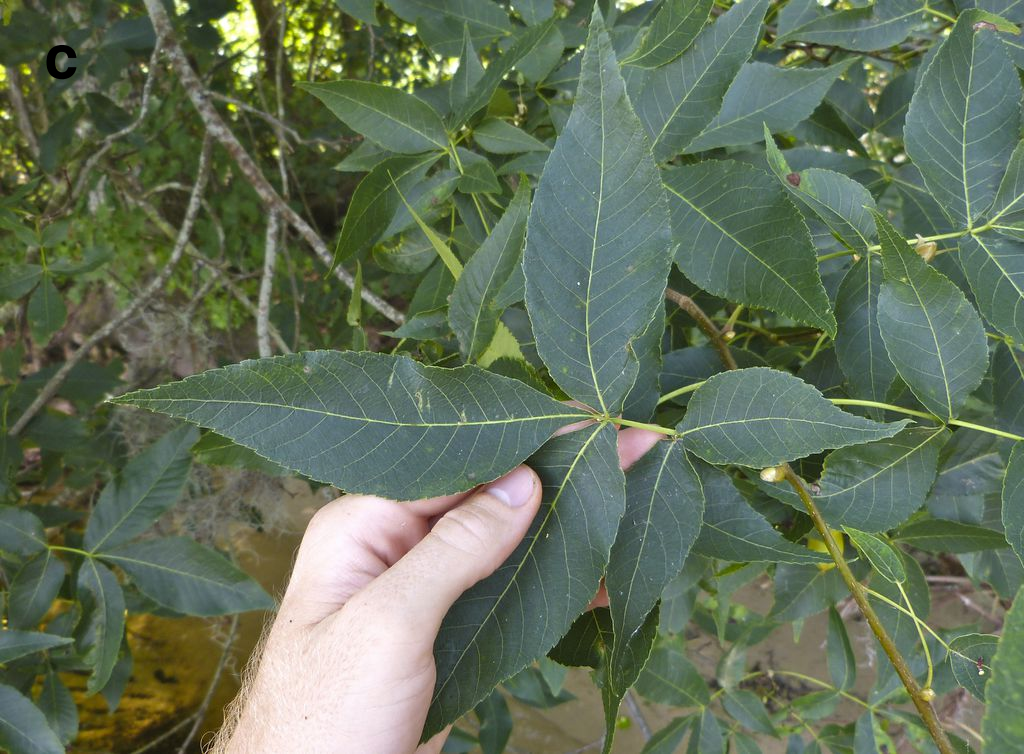
Imparipinnate leaf

**Figure 154d.**
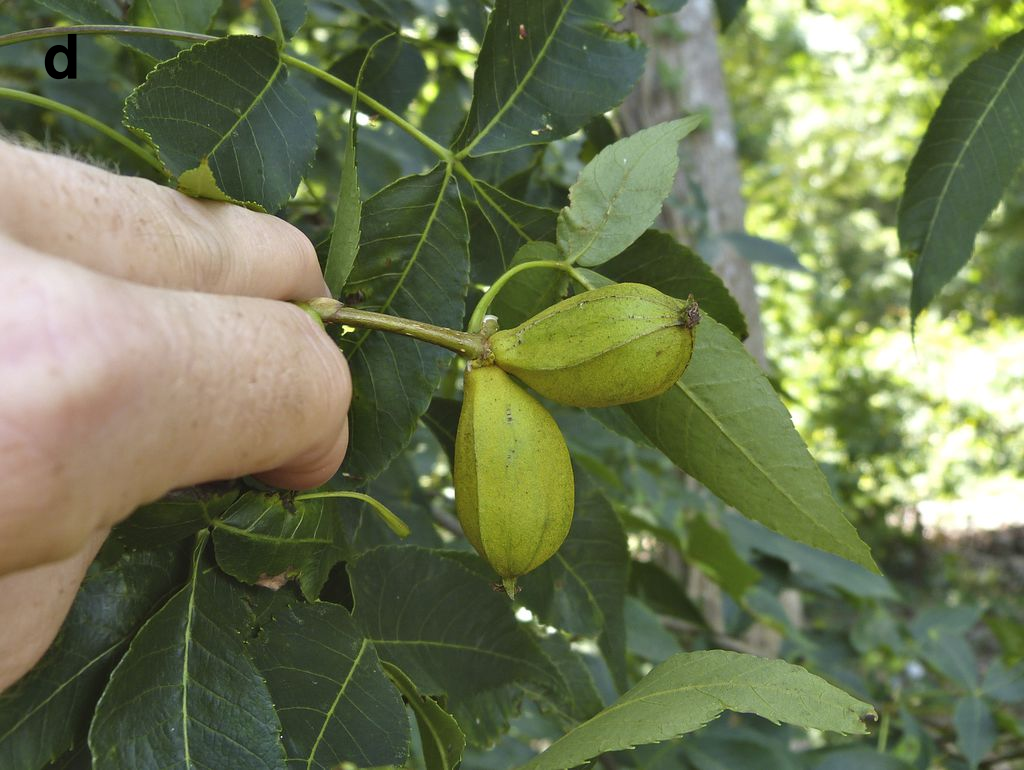
Fruits

**Figure 155a.**
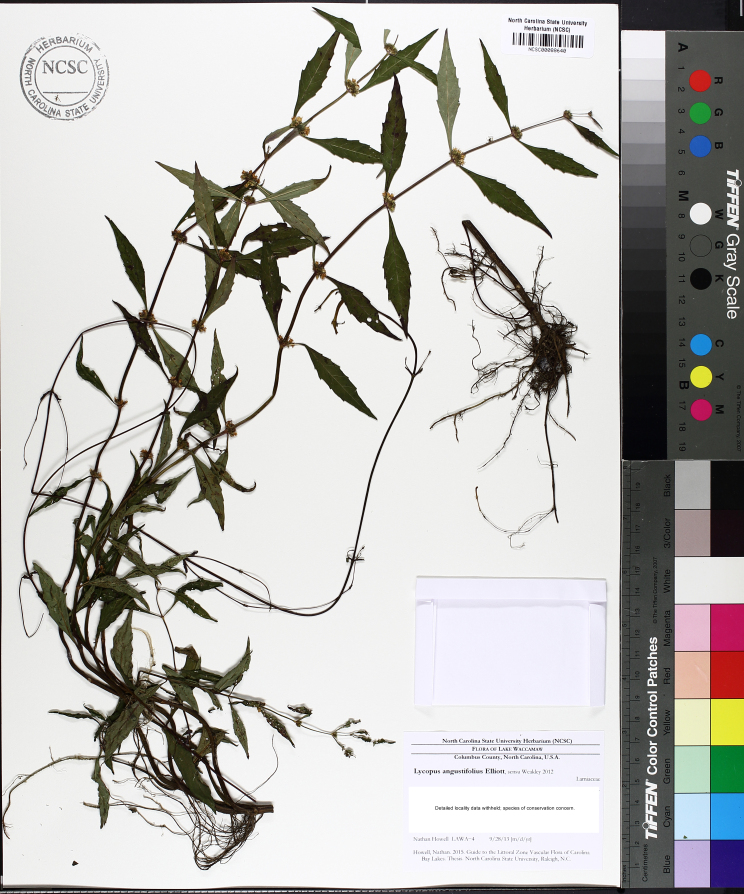
Specimen: *Howell LAWA-4* (NCSC)

**Figure 155b.**
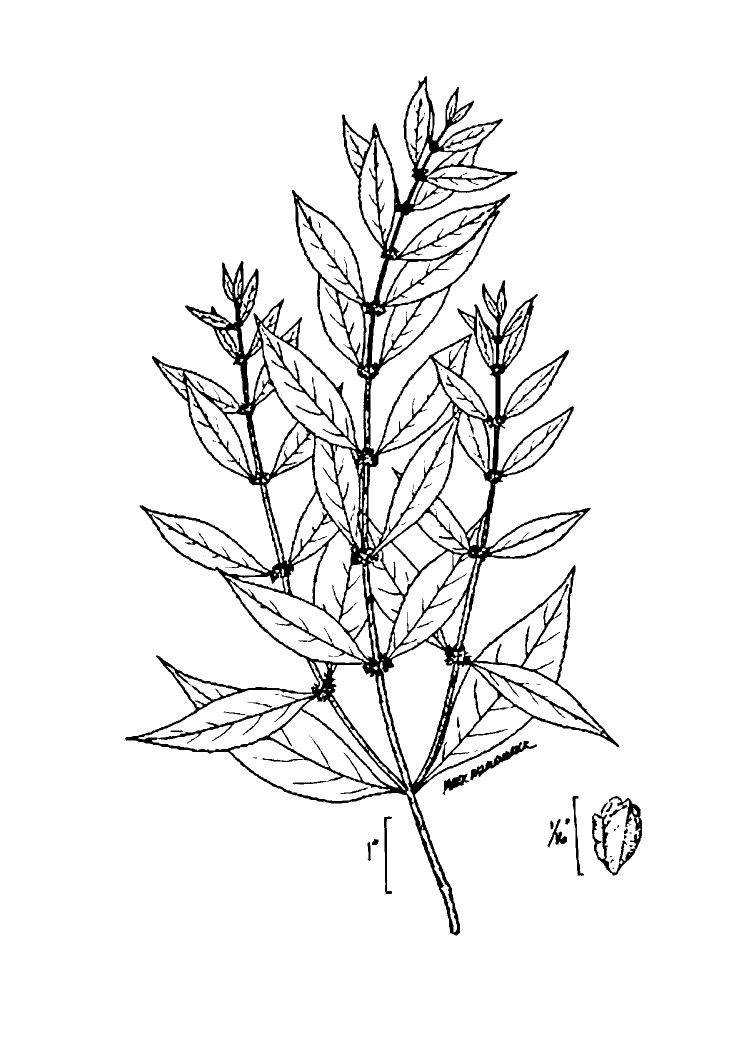
Illustration

**Figure 155c.**
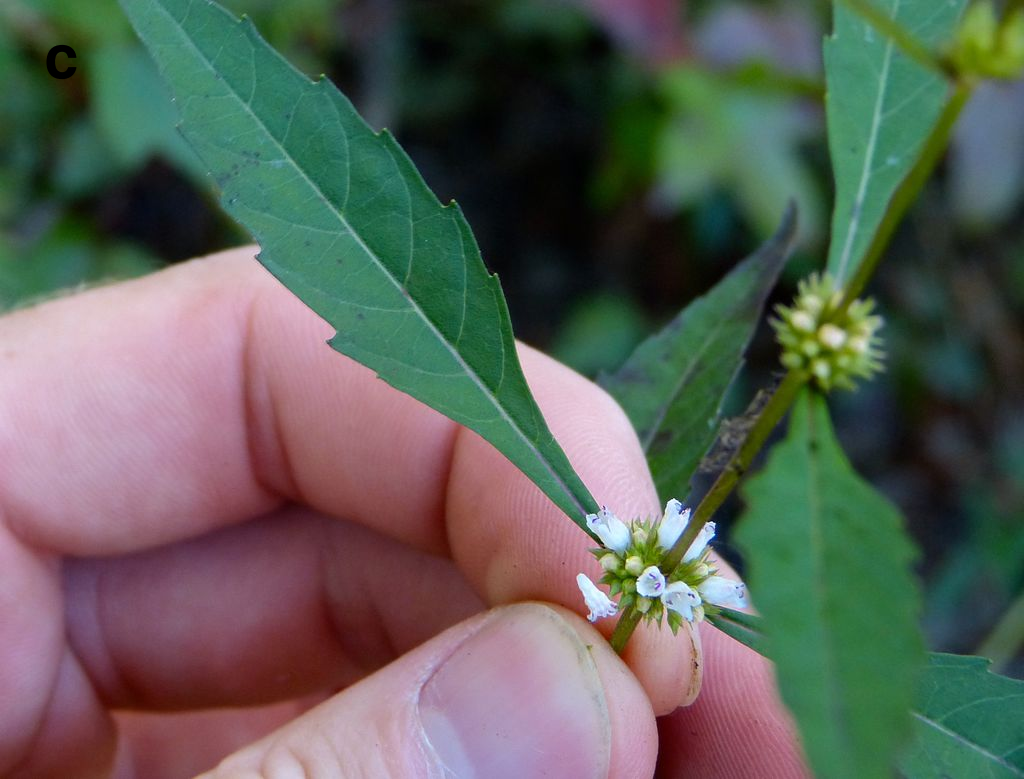
Flowers

**Figure 155d.**
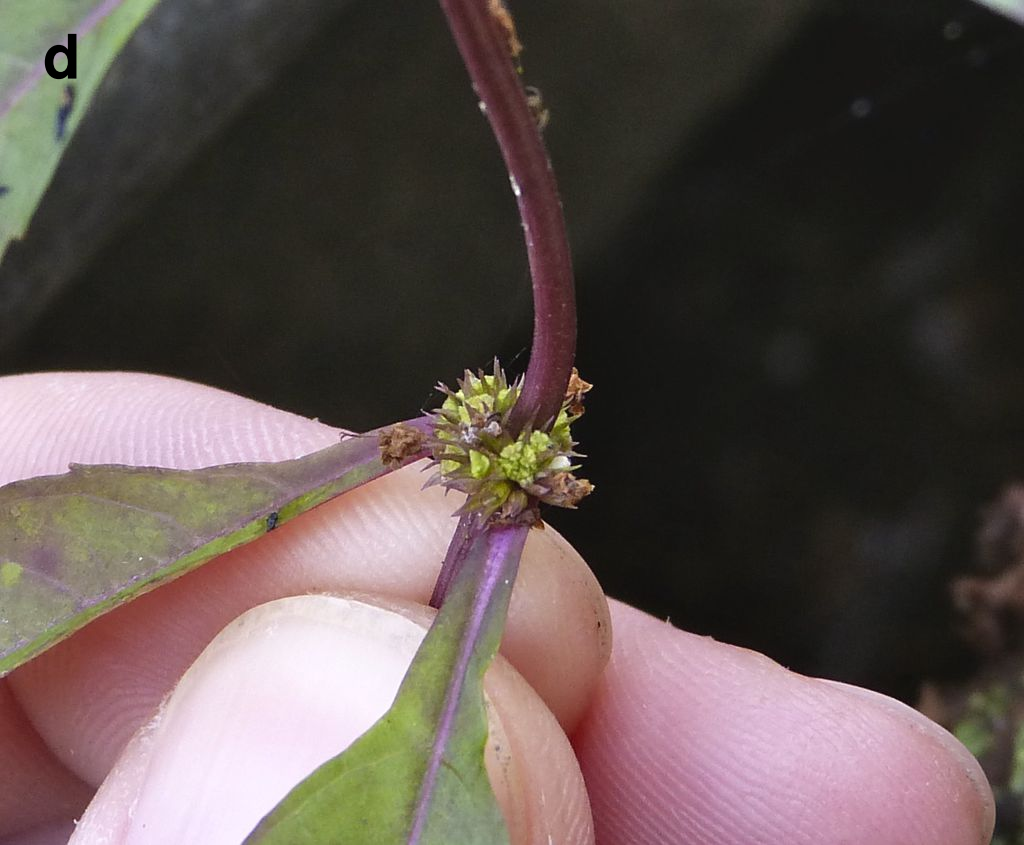
Fruits

**Figure 156a.**
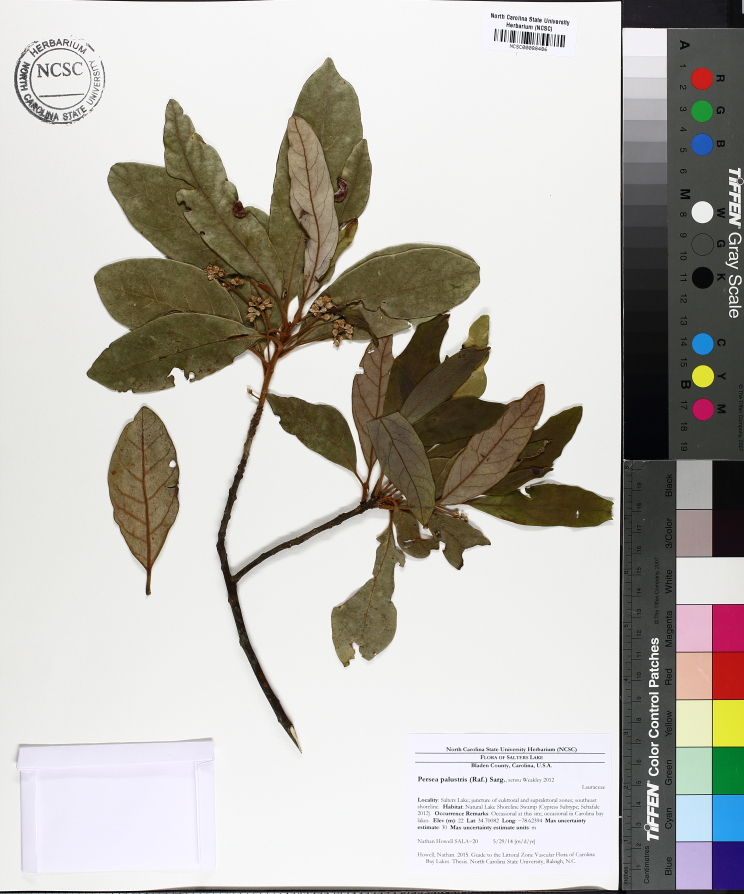
Specimen: *Howell SALA-20* (NCSC)

**Figure 156b.**
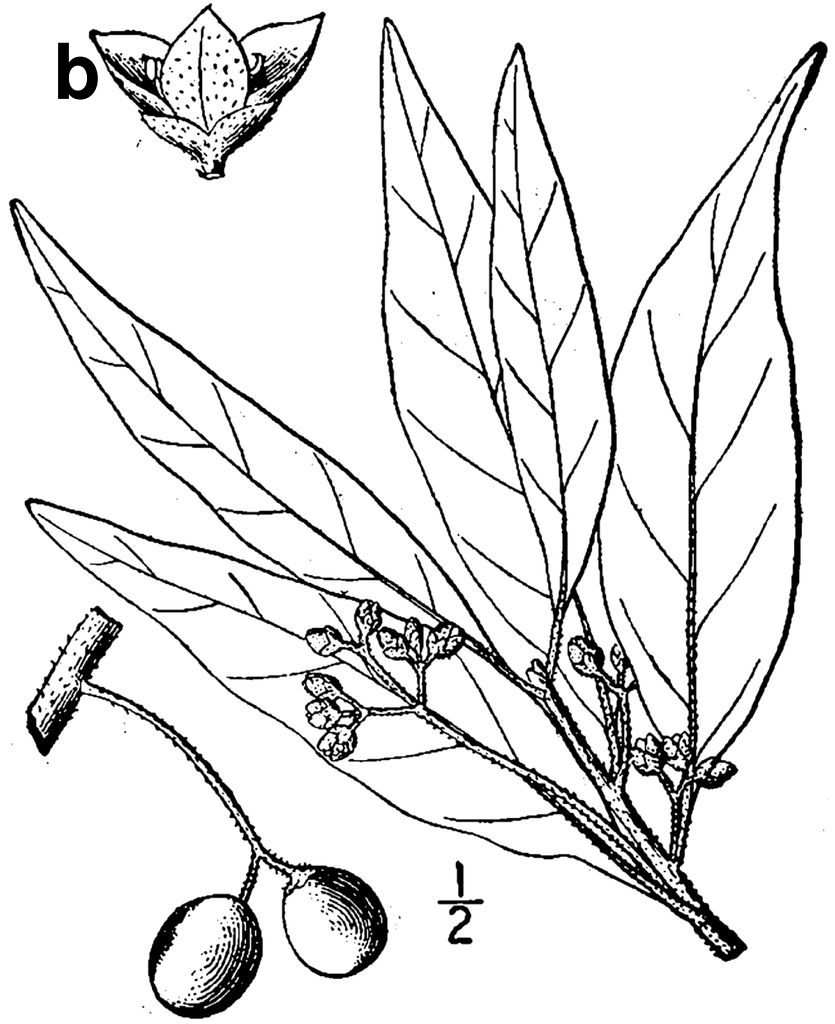
Illustration

**Figure 156c.**
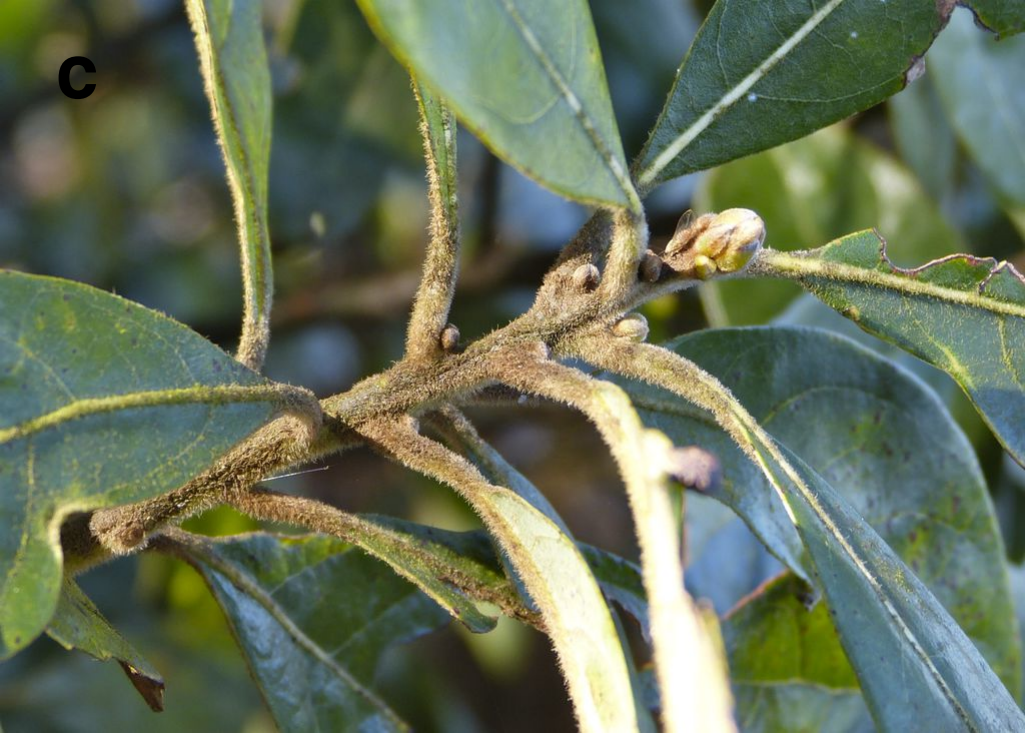
Twig (note pubescence)

**Figure 156d.**
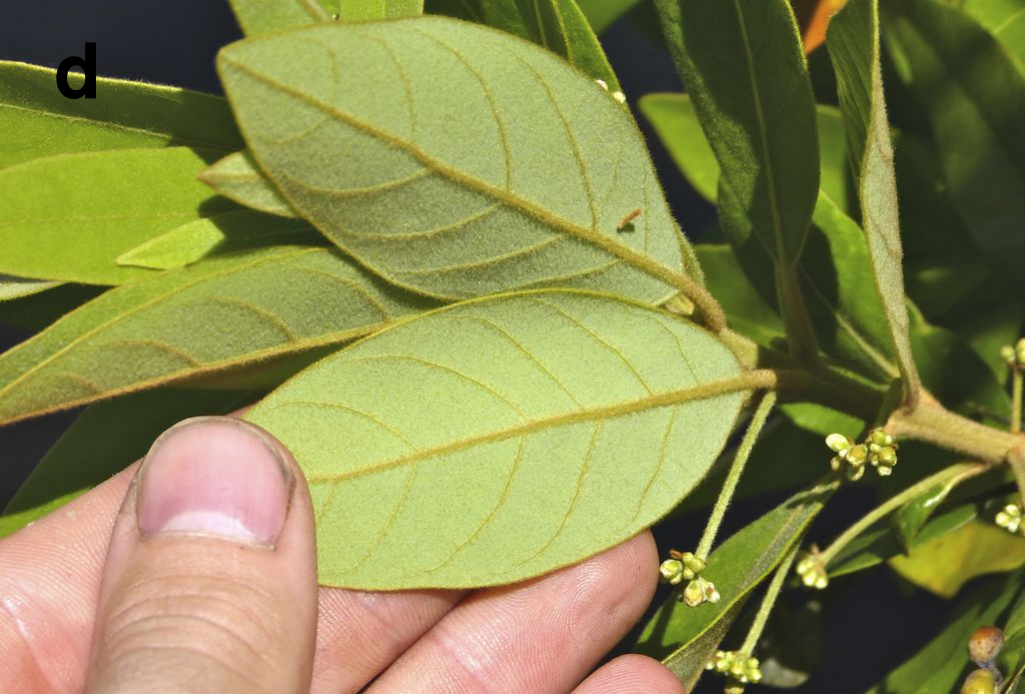
Leaf abaxial surface

**Figure 156e.**
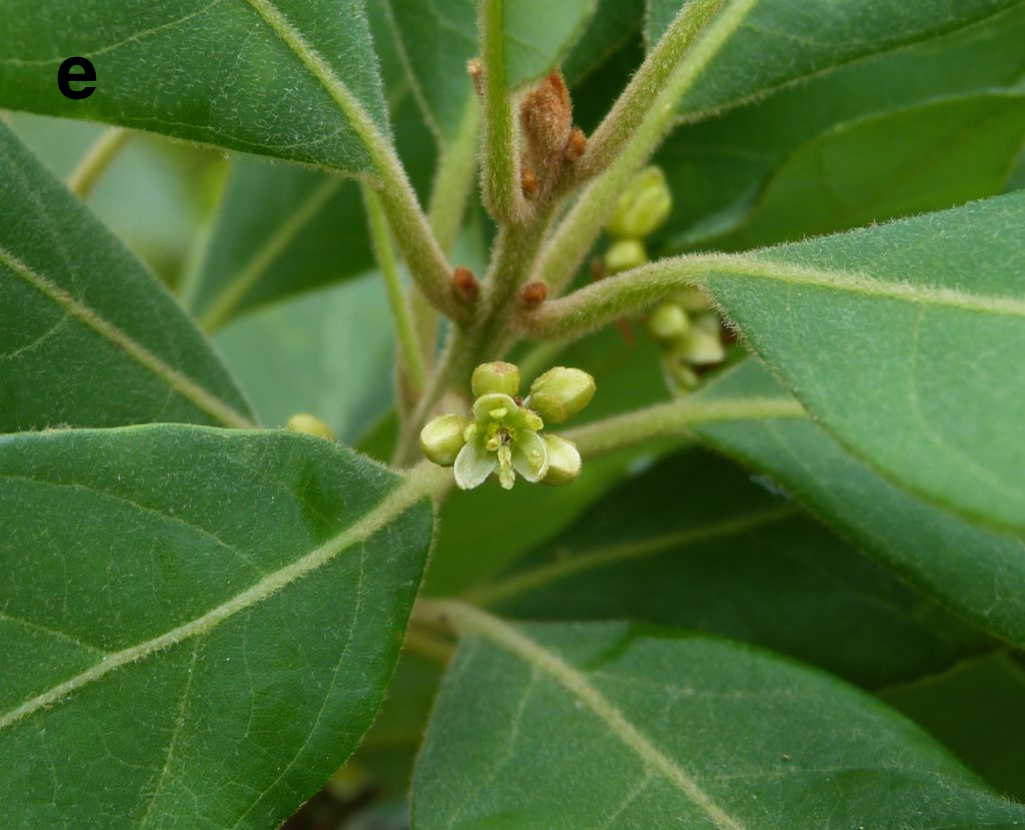
Flower

**Figure 156f.**
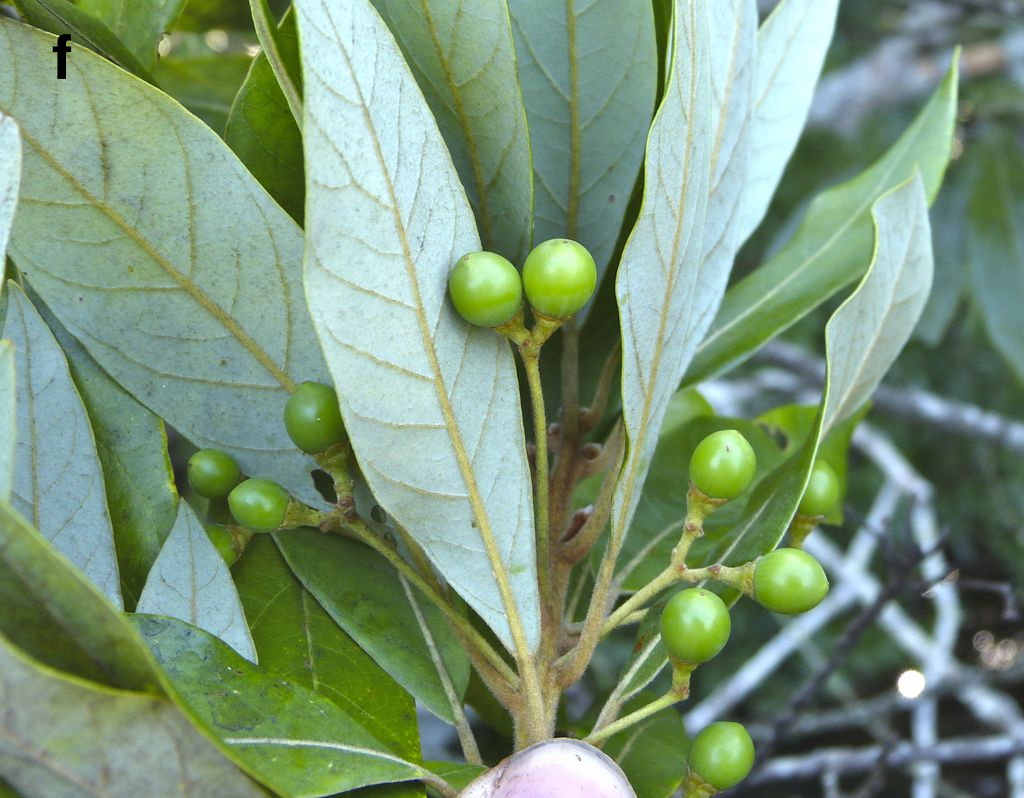
Fruits

**Figure 157a.**
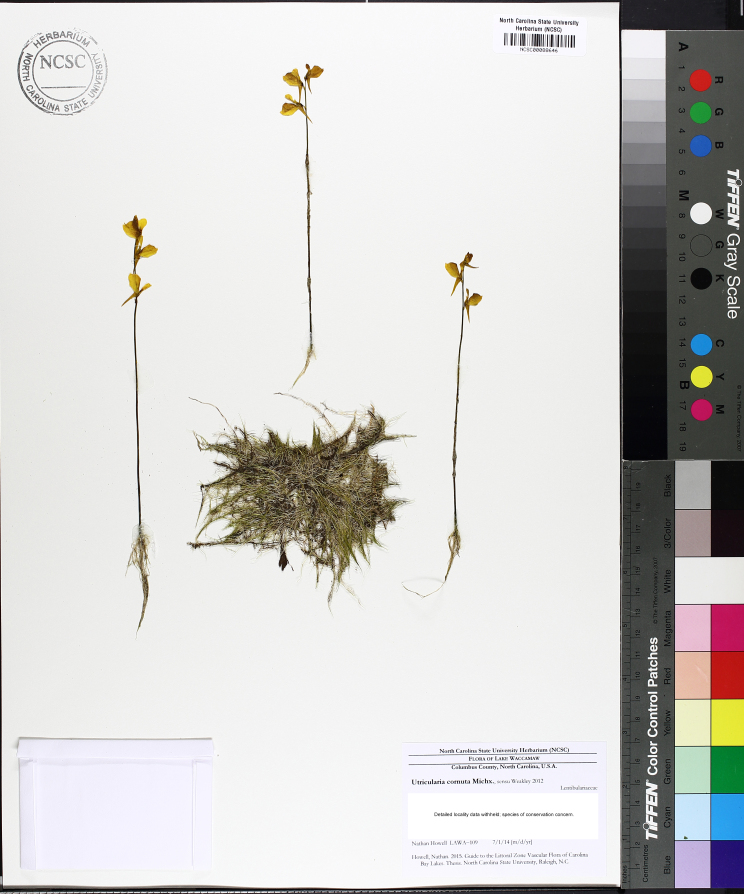
Specimen: *Howell LAWA-109* (NCSC)

**Figure 157b.**
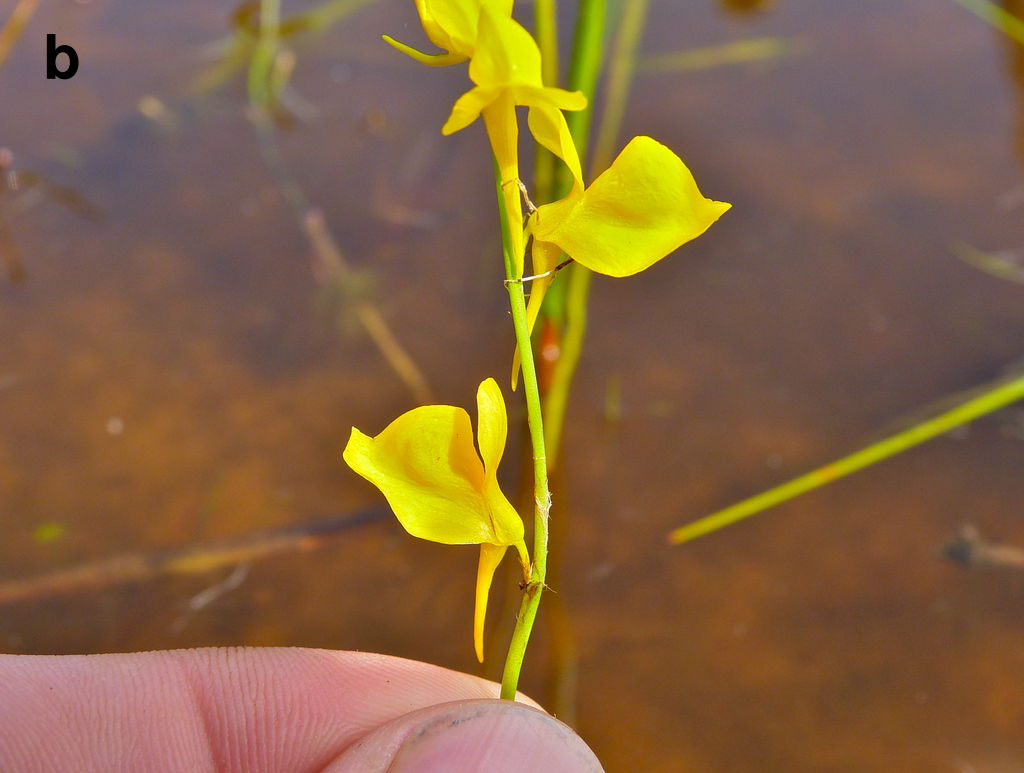
Habit

**Figure 158a.**
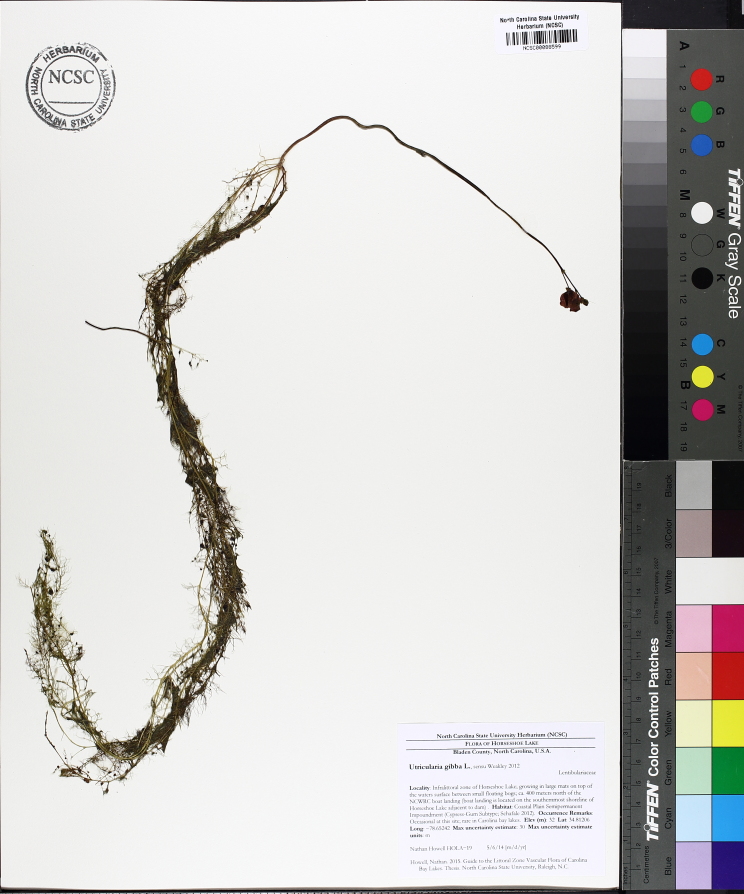
Specimen: *Howell HOLA-19* (NCSC)

**Figure 158b.**
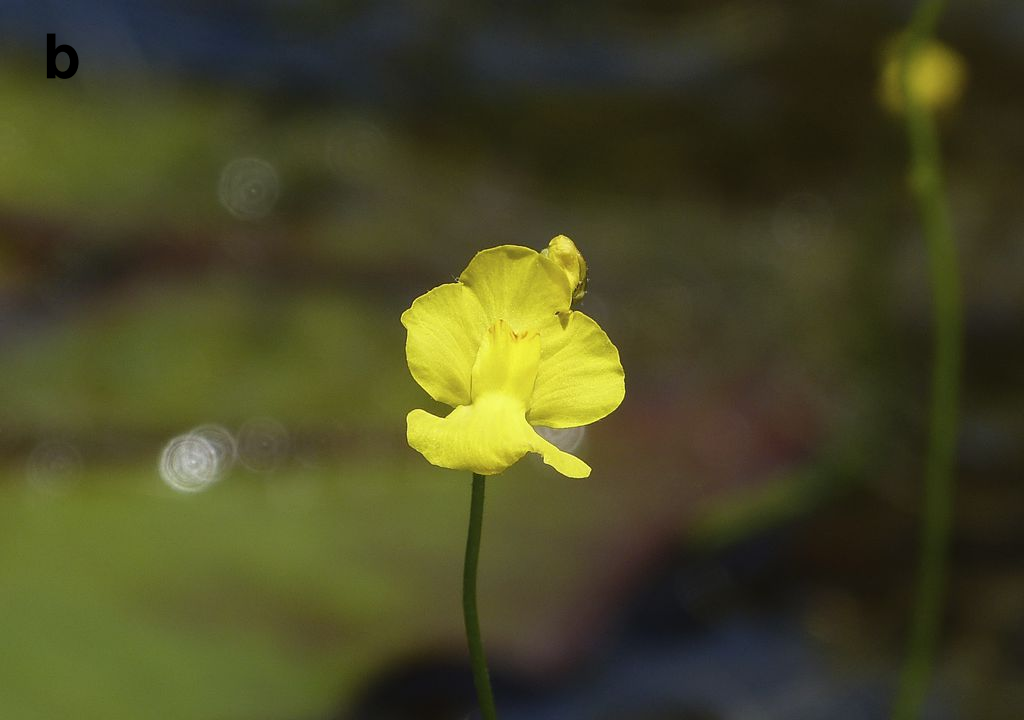
Flower

**Figure 159a.**
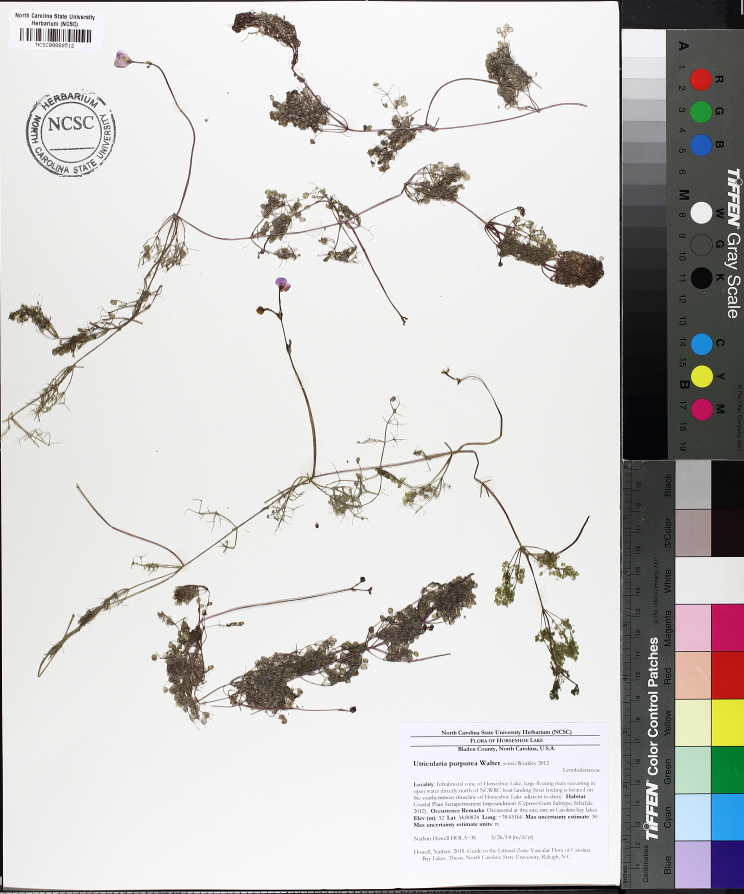
Specimen: *Howell HOLA-36* (NCSC)

**Figure 159b.**
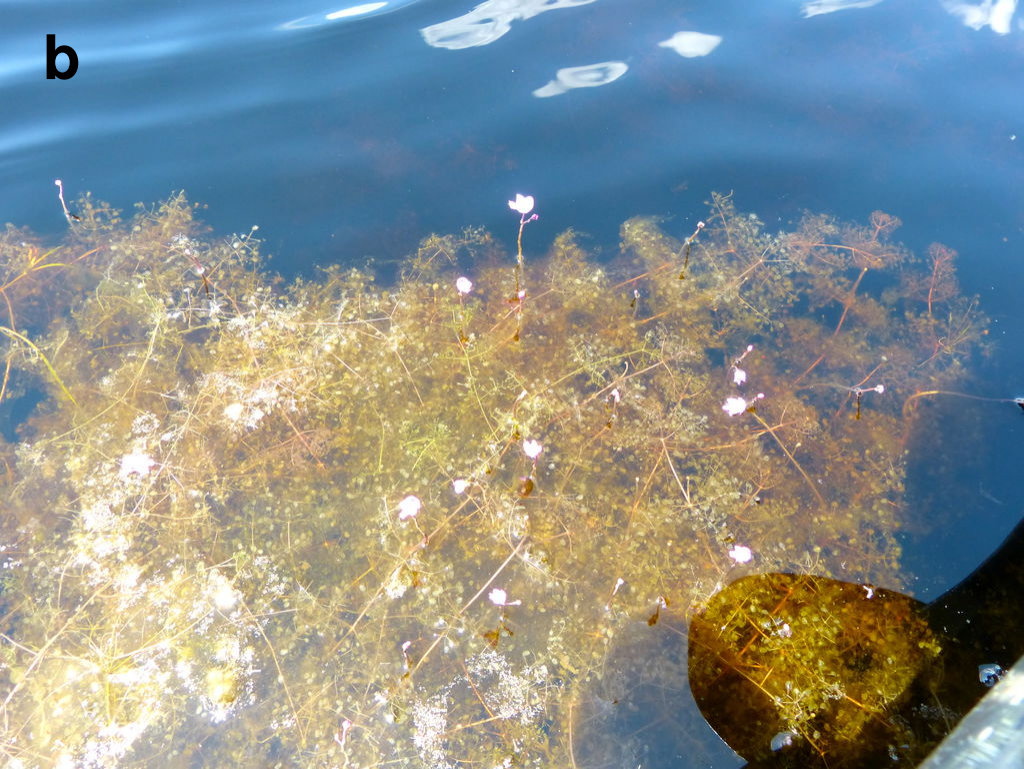
Habit

**Figure 159c.**
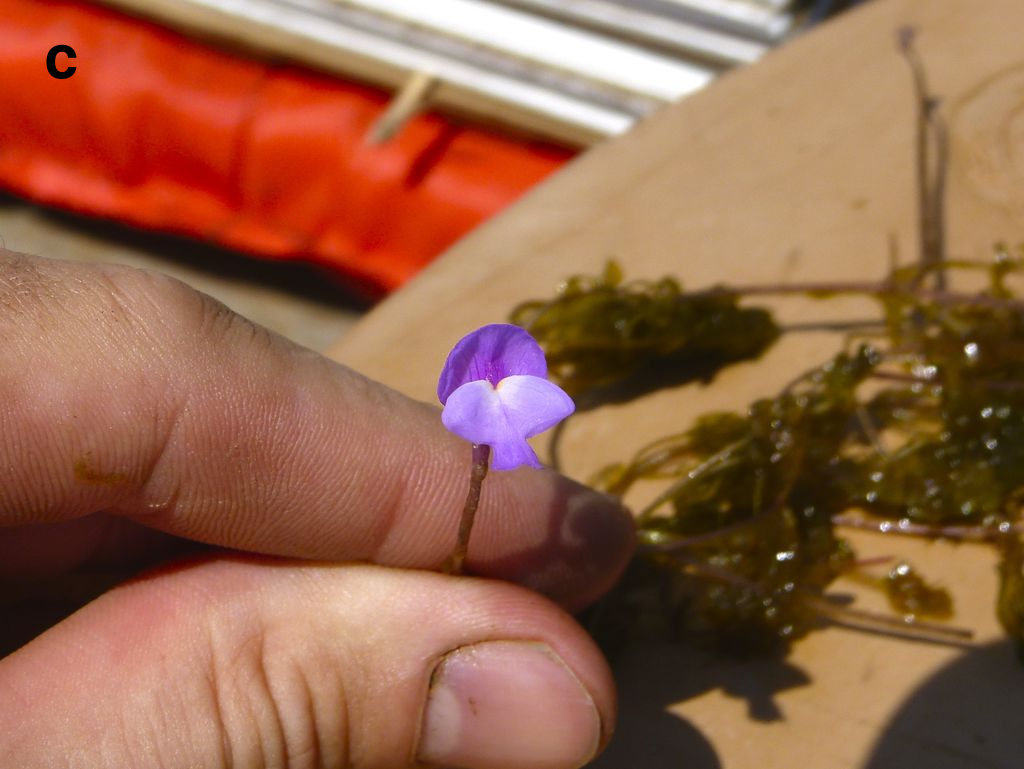
Flower

**Figure 159d.**
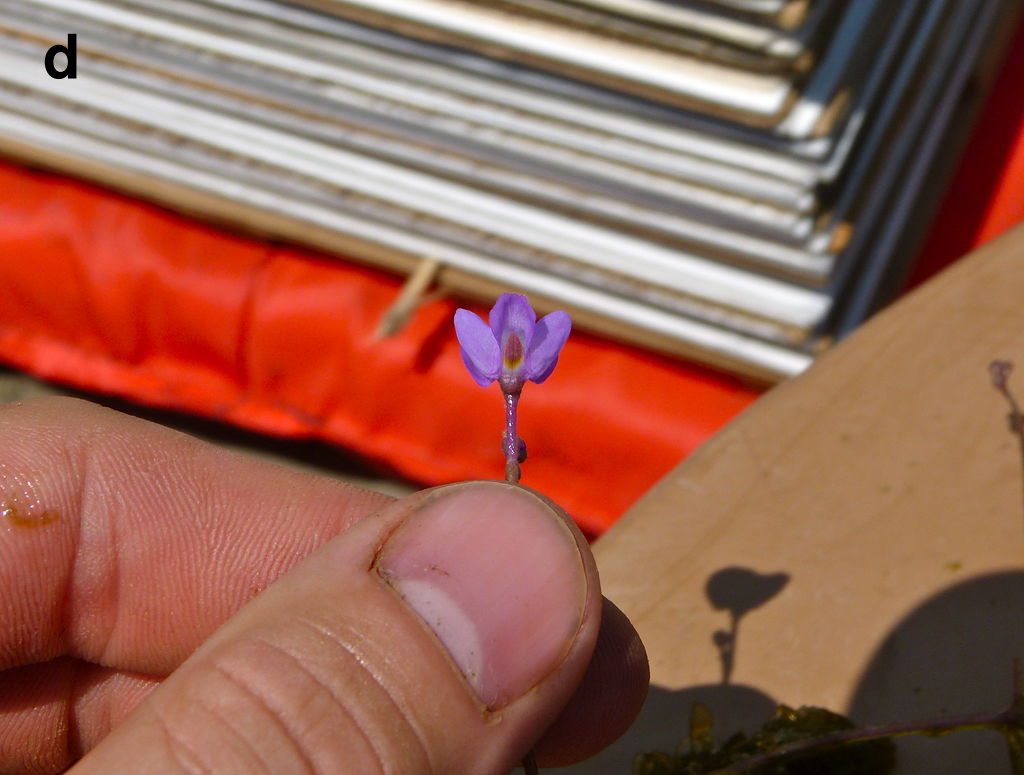
Flower

**Figure 160a.**
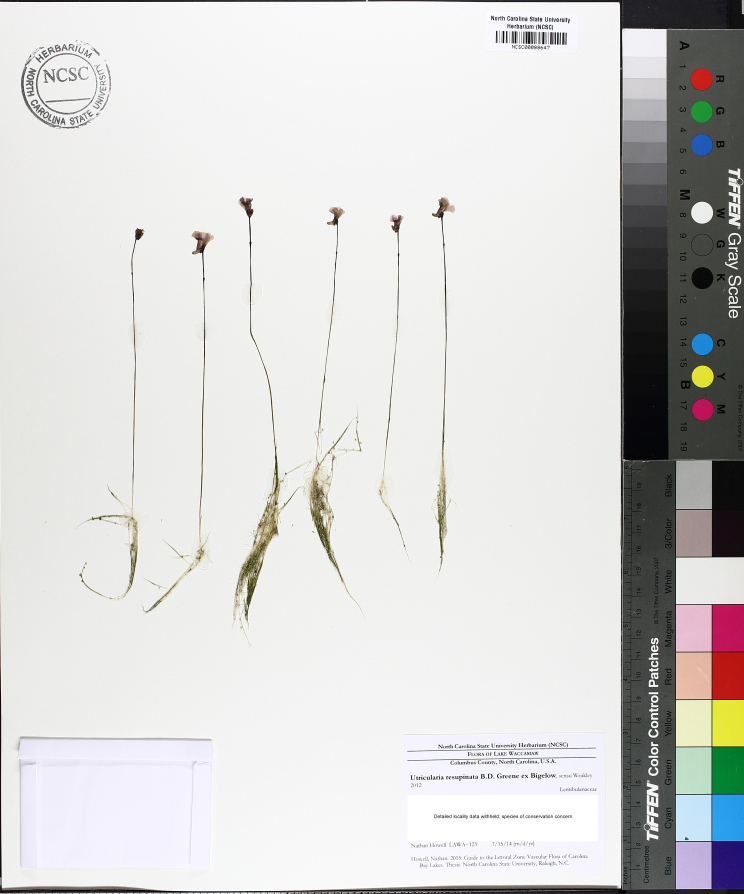
Specimen: *Howell LAWA-123* (NCSC)

**Figure 160b.**
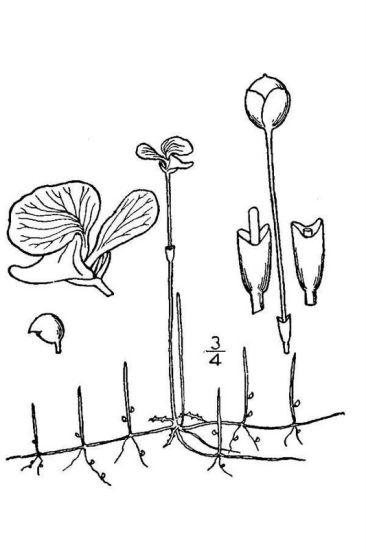
Illustration

**Figure 160c.**
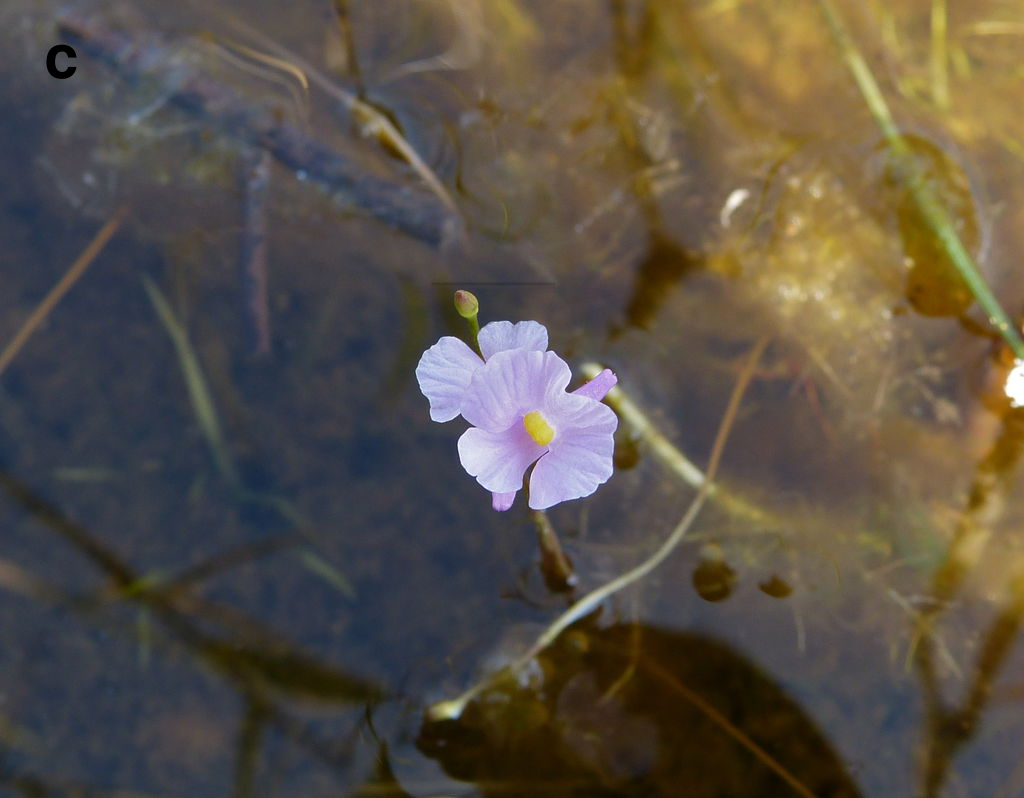
Flower (front)

**Figure 160d.**
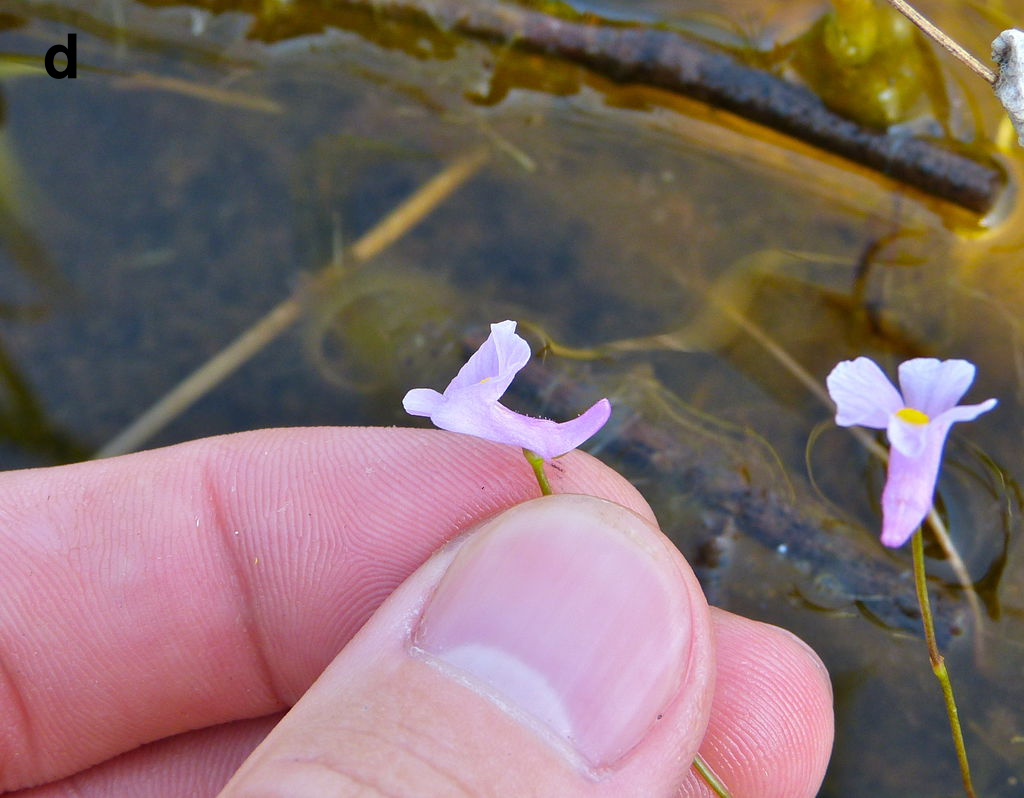
Flower (side)

**Figure 161a.**
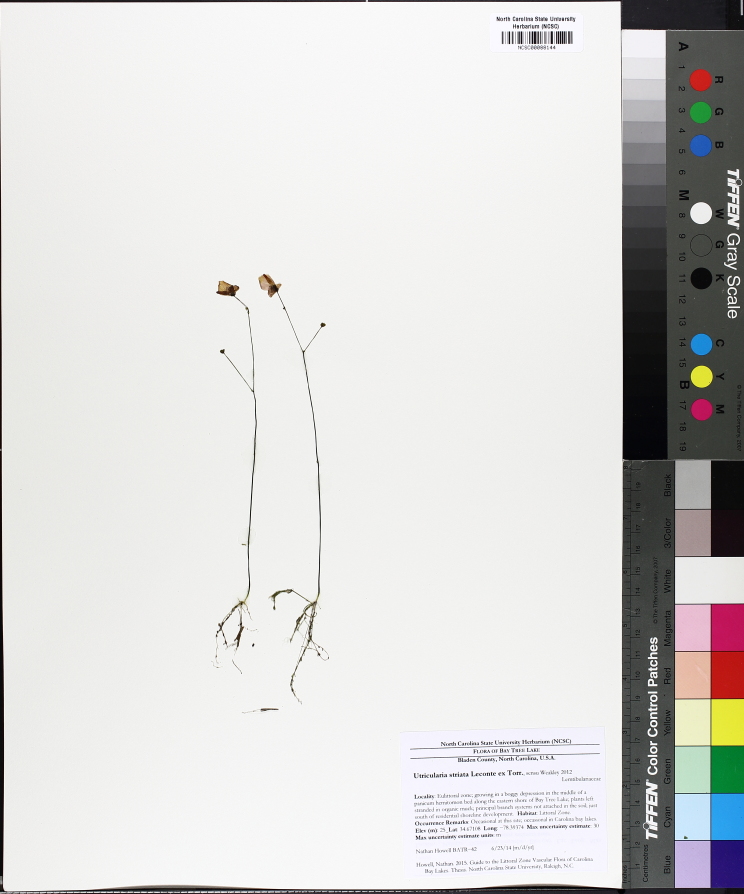
Specimen: *Howell BATR-42* (NCSC)

**Figure 161b.**
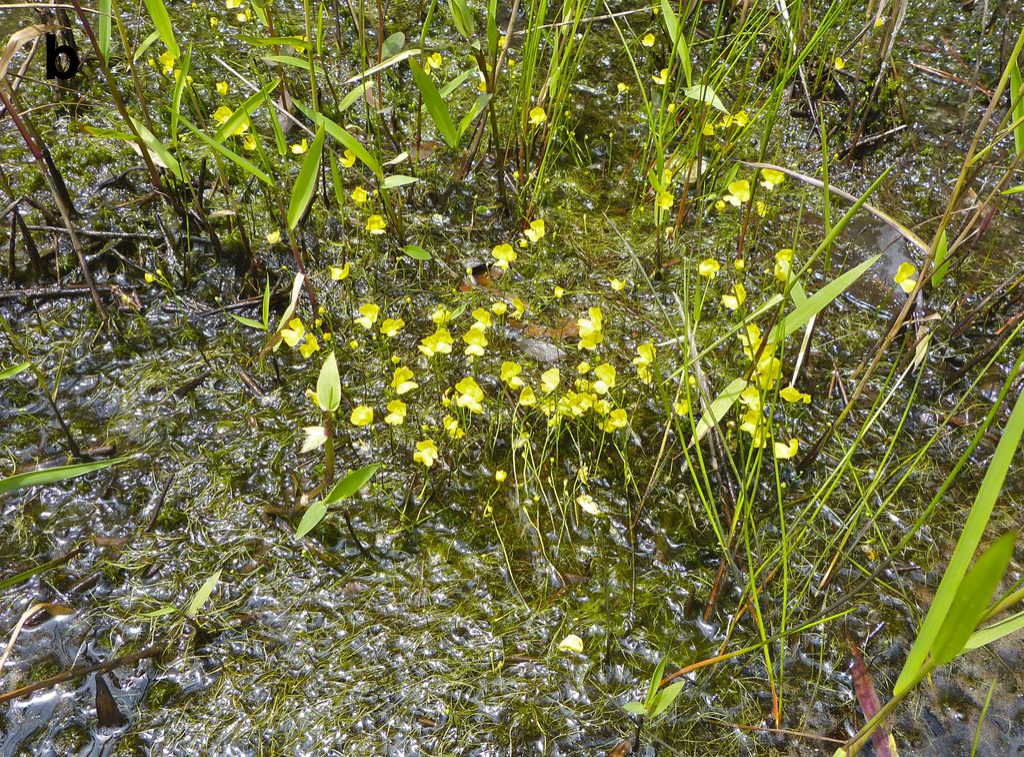
Habit

**Figure 161c.**
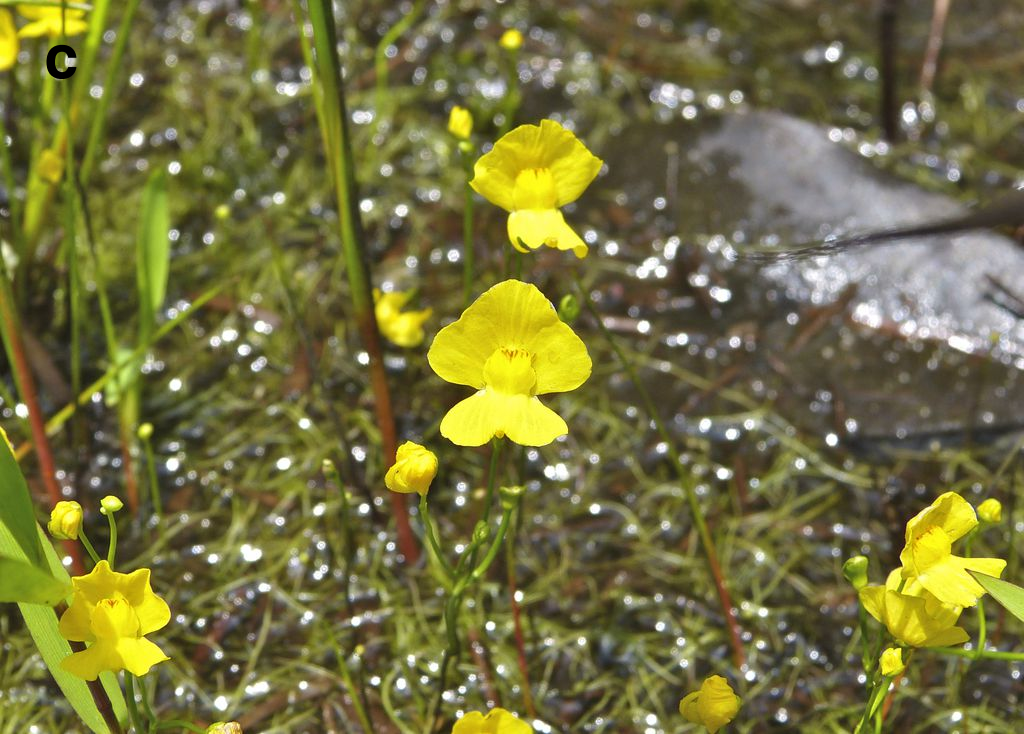
Flower

**Figure 161d.**
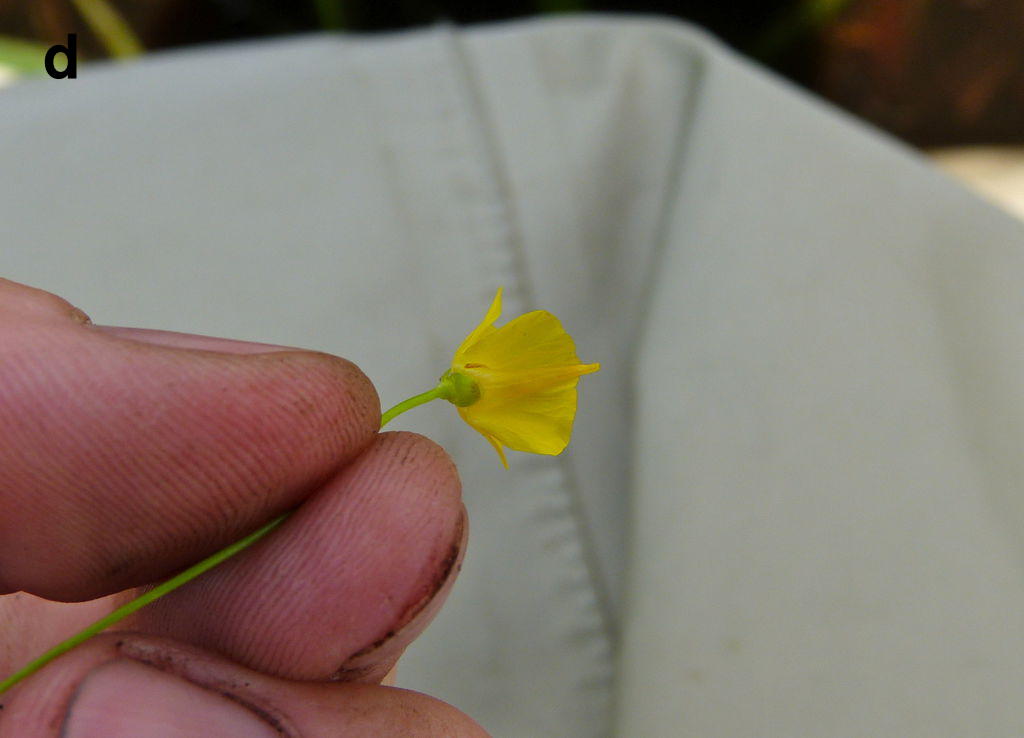
Flower

**Figure 162a.**
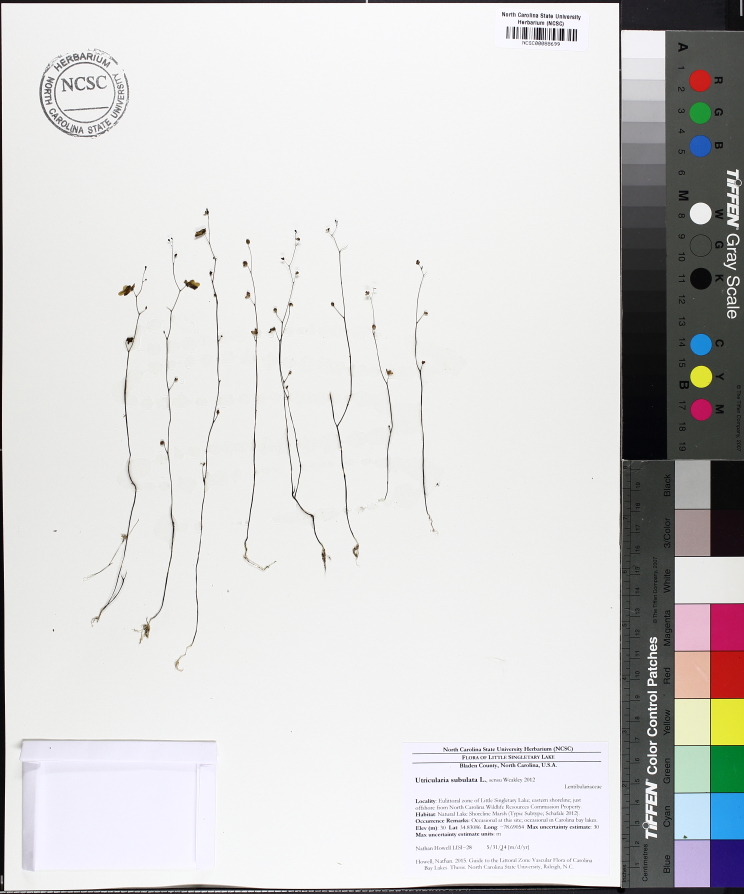
Specimen: *Howell LISI-28* (NCSC)

**Figure 162b.**
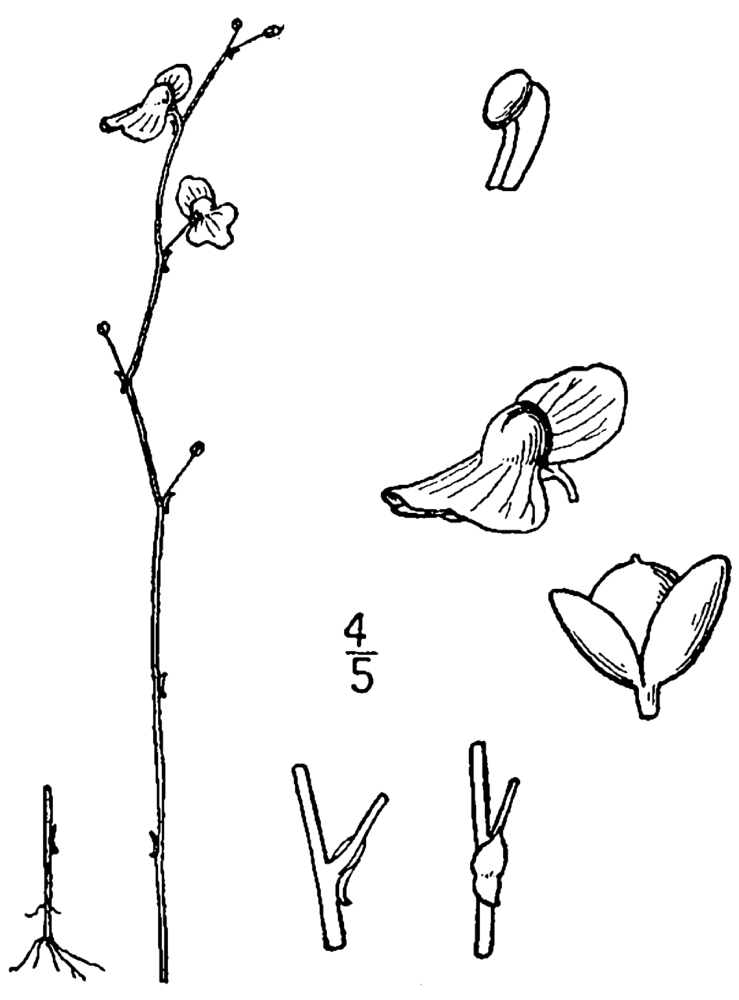
Illustration

**Figure 162c.**
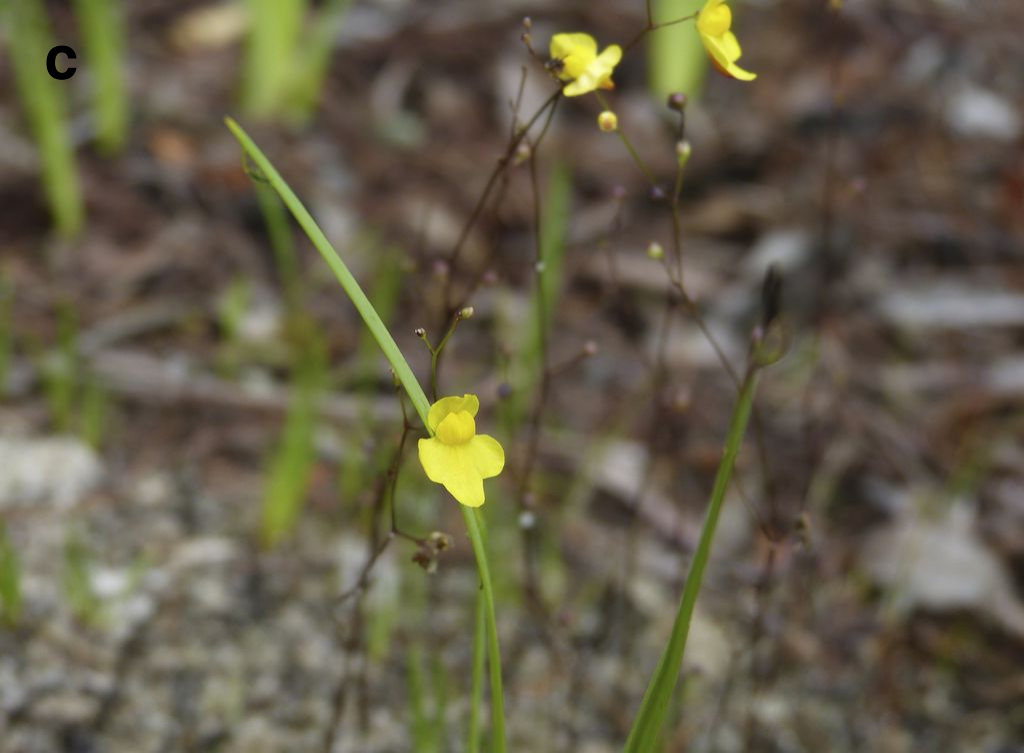
Habit

**Figure 162d.**
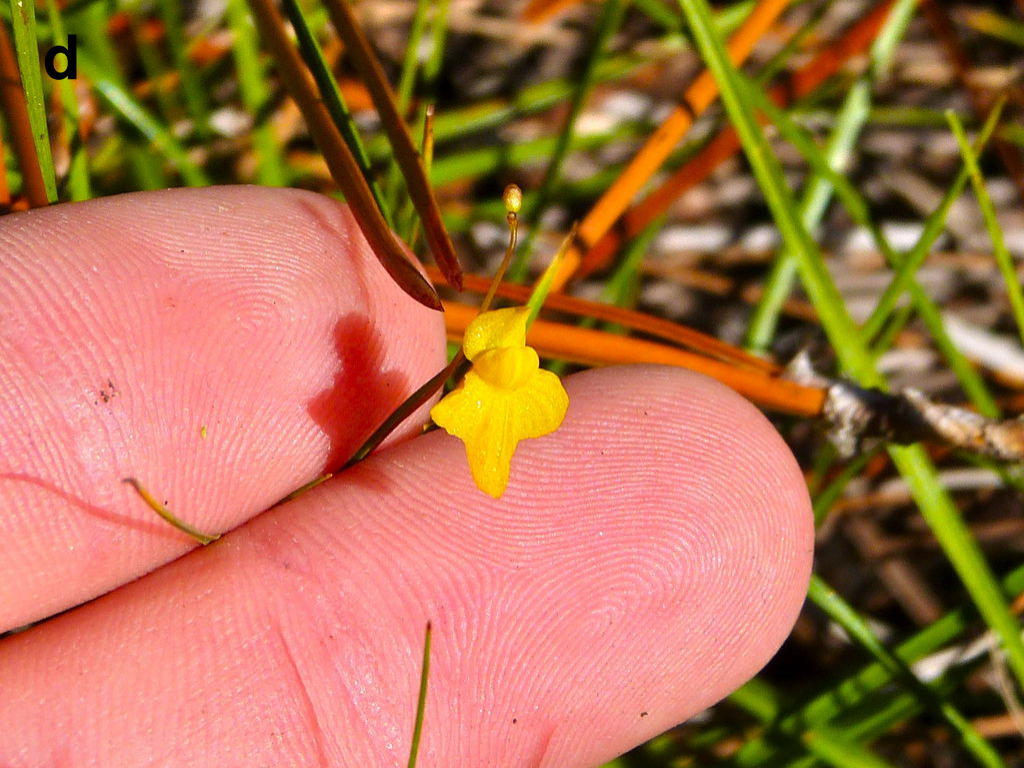
Flower (front)

**Figure 162e.**
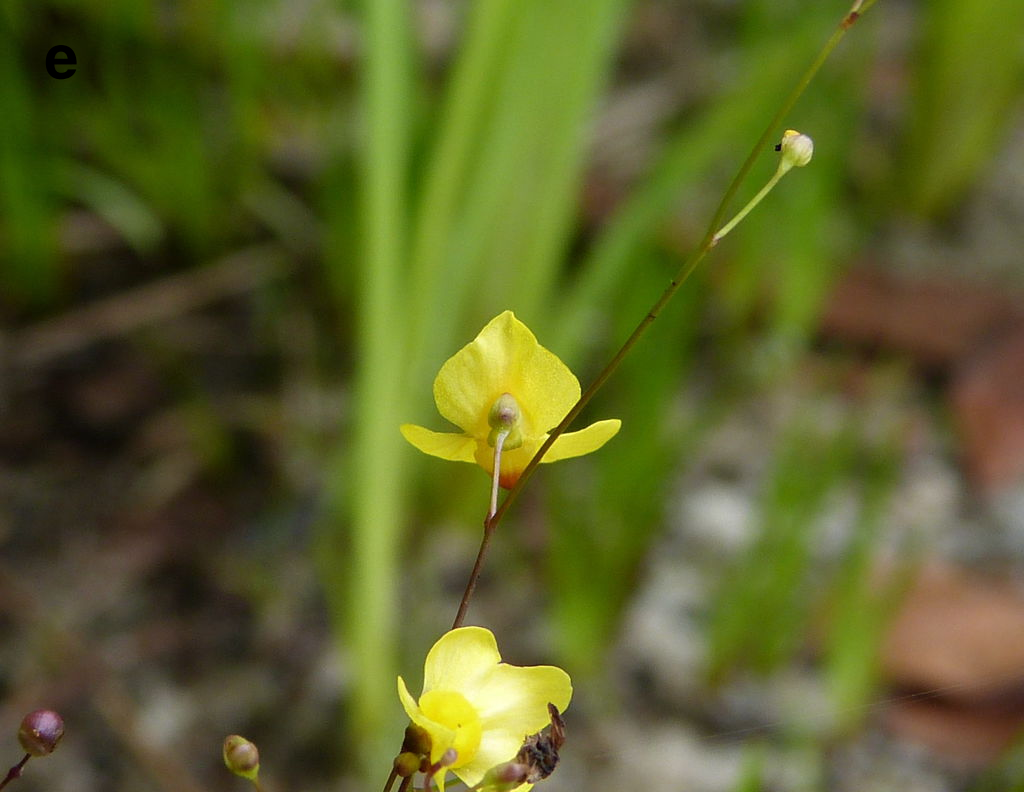
Flower (back)

**Figure 162f.**
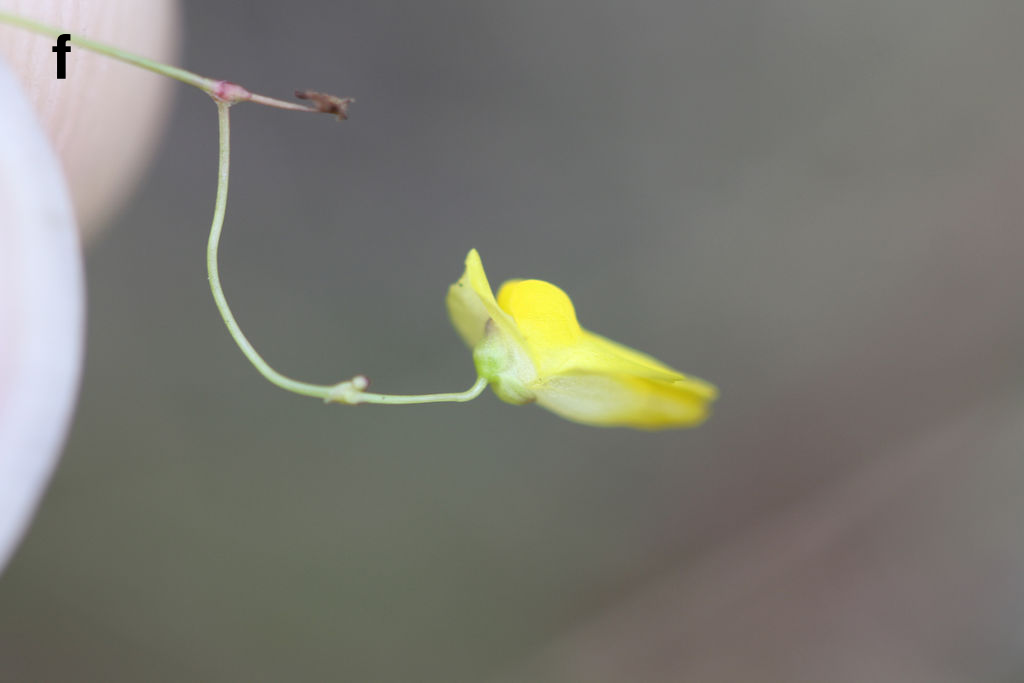
Flower (side)

**Figure 164a.**
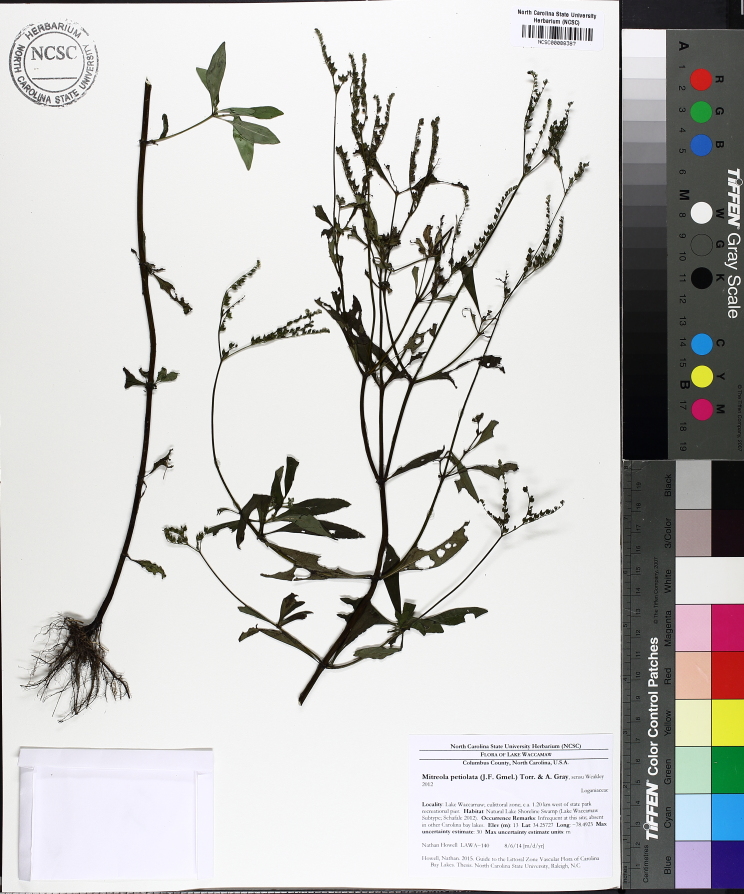
Specimen: *Howell LAWA-140* (NCSC)

**Figure 164b.**
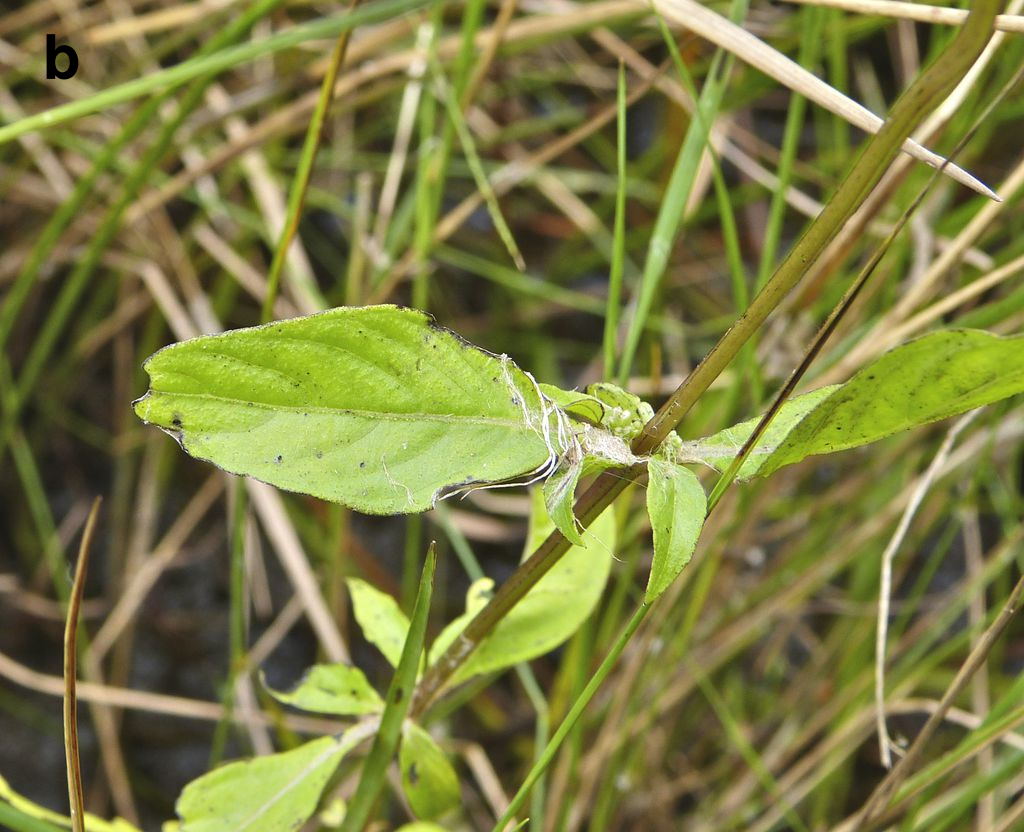
Stem and leaves

**Figure 164c.**
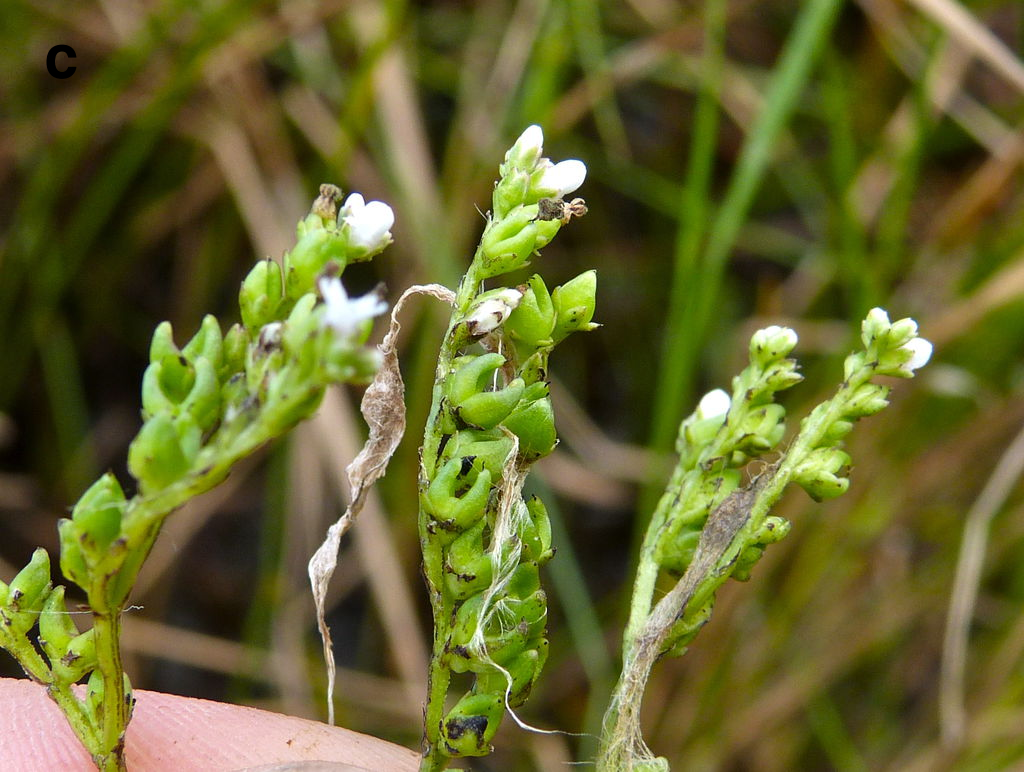
Inflorescence

**Figure 164d.**
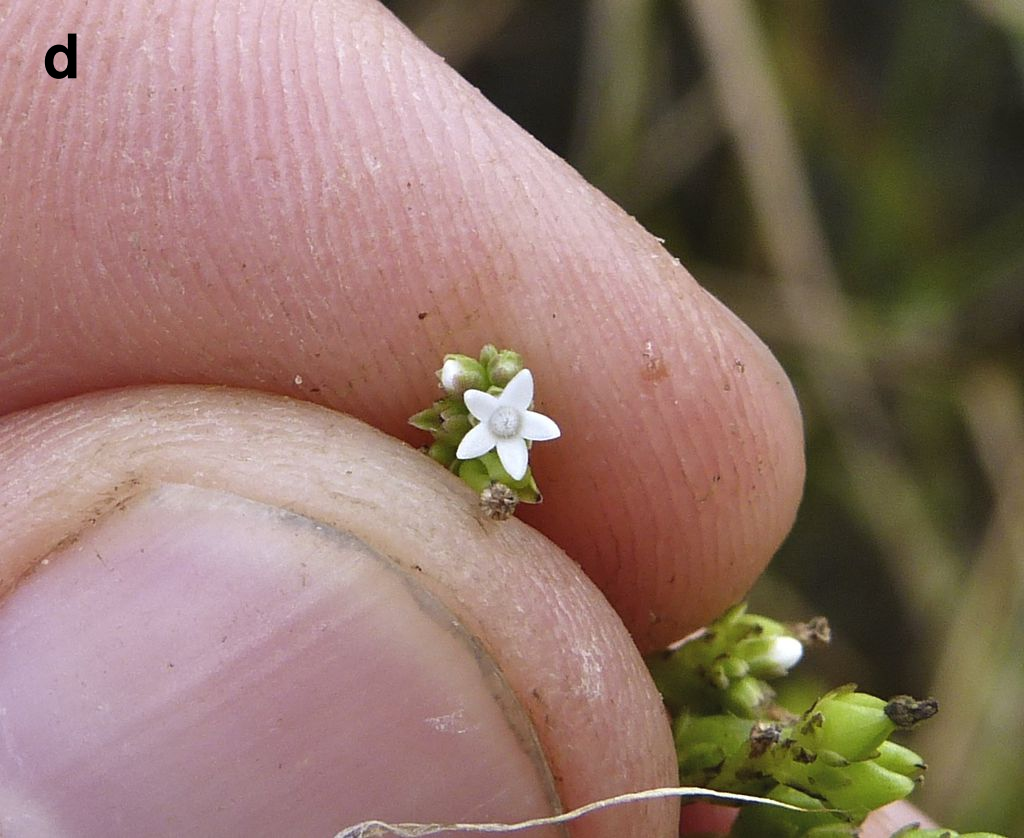
Flower

**Figure 165a.**
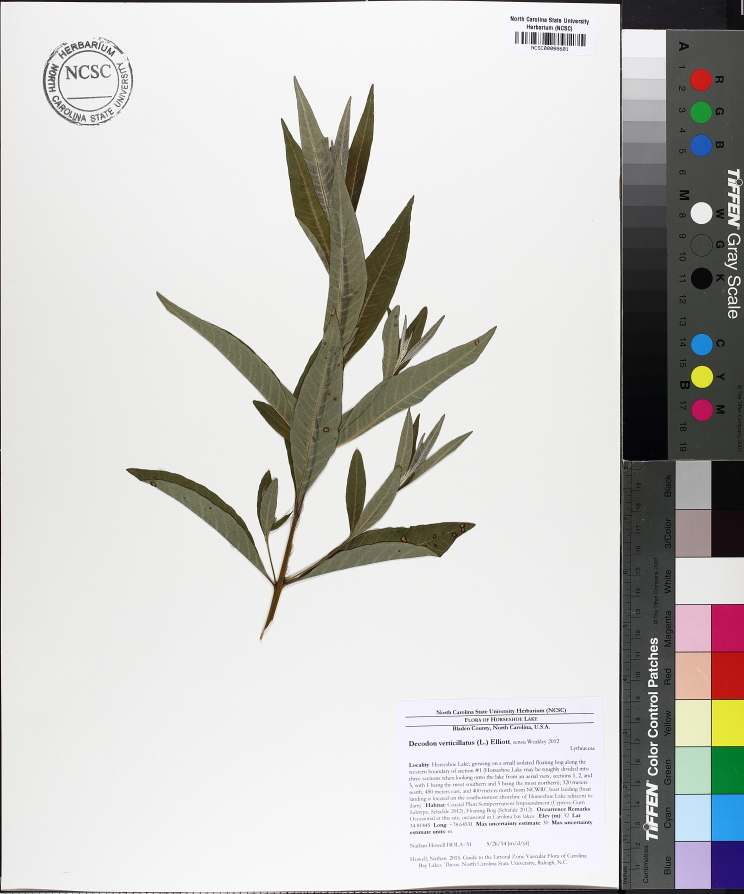
Specimen: *Howell HOLA-31* (NCSC)

**Figure 165b.**
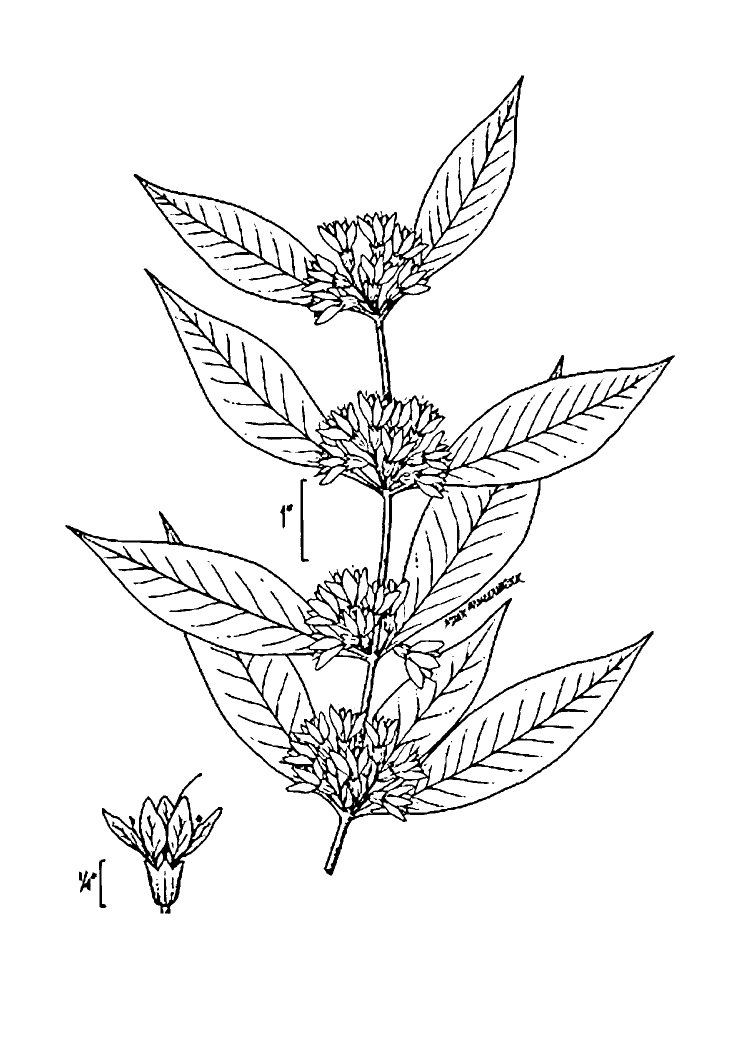
Illustration

**Figure 165c.**
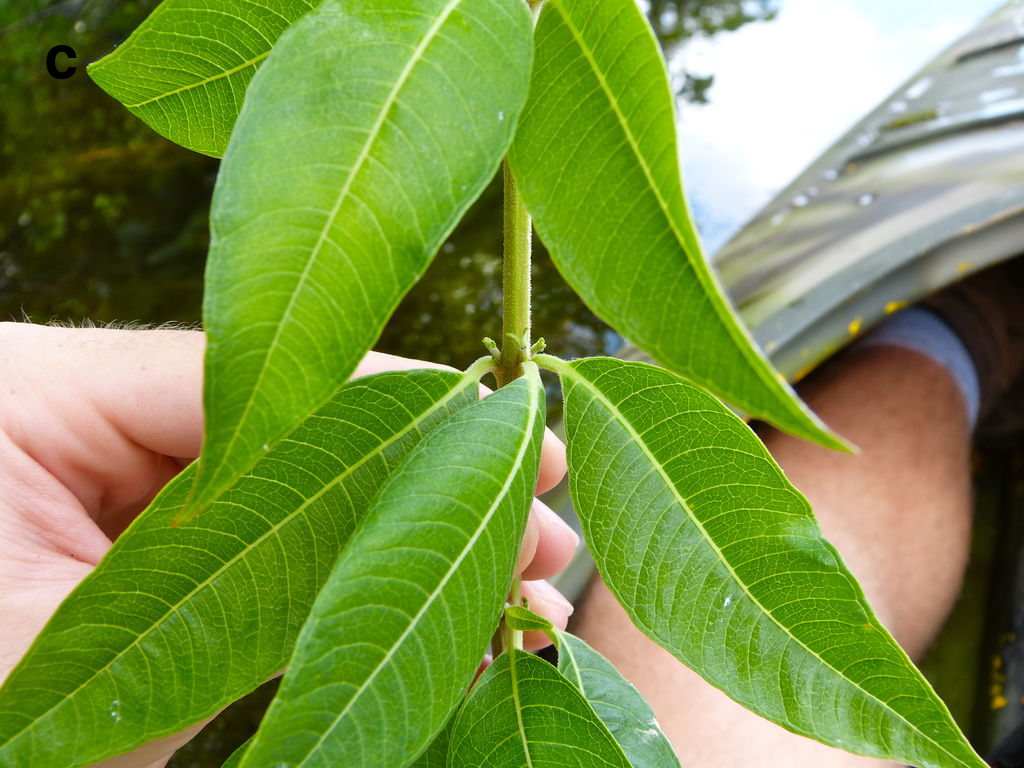
Leaves

**Figure 165d.**
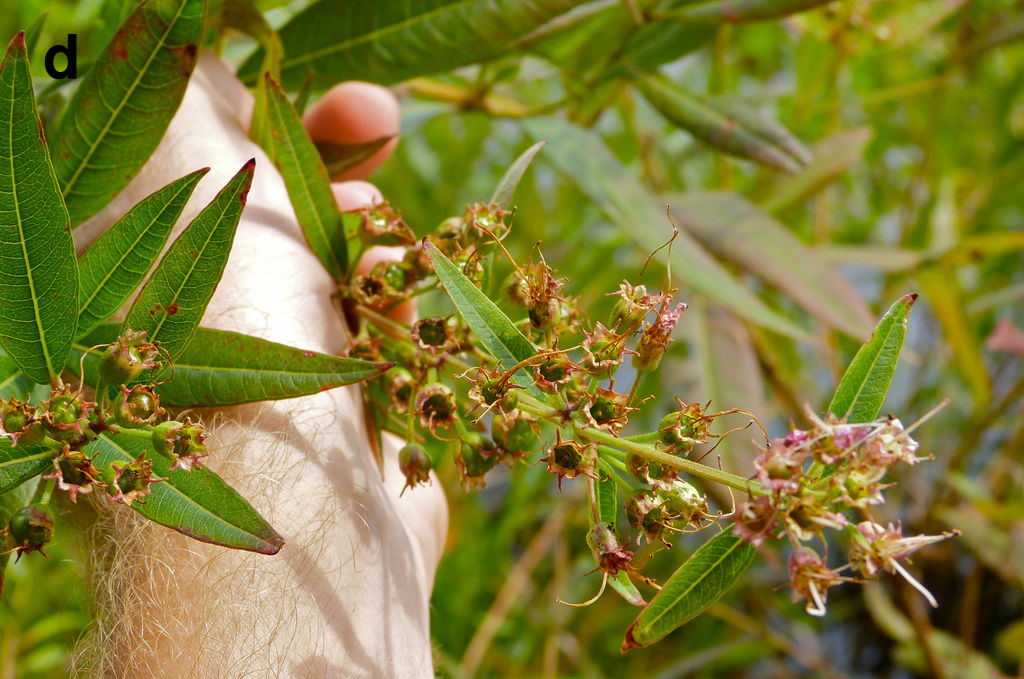
Fruits

**Figure 166a.**
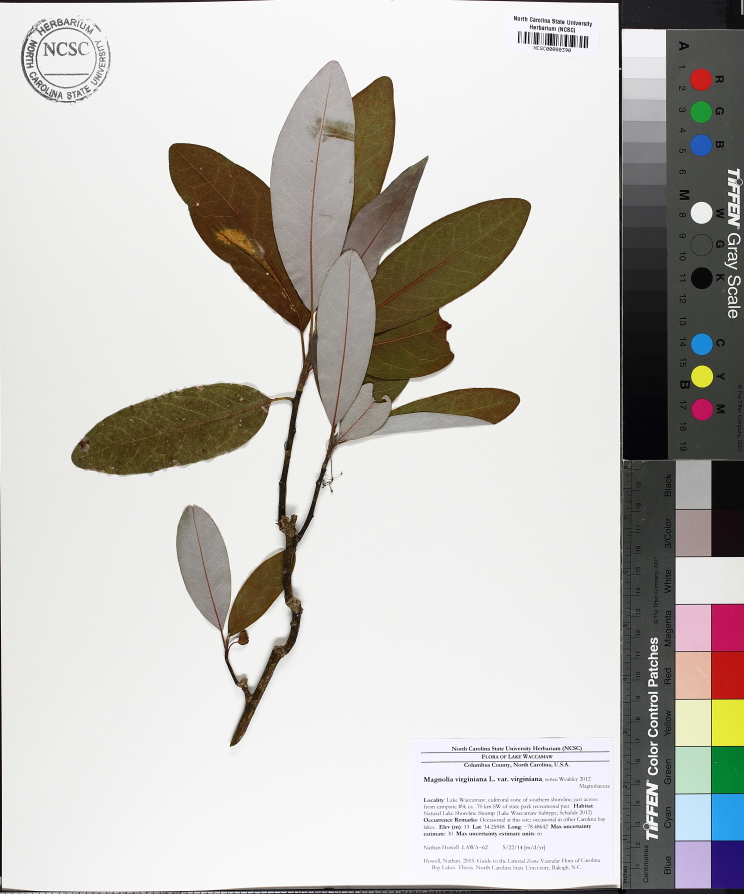
Specimen: *Howell LAWA-62* (NCSC)

**Figure 166b.**
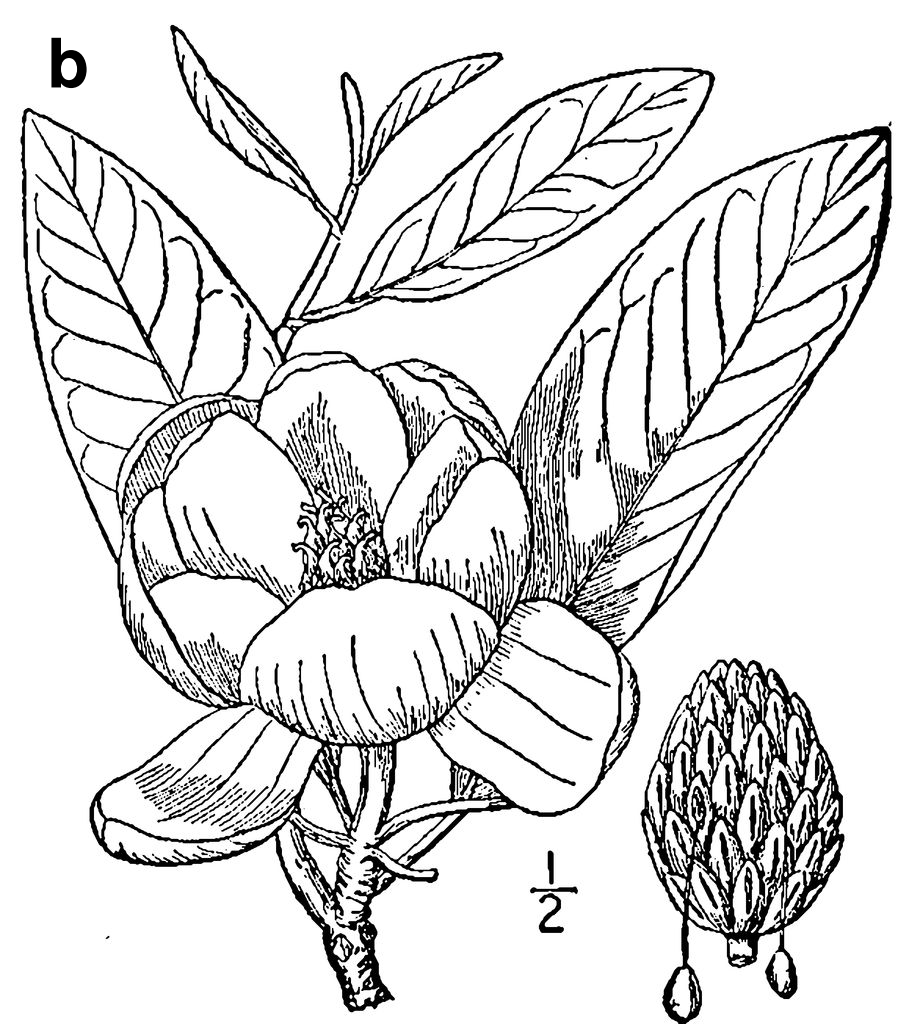
Illustration

**Figure 166c.**
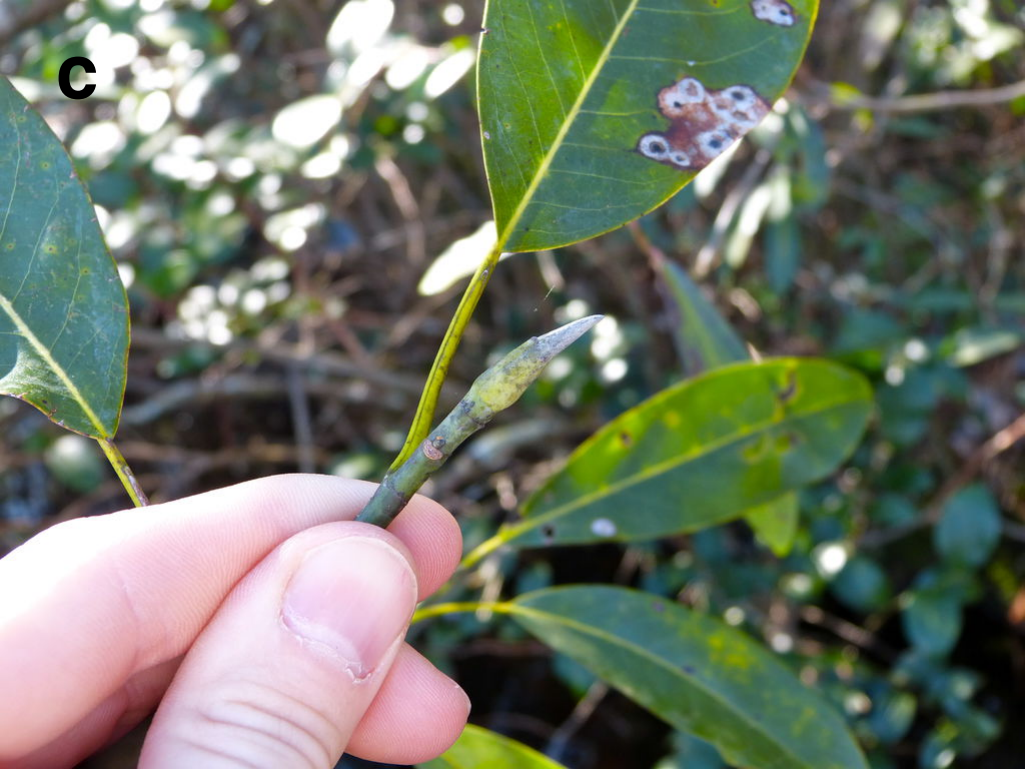
Stem and terminal bud

**Figure 166d.**
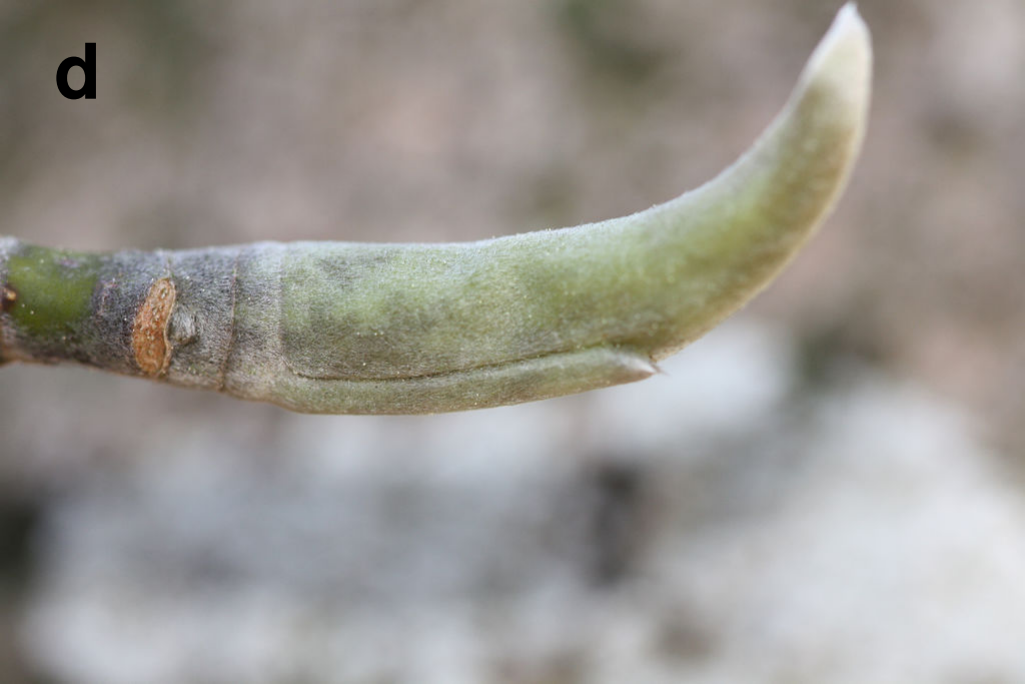
Terminal bud (detail)

**Figure 166e.**
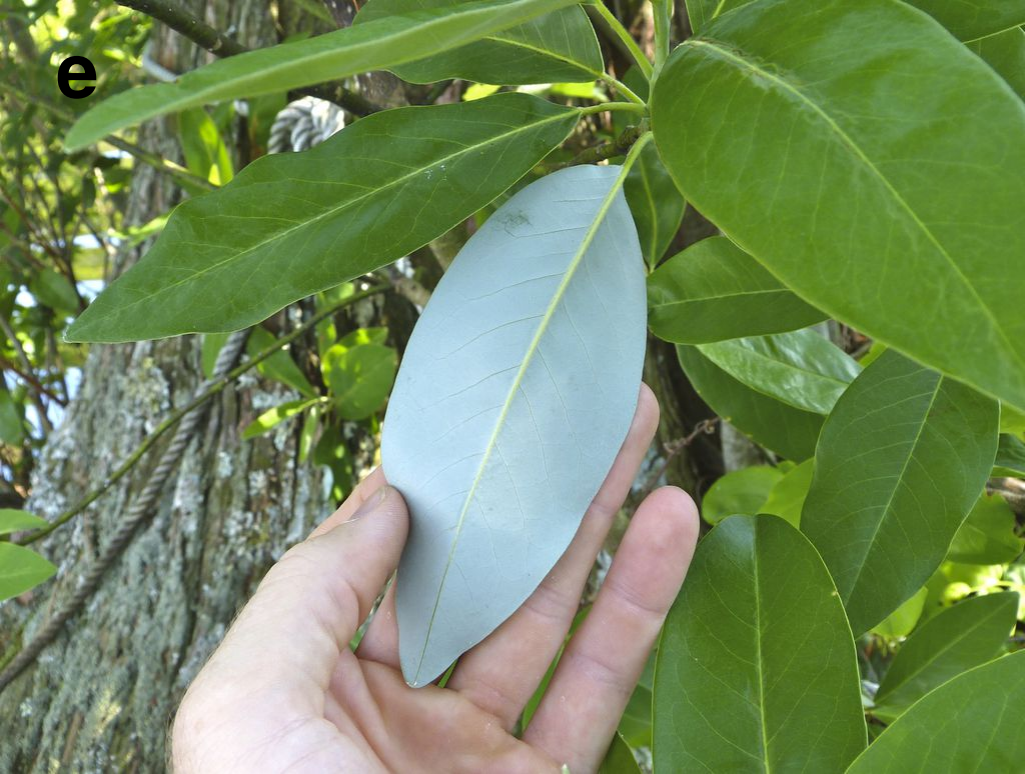
Leaves (note glaucescence below)

**Figure 166f.**
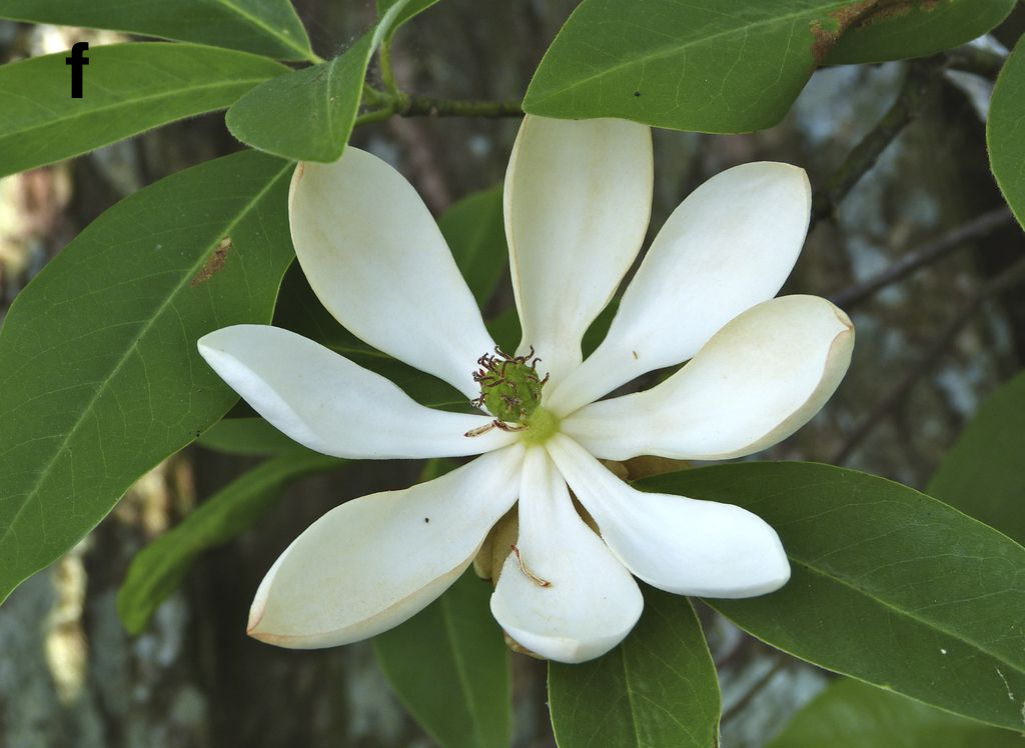
Flower

**Figure 167a.**
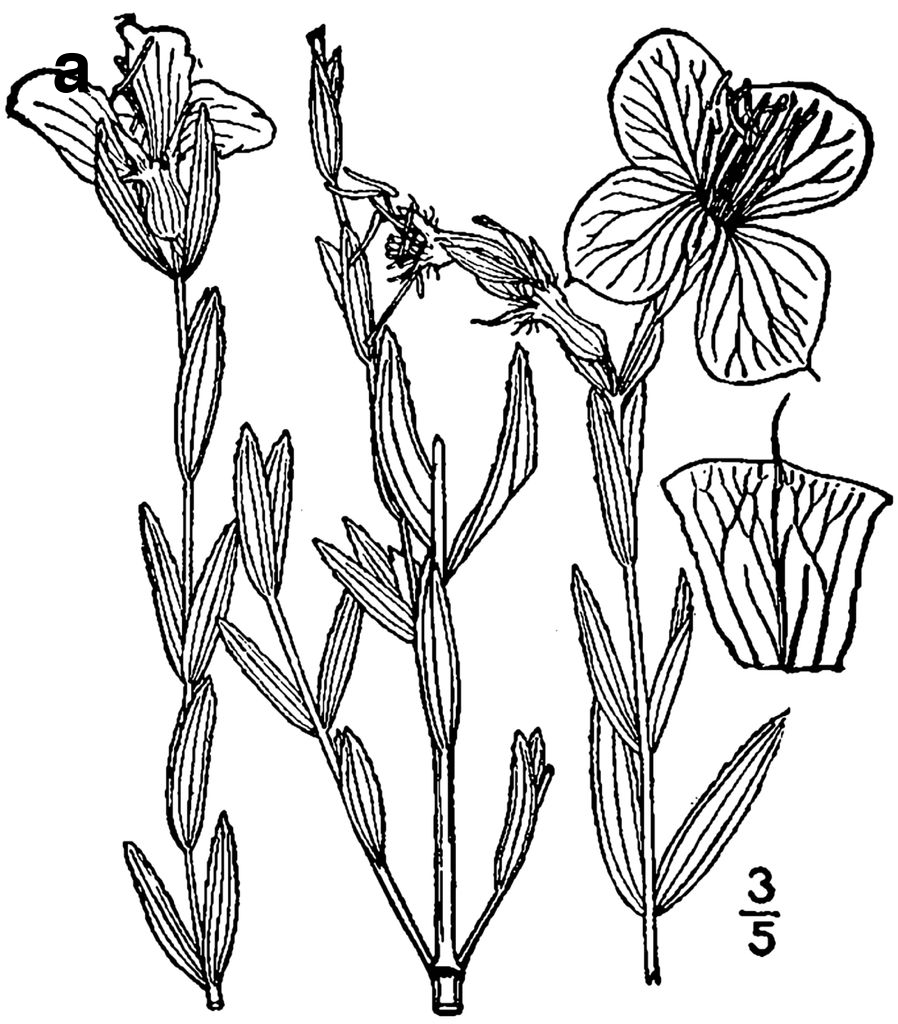
Illustration

**Figure 167b.**
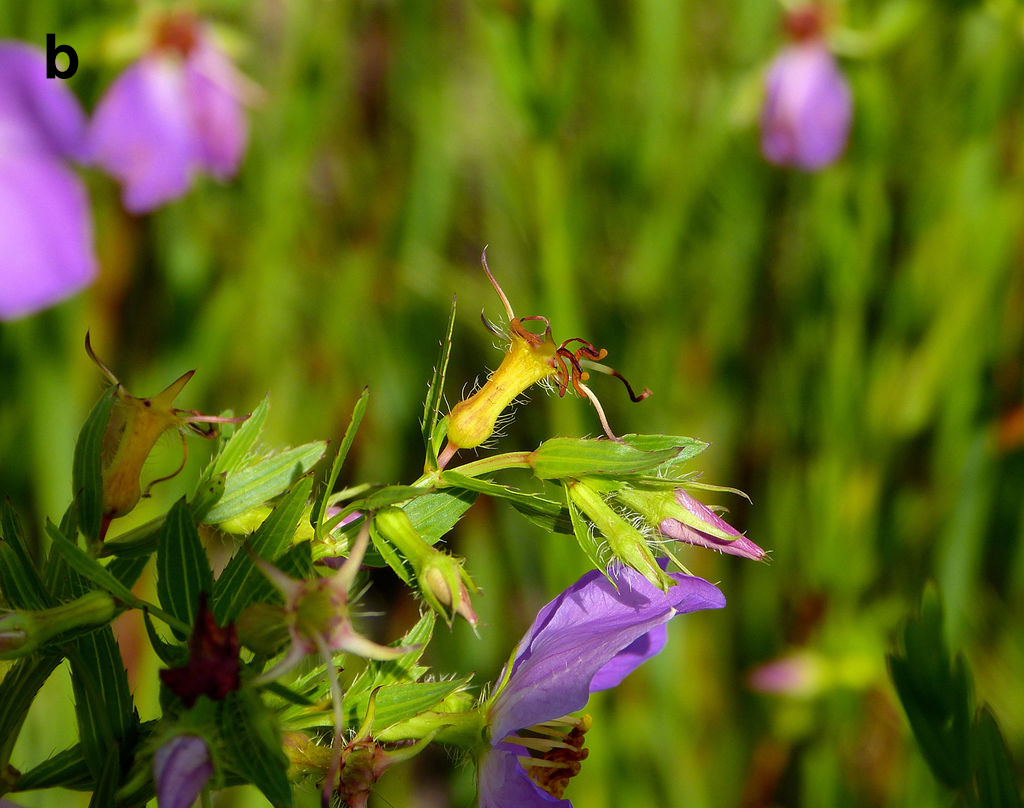
Calyx tube (lateral)

**Figure 167c.**
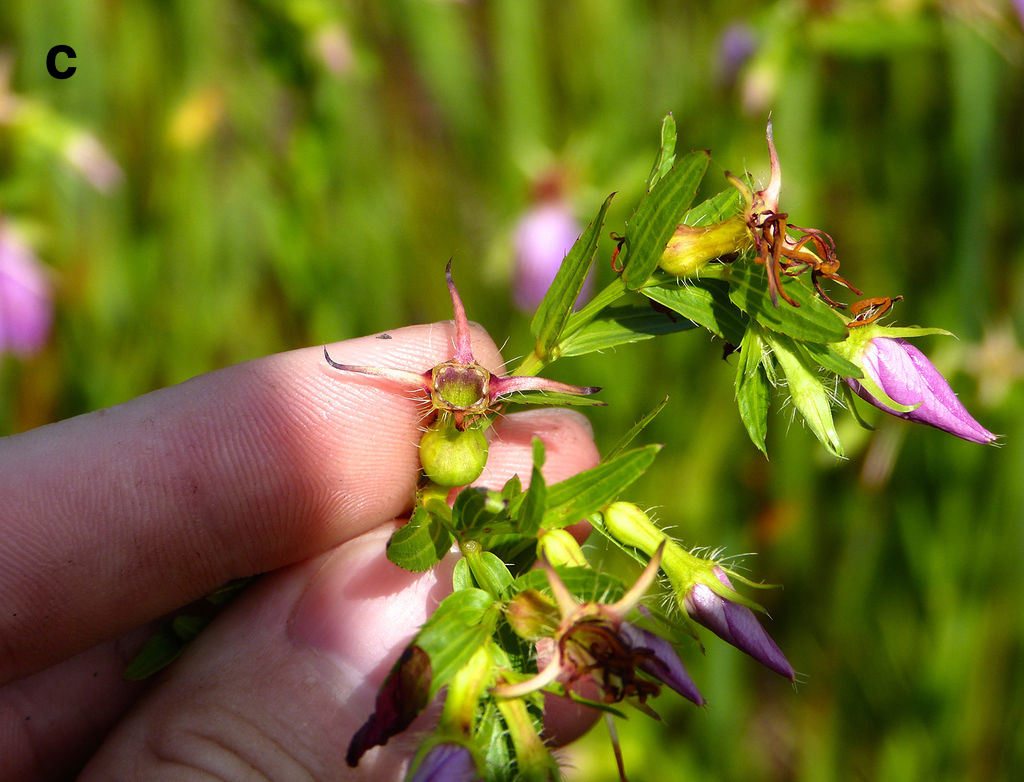
Calyx tube (latero-adaxial)

**Figure 167d.**
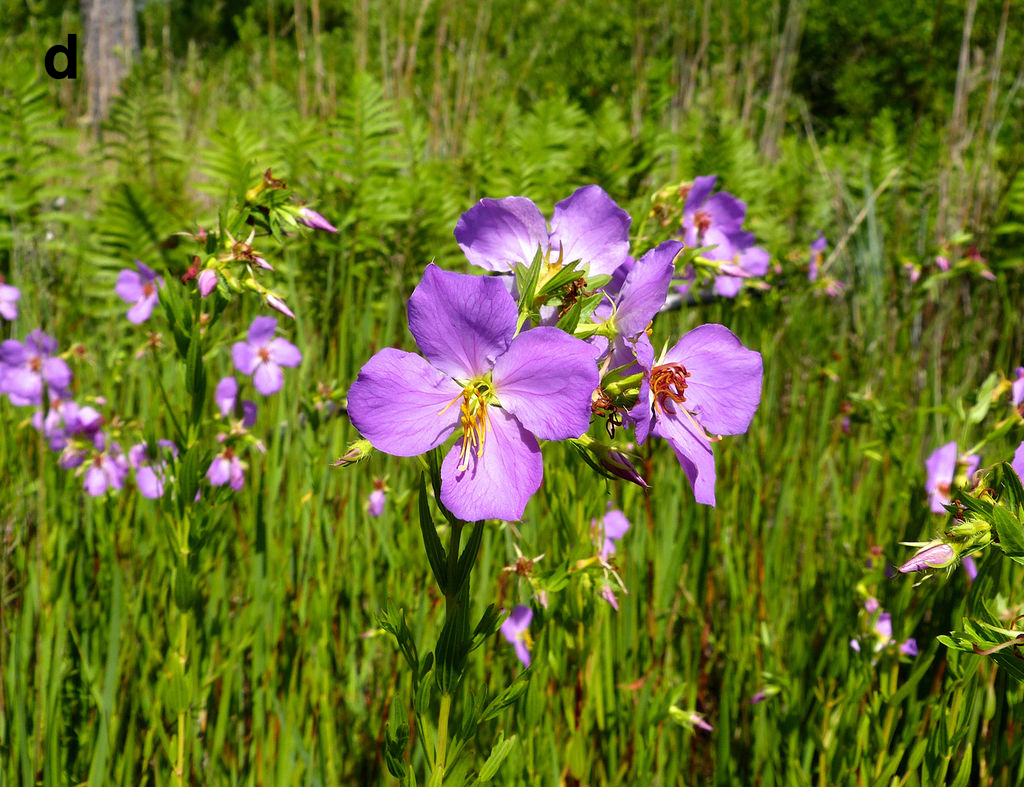
Flower (adaxial)

**Figure 168a.**
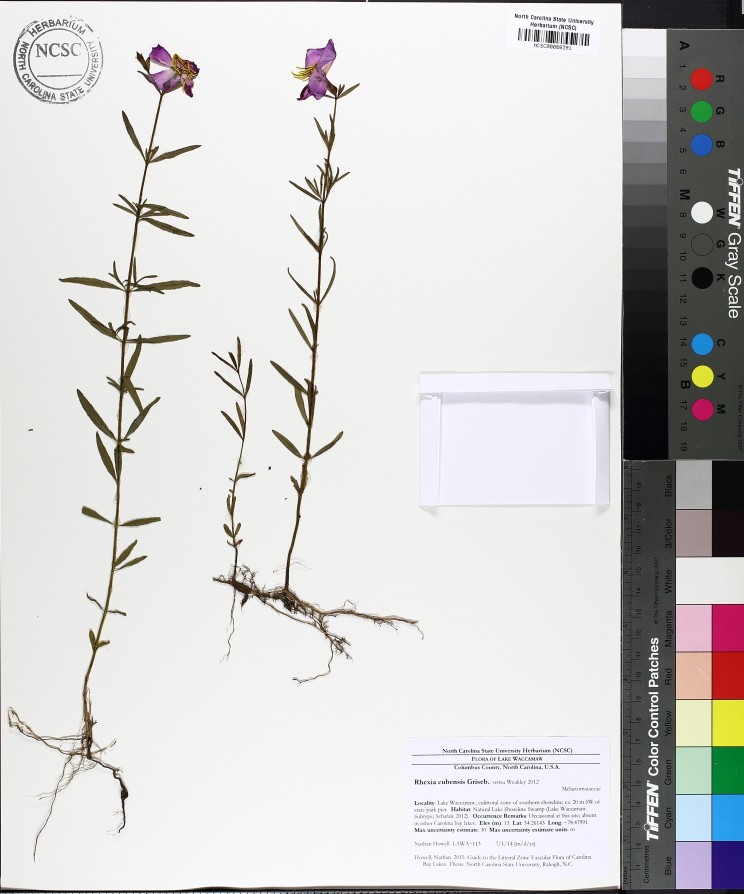
Specimen: *Howell LAWA-113* (NCSC)

**Figure 168b.**
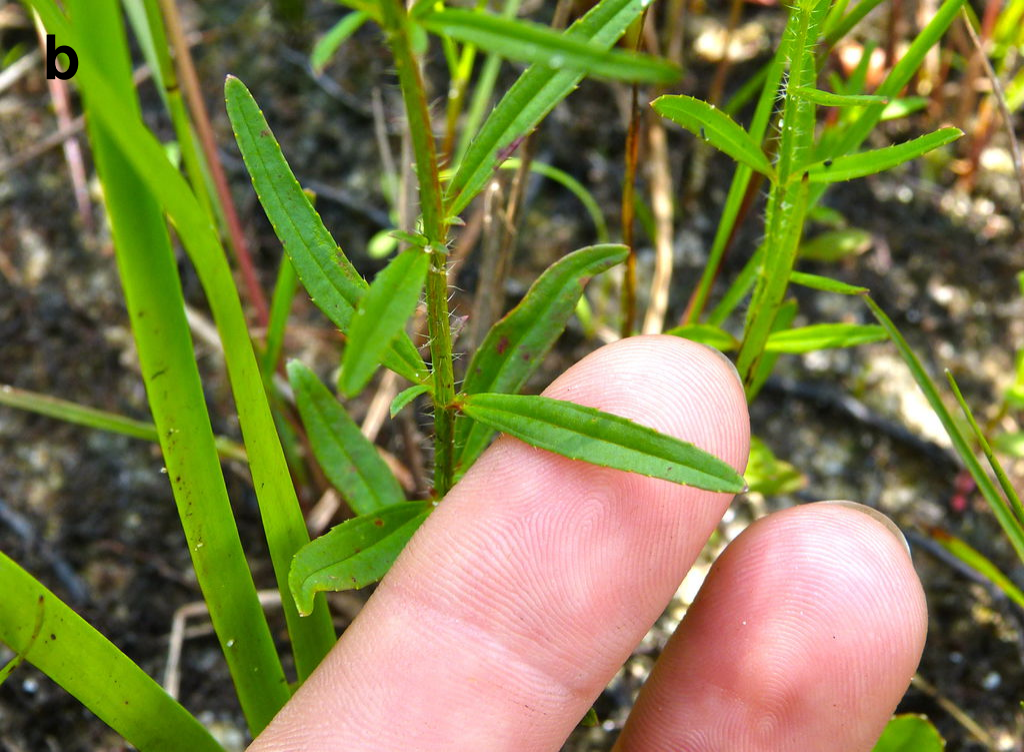
Leaves

**Figure 168c.**
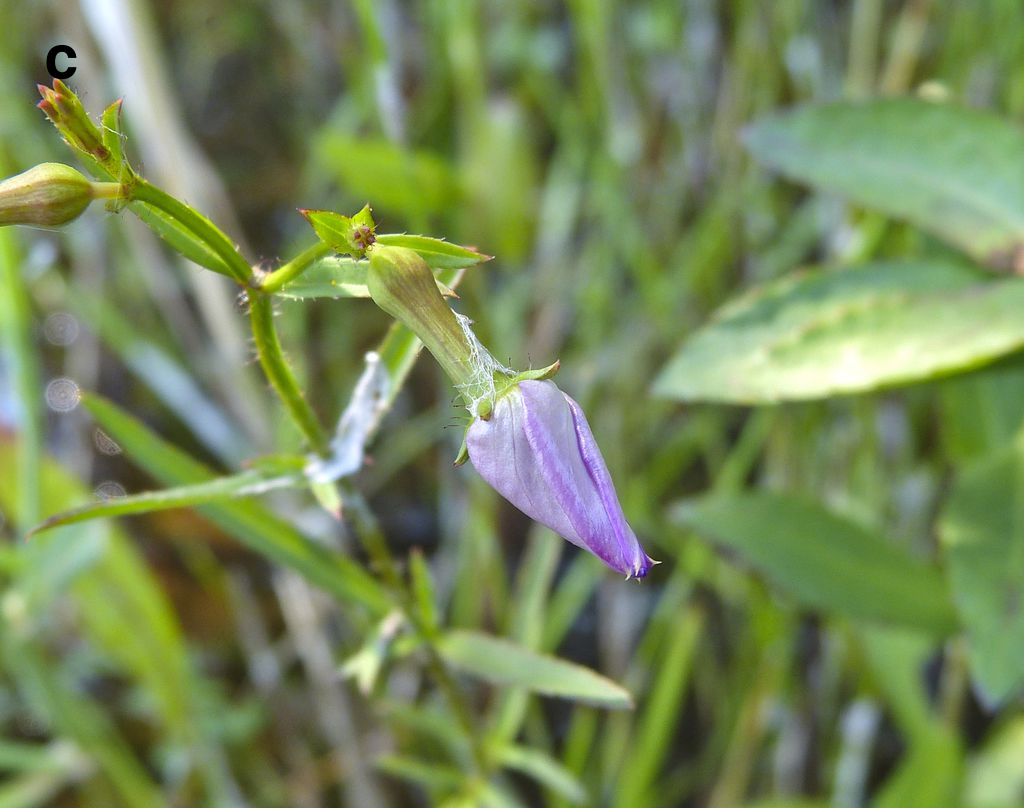
Flower (unopened)

**Figure 168d.**
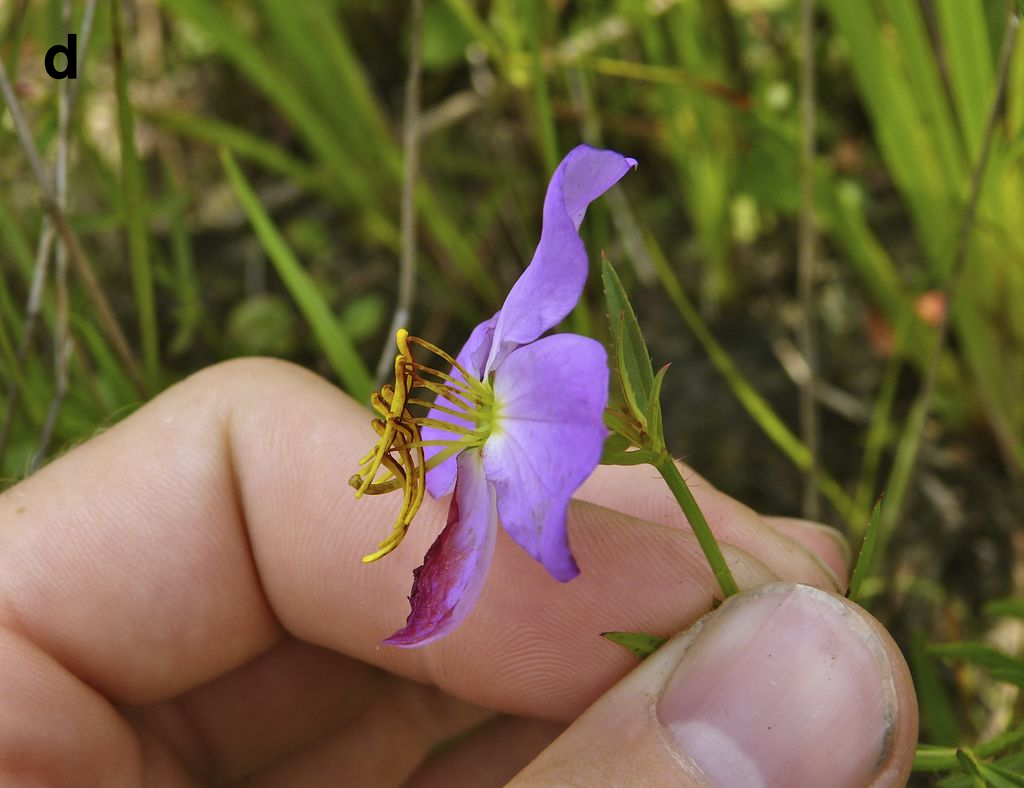
Flower (opened)

**Figure 169a.**
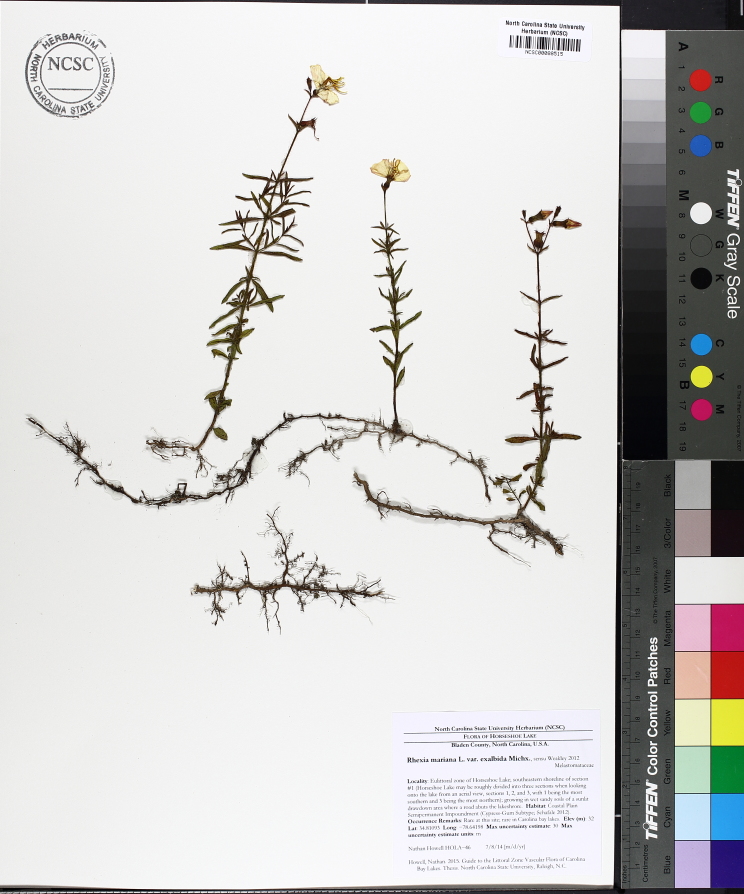
Specimen: *Howell HOLA-46* (NCSC)

**Figure 169b.**
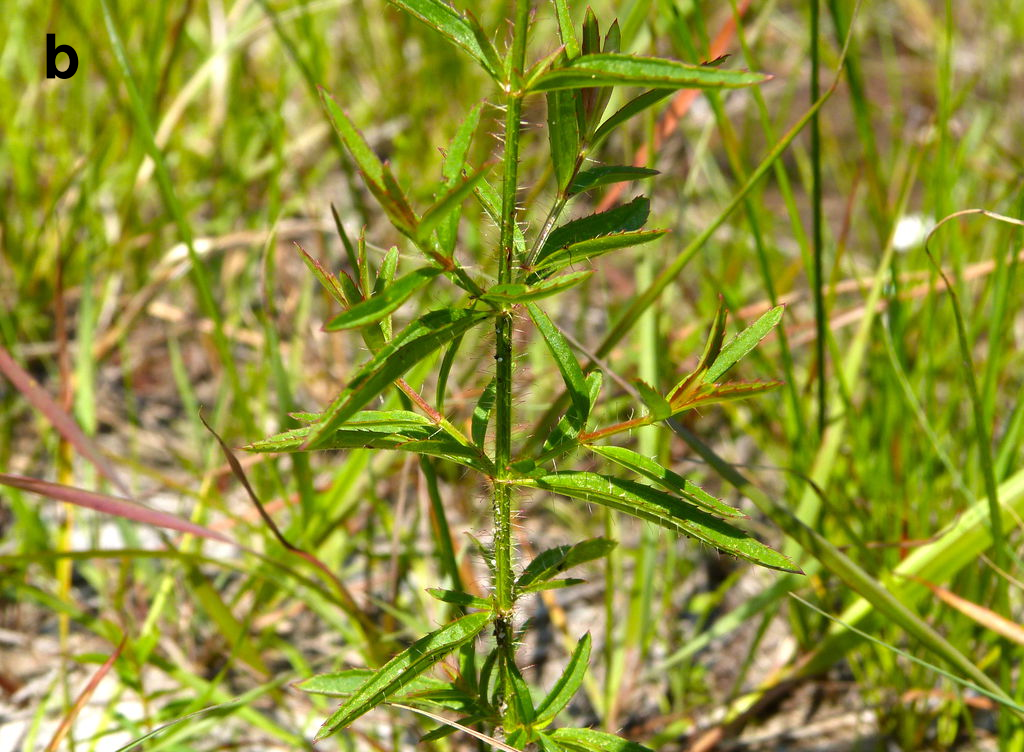
Leaves

**Figure 169c.**
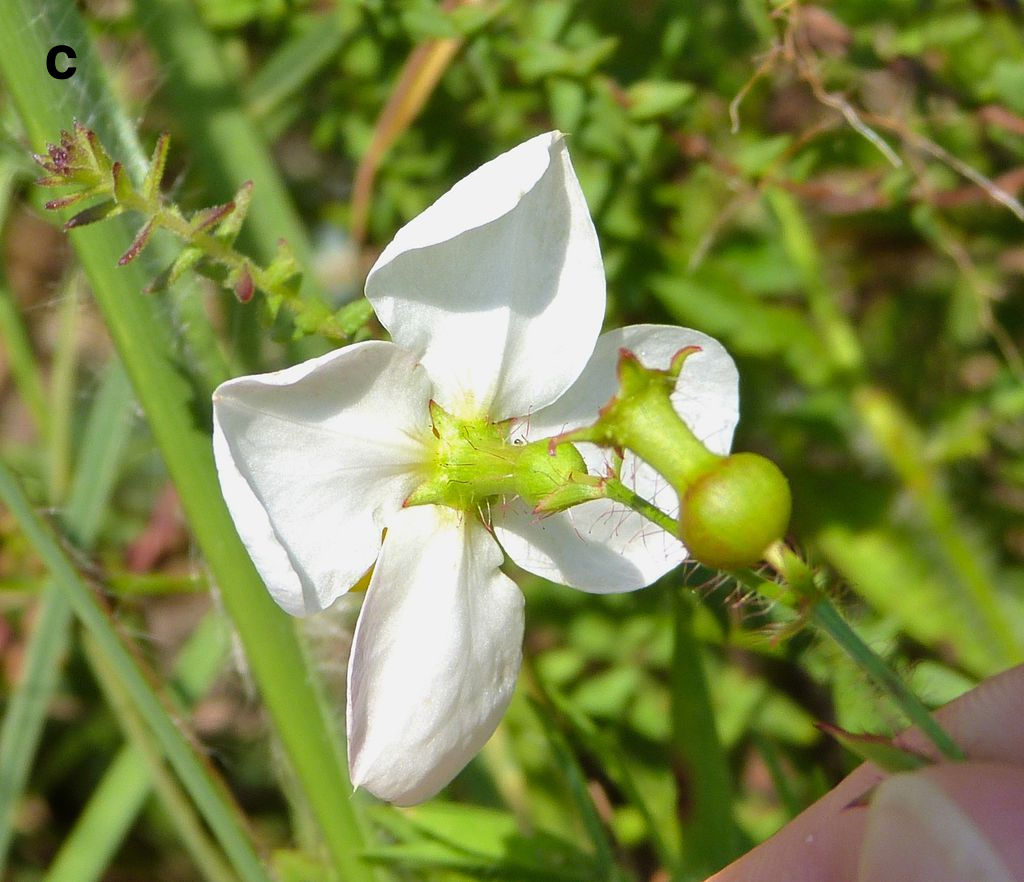
Flower (abaxial)

**Figure 169d.**
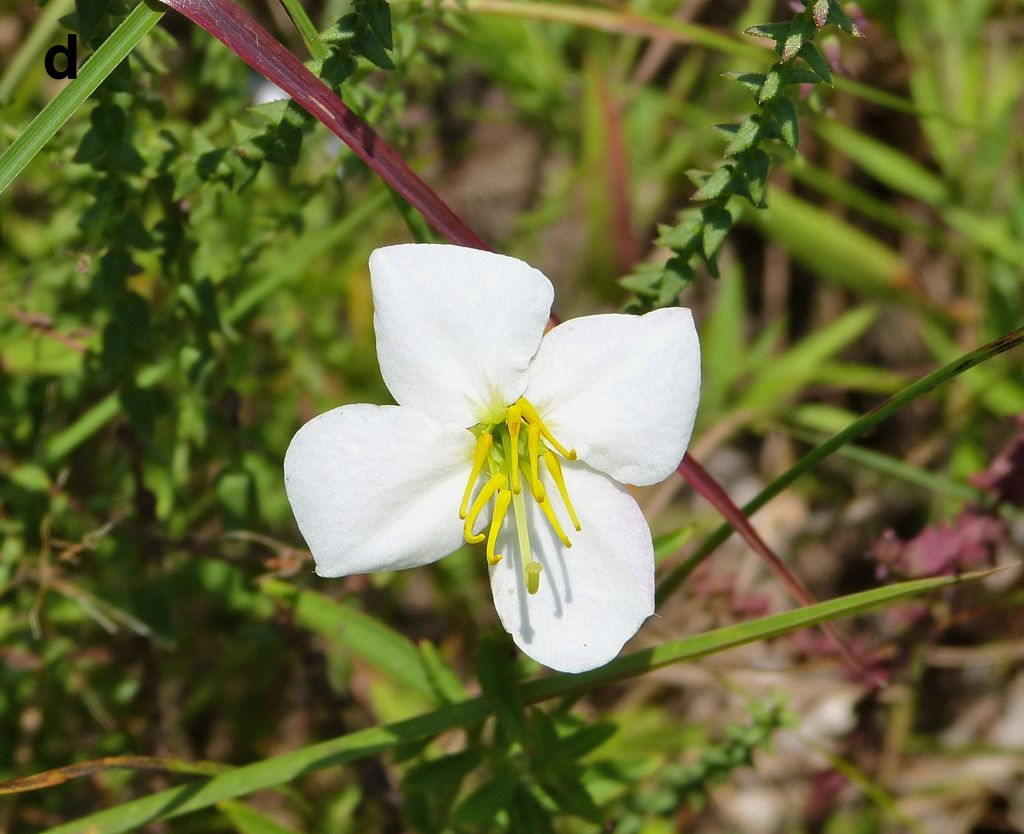
Flower (adaxial)

**Figure 170a.**
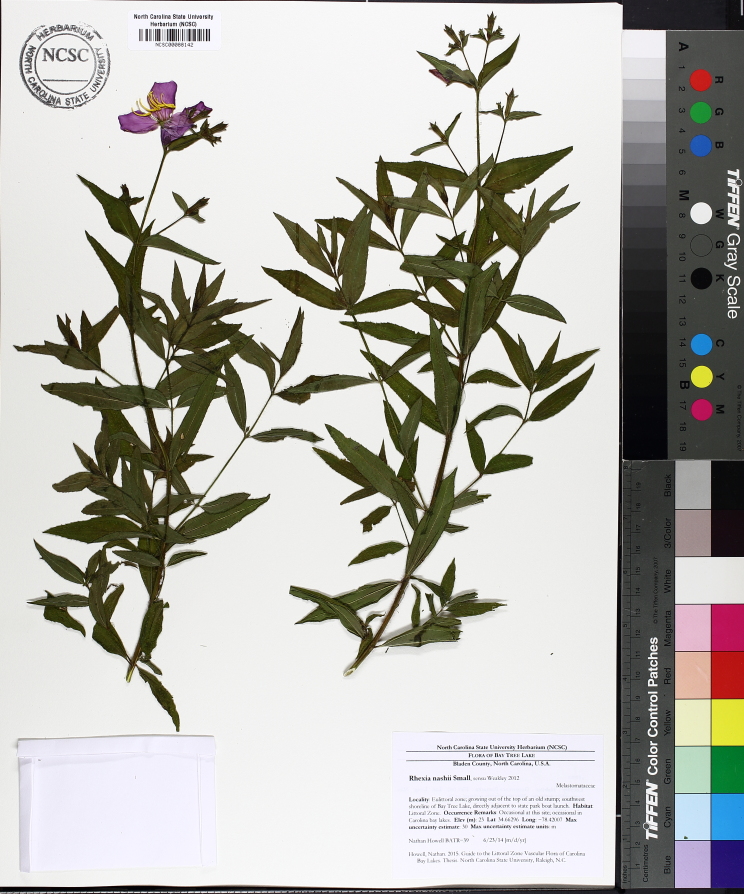
Specimen: *Howell BATR-39* (NCSC)

**Figure 170b.**
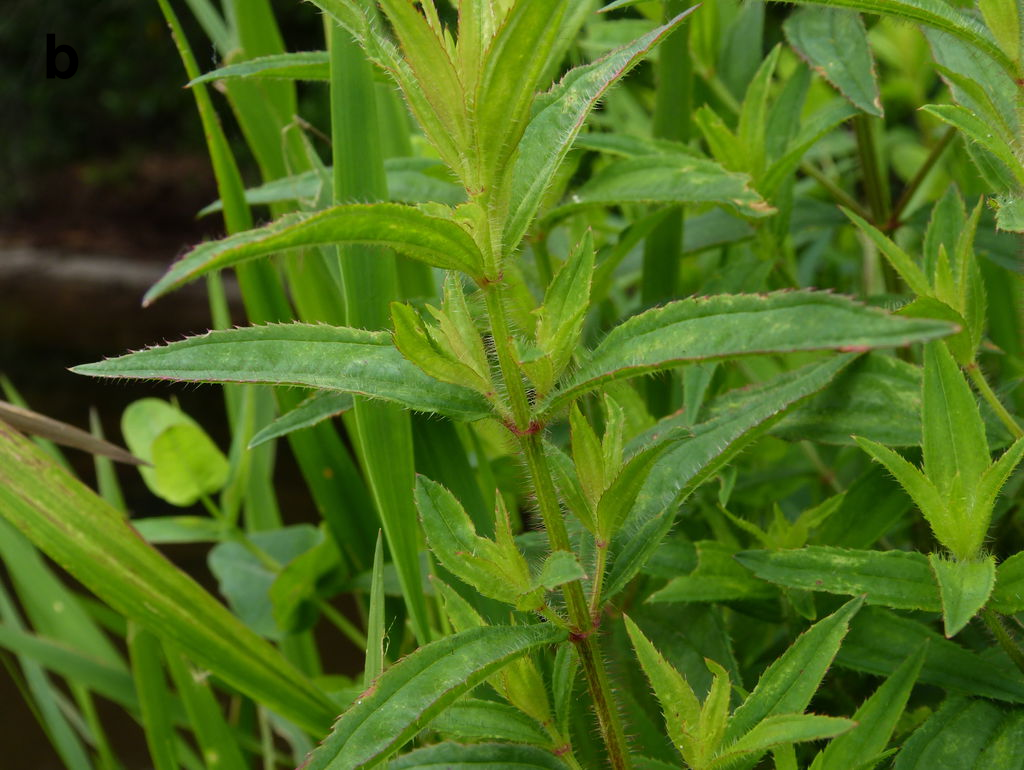
Leaves

**Figure 170c.**
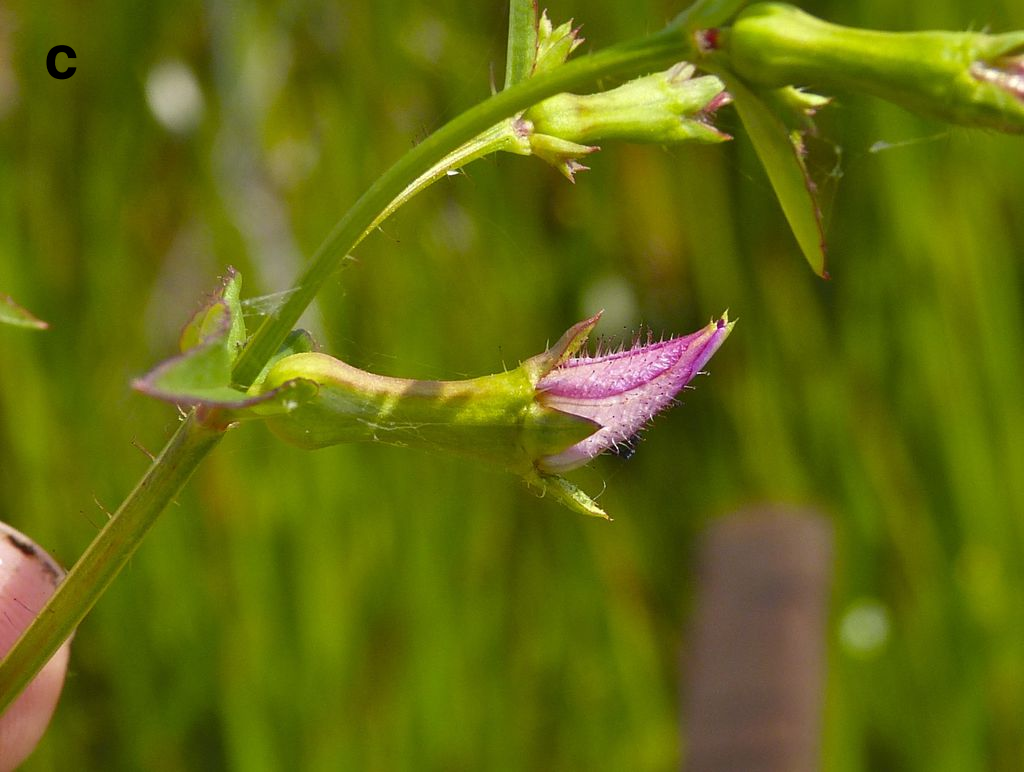
Calyx tube (and unopened corolla)

**Figure 170d.**
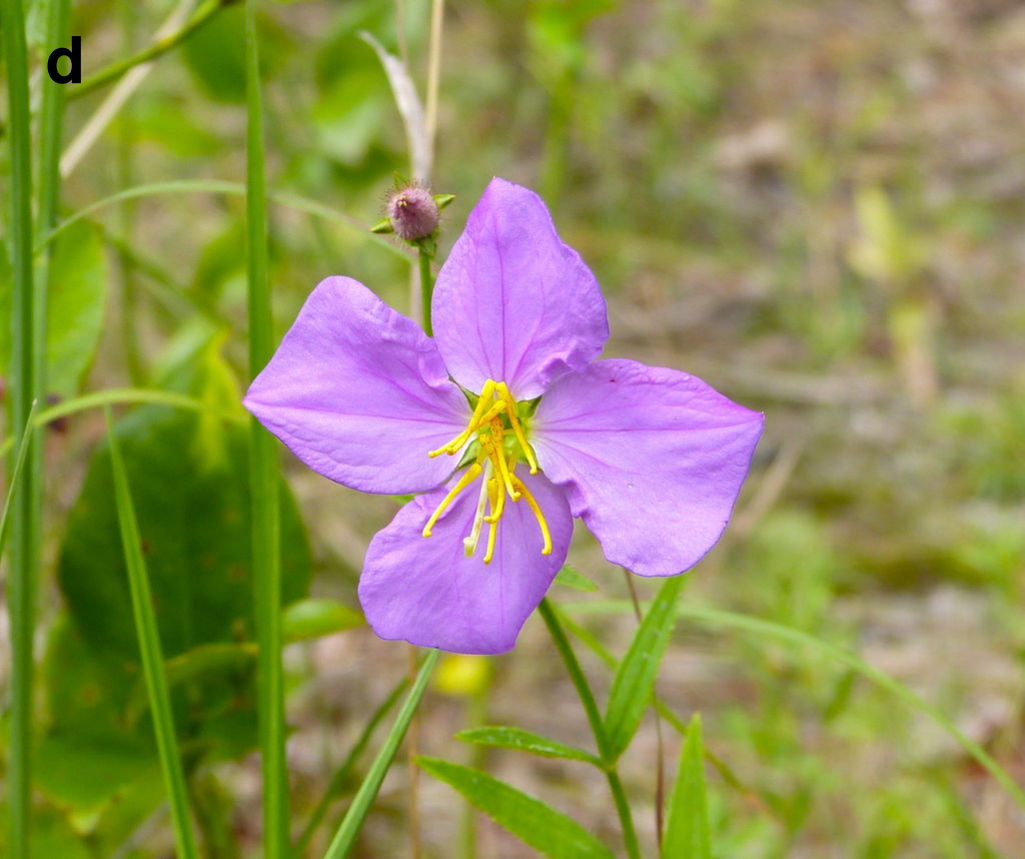
Flower

**Figure 171a.**
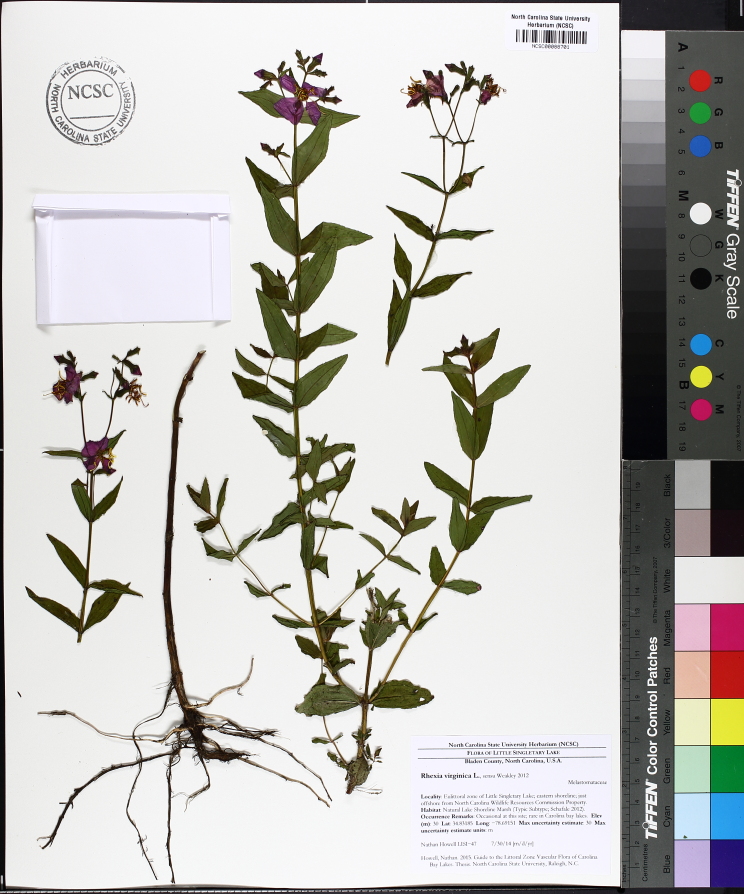
Specimen: *Howell LISI-47* (NCSC)

**Figure 171b.**
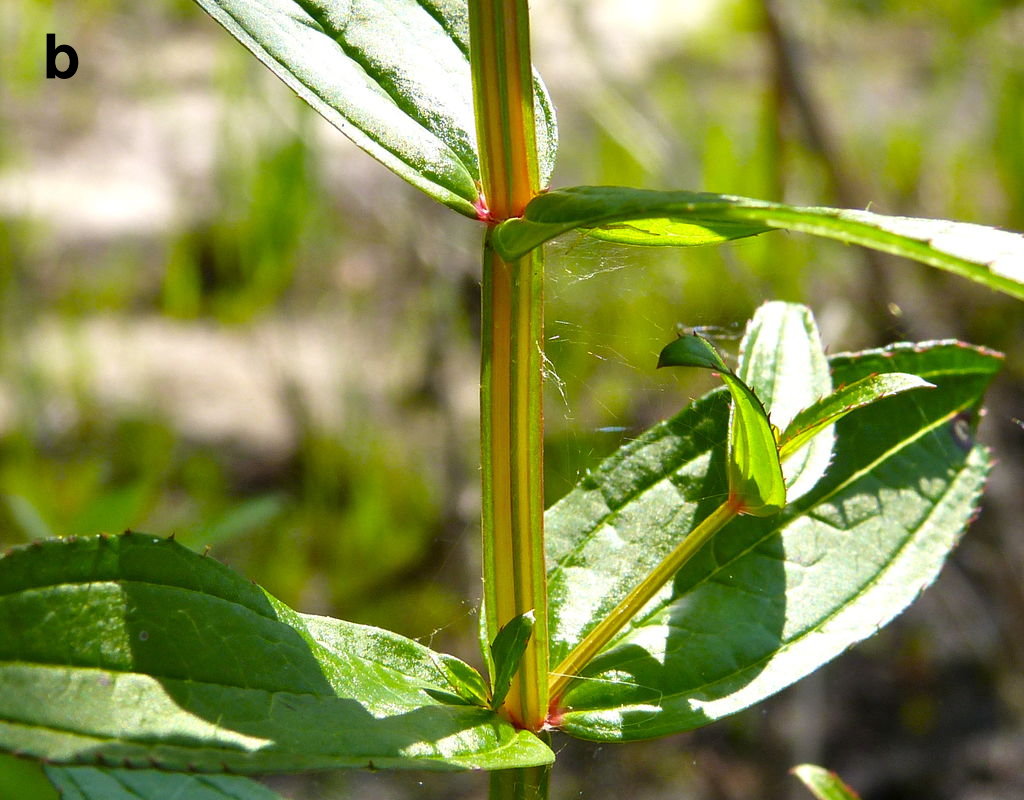
Stem and leaves

**Figure 171c.**
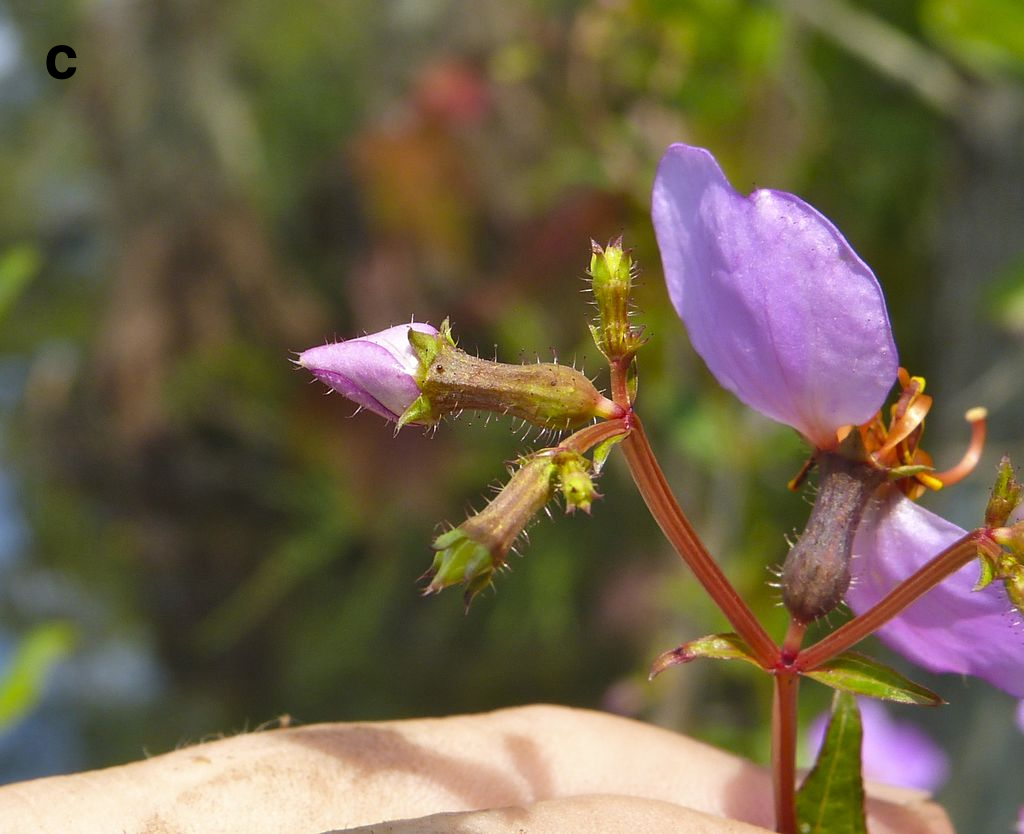
Flower (lateral)

**Figure 171d.**
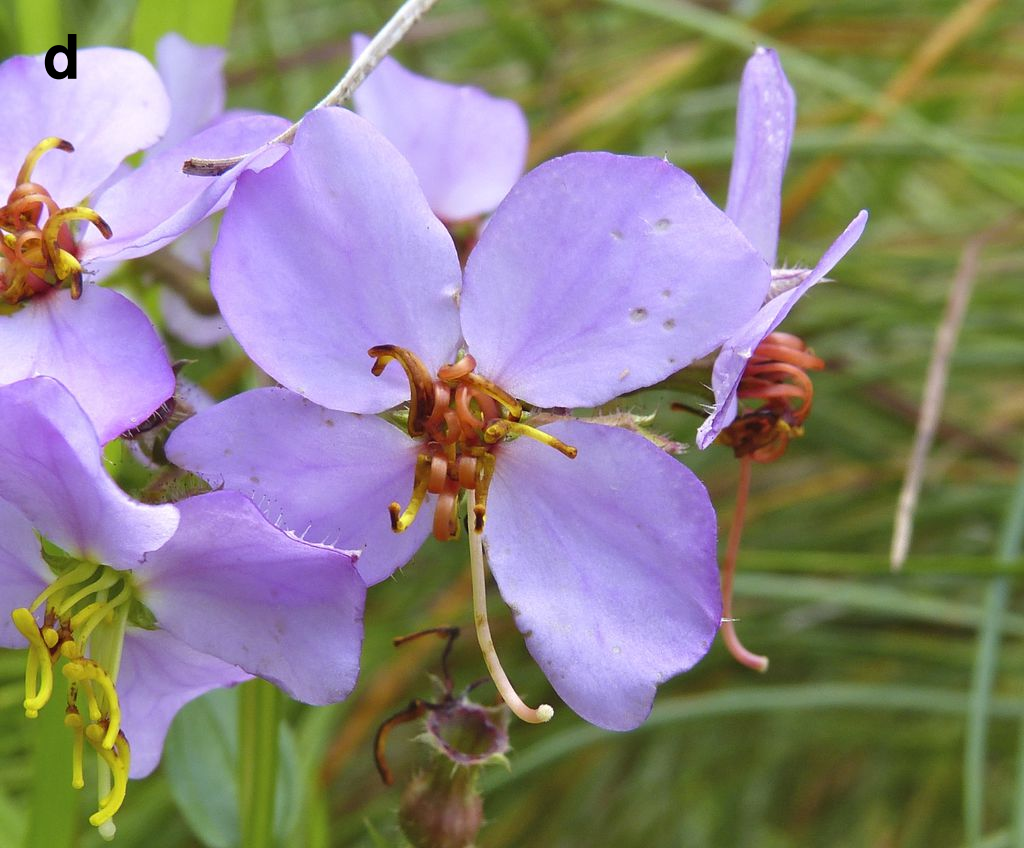
Flower (adaxial)

**Figure 172a.**
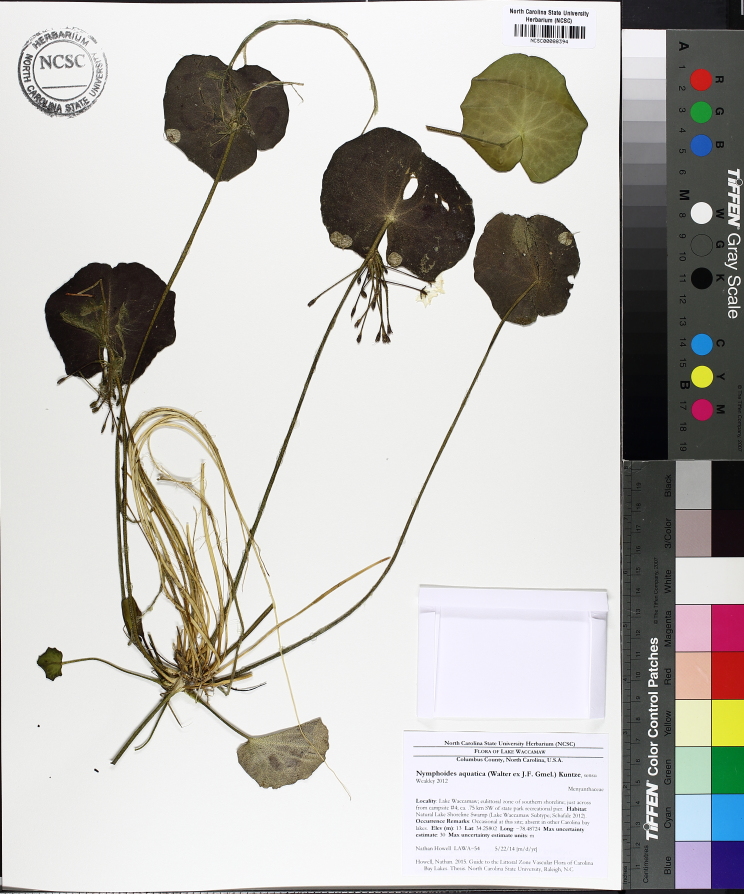
Specimen: *Howell LAWA-54* (NCSC)

**Figure 172b.**
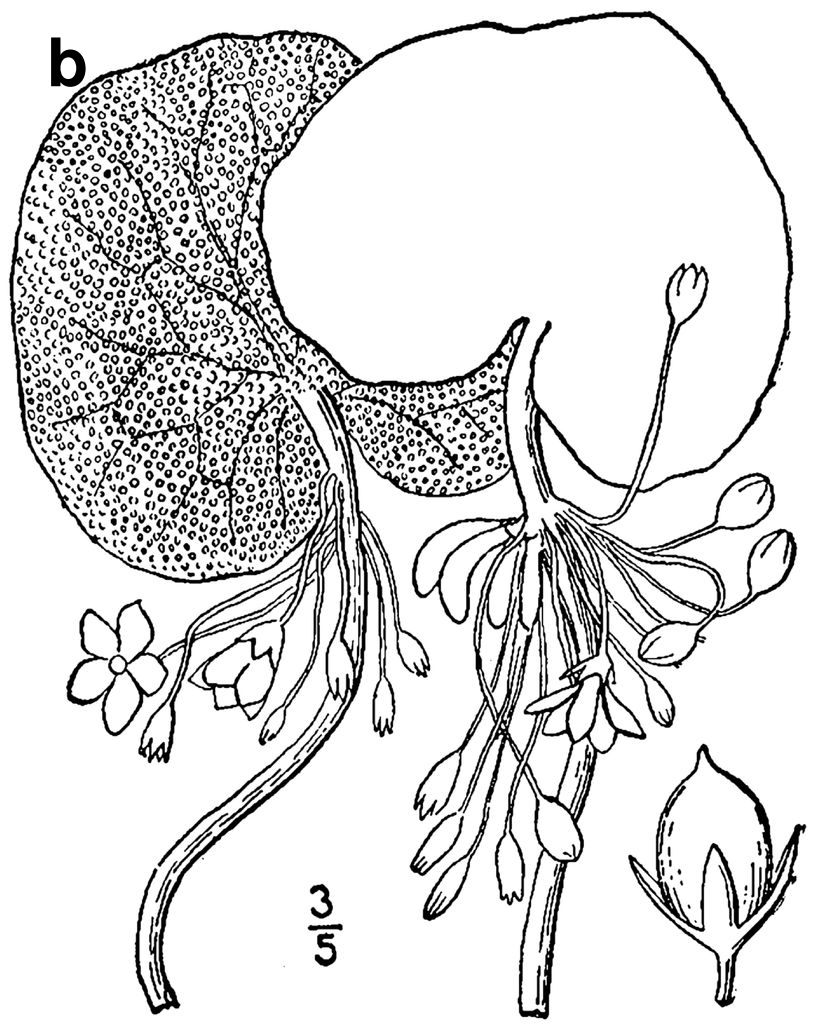
Illustration

**Figure 172c.**
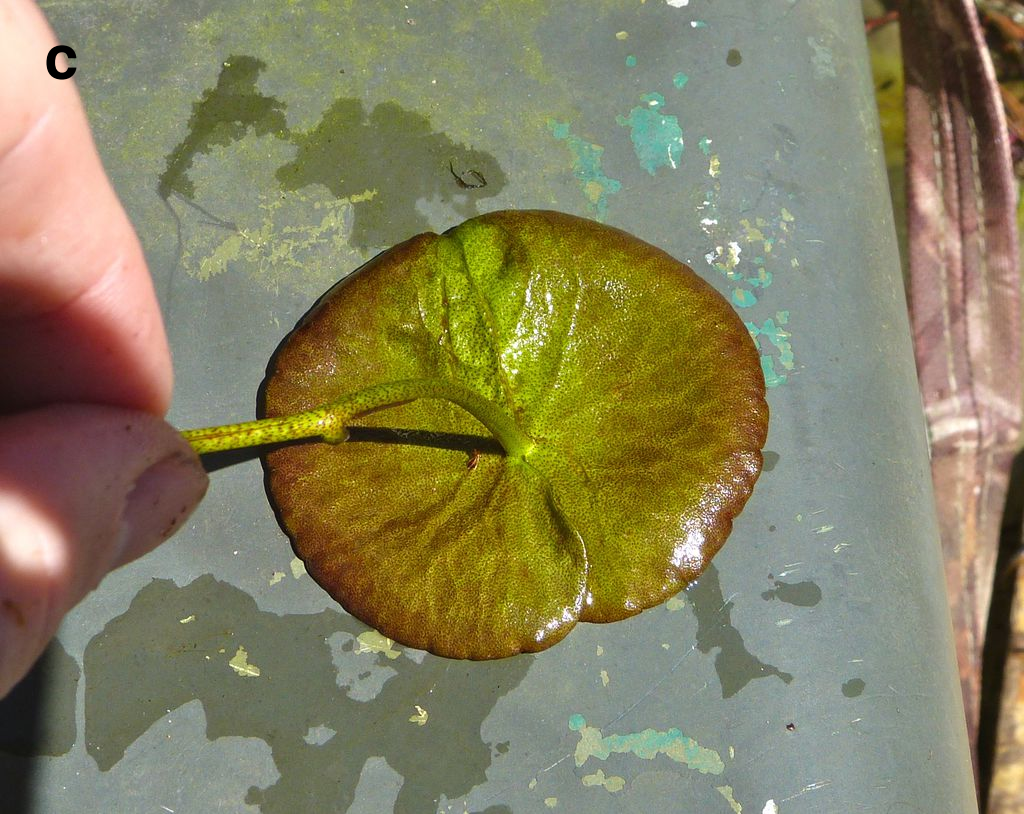
Leaf (abaxial surface)

**Figure 172d.**
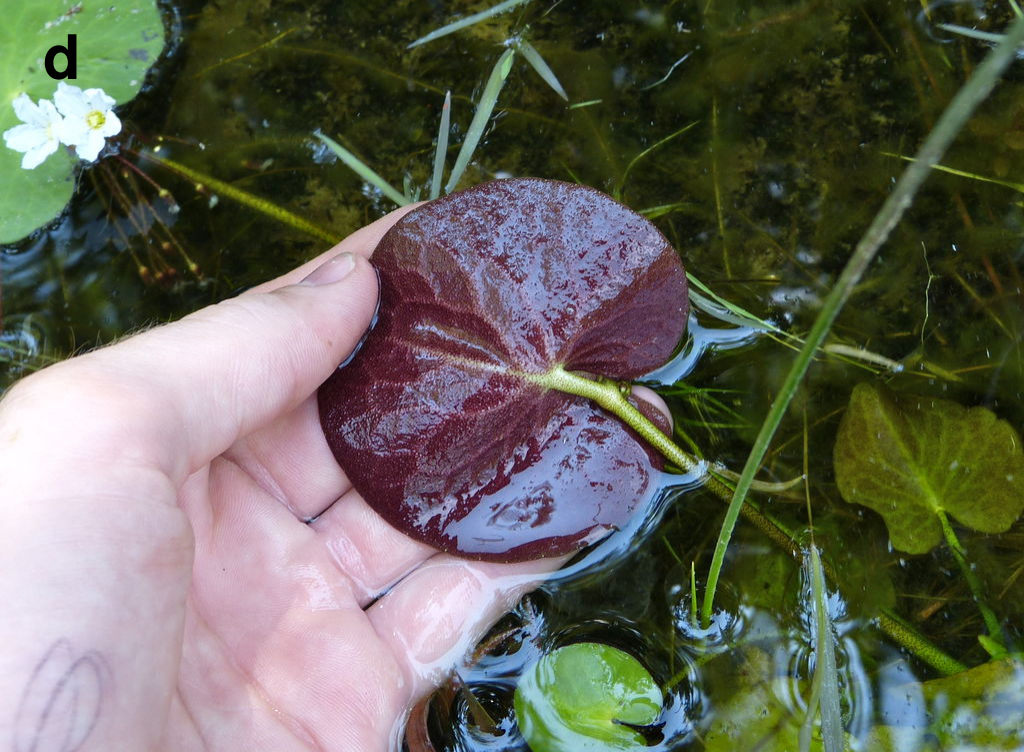
Leaf (abaxial surface)

**Figure 172e.**
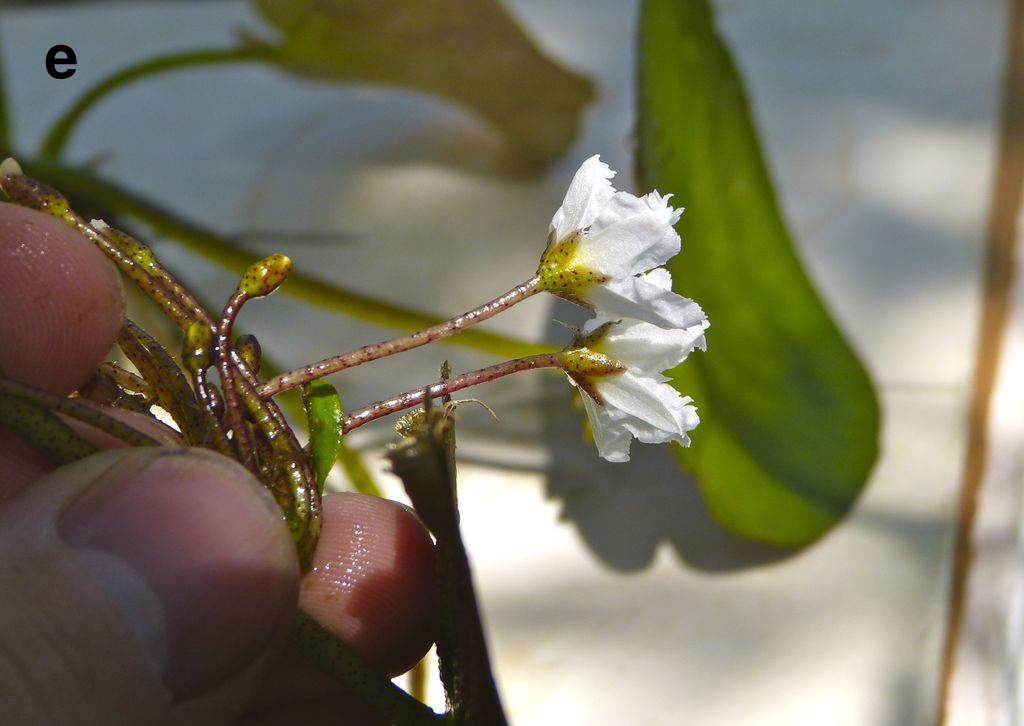
Flowers

**Figure 172f.**
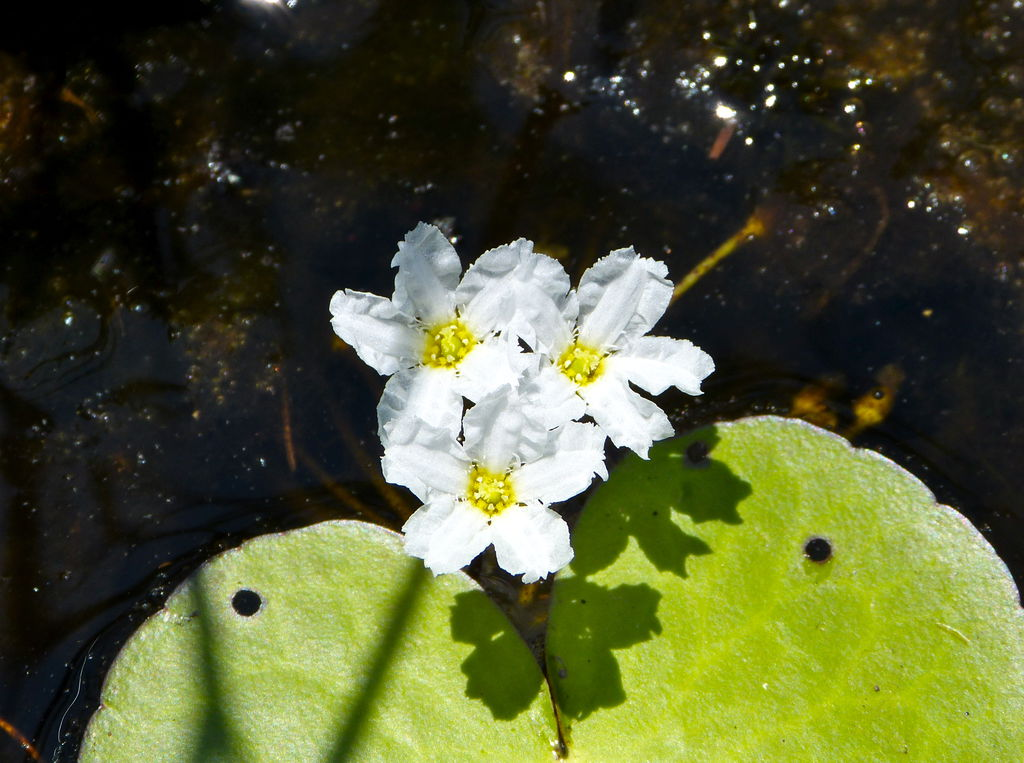
Flowers

**Figure 173a.**
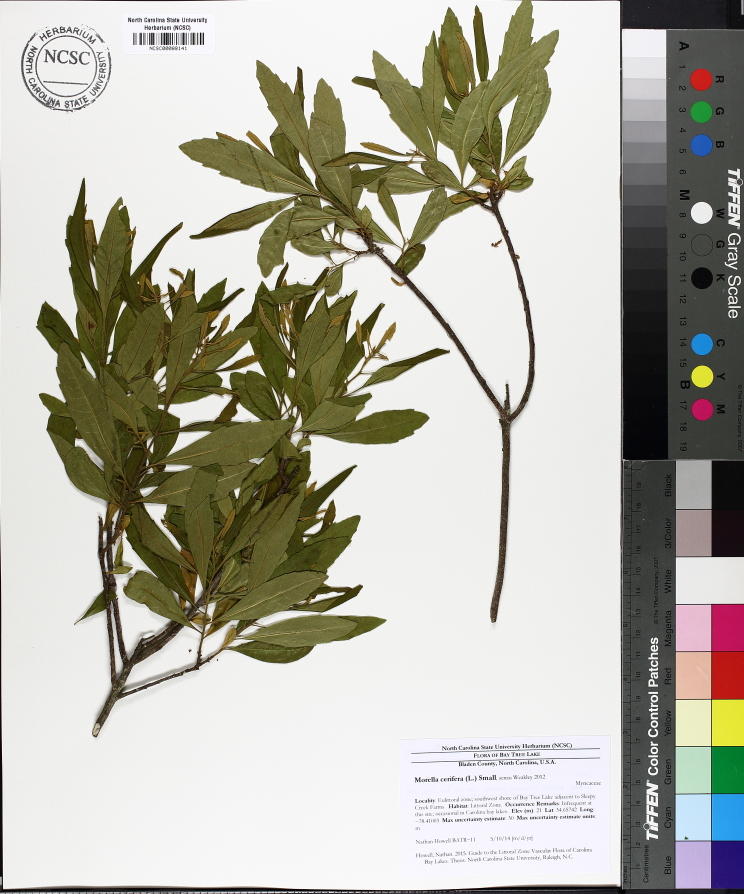
Specimen: *Howell BATR-11* (NCSC)

**Figure 173b.**
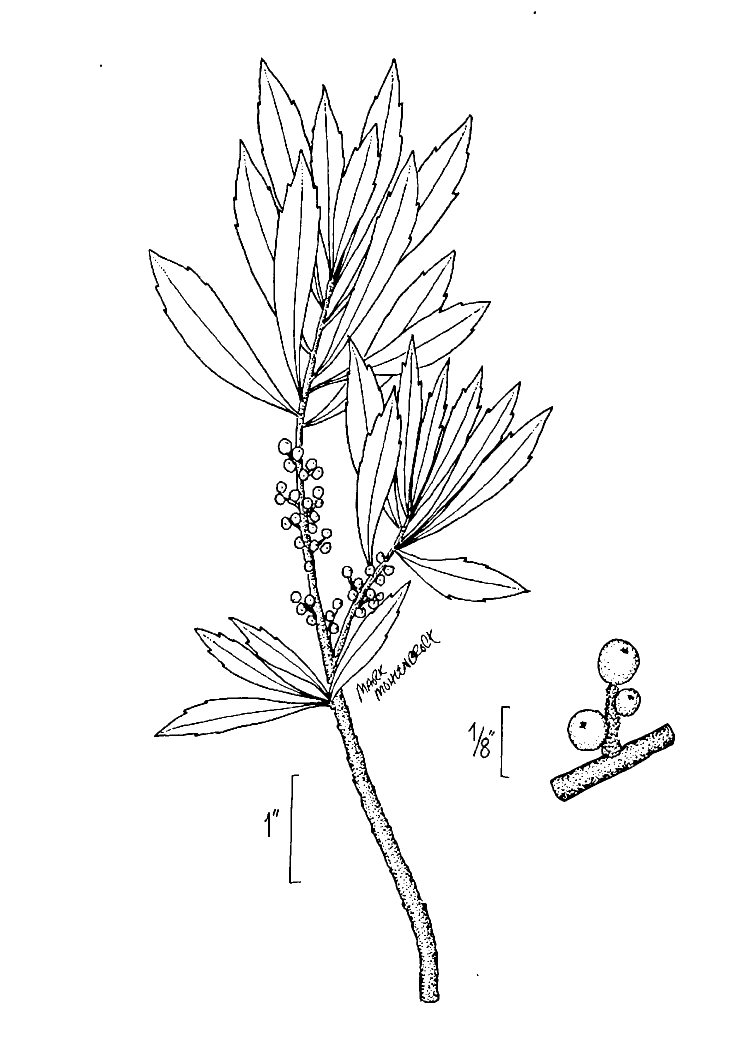
lllustration

**Figure 173c.**
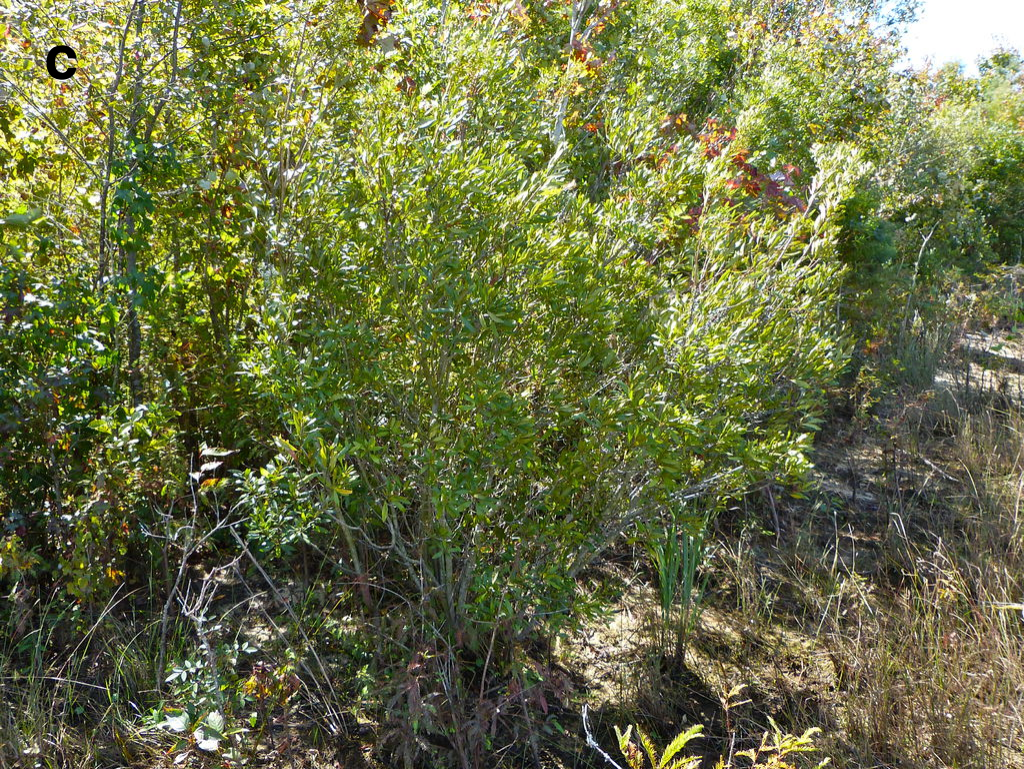
Habit

**Figure 173d.**
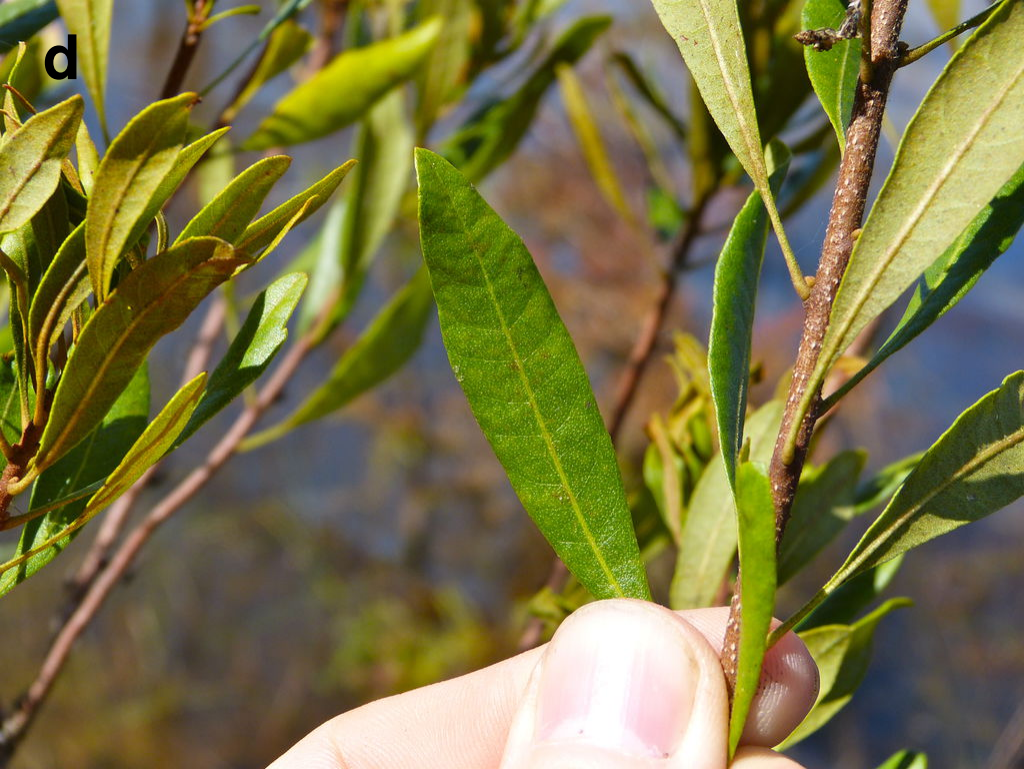
Leaf

**Figure 173e.**
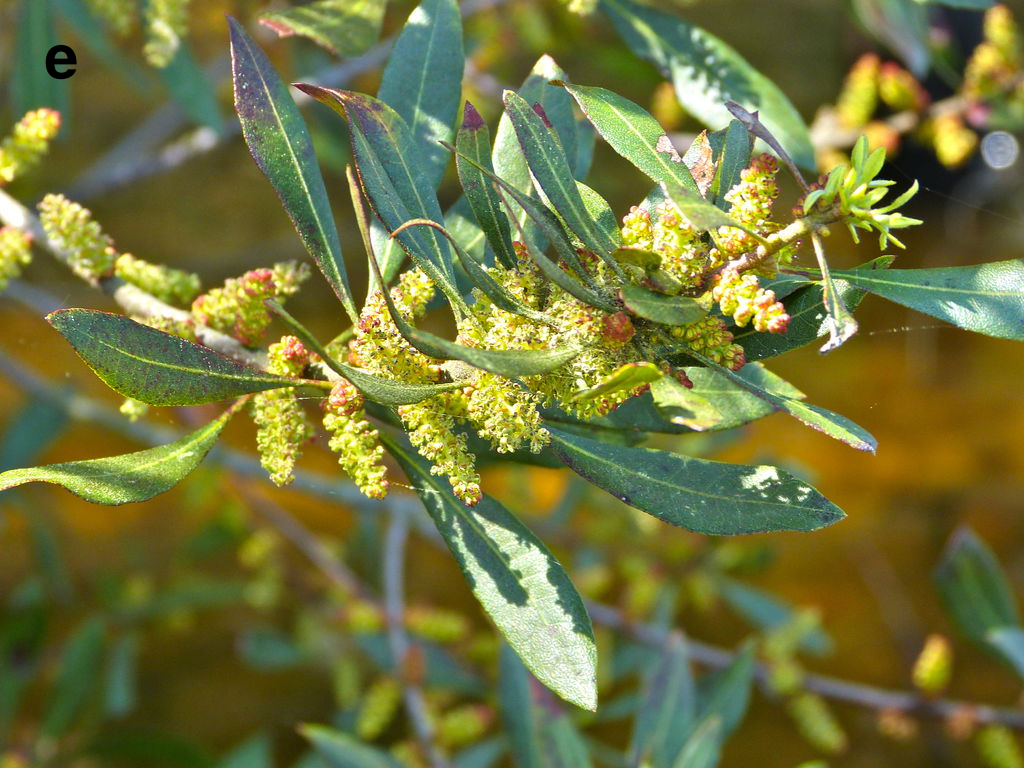
Staminate inflorescence

**Figure 173f.**
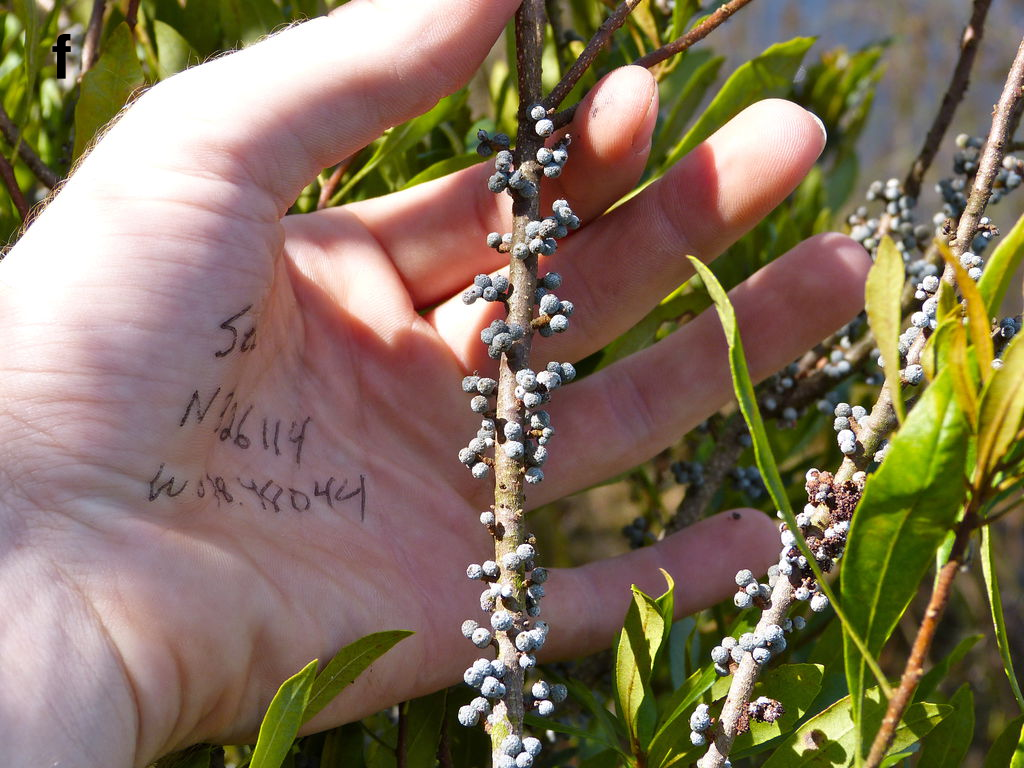
Fruits

**Figure 175a.**
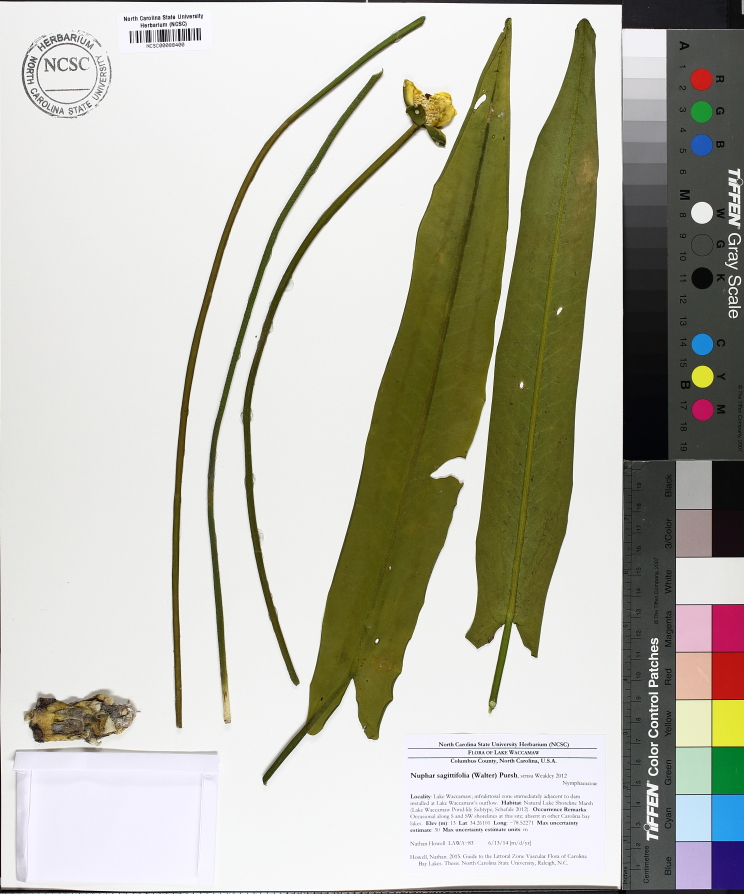
Specimen: *Howell LAWA-83* (NCSC)

**Figure 175b.**
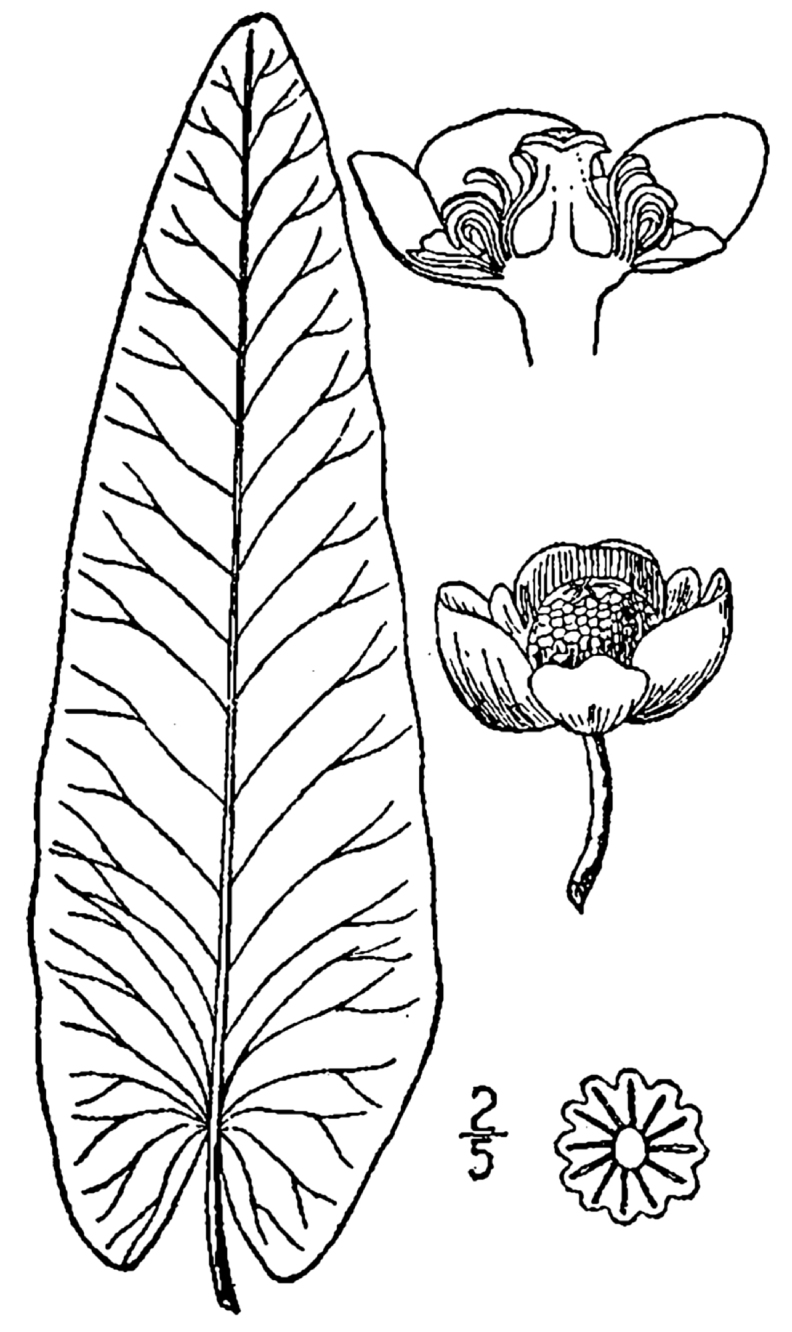
Illustration

**Figure 175c.**
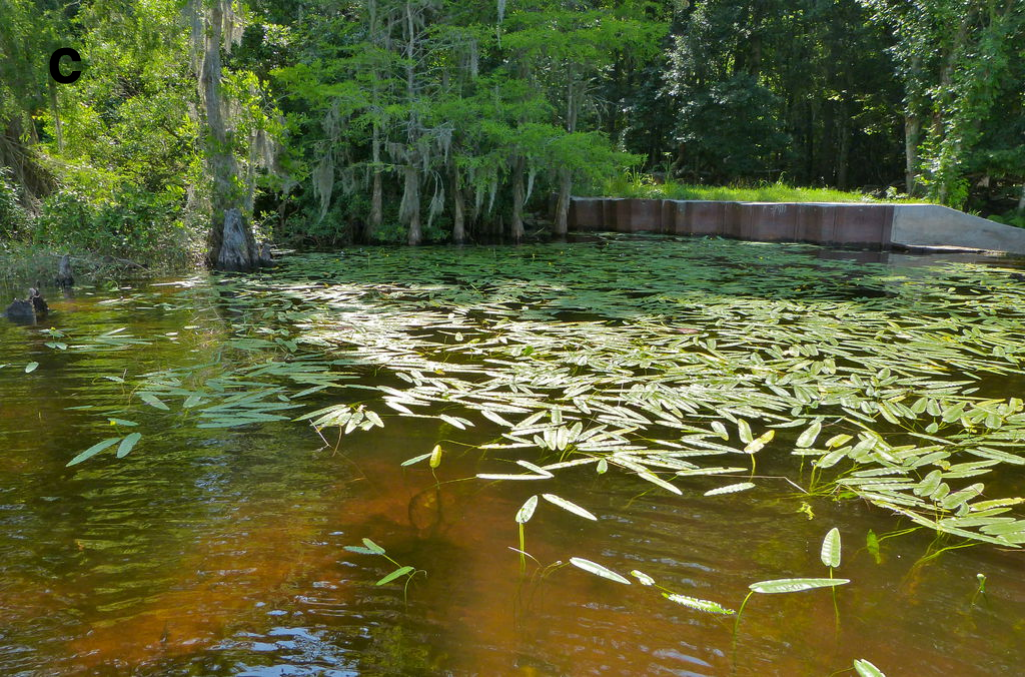
Habit

**Figure 175d.**
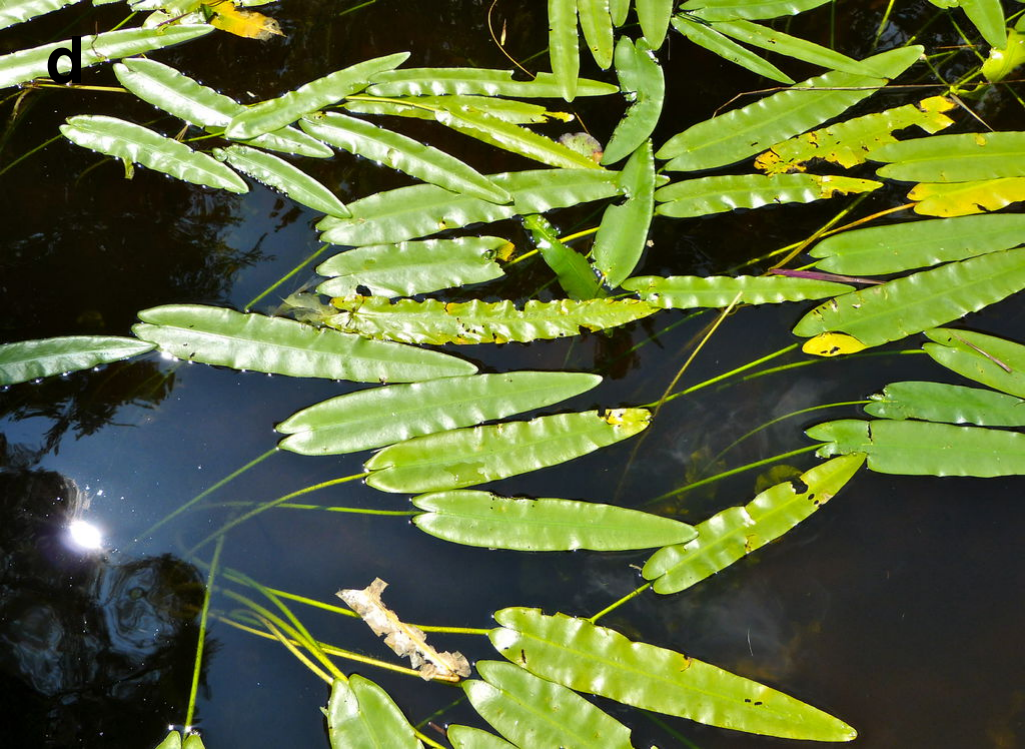
Leaves

**Figure 175e.**
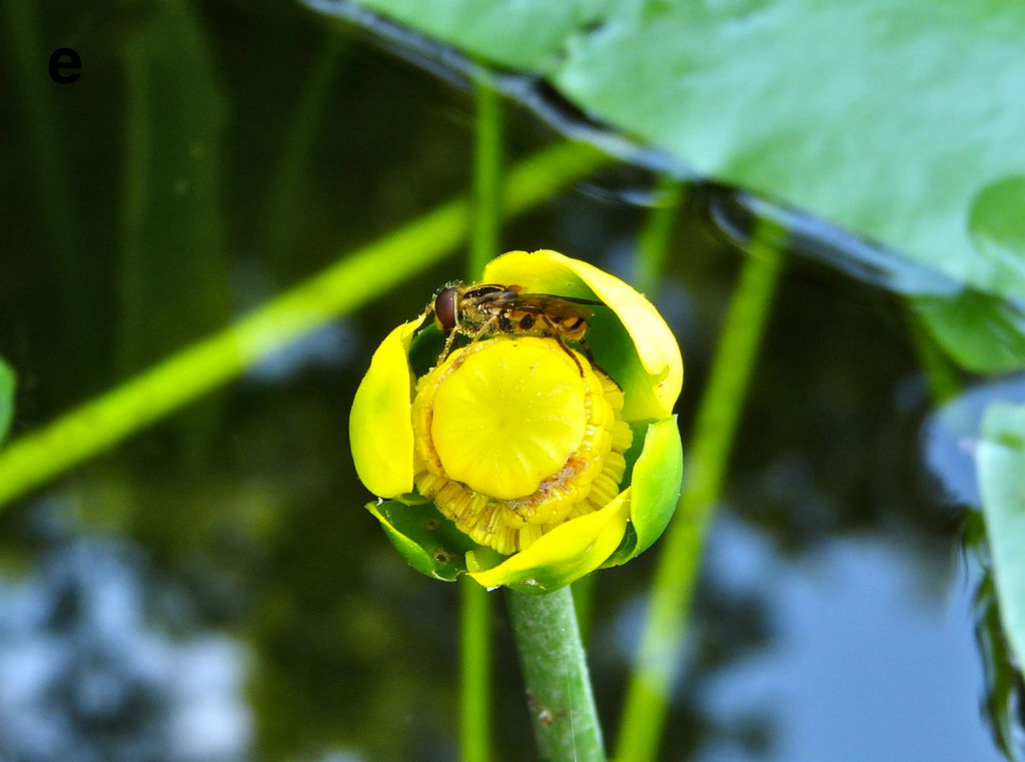
Flower

**Figure 175f.**
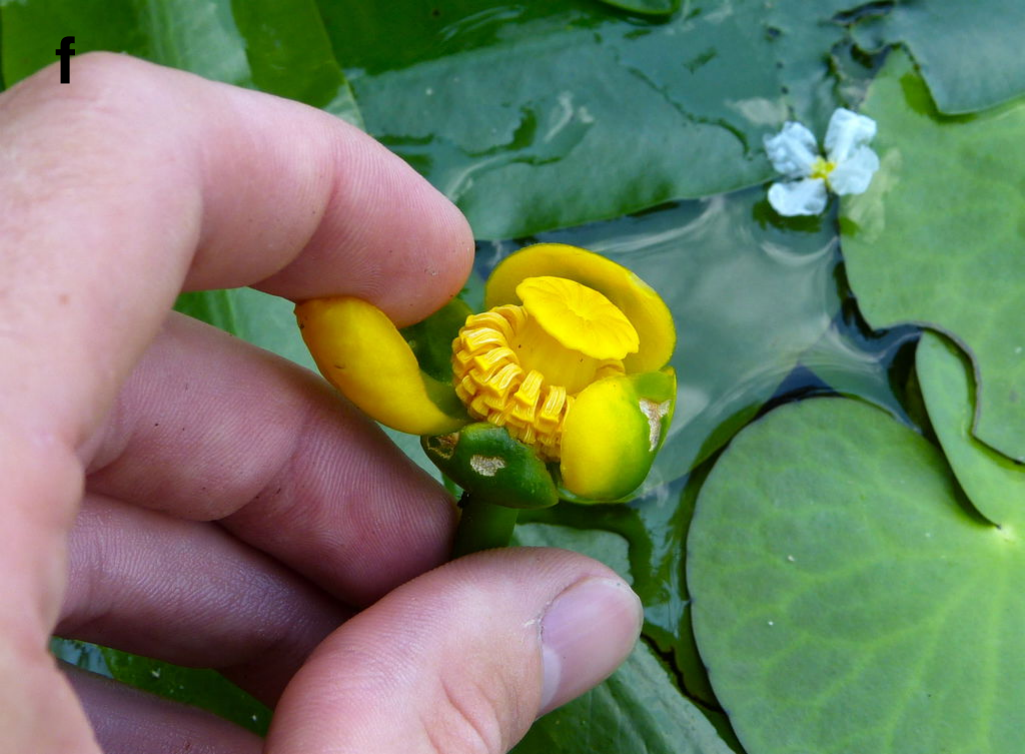
Flower

**Figure 176a.**
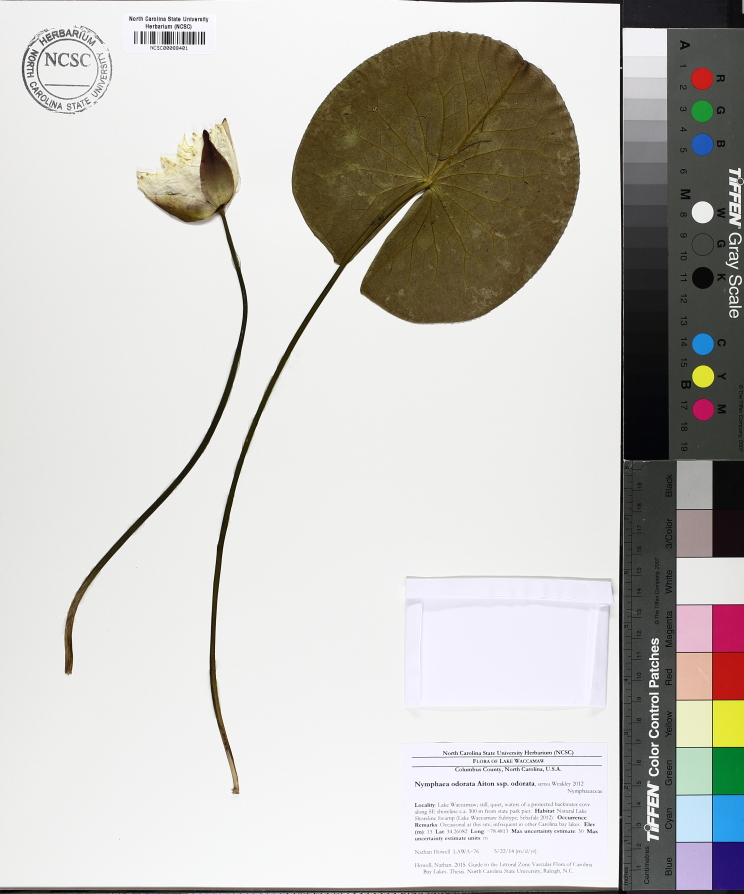
Specimen: *Howell LAWA-76* (NCSC)

**Figure 176b.**
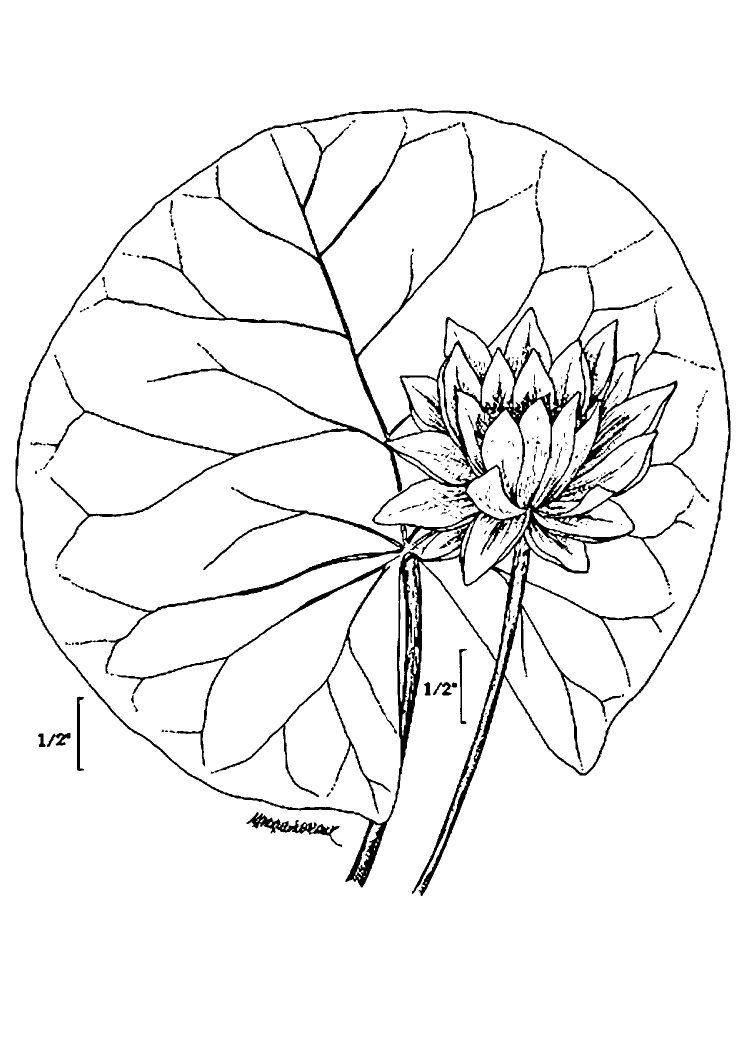
Illustration

**Figure 176c.**
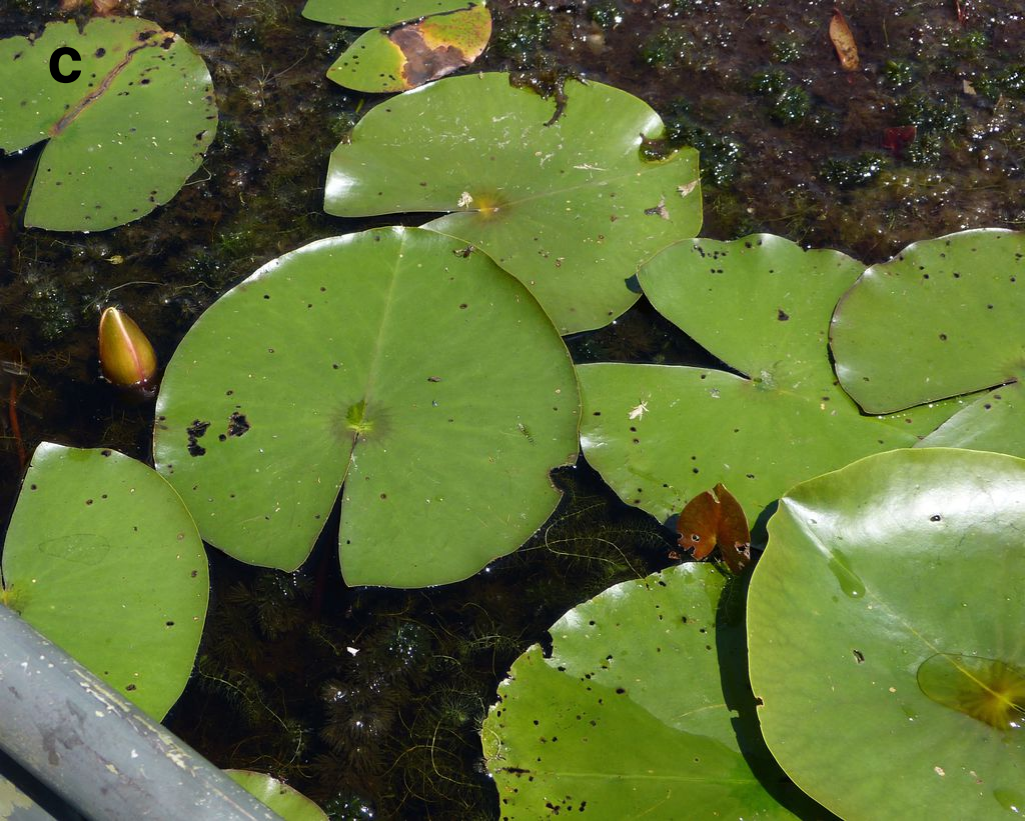
Leaves

**Figure 176d.**
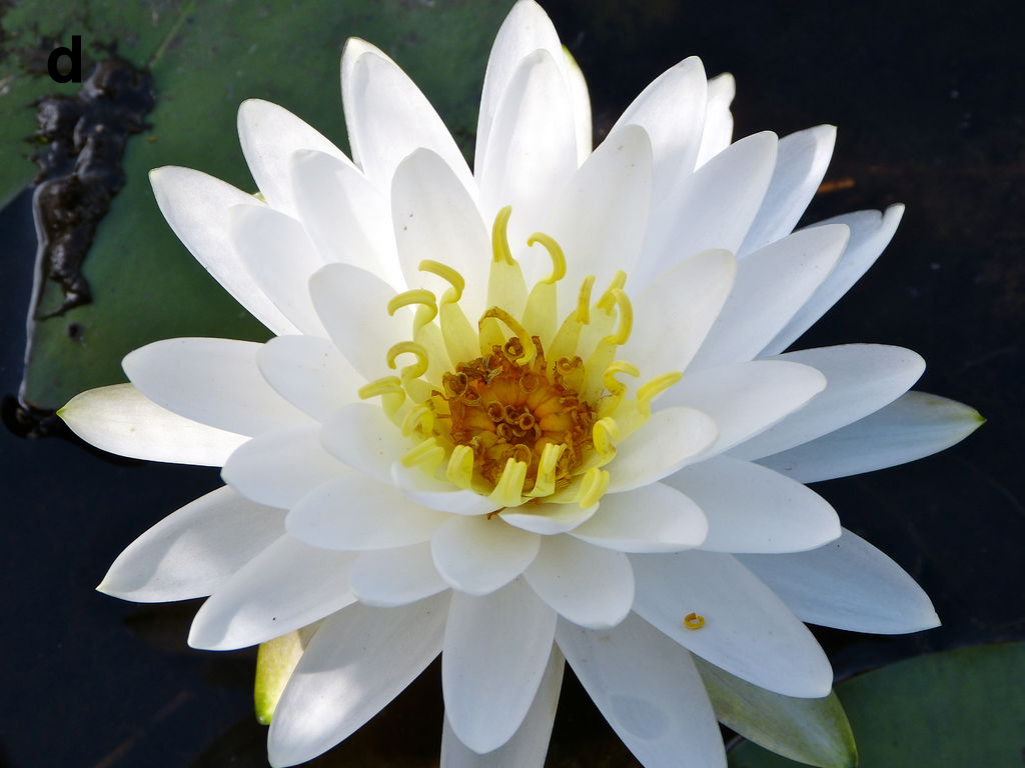
Flower

**Figure 177a.**
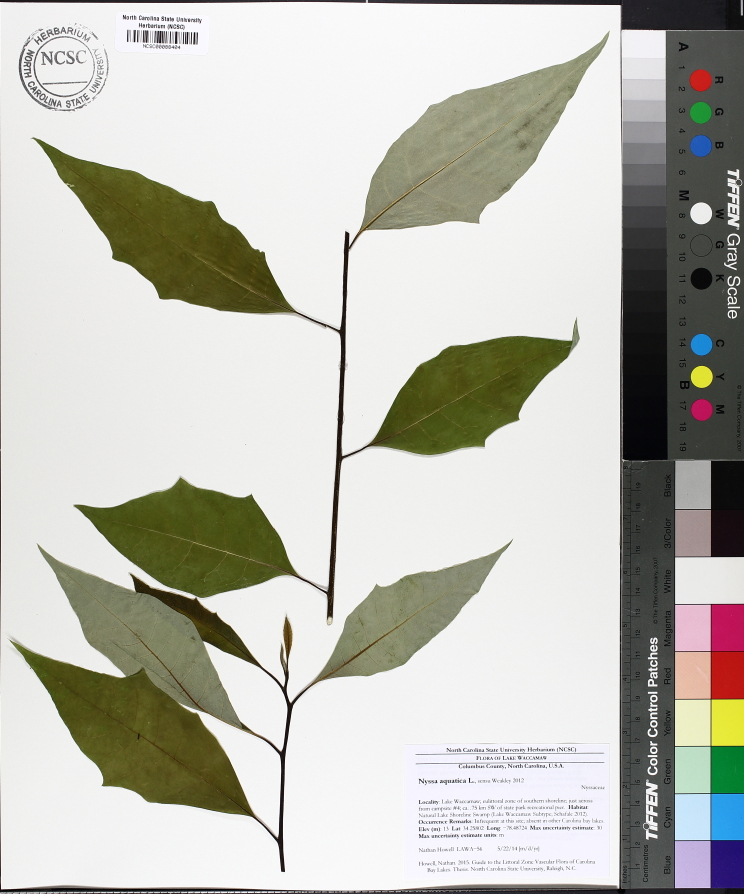
Specimen: *Howell LAWA-56* (NCSC)

**Figure 177b.**
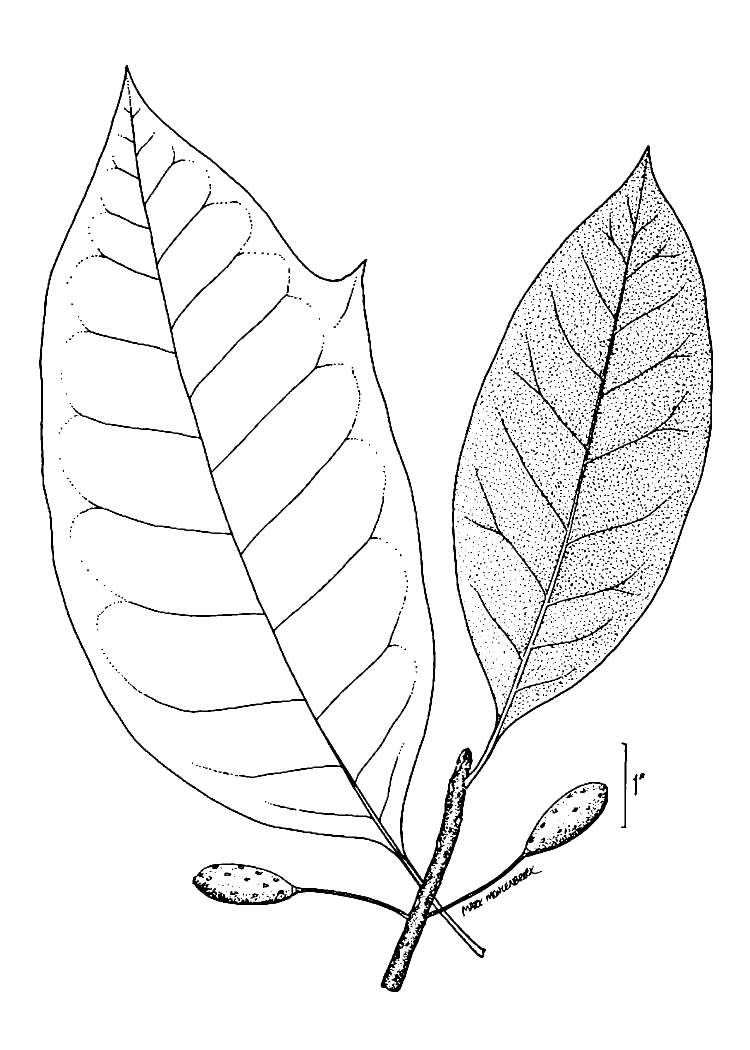
Illustration

**Figure 177c.**
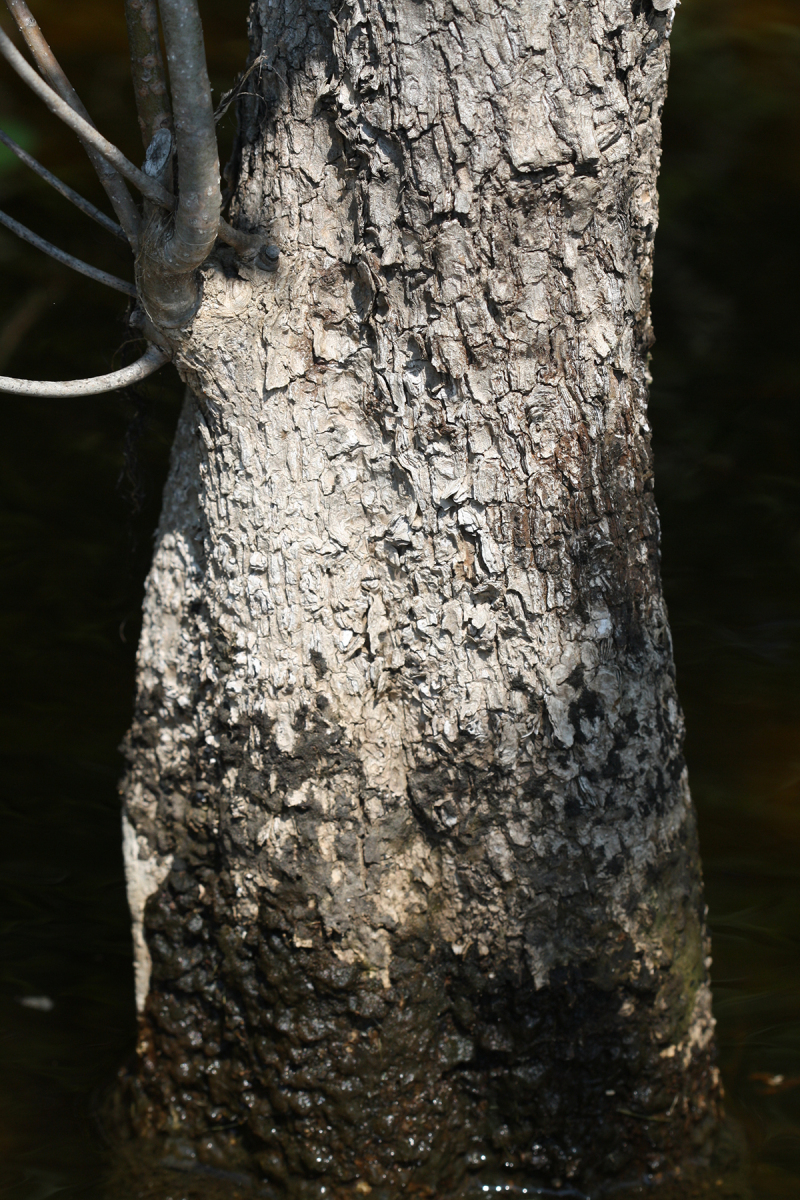
Bark

**Figure 177d.**
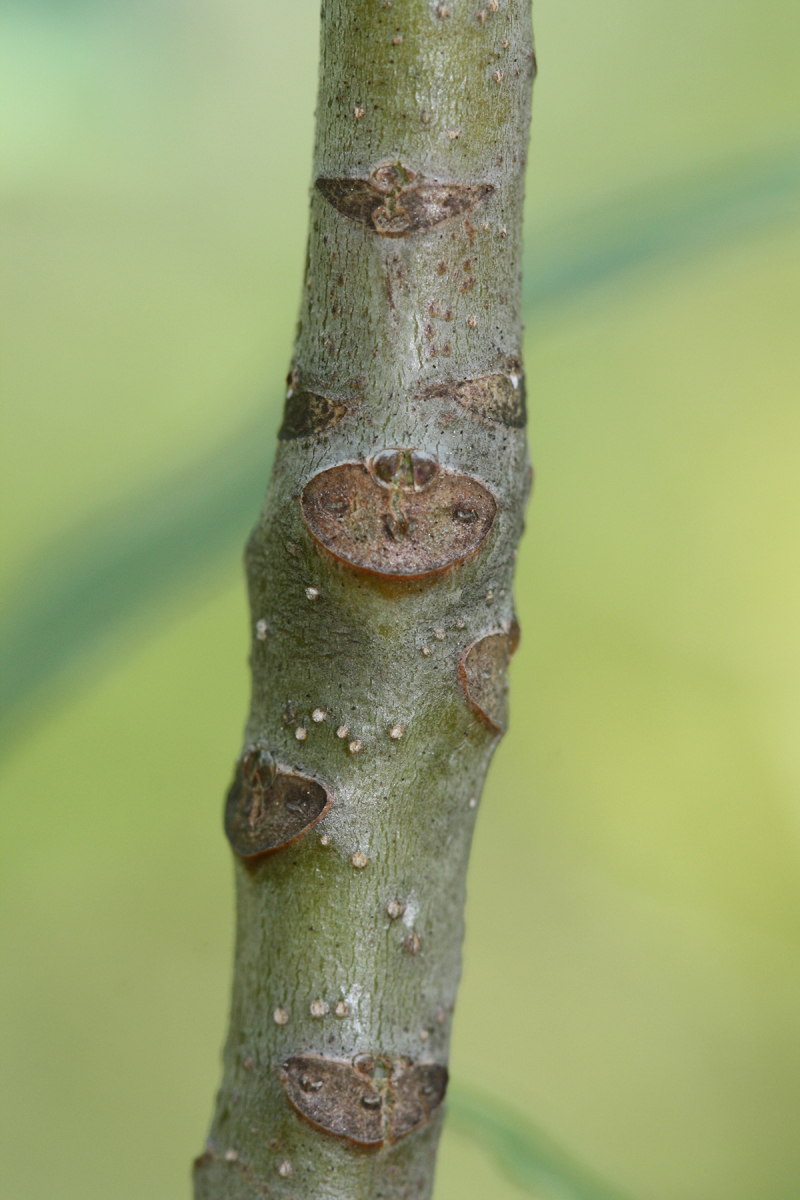
Twig

**Figure 177e.**
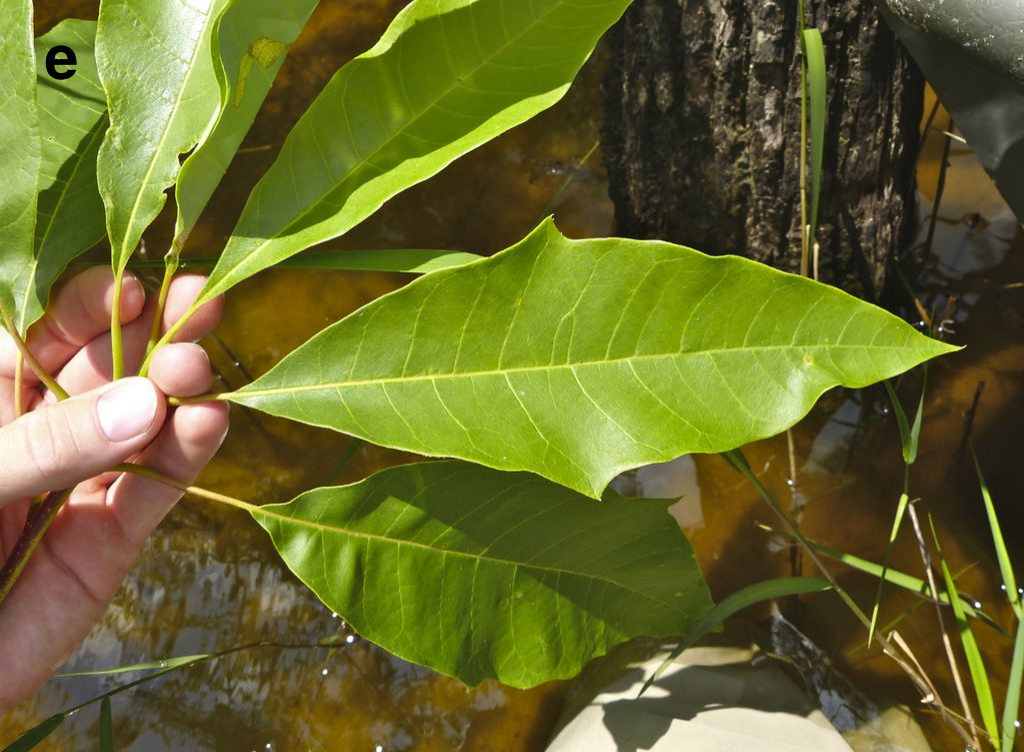
Leaves

**Figure 177f.**
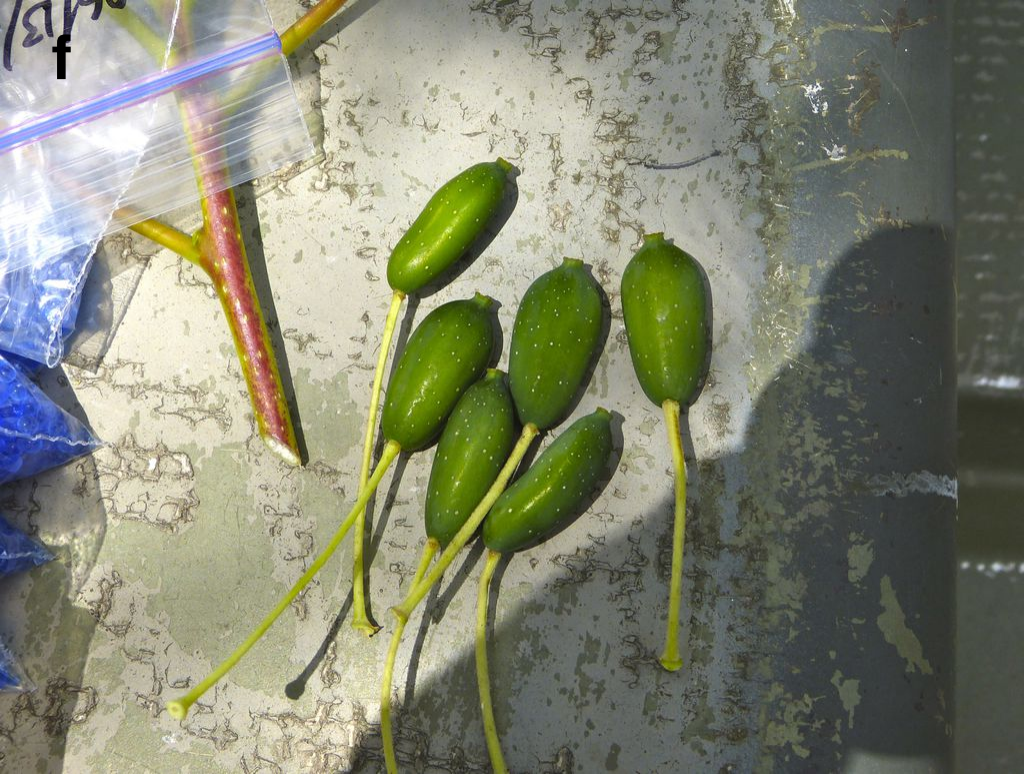
Fruit

**Figure 178a.**
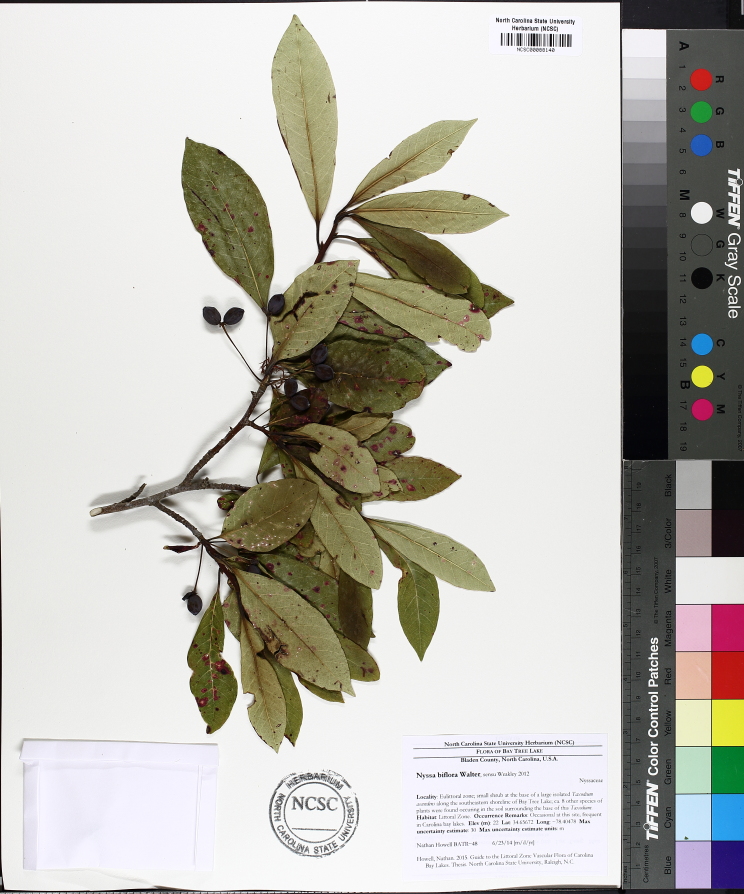
Specimen: *Howell BATR-48* (NCSC)

**Figure 178b.**
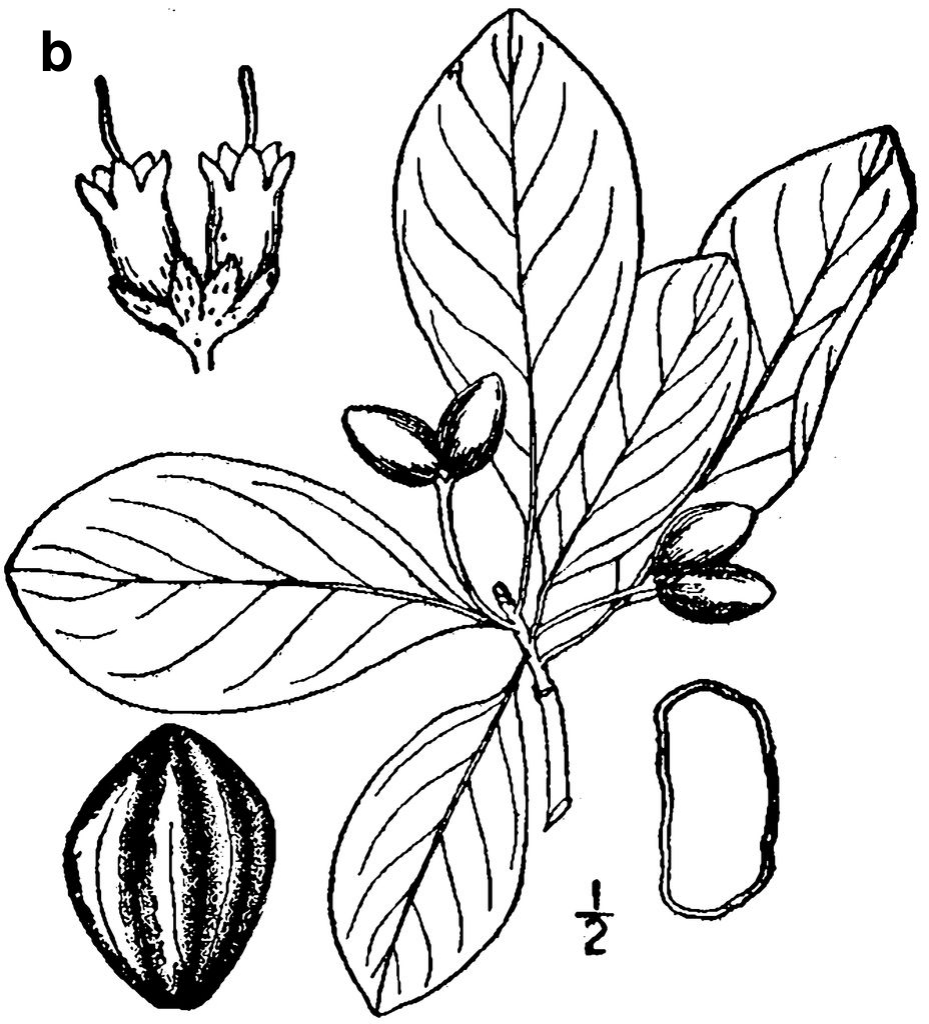
Illustration

**Figure 178c.**
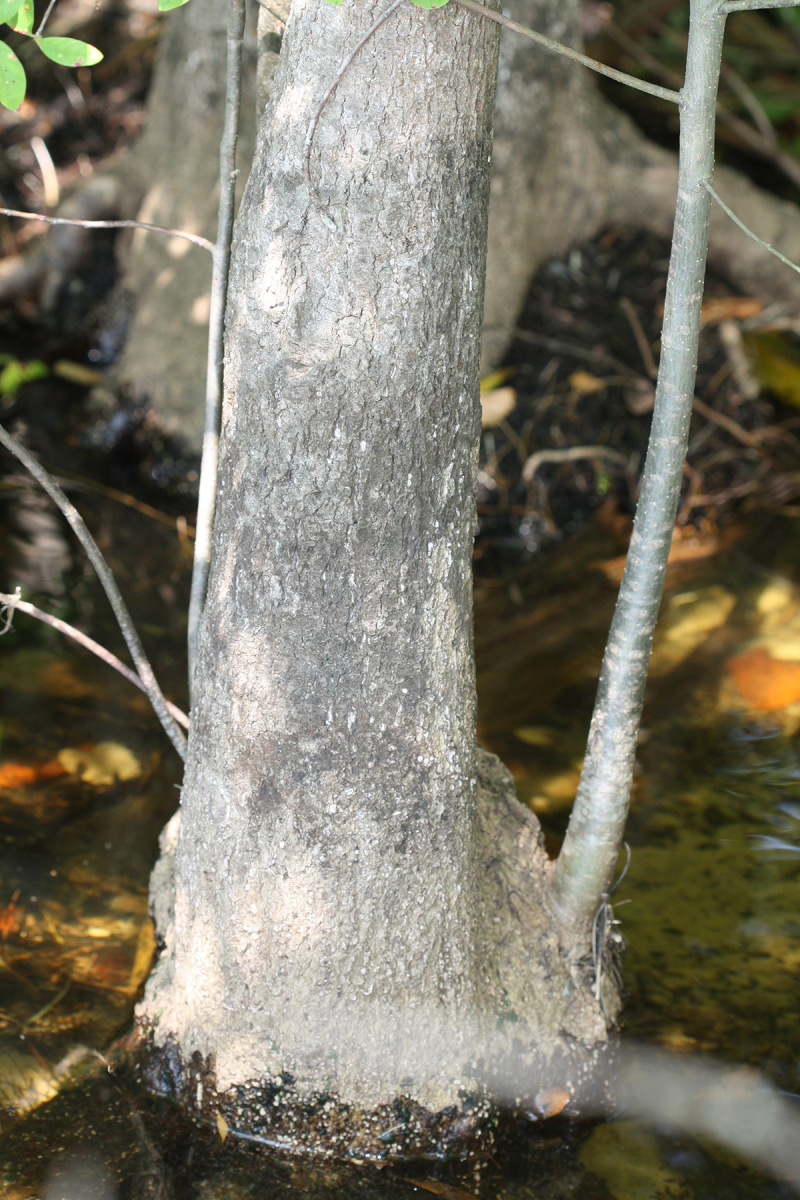
Bark

**Figure 178d.**
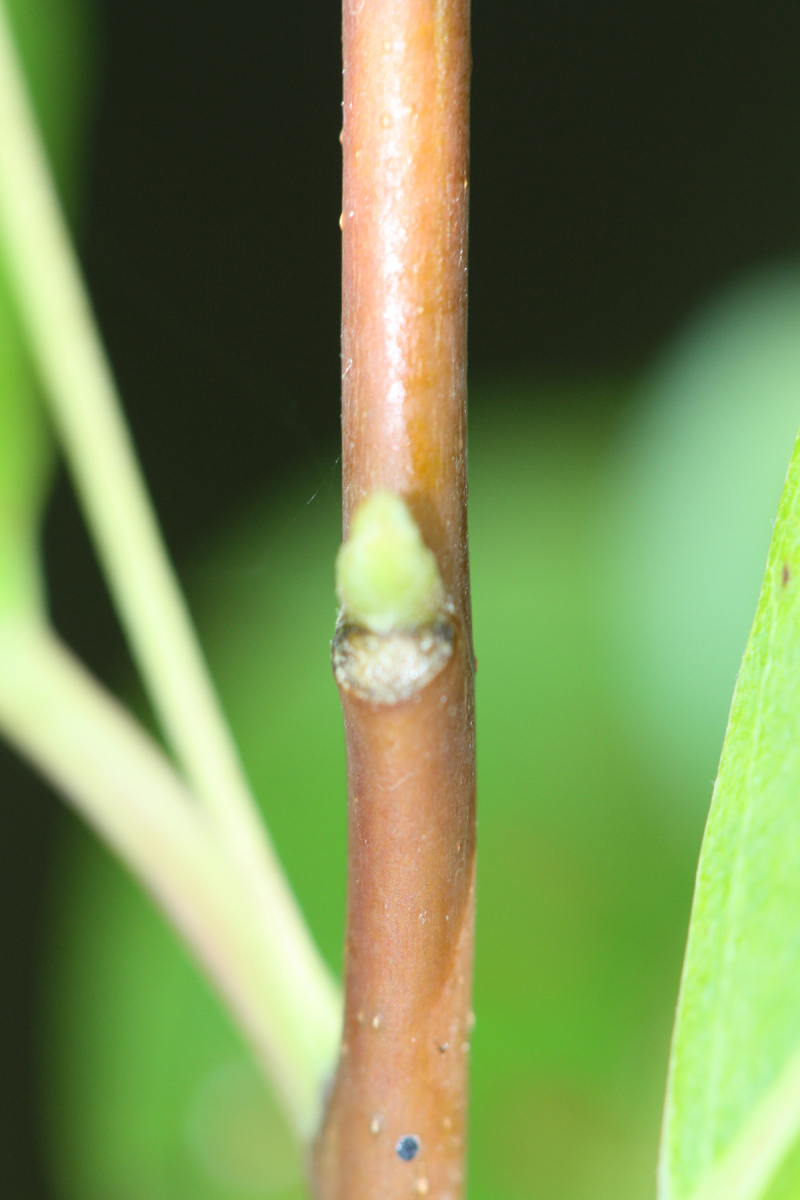
Twig

**Figure 178e.**
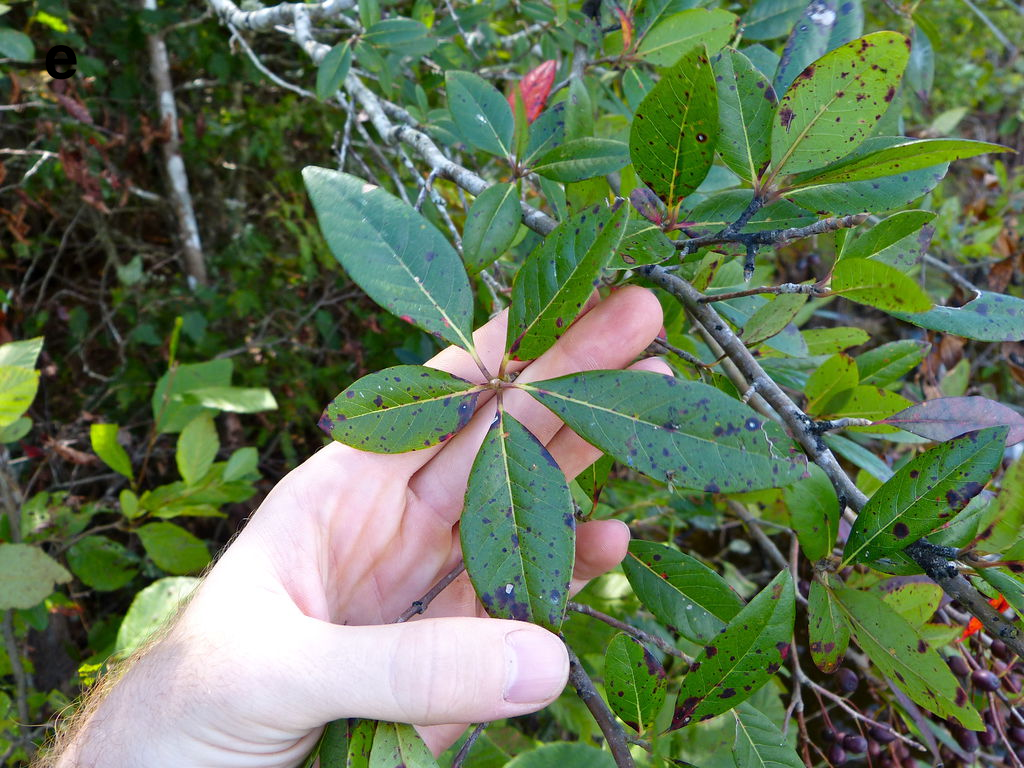
Leaves

**Figure 178f.**
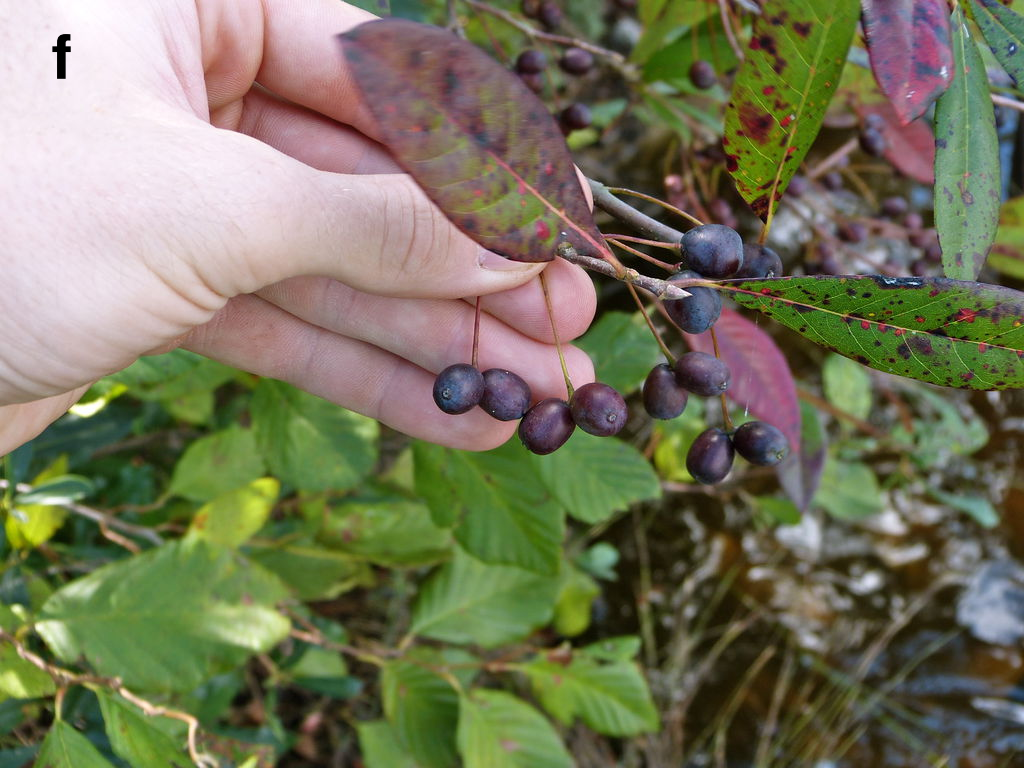
Fruits

**Figure 179a.**
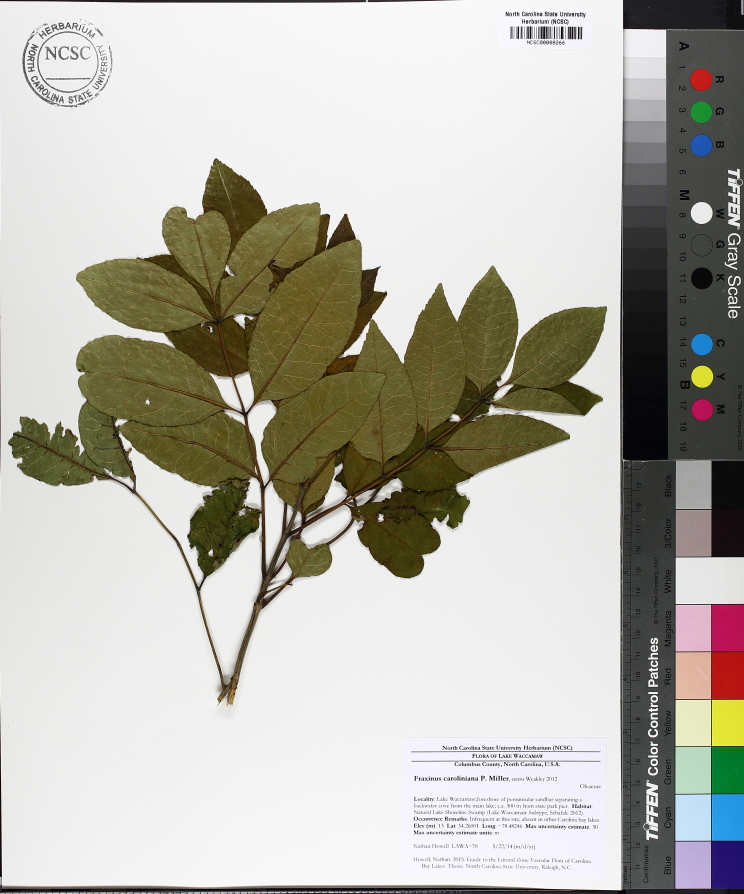
Specimen: *Howell LAWA-70* (NCSC)

**Figure 179b.**
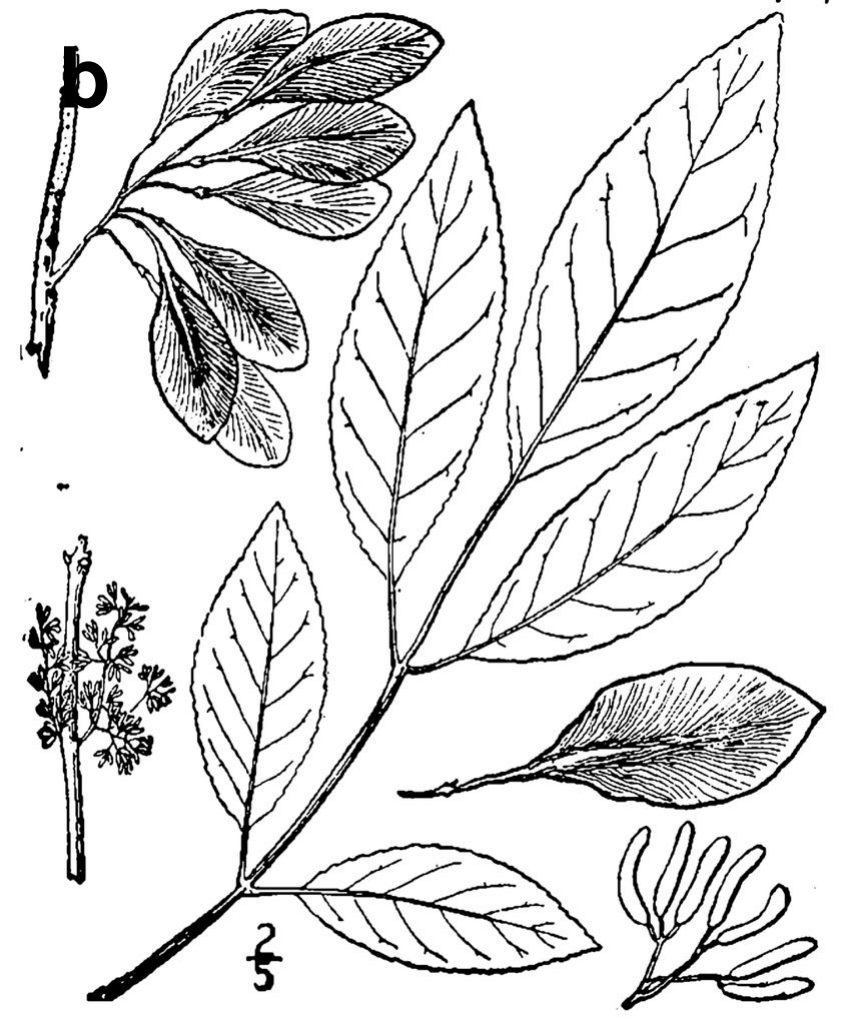
Illustration

**Figure 179c.**
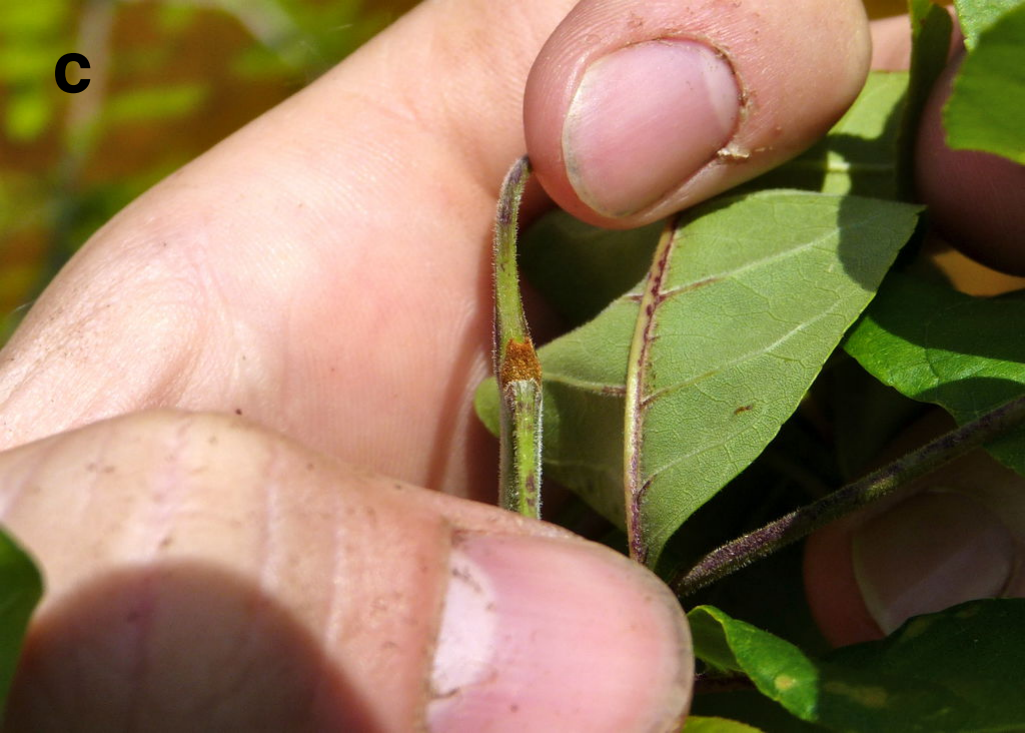
Stem

**Figure 179d.**
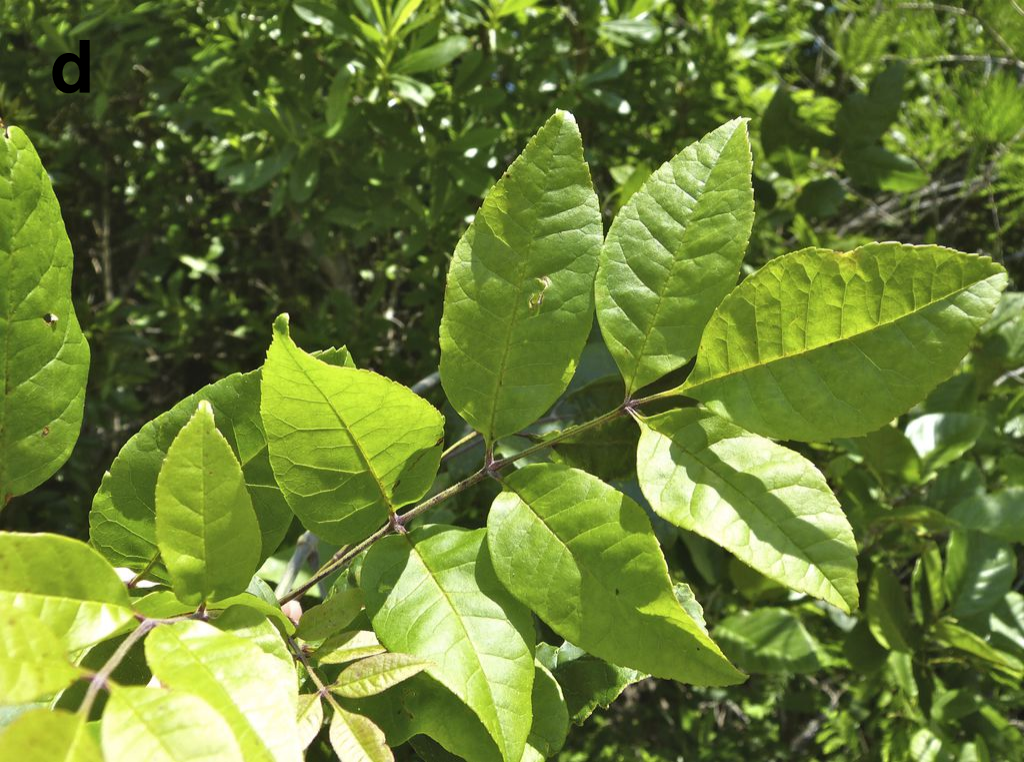
Imparipinnate leaf

**Figure 179e.**
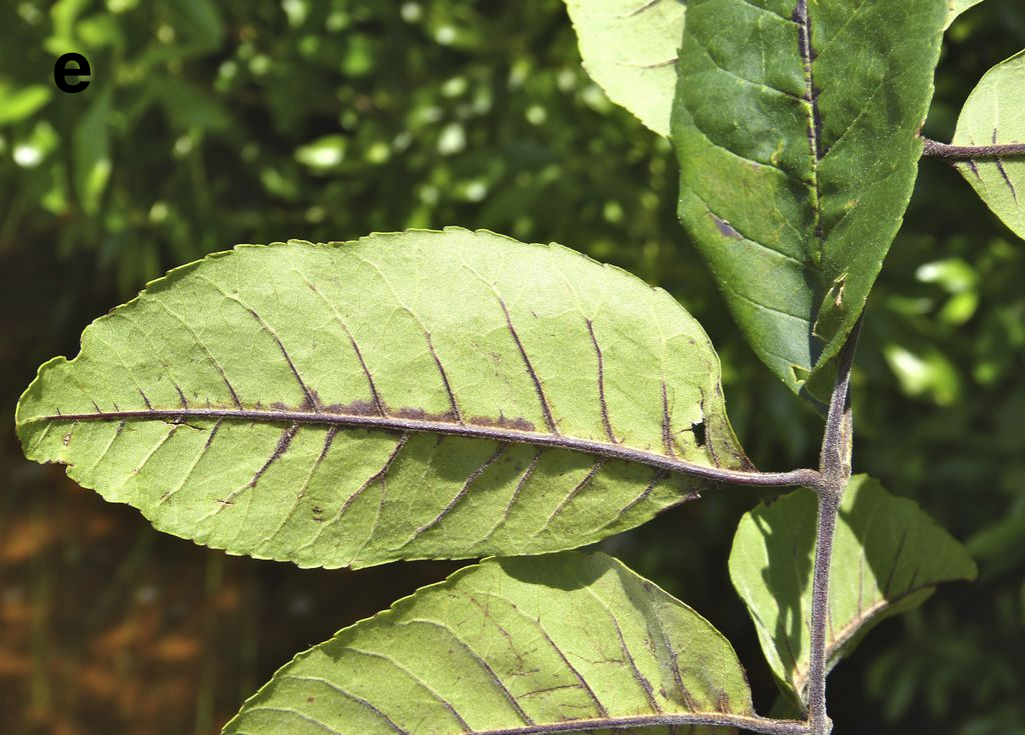
Leaflet

**Figure 179f.**
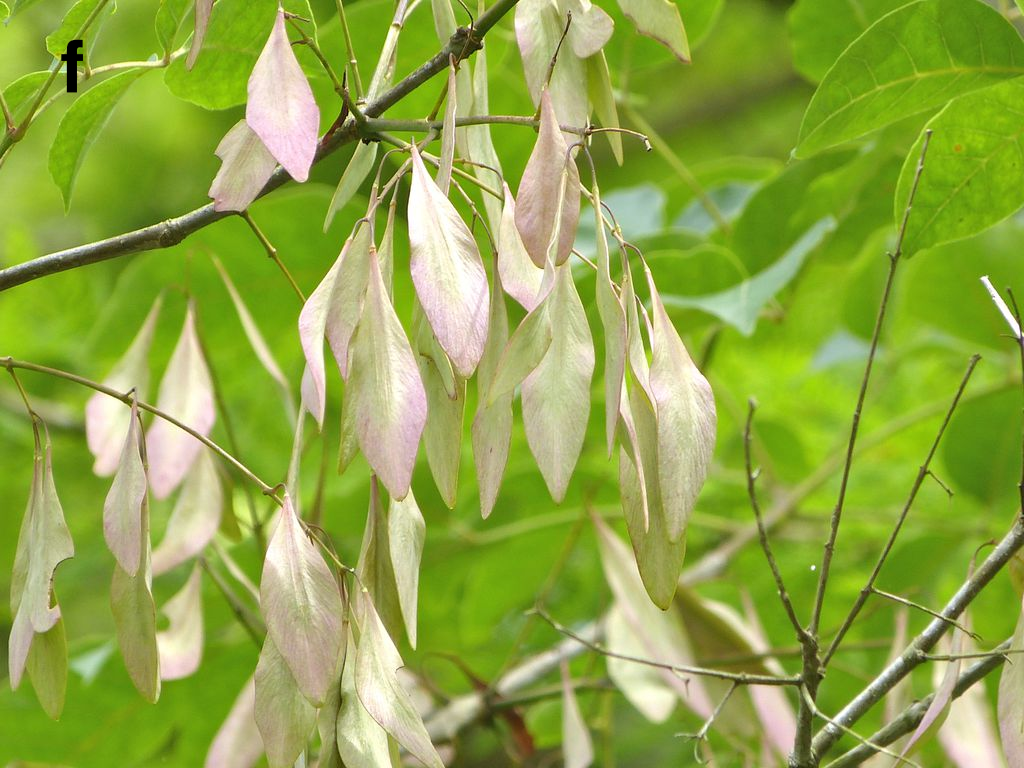
Fruits

**Figure 180a.**
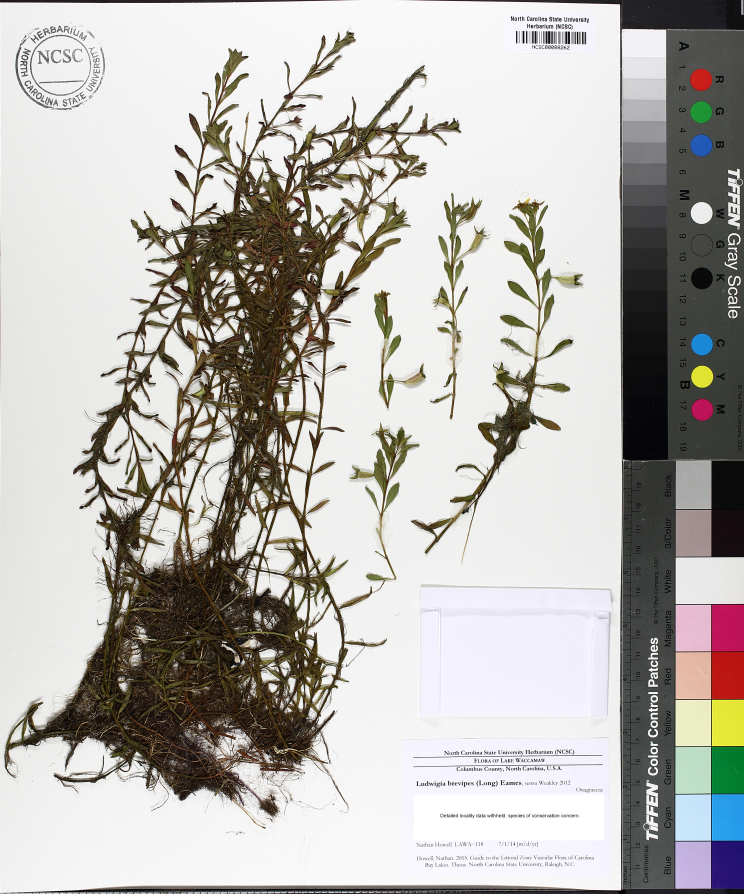
Specimen: *Howell LAWA-118* (NCSC)

**Figure 180b.**
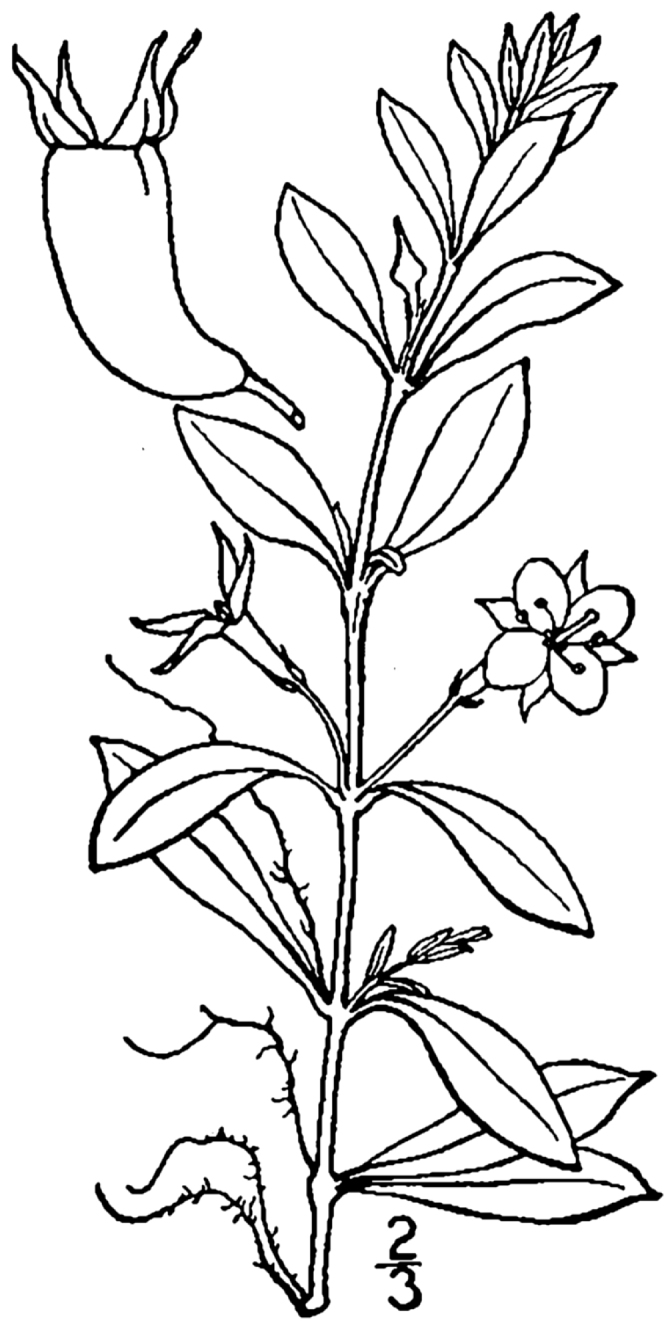
Illustration

**Figure 180c.**
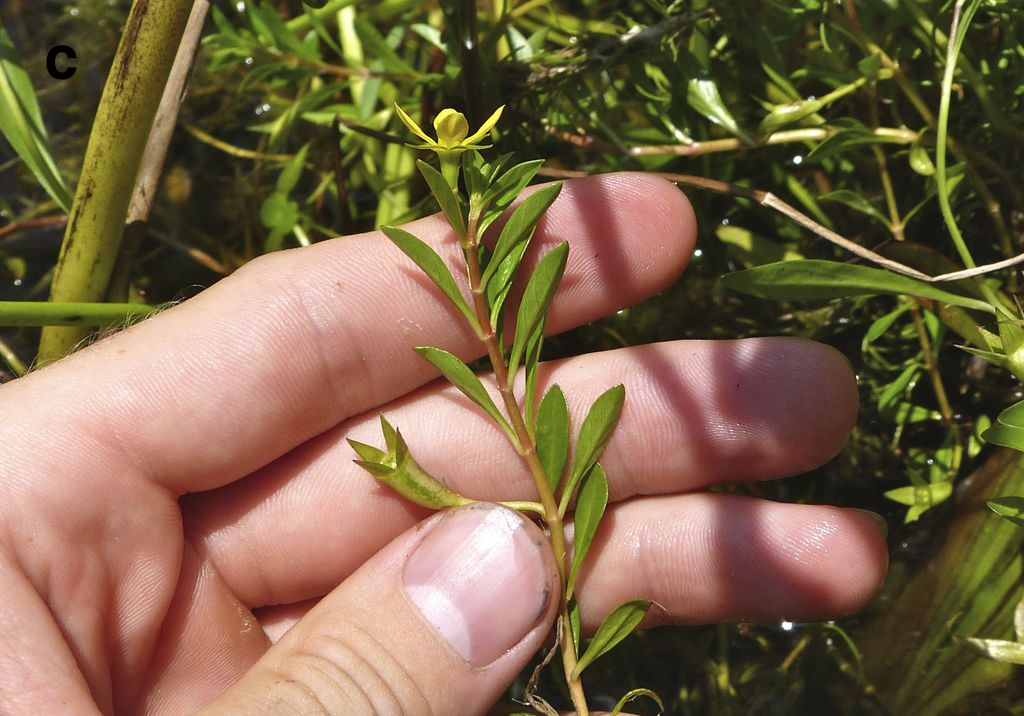
Habit

**Figure 180d.**
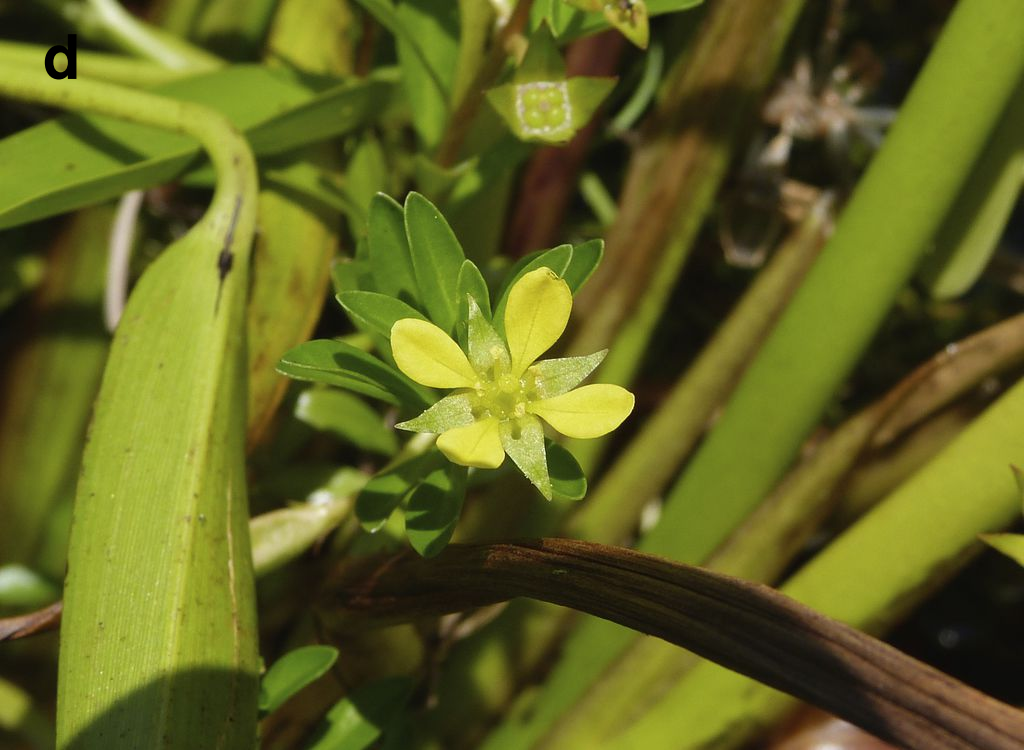
Flower

**Figure 181a.**
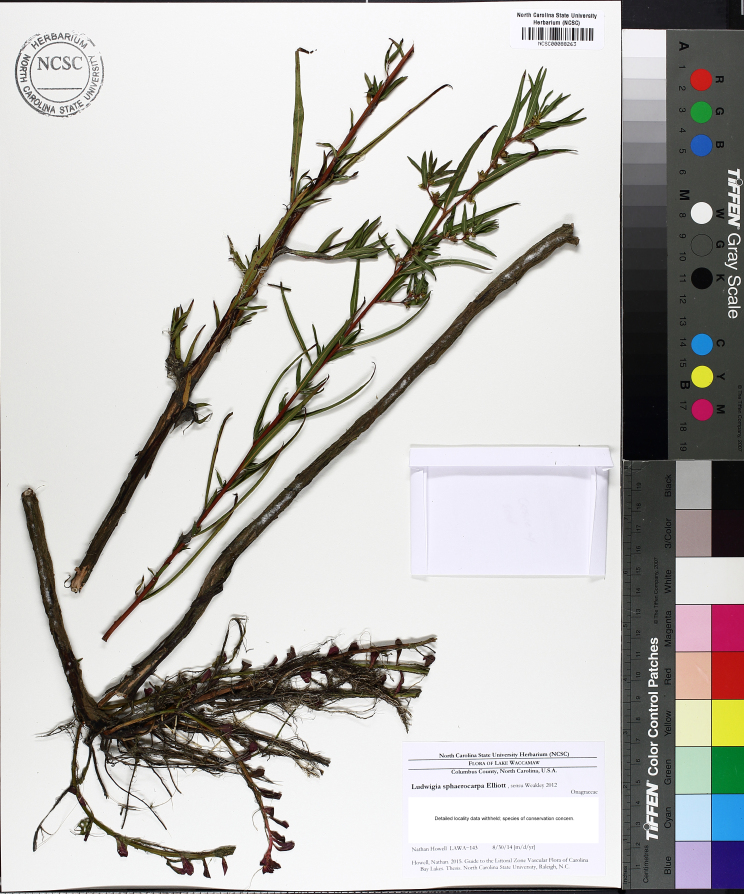
Specimen: *Howell LAWA-143* (NCSC)

**Figure 181b.**
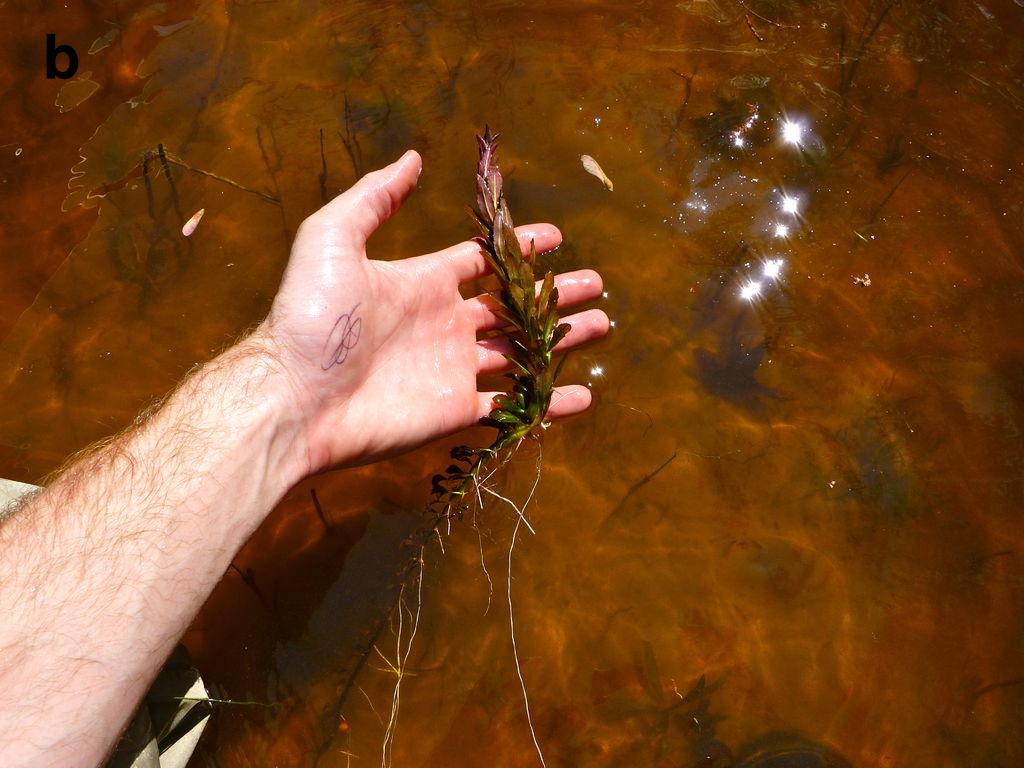
Submerged leaves

**Figure 181c.**
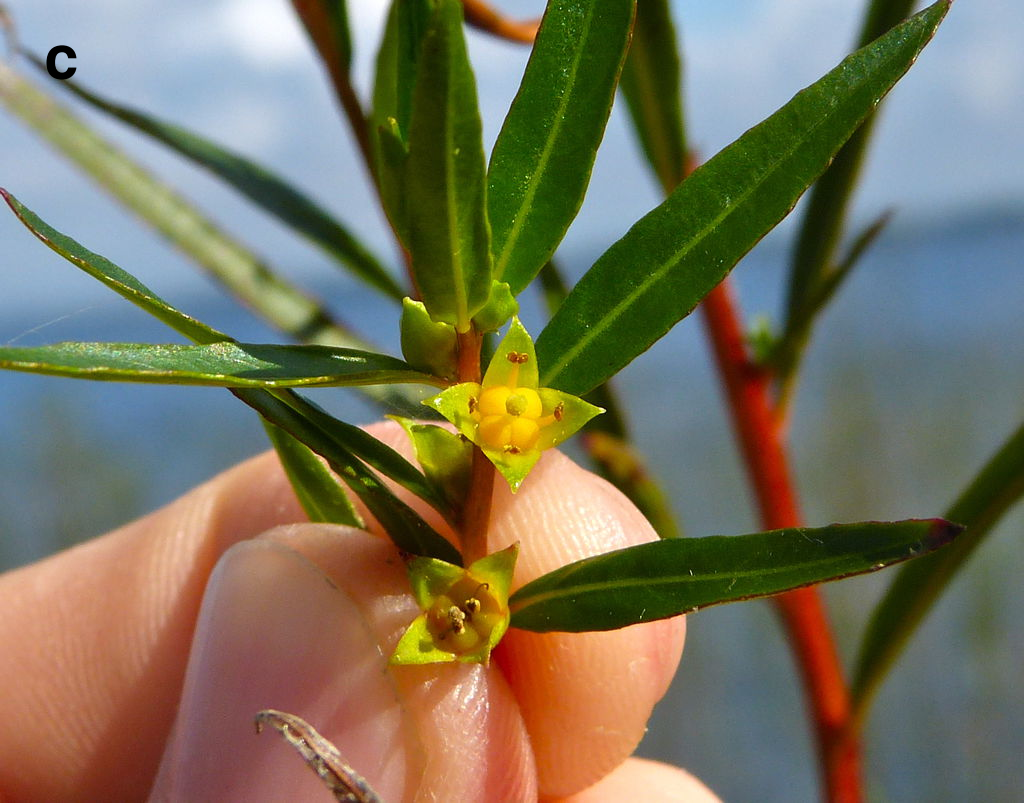
Flowers

**Figure 181d.**
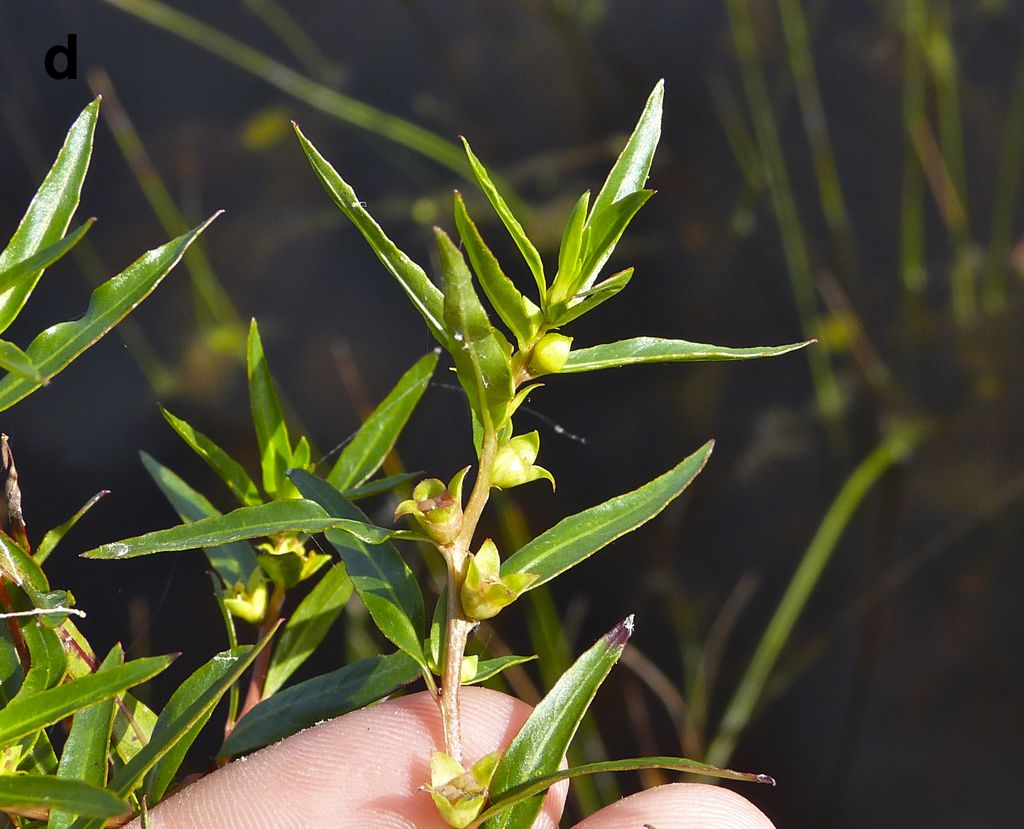
Fruits

**Figure 182a.**
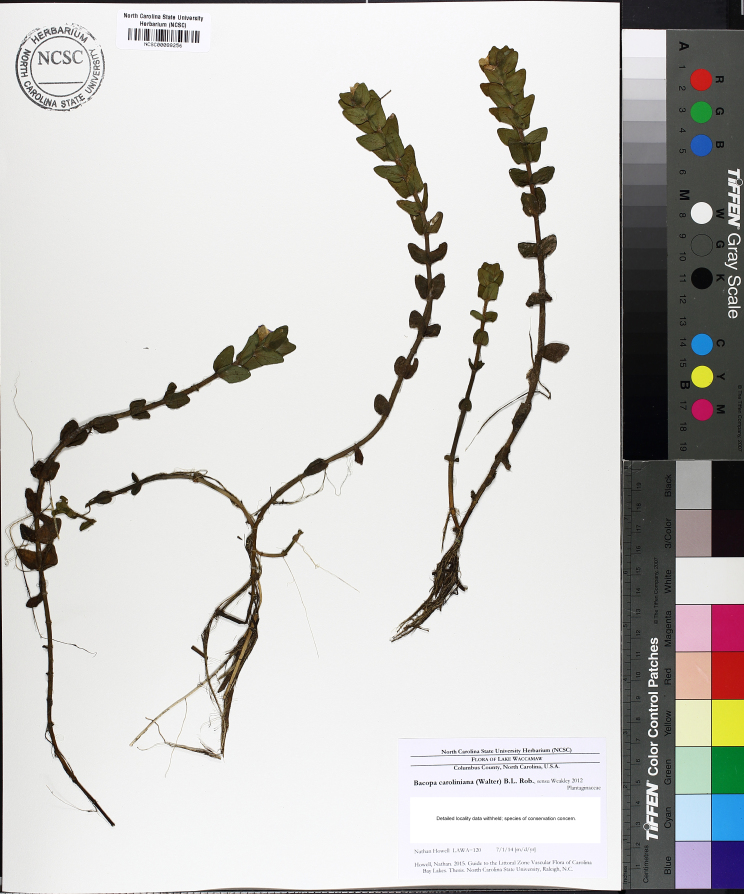
Specimen: *Howell LAWA-120* (NCSC)

**Figure 182b.**
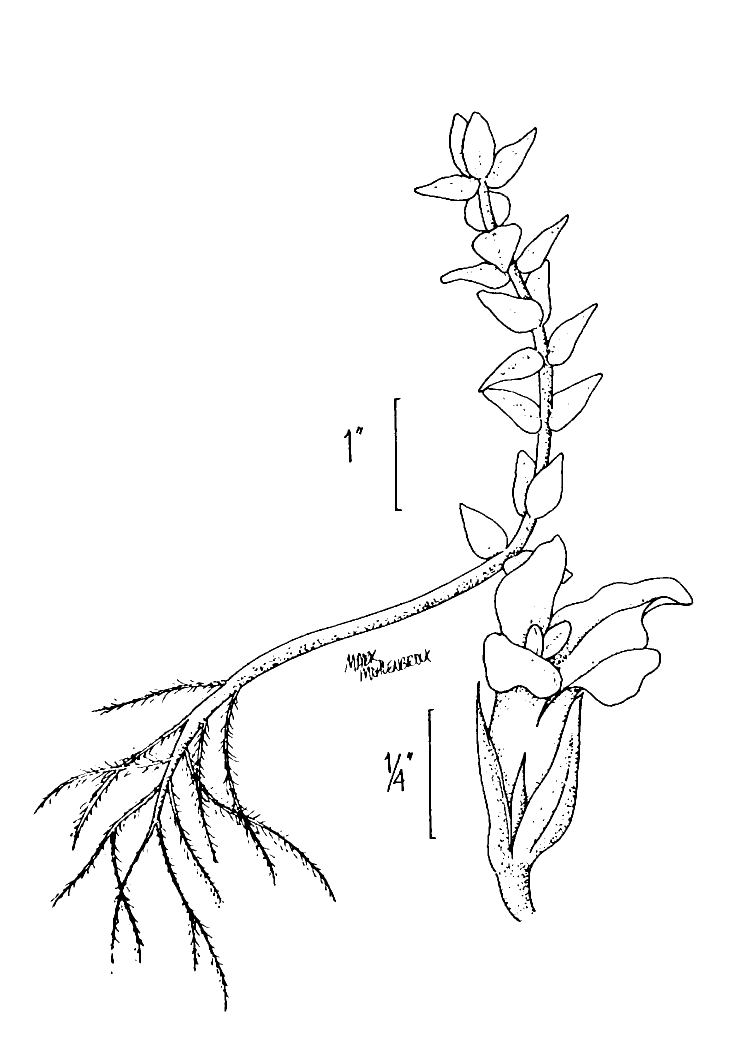
Illustration

**Figure 182c.**
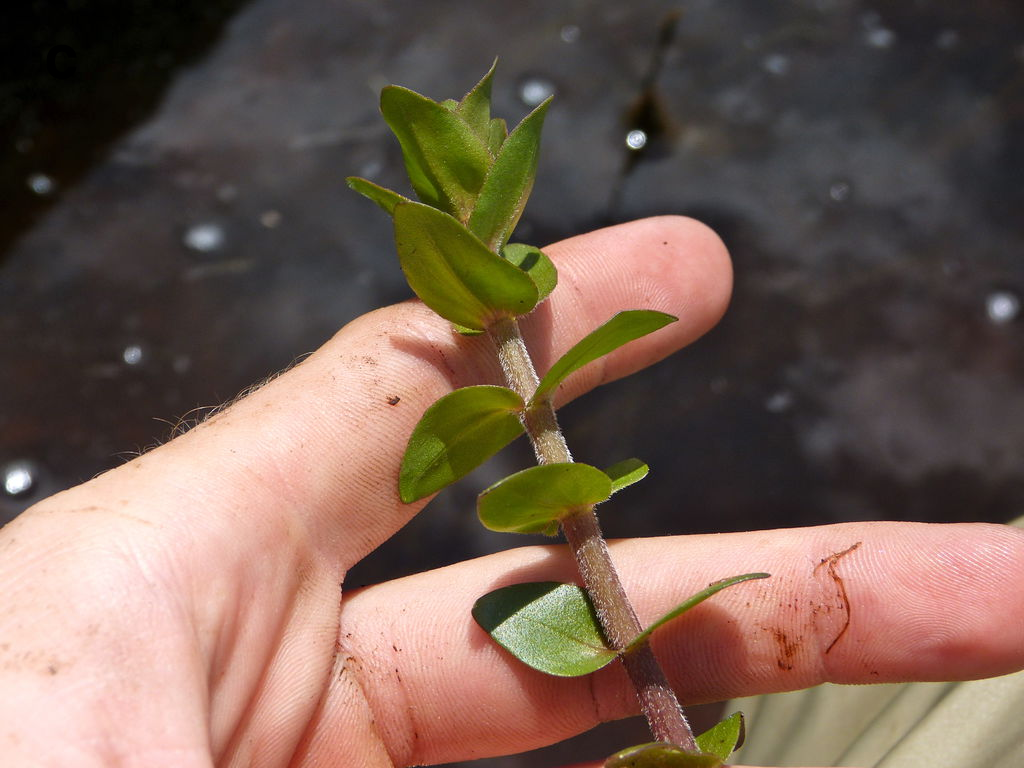
Habit

**Figure 182d.**
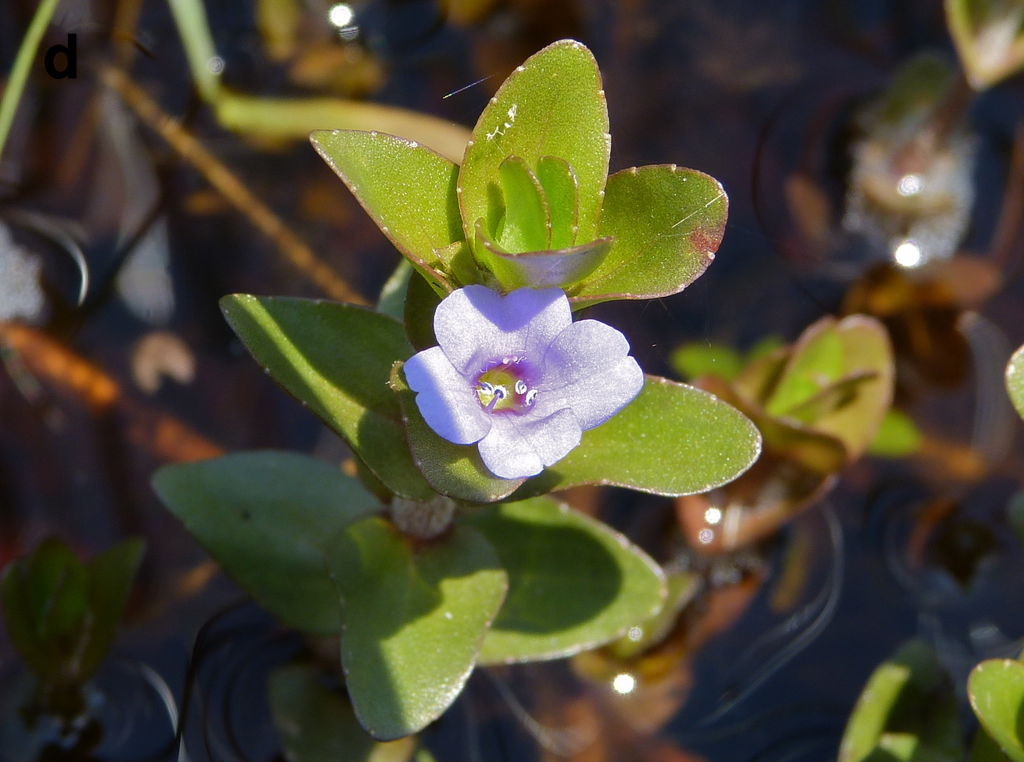
Flower

**Figure 183a.**
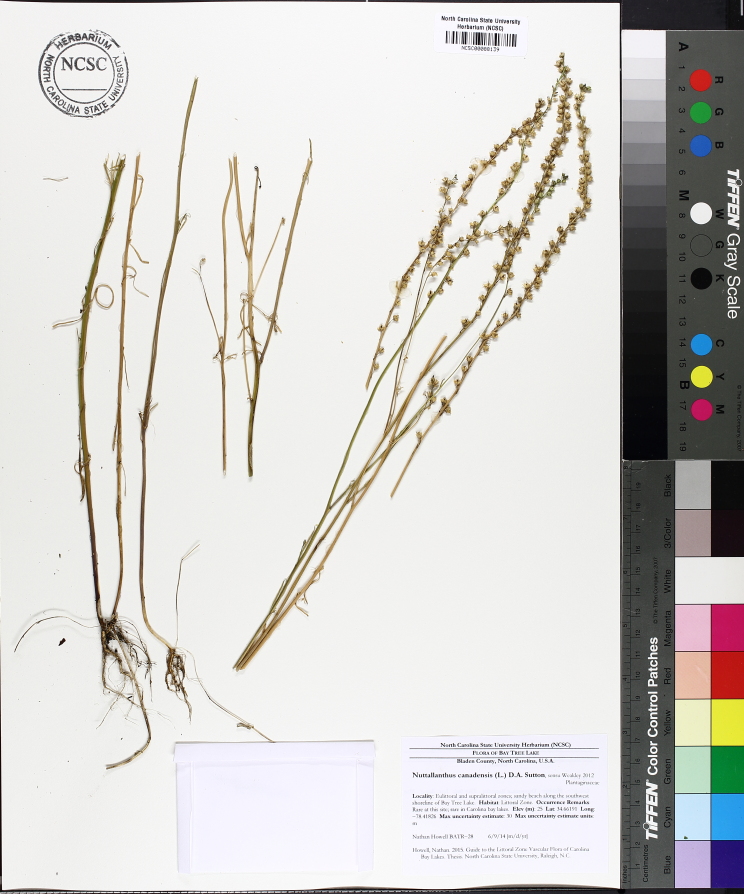
Specimen: *Howell BATR-28* (NCSC)

**Figure 183b.**
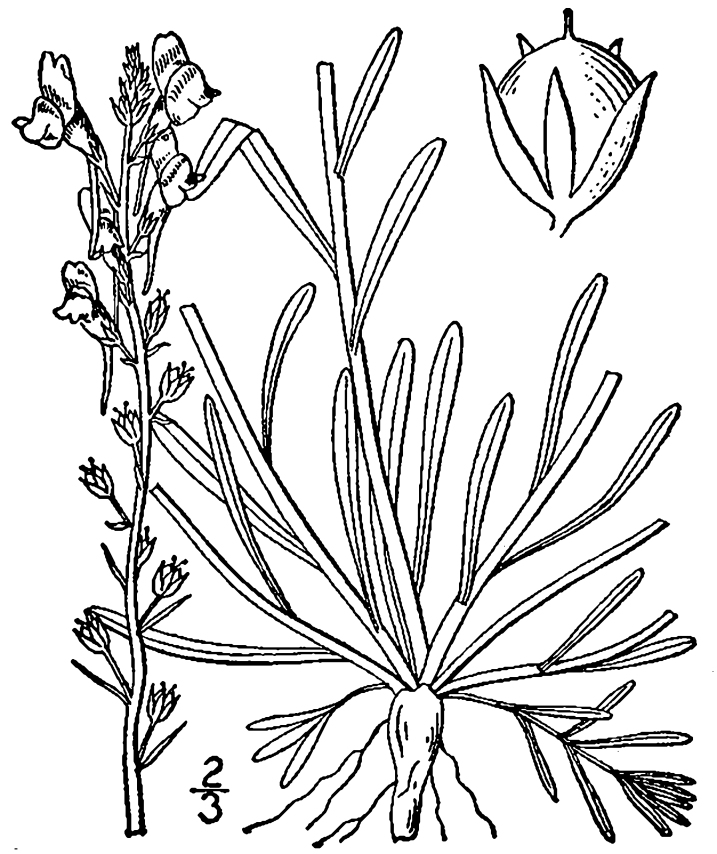
Illustration

**Figure 183c.**
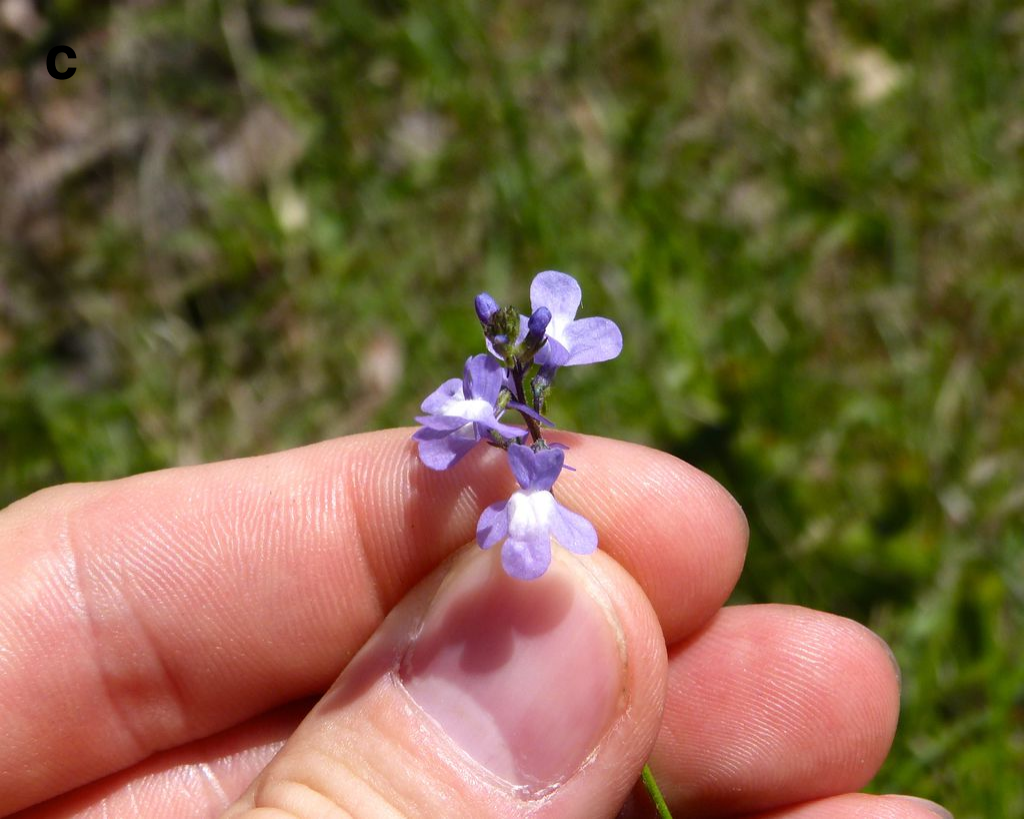
Inflorescence

**Figure 183d.**
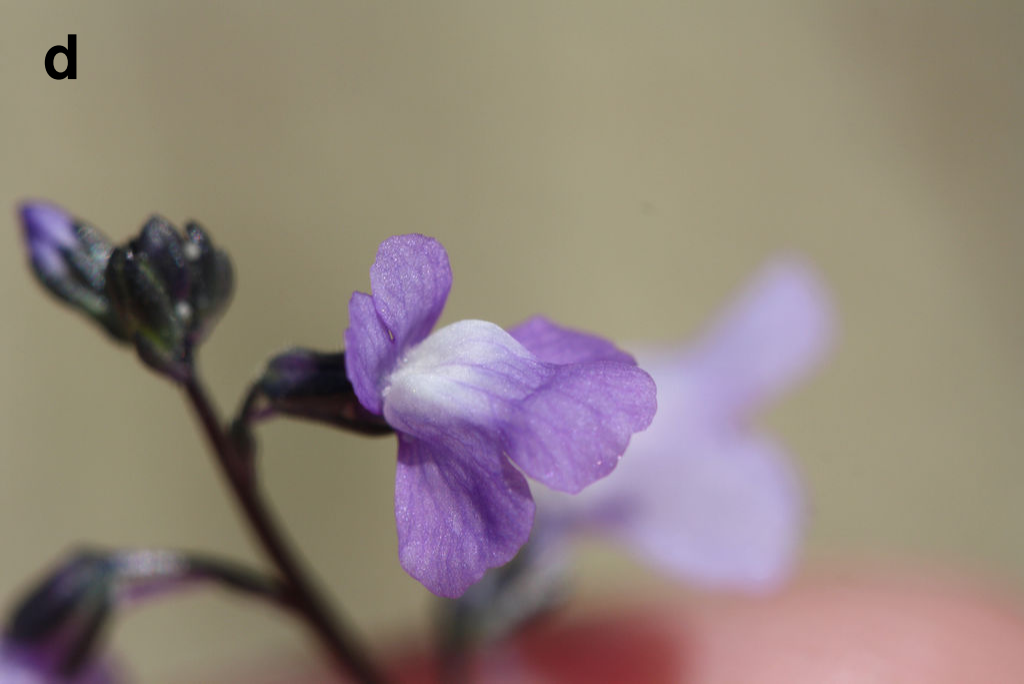
Flower

**Figure 184a.**
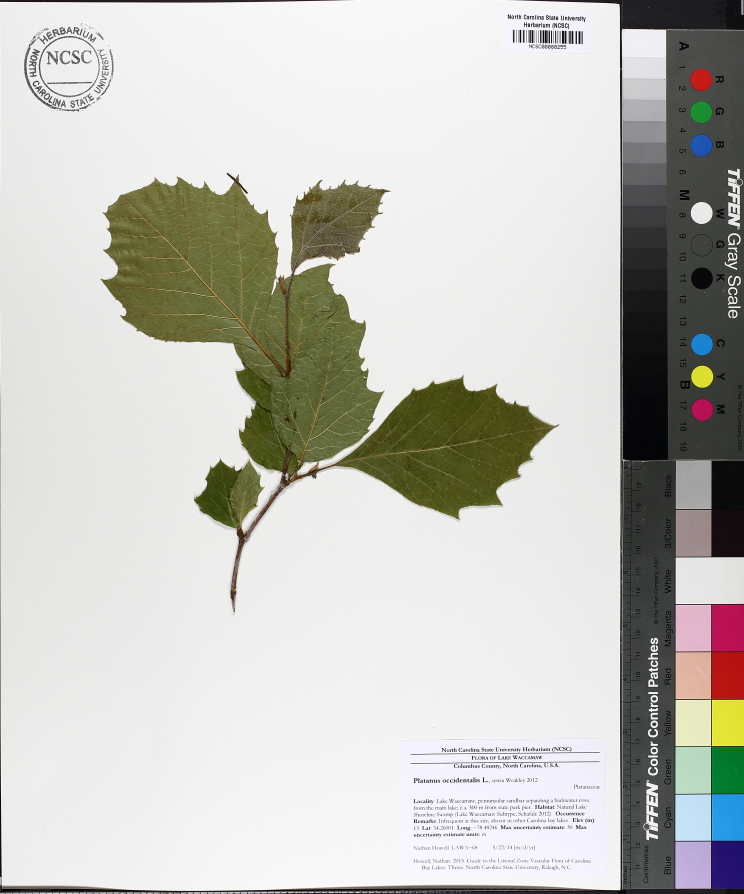
Specimen: *Howell LAWA-68* (NCSC)

**Figure 184b.**
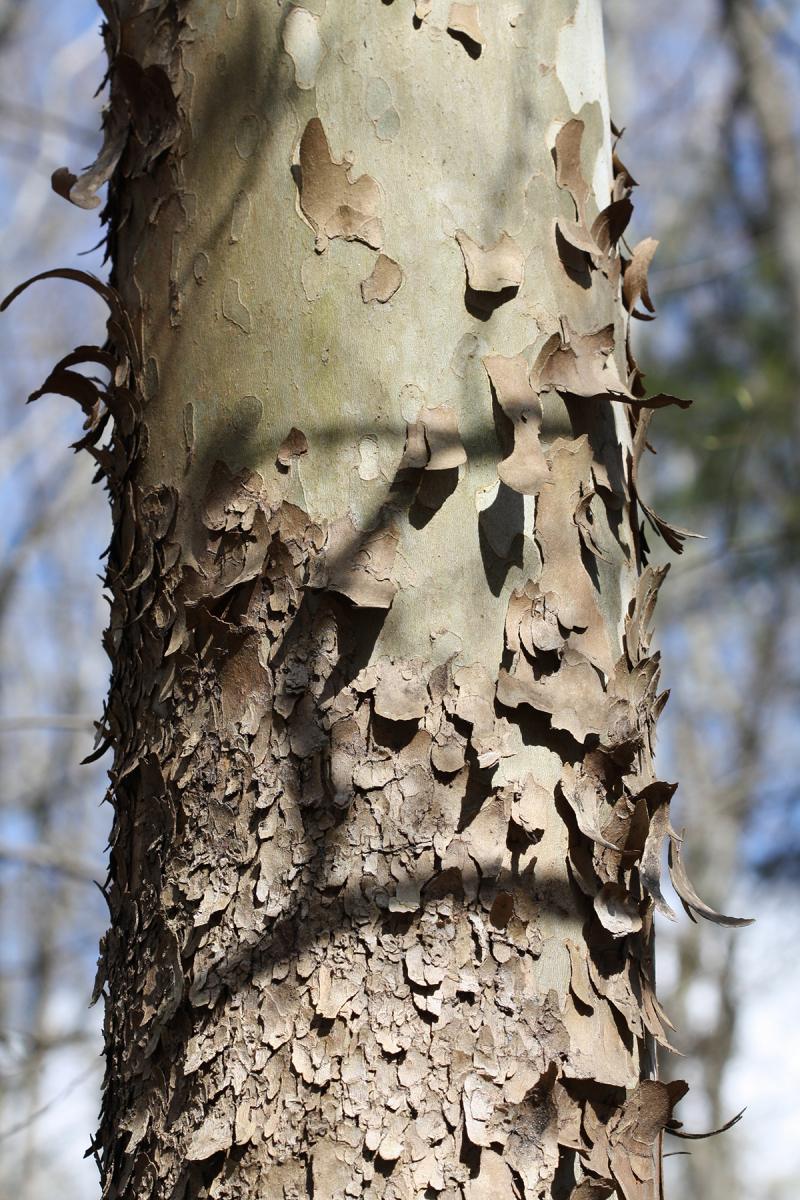
Bark

**Figure 184c.**
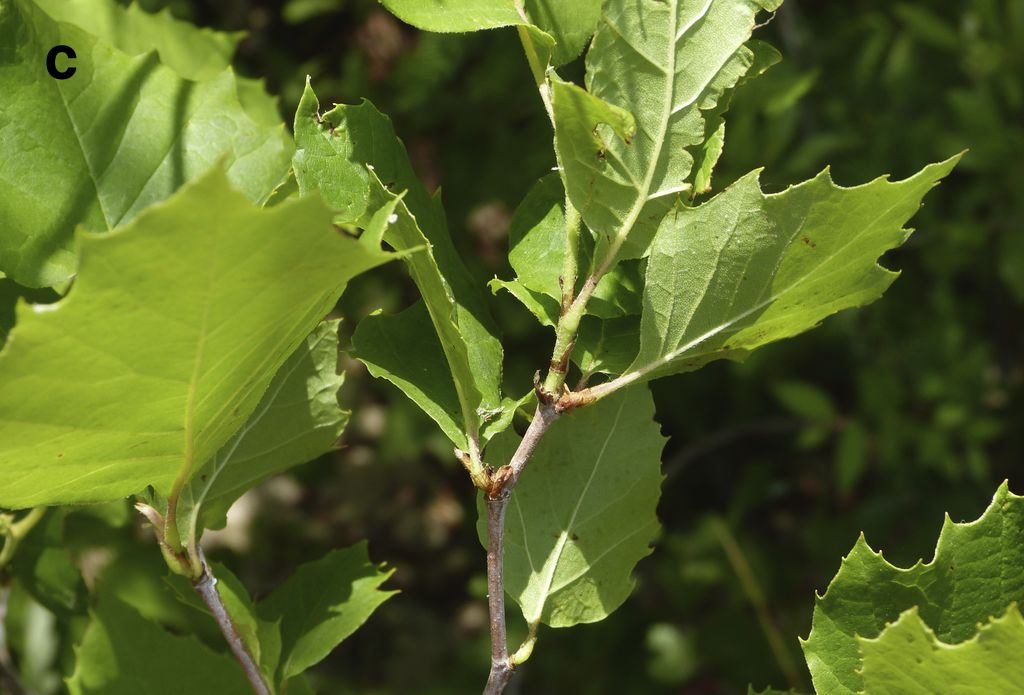
Twigs and leaves

**Figure 184d.**
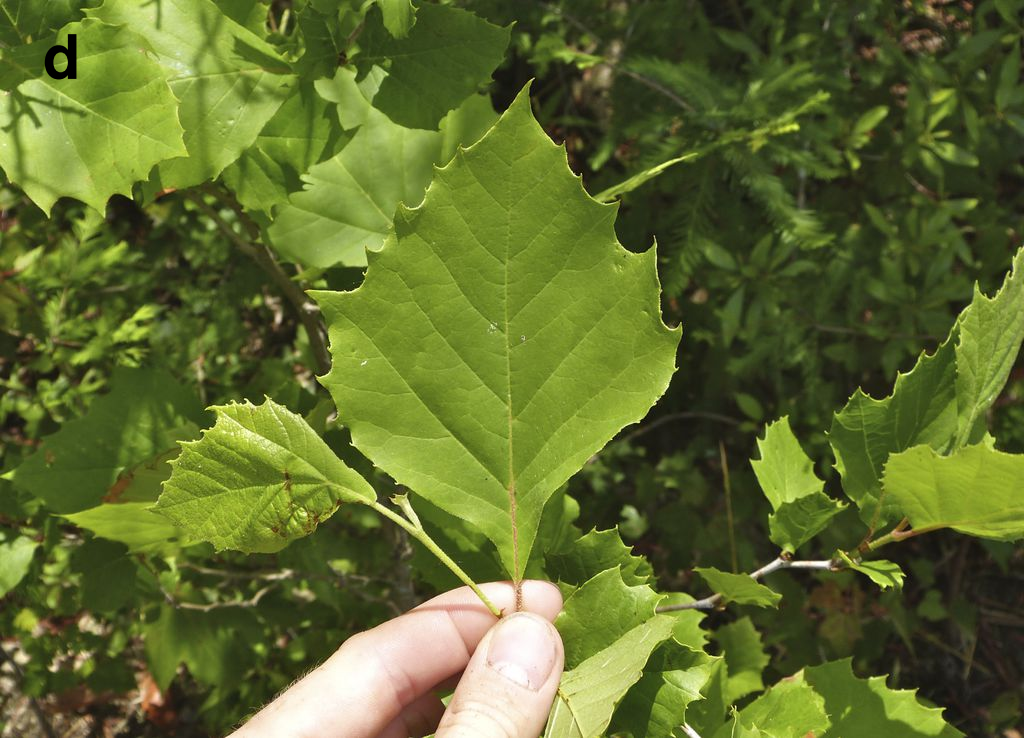
Leaves

**Figure 185a.**
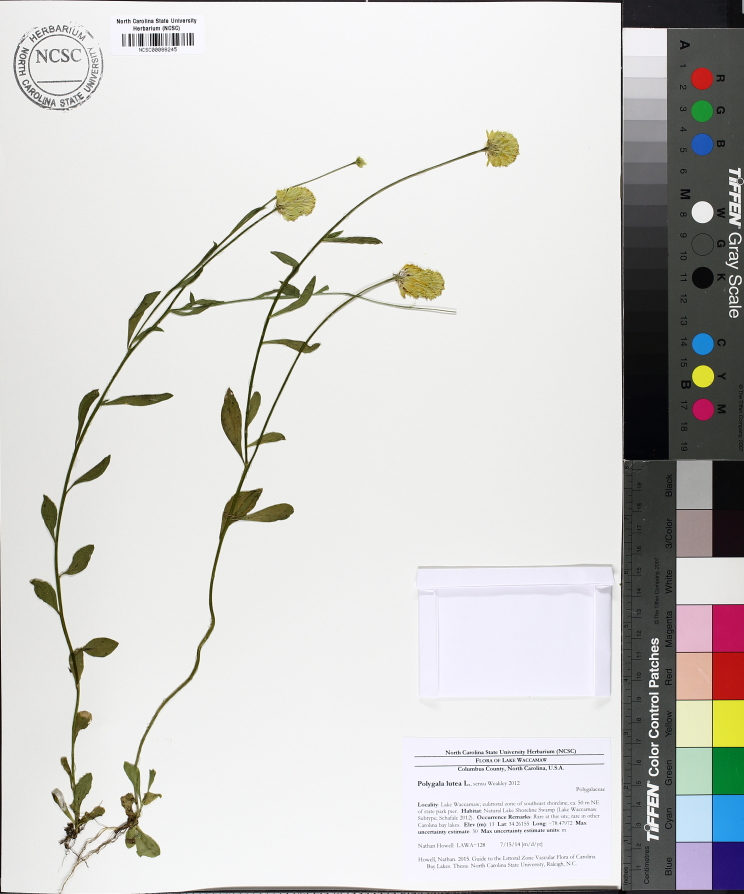
Specimen: *Howell LAWA-128* (NCSC)

**Figure 185b.**
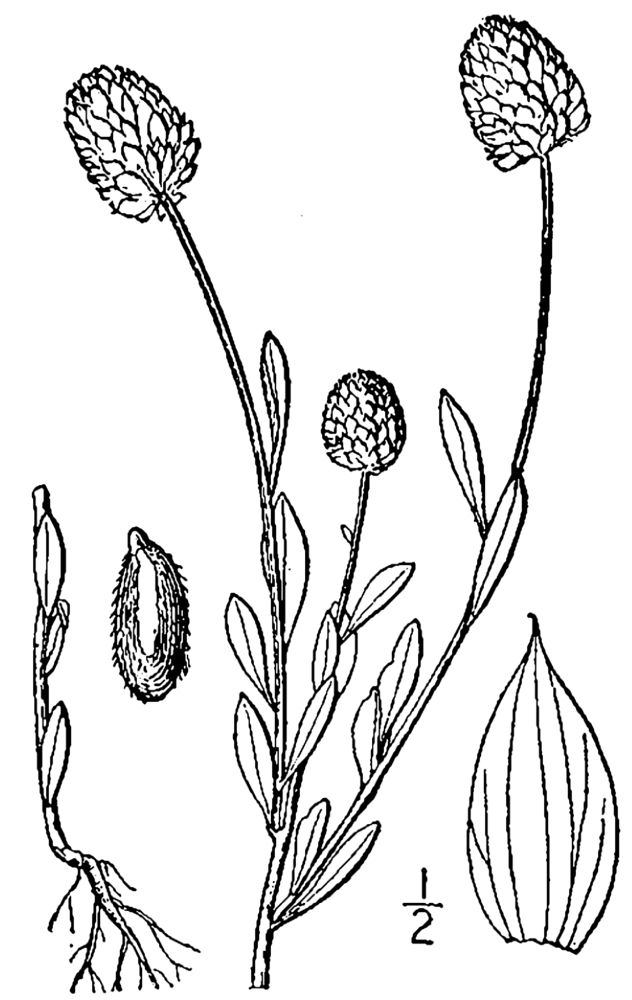
Illustration

**Figure 185c.**
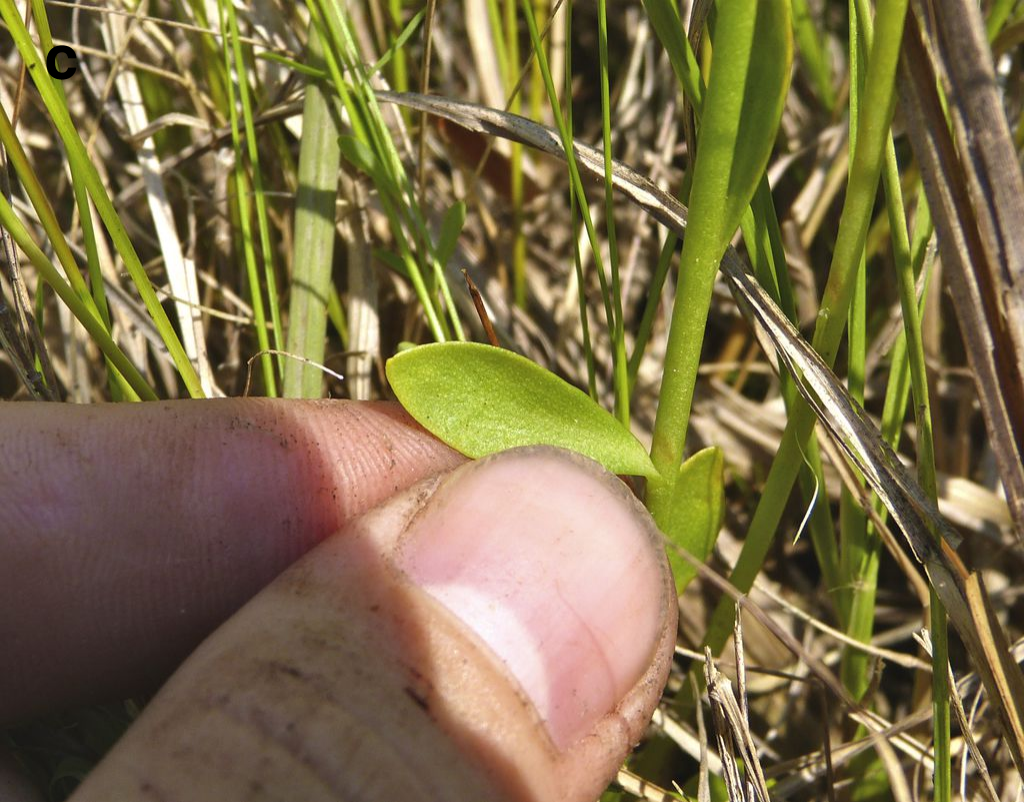
Basal leaf

**Figure 185d.**
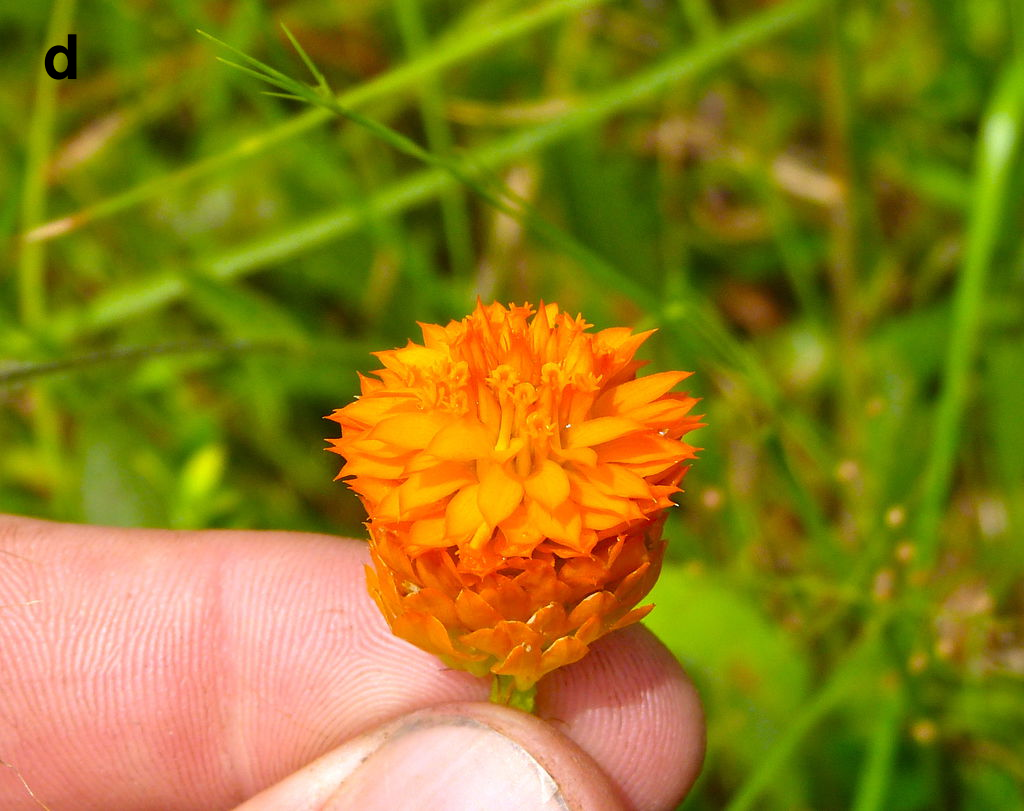
Inflorescence

**Figure 186a.**
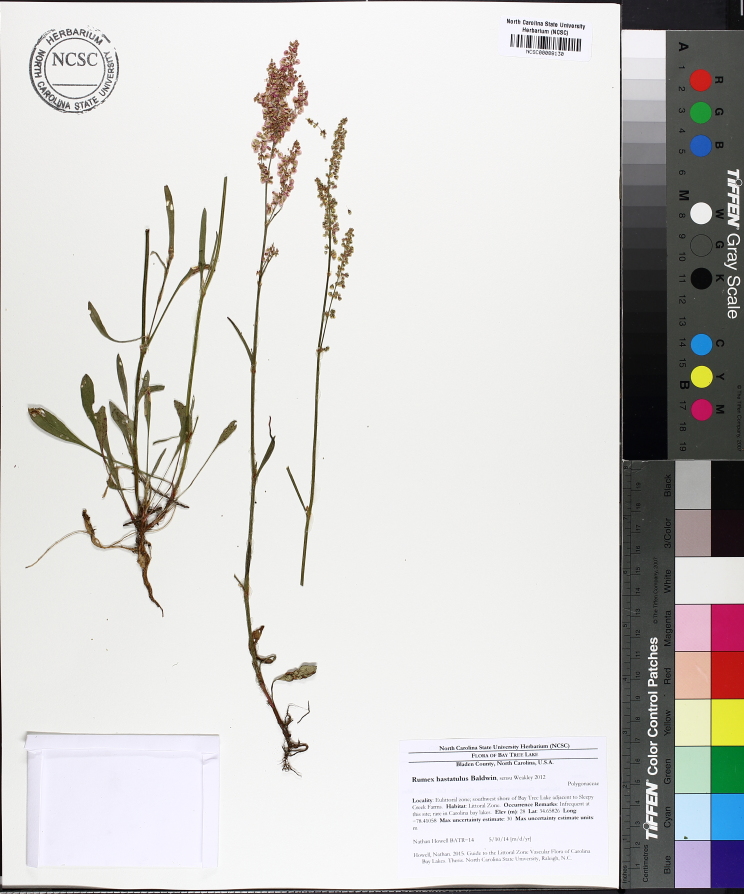
Specimen: *Howell BATR-14* (NCSC)

**Figure 186b.**
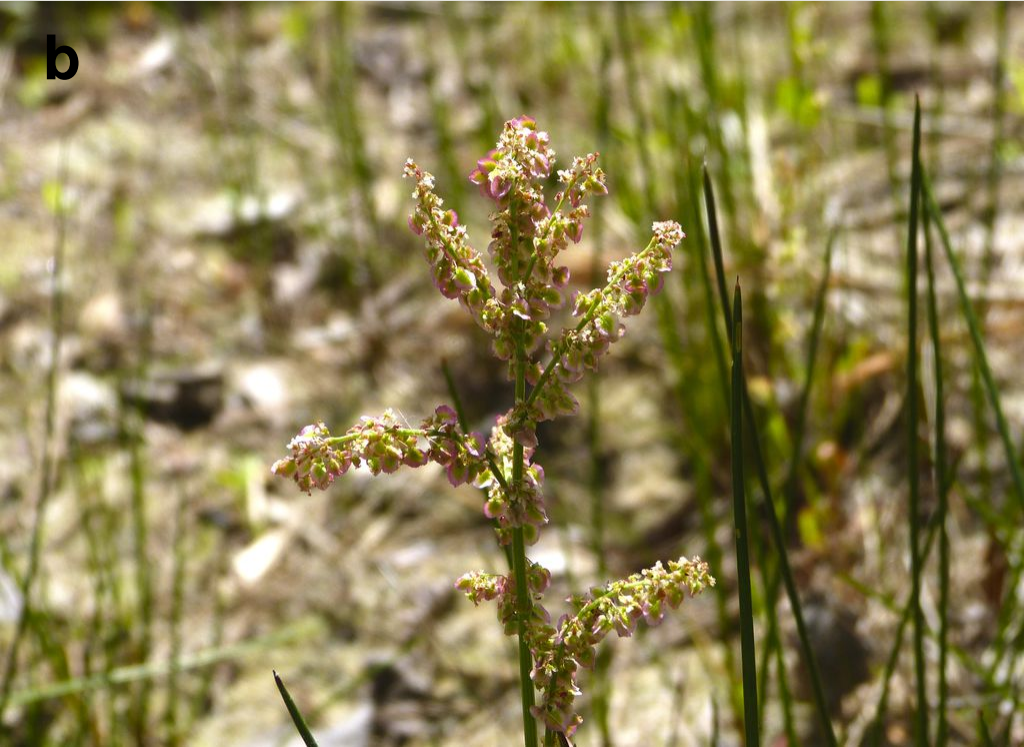
Infructescence

**Figure 187a.**
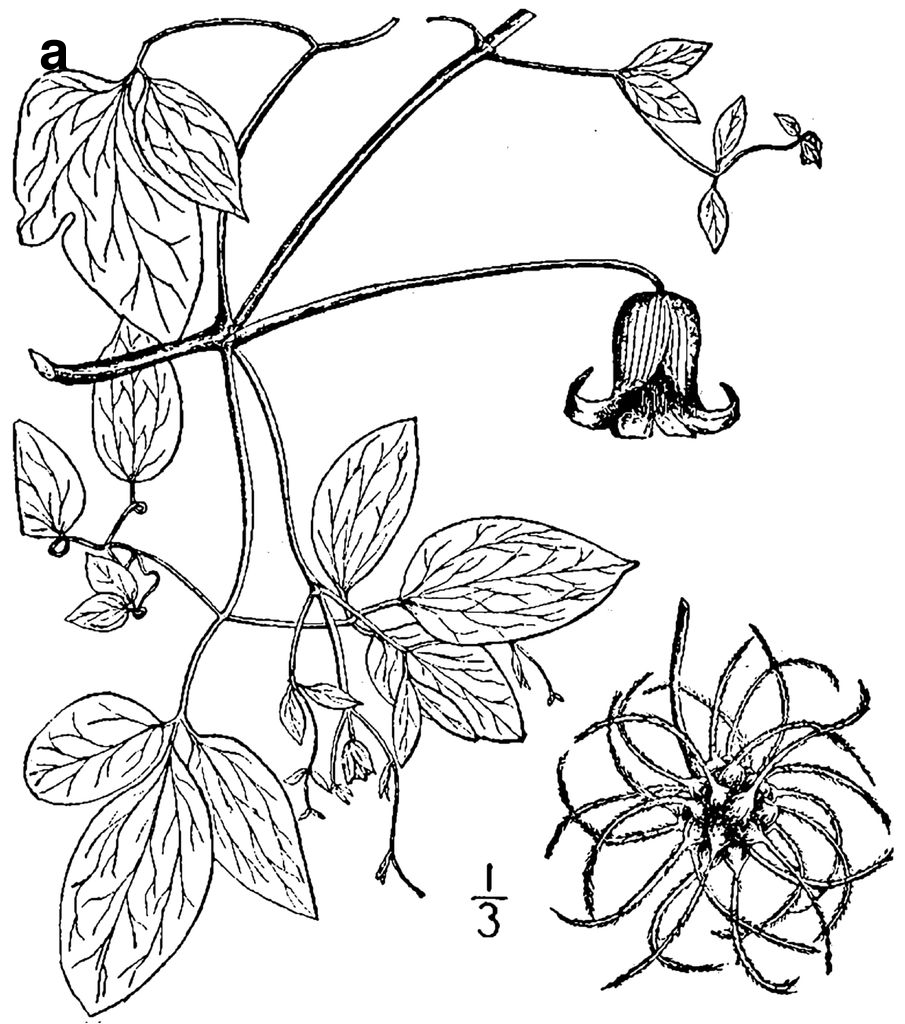
Illustration

**Figure 187b.**
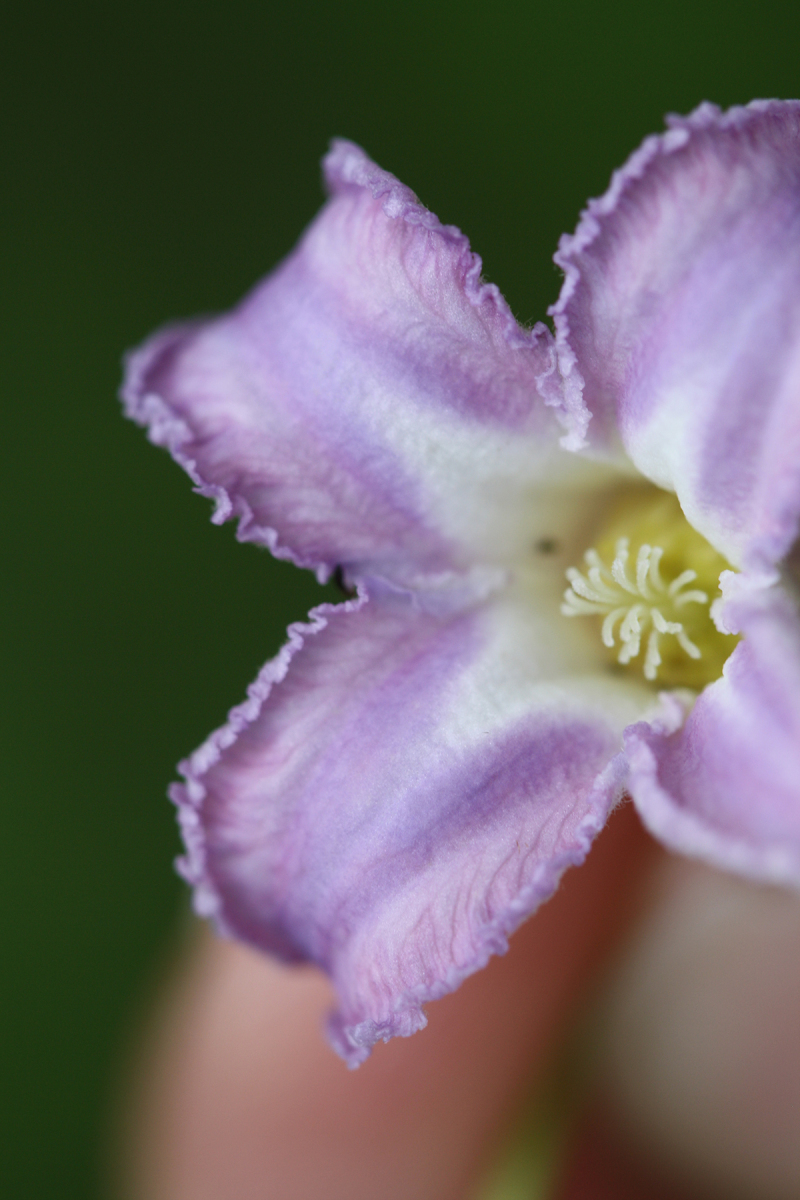
Flower

**Figure 188a.**
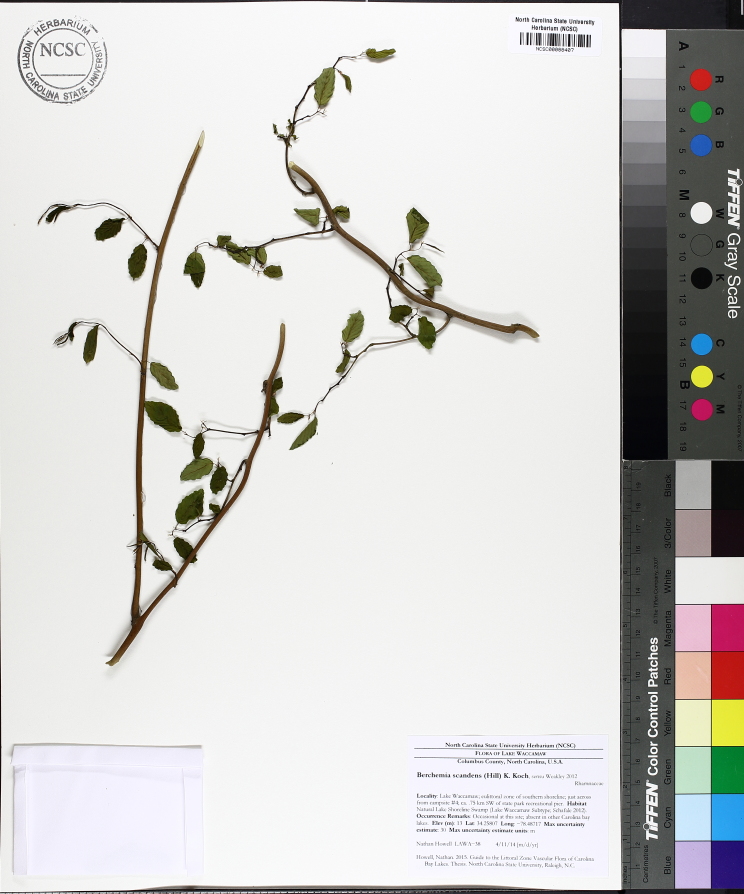
Specimen: *Howell LAWA-38* (NCSC)

**Figure 188b.**
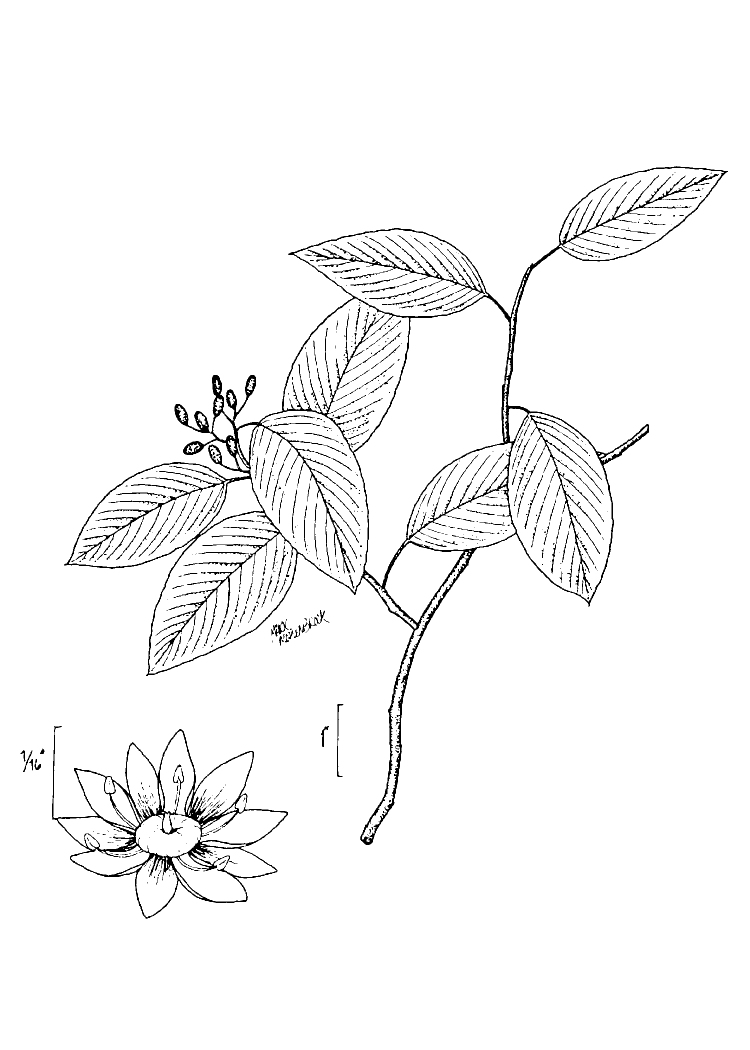
Illustration

**Figure 188c.**
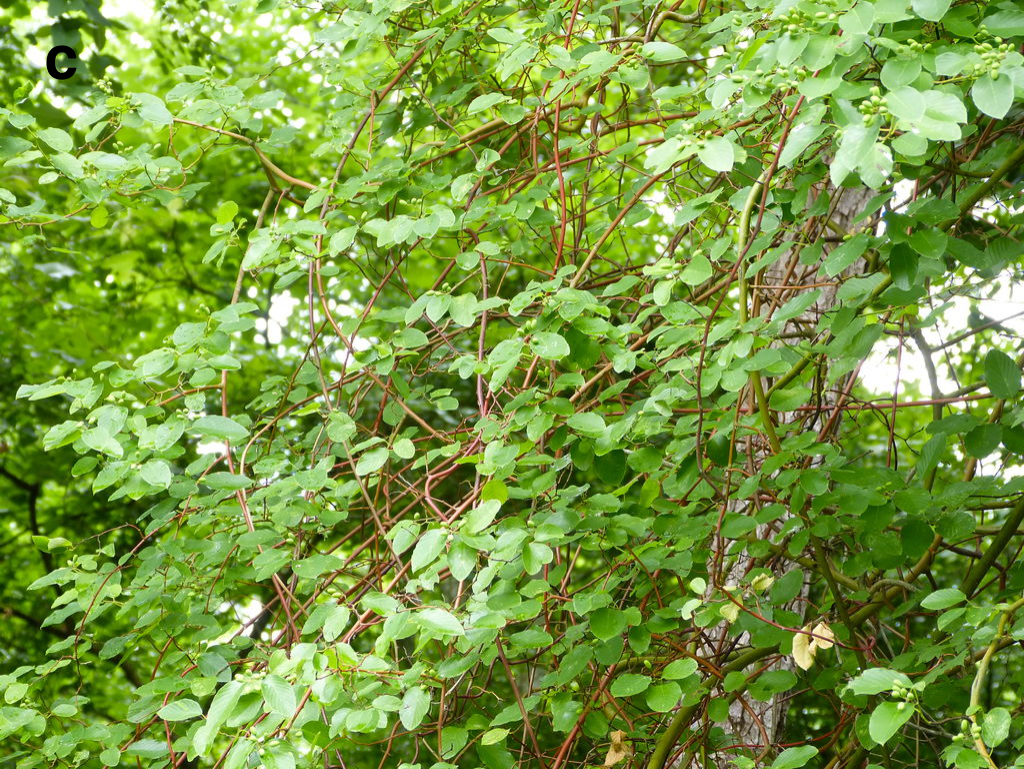
Habit

**Figure 188d.**
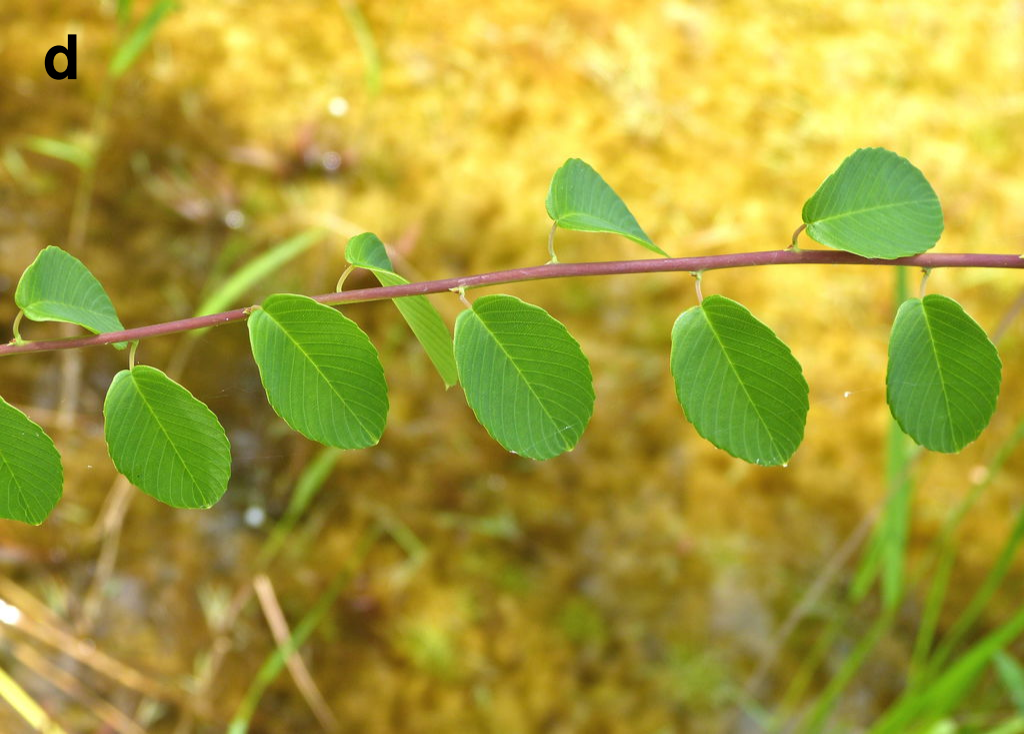
Stem and leaves

**Figure 190a.**
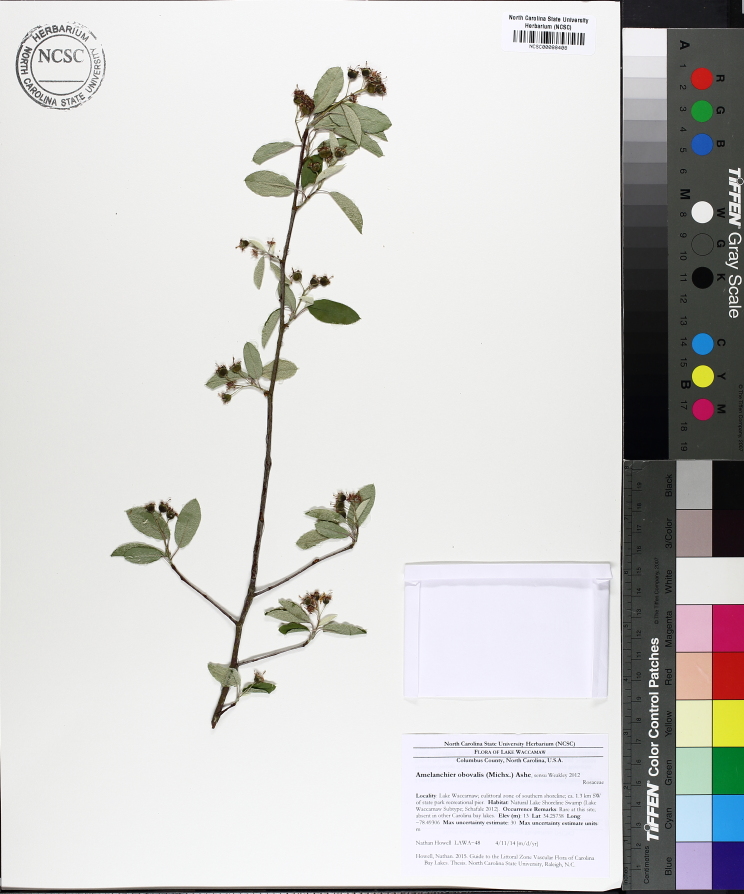
Specimen: *Howell LAWA-48* (NCSC)

**Figure 190b.**
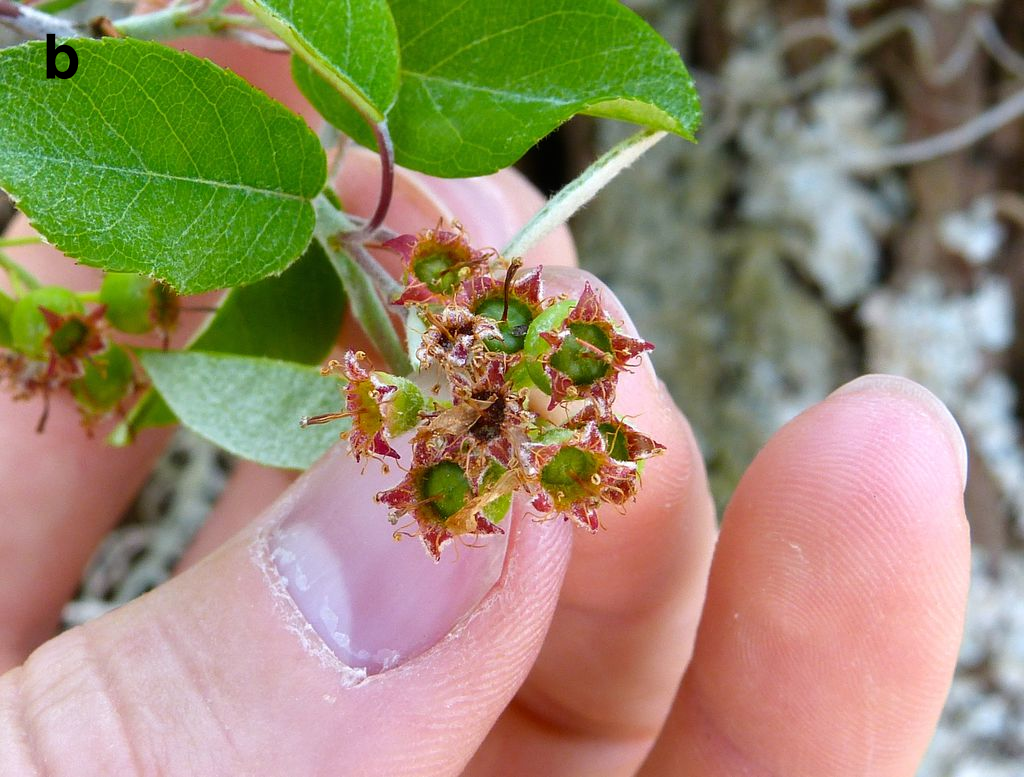
Fruits (immature)

**Figure 191a.**
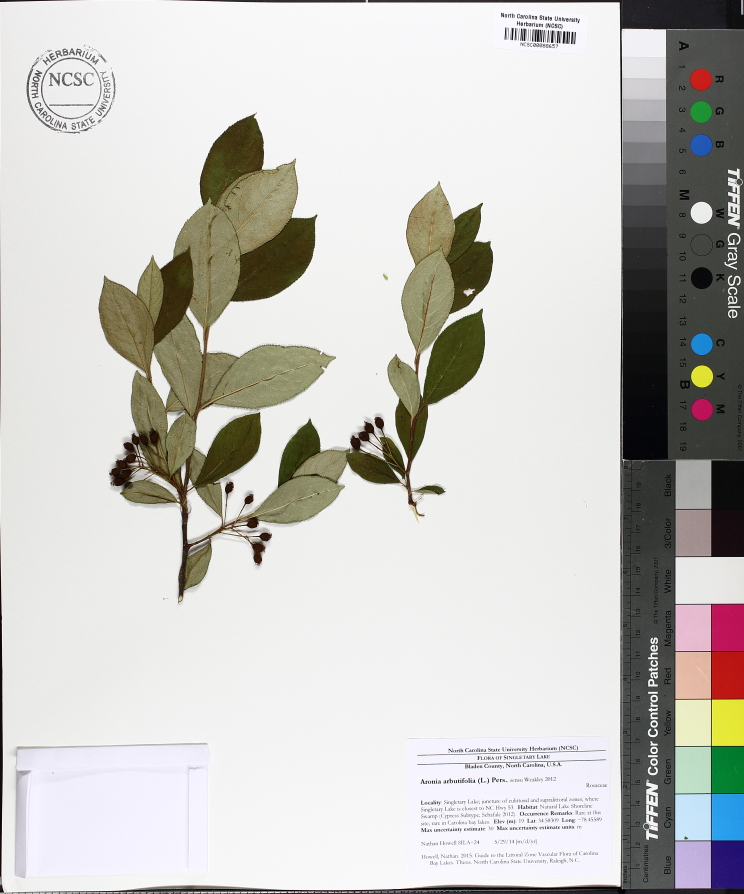
Specimen: *Howell SILA-24* (NCSC)

**Figure 191b.**
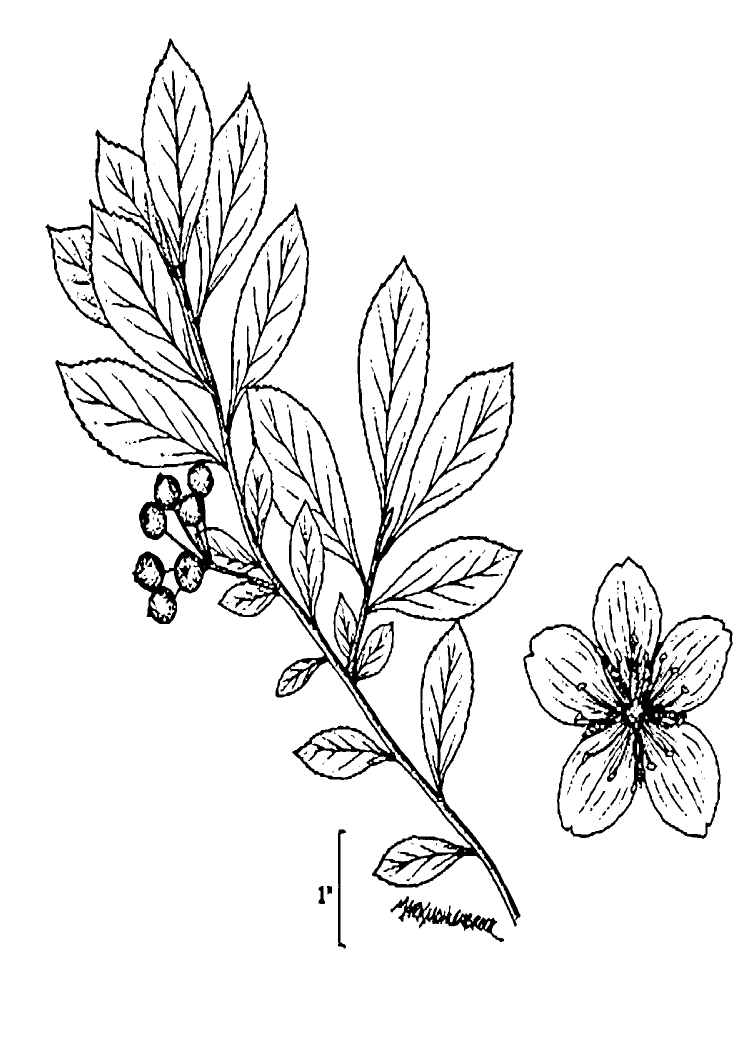
Illustration

**Figure 191c.**
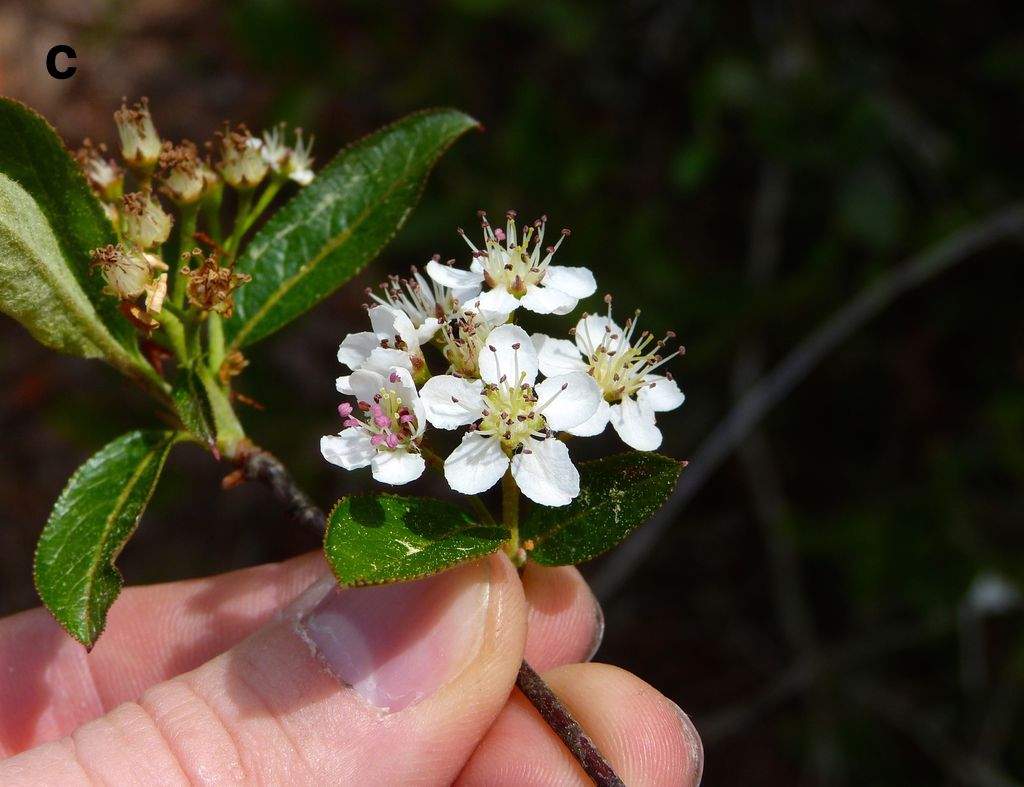
Inflorescence

**Figure 191d.**
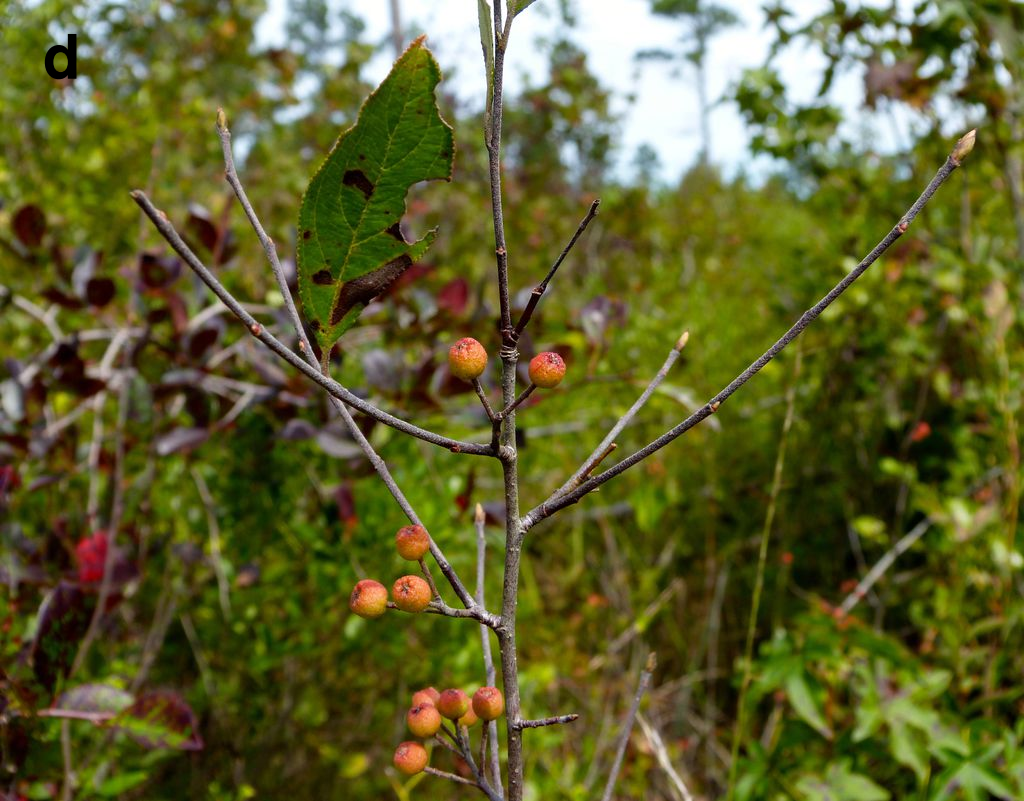
Infructescence

**Figure 192a.**
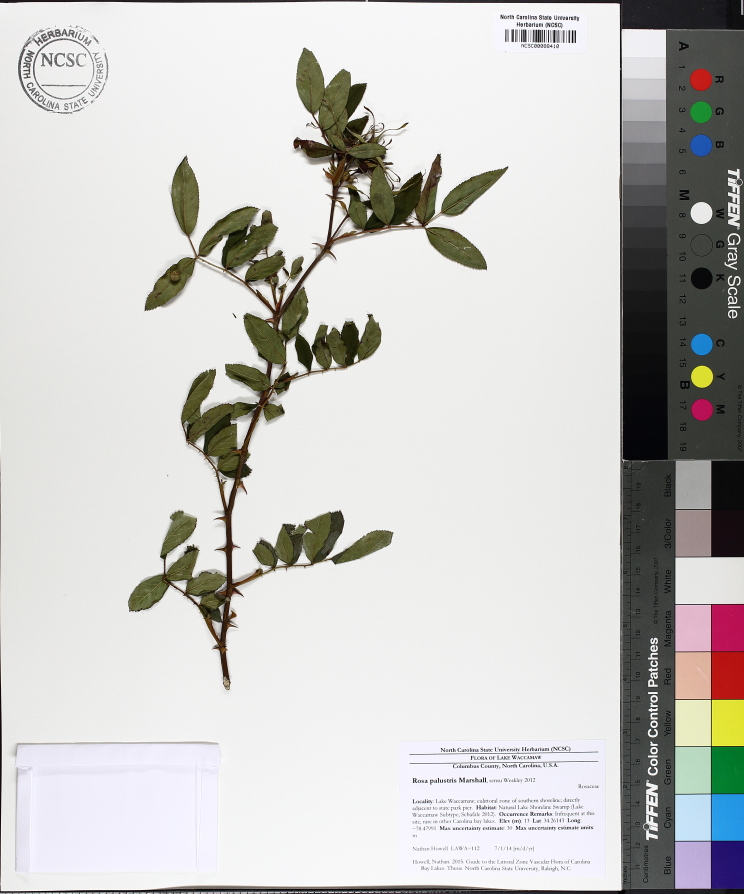
Specimen: *Howell LAWA-112* (NCSC)

**Figure 192b.**
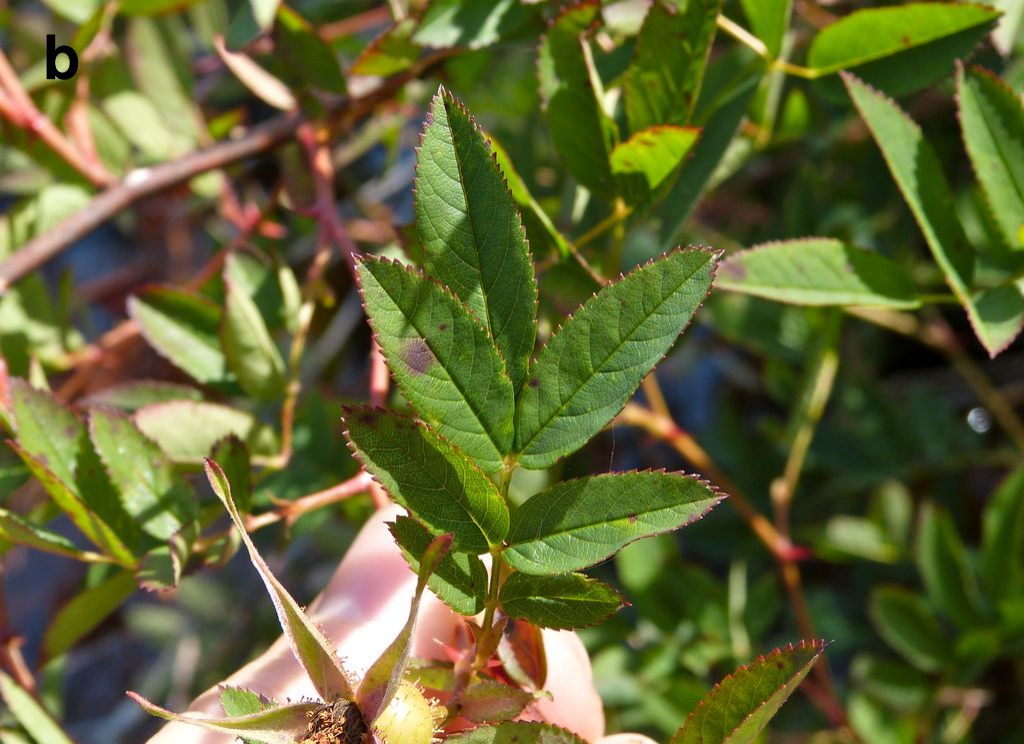
Leaf

**Figure 192c.**
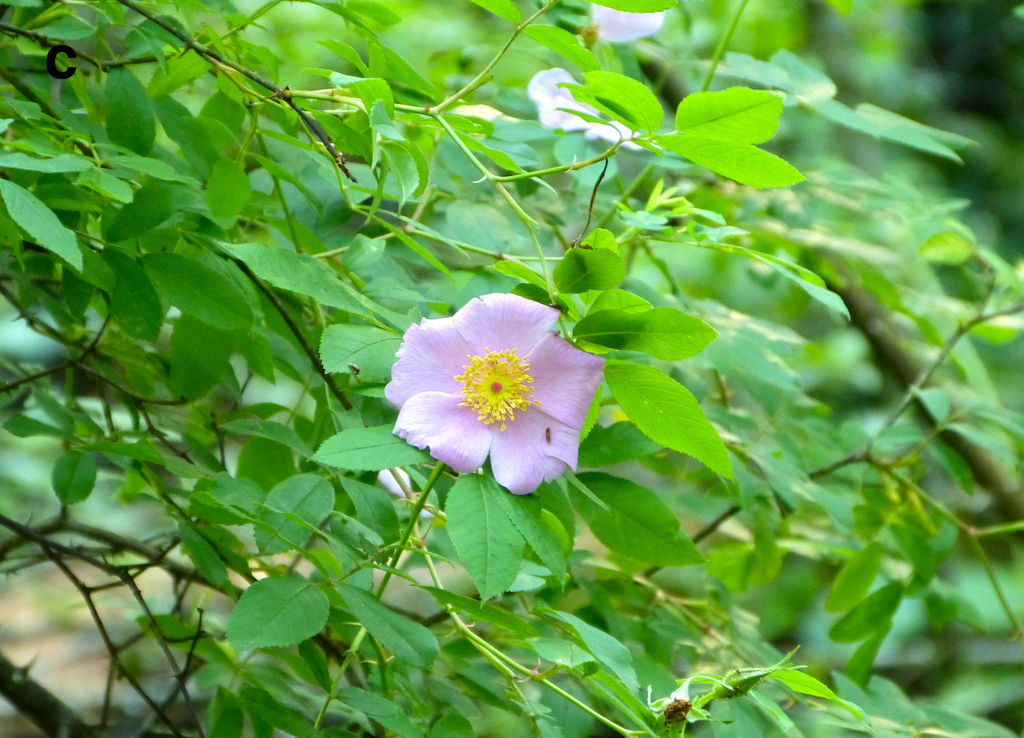
Flower

**Figure 192d.**
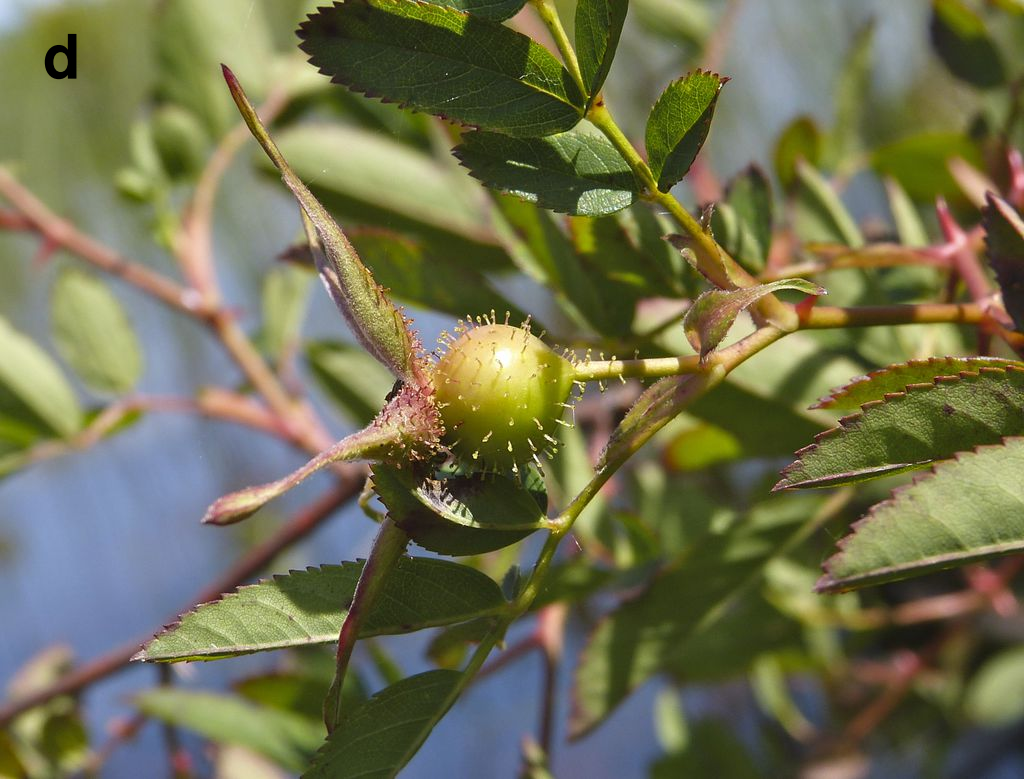
Fruit

**Figure 193a.**
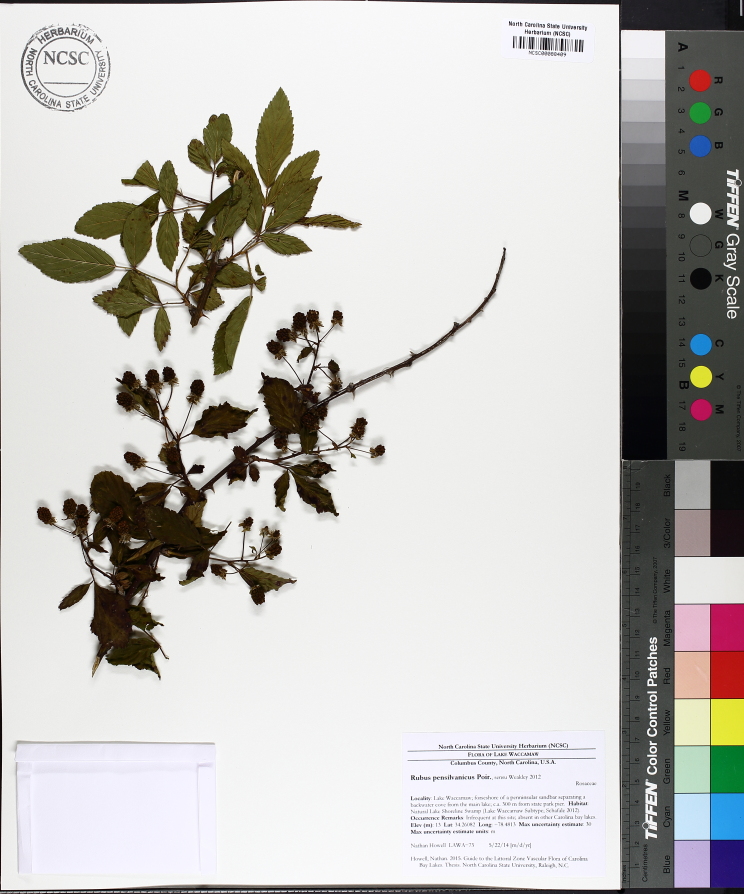
Specimen: *Howell LAWA-73* (NCSC)

**Figure 193b.**
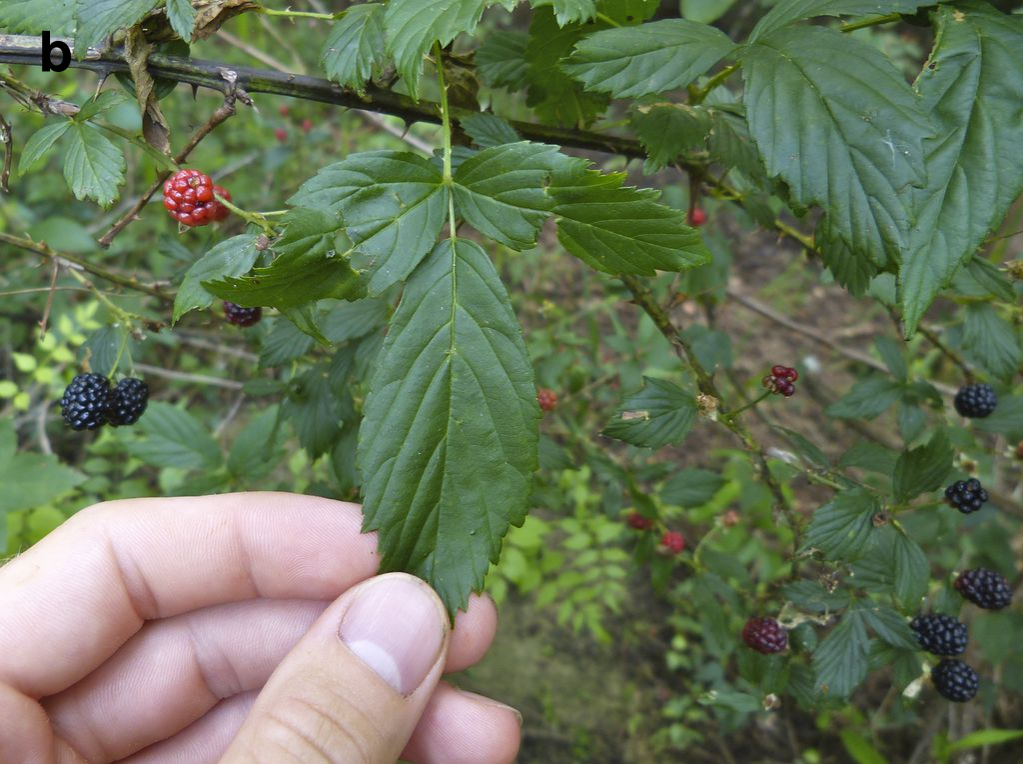
Leaf

**Figure 194a.**
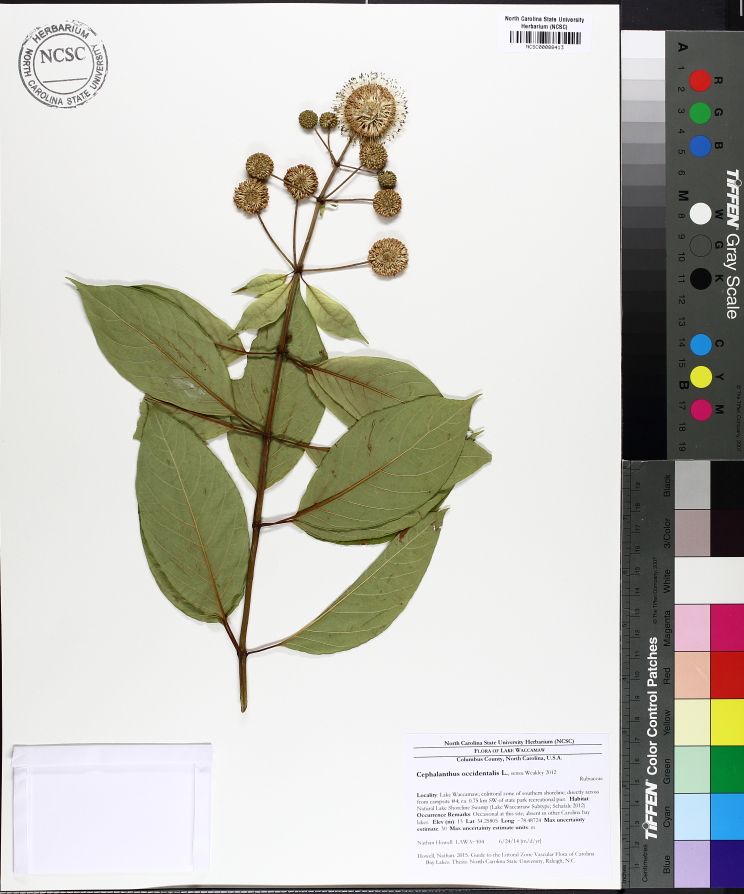
Specimen: *Howell LAWA-104* (NCSC)

**Figure 194b.**
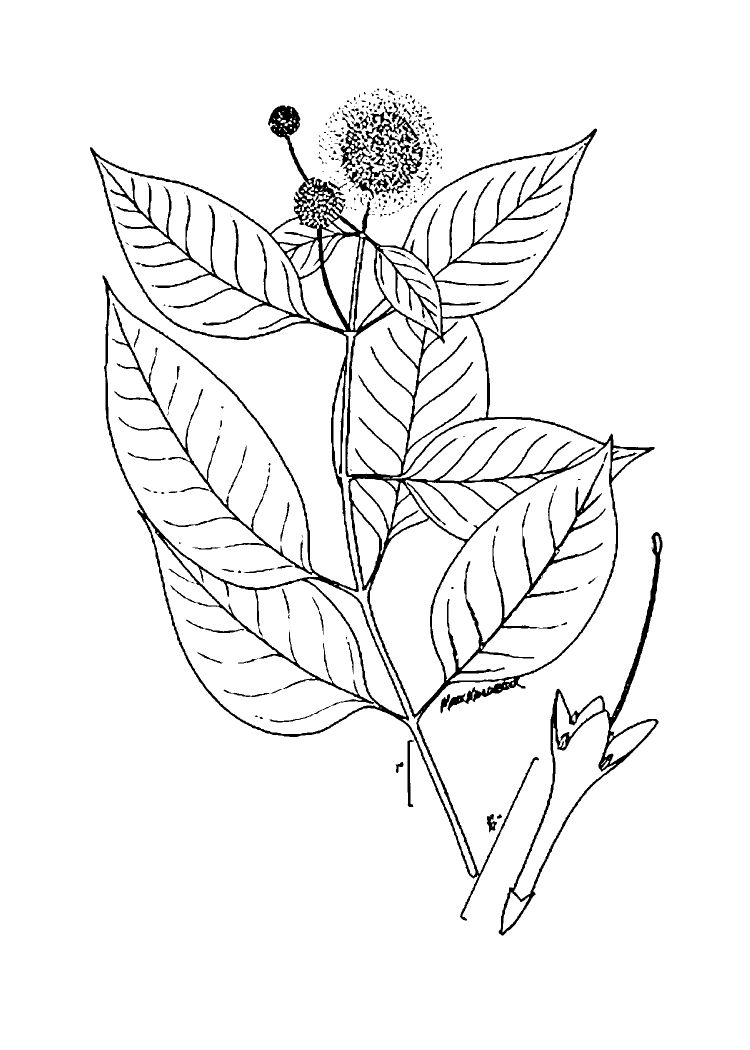
Illustration

**Figure 194c.**
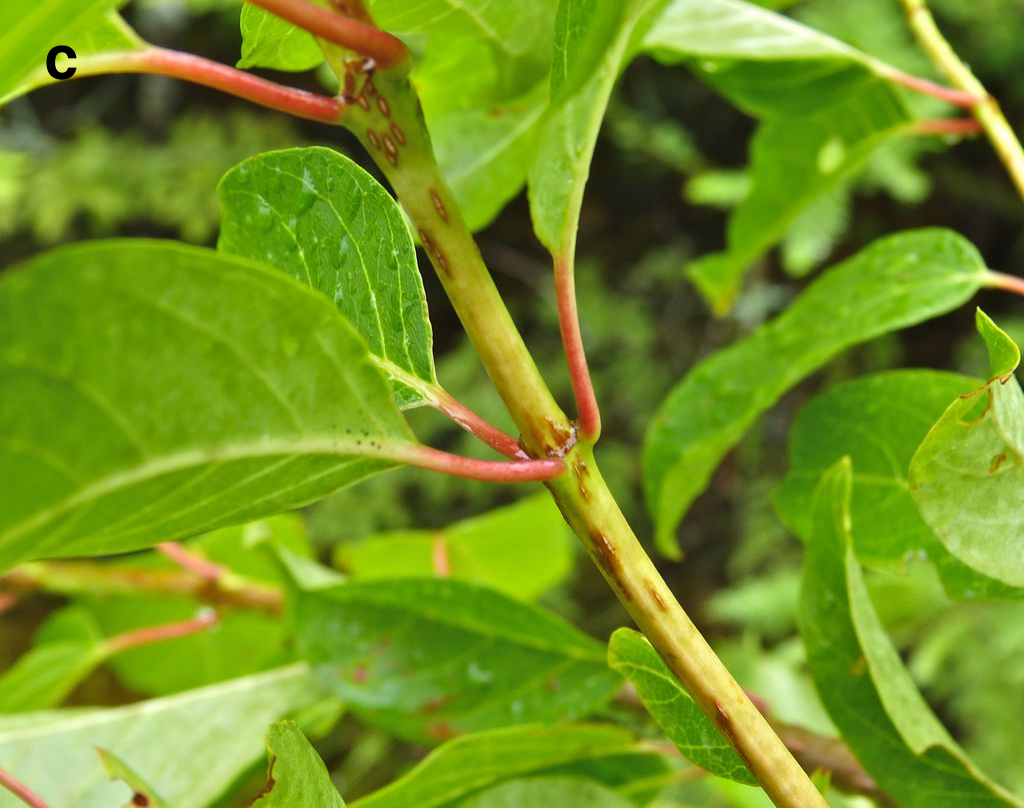
Leaves

**Figure 194d.**
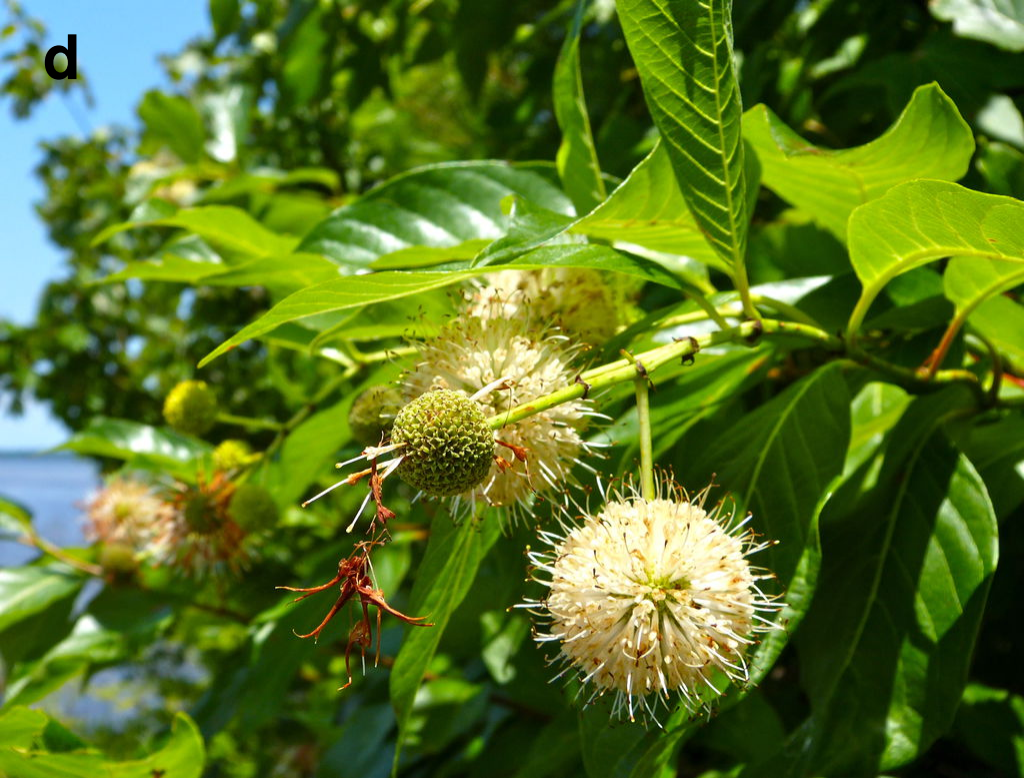
Inflorescence

**Figure 195a.**
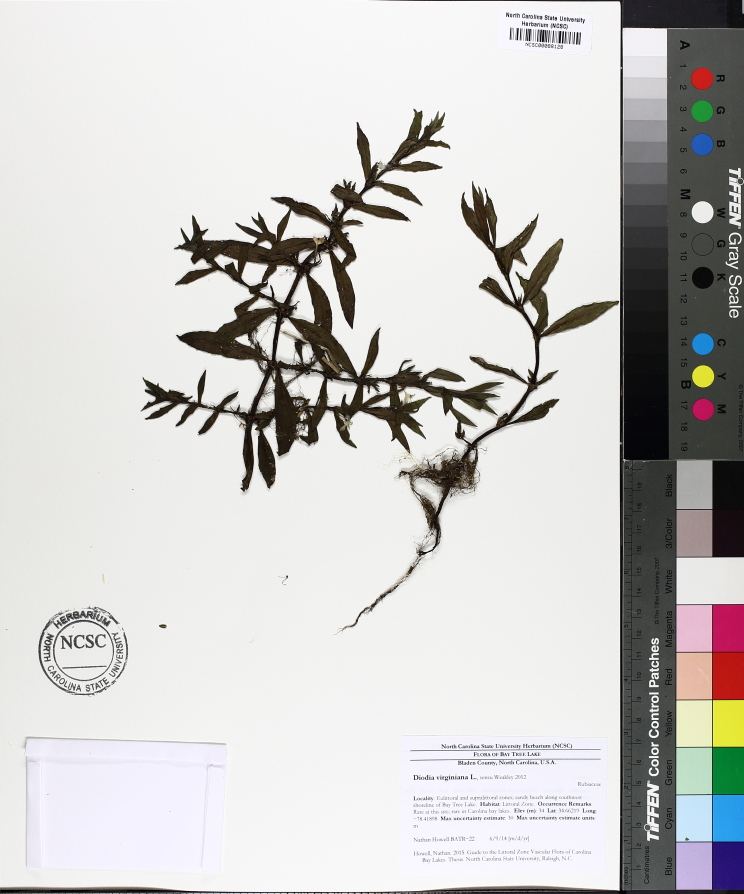
Specimen: *Howell BATR-22* (NCSC)

**Figure 195b.**
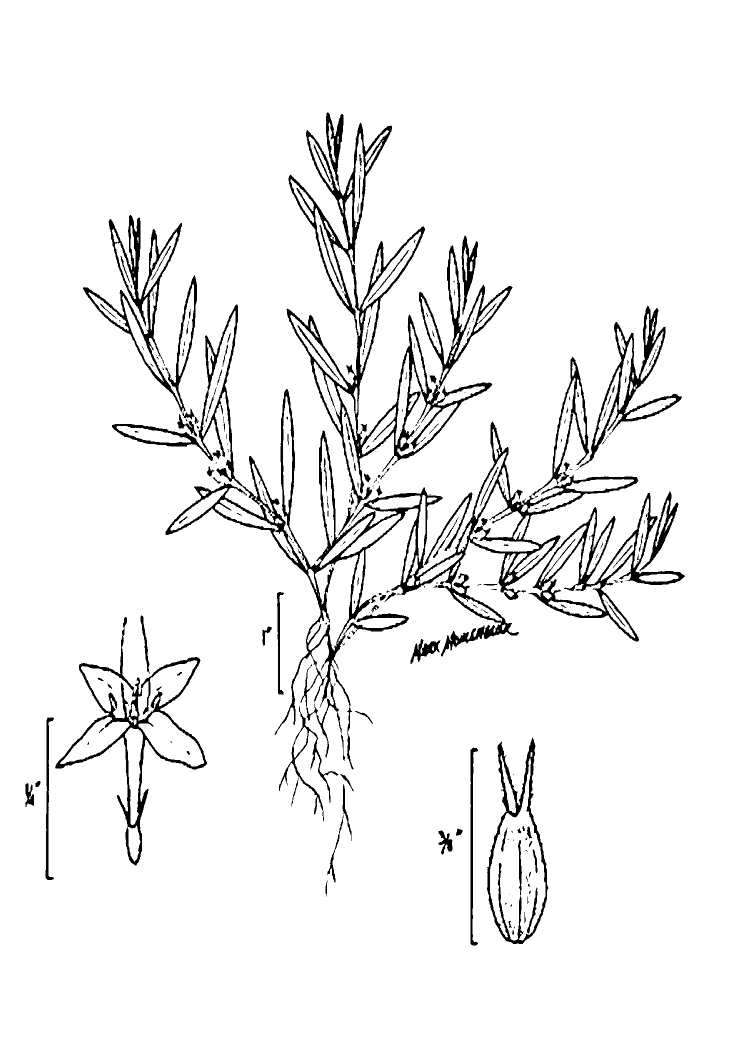
Illustration

**Figure 195c.**
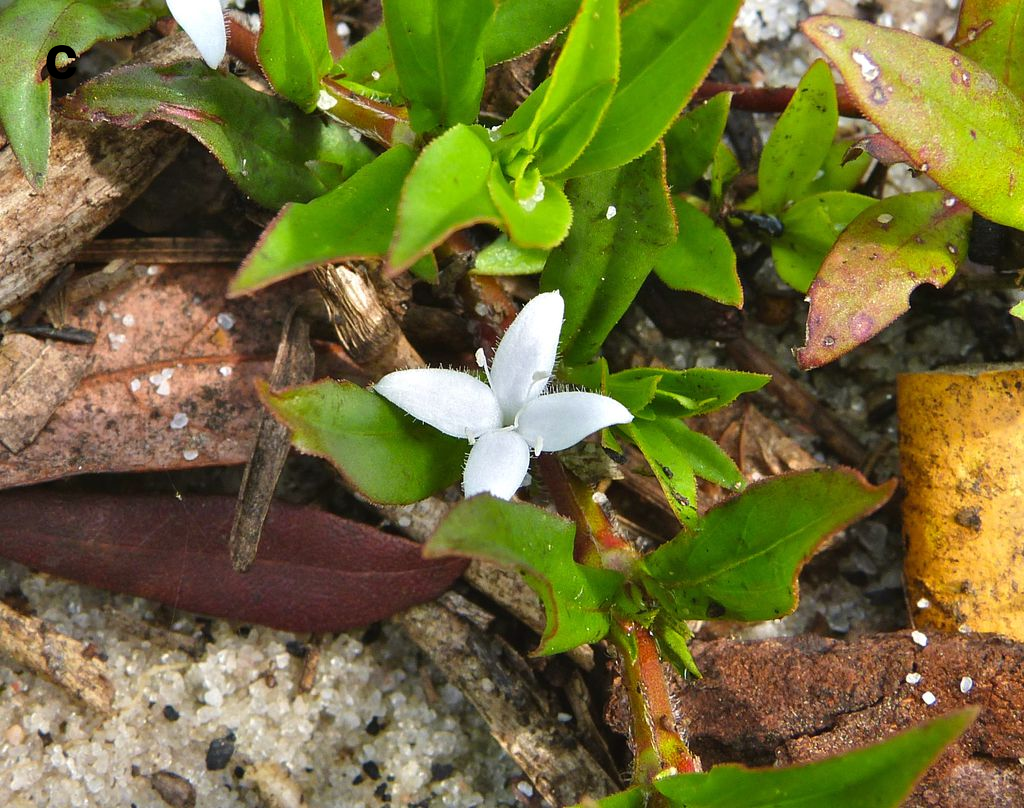
Flower (adaxial)

**Figure 195d.**
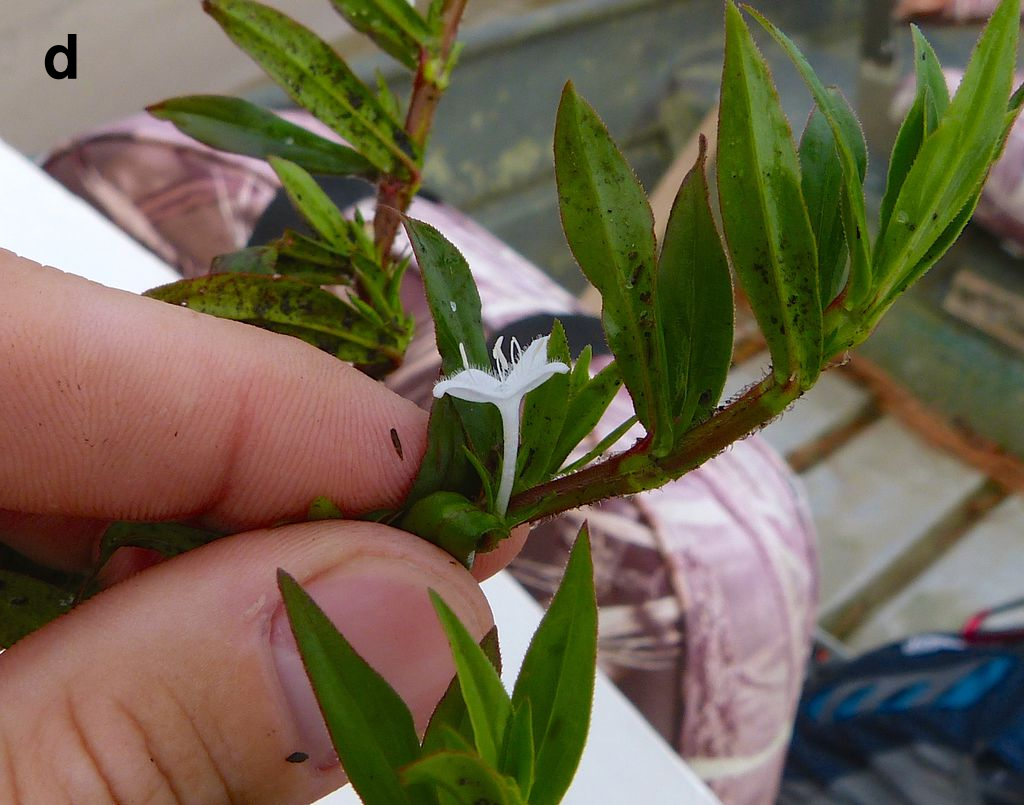
Flower (lateral)

**Figure 196a.**
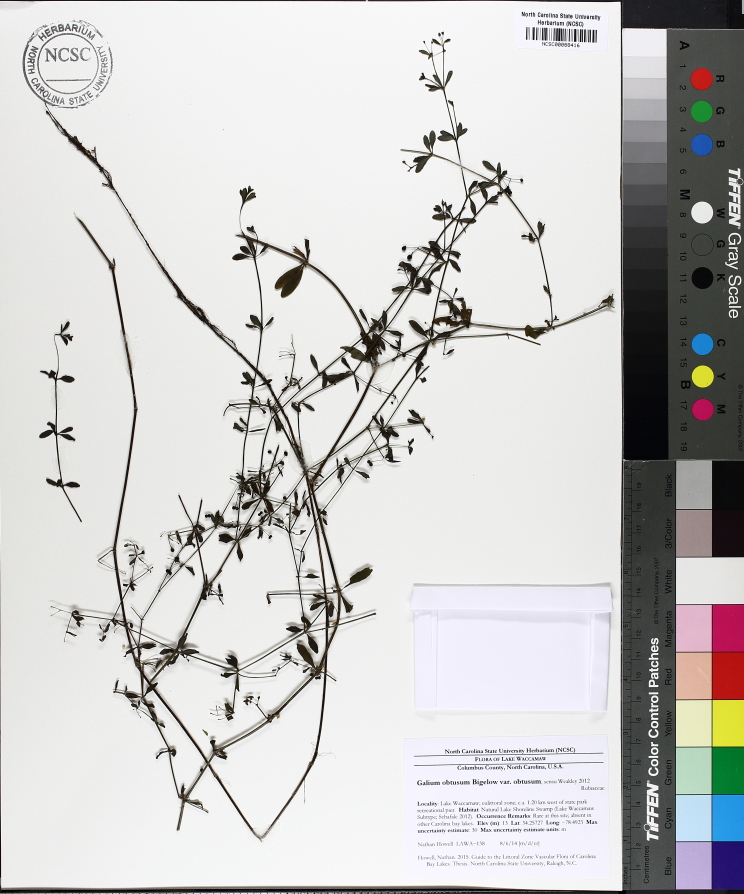
Specimen: *Howell LAWA-138* (NCSC)

**Figure 196b.**
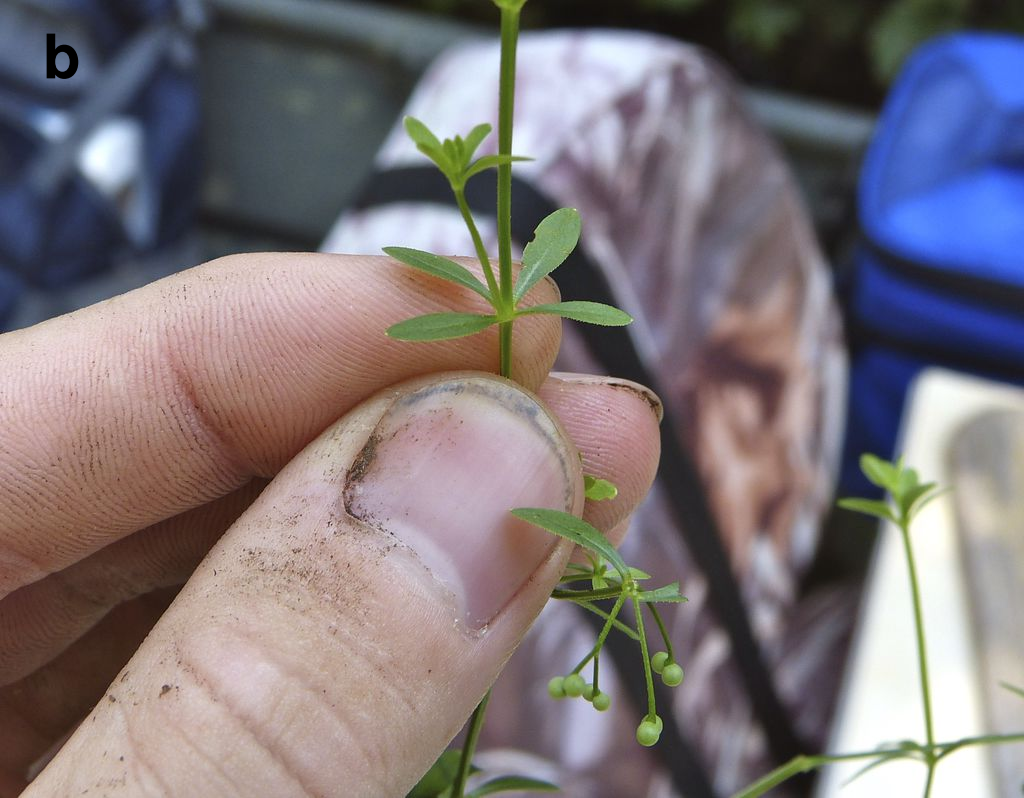
Leaves

**Figure 196c.**
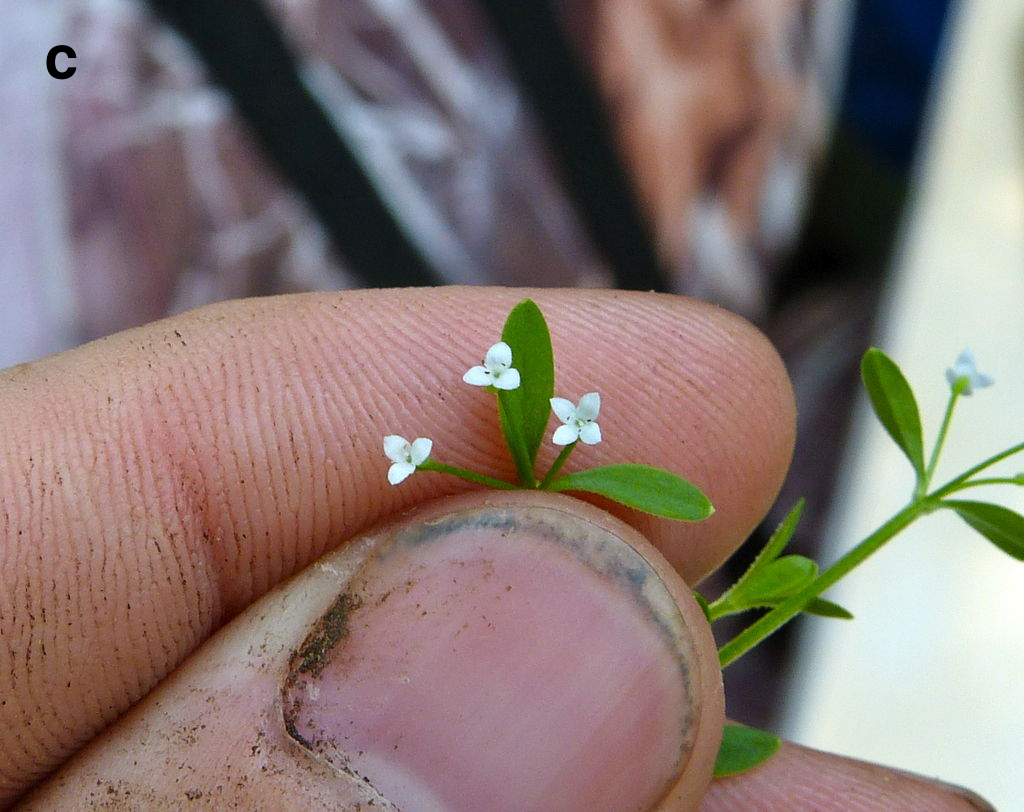
Flowers

**Figure 196d.**
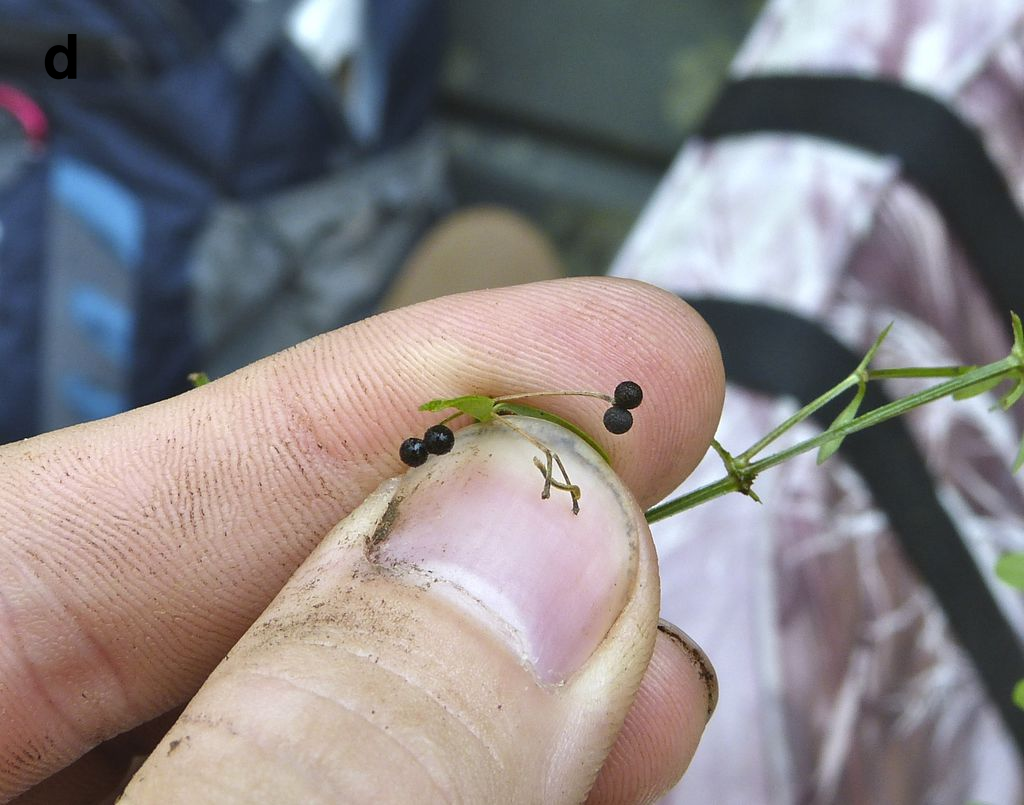
Fruits

**Figure 197a.**
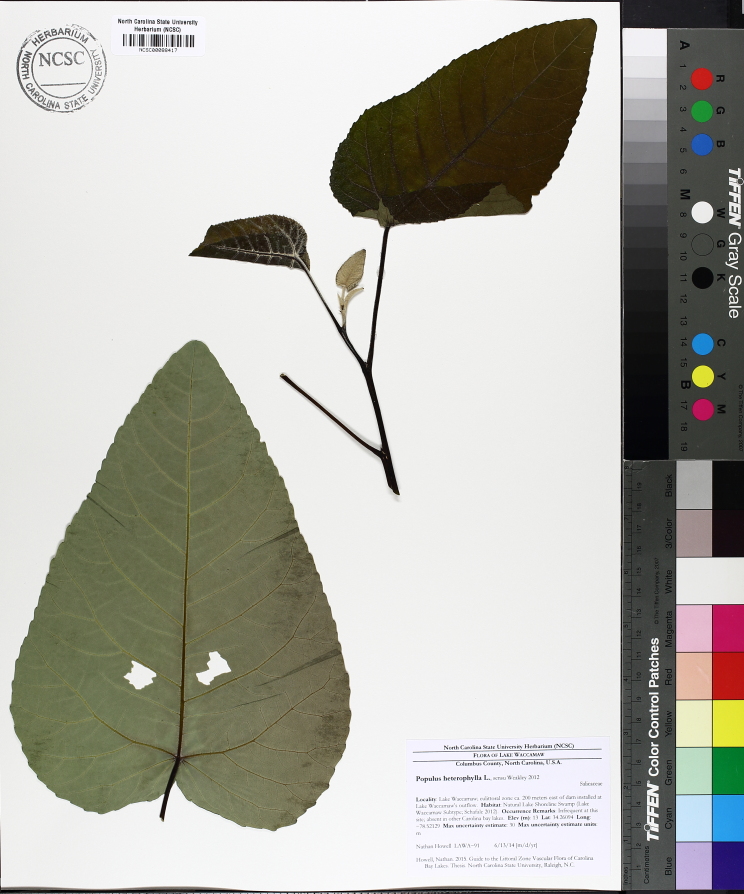
Specimen: *Howell LAWA-91* (NCSC)

**Figure 197b.**
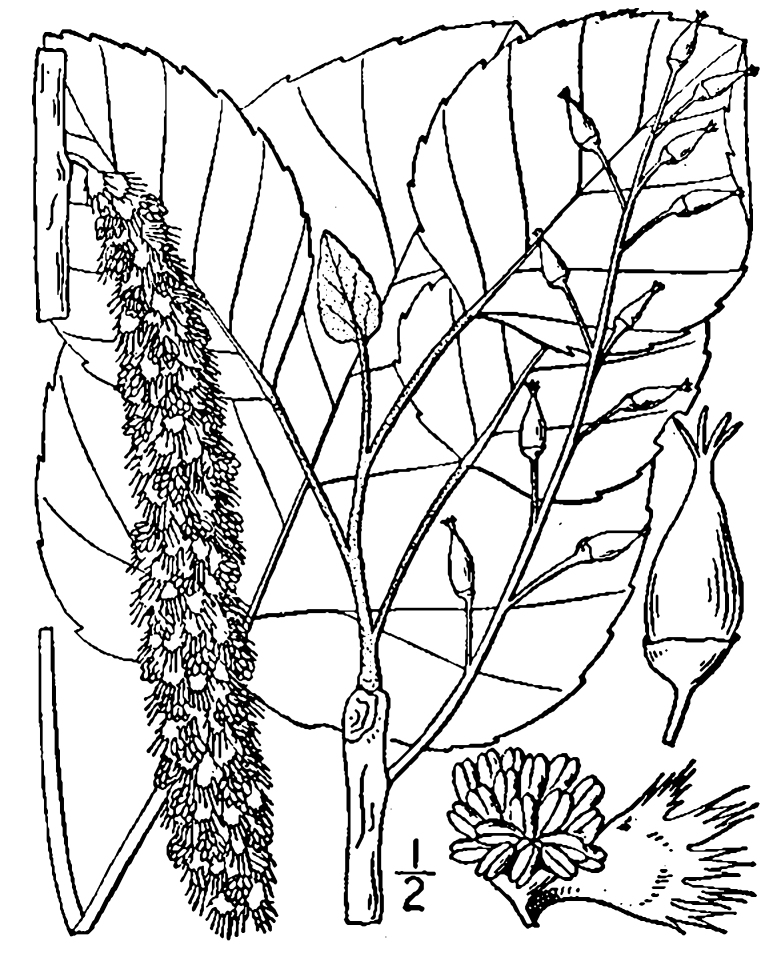
Illustration

**Figure 197c.**
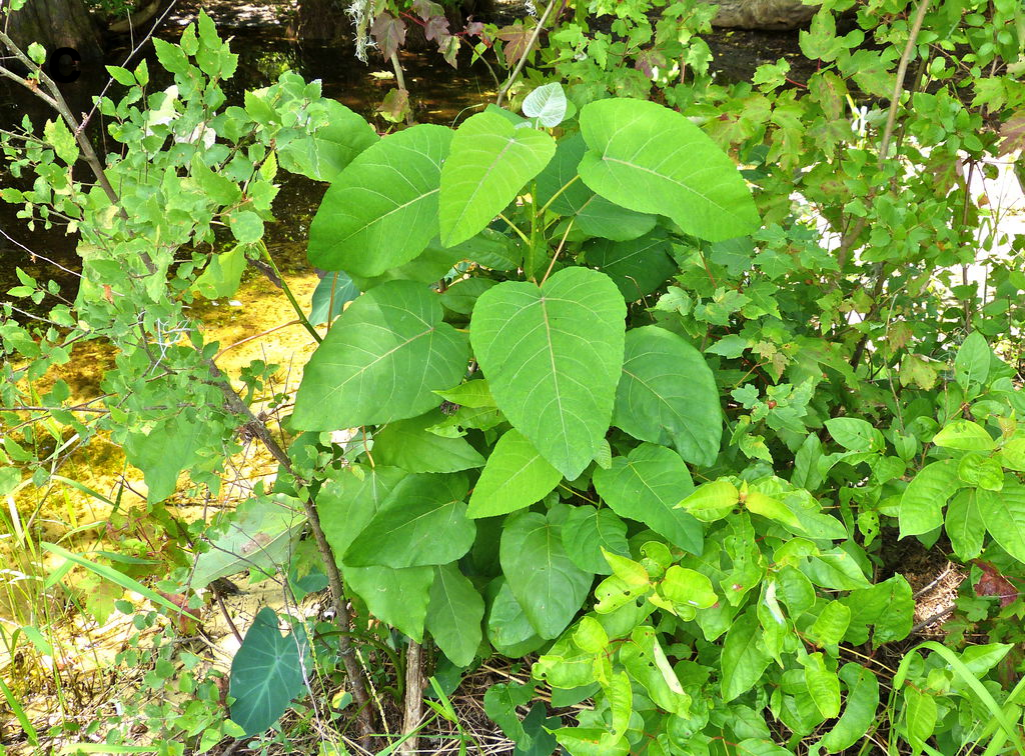
Young tree

**Figure 197d.**
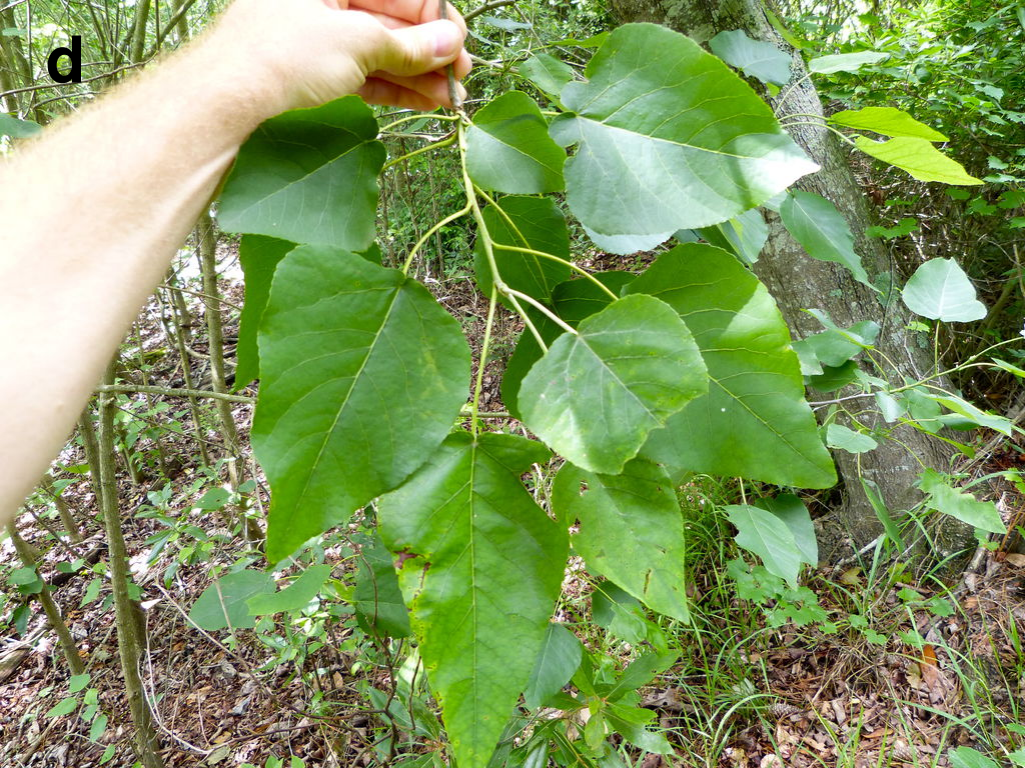
Leaves

**Figure 197e.**
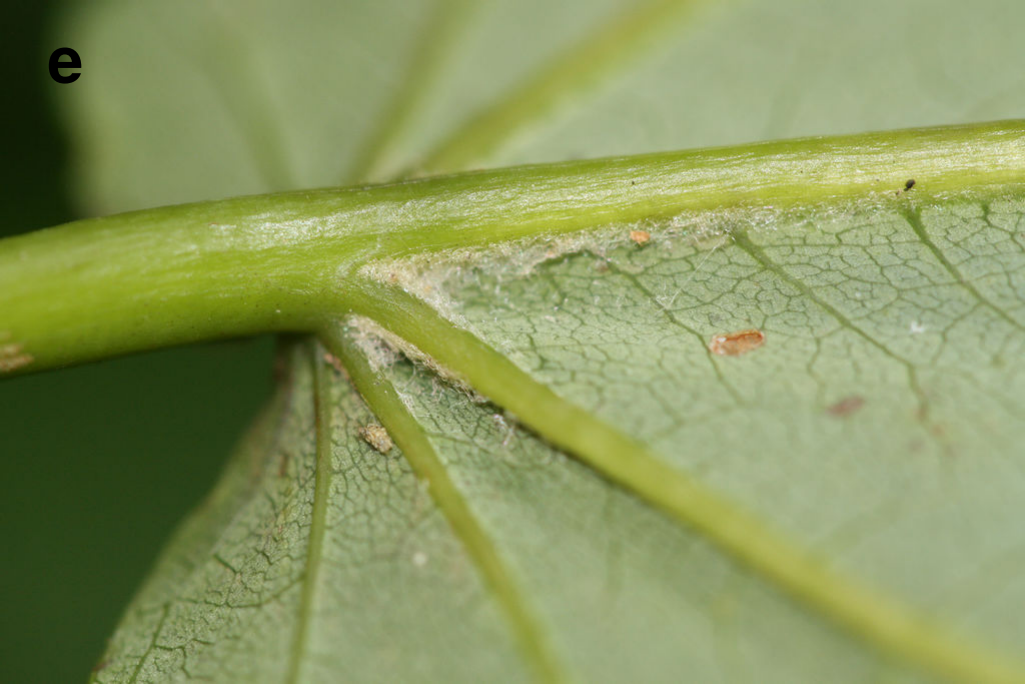
Abaxial leaf surface

**Figure 197f.**
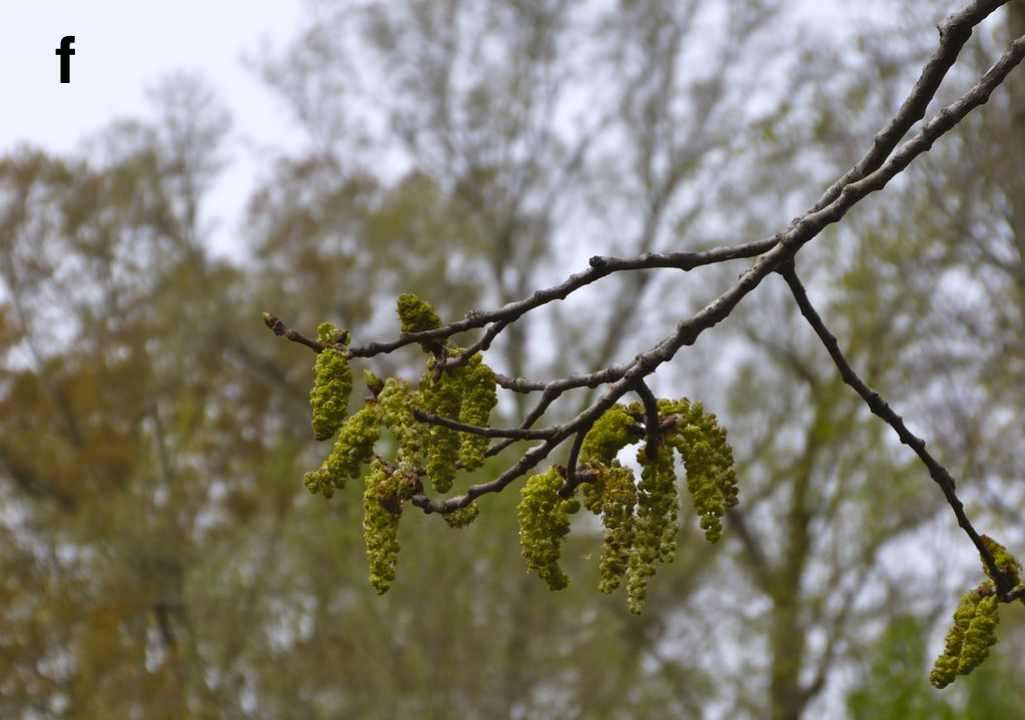
Inflorescences

**Figure 198a.**
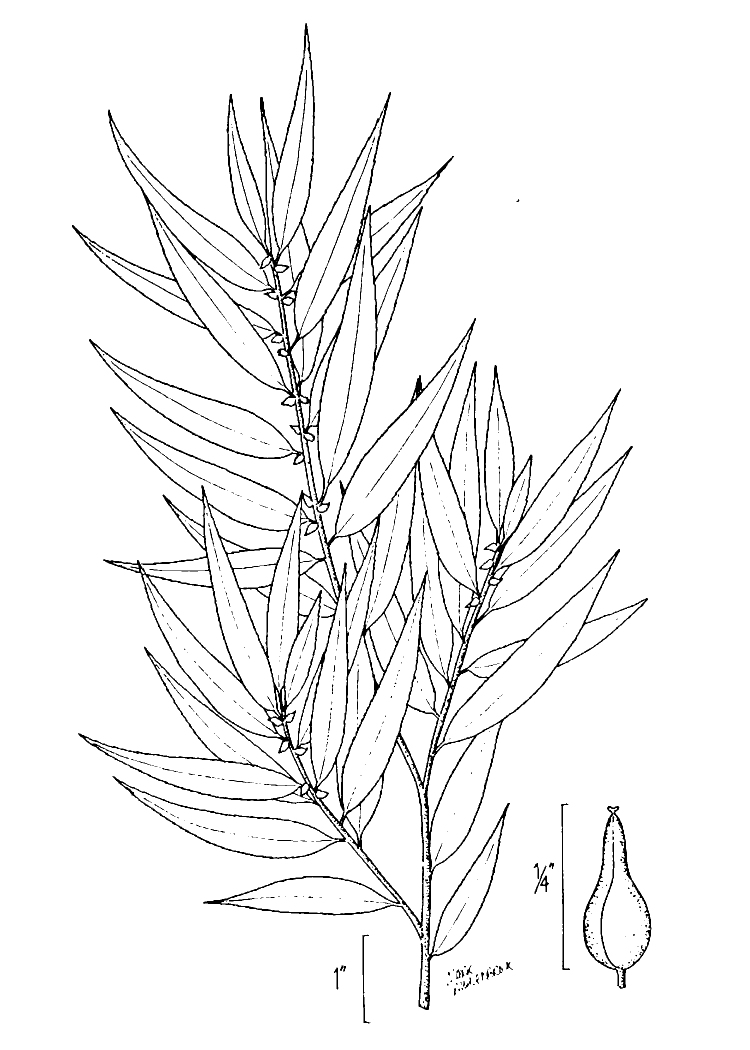
Illustration

**Figure 198b.**
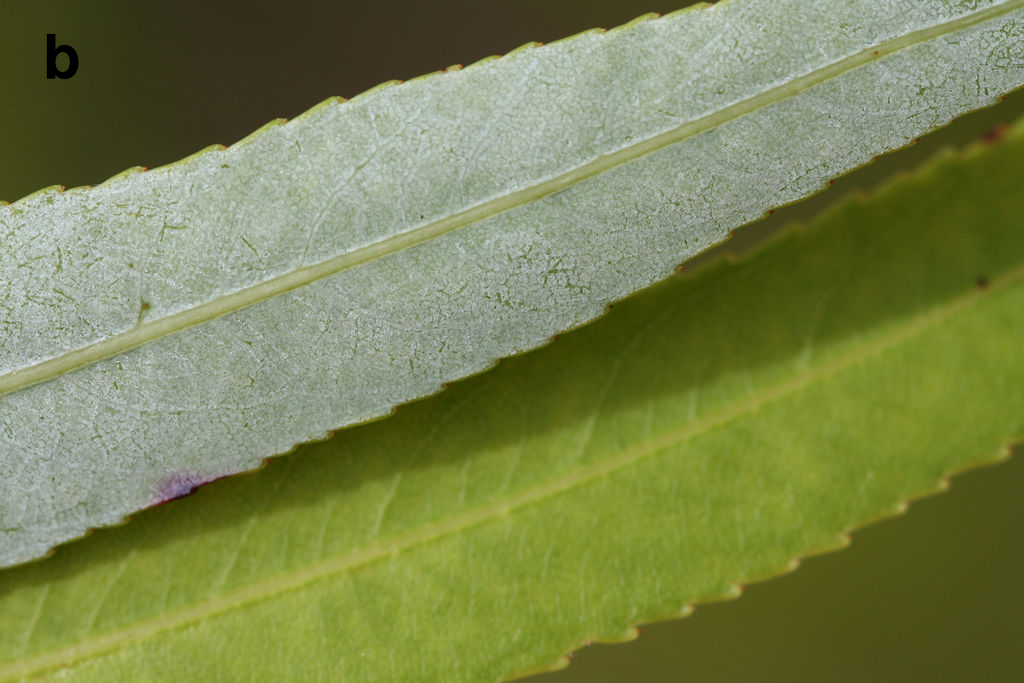
Leaves (abaxial surface [top], adaxial surface [bottom])

**Figure 199a.**
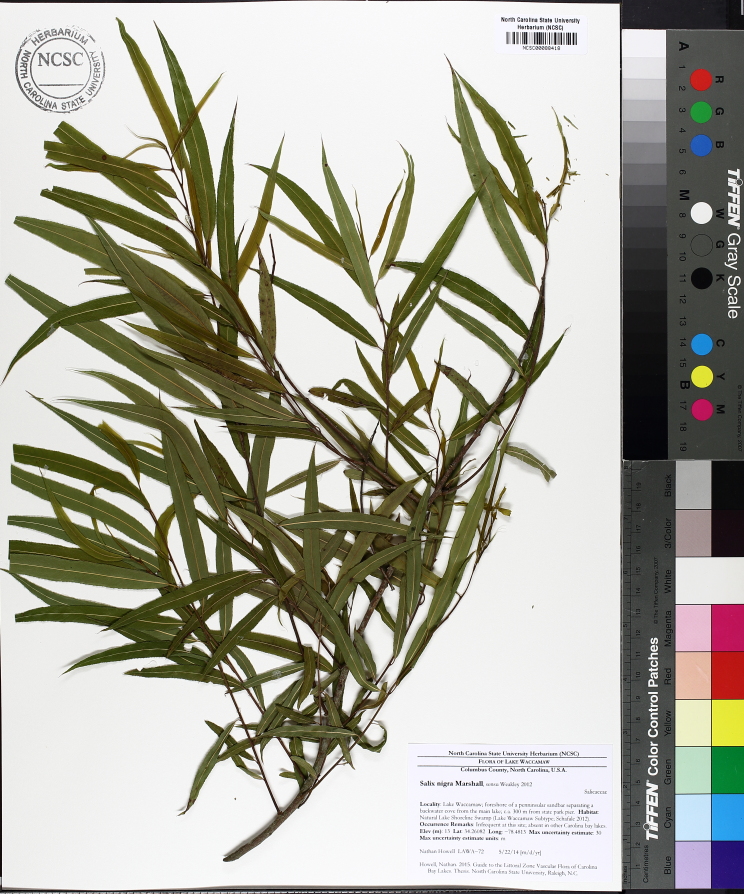
Specimen: *Howell LAWA-72* (NCSC)

**Figure 199b.**
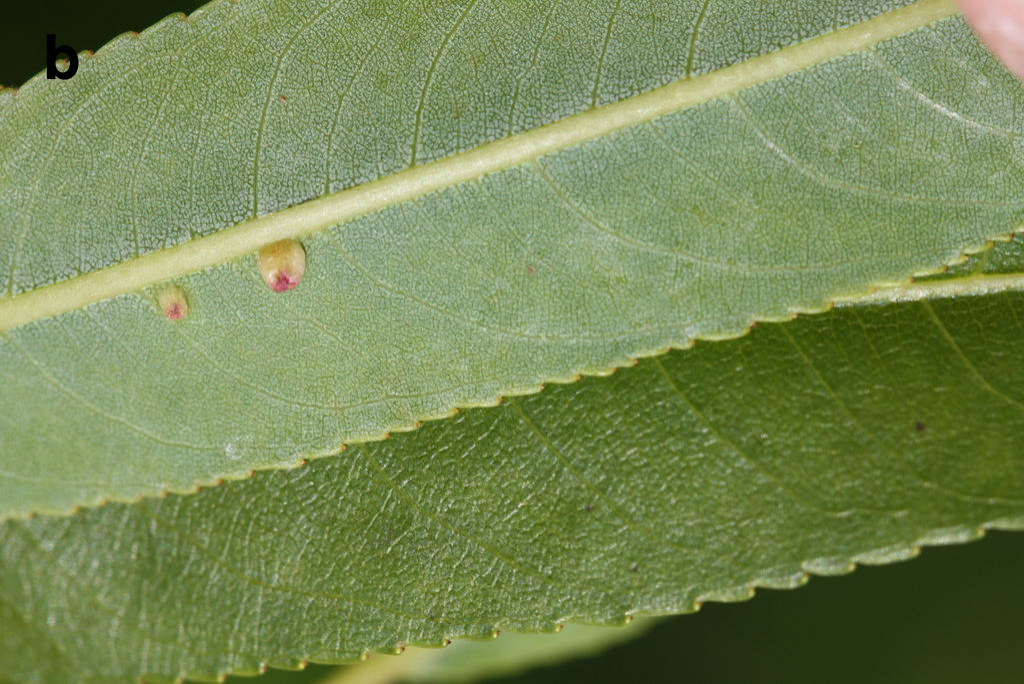
Leaves (abaxial surface [top], adaxial surface [bottom])

**Figure 200a.**
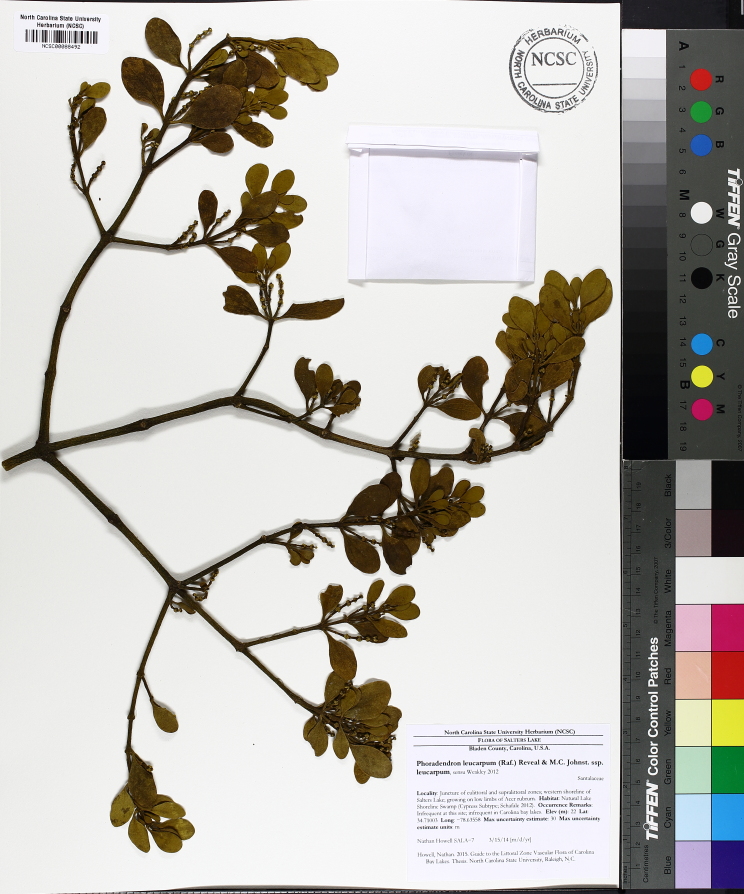
Specimen: *Howell SALA-7* (NCSC)

**Figure 200b.**
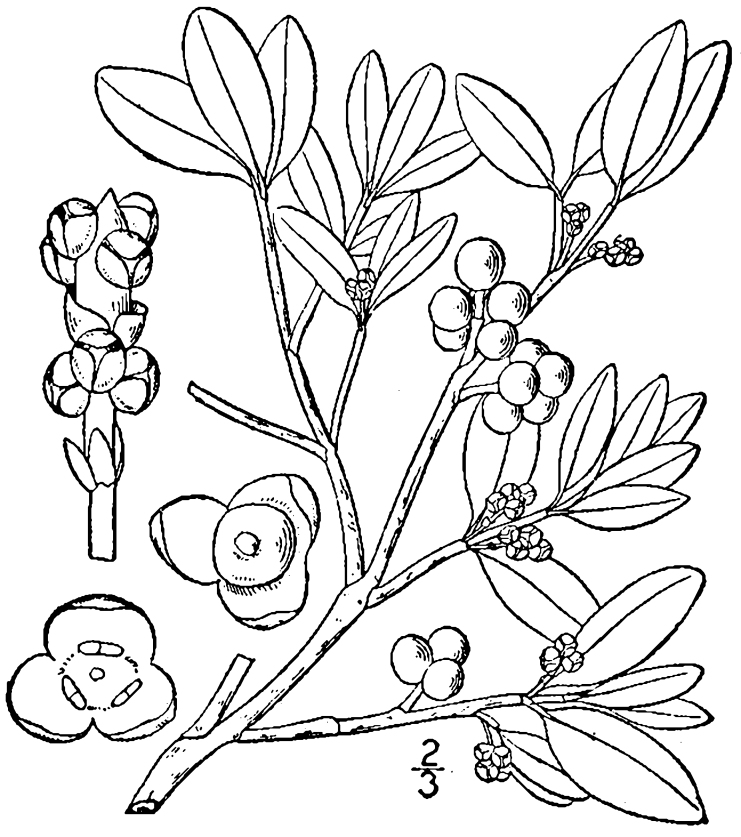
Illustration

**Figure 200c.**
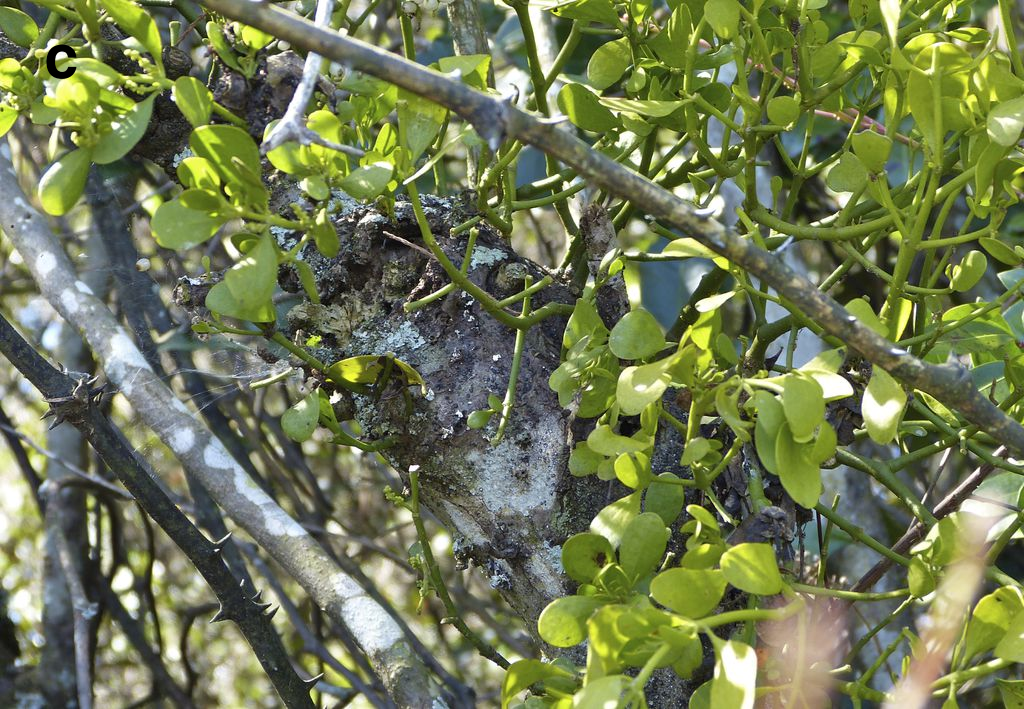
Habit

**Figure 200d.**
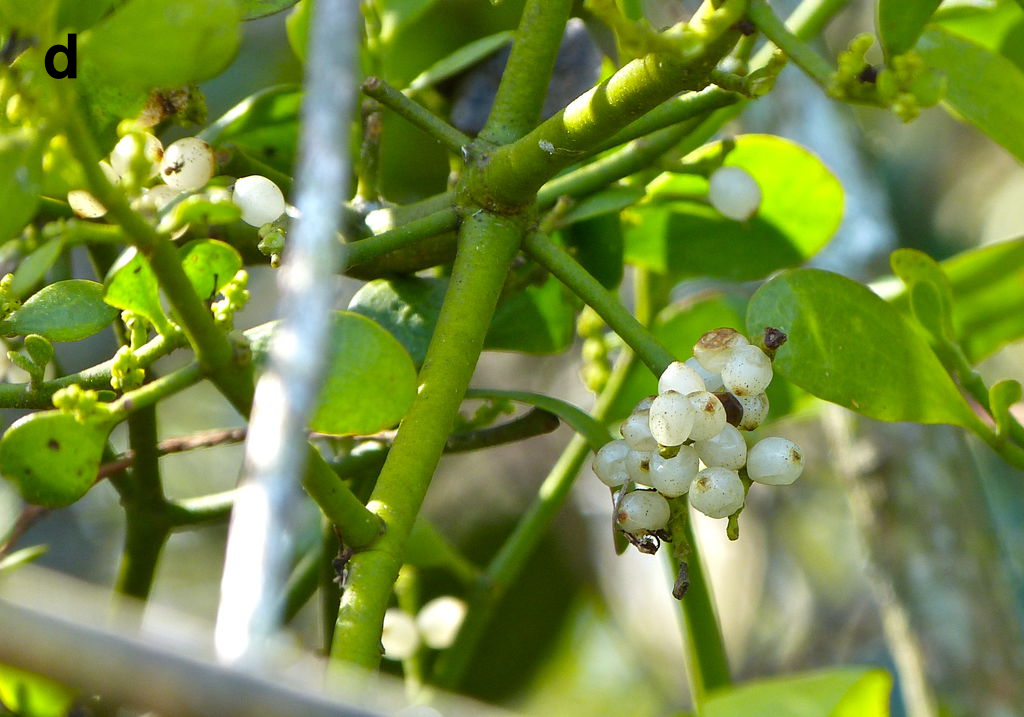
Fruits

**Figure 201a.**
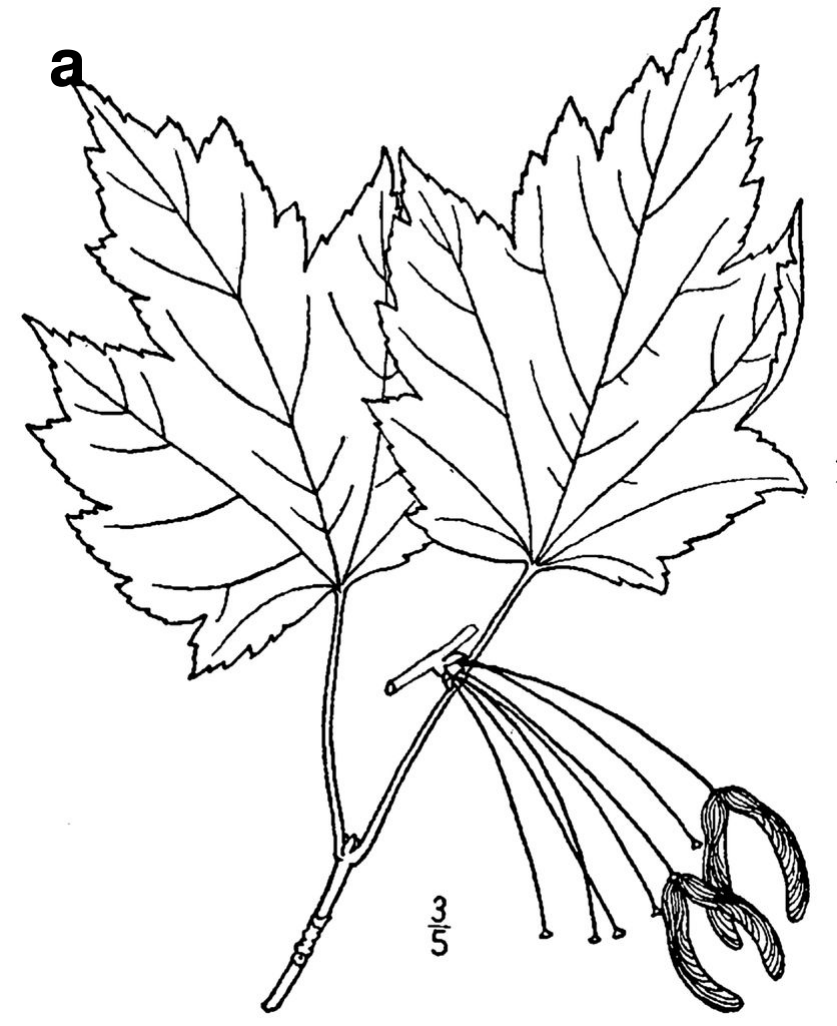
Illustration

**Figure 201b.**
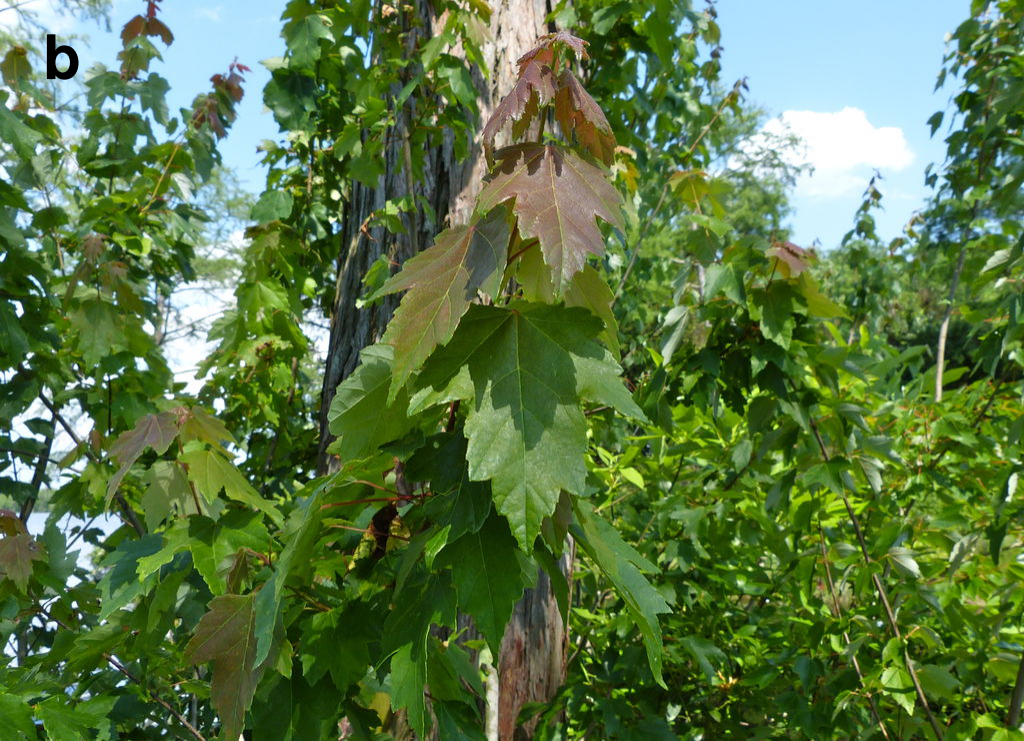
Leaves

**Figure 202a.**
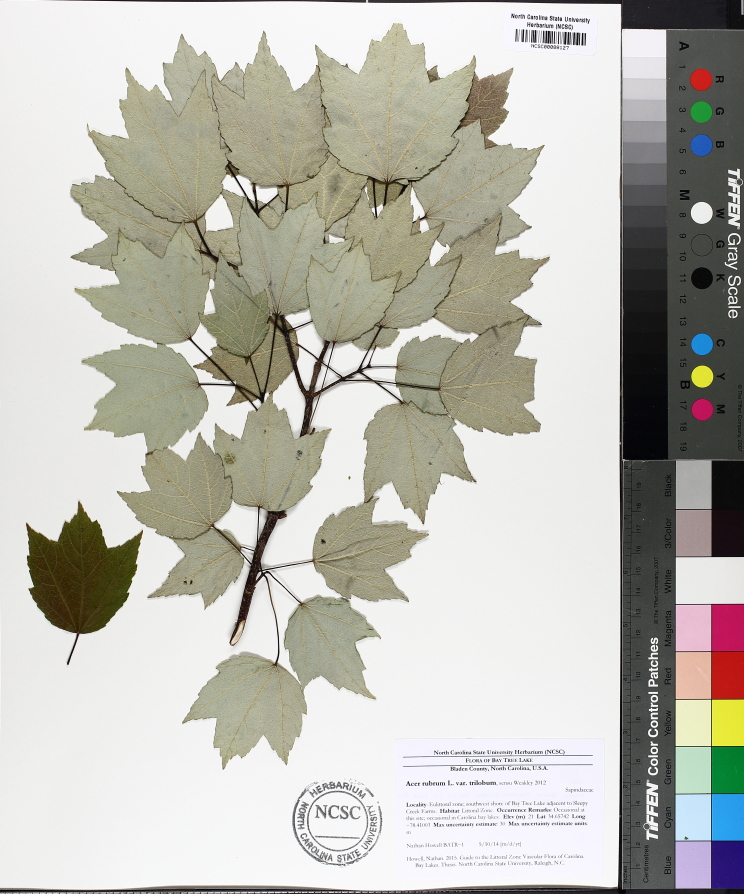
Specimen: *Howell BATR-1* (NCSC)

**Figure 202b.**
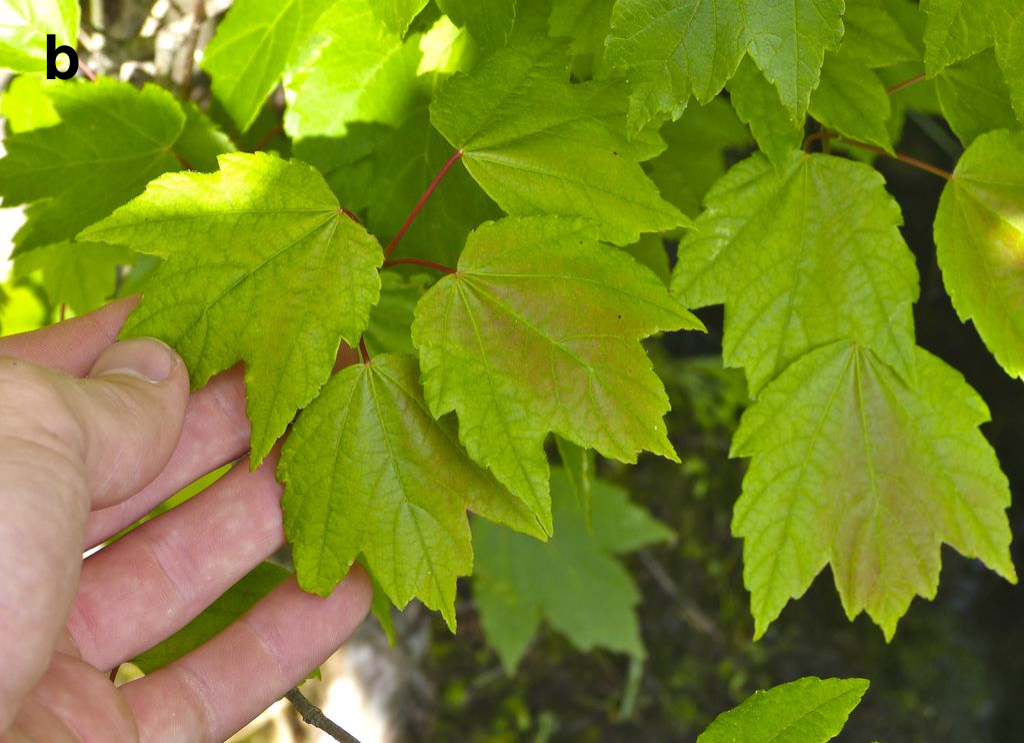
Leaves

**Figure 204a.**
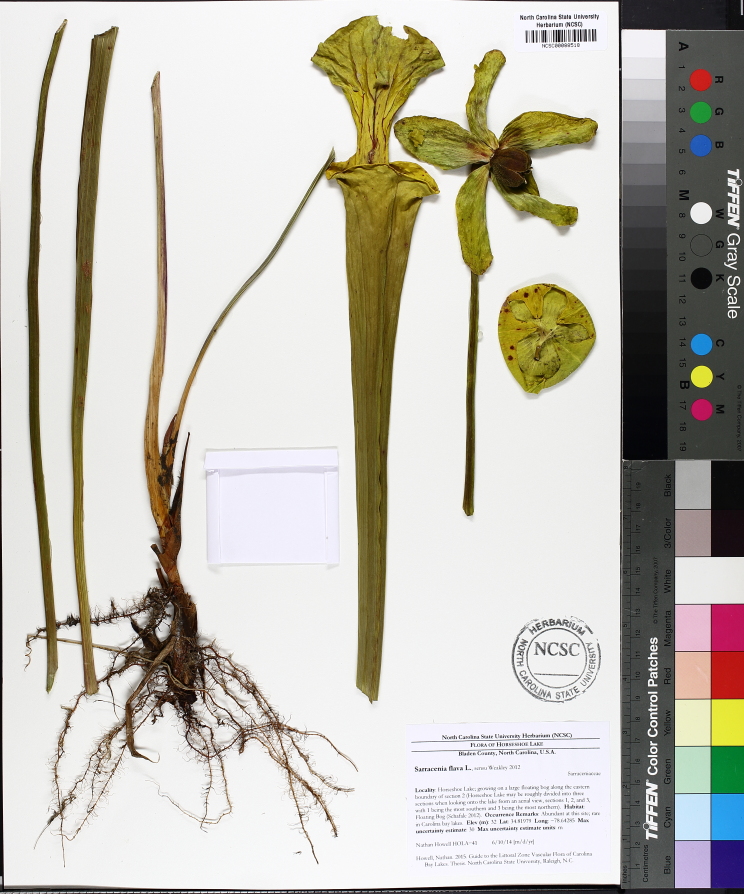
Specimen: *Howell HOLA-41* (NCSC)

**Figure 204b.**
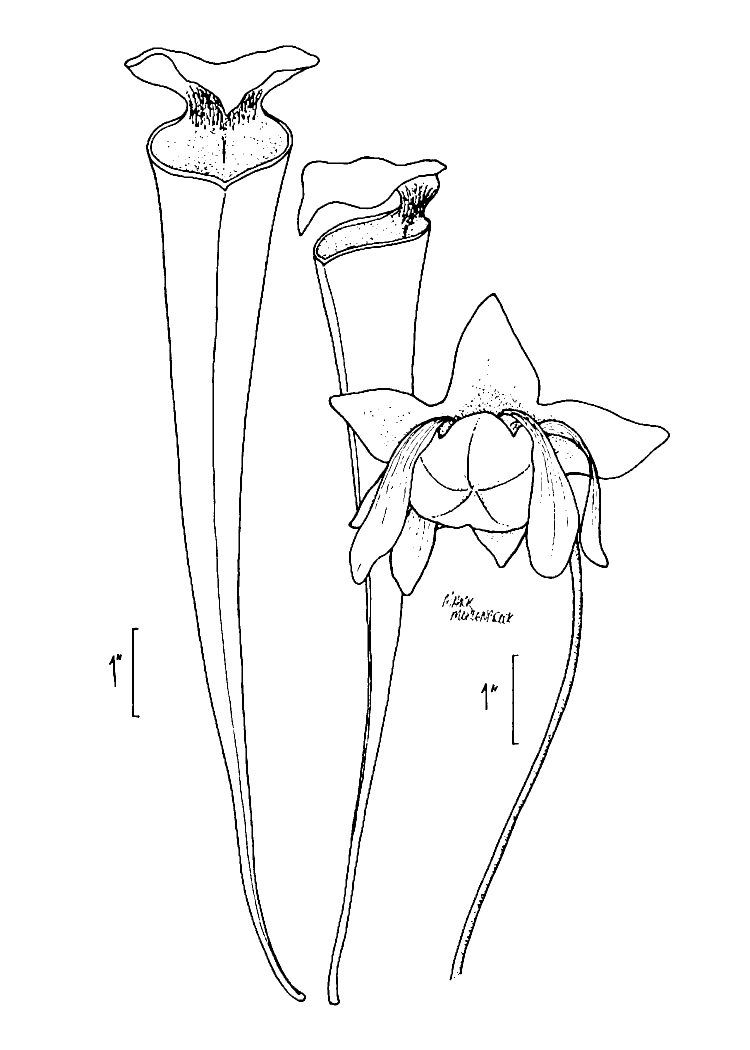
Illustration

**Figure 204c.**
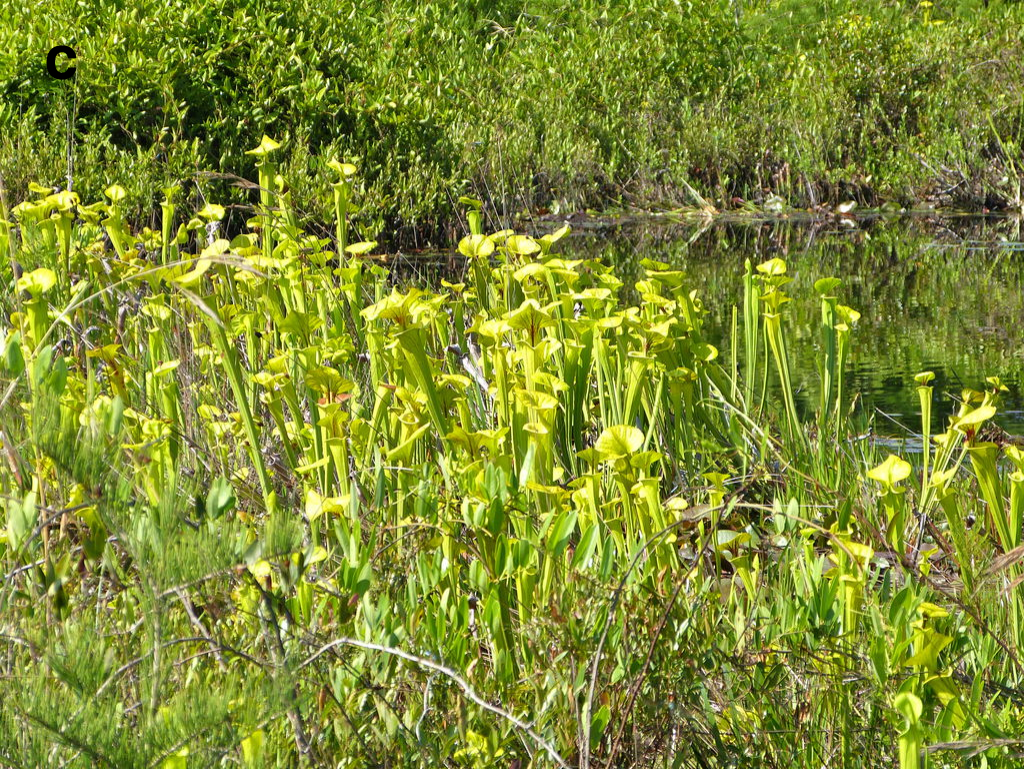
Habit

**Figure 204d.**
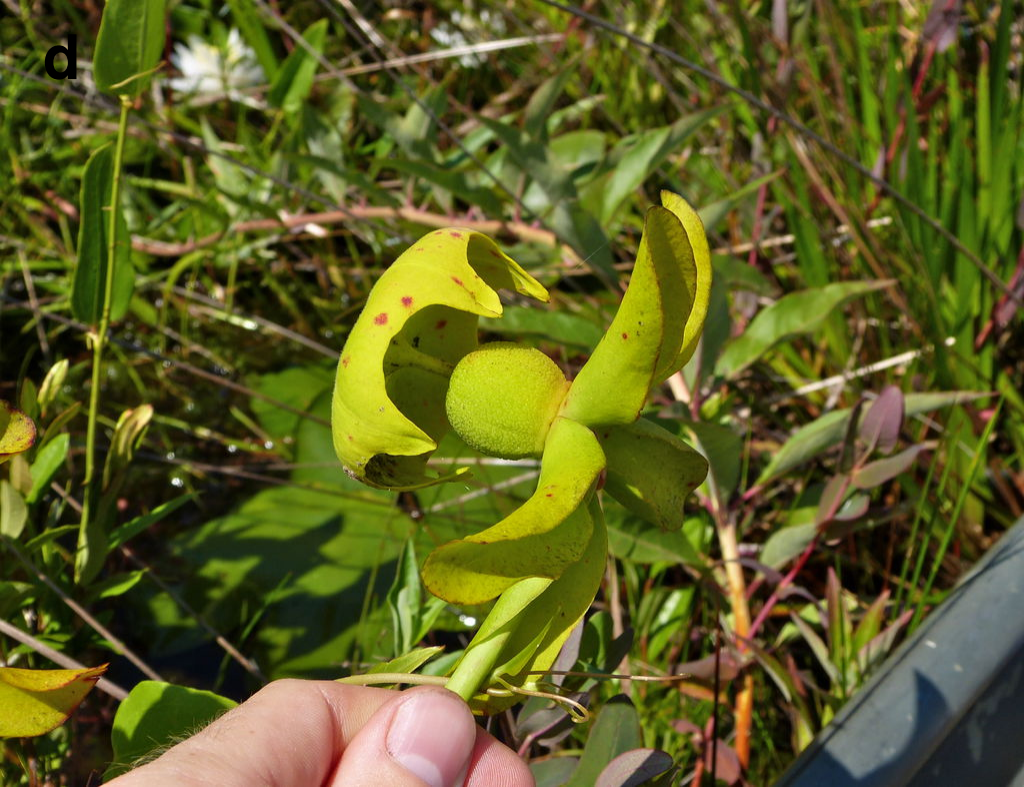
Flower (petals removed)

**Figure 205a.**
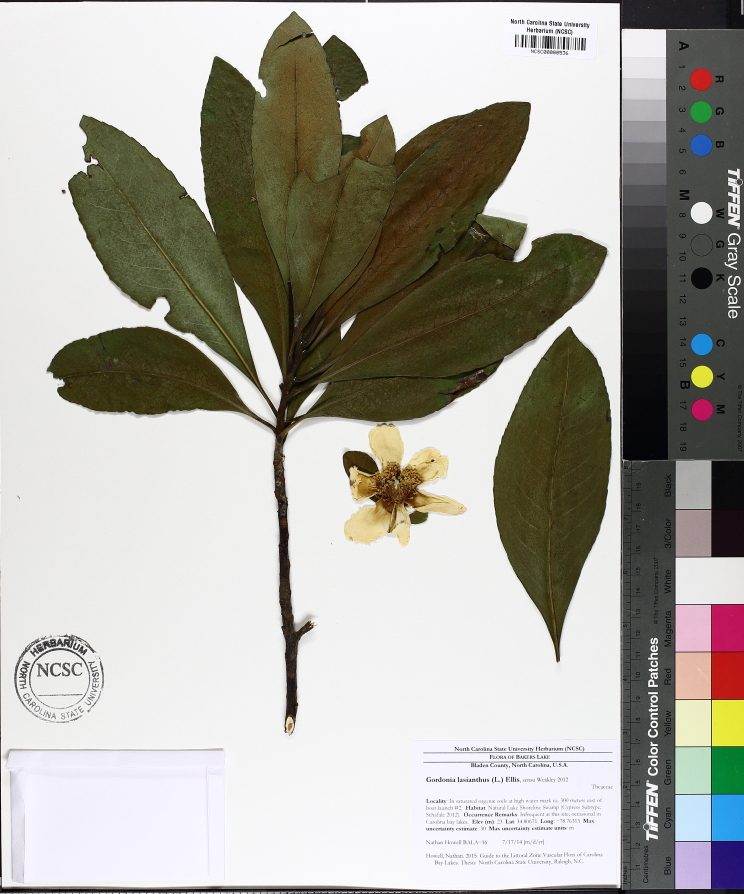
Specimen: *Howell BALA-16* (NCSC)

**Figure 205b.**
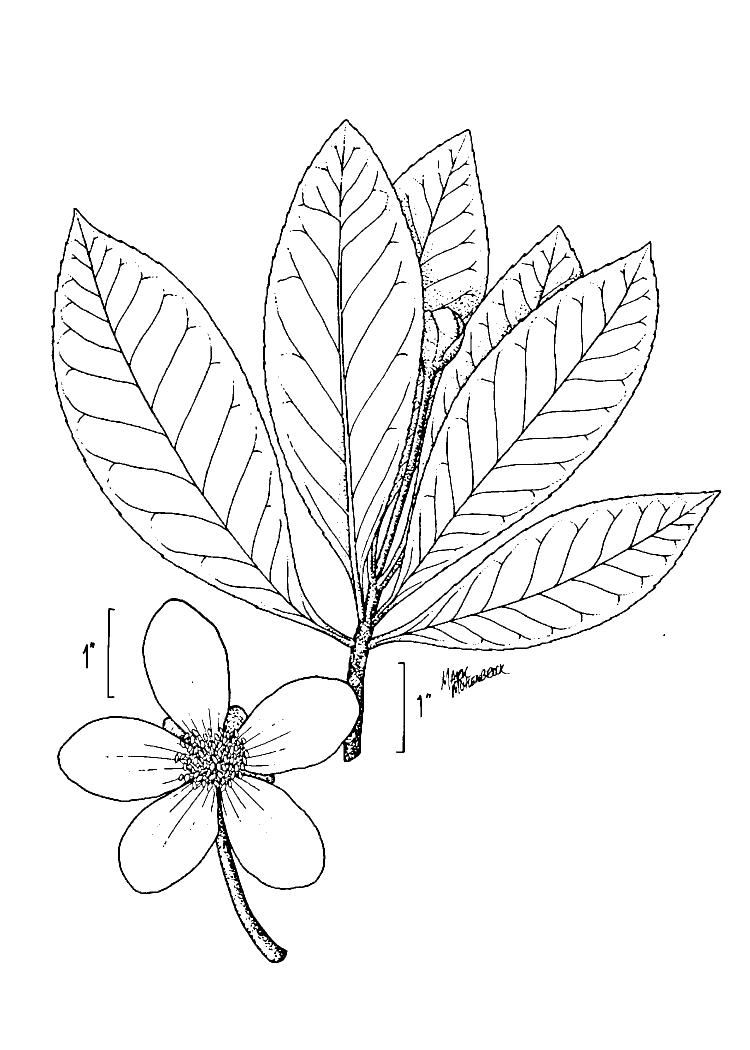
Illustration

**Figure 205c.**
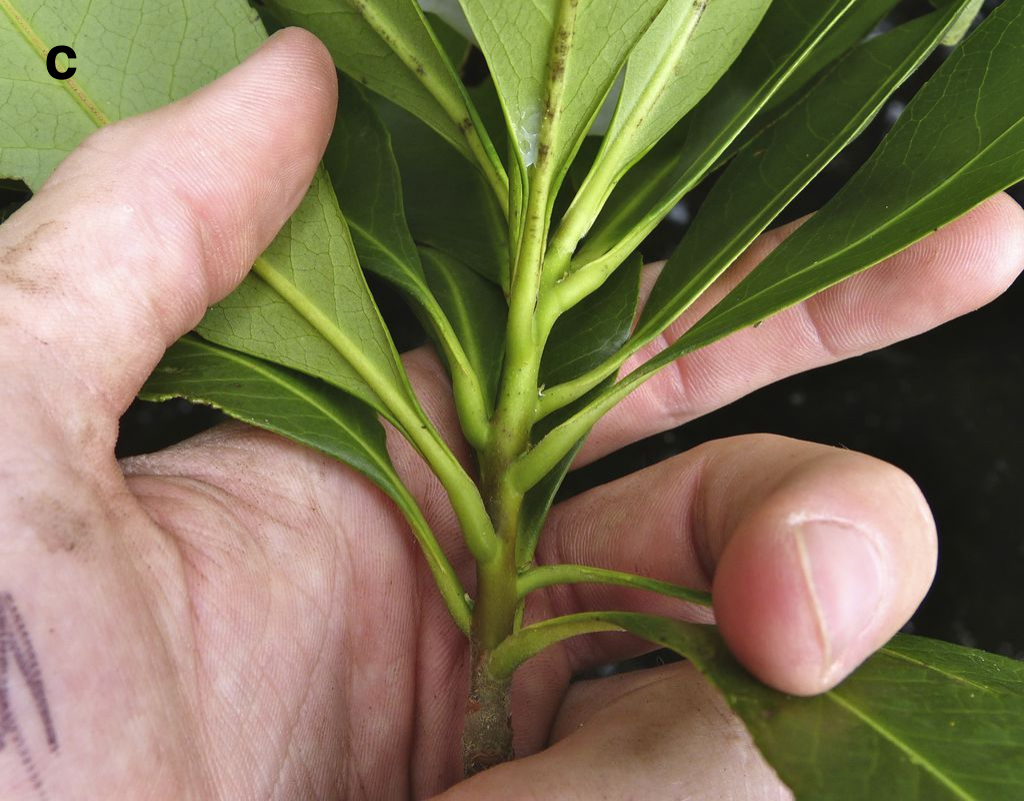
Twig and leaves

**Figure 205d.**
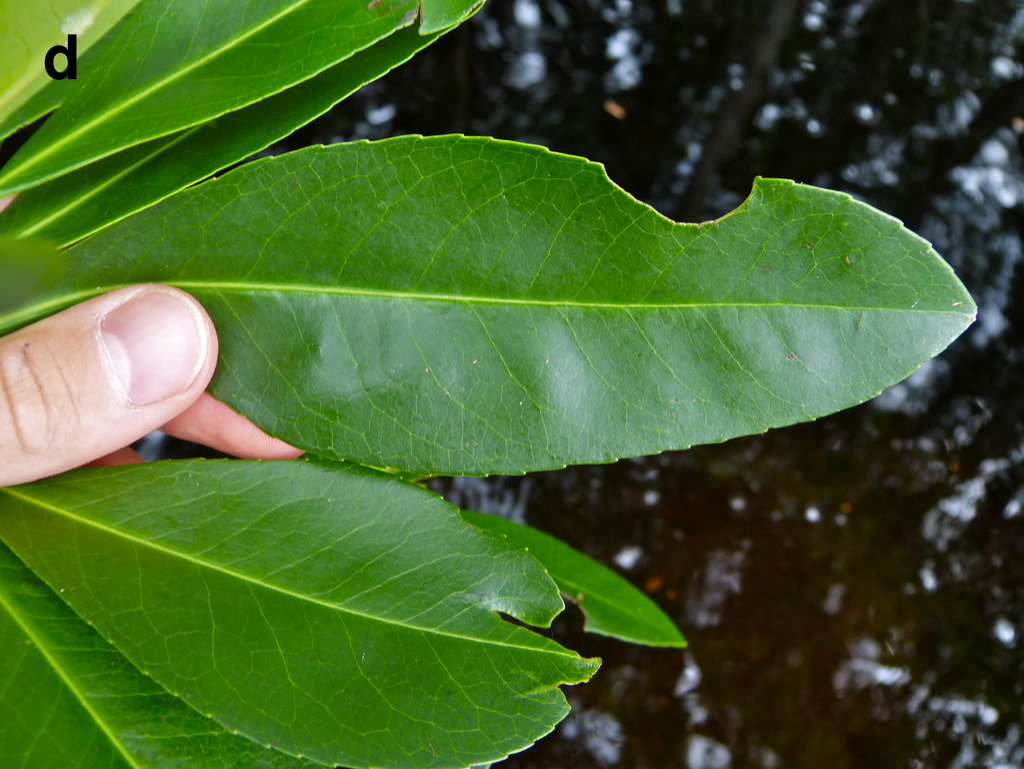
Leaves

**Figure 205e.**
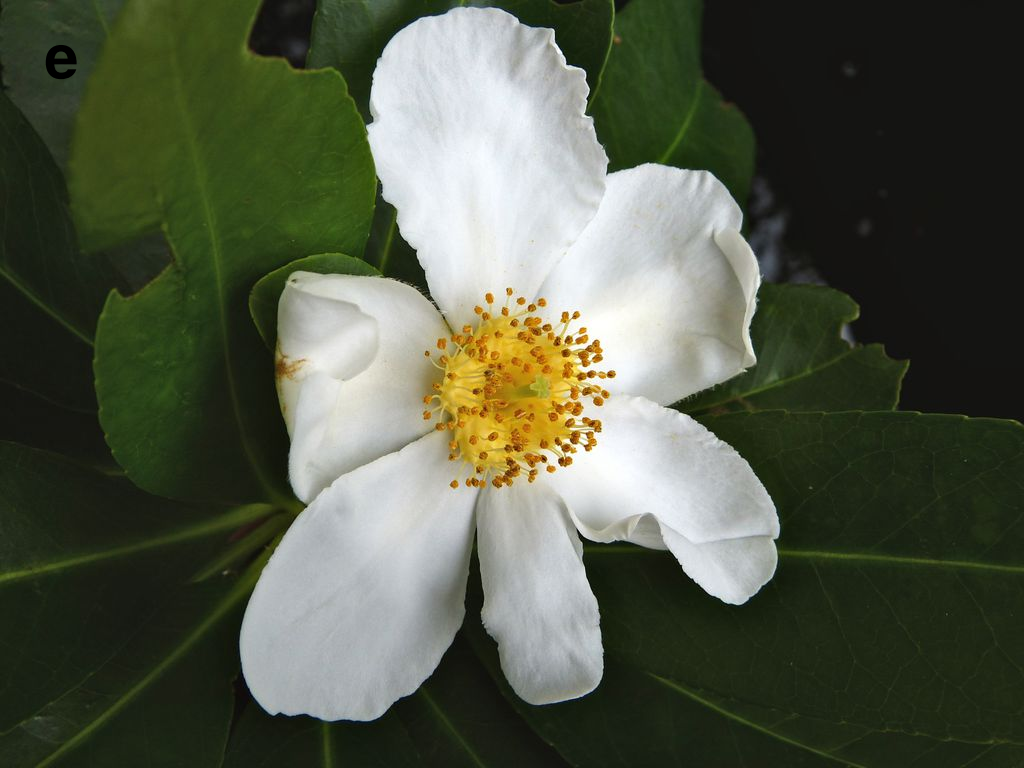
Flower

**Figure 205f.**
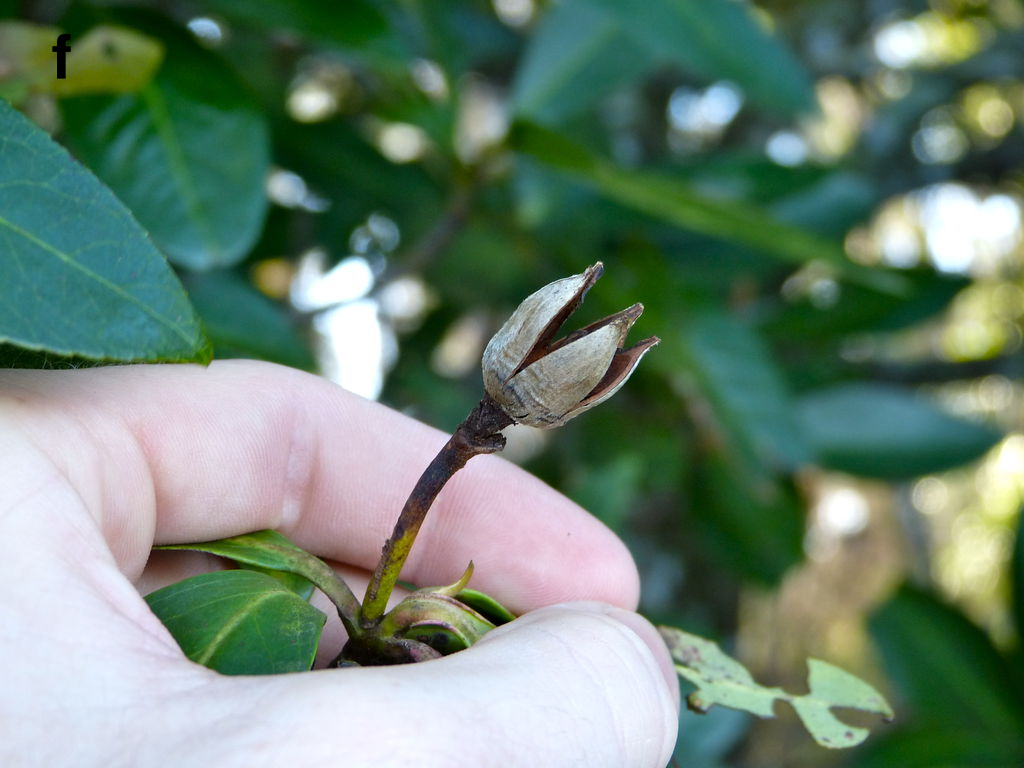
Fruit

**Figure 206a.**
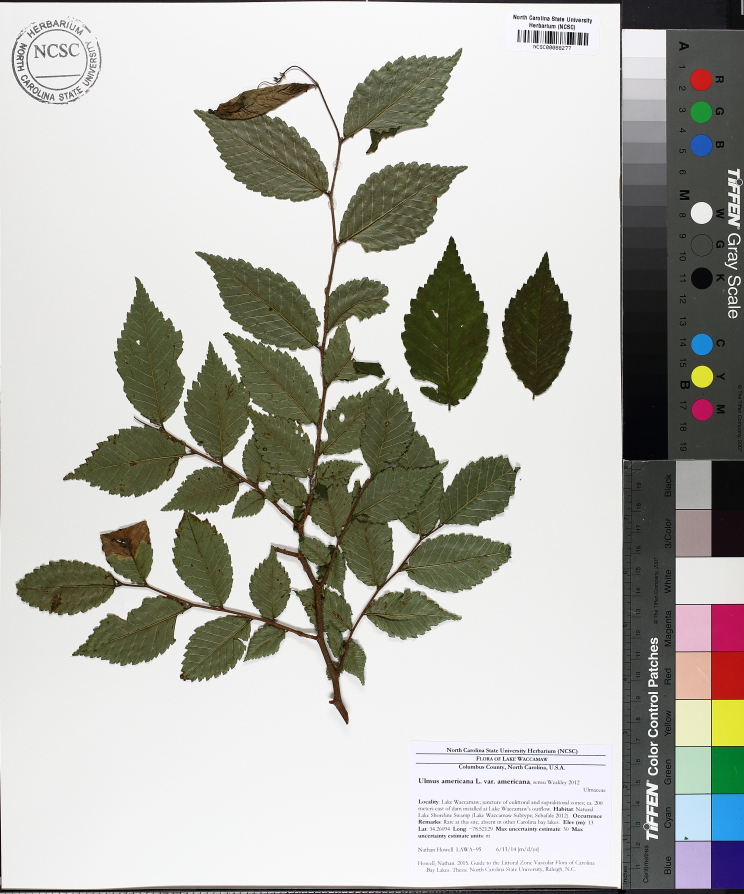
Specimen: *Howell LAWA-95* (NCSC)

**Figure 206b.**
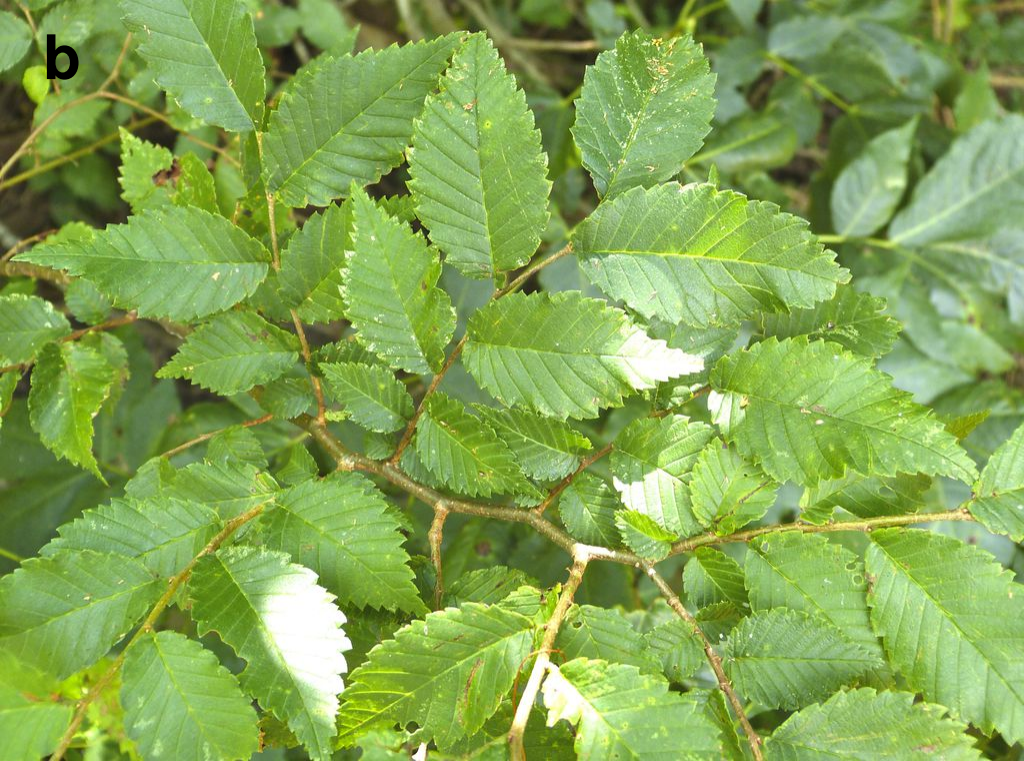
Leaves

**Figure 207a.**
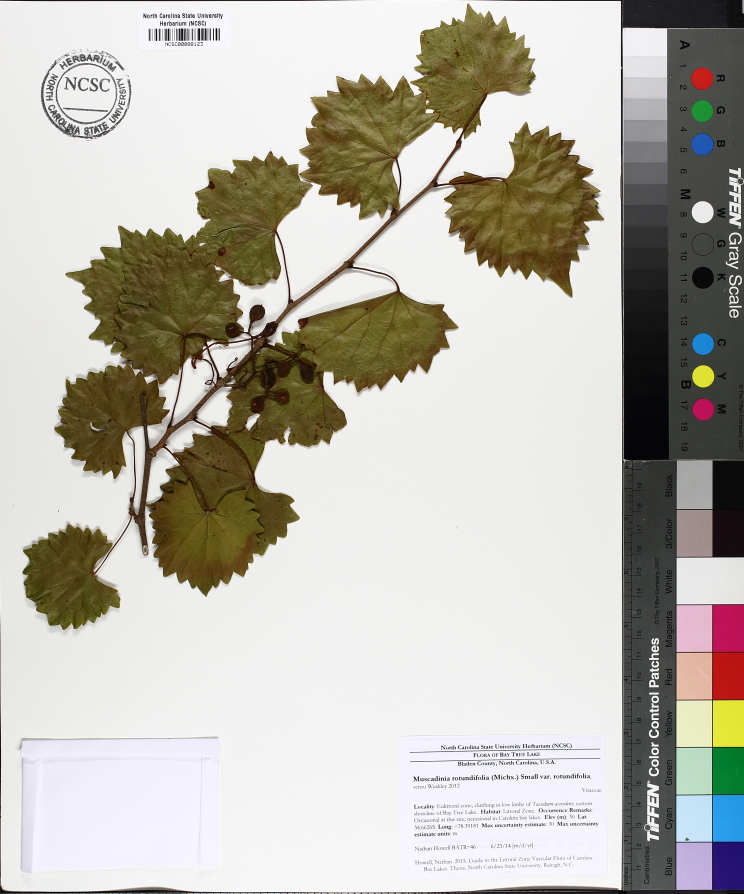
Specimen: *Howell BATR-46* (NCSC)

**Figure 207b.**
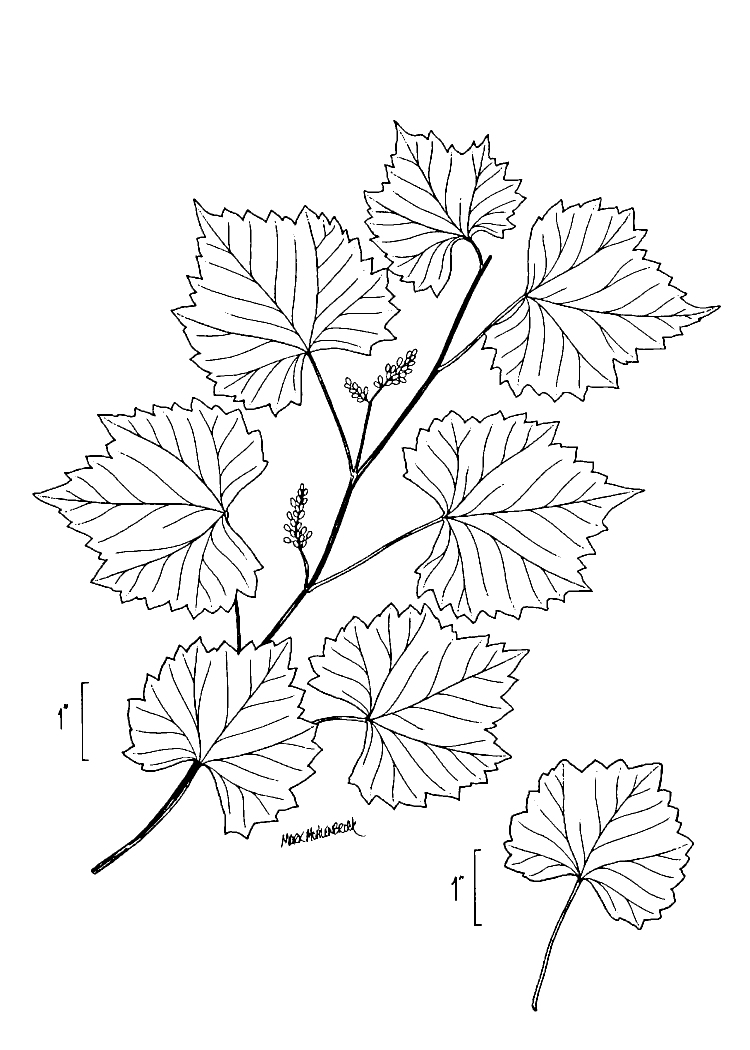
Illustration

**Figure 207c.**
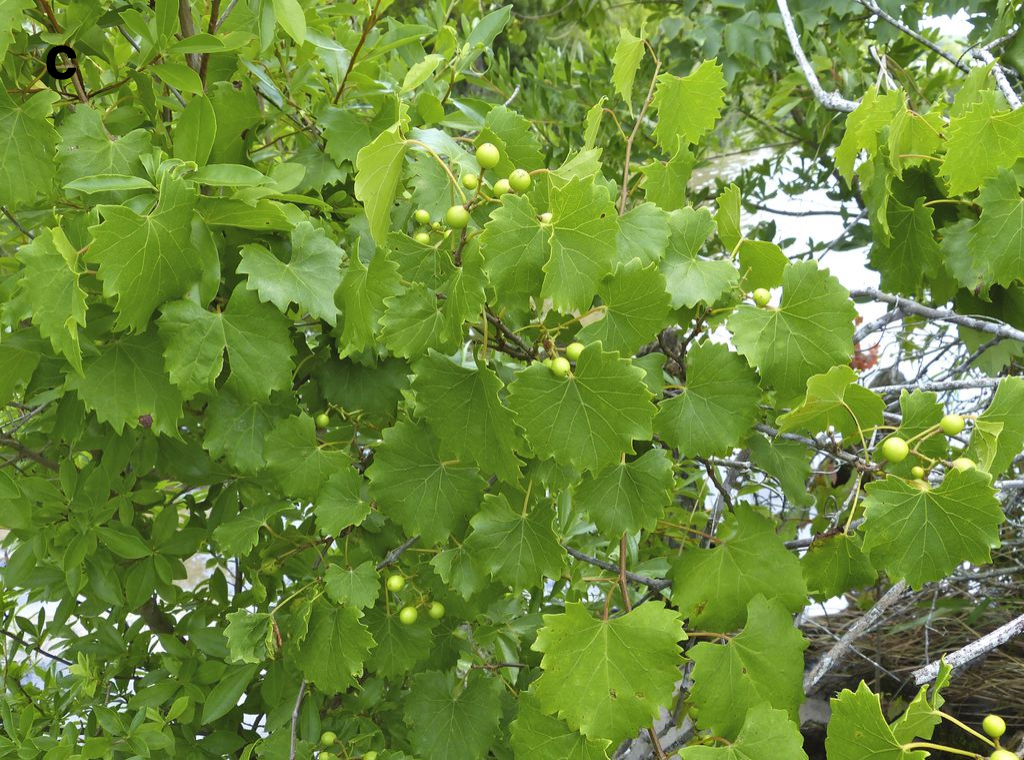
Habit

**Figure 207d.**
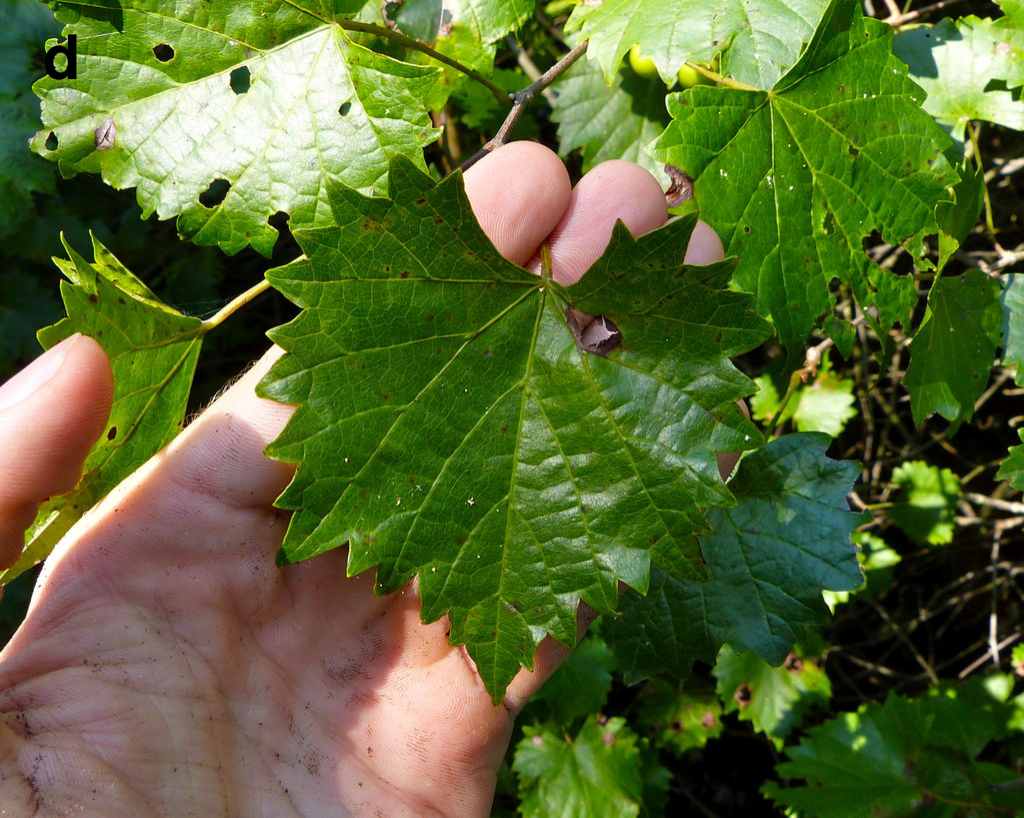
Leaf

**Figure 207e.**
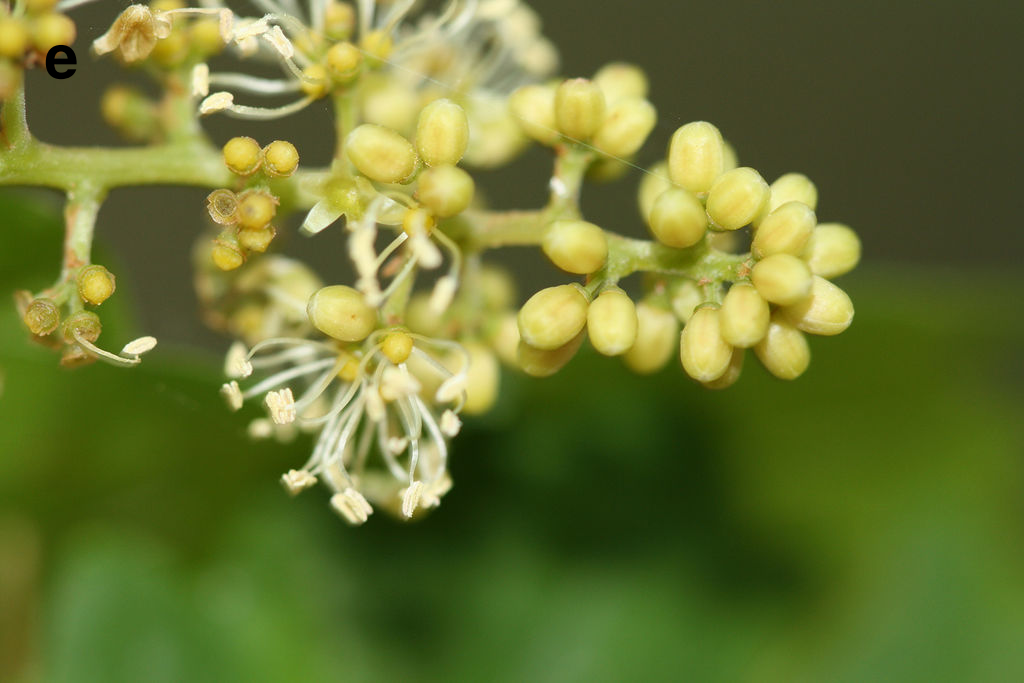
Flowers

**Figure 207f.**
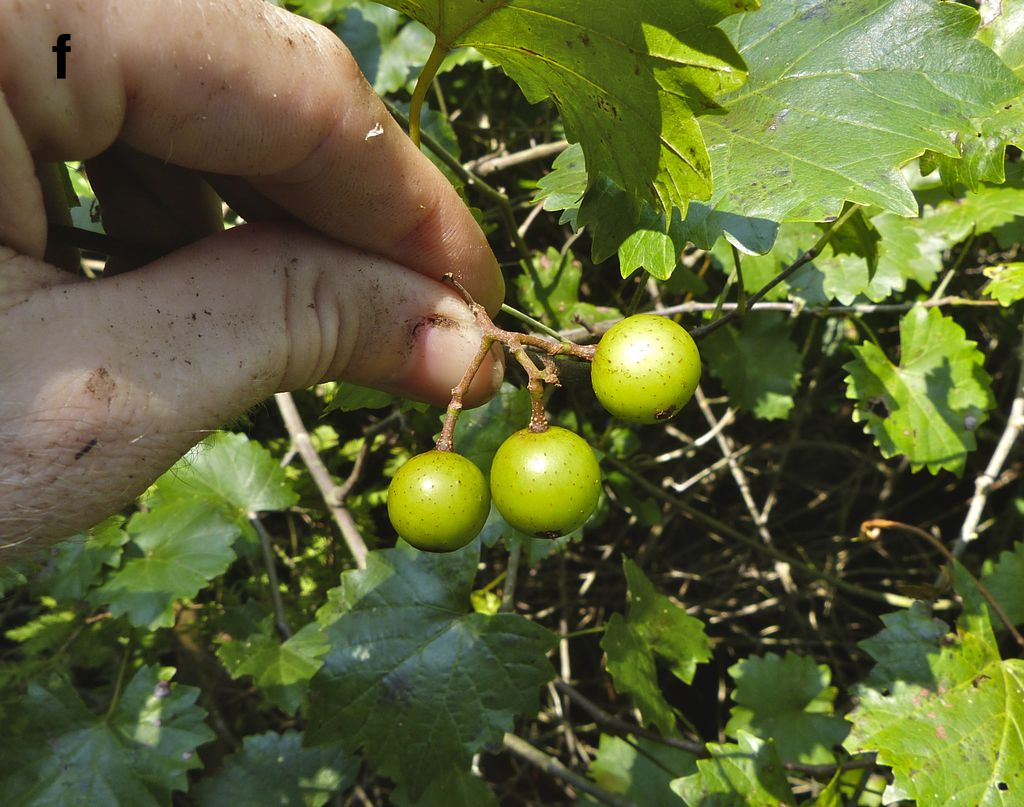
Fruits

**Figure 208a.**
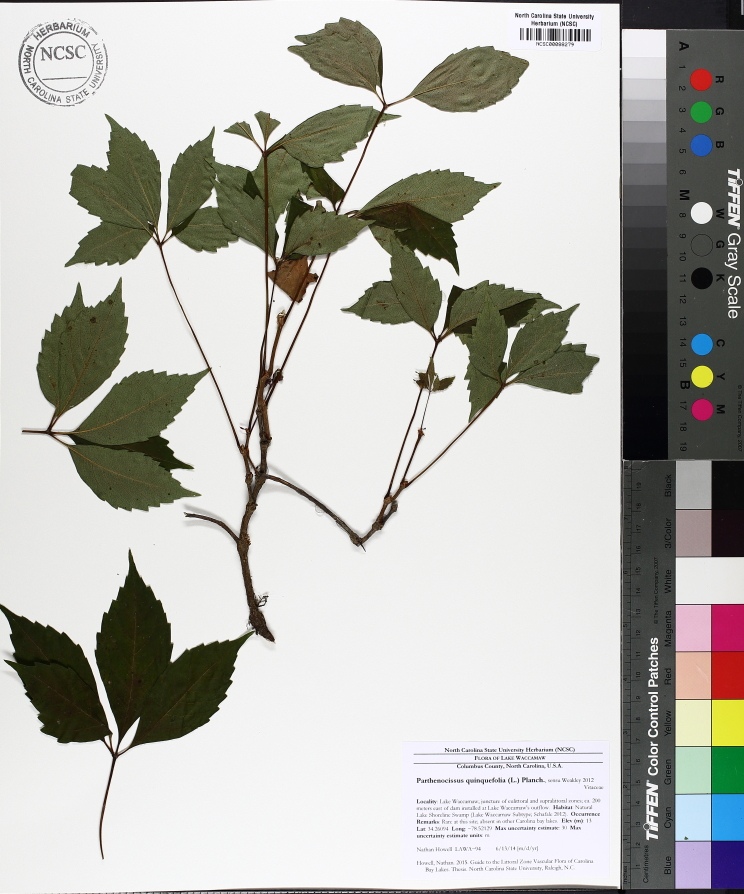
Specimen: *Howell LAWA-94* (NCSC)

**Figure 208b.**
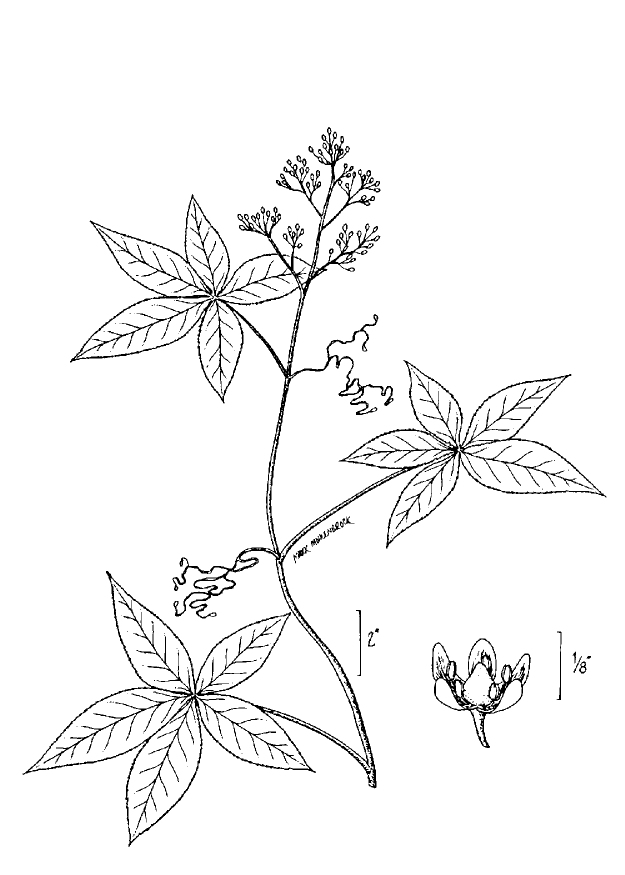
Illustration

**Figure 208c.**
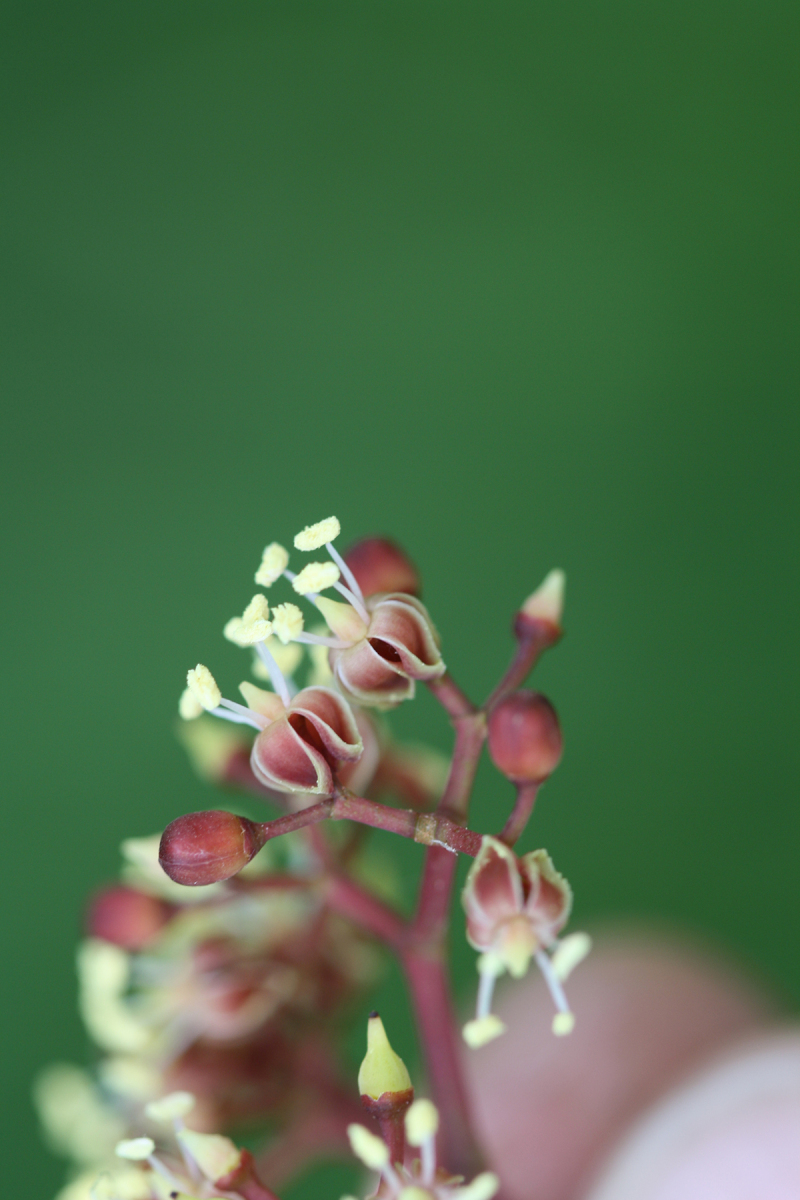
Flowers

**Figure 208d.**
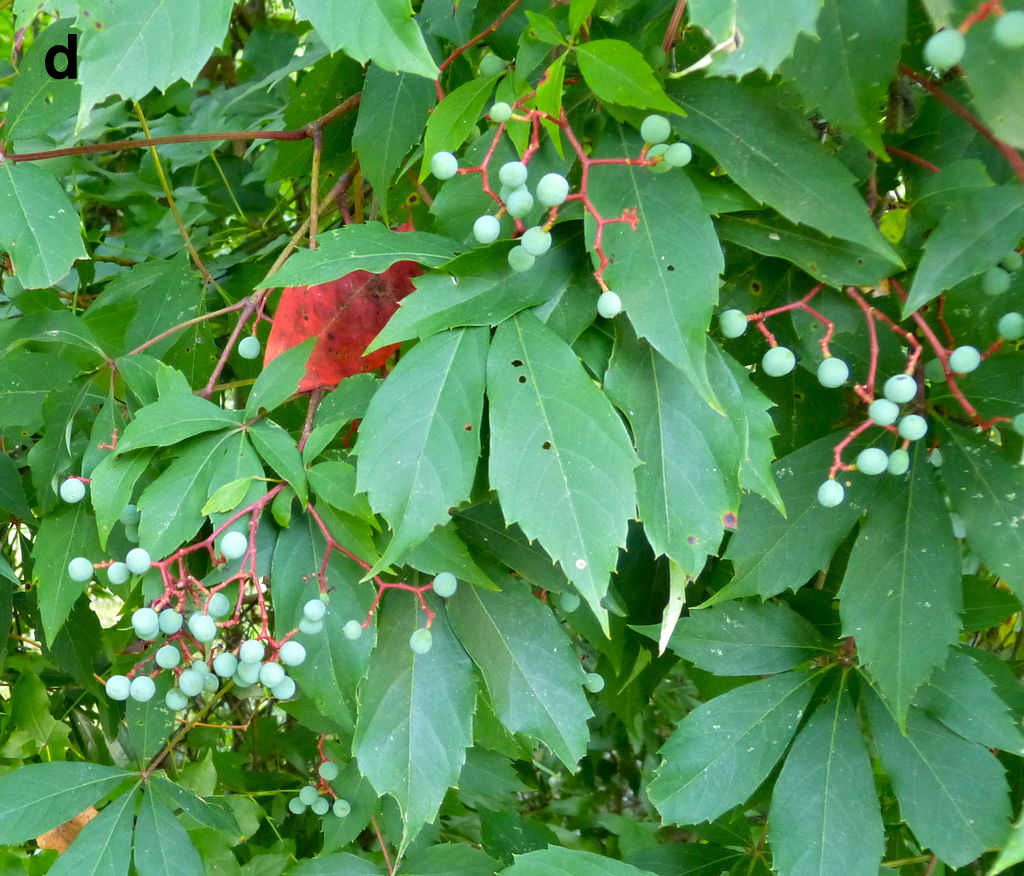
Fruits

**Figure 209.**
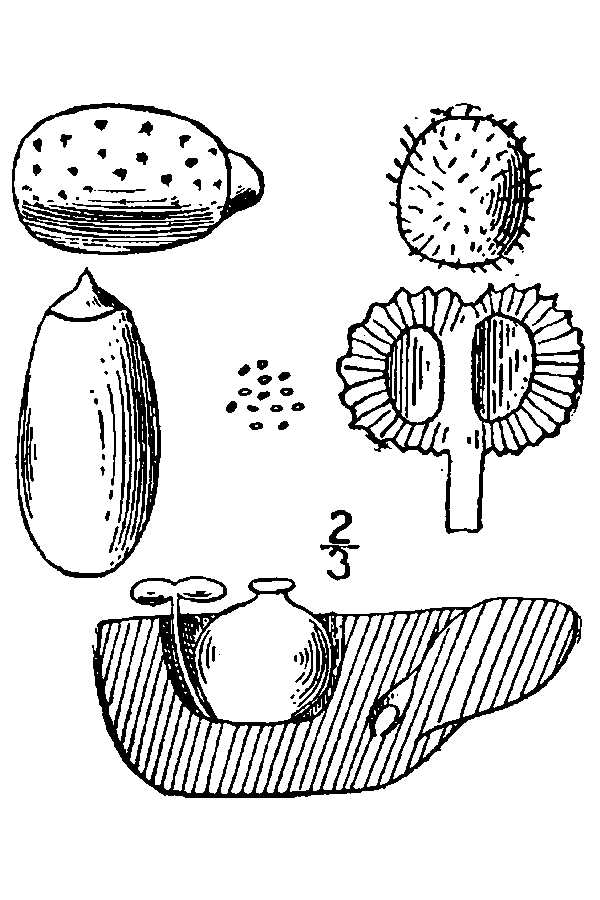
*Wolffia brasiliensis* (from Britton and Brown 1913)

**Figure 210a.**
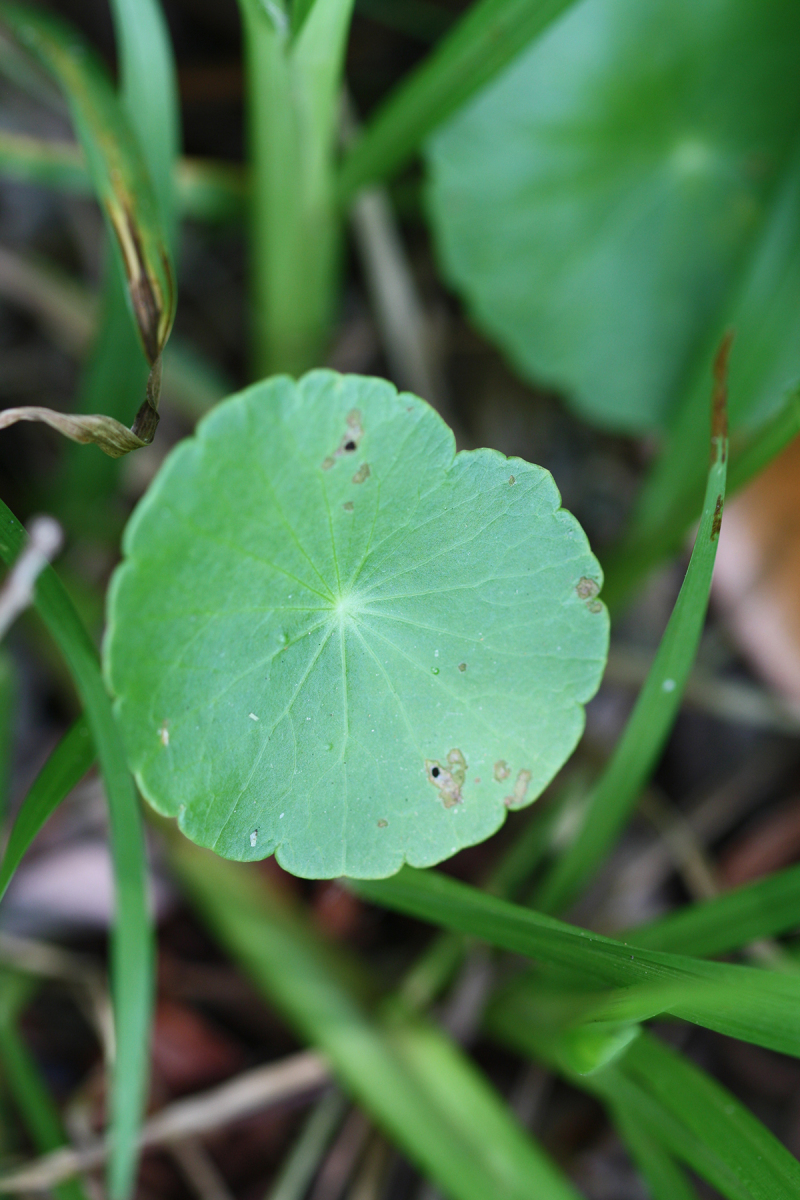
Peltate leaf

**Figure 210b.**
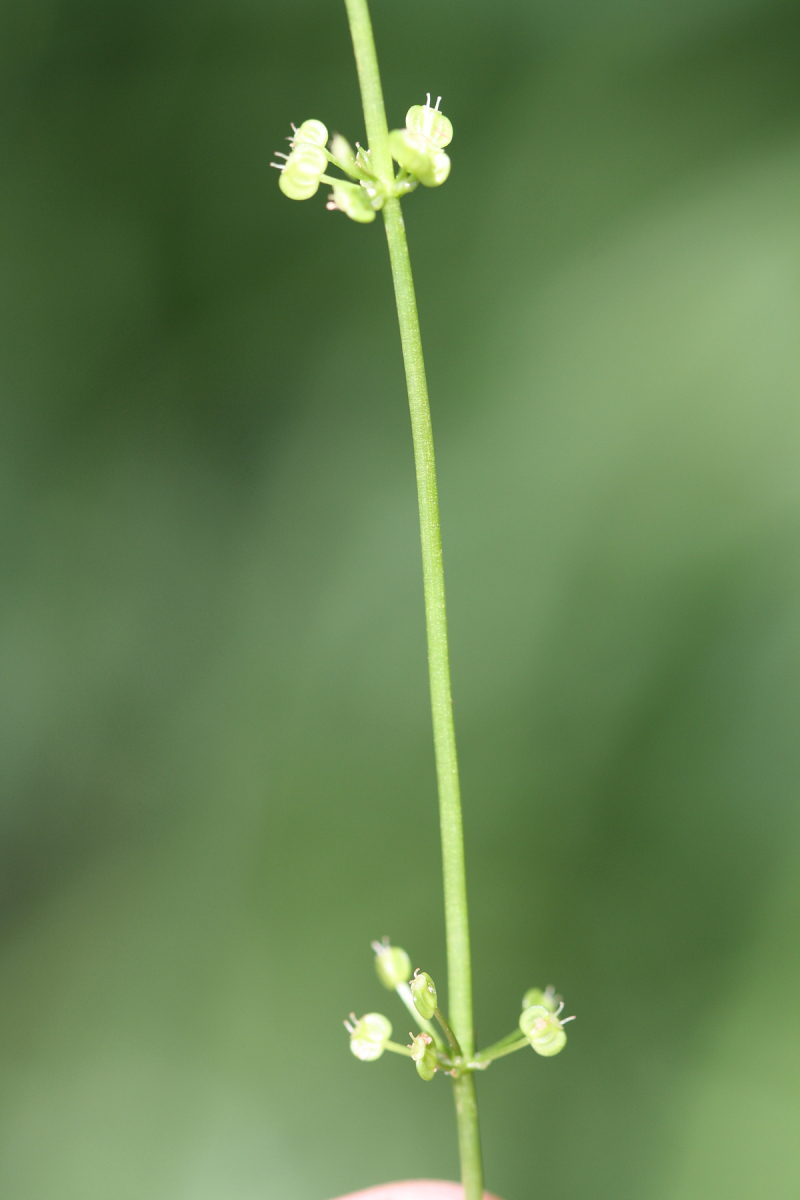
Infructescence

**Figure 210c.**
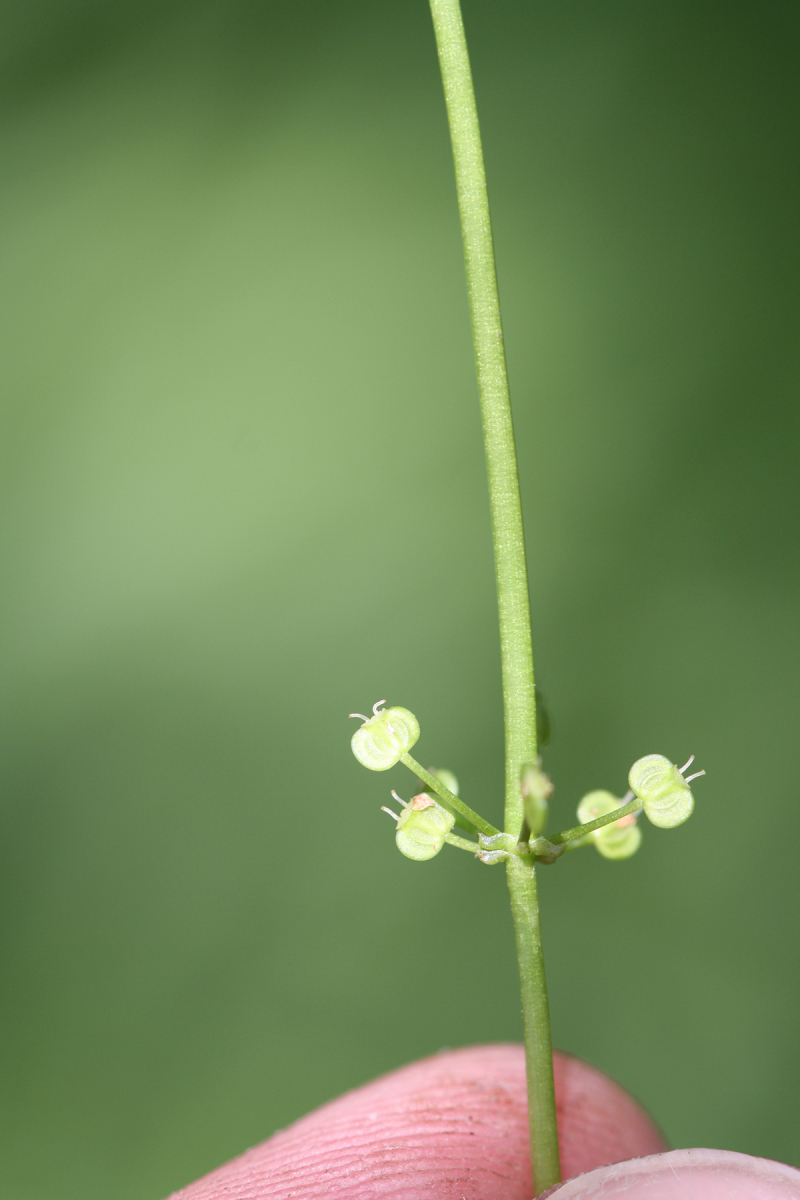
Infructescence

**Figure 210d.**
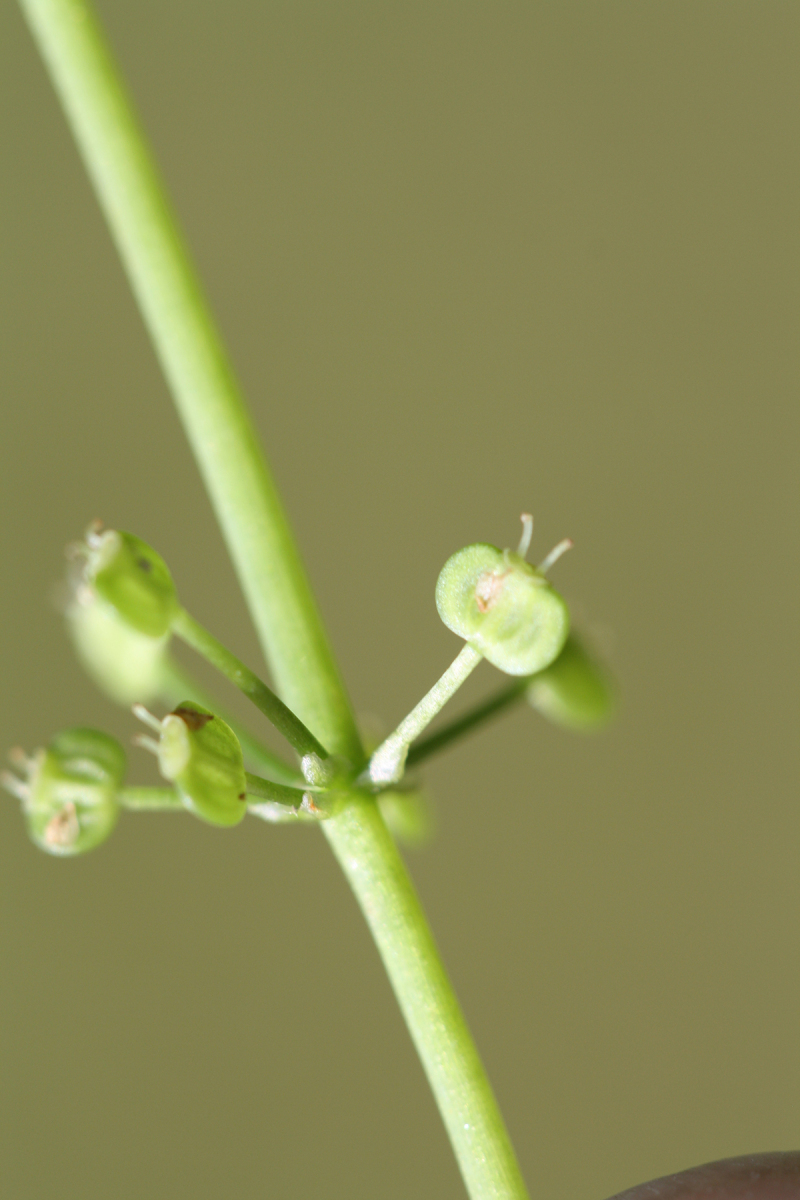
Infructescence

**Figure 211a.**
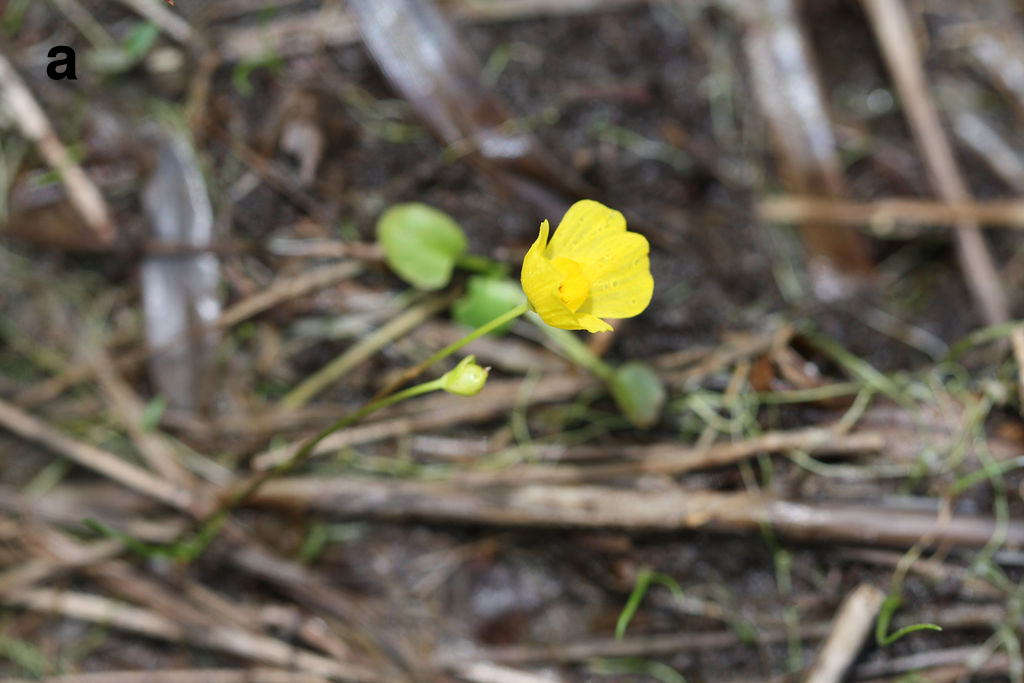
Flower

**Figure 211b.**
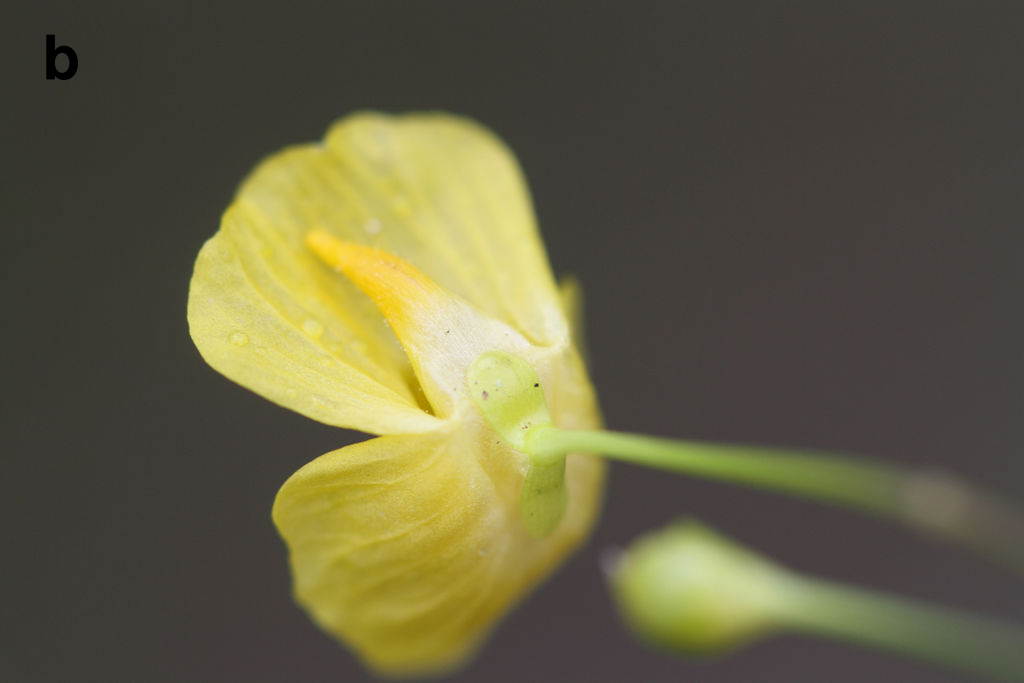
Flower, showing petal exceeding spur

**Table 1.** Water Quality Data for Bay Tree Lake (Bladen County, North Carolina). Frey (1949) sampled Bay Tree Lake 6 times during the Summer and Fall of 1947. Weiss and Kuenzler (1976) sampled Bay Tree Lake twice in 1974 (March 22 and June 6) and 4 times in 1975 (April 7, June 10, August 5, October 6). North Carolina Division of Water Quality, Environmental Sciences Section, Intensive Survey Unit (2009) (DWQ) sampled Bay Tree Lake 4 times in 2008 (June 24, July 29, August 18, October 2). Value ranges have been provided where applicable to show variability. Units are as follows: km^2 ^ = squared kilometers, ha = hectares, km = kilometers, m = meters, °C = degrees celsius, mg/L – milligrams per liter, meq/L = milliequivalents per liter, s.u. = standard units, μg/L = micrograms per liter.

	**Frey (1949)**	**Weiss & Kuenzler (1976)**	**DWQ 2009**
**Trophic Status**	−	−	Dystrophic
**Watershed Area (km^2^)**	−	−	10.36
**Surface Area (ha)**	−	573.84	−
**Max Width (km)**	−	1.77	−
**Max Length (km)**	−	3.05	−
**Max Depth (m)**	−	1.83	−
**Mean Depth (m)**	−	−	0.9
**Secchi Depth (m)**	0.55	0.3−0.4	1.4−1.8
**Min Temp. (°C)**	−	13.3	23.2
**Max Temp. (°C)**	−	30.5	30
**Dissolved Oxygen (mg/L)**	6.4	7.1−10.9	6.8−8
**Alkalinity (meq/L)**	−	0.159−0.231	−
**pH (s.u.)**	4.4	6.3−7.1	4.1−4.5
**Total N (mg/L)**	−	0.48−1.568	−
**Total P (mg/L)**	−	0.13−0.238	−
**Chlorophyll-A (μg/L)**	−	−	2−6

**Table 2.** Water Quality Data for Jones Lake (Bladen County, North Carolina). Frey (1949) sampled Jones Lake 9 times during the Summer and Fall of 1947. Weiss and Kuenzler (1976) sampled Jones Lake twice in 1974 (March 22 and June 6) and 4 times in 1975 (April 7, June 10, August 5, October 6). North Carolina Division of Water Quality, Environmental Sciences Section, Intensive Survey Unit 2009 (DWQ) sampled Jones Lake 5 times in 2008 (May 29, June 25, July 15, September 10, September 24). Value ranges have been provided where applicable to show variability. Units are as follows: km^2 ^ = squared kilometers, ha = hectares, km = kilometers, m = meters, °C = degrees celsius, mg/L – milligrams per liter, meq/L = milliequivalents per liter, s.u. = standard units, μg/L = micrograms per liter.

	**Frey (1949)**	**Weiss & Kuenzler (1976)**	**DWQ 2009**
**Trophic Status**	−	−	Dystrophic
**Watershed Area (km^2^)**	−	−	5.18
**Surface Area (ha)**	−	90.65	−
**Max Width (km)**	−	0.48	−
**Max Length (km)**	−	0.80	−
**Max Depth (m)**	−	2.13	−
**Mean Depth (m)**	−	−	0.9
**Secchi Depth (m)**	0.73	0.3−1.22	1.3−2.4
**Min Temp. (°C)**	−	14	21.9
**Max Temp. (°C)**	−	30.5	29.6
**Dissolved Oxygen (mg/L)**	5.7	6.7−10.6	6.2−7.5
**Alkalinity (meq/L)**	−	0−0.002	−
**pH (s.u.)**	4.34	3.1−4.8	3.6−4.2
**Total N (mg/L)**	−	0.32−0.73	−
**Total P (mg/L)**	−	0.013−0.025	−
**Chlorophyll-A (μg/L)**	−	−	1−11

**Table 3.** Water Quality Data for Lake Waccamaw (Columbus County, North Carolina). Frey (1949) sampled Lake Waccamaw 8 times during the Summer and Fall of 1947. Weiss and Kuenzler (1976) sampled Lake Waccamaw twice in 1974 (March 22 and June 6) and 4 times in 1975 (April 7, June 10, August 5, October 6). North Carolina Division of Water Quality, Environmental Sciences Section, Intensive Survey Unit (2012) (DWQ) sampled Lake Waccamaw 5 times in 2011 (May 4, June 8, September 1, July 20, and August 17). Value ranges have been provided where applicable to show variability. Units are as follows: km^2 ^ = squared kilometers, ha = hectares, km = kilometers, m = meters, °C = degrees celsius, mg/L – milligrams per liter, meq/L = milliequivalents per liter, s.u. = standard units, μg/L = micrograms per liter.

	**Frey (1949)**	**Weiss & Kuenzler (1976)**	**DWQ 2012**
**Trophic Status**	−	−	Mesotrophic
**Watershed Area (km^2^)**	−	−	181.29
**Surface Area (ha)**	−	3617.08	−
**Max Width (km)**	−	5.47	−
**Max Length (km)**	−	8.36	−
**Max Depth (m)**	−	3.35	−
**Mean Depth (m)**	−	−	1.5
**Secchi Depth (m)**	1.34	0.61−2.38	1.1−1.9
**Min Temp. (°C)**	−	14	23.5
**Max Temp. (°C)**	−	31.5	29.9
**Dissolved Oxygen (mg/L)**	5.2	7.8−11	6.9−8.1
**Alkalinity (meq/L)**	−	0.14−0.24	−
**pH (s.u.)**	6.95	6.8−7.5	7.0−8.5
**Total N (mg/L)**	−	0.297−1.56	−
**Total P (mg/L)**	−	0.017 − .055	−
**Chlorophyll-A (μg/L)**	−	−	2.8−8

**Table 4.** Water Quality Data for Salters Lake (Bladen County, North Carolina). Frey (1949) sampled Salters Lake 7 times during the Summer and Fall of 1947. Weiss and Kuenzler (1976) sampled Salters Lake twice in 1974 (March 22 and June 6). North Carolina Division of Water Quality, Environmental Sciences Section, Intensive Survey Unit 2009 (DWQ) sampled Salters Lake 4 times in 2008 (June 25, July 15, August 20, September 24). Value ranges have been provided where applicable to show variability. Units are as follows: km^2 ^ = squared kilometers, ha = hectares, km = kilometers, m = meters, °C = degrees celsius, mg/L – milligrams per liter, meq/L = milliequivalents per liter, s.u. = standard units, μg/L = micrograms per liter.

	**Frey (1949)**	**Weiss & Kuenzler (1976)**	**DWQ 2009**
**Trophic Status**	−	−	Dystrophic
**Watershed Area (km^2^)**	−	−	7.77
**Surface Area (ha)**	−	127.47	−
**Max Width (km)**	−	0.80	−
**Max Length (km)**	−	1.12	−
**Max Depth (m)**	−	1.82	−
**Mean Depth (m)**	−	−	2.13
**Secchi Depth (m)**	0.55	0.6−0.91	−
**Min Temp. (°C)**	−	15	21.7
**Max Temp. (°C)**	−	25.4	31.2
**Dissolved Oxygen (mg/L)**	6	7.9−10.1	6.5 – 8.1
**Alkalinity (meq/L)**	−	0.0019	−
**pH (s.u.)**	4.49	4.1−4.8	3.6 – 4.1
**Total N (mg/L)**	−	0.293−0.374	−
**Total P (mg/L)**	−	0.015−0.016	−
**Chlorophyll-A (μg/L)**	−	−	4.7 – 26

**Table 5.** Water Quality Data for Singletary Lake (Bladen County, North Carolina). Frey (1949) sampled Singletary Lake 10 times during the Summer and Fall of 1947. Weiss and Kuenzler (1976) sampled Singletary Lake twice in 1974 (March 22 and June 6) and four times in 1975 (April 7, June 10, August 5, October 6). North Carolina Division of Water Quality, Environmental Sciences Section, Intensive Survey Unit (2009) (DWQ) sampled Singletary Lake 5 times in 2008 (June 25, July 15, August 20, September 24). Value ranges have been provided where applicable to show variability. Units are as follows: km^2 ^ = squared kilometers, ha = hectares, km = kilometers, m = meters, °C = degrees celsius, mg/L – milligrams per liter, meq/L = milliequivalents per liter, s.u. = standard units, μg/L = micrograms per liter.

	**Frey (1949)**	**Weiss & Kuenzler (1976)**	**DWQ 2009**
**Trophic Status**	−	−	Dystrophic
**Watershed Area (km^2^)**	−	−	5.18
**Surface Area (ha)**	−	231.48	−
**Max Width (km)**	−	0.64	−
**Max Length (km)**	−	2.09	−
**Max Depth (m)**	−	2.74	−
**Mean Depth (m)**	−	−	2.13
**Secchi Depth (m)**	0.76	0.48−1.21	0.6−1
**Min Temp. (°C)**	−	13.8	24.8
**Max Temp. (°C)**	−	31	30.6
**Dissolved Oxygen (mg/L)**	6.6	7.3−11.2	6−7.8
**Alkalinity (meq/L)**	−	0.0019	−
**pH (s.u.)**	4.5	3.2−4.6	3.9−4.2
**Total N (mg/L)**	−	0.255−0.515	−
**Total P (mg/L)**	−	0.018−0.075	−
**Chlorophyll-A (μg/L)**	−	−	4.8−44

**Table 6.** Water Quality Data for White Lake (Bladen County, North Carolina). Frey (1949) sampled White Lake 8 times during the Summer and Fall of 1947. Weiss and Kuenzler (1976) sampled White Lake twice in 1974 (March 22 and June 6). North Carolina Division of Water Quality, Environmental Sciences Section, Intensive Survey Unit (2009) (DWQ) sampled White Lake 5 times in 2008 (May 27, June 24, July 29, August 11, and October 2). Value ranges have been provided where applicable to show variability. Units are as follows: km^2 ^ = squared kilometers, ha = hectares, km = kilometers, m = meters, °C = degrees celsius, mg/L – milligrams per liter, meq/L = milliequivalents per liter, s.u. = standard units, μg/L = micrograms per liter.

	**Frey (1949)**	**Weiss & Kuenzler (1976)**	**DWQ 2009**
**Trophic Status**	−	−	Oligotrophic
**Watershed Area (mi^2^)**	−	−	−
**Surface Area (ha)**	−	432.2	−
**Max Width (km)**	−	1.61	−
**Max Length (km)**	−	2.57	−
**Max Depth (m)**	−	3.35	−
**Mean Depth (m)**	−	−	3.04
**Secchi Depth (m)**	3.35	3.35	3.35
**Min Temp. (°C)**	−	15.1	22.3
**Max Temp. (°C)**	−	26.1	30.1
**Dissolved Oxygen (mg/L)**	6.7	8.6−10.1	6.8−8.2
**Alkalinity (meq/L)**	−	0.0019−.0099	−
**pH (s.u.)**	4.92	4.6−4.8	4.6−5.2
**Total N (mg/L)**	−	0.123−0.211	−
**Total P (mg/L)**	−	0.010−0.017	−
**Chlorophyll-A (μg/L)**	−	−	4.8−44

**Table 7.** Plant community types occurring within the littoral zone of Carolina bay lakes. Community types follow Schafale (2012); rank designations follow Robinson and Finnegan (2014). Community types are presented in order of increasing species richness. The Natural Lake Shoreline Marsh (Lake Waccamaw Pondlily Subtype) typically supports a couple of dominant taxa (i.e., *Nuphar sagittifolia* and *Eriocaulon aquaticum*) with several other co-dominants. The Natural Lake Shoreline Swamp (Lake Waccamaw Subtype) is known to contain 140+ taxa.

**Species** **Richness**	**Plant Community Types**	**State** **Rank**	**Global** **Rank**
Lowest Highest	Natural Lake Shoreline Marsh (Lake Waccamaw Pondlily Subtype)	S1	G1
	Coastal Plain Semipermanent Impoundment	S4	G4G5
	floating Bog	S1	G1?
	Natural Lake Shoreline Swamp (Cypress Subtype)	S2	G3
	Natural Lake Shoreline Marsh (Typic Subtype)	S1	G1
	Natural Lake Shoreline Swamp (Lake Waccamaw Subtype)	S1	G1
**S1** = Critically Imperiled, 1–5 occurrences in state; **S2** = Imperiled, 6–20 occurrences in state; **S4** = Apparently Secure, 101–1000 occurrences in state; **G1** = Critically Imperiled, 1–5 occurrences in the world; **G3** = Vulnerable, 21–100 occurrences in the world; **G4** = Apparently Secure, 101–1000 occurrences in the world; **G5** = Secure, 1001+ occurrences in the world.			

**Table 8.** Summary of vascular plant taxa collected or reported from Carolina bay lake littoral zones

			**Species and Subspecies/Varieties**		
**Group**	**Families**	**Genera**	**Native**	**Exotic**	**Total**
**Basal Angiosperms & Magnoliids**	4	6	6	0	6
**Pteridophytes**	6	7	7	0	7
**Gymnosperms**	2	3	5	0	5
**Monocotyledons**	17	41	84	2	86
**Eudicotyledons**	51	79	98	3	101
**Total**	80	136	200	5	205

**Table 9.** List of North Carolina Significantly Rare and Watch List taxa collected or reported from Carolina bay lake littoral zones. Status and rank designations follow Robinson and Finnegan (2014). Taxa for which voucher specimens have been collected (by the first author or others) are indicated with a check mark (✓) in the second column. The taxonomy followed in this work and that of Robinson and Finnegan (2014) differ in one instance in the following table: Luziola fluitans (Michx.) Terrell & H. Rob. var. fluitans (as *Luziola fluitans* (Michx.) Terrell & H. Rob. sensu Robinson and Finnegan 2014). See Martínez-y-Pérez et al. (2008) in addition to the FNA treatment for reasons of further division to an infraspecific rank.

	**Taxon**	**Vouchered ?**	**State Status**	**Fed. Status**	**State Rank**	**Global Rank**
	**Significantly Rare:**					
1	*Bacopa caroliniana* (Walter) B.L. Rob.	✓	T	−	S1	G4G5
2	Boltonia asteroides (L.) L’Hér var. glastifolia (Hill) Fernald	✓	SR−O	−	S2	G5TNR
3	*Cladium mariscoides* (Muhl) Torr.	✓	SR−O	−	S3	G5
4	*Eleocharis vivipara* Link	✓	E	−	S1	G5
5	*Epidendrum magnoliae* Muhl.	✓	T	−	S1S2	G4
6	*Eriocaulon aquaticum* (Hill) Druce	✓	SC−V	−	S2	G5
7	*Ludwigia brevipes* (Long) Eames	✓	SR−T	FSC	S1S2	G2G3
8	*Ludwigia sphaerocarpa* Elliott	✓	E	−	S1	G5
9	Luziola fluitans (Michx.) Terrell & H. Rob. var. fluitans	✓	SR−P	−	S2	G4,G5
10	*Lycopus angustifolius* Elliott	✓	SR−P	−	S1	G4?Q
11	*Rhexia aristosa* Britton		SC−V	FSC	S3	G3,G4
12	*Rhynchospora alba* (L.) Vahl	✓	SR−P	−	S2	G5
13	*Sagittaria filiformis* J.G. Sm.	✓	SR−P	−	SH	G4,G5
14	*Sagittaria isoetiformis* J.G. Sm.	✓	T	−	S2	G4?
15	*Sagittaria weatherbiana* Fernald	✓	E	FSC	S2	G3G4
16	*Sclerolepis uniflora* (Walter) Britton, Sterns & Poggenb.	✓	SR−T	−	S2	G4
17	*Spiranthes laciniata* (Small) Ames	✓	SC−V	−	S2	G4,G5
18	*Utricularia cornuta* Michx.	✓	T	−	S1S2	G5
19	*Utricularia resupinata* B.D. Greene ex Bigelow	✓	E	−	S1	G4
	**Watch List:**					
1	Dichanthelium dichotomum (L.) Gould var. roanokense (Ashe) LeBlond	✓	W1	−	S2	G5T4?
2	*Dichanthelium erectifolium* (Nash) Gould & C.A. Clark	✓	W1	−	S2	G4
3	*Dryopteris ludoviciana* (Kunze) Small	✓	W1	−	S2	G4
4	*Eleocharis equisetoides* (Elliott) Torr.	✓	W1	−	S3	G4
5	*Habaneria repens* Nutt.		W1	−	S2	G5
6	*Nelumbo lutea* Willd.	✓	W7	−	S2	G4
7	*Nuphar sagittifolia* (Walter) Pursh	✓	W1	FSC	S2	G5T2
8	*Rhexia cubensis* Griseb.	✓	W1	−	S3	G4G5
9	*Rhynchospora inundata* (Oakes) Fernald	✓	W1	−	S3	G4?
10	*Rhynchospora nitens* (Vahl) A. Gray	✓	W1	−	S3	G4?
11	*Xyris iridifolia* Chapm.		W7	−	S2	G4G5T4T
12	*Xyris smalliana* Nash	✓	W1	−	S3	G5
STATE STATUS: **E** = Endangered; **T** = Threatened; **SC-V** = Special Concern-Vulnerable; **SR** = Significantly Rare: **−T** = Throughout; **−P** = Periphery of Range; **−O** = Other; **W =** Watchlist: **W1** = rare but relatively secure; **W7** = rare and poorly known. FEDERAL STATUS: **FSC** = Federal Species of Concern. STATE RANK: **SH** = historical (known only from historical populations in the state); **S1** = Critically Imperiled, 1–5 populations in the state; **S2** = Imperiled, 6–20 populations in the state; **S3** = Vulnerable, 21–100 populations in the state. FEDERAL RANK: **G2** = Imperiled, 6–20 populations in the world; **G3** = Vulnerable, 21–100 populations in the world; **G4** = Apparently Secure, 101–1000 populations in the world; **G5** = Secure, 1001+ populations in the world; **T#** = Global rank of a subspecies or variety; **NR** = Not Ranked; **Q** = Questionable taxonomy; **?** = Uncertain.						

**Table 10.** Sørenson’s Similarity Index for Carolina bay lakes. Values in this table are represented as percentiles (i.e., when looking in the second column from the left under Bakers Lake, Bakers Lake is considered to be 16.4% similar to Bay Tree Lake, 23.5% similar to Horseshoe Lake, and 40.8% similar to Jones Lake). Based solely on littoral zone plant taxa, Jones Lake and Singletary Lake are 83.3% alike.

	**Bakers** **Lake**	**Bay Tree** **Lake**	**Horseshoe** **Lake**	**Jones** **Lake**	**Lake** **Waccamaw**	**Little** **SingletaryLake**	**Salters** **Lake**	**Singletary** **Lake**
Bakers Lake	100	16.4	23.5	40.8	12.4	39.3	41.0	41.5
Bay Tree Lake	16.4	100	37.4	38.6	33.0	46.3	32.4	41.3
Horseshoe Lake	23.5	37.4	100	38.6	26.7	42.2	24.7	48.3
Jones Lake	40.8	38.6	38.5	100	20.5	42.5	55.6	83.3
Lake Waccamaw	12.4	33.0	26.7	20.5	100	22.5	21.3	28.9
Little Singletary Lake	39.3	46.3	42.2	42.5	22.5	100	29.5	56.0
Salters Lake	41.0	32.4	24.7	55.5	21.3	29.5	100	51.7
Singletary Lake	41.5	41.3	48.3	83.3	28.9	56.0	51.7	100

**Table 11.** Number of taxa (species, subspecies, and varieties) by major taxonomic group across study sites. Sites are arranged from taxonomically richest to most depauperate. BALA = Bakers Lake; BATR = Bay Tree Lake; HOLA = Horseshoe Lake; JOLA = Jones Lake; LAWA = Lake Waccamaw; LISI = Little Singletary Lake; SALA = Salters Lake; SILA = Singletary Lake.

	**LAWA**	**BATR**	**HOLA**	**LISI**	**SILA**	**JOLA**	**SALA**	**BALA**
Pteridophytes	7	3	1	2	1	1	1	1
Gymnosperms	2	3	3	3	5	4	2	1
Basal angiosperms	3	--	3	--	1	--	--	--
Magnoliids	2	--	--	1	2	2	2	2
Monocots	60	23	21	17	9	10	5	3
Eudicots	71	27	24	16	18	16	12	11
*Total*	145	56	52	39	36	33	22	18

**Table 12.** Descriptions for estimating the abundance of taxa (adapted from Palmer et al. 1995)

**Density**	**Description**
Abundant	Dominant or co-dominant in one or more communities.
Frequent	Easily seen or found in one or more common communities but not dominant in any common community
Occasional	Widely scattered but not difficult to find
Infrequent	Difficult to find with few individuals or colonies but found in several locations
Rare	Very difficult to find and limited to one or very few locations or uncommon communities
