# Supplementary material for: Guide to the littoral zone vascular flora of Carolina bay lakes (U.S.A.)
Source: Biodivers Data J. 2016 Apr 5;(4):e7964. doi: 10.3897/BDJ.4.e7964 (PMC4911545; doi:10.3897/BDJ.4.e7964)
Supplement: Supplementary material 1 — Carolina bay lakes literature [file biodiversity_data_journal-4-e7964-s001.doc]

Appendix A. Carolina bay lakes literature.

Casterlin, M. E., W.W. Reynolds, D.G. Lindquist, and C.G. Yarbrough. 1984. Algal and

physiochemical indicators of eutrophication in a lake harboring endemic species: Lake Waccamaw, North Carolina. Journal of the Elisha Mitchell Scientific Society 100(3): 83-103.

Eyles, D.E. 1941. A phytosociological study of the *Castalia*-*Myriophyllum* community of Georgia Coastal Plain Boggy Ponds. American Midland Naturalist 26(2): 421−438.

Frey, D.G. 1949. Morphometry and hydrography of some natural lakes of the North Carolina coastal plain: the bay lake as a morphometric type. Journal of the Elisha Mitchell Scientific Society 65(1) 1–37.

Frey, D.G. 1951a. Pollen succession in the sediments of Singletary Lake, North Carolina. Ecology 32(3): 518−533.

Frey, D.G. 1951b. The fishes of North Carolina’s bay lakes and their intraspecific variation. Journal of the Elisha Mitchell Scientific Society 67(1): 1−44.

Frey, D.G. 1954. Evidence for the recent enlargement of the “Bay” lakes of North Carolina. Ecology 35(1): 78−88.

Hubbs, C.L. and E.C. Raney. 1946. Endemic fish fauna of Lake Waccamaw, North Carolina. University of Michigan Press, Ann Harbor, MI, USA.

Louder, D.E. 1962. An annotated check list of the North Carolina bay lakes fishes. Journal of the Elisha Mitchell Scientific Society 78: 68−73.

Newman, M.C. and J.F. Schalles. 1990. The water chemistry of Carolina bays: A regional survey. Archiv für Hydrobiologie 118(2): 147−168.

Prouty, W.F. 1935. “Carolina Bays” and Elliptical Lake Basins. The Journal of Geology 43(2): 200−207.

Stager, J.C. and L.B. Cahoon. 1987. The age and trophic history of Lake Waccamaw, North Carolina. Journal of the Elisha Mitchell Scientific Society 103(1): 1−13.

Wells, B.W. and S.G. Boyce. 1953. Carolina bays: additional data on their origin, age and history. Journal of the Elisha Mitchell Scientific Society 69(2): 119−141.
