# Supplementary material for: Guide to the littoral zone vascular flora of Carolina bay lakes (U.S.A.)
Source: Biodivers Data J. 2016 Apr 5;(4):e7964. doi: 10.3897/BDJ.4.e7964 (PMC4911545; doi:10.3897/BDJ.4.e7964)
Supplement: Supplementary material 2 — Floras, manuals, guides, and broader floristic works on site-specific and broad-scale aquatic/wetland habitats of the eastern United States. [file biodiversity_data_journal-4-e7964-s002.doc]

Appendix B. Floras, manuals, guides, and broader floristic works on site-specific and broad-scale aquatic/wetland habitats of the eastern United States.

Aulbach-Smith, C., S.J. De Kozlowski, and L.A. Dyck. 1990. Aquatic and wetland plants of South Carolina. South Carolina Aquatic Plant Management Council in cooperation with the South Carolina Water Resources Commission, US.

Delong, M.K., S.K. Jog, J.R. Johansen, and G.J. Wilder. 2005. Floristic survey of a highly disturbed wetland within Shaker Median Park, Beachwood (Cuyahoga County), Ohio. Ohio Journal of Science 105(5): 102−115.

Dressler, R.L., D.W. Hall, K.D. Perkins, N.H. Williams. 1987. Identification manual for wetland plant species of Florida. University of Florida-Institute of Food and Agricultural Sciences, Gainesville.

Ferren Jr., W.R. and A.E. Schuyler. 1980. Intertidal vascular plants of river systems near Philadelphia. Proceedings of the academy of natural sciences of Philadelphia 132: 86–120.

Godfrey, R.K. and J.W. Wooten. 1979. Aquatic and Wetland Plants of Southeastern

United States. Volume 1. Monocotyledons. University of Georgia Press, Athens.

Godfrey, R.K. and J.W. Wooten. 1981. Aquatic and Wetland Plants of Southeastern

United States. Volume 2. Dicotyledons. University of Georgia Press, Athens.

Hellquist, C.E. and G.E. Crow. 2003. The vascular flora of mud pond peatland, Carroll County, New Hampshire. Rhodora 105(922): 153–177.

Holt, C.R., G.W. Folkerts, and D.R. Folkerts. 2011. A floristic study of a steephead stream in northwestern Florida. Southeastern Naturalist 10(2): 289–302.

Jog, S.K. 2003. Vascular plant flora of highland heights community park: a floristic survey and trend analysis of a suburban wetland. PhD Dissertation. Cleveland State University.

Kiernan, B.D. 2000. Characterization of alder/willow wetlands in the adirondack mountains of New York State. M.S. Thesis. New York College of Environmental Science and Forestry.

Leck, M.A. and C.F. Leck. 2005. Vascular plants of a Delaware River tidal freshwater wetland and adjacent terrestrial areas: seed bank and vegetation comparisons of reference and constructed marshes and annotated species list. Journal of the Torrey Botanical Society 132(2): 323–354.

MacRoberts, B.R., M.H. MacRoberts, D.C. Rudolph, and D.W. Peterson. 2014. Floristics of ephemeral ponds in east-central Texas. Southeastern Naturalist 13(5): 15−25.

Mattox, J.E. 1994. Wetland vascular flora of the pine bush, Albany and Schenectady counties, New York State, in the 19^th^ and 20^th^ centuries. M.S. Thesis. Bard College.

Nichols, W.F., G.E. Moore, N.P. Ritter, and C.R. Peter. 2013. A globally rare coastal salt pond marsh system at Odiorne Point State Park, Rye, New Hampshire. Rhodora 115(961): 1–27.

Reznicek, A.A. 1994. The disjunct coastal plain flora of the great lakes region. Biological Conservation 68(3): 203−215.

Sorrie, B.A. 1994. Coastal plain ponds in New England. Biological Conservation 68(3): 225–233.

Tiner Jr, R.W., A. Rorer, and R.H. Wiegand. 1988. Field guide to nontidal wetland identification. Cooperative publication of the United States Fish and Wildlife Service and Maryland Department of Natural Resources, Water Resources Administration. Institute for wetland and environmental education and research, Inc., Leverett, MA.

Tiner, R.W. 1993. Field guide to coastal wetland plants of the southeastern United States. University of Massachusetts Press, Amherst, MA.

Wood, D.W. 1966. Vascular plant flora of Haunck’s Pond, Middle Bass Island, Ottawa County, Ohio. PhD Dissertation. The Ohio State University.
