# Supplementary material for: Guide to the littoral zone vascular flora of Carolina bay lakes (U.S.A.)
Source: Biodivers Data J. 2016 Apr 5;(4):e7964. doi: 10.3897/BDJ.4.e7964 (PMC4911545; doi:10.3897/BDJ.4.e7964)
Supplement: Supplementary material 3 — Sample taxon entry with brief descriptions of working parts. [file biodiversity_data_journal-4-e7964-s003.doc]

Appendix C. Sample taxon entry with brief descriptions of working parts.

***Juncus*^1^ *funkus*^2^ (L.) Thornhill^3^ [=^4^ RAB^5^; < ^6^GW^7^; > ^8^ FNA^9^; Weakley^10^]**

***Scirpus funkus* L.^11^**

**W1; S1,G2^12^. Eulittoral zone^13^ (NLSS−C^14^, NLSS−LW^15^, NLSM−T^16^, NLSM−LWP^17^, CPSI−CG^18^, FB^19^). May−Jul^20^; Sep−Oct^21^. (Fig X. )^22^**

**Bakers Lake^23^ (Rare^24^): *Howell BALA 14* ^25^ (NCSC^26^!^27^)**

**Bay Tree Lake^28^ (Infrequent^29^): *Howell BATR-15* (NCSC!)**

**Horseshoe Lake^30^ (Occasional^31^): *Howell HOLA-16* (NCSC!)**

**Lake Wacaamaw^32^ (Frequent^33^): *Howell LAWA-17* (NCSC!); *Wilbur 7765* (DUKE^34^!); *LeBlond* 7643 (NCU^35^!)**

**Little Singletary Lake^36^ (Abundant^37^):** ♦ **^38^**

**Jones Lake^39^ (Frequent):** ¤ **^40^**

**Salters Lake^41^ (Infrequent):** • **^42^**

**Singletary Lake^43^ (Rare):** ► **^44^**

1. Genus
2. Specific epithet
3. Species author
4. Concept of this taxon is the same as the concept originally published in the floristic work (s) following the = symbol.
5. Radford, Ahles, and Bell (1968)
6. Concept of this taxon is narrower than the concept originally published in the floristic work (s) following the < symbol.
7. Godfrey and Wooten (1979 [Monocots],1981 [Dicots])
8. Concept of this taxon is broader than the concept originally published in the floristic work(s) following the > symbol.
9. Flora of North America
10. Weakley (2012)
11. Basionym (i.e., the name of the plant given by the person who first descibed it; in this case, Carl Linnaeus first named the plant *Scirpus funkus* and the plant was later moved to a new genus and named *Juncus funkus* by Thornhill).
12. Rarity of plant. For a more detailed explanation of state and federal rarity status, see Table 3.
13. Each habitat description will start off with where it typically occurs in reference to the littoral zone (see lacustrine zonation in Figure 1).
14. This plant occurs in the Natural Lake Shoreline Swamp (Cypress Subtype) community type (Schafale 2012).
15. This plant occurs in the Natural Lake Shoreline Swamp (Lake Waccamaw Subtype) community type (Schafale 2012).
16. This plant occurs in the Natural Lake Shoreline Marsh (Typic Subtype) community type (Schafale 2012).
17. This plant occurs in the Natural Lake Shoreline Marsh (Lake Waccamaw Pondlily Subtype) community type (Schafale 2012).
18. This plant occurs in the Coastal Plain Semipermanent Impoundment (Cypress−Gum Subtype) community type (Schafale 2012).
19. This plant occurs in the Floating Bog community type (Schafale 2012).
20. This plant can be found in flower during the months of May−July.
21. This plant can be found in fruit during September−October.
22. A black and white line drawing or digital photograph is provided for this taxon in Figure X.
23. This taxon was collected/reported/observed from Bakers Lake.
24. This taxon is “very difficult to find and is limited to one or very few locations or communities” (Palmer et al. 1995).
25. This was the 14^th^ specimen collected from Bakers Lake by the present author.
26. This specimen was deposited into North Carolina State Unviversity’s vascular plant herbarium.
27. The present author agrees with this taxons most recent identification.
28. This taxon was collected/reported/observed from Bay Tree Lake.
29. This taxon is “difficult to find with few individuals of colonies but found in several locations” (Palmer et al. 1995).
30. This taxon was collected/reported/observed from Horseshoe Lake.
31. This taxon is “Widely scattered but not difficult to find” (Palmer et al. 1995).
32. This taxon was collected/reported/observed from Lake Waccamaw.
33. This taxon is “easily seen or found in one or more common communities, but not dominant in any common community” (Palmer et al. 1995).
34. This taxon was collected and deposited into Duke University’s vascular plant herbarium.
35. This taxon was collected and deposited into the University of North Carolina at Chapel Hill’s vascular plant herbarium.
36. This taxon was collected/reported/observed from Little Singletary Lake.
37. This taxon is “Dominant or co-dominant in one or more common communities” (Palmer et al. 1995).
38. The Carolina Vegetation Survey reported this taxon from this site.
39. This taxon was collected/reported/observed from Jones Lake.
40. The North Carolina Crop Science Department reported this taxon from this site.
41. This taxon was collected/reported/observed from Salters Lake.
42. The present author has personally observed this taxon at this site but failed to collect a viable voucher specimen.
43. This taxon was collected/reported/observed from Singletary Lake.
44. The North Carolina Natural Heritage Program reported this taxon from this site.
