# Supplementary material for: Guide to the littoral zone vascular flora of Carolina bay lakes (U.S.A.)
Source: Biodivers Data J. 2016 Apr 5;(4):e7964. doi: 10.3897/BDJ.4.e7964 (PMC4911545; doi:10.3897/BDJ.4.e7964)
Supplement: Supplementary material 4 — Literature highlighting the ecological, biological, and cultural importance of Carolina bays. [file biodiversity_data_journal-4-e7964-s004.doc]

Appendix D. Literature highlighting the ecological, biological, and cultural importance of Carolina bays. For additional literature concerning Carolina bays, see Ross (2000).

Battaglia, L.L. and B.S. Collins. 2005. Linking hydroperiod and vegetation response in Carolina bay wetlands. Plant Ecology 184(1): 173−185.

Bennett, S.H. and J.B. Nelson. 1991. Distribution and status of Carolina bays in South Carolina. South Carolina Wildlife & Marine Resources Department. Nongame and Heritage Trust Publication, Columbia, SC.

Brooks, M.J., B.E. Taylor, and A.H. Ivester. 2010. Carolina bays: time capsules of culture and climate change. Southeastern Archaeology 29(1): 146−163.

Buhlmann, K.A. and J.W. Gibbons. 2001. Terrestrial habitat use by aquatic turtles from a seasonally fluctuating wetland: implications for wetland conservation boundaries. Chelonian Conservation and Biology 4(1): 115−127.

Burke, V.J. and J.W. Gibbons. 1995. Terrestrial buffer zones and wetland conservation: a case study of freshwater turtles in a Carolina bay. Conservation Biology 9(6): 1365−1369.

Clark, M.K., D.S. Lee, and J.B. Funderburg. 1985. The mammal fauna of Carolina bays, pocosins, and associated communities in North Carolina: an overview. Brimleyana (11): 1−38.

Czapka, S.J. and J.C. Kilgo. 2011. Importance of Carolina bays to the avifauna of pinelands in the southeastern United States. Southeastern Naturalist 10(2): 321−332.

De Steven, D. and M.M. Toner. 2004. Vegetation of upper coastal plain depression wetlands: environmental templates and wetland dynamics within a landscape framework. Wetlands 24(1): 23−42.

Dodd, C.K. 1992. Biological diversity of a temporary pond herpetofauna in north Florida sandhills. Biodiversity and Conservation 1(3): 125−142.

Edwards, A.L. and A.S. Weakley. 2001. Population biology and management of rare plants in depression wetlands of the southeastern coastal plain, USA. Natural Areas Journal 21(1): 12−35.

Gibbons, J.W. 2003. Terrestrial habitat: a vital component for herpetofauna of isolated wetlands. Wetlands 23(3): 630−635.

Gibbons, J.W., J.W. Coker, and T.M. Murphy Jr. 1977. Selected aspects of the life history of the rainbow snake (*Farancia erytrogramma*). Herpetologica 33(3): 276−281.

Kirkman, L.K. 1992. Cyclical vegetation dynamics in Carolina bay wetlands. PhD. Dissertation. University of Georgia, Athens.

Kirkman, L.K. and R.R. Sharitz. 1994. Vegetation disturbance and maintenance of diversity in intermittently flooded Carolina bays in South Carolina. Ecological Applications 4(1): 177−188.

Kirkman, L.K., M.B. Drew, L.T. West, and E.R. Blood. 1998. Ecotone characterization between upland longleaf pine/wiregrass stands and seasonally-ponded isolated wetlands. Wetlands 18(3): 346−364.

Lacey, E.P., A. Royo, R. Bates, and D. Herr. 2001. The role of population dynamic models in biogeographic studies: an illustration from a study of *Lobelia boykinii,* a rare species endemic to the Carolina bays. Castanea 66(1/2): 115−125.

LaClaire, L.V. and R. Franz. 1991. Importance of isolated wetlands in upland landscapes. In: The role of aquatic plants in Floridas lakes and rivers. Proceedings of the Second Annual Meeting of the Florida Lake Management Society. Florida Lake Management Society, Lakeland, Florida.

LeBlond, R.J. and G.S. Grant. 2005. Natural Area Inventory of Bladen County, North Carolina. Department of Environment and Natural Resources, Office of Conservation and Community Affairs, North Carolina Natural Heritage Program. Raleigh, NC.

Mahoney, D.L., M.A. Mort, and B.E. Taylor. 1990. Species richness of calanoid copepods, cladocerans and other brachiopods in Carolina bay temporary ponds. American Midland Naturalist 123 (2): 244–258.

Mamo, L.B. and E.G. Bolen. 1999. Effects of area, isolation, and landscape on the avifauna of Carolina bays. Journal of Field Ornithology 70(3): 310−320.

Moler, P.E. and R. Franz. 1987. Wildlife values of small, isolated wetlands in the southeastern coastal plain. Pages 234−241 in R.R. Odum, K.A. Riddleberger, and J.C. Ozier (editors). Proceedings of the Third Southeastern Nongame and Endangered Wildlife Symposium. Georgia Department of Natural Resources, Atlanta, GA.

Nifong, T.D. 1982. The clay subsoil bays of North Carolina. Report to the North Carolina Natural Heritage Program and North Carolina Nature Conservancy. PDF File.

Nifong, T.D. 1998. An ecosystematic analysis of Carolina bays in the coastal plain of the Carolinas. PhD Dissertation. The University of North Carolina at Chapel Hill.

Plummer, M.V. and J.D. Congdon. 1994. Radiotelemetric study of activity and movements of racers (*Coluber constrictor*) associated with a Carolina bay in South Carolina. Copeia 1994(1): 20−26.

Richardson, C.J. and J.W. Gibbons. 1993. Pocosins, Carolina bays, and mountain bogs. In: Biodiversity of the southeastern United States: lowland terrestrial communities. J. Wiley, New York.

Semlitsch, R.D., D.E. Scott, J.H.K. Pechmann, and J.W. Gibbons. 1996. Structure and dynamics of an amphibian community: evidence from a 16-year study of a natural pond. In: Long-term studies of vertebrate communities. Academic Press, San Diego.

Semlitsch, R.D. and J.R. Bodie. 1998. Are small isolated wetlands expendable? Conservation Biology 12(5): 1129−1133.

Sharitz, R.R. and J.W. Gibbons. 1982. Ecology of southeastern shrub bogs (pocosins) and Carolina bays: a community profile. U.S. Fish and Wildlife Service, Division of Biological Services, Washington, D.C.

Sutter, R.D. and R. Kral. 1994. The ecology, status, and conservation of two non-alluvial wetland communities in the south Atlantic and eastern Gulf coastal plain, USA. Biological Conservation 68(3): 235−243.

Taylor, B.E., D.A. Leeper, M.A. McClure, and A.E. DeBiase. 1999. Carolina bays: ecology of aquatic invertebrates and perspectives on conservation. In: Invertebrates in freshwater wetlands of North America: Ecology and Management. John Wiley & Sons, New York.

Whigham, D.F. 1999. Ecological issues related to wetland preservation, restoration, creation and assessment. Science of the Total Environment 240(1): 31−40.
