# Supplementary material for: Guide to the littoral zone vascular flora of Carolina bay lakes (U.S.A.)
Source: Biodivers Data J. 2016 Apr 5;(4):e7964. doi: 10.3897/BDJ.4.e7964 (PMC4911545; doi:10.3897/BDJ.4.e7964)
Supplement: Supplementary material 5 — Suggested collection methods for problematic aquatic taxa and sampling methods of floating bog communities. [file biodiversity_data_journal-4-e7964-s005.doc]

Appendix E. Suggested collection methods for problematic aquatic taxa and sampling methods of floating bog communities.

Adequate preservation of certain aquatic taxa (i.e., *Brasenia schreberi*, *Nymphaea odorata* ssp. *odorata, Sagittaria* L. *spp., Utricularia* spp.) proved challenging by conventional pressing methods. It is recommended that when pressing *Brasenia schreberi*, wax paper be used in place of newspaper and that the press be stored in a dry space. If pressed by conventional methods, the mucilagenous underwater portions of *Brasenia schreberi* will dry to newspaper/blotter paper permanently. Likewise, wax paper should also be used when pressing specimens of *Sagittaria*. The petals of *Sagittaria* are delicate, and, when pressed, will adhere to newspaper or blotting paper, thereby making their removal and measurements difficult to attain. The large roots of *Nymphaea odorata* ssp. *odorata* proved difficult to press efficiently. Prior to pressing, it is recommended that a small section of the root be cut longitudinally in half (i.e., lengthwise) in order to reduce its diameter. The roots of other floating-leaved aquatics (e.g., *Nelumbo lutea*, *Nuphar Sagittifolia*) can be treated in this same manner.

Similar to the petals of *Sagittaria*, the petals of *Utricularia* also are delicate and will adhere to pressing papers. Wax paper should be used when pressing bladderworts in order to better preserve the integrity of flowers. Taylor (1989) suggested preserving specimens of *Utricularia* in a liquid solution of 37% water, 53% alcohol, 5% formaldehyde, and 5% glycerol or “any potable spirit will do if the collector is prepared to make the sacrifice”! Specimens should be placed in the solution immmediately while in the field. Bottles should be large enough to hold most taxa found in the Coastal Plain of North Carolina (ca. 10−15 × 3 cm). Prior to the collection of *Utricularia* specimens, detailed notes should be taken concerning corolla color, size, shape, and habit; digital photographs of plant habit and corolla morphology are always a nice addition. Fruits and seeds are also important characteristics needed for identification and should be noted when available.

The floating bogs of Horseshoe Lake, while breathtakingly beautiful and rich with emergent,

herbaceous, macrophytes, were difficult to sample. Because these bogs float on top of the water column, they are difficult to traverse. The present author made several attempts to walk on the bogs, but never made it much further than about five to ten meters. Walking on the bogs is smilar to walking on a waterbed, when firmly planting one foot down onto the bog, vegetaton five to ten meters away will subsequently begin to rise. On several occassions, one leg of the current author broke through the upper portions of the bog and penetrated into water column beneath the bog. After consulting several well-seasoned botanists on the subject of how best to sample this community, there were no decisive solutions.

Here, I provide a couple of ideas for future work on this community:

(1) Snowshoes – By increasing the surface area of footwear, researchers could walk further distances on the bogs; however, walking on bogs with snowshoe-like footwear would result in some plant mortality.

(2) Scaffolding System – Some floating bogs are small enough to build a scaffolding framework around their periphery. If the framework were permanently constructed, researchers need only bring the flooring (e.g., 2 × 6 × 12 boards, bamboo) at the time of each sampling visit. The flooring could be placed ca. 1 ft above the vegetation and researchers could sample the vegetation from over the sides of the scaffold.
