# Supplementary material for: Guide to the littoral zone vascular flora of Carolina bay lakes (U.S.A.)
Source: Biodivers Data J. 2016 Apr 5;(4):e7964. doi: 10.3897/BDJ.4.e7964 (PMC4911545; doi:10.3897/BDJ.4.e7964)
Supplement: Supplementary material 6 — Checklist of the littoral zone vascular flora of unaltered Carolina bay lake shorelines (i.e., Bakers Lake, Bay Tree Lake, Horseshoe Lake, Jones Lake, Lake Waccamaw, Little Singletary Lake, Salters Lake, Singletary Lake). [file biodiversity_data_journal-4-e7964-s006.doc]

Appendix F. Checklist of the littoral zone vascular flora of unaltered Carolina bay lake shorelines (i.e., Bakers Lake, Bay Tree Lake, Horseshoe Lake, Jones Lake, Lake Waccamaw, Little Singletary Lake, Salters Lake, Singletary Lake). Taxa are organized by major plant groups (i.e., pteridophytes, gymnosperms, basal angiosperms, magnoliids, monocotyledons, and eudicotyledons), then alphabetically by family, genus, and species. Parentheses around a taxon indicate that it is unvouchered (i.e., it has been reported by state agencies or has been observed by the present author, but has not been collected as a voucher specimen; see text for details). For taxa collected from Carolina bay lake littoral zones *by the present author*, abundance estimates sensu Palmer et al. (1995) are provided. Abundance estimates in this checklist reflect the abundance in which the taxa occur within each lake. Status and rank designations are also provided for rare taxa monitored by the NC Natural Heritage Program (Robinson and Finnegan 2014). The term “restricted” is used here only to indicate the presence of a taxon within a particular lake among all those surveyed and not in a global sense (e.g., a taxon here considered restricted to Lake Waccamaw has not been found in the other lakes surveyed, but may exist in other localities in the state or country). A = Abundant; F= Frequent; I=Infrequent; O = Occasional; R = Rare; = 🗹 restricted to lake indicated; () = unvouchered (i.e., reported by state agencies or observed by the present author, but not collected as a voucher specimen; see text for details); H = taxon has been collected and vouchered in the past but not by the present author. BALA = Bakers Lake; BATR = Bay Tree Lake; HOLA = Horseshoe Lake; JOLA = Jones Lake; LAWA = Lake Waccamaw; LISI = Little Singletary; SALA = Salters Lake; SILA = Singletary Lake.

|  | **Family** | **BALA** | **BATR** | **HOLA** | **JOLA** | **LAWA** | **LISI** | **SALA** | **SILA** |
| --- | --- | --- | --- | --- | --- | --- | --- | --- | --- |
| **Pteridophytes** |  |  |  |  |  |  |  |  |  |
| *Anchistea virginica* (L.) Sm. | Blechnaceae | I | O |  | R | I | O |  |  |
| *Lorinseria areolata* (L.) C. Presl. | Blechnaceae |  | O |  |  | I | O |  | H |
| *Dryopteris ludoviciana* (Kunze) Small W1; S2, G4 | Dryopteraceae |  |  |  |  | H🗹 |  |  |  |
| *Lycopodiella appressa* (Chapm.) Cranfill | Lycopodiaceae |  | H | I |  | I |  |  |  |
| *Onoclea sensibilis* L. | Onocleaceae |  |  |  |  | H🗹 |  |  |  |
| *Osmunda spectabilis* Willd. | Osmundaceae |  |  |  |  | I🗹 |  |  |  |
| *Pleopeltis polypodiodes* (L.) E.G. Andrews & Windham ssp. *michauxiana* (Weath.) E.G. Andrews & Windham | Polypodiaceae |  |  |  |  | I |  | I |  |

| Appendix F continued |  |  |  |  |  |  |  |  |  |
| --- | --- | --- | --- | --- | --- | --- | --- | --- | --- |
|  | **Family** | **BALA** | **BATR** | **HOLA** | **JOLA** | **LAWA** | **LISI** | **SALA** | **SILA** |
| **Gymnosperms** |  |  |  |  |  |  |  |  |  |
| *Chamaecyparis thyoides* (L.) Britton, Sterns & Poggenb. | Cupressaceae |  | I | O | O |  | O |  | I |
| *Taxodium ascendens* Brongn. | Cupressaceae | A | A | A | A | A | A | A | A |
| *Taxodium distichum* (L.) L.C. Rich. | Cupressaceae |  | H |  | H | H |  | H | H |
| *Pinus serotina* Michx. | Pinaceae |  |  |  | R |  |  |  | R |
| *Pinus taeda* L. | Pinaceae |  |  |  |  | R | I |  | R |
|  |  |  |  |  |  |  |  |  |  |
| **Basal Angiosperms** |  |  |  |  |  |  |  |  |  |
| *Brasenia schreberi* J.F. Gmel. | Cabombaceae |  |  | I🗹 |  |  |  |  |  |
| *Cabomba caroliniana* A. Gray | Cabombaceae |  |  | I🗹 |  |  |  |  |  |
| *Nuphar sagittifolia* (Walter) Pursh W1, FSC; S2, G5T2 | Nymphaeaceae |  |  |  |  | O🗹 |  |  |  |
| *Nymphaea odorata* W.T. Aiton ssp. *odorata* | Nymphaeaceae |  |  | F |  | O |  |  | H |
|  |  |  |  |  |  |  |  |  |  |
| **Magnoliids** |  |  |  |  |  |  |  |  |  |
| *Magnolia virginiana* L. var. *virginiana* | Magnoliaceae | O |  |  | O | O |  | O | O |
| *Persea palustris* (Raf.) Sarg. | Lauraceae | O |  |  | O | O | O | O | O |
|  |  |  |  |  |  |  |  |  |  |
| **Monocotyledons** |  |  |  |  |  |  |  |  |  |
| *Sagittaria filiformis* J.G. Smith SR−P; SH, G4G5 | Alismataceae |  |  |  |  | H🗹 |  |  |  |
| *Sagittaria graminea* Michx. | Alismataceae |  |  |  |  | F🗹 |  |  |  |

| Appendix F continued |  |  |  |  |  |  |  |  |  |
| --- | --- | --- | --- | --- | --- | --- | --- | --- | --- |
|  | **Family** | **BALA** | **BATR** | **HOLA** | **JOLA** | **LAWA** | **LISI** | **SALA** | **SILA** |
| *Sagittaria isoetiformis* J.G. Smith T; S2, G4? | Alismataceae |  |  | I |  | H |  |  |  |
| *Sagittaria weatherbiana* Fernald E, FSC; S2, G3G4 | Alismataceae |  |  |  |  | H🗹 |  |  |  |
| * *Colocasia esculenta* (L.) Schott | Aracaceae |  |  |  |  | I🗹 |  |  |  |
| *Wolffia* spp. Horkel ex Schleid. | Aracaceae |  |  |  |  | (🗹) |  |  |  |
| *Tillandsia usneoides* (L.) L. | Bromeliaceae | () | F | () | O | A | O | O | F |
| *Burmannia capitata* (Walter ex J.F. Gmel.) Mart. | Burmanniaceae |  |  |  |  | H🗹 |  |  |  |
| *Carex alata* Torr. | Cyperaceae |  |  |  |  | R🗹 |  |  |  |
| *Carex longii* Mack. | Cyperaceae |  | R🗹 |  |  |  |  |  |  |
| *Carex lupulina* Muhl. Ex Willd. | Cyperaceae |  |  |  |  | R🗹 |  |  |  |
| *Carex striata* Michx. var. *brevis* L.H. Bailey | Cyperaceae |  |  | H🗹 |  |  |  |  |  |
| *Cladium mariscoides* (Muhl.) Torr. SR−O; S3, G5 | Cyperaceae |  |  |  |  | A🗹 |  |  |  |
| *Cyperus erythrorhizos* Muhl. | Cyperaceae |  |  | H🗹 |  |  |  |  |  |
| *Cyperus odoratus* L. var. odoratus | Cyperaceae |  | R🗹 |  |  |  |  |  |  |
| *Cyperus polystachyos* Rottb. | Cyperaceae |  |  |  | R🗹 |  |  |  |  |
| *Dulichium arundinaceum* (L.) Britton var. *arundinaceum* | Cyperaceae |  |  | O |  | O | I |  |  |
| *Eleocharis baldwinii* (Torr.) Chapm. | Cyperaceae |  | I |  |  |  | R |  |  |
| *Eleocharis baldwinii* (Torr.) Chapm./*E. vivipara* Link | Cyperaceae |  |  | () |  |  |  |  |  |
| *Eleocharis equisetoides* (Elliott) Torr. W1; S3, G4 | Cyperaceae |  |  |  |  | I | R |  |  |

| Appendix F continued |  |  |  |  |  |  |  |  |  |
| --- | --- | --- | --- | --- | --- | --- | --- | --- | --- |
|  | **Family** | **BALA** | **BATR** | **HOLA** | **JOLA** | **LAWA** | **LISI** | **SALA** | **SILA** |
| *Eleocharis olivacea* Torr. var. olivacea | Cyperaceae |  |  |  |  | R🗹 |  |  |  |
| *Eleocharis vivipara* Link E; S1, G5 | Cyperaceae |  |  |  |  |  | R🗹 |  |  |
| *Fimbristylis autumnalis* (L.) Roem. & Schult. | Cyperaceae |  |  |  |  | H🗹 |  |  |  |
| *Fuirena pumila* Michx. | Cyperaceae |  | R🗹 |  |  |  |  |  |  |
| *Rhynchospora alba* (L.) Vahl SR−P; S2, G5 | Cyperaceae |  |  | O🗹 |  |  |  |  |  |
| *Rhynchospora corniculata* (Lam.) A. Gray var. *corniculata* | Cyperaceae |  |  |  |  | O🗹 |  |  |  |
| *Rhynchospora distans* (Michx.) Vahl | Cyperaceae | R |  |  |  | H | R |  |  |
| *Rhynchospora elliottii* A. Dietr. | Cyperaceae |  |  |  |  | H🗹 |  |  |  |
| *Rhynchospora inexpansa* (Michx.) Vahl | Cyperaceae |  |  |  | H🗹 |  |  |  |  |
| *Rhynchospora inundata* (Oakes) Fernald W1; S3, G4? | Cyperaceae |  |  | I🗹 |  |  |  |  |  |
| *Rhynchospora latifolia* (Baldwin) W.W. Thomas | Cyperaceae |  |  |  |  | H🗹 |  |  |  |
| *Rhynchospora macrostachya* Torr. ex A. Gray | Cyperaceae |  |  |  |  | I🗹 |  |  |  |
| *Rhynchospora nitens* (Vahl.) A. Gray W1; S3, G4? | Cyperaceae |  |  |  |  | H🗹 |  |  |  |
| *Scirpus cyperinus* (L.) Kunth | Cyperaceae |  | O |  | O | O | () |  |  |
| *Eriocaulon aquaticum* (Hill) Druce SC−V; S2, G5 | Eriocaulaceae |  |  |  |  | A🗹 |  |  |  |
| *Eriocaulon compressum* Lam. | Eriocaulaceae |  |  |  |  | (🗹) |  |  |  |
| *Lachnanthes caroliniana* (Lam.) Dandy | Haemodoraceae |  | I | I |  | O | I |  |  |

| Appendix F continued |  |  |  |  |  |  |  |  |  |
| --- | --- | --- | --- | --- | --- | --- | --- | --- | --- |
|  | **Family** | **BALA** | **BATR** | **HOLA** | **JOLA** | **LAWA** | **LISI** | **SALA** | **SILA** |
| * *Hydrilla verticillata* (L. f.) Royle | Hydrocharitaceae |  |  |  |  | (🗹) |  |  |  |
| *Najas guadalupensis* (Spreng) Magnus var. *guadalupensis* | Hydrocharitaceae |  |  |  |  | H🗹 |  |  |  |
| *Hypoxis curtissii* Rose | Hypoxidaceae |  |  |  |  | R🗹 |  |  |  |
| *Juncus acuminatus* Michx. | Juncaceae |  | R🗹 |  |  |  |  |  |  |
| *Juncus biflorus* Elliott | Juncaceae |  |  |  |  |  | R |  | H |
| *Juncus canadensis* J. Gay ex Laharpe | Juncaceae |  |  |  |  | I🗹 |  |  |  |
| *Juncus coriaceus* Mack. | Juncaceae |  |  | H🗹 |  |  |  |  |  |
| *Juncus effusus* L. subsp. *Solutus* (Fernald & Wiegand) Hämet-Ahti | Juncaceae |  | O | O |  |  | O |  |  |
| *Juncus pelocarpus* E. Meyer | Juncaceae |  | F | H | O | F |  |  | O |
| *Juncus repens* Michx. | Juncaceae |  | O | O |  | O | O |  | O |
| *Juncus scirpoides* Lam. Var. *compositus* | Juncaceae |  | I |  |  |  | I |  |  |
| *Mayaca fluviatilis* Aubl. | Mayacaceae |  |  |  |  | (🗹) |  |  |  |
| *Calopogon tuberosus* (L.) Britton, Sterns & Poggenb. Var. *tuberosus* | Orchidaceae |  |  | O🗹 |  |  |  |  |  |
| *Epidendrum magnoliae* Muhl. T; S1S2, G4 | Orchidaceae |  |  |  |  | (H🗹) |  |  |  |
| *Habenaria repens* Nutt. W1; S2, G5 | Orchidaceae |  |  |  |  | (🗹) |  |  |  |
| *Pogonia ophioglossoides* (L.) Ker Gawl. | Orchidaceae |  |  | O🗹 |  |  |  |  |  |

| Appendix F continued |  |  |  |  |  |  |  |  |  |
| --- | --- | --- | --- | --- | --- | --- | --- | --- | --- |
|  | **Family** | **BALA** | **BATR** | **HOLA** | **JOLA** | **LAWA** | **LISI** | **SALA** | **SILA** |
| *Spiranthes laciniata* (Small) Ames SC−V; S2, G4G5 | Orchidaceae |  |  |  |  | O🗹 |  |  |  |
| *Agrostis hyemalis* (Walter) Britton, Sterns & Poggenb. | Poaceae |  |  |  |  |  | R🗹 |  |  |
| *Andropogon glaucopsis* Steud. | Poaceae |  |  | () | I |  |  |  |  |
| *Andropogon virginicus* L. var. *virginicus* | Poaceae |  |  |  |  | (🗹) |  |  |  |
| *Arundinaria tecta* (Walter) Muhl. | Poaceae |  |  |  |  | H🗹 |  |  |  |
| *Coleataenia longifolia* (Torr.) Soreng var. *longifolia* | Poaceae |  | I |  |  | O |  |  |  |
| *Coleataenia tenera* (Bey. ex Trin.) Soreng | Poaceae |  |  |  |  | (🗹) |  |  |  |
| *Dichanthelium boreale* (Nash) Freckmann | Poaceae |  |  |  |  | H🗹 |  |  |  |
| *Dichanthelium dichotomum* (L.) Gould var. *roanokense* (Ashe) LeBlond W1; S2, G5T4? | Poaceae |  |  |  |  | H🗹 |  |  |  |
| *Dichanthelium erectifolium* (Nash) Gould & C.A. Clark W1; S2, G4 | Poaceae |  |  |  |  | I🗹 |  |  |  |
| *Dichanthelium mattamuskeetense* (Ashe) Mohlenbr. | Poaceae |  |  |  |  | H🗹 |  |  |  |
| *Dichanthelium portoricense* (Desv. ex Ham.) B.F. Hansen & Wunderlin | Poaceae |  | I |  |  | H |  |  |  |
| *Dichanthelium species 3* (=lancearium) | Poaceae |  |  |  |  | H🗹 |  |  |  |

| Appendix F continued |  |  |  |  |  |  |  |  |  |
| --- | --- | --- | --- | --- | --- | --- | --- | --- | --- |
|  | **Family** | **BALA** | **BATR** | **HOLA** | **JOLA** | **LAWA** | **LISI** | **SALA** | **SILA** |
| *Eragrostis elliottii* S. Watson | Poaceae |  | R |  |  | () |  |  |  |
| *Eragrostis refracta* (Muhl. Ex Elliott) Scribn. | Poaceae |  |  |  |  | (H🗹) |  |  |  |
| *Luziola fluitans* (Michx.) Terrell & H. Rob. var. *fluitans* SR−P; S2, G4G5 | Poaceae |  |  |  |  | O🗹 |  |  |  |
| *Panicum hemitomon* Schult. | Poaceae |  | A | I |  | A | I | O | O |
| *Panicum verrucosum* Muhl. | Poaceae |  | I |  | R |  | R |  |  |
| *Panicum virgatum* L. var. *virgatum* | Poaceae |  | H🗹 |  |  |  |  |  |  |
| *Saccharum giganteum* (Walter) Pers. | Poaceae |  |  |  | R | O |  |  |  |
| *Sacciolepis striata* (L.) Nash | Poaceae |  | O | H |  | O |  |  | O |
| *Sphenopholis obtusata* (Michx.) Scribn. | Poaceae |  |  |  |  | (H🗹) |  |  |  |
| *Pontederia cordata* (L.) var. *cordata* | Pontederiaceae |  | () |  |  | O |  |  |  |
| *Pontederia cordata* (L.) var. *lancifolia* (Muhl.) Torr. | Pontederiaceae |  |  |  |  | (🗹) |  |  |  |
| *Potamogeton pulcher* Tuck. | Potamogetonaceae |  |  |  |  | (🗹) |  |  |  |
| *Potamogeton pusillus* L. | Potamogetonaceae |  |  |  |  | (🗹) |  |  |  |
| *Smilax glauca* Walter | Smilacaceae |  | R🗹 |  |  |  |  |  |  |
| *Smilax laurifolia* L. | Smilacaceae | F | O | O | F | I |  | F | F |
| *Smilax rotundifolia* L. | Smilacaceae |  |  |  |  | (🗹) |  |  |  |
| *Smilax walteri* Pursh | Smilacaceae |  |  | O |  | O |  |  |  |
| *Xyris fimbriata* Elliott | Xyridaceae |  |  | H |  | () |  |  | H |

| Appendix F continued |  |  |  |  |  |  |  |  |  |
| --- | --- | --- | --- | --- | --- | --- | --- | --- | --- |
|  | **Family** | **BALA** | **BATR** | **HOLA** | **JOLA** | **LAWA** | **LISI** | **SALA** | **SILA** |
| *Xyris iridifolia* Chapm. W7; S2, G4G5T4 | Xyridaceae |  |  |  |  |  |  | (🗹) |  |
| *Xyris jupicai* Rich. | Xyridaceae |  |  |  |  |  | I🗹 |  |  |
| *Xyris smalliana* Nash W1; S3, G5 | Xyridaceae |  |  | H | O | A |  | H | O |
|  |  |  |  |  |  |  |  |  |  |
| **Eudicotyledons** |  |  |  |  |  |  |  |  |  |
| *Liquidambar styraciflua* L. | Altingiaceae |  | I |  |  | O |  |  |  |
| **Alternanthera philoxeroides* (Mart.) Griseb. | Amaranthaceae |  |  |  |  | I🗹 |  |  |  |
| *Rhus copallinum* L. var. *copallinum* | Anacardiaceae | R🗹 |  |  |  |  |  |  |  |
| *Toxicodendron radicans* (L.) Kuntze var. *radicans* | Anacardiaceae |  | I |  |  | O |  |  |  |
| *Centella asiatica* (L.) Urban | Apiaceae |  |  |  |  | F🗹 |  |  |  |
| *Cicuta maculata* L. | Apiaceae |  |  |  |  | R🗹 |  |  |  |
| *Ilex coriacea* (Pursh) Chapm. | Aquifoliaceae |  | R |  | O |  |  |  |  |
| *Ilex glabra* (L.) A. Gray | Aquifoliaceae | R | R |  |  | I |  |  |  |
| *Hydrocotyle umbellata* L. | Araliaceae |  | () |  |  | () |  |  |  |
| *Baccharis halimifolia* L. | Asteraceae |  |  |  |  | I🗹 |  |  |  |
| *Bidens laevis* (L.) Britton, Sterns & Poggenb. | Asteraceae |  |  |  |  | H🗹 |  |  |  |
| *Boltonia asteroides* (L.) L’Hér. Var. *glastifolia* (Hill) Fernald SR−O; S2, G5TNR | Asteraceae |  |  |  |  | I🗹 |  |  |  |
| *Erigeron vernus* (L.) Torr. & A. Gray | Asteraceae |  |  |  |  | (🗹) |  |  |  |

| Appendix F continued |  |  |  |  |  |  |  |  |  |
| --- | --- | --- | --- | --- | --- | --- | --- | --- | --- |
|  | **Family** | **BALA** | **BATR** | **HOLA** | **JOLA** | **LAWA** | **LISI** | **SALA** | **SILA** |
| *Eupatorium capillifolium* (Lam.) Small ex Porter & Britton | Asteraceae |  |  |  |  | I🗹 |  |  |  |
| *Eupatorium mohrii* Greene x *paludicola* E.E. Schilling & LeBlond | Asteraceae |  |  |  |  | R🗹 |  |  |  |
| *Euthamia caroliniana* (L.) Greene ex Porter & Britton | Asteraceae |  |  |  |  | R🗹 |  |  |  |
| * *Hypochaeris radicata* L. | Asteraceae |  | R🗹 |  |  |  |  |  |  |
| *Krigia virginica* (L.) Willd. | Asteraceae |  | R🗹 |  |  |  |  |  |  |
| *Mikania scandens* (L.) Willd. | Asteraceae |  |  |  |  | I🗹 |  |  |  |
| *Pluchea baccharis* (P. Miller) Pruski | Asteraceae |  |  |  |  | F🗹 |  |  |  |
| *Sclerolepis uniflora* (Walter) Britton, Sterns & Poggenb. SR−T; S2, G4 | Asteraceae |  |  |  |  | F🗹 |  |  |  |
| *Solidago fistulosa* P. Miller | Asteraceae |  | R | H |  |  |  |  |  |
| *Alnus serrulata* (Aiton) Willd. | Betulaceae |  |  |  |  | O🗹 |  |  |  |
| *Betula nigra* L. | Betulaceae |  | O |  |  | O | I |  |  |
| *Campsis radicans* (L.) Bureau | Bignoniaceae |  |  |  |  | I🗹 |  |  |  |
| *Lobelia nuttallii* Roem. & Schult. | Campanulaceae |  |  | R🗹 |  |  |  |  |  |
| *Lobelia glandulosa* Walter | Campanulaceae |  |  |  |  | (🗹) |  |  |  |
| *Stipulicida setacea* Michx. var. setacea | Caryophyllaceae |  | R🗹 |  |  |  |  |  |  |
| *Clethra alnifolia* L. | Clethraceae |  | O |  | O | O | O |  | O |
| *Cyrilla racemiflora* L. | Cyrillaceae |  | F | O | () | F | F |  | F |
| *Drosera intermedia* Hayne | Droseraceae |  |  | F |  |  | O |  |  |

| Appendix F continued |  |  |  |  |  |  |  |  |  |
| --- | --- | --- | --- | --- | --- | --- | --- | --- | --- |
|  | **Family** | **BALA** | **BATR** | **HOLA** | **JOLA** | **LAWA** | **LISI** | **SALA** | **SILA** |
| *Diospyros virginiana* L. | Ebenaceae |  |  |  |  | R🗹 |  |  |  |
| *Chamaedaphne calyculata* (L.) Moench | Ericaceae | O |  | O |  |  | O |  | H |
| *Eubotrys racemosa* (L.) Nutt. | Ericaceae |  |  | O | O | O | O | O | O |
| *Lyonia ligustrina* (L.) DC. var. *foliosiflora* (Michx.) Fernald | Ericaceae | I |  |  | I |  |  | I |  |
| *Lyonia lucida* (L.) K. Koch | Ericaceae | O | O | O | F | O | O | F | F |
| *Rhododendron viscosum* (L.) Torr. var. *serrulatum* (Small) H.E. Ahles | Ericaceae |  |  |  |  |  |  |  | R🗹 |
| *Vaccinium formosum* Andrews | Ericaceae |  | O |  | O |  |  | O | O |
| *Vaccinium fuscatum* Aiton | Ericaceae | O |  |  |  | O | O | O | O |
| *Zenobia pulverulenta* (W. Bartram ex Willd.) Pollard | Ericaceae | I |  | O | O |  | O |  | H |
| * *Triadica sebifera* (L.) Small | Euphorbiaceae |  |  |  |  | I🗹 |  |  |  |
| *Wisteria frutescens* (L.) Poiret | Fabaceae |  | O |  |  | O |  |  | I |
| *Quercus nigra* L. | Fagaceae |  |  |  |  | H🗹 |  |  |  |
| *Gelsemium sempervirens* (L.) J. St.-Hil. | Gelsemiaceae |  | O | R | I | O | I |  | I |
| *Decumaria barbara* L. | Hydrangeaceae |  |  |  |  | R🗹 |  |  |  |
| *Hypericum canadense* L. | Hypericaceae |  |  | R🗹 |  |  |  |  |  |
| *Hypericum mutilum* L. var. *mutilum* | Hypericaceae |  |  |  |  | R🗹 |  |  |  |
| *Hypericum virginicum* L. | Hypericaceae |  | O | O |  |  | O |  |  |
| *Hypericum walteri* J.F. Gmel. | Hypericaceae |  |  |  |  | O🗹 |  |  |  |
| *Itea virginica* L. | Iteaceae | I |  | O | O | O |  |  | O |

| Appendix F continued |  |  |  |  |  |  |  |  |  |
| --- | --- | --- | --- | --- | --- | --- | --- | --- | --- |
|  | **Family** | **BALA** | **BATR** | **HOLA** | **JOLA** | **LAWA** | **LISI** | **SALA** | **SILA** |
| *Carya glabra* (Mill.) Sweet | Juglandaceae |  |  |  |  | R🗹 |  |  |  |
| *Lycopus angustifolius* Elliott SR−P; S1, G4?Q | Lamiaceae |  |  |  |  | I🗹 |  |  |  |
| *Utricularia cornuta* Michx. T; S1S2, G5 | Lentibulariaceae |  |  |  |  | R🗹 |  |  |  |
| *Utricularia gibba* L. | Lentibulariaceae |  |  | O🗹 |  |  |  |  |  |
| *Utricularia purpurea* Walter | Lentibulariaceae |  |  | O🗹 |  |  |  |  |  |
| *Utricularia resupinata* B.D. Greene ex Bigelow E; S1, G4 | Lentibulariaceae |  |  |  |  | R🗹 |  |  |  |
| *Utricularia striata* Leconte ex Torr. | Lentibulariaceae |  | O | O |  | O |  |  |  |
| *Utricularia subulata* L. | Lentibulariaceae |  |  |  |  | () | I |  |  |
| *Lindernia dubia* (L.) Pennell var. *dubia* | Linderniaceae |  |  |  |  | H🗹 |  |  |  |
| *Mitreola petiolata* (J.F. Gmel.) Torr. & A. Gray | Loganiaceae |  |  |  |  | I🗹 |  |  |  |
| *Decodon verticillatus* (L.) Elliott | Lythraceae |  | () | O | () |  |  | () |  |
| *Rhexia aristosa* Britton SC−V; FSC; S3, G3G4 | Melastomataceae |  |  | (🗹) |  |  |  |  |  |
| *Rhexia cubensis* Griseb. W1; S3, G4G5 | Melastomataceae |  |  |  |  | O🗹 |  |  |  |
| *Rhexia mariana* L. var. *exalbida* Michx. | Melastomataceae |  |  | R🗹 |  |  |  |  |  |
| *Rhexia nashii* Small | Melastomataceae |  | O | O | I |  | O |  | I |
| *Rhexia virginica* L. | Melastomataceae |  |  |  |  |  | I🗹 |  |  |
| *Nymphoides aquatica* (Walter ex J.F. Gmel.) Kuntze | Menyanthaceae |  |  |  |  | O🗹 |  |  |  |

| Appendix F continued |  |  |  |  |  |  |  |  |  |
| --- | --- | --- | --- | --- | --- | --- | --- | --- | --- |
|  | **Family** | **BALA** | **BATR** | **HOLA** | **JOLA** | **LAWA** | **LISI** | **SALA** | **SILA** |
| *Morella cerifera* (L.) Small | Myricaceae |  | I |  | I | O |  | I |  |
| *Nelumbo lutea* Willd. W7; S2, G4 | Nelumbonaceae |  |  |  |  | H🗹 |  |  |  |
| *Nyssa aquatica* L. | Nyssaceae |  |  |  |  | I🗹 |  |  |  |
| *Nyssa biflora* Walter | Nyssaceae | F | O | O |  | O | O | () | O |
| *Fraxinus caroliniana* P. Miller | Oleaceae |  |  |  |  | I🗹 |  |  |  |
| *Ludwigia brevipes* (Long) Eames SR−T, FSC; S1S2, G2G3 | Onagraceae |  |  |  |  | I🗹 |  |  |  |
| *Ludwigia sphaerocarpa* Elliott E; S1, G5 | Onagraceae |  |  |  |  | O🗹 |  |  |  |
| *Bacopa caroliniana* (Walter) B.L. Rob. T; S1, G4G5 | Plantaginaceae |  |  |  |  | R🗹 |  |  |  |
| *Nuttallanthus canadensis* (L.) D.A. Sutton | Plantaginaceae |  | R🗹 |  |  |  |  |  |  |
| *Platanus occidentalis* L. | Platanaceae |  |  |  |  | I🗹 |  |  |  |
| *Polygala lutea* L. | Polygalaceae |  |  | R |  | R |  |  |  |
| *Rumex hastatulus* Baldwin | Polygonaceae |  | I🗹 |  |  |  |  |  |  |
| *Clematis crispa* L. | Ranunculaceae |  |  |  |  | H🗹 |  |  |  |
| *Berchemia scandens* (Hill) K. Koch | Rhamnaceae |  |  |  |  | O🗹 |  |  |  |
| *Amelanchier canadensis* (L.) Medik. | Rosaceae |  | H🗹 |  |  |  |  |  |  |
| *Amelanchier obovalis* (Michx.) Ashe | Rosaceae |  |  |  |  | R🗹 |  |  |  |
| *Aronia arbutifolia* (L.) Pers. | Rosaceae |  |  |  |  |  |  | H | R🗹 |
| *Rosa palustris* Marshall | Rosaceae |  |  |  |  | I |  |  | H |

| Appendix F continued |  |  |  |  |  |  |  |  |  |
| --- | --- | --- | --- | --- | --- | --- | --- | --- | --- |
|  | **Family** | **BALA** | **BATR** | **HOLA** | **JOLA** | **LAWA** | **LISI** | **SALA** | **SILA** |
| *Rubus pensilvanicus* Poir. | Rosaceae |  |  |  |  | I🗹 |  |  |  |
| *Cephalanthus occidentalis* L. | Rubiaceae |  |  |  |  | O🗹 |  |  |  |
| *Diodia virginiana* L. | Rubiaceae |  | R🗹 |  |  |  |  |  |  |
| *Galium obtusum* Bigelow var. *obtusum* | Rubiaceae |  |  |  |  | R🗹 |  |  |  |
| *Populus heterophylla* L. | Salicaceae |  |  |  |  | I🗹 |  |  |  |
| *Salix caroliniana* Michx. | Salicaceae |  |  |  |  | H🗹 |  |  |  |
| *Salix nigra* Marshall | Salicaceae |  |  |  |  | I🗹 |  |  |  |
| *Phoradendron leucarpum* (Raf.) Reveal & M.C. Johnst. ssp. *leucarpum* | Santalaceae |  |  |  | I |  |  | I |  |
| *Acer rubrum* L. var.  *rubrum* | Sapindaceae | R🗹 |  |  |  |  |  |  |  |
| *Acer rubrum* L. var. *trilobum* Torr. & A. Gray ex K. Koch | Sapindaceae |  | O | O | O | O | O | O | O |
| *Aesculus pavia* L. var. *pavia* | Sapindaceae |  |  |  |  | H🗹 |  |  |  |
| *Sarracenia flava* L. | Sarraceniaceae |  |  | A🗹 |  |  |  |  |  |
| *Gordonia lasianthus* (L.) Ellis | Theaceae | I |  | H | O |  | I |  | I |
| *Ulmus americana* L. var. *americana* | Ulmaceae |  |  |  |  | R🗹 |  |  |  |
| *Muscadinia rotundifolia* (Michx.) Small var. *rotundifolia* | Vitaceae |  | O |  |  | O |  | I |  |
| *Parthenocissus quinquefolia* (L.) Planch. | Vitaceae |  |  |  |  | I🗹 |  |  |  |
