# Supplementary material for: Guide to the littoral zone vascular flora of Carolina bay lakes (U.S.A.)
Source: Biodivers Data J. 2016 Apr 5;(4):e7964. doi: 10.3897/BDJ.4.e7964 (PMC4911545; doi:10.3897/BDJ.4.e7964)
Supplement: Supplementary material 7 — Provisional checklist of the littoral zone vascular flora from White Lake based on historical vouchers, personal observations, and literature reviews. [file biodiversity_data_journal-4-e7964-s007.doc]

Appendix G. Provisional checklist of the littoral zone vascular flora from White Lake based on historical vouchers, personal observations, and literature reviews. This checklist does not represent a complete inventory of this locality, but rather serves as a baseline for future research. Taxa are arranged by major groups (i.e., gymnosperms, magnoliids, monocotyledons, and eudicotyledons), then alphabetically by family, genus, and species. Basal angiosperms and pteridophytes were not represented by vouchers, observations, or reports and are therefore not included in the following checklist. Brackets around a taxon indicate that it is unvouchered (i.e., it has been reported by outside agencies or has been observed by the present author, but has not been collected). Status and rank designations are also provided for rare taxa monitored by the NC Natural Heritage Program (Robinson and Finnegan 2014).

**Gymnosperms**

CUPRESSACEAE

***Chamaecyparis thyoides*** (L.) Britton, Sterns & Poggenb. (*Anonymous*, NCSC!)

***Taxodium ascendens*** Brongn. (*Godfrey 8715*, DUKE!)

***Taxodium distichum*** (L.) Rich. (*Blomquist s.n.*, DUKE!)

**Magnoliids**

MAGNOLIACEAE

***Magnolia virginiana*** L. (*Wells & Shunk s.n.*, NCSC!)

LAURACEAE

***Persea palustris*** (Raf.) Sarg. (*Wells s.n.*, NCSC!)

**Monocotyledons**

ALISMATACEAE

***Sagittaria isoetiformis*** J.G. Sm. (*Oosting 8717*, DUKE!; T; S2, G4?)

BROMELIACEAE

[***Tillandsia usneoides*** (L.) L.] (N. Howell, pers. obs.)

CYPERACEAE

***Carex glaucescens*** Elliott (*Radford & Radford 3013*, NCU!)

***Carex longii*** Mack. (*Oosting 8716*, DUKE!)

***Cyperus grayi*** Torr. (*McCrary 1346*, NCU!)

***Eleocharis baldwinii*** (Torr.) Chapm. (*Blomquist 8718*, DUKE!; *Brun s.n.*, NCSC!)

ERIOCAULACEAE

***Eriocaulon aquaticum*** (Hill) Druce (*Demont & Robinson s.n.*, NCU!; *Godfrey s.n.*, NCSC!; *Hueske s.n.*, NCU!; SC-V; S2, G5)

HALORAGACEAE

***Myriophyllum humile*** (Raf.) Morong (*Howell* *WHLA 01*, NCSC!)

***Myriophyllum tenellum*** Bigelow (LeBlond & Sorrie 2001; E; S1, G5)

JUNCACEAE

***Juncus debilis*** A. Gray (*Godfrey & Fox 8719*, DUKE!; )

POACEAE

***Panicum hemitomon*** Schult. (*Blomquist 1525*, DUKE!; *Fox et al. 2698*, DUKE!; *Godfrey s.n.*, NCSC!)

**Eudicotyledons**

CYRILLACEAE

[***Cyrilla racemiflora*** L.] (N. Howell, pers. obs.)

FABACEAE

***Wisteria frutescens*** (L.) Poir. (*Wilbur 3588*, NCSC!)

FAGACEAE

***Quercus margaretta*** Ashe ex Small (*Fox s.n.*, NCSC!)

HYPERICACEAE

***Hypericum walteri*** J.F. Gmel. (*McCrary 1348*, NCU!)

ITEACEAE

***Itea virginica*** L. (*Oosting s.n.*, DUKE!)

LENTIBULARIACEAE

***Utricularia floridana*** Nash. (Taylor 1989; T; S1S2, G5)

MELASTOMATACEAE

***Rhexia nashii*** Small (*Radford & Radford 3006*, NCU!)

ONAGRACEAE

***Ludwigia leptocarpa*** (Nutt.) H. Hara (*McRary 1318*, NCU!)
